# Modulation of the diet and gastrointestinal microbiota normalizes systemic inflammation and β-cell chemokine expression associated with autoimmune diabetes susceptibility

**DOI:** 10.1371/journal.pone.0190351

**Published:** 2018-01-02

**Authors:** Angela M. Henschel, Susanne M. Cabrera, Mary L. Kaldunski, Shuang Jia, Rhonda Geoffrey, Mark F. Roethle, Vy Lam, Yi-Guang Chen, Xujing Wang, Nita H. Salzman, Martin J. Hessner

**Affiliations:** 1 The Max McGee National Research Center for Juvenile Diabetes at the Medical College of Wisconsin, Milwaukee, Wisconsin, United States of America; 2 The Department of Pediatrics at the Medical College of Wisconsin, and The Children’s Research Institute of Children’s Hospital of Wisconsin, Milwaukee, Wisconsin, United States of America; 3 National Institute of Diabetes and Digestive and Kidney Diseases, the National Institutes of Health, Bethesda, Maryland, United States of America; Baylor College of Medicine, UNITED STATES

## Abstract

Environmental changes associated with modern lifestyles may underlie the rising incidence of Type 1 diabetes (T1D). Our previous studies of T1D families and the BioBreeding (BB) rat model have identified a peripheral inflammatory state that is associated with diabetes susceptibility, consistent with pattern recognition receptor ligation, but is independent of disease progression. Here, compared to control strains, islets of spontaneously diabetic BB DR*lyp/lyp* and diabetes inducible BB DR+/+ weanlings provided a standard cereal diet expressed a robust proinflammatory transcriptional program consistent with microbial antigen exposure that included numerous cytokines/chemokines. The dependence of this phenotype on diet and gastrointestinal microbiota was investigated by transitioning DR+/+ weanlings to a gluten-free hydrolyzed casein diet (HCD) or treating them with antibiotics to alter/reduce pattern recognition receptor ligand exposure. Bacterial 16S rRNA gene sequencing revealed that these treatments altered the ileal and cecal microbiota, increasing the Firmicutes:Bacteriodetes ratio and the relative abundances of lactobacilli and butyrate producing taxa. While these conditions did not normalize the inherent hyper-responsiveness of DR+/+ rat leukocytes to *ex vivo* TLR stimulation, they normalized plasma cytokine levels, plasma TLR4 activity levels, the proinflammatory islet transcriptome, and β-cell chemokine expression. In lymphopenic DR*lyp/lyp* rats, HCD reduced T1D incidence, and the introduction of gluten to this diet induced islet chemokine expression and abrogated protection from diabetes. Overall, these studies link BB rat islet-level immunocyte recruiting potential, as measured by β-cell chemokine expression, to a genetically controlled immune hyper-responsiveness and innate inflammatory state that can be modulated by diet and the intestinal microbiota.

## Introduction

Type 1 diabetes (T1D) is a complex disease that arises through T-cell dependent killing of the insulin-producing β-cells. T1D incidence has increased over the past half century and a decreased proportion of “high-risk” human leukocyte antigen (HLA) genotypes, which convey the greatest genetic risk, are observed among present day diagnoses [1–3]. These observations suggest the presence of increasing environmental pressures. Modern lifestyles now include highly processed diets as well as aseptic and antibiotic practices that have likely mediated changes in the intestinal microbiota [4]. It is hypothesized that these have altered immune function and enlarged the pool of individuals susceptible to autoimmunity [5, 6].

Consistent with other studies implicating innate immunity in T1D pathogenesis (reviewed in [7]), we have identified an innate inflammatory state in T1D families [8–10]. This state includes elevated plasma cytokine/chemokine levels and is consistent with pattern recognition receptor (PRR) ligation, yet is independent of HLA, development of auto-antibody (Ab), and T1D progression. Among healthy auto-Ab negative (-) siblings possessing high-risk HLA haplotypes, we find that this state is temporally supplanted by an IL-10/TGF-β mediated regulatory state that includes age-dependent increases in activated CD4+/CD45RA-/FoxP3^high^ regulatory T-cell (Treg) frequencies [10]. These studies offer insight into the juvenile nature of T1D and suggest that failures in endogenous regulatory mechanisms that normally manage inherited T1D risk may underlie disease progression [10].

Like human T1D, diabetes in BioBreeding (BB) rats is dependent on T-cells and a high-risk major histocompatibility (MHC) haplotype (*Iddm1* locus) [11]. BB DR*lyp/lyp* rats are lymphopenic due to a mutation in *Gimap5* (*Iddm2* locus) [12], a gene necessary for post-thymic T-cell survival [13]. Consequently, DR*lyp/lyp* rats are Treg deficient and 100% of animals develop T1D independent of gender [14]. BB DR+/+ littermates are *Gimap5+/+*, are not lymphopenic, and do not develop insulitis or spontaneous T1D. However, T1D can be triggered in young DR+/+ rats by infection with Kilham’s rat virus (KRV) and other viruses [15, 16]. T1D susceptibility in BB rats extends beyond *Iddm1* and *Iddm2*, as MHC-matched Wistar-Furth (WF), PVG.R8, and Fischer (F344) rats are not susceptible to viral induced T1D and lymphopenic *Gimap5-/-* F344 rats do not develop spontaneous diabetes [16, 17].

Similar to T1D families, we have identified an innate inflammatory state in BB rats that includes elevated plasma cytokine levels and is consistent with PRR ligand exposure, yet is independent of MHC, lymphopenia, insulitis, and disease progression [15, 18]. This state in DR+/+ rats, is also temporally supplanted by an IL-10/TGF-β-mediated immunoregulatory state that coincides with the inability of KRV to induce T1D in older animals [15]. These findings suggest that autoimmunity is promoted when the endogenous innate state is augmented by additional inflammatory signals (e.g. viral infection) prior to the establishment of counter-regulatory mechanisms.

Studies of rodent models support a role for the gut microbiota in T1D pathogenesis. When raised under specific pathogen free conditions, treatment of animals with antibiotics or probiotics can prevent diabetes, presumably through altering the level or nature of PRR ligand exposure and/or selection for protective microbial lineages [19, 20]. Germ-free animals exhibit high rates of diabetes incidence, suggesting that microbes are not necessary for disease progression [21, 22]. Finally, conventionally raised animals lacking MyD88, an adaptor protein through which many innate immune receptors transduce signal, fail to develop diabetes [21]. Collectively, the literature suggest that interactions between the innate immune system and microbiota can both positively and negatively modulate disease development.

In this study we examined the influence of the diet and gut microbiota on the endogenous innate state present in BB rats relative to MHC-matched F344 control rats. Independent of insulitis or T1D progression, islets of BB rats provided a standard cereal diet (normal diet, ND) expressed a transcriptional program consistent with PRR ligand exposure. In order to alter or reduce microbial antigen exposure, weanlings were provided a hydrolyzed casein diet (HCD) or treated with antibiotics. These conditions did not normalize the inherent hyper-responsiveness of BB rat leukocytes to *ex vivo* TLR stimulation, however, HCD normalized chemokine expression by BB rat β-cells and reduced T1D incidence in DR*lyp/lyp* rats. Notably, these effects could be abrogated by the introduction of gluten to HCD. Overall, these studies link BB rat islet-level immunocyte recruiting potential, as measured by β-cell chemokine expression, to a genetically controlled immune hyper-responsiveness and innate inflammatory state that is influenced by diet and intestinal microbiota.

## Materials and methods

### Animals

This study utilized DR+/+ and DR*lyp/lyp* rats, as well as Fischer (F344) control rats that possess either *Iddm1* (designated F+/+) or both *Iddm1* and *Iddm2* (designated F*lyp/lyp*) [17], These strains, which were sourced from colonies maintained at the Medical College of Wisconsin, were provided chow and water *ad libitum* as previously described [12, 17]. Twenty-one day old littermates were weaned onto a normal cereal diet (ND) containing both plant and animal protein sources (LabDiet 5L0D, Purina, St. Louis, MO, USA), a hydrolyzed bovine casein diet (HCD, Modified AIN-93G diet, Dyets Inc., Bethlehem, PA, USA), or a custom HCD where 50% of the casein protein was replaced with gluten (HCD+G, Dyets Inc). The composition of these diets is provided (S1 Table). Alternatively, BB littermates were provided ND and treated with bacitracin and streptomycin (B/S) provided in the drinking water (2mg/ml each, Sigma-Aldrich, St. Louis, MO, USA). Weight and blood glucose (measured with an Ascensia Elite XL glucometer; Bayer, Tarrytown, New York, USA) were monitored from 40 days of age; diabetes was defined as tail vein blood glucose levels ≥250 mg/dl on two consecutive days. Blood was collected by heart puncture from rats under isoflurane anesthesia into EDTA vacutainer tubes (Becton-Dickinson, Franklin Lakes, NJ, USA). Plasma was separated by centrifugation and stored at -80°C. Tissues were collected under isoflurane anesthesia. While under anesthesia animals were sacrificed by pneumothorax followed by removal of the left ventrical; all efforts were made to minimize suffering. The study utilized 279 rats. The National Institutes of Health Guide for the Care and Use of Laboratory animals was followed and all protocols were approved by the Medical College of Wisconsin Institutional Animal Care and Use Committee (Assurance Number A3102-01).

### Islet gene expression profiling and plasma induced transcription analysis

Rat islets were isolated as described [23] and total RNA was extracted with Trizol (Invitrogen, Carlsbad, CA, USA). RNA was amplified/labeled (Affymetrix 3' IVT Express Kit, Affymetrix, Santa Clara, CA, USA), then hybridized to the Affymetrix RG230 2.0 array per the manufacturers’ protocol.

Plasma induced transcription assays were conducted as described [15, 18], using “reporter” PBMCs drawn from 180 day old male Brown Norway (BN) rats and purified through Histopaque (Sigma-Aldrich) density gradient centrifugation. Transcription was induced by culturing PBMCs for 6 h at 37°C in 5% CO_2_ with 20% plasma in RPMI 1640 medium. Tested plasma included autologous BN (self-baseline control) as well as plasma collected from 30 day old: DR+/+ rats provided ND (hereafter designated DR+/+ ND), DR+/+ rats provided HCD (DR+/+ HCD), DR+/+ rats treated with B/S (DR+/+ B/S), and F+/+ rats provided ND (F+/+ ND). RNA was prepared and induced transcription was measured using the Affymetrix RG230 2.0 array as described above.

Array images were quantified with Affymetrix Expression Console Software and normalized and analyzed with Partek Genomic Suite (Partek Inc, St. Louis, MO, USA). Longitudinal islet data were analyzed using Short Time Series Expression Miner (STEM [24]); transcripts were assigned to model profiles utilizing input parameters: absolute difference between the values of any two time points = |log_2_ ratio| > 0.5 (1.4 fold); maximum number of units a model profile may change between time points = 1. Gene expression differences were also evaluated by principle component analyses as well as by non-parametric rank product tests and determination of false discovery rates (FDR) to assess the rate of type I errors in multiple testing [25]. Ontological analyses were conducted with the Database for Annotation, Visualization, and Integrated Discovery version 6.7 (DAVID) [26], and the Ingenuity Pathway Analysis (IPA) package (Ingenuity Systems, Redwood City, CA, USA). Hierarchical clustering was conducted with Genesis [27]. Data files are available at The National Center for Biotechnology Information Gene Expression Omnibus (accession number: GSE80518).

### Direct detection of inflammatory mediators

Plasma cytokine levels were measured with the BeadLyte assay (Millipore, Billerica, MA, USA) per the manufacturer's protocol and a Bio-Plex Luminex 100 XYP instrument. Concentrations were calculated with the Bio-Plex Manager 4.1 software and a 5 parameter curve fitting algorithm applied for standard curve calculations. Plasma high mobility group box 1 protein (HMGB1) levels were measured by ELISA (R&D Systems, Minneapolis, MN, USA).

TLR4 receptor activity was measured using TLR4/NF-kB/secreted embryonic alkaline phosphatase (SEAP) Stable Reporter Cells (IML-104) per the manufacturer’s protocol (Invivogen, San Diego, CA, USA). These are HEK-293 cells that express human TLR4 as well as a secreted alkaline phosphatase under control of a NF-kB dominant promoter after TLR4 activation. Plasma (40 μl) was added to 200ul of detection media (Invivogen) containing approximately 140,000 reporter cells or control cells and incubated at 37°C with 5% CO_2_ for 15–17 hours. SEAP activity was measured in the conditioned medium using an alkaline phosphatase fluorometric assay (Abcam, Cambridge, MA, USA) following the manufacturer’s protocol.

Two-color immunofluorescent staining of pancreatic sections was conducted to localize chemokine expression to β-cells, as previously described [28]. Briefly, primary staining was conducted with goat polyclonal anti-Ccl11 Abs (R&D Systems, #AF-420-NA), or rabbit polyclonal anti-Cxcl10 Abs (Bioss, Atlanta, GA, USA, #bs-1502R) in combination with mouse monoclonal anti-insulin Abs (Sigma-Aldrich). Secondary staining was accomplished with Alexa594-conjugated donkey anti-goat IgG, or Alexa594-conjugated donkey anti-rabbit IgG, and FITC-conjugated donkey anti-mouse IgG (all from Jackson ImmunoResearch Laboratories, West Grove PA, USA). Fluorescence data was collected from 12–30 islets of ≥3 rats per experimental condition with a 40X objective lens, quantified with MetaMorph Software (Molecular Devices, Sunnyvale, CA), and expressed as relative fluorescence units (RFU).

### Analysis of ileal and cecal microbiota

Ileal and cecal sections were dissected from 30 day old DR+/+ ND, DR+/+ HCD, DR+/+ B/S, and F+/+ ND rats. Samples were immediately homogenized in PBS and total (host and bacterial) genomic DNA was extracted using the MoBIO PowerSoil Isolation Kit (Mo Bio Laboratories, Inc, Carlsbad, CA, USA) as described [29]. Bacterial abundance was assessed through quantitative PCR (qPCR) using universal eubacterial primers (1369F and 1492R) that target sequences adjacent to variable region 9 (V9) of the 16S ribosomal RNA (16S rRNA) gene [30].

Composition of the microbiota was determined through sequencing of the 16S rRNA gene V4 region (Diversigen, Baylor College of Medicine, Waco, TX, USA). PCR products were produced, then sequenced on the MiSeq platform (Illumina, San Diego, CA, USA) using the 2x250-bp protocol, yielding pair-end reads that overlap by ~247 bp [31]. The raw binary base call (BCL) files were called into fastqs by Casava v1.8.3 (Illumina). The read pairs were demultiplexed through the use of unique molecular barcodes, filtered for PhiX using Bowtie2 v2.2.1 [32] and reconstituted into two fastq files for each read using standard BASH. Sequencing reads were merged, allowing 4 mismatches per ≥50 bases, then processed using USEARCH v7.0.1001 [33]. Sequences were demultiplexed using QIIME v1.8.0 [34] and then clustered using the UPARSE pipeline [33]. Operational taxonomic unit (OTU) classification was accomplished by mapping the UPARSE OTU table to the SILVA database [35]. Relative abundances were determined by mapping the demultiplexed reads to the UPARSE OTUs. An OTU table was constructed using a custom script and the output files generated in the two previous steps. The OTU table was used to calculate alpha diversity, beta diversity and taxonomic summaries [36–38]. The Vegan (https://cran.r-project.org/web/packages/vegan/index.html) and Ecodist [39] packages in R 3.0.2 were used for data analysis. Intersample (beta) diversity was assessed with the Bray–Curtis metric and the statistical significance of differences in microbiota diversity between groups was assessed with Adonis (in Vegan). Non-metric multidimensional scaling ordination (in Ecodist) was used to visualize group clustering and diversity distance between samples. Abundance data were log transformed (log_10_ of sequence count + 1) and differences in OTU abundance between groups were assessed by heteroscedastic, two-sided Student’s *t*-tests. The Linear Discriminant Analysis (LDA) approach within the LefSe tool was used to identify significantly differentiating taxa among the ileal and cecal communities of the four experimental conditions [40].

### TLR stimulation of DR+/+ and F+/+ rat PBMC

PBMC from 30-day-old DR+/+ ND, DR+/+ HCD, DR+/+ B/S and F+/+ ND rats were isolated from whole blood by Histopaque (Sigma-Aldrich) density gradient centrifugation. Cells were plated into low attachment 24-well plates (Sigma-Aldrich) at a density of 5x10^5^ cells/0.5mL in RPMI medium/20% fetal calf serum with increasing concentrations of LPS (0, 0.1, 1, 10, and 100 ng/ml; Sigma-Aldrich). After 24 hours of culture at 37°C in 5% CO_2_, the conditioned media was recovered after centrifugation and stored at -80°C. IL-1β and TNF-α levels in the conditioned media were measured by ELISA (R&D Systems).

## Results

### The BB rat islet transcriptome is consistent with PRR ligand exposure

To investigate T1D susceptibility at the target tissue level, we longitudinally examined the islet transcriptomes of DR*lyp/lyp*, DR*+/+*, F*lyp/lyp*, and F*+/+* rats provided ND. The study encompassed day 20 (weaning), day 30 (prior to histological detection of Ccl11 expression by BB β-cells [28]), day 40 (prior to insulitis in DR*lyp/lyp* rats), and day 50 (after insulitis but prior to diabetes onset in DR*lyp/lyp* rats).

**Fig 1:**
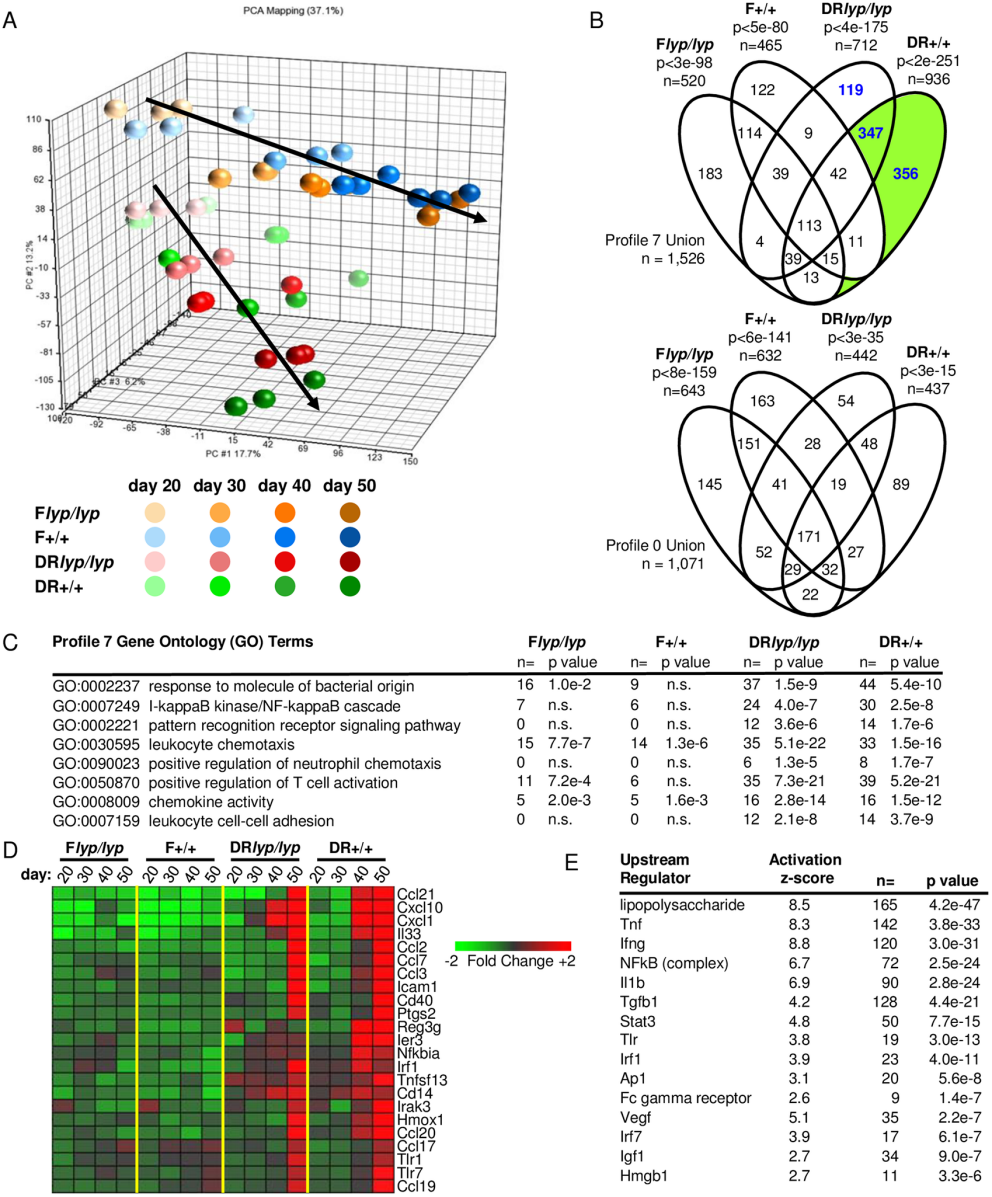
Longitudinal analysis of DR*lyp/lyp*, DR*+/+*, F*lyp/lyp* and F*+/+* rat islet transcriptomes. Gene expression profiling was conducted on a single pool of islet RNA that was generated through an equal RNA contribution from 6–8 rats for each experimental condition. Each RNA pool was analyzed in triplicate. A. Unsupervised principal component analysis of DR*lyp/lyp*, DR*+/+*, F*lyp/lyp* and F*+/+* rat islet gene expression profiles at 20, 30, 40 and 50 days of age. The analysis was conducted using the normalized intensities for all 31,042 probe sets present on the array. B. Time series data for each strain were analyzed with STEM. The maximum number of model profiles to which transcripts could be assigned was set at 8. The temporal analysis of each strain identified two significant model profiles, Profile 7 (transcripts showing increasing temporal expression) and Profile 0 (transcripts exhibiting decreased temporal expression). The number of transcripts associated with each model profile, the significance at which the profile was identified, and the relationship of the probe sets associated with the model profiles for each strain are indicated. Consistent with the STEM analysis, and the temporal divergence in the BB versus F344 islet transcriptomes observed in the principal component analysis, pairwise comparisons of the islet expression data between DR+/+ and age-matched F+/+ rats show that the number of genes exhibiting significant differences in expression increased with age: day 20 n = 769; day 30 n = 884; day 40 n = 1,075; day 50 n = 1,297 (|log_2_ ratio| ≥ 0.5, FDR ≤ 0.05). C. GO Biological Processes significantly enriched within Profile 7 of DR*lyp/lyp* and DR+/+ rats. Representative pathway terms, the number of identified genes and the significance of enrichment are tabulated for each temporal series. n.s., not significant. D. Mean expression levels of a subset of well annotated transcripts belonging to Profile 7 of DR*lyp/lyp* and DR+/+ rats. E. Upstream regulator analysis of the 703 probe sets temporally up-regulated in BB rat islets independent of insulitis and T1D progression (green shaded portions of Fig 1B). The p-value (determined with a Fisher exact test) reflects the significance of the overlap between the regulated probe sets within the data set and genes regulated by the transcriptional regulator. A p-value of ≤ 0.05 and Z-score greater than 2.0 is significant.

Unsupervised principal component analysis found the islet transcriptomes of the four strains most similar at day 20, with the BB and Fischer lineages diverging from one another as a function of age (Fig 1A). For each strain, the islet transcriptomes of young and old rats were highly distinct. Therefore, the longitudinal data for each stain were analyzed with Short Time Series Expression Miner (STEM), which clusters transcripts exhibiting similar temporal expression patterns into model profiles and subjects them to gene ontology (GO) enrichment analysis. Within each of the four data sets, two statistically significant model profiles were identified: model profile 7 (transcripts exhibiting increased temporal expression) and model profile 0 (transcripts exhibiting decreased temporal expression). The relationships between the transcripts assigned to Profile 7 and Profile 0 across the four sub strains are illustrated in Fig 1B.

Among the four analyses, 2,576 regulated transcripts were assigned to either Profile 7 or Profile 0 (S2 Table). Since a significant proportion of these were exclusive to Profile 7 of BB rats (n = 822, 31.7%, p<1x10^-6^, χ^2^-test, Fig 1B, blue font), the analysis was focused on this data subset. GO terms significantly associated with Profile 7 of BB rats reflected temporal induction of a transcriptional program consistent with bacterial antigen exposure and innate immune activation (Fig 1C). Annotated transcripts included innate receptors (*Tlr1*, *Tlr7*, *Cd14*), signal transducers (*Irak3*, *Irf1*), tumor necrosis factor (TNF) family members (*Tnfsf13*), and proteins related to host stress response (*Reg3g*, *Ier3)*. Notably, the analyses revealed that both DR*lyp/lyp* and DR+/+ rat islets temporally express numerous chemokines, including *Cxcl1*, *Ccl3* and *Ccl21* involved in neutrophil chemotaxis, as well as *Ccl2*, *Ccl19*, *Ccl20 and Cxcl10* involved in monocyte and lymphocyte chemotaxis (Fig 1D).

Ingenuity Pathway Analysis (IPA) upstream regulator analysis was conducted on the subset of transcripts unique to DR*+/+* Profile 7, inclusive of the DR*lyp/lyp*:DR*+/+* intersection (Fig 1B, green shaded). This analysis identified LPS with the greatest significance, as well as several cytokines, as candidate mediators underlying Profile 7 (Fig 1E). The proinflammatory transcriptional program in BB islets arises in a time dependent manner and is present in DR+/+ rats, which do not develop insulitis or spontaneous diabetes. It is therefore independent of infiltrating immune cells and thus hypothesized to be a consequence of elevated PRR ligand exposure.

### HCD and B/S treatment alter the ileal and cecal biome composition

**Fig 2:**
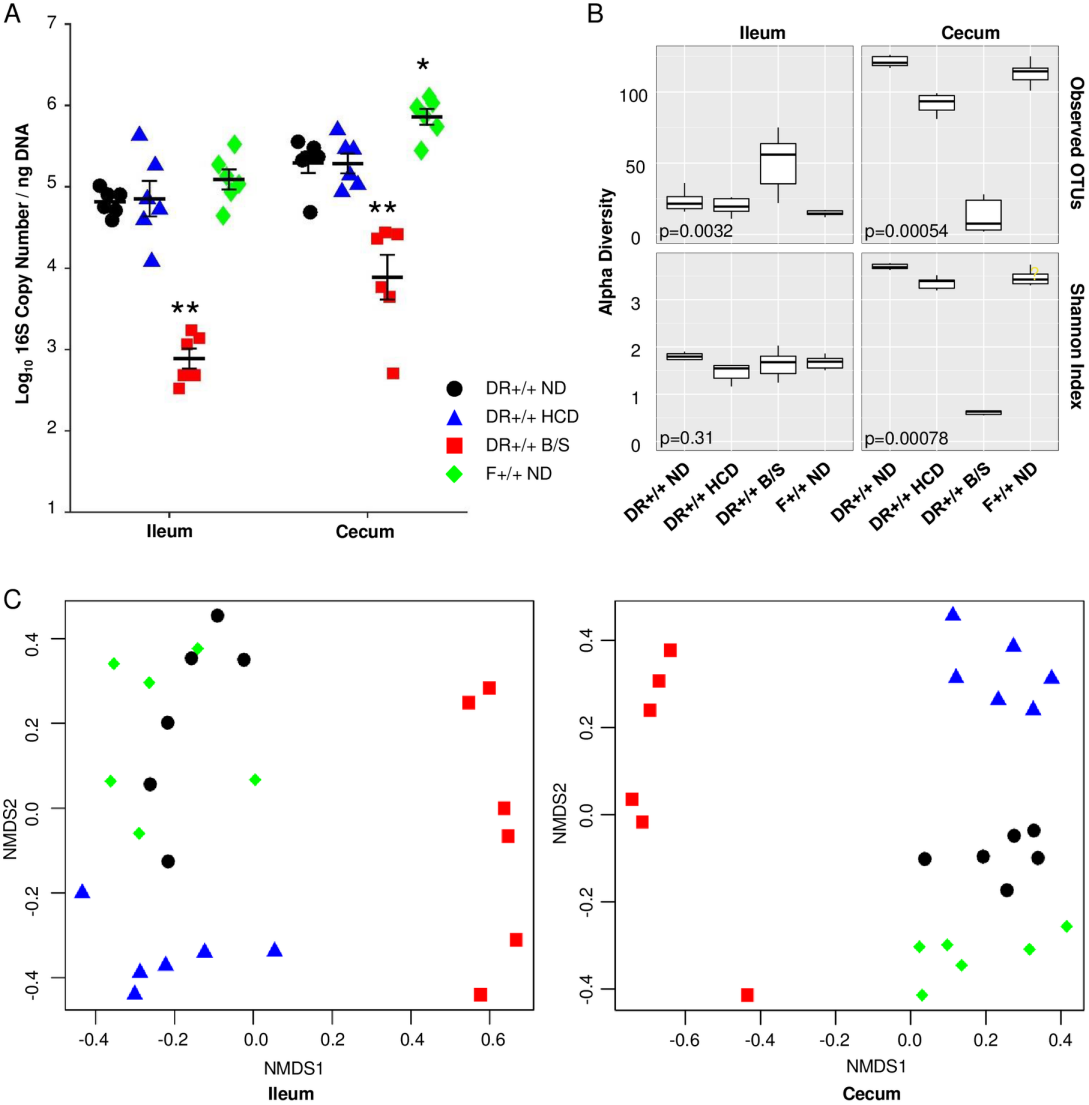
Influence of HCD and B/S treatment on the intestinal microbiota of DR+/+ littermates. Twenty one day old DR+/+ weanlings (n = 6 per experimental condition) were provided ND, HCD, or ND and treated with B/S for 10 days. Total DNA was extracted from whole cecum and ileum and analyzed. A. Bacterial abundance was assessed through quantitative PCR targeting the V9 region of the bacterial 16S rRNA gene. A standard curve was used to determine the 16S rDNA copy number for each sample and normalized to ng total DNA. (ANOVA: * p < 0.05 and ** p < 0.001 versus DR+/+ ND). B. Alpha diversity measures. Box-and-whisker plots comparing the species-level richness as operational taxonomic units (OTU) and Shannon index. The top and bottom of the boxes show the 75th and 25th percentile and the ends of the whiskers show the maximum and minimum values. Lines within the boxes represent median values (50th percentile). C. Beta diversity was assessed among experimental conditions using Bray-Curtis dissimilarity index and displayed as non-metric multidimensional scaling (NMDS) plots. DR+/+ ND (black), DR+/+ HCD (blue), DR+/+ B/S (red), F+/+ ND (green).

HCD has anti-inflammatory and anti-diabetogenic effects in rodent models of T1D, including the BB rat [22, 41–43]. Further, antibiotic treatment also protects lymphopenic BB rats from diabetes progression [19, 44]. To investigate relationship between diet, intestinal microbiota and the endogenous innate inflammatory state present in BB rats, without the confounding variable of diabetes progression, 20 day old DR+/+ littermates were weaned onto normal cereal diet (ND), or HCD, or ND plus bacitracin/streptomycin (B/S). This antibiotic combination targets Gram-positive and Gram-negative bacteria, exhibits poor absorption from the intestinal tract [45], and was determined not to impair normal weight gain. At 30 days of age, ileal and cecal DNA was prepared and microbiota was analyzed and compared to that of F+/+ ND rats. Expectedly, bacterial abundance was reduced 10–100 –fold in B/S treated rats compared to DR+/+ ND rats (p < 0.001), as assessed through quantitative PCR of the bacterial 16S rRNA gene V9 region. Compared to DR+/+ ND rats, cecum of F+/+ ND rats exhibited greater bacterial abundance (Fig 2A). The composition of the ileal and cecal microbiota was determined through sequencing of the bacterial 16S rRNA gene V4 region. A total of 219,346 quality reads were obtained from the 48 samples. Among these 173,167 (78.95%) were mapped to at least the family level. The sequences were collapsed into operational taxonomic units (OTUs) based upon sequence identity ≥97%; these represented a total of 11 phyla, 66 families, and 117 unique OTU (S3 Table).

In the ileum, the OTU richness was significantly different across the experimental conditions, but the Shannon index was not (Fig 2B). In the cecum, these alpha diversity measures (richness and evenness of taxa within a population) were significantly different across the experimental conditions (p<0.01), and lower within the cecum of B/S treated DR+/+ rats relative to the other conditions. The Bray-Curtis dissimilarity index [46], was used to assess beta diversity (overlap in taxa shared between populations), where ileal and cecal communities of DR+/+ littermates provided HCD or treated with B/S were significantly different from DR+/+ rats provided ND (Adonis, p<0.001, Fig 2C).

**Fig 3:**
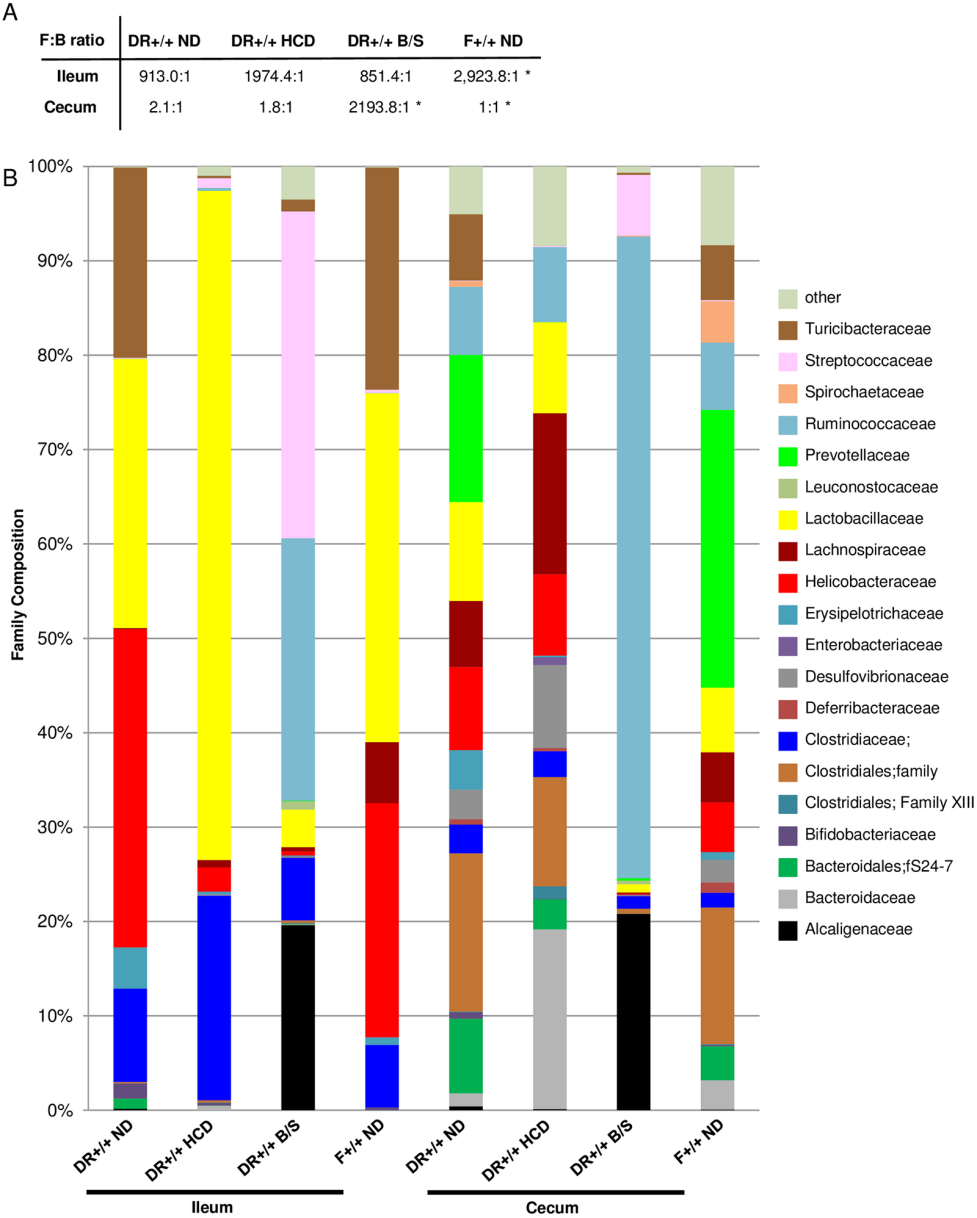
Phylum and family analysis of ileal and cecal communities. A. Indicated are the average phylum Firmicutes: phylum Bacteroidetes (F:B) ratios, calculated from the totals of the individual OTUs for each experimental condition. * indicates p < 0.05 versus DR+/+ ND, t-test. B. Family distribution. The stacked bar plots show the average Family composition of the 6 rats per experimental condition that were analyzed.

At the phylum level, human T1D patients exhibit increased relative abundances of Bacteroidetes, as well as reductions in the Firmicutes:Bacteroidetes ratio compared to healthy controls [47–50]. In diabetes susceptible DR+/+ ND rats, the ileal Firmicutes:Bacteroidetes ratio was lower than in F+/+ ND rats (Fig 3A). The cecal Firmicutes:Bacteroidetes ratio was similar across the experimental groups, with the exception of the B/S treated DR+/+ rats, where it was significantly increased.

At the family level, the ileal and cecal communities differed across the experimental conditions (Fig 3B). Relative to DR+/+ ND rats, the ileal community of DR+/+ HCD rats exhibited reductions in the family Helicobacteraceae (33.7% versus 2.6%), as did the ileal and cecal communities of DR+/+ B/S rats (33.7% versus 0.5%, and 8.8% versus 0.1%). Compared to DR+/+ ND rats, Lactobacillaceae family members comprised a greater proportion of the ileal community in DR+/+ HCD rats (28.5% versus 70.9%). Compared to DR+/+ ND rats, DR+/+ B/S rats exhibited increases in Ruminococcaceae family members in both the ileum (0.01% versus 27.8%) and cecum (7.2% versus 68.0%).

**Fig 4:**
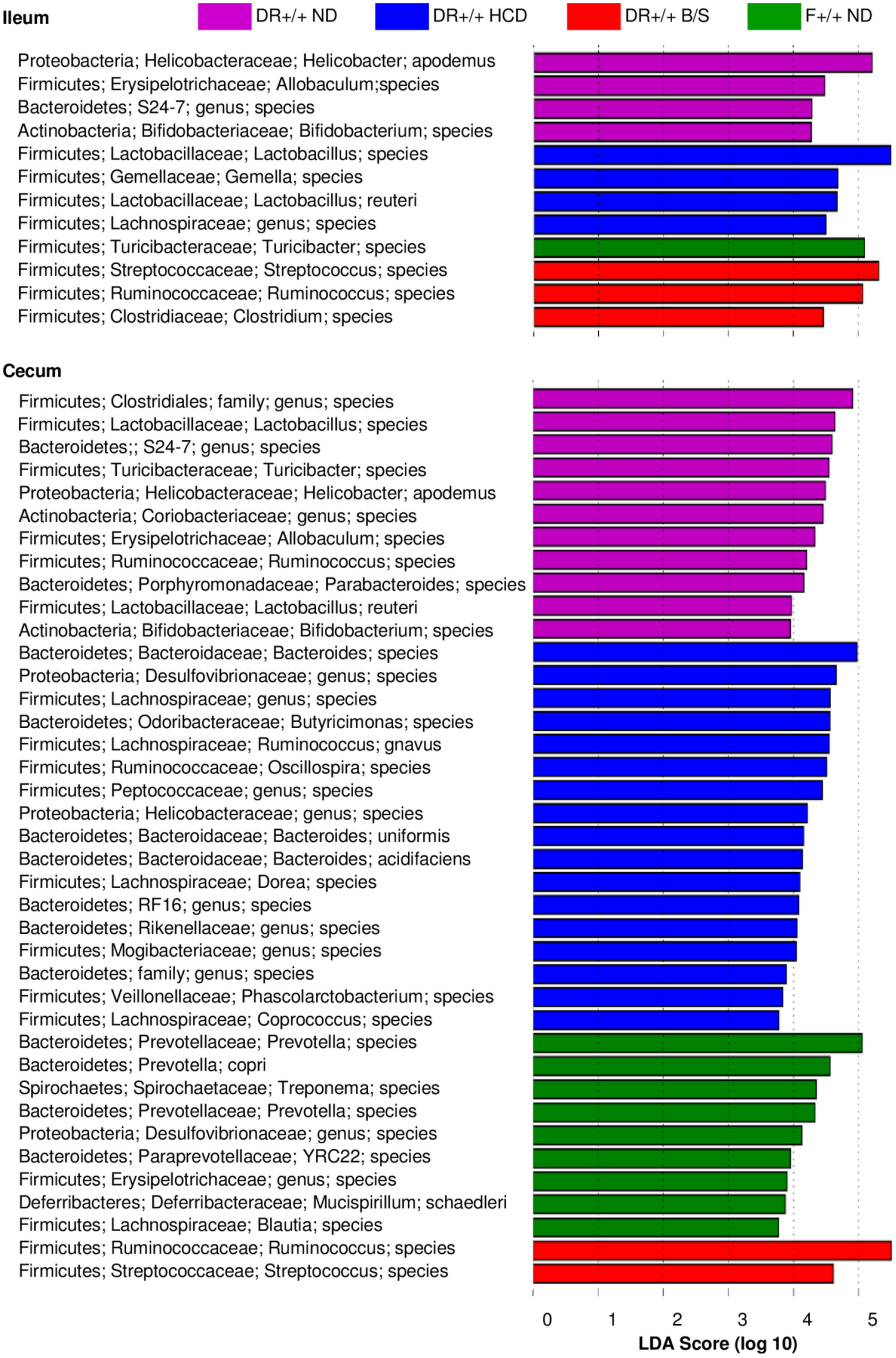
LDA effect size of the significantly differentiating microbial taxa within ileal and cecal communities of DR+/+ ND, DR+/+ HCD, DR+/+ B/S, and F+/+ ND rats. The LefSe package was used to generate the LDA effect size. The following thresholds were deemed significant: LDA cut-off = 2; Wilcoxon p-value = 0.05.

Differentiating features among the ileal and cecal communities among the four experimental conditions were identified using the Linear Discriminant Analysis (LDA) -based LefSe approach [40]. A total of 12 differentiating features were identified within the ileal samples that possessed an absolute LDA score >4; a total of 39 differentiating features were identified within the cecal samples that possessed an absolute LDA score of >3.5 (Fig 4). Among these, *Helicobacter apodemus* was identified as a differentiating feature of the DR+/+ ND ileum and cecum; notably, *Helicobacteraceae* family members act as pathogenic agents in colitic diseases [51]. Within the family Lactobacillaceae, *Lactobacillus reuteri* was a differentiating feature of the DR+/+ HCD ileal community. Lactobacilli possess broad beneficial properties and have been employed in probiotic interventions aimed at normalization of inflammatory processes. Ruminococcus species were a distinguishing feature of ileal and cecal samples of DR+/+ B/S rats, and in studies of healthy human subjects abundances of the family Ruminococcaceae have negatively correlated with plasma inflammatory markers (lipopolysaccharide-binding protein, C-reactive protein and IL-6) [52]. Overall, DR+/+ HCD rats or DR+/+ B/S rats experienced significant alterations in the microbiota of the ileum and cecum that, based upon other studies, including those examining alteration of the intestinal microbiota in the context of obesity [53], were consistent with anti-inflammatory effects [22, 41, 42, 54–56].

### HCD and B/S treatment normalize the systemic innate inflammatory state measured in plasma

**Fig 5:**
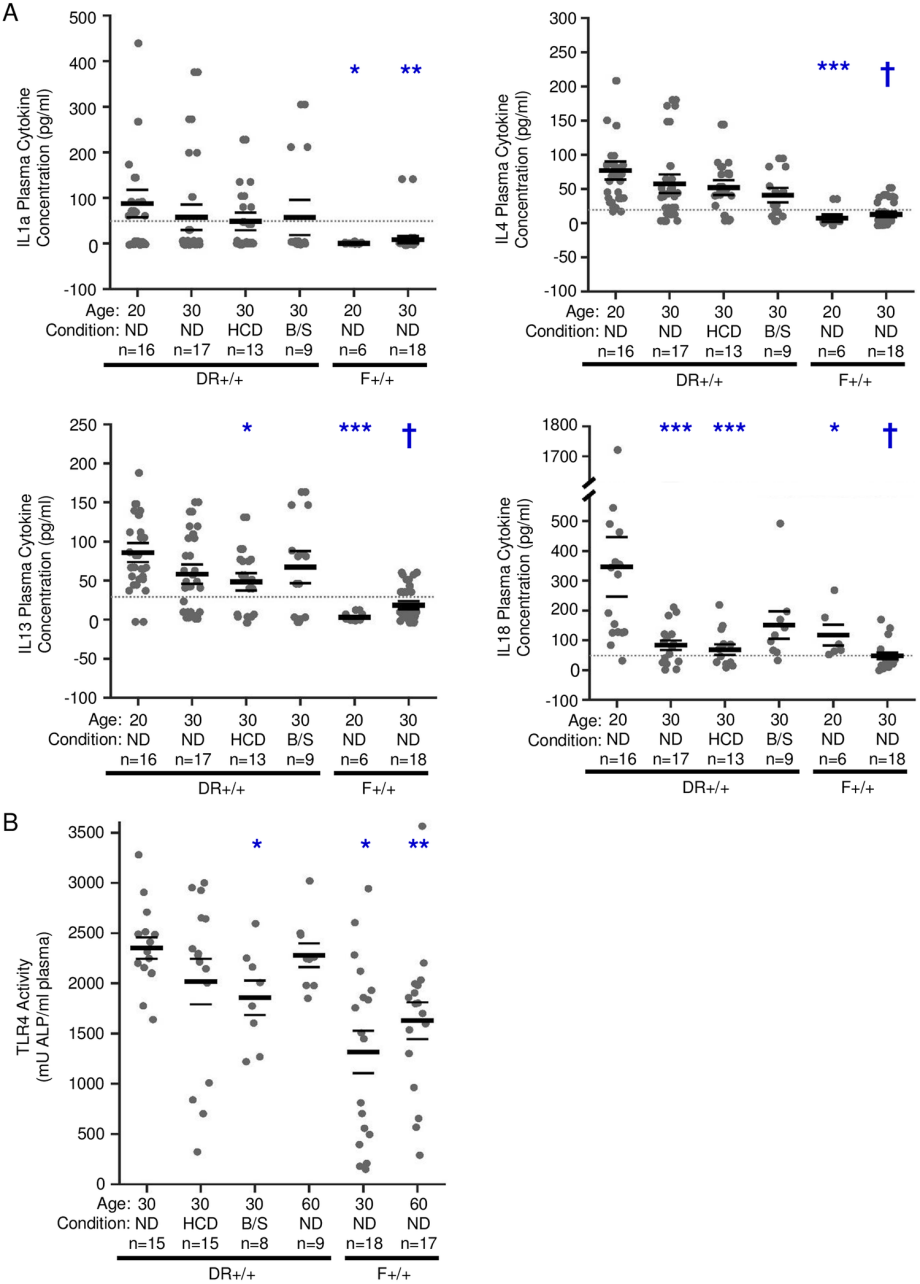
Plasma levels of immune mediators. A. Plasma cytokines were measured by multiplex ELISA. As indicated, plasma samples were collected at 20 days of age from F+/+ ND and DR+/+ ND rats and at 30 days of age in F+/+ ND, DR+/+ ND, DR+/+ HCD and DR+/+ B/S rats. The lowest reliable detection level (lowest standard) is shown with a dotted line. Significance based on rank-sum test: * p < 0.05, ** p < 0.01,***p < 0.001, † p < 0.0001 versus 20-day-old DR*+/+* rats. Error bars = SEM. B. Total TLR4 receptor activity induced by plasma collected at 30 and 60 days of age from F+/+ ND, DR+/+ ND, 30 days of age from DR+/+ HCD and DR+/+ B/S rats. * p < 0.05 when compared to day 30 DR +/+ ND. ** p < 0.05 when compared to day 60 DR+/+ ND. Error bars = SEM.

Plasma levels of IL-1a, IL-4, IL-13, and IL-18 were investigated. The analysis focused on these cytokines because previously, using a 25-plex ELISA, they were found detectable in 30 day old DR+/+ ND rats and observed to temporally decline by day 60 [15]. Here, levels of these cytokines were higher in 20 day old DR+/+ weanlings compared to 20 and 30 day old F+/+ ND rats (Fig 5A). While never as low as 30 day old F+/+ ND rats, plasma IL-1a, IL-4, IL-13, and IL-18 levels declined between 20 and 30 days in DR+/+ ND rats, but only IL-18 reached statistical significance. Compared to 20 day old DR+/+ ND rats, 30 day old DR+/+ HCD rats experienced significantly reduced IL-13 levels. These analyses corroborated the presence of a peripheral innate inflammatory state in young DR+/+ rats relative to F+/+ rats that declines in an age-dependent manner, but they did not confirm that HCD or B/S treatment robustly altered the immune state.

HMGB1 (high mobility group box 1) and lipopolysaccharide (LPS), endogenous and exogenous TLR4 ligands, respectively, were identified as potential mediators underlying the BB rat islet transcriptomes (Fig 1E). HMGB1 is a chromatin-binding protein that maintains DNA structure; however, when passively released from damaged cells or secreted from activated immune cells it acts as a damage-associated molecular pattern [57]. HMGB1 levels were not different when comparing DR+/+ to F+/+ plasma at 30 days of age (77.0+/- 3.1 ng/ml versus 75.5 +/- 7.2 ng/ml, mean +/- SEM) or ≥50 days of age (81.3 +/- 10 ng/ml versus 85.9+/- 6.8 ng/ml). Further, HCD or B/S treatment for 10 days did not significantly alter HMGB1 plasma levels from that of day 30 DR+/+ rats provided ND. Thus HMGB1 was excluded as a possible mediator underlying the BB rat islet transcriptomes. Plasma LPS concentrations in 30 and ≥50 day old DR+/+ and F+/+ rats were not detected by the limulus amoebocyte lysate assay. However, the sensitivity of the limulus amoebocyte lysate assay is known to be impaired by plasma components [58], therefore we used a reporter cell line that specifically recognizes ligands that activate TLR4. Plasma of 30 and ≥50 day old DR+/+ rats induced higher TLR4 receptor activity compared to age-matched F+/+ rats (Fig 5B). Paralleling the reductions observed in the intestinal bacterial abundance (Fig 2A), B/S treatment significantly reduced plasma TLR4 activity in 30 day old DR+/+ rats (Fig 5B). While not reaching statistical significance, TLR4 activity trended lower in DR+/+ rats fed HCD, consistent with the reported capacity of HCD to improve intestinal barrier function in BB rats [41].

Multiplex ELISAs are not always informative of the inflammatory state due to the low cytokine/chemokine levels typically present in the periphery and the combinatorial effects of plasma-born mediators, including PRR ligands. To fill this gap, we have extensively used a sensitive bioassay, where subject plasma is co-cultured with a well-controlled PBMC reporter population to induce a transcriptional response [59]. This in turn is sensitively and comprehensively measured with a transcriptome-scale array followed by the application of pathway analyses that enable the interpretation of the data in terms of inflammatory and regulatory immune activities. When we previously applied this approach to longitudinal samples collected from DR*lyp/lyp* and DR+/+ rats provided ND, distinct temporal responses were observed [15, 18]. Specifically, plasma of lymphopenic DR*lyp/lyp* rats induced a partially IL-1 dependent proinflammatory transcriptional program by 50 days of age; with later and weaker induction of IL-10/TGF-β -mediated regulatory transcription. In contrast, plasma of nonlymphopenic DR+/+ rats more rapidly induced proinflammatory transcription, however this was paralleled by robust induction of IL-10/TGF-β –dependent regulatory transcription by 40 days of age that coincided with the inability to induce T1D with KRV [15].

**Fig 6:**
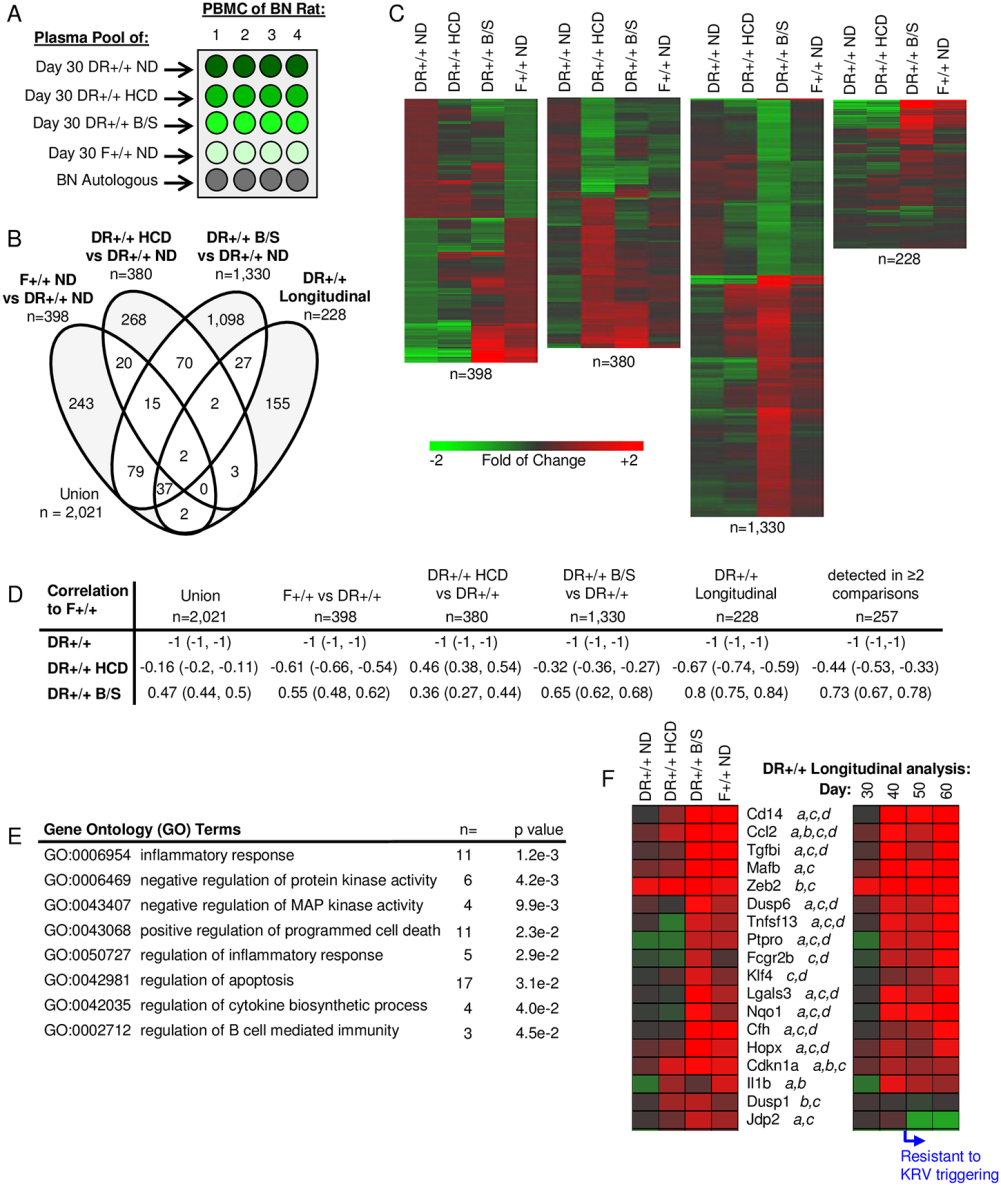
Partial normalization of the extracellular milieu by HCD and B/S treatment as measured by plasma induced transcription. A. Experimental design: responder PBMCs from four BN rats were cultured separately under 5 different conditions (n = 4 cultures for each condition): BN autologous plasma and plasma pools of 30-day-old rats: DR+/+ ND, DR+/+ HCD, DR+/+ B/S, and F+/+ ND. Plasma pools were each generated from equal volumes from six individual rats. Gene expression was measured independently in each culture and all data were normalized with that of the autologous induction. B. Venn diagram illustrating the relationship of the probe sets regulated to thresholds (|log_2_ ratio| ≥ 0.263 and Rank Product p-value ≤ 0.05) when comparing DR+/+ ND rats to F+/+ ND rats, DR+/+ HCD rats and DR+/+ B/S rats. Included in the analysis is the previously described longitudinal plasma induced transcriptional analyses of DR+/+ ND rats at 30, 40, 50 and 60 days of age [15]. C. One-way hierarchical clustering (probe sets only) for each of the data sets illustrated in Panel B. D. Pearsons Correlation Coefficients between the data sets illustrated in panel C. Indicated in parentheses are, respectively, the lower and upper 0.99 confidence intervals. E. GO Biological Processes and Molecular Functions significantly enriched among the 257 probe sets detected in ≥2 of the comparisons (unshaded portions of the venn diagram illustrated in Panel B). Representative pathway terms, the number of identified genes and significance of enrichment are tabulated. F. Mean expression levels of a subset of well annotated transcripts detected in ≥2 of the comparisons (*a*, *b*, *c*, and *d* denote the comparisons within which the transcript is detected; *a*: F+/+ ND versus DR+/+ ND, *b*: DR+/+ HCD versus DR+/+ ND, *c*: DR+/+ B/S versus DR+/+ ND, and *d* DR+/+ longitudinal analysis.

Plasma induced transcription was used to characterize the extracellular milieu at 30 days of age in DR+/+ HCD rats or DR+/+ B/S rats treated from weaning, as well as age matched DR+/+ and F+/+ rats provided ND (Fig 6A). This analysis identified a union of 2,021 significantly regulated transcripts when including the 228 temporally up-regulated probe sets identified during our previous plasma induced transcription analysis of DR+/+ ND rats at 30, 40, 50 and 60 days of age [15] (Fig 6B, S4 Table). The longitudinal data set was included to determine how HCD and B/S treatment influenced immune balance relative to the temporally acquired regulatory state that coincides with resistance to KRV triggering of T1D.

Both HCD and B/S treatment partially normalized the plasma milieu of DR+/+ rats, resulting in signatures more similar to those of F+/+ ND rats. This is reflected by the heat maps (Fig 6C) and Pearson’s correlation coefficients (Fig 6D) for each of the four data subsets. Overall, a greater normalization occurred in the plasma induced signatures of DR+/+ B/S rats, paralleling the reductions in GI microbiota and the lower TLR4 reporter activity observed in these animals, and indicating that PRR ligand exposure contributes to the underlying DR+/+ innate inflammatory state.

The plasma induced signatures of DR+/+ HCD rats significantly overlapped with those treated with B/S (89/380, 23.4%, p<1x10^-6^, χ^2^-test). Further, the signatures of the B/S treated DR+/+ rats significantly overlapped with the transcripts temporally up-regulated by DR+/+ rats at 30, 40, 50 and 60 days of age (68/228, 29.8%, p<1x10^-6^, χ^2^-test). Given these relationships, pathway analysis was conducted on the 257 probe sets commonly regulated among the 4 data sets. Consistent with our identification of many IL-10/TGF-β–regulated transcripts within the longitudinal data set [15], IPA upstream regulator analysis identified TGF-β (46 transcripts, p = 4.0e-10) and IL-10 (20 transcripts, p = 1.5e-11) as candidate mediators underlying the intersecting data set. GO terms associated with this data set included negative regulation of kinase activity, regulation of inflammatory response and regulation of cytokine biosynthetic process (Fig 6E). Specifically, plasma of DR+/+ rats provided HCD or treated with B/S induced transcription of negative regulators of cell cycle progression, including the cyclin-dependent kinase inhibitor *Cdkn1a* (p21) that is induced by and plays a role in TGF-β–mediated growth inhibition [60], as well as negative regulators of cell signaling (*Dusp1*, *Dusp6*). Further, many of the annotated transcripts encode proteins that function to temper inflammation (Fig 6F). These include *Lgals3*, a glycan binding protein important in regulating inflammation [61], *Hopx*, a transcription cofactor important for regulatory T cell function [62], the inhibitory Fc gamma receptor 2b (*Fcgr2b*), and *Klf4*, a transcription factor that regulates IL-10 expression [63] and inhibits IL-1β expression [64]. Importantly, transcripts known to be regulated by IL-10 and/or TGF-β were also identified including *Mafb*, a transcription factor that blocks IRF3-dependent transcription in response to virus [65], and *Tgfbi* (TGF-β-induced), a secreted protein that modulates cell adhesion [66] and is involved in the induction of regulatory T-cells [67].

### HCD and B/S treatment normalizes the innate inflammatory state of DR+/+ rat islets

**Fig 7:**
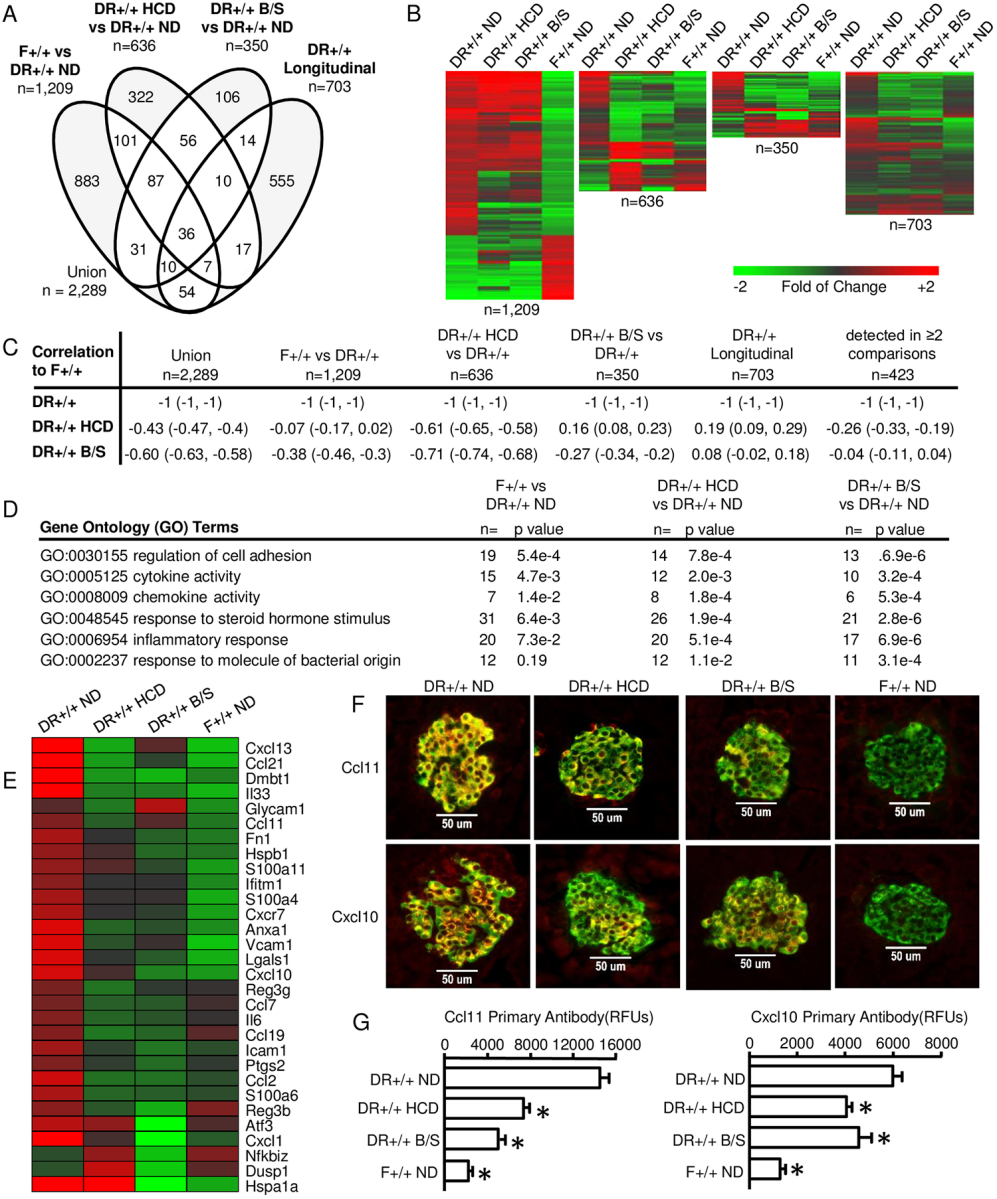
Partial normalization of the DR+/+ islet transcriptome by HCD and B/S treatment. Pools of islet RNA were prepared from an equal RNA contribution of individual islet RNAs prepared from 40 day old rats for each condition: DR+/+ ND (n = 12 rats), DR+/+ HCD (n = 7 rats), DR+/+ B/S (n = 12 rats), and F+/+ ND (n = 6 rats). These pools, analyzed in duplicate, were independent of those used in the longitudinal analysis (Fig 1). A. Venn diagram illustrating the relationship between probe sets regulated (to the thresholds of |log_2_ ratio| ≥ 0.5 (1.4-fold) and a FDR of ≤ 0.10) between DR+/+ ND versus F+/+ ND, DR+/+ ND versus DR+/+ HCD and DR+/+ ND versus DR+/+ B/S as well as those temporally regulated in DR+/+ islets, specifically classified into model profile 7 in DR+/+ but not regulated in F+/+ or F*lyp/lyp*. Probe sets identified in 2 or more analyses are highlighted in white. B. One-way hierarchical clustering (probe sets only) for the regulated probe sets identified between DR+/+ ND versus F+/+ ND (n = 1,209 probe sets), DR+/+ HCD (n = 636 probe sets) and DR+/+ B/S (n = 350 probe sets). The fourth heatmap illustrates expression levels of the 703 previously identified temporally up-regulated probe sets unique to DR+/+ rats (Fig 1B, green shaded). C. Pearson Correlation Coefficients between the data sets illustrated in panel A. Indicated in parentheses are, respectively, the lower and upper 0.99 confidence intervals. D. Pathway enrichment of terms significantly regulated in islets of F+/+ ND, DR+/+ HCD or DR+/+ B/S versus DR+/+ ND. E. Well-annotated genes related to inflammatory reponse, cell adhesion, and chemotaxis commonly detected in 2 or more comparisons represented in the Venn Diagram. F. Dual immunofluorescence staining was performed on pancreatic sections of 40 day old animals belonging to the four experimental conditions for identification of Ccl11 and Cxcl10 expressing β-cells. At least three animals were evaluated for each experimental condition, and 12–30 islets per animal were examined under each staining protocol. Top series: staining of sections with anti-insulin (FITC) and anti-Ccl11 (Alexa594). Bottom series: staining of sections with anti-insulin (FITC) and anti-Cxcl10 (Alexa594). Experimental conditions are indicated by column. Negative controls lacking primary Abs failed to show staining (not shown). All imaging was conducted under a 40x dry objective lens. G. Quantification of fluorescence intensity in islets of the four experimental conditions. * indicates p < 0.05 versus DR+/+ ND. One-way ANOVA with Dunnett’s post hoc test for multiple comparisons vs single control group.

HCD and B/S treatment partially normalized plasma-based measures of the DR+/+ innate inflammatory state; therefore, we hypothesized that this effect may extend to the islet. The longitudinal analysis of DR+/+ ND rats revealed an inflammatory islet transcriptional program by 40 days of age. Therefore islet transcriptomes of 40 day old F+/+ ND rats, DR+/+ HCD rats, and DR+/+ B/S rats were compared to those of age matched DR+/+ ND rats. A total of 2,289 regulated transcripts were identified when the 703 temporally up-regulated probe sets unique to DR+/+ rats (Fig 1B, green shaded) were included in the analysis. The relationships among these data sets are shown in Fig 7A (S5 Table). As reflected by the heat maps (Fig 7B) and Pearson’s correlation coefficients (Fig 7C), both HCD and B/S treatment partially normalized the DR+/+ islet transcriptome towards that of F+/+ ND rats. However, unlike that observed in the plasma induced transcription analysis, providing HCD elicited a larger effect in DR+/+ rat islets than did B/S treatment.

Reflecting this normalization, GO terms identified by comparison of the HCD and antibiotic treated DR+/+ rat islets to those of DR+/+ ND rat islets were reflective of activities related to PRR ligand exposure, chemokine/cytokine activity, regulation of cell adhesion and inflammatory response (Fig 7D). The islets of DR+/+ HCD and DR+/+ B/S included normalized expression levels of transcripts for chemokines and cytokines (*Cxcl1*, *Cxcl10*, *Ccl2*, *Ccl21*, *IL33*), adhesion molecules (*Vcam1*, *Icam1*), and other molecules involved in inflammatory processes (Fig 7E).

Pancreatic islets are comprised of α, β, γ, and δ cells which respectively produce glucagon, insulin, pancreatic polypeptide, and somatostatin and possess respective distributions of ~21, ~68, ~5, and ~6 percent. In order to colocalize chemokine expression to β-cells and confirm that protein expression levels of these mediators were consistent with those observed in the gene expression analyses, pancreata of 40 day old DR+/+ ND, DR+/+ HCD, DR+/+ B/S and F+/+ ND rats were subjected to dual immunofluorescence staining using anti-Ccl11 or anti-Cxcl10 Abs in combination with anti-insulin Abs (Fig 7F and 7G). Consistent with our previous histological studies localizing Ccl11 expression to β-cells of BB rats, expression of Cxcl10 was also localized to β-cells of DR+/+ ND rats. Furthermore expression of these chemokines was reduced in β-cells of DR+/+ B/S and DR+/+ HCD rats. Similar to our analysis of WF rats [28], which also lack T1D susceptibility, β-cells of F+/+ ND rats did not express detectable levels of these mediators. Consistent with the concept that β-cells are active participants in the autoimmune process, these analyses revealed that in a susceptible genetic background, diet and microbiota composition influence the immune recruiting potential at the islet level.

### Impact of HCD and B/S on diabetes incidence in lymphopenic DR*lyp/lyp* rats

**Fig 8:**
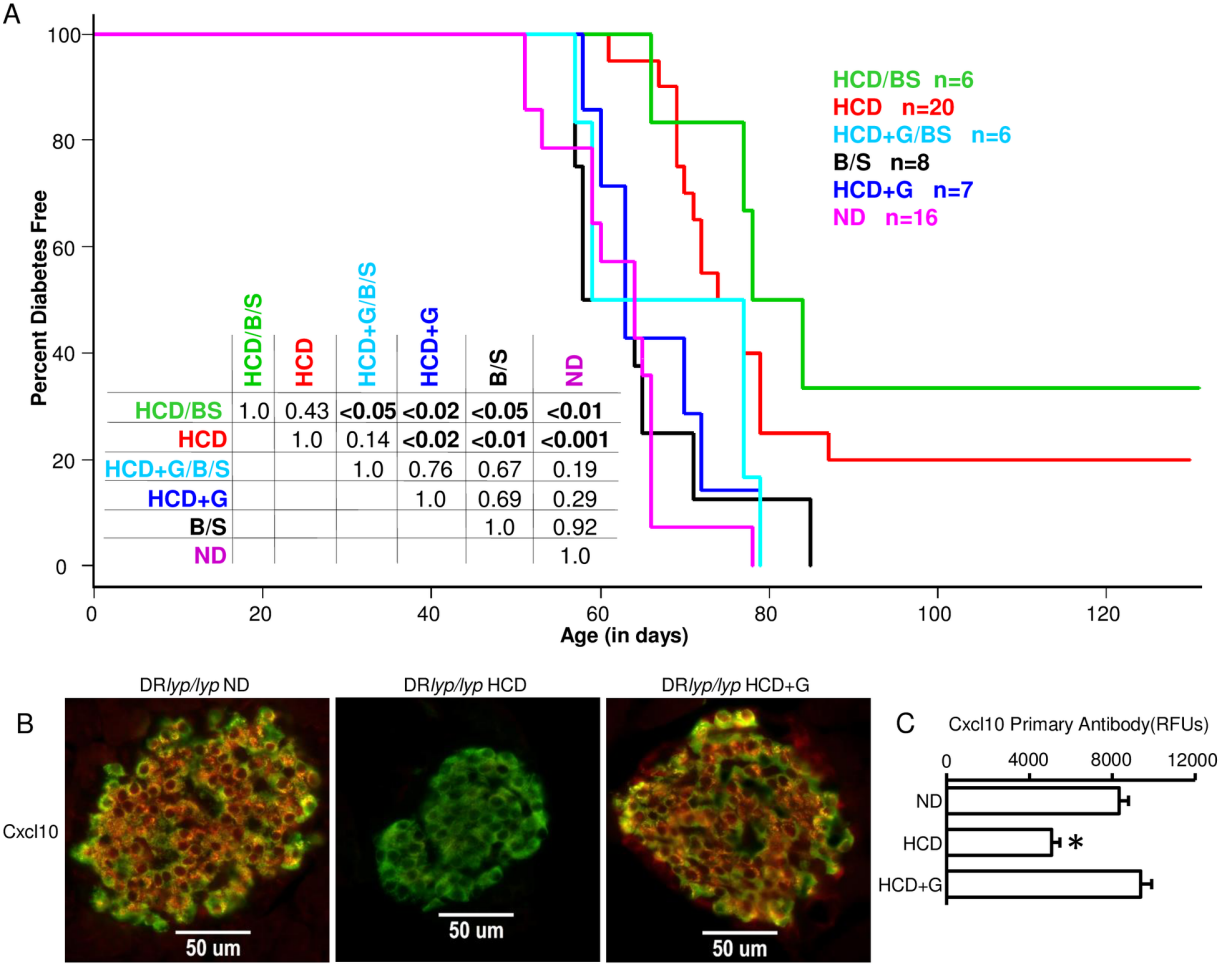
Impact of HCD and B/S on diabetes incidence in lymphopenic DR*lyp/lyp* rats. A. Survival plots of DR*lyp/lyp* rats provided ND, HCD, B/S, HCD+B/S, HCD+G, and HCD+G/B/S at weaning. T1D onset was defined as the first of two consecutive days with fasting blood glucose ≥ 250mg/dl. The median age of survival of the ND group was 62.5 days (n = 16, range 51–78 days), for HCD it was 84.6 days (n = 20, range 61–130 days), for B/S it was 63.1 days (n = 8, range 50–85 days), for HCD+B/S it was 94.2 days (n = 6, range 66–130 days), for HCD+G it was 66.4 days (n = 7, range 58–79 days), and for HCD+G/B/S it was 68 days (n = 6, range 57–79 days). The inset table shows the results of log rank tests conducted between each group. B. Dual immunofluorescence staining was performed on pancreatic sections of 40 day old animals belonging to the experimental conditions for identification of Cxcl10 expressing β-cells. Three animals were evaluated for each experimental condition, and 12–30 islets per animal were examined. Sections were stained with anti-insulin (FITC) and anti-Cxcl10 (Alexa594). Experimental conditions are indicated by column. Negative controls lacking primary Abs failed to show staining (not shown). All imaging was conducted under a 40x dry objective lens. C. Quantification of fluorescence intensity in islets of the experimental conditions. * indicates p < 0.05 versus DR+/+ ND. One-way ANOVA with Dunnett’s post hoc test for multiple comparisons vs single control group.

Consistent with our past studies [18, 28], 100% of DR*lyp/lyp* rats provided ND developed T1D by day 78, with a mean time to onset of 62.5+/-7.4 days (Fig 8A). Providing DR*lyp/lyp* rats HCD from weaning prevented T1D in 20% of rats and significantly delayed onset (mean time to onset 73.3+/-6.2 days). DR*lyp/lyp* rats provided ND and treated with B/S did not exhibit a delay in T1D progression (63.1+/-10.7 days). Providing DR*lyp/lyp* rats with HCD and treating them with B/S from weaning prevented T1D in 33% of the animals and delayed onset (mean time to onset 76.3+/-7.5 days). While the combination treatment protected a greater percentage of animals from diabetes (33.3% versus 20%), the difference was not significantly different from HCD alone with the number of animals tested. As expected, F*lyp/lyp* and F*+/+* rats provided ND did not develop diabetes.

Patrick et al., [22] reported that lymphopenic, diabetes prone BB rats provided HCD under germ-free conditions exhibited even greater protection from diabetes than those provided cereal diet under specific pathogen free conditions, suggesting biome-independent effects of HCD on diabetes progression. Further, the gluten associated amylase-trypsin inhibitors (ATIs) present in wheat have been shown capable of directly activating TLR4 on myeloid cells, promoting intestinal innate inflammation and elevated peripheral cytokine levels [68]. To investigate whether the absence of gluten contributed to the protective effects of HCD in DR*lyp/lyp* rats, a HCD containing 50% the gluten level of ND was prepared (HCD+G). Notably, when DR*lyp/lyp* weanlings were provided HCD+G, or provided HCD+G and treated with B/S, the protective effect of HCD was completely abrogated (Fig 8A). To determine whether the dietary gluten influenced chemokine expression in DR*lyp/lyp* β-cells, pancreata of 40 day old DR*lyp/lyp* rats provided HCD or HCD+G were subjected to dual immunofluorescence staining using anti-Cxcl10 Abs in combination with anti-insulin Abs. As shown in Fig 8B, the addition of gluten to HCD increased Cxcl10 expression in DR*lyp/lyp* β-cells, whether this is biome dependent or independent remains to be determined.

### TLR-stimulated cytokine production by DR+/+ and F+/+ PBMC

**Fig 9:**
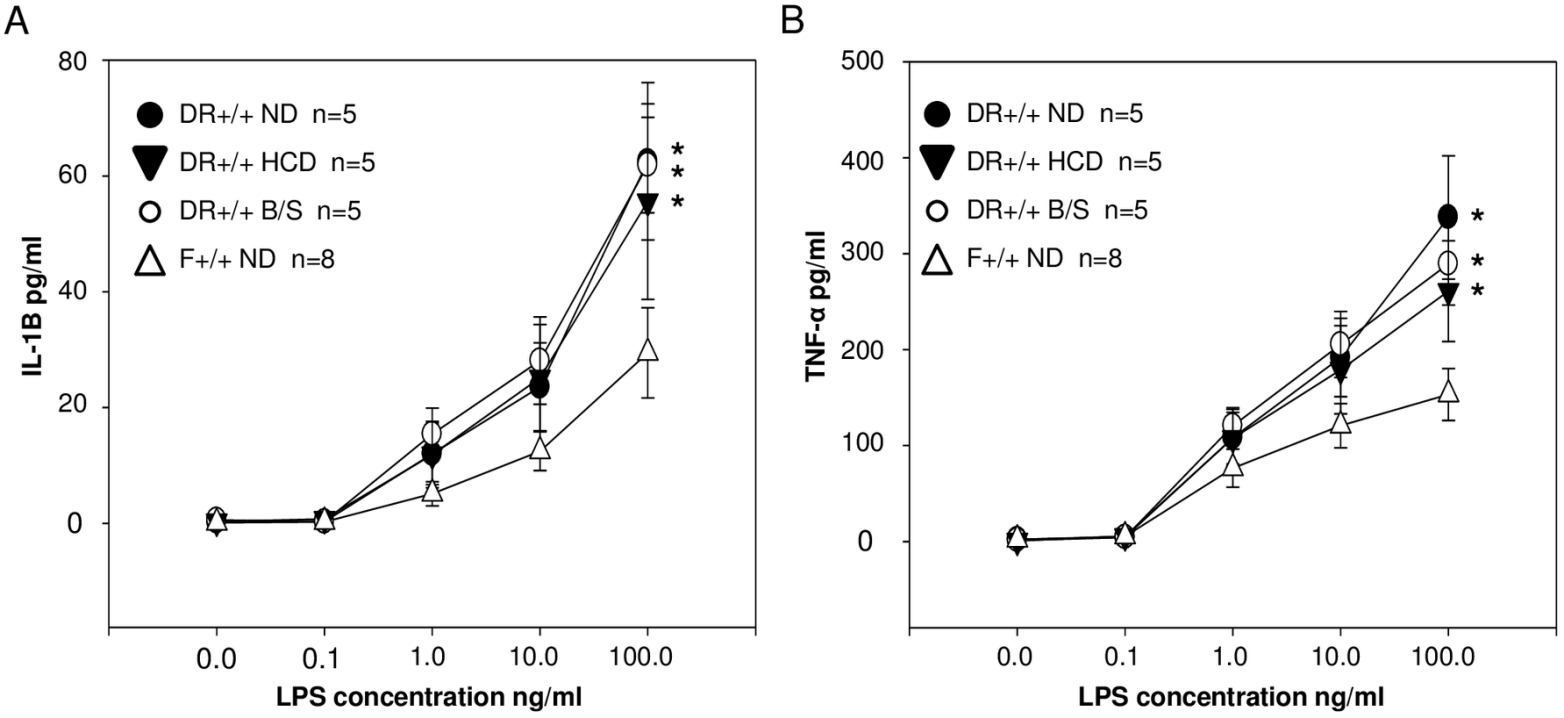
Dose response of DR+/+ ND, DR+/+ HCD, DR+/+ B/S and F+/+ PBMCs to LPS stimulated IL-1β (A) and TNF-α (B) secretion. LPS was added to PBMC cultures at the indicated concentrations. Each culture consisted of 5x10^5^ cells. After 24 hours of culture cytokine concentrations were measured in the conditioned medium by ELISA. The number of replicates experiments for each condition are indicated; flow cytometry showed that the percent of myleoid cells within isolated PBMC did not differ between DR+/+ ND and F+/+ ND rats. *ANOVA p<0.05 versus F+/+ ND.

When compared to PBMCs of unrelated healthy controls, PBMCs drawn from T1D probands and their unaffected first degree relatives exhibit hypersecretion of IL-1α and TNF-α upon stimulation with LPS [69]. We hypothesized that the innate inflammatory state associated with BB rat T1D susceptibility involves both an altered exposure to PRR ligands and an inherited hyper-responsiveness to PRR ligation. Therefore, PBMCs of 30 day old DR+/+ ND, DR+/+ HCD, DR+/+ B/S and F+/+ ND rats were treated for 24 hours with a range of LPS concentrations (0 to 100 ng/mL), after which IL-1β and TNF-α levels were measured in the conditioned medium (Fig 9). Stimulation with LPS resulted in significantly higher IL-1β and TNF-α response curves by DR+/+ ND PBMC compared to F+/+ ND PBMC. While the aforementioned studies show that inflammatory phenotypes in BB rats can be environmentally modulated *in vivo*, the responsiveness of PBMCs drawn from DR+/+ HCD rats or DR+/+ B/S rats were not different from DR+/+ ND rats, and were significantly higher than F+/+ ND rats, consistent with the concept that hyper-responsiveness to PRR ligation is genetically controlled.

## Discussion

We previously reported that BB rat β-cells express Ccl11 [28, 70]. Since healthy tissues typically do not express immunocyte recruiting chemokines, we have considered islet Ccl11 expression evidence of an underlying pathology associated with T1D susceptibility. To increase our understanding of T1D pathogenesis at the target tissue level, this study began by defining the transcriptional natural history of pancreatic islets in BB rats. This effort identified the expression of Ccl11 as a single aspect of a broad innate transcriptional program consistent with exposure to microbial antigens and NF-κB activation. In DR*lyp/lyp* rats, which are Treg deficient, this transcriptional program is associated with the islet recruitment of innate (eosinophils) and adaptive immunocytes (T-cells) and T1D progression [28, 70]. This inflammatory transcriptional program is also present in islets of DR+/+ rats; notably, protocols that deplete Treg cells induce diabetes in nonlymphopenic BB rats [71]. Conversely, adoptive transfer of Treg to young diabetes prone lymphopenic BB rats is protective [11, 14]. These observations, and our past studies [15, 18], support the existence of an innate inflammatory state in BB rats that is associated with diabetes susceptibility yet is independent of *Iddm2*, insulitis and T1D onset.

Our past blood based studies have identified a similar innate inflammatory state associated with human T1D susceptibility that is present in T1D patient and their healthy siblings [8–10]. The findings described here show that in the BB rat, this innate state extends to the β-cell level. Notably, long-term NF-κB activation in murine β-cells has been demonstrated to induce spontaneous immune-mediated diabetes [72]. Relevant to these observations, histological analyses have shown that β-cells of new onset T1D patients express CXCL10, while the islet-infiltrating lymphocytes express its receptor (CXCR3) [73]. Furthermore, transcriptomic analyses of whole pancreas and islets isolated from T1D patients has also revealed evidence of innate immune activity and NF-κB activation [74]. This was reflected by increased expression of chemokines (*CXCL1*, *CXCL10*, *CCL2*, *CCL3*, *CCL11*, *CCL19)*, immune receptors and bacterial response genes (*MUC1*, *REG3γ*, *CD14*, *TLR1*), adhesion molecules (*VCAM1*, *ICAM1*) and other transcripts that parallel our observations in DR+/+ islets. These gene expression patterns were present even in long-standing diabetes patients, suggesting that human insulitis and β-cell destruction may also involve chronic innate inflammatory processes that are independent of T1D progression itself [74].

Our findings suggest that BB rats possess elevated peripheral levels of PRR ligands and the islets experience elevated activation of PRRs. Intestinal barrier dysfunction can mediate increased exposure to microbes and their associated antigens, and increased intestinal permeability has been associated with T1D in humans [75–78] and rodent models [22, 41, 79, 80]. Importantly, exposure of β-cells to cytokines or LPS activates NF-κB, promotes proapoptotic responses and impairs the synthesis and release of insulin [81, 82]. Notably, we did not observe differences in oral glucose tolerance tests (OGTT) and proinsulin:C-peptide ratios between F+/+ and DR+/+ rats at ≤30 and ≥50 days of age, suggesting that the inflammatory state present in DR+/+ rats is not sufficient to impair glucose homeostasis or induce overt β-cell dysfunction.

Intestinal barrier function is influenced by the composition and activity of the gut microbiota. Further, PRR ligands such as LPS, derived from different lineages of microbes, can exhibit varying capacities to activate mammalian innate immune receptors [83]. Therefore we aimed to alter the composition of the intestinal microbiota and the nature of PRR ligand exposure by providing DR+/+ rats with HCD or treating them with B/S. Compared to DR+/+ ND rats, either strategy resulted in significant alterations to the microbiota. These changes encompassed increases in the Firmicutes:Bacteroidetes ratio, driven in part by greater relative abundances of *Lactobacillaceae* and reductions in *Helicobacteraceae*. These observations are consistent with associations of elevated Firmicutes:Bacteroidetes ratios and probiotic administration of *Lactobacilli* with diabetes protection (reviewed in [84]). Further, *Butyricimonas*, a butyrate producing genera, was enriched in the cecal community of DR+/+ HCD rats. Butyrate is a short-chain fatty acid that can be synthesized through utilization of the lactate released by genera such as *Lactobacillus* and *Bifidobacterium;* it is beneficial because it promotes Treg differentiation, is necessary for sufficient mucin synthesis, and enhances the intestinal barrier integrity by regulating tight junction assembly [85]. Further, reduced abundances of butyrate-producing bacteria have been reported in fecal samples of individuals possessing β-cell autoimmunity (reviewed in [84]). Overall, the ileal and cecal communities of DR+/+ HCD or DR+/+ B/S rats exhibited compositions consistent with a reduced proinflammatory bias compared to DR+/+ ND rats.

The ileal and cecal communities of DR+/+ ND and F+/+ ND rats were most similar, yet the peripheral inflammatory state was higher in DR+/+ rats, as measured by plasma cytokine levels (IL-1a, IL-4, IL-13, IL-18) and plasma induced transcription analysis. This result is consistent with the BB rat lineage possessing genetic variation that influences immune responsiveness and possibly increased intestinal permeability [86, 87] as is suggested by the measurement of higher HMGB1-independent plasma TLR4 reporter activity in DR+/+ rats. Previously, we reported that the susceptibility of DR+/+ rats to KRV triggered diabetes declines with age and is coincident with the temporal acquisition of an IL-10- and TGF-β-mediated immunoregulated state [15]. Here, the elevated plasma cytokine levels in DR+/+ ND rats were found to decline in an age-dependent manner between weaning and 30 days of age. While ELISA measurements of plasma cytokine levels in DR+/+ HCD or DR+/+ B/S rats were generally lower, plasma induced transcription assays more clearly revealed that either protocol significantly normalized the endogenous inflammatory state of DR+/+ rats towards that of F+/+ rats. This normalization was accompanied by a reduction in TLR4 reporter activity, further associating the inflammatory state in BB rats with PRR ligand exposure.

Unlike the decrease in peripheral inflammatory activity measured in DR+/+ ND rat plasma by ELISA and plasma induced transcription, a temporal increase in inflammatory gene expression was observed in BB rat islets. This may be a consequence of chronic elevated exposure to microbial antigens, which is supported by the elevated TLR4-reporter activity measured at both 30 and 60 days of age in DR+/+ ND rats compared to F+/+ ND rats. Consistent with this hypothesis, the inflammatory transcriptome expressed by DR+/+ ND rat islets at 40 days of age was partially normalized by HCD, and to a lesser extent by B/S treatment. Patrick et al. [22], reported that germ-free diabetic prone BB rats provided HCD exhibited greater T1D-free survival compared to those provided a cereal diet; further, the cereal fed rats exhibited increased T-cell insulitis, a Th1 cytokine bias, and deficiencies in anti-inflammatory M2 macrophages. These observations, suggest that independent of microbiota, HCD itself is protective, an effect possibly mediated by the absence of gluten and/or TLR4-activating ATI, as implicated by our studies of T1D incidence and β-cell chemokine expression in DR*lyp/lyp* rats. While the precise mechanism/s through which HCD elicits its protective effects remains incompletely defined, our analyses show that it significantly altered the gut microbiota, normalized innate immune activation at the islet level, and resulted in more rapid induction of immunoregulatory activity as measured through plasma induced transcription.

It is unclear why BB and LEW.1WR1 rats are susceptible to T1D, while other MHC-matched strains, such as WF, F+/+, and PVG.R8 rats, are not [16]. Overall increases in innate inflammatory activity may be mediated through the inheritance of potentiating genetic variants in BB rats, LEW.1WR1 rats [86–88], and human T1D families. In line with this hypothesis, PBMC of healthy siblings of T1D patients [69] and PBMC of DR+/+ rats are hyper-responsive to PRR ligand exposure. Notably, β-cells of both BB and LEW.1WR1 rats express Ccl11 (J.P. Mordes, E.P. Blankenhorn, and M.J. Hessner unpublished results) and T1D can be delayed/prevented in these spontaneous and viral triggered rat models by therapeutics that target innate immune processes, including the mast cell inhibitors cromolyn and salbutamol, IL-1RN, and salicylate, a potent inhibitor of NF-κB activity [7, 89]. Our working model of diabetes pathogenesis centers on the presence of an innate inflammatory state stemming from inheritance of immune potentiating genetic variants and altered PRR ligand exposure. Our human and rodent studies suggest that this state is normally counter regulated through endogenous age-dependent compensatory mechanisms [10, 15]. Here, alteration of the diet and microbiota reinforced this default protective regulatory response, suggesting that prevention of human T1D may be fostered by prebiotic or probiotic strategies that mitigate innate inflammation.

In NOD mice, alterations to the gut microbiota modulate cathelicidin-related antimicrobial peptide production in islets [90], demonstrating that diet and gut microbiota can directly shape the pancreatic inflammatory environment. To our knowledge, the results reported here are the first to link alterations in the diet and gut microbiota to a systemic innate inflammatory state that extends to the immunocyte recruiting potential of β-cells. The relationships between inherited susceptibility, endogenous counter regulatory mechanisms, and diet/microbiota remain incompletely defined, but are highly relevant as early probiotic supplementation has now been associated with a reduced the risk of islet autoimmunity in children at high genetic risk for T1D [91].

## Supporting information

S1 Table Composition of diets. (PDF)

S2 Table Longitudinal islet gene expression analysis. (PDF)

S3 Table Operational taxonomic unit (OTU) table. (PDF)

S4 Table Plasma induced transcription analysis. (PDF)

S5 Table Islet gene expression analysis of DR+/+ND, DR+/+HCD, DR+/+B/S, and F+/+ islets at 40 days of age. (PDF)
