# Supplementary material for: Modulation of the diet and gastrointestinal microbiota normalizes systemic inflammation and β-cell chemokine expression associated with autoimmune diabetes susceptibility
Source: PLoS One. 2018 Jan 2;13(1):e0190351. doi: 10.1371/journal.pone.0190351 (PMC5749787; doi:10.1371/journal.pone.0190351)
Supplement: S1 Table — (PDF) [file pone.0190351.s001.pdf]

S1 Table. Composition of Diets

	HCD	HCD +gluten	Normal Chow
	%	%	%
Protein source:			
hydrolyzed casein (HCD)	100	50	
wheat gluten		50	
corn, wheat, soybean, fish, oats, alfalfa			100
Protein	20	10	24.6
		12.1	
Cornstarch	39.8	37.6	29.4
Sucrose	10	10	1.2
Other sugars	13.2	13.2	19.4
fats	7	7	6.4
fiber; primarily cellulose	5	5	4.2
mineral	3.5	3.5	6.1
vitamin	1	1	<1
choline bitartrate	0.25	0.25	
choline chloride			0.22
total kcal/kg	3747	3754	4170
