# Supplementary material for: Modulation of the diet and gastrointestinal microbiota normalizes systemic inflammation and β-cell chemokine expression associated with autoimmune diabetes susceptibility
Source: PLoS One. 2018 Jan 2;13(1):e0190351. doi: 10.1371/journal.pone.0190351 (PMC5749787; doi:10.1371/journal.pone.0190351)
Supplement: S2 Table — (PDF) [file pone.0190351.s002.pdf]

S2 Table. Longitudinal Islet Gene Expression Analysis [1]

Affymetrix Probeset ID	Gene Symbol	Gene Name	unique BB on P7 n=822	Figure 1B P7 Venn n=1526	DRlyp/lyp STEM P7 n=712	DR+/+ STEM P7 n=936	Flyp/lyp STEM P7 n=520	F+/+ STEM P7 n=465	Figure 1B P0 Venn n=1071	DRlyp/lyp STEM P0 n=442	DR+/+ STEM P0 n=437	Flyp/lyp STEM P0 n=643	F+/+ STEM P0 n=632	Fig1D n=25	Mean log2 intensity DRlyp/lyp 20	Mean log2 intensity DRlyp/lyp 30	Mean log2 intensity DRlyp/lyp 40	Mean log2 intensity DRlyp/lyp 50	Mean log2 intensity DR+/+ 20	Mean log2 intensity DR+/+ 30	Mean log2 intensity DR+/+ 40	Mean log2 intensity DR+/+ 50	Mean log2 intensity Flyp/lyp 20	Mean log2 intensity Flyp/lyp 30	Mean log2 intensity Flyp/lyp 40	Mean log2 intensity Flyp/lyp 50	Mean log2 intensity Flyp20	Mean log2 intensity Flyp30	Mean log2 intensity Flyp40	Mean log2 intensity Flyp50	Mean log2 intensity F+/+ 20	Mean log2 intensity F+/+ 30	Mean log2 intensity F+/+ 40	Mean log2 intensity F+/+ 50
1367594_at	Bgn	biglycan	0	BBnF+/+_42	1	1	0	1	0	0	0	0	0	0	6.86	7.20	7.18	7.42	6.99	7.17	7.33	7.56	6.65	6.73	6.84	7.12	6.79	6.89	7.09	7.30	6.72	7.06	7.25	7.27
1367661_at	S100a6	S100 calcium binding protein A6	0	BBnF+/+_42	1	1	0	1	0	0	0	0	0	0	6.92	7.35	7.57	7.98	7.07	7.18	8.03	8.24	7.09	7.41	7.09	7.63	6.84	7.11	7.57	7.76	6.81	7.01	7.33	7.19
1367845_at	Nefm	neurofilament, medium polypeptide	0	BBnF+/+_42	1	1	0	1	0	0	0	0	0	0	5.39	5.55	5.76	6.00	5.28	5.38	5.94	5.86	5.15	6.15	5.79	6.08	5.00	5.42	5.63	5.89	5.14	5.37	5.63	5.95
1368007_at	Dmbt1 /// LO	deleted in malignant brain tumors 1 /// deleted in malignant brain tumors 1 protein-like	0	BBnF+/+_42	1	1	0	1	0	0	0	0	0	0	4.70	4.70	5.23	6.54	4.53	4.83	7.63	6.08	4.52	4.65	4.69	4.94	4.45	4.47	4.75	5.50	4.53	4.40	4.94	4.83
1369728_at	LOC680097	similar to germinal histone H4 gene	0	BBnF+/+_42	1	1	0	1	0	0	0	0	0	0	4.11	4.48	4.53	4.91	4.21	4.01	5.74	4.83	4.13	4.30	4.61	4.59	4.04	4.61	4.52	4.49	3.98	4.32	4.42	4.57
1370041_at	Stmn2	stathmin-like 2	0	BBnF+/+_42	1	1	0	1	0	0	0	0	0	0	5.41	5.67	5.90	6.31	5.60	5.51	6.12	6.27	6.03	7.46	6.48	6.84	6.32	6.58	6.52	6.48	5.96	5.91	6.29	6.63
1370042_at	Stmn2	stathmin-like 2	0	BBnF+/+_42	1	1	0	1	0	0	0	0	0	0	6.10	6.48	6.74	6.87	6.14	6.40	6.64	6.73	6.65	7.71	7.14	7.36	6.58	7.16	7.13	7.21	6.63	6.71	6.83	7.08
1370113_at	Birc3	baculoviral IAP repeat-containing 3	0	BBnF+/+_42	1	1	0	1	0	0	0	0	0	0	5.13	5.21	5.36	6.56	5.30	5.22	5.39	6.78	5.12	5.19	5.32	5.36	5.17	5.11	5.35	5.56	4.75	5.18	5.21	5.28
1370449_at	P2ry14	purinergic receptor P2Y, G-protein coupled, 14	0	BBnF+/+_42	1	1	0	1	0	0	0	0	0	0	3.92	4.25	4.50	4.81	3.99	4.32	4.40	4.73	3.77	3.64	4.05	3.99	3.88	4.36	4.13	4.29	3.61	3.95	3.92	4.20
1370832_at	Ccl4	chemokine (C-C motif) ligand 4	0	BBnF+/+_42	1	1	0	1	0	0	0	0	0	0	5.08	5.18	5.29	5.86	5.00	5.03	5.55	5.81	5.03	5.11	5.24	5.41	4.96	5.22	5.31	5.56	4.97	5.20	5.42	5.65
1370967_at	LOC680329	immunoglobulin lambda-like polypeptide 5-like	0	BBnF+/+_42	1	1	0	1	0	0	0	0	0	0	2.96	3.64	3.58	9.51	2.83	3.19	7.24	10.84	4.22	3.19	4.54	5.10	3.19	3.02	5.16	7.27	2.86	3.03	3.53	4.82
1370972_x_at	RT1-CE5	RT1 class I, locus CE5	0	BBnF+/+_42	1	1	0	1	0	0	0	0	0	0	5.93	6.09	6.22	6.68	5.87	6.17	6.28	6.80	6.01	6.21	6.28	6.55	7.20	7.07	7.14	7.28	6.02	6.01	6.38	6.46
1371079_at	Fcgr2b	Fc fragment of IgG, low affinity IIb, receptor (CD32)	0	BBnF+/+_42	1	1	0	1	0	0	0	0	0	0	2.85	3.14	3.09	6.49	2.92	3.34	3.45	7.34	3.34	3.19	3.66	3.66	2.83	3.11	3.61	4.57	3.04	3.33	3.36	3.61
1371152_a_at	Oas1a	2'-5' oligoadenylate synthetase 1A	0	BBnF+/+_42	1	1	0	1	0	0	0	0	0	0	3.92	3.88	4.21	4.82	3.69	3.83	4.30	4.18	3.91	4.35	4.06	4.37	4.34	4.23	4.51	4.77	3.98	3.82	4.25	4.51
1371209_at	RT1-CE5	RT1 class I, locus CE5	0	BBnF+/+_42	1	1	0	1	0	0	0	0	0	0	2.26	2.17	2.54	4.02	2.16	2.13	2.43	4.04	2.68	3.38	3.51	4.08	1.99	2.06	1.97	1.94	2.53	2.97	3.57	4.14
1372404_at	Rac2	ras-related C3 botulinum toxin substrate 2 (rho family, small GTP binding protein Rac2)	0	BBnF+/+_42	1	1	0	1	0	0	0	0	0	0	5.48	5.54	5.71	8.70	5.50	5.59	6.88	9.94	5.75	5.40	5.94	6.04	5.48	5.51	6.16	7.20	5.31	5.72	5.75	5.96
1372808_at	Mthfd2 /// R	methylenetetrahydrofolate dehydrogenase (NADP+ dependent) 2, methenyltetrahydrofolate	0	BBnF+/+_42	1	1	0	1	0	0	0	0	0	0	6.94	7.70	7.57	7.68	6.93	7.76	7.74	7.64	7.17	7.88	7.88	7.76	7.26	7.96	7.99	7.85	7.39	7.97	8.09	8.06
1373085_at	Cbr3	carbonyl reductase 3	0	BBnF+/+_42	1	1	0	1	0	0	0	0	0	0	4.04	4.57	4.65	4.97	4.11	4.45	4.84	5.09	4.18	4.37	4.42	4.58	4.26	4.34	4.63	4.85	4.00	4.33	4.45	4.51
1373226_at	Tor4a	torsin family 4, member A	0	BBnF+/+_42	1	1	0	1	0	0	0	0	0	0	5.44	5.78	5.96	5.96	5.77	5.80	5.84	6.28	5.84	6.06	6.09	6.20	5.93	5.98	6.35	6.36	5.73	6.10	6.30	6.38
1373992_at	MGC108823	similar to interferon-inducible GTPase	0	BBnF+/+_42	1	1	0	1	0	0	0	0	0	0	2.41	2.51	2.62	7.80	2.33	2.23	2.57	4.83	2.26	2.13	2.28	2.28	2.38	2.18	2.42	3.41	2.07	2.19	2.20	2.59
1377637_at	B3gnt5	UDP-GlcNAc:betaGal beta-1,3-N-acetylglucosaminyltransferase 5	0	BBnF+/+_42	1	1	0	1	0	0	0	0	0	0	2.55	2.49	2.78	6.32	2.43	2.58	3.46	7.49	3.10	2.55	3.08	2.98	2.52	2.35	2.88	4.39	2.39	2.69	2.71	3.07
1378633_at	Lpxn	leupaxin	0	BBnF+/+_42	1	1	0	1	0	0	0	0	0	0	4.62	5.04	5.06	6.55	4.80	5.00	5.43	7.60	4.92	5.05	5.17	5.35	4.81	4.97	5.40	5.89	4.79	4.83	5.15	5.30
1380366_at	Igh-6 /// LOC	immunoglobulin heavy chain 6 /// similar to single chain Fv antibody fragment scFv 7-10A	0	BBnF+/+_42	1	1	0	1	0	0	0	0	0	0	3.23	3.27	3.56	8.32	3.08	3.01	5.00	9.46	4.17	3.23	4.38	3.95	3.22	3.20	3.47	5.40	2.96	3.25	3.29	4.20
1381556_at	Ddx60	DEAD (Asp-Glu-Ala-Asp) box polypeptide 60	0	BBnF+/+_42	1	1	0	1	0	0	0	0	0	0	2.75	2.94	3.29	6.10	2.69	2.83	3.05	4.28	2.90	3.35	3.19	3.52	3.47	3.25	3.76	4.96	2.83	2.90	3.59	3.83
1382184_at	Faim3	Fas apoptotic inhibitory molecule 3	0	BBnF+/+_42	1	1	0	1	0	0	0	0	0	0	4.45	4.39	4.36	7.67	4.36	4.50	5.43	8.79	4.59	3.95	4.29	4.59	4.15	4.32	4.34	5.22	3.91	4.13	4.17	4.43
1382314_at	Isg15	ISG15 ubiquitin-like modifier	0	BBnF+/+_42	1	1	0	1	0	0	0	0	0	0	5.20	5.37	5.59	8.28	5.51	5.53	5.61	6.16	5.51	5.76	5.61	6.00	5.50	5.72	5.95	6.40	5.35	5.58	5.89	6.14
1384787_at	Cd3g	CD3 molecule, gamma	0	BBnF+/+_42	1	1	0	1	0	0	0	0	0	0	3.42	3.38	3.41	5.36	3.42	3.58	4.56	6.83	3.63	3.49	3.82	3.85	3.55	3.58	3.75	4.52	3.38	3.76	3.69	3.99
1387022_at	Aldh1a1	aldehyde dehydrogenase 1 family, member A1	0	BBnF+/+_42	1	1	0	1	0	0	0	0	0	0	6.00	6.60	6.77	6.72	6.45	6.65	6.75	7.08	5.66	5.48	5.52	6.17	5.89	6.26	6.17	6.18	5.69	6.03	6.16	6.39
1387025_at	Dync1i1	dynein cytoplasmic 1 intermediate chain 1	0	BBnF+/+_42	1	1	0	1	0	0	0	0	0	0	5.13	5.38	5.54	5.66	5.11	5.48	5.63	5.52	5.15	5.86	5.59	5.62	5.04	5.41	5.49	5.65	5.13	5.39	5.58	5.70
1387902_a_at	LOC500183	similar to NGF-binding Ig light chain	0	BBnF+/+_42	1	1	0	1	0	0	0	0	0	0	3.31	4.28	5.49	10.59	3.35	4.13	8.39	11.75	5.83	4.17	6.25	6.16	4.67	4.73	6.39	8.66	3.23	5.16	5.23	6.35
1388166_at	Igh-6	immunoglobulin heavy chain 6	0	BBnF+/+_42	1	1	0	1	0	0	0	0	0	0	3.62	3.80	3.93	7.66	4.01	3.96	5.54	9.04	4.73	3.65	4.64	4.33	3.80	3.64	4.11	5.27	3.79	4.16	3.87	4.68
1388254_a_at	RT1-CE5	RT1 class I, locus CE5	0	BBnF+/+_42	1	1	0	1	0	0	0	0	0	0	2.42	2.25	2.33	2.32	2.29	2.38	2.34	2.42	2.36	2.32	2.46	2.46	2.46	2.36	2.39	2.43	2.32	2.33	2.45	2.38
1388255_x_at	RT1-CE5	RT1 class I, locus CE5	0	BBnF+/+_42	1	1	0	1	0	0	0	0	0	0	2.74	2.81	3.02	3.57	2.62	2.66	3.06	3.57	3.24	3.19	3.38	3.68	2.71	2.86	2.47	2.73	2.88	3.33	3.41	3.58
1389653_at	Pcdhb9	protocadherin beta 9	0	BBnF+/+_42	1	1	0	1	0	0	0	0	0	0	3.63	3.83	4.35	4.52	3.78	3.74	4.19	4.60	3.83	3.87	3.97	4.29	3.77	3.94	4.00	4.10	3.70	3.58	3.90	4.18
1390226_at	Tmem173	transmembrane protein 173	0	BBnF+/+_42	1	1	0	1	0	0	0	0	0	0	5.69	6.08	6.23	6.66	5.82	6.07	6.33	6.87	6.02	6.05	6.16	6.31	5.99	6.07	6.38	6.33	5.86	6.05	6.36	6.23
1390687_at	Plek	pleckstrin	0	BBnF+/+_42	1	1	0	1	0	0	0	0	0	0	4.35	4.47	4.68	7.15	4.44	4.56	5.32	8.11	4.53	4.45	4.83	4.86	4.45	4.54	5.41	6.14	4.24	4.71	4.73	4.81
1391446_at	Ms4a1	membrane-spanning 4-domains, subfamily A, member 1	0	BBnF+/+_42	1	1	0	1	0	0	0	0	0	0	3.09	3.29	3.07	7.63	3.03	3.15	4.92	9.20	3.84	3.11	4.51	3.79	3.02	3.19	4.20	5.92	2.89	3.26	3.37	4.25
1391637_at	Arhgap15	Rho GTPase activating protein 15	0	BBnF+/+_42	1	1	0	1	0	0	0	0	0	0	2.99	3.45	3.43	4.78	3.27	3.32	3.73	5.85	3.32	3.36	3.35	3.56	3.24	3.34	3.71	4.13	2.93	3.38	3.52	3.67
1394122_at	Rhoh	ras homolog family member H	0	BBnF+/+_42	1	1	0	1	0	0	0	0	0	0	4.49	4.89	4.93	6.49	4.72	4.89	5.30	7.89	4.81	4.76	4.93	4.96	4.89	4.84	4.95	5.42	4.53	4.91	5.00	5.14
1398245_at	Sncg	synuclein, gamma (breast cancer-specific protein 1)	0	BBnF+/+_42	1	1	0	1	0	0	0	0	0	0	5.73	6.20	6.81	6.98	5.63	6.29	7.18	7.45	5.24	7.40	6.00	6.55	5.16	6.11	6.38	6.47	5.44	5.80	6.11	6.48
1398378_at	Gstk1	glutathione S-transferase kappa 1	0	BBnF+/+_42	1	1	0	1	0	0	0	0	0	0	6.80	7.14	7.36	7.28	6.76	7.08	7.28	7.27	6.73	6.98	7.03	7.15	6.70	7.27	7.15	7.14	6.60	6.89	7.02	7.12
1398390_at	Cxcl13	chemokine (C-X-C motif) ligand 13	0	BBnF+/+_42	1	1	0	1	0	0	0	0	0	0	2.92	2.68	2.86	8.64	2.59	2.76	3.81	10.02	4.14	3.09	2.84	3.86	2.78	2.69	3.28	5.13	2.83	2.78	2.94	3.97
1367570_at	Tagln	transgelin	0	BBnFlyp/lyp_39	1	1	1	0	0	0	0	0	0	0	6.65	7.10	7.26	7.87	6.72	6.75	7.71	8.56	5.91	5.64	5.93	6.59	5.75	5.84	5.99	6.13	6.16	6.01	5.87	6.24
1367631_at	Ctgf	connective tissue growth factor	0	BBnFlyp/lyp_39	1	1	1	0	0	0	0	0	0	0	7.70	8.24	8.42	8.51	7.72	8.04	8.49	8.85	7.82	8.24	8.33	8.61	8.04	8.45	8.57	8.67	7.95	8.62	8.91	8.54
1368758_a_at	Galr2 /// LOC	galanin receptor 2 /// galanin receptor type 2-like	0	BBnFlyp/lyp_39	1	1	1	0	0	0	0	0	0	0	5.24	5.79	5.96	5.95	5.33	5.86	6.06	5.84	5.46	5.84	5.86	6.12	5.05	5.88	5.96	6.00	5.24	5.65	5.81	5.61
1369867_at	St8sia1	ST8 alpha-N-acetyl-neuraminide alpha-2,8-sialyltransferase 1	0	BBnFlyp/lyp_39	1	1	1	0	0	0	0	0	0	0	5.40	5.72	5.98	6.13	5.45	5.47	6.00	5.78	5.38	6.13	6.10	6.12	5.20	5.82	5.81	6.08	5.49	5.93	5.97	5.79
1369983_at	Ccl5	chemokine (C-C motif) ligand 5	0	BBnFlyp/lyp_39	1	1	1	0	0	0	0	0	0	0	4.77	4.98	5.18	6.88	4.78	4.75	5.08	5.67	4.88	5.00	5.58	5.40	5.49	5.27	5.55	6.40	4.91	5.08	5.19	5.32
1370056_at	LOC1009111	lymphocyte antigen 6B-like /// lymphocyte antigen 6B-like /// Ly6-C antigen	0	BBnFlyp/lyp_39	1	1	1	0	0	0	0	0	0	0	5.87	5.76	6.11	7.09	5.76	5.75	6.15	7.41	5.73	5.95	6.00	6.24	5.90	6.02	6.10	6.41	5.70	5.96	5.95	6.06
1370154_at	Lyz2	lysozyme 2	0	BBnFlyp/lyp_39	1	1	1	0	0	0	0	0	0	0	8.81	9.06	9.20	11.16	8.77	8.97	9.45	11.63	8.64	8.89	9.02	9.17	8.69	8.75	10.06	10.89	8.38	8.81	9.03	8.66
1370342_at	Kcnk2	potassium channel, subfamily K, member 2	0	BBnFlyp/lyp_39	1	1	1	0	0	0	0	0	0	0	4.70	5.25	5.48	5.71	4.83	4.95	5.29	5.78	4.50	4.98	5.14	5.81	5.17	4.92	5.16	5.64	4.91	5.02	5.27	5.54
1370821_at	Tpmt	thiopurine S-methyltransferase	0	BBnFlyp/lyp_39	1	1	1	0	0	0	0	0	0	0	5.13	5.24	5.25	5.74	5.28	5.12	5.49	6.88	5.17	5.45	5.53	5.67	5.27	5.42	5.52	5.68	5.27	5.56	5.49	5.55
1370882_at	LOC1009096	class II histocompatibility antigen, M beta 1 chain-like /// RT1 class II, locus DMb	0	BBnFlyp/lyp_39	1	1	1	0	0	0	0	0	0	0	5.45	6.05	6.07	8.05	5.64	5.99	6.32	8.50	5.53	5.87	6.11	6.22	5.65	6.04	6.46	6.81	5.55	6.02	6.15	5.87
1370883_at	RT1-Da	RT1 class II, locus Da	0	BBnFlyp/lyp_39	1	1	1	0	0	0	0	0	0	0	5.86	6.98	7.17	9.21	5.99	6.86	7.38	9.73	6.40	6.74	7.18	7.09	6.65	6.91	7.81	8.43	6.19	6.95	7.06	6.76
1370892_at	C4a /// C4b /	complement component 4A (Rodgers blood group) /// complement component 4B (Chido blo	0	BBnFlyp/lyp_39	1	1	1	0	0	0	0	0	0	0	3.95	4.13	4.18	6.07	3.98	4.26	4.64	5.87	4.07	4.25	4.43	4.72	4.48	4.31	4.95	5.94	4.18	4.21	4.54	4.59
1370894_at	Cldn7	claudin 7	0	BBnFlyp/lyp_39	1	1	1	0	0	0	0	0	0	0	7.26	7.67	8.10	7.87	7.25	7.61	8.12	8.06	6.62	7.47	7.39	7.78	6.40	7.51	7.37	8.25	6.90	6.97	7.25	7.30
1371065_at	LOC688090 /	similar to RT1 class II histocompatibility antigen, B-1 beta chain precursor (RT1.B-beta(1)) ///	0	BBnFlyp/lyp_39	1	1	1	0	0	0	0	0	0	0	5.44	6.69	6.95	9.23	5.53	6.36	7.18	9.67	5.97	6.46	6.84	6.89	3.99	3.74	3.87	3.83	5.84	6.59	6.80	6.43
1373032_at	Mustn1	musculoskeletal, embryonic nuclear protein 1	0	BBnFlyp/lyp_39	1	1	1	0	0	0	0	0	0	0	6.13	6.57	6.70	6.92	5.98	6.38	6.91	7.50	5.47	5.80	5.81	6.07	5.67	5.85	5.72	5.78	5.74	5.69	5.79	5.91
1373504_at	Glipr1	GLI pathogenesis-related 1	0	BBnFlyp/lyp_39	1	1	1	0	0	0	0	0	0	0	5.44	5.68	5.85	6.87	5.24	5.45	5.89	7.74	5.21	5.63	5.60	5.72	5.32	5.62	5.92	6.32	5.52	5.46	5.60	5.53
1373575_at	Fcer1g	Fc fragment of IgE, high affinity I, receptor for; gamma polypeptide	0	BBnFlyp/lyp_39	1	1	1	0	0	0	0	0	0	0	6.15	6.43	6.71	8.18	6.01	6.49	6.57	7.54	6.01	6.44	6.60	6.66	6.21	6.48	6.78	7.18	5.99	6.52	6.67	6.40
1373592_at	Serpinb9	serpin peptidase inhibitor, clade B (ovalbumin), member 9	0	BBnFlyp/lyp_39	1	1	1	0	0	0	0	0	0	0	6.82	6.91	7.00	7.59	6.78	6.88	7.09	7.60	6.55	6.78	6.83	7.13	6.65	6.83	7.08	7.23	6.66	6.75	6.87	7.08
1373975_at	Inmt	indolethylamine N-methyltransferase	0	BBnFlyp/lyp_39	1	1	1	0	0	0	0	0	0	0	5.14	5.96	5.83	5.85	5.06	5.89	5.95	6.02	5.59	6.50	6.47	6.44	5.25	6.64	6.74	6.73	5.55	6.98	7.20	6.81
1375001_at	Cytl1	cytokine like 1	0	BBnFlyp/lyp_39	1	1	1	0	0	0	0	0	0	0	5.40	5.89	6.28	6.57	5.40	5.69	6.80	7.26	4.69	4.67	5.03	5.50	4.97	4.80	4.68	5.04	5.06	5.02	4.52	5.00
1375944_at	Acss2	acyl-CoA synthetase short-chain family member 2	0	BBnFlyp/lyp_39	1	1	1	0	0	0	0	0	0	0	7.17	7.29	7.61	7.69	7.06	7.31	7.51	7.65	7.32	7.59	7.69	7.85	7.16	7.51	7.67	7.77	7.26	7.48	7.67	7.73
1376652_at	C1qa	complement component 1, q subcomponent, A chain	0	BBnFlyp/lyp_39	1	1	1	0	0	0	0	0	0	0	6.14	6.43	6.59	7.89	6.21	6.38	6.58	7.79	6.06	6.42	6.71	6.80	6.13	6.27	6.79	7.18	6.11	6.42	6.58	6.40
1379692_at	Cd22	CD22 molecule	0	BBnFlyp/lyp_39	1	1	1	0	0	0	0	0	0	0	4.71	4.75	4.55	5.78	4.69	4.92	5.12	6.38	4.62	4.60	4.92	5.16	4.57	4.66	4.87	5.17	4.64	4.88	4.54	4.90
1379766_at	---	---	0	BBnFlyp/lyp_39	1	1	1	0	0	0	0	0	0	0	3.17	3.28	3.47	5.72	2.99	3.23	3.72	6.17	2.98	3.13	3.29	3.52	3.15	3.28	3.79	4.41	3.10	3.22	3.21	3.27
1381441_at	LOC687813	similar to Tnf receptor-associated factor 1	0	BBnFlyp/lyp_39	1	1	1	0	0	0	0	0	0	0	3.59	3.43	3.69	4.19	3.56	3.47	3.79	4.10	3.70	3.69	3.76	4.27	4.31	4.05	4.09	4.11	3.95	3.65	3.88	3.94
1382692_at	Clec7a	C-type lectin domain family 7, member A	0	BBnFlyp/lyp_39	1	1	1	0	0	0	0	0	0	0	4.39	4.86	5.12	5.74	4.36	4.82	5.03	5.24	4.31	4.77	5.12	5.36	4.52	4.87	5.30	5.36	4.53	4.96	5.34	4.90
1382723_at	Kcnk2	potassium channel, subfamily K, member 2	0	BBnFlyp/lyp_39	1	1	1	0	0	0	0	0	0	0	5.54	5.54	5.42	5.55	5.43	5.50	5.65	5.58	5.50	5.50	5.50	5.75	5.48	5.62	5.55	5.64	5.55	5.52	5.61	5.60
1383708_at	Itgbl1	integrin, beta-like 1	0	BBnFlyp/lyp_39	1	1	1	0	0	0	0	0	0	0	3.87	4.48	4.53	4.48	3.99	4.19	4.52	4.69	3.30	3.44	3.72	3.97	3.37	3.40	4.03	3.97	3.60	3.64	3.94	3.89
1384667_x_at	Galr2 /// LOC	galanin receptor 2 /// galanin receptor type 2-like	0	BBnFlyp/lyp_39	1	1	1	0	0	0	0	0	0	0	4.10	4.70	5.31	4.86	4.25	4.68	4.78	4.84	4.35	4.95	4.78	4.84	4.20	4.68	4.83	4.92	4.04	4.30	4.61	4.33
1387687_at	Igsf6	immunoglobulin superfamily, member 6	0	BBnFlyp/lyp_39	1	1	1	0	0	0	0	0	0	0	2.73	2.77	2.83	3.39	2.68	2.83	2.82	3.69	2.61	2.82	2.89	3.16	2.80	2.81	3.00	3.38	2.86	2.90	3.01	2.93
1388272_at	IGH-1A	---	0	BBnFlyp/lyp_39	1	1	1	0	0	0	0	0	0	0	3.05	3.27	3.46	5.62	3.11	3.26	5.31	8.45	2.93	3.35	3.37	4.08	2.94	3.52	3.27	5.58	3.10	3.17	3.31	3.48
1388985_at	---	---	0	BBnFlyp/lyp_39	1	1	1	0	0	0	0	0	0	0	6.69	6.60	6.79	7.19	6.77	6.62	6.89	7.48	6.38	6.47	6.52	6.90	6.17	6.63	6.61	6.98	6.42	6.53	6.73	6.78
1389553_at	Clec4a3	C-type lectin domain family 4, member A3	0	BBnFlyp/lyp_39	1	1	1	0	0	0	0	0	0	0	4.11	4.61	4.69	6.36	4.06	4.50	4.66	5.57	4.01	4.39	4.55	4.81	4.16	4.22	4.88	5.20	4.23	4.26	4.73	4.35
1389873_at	Pycard	PYD and CARD domain containing	0	BBnFlyp/lyp_39	1	1	1	0	0	0	0	0	0	0	6.38	6.86	7.00	7.63	6.49	6.67	6.91	7.82	6.83	7.02	7.19	7.60	7.23	6.95	7.23	7.52	7.08	7.06	7.30	7.35
1390510_at	LOC1009125	membrane-spanning 4-domains subfamily A member 6B-like /// membrane-spanning 4-doma	0	BBnFlyp/lyp_39	1	1	1	0	0	0	0	0	0	0	4.71	5.01	4.99	6.57	5.06	4.75	5.90	7.22	4.37	4.55	4.92	4.74	4.57	4.55	5.19	5.99	4.54	4.67	4.84	4.71
1391684_at	Tmem14a	transmembrane protein 14A	0	BBnFlyp/lyp_39	1	1	1	0	0	0	0	0	0	0	6.74	7.23	7.31	7.29	6.79	7.16	7.30	7.30	6.82	7.35	7.26	7.28	6.54	7.21	7.34	7.30	6.78	6.97	7.26	7.25
1393436_at	Scgb1c1	secretoglobin, family 1C, member 1	0	BBnFlyp/lyp_39	1	1	1	0	0	0	0	0	0	0	4.07	4.05	4.41	4.59	4.14	4.11	4.76	5.14	3.78	3.64	3.70	4.32	4.07	3.85	3.69	4.09	3.53	3.44	3.66	3.64
1398256_at	Il1b	interleukin 1 beta	0	BBnFlyp/lyp_39	1	1	1	0	0	0	0	0	0	0	4.34	4.64	4.84	5.85	4.59	4.65	4.94	5.36	4.43	4.59	4.96	4.91	4.47	4.63	4.96	5.30	4.56	4.63	4.92	4.79
1398540_at	Rgs1	regulator of G-protein signaling 1	0	BBnFlyp/lyp_39	1	1	1	0	0	0	0	0	0	0	4.62	5.59	5.83	6.69	4.89	5.46	5.79	7.40	5.19	5.57	5.97	5.92	5.09	5.47	6.01	6.94	4.90	5.74	6.21	5.59
1367595_s_at	B2m	beta-2 microglobulin	0	common_113	1	1	1	1	0	0	0	0	0	0	10.80	11.11	11.45	12.67	10.84	11.02	11.40	12.05	11.14	11.52	11.57	11.84	11.25	11.50	11.84	12.00	11.01	11.37	11.49	11.66
1367679_at	Cd74	Cd74 molecule, major histocompatibility complex, class II invariant chain	0	common_113	1	1	1	1	0	0	0	0	0	0	7.25	8.37	8.68	11.04	7.37	8.24	8.99	11.40	7.77	8.08	8.63	8.67	7.65	8.16	8.85	9.59	7.60	8.37	8.49	8.31
1367705_at	Glrx	glutaredoxin (thioltransferase)	0	common_113	1	1	1	1	0	0	0	0	0	0	6.65	7.32	7.58	7.82	6.72	7.26	7.57	7.81	7.23	7.92	8.26	8.63	7.01	8.00	8.28	8.47	7.24	8.03	8.31	8.66
1367721_at	Sdc4	syndecan 4	0	common_113	1	1	1	1	0	0	0	0	0	0	8.75	9.08	9.37	9.54	8.84	8.96	9.37	9.30	8.56	8.83	8.96	9.27	8.94	8.94	9.16	9.27	8.66	8.75	9.02	9.20
1367942_at	Acp5	acid phosphatase 5, tartrate resistant	0	common_113	1	1	1	1	0	0	0	0	0	0	3.42	4.03	4.17	6.19	3.69	3.99	4.50	7.04	3.82	3.88	4.26	4.45	3.67	4.16	4.69	5.15	3.71	4.28	4.21	4.34
1367998_at	Slpi	secretory leukocyte peptidase inhibitor	0	common_113	1	1	1	1	0	0	0	0	0	0	4.15	5.09	5.72	8.88	4.62	4.86	6.06	8.78	4.63	4.82	5.75	5.28	3.96	5.41	8.10	9.16	4.44	5.41	5.51	5.44
1368027_at	Tbxas1	thromboxane A synthase 1, platelet	0	common_113	1	1	1	1	0	0	0	0	0	0	5.64	6.23	6.16	6.50	5.85	6.24	6.37	6.34	6.05	6.33	6.54	6.74	5.65	6.47	6.54	6.69	6.19	6.84	6.98	7.06
1368078_at	Esm1	endothelial cell-specific molecule 1	0	common_113	1	1	1	1	0	0	0	0	0	0	6.14	7.58	7.86	7.80	6.44	7.67	7.70	8.14	6.45	7.87	8.58	8.90	6.28	7.98	8.51	8.79	6.72	8.24	8.76	8.89
1368124_at	Dusp5	dual specificity phosphatase 5	0	common_113	1	1	1	1	0	0	0	0	0	0	5.84	6.20	6.32	6.43	6.02	6.11	7.00	6.71	6.15	6.58	7.00	6.89	6.25	6.55	6.78	6.96	6.21	6.58	7.09	6.86
1368137_at	Mapt	microtubule-associated protein tau	0	common_113	1	1	1	1	0	0	0	0	0	0	6.97	7.44	7.61	7.44	6.98	7.37	7.54	7.42	7.21	7.46	7.62	7.67	7.02	7.55	7.60	7.49	6.91	7.44	7.50	7.57
1368138_at	Mapt	microtubule-associated protein tau	0	common_113	1	1	1	1	0	0	0	0	0	0	6.17	6.65	6.79	6.77	6.20	6.54	6.84	6.63	6.41	6.78	6.83	6.94	6.29	6.82	6.85	6.90	6.32	6.69	6.84	6.86
1368157_at	Stmn3	stathmin-like 3	0	common_113	1	1	1	1	0	0	0	0	0	0	6.68	7.20	7.63	7.60	6.74	7.21	7.46	7.38	7.03	7.64	7.65	7.75	6.92	7.58	7.66	7.74	6.81	7.25	7.37	7.63
1368332_at	Gbp2	guanylate binding protein 2, interferon-inducible	0	common_113	1	1	1	1	0	0	0	0	0	0	2.97	3.38	3.72	7.67	3.23	3.27	3.60	4.92	3.04	3.63	3.52	3.72	3.16	3.45	4.15	4.68	3.23	3.20	3.75	3.77
1368399_a_at	Cpq	carboxypeptidase Q	0	common_113	1	1	1	1	0	0	0	0	0	0	7.44	7.75	7.99	8.03	7.41	7.66	7.91	8.00	7.42	7.82	8.02	8.47	7.46	7.88	8.10	8.44	7.40	7.88	8.12	8.49
1368437_at	Car4	carbonic anhydrase 4	0	common_113	1	1	1	1	0	0	0	0	0	0	4.46	5.32	5.79	5.99	4.44	5.09	5.92	6.07	4.58	4.85	5.41	5.82	4.46	5.08	5.57	5.68	4.79	5.26	5.74	6.01
1368499_at	Sycp2	synaptonemal complex protein 2	0	common_113	1	1	1	1	0	0	0	0	0	0	3.89	4.32	4.52	4.53	3.87	4.12	4.32	4.54	3.55	4.08	4.21	4.52	3.88	4.05	4.05	4.55	3.61	3.88	4.59	4.59
1368518_at	Cd53	Cd53 molecule	0	common_113	1	1	1	1	0	0	0	0	0	0	4.03	4.73	5.04	8.18	4.19	4.32	6.04	9.33	4.58	4.67	5.33	5.38	4.54	4.59	5.63	6.82	4.36	4.89	5.03	5.24
1368558_s_at	Aif1	allograft inflammatory factor 1	0	common_113	1	1	1	1	0	0	0	0	0	0	3.84	4.37	4.83	6.60	3.73	4.25	4.55	6.48	3.59	4.23	4.35	4.58	3.93	4.31	5.09	5.73	3.64	4.19	4.56	4.20
1369043_at	Kcna4	potassium voltage-gated channel, shaker-related subfamily, member 4	0	common_113	1	1	1	1	0	0	0	0	0	0	5.75	6.00	6.40	6.55	5.72	5.66	6.09	6.48	5.19	5.81	6.38	6.79	6.27	6.07	6.03	6.64	5.69	5.98	6.56	6.53
1369113_at	Grem1	gremlin 1	0	common_113	1	1	1	1	0	0	0	0	0	0	4.40	5.20	5.52	5.73	4.36	5.24	5.49	6.45	2.84	3.26	3.11	3.89	2.90	2.88	4.00	4.16	3.00	3.70	4.74	4.18
1369489_at	Aif1	allograft inflammatory factor 1	0	common_113	1	1	1	1	0	0	0	0	0	0	5.23	5.21	5.21	5.20	5.23	5.42	5.35	5.28	5.14	5.19	5.27	5.52	5.19	5.32	5.26	5.38	5.08	5.35	5.31	5.58
1369493_at	Prlr	prolactin receptor	0	common_113	1	1	1	1	0	0	0	0	0	0	5.10	5.41	5.57	5.74	5.14	5.40	5.77	5.84	4.69	5.29	5.60	5.78	4.90	5.59	5.74	5.80	5.05	5.29	5.79	5.97
1369665_a_at	Il18	interleukin 18	0	common_113	1	1	1	1	0	0	0	0	0	0	4.36	4.59	4.83	5.85	4.63	4.43	4.98	5.96	4.65	4.89	5.20	5.52	4.71	4.90	5.36	6.14	4.55	4.85	5.21	5.41
1369792_at	Gpr6	G protein-coupled receptor 6	0	common_113	1	1	1	1	0	0	0	0	0	0	5.26	5.65	5.76	5.85	5.36	5.61	5.88	5.87	5.55	5.71	6.14	6.53	5.21	5.70	6.25	6.29	5.68	5.91	6.20	6.55
1369964_at	Coro1a	coronin, actin binding protein 1A	0	common_113	1	1	1	1	0	0	0	0	0	0	4.14	4.45	4.92	8.39	4.30	4.60	6.45	9.42	4.54	4.33	4.94	5.01	4.59	4.35	5.14	6.27	4.17	4.55	4.81	5.04
1370054_at	Cdkn2c	cyclin-dependent kinase inhibitor 2C (p18, inhibits CDK4)	0	common_113	1	1	1	1	0	0	0	0	0	0	7.05	7.55	7.65	7.68	7.20	7.61	7.58	7.82	7.18	7.73	7.92	7.88	7.19	7.72	7.90	7.94	7.25	7.75	7.80	8.01
1370090_at	Lcp2	lymphocyte cytosolic protein 2	0	common_113	1	1	1	1	0	0	0	0	0	0	3.61	3.74	3.73	4.81	3.45	3.52	4.15	5.26	3.41	3.73	3.74	4.20	3.58	3.57	3.88	4.44	3.40	3.70	3.94	3.89
1370097_a_at	Cxcr4	chemokine (C-X-C motif) receptor 4	0	common_113	1	1	1	1	0	0	0	0	0	0	4.41	5.07	5.21	7.17	4.47	4.96	5.98	8.62	4.74	4.99	5.36	5.64	4.55	5.05	5.68	6.73	4.79	4.85	5.06	5.65
1370252_at	Avpi1	arginine vasopressin-induced 1	0	common_113	1	1	1	1	0	0	0	0	0	0	6.33	6.56	6.94	6.94	6.36	6.53	6.84	6.87	6.36	6.66	6.81	7.06	6.59	6.62	6.81	7.01	6.31	6.57	6.85	7.05
1370384_a_at	Prlr	prolactin receptor	0	common_113	1	1	1	1	0	0	0	0	0	0	8.93	9.55	9.89	10.13	9.03	9.50	9.78	10.19	8.63	9.32	10.03	10.26	8.69	9.63	9.97	10.25	8.90	9.62	10.06	10.50
1370522_at	Gcgr	glucagon receptor	0	common_113	1	1	1	1	0	0	0	0	0	0	5.90	6.37	6.62	6.87	6.17	6.24	6.81	6.89	6.76	7.22	7.47	7.81	6.97	7.30	7.60	7.77	6.87	7.25	7.66	7.86
1370590_at	Gpsm1	G-protein signaling modulator 1	0	common_113	1	1	1	1	0	0	0	0	0	0	8.29	8.69	8.79	8.84	8.06	8.57	9.00	8.77	8.32	8.94	9.01	8.86	7.44	9.02	8.91	8.77	8.07	8.71	8.60	8.69
1370595_a_at	Kcnip4	Kv channel interacting protein 4	0	common_113	1	1	1	1	0	0	0	0	0	0	2.46	2.80	3.05	3.01	2.61	2.92	3.15	3.09	2.60	2.85	2.95	3.13	2.51	2.97	3.19	3.06	2.62	2.95	2.95	3.17
1370603_a_at	Ptprc	protein tyrosine phosphatase, receptor type, C	0	common_113	1	1	1	1	0	0	0	0	0	0	2.47	2.64	2.92	5.39	2.47	2.70	3.55	6.76	2.56	2.60	2.75	2.91	2.45	2.40	2.96	3.78	2.46	2.75	2.73	2.94
1370789_a_at	Prlr	prolactin receptor	0	common_113	1	1	1	1	0	0	0	0	0	0	6.49	7.11	7.46	7.47	6.70	7.16	7.43	7.61	6.22	6.87	7.75	7.94	5.46	7.29	7.72	7.73	6.50	7.27	7.15	7.83

Affymetrix Probeset ID	Gene Symbol	Gene Name	unique BB on P7 n=822	Figure 1B P7 Venn n=1526	DRlyp/lyp STEM P7 n=712	DR+/+ STEM P7 n=936	Flyp/lyp STEM P7 n=520	F+/+ STEM P7 n=465	Figure 1B P0 Venn n=1071	DRlyp/lyp STEM P0 n=442	DR+/+ STEM P0 n=437	Flyp/lyp STEM P0 n=643	F+/+ STEM P0 n=632	Fig1D n=25	Mean log2 intensity DRlyp/lyp 20	Mean log2 intensity DRlyp/lyp 30	Mean log2 intensity DRlyp/lyp 40	Mean log2 intensity DRlyp/lyp 50	Mean log2 intensity DR+/+ 20	Mean log2 intensity DR+/+ 30	Mean log2 intensity DR+/+ 40	Mean log2 intensity DR+/+ 50	Mean log2 intensity Flyp/lyp 20	Mean log2 intensity Flyp/lyp 30	Mean log2 intensity Flyp/lyp 40	Mean log2 intensity Flyp/lyp 50	Mean log2 intensity Flyp20	Mean log2 intensity Flyp30	Mean log2 intensity Flyp40	Mean log2 intensity Flyp50	Mean log2 intensity F+/+ 20	Mean log2 intensity F+/+ 30	Mean log2 intensity F+/+ 40	Mean log2 intensity F+/+ 50
1371163_at	Slfn3	schlafen 3	0	common_113	1	1	1	1	0	0	0	0	0	0	3.23	3.39	3.35	3.36	3.28	3.35	3.47	3.25	3.26	3.31	3.35	3.53	3.27	3.42	3.30	3.32	3.29	3.41	3.41	3.36
1371447_at	Plac8	placenta-specific 8	0	common_113	1	1	1	1	0	0	0	0	0	0	6.22	6.57	6.87	10.17	6.22	6.36	8.28	10.90	6.71	6.64	7.60	7.29	6.17	6.69	7.47	8.96	6.07	6.71	6.96	7.32
1372064_at	Cxcl16	chemokine (C-X-C motif) ligand 16	0	common_113	1	1	1	1	0	0	0	0	0	0	6.41	6.73	6.88	7.62	6.46	6.75	6.92	7.15	6.40	6.81	6.84	7.17	6.44	6.83	7.07	7.22	6.41	6.91	7.12	7.13
1372383_at	Gpsm1	G-protein signaling modulator 1	0	common_113	1	1	1	1	0	0	0	0	0	0	10.55	10.81	11.00	11.12	10.28	10.88	11.11	10.98	10.56	11.09	11.18	11.08	10.46	11.03	11.08	11.07	10.34	10.91	11.06	11.21
1372478_at	Cmtm7	CKLF-like MARVEL transmembrane domain containing 7	0	common_113	1	1	1	1	0	0	0	0	0	0	7.31	7.69	7.86	7.93	7.48	7.69	7.78	8.00	7.20	7.65	8.05	8.10	7.29	7.72	7.90	7.97	7.26	7.96	8.13	8.30
1372626_at	Tpd52l1	tumor protein D52-like 1	0	common_113	1	1	1	1	0	0	0	0	0	0	5.91	6.39	7.13	7.73	6.10	6.24	7.24	7.70	5.80	6.60	7.50	8.79	6.60	6.95	7.71	8.51	6.55	7.14	8.11	8.91
1372647_at	---	---	0	common_113	1	1	1	1	0	0	0	0	0	0	5.21	5.85	6.03	6.06	5.39	5.84	6.17	6.08	4.75	5.38	5.39	5.58	4.96	5.34	5.72	5.86	5.11	5.32	5.64	5.68
1373636_at	Spock1	sparc/osteonectin, cwcv and kazal-like domains proteoglycan (testican) 1	0	common_113	1	1	1	1	0	0	0	0	0	0	4.85	5.12	5.44	5.84	5.07	5.18	5.46	5.71	6.14	6.38	6.88	7.26	6.81	6.82	7.06	7.24	6.34	6.64	6.86	7.01
1373661_a_at	Cxcr4	chemokine (C-X-C motif) receptor 4	0	common_113	1	1	1	1	0	0	0	0	0	0	5.32	5.98	6.04	7.74	5.48	5.97	6.69	9.01	5.64	5.82	6.19	6.40	5.68	5.87	6.48	7.22	5.67	5.68	5.96	6.28
1373886_at	Reep1	receptor accessory protein 1	0	common_113	1	1	1	1	0	0	0	0	0	0	3.60	4.08	4.40	4.53	3.53	3.93	4.33	4.43	3.67	4.46	4.42	4.97	3.71	4.03	4.00	4.73	3.18	3.77	4.08	4.40
1374434_at	LOC498222	similar to specifically androgen-regulated protein	0	common_113	1	1	1	1	0	0	0	0	0	0	3.90	4.20	4.20	4.43	3.67	3.95	5.02	4.60	3.72	4.03	4.08	4.36	4.30	4.04	4.33	4.39	3.79	4.01	4.05	4.36
1374650_at	Nedd9	neural precursor cell expressed, developmentally down-regulated 9	0	common_113	1	1	1	1	0	0	0	0	0	0	6.49	6.61	7.19	7.19	6.61	6.56	6.74	7.53	6.12	6.36	6.79	7.27	6.49	6.34	6.72	7.02	6.12	6.84	7.10	7.10
1374730_at	Tyrobp	Tyro protein tyrosine kinase binding protein	0	common_113	1	1	1	1	0	0	0	0	0	0	5.19	5.70	5.88	7.37	5.45	5.67	5.86	7.12	5.19	5.50	6.00	5.89	5.47	5.51	6.20	6.65	5.27	5.66	5.90	5.70
1374892_at	Sat2	spermidine/spermine N1-acetyltransferase family member 2	0	common_113	1	1	1	1	0	0	0	0	0	0	7.24	7.79	8.04	7.78	7.21	7.65	7.93	7.79	7.49	8.08	8.03	8.08	7.34	8.08	8.13	8.11	7.48	7.93	8.03	8.02
1374959_at	Nqo2	NAD(P)H dehydrogenase, quinone 2	0	common_113	1	1	1	1	0	0	0	0	0	0	5.09	5.40	5.55	5.89	5.12	5.12	5.58	5.68	6.86	7.31	7.42	7.54	6.70	7.27	7.47	7.56	6.95	7.35	7.47	7.60
1375052_at	C2cd4b	C2 calcium-dependent domain containing 4B	0	common_113	1	1	1	1	0	0	0	0	0	0	8.03	8.70	8.75	8.78	8.25	8.66	9.07	8.72	7.92	8.06	8.46	8.42	7.77	7.90	8.39	8.51	7.88	8.05	8.47	8.45
1375089_at	Filip1	filamin A interacting protein 1	0	common_113	1	1	1	1	0	0	0	0	0	0	4.93	5.35	5.46	5.77	5.03	5.09	5.46	5.66	5.35	5.68	6.20	5.88	4.48	5.70	6.02	6.03	5.70	5.67	5.72	5.94
1376094_at	Hint3	histidine triad nucleotide binding protein 3	0	common_113	1	1	1	1	0	0	0	0	0	0	7.31	7.68	7.80	7.86	7.27	7.63	7.78	7.75	7.33	7.66	7.79	7.85	7.25	7.69	7.90	7.90	7.36	7.64	7.86	7.94
1376285_at	Gulp1	GULP, engulfment adaptor PTB domain containing 1	0	common_113	1	1	1	1	0	0	0	0	0	0	8.88	9.29	9.49	9.42	8.88	9.42	9.39	9.36	8.87	9.27	9.44	9.47	8.60	9.18	9.43	9.42	8.73	9.29	9.44	9.51
1376579_at	Lap3	leucine aminopeptidase 3	0	common_113	1	1	1	1	0	0	0	0	0	0	6.97	7.19	7.37	7.56	6.71	6.89	7.11	7.40	7.07	7.40	7.35	7.61	7.07	7.31	7.48	7.58	7.21	7.37	7.56	7.77
1377163_at	Inhbb	inhibin beta-B	0	common_113	1	1	1	1	0	0	0	0	0	0	4.68	5.13	5.17	5.22	4.43	5.09	5.30	5.09	4.38	4.79	5.15	5.23	4.72	4.96	5.07	5.22	4.68	4.89	5.11	5.25
1377390_at	Bace2	beta-site APP-cleaving enzyme 2	0	common_113	1	1	1	1	0	0	0	0	0	0	6.89	7.22	7.63	7.50	6.71	7.15	7.44	7.32	6.65	7.13	7.11	7.39	6.43	6.83	7.07	7.39	6.54	6.90	7.10	7.40
1377626_at	LOC10091103	uncharacterized LOC100911030	0	common_113	1	1	1	1	0	0	0	0	0	0	3.59	3.73	4.21	7.36	3.59	4.03	4.72	5.46	3.70	3.89	3.90	4.27	3.85	3.97	4.36	4.79	3.73	3.91	4.23	4.07
1377729_at	---	---	0	common_113	1	1	1	1	0	0	0	0	0	0	5.90	6.34	6.45	6.48	5.78	6.13	6.41	6.30	6.14	6.71	6.70	6.66	6.08	6.49	6.60	6.63	5.92	6.57	6.63	6.63
1378430_at	Moxd1	monooxygenase, DBH-like 1	0	common_113	1	1	1	1	0	0	0	0	0	0	6.87	7.37	7.60	7.55	6.92	7.33	7.43	7.38	6.29	6.94	7.00	7.22	6.48	7.17	7.40	7.28	6.96	7.63	7.65	7.69
1378856_at	LOC679974 /	similar to transcription elongation factor A (SII)-like 3 /// transcription elongation factor A (SI	0	common_113	1	1	1	1	0	0	0	0	0	0	5.93	6.10	6.45	6.23	5.84	5.98	6.38	6.07	5.91	6.35	6.54	6.78	5.90	6.42	6.49	6.63	5.99	6.16	6.49	6.64
1379285_at	Rtp4	receptor (chemosensory) transporter protein 4	0	common_113	1	1	1	1	0	0	0	0	0	0	3.87	3.80	4.16	6.30	3.72	3.96	4.12	4.65	3.58	4.11	4.03	4.05	3.87	4.20	4.32	4.51	3.66	3.77	4.20	4.35
1379463_at	Filip1	filamin A interacting protein 1	0	common_113	1	1	1	1	0	0	0	0	0	0	6.44	6.83	7.08	7.49	6.08	6.70	7.38	7.30	6.85	7.51	7.86	7.73	6.61	7.74	7.77	7.98	7.22	7.41	7.67	8.21
1379568_at	Ifit2	interferon-induced protein with tetratricopeptide repeats 2	0	common_113	1	1	1	1	0	0	0	0	0	0	4.22	4.40	4.59	5.20	4.34	4.41	4.52	4.90	4.17	4.52	4.66	4.96	4.22	4.69	4.97	5.14	4.43	4.70	4.98	5.19
1381773_at	Abhd3	abhydrolase domain containing 3	0	common_113	1	1	1	1	0	0	0	0	0	0	3.59	3.73	3.77	3.75	3.65	3.70	3.94	3.85	3.68	3.77	3.77	3.87	3.48	3.83	3.90	3.97	3.60	3.86	3.66	3.97
1381993_at	Clic2	chloride intracellular channel 2	0	common_113	1	1	1	1	0	0	0	0	0	0	6.94	7.47	7.68	8.38	7.00	7.36	7.51	8.40	6.41	7.07	7.24	7.51	6.65	7.14	7.57	7.93	6.39	6.84	7.25	7.29
1382137_at	Abhd3	abhydrolase domain containing 3	0	common_113	1	1	1	1	0	0	0	0	0	0	5.34	5.70	6.18	6.25	5.39	5.61	5.94	6.27	5.18	5.83	6.27	6.69	5.11	5.97	6.05	6.56	5.28	6.06	6.39	6.95
1382311_at	Tifa	TRAF-interacting protein with forkhead-associated domain	0	common_113	1	1	1	1	0	0	0	0	0	0	4.63	4.58	4.79	5.95	4.52	4.50	5.31	6.29	4.23	4.40	4.78	5.24	4.58	4.49	5.18	5.83	4.41	4.64	4.98	4.88
1382855_at	Tcfl5	transcription factor-like 5 (basic helix-loop-helix)	0	common_113	1	1	1	1	0	0	0	0	0	0	6.53	6.92	7.16	7.11	6.54	6.99	7.23	7.09	6.62	6.98	7.08	7.18	6.75	7.10	7.25	7.26	6.52	6.98	7.24	7.25
1383912_at	Abracl	ABRA C-terminal like	0	common_113	1	1	1	1	0	0	0	0	0	0	6.02	6.16	6.37	6.77	5.99	5.98	6.26	7.12	6.08	6.50	6.55	6.86	6.04	6.37	6.56	7.08	6.19	6.36	6.53	6.75
1384180_at	Ifit2	interferon-induced protein with tetratricopeptide repeats 2	0	common_113	1	1	1	1	0	0	0	0	0	0	5.36	5.49	5.88	6.53	5.42	5.61	5.65	6.25	5.49	6.00	5.91	6.27	5.64	6.02	6.25	6.62	5.44	5.88	6.41	6.43
1385743_at	Cklf	chemokine-like factor	0	common_113	1	1	1	1	0	0	0	0	0	0	2.27	2.20	2.46	2.31	2.42	2.30	2.38	2.39	2.14	2.36	2.38	2.43	2.19	2.40	2.43	2.35	2.29	2.38	2.29	2.34
1385835_at	Plxdc1	plexin domain containing 1	0	common_113	1	1	1	1	0	0	0	0	0	0	5.22	5.77	6.13	6.05	5.40	5.80	5.97	5.87	5.11	5.27	5.72	5.86	4.75	5.46	5.77	5.88	5.03	5.52	5.54	5.93
1386908_at	Glrx	glutaredoxin (thioltransferase)	0	common_113	1	1	1	1	0	0	0	0	0	0	7.87	8.51	8.80	9.11	7.93	8.56	8.76	8.95	8.48	9.18	9.45	9.76	8.27	9.17	9.52	9.71	8.54	9.30	9.57	9.96
1387071_a_at	Mapt	microtubule-associated protein tau	0	common_113	1	1	1	1	0	0	0	0	0	0	6.33	6.87	7.14	7.03	6.34	6.85	7.01	6.80	6.42	7.17	7.21	7.14	6.46	7.05	7.15	7.17	6.36	7.04	7.07	7.19
1387134_at	Slfn3	schlafen 3	0	common_113	1	1	1	1	0	0	0	0	0	0	3.81	4.26	5.07	7.73	3.81	3.92	4.64	6.57	4.03	4.78	4.73	5.48	4.26	4.98	5.61	6.76	3.77	4.29	5.39	5.75
1387218_at	Tff3	trefoil factor 3, intestinal	0	common_113	1	1	1	1	0	0	0	0	0	0	7.87	8.46	8.48	8.66	7.90	8.42	8.44	8.37	8.04	8.67	8.68	8.65	7.38	8.37	8.63	8.63	7.76	8.18	8.41	8.26
1387529_a_at	Tagln3	transgelin 3	0	common_113	1	1	1	1	0	0	0	0	0	0	5.49	5.77	6.09	6.16	5.53	5.81	6.12	6.14	6.39	6.79	6.75	7.42	6.41	6.65	6.90	7.13	6.43	6.44	6.86	7.01
1387599_a_at	Nqo1	NAD(P)H dehydrogenase, quinone 1	0	common_113	1	1	1	1	0	0	0	0	0	0	5.78	6.29	6.91	7.11	6.13	6.43	6.99	7.08	5.88	6.67	7.03	7.56	6.26	6.49	6.82	7.24	6.07	6.57	7.12	7.43
1387770_at	Ifi27	interferon, alpha-inducible protein 27	0	common_113	1	1	1	1	0	0	0	0	0	0	7.83	8.13	8.63	9.57	8.09	8.16	8.38	8.87	9.70	10.04	10.04	10.42	10.02	10.07	10.34	10.57	9.70	9.87	10.07	10.27
1387818_at	Casp4	caspase 4, apoptosis-related cysteine peptidase	0	common_113	1	1	1	1	0	0	0	0	0	0	4.73	4.83	4.81	5.50	4.50	4.89	4.92	5.16	4.57	4.92	5.13	5.32	4.61	5.28	5.57	5.52	4.67	5.15	5.23	5.29
1387938_at	Baalc	brain and acute leukemia, cytoplasmic	0	common_113	1	1	1	1	0	0	0	0	0	0	5.57	5.78	6.18	6.07	5.52	5.79	5.97	6.16	5.41	5.79	5.86	6.02	5.61	5.48	5.91	6.00	5.02	5.44	5.92	6.02
1387956_s_at	Cklf	chemokine-like factor	0	common_113	1	1	1	1	0	0	0	0	0	0	4.04	4.01	4.36	4.80	3.81	3.85	4.42	4.91	4.14	4.17	4.49	4.65	3.95	4.30	4.50	4.77	4.04	3.99	4.42	4.67
1387984_at	Cklf	chemokine-like factor	0	common_113	1	1	1	1	0	0	0	0	0	0	5.37	5.58	5.73	6.19	5.19	5.39	5.71	6.14	5.39	5.54	5.73	5.90	4.98	5.75	5.93	6.23	5.45	5.38	5.73	6.03
1388138_at	Thbs4	thrombospondin 4	0	common_113	1	1	1	1	0	0	0	0	0	0	5.30	6.48	7.26	6.98	5.44	6.85	7.15	7.31	4.18	5.08	4.93	5.96	3.90	4.73	5.90	6.14	4.68	4.76	5.72	5.97
1388447_at	Lbh	limb bud and heart development	0	common_113	1	1	1	1	0	0	0	0	0	0	6.33	6.40	6.66	6.83	6.48	6.36	6.61	6.93	6.32	6.55	6.82	7.15	6.58	6.80	6.86	7.09	6.37	6.80	7.00	7.23
1388883_at	Pold4	polymerase (DNA-directed), delta 4, accessory subunit	0	common_113	1	1	1	1	0	0	0	0	0	0	7.48	7.84	8.14	8.32	7.52	7.91	8.06	8.69	7.69	8.14	8.34	8.70	7.58	8.14	8.46	8.57	7.63	8.22	8.50	8.63
1388930_at	Tmem123	transmembrane protein 123	0	common_113	1	1	1	1	0	0	0	0	0	0	9.86	10.18	10.36	10.55	9.82	10.11	10.31	10.60	9.92	10.18	10.48	10.58	9.80	10.12	10.44	10.61	9.86	10.21	10.45	10.67
1388963_at	Astn1	astrotactin 1	0	common_113	1	1	1	1	0	0	0	0	0	0	5.65	6.25	6.98	6.92	5.59	6.18	6.72	6.75	5.28	6.18	6.55	7.38	5.35	6.30	6.73	7.27	5.00	5.96	6.51	7.25
1389034_at	Usp18	ubiquitin specific peptidase 18	0	common_113	1	1	1	1	0	0	0	0	0	0	5.46	5.64	5.83	7.35	5.58	5.72	5.73	6.17	5.05	5.44	5.49	5.84	5.35	5.51	5.96	6.28	5.13	5.23	5.57	5.65
1389234_at	Vwf	von Willebrand factor	0	common_113	1	1	1	1	0	0	0	0	0	0	4.30	4.79	5.02	5.63	4.63	4.53	5.13	5.63	4.26	5.02	5.47	5.92	4.69	5.23	5.83	5.99	4.43	4.88	5.68	6.00
1389244_x_at	Cxcr4	chemokine (C-X-C motif) receptor 4	0	common_113	1	1	1	1	0	0	0	0	0	0	5.76	6.24	6.30	8.13	5.82	6.26	6.92	9.27	5.74	6.11	6.36	6.59	5.81	6.03	6.79	7.51	5.83	5.98	6.24	6.52
1389306_at	Matn2	matrilin 2	0	common_113	1	1	1	1	0	0	0	0	0	0	6.11	6.43	6.82	7.03	6.40	6.50	6.72	7.06	5.24	5.86	6.39	6.72	5.71	5.76	6.32	6.53	5.48	5.98	6.58	6.95
1390146_at	Tmem150c	transmembrane protein 150C	0	common_113	1	1	1	1	0	0	0	0	0	0	7.64	8.11	8.28	8.34	7.51	8.00	8.13	8.31	7.38	7.90	8.20	8.52	7.48	7.99	8.15	8.40	7.42	7.99	8.29	8.61
1390397_at	Syndig1	synapse differentiation inducing 1	0	common_113	1	1	1	1	0	0	0	0	0	0	4.70	4.92	5.19	5.66	4.54	5.06	5.48	5.48	5.03	5.46	5.81	6.20	5.35	5.52	5.98	6.06	5.10	5.25	5.50	6.29
1390472_at	Naaa	N-acylethanolamine acid amidase	0	common_113	1	1	1	1	0	0	0	0	0	0	5.58	6.04	6.28	6.76	5.56	5.95	6.26	7.00	5.59	5.90	6.06	6.19	5.50	5.87	6.04	6.13	5.50	5.81	6.08	6.04
1390738_at	Bst2	bone marrow stromal cell antigen 2	0	common_113	1	1	1	1	0	0	0	0	0	0	6.46	6.86	6.92	7.67	6.40	6.74	6.95	7.15	6.32	6.76	6.71	6.94	6.23	6.83	6.82	6.96	6.29	6.77	6.83	6.89
1390797_at	Lcp2	lymphocyte cytosolic protein 2	0	common_113	1	1	1	1	0	0	0	0	0	0	3.44	3.54	3.50	4.44	3.59	3.52	3.81	4.71	3.32	3.52	3.84	3.87	3.40	3.84	3.87	4.28	3.25	3.51	3.78	3.86
1390798_at	Ptprc	protein tyrosine phosphatase, receptor type, C	0	common_113	1	1	1	1	0	0	0	0	0	0	3.47	3.90	4.32	7.82	3.75	4.03	5.95	9.04	4.09	3.92	4.19	4.79	3.76	3.88	4.49	6.28	3.54	4.02	4.18	4.66
1391083_at	Arhgap22	Rho GTPase activating protein 22	0	common_113	1	1	1	1	0	0	0	0	0	0	3.12	3.30	3.51	3.68	3.11	3.14	3.48	3.75	2.98	3.36	3.56	3.58	3.49	3.41	3.40	3.52	3.19	3.21	3.51	3.70
1391132_at	Car4	carbonic anhydrase 4	0	common_113	1	1	1	1	0	0	0	0	0	0	3.78	3.63	3.71	3.75	3.70	3.89	3.86	3.68	3.70	3.68	3.74	3.75	3.62	3.71	3.85	3.74	3.67	3.67	3.66	3.81
1391341_at	Plxdc1	plexin domain containing 1	0	common_113	1	1	1	1	0	0	0	0	0	0	6.95	7.65	8.16	8.04	6.86	7.74	7.98	8.06	6.15	6.64	7.25	7.61	6.17	7.01	7.31	7.57	6.23	6.90	7.19	7.62
1391509_at	Tacstd2	tumor-associated calcium signal transducer 2	0	common_113	1	1	1	1	0	0	0	0	0	0	2.92	3.04	3.28	3.60	2.90	2.91	4.61	4.18	2.63	2.82	2.82	3.18	2.83	3.06	3.35	3.72	2.69	2.74	3.22	2.99
1392451_at	LOC691354	hypothetical protein LOC691354	0	common_113	1	1	1	1	0	0	0	0	0	0	6.43	6.86	7.10	7.19	6.45	6.69	7.15	7.11	6.84	7.28	7.38	7.63	6.56	7.39	7.62	7.62	6.86	7.20	7.35	7.63
1392515_at	Klra17 /// Klr	killer cell lectin-like receptor, subfamily A, member 17 /// killer cell lectin-like receptor, subfa	0	common_113	1	1	1	1	0	0	0	0	0	0	4.93	5.15	5.19	5.90	4.66	4.97	5.03	5.74	5.06	5.28	5.46	5.66	5.12	5.25	5.62	6.11	4.78	4.98	5.30	5.10
1393756_at	Dmp1	dentin matrix acidic phosphoprotein 1	0	common_113	1	1	1	1	0	0	0	0	0	0	3.71	4.29	4.76	4.94	3.92	4.09	4.95	4.79	3.73	4.06	4.20	4.69	3.64	3.98	4.28	4.58	3.58	4.04	4.30	4.61
1394435_at	Vangl1	VANGL planar cell polarity protein 1	0	common_113	1	1	1	1	0	0	0	0	0	0	6.00	6.78	6.76	6.75	6.14	6.76	6.82	6.79	5.96	6.64	6.75	6.94	6.10	6.75	6.97	7.10	6.07	6.72	6.92	7.24
1394681_at	Akr1cl	aldo-keto reductase family 1, member C-like	0	common_113	1	1	1	1	0	0	0	0	0	0	4.69	5.28	5.44	5.65	4.71	5.10	5.92	5.47	4.91	5.70	5.69	5.92	4.73	5.75	5.91	5.97	4.80	5.63	5.79	5.71
1396053_at	Nedd9	neural precursor cell expressed, developmentally down-regulated 9	0	common_113	1	1	1	1	0	0	0	0	0	0	5.06	5.10	5.64	5.64	5.26	5.24	5.36	5.93	4.84	4.93	5.36	5.86	4.96	4.91	5.22	5.71	4.71	5.26	5.86	5.51
1396386_at	Bace2	beta-site APP-cleaving enzyme 2	0	common_113	1	1	1	1	0	0	0	0	0	0	5.83	6.17	6.53	6.46	5.93	6.25	6.46	6.15	5.52	6.30	6.25	6.29	5.41	6.10	6.04	6.23	5.55	5.99	5.92	6.19
1397227_at	Tmem35	transmembrane protein 35	0	common_113	1	1	1	1	0	0	0	0	0	0	6.17	6.47	7.38	7.85	5.98	6.93	7.52	8.01	4.70	6.35	7.58	8.70	5.66	7.18	7.89	8.43	4.80	6.39	7.20	7.88
1398219_at	LOC690918	hypothetical protein LOC690918	0	common_113	1	1	1	1	0	0	0	0	0	0	9.92	10.04	10.44	10.52	10.02	9.99	10.18	10.55	10.24	10.38	10.73	11.09	10.49	10.32	10.73	10.97	10.32	10.58	10.84	10.93
1398431_at	Car8	carbonic anhydrase 8	0	common_113	1	1	1	1	0	0	0	0	0	0	6.32	6.62	6.62	6.84	6.28	6.26	6.59	6.97	6.98	7.03	7.43	7.55	7.45	7.41	7.39	7.76	7.30	7.15	7.44	7.74
1367811_at	Phgdh	phosphoglycerate dehydrogenase	0	DR+/+nF+/+_11	0	1	0	1	0	0	0	0	0	0	5.53	6.00	5.74	5.92	5.58	5.87	5.99	6.61	6.03	6.14	6.35	6.47	5.75	6.80	6.36	6.39	6.01	6.65	6.61	6.94
1368465_at	Asic2	acid-sensing (proton-gated) ion channel 2	0	DR+/+nF+/+_11	0	1	0	1	0	0	0	0	0	0	3.88	3.64	4.33	3.94	3.66	3.50	3.66	5.21	3.56	3.89	3.62	3.54	3.65	3.61	3.43	4.50	3.68	3.70	3.78	4.49
1371140_a_at	Asic2	acid-sensing (proton-gated) ion channel 2	0	DR+/+nF+/+_11	0	1	0	1	0	0	0	0	0	0	2.88	2.94	3.04	3.07	2.96	3.17	2.94	3.34	3.02	2.98	2.93	3.06	2.89	2.98	3.02	3.23	3.02	2.89	2.85	3.28
1376792_at	Eva1a	eva-1 homolog A	0	DR+/+nF+/+_11	0	1	0	1	0	0	0	0	0	0	5.15	5.39	5.46	5.42	4.93	5.17	5.42	5.45	5.47	5.64	5.65	5.52	5.55	5.64	5.82	5.69	5.28	5.42	5.65	5.87
1377998_at	Cpox	coproporphyrinogen oxidase	0	DR+/+nF+/+_11	0	1	0	1	0	0	0	0	0	0	6.61	6.86	6.95	6.98	6.50	6.77	6.85	7.10	6.62	6.84	6.90	7.06	6.73	6.88	7.00	7.08	6.65	6.89	6.90	7.17
1378387_at	Tinf2	TERF1 (TRF1)-interacting nuclear factor 2	0	DR+/+nF+/+_11	0	1	0	1	0	0	0	0	0	0	5.80	6.13	6.24	6.26	5.85	6.11	6.23	6.37	6.03	6.21	6.46	6.39	5.77	6.24	6.40	6.32	6.00	6.23	6.50	6.47
1378855_a_at	LOC679974 /	similar to transcription elongation factor A (SII)-like 3 /// transcription elongation factor A (SI	0	DR+/+nF+/+_11	0	1	0	1	0	0	0	0	0	0	4.30	4.77	5.04	4.72	4.27	4.51	4.80	4.70	4.52	4.90	4.95	5.02	4.37	4.76	5.00	5.00	4.40	4.61	4.83	4.99
1383721_at	Fzd8 /// LOC1	frizzled family receptor 8 /// frizzled-8-like	0	DR+/+nF+/+_11	0	1	0	1	0	0	0	0	0	0	5.54	5.58	5.88	6.03	5.45	5.70	6.04	5.89	5.57	5.73	5.97	6.01	5.28	5.77	6.06	6.16	5.63	5.81	5.87	6.20
1388689_at	Acyp2	acylphosphatase 2, muscle type	0	DR+/+nF+/+_11	0	1	0	1	0	0	0	0	0	0	6.05	6.10	6.35	6.36	5.79	5.90	6.23	6.38	6.12	6.18	6.31	6.43	6.10	6.28	6.29	6.55	6.14	6.14	6.30	6.74
1393621_at	LOC10090975	vitamin K epoxide reductase complex subunit 1-like protein 1-like /// vitamin K epoxide redu	0	DR+/+nF+/+_11	0	1	0	1	0	0	0	0	0	0	4.76	4.89	4.90	5.12	4.78	4.70	4.85	5.23	4.78	5.10	5.09	5.12	4.85	5.29	5.15	5.22	4.84	5.03	5.25	5.36
1398480_at	Lrrc24	leucine rich repeat containing 24	0	DR+/+nF+/+_11	0	1	0	1	0	0	0	0	0	0	3.55	3.61	4.00	3.79	3.21	3.36	3.70	3.96	3.67	3.91	3.76	4.06	3.75	3.75	3.93	4.06	3.60	3.68	4.05	4.15
1367660_at	Fabp3	fatty acid binding protein 3, muscle and heart	0	DR+/+nFIS_15	0	1	1	1	0	0	0	0	0	0	5.40	5.77	6.06	5.73	5.35	5.49	6.02	5.85	5.80	6.26	6.32	6.47	6.01	6.25	6.38	6.39	5.83	6.06	6.37	6.37
1367749_at	Lum	lumican	0	DR+/+nFIS_15	0	1	1	1	0	0	0	0	0	0	6.85	7.51	7.12	7.55	6.68	7.31	7.17	8.24	5.59	6.06	6.21	6.57	5.68	6.23	6.80	7.18	5.97	6.56	6.77	6.84
1368255_at	Ntm	neurotrimin	0	DR+/+nFIS_15	0	1	1	1	0	0	0	0	0	0	5.63	5.71	5.93	5.79	5.25	5.74	5.90	5.81	5.00	5.68	6.13	6.79	5.08	5.65	6.12	6.47	5.20	5.81	6.47	6.92
1370089_at	Ppargc1a	peroxisome proliferator-activated receptor gamma, coactivator 1 alpha	0	DR+/+nFIS_15	0	1	1	1	0	0	0	0	0	0	3.12	3.14	3.44	3.53	2.82	2.85	3.40	3.84	2.79	3.12	3.75	3.77	3.04	3.57	3.18	3.96	3.14	3.78	3.94	3.78
1373674_at	Mfap5	microfibrillar associated protein 5	0	DR+/+nFIS_15	0	1	1	1	0	0	0	0	0	0	4.31	5.03	5.06	4.90	4.07	4.58	4.90	5.06	3.48	4.08	4.17	4.42	3.58	4.06	4.17	4.34	3.60	4.15	4.49	4.55
1378089_at	Tmem100	transmembrane protein 100	0	DR+/+nFIS_15	0	1	1	1	0	0	0	0	0	0	4.14	3.98	4.06	4.22	3.92	4.24	4.24	4.27	3.86	3.97	4.32	4.50	3.99	3.99	4.39	4.35	3.81	3.91	4.23	4.49
1379396_at	Elmo1	engulfment and cell motility 1	0	DR+/+nFIS_15	0	1	1	1	0	0	0	0	0	0	5.93	5.96	5.94	6.39	6.04	5.86	6.11	6.47	6.03	6.26	6.25	6.63	6.53	6.14	6.40	6.62	6.05	6.24	6.59	6.38
1380726_at	---	---	0	DR+/+nFIS_15	0	1	1	1	0	0	0	0	0	0	5.67	5.92	6.09	6.02	5.73	5.90	6.04	6.44	4.81	5.20	5.39	5.63	5.07	5.41	5.56	5.80	5.15	5.50	5.45	5.87
1381449_s_at	Tgfa	transforming growth factor alpha	0	DR+/+nFIS_15	0	1	1	1	0	0	0	0	0	0	6.90	7.20	7.36	7.23	6.88	7.00	7.18	7.48	6.46	6.87	7.02	7.16	6.74	6.87	7.06	7.23	6.55	6.88	7.01	7.02
1381504_at	Aspn	asporin	0	DR+/+nFIS_15	0	1	1	1	0	0	0	0	0	0	4.84	5.05	5.13	4.87	4.69	5.00	4.95	5.41	4.01	4.10	4.47	4.59	4.15	4.48	4.64	4.61	4.38	4.22	4.69	4.85
1382589_at	Ankrd55	ankyrin repeat domain 55	0	DR+/+nFIS_15	0	1	1	1	0	0	0	0	0	0	4.87	5.16	5.23	5.27	4.70	4.84	5.37	5.26	4.14	4.43	4.65	4.84	4.19	4.69	4.87	4.84	4.21	4.43	5.11	5.01
1387413_at	Ncf1	neutrophil cytosolic factor 1	0	DR+/+nFIS_15	0	1	1	1	0	0	0	0	0	0	3.92	3.93	3.96	3.88	3.73	3.89	4.13	4.25	3.72	3.76	4.02	4.25	3.75	4.10	3.86	4.07	3.90	3.80	4.31	4.14
1387450_at	Tgfa	transforming growth factor alpha	0	DR+/+nFIS_15	0	1	1	1	0	0	0	0	0	0	5.36	5.34	5.62	5.63	5.19	5.20	5.58	5.70	4.79	5.28	5.32	5.61	5.25	5.18	5.47	5.66	4.95	5.34	5.48	5.57
1389467_at	Tmem100	transmembrane protein 100	0	DR+/+nFIS_15	0	1	1	1	0	0	0	0	0	0	6.44	6.45	6.85	6.72	6.55	6.49	6.62	7.23	6.07	6.14	6.95	7.31	6.23	6.29	6.86	7.21	6.09	6.27	6.90	7.32
1393783_at	Cntnap2	contactin associated protein-like 2	0	DR+/+nFIS_15	0	1	1	1	0	0	0	0	0	0	2.85	2.83	3.06	3.26	2.83	2.60	2.94	3.45	2.75	2.89	3.10	3.76	2.89	3.05	2.81	3.29	2.88	2.86	2.96	3.47
1367612_at	Mgst1	microsomal glutathione S-transferase 1	0	DR+/+nFlyp/lyp_13	0	1	1	0	0	0	0	0	0	0	6.16	6.20	6.52	6.61	6.17	6.30	6.71	6.85	5.15	5.22	5.23	5.68	5.38	5.27	6.04	5.93	5.29	4.70	5.44	5.08
1367871_at	Cyp2e1	cytochrome P450, family 2, subfamily e, polypeptide 1	0	DR+/+nFlyp/lyp_13	0	1	1	0	0	0	0	0	0	0	4.21	4.16	4.53	4.51	4.05	4.22	4.76	4.63	3.86	4.09	4.02	4.67	4.11	4.26	4.52	4.51	4.06	4.12	4.42	4.33
1367940_at	Cxcr7	chemokine (C-X-C motif) receptor 7	0	DR+/+nFlyp/lyp_13	0	1	1	0	0	0	0	0	0	0	6.20	6.64	6.75	6.43	6.29	6.43	6.86	7.28	5.44	5.73	5.98	6.09	5.71	5.75	5.93	6.20	5.52	5.90	6.01	5.91
1368322_at	Sod3	superoxide dismutase 3, extracellular	0	DR+/+nFlyp/lyp_13	0	1	1	0	0	0	0	0	0	0	4.54	4.53	4.83	4.86	4.52	4.69	4.98	5.16	4.21	4.46	4.34	4.77	4.36	4.48	4.77	4.99	4.33	4.51	4.57	4.62
1368476_at	Nr3c2	nuclear receptor subfamily 3, group C, member 2	0	DR+/+nFlyp/lyp_13	0	1	1	0	0	0	0	0	0	0	5.71	6.09	6.31	6.07	5.55	5.94	6.21	6.08	5.81	6.14	6.40	6.30	5.95	6.38	6.37	6.23	5.93	6.09	6.28	6.39
1370157_at	Pln	phospholamban	0	DR+/+nFlyp/lyp_13	0	1	1	0	0	0	0	0	0	0	5.77	6.32	6.64	6.52	5.56	6.10	6.60	6.44	4.94	5.58	5.48	5.89	4.96	5.59	5.63	5.44	5.08	5.33	5.41	5.46
1370352_at	Ces1f /// Cesl	carboxylesterase 1F /// carboxylesterase-like 1	0	DR+/+nFlyp/lyp_13	0	1	1	0	0	0	0	0	0	0	4.27	4.37	4.49	4.46	3.88	4.16	4.98	4.90	3.96	4.04	4.10	4.46	3.88	4.25	4.56	4.83	4.23	3.86	4.40	4.45
1383469_at	Aldh1a3	aldehyde dehydrogenase 1 family, member A3	0	DR+/+nFlyp/lyp_13	0	1	1	0	0	0	0	0	0	0	2.72	2.85	3.17	3.01	2.64	3.10	3.21	3.21	2.44	2.77	2.84	3.03	2.58	2.70	2.97	3.06	2.69	2.75	3.12	2.99
1384382_at	Osgepl1	O-sialoglycoprotein endopeptidase-like 1	0	DR+/+nFlyp/lyp_13	0	1	1	0	0	0	0	0	0	0	4.40	4.73	4.82	4.77	4.35	4.61	4.72	4.91	4.38	4.80	4.94	4.99	4.58	4.84	4.92	4.86	4.62	4.69	5.06	5.00
1384515_at	Hey2	hairy/enhancer-of-split related with YRPW motif 2	0	DR+/+nFlyp/lyp_13	0	1	1	0	0	0	0	0	0	0	5.03	5.18	5.43	5.51	4.93	5.04	5.54	5.74	4.93	4.98	4.89	5.43	5.13	4.95	5.11	5.47	5.28	5.28	5.42	5.76
1384689_at	Pln	phospholamban	0	DR+/+nFlyp/lyp_13	0	1	1	0	0	0	0	0	0	0	2.49	2.51	2.51	2.42	2.38	2.50	2.41	2.35	2.46	2.39	2.43	2.58	2.40	2.28	2.54	2.38	2.45	2.57	2.28	2.49
1391277_at	S100a5	S100 calcium binding protein A5	0	DR+/+nFlyp/lyp_13	0	1	1	0	0	0	0	0	0	0	3.52	3.45	3.46	3.91	3.30	3.57	3.70	4.23	3.07	3.44	3.47	3.58	3.45	3.57	3.77	4.06	3.43	3.40	3.52	3.68
1396561_x_at	Piga	phosphatidylinositol glycan anchor biosynthesis, class A	0	DR+/+nFlyp/lyp_13	0	1	1	0	0	0	0	0	0	0	3.86	3.68	3.74	4.18	3.69	3.47	3.88	4.14	3.78	4.09	4.03	4.37	4.37	4.20	4.18	4.22	4.09	4.15	4.14	4.23

Affymetrix Probeset ID	Gene Symbol	Gene Name	unique BB on P7 n=822	Figure 1B P7 Venn n=1526	DRlyp/lyp STEM P7 n=712	DR+/+ STEM P7 n=936	Flyp/lyp STEM P7 n=520	F+/+ STEM P7 n=465	Figure 1B P0 Venn n=1071	DRlyp/lyp STEM P0 n=442	DR+/+ STEM P0 n=437	Flyp/lyp STEM P0 n=643	F+/+ STEM P0 n=632	Fig1D n=25	Mean log2 intensity DRlyp/lyp 20	Mean log2 intensity DRlyp/lyp 30	Mean log2 intensity DRlyp/lyp 40	Mean log2 intensity DRlyp/lyp 50	Mean log2 intensity DR+/+ 20	Mean log2 intensity DR+/+ 30	Mean log2 intensity DR+/+ 40	Mean log2 intensity DR+/+ 50	Mean log2 intensity Flyp/lyp 20	Mean log2 intensity Flyp/lyp 30	Mean log2 intensity Flyp/lyp 40	Mean log2 intensity Flyp/lyp 50	Mean log2 intensity Flyp20	Mean log2 intensity Flyp30	Mean log2 intensity Flyp40	Mean log2 intensity Flyp50	Mean log2 intensity F+/+ 20	Mean log2 intensity F+/+ 30	Mean log2 intensity F+/+ 40	Mean log2 intensity F+/+ 50
1370202_at	Pla2g16	phospholipase A2, group XVI	1	DRlyp/lypnDR+/+_347	1	1	0	0	uniqFlyp/lyp_145	0	0	1	0	0	5.70	5.75	5.73	7.09	5.59	5.52	5.85	6.99	5.65	5.44	5.22	4.99	5.98	5.51	5.74	5.91	5.42	5.27	5.26	5.18
1373911_at	Postn	periostin, osteoblast specific factor	1	DRlyp/lypnDR+/+_347	1	1	0	0	uniqFlyp/lyp_145	0	0	1	0	0	4.33	4.23	4.19	5.11	4.15	4.01	3.82	5.76	4.71	4.50	4.46	4.16	5.20	4.68	5.05	5.46	4.80	4.75	4.66	4.35
1379499_at	Ltb	lymphotoxin beta (TNF superfamily, member 3)	1	DRlyp/lypnDR+/+_347	1	1	0	0	uniqFlyp/lyp_145	0	0	1	0	0	4.91	5.17	5.11	6.94	5.13	5.11	5.45	8.04	5.17	4.84	4.78	4.65	5.04	4.79	5.21	5.89	4.84	4.84	4.57	4.59
1378015_at	Ccl21	chemokine (C-C motif) ligand 21	1	DRlyp/lypnDR+/+_347	1	1	0	0	0	0	0	0	0	1	3.70	3.65	4.18	7.40	4.11	3.88	5.14	8.87	3.89	4.04	3.91	3.88	3.90	4.22	5.02	7.15	3.68	3.94	3.62	3.93
1387969_at	Cxcl10	chemokine (C-X-C motif) ligand 10	1	DRlyp/lypnDR+/+_347	1	1	0	0	0	0	0	0	0	2	5.87	6.05	6.83	9.82	6.01	5.61	7.34	7.19	5.43	5.22	6.04	5.74	5.62	5.48	5.61	5.48	5.33	5.12	5.42	5.62
1387316_at	Cxcl1	chemokine (C-X-C motif) ligand 1 (melanoma growth stimulating activity, alpha)	1	DRlyp/lypnDR+/+_347	1	1	0	0	0	0	0	0	0	3	5.35	6.07	6.84	7.07	5.47	5.49	9.15	8.63	5.32	5.29	5.72	5.14	5.22	5.44	5.34	5.94	5.16	4.75	5.28	5.37
1373970_at	Il33	interleukin 33	1	DRlyp/lypnDR+/+_347	1	1	0	0	0	0	0	0	0	4	4.15	4.18	4.68	6.25	4.02	4.09	5.13	7.31	3.61	3.96	3.90	4.32	3.67	3.79	4.30	4.93	3.66	3.82	4.04	4.27
1367973_at	Ccl2	chemokine (C-C motif) ligand 2	1	DRlyp/lypnDR+/+_347	1	1	0	0	0	0	0	0	0	5	3.11	3.24	3.11	5.86	3.26	3.18	3.58	5.92	3.27	3.22	3.22	3.03	3.21	3.06	3.44	4.64	3.11	3.13	3.25	3.16
1379935_at	Ccl7	chemokine (C-C motif) ligand 7	1	DRlyp/lypnDR+/+_347	1	1	0	0	0	0	0	0	0	6	2.99	3.06	3.15	5.37	2.85	3.19	3.49	4.70	3.07	3.27	3.29	3.32	3.18	3.22	3.21	3.85	3.05	3.21	3.29	3.27
1369815_at	Ccl3	chemokine (C-C motif) ligand 3	1	DRlyp/lypnDR+/+_347	1	1	0	0	0	0	0	0	0	7	4.69	4.87	5.01	6.30	4.98	4.84	5.50	7.06	4.90	4.94	5.31	5.22	4.78	5.03	5.19	5.68	4.93	4.85	5.07	5.25
1387202_at	Icam1	intercellular adhesion molecule 1	1	DRlyp/lypnDR+/+_347	1	1	0	0	0	0	0	0	0	8	4.23	4.33	4.10	5.79	4.06	4.37	4.68	6.25	4.29	4.31	4.18	4.40	4.47	4.52	4.51	4.75	4.36	4.46	4.40	4.39
1368527_at	Ptgs2	prostaglandin-endoperoxide synthase 2	1	DRlyp/lypnDR+/+_347	1	1	0	0	0	0	0	0	0	10	2.13	2.19	2.03	3.03	2.30	2.02	2.34	4.58	2.06	2.16	2.11	2.12	2.06	1.89	2.04	2.57	2.07	2.13	2.12	2.08
1388587_at	Ier3	immediate early response 3	1	DRlyp/lypnDR+/+_347	1	1	0	0	0	0	0	0	0	12	7.30	7.82	7.94	7.94	7.66	7.67	8.83	8.64	7.50	7.61	7.84	7.56	7.49	7.57	7.70	7.69	7.50	7.47	7.51	7.49
1368073_at	Irf1	interferon regulatory factor 1	1	DRlyp/lypnDR+/+_347	1	1	0	0	0	0	0	0	0	14	8.23	8.51	8.48	9.59	8.51	8.53	9.13	8.75	8.25	8.56	8.47	7.96	8.32	8.23	8.34	8.10	8.23	8.02	8.04	7.94
1368490_at	Cd14	CD14 molecule	1	DRlyp/lypnDR+/+_347	1	1	0	0	0	0	0	0	0	16	6.46	6.77	7.09	7.24	6.57	6.79	7.10	6.95	6.09	6.39	6.30	6.31	6.04	6.24	6.69	6.70	6.17	6.09	6.37	5.92
1369814_at	Ccl20	chemokine (C-C motif) ligand 20	1	DRlyp/lypnDR+/+_347	1	1	0	0	0	0	0	0	0	19	3.06	3.14	3.19	4.17	3.23	3.18	3.76	4.39	3.05	3.20	3.08	2.91	3.22	2.89	3.00	3.23	3.03	3.00	2.80	3.08
1384946_at	Tlr1	toll-like receptor 1	1	DRlyp/lypnDR+/+_347	1	1	0	0	0	0	0	0	0	21	2.41	2.46	2.61	3.26	2.44	2.46	2.62	3.07	2.33	2.56	2.53	2.62	2.47	2.46	2.66	2.71	2.44	2.59	2.61	2.63
1382531_at	Tlr7	toll-like receptor 7	1	DRlyp/lypnDR+/+_347	1	1	0	0	0	0	0	0	0	22	2.53	2.50	2.56	3.12	2.51	2.46	2.70	3.48	2.46	2.50	2.68	2.54	2.44	2.64	2.75	3.07	2.53	2.42	2.60	2.78
1391925_at	Ccl19	chemokine (C-C motif) ligand 19	1	DRlyp/lypnDR+/+_347	1	1	0	0	0	0	0	0	0	23	4.50	4.58	4.64	5.32	4.57	4.56	4.69	5.50	4.49	4.46	4.52	4.55	4.70	4.64	4.92	5.25	4.47	4.62	4.65	4.84
1368192_at	Cxcr3	chemokine (C-X-C motif) receptor 3	1	DRlyp/lypnDR+/+_347	1	1	0	0	0	0	0	0	0	24	3.49	3.58	3.81	4.04	3.62	3.85	3.97	4.32	3.73	3.58	3.76	3.85	3.77	3.70	3.78	3.92	3.69	3.71	4.01	3.77
1367576_at	Gpx1	glutathione peroxidase 1	1	DRlyp/lypnDR+/+_347	1	1	0	0	0	0	0	0	0	0	9.30	9.40	9.45	9.95	9.40	9.39	9.49	10.34	9.48	9.30	9.47	9.38	9.46	9.60	9.49	9.62	9.34	9.42	9.26	9.28
1367586_at	Ldha	lactate dehydrogenase A	1	DRlyp/lypnDR+/+_347	1	1	0	0	0	0	0	0	0	0	6.93	6.76	6.86	7.70	7.00	6.66	7.08	8.53	6.69	6.83	6.54	6.66	7.00	6.75	6.81	6.80	6.78	6.62	6.69	6.70
1367605_at	Pfn1	profilin 1	1	DRlyp/lypnDR+/+_347	1	1	0	0	0	0	0	0	0	0	8.02	8.13	8.17	8.57	8.12	8.07	8.19	8.92	8.12	8.19	8.17	8.18	7.99	8.18	8.20	8.23	8.14	8.14	8.15	8.07
1367614_at	Anxa1	annexin A1	1	DRlyp/lypnDR+/+_347	1	1	0	0	0	0	0	0	0	0	7.37	7.32	7.42	7.84	7.40	7.37	7.91	8.53	6.93	6.89	6.93	7.20	7.07	6.86	7.40	7.47	7.07	7.03	7.19	7.21
1367628_at	Lgals1	lectin, galactoside-binding, soluble, 1	1	DRlyp/lypnDR+/+_347	1	1	0	0	0	0	0	0	0	0	5.55	5.80	5.80	6.13	5.70	5.83	6.07	6.75	5.66	6.54	5.64	5.82	5.93	5.91	5.93	6.01	5.67	5.66	5.85	5.92
1367673_at	Selenbp1	selenium binding protein 1	1	DRlyp/lypnDR+/+_347	1	1	0	0	0	0	0	0	0	0	5.63	6.00	6.13	6.03	5.72	5.89	6.37	6.41	5.62	5.56	5.57	5.63	6.01	5.86	5.83	5.92	5.83	5.75	5.68	5.74
1367774_at	Gsta3	glutathione S-transferase A3	1	DRlyp/lypnDR+/+_347	1	1	0	0	0	0	0	0	0	0	6.44	6.76	7.11	7.09	6.19	6.62	7.29	7.25	5.79	6.00	5.60	6.42	6.02	6.16	6.38	6.60	5.97	5.79	5.83	6.22
1367785_at	Cnn1	calponin 1, basic, smooth muscle	1	DRlyp/lypnDR+/+_347	1	1	0	0	0	0	0	0	0	0	3.14	3.25	3.18	3.79	3.06	3.12	3.74	4.53	2.82	2.83	2.87	3.15	2.86	2.96	2.91	2.98	2.97	2.88	2.80	3.12
1367786_at	Psmb8	proteasome (prosome, macropain) subunit, beta type, 8	1	DRlyp/lypnDR+/+_347	1	1	0	0	0	0	0	0	0	0	6.95	7.01	7.32	8.93	6.92	6.96	7.23	8.62	6.97	7.04	6.92	6.99	6.23	6.11	6.37	6.57	7.00	6.86	6.99	6.94
1367846_at	S100a4	S100 calcium-binding protein A4	1	DRlyp/lypnDR+/+_347	1	1	0	0	0	0	0	0	0	0	6.14	6.69	6.87	7.39	6.16	6.54	6.89	7.44	5.38	6.62	6.00	6.07	5.67	6.14	6.07	6.44	5.46	6.02	5.88	5.87
1367850_at	Fcgr2a /// LO	Fc fragment of IgG, low affinity IIa, receptor /// Low affinity immunoglobulin gamma Fc regio	1	DRlyp/lypnDR+/+_347	1	1	0	0	0	0	0	0	0	0	4.49	4.71	4.58	6.42	4.21	4.44	4.74	5.22	4.16	4.40	4.41	4.24	4.22	4.09	4.42	4.56	4.40	4.21	4.51	4.15
1367936_at	Stk10	serine/threonine kinase 10	1	DRlyp/lypnDR+/+_347	1	1	0	0	0	0	0	0	0	0	6.33	6.29	6.34	7.03	6.34	6.31	6.42	7.49	6.14	6.10	6.09	6.09	6.36	6.15	6.17	6.23	6.17	6.13	6.14	6.24
1368000_at	C3	complement component 3	1	DRlyp/lypnDR+/+_347	1	1	0	0	0	0	0	0	0	0	3.35	3.46	3.59	5.40	3.39	3.55	4.93	5.23	3.49	3.47	3.44	3.63	3.41	3.49	3.80	3.81	3.27	3.54	3.45	3.55
1368006_at	Laptm5	lysosomal protein transmembrane 5	1	DRlyp/lypnDR+/+_347	1	1	0	0	0	0	0	0	0	0	5.10	5.31	5.57	7.92	5.37	5.37	6.20	8.93	5.10	5.12	5.49	5.47	5.14	5.12	5.71	6.53	4.97	5.32	5.40	5.25
1368010_at	Ptpn6	protein tyrosine phosphatase, non-receptor type 6	1	DRlyp/lypnDR+/+_347	1	1	0	0	0	0	0	0	0	0	7.18	7.15	7.33	7.85	7.14	7.24	7.24	8.37	7.29	7.29	7.42	7.58	7.27	7.26	7.40	7.58	7.21	7.33	7.40	7.51
1368075_at	Lipa	lipase A, lysosomal acid, cholesterol esterase	1	DRlyp/lypnDR+/+_347	1	1	0	0	0	0	0	0	0	0	6.78	6.81	7.10	7.50	6.93	6.65	7.14	7.57	6.75	6.71	6.87	7.18	7.04	6.89	7.09	7.26	6.76	6.85	6.94	7.21
1368167_at	Ctse	cathepsin E	1	DRlyp/lypnDR+/+_347	1	1	0	0	0	0	0	0	0	0	3.04	3.44	3.48	6.14	3.32	3.50	4.40	7.33	3.47	3.20	3.78	3.65	3.22	3.46	3.66	4.51	3.35	3.41	3.55	3.69
1368186_a_at	Syk	spleen tyrosine kinase	1	DRlyp/lypnDR+/+_347	1	1	0	0	0	0	0	0	0	0	4.91	4.94	4.89	5.68	4.99	5.08	5.31	5.99	5.01	4.92	5.02	5.00	4.96	5.06	5.10	5.18	4.88	4.97	4.86	5.16
1368247_at	Hspa1a /// Hs	heat shock 70kD protein 1A /// heat shock 70kD protein 1B (mapped)	1	DRlyp/lypnDR+/+_347	1	1	0	0	0	0	0	0	0	0	7.99	8.12	8.72	9.73	7.71	7.04	9.82	9.93	8.14	7.75	7.90	8.83	7.32	10.42	9.56	9.38	8.81	8.44	7.98	9.44
1368270_at	Apobec1	apolipoprotein B mRNA editing enzyme, catalytic polypeptide 1	1	DRlyp/lypnDR+/+_347	1	1	0	0	0	0	0	0	0	0	2.34	2.39	2.43	3.03	2.36	2.37	2.39	3.10	2.48	2.66	2.59	2.76	2.63	2.51	2.90	2.78	2.48	2.45	2.70	2.58
1368290_at	Cyr61	cysteine-rich, angiogenic inducer, 61	1	DRlyp/lypnDR+/+_347	1	1	0	0	0	0	0	0	0	0	7.10	7.60	7.72	7.84	7.25	7.37	8.12	8.32	7.10	7.47	7.45	7.37	6.75	7.40	7.40	7.83	7.09	7.39	7.68	7.25
1368294_at	Dnase1l3	deoxyribonuclease 1-like 3	1	DRlyp/lypnDR+/+_347	1	1	0	0	0	0	0	0	0	0	4.33	4.52	4.57	7.42	4.09	4.15	5.20	7.98	4.66	4.29	5.00	4.02	4.06	4.48	6.13	7.13	4.10	4.41	4.38	4.46
1368337_at	Glycam1	glycosylation dependent cell adhesion molecule 1	1	DRlyp/lypnDR+/+_347	1	1	0	0	0	0	0	0	0	0	3.91	3.99	5.63	5.94	5.42	4.28	6.00	8.68	4.02	4.06	4.32	4.23	4.16	4.10	4.55	5.10	4.20	4.16	4.12	4.36
1368474_at	LOC10091247	vascular cell adhesion protein 1-like /// vascular cell adhesion molecule 1	1	DRlyp/lypnDR+/+_347	1	1	0	0	0	0	0	0	0	0	3.57	3.88	3.76	7.26	3.21	3.49	4.74	7.68	3.62	3.22	4.09	3.59	3.55	3.19	5.32	6.81	3.42	3.53	3.61	3.68
1368482_at	Bcl2a1	BCL2-related protein A1	1	DRlyp/lypnDR+/+_347	1	1	0	0	0	0	0	0	0	0	3.56	4.02	4.04	6.83	3.80	3.86	4.34	7.07	3.68	3.89	3.91	4.06	3.65	3.79	4.47	5.39	3.54	3.71	3.96	3.89
1368492_at	Hpgds	hematopoietic prostaglandin D synthase	1	DRlyp/lypnDR+/+_347	1	1	0	0	0	0	0	0	0	0	4.55	4.40	4.49	5.06	4.44	4.51	4.64	4.95	4.48	4.48	4.45	4.61	4.53	4.54	4.62	4.90	4.58	4.44	4.60	4.66
1368521_at	Napsa	napsin A aspartic peptidase	1	DRlyp/lypnDR+/+_347	1	1	0	0	0	0	0	0	0	0	2.87	3.13	3.04	4.99	3.01	3.05	3.44	5.62	3.01	3.03	3.13	3.13	2.87	2.92	3.19	3.50	2.91	2.95	3.18	3.29
1368530_at	Mmp12	matrix metallopeptidase 12	1	DRlyp/lypnDR+/+_347	1	1	0	0	0	0	0	0	0	0	3.56	3.60	3.81	4.86	3.46	3.77	3.96	4.27	3.30	3.47	3.54	3.78	3.27	3.36	3.73	3.87	3.35	3.64	3.62	3.76
1368555_at	Cd37	CD37 molecule	1	DRlyp/lypnDR+/+_347	1	1	0	0	0	0	0	0	0	0	2.54	2.54	2.49	3.15	2.70	2.76	2.77	4.32	2.55	2.51	2.62	2.93	2.55	2.56	2.78	3.05	2.72	2.70	2.68	2.79
1368610_at	Milr1	**mast cell immunoglobulin-like receptor 1**	1	DRlyp/lypnDR+/+_347	1	1	0	0	0	0	0	0	0	0	3.39	3.26	3.45	4.03	3.31	3.39	3.44	4.72	3.46	3.37	3.34	3.35	3.36	3.32	3.32	3.35	3.41	3.23	3.45	3.52
1368655_at	Srgn	serglycin	1	DRlyp/lypnDR+/+_347	1	1	0	0	0	0	0	0	0	0	7.92	8.00	8.02	8.79	7.94	7.91	8.17	9.21	8.28	8.32	8.31	8.31	8.29	8.46	8.34	8.34	8.38	8.52	8.39	8.39
1368679_a_at	Lyn	v-yes-1 Yamaguchi sarcoma viral related oncogene homolog	1	DRlyp/lypnDR+/+_347	1	1	0	0	0	0	0	0	0	0	4.65	4.76	4.69	5.37	4.65	4.74	4.62	5.61	5.29	5.26	5.41	5.48	4.78	5.37	5.64	5.59	5.31	5.46	5.31	5.35
1368762_at	Ubd	ubiquitin D	1	DRlyp/lypnDR+/+_347	1	1	0	0	0	0	0	0	0	0	3.51	3.86	4.34	10.63	3.90	3.84	4.98	7.45	3.87	3.57	4.03	4.15	3.84	3.82	5.07	5.69	3.69	3.70	3.78	4.11
1368914_at	Runx1	runt-related transcription factor 1	1	DRlyp/lypnDR+/+_347	1	1	0	0	0	0	0	0	0	0	4.32	4.48	4.56	5.67	4.18	4.57	4.71	6.43	3.99	4.22	4.03	4.64	3.99	4.25	4.30	4.81	4.14	4.30	4.59	4.50
1368915_at	Kmo	kynurenine 3-monooxygenase (kynurenine 3-hydroxylase)	1	DRlyp/lypnDR+/+_347	1	1	0	0	0	0	0	0	0	0	2.56	2.65	2.73	3.09	2.71	2.85	2.95	3.47	2.68	2.71	2.80	2.83	2.70	2.81	2.95	2.94	2.76	2.85	2.75	2.90
1368921_a_at	Cd44	Cd44 molecule	1	DRlyp/lypnDR+/+_347	1	1	0	0	0	0	0	0	0	0	5.00	5.01	4.98	5.82	5.06	5.17	5.33	6.09	4.80	4.89	5.03	5.04	4.95	4.81	5.27	5.26	4.80	4.93	5.00	4.95
1368941_at	Ptger4	prostaglandin E receptor 4 (subtype EP4)	1	DRlyp/lypnDR+/+_347	1	1	0	0	0	0	0	0	0	0	3.16	2.98	3.27	3.28	3.19	3.05	3.35	3.47	3.09	3.00	3.13	3.22	3.06	3.33	3.20	3.27	3.15	3.14	3.12	3.17
1368990_at	Cyp1b1	cytochrome P450, family 1, subfamily b, polypeptide 1	1	DRlyp/lypnDR+/+_347	1	1	0	0	0	0	0	0	0	0	4.29	4.22	4.30	4.84	4.25	4.39	4.72	4.98	4.24	4.32	4.36	4.46	4.23	4.23	4.35	4.53	4.49	4.31	4.55	4.46
1369025_at	Cd5 /// LOC10	Cd5 molecule /// T-cell surface glycoprotein CD5-like	1	DRlyp/lypnDR+/+_347	1	1	0	0	0	0	0	0	0	0	3.78	3.79	3.87	4.60	3.96	3.93	4.24	5.43	3.77	3.86	4.02	3.80	3.99	4.06	4.26	4.24	3.79	3.97	3.93	3.98
1369093_at	Reln	reelin	1	DRlyp/lypnDR+/+_347	1	1	0	0	0	0	0	0	0	0	2.29	2.31	2.30	2.48	2.38	2.37	2.34	2.60	2.32	2.24	2.29	2.42	2.22	2.30	2.51	2.30	2.32	2.32	2.31	2.48
1369105_a_at	Pkib	protein kinase (cAMP-dependent, catalytic) inhibitor beta	1	DRlyp/lypnDR+/+_347	1	1	0	0	0	0	0	0	0	0	5.63	6.01	6.28	6.61	5.56	5.86	6.25	6.90	4.24	4.40	4.64	4.44	4.66	4.56	4.78	4.83	4.09	4.40	4.58	4.54
1369110_x_at	RT1-EC2	RT1 class Ib, locus EC2	1	DRlyp/lypnDR+/+_347	1	1	0	0	0	0	0	0	0	0	4.71	4.83	5.16	6.61	4.66	4.85	5.26	6.30	4.93	5.15	5.17	5.55	4.76	4.89	4.83	5.02	4.76	4.99	5.27	5.42
1369181_at	Cybb	cytochrome b-245, beta polypeptide	1	DRlyp/lypnDR+/+_347	1	1	0	0	0	0	0	0	0	0	3.60	3.48	3.62	4.90	3.39	3.64	3.75	5.83	3.75	3.50	3.63	3.82	3.31	3.56	3.98	4.09	3.55	3.65	3.82	3.63
1369186_at	Casp1	caspase 1	1	DRlyp/lypnDR+/+_347	1	1	0	0	0	0	0	0	0	0	4.86	4.78	4.80	5.83	4.78	4.86	4.95	6.62	4.74	4.71	4.84	5.03	5.06	4.87	5.05	5.33	4.80	4.94	4.81	5.00
1369190_at	Cd2	Cd2 molecule	1	DRlyp/lypnDR+/+_347	1	1	0	0	0	0	0	0	0	0	3.49	3.49	3.59	4.27	3.78	3.78	3.79	4.37	3.60	3.51	3.70	3.70	3.60	3.65	3.84	3.78	3.57	3.68	3.56	3.63
1369204_at	Hck	hemopoietic cell kinase	1	DRlyp/lypnDR+/+_347	1	1	0	0	0	0	0	0	0	0	3.76	3.82	4.17	5.80	3.73	3.86	4.22	5.17	3.77	3.75	4.02	3.84	3.53	3.91	4.03	4.46	3.68	3.98	3.94	3.90
1369262_at	Casp8	caspase 8	1	DRlyp/lypnDR+/+_347	1	1	0	0	0	0	0	0	0	0	5.26	5.32	5.29	5.85	5.28	5.25	5.49	6.10	5.33	5.20	5.40	5.42	5.18	5.22	5.38	5.36	5.35	5.27	5.35	5.31
1369268_at	Atf3	activating transcription factor 3	1	DRlyp/lypnDR+/+_347	1	1	0	0	0	0	0	0	0	0	6.54	7.26	7.18	7.14	7.37	6.94	9.11	8.75	7.21	7.17	7.68	6.72	6.89	6.86	7.41	7.29	7.22	7.12	7.08	6.81
1369281_at	Tnfsf11	tumor necrosis factor (ligand) superfamily, member 11	1	DRlyp/lypnDR+/+_347	1	1	0	0	0	0	0	0	0	0	2.22	2.37	2.23	2.79	2.17	2.45	2.39	3.25	2.33	2.40	2.22	2.52	2.23	2.29	2.68	2.77	2.17	2.35	2.36	2.53
1369282_at	Tnfsf11	tumor necrosis factor (ligand) superfamily, member 11	1	DRlyp/lypnDR+/+_347	1	1	0	0	0	0	0	0	0	0	2.02	2.25	2.14	2.89	2.19	2.18	2.48	3.50	1.93	2.22	2.20	2.38	2.20	2.12	2.23	2.62	2.05	2.15	2.19	2.23
1369294_at	Bst1	bone marrow stromal cell antigen 1	1	DRlyp/lypnDR+/+_347	1	1	0	0	0	0	0	0	0	0	5.27	5.13	5.24	6.60	5.27	5.18	5.47	6.77	5.39	5.05	5.27	5.37	5.07	4.96	5.18	5.55	5.30	5.13	5.40	5.37
1369306_at	Klrd1	killer cell lectin-like receptor, subfamily D, member 1	1	DRlyp/lypnDR+/+_347	1	1	0	0	0	0	0	0	0	0	2.12	2.24	2.32	2.80	2.13	2.18	2.28	2.71	2.32	2.24	2.44	2.25	2.50	2.42	2.48	3.22	2.28	2.28	2.39	2.31
1369387_at	Vav1	vav 1 guanine nucleotide exchange factor	1	DRlyp/lypnDR+/+_347	1	1	0	0	0	0	0	0	0	0	3.73	3.69	3.75	4.52	3.79	3.92	3.93	4.89	3.69	3.71	3.81	4.02	3.72	3.81	3.96	3.96	3.83	3.86	3.68	3.90
1369552_at	Samsn1	SAM domain, SH3 domain and nuclear localization signals, 1	1	DRlyp/lypnDR+/+_347	1	1	0	0	0	0	0	0	0	0	2.86	3.04	3.30	3.82	3.05	2.99	3.26	3.73	3.09	3.00	3.25	3.08	2.91	3.03	3.12	3.36	2.99	3.15	3.07	3.39
1369683_at	Bid	BH3 interacting domain death agonist	1	DRlyp/lypnDR+/+_347	1	1	0	0	0	0	0	0	0	0	2.96	3.17	3.20	3.61	3.15	3.30	3.29	4.23	2.98	3.24	3.24	3.09	2.92	3.04	3.24	3.25	2.98	3.11	3.01	3.07
1369872_a_at	Fcer2	Fc fragment of IgE, low affinity II, receptor for (CD23)	1	DRlyp/lypnDR+/+_347	1	1	0	0	0	0	0	0	0	0	3.21	3.09	3.05	3.51	3.02	3.21	3.17	3.63	3.03	3.09	3.23	3.27	3.31	3.12	3.16	3.02	2.99	3.05	3.18	3.29
1370026_at	Cryab	crystallin, alpha B	1	DRlyp/lypnDR+/+_347	1	1	0	0	0	0	0	0	0	0	5.35	5.48	5.59	5.90	5.36	5.45	6.15	6.42	5.21	5.24	5.09	5.30	5.29	5.32	5.63	5.48	5.35	5.14	5.32	5.40
1370057_at	Csrp1	cysteine and glycine-rich protein 1	1	DRlyp/lypnDR+/+_347	1	1	0	0	0	0	0	0	0	0	7.17	7.26	7.28	7.78	7.11	7.15	7.58	8.23	6.87	7.12	7.12	7.24	6.99	7.22	7.15	7.32	7.06	7.08	7.05	7.14
1370096_at	Prf1	perforin 1 (pore forming protein)	1	DRlyp/lypnDR+/+_347	1	1	0	0	0	0	0	0	0	0	3.59	3.53	3.60	4.67	3.49	3.86	3.72	4.27	3.57	3.67	3.81	3.80	3.52	3.63	3.75	4.07	3.45	3.70	3.70	3.86
1370104_at	Pde6h	phosphodiesterase 6H, cGMP-specific, cone, gamma	1	DRlyp/lypnDR+/+_347	1	1	0	0	0	0	0	0	0	0	3.46	3.41	3.45	4.76	3.71	3.63	3.91	5.68	3.46	3.44	3.75	3.71	3.53	3.60	3.56	4.11	3.46	3.66	3.53	3.60
1370186_at	Psmb9	proteasome (prosome, macropain) subunit, beta type, 9	1	DRlyp/lypnDR+/+_347	1	1	0	0	0	0	0	0	0	0	7.17	7.42	7.55	9.28	7.19	7.36	7.46	8.40	7.19	7.43	7.27	7.26	6.84	7.06	7.19	7.26	7.26	7.22	7.26	7.17
1370215_at	C1qb	complement component 1, q subcomponent, B chain	1	DRlyp/lypnDR+/+_347	1	1	0	0	0	0	0	0	0	0	5.00	5.19	5.11	6.65	4.97	5.06	5.28	6.69	5.01	5.14	5.14	5.42	5.08	4.92	5.40	5.84	4.65	5.08	5.10	4.66
1370219_at	Cyba	cytochrome b-245, alpha polypeptide	1	DRlyp/lypnDR+/+_347	1	1	0	0	0	0	0	0	0	0	6.19	6.34	6.29	7.32	6.20	6.35	6.44	7.79	6.04	6.16	6.21	6.19	6.15	6.19	6.35	6.55	6.05	6.27	6.11	6.20
1370249_at	Tspo	translocator protein	1	DRlyp/lypnDR+/+_347	1	1	0	0	0	0	0	0	0	0	5.91	5.90	5.97	6.98	5.91	5.96	6.44	7.35	5.71	5.93	5.86	5.78	5.95	5.87	6.08	6.26	5.92	5.79	5.88	5.81
1370355_at	Scd1	stearoyl-Coenzyme A desaturase 1	1	DRlyp/lypnDR+/+_347	1	1	0	0	0	0	0	0	0	0	2.91	2.98	2.97	3.41	3.12	3.11	3.17	3.71	2.98	2.96	3.26	3.15	3.00	2.84	2.99	3.15	2.94	3.03	2.97	3.24
1370371_a_at	Ceacam1 /// (	carcinoembryonic antigen-related cell adhesion molecule 1 (biliary glycoprotein) /// carcinoe	1	DRlyp/lypnDR+/+_347	1	1	0	0	0	0	0	0	0	0	3.22	3.19	3.30	3.81	3.20	3.07	3.26	5.07	3.15	3.21	3.28	3.51	3.10	3.11	3.03	3.50	3.22	3.09	3.17	3.50
1370382_at	RT1-Db1	RT1 class II, locus Db1	1	DRlyp/lypnDR+/+_347	1	1	0	0	0	0	0	0	0	0	1.86	2.00	2.00	2.37	1.87	1.99	2.06	2.79	1.97	1.99	2.07	2.00	2.60	2.63	2.98	3.40	1.96	2.13	2.05	2.08
1370383_s_at	RT1-Db1	RT1 class II, locus Db1	1	DRlyp/lypnDR+/+_347	1	1	0	0	0	0	0	0	0	0	5.48	6.06	6.37	8.46	5.49	5.89	6.59	8.97	5.66	6.00	6.33	6.45	6.08	6.53	7.08	7.50	5.59	6.09	6.34	6.18
1370394_at	IgG-2a	gamma-2a immunoglobulin heavy chain	1	DRlyp/lypnDR+/+_347	1	1	0	0	0	0	0	0	0	0	3.15	3.19	3.11	5.44	3.23	3.06	3.52	8.25	3.30	3.34	3.49	3.71	3.26	3.26	3.23	5.44	3.14	3.46	3.16	3.61
1370419_a_at	Sh3kbp1	SH3-domain kinase binding protein 1	1	DRlyp/lypnDR+/+_347	1	1	0	0	0	0	0	0	0	0	5.56	5.75	5.73	6.15	5.65	5.50	5.73	6.60	5.62	5.59	5.55	5.74	5.56	5.43	5.76	5.72	5.64	5.50	5.75	5.85
1370422_at	Ripk3	receptor-interacting serine-threonine kinase 3	1	DRlyp/lypnDR+/+_347	1	1	0	0	0	0	0	0	0	0	3.66	3.76	3.69	4.66	3.70	3.75	3.81	4.80	3.68	3.71	3.75	3.95	3.71	3.59	3.65	3.98	3.74	3.55	3.79	3.84
1370428_x_at	RT1-A2 /// RT	RT1 class Ia, locus A2 /// RT1 class I, locus A3 /// RT1 class Ib, locus EC2	1	DRlyp/lypnDR+/+_347	1	1	0	0	0	0	0	0	0	0	8.90	8.99	9.23	10.12	8.91	9.02	9.09	9.55	9.24	9.36	9.28	9.36	2.71	2.75	3.09	2.98	9.12	9.21	9.02	9.16
1370429_at	RT1-EC2	RT1 class Ib, locus EC2	1	DRlyp/lypnDR+/+_347	1	1	0	0	0	0	0	0	0	0	5.45	5.86	6.04	6.55	5.49	5.96	5.87	6.21	5.59	5.76	5.84	6.17	4.25	4.76	4.75	5.17	5.50	5.69	5.72	5.69
1370516_at	Slc15a3	solute carrier family 15, member 3	1	DRlyp/lypnDR+/+_347	1	1	0	0	0	0	0	0	0	0	4.43	4.32	4.37	5.50	4.25	4.27	4.59	5.87	4.32	4.18	4.36	4.37	4.16	4.23	4.38	4.49	4.19	4.35	4.57	4.35
1370621_at	Cd247	Cd247 molecule	1	DRlyp/lypnDR+/+_347	1	1	0	0	0	0	0	0	0	0	3.24	3.34	3.55	4.10	3.36	3.48	3.91	4.74	3.23	3.44	3.51	3.51	3.36	3.39	3.50	3.79	3.31	3.52	3.69	3.50
1370822_at	RT1-Ba	RT1 class II, locus Ba	1	DRlyp/lypnDR+/+_347	1	1	0	0	0	0	0	0	0	0	6.10	7.01	7.14	9.32	6.19	6.83	7.46	9.92	6.48	6.76	7.19	7.16	6.61	7.15	8.01	8.62	6.41	7.05	7.01	6.82
1370885_at	Ctsz	cathepsin Z	1	DRlyp/lypnDR+/+_347	1	1	0	0	0	0	0	0	0	0	6.68	6.69	6.85	7.53	6.73	6.74	6.76	7.70	6.42	6.36	6.48	6.40	6.18	6.44	6.50	6.59	6.37	6.29	6.28	6.08
1370896_a_at	Myh11	myosin, heavy chain 11, smooth muscle	1	DRlyp/lypnDR+/+_347	1	1	0	0	0	0	0	0	0	0	6.61	7.35	7.49	7.87	6.50	7.03	7.85	8.66	5.87	6.11	6.29	6.75	6.09	6.21	6.33	6.44	6.11	6.32	6.21	6.51
1370904_at	LOC1009095	class II histocompatibility antigen, M alpha chain-like /// RT1 class II, locus DMa	1	DRlyp/lypnDR+/+_347	1	1	0	0	0	0	0	0	0	0	5.32	5.59	5.64	7.20	5.34	5.51	5.83	7.89	5.24	5.46	5.60	5.70	5.45	5.57	5.91	6.09	5.28	5.52	5.55	5.22
1370912_at	Hspa1a	heat shock 70kD protein 1A	1	DRlyp/lypnDR+/+_347	1	1	0	0	0	0	0	0	0	0	9.14	9.12	9.59	10.64	8.74	7.86	10.64	10.56	9.00	8.57	8.74	9.62	7.57	10.05	9.24	9.06	9.55	9.32	8.81	10.06
1370924_at	Tcrb	T-cell receptor beta chain	1	DRlyp/lypnDR+/+_347	1	1	0	0	0	0	0	0	0	0	4.16	4.04	4.26	5.92	4.15	4.30	5.38	7.25	4.27	4.16	4.35	4.60	4.20	4.26	4.55	5.46	4.24	4.47	4.46	4.57
1370987_at	Spn	sialophorin	1	DRlyp/lypnDR+/+_347	1	1	0	0	0	0	0	0	0	0	4.54	4.85	4.95	6.10	4.74	4.84	5.30	6.80	4.81	5.09	5.19	5.24	5.02	5.11	5.15	5.50	5.08	5.08	5.23	5.11
1371015_at	Mx1	myxovirus (influenza virus) resistance 1	1	DRlyp/lypnDR+/+_347	1	1	0	0	0	0	0	0	0	0	2.46	2.48	2.60	4.90	2.51	2.68	2.86	4.56	2.64	2.64	2.74	2.89	2.50	2.83	2.86	4.30	2.83	2.66	2.72	3.00
1371016_at	LOC290071	similar to RIKEN cDNA A430107P09 gene	1	DRlyp/lypnDR+/+_347	1	1	0	0	0	0	0	0	0	0	3.13	2.99	3.11	5.94	3.15	2.99	5.02	7.58	2.93	2.99	2.81	3.35	3.26	3.08	3.22	4.28	3.20	3.18	3.29	3.53
1371078_at	LOC1003649	MHC class Ib alpha chain-like /// similar to RT1 class I, CE11 /// similar to H-2 class I histocom	1	DRlyp/lypnDR+/+_347	1	1	0	0	0	0	0	0	0	0	4.05	4.42	4.45	5.75	4.30	4.35	4.51	5.24	4.48	4.54	4.53	4.76	4.31	4.56	4.69	4.82	4.20	4.53	4.56	4.53
1371111_at	RT1-EC2	RT1 class Ib, locus EC2	1	DRlyp/lypnDR+/+_347	1	1	0	0	0	0	0	0	0	0	6.46	6.36	6.50	6.63	6.35	6.30	6.50	6.48	6.32	6.40	6.41	6.43	6.25	6.37	6.39	6.44	6.27	6.38	6.29	6.42
1371115_at	Ptpre	protein tyrosine phosphatase, receptor type, E	1	DRlyp/lypnDR+/+_347	1	1	0	0	0	0	0	0	0	0	2.83	2.92	3.10	3.65	2.97	2.83	3.07	3.46	2.73	2.88	2.82	3.06	2.76	2.91	2.91	3.05	2.76	2.87	2.95	2.96
1371123_x_at	RT1-S3	RT1 class Ib, locus S3	1	DRlyp/lypnDR+/+_347	1	1	0	0	0	0	0	0	0	0	6.43	6.39	6.62	7.92	6.44	6.35	6.47	7.06	6.75	6.74	6.51	6.69	6.76	6.82	6.84	6.84	6.75	6.52	6.63	6.59
1371171_at	RT1-EC2	RT1 class Ib, locus EC2	1	DRlyp/lypnDR+/+_347	1	1	0	0	0	0	0	0	0	0	4.42	4.64	4.58	5.04	4.49	4.48	4.99	4.91	4.48	4.74	4.69	4.71	5.14	5.37	5.38	5.37	4.57	4.55	4.54	4.77
1371256_at	Ptpn18	protein tyrosine phosphatase, non-receptor type 18	1	DRlyp/lypnDR+/+_347	1	1	0	0	0	0	0	0	0	0	5.57	5.68	5.75	6.39	5.64	5.76	5.98	6.89	5.51	5.54	5.59	5.62	5.65	5.73	5.67	5.90	5.58	5.56	5.40	5.46
1371262_at	IgG-2a /// Igh	gamma-2a immunoglobulin heavy chain /// immunoglobulin heavy chain, alpha /// Ighg prot	1	DRlyp/lypnDR+/+_347	1	1	0	0	0	0	0	0	0	0	4.02	4.10	4.23	7.75	4.01	4.09	5.85	9.48	4.28	4.03	4.47	4.70	3.97	4.09	4.35	5.45	4.05	4.16	4.18	4.41
1371382_at	Flna	filamin A, alpha	1	DRlyp/lypnDR+/+_347	1	1	0	0	0	0	0	0	0	0	6.90	6.79	6.89	7.37	7.02	6.85	7.13	7.73	6.68	6.41	6.63	6.74	6.74	6.64	6.77	6.86	6.76	6.83	6.66	6.77
1371774_at	Sat1	spermidine/spermine N1-acetyl transferase 1	1	DRlyp/lypnDR+/+_347	1	1	0	0	0	0	0	0	0	0	8.73	9.17	9.22	9.36	9.06	9.15	9.84	9.78	8.65	8.90	9.20	8.75	8.65	8.85	8.85	8.97	8.45	8.79	8.84	8.65
1372013_at	Ifitm1	interferon induced transmembrane protein 1	1	DRlyp/lypnDR+/+_347	1	1	0	0	0	0	0	0	0	0	6.78	6.70	6.78	8.59	6.85	6.67	7.05	8.78	6.56	6.39	6.37	6.31	6.65	6.51	7.36	7.83	6.59	6.48	6.49	6.34
1372070_at	Ifi30	interferon gamma inducible protein 30	1	DRlyp/lypnDR+/+_347	1	1	0	0	0	0	0	0	0	0	8.88	8.83	8.85	9.55	8.94	8.88	8.86	9.97	9.06	8.97	8.88	8.78	8.90	8.95	9.01	9.02	8.98	8.95	8.77	8.68
1372182_at	Pfkp	phosphofructokinase, platelet	1	DRlyp/lypnDR+/+_347	1	1	0	0	0	0	0	0	0	0	6.65	6.87	7.14	7.55	6.56	6.87	7.12	7.28	6.85	7.22	7.10	7.05	6.78	7.12	7.12	7.00	6.90	6.95	6.95	7.10
1372200_at	Gpsm3	G-protein signaling modulator 3	1	DRlyp/lypnDR+/+_347	1	1	0	0	0	0	0	0	0	0	4.46	4.49	4.62	5.48	4.58	4.59	4.82	5.42	4.61	4.65	4.88	4.89	5.06	4.86	4.86	4.98	4.65	4.85	4.87	4.65

Affymetrix Probeset ID	Gene Symbol	Gene Name	unique BB on P7 n=822	Figure 1B P7 Venn n=1526	DRlyp/lyp STEM P7 n=712	DR+/+ STEM P7 n=936	Flyp/lyp STEM P7 n=520	F+/+ STEM P7 n=465	Figure 1B P0 Venn n=1071	DRlyp/lyp STEM P0 n=442	DR+/+ STEM P0 n=437	Flyp/lyp STEM P0 n=643	F+/+ STEM P0 n=632	Fig1D n=25	Mean log2 intensity DRlyp/lyp 20	Mean log2 intensity DRlyp/lyp 30	Mean log2 intensity DRlyp/lyp 40	Mean log2 intensity DRlyp/lyp 50	Mean log2 intensity DR+/+ 20	Mean log2 intensity DR+/+ 30	Mean log2 intensity DR+/+ 40	Mean log2 intensity DR+/+ 50	Mean log2 intensity Flyp/lyp 20	Mean log2 intensity Flyp/lyp 30	Mean log2 intensity Flyp/lyp 40	Mean log2 intensity Flyp/lyp 50	Mean log2 intensity Flyp20	Mean log2 intensity Flyp30	Mean log2 intensity Flyp40	Mean log2 intensity Flyp50	Mean log2 intensity F+/+ 20	Mean log2 intensity F+/+ 30	Mean log2 intensity F+/+ 40	Mean log2 intensity F+/+ 50
1372256_at	Crip1	cysteine-rich protein 1 (intestinal)	1	DRlyp/lypnDR+/+_347	1	1	0	0	0	0	0	0	0	0	8.47	8.59	8.53	9.26	8.53	8.39	8.81	9.94	8.03	8.04	8.01	8.26	8.31	8.11	8.14	8.44	8.26	8.08	8.07	8.10
1372846_at	Cyb561a3	cytochrome b561 family, member A3	1	DRlyp/lypnDR+/+_347	1	1	0	0	0	0	0	0	0	0	6.57	6.60	6.73	7.43	6.49	6.62	6.83	7.92	6.76	6.81	7.07	7.09	6.74	6.86	6.96	7.24	6.64	6.89	7.02	7.07
1372852_at	Ptprcap	protein tyrosine phosphatase, receptor type, C-associated protein	1	DRlyp/lypnDR+/+_347	1	1	0	0	0	0	0	0	0	0	5.04	4.82	5.16	7.08	4.98	4.99	5.74	8.15	5.03	5.05	5.29	5.39	5.10	5.26	5.32	5.85	5.02	5.10	5.15	5.27
1372930_at	Sp110	SP110 nuclear body protein	1	DRlyp/lypnDR+/+_347	1	1	0	0	0	0	0	0	0	0	7.76	7.77	7.80	8.35	7.73	7.78	7.84	8.36	7.69	7.73	7.75	7.82	7.65	7.71	7.86	7.90	7.53	7.65	7.74	7.73
1372935_at	Tmem119	transmembrane protein 119	1	DRlyp/lypnDR+/+_347	1	1	0	0	0	0	0	0	0	0	4.42	4.38	4.47	5.12	4.29	4.51	4.33	5.37	4.35	4.45	4.46	4.79	4.46	4.41	4.62	4.96	4.21	4.65	4.68	4.53
1373025_at	C1qc	complement component 1, q subcomponent, C chain	1	DRlyp/lypnDR+/+_347	1	1	0	0	0	0	0	0	0	0	5.43	5.94	6.06	7.29	5.63	6.00	6.16	7.05	5.89	5.91	6.09	6.07	5.85	5.79	6.41	6.63	5.73	5.99	6.00	5.81
1373065_at	Ptpn18	protein tyrosine phosphatase, non-receptor type 18	1	DRlyp/lypnDR+/+_347	1	1	0	0	0	0	0	0	0	0	6.83	6.81	6.82	7.83	6.74	6.84	7.10	8.55	6.65	6.67	6.73	6.55	6.86	6.81	6.87	7.09	6.67	6.64	6.80	6.49
1373175_at	Plbd1	phospholipase B domain containing 1	1	DRlyp/lypnDR+/+_347	1	1	0	0	0	0	0	0	0	0	3.92	3.92	3.91	5.23	3.79	3.72	4.20	5.84	4.01	3.94	4.26	4.15	4.01	3.92	4.49	4.75	4.03	4.09	4.20	3.94
1373421_at	Tgif1	TGFB-induced factor homeobox 1	1	DRlyp/lypnDR+/+_347	1	1	0	0	0	0	0	0	0	0	5.97	5.90	6.04	6.49	5.92	5.84	6.26	6.93	6.15	6.05	6.04	6.14	6.26	6.06	6.16	6.27	6.24	5.87	6.16	6.10
1373490_at	Gmfg	glia maturation factor, gamma	1	DRlyp/lypnDR+/+_347	1	1	0	0	0	0	0	0	0	0	5.33	5.45	5.49	7.02	5.34	5.27	5.86	8.04	5.21	5.17	5.37	5.31	5.24	5.28	5.58	6.36	5.18	5.25	5.28	5.40
1373785_at	Pld4	phospholipase D family, member 4	1	DRlyp/lypnDR+/+_347	1	1	0	0	0	0	0	0	0	0	3.48	3.62	3.56	4.08	3.64	3.49	3.69	4.00	3.56	3.62	3.54	3.72	3.42	3.62	3.53	3.91	3.58	3.68	3.60	3.55
1373881_at	Arhgdib	Rho, GDP dissociation inhibitor (GDI) beta	1	DRlyp/lypnDR+/+_347	1	1	0	0	0	0	0	0	0	0	5.98	6.31	6.22	7.55	6.12	6.28	6.67	8.63	6.14	6.23	6.45	6.46	6.01	6.32	6.44	6.74	6.23	6.29	6.43	6.38
1373957_at	Reln	reelin	1	DRlyp/lypnDR+/+_347	1	1	0	0	0	0	0	0	0	0	3.80	3.73	3.75	4.58	3.82	3.78	4.02	4.85	3.73	3.87	4.09	3.83	4.05	3.75	4.26	4.43	4.05	3.91	3.67	3.73
1374061_at	Cd302	CD302 molecule	1	DRlyp/lypnDR+/+_347	1	1	0	0	0	0	0	0	0	0	4.88	5.35	5.26	5.81	4.87	4.96	5.31	6.18	4.62	4.57	4.60	4.68	4.82	4.97	5.17	5.45	4.45	4.91	4.82	4.78
1374334_at	Igha /// LOC3	immunoglobulin heavy chain, alpha /// similar to immunoglobulin heavy chain	1	DRlyp/lypnDR+/+_347	1	1	0	0	0	0	0	0	0	0	4.52	4.73	5.27	9.72	4.69	4.96	7.06	9.48	5.01	4.79	4.95	5.04	5.43	5.32	5.70	6.46	4.59	5.45	5.66	5.36
1374544_at	MGC112715	hypothetical protein LOC690899	1	DRlyp/lypnDR+/+_347	1	1	0	0	0	0	0	0	0	0	4.55	4.94	4.82	5.87	4.59	5.00	4.93	5.85	4.64	4.52	4.71	4.71	4.39	4.51	4.82	5.00	4.55	4.76	4.94	4.74
1374838_at	Sp140	SP140 nuclear body protein	1	DRlyp/lypnDR+/+_347	1	1	0	0	0	0	0	0	0	0	5.84	5.97	6.04	6.66	5.86	5.93	6.14	6.90	5.88	5.95	5.92	6.05	6.11	5.97	6.12	6.11	5.95	6.05	5.97	5.96
1374943_at	Fam210b	family with sequence similarity 210, member B	1	DRlyp/lypnDR+/+_347	1	1	0	0	0	0	0	0	0	0	5.72	6.12	6.22	6.35	5.71	6.25	6.28	6.32	5.94	6.24	6.38	6.37	6.07	6.26	6.44	6.51	5.86	6.12	6.30	6.36
1375367_at	Pdlim2	PDZ and LIM domain 2	1	DRlyp/lypnDR+/+_347	1	1	0	0	0	0	0	0	0	0	4.25	4.31	4.38	4.88	4.41	4.31	4.59	5.26	4.17	4.12	4.12	4.24	4.27	4.15	4.33	4.28	4.30	4.19	4.13	4.25
1375989_a_at	Nfkb2	nuclear factor of kappa light polypeptide gene enhancer in B-cells 2, p49/p100	1	DRlyp/lypnDR+/+_347	1	1	0	0	0	0	0	0	0	0	4.54	4.72	4.75	5.27	4.72	4.72	4.94	5.30	4.68	4.70	4.98	4.88	4.65	4.65	4.92	4.99	4.59	4.83	4.83	4.91
1376030_at	Acap1	ArfGAP with coiled-coil, ankyrin repeat and PH domains 1	1	DRlyp/lypnDR+/+_347	1	1	0	0	0	0	0	0	0	0	3.00	3.12	3.12	3.84	2.84	3.21	3.54	4.51	2.96	3.03	3.20	3.25	2.98	2.83	3.09	3.29	3.05	3.12	3.02	3.01
1376160_at	Relb	v-rel reticuloendotheliosis viral oncogene homolog B	1	DRlyp/lypnDR+/+_347	1	1	0	0	0	0	0	0	0	0	4.46	4.42	4.41	4.94	4.38	4.49	4.72	4.89	4.47	4.57	4.69	4.80	4.56	4.77	4.76	4.86	4.57	4.91	4.81	4.83
1376255_at	Map4k1	mitogen activated protein kinase kinase kinase kinase 1	1	DRlyp/lypnDR+/+_347	1	1	0	0	0	0	0	0	0	0	3.92	4.27	4.44	5.13	4.10	4.19	4.44	5.00	3.94	4.11	4.32	4.35	4.15	4.37	4.09	4.42	4.07	4.11	4.21	4.40
1376327_at	Tnfrsf14	tumor necrosis factor receptor superfamily, member 14	1	DRlyp/lypnDR+/+_347	1	1	0	0	0	0	0	0	0	0	3.13	3.24	3.32	5.21	3.19	3.21	3.73	6.27	3.26	3.16	3.34	3.27	3.41	3.17	3.45	3.99	3.14	3.51	3.36	3.40
1376688_a_at	LOC1009104	paired immunoglobulin-like type 2 receptor alpha-like	1	DRlyp/lypnDR+/+_347	1	1	0	0	0	0	0	0	0	0	3.93	4.29	4.21	4.89	3.64	4.10	4.14	5.24	3.84	4.03	4.17	4.33	3.94	4.12	4.33	4.57	3.74	4.21	4.34	3.89
1376708_at	---	---	1	DRlyp/lypnDR+/+_347	1	1	0	0	0	0	0	0	0	0	5.41	5.42	5.48	6.02	5.24	5.50	5.53	6.01	6.32	6.12	6.13	6.19	6.21	6.03	6.16	6.42	5.80	5.74	5.89	5.84
1376766_at	Fmnl1	formin-like 1	1	DRlyp/lypnDR+/+_347	1	1	0	0	0	0	0	0	0	0	4.61	4.84	5.03	5.44	4.69	4.88	5.15	5.90	4.92	5.02	5.00	5.12	5.59	5.19	5.26	5.29	5.07	5.05	5.02	4.97
1376795_at	Pik3ap1	phosphoinositide-3-kinase adaptor protein 1	1	DRlyp/lypnDR+/+_347	1	1	0	0	0	0	0	0	0	0	4.97	4.90	5.10	6.00	4.74	4.97	5.36	6.48	4.77	4.79	4.94	5.06	4.91	4.93	5.08	5.30	4.84	4.72	5.05	5.06
1376835_at	---	---	1	DRlyp/lypnDR+/+_347	1	1	0	0	0	0	0	0	0	0	6.28	6.40	6.62	6.93	6.40	6.54	6.65	7.15	6.39	6.51	6.62	6.53	6.58	6.45	6.64	6.75	6.52	6.50	6.57	6.68
1376895_at	Il16	interleukin 16	1	DRlyp/lypnDR+/+_347	1	1	0	0	0	0	0	0	0	0	3.51	3.50	3.64	4.65	3.55	3.63	3.99	5.71	3.60	3.49	3.65	3.45	3.63	3.47	3.83	4.02	3.57	3.42	3.71	3.82
1377023_at	Dusp2	dual specificity phosphatase 2	1	DRlyp/lypnDR+/+_347	1	1	0	0	0	0	0	0	0	0	4.09	4.10	4.19	5.32	4.12	4.20	4.55	6.03	4.32	4.14	4.07	4.31	4.10	4.24	4.15	4.44	4.04	4.34	4.25	4.39
1377110_at	Arl5c	ADP-ribosylation factor-like 5C	1	DRlyp/lypnDR+/+_347	1	1	0	0	0	0	0	0	0	0	2.07	2.17	2.21	3.44	2.16	2.03	2.66	4.29	2.07	2.28	2.32	2.24	2.26	2.07	2.21	2.55	2.19	2.24	2.13	2.47
1377146_at	Vip	vasoactive intestinal peptide	1	DRlyp/lypnDR+/+_347	1	1	0	0	0	0	0	0	0	0	3.16	3.40	4.47	4.87	3.34	3.60	5.29	5.05	2.86	4.03	3.29	3.39	2.65	4.91	2.67	2.78	3.29	2.79	2.64	5.41
1377153_a_at	Klhl6	kelch-like family member 6	1	DRlyp/lypnDR+/+_347	1	1	0	0	0	0	0	0	0	0	3.89	3.83	3.89	5.05	4.00	3.93	4.20	6.33	3.67	3.59	3.77	3.94	3.74	3.87	3.89	4.28	3.80	3.91	3.85	3.90
1377239_at	Apbb1ip	amyloid beta (A4) precursor protein-binding, family B, member 1 interacting protein	1	DRlyp/lypnDR+/+_347	1	1	0	0	0	0	0	0	0	0	4.21	4.54	4.51	5.96	4.36	4.41	4.87	6.59	4.87	4.58	4.70	4.61	4.80	4.54	4.77	4.91	4.51	4.59	4.63	4.41
1377334_at	RT1-Ba	RT1 class II, locus Ba	1	DRlyp/lypnDR+/+_347	1	1	0	0	0	0	0	0	0	0	5.76	6.61	6.82	9.05	5.87	6.38	7.14	9.56	6.18	6.60	6.94	7.09	4.50	4.97	5.39	5.83	6.26	6.76	6.92	6.88
1377492_at	Rprml	reprimo-like	1	DRlyp/lypnDR+/+_347	1	1	0	0	0	0	0	0	0	0	5.94	6.30	6.69	6.76	5.98	6.15	6.76	6.74	5.40	5.66	5.51	5.84	5.65	5.75	5.85	5.88	5.73	5.64	5.80	5.93
1377759_at	Bid	BH3 interacting domain death agonist	1	DRlyp/lypnDR+/+_347	1	1	0	0	0	0	0	0	0	0	4.86	5.07	5.11	6.06	4.87	5.12	5.42	6.84	4.88	4.99	4.98	4.98	4.84	4.83	5.11	5.23	4.70	5.05	4.88	4.92
1377916_at	Slfn2	schlafen 2	1	DRlyp/lypnDR+/+_347	1	1	0	0	0	0	0	0	0	0	5.22	5.35	5.44	7.04	5.26	5.26	5.91	7.62	5.13	5.39	5.30	5.50	5.28	5.45	5.54	6.16	5.12	5.29	5.44	5.53
1377943_at	---	---	1	DRlyp/lypnDR+/+_347	1	1	0	0	0	0	0	0	0	0	2.72	2.95	3.09	3.96	2.80	2.94	2.91	4.12	3.01	2.95	3.10	3.22	2.74	2.92	3.27	3.33	2.79	2.93	3.07	3.06
1377950_at	LOC1009109	interferon-inducible GTPase 1-like /// similar to interferon-inducible GTPase	1	DRlyp/lypnDR+/+_347	1	1	0	0	0	0	0	0	0	0	3.26	3.24	3.74	6.02	3.20	3.44	3.31	4.91	2.55	2.64	2.67	2.72	2.75	2.72	2.66	2.66	2.49	2.65	2.77	2.60
1378047_at	Spic	Spi-C transcription factor (Spi-1/PU.1 related)	1	DRlyp/lypnDR+/+_347	1	1	0	0	0	0	0	0	0	0	2.46	2.48	2.36	3.23	2.38	2.35	2.61	3.72	2.43	2.40	2.55	2.43	2.41	2.34	2.68	3.07	2.39	2.32	2.42	2.39
1378150_at	---	---	1	DRlyp/lypnDR+/+_347	1	1	0	0	0	0	0	0	0	0	4.00	4.17	4.03	4.71	4.10	3.96	4.37	5.19	4.01	4.14	4.16	4.14	3.76	4.04	4.28	4.33	3.88	4.25	4.12	4.03
1378419_at	Rnase2	ribonuclease, RNase A family, 2 (liver, eosinophil-derived neurotoxin)	1	DRlyp/lypnDR+/+_347	1	1	0	0	0	0	0	0	0	0	2.32	2.29	2.23	3.22	2.14	2.31	2.38	3.15	2.20	2.23	2.26	2.19	2.08	2.35	2.25	2.26	2.13	2.22	2.13	2.36
1378451_at	Cyba	cytochrome b-245, alpha polypeptide	1	DRlyp/lypnDR+/+_347	1	1	0	0	0	0	0	0	0	0	3.82	3.79	3.63	3.97	3.90	3.81	3.79	3.79	3.74	3.62	3.92	3.93	3.80	3.76	3.81	4.04	3.68	3.93	3.80	3.82
1379295_at	Gngt2	guanine nucleotide binding protein (G protein), gamma transducing activity polypeptide 2	1	DRlyp/lypnDR+/+_347	1	1	0	0	0	0	0	0	0	0	5.16	5.29	5.27	5.95	5.17	5.22	5.26	6.16	4.98	5.11	5.06	5.33	5.06	5.25	5.40	5.67	5.16	5.20	5.32	5.20
1379563_at	Irf5	interferon regulatory factor 5	1	DRlyp/lypnDR+/+_347	1	1	0	0	0	0	0	0	0	0	4.23	4.35	4.30	4.76	4.37	4.37	4.51	4.99	4.24	4.28	4.24	4.39	4.11	4.30	4.35	4.41	4.26	4.32	4.28	4.38
1379606_at	Rab30	RAB30, member RAS oncogene family	1	DRlyp/lypnDR+/+_347	1	1	0	0	0	0	0	0	0	0	4.76	4.78	4.72	5.33	4.63	4.73	4.87	6.00	4.75	4.80	4.74	4.76	4.93	4.91	4.85	4.99	4.67	4.84	4.71	5.10
1379653_a_at	Sh2d1a	SH2 domain containing 1A	1	DRlyp/lypnDR+/+_347	1	1	0	0	0	0	0	0	0	0	2.08	1.94	2.03	2.49	1.93	1.88	2.12	3.88	1.97	1.98	1.91	1.93	1.84	1.93	1.92	2.46	1.94	1.85	1.96	2.12
1379659_at	Bmp2k	BMP-2 inducible kinase	1	DRlyp/lypnDR+/+_347	1	1	0	0	0	0	0	0	0	0	4.29	4.40	4.53	5.04	4.35	4.38	4.57	5.45	4.68	4.80	4.98	4.97	5.03	4.87	4.93	5.00	4.67	4.90	5.00	4.91
1379732_at	Stx11	syntaxin 11	1	DRlyp/lypnDR+/+_347	1	1	0	0	0	0	0	0	0	0	2.38	2.55	2.57	3.22	2.55	2.67	2.67	3.84	2.45	2.35	2.48	2.66	2.35	2.57	2.64	2.76	2.41	2.42	2.65	2.62
1379791_at	Cd3e	CD3 molecule, epsilon	1	DRlyp/lypnDR+/+_347	1	1	0	0	0	0	0	0	0	0	3.47	3.43	3.44	4.75	3.50	3.76	4.11	5.94	3.40	3.39	3.62	3.63	3.34	3.39	3.56	4.01	3.37	3.46	3.57	3.59
1379957_at	Slfn13	schlafen family member 13	1	DRlyp/lypnDR+/+_347	1	1	0	0	0	0	0	0	0	0	5.00	4.95	5.11	5.67	4.81	5.09	5.16	5.71	5.52	5.52	5.57	5.46	5.28	5.62	5.55	5.85	5.41	5.52	5.73	5.40
1380021_at	Dennd2d	DENN/MADD domain containing 2D	1	DRlyp/lypnDR+/+_347	1	1	0	0	0	0	0	0	0	0	4.64	5.01	5.13	5.86	4.90	5.05	5.36	6.25	4.97	4.86	5.03	4.99	4.79	4.92	5.06	5.38	4.72	4.95	4.76	5.18
1380063_at	Ch25h	cholesterol 25-hydroxylase	1	DRlyp/lypnDR+/+_347	1	1	0	0	0	0	0	0	0	0	5.26	5.32	5.36	5.76	5.26	5.35	5.51	6.39	5.07	5.09	5.30	5.30	5.12	5.19	5.53	5.93	5.17	5.35	5.40	5.29
1380333_at	Ptpn22	protein tyrosine phosphatase, non-receptor type 22 (lymphoid)	1	DRlyp/lypnDR+/+_347	1	1	0	0	0	0	0	0	0	0	2.96	3.23	2.85	3.19	2.93	3.06	3.10	3.37	3.22	3.03	3.19	3.41	3.10	2.97	3.00	3.03	3.03	3.15	3.11	3.16
1380377_at	Btk	Bruton agammaglobulinemia tyrosine kinase	1	DRlyp/lypnDR+/+_347	1	1	0	0	0	0	0	0	0	0	2.63	2.59	2.55	4.27	2.62	2.73	3.04	5.43	2.65	2.41	2.80	2.82	2.51	2.67	2.95	3.37	2.64	2.69	2.72	2.82
1380432_at	Cmah	cytidine monophospho-N-acetylneuraminic acid hydroxylase	1	DRlyp/lypnDR+/+_347	1	1	0	0	0	0	0	0	0	0	2.28	2.44	2.41	4.31	2.25	2.38	2.66	5.08	2.41	2.26	2.62	2.34	2.48	2.42	2.43	2.82	2.43	2.33	2.59	2.51
1380521_at	Hcls1	hematopoietic cell specific Lyn substrate 1	1	DRlyp/lypnDR+/+_347	1	1	0	0	0	0	0	0	0	0	6.04	6.09	5.99	7.01	6.10	6.25	6.32	7.44	6.00	6.02	6.14	6.41	5.96	6.08	6.14	6.25	6.04	6.08	6.13	6.22
1380537_at	Nckap1l	NCK associated protein 1 like	1	DRlyp/lypnDR+/+_347	1	1	0	0	0	0	0	0	0	0	3.58	3.55	3.39	4.74	3.42	3.59	3.68	5.59	3.67	3.68	3.59	3.88	3.64	3.73	3.70	3.97	3.82	3.67	3.82	3.70
1380930_at	RGD1564142	similar to chromosome 10 open reading frame 64	1	DRlyp/lypnDR+/+_347	1	1	0	0	0	0	0	0	0	0	2.52	2.74	2.71	3.82	2.76	2.64	2.76	3.90	2.65	2.64	2.64	2.89	2.50	2.67	2.65	3.08	2.65	2.58	2.75	2.72
1381219_at	Myh11	myosin, heavy chain 11, smooth muscle	1	DRlyp/lypnDR+/+_347	1	1	0	0	0	0	0	0	0	0	2.80	2.71	2.68	2.76	2.66	2.62	2.78	2.84	2.58	2.50	2.50	2.66	2.60	2.47	2.66	2.57	2.56	2.54	2.56	2.49
1381311_at	Emr1	EGF-like module containing, mucin-like, hormone receptor-like 1	1	DRlyp/lypnDR+/+_347	1	1	0	0	0	0	0	0	0	0	2.46	2.58	2.54	3.05	2.44	2.45	2.39	2.98	2.54	2.71	2.78	2.90	2.64	2.71	2.72	2.98	2.48	2.83	3.26	2.73
1381327_a_at	Cr2	complement component (3d/Epstein Barr virus) receptor 2	1	DRlyp/lypnDR+/+_347	1	1	0	0	0	0	0	0	0	0	1.74	1.83	1.86	4.44	1.90	1.85	2.18	5.90	2.41	1.96	2.26	2.09	1.78	1.89	1.98	2.47	1.74	1.90	2.03	2.01
1381328_at	Cr2	complement component (3d/Epstein Barr virus) receptor 2	1	DRlyp/lypnDR+/+_347	1	1	0	0	0	0	0	0	0	0	1.86	1.85	1.85	3.71	1.77	1.81	2.00	5.99	1.95	1.94	1.92	1.84	1.81	1.95	1.96	2.19	1.95	1.86	1.88	1.89
1381357_at	Ptpn22	protein tyrosine phosphatase, non-receptor type 22 (lymphoid)	1	DRlyp/lypnDR+/+_347	1	1	0	0	0	0	0	0	0	0	1.85	1.97	2.07	3.20	1.90	1.97	2.52	4.81	2.02	1.98	2.03	2.07	1.89	1.97	2.06	2.51	1.91	1.92	2.02	2.03
1381399_at	LOC1003606	ribosomal protein L37-like /// ribosomal protein L37-like /// ribosomal protein L37-like /// rib	1	DRlyp/lypnDR+/+_347	1	1	0	0	0	0	0	0	0	0	3.26	3.30	3.22	3.79	3.00	2.88	3.29	3.86	3.35	3.28	3.42	3.50	3.47	3.43	3.38	3.45	3.40	3.29	3.60	3.51
1381593_x_at	RT1-Ba	RT1 class II, locus Ba	1	DRlyp/lypnDR+/+_347	1	1	0	0	0	0	0	0	0	0	7.13	7.05	6.76	6.95	6.97	7.11	7.07	6.94	7.12	6.97	7.06	7.28	7.12	7.41	7.18	7.07	6.88	7.39	7.05	7.25
1381875_at	Nmi	N-myc (and STAT) interactor	1	DRlyp/lypnDR+/+_347	1	1	0	0	0	0	0	0	0	0	5.06	5.14	5.21	5.74	4.90	4.98	5.11	5.81	5.55	5.66	5.54	5.74	5.58	5.60	5.73	5.78	5.45	5.60	5.68	5.60
1382026_at	Arhgap9	Rho GTPase activating protein 9	1	DRlyp/lypnDR+/+_347	1	1	0	0	0	0	0	0	0	0	2.70	2.92	2.93	5.13	3.12	3.09	3.60	5.99	2.91	3.04	3.04	3.11	3.12	3.04	3.19	3.92	3.13	3.02	2.92	3.11
1382043_at	Unc93b1	unc-93 homolog B1 (C. elegans)	1	DRlyp/lypnDR+/+_347	1	1	0	0	0	0	0	0	0	0	6.57	6.71	6.74	7.51	6.55	6.69	6.68	7.65	6.62	6.70	6.72	6.79	6.66	6.74	6.72	6.94	6.63	6.76	6.84	6.70
1382192_at	Lyve1	lymphatic vessel endothelial hyaluronan receptor 1	1	DRlyp/lypnDR+/+_347	1	1	0	0	0	0	0	0	0	0	3.09	2.91	2.99	5.17	3.10	2.97	3.33	5.36	2.80	3.20	2.95	2.72	2.96	3.32	4.13	5.78	3.02	3.17	2.88	3.00
1382274_at	Rarres1	retinoic acid receptor responder (tazarotene induced) 1	1	DRlyp/lypnDR+/+_347	1	1	0	0	0	0	0	0	0	0	5.22	5.39	5.47	7.19	5.50	5.32	5.68	7.67	5.26	5.32	5.31	5.41	5.49	5.48	5.66	6.23	5.18	5.45	5.22	5.58
1382283_at	Wipf1	WAS/WASL interacting protein family, member 1	1	DRlyp/lypnDR+/+_347	1	1	0	0	0	0	0	0	0	0	4.31	4.27	4.48	5.58	4.39	4.34	4.56	6.41	4.47	4.07	4.09	4.29	4.56	4.10	4.26	4.63	4.27	4.23	4.22	4.22
1382337_at	Stap1	signal transducing adaptor family member 1	1	DRlyp/lypnDR+/+_347	1	1	0	0	0	0	0	0	0	0	2.29	2.24	2.49	3.05	2.27	2.14	2.19	3.66	2.28	2.22	2.45	2.26	2.09	2.30	2.30	2.65	2.16	2.19	2.26	2.48
1382410_at	Cd209b	CD209b antigen	1	DRlyp/lypnDR+/+_347	1	1	0	0	0	0	0	0	0	0	3.73	4.00	3.99	7.71	3.94	4.06	5.04	7.70	3.94	3.98	5.76	4.32	4.02	4.12	5.41	7.11	3.98	4.24	4.15	4.45
1382442_at	Sept6	septin 6	1	DRlyp/lypnDR+/+_347	1	1	0	0	0	0	0	0	0	0	2.85	2.72	2.79	4.16	2.70	2.83	3.22	5.05	2.65	2.77	2.79	2.95	2.72	2.85	2.95	3.15	2.76	2.80	2.95	2.61
1382546_at	Phf11	PHD finger protein 11	1	DRlyp/lypnDR+/+_347	1	1	0	0	0	0	0	0	0	0	4.69	5.01	5.05	5.78	5.01	4.88	5.46	6.03	5.00	4.87	5.00	5.20	5.00	5.04	5.06	5.31	5.04	5.04	5.20	5.00
1382566_at	Il7r /// LOC10	interleukin 7 receptor /// interleukin-7 receptor subunit alpha-like	1	DRlyp/lypnDR+/+_347	1	1	0	0	0	0	0	0	0	0	3.74	3.66	3.80	4.57	3.68	3.73	4.20	4.84	3.57	3.75	3.85	3.80	3.62	3.69	3.99	4.44	3.88	3.83	4.11	3.96
1382622_at	Cst7	cystatin F (leukocystatin)	1	DRlyp/lypnDR+/+_347	1	1	0	0	0	0	0	0	0	0	2.94	2.98	3.14	4.91	2.94	3.11	3.28	4.34	2.90	3.02	3.09	3.34	2.87	3.06	3.51	3.58	2.99	3.10	3.14	3.10
1382663_at	Batf	basic leucine zipper transcription factor, ATF-like	1	DRlyp/lypnDR+/+_347	1	1	0	0	0	0	0	0	0	0	3.69	3.84	3.85	4.84	3.81	3.77	4.00	5.35	3.84	3.91	3.89	4.22	3.94	3.81	3.98	4.23	3.79	3.93	3.97	3.98
1382732_at	Lyve1	lymphatic vessel endothelial hyaluronan receptor 1	1	DRlyp/lypnDR+/+_347	1	1	0	0	0	0	0	0	0	0	2.62	2.81	2.67	3.67	2.80	2.80	2.76	3.74	2.69	2.80	2.82	2.70	2.52	2.77	3.46	4.10	2.69	2.67	2.72	2.64
1382803_at	Map4k1	mitogen activated protein kinase kinase kinase kinase 1	1	DRlyp/lypnDR+/+_347	1	1	0	0	0	0	0	0	0	0	3.23	3.22	3.33	3.63	3.44	3.60	3.60	3.79	3.35	3.37	3.35	3.53	3.38	3.52	3.47	3.52	3.41	3.46	3.38	3.63
1383015_at	Tbc1d10c	TBC1 domain family, member 10C	1	DRlyp/lypnDR+/+_347	1	1	0	0	0	0	0	0	0	0	5.15	5.33	5.31	5.86	5.34	4.98	5.32	6.61	5.23	5.19	5.27	5.02	5.08	5.00	5.41	5.51	5.14	5.07	5.13	5.21
1383131_at	Itgb2	integrin, beta 2	1	DRlyp/lypnDR+/+_347	1	1	0	0	0	0	0	0	0	0	4.32	4.65	4.73	6.60	4.62	4.60	4.92	7.42	4.31	4.38	4.79	4.77	4.36	4.43	4.78	5.12	4.38	4.52	4.72	4.76
1383163_at	Igj	immunoglobulin joining chain	1	DRlyp/lypnDR+/+_347	1	1	0	0	0	0	0	0	0	0	4.03	4.31	4.32	7.34	4.01	4.33	5.21	8.79	4.59	4.56	4.44	4.79	4.55	4.56	4.58	5.28	4.18	4.36	4.76	4.75
1383241_at	C1r	complement component 1, r subcomponent	1	DRlyp/lypnDR+/+_347	1	1	0	0	0	0	0	0	0	0	7.17	7.25	7.34	8.26	7.20	7.16	7.19	7.89	6.50	6.75	6.63	6.83	6.68	6.71	6.92	7.29	6.75	6.74	6.81	6.80
1383291_at	C7 /// Tubb4	complement component 7 /// tubulin, beta 4B class IVb	1	DRlyp/lypnDR+/+_347	1	1	0	0	0	0	0	0	0	0	5.48	5.71	5.52	6.30	5.44	5.34	5.52	6.17	4.80	4.97	4.85	4.87	5.34	4.93	5.37	6.18	5.06	5.05	4.97	5.00
1383302_at	Dnajb1	DnaJ (Hsp40) homolog, subfamily B, member 1	1	DRlyp/lypnDR+/+_347	1	1	0	0	0	0	0	0	0	0	8.05	8.26	8.20	8.73	8.06	7.75	9.17	9.09	8.07	7.96	8.00	8.26	7.36	8.52	8.77	8.28	8.32	8.04	7.69	8.12
1383320_at	Lck	lymphocyte-specific protein tyrosine kinase	1	DRlyp/lypnDR+/+_347	1	1	0	0	0	0	0	0	0	0	6.07	6.10	6.12	7.15	6.11	6.15	6.68	8.24	6.28	6.22	6.27	6.42	6.53	6.30	6.45	6.72	6.38	6.29	6.39	6.37
1383407_at	Sash3	SAM and SH3 domain containing 3	1	DRlyp/lypnDR+/+_347	1	1	0	0	0	0	0	0	0	0	3.74	3.92	4.09	4.88	3.89	4.02	4.34	6.18	3.94	3.92	4.19	4.21	3.80	3.97	4.11	4.39	3.93	3.97	4.00	4.15
1383426_at	Pstpip1	proline-serine-threonine phosphatase-interacting protein 1	1	DRlyp/lypnDR+/+_347	1	1	0	0	0	0	0	0	0	0	3.97	4.05	3.98	4.57	4.12	4.03	4.09	5.23	3.77	3.94	4.00	3.96	3.82	4.01	3.90	4.01	3.89	3.79	3.90	3.89
1383506_at	Bcl11a	B-cell CLL/lymphoma 11A (zinc finger protein)	1	DRlyp/lypnDR+/+_347	1	1	0	0	0	0	0	0	0	0	2.95	2.96	2.90	3.60	3.00	3.16	3.11	4.25	3.05	2.91	3.18	3.16	2.83	3.10	3.05	3.01	2.89	2.96	3.11	3.21
1383564_at	Irf7	interferon regulatory factor 7	1	DRlyp/lypnDR+/+_347	1	1	0	0	0	0	0	0	0	0	5.55	5.65	6.08	7.30	5.71	5.79	5.94	6.28	5.65	5.80	5.83	6.11	5.89	5.94	6.14	6.46	5.67	5.78	6.03	6.08
1383658_at	Laptm5	lysosomal protein transmembrane 5	1	DRlyp/lypnDR+/+_347	1	1	0	0	0	0	0	0	0	0	6.43	6.50	6.61	7.59	6.39	6.53	6.88	8.11	6.47	6.36	6.64	6.74	6.33	6.34	6.66	7.05	6.31	6.62	6.57	6.67
1383786_at	Rab32	RAB32, member RAS oncogene family	1	DRlyp/lypnDR+/+_347	1	1	0	0	0	0	0	0	0	0	4.78	4.68	4.74	5.24	4.79	4.80	4.80	5.42	4.60	4.51	4.72	4.72	4.96	4.64	5.14	5.16	4.82	4.82	4.83	4.89
1383833_at	Fam55b	family with sequence similarity 55, member B	1	DRlyp/lypnDR+/+_347	1	1	0	0	0	0	0	0	0	0	3.33	3.42	3.36	3.92	3.45	3.05	3.54	4.61	3.37	3.33	3.39	3.32	3.51	3.44	3.39	3.56	3.42	3.49	3.35	3.36
1383863_at	Lmo2	LIM domain only 2	1	DRlyp/lypnDR+/+_347	1	1	0	0	0	0	0	0	0	0	7.39	7.42	7.46	7.90	7.37	7.49	7.45	8.25	7.10	7.32	7.42	7.50	7.31	7.17	7.42	7.57	7.28	7.45	7.53	7.53
1383906_at	Neurl3	neuralized homolog 3 (Drosophila)	1	DRlyp/lypnDR+/+_347	1	1	0	0	0	0	0	0	0	0	4.55	4.61	4.77	5.93	4.62	4.64	4.89	5.77	4.58	4.63	4.62	4.74	4.85	4.49	4.89	5.07	4.37	4.62	4.54	4.68
1383952_at	Mical1	microtubule associated monooxygenase, calponin and LIM domain containing 1	1	DRlyp/lypnDR+/+_347	1	1	0	0	0	0	0	0	0	0	3.58	3.65	3.65	4.21	3.76	3.71	3.90	4.63	3.75	3.62	3.58	3.50	3.63	3.65	3.76	4.02	3.60	3.62	3.70	3.83
1384017_at	---	---	1	DRlyp/lypnDR+/+_347	1	1	0	0	0	0	0	0	0	0	5.24	5.24	5.13	5.81	5.19	5.00	5.23	6.11	4.88	4.73	5.02	4.95	5.06	4.96	5.14	5.31	4.88	4.99	5.10	5.20
1384208_at	Igj	immunoglobulin joining chain	1	DRlyp/lypnDR+/+_347	1	1	0	0	0	0	0	0	0	0	2.81	2.94	2.86	3.46	3.13	2.95	3.29	3.95	2.88	2.92	3.07	3.24	2.97	3.05	2.93	3.01	3.08	2.98	2.93	3.06
1384218_at	LOC362795	immunoglobulin G heavy chain	1	DRlyp/lypnDR+/+_347	1	1	0	0	0	0	0	0	0	0	3.65	3.69	3.67	5.06	3.70	3.69	4.34	6.98	3.60	3.64	3.83	3.86	3.54	3.74	3.72	3.92	3.62	3.92	3.78	3.84
1384292_at	Dok1	docking protein 1	1	DRlyp/lypnDR+/+_347	1	1	0	0	0	0	0	0	0	0	4.25	4.66	4.54	4.90	4.40	4.54	4.48	5.05	4.52	4.43	4.47	4.41	4.54	4.53	4.58	4.49	4.49	4.50	4.58	4.54
1384298_at	Myo1f	myosin IF	1	DRlyp/lypnDR+/+_347	1	1	0	0	0	0	0	0	0	0	3.28	3.34	3.47	4.09	3.35	3.39	3.50	4.00	3.19	3.39	3.43	3.54	3.34	3.18	3.49	3.89	3.41	3.48	3.43	3.41
1384350_at	Nckap1l	NCK associated protein 1 like	1	DRlyp/lypnDR+/+_347	1	1	0	0	0	0	0	0	0	0	3.58	3.59	3.64	4.55	3.60	3.64	3.97	5.39	3.65	3.62	3.58	3.76	3.57	3.63	3.79	3.87	3.51	3.75	3.76	3.62
1384476_at	Cmah	cytidine monophospho-N-acetylneuraminic acid hydroxylase	1	DRlyp/lypnDR+/+_347	1	1	0	0	0	0	0	0	0	0	5.02	4.92	4.91	5.42	4.90	4.96	5.17	5.74	4.90	5.03	5.16	5.20	4.96	5.05	5.15	5.37	5.00	5.04	5.15	5.25
1384555_at	Dennd1c	DENN/MADD domain containing 1C	1	DRlyp/lypnDR+/+_347	1	1	0	0	0	0	0	0	0	0	4.15	4.18	4.19	4.93	4.10	4.24	4.62	5.58	4.43	4.27	4.47	4.40	4.19	4.29	4.39	4.63	4.30	4.30	4.45	4.41
1384837_at	Cd69	Cd69 molecule	1	DRlyp/lypnDR+/+_347	1	1	0	0	0	0	0	0	0	0	2.88	3.01	3.24	6.84	3.02	3.25	5.15	8.71	3.20	3.06	3.18	3.46	3.03	3.05	3.30	5.04	3.09	3.31	3.13	3.73
1385019_at	Bank1	B-cell scaffold protein with ankyrin repeats 1	1	DRlyp/lypnDR+/+_347	1	1	0	0	0	0	0	0	0	0	3.82	3.82	3.60	5.74	3.77	3.77	4.18	7.08	3.84	3.73	4.04	3.81	3.73	3.75	3.92	4.54	3.65	3.80	3.89	3.95
1385047_x_at	LOC685048 /	similar to paired immunoglobin-like type 2 receptor beta /// similar to cell surface receptor F	1	DRlyp/lypnDR+/+_347	1	1	0	0	0	0	0	0	0	0	4.41	4.55	4.69	4.93	4.45	4.67	4.75	5.30	4.73	4.46	4.66	4.79	4.27	4.56	4.89	5.03	4.61	4.78	4.61	4.73
1385051_at	Gbp4 /// LOC	guanylate binding protein 4 /// guanylate-binding protein 4-like	1	DRlyp/lypnDR+/+_347	1	1	0	0	0	0	0	0	0	0	1.96	1.89	1.91	3.70	2.04	1.95	2.18	3.15	1.93	1.83	2.08	2.15	2.12	2.00	2.10	2.54	2.15	2.03	2.05	2.11
1385160_at	Stab2	stabilin 2	1	DRlyp/lypnDR+/+_347	1	1	0	0	0	0	0	0	0	0	4.56	4.46	4.51	5.07	4.55	4.60	4.81	5.11	4.82	4.72	4.84	4.73	4.72	4.69	4.82	5.29	4.71	4.68	4.91	4.78
1385213_at	Epsti1	epithelial stromal interaction 1 (breast)	1	DRlyp/lypnDR+/+_347	1	1	0	0	0	0	0	0	0	0	3.19	3.32	3.43	4.63	3.29	3.16	3.66	5.16	3.19	3.19	3.29	3.39	3.21	3.16	3.48	3.83	3.18	3.17	3.24	3.33
1385440_at	Hcst	hematopoietic cell signal transducer	1	DRlyp/lypnDR+/+_347	1	1	0	0	0	0	0	0	0	0	3.00	3.46	3.33	4.36	3.09	3.13	3.60	4.80	3.08	3.15	3.47	3.56	3.10	3.24	3.29	3.91	3.04	3.32	3.04	3.34
1385441_at	Vpreb3	pre-B lymphocyte 3	1	DRlyp/lypnDR+/+_347	1	1	0	0	0	0	0	0	0	0	4.37	4.31	4.49	5.02	4.50	4.71	4.64	5.46	4.44	4.63	4.50	4.54	4.48	4.50	4.39	4.54	4.56	4.68	4.58	4.53
1385700_at	Cr2	complement component (3d/Epstein Barr virus) receptor 2	1	DRlyp/lypnDR+/+_347	1	1	0	0	0	0	0	0	0	0	3.25	3.38	3.59	5.06	3.42	3.36	3.72	6.45	3.45	3.42	3.35	3.59	3.26	3.32	3.50	3.87	3.11	3.38	3.19	3.69

Affymetrix Probeset ID	Gene Symbol	Gene Name	unique BB on P7 n=822	Figure 1B P7 Venn n=1526	DRlyp/lyp STEM P7 n=712	DR+/+ STEM P7 n=936	Flyp/lyp STEM P7 n=520	F+/+ STEM P7 n=465	Figure 1B P0 Venn n=1071	DRlyp/lyp STEM P0 n=442	DR+/+ STEM P0 n=437	Flyp/lyp STEM P0 n=643	F+/+ STEM P0 n=632	Fig1D n=25	Mean log2 intensity DRlyp/lyp 20	Mean log2 intensity DRlyp/lyp 30	Mean log2 intensity DRlyp/lyp 40	Mean log2 intensity DRlyp/lyp 50	Mean log2 intensity DR+/+ 20	Mean log2 intensity DR+/+ 30	Mean log2 intensity DR+/+ 40	Mean log2 intensity DR+/+ 50	Mean log2 intensity Flyp/lyp 20	Mean log2 intensity Flyp/lyp 30	Mean log2 intensity Flyp/lyp 40	Mean log2 intensity Flyp/lyp 50	Mean log2 intensity Flyp20	Mean log2 intensity Flyp30	Mean log2 intensity Flyp40	Mean log2 intensity Flyp50	Mean log2 intensity F+/+ 20	Mean log2 intensity F+/+ 30	Mean log2 intensity F+/+ 40	Mean log2 intensity F+/+ 50
1385702_at	Ifi204	interferon activated gene 204	1	DRlyp/lypnDR+/+_347	1	1	0	0	0	0	0	0	0	0	2.54	2.54	2.57	3.89	2.56	2.55	2.59	3.54	2.53	2.63	2.81	2.77	2.77	2.50	2.88	3.35	2.77	2.79	2.62	2.75
1385813_at	Mzb1	marginal zone B and B1 cell-specific protein	1	DRlyp/lypnDR+/+_347	1	1	0	0	0	0	0	0	0	0	3.57	3.73	3.81	6.43	3.68	3.91	4.44	7.64	3.97	3.69	4.13	4.10	3.93	3.81	3.94	4.32	4.01	3.84	3.99	4.08
1385831_at	Sash3	SAM and SH3 domain containing 3	1	DRlyp/lypnDR+/+_347	1	1	0	0	0	0	0	0	0	0	2.04	2.10	2.15	2.41	2.08	2.04	2.13	2.83	2.12	2.23	2.16	2.22	2.04	2.01	2.36	2.07	2.03	2.12	2.32	2.12
1385832_s_at	Sash3	SAM and SH3 domain containing 3	1	DRlyp/lypnDR+/+_347	1	1	0	0	0	0	0	0	0	0	3.15	3.32	3.36	5.90	3.17	3.48	4.14	7.38	3.53	3.10	3.62	3.65	3.12	3.12	3.62	4.44	3.07	3.46	3.41	3.52
1385962_x_at	Blk	B lymphoid tyrosine kinase	1	DRlyp/lypnDR+/+_347	1	1	0	0	0	0	0	0	0	0	3.09	3.11	3.21	3.85	3.21	3.22	3.29	4.60	3.12	3.14	3.20	3.30	3.00	3.19	3.12	3.31	3.16	3.17	3.11	3.32
1386052_at	---	---	1	DRlyp/lypnDR+/+_347	1	1	0	0	0	0	0	0	0	0	2.02	2.03	2.20	3.05	1.85	2.10	2.36	2.65	2.01	2.04	2.03	1.94	2.03	1.93	2.03	2.72	1.98	1.95	2.09	2.12
1386879_at	Lgals3	lectin, galactoside-binding, soluble, 3	1	DRlyp/lypnDR+/+_347	1	1	0	0	0	0	0	0	0	0	6.65	6.61	6.80	7.87	6.46	6.53	7.11	7.69	6.38	6.88	6.36	6.68	6.50	6.40	6.69	6.97	6.56	6.21	6.46	6.38
1386890_at	S100a10	S100 calcium binding protein A10	1	DRlyp/lypnDR+/+_347	1	1	0	0	0	0	0	0	0	0	7.89	8.04	8.09	8.45	7.93	7.96	8.21	8.68	7.55	7.95	7.71	7.90	7.31	7.56	7.77	7.91	7.59	7.65	7.65	7.77
1386925_at	Arpc1b	actin related protein 2/3 complex, subunit 1B	1	DRlyp/lypnDR+/+_347	1	1	0	0	0	0	0	0	0	0	7.86	7.87	7.87	8.42	7.87	7.85	7.85	8.87	7.97	7.96	7.87	7.86	7.99	7.90	7.95	7.99	8.04	7.96	7.82	7.90
1387005_at	Ctss	cathepsin S	1	DRlyp/lypnDR+/+_347	1	1	0	0	0	0	0	0	0	0	6.34	6.94	7.06	8.49	6.46	6.69	7.06	8.52	7.01	7.20	7.38	7.37	7.15	7.20	7.67	8.03	6.78	7.31	7.51	7.08
1387173_at	Cma1	chymase 1, mast cell	1	DRlyp/lypnDR+/+_347	1	1	0	0	0	0	0	0	0	0	2.74	2.75	2.86	3.29	2.75	2.97	2.89	3.32	2.99	2.80	3.06	2.95	2.93	2.85	3.02	2.82	2.80	2.95	2.96	2.98
1387189_at	Slc22a3	solute carrier family 22 (extraneuronal monoamine transporter), member 3	1	DRlyp/lypnDR+/+_347	1	1	0	0	0	0	0	0	0	0	2.26	2.46	2.50	3.33	2.49	2.44	2.61	3.22	2.21	2.42	2.44	2.37	2.31	2.34	2.47	2.82	2.28	2.39	2.47	2.47
1387198_at	Inpp5d	inositol polyphosphate-5-phosphatase D	1	DRlyp/lypnDR+/+_347	1	1	0	0	0	0	0	0	0	0	4.07	4.12	4.22	5.15	3.99	4.37	4.48	5.73	4.05	4.17	4.12	4.22	4.24	4.15	4.15	4.44	4.10	4.34	4.12	4.31
1387227_at	Wipf1	WAS/WASL interacting protein family, member 1	1	DRlyp/lypnDR+/+_347	1	1	0	0	0	0	0	0	0	0	6.51	6.36	6.23	7.50	6.52	6.35	6.59	8.41	6.21	6.02	5.96	6.06	6.68	6.12	6.25	6.49	6.26	6.09	6.22	6.12
1387277_at	Lyn	v-yes-1 Yamaguchi sarcoma viral related oncogene homolog	1	DRlyp/lypnDR+/+_347	1	1	0	0	0	0	0	0	0	0	5.21	5.50	5.34	5.64	5.52	5.40	5.60	5.78	5.55	5.68	5.94	5.72	5.65	5.91	5.60	5.80	5.75	5.74	5.89	5.97
1387394_at	Il2rb	interleukin 2 receptor, beta	1	DRlyp/lypnDR+/+_347	1	1	0	0	0	0	0	0	0	0	3.40	3.23	3.51	4.33	3.29	3.31	3.53	4.17	3.32	3.52	3.51	3.61	3.52	3.37	3.60	3.89	3.13	3.60	3.48	3.52
1387459_at	Pkib	protein kinase (cAMP-dependent, catalytic) inhibitor beta	1	DRlyp/lypnDR+/+_347	1	1	0	0	0	0	0	0	0	0	5.14	5.55	5.76	5.95	5.19	5.34	5.75	6.37	3.67	3.89	4.10	4.06	4.10	4.08	3.91	4.27	3.81	3.69	4.26	3.89
1387472_at	Cd3d	CD3 molecule, delta	1	DRlyp/lypnDR+/+_347	1	1	0	0	0	0	0	0	0	0	2.83	2.74	2.95	3.97	2.92	3.03	3.79	5.26	2.91	2.82	2.85	2.86	2.72	2.89	2.90	3.53	2.82	2.95	2.86	3.16
1387502_at	Stk17b	serine/threonine kinase 17b	1	DRlyp/lypnDR+/+_347	1	1	0	0	0	0	0	0	0	0	4.84	4.61	4.56	6.59	4.67	4.53	5.32	7.92	4.70	4.37	4.90	4.74	4.67	4.66	5.07	5.58	4.55	4.79	4.79	4.87
1387627_at	Cd86	CD86 molecule	1	DRlyp/lypnDR+/+_347	1	1	0	0	0	0	0	0	0	0	2.92	3.10	2.89	3.31	2.89	3.22	3.19	3.49	3.13	3.00	3.11	3.24	3.04	3.24	3.13	3.29	3.21	3.19	3.21	3.23
1387659_at	Gda	guanine deaminase	1	DRlyp/lypnDR+/+_347	1	1	0	0	0	0	0	0	0	0	5.00	5.40	5.75	6.41	4.92	5.35	5.75	6.45	5.08	5.21	5.26	5.32	5.30	5.33	5.56	5.57	5.15	5.28	5.29	5.39
1387749_at	Cd79b	Cd79b molecule, immunoglobulin-associated beta	1	DRlyp/lypnDR+/+_347	1	1	0	0	0	0	0	0	0	0	3.45	3.64	3.66	6.55	3.38	3.56	4.41	7.83	3.67	3.56	3.94	4.01	3.37	3.42	3.87	4.87	3.55	3.49	3.55	3.81
1387794_at	Fcn1	ficolin (collagen/fibrinogen domain containing) 1	1	DRlyp/lypnDR+/+_347	1	1	0	0	0	0	0	0	0	0	3.02	2.70	2.84	5.84	2.94	3.06	3.56	6.06	2.94	2.94	3.53	3.07	3.01	3.02	3.84	4.63	3.07	3.13	3.06	3.10
1387799_at	Fxyd2	FXYD domain-containing ion transport regulator 2	1	DRlyp/lypnDR+/+_347	1	1	0	0	0	0	0	0	0	0	3.45	3.53	3.53	4.24	3.51	3.59	3.57	4.38	3.56	3.60	3.86	3.71	3.61	3.59	3.69	4.39	3.53	3.67	3.76	3.56
1387893_at	C1s	complement component 1, s subcomponent	1	DRlyp/lypnDR+/+_347	1	1	0	0	0	0	0	0	0	0	6.39	6.66	6.73	8.06	6.30	6.66	6.81	7.78	6.15	6.55	6.10	6.42	6.15	6.34	6.83	7.50	6.08	6.28	6.43	6.34
1387952_a_at	Cd44	Cd44 molecule	1	DRlyp/lypnDR+/+_347	1	1	0	0	0	0	0	0	0	0	4.02	4.31	4.22	4.99	4.09	4.23	4.58	5.43	4.13	4.14	4.07	4.28	3.91	4.13	4.45	4.27	3.99	4.28	4.19	4.03
1387957_a_at	Sh3kbp1	SH3-domain kinase binding protein 1	1	DRlyp/lypnDR+/+_347	1	1	0	0	0	0	0	0	0	0	6.78	6.88	6.99	7.30	6.81	6.88	7.05	7.72	6.43	6.52	6.71	6.96	6.49	6.74	6.85	7.06	6.61	6.67	6.75	7.09
1387965_at	Havcr1	hepatitis A virus cellular receptor 1	1	DRlyp/lypnDR+/+_347	1	1	0	0	0	0	0	0	0	0	3.14	3.10	3.21	3.95	3.25	3.01	3.29	4.97	2.99	3.12	3.20	3.35	3.01	3.17	3.31	3.73	3.00	3.24	3.17	3.47
1388026_at	Cd247	Cd247 molecule	1	DRlyp/lypnDR+/+_347	1	1	0	0	0	0	0	0	0	0	4.24	4.10	4.24	4.82	4.05	4.18	4.82	4.89	3.98	4.17	4.12	4.56	3.94	4.14	4.35	4.71	4.00	4.11	4.47	4.42
1388071_x_at	RT1-EC2	RT1 class Ib, locus EC2	1	DRlyp/lypnDR+/+_347	1	1	0	0	0	0	0	0	0	0	7.64	7.74	8.01	8.62	7.51	7.75	8.04	8.19	7.67	8.03	8.03	8.36	10.88	10.96	11.03	11.10	7.81	7.82	7.98	8.12
1388131_at	Tubb2b	tubulin, beta 2B class IIb	1	DRlyp/lypnDR+/+_347	1	1	0	0	0	0	0	0	0	0	5.74	5.57	5.75	6.13	5.64	5.80	6.03	6.17	5.78	6.55	5.61	5.56	5.63	6.07	5.52	5.64	5.89	5.62	5.55	5.90
1388164_at	RT1-S3	RT1 class Ib, locus S3	1	DRlyp/lypnDR+/+_347	1	1	0	0	0	0	0	0	0	0	6.94	6.89	7.02	8.23	6.93	6.90	6.94	7.35	7.23	7.23	6.87	6.96	7.60	7.16	7.25	7.35	7.21	6.98	6.93	6.92
1388181_at	Igd	immunoglobulin delta heavy chain constant region	1	DRlyp/lypnDR+/+_347	1	1	0	0	0	0	0	0	0	0	3.09	3.14	3.29	4.44	3.29	3.24	3.18	5.39	3.09	3.07	3.05	3.19	2.93	3.09	3.05	3.30	3.06	3.10	3.06	3.21
1388202_at	RT1-EC2	RT1 class Ib, locus EC2	1	DRlyp/lypnDR+/+_347	1	1	0	0	0	0	0	0	0	0	3.40	3.46	3.58	4.31	3.42	3.47	3.85	3.97	3.53	3.65	3.64	3.67	4.06	4.59	4.04	4.21	3.58	3.45	3.66	3.68
1388212_a_at	RT1-S3	RT1 class Ib, locus S3	1	DRlyp/lypnDR+/+_347	1	1	0	0	0	0	0	0	0	0	7.03	7.00	7.15	8.49	6.96	7.02	7.00	7.61	7.36	7.36	7.09	7.12	7.39	7.45	7.41	7.52	7.33	7.20	7.18	7.25
1388213_a_at	RT1-S3	RT1 class Ib, locus S3	1	DRlyp/lypnDR+/+_347	1	1	0	0	0	0	0	0	0	0	6.12	6.00	6.18	7.66	5.92	5.96	6.02	6.73	6.43	6.38	6.15	6.19	6.49	6.54	6.53	6.57	6.34	6.18	6.31	6.28
1388275_at	LOC1009127	T-cell receptor beta chain T17T-22-like /// T-cell receptor beta chain	1	DRlyp/lypnDR+/+_347	1	1	0	0	0	0	0	0	0	0	3.07	3.18	3.23	5.65	3.33	3.29	4.91	7.28	3.00	3.25	3.31	3.53	3.35	3.24	3.51	4.68	3.23	3.49	3.41	3.73
1388277_at	LOC1003617	lambda-chain C1-region-like /// Ig lambda chain V-VI region SUT-like	1	DRlyp/lypnDR+/+_347	1	1	0	0	0	0	0	0	0	0	4.48	4.46	4.44	5.81	4.58	4.80	5.15	7.17	4.75	4.59	4.93	4.95	4.70	4.47	4.86	5.71	4.59	4.72	4.56	5.13
1388433_at	Krt19	keratin 19	1	DRlyp/lypnDR+/+_347	1	1	0	0	0	0	0	0	0	0	8.20	8.48	8.76	8.69	8.35	8.41	9.13	8.71	5.56	5.36	5.26	5.57	5.67	5.10	5.80	6.01	5.76	5.25	5.45	5.18
1388460_at	Capg	capping protein (actin filament), gelsolin-like	1	DRlyp/lypnDR+/+_347	1	1	0	0	0	0	0	0	0	0	4.45	4.56	4.58	5.80	4.27	4.29	4.92	6.30	4.30	4.43	4.51	4.67	4.46	4.52	4.74	4.90	4.50	4.38	4.59	4.63
1388673_at	Lsp1	lymphocyte-specific protein 1	1	DRlyp/lypnDR+/+_347	1	1	0	0	0	0	0	0	0	0	3.75	4.00	4.08	5.72	3.79	3.94	4.35	5.89	3.66	3.73	3.93	3.84	3.80	3.82	3.87	4.35	3.76	3.92	3.97	4.10
1388694_at	LOC1009097	RT1 class I histocompatibility antigen, AA alpha chain-like /// RT1 class I, locus T24, gene 3	1	DRlyp/lypnDR+/+_347	1	1	0	0	0	0	0	0	0	0	4.08	4.10	4.29	4.63	4.08	4.12	4.11	4.62	4.17	4.20	4.24	4.46	3.96	4.33	4.45	4.61	4.13	4.30	4.27	4.52
1388722_at	Dnajb1	DnaJ (Hsp40) homolog, subfamily B, member 1	1	DRlyp/lypnDR+/+_347	1	1	0	0	0	0	0	0	0	0	8.85	9.06	9.11	9.62	8.91	8.79	9.81	9.70	8.87	8.82	8.80	9.06	8.55	9.15	9.48	9.24	8.92	8.90	8.69	9.18
1388740_at	Fermt3	fermitin family member 3	1	DRlyp/lypnDR+/+_347	1	1	0	0	0	0	0	0	0	0	4.90	4.84	5.10	6.02	5.03	5.10	5.37	6.61	4.89	4.87	5.02	4.99	5.17	4.95	5.07	5.37	4.87	4.99	5.20	5.20
1388784_at	Csf1r	colony stimulating factor 1 receptor	1	DRlyp/lypnDR+/+_347	1	1	0	0	0	0	0	0	0	0	4.54	4.84	4.90	5.96	4.38	4.90	5.09	6.25	4.35	4.41	4.75	4.74	4.68	4.50	5.16	5.71	4.40	4.77	4.90	4.57
1388879_at	Abi3bp	ABI family, member 3 (NESH) binding protein	1	DRlyp/lypnDR+/+_347	1	1	0	0	0	0	0	0	0	0	4.83	5.04	5.13	5.75	4.80	4.89	5.29	6.13	3.95	4.11	4.05	4.19	4.16	4.24	4.70	5.04	4.24	4.39	4.38	4.45
1388940_at	Arhgap25	Rho GTPase activating protein 25	1	DRlyp/lypnDR+/+_347	1	1	0	0	0	0	0	0	0	0	3.47	3.49	3.35	4.25	3.44	3.46	3.65	4.58	3.39	3.32	3.61	3.54	3.52	3.60	3.52	3.65	3.55	3.48	3.43	3.75
1388945_at	Pxdc1	PX domain containing 1	1	DRlyp/lypnDR+/+_347	1	1	0	0	0	0	0	0	0	0	5.72	5.66	5.73	6.66	5.59	5.67	5.90	7.34	5.70	5.82	5.82	5.95	5.73	5.85	5.73	6.04	5.87	5.94	5.83	6.00
1389092_at	Il2rg	interleukin 2 receptor, gamma	1	DRlyp/lypnDR+/+_347	1	1	0	0	0	0	0	0	0	0	4.95	5.01	4.86	6.56	4.94	4.82	5.32	7.37	4.88	4.89	5.02	5.06	4.82	5.00	5.30	5.70	5.06	5.02	5.08	5.08
1389179_at	Cidea	cell death-inducing DFFA-like effector a	1	DRlyp/lypnDR+/+_347	1	1	0	0	0	0	0	0	0	0	3.61	3.90	4.09	4.14	3.62	3.60	4.41	4.31	3.44	3.50	3.54	3.76	3.79	3.51	5.15	4.03	3.84	3.59	4.60	3.71
1389210_at	Lcp1	lymphocyte cytosolic protein 1	1	DRlyp/lypnDR+/+_347	1	1	0	0	0	0	0	0	0	0	5.30	5.48	5.58	8.02	5.32	5.42	6.11	9.05	5.77	5.61	5.83	5.87	5.75	5.91	6.14	6.47	5.70	5.81	5.86	5.89
1389413_at	Evi2a	ecotropic viral integration site 2A	1	DRlyp/lypnDR+/+_347	1	1	0	0	0	0	0	0	0	0	3.20	3.15	2.99	4.46	3.25	2.97	3.28	5.82	3.24	2.94	3.12	3.15	3.37	3.30	3.23	3.66	3.24	3.03	3.12	3.28
1389581_at	Il33	interleukin 33	1	DRlyp/lypnDR+/+_347	1	1	0	0	0	0	0	0	0	0	3.68	3.62	3.82	4.91	3.55	3.63	4.12	6.02	3.55	3.53	3.55	3.84	3.52	3.60	3.91	4.09	3.43	3.59	3.73	3.76
1389605_at	Jakmip1	janus kinase and microtubule interacting protein 1	1	DRlyp/lypnDR+/+_347	1	1	0	0	0	0	0	0	0	0	3.08	3.03	3.16	3.65	3.01	2.92	3.23	4.60	2.92	3.02	3.20	3.25	2.92	2.90	3.22	3.35	2.99	2.83	3.06	3.18
1389732_at	Dram	damage-regulated autophagy modulator	1	DRlyp/lypnDR+/+_347	1	1	0	0	0	0	0	0	0	0	6.66	6.96	6.96	7.38	6.70	6.85	6.92	7.27	6.65	6.85	6.85	6.91	6.83	7.16	6.99	7.02	6.51	6.90	6.89	6.95
1389997_at	Cd3e	CD3 molecule, epsilon	1	DRlyp/lypnDR+/+_347	1	1	0	0	0	0	0	0	0	0	3.14	3.17	3.16	3.58	3.19	2.99	3.55	4.58	3.05	3.02	3.17	3.29	3.09	3.21	3.08	3.23	3.12	3.12	3.06	3.28
1390282_at	Cyp2s1	cytochrome P450, family 2, subfamily s, polypeptide 1	1	DRlyp/lypnDR+/+_347	1	1	0	0	0	0	0	0	0	0	3.93	3.95	3.84	4.41	3.88	4.23	4.19	4.62	4.15	4.17	4.07	4.10	4.04	4.16	4.30	4.09	4.14	4.26	4.25	4.23
1390513_at	LOC690130	similar to ras homolog gene family, member f	1	DRlyp/lypnDR+/+_347	1	1	0	0	0	0	0	0	0	0	3.89	3.95	3.99	4.69	4.03	4.31	4.30	4.95	4.01	3.89	3.95	4.00	4.07	3.88	3.99	4.09	3.86	3.99	3.88	4.14
1390529_at	Cd83	CD83 molecule	1	DRlyp/lypnDR+/+_347	1	1	0	0	0	0	0	0	0	0	7.06	7.22	7.13	8.53	7.17	7.25	7.44	9.42	7.11	7.13	7.14	7.14	7.06	7.06	7.21	7.45	7.02	6.99	7.08	7.15
1390707_at	Rgs10	regulator of G-protein signaling 10	1	DRlyp/lypnDR+/+_347	1	1	0	0	0	0	0	0	0	0	4.62	5.12	5.27	6.14	4.39	5.17	5.58	6.93	4.43	4.97	4.61	4.94	4.54	4.74	5.05	5.31	4.59	4.79	4.74	4.63
1391015_at	Ccdc69	coiled-coil domain containing 69	1	DRlyp/lypnDR+/+_347	1	1	0	0	0	0	0	0	0	0	3.90	4.05	4.16	4.71	4.06	4.41	4.42	5.08	4.01	4.11	4.27	4.39	4.04	4.27	4.28	4.38	4.20	4.27	4.32	4.32
1391072_at	Fcer2	Fc fragment of IgE, low affinity II, receptor for (CD23)	1	DRlyp/lypnDR+/+_347	1	1	0	0	0	0	0	0	0	0	2.61	2.80	2.60	4.72	2.85	2.85	3.09	5.19	2.91	2.63	2.67	2.84	2.70	2.81	2.87	3.05	2.60	2.72	2.93	2.81
1391162_at	Dcdc2	doublecortin domain containing 2	1	DRlyp/lypnDR+/+_347	1	1	0	0	0	0	0	0	0	0	4.23	4.27	4.73	4.82	4.35	4.41	5.34	5.10	4.08	3.86	3.78	4.56	4.36	4.19	4.74	4.77	4.39	3.98	4.33	4.40
1391214_at	Fam26f	family with sequence similarity 26, member F	1	DRlyp/lypnDR+/+_347	1	1	0	0	0	0	0	0	0	0	2.94	2.91	2.86	3.68	2.89	3.01	3.20	3.85	2.86	2.85	2.91	2.99	2.81	2.94	2.97	3.26	3.01	3.04	2.88	3.29
1391612_at	Il22ra2	interleukin 22 receptor, alpha 2	1	DRlyp/lypnDR+/+_347	1	1	0	0	0	0	0	0	0	0	2.37	2.62	2.49	4.97	2.37	2.49	3.12	6.71	2.62	2.40	2.49	2.48	2.38	2.42	3.71	5.74	2.45	2.45	2.51	2.59
1391737_at	Ncf4	neutrophil cytosolic factor 4	1	DRlyp/lypnDR+/+_347	1	1	0	0	0	0	0	0	0	0	4.13	4.11	4.06	4.90	4.06	4.25	4.27	5.49	4.04	4.01	4.19	4.10	4.10	4.00	4.25	4.35	3.99	4.22	4.22	4.12
1391878_at	RGD1560020	similar to Myb proto-oncogene protein (C-myb) (predicted)	1	DRlyp/lypnDR+/+_347	1	1	0	0	0	0	0	0	0	0	2.97	3.10	3.24	3.52	3.02	3.15	3.24	4.65	2.90	2.81	2.82	2.69	2.79	2.95	3.02	2.99	3.13	2.69	2.95	2.90
1392032_at	Grap	GRB2-related adaptor protein	1	DRlyp/lypnDR+/+_347	1	1	0	0	0	0	0	0	0	0	4.66	4.87	4.75	5.41	4.81	4.97	5.03	5.94	4.76	4.79	4.90	4.92	4.70	4.97	4.86	4.98	4.85	4.97	4.90	5.05
1392053_at	Mmrn1	multimerin 1	1	DRlyp/lypnDR+/+_347	1	1	0	0	0	0	0	0	0	0	2.61	3.11	2.98	3.78	2.63	2.57	3.21	5.11	2.36	2.78	2.99	2.72	2.76	2.92	3.25	2.98	2.65	2.78	2.75	3.06
1392171_at	Chi3l1	chitinase 3-like 1 (cartilage glycoprotein-39)	1	DRlyp/lypnDR+/+_347	1	1	0	0	0	0	0	0	0	0	4.30	4.19	4.56	5.58	4.12	4.29	4.50	6.19	4.02	4.28	4.36	4.49	4.22	4.16	4.38	4.80	4.07	4.20	4.39	4.48
1392233_at	Cr2	complement component (3d/Epstein Barr virus) receptor 2	1	DRlyp/lypnDR+/+_347	1	1	0	0	0	0	0	0	0	0	2.48	2.49	2.37	3.81	2.41	2.54	2.57	5.72	2.57	2.46	2.60	2.49	2.49	2.20	2.61	2.75	2.40	2.49	2.28	2.45
1392308_at	Pla2g2d	phospholipase A2, group IID	1	DRlyp/lypnDR+/+_347	1	1	0	0	0	0	0	0	0	0	2.48	2.60	2.60	3.34	2.36	2.56	2.81	3.71	2.74	2.61	2.70	2.68	2.58	2.55	2.65	2.72	2.56	2.50	2.63	2.62
1392322_at	Gimap7	GTPase, IMAP family member 7	1	DRlyp/lypnDR+/+_347	1	1	0	0	0	0	0	0	0	0	2.72	2.61	2.63	3.55	2.53	2.67	3.18	4.96	2.36	2.67	2.65	2.64	2.51	2.54	2.78	2.88	2.50	2.55	2.55	2.67
1392334_at	RT1-Ba	RT1 class II, locus Ba	1	DRlyp/lypnDR+/+_347	1	1	0	0	0	0	0	0	0	0	7.16	7.17	6.93	7.04	7.14	7.31	7.14	7.02	7.20	7.07	7.20	7.25	7.22	7.32	7.23	7.09	7.04	7.43	7.16	7.30
1392444_at	Chst5	carbohydrate (N-acetylglucosamine 6-O) sulfotransferase 5	1	DRlyp/lypnDR+/+_347	1	1	0	0	0	0	0	0	0	0	4.64	4.95	5.34	5.73	4.76	5.01	5.30	5.63	4.11	4.14	4.22	4.45	4.23	4.24	4.15	4.33	4.16	4.15	4.27	4.24
1392494_at	LOC499331	similar to hypothetical protein D030056L22	1	DRlyp/lypnDR+/+_347	1	1	0	0	0	0	0	0	0	0	6.86	7.04	7.06	7.37	6.79	7.06	7.11	7.75	6.81	6.88	7.06	7.02	6.87	6.96	7.17	7.11	6.82	6.91	6.95	7.00
1392547_at	MGC105649	hypothetical LOC302884	1	DRlyp/lypnDR+/+_347	1	1	0	0	0	0	0	0	0	0	3.78	3.90	4.05	5.23	3.71	4.00	4.16	4.36	3.67	4.23	3.88	4.00	4.18	4.33	4.28	4.69	3.75	4.06	4.14	4.06
1392754_at	Adam8	ADAM metallopeptidase domain 8	1	DRlyp/lypnDR+/+_347	1	1	0	0	0	0	0	0	0	0	4.51	4.51	4.46	5.00	4.34	4.46	4.97	5.03	4.23	4.57	4.58	4.64	4.36	4.27	4.65	4.90	4.57	4.40	4.67	4.74
1392886_a_at	Fcrla	Fc receptor-like A	1	DRlyp/lypnDR+/+_347	1	1	0	0	0	0	0	0	0	0	3.93	4.13	4.20	6.33	4.27	4.60	4.86	7.29	4.35	4.24	4.53	4.59	3.98	4.25	4.36	5.04	4.18	4.09	4.88	4.36
1393049_at	Myo1g	myosin IG	1	DRlyp/lypnDR+/+_347	1	1	0	0	0	0	0	0	0	0	4.45	4.50	4.67	5.58	4.50	4.53	5.03	5.87	4.51	4.34	4.57	4.73	4.59	4.36	4.61	4.84	4.48	4.67	4.63	4.80
1393144_at	Nmi	N-myc (and STAT) interactor	1	DRlyp/lypnDR+/+_347	1	1	0	0	0	0	0	0	0	0	6.90	6.99	7.17	7.42	6.89	6.82	7.12	7.25	6.63	6.89	6.91	7.05	6.78	7.02	7.01	7.17	6.79	6.85	7.01	6.94
1393280_at	Ly86	lymphocyte antigen 86	1	DRlyp/lypnDR+/+_347	1	1	0	0	0	0	0	0	0	0	3.15	3.48	3.67	6.12	3.37	3.53	3.87	6.69	3.36	3.39	3.78	3.70	3.25	3.57	4.14	4.80	3.24	3.58	3.48	3.61
1393297_at	Pou2af1	POU class 2 associating factor 1	1	DRlyp/lypnDR+/+_347	1	1	0	0	0	0	0	0	0	0	2.23	2.16	2.34	6.20	2.31	2.27	3.55	7.67	2.41	2.16	2.92	2.58	2.21	2.16	2.42	3.31	2.22	2.21	2.37	2.63
1393336_at	Swap70	SWAP switching B-cell complex 70	1	DRlyp/lypnDR+/+_347	1	1	0	0	0	0	0	0	0	0	6.37	6.48	6.39	7.30	6.46	6.34	6.54	8.34	6.41	6.30	6.34	6.30	6.44	6.41	6.35	6.46	6.35	6.45	6.24	6.28
1393347_at	Itgal	integrin, alpha L	1	DRlyp/lypnDR+/+_347	1	1	0	0	0	0	0	0	0	0	5.83	5.66	5.87	6.99	6.01	5.79	5.89	7.14	5.52	5.54	5.57	5.73	5.86	5.55	5.60	5.86	5.70	5.45	5.70	5.68
1393379_a_at	Blk	B lymphoid tyrosine kinase	1	DRlyp/lypnDR+/+_347	1	1	0	0	0	0	0	0	0	0	4.28	4.32	4.36	5.47	4.41	4.53	4.55	6.24	4.41	4.37	4.61	4.51	4.36	4.47	4.54	4.67	4.23	4.39	4.54	4.60
1393400_at	Zap70	zeta-chain (TCR) associated protein kinase 70	1	DRlyp/lypnDR+/+_347	1	1	0	0	0	0	0	0	0	0	5.04	5.20	5.33	5.95	5.26	5.50	5.74	6.68	5.05	5.24	5.31	5.38	5.18	5.27	5.40	5.82	5.03	5.38	5.24	5.56
1393460_at	Lrrc33	leucine rich repeat containing 33	1	DRlyp/lypnDR+/+_347	1	1	0	0	0	0	0	0	0	0	6.37	6.48	6.46	6.93	6.57	6.58	6.57	7.33	6.20	6.21	6.36	6.25	6.29	6.33	6.32	6.53	6.19	6.39	6.39	6.24
1393638_at	---	---	1	DRlyp/lypnDR+/+_347	1	1	0	0	0	0	0	0	0	0	3.30	3.44	3.54	4.69	3.40	3.36	4.15	5.62	3.30	2.97	3.39	3.40	3.62	3.07	3.33	4.03	3.04	3.21	3.33	3.26
1393663_at	Slc36a2	solute carrier family 36 (proton/amino acid symporter), member 2	1	DRlyp/lypnDR+/+_347	1	1	0	0	0	0	0	0	0	0	3.51	3.63	3.56	4.10	3.61	3.64	3.69	4.18	3.60	3.63	3.66	3.64	3.47	3.63	3.89	4.07	3.61	3.58	3.66	3.77
1393688_at	LOC685048 /	similar to paired immunoglobin-like type 2 receptor beta /// similar to cell surface receptor F	1	DRlyp/lypnDR+/+_347	1	1	0	0	0	0	0	0	0	0	2.70	2.87	2.76	3.27	2.66	2.88	2.85	3.47	2.94	2.72	2.86	2.92	2.69	2.92	3.07	3.18	2.76	2.88	2.89	2.89
1393708_at	Bhlhe22	basic helix-loop-helix family, member e22	1	DRlyp/lypnDR+/+_347	1	1	0	0	0	0	0	0	0	0	2.62	2.70	2.76	3.19	2.69	2.80	3.09	3.33	2.69	2.55	2.61	3.00	2.75	2.75	2.92	3.37	2.78	2.79	2.75	2.62
1393863_at	Cd180	CD180 molecule	1	DRlyp/lypnDR+/+_347	1	1	0	0	0	0	0	0	0	0	2.17	2.04	2.19	2.67	2.18	2.14	2.20	3.17	2.12	2.16	2.18	2.27	2.19	2.11	2.07	2.19	2.14	2.11	2.33	2.32
1393926_at	Stap1	signal transducing adaptor family member 1	1	DRlyp/lypnDR+/+_347	1	1	0	0	0	0	0	0	0	0	2.78	2.82	2.75	4.04	2.94	2.78	3.12	5.58	2.75	2.76	2.85	2.86	2.74	2.75	2.99	2.97	2.79	2.80	2.82	2.92
1393929_at	Ccr6	chemokine (C-C motif) receptor 6	1	DRlyp/lypnDR+/+_347	1	1	0	0	0	0	0	0	0	0	3.61	3.74	3.69	5.47	3.66	3.93	3.88	6.16	3.79	3.72	3.91	3.88	3.68	3.51	4.10	4.51	3.41	3.88	3.66	3.94
1394051_at	P2ry10	purinergic receptor P2Y, G-protein coupled, 10	1	DRlyp/lypnDR+/+_347	1	1	0	0	0	0	0	0	0	0	1.73	1.90	1.78	3.57	1.90	1.79	2.13	4.97	1.93	1.80	1.94	2.08	1.89	1.89	1.99	2.59	1.86	1.87	1.86	1.83
1394264_at	Chst5	carbohydrate (N-acetylglucosamine 6-O) sulfotransferase 5	1	DRlyp/lypnDR+/+_347	1	1	0	0	0	0	0	0	0	0	3.31	3.78	3.85	3.78	3.49	3.71	3.85	4.12	3.09	3.17	3.19	3.28	3.07	2.97	3.14	3.07	3.15	3.32	3.20	3.36
1394306_at	Zap70	zeta-chain (TCR) associated protein kinase 70	1	DRlyp/lypnDR+/+_347	1	1	0	0	0	0	0	0	0	0	5.21	5.03	5.19	5.35	5.15	5.16	5.32	5.33	4.98	5.12	5.11	5.27	5.05	5.18	5.18	5.32	5.11	5.21	5.28	5.26
1394451_at	Anxa1	annexin A1	1	DRlyp/lypnDR+/+_347	1	1	0	0	0	0	0	0	0	0	6.73	6.88	7.04	7.32	6.94	6.77	7.99	8.07	6.36	6.33	6.45	6.52	6.49	6.55	6.56	6.81	6.59	6.33	6.47	6.42
1394501_at	Siglec10	sialic acid binding Ig-like lectin 10	1	DRlyp/lypnDR+/+_347	1	1	0	0	0	0	0	0	0	0	3.00	3.06	3.03	3.62	3.11	3.14	3.13	3.99	3.16	3.12	3.07	3.08	3.17	3.08	3.06	3.24	3.08	3.14	3.02	3.11
1394523_at	Plbd1	phospholipase B domain containing 1	1	DRlyp/lypnDR+/+_347	1	1	0	0	0	0	0	0	0	0	5.21	5.11	5.20	5.35	4.99	5.06	5.25	5.24	5.08	5.06	5.38	5.62	5.05	5.21	5.17	5.32	5.07	5.21	5.20	5.35
1394699_at	---	---	1	DRlyp/lypnDR+/+_347	1	1	0	0	0	0	0	0	0	0	4.17	4.43	4.58	4.67	4.24	4.41	5.15	4.68	4.50	4.47	4.59	4.84	4.58	4.61	5.14	5.16	4.69	4.60	5.02	4.77
1394819_at	Sp110	SP110 nuclear body protein	1	DRlyp/lypnDR+/+_347	1	1	0	0	0	0	0	0	0	0	4.70	4.65	4.76	5.17	4.71	4.71	4.64	5.27	4.64	4.55	4.75	4.73	4.23	4.61	4.84	4.86	4.62	4.64	4.49	4.47
1395301_x_at	LOC1009108	paired immunoglobulin-like type 2 receptor alpha-like /// similar to cell surface receptor FDF.	1	DRlyp/lypnDR+/+_347	1	1	0	0	0	0	0	0	0	0	4.57	4.71	4.62	5.10	4.15	4.50	5.10	5.27	4.53	4.44	4.89	4.85	4.34	4.41	4.80	5.48	4.38	4.62	4.66	4.72
1396132_at	Fcrla	Fc receptor-like A	1	DRlyp/lypnDR+/+_347	1	1	0	0	0	0	0	0	0	0	2.32	2.49	2.51	4.15	2.44	2.49	2.89	5.11	2.70	2.54	2.77	2.65	2.50	2.48	2.64	2.90	2.52	2.49	2.45	2.50
1396268_at	Sp110	SP110 nuclear body protein	1	DRlyp/lypnDR+/+_347	1	1	0	0	0	0	0	0	0	0	5.67	5.78	5.93	6.36	5.71	5.73	5.90	6.47	5.63	5.85	5.94	5.91	5.41	5.78	5.92	5.86	5.67	5.81	5.67	5.85
1397507_at	Apbb1ip	amyloid beta (A4) precursor protein-binding, family B, member 1 interacting protein	1	DRlyp/lypnDR+/+_347	1	1	0	0	0	0	0	0	0	0	2.10	2.16	2.21	2.72	2.06	2.13	2.24	2.95	2.08	2.18	2.20	2.19	2.15	2.14	2.33	2.18	2.19	2.16	2.10	2.19
1397548_at	Hmha1	histocompatibility (minor) HA-1	1	DRlyp/lypnDR+/+_347	1	1	0	0	0	0	0	0	0	0	4.49	4.50	4.55	5.11	4.42	4.55	4.67	5.49	4.65	4.53	4.64	4.79	4.44	4.60	4.54	4.79	4.55	4.60	4.63	4.67
1397866_at	Serpinb6b	serine (or cysteine) peptidase inhibitor, clade B, member 6b	1	DRlyp/lypnDR+/+_347	1	1	0	0	0	0	0	0	0	0	3.55	3.66	3.56	5.86	3.59	3.42	4.12	6.80	3.73	3.72	3.87	4.02	3.91	3.80	4.15	4.84	3.75	3.87	3.86	3.99
1398246_s_at	Fcgr2a /// LO	Fc fragment of IgG, low affinity IIa, receptor /// low affinity immunoglobulin gamma Fc regio	1	DRlyp/lypnDR+/+_347	1	1	0	0	0	0	0	0	0	0	5.45	5.36	5.55	6.91	5.38	5.61	5.44	6.15	5.33	5.23	5.18	5.11	5.61	5.11	5.38	5.28	5.22	5.27	5.36	4.86
1398297_at	Mapk12	mitogen-activated protein kinase 12	1	DRlyp/lypnDR+/+_347	1	1	0	0	0	0	0	0	0	0	6.12	6.34	6.67	6.40	5.85	6.34	6.62	6.44	5.52	5.90	5.96	5.93	5.63	5.88	6.02	6.04	5.61	5.79	5.90	5.74
1398405_at	Sept6	septin 6	1	DRlyp/lypnDR+/+_347	1	1	0	0	0	0	0	0	0	0	3.37	3.20	3.35	3.57	3.50	3.42	3.48	3.53	3.41	3.55	3.28	3.58	3.35	3.44	3.39	3.24	3.38	3.38	3.31	3.43
1398407_at	Tnfaip8	tumor necrosis factor, alpha-induced protein 8	1	DRlyp/lypnDR+/+_347	1	1	0	0	0	0	0	0	0	0	2.95	2.94	3.24	4.52	2.84	3.09	3.54	5.61	3.00	3.00	2.83	2.90	2.81	2.85	3.00	3.26	2.90	2.87	3.01	2.98
1368544_a_at	Nol3	nucleolar protein 3 (apoptosis repressor with CARD domain)	0	DRlyp/lypnF+/+_9	1	0	0	1	0	0	0	0	0	0	3.54	3.91	4.14	4.35	3.86	4.02	4.30	4.16	3.61	4.26	3.96	4.55	3.54	3.82	4.27	4.59	3.77	4.04	4.35	4.41
1369044_a_at	Pde4b	phosphodiesterase 4B, cAMP specific	0	DRlyp/lypnF+/+_9	1	0	0	1	0	0	0	0	0	0	4.23	4.28	4.40	4.73	4.42	4.31	4.26	5.26	4.22	4.12	4.51	4.51	4.00	4.24	4.62	4.51	4.09	4.36	4.29	4.59
1372691_at	Upp1	uridine phosphorylase 1	0	DRlyp/lypnF+/+_9	1	0	0	1	0	0	0	0	0	0	5.45	5.52	5.67	6.39	5.42	5.61	5.73	5.89	5.38	5.65	5.73	5.82	5.38	5.63	5.93	5.87	5.28	5.47	5.73	5.86
1373240_at	Dhrs3	dehydrogenase/reductase (SDR family) member 3	0	DRlyp/lypnF+/+_9	1	0	0	1	0	0	0	0	0	0	5.72	5.94	6.06	6.23	5.86	6.08	6.25	6.30	5.57	5.78	5.84	5.99	5.81	5.80	5.97	6.23	5.61	5.94	6.02	6.19

Affymetrix Probeset ID	Gene Symbol	Gene Name	unique BB on P7 n=822	Figure 1B P7 Venn n=1526	DRlyp/lyp STEM P7 n=712	DR+/+ STEM P7 n=936	Flyp/lyp STEM P7 n=520	F+/+ STEM P7 n=465	Figure 1B P0 Venn n=1071	DRlyp/lyp STEM P0 n=442	DR+/+ STEM P0 n=437	Flyp/lyp STEM P0 n=643	F+/+ STEM P0 n=632	Fig1D n=25	Mean log2 intensity DRlyp/lyp 20	Mean log2 intensity DRlyp/lyp 30	Mean log2 intensity DRlyp/lyp 40	Mean log2 intensity DRlyp/lyp 50	Mean log2 intensity DR+/+ 20	Mean log2 intensity DR+/+ 30	Mean log2 intensity DR+/+ 40	Mean log2 intensity DR+/+ 50	Mean log2 intensity Flyp/lyp 20	Mean log2 intensity Flyp/lyp 30	Mean log2 intensity Flyp/lyp 40	Mean log2 intensity Flyp/lyp 50	Mean log2 intensity Flyp20	Mean log2 intensity Flyp30	Mean log2 intensity Flyp40	Mean log2 intensity Flyp50	Mean log2 intensity F+/+ 20	Mean log2 intensity F+/+ 30	Mean log2 intensity F+/+ 40	Mean log2 intensity F+/+ 50
1376908_at	Ifit3	interferon-induced protein with tetratricopeptide repeats 3	0	DRlyp/lypnF+/+_9	1	0	0	1	0	0	0	0	0	0	2.70	2.66	2.85	3.60	2.62	2.61	2.79	3.08	2.69	2.86	2.96	3.07	2.73	3.01	3.09	3.50	2.51	2.88	3.08	3.25
1390385_at	Glce	glucuronic acid epimerase	0	DRlyp/lypnF+/+_9	1	0	0	1	0	0	0	0	0	0	7.26	7.69	7.77	7.65	7.43	7.74	7.63	7.50	7.38	7.60	7.68	7.72	7.44	7.59	7.81	7.73	7.31	7.70	7.72	7.85
1390956_at	---	---	0	DRlyp/lypnF+/+_9	1	0	0	1	0	0	0	0	0	0	5.11	5.15	5.23	5.88	5.11	5.19	5.50	5.55	4.98	5.02	5.25	5.48	4.85	5.07	5.01	5.34	4.90	5.20	5.23	5.41
1392996_at	Cpeb1	cytoplasmic polyadenylation element binding protein 1	0	DRlyp/lypnF+/+_9	1	0	0	1	0	0	0	0	0	0	7.15	7.41	7.68	7.61	7.14	7.43	7.55	7.45	7.38	7.66	7.73	7.80	7.44	7.80	7.85	7.89	7.30	7.68	7.79	7.88
1393217_at	Abcg3l1	ATP-binding cassette, subfamily G (WHITE), member 3-like 1	0	DRlyp/lypnF+/+_9	1	0	0	1	0	0	0	0	0	0	6.13	6.33	6.45	6.67	5.92	6.30	6.38	6.36	6.31	6.49	6.40	6.55	6.32	6.42	6.49	6.68	6.12	6.36	6.49	6.62
1367653_a_at	Mdh1	malate dehydrogenase 1, NAD (soluble)	0	DRlyp/lypnFIS_39	1	0	1	1	0	0	0	0	0	0	11.24	11.29	11.61	11.82	11.25	11.30	11.56	11.73	11.19	11.49	11.72	12.08	11.37	11.49	11.77	11.95	11.35	11.52	11.83	12.00
1367755_at	Cdo1	cysteine dioxygenase, type I	0	DRlyp/lypnFIS_39	1	0	1	1	0	0	0	0	0	0	8.15	8.51	8.80	8.69	8.31	8.52	8.65	8.79	7.83	8.24	8.60	8.80	7.67	8.29	8.74	8.74	7.74	8.43	8.53	8.80
1368145_at	Pcp4	Purkinje cell protein 4	0	DRlyp/lypnFIS_39	1	0	1	1	0	0	0	0	0	0	9.07	9.31	9.62	9.69	9.34	9.50	9.51	9.74	7.92	8.19	8.58	8.76	7.79	8.38	8.58	8.64	7.97	8.33	8.56	8.79
1368182_at	Acsl6	acyl-CoA synthetase long-chain family member 6	0	DRlyp/lypnFIS_39	1	0	1	1	0	0	0	0	0	0	6.63	7.00	7.33	7.20	6.87	6.95	7.16	7.23	6.75	7.09	7.34	7.43	6.80	6.92	7.41	7.45	6.89	7.12	7.44	7.44
1368200_at	Cx3cl1	chemokine (C-X3-C motif) ligand 1	0	DRlyp/lypnFIS_39	1	0	1	1	0	0	0	0	0	0	6.32	6.43	6.73	7.14	6.48	6.58	6.75	6.65	5.92	6.29	6.35	6.65	6.27	6.27	6.61	6.64	6.09	6.46	6.76	6.51
1368354_at	Gstt1	glutathione S-transferase theta 1	0	DRlyp/lypnFIS_39	1	0	1	1	0	0	0	0	0	0	6.97	7.43	7.50	7.48	7.06	7.46	7.55	7.43	6.73	7.24	7.38	7.45	6.63	7.36	7.53	7.45	6.95	7.37	7.38	7.50
1369654_at	Prkaa2	protein kinase, AMP-activated, alpha 2 catalytic subunit	0	DRlyp/lypnFIS_39	1	0	1	1	0	0	0	0	0	0	6.26	6.72	6.95	6.97	6.33	6.78	6.80	6.82	6.45	6.86	7.06	7.21	6.15	6.77	7.12	7.11	6.51	6.86	6.89	7.03
1369699_at	Glp1r	glucagon-like peptide 1 receptor	0	DRlyp/lypnFIS_39	1	0	1	1	0	0	0	0	0	0	8.41	8.84	8.95	9.05	8.32	8.89	8.97	8.77	8.18	8.64	8.75	8.94	6.81	7.58	7.81	7.92	8.10	8.81	8.95	9.03
1370251_at	Avpi1	arginine vasopressin-induced 1	0	DRlyp/lypnFIS_39	1	0	1	1	0	0	0	0	0	0	4.58	4.76	5.10	4.99	4.60	4.82	4.95	4.96	4.48	4.71	4.83	5.11	4.65	4.64	4.88	4.89	4.43	4.80	4.93	5.06
1370747_at	Fgf9	fibroblast growth factor 9	0	DRlyp/lypnFIS_39	1	0	1	1	0	0	0	0	0	0	5.04	5.30	5.65	5.61	5.09	5.29	5.52	5.44	4.90	5.34	5.67	5.98	5.12	5.49	5.89	6.04	4.90	5.32	5.73	5.94
1372277_at	Aldh4a1	aldehyde dehydrogenase 4 family, member A1	0	DRlyp/lypnFIS_39	1	0	1	1	0	0	0	0	0	0	6.25	6.39	6.50	7.15	6.32	6.37	6.59	6.72	5.98	6.28	6.35	6.76	5.99	6.31	6.45	6.55	5.92	6.33	6.63	6.63
1372311_at	Gnpda1	glucosamine-6-phosphate deaminase 1	0	DRlyp/lypnFIS_39	1	0	1	1	0	0	0	0	0	0	7.05	7.21	7.37	7.58	7.01	7.36	7.38	7.41	7.43	7.72	7.75	7.97	7.18	7.75	7.98	7.99	7.44	7.80	7.91	8.09
1372901_at	---	---	0	DRlyp/lypnFIS_39	1	0	1	1	0	0	0	0	0	0	7.48	7.90	8.19	8.26	7.69	7.93	8.02	8.03	7.62	7.92	7.99	8.23	7.73	7.83	8.15	8.30	7.61	7.93	8.13	8.28
1373368_at	PCOLCE2	procollagen C-endopeptidase enhancer 2	0	DRlyp/lypnFIS_39	1	0	1	1	0	0	0	0	0	0	5.18	5.29	5.58	5.69	5.43	5.57	5.56	5.65	4.84	5.31	5.30	5.56	5.04	5.11	5.35	5.20	5.05	5.28	5.26	5.59
1373902_at	Rimklb	ribosomal modification protein rimK-like family member B	0	DRlyp/lypnFIS_39	1	0	1	1	0	0	0	0	0	0	6.11	6.45	6.63	6.49	6.06	6.31	6.50	6.53	5.73	6.17	6.48	6.51	5.87	6.07	6.22	6.43	5.74	6.23	6.50	6.64
1374006_at	Kat3	kynurenine aminotransferase III	0	DRlyp/lypnFIS_39	1	0	1	1	0	0	0	0	0	0	5.42	5.94	5.91	6.07	5.84	5.81	5.89	5.98	5.65	6.02	6.27	6.13	5.95	6.02	5.92	5.95	5.74	6.12	6.31	6.17
1376345_at	Caly	calcyon neuron-specific vesicular protein	0	DRlyp/lypnFIS_39	1	0	1	1	0	0	0	0	0	0	6.67	6.89	7.23	7.18	6.78	6.94	7.22	7.21	6.53	7.05	7.31	7.58	6.43	7.05	7.32	7.41	6.67	7.05	7.32	7.56
1376501_at	Arhgap8	Rho GTPase activating protein 8	0	DRlyp/lypnFIS_39	1	0	1	1	0	0	0	0	0	0	6.11	6.28	6.49	6.61	6.12	6.35	6.43	6.29	6.15	6.56	6.48	6.68	6.35	6.39	6.54	6.53	6.13	6.53	6.63	6.61
1376733_at	Igsf11	immunoglobulin superfamily, member 11	0	DRlyp/lypnFIS_39	1	0	1	1	0	0	0	0	0	0	9.05	9.24	9.41	9.68	9.09	9.09	9.43	9.51	9.08	9.36	9.70	9.83	9.05	9.41	9.61	9.80	9.17	9.45	9.71	9.89
1377980_at	RGD1305725	similar to chromosome 20 open reading frame 102	0	DRlyp/lypnFIS_39	1	0	1	1	0	0	0	0	0	0	7.42	7.68	8.00	8.00	7.48	7.75	7.92	7.92	7.62	7.87	8.21	8.36	7.71	7.90	8.15	8.31	7.69	8.04	8.25	8.34
1379400_at	Aldh1l2	aldehyde dehydrogenase 1 family, member L2	0	DRlyp/lypnFIS_39	1	0	1	1	0	0	0	0	0	0	6.27	6.87	6.85	6.88	6.60	6.77	6.90	6.99	6.29	6.67	6.83	7.15	6.19	7.16	6.99	7.02	6.44	7.15	7.30	7.55
1382604_at	Polr3g	polymerase (RNA) III (DNA directed) polypeptide G	0	DRlyp/lypnFIS_39	1	0	1	1	0	0	0	0	0	0	7.12	7.38	7.63	7.67	7.28	7.36	7.50	7.66	6.83	7.13	7.32	7.85	6.71	7.10	7.39	7.66	7.01	7.18	7.50	7.79
1383191_at	Ppp1r1a	protein phosphatase 1, regulatory (inhibitor) subunit 1A	0	DRlyp/lypnFIS_39	1	0	1	1	0	0	0	0	0	0	9.33	9.72	9.89	9.92	9.42	9.82	9.79	9.85	9.14	9.55	9.85	9.90	8.86	9.42	9.78	9.84	9.08	9.64	9.82	9.88
1383432_at	Dapl1	death associated protein-like 1	0	DRlyp/lypnFIS_39	1	0	1	1	0	0	0	0	0	0	5.30	5.19	5.68	5.75	5.50	5.27	5.55	5.77	4.61	4.87	5.22	5.90	4.99	5.26	5.62	5.86	4.88	5.06	5.52	5.81
1383783_at	Pcdh9	protocadherin 9	0	DRlyp/lypnFIS_39	1	0	1	1	0	0	0	0	0	0	7.42	7.86	8.07	8.00	7.54	7.77	7.85	8.01	7.42	7.79	8.15	8.73	7.63	7.97	8.30	8.71	7.56	7.92	8.27	8.56
1384655_at	Kirrel3	kin of IRRE like 3 (Drosophila)	0	DRlyp/lypnFIS_39	1	0	1	1	0	0	0	0	0	0	5.23	5.64	5.64	5.80	5.30	5.50	5.53	5.65	4.94	5.36	5.54	5.81	5.33	5.43	5.61	5.80	4.90	5.34	5.58	5.58
1386968_at	Ppp1r1a	protein phosphatase 1, regulatory (inhibitor) subunit 1A	0	DRlyp/lypnFIS_39	1	0	1	1	0	0	0	0	0	0	11.47	11.75	11.96	11.95	11.58	11.81	11.83	11.88	11.41	11.68	11.83	11.95	11.36	11.47	11.88	11.90	11.34	11.65	11.83	11.84
1387177_at	Vipr2	vasoactive intestinal peptide receptor 2	0	DRlyp/lypnFIS_39	1	0	1	1	0	0	0	0	0	0	6.79	7.13	7.19	7.29	6.90	7.07	7.21	7.24	6.62	7.06	7.26	7.36	6.69	7.03	7.07	7.36	6.56	7.13	7.39	7.36
1387946_at	Lgals3bp	lectin, galactoside-binding, soluble, 3 binding protein	0	DRlyp/lypnFIS_39	1	0	1	1	0	0	0	0	0	0	8.66	8.65	9.00	9.48	8.67	8.59	8.80	8.86	8.08	8.19	8.33	8.69	8.27	8.25	8.65	8.79	8.06	8.12	8.39	8.68
1389632_at	Rhobtb1	Rho-related BTB domain containing 1	0	DRlyp/lypnFIS_39	1	0	1	1	0	0	0	0	0	0	7.98	8.17	8.27	8.60	7.99	8.09	8.37	8.40	8.06	8.46	8.65	8.79	8.50	8.36	8.68	8.86	8.31	8.45	8.60	8.84
1390172_at	Dhtkd1	dehydrogenase E1 and transketolase domain containing 1	0	DRlyp/lypnFIS_39	1	0	1	1	0	0	0	0	0	0	5.92	6.22	6.46	6.42	5.90	6.14	6.37	6.31	5.65	6.04	6.11	6.31	5.77	6.14	6.22	6.30	5.61	5.97	6.28	6.47
1390221_at	Prdm1	PR domain containing 1, with ZNF domain	0	DRlyp/lypnFIS_39	1	0	1	1	0	0	0	0	0	0	5.87	6.14	6.20	6.49	6.17	6.53	6.43	6.37	5.62	5.75	6.14	6.20	6.11	5.81	6.37	6.37	5.98	5.78	6.05	6.43
1390973_at	Trim45	tripartite motif-containing 45	0	DRlyp/lypnFIS_39	1	0	1	1	0	0	0	0	0	0	6.63	6.93	7.12	7.20	6.75	6.76	6.85	6.91	6.82	7.03	7.24	7.35	6.84	6.99	7.12	7.24	6.89	6.98	7.19	7.39
1392865_at	Fgf9	fibroblast growth factor 9	0	DRlyp/lypnFIS_39	1	0	1	1	0	0	0	0	0	0	6.34	6.60	6.91	7.01	6.47	6.50	6.89	6.88	6.18	6.75	6.93	7.33	6.48	6.82	7.07	7.41	6.24	6.57	7.00	7.28
1392994_at	Nmrk1	nicotinamide riboside kinase 1	0	DRlyp/lypnFIS_39	1	0	1	1	0	0	0	0	0	0	5.27	5.62	5.78	5.63	5.38	5.58	5.66	5.61	5.60	6.02	6.06	6.23	5.72	6.05	6.18	6.27	5.41	6.00	6.15	6.23
1395601_at	Nudt12	nudix (nucleoside diphosphate linked moiety X)-type motif 12	0	DRlyp/lypnFIS_39	1	0	1	1	0	0	0	0	0	0	4.37	4.77	4.93	4.85	4.59	4.74	4.67	4.67	4.64	4.99	4.93	5.20	4.80	4.94	5.04	5.12	4.69	4.81	5.12	5.20
1397688_at	Pcdh9	protocadherin 9	0	DRlyp/lypnFIS_39	1	0	1	1	0	0	0	0	0	0	5.03	5.31	5.42	5.36	5.25	5.27	5.31	5.42	5.02	5.29	5.71	6.10	5.34	5.33	5.72	5.97	5.30	5.39	5.95	6.07
1398425_at	Fam110b	family with sequence similarity 110, member B	0	DRlyp/lypnFIS_39	1	0	1	1	0	0	0	0	0	0	7.27	7.51	7.77	7.71	7.32	7.35	7.60	7.64	7.18	7.52	7.85	8.13	7.28	7.52	7.72	7.93	7.25	7.68	8.01	7.98
1399111_at	Cyb561d2	cytochrome b561 family, member D2	0	DRlyp/lypnFIS_39	1	0	1	1	0	0	0	0	0	0	6.65	6.94	7.17	7.13	6.73	7.02	7.08	6.98	6.61	6.90	7.16	7.27	6.55	6.95	7.22	7.24	6.62	7.01	7.08	7.28
1368307_at	Ggt7	gamma-glutamyltransferase 7	0	DRlyp/lypnFlyp/lyp_4	1	0	1	0	0	0	0	0	0	0	5.52	5.73	6.02	6.03	5.83	5.82	5.87	5.95	6.08	6.34	6.41	6.72	6.31	6.23	6.49	6.64	6.09	6.32	6.35	6.51
1386964_at	Bpifa2f	BPI fold containing family A, member 2F	0	DRlyp/lypnFlyp/lyp_4	1	0	1	0	0	0	0	0	0	0	6.72	6.90	7.02	7.24	6.87	7.01	7.11	7.31	7.10	7.29	7.39	7.63	7.43	7.17	7.42	7.39	7.10	6.89	7.27	6.94
1388203_x_at	RT1-CE10 ///	RT1 class I, locus CE10 /// RT1 class Ib, locus EC2	0	DRlyp/lypnFlyp/lyp_4	1	0	1	0	0	0	0	0	0	0	4.16	4.21	4.35	4.97	4.08	4.19	4.47	4.57	4.13	4.36	4.40	4.64	4.36	4.52	4.53	4.65	4.15	4.28	4.41	4.61
1388282_s_at	Klra5 /// LOC	killer cell lectin-like receptor, subfamily A, member 5 /// killer cell lectin-like receptor 5-like /	0	DRlyp/lypnFlyp/lyp_4	1	0	1	0	0	0	0	0	0	0	2.54	2.63	2.61	3.14	2.44	2.43	2.83	2.87	2.49	2.67	2.87	3.20	2.63	2.69	2.92	3.14	2.54	2.47	2.79	2.72
1367608_at	Cryba4	crystallin, beta A4	0	Flyp/lypnF+/+_114	0	0	1	1	0	0	0	0	0	0	5.19	5.16	5.26	5.29	5.32	5.24	5.25	5.39	4.94	5.18	5.33	5.55	4.93	5.08	5.33	5.53	5.02	5.37	5.42	5.62
1367740_at	Ckb	creatine kinase, brain	0	Flyp/lypnF+/+_114	0	0	1	1	0	0	0	0	0	0	6.69	6.90	7.10	7.18	6.72	6.84	7.09	7.15	6.67	7.01	7.24	7.49	6.80	7.10	7.31	7.40	6.81	7.00	7.20	7.40
1367764_at	Ccng1	cyclin G1	0	Flyp/lypnF+/+_114	0	0	1	1	0	0	0	0	0	0	8.93	8.93	9.24	9.34	8.88	8.99	9.23	9.35	9.21	9.29	9.48	9.82	9.19	9.38	9.65	9.83	9.21	9.46	9.59	10.03
1367946_at	Pdlim1	PDZ and LIM domain 1	0	Flyp/lypnF+/+_114	0	0	1	1	0	0	0	0	0	0	7.94	7.91	8.04	8.14	8.01	7.93	8.13	8.29	7.78	7.93	8.25	8.36	8.15	8.11	7.99	8.22	7.93	8.01	8.26	8.45
1368258_at	Apln	apelin	0	Flyp/lypnF+/+_114	0	0	1	1	0	0	0	0	0	0	5.37	5.43	5.45	5.45	5.26	5.67	5.30	5.51	5.29	5.55	5.62	5.94	5.27	5.56	5.73	5.87	5.26	5.54	5.70	5.93
1368398_at	Cacna1h	calcium channel, voltage-dependent, T type, alpha 1H subunit	0	Flyp/lypnF+/+_114	0	0	1	1	0	0	0	0	0	0	5.16	5.31	5.59	5.35	5.17	5.37	5.56	5.27	5.37	5.42	5.92	6.42	5.23	5.49	5.80	5.89	5.45	5.80	6.22	6.38
1368438_at	Pde10a	phosphodiesterase 10A	0	Flyp/lypnF+/+_114	0	0	1	1	0	0	0	0	0	0	7.30	7.42	7.59	7.68	7.35	7.26	7.67	7.52	6.82	6.94	7.38	7.52	6.45	7.11	7.38	7.44	7.04	7.27	7.52	7.75
1368440_at	Slc3a1	solute carrier family 3, member 1	0	Flyp/lypnF+/+_114	0	0	1	1	0	0	0	0	0	0	5.09	5.35	5.47	5.37	5.18	5.27	5.46	5.41	5.02	5.32	5.40	5.59	5.44	5.36	5.49	5.68	5.08	5.22	5.68	5.83
1368550_at	Foxq1	forkhead box Q1	0	Flyp/lypnF+/+_114	0	0	1	1	0	0	0	0	0	0	5.08	5.36	5.56	5.63	5.28	5.36	5.59	5.49	4.87	5.04	5.18	5.40	4.95	5.23	5.32	5.31	4.74	5.22	5.33	5.34
1368659_at	Agxt2	alanine-glyoxylate aminotransferase 2	0	Flyp/lypnF+/+_114	0	0	1	1	0	0	0	0	0	0	3.24	3.34	3.44	3.41	3.44	3.52	3.37	3.49	3.24	3.56	3.66	3.74	3.36	3.32	3.56	3.59	3.34	3.61	3.50	3.85
1368709_at	Fut1	fucosyltransferase 1	0	Flyp/lypnF+/+_114	0	0	1	1	0	0	0	0	0	0	4.79	6.14	6.01	5.81	4.71	6.24	5.97	5.72	5.30	6.33	6.22	6.32	5.19	6.42	6.58	6.42	5.78	6.81	6.89	7.03
1368853_at	Vsnl1	visinin-like 1	0	Flyp/lypnF+/+_114	0	0	1	1	0	0	0	0	0	0	8.57	8.94	9.03	8.81	8.45	8.85	8.73	8.80	8.20	8.69	8.77	8.93	7.79	8.53	8.80	8.76	8.18	8.66	8.69	8.62
1368854_at	Vsnl1	visinin-like 1	0	Flyp/lypnF+/+_114	0	0	1	1	0	0	0	0	0	0	10.24	10.55	10.62	10.51	10.14	10.49	10.46	10.42	9.87	10.35	10.40	10.57	9.56	10.26	10.38	10.45	9.86	10.32	10.43	10.35
1369011_at	Apoa5	apolipoprotein A-V	0	Flyp/lypnF+/+_114	0	0	1	1	0	0	0	0	0	0	3.26	3.13	3.15	3.19	3.09	3.30	3.65	3.29	3.10	3.32	3.40	3.64	3.23	3.27	3.41	3.34	3.16	3.42	3.66	3.51
1369275_s_at	Cyp2a1 /// Cy	cytochrome P450, family 2, subfamily a, polypeptide 1 /// cytochrome P450, family 2, subfan	0	Flyp/lypnF+/+_114	0	0	1	1	0	0	0	0	0	0	4.78	4.95	4.98	5.20	5.07	5.21	5.17	5.20	4.73	5.03	5.19	5.37	4.86	5.03	5.08	5.30	4.64	5.03	5.10	5.32
1369304_at	Pts	6-pyruvoyl-tetrahydropterin synthase	0	Flyp/lypnF+/+_114	0	0	1	1	0	0	0	0	0	0	6.88	7.28	7.35	7.34	6.83	7.11	7.30	7.26	6.89	7.35	7.48	7.52	6.87	7.34	7.43	7.49	6.92	7.33	7.52	7.57
1369350_at	Kcnq2	potassium voltage-gated channel, KQT-like subfamily, member 2	0	Flyp/lypnF+/+_114	0	0	1	1	0	0	0	0	0	0	4.11	4.19	4.20	4.37	3.92	4.14	4.31	4.19	4.11	4.11	4.19	4.18	4.06	4.16	4.08	4.19	4.04	4.12	4.30	4.24
1369437_at	Slco4a1	solute carrier organic anion transporter family, member 4a1	0	Flyp/lypnF+/+_114	0	0	1	1	0	0	0	0	0	0	5.03	5.24	5.25	5.37	5.04	5.04	5.08	5.22	4.67	5.14	5.33	5.47	4.96	5.18	5.04	5.29	4.84	5.07	5.27	5.16
1369662_at	Scn2a1	sodium channel, voltage-gated, type II, alpha 1	0	Flyp/lypnF+/+_114	0	0	1	1	0	0	0	0	0	0	4.46	4.60	4.87	4.78	4.54	4.52	4.44	4.59	4.08	4.69	4.61	4.97	5.07	4.63	4.83	4.97	3.95	4.41	4.67	4.46
1369737_at	Crem	cAMP responsive element modulator	0	Flyp/lypnF+/+_114	0	0	1	1	0	0	0	0	0	0	4.02	4.00	4.46	4.31	3.92	3.99	4.31	4.70	3.65	4.06	4.65	4.72	3.66	4.08	4.05	4.68	3.94	4.50	4.71	4.78
1369738_s_at	Crem	cAMP responsive element modulator	0	Flyp/lypnF+/+_114	0	0	1	1	0	0	0	0	0	0	5.84	5.88	6.05	6.06	6.05	5.89	6.10	6.28	5.74	5.96	6.29	6.31	5.61	6.04	6.08	6.20	5.76	6.30	6.33	6.30
1369785_at	Ppat	phosphoribosyl pyrophosphate amidotransferase	0	Flyp/lypnF+/+_114	0	0	1	1	0	0	0	0	0	0	8.24	8.24	8.47	8.25	8.05	8.11	8.31	8.39	8.13	8.43	8.74	8.62	8.39	8.59	8.41	8.56	8.34	8.70	8.85	8.72
1370455_a_at	Olfm3	olfactomedin 3	0	Flyp/lypnF+/+_114	0	0	1	1	0	0	0	0	0	0	3.03	3.55	3.75	3.41	3.17	3.07	3.23	3.55	3.02	3.33	3.50	3.76	2.99	3.65	3.77	3.52	3.04	3.51	3.83	3.54
1370517_at	Nptx1	neuronal pentraxin I	0	Flyp/lypnF+/+_114	0	0	1	1	0	0	0	0	0	0	4.31	4.45	4.73	4.62	4.52	4.41	4.78	4.50	4.85	5.17	5.71	6.14	4.84	5.46	5.72	6.20	5.08	5.27	5.73	6.23
1370566_at	LOC1003659!	Rdh2 protein-like /// retinol dehydrogenase 16 (all-trans)	0	Flyp/lypnF+/+_114	0	0	1	1	0	0	0	0	0	0	3.59	3.62	3.68	3.85	3.48	3.79	3.74	3.84	3.53	3.50	3.78	4.09	3.74	3.77	3.76	4.07	3.51	3.70	3.95	4.03
1370669_a_at	Pde10a	phosphodiesterase 10A	0	Flyp/lypnF+/+_114	0	0	1	1	0	0	0	0	0	0	5.41	5.51	5.51	5.75	5.56	5.41	5.63	5.42	5.00	4.93	5.10	5.45	4.52	5.06	5.26	5.18	4.95	5.21	5.33	5.50
1371139_at	Pls3	plastin 3	0	Flyp/lypnF+/+_114	0	0	1	1	0	0	0	0	0	0	6.93	7.02	7.09	6.80	6.88	7.01	6.97	7.13	6.69	6.85	7.21	7.29	6.35	6.97	7.32	7.19	6.81	7.07	7.05	7.22
1371757_s_at	LOC1003652:	keratin 6A-like /// similar to keratin complex 2, basic, gene 6a /// similar to keratin complex	0	Flyp/lypnF+/+_114	0	0	1	1	0	0	0	0	0	0	4.06	4.15	4.14	4.19	4.29	4.37	4.34	4.07	4.14	4.60	5.18	5.44	4.41	4.66	5.37	5.40	4.17	4.92	5.42	5.37
1371950_at	Pdzd11	PDZ domain containing 11	0	Flyp/lypnF+/+_114	0	0	1	1	0	0	0	0	0	0	6.55	6.77	6.77	6.96	6.49	6.74	6.85	6.93	6.58	6.91	6.98	7.10	6.19	6.81	6.91	7.00	6.60	6.88	7.10	7.13
1372176_at	Prkca	protein kinase C, alpha	0	Flyp/lypnF+/+_114	0	0	1	1	0	0	0	0	0	0	4.53	4.73	4.79	4.87	4.87	4.86	4.98	4.97	4.51	4.89	5.12	5.09	4.56	4.86	4.98	5.08	4.66	5.04	5.09	5.25
1372325_at	Emilin1	elastin microfibril interfacer 1	0	Flyp/lypnF+/+_114	0	0	1	1	0	0	0	0	0	0	7.56	7.66	7.74	7.68	7.61	7.63	7.57	7.51	8.13	8.25	8.51	8.69	8.25	8.22	8.46	8.56	8.07	8.36	8.54	8.64
1372726_at	Hist1h4b /// I	histone cluster 1, H4b /// histone cluster 1, H4m /// histone cluster 2, H4 /// histone cluster 2	0	Flyp/lypnF+/+_114	0	0	1	1	0	0	0	0	0	0	7.27	7.54	7.75	7.59	7.24	7.55	7.63	7.61	7.36	7.87	7.89	8.20	7.31	7.89	8.01	8.12	7.60	8.08	8.22	8.40
1372947_at	Pls3	plastin 3	0	Flyp/lypnF+/+_114	0	0	1	1	0	0	0	0	0	0	9.55	9.67	9.76	9.68	9.50	9.70	9.71	9.89	9.42	9.69	9.89	10.06	9.40	9.78	9.85	9.94	9.52	9.84	10.05	10.16
1372966_at	Mfsd2a	major facilitator superfamily domain containing 2A	0	Flyp/lypnF+/+_114	0	0	1	1	0	0	0	0	0	0	5.10	5.14	5.44	5.19	4.95	5.21	5.31	5.45	4.99	5.22	5.64	5.91	5.05	5.16	5.29	5.51	5.20	5.48	5.72	5.62
1373161_at	Tmem98	transmembrane protein 98	0	Flyp/lypnF+/+_114	0	0	1	1	0	0	0	0	0	0	8.79	8.98	9.18	9.13	8.89	8.95	9.06	9.12	8.42	8.68	8.99	9.09	8.28	8.73	8.85	9.09	8.50	8.76	8.95	9.10
1373335_at	Zdhhc9	zinc finger, DHHC-type containing 9	0	Flyp/lypnF+/+_114	0	0	1	1	0	0	0	0	0	0	7.83	8.07	8.13	8.15	7.81	8.06	8.12	8.18	8.03	8.28	8.50	8.59	8.00	8.22	8.47	8.55	7.96	8.32	8.45	8.78
1373344_at	RGD1562200	similar to GS2 gene	0	Flyp/lypnF+/+_114	0	0	1	1	0	0	0	0	0	0	5.41	5.67	5.66	5.78	5.58	5.67	5.74	5.61	5.30	5.80	5.91	5.77	5.18	5.66	5.79	5.85	5.39	5.68	5.89	5.79
1373484_at	Klhl13	kelch-like family member 13	0	Flyp/lypnF+/+_114	0	0	1	1	0	0	0	0	0	0	7.06	7.35	7.45	7.55	7.16	7.24	7.46	7.62	7.08	7.36	7.55	7.68	7.00	7.43	7.51	7.61	7.11	7.36	7.55	7.68
1373686_at	Serpina6	serpin peptidase inhibitor, clade A (alpha-1 antiproteinase, antitrypsin), member 6	0	Flyp/lypnF+/+_114	0	0	1	1	0	0	0	0	0	0	3.66	5.24	5.17	4.73	3.31	5.18	5.28	4.79	3.04	3.78	3.87	4.68	2.93	4.84	4.54	4.64	3.02	4.32	4.43	4.93
1373774_at	Rit2	Ras-like without CAAX 2	0	Flyp/lypnF+/+_114	0	0	1	1	0	0	0	0	0	0	6.49	6.61	6.91	7.00	6.70	6.85	6.96	6.93	6.74	7.46	7.75	8.09	7.32	7.67	7.86	8.15	6.80	7.32	7.74	8.00
1374218_at	LOC10091284	uncharacterized LOC100912843	0	Flyp/lypnF+/+_114	0	0	1	1	0	0	0	0	0	0	4.06	4.12	4.06	4.12	3.93	3.97	4.33	4.10	3.79	4.02	4.16	4.40	3.97	4.19	4.14	4.36	3.86	3.98	4.42	4.37
1374541_at	Gys1	glycogen synthase 1, muscle	0	Flyp/lypnF+/+_114	0	0	1	1	0	0	0	0	0	0	5.62	5.81	5.97	6.07	5.70	5.92	6.11	5.99	5.67	5.99	6.16	6.33	5.77	6.08	6.08	6.23	5.73	6.17	6.31	6.30
1374585_at	Echdc1	enoyl CoA hydratase domain containing 1	0	Flyp/lypnF+/+_114	0	0	1	1	0	0	0	0	0	0	9.47	9.37	9.62	9.81	9.63	9.40	9.58	9.93	9.51	9.64	9.91	10.26	9.84	9.73	9.94	10.12	9.50	9.64	9.89	10.07
1374600_at	Hist1h4b /// I	histone cluster 1, H4b /// histone cluster 1, H4m /// histone cluster 2, H4 /// histone cluster 2	0	Flyp/lypnF+/+_114	0	0	1	1	0	0	0	0	0	0	6.20	6.39	6.58	6.59	6.21	6.39	6.43	6.77	6.25	6.65	6.65	6.82	6.09	6.61	6.65	6.84	6.46	6.80	6.79	6.95
1374672_at	Tnni3k	TNNI3 interacting kinase	0	Flyp/lypnF+/+_114	0	0	1	1	0	0	0	0	0	0	5.71	5.81	5.92	5.94	5.81	5.76	5.94	5.77	5.70	5.94	6.18	6.33	6.16	6.22	6.32	6.38	5.79	5.92	6.20	6.29
1374929_at	Ccdc126 /// L	coiled-coil domain containing 126 /// coiled-coil domain containing 126-like	0	Flyp/lypnF+/+_114	0	0	1	1	0	0	0	0	0	0	7.31	7.33	7.72	7.60	7.59	7.55	7.73	7.80	7.10	7.40	7.62	7.91	7.40	7.41	7.61	7.79	7.26	7.43	7.69	8.07
1374966_at	Dcx	doublecortin	0	Flyp/lypnF+/+_114	0	0	1	1	0	0	0	0	0	0	8.47	8.20	8.60	8.75	8.54	8.25	8.49	8.83	8.44	8.38	8.87	9.47	9.02	8.54	8.85	9.26	8.66	8.56	8.89	9.32
1375877_at	Syt4	synaptotagmin IV	0	Flyp/lypnF+/+_114	0	0	1	1	0	0	0	0	0	0	8.69	8.78	9.04	9.15	8.75	8.78	9.05	9.06	8.40	8.89	9.29	9.59	8.52	9.07	9.27	9.58	8.54	9.15	9.34	9.71
1376105_at	Col14a1	collagen, type XIV, alpha 1	0	Flyp/lypnF+/+_114	0	0	1	1	0	0	0	0	0	0	7.44	7.44	7.12	7.04	7.57	7.53	7.00	7.41	7.88	7.84	8.18	8.57	8.02	8.11	8.30	8.59	7.89	8.04	8.25	8.78
1376170_at	Col14a1	collagen, type XIV, alpha 1	0	Flyp/lypnF+/+_114	0	0	1	1	0	0	0	0	0	0	6.50	6.38	6.28	6.03	6.70	6.56	6.02	6.39	7.55	7.58	7.72	8.19	7.97	7.80	7.89	8.19	7.67	7.66	7.90	8.38
1376191_at	Hpgd	hydroxyprostaglandin dehydrogenase 15 (NAD)	0	Flyp/lypnF+/+_114	0	0	1	1	0	0	0	0	0	0	8.83	9.00	9.19	9.21	8.92	9.00	9.16	9.31	8.52	8.63	8.92	9.07	8.13	8.65	8.88	9.08	8.34	8.75	8.95	9.30
1376355_at	FAM120C	family with sequence similarity 120C	0	Flyp/lypnF+/+_114	0	0	1	1	0	0	0	0	0	0	4.99	5.02	5.05	5.00	4.78	4.94	5.13	5.16	4.89	5.08	5.26	5.21	4.51	5.11	5.23	5.09	4.88	5.11	5.32	5.09
1376649_at	Snf1lk2	SNF1-like kinase 2	0	Flyp/lypnF+/+_114	0	0	1	1	0	0	0	0	0	0	8.98	9.01	9.14	9.20	8.91	9.03	9.13	9.13	8.91	9.19	9.33	9.34	9.08	9.24	9.23	9.33	8.94	9.36	9.48	9.44
1376654_at	Plcxd3	phosphatidylinositol-specific phospholipase C, X domain containing 3	0	Flyp/lypnF+/+_114	0	0	1	1	0	0	0	0	0	0	7.14	6.85	7.10	7.37	7.15	7.08	6.96	7.00	7.09	7.36	7.52	7.82	6.55	7.39	7.44	7.71	7.32	7.52	7.38	7.97
1376787_at	Lingo2	leucine rich repeat and Ig domain containing 2	0	Flyp/lypnF+/+_114	0	0	1	1	0	0	0	0	0	0	6.37	6.27	6.38	6.35	6.16	6.38	6.41	6.45	6.16	6.28	6.58	7.00	6.07	6.31	6.54	6.73	6.34	6.48	6.83	7.02
1376958_at	LOC1009111(	leukocyte elastase inhibitor A-like	0	Flyp/lypnF+/+_114	0	0	1	1	0	0	0	0	0	0	3.62	3.59	3.60	3.67	3.48	3.72	3.64	3.32	3.66	4.36	5.01	5.84	3.71	5.16	5.84	5.90	3.75	5.05	5.82	6.55
1377340_at	Tfpi2	tissue factor pathway inhibitor 2	0	Flyp/lypnF+/+_114	0	0	1	1	0	0	0	0	0	0	3.57	3.41	3.36	3.65	3.41	3.19	3.61	3.78	3.36	3.65	3.84	4.40	3.73	3.65	4.17	4.45	3.44	3.59	4.05	4.39
1377375_at	Aass	aminoadipate-semialdehyde synthase	0	Flyp/lypnF+/+_114	0	0	1	1	0	0	0	0	0	0	9.09	9.19	9.39	9.56	9.07	9.17	9.31	9.43	8.51	9.04	9.29	9.55	8.82	9.14	9.34	9.43	8.85	9.16	9.34	9.51
1378000_at	Tmem178b	transmembrane protein 178B	0	Flyp/lypnF+/+_114	0	0	1	1	0	0	0	0	0	0	7.69	7.84	8.03	8.11	7.78	7.71	7.93	8.00	7.21	7.65	7.80	8.11	7.41	7.67	7.84	8.12	7.14	7.48	7.75	7.94
1378002_at	Hspa4l	heat shock protein 4-like	0	Flyp/lypnF+/+_114	0	0	1	1	0	0	0	0	0	0	7.23	7.33	7.50	7.61	7.22	7.23	7.48	7.37	7.32	7.50	7.75	7.82	7.24	7.50	7.73	7.74	7.29	7.64	7.83	7.94
1378675_at	Tgm3	transglutaminase 3, E polypeptide	0	Flyp/lypnF+/+_114	0	0	1	1	0	0	0	0	0	0	7.08	7.38	7.47	7.52	7.12	7.56	7.52	7.50	6.51	6.52	6.83	7.06	6.50	6.67	6.58	6.84	6.92	6.87	7.14	7.41
1378925_at	Crem	cAMP responsive element modulator	0	Flyp/lypnF+/+_114	0	0	1	1	0	0	0	0	0	0	6.71	6.63	7.14	7.06	6.71	6.65	6.90	7.32	6.29	6.70	7.37	7.39	5.93	6.69	6.77	7.32	6.39	7.26	7.56	7.48
1379197_at	Cacna1h	calcium channel, voltage-dependent, T type, alpha 1H subunit	0	Flyp/lypnF+/+_114	0	0	1	1	0	0	0	0	0	0	3.63	3.65	3.78	3.83	3.94	3.86	3.93	3.74	3.76	3.78	3.78	3.99	3.79	3.86	3.82	3.93	3.64	3.89	3.82	3.91
1379386_at	Gtse1	G-2 and S-phase expressed 1	0	Flyp/lypnF+/+_114	0	0	1	1	0	0	0	0	0	0	6.26	6.20	6.19	6.43	6.06	6.31	6.37	6.47	5.98	6.24	6.51	6.55	6.23	6.26	6.36	6.43	6.03	6.33	6.40	6.53
1379414_at	Ube2ql1	ubiquitin-conjugating enzyme E2Q family-like 1	0	Flyp/lypnF+/+_114	0	0	1	1	0	0	0	0	0	0	6.32	6.57	6.81	6.78	6.50	6.54	6.92	6.82	6.49	6.77	7.05	7.29	6.34	6.78	7.02	7.17	6.43	6.73	7.03	7.36
1380637_at	Megf11	multiple EGF-like-domains 11	0	Flyp/lypnF+/+_114	0	0	1	1	0	0	0	0	0	0	4.47	4.70	4.81	4.88	4.43	4.78	4.76	4.77	4.22	4.53	4.60	4.81	4.31	4.50	4.73	4.93	4.13	4.69	4.68	5.14
1381465_at	Tmem229a	transmembrane protein 229A	0	Flyp/lypnF+/+_114	0	0	1	1	0	0	0	0	0	0	4.14	4.10	4.15	4.12	4.19	4.37	4.23	4.00	4.03	4.12	4.12	4.36	3.99	4.22	4.14	4.19	4.10	4.38	4.16	4.20
1381931_at	Ablim1	actin-binding LIM protein 1	0	Flyp/lypnF+/+_114	0	0	1	1	0	0	0	0	0	0	4.07	4.23	4.12	4.20	4.06	3.95	4.33	4.29	3.86	4.23	4.27	4.63	4.01	4.13	4.08	4.45	4.08	4.00	4.55	4.36
1382469_at	---	---	0	Flyp/lypnF+/+_114	0	0	1	1	0	0	0	0	0	0	6.66	6.93	6.96	7.08	6.59	6.99	6.94	6.90	6.55	6.95	7.04	7.12	6.50	6.97	6.95	7.07	6.55	7.02	7.02	7.20

Affymetrix Probeset ID	Gene Symbol	Gene Name	unique BB on P7 n=822	Figure 1B P7 Venn n=1526	DRlyp/lyp STEM P7 n=712	DR+/+ STEM P7 n=936	Flyp/lyp STEM P7 n=520	F+/+ STEM P7 n=465	Figure 1B P0 Venn n=1071	DRlyp/lyp STEM P0 n=442	DR+/+ STEM P0 n=437	Flyp/lyp STEM P0 n=643	F+/+ STEM P0 n=632	Fig1D n=25	Mean log2 intensity DRlyp/lyp 20	Mean log2 intensity DRlyp/lyp 30	Mean log2 intensity DRlyp/lyp 40	Mean log2 intensity DRlyp/lyp 50	Mean log2 intensity DR+/+ 20	Mean log2 intensity DR+/+ 30	Mean log2 intensity DR+/+ 40	Mean log2 intensity DR+/+ 50	Mean log2 intensity Flyp/lyp 20	Mean log2 intensity Flyp/lyp 30	Mean log2 intensity Flyp/lyp 40	Mean log2 intensity Flyp/lyp 50	Mean log2 intensity Flyp20	Mean log2 intensity Flyp30	Mean log2 intensity Flyp40	Mean log2 intensity Flyp50	Mean log2 intensity F+/+ 20	Mean log2 intensity F+/+ 30	Mean log2 intensity F+/+ 40	Mean log2 intensity F+/+ 50
1383699_at	Rit2	Ras-like without CAAX 2	0	Flyp/lypnF+/+_114	0	0	1	1	0	0	0	0	0	0	5.69	5.70	5.98	6.02	5.72	5.93	6.04	5.99	5.75	6.42	6.70	7.09	6.13	6.67	6.87	7.15	5.82	6.36	6.74	6.93
1383713_at	LOC1003611	rCG29620-like	0	Flyp/lypnF+/+_114	0	0	1	1	0	0	0	0	0	0	5.36	5.35	5.52	5.67	5.62	5.43	5.56	5.37	4.78	4.89	5.06	5.35	5.02	5.02	5.13	5.22	4.76	5.38	5.41	5.43
1384004_at	Gpr158	G protein-coupled receptor 158	0	Flyp/lypnF+/+_114	0	0	1	1	0	0	0	0	0	0	8.10	8.18	8.34	8.58	8.10	8.12	8.30	8.45	8.07	8.36	8.52	8.84	8.41	8.30	8.68	8.86	8.18	8.36	8.68	8.94
1384877_at	Aqp11	aquaporin 11	0	Flyp/lypnF+/+_114	0	0	1	1	0	0	0	0	0	0	4.76	4.98	5.23	5.06	4.83	4.95	5.16	4.97	4.57	4.82	5.08	5.15	4.52	5.09	4.93	4.97	4.48	4.83	5.10	5.05
1385061_at	Tmem229a	transmembrane protein 229A	0	Flyp/lypnF+/+_114	0	0	1	1	0	0	0	0	0	0	3.17	3.55	3.75	3.72	3.11	3.54	3.35	3.52	3.25	3.78	4.11	4.26	3.12	3.79	3.66	4.22	3.27	3.93	4.33	4.39
1385464_at	Foxq1	forkhead box Q1	0	Flyp/lypnF+/+_114	0	0	1	1	0	0	0	0	0	0	3.77	3.98	4.13	4.08	3.75	3.85	4.30	3.94	3.42	3.58	3.62	3.90	3.69	3.86	3.74	3.92	3.28	3.58	3.51	3.94
1385481_at	Dsel	dermatan sulfate epimerase-like	0	Flyp/lypnF+/+_114	0	0	1	1	0	0	0	0	0	0	6.19	6.54	6.63	6.55	6.20	6.42	6.42	6.45	6.17	6.57	6.62	6.92	6.25	6.58	6.61	6.73	6.24	6.66	6.81	6.87
1387160_at	Kcne3	potassium voltage-gated channel, Isk-related family, member 3	0	Flyp/lypnF+/+_114	0	0	1	1	0	0	0	0	0	0	5.08	5.01	5.03	5.00	5.09	5.10	5.13	5.08	4.97	4.86	5.11	5.56	4.89	4.92	5.15	5.55	4.80	4.92	5.27	5.36
1387179_at	Adcy8	adenylate cyclase 8 (brain)	0	Flyp/lypnF+/+_114	0	0	1	1	0	0	0	0	0	0	4.47	4.82	4.94	4.70	4.60	4.39	4.66	4.42	5.28	5.87	5.87	5.88	4.99	5.63	5.65	5.80	4.89	5.70	5.67	5.76
1387437_at	Fbxo2	F-box protein 2	0	Flyp/lypnF+/+_114	0	0	1	1	0	0	0	0	0	0	5.25	5.53	5.70	5.58	5.24	5.46	5.51	5.49	5.43	5.53	5.73	6.02	5.25	5.47	5.73	5.78	5.29	5.69	5.82	5.79
1387601_at	Dcx	doublecortin	0	Flyp/lypnF+/+_114	0	0	1	1	0	0	0	0	0	0	3.11	2.98	3.19	3.21	3.16	2.93	3.05	3.29	2.97	3.16	3.21	3.48	3.21	3.18	3.38	3.46	3.17	3.20	3.25	3.54
1387631_at	Hpgd	hydroxyprostaglandin dehydrogenase 15 (NAD)	0	Flyp/lypnF+/+_114	0	0	1	1	0	0	0	0	0	0	7.98	8.19	8.37	8.47	8.05	8.06	8.27	8.60	7.69	7.78	8.09	8.59	7.13	7.81	8.13	8.34	7.43	7.89	8.17	8.44
1387662_at	Syt4	synaptotagmin IV	0	Flyp/lypnF+/+_114	0	0	1	1	0	0	0	0	0	0	7.16	7.19	7.52	7.49	7.16	7.23	7.45	7.20	6.71	7.05	7.65	7.93	5.93	7.45	7.74	7.85	6.81	7.50	7.56	7.88
1387714_at	Crem	cAMP responsive element modulator	0	Flyp/lypnF+/+_114	0	0	1	1	0	0	0	0	0	0	3.91	3.93	4.04	3.99	3.82	3.75	4.05	4.12	3.63	3.95	4.16	4.18	3.63	3.93	3.82	4.02	3.60	4.12	4.12	4.19
1387817_at	Nsg1	neuron specific gene family member 1	0	Flyp/lypnF+/+_114	0	0	1	1	0	0	0	0	0	0	7.49	7.75	7.95	7.99	7.47	7.70	7.96	7.90	7.59	8.18	8.26	8.46	7.43	7.94	8.10	8.28	7.54	8.04	8.13	8.34
1387859_at	LOC1009110	cysteine desulfurase, mitochondrial-like /// NFS1 nitrogen fixation 1 homolog (S. cerevisiae)	0	Flyp/lypnF+/+_114	0	0	1	1	0	0	0	0	0	0	8.99	9.26	9.46	9.46	9.01	9.26	9.36	9.35	9.13	9.42	9.53	9.66	9.02	9.34	9.58	9.62	9.11	9.40	9.59	9.65
1389067_at	Slco4a1	solute carrier organic anion transporter family, member 4a1	0	Flyp/lypnF+/+_114	0	0	1	1	0	0	0	0	0	0	7.63	7.86	8.23	8.12	7.78	7.86	7.89	8.23	7.53	7.91	8.15	8.31	7.77	7.75	7.86	8.03	7.50	7.93	8.15	8.12
1389142_at	Sqrdl	sulfide quinone reductase-like (yeast)	0	Flyp/lypnF+/+_114	0	0	1	1	0	0	0	0	0	0	9.24	9.47	9.64	9.64	9.27	9.42	9.58	9.61	8.69	9.06	9.20	9.43	8.52	8.98	9.25	9.28	9.11	9.21	9.71	9.73
1389253_at	Vnn1	vanin 1	0	Flyp/lypnF+/+_114	0	0	1	1	0	0	0	0	0	0	4.05	4.07	4.05	4.29	4.02	4.02	4.05	4.39	3.98	4.07	4.66	5.14	4.44	4.46	4.75	4.96	4.52	4.42	5.01	5.41
1389651_at	Apln	apelin	0	Flyp/lypnF+/+_114	0	0	1	1	0	0	0	0	0	0	3.40	3.85	3.71	3.87	3.63	3.90	3.74	3.73	3.74	3.89	4.36	4.63	3.99	4.13	4.56	4.96	3.46	3.93	4.60	4.53
1389682_at	Chac2	ChaC, cation transport regulator homolog 2 (E. coli)	0	Flyp/lypnF+/+_114	0	0	1	1	0	0	0	0	0	0	5.84	5.92	6.18	6.27	5.86	5.93	6.24	6.21	5.98	5.99	6.21	6.60	5.98	5.86	6.24	6.56	5.99	5.87	6.19	6.66
1389725_at	Tm7sf2	transmembrane 7 superfamily member 2	0	Flyp/lypnF+/+_114	0	0	1	1	0	0	0	0	0	0	6.24	6.35	6.47	6.44	6.25	6.36	6.52	6.50	6.21	6.44	6.64	6.73	6.19	6.49	6.59	6.69	6.14	6.45	6.57	6.71
1390208_at	Htatip2	HIV-1 Tat interactive protein 2	0	Flyp/lypnF+/+_114	0	0	1	1	0	0	0	0	0	0	6.42	6.60	6.69	6.89	6.59	6.56	6.67	6.83	6.48	6.69	6.80	7.05	6.41	6.61	6.92	6.99	6.49	6.68	6.84	7.05
1391546_at	---	---	0	Flyp/lypnF+/+_114	0	0	1	1	0	0	0	0	0	0	5.39	5.60	5.75	5.86	5.43	5.54	5.88	5.82	5.80	5.87	6.35	6.97	5.92	6.06	6.30	6.79	5.57	5.92	6.34	6.92
1391573_at	Tnfrsf21	tumor necrosis factor receptor superfamily, member 21	0	Flyp/lypnF+/+_114	0	0	1	1	0	0	0	0	0	0	5.81	5.93	6.05	6.18	5.78	6.07	6.18	6.13	5.48	5.89	6.03	6.41	5.88	6.10	6.13	6.40	5.65	6.32	6.54	6.78
1391679_at	LOC691083	hypothetical protein LOC691083	0	Flyp/lypnF+/+_114	0	0	1	1	0	0	0	0	0	0	5.29	5.26	5.54	5.59	5.46	5.67	5.53	5.57	5.33	5.47	5.71	6.03	5.52	5.64	5.62	5.75	5.40	5.64	5.80	5.93
1391766_at	Hs3st5	heparan sulfate (glucosamine) 3-O-sulfotransferase 5	0	Flyp/lypnF+/+_114	0	0	1	1	0	0	0	0	0	0	2.95	2.88	2.97	3.32	3.21	2.96	3.24	3.12	2.89	2.68	3.00	3.57	3.07	2.97	2.93	3.44	2.89	2.96	3.38	3.39
1391976_at	Six4	SIX homeobox 4	0	Flyp/lypnF+/+_114	0	0	1	1	0	0	0	0	0	0	5.11	5.33	5.51	5.14	5.10	4.91	5.29	5.21	4.33	4.77	5.01	5.00	4.81	4.85	4.97	4.97	4.65	4.96	5.17	5.07
1392883_at	Limch1	LIM and calponin homology domains 1	0	Flyp/lypnF+/+_114	0	0	1	1	0	0	0	0	0	0	6.51	6.42	6.65	6.80	6.50	6.35	6.69	6.77	6.59	6.53	6.96	7.23	6.53	6.67	7.03	7.13	6.83	6.70	6.82	7.26
1393030_at	LOC1009104	protein PRRC2C-like /// hypothetical protein LOC683963	0	Flyp/lypnF+/+_114	0	0	1	1	0	0	0	0	0	0	3.38	3.34	3.60	3.66	3.23	3.50	3.42	3.49	3.92	4.46	4.32	4.75	3.98	4.13	4.28	4.68	3.84	4.25	4.33	4.47
1393291_at	Ube2ql1	ubiquitin-conjugating enzyme E2Q family-like 1	0	Flyp/lypnF+/+_114	0	0	1	1	0	0	0	0	0	0	6.09	6.35	6.51	6.52	6.06	6.23	6.54	6.51	6.24	6.41	6.76	7.03	6.03	6.58	6.82	6.99	6.20	6.45	6.81	7.09
1393321_at	---	---	0	Flyp/lypnF+/+_114	0	0	1	1	0	0	0	0	0	0	5.49	5.77	5.78	5.83	5.48	5.80	5.80	5.74	5.30	5.73	5.78	5.91	5.49	5.76	5.72	5.87	5.39	5.68	5.89	5.98
1393550_at	Crem	cAMP responsive element modulator	0	Flyp/lypnF+/+_114	0	0	1	1	0	0	0	0	0	0	5.86	6.00	6.07	5.69	6.04	5.87	5.83	5.85	5.27	5.56	5.77	5.87	5.10	5.27	5.54	5.83	5.34	5.70	6.10	5.77
1393584_at	Tnfrsf21	tumor necrosis factor receptor superfamily, member 21	0	Flyp/lypnF+/+_114	0	0	1	1	0	0	0	0	0	0	6.31	6.52	6.43	6.61	6.18	6.45	6.55	6.54	5.92	6.31	6.45	6.57	6.22	6.63	6.59	6.61	6.16	6.62	6.78	6.86
1393592_at	Hs3st5	heparan sulfate (glucosamine) 3-O-sulfotransferase 5	0	Flyp/lypnF+/+_114	0	0	1	1	0	0	0	0	0	0	2.83	2.80	2.95	3.04	2.98	2.90	2.90	3.21	2.80	2.87	2.90	3.46	2.90	2.94	3.01	3.42	2.88	2.86	2.99	3.54
1393841_at	Lingo2	leucine rich repeat and Ig domain containing 2	0	Flyp/lypnF+/+_114	0	0	1	1	0	0	0	0	0	0	5.35	5.36	5.42	5.23	5.14	5.33	5.14	5.67	5.21	5.47	5.90	6.44	5.05	5.43	5.64	6.03	5.29	5.53	6.10	6.37
1393893_at	Morn5	MORN repeat containing 5	0	Flyp/lypnF+/+_114	0	0	1	1	0	0	0	0	0	0	2.80	2.95	3.01	2.88	2.85	2.88	3.00	2.97	2.76	3.15	3.01	3.41	2.85	3.24	3.17	3.36	2.81	3.19	3.33	3.33
1393952_at	Ccdc68	coiled-coil domain containing 68	0	Flyp/lypnF+/+_114	0	0	1	1	0	0	0	0	0	0	3.11	3.25	3.29	3.25	3.13	3.18	3.33	3.53	3.07	3.43	3.49	3.85	3.02	3.06	3.50	3.66	3.00	3.18	3.66	3.63
1394434_at	Snf1lk2	SNF1-like kinase 2	0	Flyp/lypnF+/+_114	0	0	1	1	0	0	0	0	0	0	5.25	5.46	5.51	5.71	5.23	5.34	5.71	5.48	5.29	5.71	5.96	6.05	5.13	5.83	5.73	5.95	5.53	5.79	6.01	6.22
1394459_at	---	---	0	Flyp/lypnF+/+_114	0	0	1	1	0	0	0	0	0	0	8.34	8.71	8.85	8.88	8.30	8.66	8.76	8.68	8.31	8.62	8.70	8.82	8.17	8.62	8.70	8.83	8.35	8.62	8.80	8.87
1394946_at	Rgd1310049	uncharacterized LOC292199	0	Flyp/lypnF+/+_114	0	0	1	1	0	0	0	0	0	0	7.01	7.27	7.52	7.51	7.09	7.01	7.40	7.50	7.31	7.87	8.22	8.52	7.77	8.05	7.99	8.43	7.66	8.20	8.42	8.51
1397184_at	Rgd1310049	uncharacterized LOC292199	0	Flyp/lypnF+/+_114	0	0	1	1	0	0	0	0	0	0	6.97	7.20	7.34	7.37	7.05	7.06	7.26	7.38	7.24	7.64	8.01	8.26	7.72	7.89	7.82	8.18	7.58	7.92	8.23	8.31
1397333_at	Samd12	sterile alpha motif domain containing 12	0	Flyp/lypnF+/+_114	0	0	1	1	0	0	0	0	0	0	3.40	3.55	3.60	3.70	3.29	3.45	3.62	3.65	3.20	3.52	3.58	3.80	3.48	3.51	3.51	3.73	3.31	3.57	3.90	3.94
1398276_at	Dlg2	discs, large homolog 2 (Drosophila)	0	Flyp/lypnF+/+_114	0	0	1	1	0	0	0	0	0	0	4.49	4.45	4.75	4.73	4.48	4.55	4.59	4.64	4.40	4.52	4.66	4.92	4.55	4.66	5.00	5.07	4.53	4.32	4.69	5.10
1398662_at	Fam167a	family with sequence similarity 167, member A	0	Flyp/lypnF+/+_114	0	0	1	1	0	0	0	0	0	0	3.97	4.09	4.14	4.19	4.09	4.29	4.32	4.23	3.99	4.25	4.45	4.63	4.13	4.39	4.21	4.41	4.19	4.46	4.45	4.76
1380336_at	Irak3	interleukin-1 receptor-associated kinase 3	1	uniqDR+/+_356	0	1	0	0	Flyp/lypnF+/+_151	0	0	1	1	17	4.53	4.32	4.27	5.02	4.60	4.06	4.63	5.69	4.63	4.33	4.10	4.12	4.91	4.44	4.38	4.55	4.76	4.30	4.28	4.11
1388924_at	Angptl4	angiopoietin-like 4	1	uniqDR+/+_356	0	1	0	0	Flyp/lypnF+/+_151	0	0	1	1	0	5.79	5.64	5.19	6.08	5.56	5.43	5.70	6.48	5.77	5.38	4.94	4.93	5.92	5.69	5.63	6.05	5.90	5.35	5.08	5.07
1367574_at	Vim	vimentin	1	uniqDR+/+_356	0	1	0	0	uniqF+/+_163	0	0	0	1	0	8.98	8.85	8.74	9.24	8.86	8.89	8.89	9.65	8.57	8.60	8.27	8.40	8.76	8.50	8.53	8.45	8.81	8.50	8.36	8.27
1371499_at	Cd9	CD9 molecule	1	uniqDR+/+_356	0	1	0	0	uniqF+/+_163	0	0	0	1	0	6.57	6.50	6.55	6.86	6.60	6.39	6.92	7.51	6.53	6.52	6.00	6.21	6.83	6.37	6.38	6.41	6.82	6.13	5.98	6.17
1374104_at	Cldn5	claudin 5	1	uniqDR+/+_356	0	1	0	0	uniqF+/+_163	0	0	0	1	0	5.20	5.36	5.08	5.64	5.12	4.86	5.51	6.62	4.39	4.25	4.54	4.74	5.01	4.38	5.01	5.21	5.07	4.63	4.49	4.50
1382947_at	Rab1b	RAB1B, member RAS oncogene family	1	uniqDR+/+_356	0	1	0	0	uniqF+/+_163	0	0	0	1	0	6.80	6.82	6.92	6.85	6.88	6.90	6.97	6.90	6.74	6.83	6.84	7.01	6.64	6.84	6.75	6.92	6.76	6.94	6.78	6.88
1385270_s_at	LOC10036378	RAB1B, member RAS oncogene family-like /// RAB1A, member RAS oncogene family /// RAB	1	uniqDR+/+_356	0	1	0	0	uniqF+/+_163	0	0	0	1	0	5.29	5.30	5.41	5.42	5.11	5.02	5.66	5.75	4.99	5.42	5.53	5.73	4.25	5.37	5.19	5.61	5.23	5.05	4.93	4.61
1389402_at	Csrnp1	cysteine-serine-rich nuclear protein 1	1	uniqDR+/+_356	0	1	0	0	uniqF+/+_163	0	0	0	1	0	6.02	6.23	6.14	5.84	6.21	6.26	6.91	6.63	6.07	6.14	6.22	5.71	6.20	5.62	5.73	5.83	6.06	6.11	6.08	5.44
1393669_at	LOC10036378	RAB1B, member RAS oncogene family-like /// RAB1A, member RAS oncogene family /// RAB	1	uniqDR+/+_356	0	1	0	0	uniqF+/+_163	0	0	0	1	0	5.68	5.79	5.92	5.80	5.47	5.38	6.03	6.29	5.49	6.03	5.75	5.96	4.29	5.73	5.85	6.13	5.59	5.44	5.25	4.84
1367974_at	Anxa3	annexin A3	1	uniqDR+/+_356	0	1	0	0	uniqFlyp/lyp_145	0	0	1	0	0	7.33	6.72	6.66	7.23	7.19	6.51	6.95	7.69	7.45	6.77	6.36	6.06	7.43	6.75	6.82	6.68	7.41	6.74	6.28	6.22
1367975_at	Anxa3	annexin A3	1	uniqDR+/+_356	0	1	0	0	uniqFlyp/lyp_145	0	0	1	0	0	3.69	3.29	3.57	3.84	3.48	3.36	3.50	3.83	3.75	3.44	3.21	3.33	3.91	3.50	3.31	3.63	3.52	3.34	3.44	3.54
1368280_at	Ctsc	cathepsin C	1	uniqDR+/+_356	0	1	0	0	uniqFlyp/lyp_145	0	0	1	0	0	5.90	5.68	5.59	6.25	5.79	5.68	5.75	6.29	5.76	5.31	5.21	5.25	5.64	5.84	5.47	5.40	5.68	5.39	5.22	5.24
1368308_at	Myc	myelocytomatosis oncogene	1	uniqDR+/+_356	0	1	0	0	uniqFlyp/lyp_145	0	0	1	0	0	4.49	4.79	4.32	5.16	4.55	4.37	4.76	6.23	4.66	4.23	4.36	4.06	4.87	4.76	4.14	4.52	4.35	4.69	4.37	4.16
1395313_s_at	Anxa3	annexin A3	1	uniqDR+/+_356	0	1	0	0	uniqFlyp/lyp_145	0	0	1	0	0	6.21	6.15	5.99	6.35	6.08	6.22	6.20	6.54	6.27	6.13	6.12	6.01	6.44	6.28	6.13	6.21	6.45	6.21	6.11	6.21
1377698_at	Cd40	CD40 molecule, TNF receptor superfamily member 5	1	uniqDR+/+_356	0	1	0	0	0	0	0	0	0	9	5.08	4.97	4.85	6.15	5.03	4.86	4.99	6.64	4.72	4.84	4.91	4.95	4.83	4.88	4.86	4.99	4.73	4.69	4.84	4.85
1370631_at	Reg3g	regenerating islet-derived 3 gamma	1	uniqDR+/+_356	0	1	0	0	0	0	0	0	0	11	4.26	3.53	3.98	3.71	3.60	3.59	5.72	4.87	3.78	3.63	3.60	3.81	4.60	3.75	3.81	4.19	3.45	3.44	3.46	3.64
1389538_at	Nfkbia	nuclear factor of kappa light polypeptide gene enhancer in B-cells inhibitor, alpha	1	uniqDR+/+_356	0	1	0	0	0	0	0	0	0	13	9.18	9.39	9.50	9.46	9.31	9.27	10.04	9.83	9.19	9.26	9.30	9.07	9.37	9.32	9.00	8.99	9.13	9.23	9.08	8.75
1377353_a_at	Tnfsf13	tumor necrosis factor (ligand) superfamily, member 13	1	uniqDR+/+_356	0	1	0	0	0	0	0	0	0	15	4.75	4.65	4.58	4.98	4.55	4.50	4.74	5.31	4.16	4.37	4.07	4.22	4.57	4.23	4.23	4.51	4.18	4.19	4.38	4.03
1370080_at	Hmox1	heme oxygenase (decycling) 1	1	uniqDR+/+_356	0	1	0	0	0	0	0	0	0	18	5.07	4.99	4.85	5.98	4.98	5.14	5.59	6.23	5.03	4.98	5.07	5.18	5.00	4.89	5.05	5.54	5.10	4.84	5.01	5.09
1370118_at	Ccl17	chemokine (C-C motif) ligand 17	1	uniqDR+/+_356	0	1	0	0	0	0	0	0	0	20	5.74	5.78	5.64	6.25	5.53	5.62	5.76	6.22	5.57	5.69	5.79	6.01	5.84	5.77	5.71	6.09	5.70	5.90	5.86	5.99
1369911_at	Cxcr5	chemokine (C-X-C motif) receptor 5	1	uniqDR+/+_356	0	1	0	0	0	0	0	0	0	25	3.22	3.18	3.29	3.59	3.43	3.43	3.39	3.92	3.27	3.16	3.25	3.55	3.24	3.32	3.28	3.35	3.18	3.23	3.45	3.56
1367557_s_at	Gapdh /// LO	glyceraldehyde-3-phosphate dehydrogenase /// similar to Glyceraldehyde-3-phosphate dehy	1	uniqDR+/+_356	0	1	0	0	0	0	0	0	0	0	9.24	9.35	9.46	9.66	9.28	9.31	9.53	9.91	9.42	9.56	9.60	9.78	9.13	9.47	9.58	9.60	9.37	9.51	9.58	9.65
1367577_at	Hspb1	heat shock protein 1	1	uniqDR+/+_356	0	1	0	0	0	0	0	0	0	0	7.73	7.77	7.83	8.10	7.78	7.69	8.20	8.53	7.40	7.75	7.86	7.90	7.47	8.06	7.87	8.02	7.78	7.83	7.86	8.22
1367652_at	Igfbp3	insulin-like growth factor binding protein 3	1	uniqDR+/+_356	0	1	0	0	0	0	0	0	0	0	8.57	8.64	8.59	8.88	8.48	8.47	8.73	9.19	8.56	8.54	8.59	8.67	8.88	8.71	8.73	9.06	8.66	8.71	8.65	8.86
1367655_at	LOC1003594	thymosin, beta 10-like /// thymosin, beta 10-like /// thymosin, beta 10-like /// hypothetical p	1	uniqDR+/+_356	0	1	0	0	0	0	0	0	0	0	6.90	6.89	6.92	7.09	6.72	6.72	6.84	7.59	6.94	6.96	6.80	6.73	6.56	6.70	6.78	6.84	6.76	6.63	6.67	6.50
1367675_at	Cib1	calcium and integrin binding 1 (calmyrin)	1	uniqDR+/+_356	0	1	0	0	0	0	0	0	0	0	7.48	7.38	7.57	7.75	7.52	7.48	7.45	8.01	7.67	7.56	7.60	7.57	7.72	7.52	7.61	7.64	7.71	7.53	7.61	7.57
1367683_at	Kpna2 /// LO	karyopherin alpha 2 /// karyopherin alpha 2-like	1	uniqDR+/+_356	0	1	0	0	0	0	0	0	0	0	7.53	7.53	7.66	7.86	7.52	7.62	7.54	8.12	7.59	7.80	7.88	7.79	7.59	7.68	7.81	7.81	7.73	7.78	7.73	7.93
1367700_at	Fmod	fibromodulin	1	uniqDR+/+_356	0	1	0	0	0	0	0	0	0	0	4.15	4.27	4.44	4.28	3.88	4.22	4.33	4.60	4.04	3.88	3.87	3.93	4.31	4.05	3.81	3.91	4.10	4.16	3.92	3.93
1367791_at	Ramp1	receptor (G protein-coupled) activity modifying protein 1	1	uniqDR+/+_356	0	1	0	0	0	0	0	0	0	0	5.15	5.22	5.16	5.48	5.38	5.12	5.43	6.38	5.04	4.83	4.85	5.06	4.91	4.98	4.91	5.19	4.96	4.87	4.79	4.88
1367794_at	A2m /// LOC1	alpha-2-macroglobulin /// alpha-2-macroglobulin-like	1	uniqDR+/+_356	0	1	0	0	0	0	0	0	0	0	4.47	4.96	4.71	5.35	4.20	4.74	4.83	5.27	4.75	4.96	5.53	4.87	5.04	5.66	5.48	5.32	4.81	5.06	4.97	5.14
1367847_at	Nupr1	nuclear protein, transcriptional regulator, 1	1	uniqDR+/+_356	0	1	0	0	0	0	0	0	0	0	7.13	7.31	7.29	7.20	6.95	6.83	7.34	7.42	7.65	7.94	7.71	7.37	8.90	8.93	7.76	7.48	7.72	8.09	7.56	7.97
1367912_at	Ltbp1	latent transforming growth factor beta binding protein 1	1	uniqDR+/+_356	0	1	0	0	0	0	0	0	0	0	5.78	5.87	5.90	5.96	5.60	5.75	5.95	6.44	5.24	5.37	5.32	5.59	5.62	5.58	5.65	5.71	5.54	5.58	5.57	5.83
1367914_at	Emp3	epithelial membrane protein 3	1	uniqDR+/+_356	0	1	0	0	0	0	0	0	0	0	6.36	6.30	6.12	6.63	6.31	6.22	6.30	7.11	6.14	6.06	6.10	6.15	6.34	6.13	6.20	6.15	6.29	6.15	6.09	6.09
1367951_at	Pgam2	phosphoglycerate mutase 2 (muscle)	1	uniqDR+/+_356	0	1	0	0	0	0	0	0	0	0	3.35	3.40	3.55	3.65	3.39	3.46	3.62	3.97	3.64	3.48	3.43	3.45	3.30	3.40	3.52	3.38	3.39	3.68	3.35	3.52
1367999_at	Aldh2	aldehyde dehydrogenase 2 family (mitochondrial)	1	uniqDR+/+_356	0	1	0	0	0	0	0	0	0	0	5.88	5.83	5.88	6.16	5.92	5.78	6.20	6.54	6.02	6.04	5.87	6.04	5.96	6.06	6.08	6.20	6.05	6.04	6.04	6.04
1368028_at	Prph	peripherin	1	uniqDR+/+_356	0	1	0	0	0	0	0	0	0	0	5.09	4.77	5.10	5.21	4.88	4.97	5.38	5.25	4.49	6.81	4.56	4.70	4.54	5.06	4.58	4.64	4.95	4.91	4.71	5.17
1368109_at	St3gal5	ST3 beta-galactoside alpha-2,3-sialyltransferase 5	1	uniqDR+/+_356	0	1	0	0	0	0	0	0	0	0	6.58	6.68	6.69	6.83	6.45	6.56	6.72	6.98	6.68	6.72	6.67	6.74	6.90	6.74	6.84	6.67	6.84	6.70	6.85	6.88
1368114_at	Fgf13	fibroblast growth factor 13	1	uniqDR+/+_356	0	1	0	0	0	0	0	0	0	0	2.81	2.82	3.18	3.23	2.80	2.95	3.11	3.70	2.79	3.10	2.82	2.86	2.76	3.10	2.96	2.82	2.82	2.97	2.99	3.28
1368168_at	Slc34a2	solute carrier family 34 (sodium phosphate), member 2	1	uniqDR+/+_356	0	1	0	0	0	0	0	0	0	0	3.86	4.17	4.14	4.22	3.84	3.91	5.07	4.58	3.77	3.61	3.52	4.30	3.83	3.92	4.13	4.31	4.05	3.73	4.01	3.80
1368207_at	Fxyd5	FXYD domain-containing ion transport regulator 5	1	uniqDR+/+_356	0	1	0	0	0	0	0	0	0	0	6.57	6.37	6.44	7.07	6.57	6.38	6.70	7.57	6.44	6.35	6.36	6.51	6.62	6.39	6.55	6.63	6.62	6.44	6.54	6.52
1368251_at	Jak3	Janus kinase 3	1	uniqDR+/+_356	0	1	0	0	0	0	0	0	0	0	4.75	4.58	4.74	5.03	4.66	4.55	4.66	5.22	4.76	4.86	4.70	4.90	4.90	4.91	4.87	4.87	4.83	4.83	4.74	4.88
1368304_at	Fmo3	flavin containing monooxygenase 3	1	uniqDR+/+_356	0	1	0	0	0	0	0	0	0	0	4.97	5.00	5.17	5.00	4.81	4.91	5.61	5.14	4.66	4.55	4.36	4.72	4.92	4.63	4.70	4.95	4.95	4.50	4.67	4.93
1368387_at	Bdh1	3-hydroxybutyrate dehydrogenase, type 1	1	uniqDR+/+_356	0	1	0	0	0	0	0	0	0	0	4.62	4.78	5.02	4.99	4.67	4.52	4.61	5.45	5.40	5.43	5.30	5.64	5.27	5.26	5.31	5.55	5.20	5.33	5.58	5.62
1368407_at	Hpse	heparanase	1	uniqDR+/+_356	0	1	0	0	0	0	0	0	0	0	4.81	4.78	4.91	5.19	4.71	4.64	5.09	5.61	3.50	3.42	3.68	3.83	3.55	3.44	3.41	3.63	3.48	3.63	3.53	3.64
1368418_a_at	Cp	ceruloplasmin (ferroxidase)	1	uniqDR+/+_356	0	1	0	0	0	0	0	0	0	0	6.16	5.80	5.94	6.71	6.16	5.94	6.21	6.67	5.68	5.65	5.63	5.90	5.89	5.71	6.19	6.43	5.87	5.61	5.78	5.72
1368419_at	Cp	ceruloplasmin (ferroxidase)	1	uniqDR+/+_356	0	1	0	0	0	0	0	0	0	0	5.88	5.36	5.58	6.69	5.88	5.42	5.78	6.62	5.17	5.15	4.68	5.26	5.58	5.02	5.73	6.09	5.37	4.66	4.88	5.03
1368420_at	Cp	ceruloplasmin (ferroxidase)	1	uniqDR+/+_356	0	1	0	0	0	0	0	0	0	0	3.23	3.25	3.25	4.60	3.22	2.95	3.95	4.22	3.24	3.22	3.21	3.71	3.22	3.24	3.87	4.11	3.14	3.24	3.51	3.35
1368422_at	Meox2	mesenchyme homeobox 2	1	uniqDR+/+_356	0	1	0	0	0	0	0	0	0	0	3.26	3.21	3.15	3.65	3.37	3.17	3.30	4.22	2.91	2.76	2.66	2.72	2.98	2.73	3.06	3.69	3.05	2.92	2.97	2.88
1368563_at	Aspa	aspartoacylase	1	uniqDR+/+_356	0	1	0	0	0	0	0	0	0	0	3.85	4.03	4.01	4.22	3.71	4.09	3.98	4.29	4.14	3.89	4.19	4.05	3.98	4.14	4.09	4.25	4.05	4.16	4.34	4.19
1368605_at	Sh2b2	SH2B adaptor protein 2	1	uniqDR+/+_356	0	1	0	0	0	0	0	0	0	0	3.25	3.47	3.25	3.84	3.57	3.44	3.53	4.64	3.18	3.42	3.42	3.36	3.32	3.60	3.47	3.60	3.16	3.38	3.44	3.65
1368612_at	Itgb4	integrin, beta 4	1	uniqDR+/+_356	0	1	0	0	0	0	0	0	0	0	4.31	4.27	4.31	4.63	4.18	4.12	4.56	4.85	3.87	4.21	3.80	4.28	4.42	4.26	4.27	4.35	4.31	4.04	4.04	4.13
1368674_at	Pygl	phosphorylase, glycogen, liver	1	uniqDR+/+_356	0	1	0	0	0	0	0	0	0	0	4.60	4.76	4.79	5.00	4.63	4.81	4.66	5.44	4.36	4.60	4.41	4.57	4.37	4.60	4.62	4.51	4.48	4.40	4.55	4.59
1368723_at	Lat	linker for activation of T cells	1	uniqDR+/+_356	0	1	0	0	0	0	0	0	0	0	4.64	4.44	4.43	5.18	4.45	4.50	5.02	6.21	4.24	4.47	4.54	4.58	4.48	4.67	4.46	4.70	4.58	4.55	4.64	4.77
1368732_at	Tap2	transporter 2, ATP-binding cassette, sub-family B (MDR/TAP)	1	uniqDR+/+_356	0	1	0	0	0	0	0	0	0	0	5.18	5.49	5.29	6.00	5.00	5.37	5.47	5.79	5.16	5.45	5.27	5.45	4.77	5.34	4.97	5.51	5.27	5.24	5.20	5.28
1368760_at	Cxcl2	chemokine (C-X-C motif) ligand 2	1	uniqDR+/+_356	0	1	0	0	0	0	0	0	0	0	4.62	5.02	5.02	4.98	4.73	4.84	5.99	5.72	4.91	4.96	5.17	5.20	5.04	5.00	5.18	5.33	4.93	5.05	5.25	5.00
1368777_at	Bard1	BRCA1 associated RING domain 1	1	uniqDR+/+_356	0	1	0	0	0	0	0	0	0	0	4.02	4.07	4.12	4.31	3.68	4.11	4.13	4.34	3.93	4.15	4.14	4.15	3.74	4.14	4.35	4.39	3.98	4.26	4.12	4.24
1368783_at	Icos	inducible T-cell co-stimulator	1	uniqDR+/+_356	0	1	0	0	0	0	0	0	0	0	3.56	3.59	3.74	4.05	3.63	3.67	3.97	4.87	3.50	3.47	3.64	3.64	3.62	3.57	3.58	3.81	3.43	3.71	3.57	3.62
1368870_at	Id2	inhibitor of DNA binding 2	1	uniqDR+/+_356	0	1	0	0	0	0	0	0	0	0	6.44	6.44	6.51	6.77	6.63	6.49	6.85	7.06	5.62	5.43	5.55	5.38	5.93	5.66	5.89	5.72	5.73	5.57	5.49	5.37
1368906_at	Pgk1	phosphoglycerate kinase 1	1	uniqDR+/+_356	0	1	0	0	0	0	0	0	0	0	1.91	1.98	1.88	1.99	2.01	1.98	1.88	1.98	1.86	2.02	2.00	2.11	1.96	1.87	1.84	1.93	1.87	1.82	2.07	1.94
1368930_at	Kcnn4	potassium intermediate/small conductance calcium-activated channel, subfamily N, member	1	uniqDR+/+_356	0	1	0	0	0	0	0	0	0	0	2.59	2.56	2.70	2.92	2.52	2.54	2.97	3.18	2.37	2.45	2.45	2.67	2.58	2.44	2.50	2.70	2.49	2.51	2.52	2.64
1368948_at	Msn	moesin	1	uniqDR+/+_356	0	1	0	0	0	0	0	0	0	0	4.75	4.82	4.79	4.87	4.66	4.72	4.85	5.20	4.66	4.74	4.57	4.75	4.36	4.75	4.82	4.59	4.66	4.79	4.67	4.58
1368975_at	Cd38	CD38 molecule	1	uniqDR+/+_356	0	1	0	0	0	0	0	0	0	0	2.41	2.35	2.51	3.17	2.33	2.34	2.57	3.23	2.49	2.61	2.37	2.31	2.44	2.43	2.32	2.59	2.39	2.38	2.41	2.39
1368976_at	Cd38	CD38 molecule	1	uniqDR+/+_356	0	1	0	0	0	0	0	0	0	0	2.22	2.30	2.20	2.22	2.17	2.18	2.43	2.46	2.07	2.16	2.21	2.37	2.24	2.16	2.26	2.22	2.13	2.18	2.17	2.28
1368983_at	Hbegf	heparin-binding EGF-like growth factor	1	uniqDR+/+_356	0	1	0	0	0	0	0	0	0	0	4.04	3.95	3.99	4.18	3.82	4.09	4.56	4.50	3.90	4.06	4.23	4.30	4.22	4.16	4.29	4.43	4.18	4.18	4.36	4.40
1369129_at	Rasgrp1	RAS guanyl releasing protein 1 (calcium and DAG-regulated)	1	uniqDR+/+_356	0	1	0	0	0	0	0	0	0	0	2.49	2.44	2.55	2.51	2.54	2.64	2.51	2.94	2.33	2.51	2.68	2.42	2.38	2.50	2.57	2.57	2.52	2.53	2.46	2.61
1369130_at	Rasgrp1	RAS guanyl releasing protein 1 (calcium and DAG-regulated)	1	uniqDR+/+_356	0	1	0	0	0	0	0	0	0	0	2.70	2.62	2.60	2.81	2.54	2.56	2.70	3.20	2.56	2.64	2.54	2.57	2.70	2.65	2.70	2.64	2.57	2.70	2.66	2.63
1369214_a_at	Ciita	class II, major histocompatibility complex, transactivator	1	uniqDR+/+_356	0	1	0	0	0	0	0	0	0	0	4.55	4.50	4.37	4.99	4.33	4.56	4.71	5.14	4.35	4.53	4.54	4.74	4.31	4.57	4.58	4.89	4.37	4.47	4.57	4.63
1369393_at	Map3k8	mitogen-activated protein kinase kinase kinase 8	1	uniqDR+/+_356	0	1	0	0	0	0	0	0	0	0	4.02	4.06	3.88	4.37	3.94	4.08	4.00	4.89	3.69	3.75	3.75	3.43	3.91	3.57	3.71	3.68	3.64	3.58	3.90	3.71
1369425_at	Cdh13	cadherin 13	1	uniqDR+/+_356	0	1	0	0	0	0	0	0	0	0	4.74	4.94	4.99	4.79	4.54	4.69	4.99	5.06	4.48	4.53	4.60	4.76	4.63	4.70	4.65	4.37	4.63	4.55	4.70	4.76
1369519_at	Edn1	endothelin 1	1	uniqDR+/+_356	0	1	0	0	0	0	0	0	0	0	4.87	5.14	5.18	5.13	4.85	4.90	5.21	5.39	5.07	4.99	5.46	5.42	5.03	5.20	5.49	5.51	5.07	5.32	5.23	5.25
1369555_at	Ccr4	chemokine (C-C motif) receptor 4	1	uniqDR+/+_356	0	1	0	0	0	0	0	0	0	0	3.02	2.98	2.97	3.20	2.95	3.06	3.04	3.49	2.86	2.86	2.90	3.05	3.02	2.95	2.98	3.02	2.85	3.05	3.07	3.19
1369584_at	Socs3	suppressor of cytokine signaling 3	1	uniqDR+/+_356	0	1	0	0	0	0	0	0	0	0	4.51	4.75	4.67	4.57	4.50	4.81	5.07	4.94	4.77	5.18	5.25	4.86	4.53	4.87	5.07	4.90	4.72	4.67	4.69	4.53

Affymetrix Probeset ID	Gene Symbol	Gene Name	unique BB on P7 n=822	Figure 1B P7 Venn n=1526	DRlyp/lyp STEM P7 n=712	DR+/+ STEM P7 n=936	Flyp/lyp STEM P7 n=520	F+/+ STEM P7 n=465	Figure 1B P0 Venn n=1071	DRlyp/lyp STEM P0 n=442	DR+/+ STEM P0 n=437	Flyp/lyp STEM P0 n=643	F+/+ STEM P0 n=632	Fig1D n=25	Mean log2 intensity DRlyp/lyp 20	Mean log2 intensity DRlyp/lyp 30	Mean log2 intensity DRlyp/lyp 40	Mean log2 intensity DRlyp/lyp 50	Mean log2 intensity DR+/+ 20	Mean log2 intensity DR+/+ 30	Mean log2 intensity DR+/+ 40	Mean log2 intensity DR+/+ 50	Mean log2 intensity Flyp/lyp 20	Mean log2 intensity Flyp/lyp 30	Mean log2 intensity Flyp/lyp 40	Mean log2 intensity Flyp/lyp 50	Mean log2 intensity Flyp20	Mean log2 intensity Flyp30	Mean log2 intensity Flyp40	Mean log2 intensity Flyp50	Mean log2 intensity F+/+ 20	Mean log2 intensity F+/+ 30	Mean log2 intensity F+/+ 40	Mean log2 intensity F+/+ 50
1369625_at	Aqp1	aquaporin 1	1	uniqDR+/+_356	0	1	0	0	0	0	0	0	0	0	4.91	4.70	4.51	4.94	4.49	4.44	4.87	5.66	3.53	4.13	3.79	4.05	3.81	3.92	3.81	4.09	4.79	4.37	4.37	4.33
1369791_at	Clcn4	chloride channel, voltage-sensitive 4	1	uniqDR+/+_356	0	1	0	0	0	0	0	0	0	0	3.61	3.51	3.59	3.61	3.55	3.61	3.70	3.65	3.66	3.51	3.58	3.73	3.66	3.61	3.71	3.53	3.62	3.70	3.69	3.81
1369801_at	Sell	selectin L	1	uniqDR+/+_356	0	1	0	0	0	0	0	0	0	0	3.76	3.85	3.65	4.43	3.71	3.86	4.48	5.25	3.79	3.88	3.87	4.08	3.86	3.88	3.94	4.37	3.83	3.95	4.06	4.10
1369877_at	Cd8a	CD8a molecule	1	uniqDR+/+_356	0	1	0	0	0	0	0	0	0	0	2.86	2.89	2.88	3.16	2.91	2.93	3.18	4.05	2.82	2.92	2.94	2.99	2.80	2.84	2.96	3.05	2.82	2.96	2.92	3.10
1369959_at	Zfp36l1	zinc finger protein 36, C3H type-like 1	1	uniqDR+/+_356	0	1	0	0	0	0	0	0	0	0	9.62	9.53	9.40	10.07	9.83	9.54	9.76	10.69	9.23	9.12	9.12	9.19	9.65	9.10	9.21	9.52	9.52	9.24	9.24	9.13
1369979_at	Skap2	src kinase associated phosphoprotein 2	1	uniqDR+/+_356	0	1	0	0	0	0	0	0	0	0	7.01	7.06	7.03	7.28	7.04	6.99	7.05	7.54	7.13	7.23	7.33	7.32	7.16	7.29	7.35	7.41	7.12	7.29	7.32	7.37
1370033_at	Myl1	myosin, light chain 1	1	uniqDR+/+_356	0	1	0	0	0	0	0	0	0	0	2.66	2.84	2.96	3.06	2.62	2.71	3.14	3.41	2.43	2.49	2.51	2.80	2.50	2.63	2.57	2.70	2.50	2.48	2.68	2.66
1370047_at	Enpp1	ectonucleotide pyrophosphatase/phosphodiesterase 1	1	uniqDR+/+_356	0	1	0	0	0	0	0	0	0	0	5.54	5.85	5.85	5.78	5.42	5.72	5.78	6.27	4.47	4.61	4.78	4.82	4.38	4.70	4.80	4.79	4.84	4.80	4.74	4.42
1370058_at	Nefl	neurofilament, light polypeptide	1	uniqDR+/+_356	0	1	0	0	0	0	0	0	0	0	3.07	3.23	3.22	3.32	3.15	3.29	3.79	3.50	2.97	4.54	3.14	3.18	2.86	3.38	2.85	3.04	3.23	3.07	2.97	3.48
1370059_at	Nefl	neurofilament, light polypeptide	1	uniqDR+/+_356	0	1	0	0	0	0	0	0	0	0	2.72	2.94	3.22	3.31	2.85	3.06	3.48	3.35	2.54	4.49	2.71	2.86	2.58	3.32	2.82	2.66	2.86	2.74	2.82	3.24
1370127_at	Pold1	polymerase (DNA directed), delta 1, catalytic subunit	1	uniqDR+/+_356	0	1	0	0	0	0	0	0	0	0	5.35	5.41	5.41	5.61	5.22	5.33	5.34	5.76	5.19	5.37	5.24	5.32	5.55	5.41	5.25	5.33	5.32	5.44	5.42	5.28
1370133_at	Rgs19	regulator of G-protein signaling 19	1	uniqDR+/+_356	0	1	0	0	0	0	0	0	0	0	5.15	5.04	4.99	5.25	5.06	4.95	5.30	5.47	5.04	5.07	5.15	5.08	4.95	4.97	5.04	5.11	4.86	5.15	4.86	4.92
1370136_at	Lbr	lamin B receptor	1	uniqDR+/+_356	0	1	0	0	0	0	0	0	0	0	4.80	4.73	4.74	4.70	4.76	4.85	4.83	5.30	4.70	4.70	4.54	4.46	4.63	4.69	4.73	4.54	4.79	4.72	4.58	4.45
1370138_at	Lef1	lymphoid enhancer binding factor 1	1	uniqDR+/+_356	0	1	0	0	0	0	0	0	0	0	2.46	2.70	2.62	2.92	2.61	2.77	3.16	3.90	2.54	2.66	2.56	2.59	2.82	2.54	2.88	2.75	2.61	2.65	2.63	2.74
1370174_at	Ppp1r15a	protein phosphatase 1, regulatory subunit 15A	1	uniqDR+/+_356	0	1	0	0	0	0	0	0	0	0	7.54	7.72	7.65	7.65	7.57	7.56	8.37	7.89	7.67	7.61	7.70	7.39	7.58	7.53	7.52	7.58	7.57	7.54	7.48	7.24
1370228_at	Srprb /// Tf	signal recognition particle receptor, B subunit /// transferrin	1	uniqDR+/+_356	0	1	0	0	0	0	0	0	0	0	6.84	6.79	6.99	7.35	6.84	6.86	7.42	7.89	6.25	6.12	5.98	6.65	6.06	6.13	6.78	6.92	6.35	6.14	6.44	6.09
1370234_at	Fn1	fibronectin 1	1	uniqDR+/+_356	0	1	0	0	0	0	0	0	0	0	6.76	6.69	6.75	6.97	6.76	6.64	6.95	7.29	6.48	6.23	6.32	6.36	6.75	6.53	6.53	6.44	6.59	6.61	6.47	6.46
1370291_at	Pdlim3	PDZ and LIM domain 3	1	uniqDR+/+_356	0	1	0	0	0	0	0	0	0	0	5.22	5.44	5.63	5.50	5.28	5.41	5.67	5.86	5.46	5.28	5.33	5.44	5.44	5.64	5.42	5.25	5.67	5.66	5.11	5.49
1370391_at	Crabp2 /// LC	cellular retinoic acid binding protein 2 /// cellular retinoic acid-binding protein 2-like	1	uniqDR+/+_356	0	1	0	0	0	0	0	0	0	0	4.13	4.00	4.12	4.14	4.12	4.12	4.27	4.79	3.72	3.94	3.98	4.20	4.21	3.87	3.96	4.10	3.96	3.99	3.92	4.07
1370406_a_at	Cd55	Cd55 molecule	1	uniqDR+/+_356	0	1	0	0	0	0	0	0	0	0	3.37	3.35	3.52	3.42	3.27	3.23	3.49	3.86	3.05	3.14	3.10	3.09	3.11	3.04	3.20	3.28	3.11	3.11	2.94	2.99
1370469_at	Ptpn7	protein tyrosine phosphatase, non-receptor type 7	1	uniqDR+/+_356	0	1	0	0	0	0	0	0	0	0	5.19	5.01	5.04	5.56	5.27	5.24	5.34	5.82	5.27	5.27	5.28	5.40	5.31	5.26	5.29	5.39	5.37	5.33	5.33	5.34
1370544_at	Eml2	echinoderm microtubule associated protein like 2	1	uniqDR+/+_356	0	1	0	0	0	0	0	0	0	0	5.15	5.51	5.28	6.05	4.93	5.40	5.41	5.67	5.46	5.70	5.56	5.76	5.04	5.35	5.48	5.41	5.38	5.43	5.86	5.68
1370563_at	Akr1c14	aldo-keto reductase family 1, member C14	1	uniqDR+/+_356	0	1	0	0	0	0	0	0	0	0	3.64	3.80	3.76	4.00	3.74	3.65	4.11	4.12	4.08	3.92	3.81	4.06	4.24	4.03	4.06	4.19	3.99	3.90	4.16	4.15
1370708_a_at	Akr1c14	aldo-keto reductase family 1, member C14	1	uniqDR+/+_356	0	1	0	0	0	0	0	0	0	0	2.68	2.70	2.61	2.83	2.44	2.50	3.16	3.07	2.62	2.70	2.83	2.91	2.89	2.85	2.82	3.08	2.71	2.73	2.75	2.71
1370769_a_at	Icos	inducible T-cell co-stimulator	1	uniqDR+/+_356	0	1	0	0	0	0	0	0	0	0	2.47	2.34	2.29	2.74	2.32	2.46	2.66	3.38	2.34	2.35	2.40	2.52	2.33	2.52	2.56	2.67	2.34	2.58	2.46	2.44
1370826_at	Nap1l1	nucleosome assembly protein 1-like 1	1	uniqDR+/+_356	0	1	0	0	0	0	0	0	0	0	8.45	8.52	8.51	8.70	8.37	8.50	8.45	9.02	8.42	8.52	8.60	8.44	8.18	8.69	8.57	8.54	8.43	8.63	8.56	8.57
1370875_at	Ezr	ezrin	1	uniqDR+/+_356	0	1	0	0	0	0	0	0	0	0	7.64	7.59	7.59	7.99	7.58	7.58	7.69	8.29	7.51	7.45	7.50	7.45	7.75	7.48	7.55	7.52	7.63	7.43	7.61	7.41
1370956_at	Dcn	decorin	1	uniqDR+/+_356	0	1	0	0	0	0	0	0	0	0	7.72	8.13	7.99	7.76	7.53	8.01	7.86	8.26	7.22	7.34	7.14	7.29	7.42	7.44	7.61	7.55	7.46	7.47	7.58	7.49
1370963_at	Gas7	growth arrest specific 7	1	uniqDR+/+_356	0	1	0	0	0	0	0	0	0	0	4.03	3.85	3.69	4.29	3.93	3.89	3.92	4.87	3.42	3.46	3.53	3.58	3.62	3.25	3.62	3.84	3.61	3.37	3.71	3.67
1370990_at	Crlf3	cytokine receptor-like factor 3	1	uniqDR+/+_356	0	1	0	0	0	0	0	0	0	0	7.12	7.10	7.26	7.45	7.08	7.11	7.27	7.89	7.56	7.54	7.54	7.48	7.49	7.49	7.68	7.65	7.48	7.42	7.59	7.57
1371034_at	Onecut1	one cut homeobox 1	1	uniqDR+/+_356	0	1	0	0	0	0	0	0	0	0	3.92	4.12	4.36	4.61	4.24	4.11	4.90	5.11	3.66	3.70	3.22	4.08	4.10	3.63	4.32	4.57	4.02	3.44	3.92	4.07
1371040_at	Slc1a5	solute carrier family 1 (neutral amino acid transporter), member 5	1	uniqDR+/+_356	0	1	0	0	0	0	0	0	0	0	5.92	5.82	5.96	6.20	5.92	5.98	6.21	6.60	6.24	6.15	6.04	6.04	6.26	6.27	6.20	6.06	6.09	6.15	6.11	6.05
1371089_at	Gsta5	glutathione S-transferase Yc2 subunit	1	uniqDR+/+_356	0	1	0	0	0	0	0	0	0	0	2.28	2.39	2.26	2.74	2.27	2.39	2.48	3.23	2.45	2.27	2.29	2.38	2.27	2.31	2.36	2.49	2.23	2.18	2.27	2.55
1371210_s_at	RT1-CE5 /// R	RT1 class I, locus CE5 /// RT1 class Ib, locus EC2	1	uniqDR+/+_356	0	1	0	0	0	0	0	0	0	0	3.13	3.17	3.36	3.48	2.81	2.83	3.18	3.56	3.12	3.24	3.11	3.37	2.90	3.04	2.95	3.15	3.08	3.11	3.20	3.25
1371336_at	Hn1	hematological and neurological expressed 1	1	uniqDR+/+_356	0	1	0	0	0	0	0	0	0	0	7.15	7.15	7.12	7.45	7.08	7.11	7.21	7.87	7.24	7.36	7.21	7.16	7.23	7.27	7.24	7.26	7.31	7.20	7.15	7.06
1371414_at	Gsn	gelsolin	1	uniqDR+/+_356	0	1	0	0	0	0	0	0	0	0	8.41	8.83	8.76	8.69	8.10	8.65	8.74	8.86	7.99	8.20	8.05	8.05	7.97	8.15	8.20	8.43	8.07	8.29	8.35	8.13
1371566_at	Fbxl22	F-box and leucine-rich repeat protein 22	1	uniqDR+/+_356	0	1	0	0	0	0	0	0	0	0	2.50	2.74	2.68	2.55	2.66	2.57	3.12	3.00	2.87	2.70	2.66	2.60	2.73	2.40	2.68	2.41	2.56	2.63	2.73	2.77
1371872_at	---	---	1	uniqDR+/+_356	0	1	0	0	0	0	0	0	0	0	9.75	9.73	9.78	9.84	9.66	9.74	9.76	9.88	9.46	9.43	9.50	9.49	9.56	9.55	9.56	9.47	9.45	9.47	9.56	9.57
1372107_at	Fhl1	four and a half LIM domains 1	1	uniqDR+/+_356	0	1	0	0	0	0	0	0	0	0	6.07	6.59	6.58	6.95	6.19	6.41	6.81	7.62	6.01	6.28	6.27	6.37	6.14	6.25	6.41	6.42	6.10	6.20	6.25	6.39
1372133_at	Rras2	related RAS viral (r-ras) oncogene homolog 2	1	uniqDR+/+_356	0	1	0	0	0	0	0	0	0	0	6.98	7.12	7.18	7.45	7.03	7.22	7.12	7.77	6.68	6.73	6.85	6.74	6.80	6.91	6.90	6.78	6.70	6.79	6.75	6.80
1372135_at	LOC684352	similar to twinfilin-like protein	1	uniqDR+/+_356	0	1	0	0	0	0	0	0	0	0	5.49	5.61	5.65	5.83	5.64	5.52	5.72	6.20	5.46	5.73	5.70	5.65	5.55	5.63	5.63	5.69	5.50	5.64	5.61	5.55
1372161_at	LOC685612	similar to Emu2	1	uniqDR+/+_356	0	1	0	0	0	0	0	0	0	0	4.69	4.63	4.71	4.78	4.34	4.86	4.87	4.78	4.33	4.39	4.62	4.72	4.49	4.31	4.62	4.76	4.61	4.66	4.73	4.66
1372219_at	Tpm2	tropomyosin 2, beta	1	uniqDR+/+_356	0	1	0	0	0	0	0	0	0	0	7.11	7.41	7.34	7.84	7.17	7.11	7.56	8.58	6.39	6.21	6.29	6.71	6.35	6.33	6.65	6.59	6.57	6.39	6.21	6.59
1372246_at	Ostf1	osteoclast stimulating factor 1	1	uniqDR+/+_356	0	1	0	0	0	0	0	0	0	0	8.09	8.05	8.17	8.50	8.02	8.04	8.18	8.80	7.93	8.09	8.20	8.18	8.01	8.17	8.23	8.35	8.08	8.18	8.21	8.27
1372273_at	Gypc	glycophorin C (Gerbich blood group)	1	uniqDR+/+_356	0	1	0	0	0	0	0	0	0	0	5.51	5.30	5.22	5.82	5.50	5.42	5.43	6.29	4.94	4.83	4.68	4.96	5.34	4.93	5.19	5.30	5.10	4.91	4.95	5.20
1372303_at	Fam49b	family with sequence similarity 49, member B	1	uniqDR+/+_356	0	1	0	0	0	0	0	0	0	0	8.35	8.42	8.51	8.76	8.43	8.56	8.41	9.17	8.37	8.47	8.45	8.36	8.50	8.43	8.44	8.44	8.39	8.48	8.46	8.41
1372397_at	Tiprl	TIP41, TOR signaling pathway regulator-like (S. cerevisiae)	1	uniqDR+/+_356	0	1	0	0	0	0	0	0	0	0	7.45	7.59	7.72	7.64	7.13	7.54	7.69	7.65	8.08	8.12	8.11	8.19	8.26	8.13	8.07	8.18	8.05	8.05	8.18	8.14
1372406_at	Mcm3	minichromosome maintenance complex component 3	1	uniqDR+/+_356	0	1	0	0	0	0	0	0	0	0	4.65	4.80	4.55	5.60	4.69	4.78	4.90	6.29	4.52	4.63	4.64	4.63	4.66	4.60	4.53	4.73	4.62	4.54	4.62	4.57
1372408_at	Gga2	golgi associated, gamma adaptin ear containing, ARF binding protein 2	1	uniqDR+/+_356	0	1	0	0	0	0	0	0	0	0	8.55	8.60	8.63	8.90	8.57	8.56	8.65	9.11	8.54	8.50	8.55	8.54	8.68	8.57	8.59	8.64	8.45	8.47	8.57	8.61
1372531_at	Ppfibp2	PTPRF interacting protein, binding protein 2 (liprin beta 2)	1	uniqDR+/+_356	0	1	0	0	0	0	0	0	0	0	3.66	3.72	3.96	3.97	4.02	3.89	4.03	4.43	3.72	3.82	3.75	4.08	3.67	3.71	3.94	4.01	3.80	3.68	3.88	4.07
1372725_at	Plscr2	phospholipid scramblase 2	1	uniqDR+/+_356	0	1	0	0	0	0	0	0	0	0	3.14	3.19	3.36	3.44	3.18	3.00	3.50	3.94	3.62	3.40	3.27	3.70	3.59	3.80	3.62	3.70	3.60	3.59	3.83	4.00
1372770_at	Sri	sorcin	1	uniqDR+/+_356	0	1	0	0	0	0	0	0	0	0	7.79	7.87	7.93	8.01	7.76	7.89	7.86	8.29	7.54	7.65	7.69	7.65	7.14	7.77	7.76	7.64	7.53	7.61	7.54	7.48
1372904_at	Mob3a	MOB kinase activator 3A	1	uniqDR+/+_356	0	1	0	0	0	0	0	0	0	0	5.57	5.59	5.57	6.10	5.53	5.57	5.56	6.32	5.65	5.67	5.71	5.61	5.89	5.51	5.69	5.74	5.61	5.68	5.61	5.49
1373102_at	Cdh13	cadherin 13	1	uniqDR+/+_356	0	1	0	0	0	0	0	0	0	0	5.49	5.76	5.68	5.72	5.37	5.48	5.69	5.74	5.12	5.27	5.45	5.53	5.35	5.44	5.42	5.42	5.30	5.26	5.19	5.33
1373252_at	LOC300225	similar to formin-like 3 protein	1	uniqDR+/+_356	0	1	0	0	0	0	0	0	0	0	6.33	6.35	6.31	6.57	6.34	6.20	6.38	6.89	6.07	6.10	6.08	6.30	6.46	6.25	6.14	6.29	6.20	6.20	6.39	6.22
1373290_at	Ezh2	enhancer of zeste homolog 2 (Drosophila)	1	uniqDR+/+_356	0	1	0	0	0	0	0	0	0	0	4.90	5.04	4.95	5.21	4.94	5.08	5.01	5.81	5.10	5.30	5.08	5.04	5.22	4.87	5.01	5.07	5.11	4.93	4.99	5.05
1373473_a_at	Nap1l1	nucleosome assembly protein 1-like 1	1	uniqDR+/+_356	0	1	0	0	0	0	0	0	0	0	7.78	7.96	8.00	8.04	7.68	7.89	7.91	8.36	8.10	8.18	8.13	7.93	7.86	8.29	8.17	7.99	7.99	8.10	8.01	7.92
1373485_at	---	---	1	uniqDR+/+_356	0	1	0	0	0	0	0	0	0	0	4.41	4.27	4.29	4.52	4.32	4.47	4.36	4.83	4.08	4.07	4.18	4.19	4.22	4.03	4.34	4.45	4.38	4.18	4.16	4.08
1373528_at	Pik3cd	phosphatidylinositol-4,5-bisphosphate 3-kinase, catalytic subunit delta	1	uniqDR+/+_356	0	1	0	0	0	0	0	0	0	0	5.83	5.69	5.64	6.49	5.79	5.93	5.99	6.83	5.94	5.84	5.89	5.93	5.75	5.83	5.77	6.00	5.80	5.95	5.95	5.90
1373530_at	Ccne1	cyclin E1	1	uniqDR+/+_356	0	1	0	0	0	0	0	0	0	0	4.59	4.86	4.92	4.97	4.75	4.81	4.98	5.36	4.62	4.86	4.96	4.96	4.70	4.82	5.13	5.09	4.63	4.82	5.04	5.10
1373557_at	Mcm4	minichromosome maintenance complex component 4	1	uniqDR+/+_356	0	1	0	0	0	0	0	0	0	0	7.32	7.37	7.37	7.48	7.13	7.48	7.40	7.83	7.19	7.37	7.46	7.34	7.32	7.35	7.36	7.26	7.19	7.40	7.44	7.49
1373666_at	Rapgef5	Rap guanine nucleotide exchange factor (GEF) 5	1	uniqDR+/+_356	0	1	0	0	0	0	0	0	0	0	5.95	5.95	6.01	6.00	5.84	5.91	6.15	6.36	5.67	5.72	5.66	5.78	5.83	5.61	5.78	5.82	5.71	5.60	5.73	5.98
1373903_at	Rcsd1	RCSD domain containing 1	1	uniqDR+/+_356	0	1	0	0	0	0	0	0	0	0	5.76	5.75	5.68	6.09	5.77	5.84	5.95	6.98	5.59	5.70	5.68	5.93	6.02	5.86	5.83	5.96	5.90	5.69	6.03	5.96
1374055_at	Erf	Ets2 repressor factor	1	uniqDR+/+_356	0	1	0	0	0	0	0	0	0	0	7.12	7.11	6.98	7.42	6.82	6.85	7.25	7.33	7.09	7.13	6.98	7.09	7.02	7.21	6.88	7.15	7.00	7.21	7.20	6.96
1374101_at	Tbc1d10a	TBC1 domain family, member 10a	1	uniqDR+/+_356	0	1	0	0	0	0	0	0	0	0	5.50	5.51	5.48	5.91	5.45	5.25	5.68	5.90	5.79	5.81	5.61	5.95	5.61	5.75	5.53	5.79	5.79	5.42	5.80	5.83
1374113_at	Baz1a	bromodomain adjacent to zinc finger domain, 1A	1	uniqDR+/+_356	0	1	0	0	0	0	0	0	0	0	5.84	5.80	5.81	6.29	5.89	5.73	6.05	6.77	6.02	5.85	6.00	6.02	5.84	5.90	6.08	6.11	5.96	5.95	5.96	6.12
1374237_at	Lmod1	leiomodin 1 (smooth muscle)	1	uniqDR+/+_356	0	1	0	0	0	0	0	0	0	0	3.93	3.98	4.25	4.22	3.78	3.92	4.02	4.40	3.72	3.96	3.93	3.93	3.65	3.98	3.91	3.83	3.73	3.96	3.94	3.93
1374293_at	H2afj /// LOC	H2A histone family, member J /// histone H2A.J-like	1	uniqDR+/+_356	0	1	0	0	0	0	0	0	0	0	7.37	7.50	7.69	7.76	7.48	7.40	7.55	7.92	7.64	7.69	7.82	7.89	7.60	7.68	7.79	7.95	7.66	7.67	7.77	7.82
1374540_at	Cdca7	cell division cycle associated 7	1	uniqDR+/+_356	0	1	0	0	0	0	0	0	0	0	4.94	4.81	4.67	5.53	4.85	4.90	4.89	6.62	4.54	4.65	4.52	4.78	4.70	4.56	4.63	4.78	4.60	4.60	4.57	4.74
1374649_at	Rasgrp2	RAS guanyl releasing protein 2 (calcium and DAG-regulated)	1	uniqDR+/+_356	0	1	0	0	0	0	0	0	0	0	4.83	4.59	4.70	5.14	4.78	4.77	4.81	5.98	5.00	4.68	4.76	4.72	4.97	4.68	4.80	4.90	4.95	4.84	4.74	4.65
1374705_at	Col4a5	collagen, type IV, alpha 5	1	uniqDR+/+_356	0	1	0	0	0	0	0	0	0	0	6.67	6.81	6.89	7.04	6.58	6.81	7.07	7.46	6.28	6.43	6.45	6.63	6.54	6.54	6.70	6.57	6.45	6.46	6.57	6.66
1374735_at	Arhgap4	Rho GTPase activating protein 4	1	uniqDR+/+_356	0	1	0	0	0	0	0	0	0	0	4.85	4.98	4.95	5.34	4.66	4.91	4.96	5.57	4.80	4.89	4.97	5.15	4.83	4.86	4.96	5.20	4.72	4.89	5.02	5.05
1374816_at	Gcom1	GRINL1A complex locus 1	1	uniqDR+/+_356	0	1	0	0	0	0	0	0	0	0	5.64	5.53	5.72	6.01	5.55	5.54	5.85	6.72	5.83	5.63	5.71	5.81	5.87	5.93	5.93	6.13	5.82	5.67	5.62	6.02
1374830_at	Ccdc3	coiled-coil domain containing 3	1	uniqDR+/+_356	0	1	0	0	0	0	0	0	0	0	4.56	4.86	4.63	4.80	4.61	4.86	4.95	5.20	4.33	4.25	4.37	4.29	4.48	4.48	4.56	4.35	4.50	4.49	4.37	4.42
1374872_at	Rasgrp2	RAS guanyl releasing protein 2 (calcium and DAG-regulated)	1	uniqDR+/+_356	0	1	0	0	0	0	0	0	0	0	3.76	3.64	3.56	4.28	3.76	3.50	3.98	4.44	3.85	3.62	3.60	3.79	3.69	3.60	3.67	3.95	3.78	3.64	3.61	3.58
1375015_at	LOC688280	similar to transmembrane channel-like protein 6 isoform 1	1	uniqDR+/+_356	0	1	0	0	0	0	0	0	0	0	4.42	4.33	4.16	4.55	4.08	4.26	4.36	4.67	3.98	4.24	4.12	4.25	4.27	4.12	4.25	4.12	4.23	4.22	4.21	4.15
1375170_at	S100a11	S100 calcium binding protein A11	1	uniqDR+/+_356	0	1	0	0	0	0	0	0	0	0	8.03	8.20	8.18	8.48	7.98	8.15	8.57	8.87	7.59	7.69	7.47	7.64	7.58	7.62	7.85	7.86	7.61	7.44	7.71	7.44
1375686_at	Ppil3	peptidylprolyl isomerase (cyclophilin)-like 3	1	uniqDR+/+_356	0	1	0	0	0	0	0	0	0	0	6.49	6.71	6.67	6.98	6.64	6.53	6.62	7.22	6.54	6.60	6.70	6.88	6.57	6.57	6.70	6.96	6.54	6.63	6.65	7.00
1375709_at	Sipa1	signal-induced proliferation-associated 1	1	uniqDR+/+_356	0	1	0	0	0	0	0	0	0	0	6.28	6.26	6.18	6.59	6.34	6.26	6.29	7.11	6.31	6.31	6.27	6.28	6.33	6.28	6.12	6.43	6.27	6.25	6.40	6.37
1375719_s_at	Cdh13	cadherin 13	1	uniqDR+/+_356	0	1	0	0	0	0	0	0	0	0	5.17	5.37	5.30	5.37	5.10	5.19	5.47	5.68	5.01	5.05	5.09	5.17	5.07	5.16	5.05	4.99	5.06	4.95	5.06	4.88
1375784_at	Egr4	early growth response 4	1	uniqDR+/+_356	0	1	0	0	0	0	0	0	0	0	4.71	4.73	4.64	4.53	4.65	4.71	4.73	4.52	4.52	4.59	4.50	4.76	4.60	4.70	4.62	4.63	4.60	4.66	4.53	4.78
1375908_at	Mpzl2	myelin protein zero-like 2	1	uniqDR+/+_356	0	1	0	0	0	0	0	0	0	0	4.06	4.33	4.31	4.43	4.13	4.02	4.83	4.50	3.99	3.85	3.94	4.26	4.07	4.08	4.35	4.68	3.97	4.08	4.14	4.13
1375983_at	Mamdc2	MAM domain containing 2	1	uniqDR+/+_356	0	1	0	0	0	0	0	0	0	0	4.03	4.14	4.15	4.16	3.85	3.94	4.13	4.47	3.77	4.05	4.01	4.12	3.99	4.11	4.11	4.08	4.01	4.09	4.00	4.29
1375996_at	Sept1	septin 1	1	uniqDR+/+_356	0	1	0	0	0	0	0	0	0	0	5.88	6.04	6.00	6.27	5.68	5.87	6.09	6.86	5.81	5.97	6.03	5.92	5.86	5.97	6.00	6.03	5.83	5.82	6.00	5.87
1376102_at	Tmbim1	transmembrane BAX inhibitor motif containing 1	1	uniqDR+/+_356	0	1	0	0	0	0	0	0	0	0	5.74	5.68	5.71	6.15	5.76	5.70	5.77	6.36	5.62	5.62	5.45	5.46	5.95	5.59	5.64	5.65	5.64	5.55	5.65	5.45
1376259_at	Prkcq	protein kinase C, theta	1	uniqDR+/+_356	0	1	0	0	0	0	0	0	0	0	3.20	3.34	3.24	3.58	3.23	3.37	3.59	4.00	3.12	3.21	3.30	3.48	3.22	3.31	3.33	3.31	2.97	3.35	3.41	3.34
1376311_at	Ntng1	netrin G1	1	uniqDR+/+_356	0	1	0	0	0	0	0	0	0	0	2.24	2.24	2.34	2.55	2.27	2.16	2.22	2.68	2.26	2.35	2.22	2.29	2.12	2.06	2.31	2.33	2.37	2.00	2.26	2.27
1376319_at	Sema3c	sema domain, immunoglobulin domain (Ig), short basic domain, secreted, (semaphorin) 3C	1	uniqDR+/+_356	0	1	0	0	0	0	0	0	0	0	5.48	5.38	5.67	5.55	5.54	5.39	5.66	6.01	5.52	5.43	5.49	5.57	5.54	5.60	5.54	5.60	5.51	5.49	5.80	5.80
1376667_at	Cyp26b1	cytochrome P450, family 26, subfamily b, polypeptide 1	1	uniqDR+/+_356	0	1	0	0	0	0	0	0	0	0	4.50	4.42	4.37	4.42	4.40	4.49	4.58	4.59	4.36	4.46	4.57	4.60	4.41	4.58	4.50	4.43	4.33	4.41	4.48	4.54
1376706_at	Tmem47	transmembrane protein 47	1	uniqDR+/+_356	0	1	0	0	0	0	0	0	0	0	7.00	7.31	7.26	7.38	7.02	7.11	7.29	7.51	6.97	7.10	7.15	7.11	7.02	7.31	7.16	7.20	7.00	7.22	7.17	7.30
1376775_at	Prr5l	proline rich 5 like	1	uniqDR+/+_356	0	1	0	0	0	0	0	0	0	0	3.55	3.52	3.70	4.02	3.31	3.50	3.71	3.84	3.39	3.38	3.47	3.52	3.34	3.65	3.64	3.88	3.34	3.56	3.60	3.68
1376937_at	Filip1l	filamin A interacting protein 1-like	1	uniqDR+/+_356	0	1	0	0	0	0	0	0	0	0	5.75	5.90	6.00	5.99	5.74	5.82	6.07	6.50	5.22	5.33	5.41	5.65	5.61	5.47	5.74	5.53	5.43	5.40	5.66	5.64
1377018_at	---	---	1	uniqDR+/+_356	0	1	0	0	0	0	0	0	0	0	5.54	5.65	5.78	6.02	5.50	5.58	6.44	6.51	5.66	5.60	5.46	5.64	5.36	5.67	5.94	6.06	5.47	5.41	5.70	5.41
1377169_at	LOC684173	similar to TNF receptor-associated factor 5	1	uniqDR+/+_356	0	1	0	0	0	0	0	0	0	0	5.98	5.92	5.99	6.04	5.86	6.02	5.91	6.43	5.53	5.59	5.69	5.85	5.79	5.61	5.69	5.80	5.74	5.65	5.63	5.80
1377286_at	Gimap4	GTPase, IMAP family member 4	1	uniqDR+/+_356	0	1	0	0	0	0	0	0	0	0	5.60	5.32	5.12	5.84	5.54	5.09	5.52	6.71	5.33	5.01	5.05	5.01	5.35	5.15	5.16	5.23	5.28	4.98	5.02	4.85
1377494_at	Fli1	Friend leukemia virus integration 1	1	uniqDR+/+_356	0	1	0	0	0	0	0	0	0	0	6.53	6.42	6.43	6.40	6.50	6.43	6.51	6.45	6.35	6.35	6.58	6.53	6.46	6.47	6.43	6.41	6.42	6.52	6.51	6.51
1377631_at	Col9a3	collagen, type IX, alpha 3	1	uniqDR+/+_356	0	1	0	0	0	0	0	0	0	0	3.95	4.00	3.83	4.47	4.08	3.88	4.16	4.93	4.13	3.98	3.93	4.05	4.18	3.89	3.92	4.15	4.06	3.80	3.96	4.10
1377675_at	LOC1003023	hypothetical protein LOC100302372	1	uniqDR+/+_356	0	1	0	0	0	0	0	0	0	0	5.79	5.77	5.65	6.15	5.73	5.67	5.84	6.98	5.68	5.58	5.74	5.69	5.91	5.80	5.76	5.84	5.68	5.81	5.73	5.77
1377718_at	Fancb	Fanconi anemia, complementation group B	1	uniqDR+/+_356	0	1	0	0	0	0	0	0	0	0	4.12	4.34	4.24	4.51	4.16	4.12	4.11	4.63	4.19	4.28	4.31	4.23	4.22	3.85	4.15	4.15	3.99	3.99	4.25	4.01
1377727_at	Baz1a	bromodomain adjacent to zinc finger domain, 1A	1	uniqDR+/+_356	0	1	0	0	0	0	0	0	0	0	5.26	5.26	5.35	5.75	5.42	4.69	5.23	6.31	5.58	5.42	5.29	5.36	5.11	5.04	5.53	5.43	5.55	5.14	5.13	5.17
1377735_at	Plekha3	pleckstrin homology domain-containing, family A (phosphoinositide binding specific) membe	1	uniqDR+/+_356	0	1	0	0	0	0	0	0	0	0	8.03	8.03	8.11	8.28	7.98	8.09	8.14	8.46	7.79	7.93	7.99	7.92	8.07	7.97	7.88	8.06	7.83	7.87	7.94	8.02
1377810_at	---	---	1	uniqDR+/+_356	0	1	0	0	0	0	0	0	0	0	4.69	4.77	4.87	5.16	4.71	4.72	4.84	5.61	4.75	4.55	4.58	4.59	4.55	4.59	4.60	4.63	4.55	4.45	4.55	4.54
1378028_at	Mad2l1	MAD2 mitotic arrest deficient-like 1 (yeast)	1	uniqDR+/+_356	0	1	0	0	0	0	0	0	0	0	4.11	4.47	4.30	4.49	4.31	4.54	4.44	5.03	4.33	4.58	4.60	4.53	4.14	4.28	4.71	4.43	4.33	4.67	4.64	4.41
1378032_at	Nfkbiz	nuclear factor of kappa light polypeptide gene enhancer in B-cells inhibitor, zeta	1	uniqDR+/+_356	0	1	0	0	0	0	0	0	0	0	7.65	8.24	7.96	7.96	8.23	8.19	9.45	8.99	7.99	8.26	8.41	7.54	8.18	7.65	8.01	7.87	7.91	8.08	8.12	7.24
1378057_at	Flrt3	fibronectin leucine rich transmembrane protein 3	1	uniqDR+/+_356	0	1	0	0	0	0	0	0	0	0	4.65	4.78	4.91	4.98	4.60	4.75	5.25	5.36	4.76	4.68	4.71	4.62	4.97	4.76	4.99	5.04	4.85	4.61	4.80	4.73
1378156_at	Cys1	cystin 1	1	uniqDR+/+_356	0	1	0	0	0	0	0	0	0	0	5.83	6.10	6.12	5.99	5.64	5.88	6.13	6.15	6.30	6.66	6.76	6.43	6.32	6.46	6.49	6.54	6.44	6.16	6.40	6.73
1378185_at	Nxt2	nuclear transport factor 2-like export factor 2	1	uniqDR+/+_356	0	1	0	0	0	0	0	0	0	0	6.05	6.32	6.31	6.50	6.02	6.25	6.18	6.65	5.99	6.16	6.27	6.40	6.20	6.16	6.43	6.51	5.94	6.16	6.30	6.37
1378247_at	Eaf2	ELL associated factor 2	1	uniqDR+/+_356	0	1	0	0	0	0	0	0	0	0	2.39	2.19	2.29	3.01	2.22	2.22	2.21	4.69	2.31	2.35	2.51	2.29	2.40	2.23	2.21	2.39	2.49	2.15	2.50	2.39
1378377_at	Dapp1	dual adaptor of phosphotyrosine and 3-phosphoinositides	1	uniqDR+/+_356	0	1	0	0	0	0	0	0	0	0	4.00	3.82	3.75	4.18	4.00	3.88	3.90	4.08	3.86	3.78	3.79	3.76	3.61	3.76	3.67	3.72	3.79	3.71	3.75	3.74
1378520_at	Bcl11b	B-cell CLL/lymphoma 11B (zinc finger protein)	1	uniqDR+/+_356	0	1	0	0	0	0	0	0	0	0	3.29	3.37	3.35	3.37	3.22	3.21	3.37	3.63	3.19	3.34	3.43	3.33	3.37	3.54	3.32	3.45	3.26	3.53	3.34	3.39
1378526_at	Gimap6	GTPase, IMAP family member 6	1	uniqDR+/+_356	0	1	0	0	0	0	0	0	0	0	6.81	6.71	6.71	6.84	6.69	6.60	6.68	7.23	6.60	6.43	6.43	6.60	6.71	6.59	6.54	6.60	6.52	6.35	6.46	6.49
1378699_at	Pkhd1l1	polycystic kidney and hepatic disease 1-like 1	1	uniqDR+/+_356	0	1	0	0	0	0	0	0	0	0	3.64	3.85	3.77	3.84	3.70	3.76	4.18	4.44	3.65	3.89	3.86	4.09	4.03	3.86	4.04	4.19	3.86	3.87	3.85	4.00
1379262_at	Acot9	acyl-CoA thioesterase 9	1	uniqDR+/+_356	0	1	0	0	0	0	0	0	0	0	6.97	7.16	7.26	7.43	6.96	7.13	7.31	7.55	6.99	7.21	7.40	7.41	7.11	7.34	7.42	7.39	7.01	7.23	7.36	7.41
1379275_at	Snx10	sorting nexin 10	1	uniqDR+/+_356	0	1	0	0	0	0	0	0	0	0	5.41	5.72	5.70	5.87	5.57	5.60	5.75	6.15	5.13	5.14	5.34	5.40	4.69	5.27	5.33	5.13	5.26	5.39	5.27	5.46
1379368_at	Bcl6	B-cell CLL/lymphoma 6	1	uniqDR+/+_356	0	1	0	0	0	0	0	0	0	0	5.98	5.92	5.67	6.80	5.61	5.76	6.09	7.26	5.61	5.82	5.59	5.60	5.95	6.09	5.66	5.74	5.49	5.50	5.46	5.61
1379677_at	Tnfsf13	tumor necrosis factor (ligand) superfamily, member 13	1	uniqDR+/+_356	0	1	0	0	0	0	0	0	0	0	4.15	4.17	4.28	4.66	4.06	4.07	4.29	4.65	3.99	3.98	4.12	4.20	3.99	4.06	4.01	4.44	3.98	3.95	4.18	4.02
1379740_at	---	---	1	uniqDR+/+_356	0	1	0	0	0	0	0	0	0	0	3.26	3.42	3.25	3.48	3.23	3.24	3.18	3.86	2.70	3.04	2.93	2.85	2.48	2.84	2.94	2.81	2.78	2.89	2.98	3.08

Affymetrix Probeset ID	Gene Symbol	Gene Name	unique BB on P7 n=822	Figure 1B P7 Venn n=1526	DRlyp/lyp STEM P7 n=712	DR+/+ STEM P7 n=936	Flyp/lyp STEM P7 n=520	F+/+ STEM P7 n=465	Figure 1B P0 Venn n=1071	DRlyp/lyp STEM P0 n=442	DR+/+ STEM P0 n=437	Flyp/lyp STEM P0 n=643	F+/+ STEM P0 n=632	Fig1D n=25	Mean log2 intensity DRlyp/lyp 20	Mean log2 intensity DRlyp/lyp 30	Mean log2 intensity DRlyp/lyp 40	Mean log2 intensity DRlyp/lyp 50	Mean log2 intensity DR+/+ 20	Mean log2 intensity DR+/+ 30	Mean log2 intensity DR+/+ 40	Mean log2 intensity DR+/+ 50	Mean log2 intensity Flyp/lyp 20	Mean log2 intensity Flyp/lyp 30	Mean log2 intensity Flyp/lyp 40	Mean log2 intensity Flyp/lyp 50	Mean log2 intensity Flyp20	Mean log2 intensity Flyp30	Mean log2 intensity Flyp40	Mean log2 intensity Flyp50	Mean log2 intensity F+/+ 20	Mean log2 intensity F+/+ 30	Mean log2 intensity F+/+ 40	Mean log2 intensity F+/+ 50
1379780_at	Fam65b	family with sequence similarity 65, member B	1	uniqDR+/+_356	0	1	0	0	0	0	0	0	0	0	3.50	3.44	3.52	3.72	3.44	3.52	3.63	4.48	3.52	3.55	3.63	3.49	3.58	3.51	3.50	3.66	3.51	3.55	3.52	3.55
1379932_at	Clcn4	chloride channel, voltage-sensitive 4	1	uniqDR+/+_356	0	1	0	0	0	0	0	0	0	0	4.56	4.47	4.67	4.96	4.26	4.51	4.40	5.34	4.84	5.36	5.21	5.22	5.10	5.09	5.12	5.29	4.84	5.28	5.27	5.21
1380210_at	Gimap1	GTPase, IMAP family member 1	1	uniqDR+/+_356	0	1	0	0	0	0	0	0	0	0	4.83	4.73	4.66	5.19	4.92	4.58	4.74	5.52	4.76	4.48	4.38	4.34	4.82	4.46	4.60	4.49	4.53	4.34	4.36	4.47
1380293_at	LOC361346	similar to chromosome 18 open reading frame 54	1	uniqDR+/+_356	0	1	0	0	0	0	0	0	0	0	4.91	5.02	4.84	4.96	4.81	4.95	4.81	5.29	4.18	4.28	4.28	4.43	3.88	4.25	4.59	4.36	4.39	4.40	4.48	4.83
1380467_at	Slc16a1	solute carrier family 16, member 1 (monocarboxylic acid transporter 1)	1	uniqDR+/+_356	0	1	0	0	0	0	0	0	0	0	3.28	3.57	3.54	3.54	3.41	3.57	3.64	3.59	3.70	3.61	3.55	3.77	3.29	3.59	3.69	3.60	3.39	3.65	3.46	3.62
1380568_a_at	Ddb2	damage specific DNA binding protein 2	1	uniqDR+/+_356	0	1	0	0	0	0	0	0	0	0	4.36	4.52	4.47	4.48	4.31	4.26	4.76	4.62	4.30	4.61	4.42	4.69	4.65	4.41	4.45	4.74	4.37	4.40	4.66	4.62
1380617_at	Gimap5	GTPase, IMAP family member 5	1	uniqDR+/+_356	0	1	0	0	0	0	0	0	0	0	5.70	5.74	5.58	5.96	6.43	6.15	6.43	7.44	5.39	5.16	5.02	5.18	5.66	5.12	5.44	5.36	6.18	5.88	5.97	5.88
1380798_at	Nfkbiz	nuclear factor of kappa light polypeptide gene enhancer in B-cells inhibitor, zeta	1	uniqDR+/+_356	0	1	0	0	0	0	0	0	0	0	5.01	4.73	4.98	5.19	5.09	5.04	4.88	5.02	5.05	5.08	5.06	5.13	4.95	5.04	5.01	5.23	5.12	5.15	4.98	5.08
1380828_at	Gabra1	gamma-aminobutyric acid (GABA) A receptor, alpha 1	1	uniqDR+/+_356	0	1	0	0	0	0	0	0	0	0	2.85	3.05	3.10	3.11	2.96	2.92	3.16	3.49	2.78	2.97	2.88	2.76	2.90	2.67	2.83	2.60	2.93	2.73	2.96	2.66
1381012_at	Serpinf1	serpin peptidase inhibitor, clade F (alpha-2 antiplasmin, pigment epithelium derived factor),	1	uniqDR+/+_356	0	1	0	0	0	0	0	0	0	0	5.41	5.40	5.50	5.39	5.42	5.54	5.49	5.51	5.36	5.43	5.34	5.40	5.33	5.33	5.35	5.39	5.43	5.34	5.40	5.45
1381221_at	RGD1311300	similar to T cell receptor V delta 6	1	uniqDR+/+_356	0	1	0	0	0	0	0	0	0	0	2.84	2.97	2.88	2.99	2.77	2.98	3.03	3.28	2.86	2.72	2.67	2.95	2.85	2.96	3.00	3.05	2.85	2.77	2.83	2.80
1381570_at	Nfkbid	nuclear factor of kappa light polypeptide gene enhancer in B-cells inhibitor, delta	1	uniqDR+/+_356	0	1	0	0	0	0	0	0	0	0	5.99	6.01	6.03	6.37	6.11	6.15	6.40	6.62	5.96	5.98	6.05	6.05	5.89	5.86	5.89	5.94	5.98	5.88	6.04	6.12
1382058_at	Rras2	related RAS viral (r-ras) oncogene homolog 2	1	uniqDR+/+_356	0	1	0	0	0	0	0	0	0	0	6.14	6.27	6.16	6.49	6.01	6.24	6.23	6.92	6.18	6.05	6.05	5.96	6.18	6.26	6.21	6.09	6.01	6.15	6.09	6.04
1382106_at	Ccl6	chemokine (C-C motif) ligand 6	1	uniqDR+/+_356	0	1	0	0	0	0	0	0	0	0	2.58	2.63	2.68	2.59	2.56	2.63	2.73	2.76	2.66	2.76	2.59	2.69	2.63	2.56	2.59	2.65	2.67	2.68	2.68	2.62
1382167_at	Sit1	signaling threshold regulating transmembrane adaptor 1	1	uniqDR+/+_356	0	1	0	0	0	0	0	0	0	0	3.17	2.99	2.85	3.96	3.03	3.24	3.46	5.18	2.99	3.01	3.02	3.10	3.08	3.10	3.04	3.50	2.98	3.19	3.14	3.24
1382181_at	Gsap	gamma-secretase activating protein	1	uniqDR+/+_356	0	1	0	0	0	0	0	0	0	0	5.60	5.40	5.57	5.94	5.49	5.62	5.51	6.00	5.02	5.19	5.09	5.16	5.28	5.18	5.19	5.12	5.29	5.27	5.19	5.11
1382215_at	RGD1562997	similar to Transcription initiation factor TFIID 105 kDa subunit (TAFII-105)	1	uniqDR+/+_356	0	1	0	0	0	0	0	0	0	0	4.29	4.47	4.34	4.39	3.96	4.22	4.32	4.51	4.51	4.56	4.32	4.46	4.79	4.72	4.69	4.62	4.39	4.45	4.54	4.76
1382328_at	Gimap1	GTPase, IMAP family member 1	1	uniqDR+/+_356	0	1	0	0	0	0	0	0	0	0	2.87	2.99	3.04	3.38	2.95	2.79	3.25	3.93	2.83	2.91	3.00	2.96	2.87	2.91	2.97	3.33	2.90	2.84	2.96	2.92
1382378_at	Ctsw	cathepsin W	1	uniqDR+/+_356	0	1	0	0	0	0	0	0	0	0	3.39	3.35	3.35	3.58	3.28	3.50	3.57	3.78	3.24	3.41	3.39	3.42	3.21	3.34	3.32	3.58	3.30	3.49	3.43	3.51
1382439_at	Itgb6	integrin, beta 6	1	uniqDR+/+_356	0	1	0	0	0	0	0	0	0	0	5.61	5.70	5.84	5.77	5.45	5.72	6.11	5.81	5.32	5.40	5.48	5.71	5.54	5.30	5.84	5.69	5.47	5.38	5.78	5.74
1382590_at	Rftn1	raftlin lipid raft linker 1	1	uniqDR+/+_356	0	1	0	0	0	0	0	0	0	0	6.37	6.22	6.11	6.74	6.22	6.20	6.10	7.02	6.30	6.21	6.18	6.23	6.41	6.55	6.30	6.36	6.35	6.43	6.34	6.38
1382955_at	Gpr126	G protein-coupled receptor 126	1	uniqDR+/+_356	0	1	0	0	0	0	0	0	0	0	2.83	2.91	2.86	3.26	2.72	2.78	3.28	3.10	2.68	2.88	2.82	2.88	2.93	2.81	2.97	3.02	2.85	2.69	2.86	2.91
1383008_at	Smc4	structural maintenance of chromosomes 4	1	uniqDR+/+_356	0	1	0	0	0	0	0	0	0	0	5.35	5.32	5.33	5.54	5.37	5.23	5.32	5.88	5.57	5.55	5.62	5.53	5.30	5.47	5.44	5.54	5.56	5.45	5.47	5.59
1383085_at	Sh3bgrl	SH3 domain binding glutamic acid-rich protein like	1	uniqDR+/+_356	0	1	0	0	0	0	0	0	0	0	8.42	8.61	8.62	8.84	8.40	8.36	8.49	8.95	8.57	8.47	8.64	8.69	7.69	8.50	8.64	8.82	8.48	8.52	8.50	8.73
1383145_at	Lamp3	lysosomal-associated membrane protein 3	1	uniqDR+/+_356	0	1	0	0	0	0	0	0	0	0	1.97	2.00	1.95	2.35	2.05	1.90	2.04	2.81	2.00	2.03	2.04	2.01	1.89	1.95	1.96	2.15	2.07	1.91	2.19	2.13
1383160_at	Chordc1	cysteine and histidine-rich domain (CHORD)-containing 1	1	uniqDR+/+_356	0	1	0	0	0	0	0	0	0	0	8.17	8.05	8.12	8.44	8.10	7.97	8.28	8.50	8.18	8.03	8.08	8.21	7.89	7.98	8.16	8.22	8.05	7.95	7.93	8.35
1383322_at	Rasl11b	RAS-like family 11 member B	1	uniqDR+/+_356	0	1	0	0	0	0	0	0	0	0	4.26	4.24	4.34	4.27	4.14	4.31	4.82	4.68	3.74	3.87	3.70	3.79	3.96	3.74	3.74	3.65	3.93	3.76	3.83	3.79
1383437_at	Rab17	RAB17, member RAS oncogene family	1	uniqDR+/+_356	0	1	0	0	0	0	0	0	0	0	3.54	3.58	3.48	3.64	3.42	3.33	3.97	3.90	3.51	3.33	3.27	3.72	3.46	3.39	3.57	3.63	3.24	3.20	3.40	3.40
1383630_at	Dok3	docking protein 3	1	uniqDR+/+_356	0	1	0	0	0	0	0	0	0	0	4.85	4.93	5.05	5.25	4.90	5.05	5.02	5.62	4.85	5.05	5.13	5.23	4.94	5.14	5.02	5.22	4.86	5.13	5.00	5.11
1383685_at	Heatr1 /// LO	HEAT repeat containing 1 /// HEAT repeat-containing protein 1-like	1	uniqDR+/+_356	0	1	0	0	0	0	0	0	0	0	6.25	6.14	6.23	6.34	5.95	5.91	6.11	6.44	6.14	6.19	6.29	6.36	6.26	6.17	6.15	6.35	6.19	6.05	6.31	6.41
1383785_at	---	---	1	uniqDR+/+_356	0	1	0	0	0	0	0	0	0	0	4.80	4.73	4.54	4.98	4.93	4.87	4.83	5.63	5.08	5.09	4.96	5.10	5.13	5.12	5.10	5.40	5.11	5.15	5.23	5.05
1383893_at	Atp6v1g3	ATPase, H+ transporting, lysosomal V1 subunit G3	1	uniqDR+/+_356	0	1	0	0	0	0	0	0	0	0	2.27	2.39	2.40	2.82	2.42	2.39	2.64	3.20	2.39	2.44	2.42	2.51	2.45	2.32	2.34	2.51	2.36	2.33	2.46	2.48
1383926_at	Bub1b	BUB1 mitotic checkpoint serine/threonine kinase B	1	uniqDR+/+_356	0	1	0	0	0	0	0	0	0	0	3.50	3.75	3.62	3.84	3.49	3.88	3.68	4.41	3.59	3.91	3.94	3.81	3.61	3.78	3.89	3.69	3.73	3.97	3.85	3.99
1384035_at	---	---	1	uniqDR+/+_356	0	1	0	0	0	0	0	0	0	0	2.79	2.74	2.99	3.16	2.71	2.81	4.21	3.59	2.85	2.88	2.82	2.98	3.03	2.81	3.18	3.28	2.90	2.51	3.12	2.60
1384036_s_at	---	---	1	uniqDR+/+_356	0	1	0	0	0	0	0	0	0	0	2.60	2.66	2.70	2.71	2.57	2.63	2.80	2.72	2.57	2.58	2.59	2.72	2.60	2.62	2.97	2.73	2.63	2.58	2.54	2.71
1384111_at	Stat4	signal transducer and activator of transcription 4	1	uniqDR+/+_356	0	1	0	0	0	0	0	0	0	0	2.91	2.86	3.03	3.30	3.02	3.02	3.21	3.92	2.94	3.05	3.02	3.31	2.87	2.99	2.94	3.38	3.00	3.07	3.24	3.20
1384192_at	Chst1	carbohydrate (keratan sulfate Gal-6) sulfotransferase 1	1	uniqDR+/+_356	0	1	0	0	0	0	0	0	0	0	4.87	4.72	4.65	5.23	4.78	4.63	4.76	5.63	4.77	4.85	4.82	4.82	5.03	4.69	4.66	4.81	4.80	4.69	4.87	4.86
1384392_at	Cyp26b1	cytochrome P450, family 26, subfamily b, polypeptide 1	1	uniqDR+/+_356	0	1	0	0	0	0	0	0	0	0	3.43	3.89	3.70	4.19	4.07	3.92	4.15	5.07	3.53	3.87	3.72	3.44	3.92	3.86	3.14	3.44	3.48	4.12	3.62	3.46
1384411_at	Glcci1	glucocorticoid induced transcript 1	1	uniqDR+/+_356	0	1	0	0	0	0	0	0	0	0	6.76	6.94	7.08	7.14	6.58	6.82	6.94	7.10	6.89	7.13	7.16	7.37	6.82	7.04	7.17	7.25	6.93	7.12	7.21	7.35
1384456_at	Usp24	ubiquitin specific peptidase 24	1	uniqDR+/+_356	0	1	0	0	0	0	0	0	0	0	5.74	5.91	5.88	6.01	5.70	5.72	5.93	6.29	5.88	5.80	5.95	5.95	5.60	5.93	5.86	5.98	5.90	5.92	5.93	6.01
1384493_at	Gsto2 /// LOC	glutathione S-transferase omega 2 /// glutathione S-transferase omega-2-like	1	uniqDR+/+_356	0	1	0	0	0	0	0	0	0	0	4.95	5.13	5.09	5.05	4.70	5.09	5.21	5.25	4.96	5.20	5.31	5.37	5.16	5.14	5.28	5.41	4.93	5.20	5.35	5.31
1384507_at	Fam105a	family with sequence similarity 105, member A	1	uniqDR+/+_356	0	1	0	0	0	0	0	0	0	0	4.87	4.86	4.92	5.20	4.91	4.93	4.94	5.43	4.99	4.91	4.88	4.66	5.25	5.16	4.86	5.15	4.99	4.83	4.78	4.66
1384816_at	Cxadr	coxsackie virus and adenovirus receptor	1	uniqDR+/+_356	0	1	0	0	0	0	0	0	0	0	4.76	5.11	5.33	5.02	4.48	4.91	5.17	5.03	5.35	5.46	5.40	5.52	5.32	5.49	5.53	5.55	5.41	5.31	5.39	5.58
1384895_at	Pacs2	phosphofurin acidic cluster sorting protein 2	1	uniqDR+/+_356	0	1	0	0	0	0	0	0	0	0	3.87	3.98	3.69	3.67	3.38	3.60	3.90	3.82	3.68	3.83	3.83	3.98	3.47	4.01	3.94	3.87	3.82	3.90	4.02	3.88
1384900_at	Mob3a	MOB kinase activator 3A	1	uniqDR+/+_356	0	1	0	0	0	0	0	0	0	0	4.01	3.92	4.28	4.08	4.02	3.85	4.14	4.29	3.98	3.97	4.01	3.94	3.96	3.95	4.11	3.86	3.87	3.87	3.78	3.73
1384944_at	Bcl11b	B-cell CLL/lymphoma 11B (zinc finger protein)	1	uniqDR+/+_356	0	1	0	0	0	0	0	0	0	0	2.38	2.26	2.24	2.92	2.20	2.47	2.57	4.26	2.10	2.09	2.14	2.27	2.13	2.25	2.21	2.28	1.99	2.08	2.25	2.29
1384981_at	Cd6	Cd6 molecule	1	uniqDR+/+_356	0	1	0	0	0	0	0	0	0	0	5.33	5.32	5.20	5.53	5.25	5.35	5.48	5.77	5.33	5.34	5.30	5.54	5.34	5.43	5.32	5.51	5.39	5.39	5.38	5.40
1385035_at	Usp12	ubiquitin specific peptidase 12	1	uniqDR+/+_356	0	1	0	0	0	0	0	0	0	0	4.90	5.24	5.38	5.27	5.00	5.29	5.37	5.59	5.35	5.67	5.67	5.65	5.24	5.81	5.92	5.79	5.33	5.63	5.61	5.76
1385058_at	Cldn8	claudin 8	1	uniqDR+/+_356	0	1	0	0	0	0	0	0	0	0	3.94	3.96	3.74	4.04	3.72	3.71	4.55	4.63	3.80	3.51	3.43	3.91	4.14	4.29	3.96	4.00	3.86	3.62	3.65	3.80
1385155_at	Lrrfip1	leucine rich repeat (in FLII) interacting protein 1	1	uniqDR+/+_356	0	1	0	0	0	0	0	0	0	0	3.94	4.05	4.01	3.86	4.08	4.12	3.81	4.20	3.77	3.76	3.89	3.67	3.60	4.06	3.85	3.88	3.94	3.97	3.87	3.99
1385397_at	Steap4	STEAP family member 4	1	uniqDR+/+_356	0	1	0	0	0	0	0	0	0	0	6.75	7.14	7.14	6.85	6.58	7.06	6.97	7.09	6.86	7.03	6.74	6.62	7.19	7.06	6.96	7.02	6.78	7.05	7.03	6.86
1385414_at	Cd8a	CD8a molecule	1	uniqDR+/+_356	0	1	0	0	0	0	0	0	0	0	2.51	2.46	2.59	2.95	2.35	2.56	2.78	3.54	2.54	2.45	2.53	2.58	2.57	2.58	2.58	2.78	2.50	2.71	2.65	2.67
1385426_at	Ccdc109b	coiled-coil domain containing 109B	1	uniqDR+/+_356	0	1	0	0	0	0	0	0	0	0	3.67	4.03	4.06	4.12	3.71	3.77	3.83	4.30	3.38	3.40	3.16	3.03	3.20	3.44	3.15	3.10	3.14	3.12	3.06	2.81
1385444_at	---	---	1	uniqDR+/+_356	0	1	0	0	0	0	0	0	0	0	5.40	5.95	5.89	6.01	5.47	5.92	5.85	6.18	4.66	4.98	4.90	5.27	4.78	5.06	5.26	5.29	4.83	5.31	5.16	5.08
1385465_at	Siglec5	sialic acid binding Ig-like lectin 5	1	uniqDR+/+_356	0	1	0	0	0	0	0	0	0	0	1.98	2.05	1.81	2.10	1.96	1.93	1.98	2.62	1.95	2.00	2.12	1.98	1.95	1.98	2.20	2.00	2.11	2.17	2.07	2.11
1385587_at	Mcoln2	mucolipin 2	1	uniqDR+/+_356	0	1	0	0	0	0	0	0	0	0	2.49	2.52	2.50	2.89	2.51	2.44	2.52	3.78	2.54	2.43	2.52	2.47	2.54	2.60	2.61	2.79	2.52	2.53	2.51	2.60
1385853_at	Baz1a	bromodomain adjacent to zinc finger domain, 1A	1	uniqDR+/+_356	0	1	0	0	0	0	0	0	0	0	3.28	3.27	3.49	3.56	3.25	2.89	3.05	4.18	3.37	3.57	3.22	3.12	3.50	3.31	3.39	3.21	3.37	3.10	3.49	3.22
1386162_at	Txk	TXK tyrosine kinase	1	uniqDR+/+_356	0	1	0	0	0	0	0	0	0	0	2.74	2.68	2.70	2.90	2.73	2.75	2.83	3.41	2.75	2.78	2.56	2.64	2.80	2.57	2.74	2.76	2.71	2.79	2.82	2.71
1386166_at	Atp6v1g3	ATPase, H+ transporting, lysosomal V1 subunit G3	1	uniqDR+/+_356	0	1	0	0	0	0	0	0	0	0	2.00	2.07	2.13	2.16	1.97	2.11	2.25	2.25	2.10	2.12	2.20	2.37	1.99	2.15	2.14	2.12	2.28	2.08	2.18	2.16
1386869_at	Actg2	actin, gamma 2, smooth muscle, enteric	1	uniqDR+/+_356	0	1	0	0	0	0	0	0	0	0	2.94	3.10	2.94	3.40	2.81	2.76	3.18	4.39	2.61	2.58	2.68	2.81	2.77	2.56	2.79	2.63	2.69	2.54	2.68	2.78
1386881_at	Igfbp3	insulin-like growth factor binding protein 3	1	uniqDR+/+_356	0	1	0	0	0	0	0	0	0	0	7.50	7.55	7.60	7.88	7.49	7.40	7.76	8.18	7.44	7.45	7.46	7.45	7.90	7.52	7.57	7.99	7.51	7.58	7.62	7.76
1386981_at	Slc16a1	solute carrier family 16, member 1 (monocarboxylic acid transporter 1)	1	uniqDR+/+_356	0	1	0	0	0	0	0	0	0	0	4.42	4.04	4.18	4.63	4.24	4.13	4.20	5.17	4.06	3.94	3.88	3.73	4.53	4.04	3.89	4.12	4.06	3.86	3.97	3.63
1387036_at	Hes1	hairy and enhancer of split 1 (Drosophila)	1	uniqDR+/+_356	0	1	0	0	0	0	0	0	0	0	6.75	6.64	6.66	6.69	6.65	6.68	7.09	7.21	6.48	6.53	6.56	6.51	6.79	6.69	6.45	6.56	6.57	6.60	6.50	6.51
1387040_at	Mal	mal, T-cell differentiation protein	1	uniqDR+/+_356	0	1	0	0	0	0	0	0	0	0	3.87	4.01	4.20	4.36	3.81	4.00	4.28	4.56	3.69	3.81	3.96	4.14	3.93	3.95	4.22	3.93	3.76	3.94	4.10	4.14
1387098_at	Polr1a	polymerase (RNA) I polypeptide A	1	uniqDR+/+_356	0	1	0	0	0	0	0	0	0	0	5.24	5.20	5.15	5.44	5.07	5.27	5.23	5.58	5.22	5.12	5.35	5.39	5.28	5.49	5.48	5.43	5.36	5.44	5.47	5.67
1387114_at	Prkcd	protein kinase C, delta	1	uniqDR+/+_356	0	1	0	0	0	0	0	0	0	0	6.84	6.89	6.76	7.04	6.85	6.85	6.76	7.31	6.94	7.04	6.98	6.84	7.04	7.02	7.02	7.01	6.92	6.93	6.94	6.81
1387190_at	Dgka	diacylglycerol kinase, alpha	1	uniqDR+/+_356	0	1	0	0	0	0	0	0	0	0	6.40	6.52	6.41	6.88	6.53	6.41	6.75	7.20	6.40	6.56	6.43	6.46	6.55	6.59	6.51	6.70	6.68	6.53	6.65	6.65
1387294_at	Sh3bp5	SH3-domain binding protein 5 (BTK-associated)	1	uniqDR+/+_356	0	1	0	0	0	0	0	0	0	0	7.90	8.09	8.15	8.24	8.01	8.11	8.11	8.54	7.48	7.47	7.32	7.66	7.58	7.25	7.49	7.41	7.52	7.72	7.67	7.73
1387315_at	Sftpd	surfactant protein D	1	uniqDR+/+_356	0	1	0	0	0	0	0	0	0	0	4.86	5.03	5.16	5.12	4.72	5.01	5.38	5.24	4.65	4.80	4.85	5.00	4.54	5.04	4.91	5.11	4.73	4.74	4.84	4.95
1387361_s_at	Pgk1	phosphoglycerate kinase 1	1	uniqDR+/+_356	0	1	0	0	0	0	0	0	0	0	8.50	8.48	8.54	8.74	8.43	8.50	8.54	8.94	8.57	8.73	8.69	8.77	8.53	8.68	8.77	8.81	8.64	8.71	8.71	8.87
1387365_at	Nr1h3	nuclear receptor subfamily 1, group H, member 3	1	uniqDR+/+_356	0	1	0	0	0	0	0	0	0	0	5.02	5.27	5.04	5.34	4.92	5.03	4.92	5.43	4.95	4.97	4.75	4.89	5.01	4.87	5.03	5.18	4.78	4.99	4.95	4.90
1387442_at	Egr4	early growth response 4	1	uniqDR+/+_356	0	1	0	0	0	0	0	0	0	0	6.15	6.24	6.49	6.48	6.19	6.03	7.43	6.75	6.12	6.12	6.53	6.00	5.80	6.07	6.05	6.23	5.69	5.94	6.12	5.86
1387483_at	Plcg2	phospholipase C, gamma 2	1	uniqDR+/+_356	0	1	0	0	0	0	0	0	0	0	3.81	3.72	3.71	4.23	3.85	3.77	3.67	4.88	3.62	3.41	3.72	3.64	3.67	3.61	3.59	3.73	3.55	3.61	3.58	3.62
1387566_at	Pla2g4a	phospholipase A2, group IVA (cytosolic, calcium-dependent)	1	uniqDR+/+_356	0	1	0	0	0	0	0	0	0	0	3.37	3.14	3.13	4.32	3.34	3.03	3.36	4.28	3.21	3.29	2.97	3.28	3.56	3.14	3.53	3.46	3.41	3.17	3.20	3.26
1387588_at	Ehd3	EH-domain containing 3	1	uniqDR+/+_356	0	1	0	0	0	0	0	0	0	0	4.58	4.59	4.57	4.66	4.55	4.53	4.68	4.90	4.48	4.56	4.67	4.60	4.37	4.65	4.57	4.60	4.41	4.42	4.67	4.58
1387605_at	Casp12	caspase 12	1	uniqDR+/+_356	0	1	0	0	0	0	0	0	0	0	3.20	3.15	3.25	3.52	3.05	3.30	3.49	3.57	3.18	3.44	3.25	3.63	3.27	3.26	3.60	3.55	3.33	3.32	3.52	3.57
1387621_at	Tnfrsf4	tumor necrosis factor receptor superfamily, member 4	1	uniqDR+/+_356	0	1	0	0	0	0	0	0	0	0	5.87	5.90	5.96	6.22	5.71	5.86	6.04	6.21	5.73	5.96	5.92	6.14	5.88	5.94	5.85	6.10	5.88	5.92	6.10	6.01
1387651_at	Aqp1	aquaporin 1	1	uniqDR+/+_356	0	1	0	0	0	0	0	0	0	0	5.72	5.62	5.54	5.81	5.59	5.76	5.75	5.70	5.29	5.54	5.49	5.71	5.32	5.48	5.50	5.69	5.71	5.69	5.63	5.67
1387711_at	Il10	interleukin 10	1	uniqDR+/+_356	0	1	0	0	0	0	0	0	0	0	2.46	2.25	2.33	3.04	2.44	2.42	2.41	3.07	2.28	2.46	2.41	2.41	2.37	2.35	2.59	2.22	2.42	2.23	2.32	2.44
1387739_at	Cd8b	CD8b molecule	1	uniqDR+/+_356	0	1	0	0	0	0	0	0	0	0	3.51	3.37	3.42	3.58	3.39	3.54	3.85	3.91	3.29	3.54	3.47	3.52	3.63	3.63	3.39	3.54	3.38	3.56	3.70	3.68
1387760_a_at	Onecut1	one cut homeobox 1	1	uniqDR+/+_356	0	1	0	0	0	0	0	0	0	0	3.59	3.55	3.63	3.77	3.86	3.75	4.11	4.03	3.50	3.56	3.57	3.74	3.63	3.71	3.76	3.99	3.62	3.43	3.63	3.86
1387870_at	Zfp36	zinc finger protein 36	1	uniqDR+/+_356	0	1	0	0	0	0	0	0	0	0	7.53	8.20	8.21	8.04	7.86	8.06	9.07	8.54	7.98	8.50	8.75	8.27	8.17	8.11	8.29	8.27	8.04	8.28	8.41	7.90
1387951_at	Cd55	Cd55 molecule	1	uniqDR+/+_356	0	1	0	0	0	0	0	0	0	0	3.71	4.03	3.87	4.40	4.05	3.94	3.78	4.85	3.32	3.33	3.17	2.71	3.11	2.91	3.19	3.64	3.19	3.04	3.10	2.88
1388298_at	Myl9	myosin, light chain 9, regulatory	1	uniqDR+/+_356	0	1	0	0	0	0	0	0	0	0	7.21	7.34	7.22	7.42	7.32	7.25	7.45	8.12	6.72	6.58	6.57	6.88	6.91	6.67	6.84	6.65	6.80	6.81	6.63	6.75
1388318_at	Pgk1	phosphoglycerate kinase 1	1	uniqDR+/+_356	0	1	0	0	0	0	0	0	0	0	9.37	9.40	9.52	9.63	9.32	9.41	9.46	9.89	9.53	9.54	9.53	9.59	9.48	9.53	9.60	9.63	9.52	9.56	9.55	9.71
1388340_at	Ns5atp9	NS5A (hepatitis C virus) transactivated protein 9	1	uniqDR+/+_356	0	1	0	0	0	0	0	0	0	0	5.69	5.92	5.52	6.72	5.86	6.10	5.66	7.81	5.62	5.84	6.09	5.72	5.65	5.54	5.85	5.86	5.76	5.92	5.76	5.81
1388408_at	Prr13	proline rich 13	1	uniqDR+/+_356	0	1	0	0	0	0	0	0	0	0	8.45	8.41	8.44	8.81	8.34	8.42	8.57	8.95	8.40	8.53	8.54	8.52	8.41	8.42	8.55	8.62	8.38	8.47	8.51	8.53
1388528_at	Fbl	fibrillarin	1	uniqDR+/+_356	0	1	0	0	0	0	0	0	0	0	8.33	8.24	8.37	8.48	8.29	8.23	8.26	8.74	8.32	8.29	8.25	8.28	8.34	8.33	8.31	8.33	8.36	8.31	8.22	8.38
1388569_at	Serpinf1	serpin peptidase inhibitor, clade F (alpha-2 antiplasmin, pigment epithelium derived factor),	1	uniqDR+/+_356	0	1	0	0	0	0	0	0	0	0	4.86	5.51	5.70	5.60	4.88	5.61	5.51	5.91	4.21	4.43	4.57	4.96	4.10	4.57	4.83	4.97	4.41	4.68	5.30	5.35
1388581_at	Hn1	hematological and neurological expressed 1	1	uniqDR+/+_356	0	1	0	0	0	0	0	0	0	0	7.78	7.70	7.65	7.81	7.74	7.68	7.68	8.05	7.68	7.61	7.49	7.39	7.70	7.54	7.54	7.36	7.62	7.48	7.41	7.35
1388596_at	Cotl1	coactosin-like 1 (Dictyostelium)	1	uniqDR+/+_356	0	1	0	0	0	0	0	0	0	0	7.67	7.46	7.58	7.98	7.54	7.46	7.50	8.27	7.90	7.78	7.93	8.03	8.12	7.78	7.87	7.97	7.88	7.81	8.02	8.21
1388602_at	Cfd	complement factor D (adipsin)	1	uniqDR+/+_356	0	1	0	0	0	0	0	0	0	0	4.35	4.00	4.18	5.18	4.25	4.24	4.51	5.08	3.31	3.55	3.66	3.62	3.51	3.71	4.02	4.57	3.74	3.61	3.72	3.46
1388629_at	Impdh2	IMP (inosine 5'-monophosphate) dehydrogenase 2	1	uniqDR+/+_356	0	1	0	0	0	0	0	0	0	0	8.81	8.79	8.82	8.90	8.82	8.86	8.82	9.37	8.74	8.66	8.63	8.37	8.73	8.73	8.68	8.66	8.73	8.65	8.54	8.52
1388663_at	LOC687118	similar to death effector domain-containing DNA binding protein 2	1	uniqDR+/+_356	0	1	0	0	0	0	0	0	0	0	4.86	4.92	4.80	5.27	4.72	4.77	5.37	5.25	5.09	5.08	4.94	5.15	4.89	5.02	5.15	5.00	5.05	4.90	4.84	4.95
1388837_at	Slc44a2	solute carrier family 44, member 2	1	uniqDR+/+_356	0	1	0	0	0	0	0	0	0	0	6.45	6.37	6.32	6.71	6.44	6.29	6.48	7.06	6.35	6.42	6.28	6.33	6.68	6.30	6.29	6.34	6.41	6.30	6.56	6.43
1388846_at	Bcl2l12	BCL2-like 12 (proline rich)	1	uniqDR+/+_356	0	1	0	0	0	0	0	0	0	0	3.64	3.44	3.35	4.12	3.59	3.56	3.52	4.56	3.38	3.33	3.47	3.45	3.63	3.24	3.43	3.56	3.38	3.40	3.48	3.37
1388864_at	Ddx54	DEAD (Asp-Glu-Ala-Asp) box polypeptide 54	1	uniqDR+/+_356	0	1	0	0	0	0	0	0	0	0	5.64	5.54	5.54	5.79	5.51	5.56	5.65	6.05	5.91	5.72	5.83	5.81	6.17	5.87	5.87	5.76	5.87	5.84	5.95	5.90
1388887_at	Ggta1p	glycoprotein, alpha-galactosyltransferase 1,3	1	uniqDR+/+_356	0	1	0	0	0	0	0	0	0	0	6.89	6.95	7.02	7.49	6.96	6.92	6.95	7.96	6.69	6.73	7.03	7.28	6.98	6.89	7.16	7.38	6.87	6.91	7.10	7.46
1389063_at	LOC10091200	exportin-6-like /// exportin 6	1	uniqDR+/+_356	0	1	0	0	0	0	0	0	0	0	8.72	8.71	9.14	8.95	8.72	8.72	8.67	9.68	8.68	8.72	8.69	8.54	8.82	8.63	8.65	9.00	8.65	8.67	8.64	9.06
1389071_at	Ehbp1l1	EH domain binding protein 1-like 1	1	uniqDR+/+_356	0	1	0	0	0	0	0	0	0	0	5.79	5.83	5.58	6.19	5.66	5.57	5.85	6.28	5.78	5.68	5.55	5.71	5.87	5.65	5.48	5.70	5.93	5.69	5.55	5.74
1389074_at	Fchsd2	FCH and double SH3 domains 2	1	uniqDR+/+_356	0	1	0	0	0	0	0	0	0	0	8.26	8.36	8.42	8.68	8.33	8.39	8.32	8.82	8.23	8.33	8.37	8.29	8.37	8.23	8.36	8.48	8.37	8.36	8.34	8.44
1389079_at	Dhrs7c	dehydrogenase/reductase (SDR family) member 7C	1	uniqDR+/+_356	0	1	0	0	0	0	0	0	0	0	2.43	2.35	2.34	2.91	2.43	2.40	2.36	2.95	2.17	2.12	2.20	2.41	2.16	2.38	2.14	2.22	2.26	2.18	2.31	2.14
1389110_at	LOC10036001	mitochondrial ribosomal protein S6-like	1	uniqDR+/+_356	0	1	0	0	0	0	0	0	0	0	7.48	7.45	7.59	7.81	7.36	7.44	7.72	7.87	7.65	7.85	7.81	7.75	7.53	7.70	7.78	7.78	7.75	7.62	7.66	7.67
1389123_at	Ccl6	chemokine (C-C motif) ligand 6	1	uniqDR+/+_356	0	1	0	0	0	0	0	0	0	0	4.67	5.16	5.29	5.52	4.63	4.95	5.25	5.94	4.80	4.91	5.42	5.20	4.88	4.99	5.41	5.44	4.56	5.03	5.19	4.51
1389227_at	Rhog	ras homolog family member G	1	uniqDR+/+_356	0	1	0	0	0	0	0	0	0	0	7.19	7.25	7.20	7.49	7.17	7.26	7.24	7.70	7.33	7.16	7.17	7.15	7.31	7.28	7.27	7.28	7.29	7.16	7.22	7.24
1389351_at	Lrrfip1	leucine rich repeat (in FLII) interacting protein 1	1	uniqDR+/+_356	0	1	0	0	0	0	0	0	0	0	5.69	5.87	5.99	6.22	5.82	5.62	6.12	6.82	5.48	5.66	5.53	5.74	5.78	5.67	5.78	5.89	5.52	5.63	5.78	5.57
1389359_at	Smc4	structural maintenance of chromosomes 4	1	uniqDR+/+_356	0	1	0	0	0	0	0	0	0	0	2.97	3.10	3.05	3.21	3.02	3.12	3.12	3.59	3.41	3.37	3.23	3.06	3.10	3.07	3.14	3.28	3.26	3.32	3.10	3.14
1389384_at	Ndufaf4	NADH dehydrogenase (ubiquinone) complex I, assembly factor 4	1	uniqDR+/+_356	0	1	0	0	0	0	0	0	0	0	7.26	7.43	7.43	7.46	7.03	7.38	7.48	7.71	7.33	7.37	7.42	7.45	7.28	7.51	7.49	7.47	7.34	7.41	7.39	7.60
1389425_at	LOC10036058	5',3'-nucleotidase, cytosolic	1	uniqDR+/+_356	0	1	0	0	0	0	0	0	0	0	4.58	4.66	4.72	5.04	4.72	4.39	4.68	5.63	4.68	4.60	4.61	4.60	5.05	4.78	4.67	4.77	4.76	4.52	4.59	4.48
1389595_at	Dus2l	dihydrouridine synthase 2-like, SMM1 homolog (S. cerevisiae)	1	uniqDR+/+_356	0	1	0	0	0	0	0	0	0	0	6.85	7.10	6.97	7.25	6.80	6.97	7.10	7.39	6.79	6.93	6.95	7.05	6.71	7.07	7.04	7.05	6.91	6.94	7.00	7.02
1389659_at	Ctla2a	cytotoxic T lymphocyte-associated protein 2 alpha	1	uniqDR+/+_356	0	1	0	0	0	0	0	0	0	0	5.19	5.32	5.26	5.63	5.35	5.45	5.47	5.85	5.34	5.26	5.37	5.38	5.62	5.37	5.47	5.67	5.49	5.28	5.37	5.29
1389729_at	Rilpl2	Rab interacting lysosomal protein-like 2	1	uniqDR+/+_356	0	1	0	0	0	0	0	0	0	0	8.40	8.51	8.65	8.85	8.37	8.44	8.57	9.27	8.47	8.55	8.54	8.65	8.63	8.56	8.58	8.73	8.44	8.47	8.58	8.62
1389852_at	Cenpm	centromere protein M	1	uniqDR+/+_356	0	1	0	0	0	0	0	0	0	0	4.34	4.67	4.66	4.83	4.58	4.48	4.75	4.99	4.49	4.68	4.68	4.78	4.50	4.73	4.64	4.77	4.42	4.62	4.62	4.69
1389980_at	Cnih4	cornichon homolog 4 (Drosophila)	1	uniqDR+/+_356	0	1	0	0	0	0	0	0	0	0	6.44	6.41	6.48	6.66	6.21	6.43	6.40	6.79	6.56	6.63	6.53	6.33	6.31	6.58	6.66	6.67	6.47	6.39	6.35	6.51
1390034_at	---	---	1	uniqDR+/+_356	0	1	0	0	0	0	0	0	0	0	6.25	6.19	6.29	6.50	6.20	6.16	6.25	6.72	6.21	6.21	6.26	6.16	6.27	6.14	6.25	6.22	6.29	5.99	6.24	6.27
1390049_at	Fhl1	four and a half LIM domains 1	1	uniqDR+/+_356	0	1	0	0	0	0	0	0	0	0	3.52	3.64	3.67	3.84	3.41	3.58	3.87	4.62	3.19	3.39	3.41	3.70	3.07	3.45	3.58	3.55	3.51	3.37	3.21	3.49
1390112_at	Efemp1	EGF-containing fibulin-like extracellular matrix protein 1	1	uniqDR+/+_356	0	1	0	0	0	0	0	0	0	0	2.51	2.45	2.67	2.77	2.30	2.34	2.63	3.33	2.28	2.51	2.47	2.47	2.16	2.48	2.43	2.47	2.18	2.44	2.42	2.45

Affymetrix Probeset ID	Gene Symbol	Gene Name	unique BB on P7 n=822	Figure 1B P7 Venn n=1526	DRlyp/lyp STEM P7 n=712	DR+/+ STEM P7 n=936	Flyp/lyp STEM P7 n=520	F+/+ STEM P7 n=465	Figure 1B P0 Venn n=1071	DRlyp/lyp STEM P0 n=442	DR+/+ STEM P0 n=437	Flyp/lyp STEM P0 n=643	F+/+ STEM P0 n=632	Fig1D n=25	Mean log2 intensity DRlyp/lyp 20	Mean log2 intensity DRlyp/lyp 30	Mean log2 intensity DRlyp/lyp 40	Mean log2 intensity DRlyp/lyp 50	Mean log2 intensity DR+/+ 20	Mean log2 intensity DR+/+ 30	Mean log2 intensity DR+/+ 40	Mean log2 intensity DR+/+ 50	Mean log2 intensity Flyp/lyp 20	Mean log2 intensity Flyp/lyp 30	Mean log2 intensity Flyp/lyp 40	Mean log2 intensity Flyp/lyp 50	Mean log2 intensity Flyp20	Mean log2 intensity Flyp30	Mean log2 intensity Flyp40	Mean log2 intensity Flyp50	Mean log2 intensity F+/+ 20	Mean log2 intensity F+/+ 30	Mean log2 intensity F+/+ 40	Mean log2 intensity F+/+ 50
1390195_at	Psd4	pleckstrin and Sec7 domain containing 4	1	uniqDR+/+_356	0	1	0	0	0	0	0	0	0	0	4.65	4.71	4.85	5.12	4.52	4.65	4.84	5.38	4.73	4.65	4.75	5.15	4.79	4.78	4.68	4.99	4.73	4.69	4.86	4.81
1390209_at	Syngr3	synaptogyrin 3	1	uniqDR+/+_356	0	1	0	0	0	0	0	0	0	0	3.25	3.38	3.48	3.66	3.38	3.58	3.97	3.67	3.15	4.54	3.29	3.43	3.31	3.63	3.41	3.20	3.39	3.36	3.20	3.62
1390914_at	Fli1	Friend leukemia virus integration 1	1	uniqDR+/+_356	0	1	0	0	0	0	0	0	0	0	5.99	5.84	5.85	6.49	5.90	5.78	5.92	7.04	5.81	5.81	5.61	5.70	6.20	5.77	5.59	5.96	5.96	5.96	5.91	5.65
1390948_at	Phemx	pan hematopoietic expression	1	uniqDR+/+_356	0	1	0	0	0	0	0	0	0	0	2.85	2.70	2.80	3.01	2.76	2.73	3.10	3.47	2.80	2.89	3.00	3.05	2.82	2.54	2.97	3.00	2.92	2.97	2.94	2.81
1391112_at	Ggta1p	glycoprotein, alpha-galactosyltransferase 1,3	1	uniqDR+/+_356	0	1	0	0	0	0	0	0	0	0	3.75	3.78	3.54	3.88	3.59	3.73	3.76	4.24	3.55	3.57	3.50	3.69	3.87	3.77	3.59	3.79	3.91	3.77	3.76	3.67
1391323_at	Srprb /// Tf	signal recognition particle receptor, B subunit /// transferrin	1	uniqDR+/+_356	0	1	0	0	0	0	0	0	0	0	2.42	2.48	2.48	2.43	2.30	2.32	2.68	2.55	2.46	2.31	2.44	2.48	2.42	2.26	2.41	2.42	2.46	2.47	2.63	2.51
1391442_at	---	---	1	uniqDR+/+_356	0	1	0	0	0	0	0	0	0	0	5.28	5.19	5.15	5.76	5.18	5.01	5.20	6.27	5.13	5.29	5.16	5.13	5.12	5.25	5.03	5.24	5.16	5.05	5.03	5.22
1391453_at	Ebi3	Epstein-Barr virus induced 3	1	uniqDR+/+_356	0	1	0	0	0	0	0	0	0	0	3.93	4.16	4.07	4.29	4.01	3.96	4.11	4.53	4.04	4.20	4.16	4.11	4.15	4.11	4.08	4.05	4.16	4.10	4.20	4.12
1391787_at	Traf3ip3	TRAF3 interacting protein 3	1	uniqDR+/+_356	0	1	0	0	0	0	0	0	0	0	4.47	4.36	4.48	4.73	4.58	4.51	4.44	4.95	4.55	4.44	4.35	4.55	4.43	4.55	4.47	4.60	4.38	4.59	4.44	4.48
1391797_at	Mef2b	myocyte enhancer factor 2B	1	uniqDR+/+_356	0	1	0	0	0	0	0	0	0	0	4.54	4.50	4.55	4.73	4.57	4.58	4.97	5.14	4.58	4.59	4.69	4.93	4.49	4.58	4.72	4.66	4.43	4.75	4.54	4.70
1391835_at	Baz1a	bromodomain adjacent to zinc finger domain, 1A	1	uniqDR+/+_356	0	1	0	0	0	0	0	0	0	0	2.75	2.75	2.73	2.68	2.75	2.70	2.85	2.79	2.57	2.86	2.93	2.91	2.59	2.70	2.79	2.77	2.71	2.72	2.87	2.89
1391910_at	Rftn1	raftlin lipid raft linker 1	1	uniqDR+/+_356	0	1	0	0	0	0	0	0	0	0	5.34	5.40	5.43	5.23	5.20	5.16	5.52	5.54	5.15	5.40	5.35	5.73	5.24	5.55	5.31	5.48	5.15	5.37	5.59	5.61
1391948_at	Bcl11b	B-cell CLL/lymphoma 11B (zinc finger protein)	1	uniqDR+/+_356	0	1	0	0	0	0	0	0	0	0	3.08	2.84	2.76	3.51	3.00	3.02	3.30	3.93	2.88	2.81	2.75	3.04	2.92	2.96	2.79	3.13	2.73	2.69	2.84	2.95
1392560_at	Pip5k1a	phosphatidylinositol-4-phosphate 5-kinase, type 1, alpha	1	uniqDR+/+_356	0	1	0	0	0	0	0	0	0	0	6.09	6.22	5.90	6.69	5.95	5.94	6.26	6.51	6.36	6.25	6.17	6.19	6.05	6.05	6.03	6.33	6.15	6.14	6.54	6.27
1392708_at	Stard6	StAR-related lipid transfer (START) domain containing 6	1	uniqDR+/+_356	0	1	0	0	0	0	0	0	0	0	4.96	5.30	5.45	5.31	4.92	5.26	5.54	5.43	4.70	5.02	5.17	5.20	4.41	4.94	5.18	5.16	4.72	5.03	5.10	5.19
1392722_at	Dapp1	dual adaptor of phosphotyrosine and 3-phosphoinositides	1	uniqDR+/+_356	0	1	0	0	0	0	0	0	0	0	4.86	5.17	5.21	5.67	5.17	5.17	5.20	6.03	5.07	5.20	5.19	5.06	5.33	4.99	5.06	5.35	5.07	4.80	5.06	4.95
1392976_at	Tpm2	tropomyosin 2, beta	1	uniqDR+/+_356	0	1	0	0	0	0	0	0	0	0	2.80	2.78	2.60	2.76	2.82	2.99	2.66	2.76	2.74	2.62	2.77	2.82	2.66	2.91	2.84	2.72	2.73	2.91	2.81	3.03
1393039_a_at	Rab17	RAB17, member RAS oncogene family	1	uniqDR+/+_356	0	1	0	0	0	0	0	0	0	0	5.10	5.30	5.35	5.45	5.10	5.27	5.58	5.56	5.23	5.28	5.30	5.35	5.17	5.29	5.31	5.35	5.23	5.24	5.20	5.40
1393121_at	Agr2	anterior gradient 2 homolog (Xenopus laevis)	1	uniqDR+/+_356	0	1	0	0	0	0	0	0	0	0	3.04	3.08	2.92	3.19	2.94	3.11	3.48	3.45	2.82	2.95	2.95	3.11	2.94	2.91	3.05	3.23	2.94	2.99	2.96	3.03
1393171_at	Tmem47	transmembrane protein 47	1	uniqDR+/+_356	0	1	0	0	0	0	0	0	0	0	3.64	3.61	3.50	3.61	3.45	3.61	3.71	3.97	3.41	3.50	3.61	3.65	3.61	3.73	3.68	3.68	3.44	3.75	3.71	3.89
1393201_at	Lrmp	lymphoid-restricted membrane protein	1	uniqDR+/+_356	0	1	0	0	0	0	0	0	0	0	4.81	4.78	4.73	5.19	4.73	4.66	4.84	5.85	4.75	4.70	4.88	4.77	4.71	5.03	4.72	4.89	4.74	4.89	4.73	4.98
1393210_at	LOC10036138	extracellular matrix protein 2-like	1	uniqDR+/+_356	0	1	0	0	0	0	0	0	0	0	4.42	4.58	4.58	4.59	4.41	4.68	4.62	4.96	4.00	3.95	3.82	4.26	4.34	4.05	4.27	4.18	4.08	3.99	4.20	4.04
1393315_at	Traf3ip3	TRAF3 interacting protein 3	1	uniqDR+/+_356	0	1	0	0	0	0	0	0	0	0	3.00	3.02	2.99	3.53	3.01	3.04	3.15	3.88	3.06	2.96	3.03	3.12	2.97	2.86	2.95	3.15	3.02	2.90	3.03	3.05
1393319_a_at	Rab17	RAB17, member RAS oncogene family	1	uniqDR+/+_356	0	1	0	0	0	0	0	0	0	0	2.89	2.89	2.73	3.22	2.71	2.78	3.22	3.08	2.74	2.86	2.91	3.12	2.98	2.72	2.81	2.98	2.79	2.56	3.08	2.81
1393413_at	LOC10091143	neuronal tyrosine-phosphorylated phosphoinositide-3-kinase adapter 1-like /// neuronal tyro	1	uniqDR+/+_356	0	1	0	0	0	0	0	0	0	0	5.59	5.50	5.38	6.03	5.21	5.62	5.71	5.89	5.50	5.67	5.49	5.56	5.27	5.52	5.32	5.77	5.50	5.30	5.70	5.68
1393654_at	Neil1	nei endonuclease VIII-like 1 (E. coli)	1	uniqDR+/+_356	0	1	0	0	0	0	0	0	0	0	6.15	6.31	6.33	6.53	6.22	6.21	6.34	6.74	6.22	6.32	6.41	6.25	6.22	6.35	6.44	6.43	6.29	6.22	6.36	6.37
1393682_at	Gpr34	G protein-coupled receptor 34	1	uniqDR+/+_356	0	1	0	0	0	0	0	0	0	0	1.88	1.89	1.84	2.04	1.78	1.91	1.96	2.36	1.83	1.93	1.92	1.85	2.11	2.01	2.13	2.02	2.08	1.93	1.92	1.98
1393755_at	Pik3cd	phosphatidylinositol-4,5-bisphosphate 3-kinase, catalytic subunit delta	1	uniqDR+/+_356	0	1	0	0	0	0	0	0	0	0	3.81	3.75	3.75	4.22	3.58	3.58	3.90	4.69	3.61	3.79	3.73	3.83	3.63	3.78	3.80	3.97	3.61	3.71	3.88	3.90
1394480_at	LOC10030237	hypothetical protein LOC100302372	1	uniqDR+/+_356	0	1	0	0	0	0	0	0	0	0	4.81	5.00	4.86	5.04	4.99	5.00	4.93	5.27	4.98	4.82	4.95	4.74	5.07	5.06	4.89	4.78	5.01	5.06	4.78	4.86
1394673_at	Cd33	CD33 molecule	1	uniqDR+/+_356	0	1	0	0	0	0	0	0	0	0	4.10	3.98	3.94	4.75	4.13	4.24	4.15	4.89	3.98	3.99	4.00	4.10	3.97	4.02	4.21	4.71	3.91	4.26	4.02	3.90
1394678_at	Fgd2	FYVE, RhoGEF and PH domain containing 2	1	uniqDR+/+_356	0	1	0	0	0	0	0	0	0	0	5.52	5.68	5.58	6.15	5.17	5.52	5.62	6.43	5.29	5.45	5.50	5.44	4.82	5.56	5.62	5.61	5.11	5.38	5.47	5.32
1394750_at	Fhl1	four and a half LIM domains 1	1	uniqDR+/+_356	0	1	0	0	0	0	0	0	0	0	3.26	3.36	3.59	3.42	3.24	3.35	3.32	3.40	3.20	3.14	3.32	3.45	3.16	3.40	3.39	3.38	3.33	3.45	3.48	3.44
1395116_at	Cd8a	CD8a molecule	1	uniqDR+/+_356	0	1	0	0	0	0	0	0	0	0	2.90	2.99	2.96	3.25	2.98	3.06	3.13	3.34	2.97	2.97	3.01	3.07	2.77	3.08	3.07	3.11	3.05	3.16	3.11	3.23
1395394_at	Gga2	golgi associated, gamma adaptin ear containing, ARF binding protein 2	1	uniqDR+/+_356	0	1	0	0	0	0	0	0	0	0	6.07	6.15	6.28	6.19	6.03	6.06	6.17	6.60	6.07	6.21	6.17	6.05	4.49	6.18	6.19	5.91	6.09	5.89	5.32	5.32
1395635_at	Tnfrsf26	tumor necrosis factor receptor superfamily, member 26	1	uniqDR+/+_356	0	1	0	0	0	0	0	0	0	0	4.08	3.98	3.90	4.44	3.93	3.91	4.07	5.18	3.79	3.78	3.92	3.85	4.11	3.95	3.93	4.00	4.00	3.96	4.00	4.08
1395697_at	Ezh2	enhancer of zeste homolog 2 (Drosophila)	1	uniqDR+/+_356	0	1	0	0	0	0	0	0	0	0	5.17	5.39	5.33	5.65	5.56	5.25	5.27	6.23	5.17	5.13	5.12	4.95	4.48	4.78	5.08	5.07	5.16	4.73	5.03	5.03
1395960_at	LOC688276	similar to epidermodysplasia verruciformis 2	1	uniqDR+/+_356	0	1	0	0	0	0	0	0	0	0	2.58	2.63	2.62	3.01	2.68	2.71	2.91	3.27	2.42	2.59	2.73	2.83	2.54	2.68	2.63	2.84	2.50	2.58	2.67	2.82
1396035_at	Sbspon	somatomedin B and thrombospondin, type 1 domain containing	1	uniqDR+/+_356	0	1	0	0	0	0	0	0	0	0	3.13	3.24	3.30	3.18	3.16	3.24	3.79	3.61	3.07	3.05	3.11	3.20	3.20	3.08	3.30	3.47	3.18	3.16	3.29	3.01
1396036_at	Ralgps2	Ral GEF with PH domain and SH3 binding motif 2	1	uniqDR+/+_356	0	1	0	0	0	0	0	0	0	0	4.97	4.95	4.92	5.19	4.83	4.78	4.97	5.72	4.93	4.75	4.97	4.56	4.02	4.59	4.77	4.87	4.84	4.69	4.60	4.65
1396231_at	Rasal3	RAS protein activator like 3	1	uniqDR+/+_356	0	1	0	0	0	0	0	0	0	0	3.29	3.27	3.24	3.65	3.13	3.17	3.29	3.71	3.15	3.17	3.41	3.47	3.25	3.23	3.28	3.64	3.32	3.29	3.38	3.40
1396933_s_at	Akr1c14	aldo-keto reductase family 1, member C14	1	uniqDR+/+_356	0	1	0	0	0	0	0	0	0	0	3.07	3.23	3.36	3.55	3.28	3.45	3.84	3.68	3.25	3.46	3.42	3.85	3.43	3.56	3.52	3.78	3.54	3.46	3.75	3.79
1397335_at	Sema3d	sema domain, immunoglobulin domain (Ig), short basic domain, secreted, (semaphorin) 3D	1	uniqDR+/+_356	0	1	0	0	0	0	0	0	0	0	3.67	3.55	3.59	3.72	3.28	3.70	3.71	4.08	2.87	3.23	3.33	3.20	3.02	3.34	3.29	3.19	3.17	3.28	3.31	3.20
1397411_at	Fgd2	FYVE, RhoGEF and PH domain containing 2	1	uniqDR+/+_356	0	1	0	0	0	0	0	0	0	0	4.29	4.60	4.40	4.74	4.37	4.59	4.65	4.81	4.32	4.40	4.65	4.60	4.36	4.73	4.60	4.54	4.31	4.65	4.32	4.58
1397684_at	Dkk2	dickkopf WNT signaling pathway inhibitor 2	1	uniqDR+/+_356	0	1	0	0	0	0	0	0	0	0	2.68	2.82	3.01	2.78	2.68	2.71	2.71	3.30	2.01	2.57	2.29	2.47	2.17	2.38	2.41	2.42	2.24	2.36	2.41	2.53
1398272_at	B4galnt1	beta-1,4-N-acetyl-galactosaminyl transferase 1	1	uniqDR+/+_356	0	1	0	0	0	0	0	0	0	0	3.58	3.59	3.67	3.95	3.44	3.57	3.75	4.02	3.58	3.57	3.59	3.65	3.57	3.65	3.62	3.66	3.40	3.64	3.60	3.51
1398282_at	Kynu	kynureninase	1	uniqDR+/+_356	0	1	0	0	0	0	0	0	0	0	3.15	3.10	3.12	3.50	3.20	3.22	3.06	4.51	2.92	3.10	3.34	3.23	3.15	3.19	3.27	3.42	3.06	3.18	3.27	3.27
1398356_at	Nudt21	nudix (nucleoside diphosphate linked moiety X)-type motif 21	1	uniqDR+/+_356	0	1	0	0	0	0	0	0	0	0	8.08	8.15	8.15	8.30	8.19	8.08	8.17	8.61	8.30	8.24	8.18	8.13	8.25	8.19	8.26	8.16	8.24	8.16	8.07	8.06
1398602_at	Mad2l1	MAD2 mitotic arrest deficient-like 1 (yeast)	1	uniqDR+/+_356	0	1	0	0	0	0	0	0	0	0	4.83	5.36	5.17	5.51	5.07	5.34	5.13	5.88	4.95	5.61	5.55	5.28	5.00	5.28	5.49	5.50	5.20	5.50	5.33	5.46
1398641_at	Plekha3	pleckstrin homology domain-containing, family A (phosphoinositide binding specific) membe	1	uniqDR+/+_356	0	1	0	0	0	0	0	0	0	0	6.51	6.75	6.89	6.85	6.71	6.45	6.76	7.20	6.71	6.81	6.67	6.79	6.90	6.72	6.79	6.82	6.70	6.59	6.82	6.91
1399152_at	Eps15	epidermal growth factor receptor pathway substrate 15	1	uniqDR+/+_356	0	1	0	0	0	0	0	0	0	0	5.99	6.03	6.12	6.47	5.82	5.93	6.11	6.71	6.06	5.99	6.03	6.16	6.12	5.94	6.22	6.28	6.02	5.95	6.14	6.19
1367710_at	Psme2	proteasome (prosome, macropain) activator subunit 2	1	uniqDRlyp/lyp_119	1	0	0	0	0	0	0	0	0	0	8.83	8.79	8.86	9.47	8.82	8.75	8.80	9.09	8.79	8.83	8.71	8.77	8.79	8.76	8.82	8.81	8.78	8.69	8.77	8.63
1367925_at	Mvp	major vault protein	1	uniqDRlyp/lyp_119	1	0	0	0	0	0	0	0	0	0	7.94	7.98	8.14	8.45	7.86	7.91	7.94	8.20	8.05	8.09	8.19	8.42	8.15	8.00	8.18	8.32	7.95	8.07	8.25	8.38
1368059_at	Crym	crystallin, mu	1	uniqDRlyp/lyp_119	1	0	0	0	0	0	0	0	0	0	4.00	4.18	4.20	4.59	4.22	4.22	4.53	4.70	3.98	4.09	4.14	4.34	4.11	4.09	4.23	4.43	4.03	4.18	4.21	4.31
1368134_a_at	Il4r	interleukin 4 receptor	1	uniqDRlyp/lyp_119	1	0	0	0	0	0	0	0	0	0	5.48	5.60	5.52	6.05	5.60	5.43	5.49	6.05	5.60	5.63	5.56	5.65	5.56	5.61	5.52	5.61	5.43	5.69	5.61	5.68
1368227_at	Slc28a2	solute carrier family 28 (sodium-coupled nucleoside transporter), member 2	1	uniqDRlyp/lyp_119	1	0	0	0	0	0	0	0	0	0	5.86	6.09	6.27	6.52	5.97	6.00	6.16	6.47	5.36	5.55	5.58	5.68	5.78	5.60	5.66	6.04	5.94	5.79	6.17	6.12
1368377_at	Gzmc /// LOC	granzyme C /// granzyme C-like /// similar to granzyme C /// similar to granzyme C	1	uniqDRlyp/lyp_119	1	0	0	0	0	0	0	0	0	0	3.94	4.10	3.90	4.86	3.96	4.00	4.08	3.73	4.00	3.94	3.96	3.99	4.02	3.96	3.91	3.87	4.02	3.82	4.03	3.79
1368448_at	Ltbp2	latent transforming growth factor beta binding protein 2	1	uniqDRlyp/lyp_119	1	0	0	0	0	0	0	0	0	0	5.51	5.73	5.63	6.01	5.51	6.09	5.94	5.87	5.53	5.48	5.62	5.85	5.55	5.57	5.76	5.84	5.42	5.71	5.68	5.69
1368455_at	Nkg7	natural killer cell group 7 sequence	1	uniqDRlyp/lyp_119	1	0	0	0	0	0	0	0	0	0	3.57	3.51	3.58	4.44	3.34	3.49	3.56	3.62	3.42	3.57	3.51	3.70	3.61	3.60	3.73	4.09	3.46	3.68	3.78	3.66
1368464_at	Clec10a	C-type lectin domain family 10, member A	1	uniqDRlyp/lyp_119	1	0	0	0	0	0	0	0	0	0	4.44	4.60	4.53	5.56	4.34	4.58	4.54	4.64	4.53	4.87	4.66	4.99	4.91	4.86	4.97	4.85	4.75	4.91	4.98	4.72
1368675_at	Chn2	chimerin (chimaerin) 2	1	uniqDRlyp/lyp_119	1	0	0	0	0	0	0	0	0	0	3.97	4.07	4.38	4.53	4.08	4.39	4.30	4.33	3.89	4.13	4.19	4.33	4.23	4.29	4.27	4.27	4.07	4.21	4.13	4.36
1368693_at	Fgr	Gardner-Rasheed feline sarcoma viral (v-fgr) oncogene homolog	1	uniqDRlyp/lyp_119	1	0	0	0	0	0	0	0	0	0	3.28	3.26	3.39	4.09	3.20	3.44	3.55	3.64	3.18	3.22	3.37	3.43	3.09	3.24	3.32	3.44	3.17	3.28	3.36	3.40
1368815_at	Mpz	myelin protein zero	1	uniqDRlyp/lyp_119	1	0	0	0	0	0	0	0	0	0	2.92	3.11	2.90	3.25	3.01	2.97	3.15	3.17	3.13	3.19	3.03	3.14	3.03	3.00	3.13	3.03	3.06	3.00	2.96	3.07
1368816_at	Mpz	myelin protein zero	1	uniqDRlyp/lyp_119	1	0	0	0	0	0	0	0	0	0	2.60	2.85	3.01	3.45	2.77	2.82	3.09	3.44	2.68	2.95	2.98	3.29	2.91	2.87	2.92	2.84	2.88	2.83	2.89	2.98
1368835_at	Stat1	signal transducer and activator of transcription 1	1	uniqDRlyp/lyp_119	1	0	0	0	0	0	0	0	0	0	8.68	8.62	8.61	9.87	8.58	8.58	8.57	8.80	8.60	8.68	8.72	8.72	8.41	8.75	8.87	8.74	8.54	8.70	8.79	8.76
1369036_at	Grik2	glutamate receptor, ionotropic, kainate 2	1	uniqDRlyp/lyp_119	1	0	0	0	0	0	0	0	0	0	3.48	3.68	4.13	3.92	3.78	3.73	3.87	3.87	3.67	3.93	3.91	3.83	3.68	3.91	3.79	3.97	3.79	3.82	4.01	3.89
1369048_at	Gabrd	gamma-aminobutyric acid (GABA) A receptor, delta	1	uniqDRlyp/lyp_119	1	0	0	0	0	0	0	0	0	0	6.24	6.85	7.21	6.97	6.09	6.98	7.05	6.66	6.07	6.69	6.63	6.76	5.74	6.75	6.88	6.70	6.14	6.55	6.69	6.57
1369177_at	Pi4k2a	phosphatidylinositol 4-kinase type 2 alpha	1	uniqDRlyp/lyp_119	1	0	0	0	0	0	0	0	0	0	5.66	5.64	5.72	6.19	5.96	5.75	5.62	5.87	6.03	5.85	6.04	6.17	5.13	5.78	5.89	6.02	5.90	5.86	5.99	5.81
1369202_at	Mx2	myxovirus (influenza virus) resistance 2	1	uniqDRlyp/lyp_119	1	0	0	0	0	0	0	0	0	0	2.40	2.49	2.58	2.57	2.56	2.49	2.47	2.45	2.55	2.49	2.63	2.66	2.51	2.44	2.53	2.57	2.49	2.47	2.43	2.55
1369290_at	Ccr5	chemokine (C-C motif) receptor 5	1	uniqDRlyp/lyp_119	1	0	0	0	0	0	0	0	0	0	2.15	2.14	2.25	3.09	2.19	2.31	2.13	2.58	2.08	2.09	2.22	2.38	2.16	2.19	2.23	2.45	2.17	2.26	2.05	2.40
1369497_at	LOC24906	RoBo-1	1	uniqDRlyp/lyp_119	1	0	0	0	0	0	0	0	0	0	2.56	2.65	2.73	3.39	2.73	2.89	2.67	2.92	2.54	2.66	2.67	2.86	2.63	2.65	2.62	2.99	2.68	2.58	2.63	2.69
1369520_a_at	Bcat1	branched chain amino acid transaminase 1, cytosolic	1	uniqDRlyp/lyp_119	1	0	0	0	0	0	0	0	0	0	5.79	5.83	5.61	5.78	5.59	5.55	5.63	5.55	5.72	5.56	5.64	5.69	5.59	5.70	5.65	5.62	5.66	5.68	5.74	5.62
1369726_at	Tapbp	TAP binding protein (tapasin)	1	uniqDRlyp/lyp_119	1	0	0	0	0	0	0	0	0	0	6.48	6.68	6.69	7.24	6.36	6.47	6.64	6.81	6.43	6.54	6.52	6.54	6.61	7.16	7.01	6.96	6.32	6.50	6.46	6.40
1370332_at	Unc13d	unc-13 homolog D (C. elegans)	1	uniqDRlyp/lyp_119	1	0	0	0	0	0	0	0	0	0	4.47	4.80	4.91	5.15	4.89	4.98	4.82	5.27	4.72	4.84	4.86	4.98	4.73	4.74	4.84	4.89	4.86	4.81	4.95	4.94
1370369_at	Gzmm	granzyme M (lymphocyte met-ase 1)	1	uniqDRlyp/lyp_119	1	0	0	0	0	0	0	0	0	0	4.79	4.66	4.76	5.22	4.81	4.76	4.98	4.87	4.82	4.78	5.08	5.11	4.77	4.89	4.99	5.36	4.85	4.85	5.01	4.96
1370628_at	Gzmb /// Gzm	granzyme B (granzyme 2, cytotoxic T-lymphocyte-associated serine esterase 1) /// Granzyme	1	uniqDRlyp/lyp_119	1	0	0	0	0	0	0	0	0	0	2.72	2.72	2.76	5.48	2.79	2.73	2.83	2.91	2.65	2.75	2.75	3.13	2.69	2.67	2.86	3.00	2.64	2.72	2.85	2.89
1370790_at	Ifng	interferon gamma	1	uniqDRlyp/lyp_119	1	0	0	0	0	0	0	0	0	0	2.07	2.10	2.22	2.91	2.19	2.19	2.15	2.21	2.16	2.03	2.02	2.17	2.07	2.19	2.27	2.17	2.12	2.24	2.15	2.15
1370869_at	Bcat1	branched chain amino acid transaminase 1, cytosolic	1	uniqDRlyp/lyp_119	1	0	0	0	0	0	0	0	0	0	2.94	3.08	3.28	4.18	3.15	3.21	3.45	4.19	2.87	4.56	3.24	3.39	2.93	3.49	3.21	3.32	3.05	3.24	3.24	3.56
1370913_at	Rsad2	radical S-adenosyl methionine domain containing 2	1	uniqDRlyp/lyp_119	1	0	0	0	0	0	0	0	0	0	6.26	6.28	6.11	7.68	6.30	6.35	5.90	5.90	6.36	6.12	5.88	6.17	6.04	6.06	6.14	6.00	6.20	5.95	6.00	5.96
1370964_at	Ass1	argininosuccinate synthase 1	1	uniqDRlyp/lyp_119	1	0	0	0	0	0	0	0	0	0	5.95	6.29	6.45	6.38	5.95	6.29	6.31	6.10	5.90	6.17	6.07	6.23	5.58	6.26	6.24	6.17	5.86	6.01	6.15	6.09
1371446_at	Mapkapk2	mitogen-activated protein kinase-activated protein kinase 2	1	uniqDRlyp/lyp_119	1	0	0	0	0	0	0	0	0	0	8.08	8.24	8.30	8.61	8.09	8.24	8.44	8.58	8.14	8.29	8.50	8.53	8.21	8.46	8.32	8.53	8.17	8.64	8.64	8.60
1371677_at	Spink8	serine peptidase inhibitor, Kazal type 8	1	uniqDRlyp/lyp_119	1	0	0	0	0	0	0	0	0	0	3.28	3.70	3.57	4.01	3.36	3.56	3.39	3.70	3.19	3.27	3.13	3.51	3.11	3.23	3.36	3.37	2.99	3.42	3.27	3.52
1372757_at	Stat1	signal transducer and activator of transcription 1	1	uniqDRlyp/lyp_119	1	0	0	0	0	0	0	0	0	0	9.12	9.13	9.27	10.24	9.17	9.15	9.21	9.29	9.14	9.29	9.32	9.31	9.08	9.20	9.38	9.34	9.12	9.22	9.31	9.32
1373037_at	Ube2l6	ubiquitin-conjugating enzyme E2L 6	1	uniqDRlyp/lyp_119	1	0	0	0	0	0	0	0	0	0	6.46	6.63	6.67	7.58	6.44	6.69	6.57	6.65	6.39	6.66	6.72	6.76	6.27	6.60	6.85	6.78	6.48	6.59	6.76	6.77
1373514_at	Rnf213	ring finger protein 213	1	uniqDRlyp/lyp_119	1	0	0	0	0	0	0	0	0	0	5.77	5.98	5.99	6.98	5.89	5.90	5.96	6.15	5.88	6.03	5.85	5.98	6.08	5.90	6.00	6.19	5.97	5.92	6.09	5.95
1373523_at	Fcgr3a	Fc fragment of IgG, low affinity IIIa, receptor	1	uniqDRlyp/lyp_119	1	0	0	0	0	0	0	0	0	0	4.93	5.00	5.35	6.70	5.06	5.12	5.26	5.41	5.01	4.98	5.07	5.36	4.94	5.02	5.35	5.51	4.81	5.12	5.21	5.28
1373544_at	Cxcl9	chemokine (C-X-C motif) ligand 9	1	uniqDRlyp/lyp_119	1	0	0	0	0	0	0	0	0	0	2.78	2.74	2.88	8.00	2.77	2.67	2.79	3.16	2.91	2.61	2.76	2.89	2.67	2.60	2.97	2.84	2.92	2.65	2.56	2.82
1373657_at	Slc31a2	solute carrier family 31 (copper transporters), member 2	1	uniqDRlyp/lyp_119	1	0	0	0	0	0	0	0	0	0	8.03	8.31	8.42	8.38	8.09	8.40	8.29	8.30	8.45	8.49	8.51	8.54	8.21	8.30	8.63	8.64	8.20	8.39	8.45	8.51
1373670_at	---	---	1	uniqDRlyp/lyp_119	1	0	0	0	0	0	0	0	0	0	6.13	6.06	6.11	6.97	6.12	6.09	6.09	6.25	6.18	6.24	6.14	6.17	6.25	6.17	6.16	6.30	6.10	6.13	6.21	6.24
1374070_at	Gpx2	glutathione peroxidase 2	1	uniqDRlyp/lyp_119	1	0	0	0	0	0	0	0	0	0	4.49	4.51	4.52	5.00	4.69	4.45	5.88	5.23	4.80	5.31	5.09	5.36	4.75	4.76	5.42	5.58	5.03	4.97	5.29	5.10
1374337_at	---	---	1	uniqDRlyp/lyp_119	1	0	0	0	0	0	0	0	0	0	6.20	6.11	6.18	6.96	6.17	6.12	6.21	6.21	6.08	6.22	6.11	6.22	6.33	6.18	6.15	6.33	6.06	6.07	6.19	6.16
1374718_at	Dtx3l	deltex 3-like (Drosophila)	1	uniqDRlyp/lyp_119	1	0	0	0	0	0	0	0	0	0	6.95	7.06	7.08	7.95	6.95	6.95	6.95	7.07	6.88	6.86	6.72	6.71	7.19	6.92	6.89	6.87	6.75	6.71	6.79	6.93
1374870_at	---	---	1	uniqDRlyp/lyp_119	1	0	0	0	0	0	0	0	0	0	8.99	9.21	9.21	9.62	9.12	9.12	9.32	9.30	9.14	9.27	9.44	9.60	9.18	9.22	9.25	9.67	9.18	9.24	9.47	9.62
1375003_at	Serpinb6	serpin peptidase inhibitor, clade B (ovalbumin), member 6	1	uniqDRlyp/lyp_119	1	0	0	0	0	0	0	0	0	0	7.71	8.00	8.22	8.07	7.66	7.96	8.15	8.03	8.22	8.42	8.57	8.65	8.29	8.40	8.55	8.56	8.21	8.33	8.45	8.61
1375010_at	Cd68	Cd68 molecule	1	uniqDRlyp/lyp_119	1	0	0	0	0	0	0	0	0	0	4.42	4.51	4.49	5.54	4.54	4.77	4.41	5.39	4.48	4.63	4.66	4.62	4.70	4.38	4.65	5.09	4.43	4.55	4.46	4.44
1375041_at	RGD1563982	similar to F-box only protein 27	1	uniqDRlyp/lyp_119	1	0	0	0	0	0	0	0	0	0	6.15	6.37	6.39	6.78	6.10	6.41	6.39	6.28	6.16	6.41	6.41	6.43	6.13	6.35	6.51	6.39	6.16	6.35	6.49	6.54
1375362_at	Sppl2a	signal peptide peptidase-like 2A	1	uniqDRlyp/lyp_119	1	0	0	0	0	0	0	0	0	0	7.57	7.87	7.97	8.08	7.58	7.68	7.81	7.97	7.71	7.88	7.85	8.02	7.44	7.84	7.85	8.16	7.73	7.84	7.68	7.89
1375379_at	---	---	1	uniqDRlyp/lyp_119	1	0	0	0	0	0	0	0	0	0	2.39	2.40	2.57	2.59	2.62	2.60	2.48	2.66	2.48	2.51	2.48	2.50	2.40	2.35	2.54	2.47	2.50	2.57	2.47	2.52
1375708_at	Col27a1	collagen, type XXVII, alpha 1	1	uniqDRlyp/lyp_119	1	0	0	0	0	0	0	0	0	0	8.50	8.64	8.61	9.09	8.57	8.50	8.70	8.74	8.74	8.76	8.92	9.08	8.73	8.61	8.70	9.08	8.71	8.73	9.04	9.18
1375796_at	Igtp /// Irgm2	interferon gamma induced GTPase /// immunity-related GTPase family M member 2	1	uniqDRlyp/lyp_119	1	0	0	0	0	0	0	0	0	0	3.82	4.10	3.87	5.90	4.09	3.79	3.86	4.16	3.98	3.99	3.96	3.96	4.01	4.01	4.22	4.26	3.94	3.83	3.94	4.08
1375917_at	Lilrb4	leukocyte immunoglobulin-like receptor, subfamily B, member 4	1	uniqDRlyp/lyp_119	1	0	0	0	0	0	0	0	0	0	2.72	2.65	2.84	3.47	2.71	2.74	2.73	2.97	2.64	2.80	2.88	3.05	2.98	2.65	3.03	2.97	2.59	2.61	2.86	2.86
1376144_at	Parp9	poly (ADP-ribose) polymerase family, member 9	1	uniqDRlyp/lyp_119	1	0	0	0	0	0	0	0	0	0	6.69	6.68	6.72	7.78	6.74	6.55	6.71	6.99	6.33	6.54	6.41	6.42	6.54	6.44	6.60	6.69	6.33	6.28	6.57	6.53
1376151_a_at	Irgm2	immunity-related GTPase family M member 2	1	uniqDRlyp/lyp_119	1	0	0	0	0	0	0	0	0	0	8.40	8.41	8.49	10.00	8.45	8.38	8.44	8.42	8.41	8.39	8.32	8.31	8.56	8.30	8.44	8.40	8.43	8.17	8.33	8.32
1376390_at	Ms4a11	membrane-spanning 4-domains, subfamily A, member 11	1	uniqDRlyp/lyp_119	1	0	0	0	0	0	0	0	0	0	2.66	2.86	2.79	4.56	2.68	2.69	2.89	3.24	3.04	2.93	2.76	2.89	2.79	2.78	3.31	3.48	2.88	2.93	2.79	2.85
1376394_at	Clec9a	C-type lectin domain family 9, member A	1	uniqDRlyp/lyp_119	1	0	0	0	0	0	0	0	0	0	2.50	2.73	2.69	3.62	2.60	2.70	2.72	2.99	2.52	2.69	2.69	2.79	2.65	2.73	3.03	3.48	2.60	2.61	2.79	2.92
1376693_at	---	---	1	uniqDRlyp/lyp_119	1	0	0	0	0	0	0	0	0	0	5.23	5.24	5.46	7.84	5.15	4.99	5.08	5.67	4.45	5.02	4.87	5.00	4.92	4.92	5.16	5.50	4.62	4.72	5.09	5.03
1376845_at	Ifi27l2b	interferon, alpha-inducible protein 27 like 2B	1	uniqDRlyp/lyp_119	1	0	0	0	0	0	0	0	0	0	8.45	8.71	8.85	9.52	8.56	8.89	8.70	8.89	8.37	8.87	8.55	8.70	8.37	9.03	9.01	9.24	8.49	8.78	8.64	8.61
1376920_at	LOC500013	similar to sterile alpha motif domain containing 9-like	1	uniqDRlyp/lyp_119	1	0	0	0	0	0	0	0	0	0	5.30	5.36	5.54	6.35	5.20	5.20	5.39	5.63	4.96	5.16	5.09	5.31	5.10	5.09	5.21	5.39	4.98	4.92	5.21	5.29
1377060_at	Mccc2	methylcrotonoyl-CoA carboxylase 2 (beta)	1	uniqDRlyp/lyp_119	1	0	0	0	0	0	0	0	0	0	7.18	7.59	7.73	7.64	7.41	7.50	7.57	7.69	7.47	7.67	7.71	7.84	7.60	7.68	7.77	7.86	7.43	7.64	7.85	7.88
1377116_at	Rnasel	ribonuclease L (2',5'-oligoisoadenylate synthetase-dependent)	1	uniqDRlyp/lyp_119	1	0	0	0	0	0	0	0	0	0	7.57	7.99	8.03	8.05	7.66	7.91	7.97	7.84	7.87	8.07	7.93	7.87	7.86	7.99	8.07	7.95	7.70	7.86	7.80	7.84
1377662_at	Pir	pirin (iron-binding nuclear protein)	1	uniqDRlyp/lyp_119	1	0	0	0	0	0	0	0	0	0	5.13	5.31	5.64	5.49	5.13	5.28	5.51	5.39	5.36	5.50	5.58	5.74	5.34	5.40	5.55	5.71	5.64	5.28	5.61	5.87
1378043_at	---	---	1	uniqDRlyp/lyp_119	1	0	0	0	0	0	0	0	0	0	2.44	2.17	2.14	2.47	2.30	2.43	2.40	2.31	2.48	2.23	2.35	2.32	2.29	2.30	2.28	2.25	2.17	2.34	2.40	2.42
1378193_at	Ms4a7	membrane-spanning 4-domains, subfamily A, member 7	1	uniqDRlyp/lyp_119	1	0	0	0	0	0	0	0	0	0	3.58	3.66	3.67	5.20	3.74	3.70	3.90	3.99	3.58	3.69	3.94	3.98	3.75	3.82	4.14	4.39	3.60	3.73	3.97	3.83
1378443_at	Slamf9	SLAM family member 9	1	uniqDRlyp/lyp_119	1	0	0	0	0	0	0	0	0	0	4.60	4.69	4.84	5.29	4.50	4.68	4.79	4.89	4.45	4.60	4.66	4.87	4.60	4.72	4.86	5.03	4.54	4.73	4.88	4.76
1378640_at	Uhrf1	ubiquitin-like with PHD and ring finger domains 1	1	uniqDRlyp/lyp_119	1	0	0	0	0	0	0	0	0	0	2.72	3.14	3.07	3.30	3.08	3.08	2.90	3.67	3.11	3.15	3.33	3.17	3.19	3.35	3.17	3.24	3.03	3.41	3.22	3.32
1379293_at	Gzma	granzyme A	1	uniqDRlyp/lyp_119	1	0	0	0	0	0	0	0	0	0	2.31	2.41	2.45	4.07	2.56	2.43	2.45	2.57	2.39	2.38	2.43	2.61	2.60	2.49	2.53	2.68	2.27	2.37	2.65	2.56
1379365_at	Cxcl11	chemokine (C-X-C motif) ligand 11	1	uniqDRlyp/lyp_119	1	0	0	0	0	0	0	0	0	0	7.52	7.64	7.80	10.50	7.78	7.46	7.71	7.77	7.82	7.78	7.91	7.97	8.00	7.74	7.93	7.91	7.81	7.55	7.75	7.72
1379633_a_at	Uba7	ubiquitin-like modifier activating enzyme 7	1	uniqDRlyp/lyp_119	1	0	0	0	0	0	0	0	0	0	4.91	5.01	5.14	5.89	4.84	4.86	5.00	5.11	4.62	4.78	4.73	4.86	4.94	4.72	4.65	4.91	4.62	4.58	4.74	4.86

Affymetrix Probeset ID	Gene Symbol	Gene Name	unique BB on P7 n=822	Figure 1B P7 Venn n=1526	DRlyp/lyp STEM P7 n=712	DR+/+ STEM P7 n=936	Flyp/lyp STEM P7 n=520	F+/+ STEM P7 n=465	Figure 1B P0 Venn n=1071	DRlyp/lyp STEM P0 n=442	DR+/+ STEM P0 n=437	Flyp/lyp STEM P0 n=643	F+/+ STEM P0 n=632	Fig1D n=25	Mean log2 intensity DRlyp/lyp 20	Mean log2 intensity DRlyp/lyp 30	Mean log2 intensity DRlyp/lyp 40	Mean log2 intensity DRlyp/lyp 50	Mean log2 intensity DR+/+ 20	Mean log2 intensity DR+/+ 30	Mean log2 intensity DR+/+ 40	Mean log2 intensity DR+/+ 50	Mean log2 intensity Flyp/lyp 20	Mean log2 intensity Flyp/lyp 30	Mean log2 intensity Flyp/lyp 40	Mean log2 intensity Flyp/lyp 50	Mean log2 intensity Flyp20	Mean log2 intensity Flyp30	Mean log2 intensity Flyp40	Mean log2 intensity Flyp50	Mean log2 intensity F+/+ 20	Mean log2 intensity F+/+ 30	Mean log2 intensity F+/+ 40	Mean log2 intensity F+/+ 50
1379634_at	Uba7	ubiquitin-like modifier activating enzyme 7	1	uniqDRlyp/lyp_119	1	0	0	0	0	0	0	0	0	0	4.17	4.30	4.32	4.55	4.21	4.46	4.50	4.51	4.15	4.24	4.39	4.70	4.13	4.30	4.49	4.45	4.24	4.51	4.35	4.66
1379717_at	Abcg3l4	ATP-binding cassette, subfamily G (WHITE), member 3-like 4	1	uniqDRlyp/lyp_119	1	0	0	0	0	0	0	0	0	0	7.85	8.02	8.18	8.71	7.80	8.00	8.16	8.25	7.88	8.06	8.10	8.19	7.83	8.09	8.17	8.20	7.84	7.95	8.14	8.20
1379794_at	Gzmb /// Gzn	granzyme B (granzyme 2, cytotoxic T-lymphocyte-associated serine esterase 1) /// Granzyme	1	uniqDRlyp/lyp_119	1	0	0	0	0	0	0	0	0	0	1.77	1.72	1.83	4.06	1.86	1.76	1.77	1.81	1.86	1.95	1.90	2.17	1.94	1.95	1.85	2.09	1.83	1.89	2.08	2.12
1380425_at	---	---	1	uniqDRlyp/lyp_119	1	0	0	0	0	0	0	0	0	0	6.75	7.38	7.41	7.32	6.79	7.32	7.41	7.20	7.09	7.43	7.22	7.16	7.03	7.32	7.36	7.30	6.76	7.12	7.19	7.07
1381014_at	Ifi44	interferon-induced protein 44	1	uniqDRlyp/lyp_119	1	0	0	0	0	0	0	0	0	0	5.10	5.01	5.04	6.27	5.13	5.06	5.03	5.07	4.96	5.01	4.95	5.00	5.04	5.02	5.23	5.16	4.89	4.84	4.91	4.94
1381849_at	Tulp3	tubby-like protein 3	1	uniqDRlyp/lyp_119	1	0	0	0	0	0	0	0	0	0	4.47	4.66	4.71	4.98	4.82	4.96	5.02	4.85	4.46	4.59	4.78	4.91	4.41	4.73	4.82	4.80	4.55	4.79	4.66	4.90
1382074_at	---	---	1	uniqDRlyp/lyp_119	1	0	0	0	0	0	0	0	0	0	6.63	6.75	6.80	7.21	6.70	6.67	6.66	6.95	6.60	6.75	6.84	6.83	6.71	6.75	6.85	6.90	6.73	6.84	6.95	6.89
1382153_at	Clec4a	C-type lectin domain family 4, member A	1	uniqDRlyp/lyp_119	1	0	0	0	0	0	0	0	0	0	2.62	2.47	2.83	2.98	2.68	2.65	2.53	2.96	2.47	2.52	2.85	2.78	2.55	2.68	2.89	2.96	2.66	2.70	2.73	2.77
1382454_at	Cxcl9	chemokine (C-X-C motif) ligand 9	1	uniqDRlyp/lyp_119	1	0	0	0	0	0	0	0	0	0	2.84	2.87	3.08	7.82	3.14	3.10	3.06	3.56	2.83	3.06	3.15	3.12	3.05	3.07	3.22	3.31	3.14	2.84	3.13	3.06
1382680_at	---	---	1	uniqDRlyp/lyp_119	1	0	0	0	0	0	0	0	0	0	6.27	6.67	6.89	7.20	6.41	6.71	6.72	7.10	6.13	6.45	6.59	6.81	5.97	6.41	6.65	6.89	6.09	6.48	6.67	7.01
1383323_at	Dsn1	DSN1, MIND kinetochore complex component, homolog (S. cerevisiae)	1	uniqDRlyp/lyp_119	1	0	0	0	0	0	0	0	0	0	3.61	3.75	3.74	4.12	3.74	3.92	3.94	4.15	3.68	3.66	3.92	3.87	3.63	3.64	3.69	3.70	3.75	3.71	3.91	3.85
1383424_at	Cmpk2	cytidine monophosphate (UMP-CMP) kinase 2, mitochondrial	1	uniqDRlyp/lyp_119	1	0	0	0	0	0	0	0	0	0	7.60	7.56	7.73	8.22	7.63	7.63	7.59	7.46	7.31	7.33	7.34	7.42	7.56	7.45	7.47	7.45	7.33	7.29	7.29	7.32
1383448_at	Irf9	interferon regulatory factor 9	1	uniqDRlyp/lyp_119	1	0	0	0	0	0	0	0	0	0	5.98	6.04	6.18	7.05	6.09	6.00	6.05	6.24	6.23	6.35	6.18	6.31	6.40	6.25	6.48	6.53	6.18	6.05	6.07	6.22
1383614_at	LOC1009122(	NUAK family SNF1-like kinase 2-like /// NUAK family, SNF1-like kinase, 2	1	uniqDRlyp/lyp_119	1	0	0	0	0	0	0	0	0	0	4.82	5.13	5.10	5.34	5.01	5.03	5.19	5.15	5.03	5.32	5.12	5.17	5.39	5.06	5.07	5.16	5.18	4.93	5.20	5.13
1383684_at	Asf1b	ASF1 anti-silencing function 1 homolog B (S. cerevisiae)	1	uniqDRlyp/lyp_119	1	0	0	0	0	0	0	0	0	0	3.13	3.34	3.24	3.75	3.51	3.59	3.46	3.90	3.15	3.62	3.74	3.41	3.37	3.18	3.52	3.42	3.21	3.46	3.60	3.56
1384939_at	---	---	1	uniqDRlyp/lyp_119	1	0	0	0	0	0	0	0	0	0	2.40	2.64	2.59	3.24	2.55	2.60	2.61	2.95	2.45	2.37	2.41	2.46	2.40	2.41	2.47	2.75	2.56	2.25	2.39	2.37
1385062_at	RGD1306072	hypothetical LOC304654	1	uniqDRlyp/lyp_119	1	0	0	0	0	0	0	0	0	0	4.07	4.43	4.48	4.63	4.31	4.69	4.57	4.46	4.17	4.43	4.64	4.55	4.27	4.48	4.51	4.62	4.35	4.54	4.53	4.48
1385551_at	RGD1562652	similar to class I histocompatibility antigen alpha chain - cotton-top tamarin	1	uniqDRlyp/lyp_119	1	0	0	0	0	0	0	0	0	0	6.35	6.55	6.78	6.98	6.26	6.56	6.57	6.49	6.43	6.46	6.48	6.58	6.50	6.57	6.65	6.60	6.33	6.45	6.51	6.46
1386009_at	Csf3r	colony stimulating factor 3 receptor (granulocyte)	1	uniqDRlyp/lyp_119	1	0	0	0	0	0	0	0	0	0	3.25	3.35	3.40	3.83	3.54	3.41	3.45	3.66	3.08	3.44	3.27	3.58	3.21	3.35	3.20	3.56	3.11	3.47	3.49	3.49
1386239_at	Dmrta1	DMRT-like family A1	1	uniqDRlyp/lyp_119	1	0	0	0	0	0	0	0	0	0	5.09	5.56	5.65	5.69	5.33	5.36	5.45	5.65	5.32	5.40	5.51	5.77	5.28	5.24	5.55	5.94	5.21	5.32	5.50	5.63
1386277_at	Uba7	ubiquitin-like modifier activating enzyme 7	1	uniqDRlyp/lyp_119	1	0	0	0	0	0	0	0	0	0	5.59	5.63	5.66	6.26	5.64	5.82	5.67	5.80	5.42	5.53	5.55	5.63	5.42	5.51	5.39	5.66	5.50	5.50	5.59	5.56
1387027_a_at	Lgals9	lectin, galactoside-binding, soluble, 9	1	uniqDRlyp/lyp_119	1	0	0	0	0	0	0	0	0	0	4.14	4.21	4.49	4.94	4.35	4.25	4.38	4.70	4.24	4.45	4.25	4.52	4.31	4.44	4.41	4.61	4.27	4.08	4.25	4.43
1387205_at	RT1-M3-1	RT1 class Ib, locus M3, gene 1	1	uniqDRlyp/lyp_119	1	0	0	0	0	0	0	0	0	0	7.86	8.07	8.15	8.49	7.88	8.14	8.25	8.25	7.97	8.26	8.32	8.43	7.87	8.33	8.37	8.33	7.98	8.18	8.41	8.35
1387283_at	Mx2	myxovirus (influenza virus) resistance 2	1	uniqDRlyp/lyp_119	1	0	0	0	0	0	0	0	0	0	3.43	3.33	3.50	4.41	3.16	3.33	3.38	3.74	3.48	3.56	3.40	3.67	3.40	3.59	3.65	4.09	3.36	3.46	3.65	3.86
1387354_at	Stat1	signal transducer and activator of transcription 1	1	uniqDRlyp/lyp_119	1	0	0	0	0	0	0	0	0	0	7.43	7.41	7.55	9.09	7.51	7.38	7.51	7.76	7.44	7.52	7.60	7.73	7.06	7.57	7.77	7.66	7.46	7.57	7.57	7.53
1387378_at	Fcnb	ficolin B	1	uniqDRlyp/lyp_119	1	0	0	0	0	0	0	0	0	0	4.61	4.75	4.70	5.17	4.79	4.67	4.81	4.83	4.60	4.68	4.84	4.78	4.80	4.77	4.85	4.90	4.59	4.83	4.73	4.52
1388034_at	Apitd1 /// Co	apoptosis-inducing, TAF9-like domain 1 /// cortistatin /// kinesin family member 1B /// 6-phc	1	uniqDRlyp/lyp_119	1	0	0	0	0	0	0	0	0	0	5.98	6.15	6.12	6.33	6.00	6.08	6.18	6.26	5.79	6.06	6.07	6.17	5.74	5.90	5.97	6.19	5.90	5.87	6.31	5.81
1388149_at	Tap1	transporter 1, ATP-binding cassette, sub-family B (MDR/TAP)	1	uniqDRlyp/lyp_119	1	0	0	0	0	0	0	0	0	0	6.43	6.50	6.66	8.83	6.38	6.46	6.65	6.98	6.36	6.61	6.58	6.56	6.34	6.44	6.42	6.50	6.29	6.51	6.57	6.57
1388276_at	LOC679994	similar to histone 1, H2ai	1	uniqDRlyp/lyp_119	1	0	0	0	0	0	0	0	0	0	4.36	4.39	4.55	4.98	4.36	4.12	5.36	4.88	4.20	4.41	4.54	4.67	4.10	4.67	4.62	4.64	4.54	4.45	4.54	4.42
1388574_at	Wars	tryptophanyl-tRNA synthetase	1	uniqDRlyp/lyp_119	1	0	0	0	0	0	0	0	0	0	7.83	7.89	8.01	8.84	7.88	7.84	7.94	8.05	8.00	8.03	8.13	8.21	8.07	8.07	8.12	8.21	7.97	8.12	8.22	8.35
1389470_at	C2 /// Cfb	complement component 2 /// complement factor B	1	uniqDRlyp/lyp_119	1	0	0	0	0	0	0	0	0	0	3.64	3.77	3.93	4.63	3.83	3.83	4.09	4.16	3.55	3.67	3.77	3.87	3.61	3.64	4.22	3.89	3.71	3.88	3.84	3.96
1389571_at	Stat2	signal transducer and activator of transcription 2	1	uniqDRlyp/lyp_119	1	0	0	0	0	0	0	0	0	0	6.69	6.64	6.54	7.72	6.63	6.58	6.66	7.16	6.66	6.73	6.77	6.88	6.65	6.79	6.80	6.96	6.70	6.70	6.91	6.97
1390097_at	Tspyl4	TSPY-like 4	1	uniqDRlyp/lyp_119	1	0	0	0	0	0	0	0	0	0	6.46	7.89	7.71	7.88	8.02	6.62	7.71	7.78	8.25	8.34	8.32	8.34	7.05	8.11	8.15	8.50	8.10	8.28	8.37	7.99
1390312_at	LOC500013 /,	similar to sterile alpha motif domain containing 9-like /// similar to sterile alpha motif domai	1	uniqDRlyp/lyp_119	1	0	0	0	0	0	0	0	0	0	5.86	5.95	5.84	6.78	5.78	5.91	5.82	6.18	5.54	5.56	5.65	5.87	5.65	5.72	5.93	6.04	5.66	5.67	5.84	5.88
1390383_at	Plin2	perilipin 2	1	uniqDRlyp/lyp_119	1	0	0	0	0	0	0	0	0	0	8.32	8.51	8.58	8.85	8.43	8.52	8.61	8.66	8.19	8.34	8.40	8.57	8.09	8.28	8.58	8.57	8.16	8.37	8.49	8.60
1390850_at	---	---	1	uniqDRlyp/lyp_119	1	0	0	0	0	0	0	0	0	0	3.52	3.53	3.51	3.50	3.50	3.37	3.59	3.61	3.30	3.29	3.50	3.36	3.50	3.52	3.61	3.56	3.29	3.53	3.59	3.56
1391463_at	Ddx58	DEAD (Asp-Glu-Ala-Asp) box polypeptide 58	1	uniqDRlyp/lyp_119	1	0	0	0	0	0	0	0	0	0	6.18	6.27	6.43	6.73	6.13	6.21	6.27	6.39	6.45	6.44	6.42	6.59	6.58	6.48	6.59	6.73	6.41	6.46	6.65	6.71
1391489_at	Irgm	immunity-related GTPase family, M	1	uniqDRlyp/lyp_119	1	0	0	0	0	0	0	0	0	0	5.86	5.70	6.02	7.84	5.89	5.91	5.91	5.85	4.92	4.92	4.87	5.17	5.20	5.10	5.14	5.03	5.27	5.05	5.33	5.26
1391906_at	Apitd1 /// Co	apoptosis-inducing, TAF9-like domain 1 /// cortistatin /// kinesin family member 1B /// 6-phc	1	uniqDRlyp/lyp_119	1	0	0	0	0	0	0	0	0	0	6.44	6.75	6.68	7.12	6.60	6.66	6.83	6.97	6.42	6.62	6.65	6.57	6.06	6.49	6.43	6.79	6.45	6.45	6.77	6.35
1392801_at	RGD1561145	similar to novel protein	1	uniqDRlyp/lyp_119	1	0	0	0	0	0	0	0	0	0	4.06	3.90	4.10	4.46	4.32	4.29	4.11	4.55	4.21	4.23	4.23	4.27	4.11	4.35	4.33	4.39	4.04	4.48	4.30	4.53
1392819_at	Ms4a11	membrane-spanning 4-domains, subfamily A, member 11	1	uniqDRlyp/lyp_119	1	0	0	0	0	0	0	0	0	0	4.85	4.87	4.90	5.07	4.81	5.01	5.01	5.11	4.81	4.75	4.76	4.92	4.88	4.79	4.97	5.16	4.83	4.95	4.94	5.00
1393038_at	Fcgr1a	Fc fragment of IgG, high affinity Ia, receptor (CD64)	1	uniqDRlyp/lyp_119	1	0	0	0	0	0	0	0	0	0	4.51	4.75	4.92	5.41	4.61	4.77	4.90	5.11	4.63	4.66	4.85	4.94	4.48	4.66	4.99	5.10	4.72	4.72	4.97	4.97
1393044_at	Cmpk2	cytidine monophosphate (UMP-CMP) kinase 2, mitochondrial	1	uniqDRlyp/lyp_119	1	0	0	0	0	0	0	0	0	0	5.17	5.28	5.28	5.56	5.29	5.42	5.15	5.09	4.94	4.87	4.91	5.01	5.04	4.96	5.01	5.03	4.98	4.97	4.98	4.86
1393917_at	Cd163	CD163 molecule	1	uniqDRlyp/lyp_119	1	0	0	0	0	0	0	0	0	0	2.87	2.93	3.02	3.45	3.05	2.93	2.85	2.85	3.24	2.86	3.09	2.94	3.53	3.02	3.00	2.99	3.14	3.16	2.87	3.20
1394954_at	Slc31a2	solute carrier family 31 (copper transporters), member 2	1	uniqDRlyp/lyp_119	1	0	0	0	0	0	0	0	0	0	6.88	7.39	7.54	7.34	7.19	7.43	7.44	7.30	7.32	7.54	7.64	7.65	6.87	7.46	7.68	7.62	7.18	7.41	7.41	7.52
1395124_at	Wars	tryptophanyl-tRNA synthetase	1	uniqDRlyp/lyp_119	1	0	0	0	0	0	0	0	0	0	6.14	6.19	6.26	6.80	6.12	6.27	6.18	6.21	6.21	6.28	6.35	6.50	5.86	6.33	6.40	6.43	6.21	6.36	6.33	6.30
1395184_at	Clec12a	C-type lectin domain family 12, member A	1	uniqDRlyp/lyp_119	1	0	0	0	0	0	0	0	0	0	2.76	2.77	2.90	3.67	2.84	2.92	3.02	3.02	2.71	2.68	2.70	3.15	2.67	3.00	3.06	3.11	2.88	3.07	3.19	3.01
1395269_s_at	Gabrd	gamma-aminobutyric acid (GABA) A receptor, delta	1	uniqDRlyp/lyp_119	1	0	0	0	0	0	0	0	0	0	7.13	7.56	7.81	7.70	7.22	7.73	7.83	7.46	7.14	7.40	7.42	7.58	6.90	7.47	7.51	7.60	7.18	7.43	7.41	7.48
1395270_x_at	Gabrd	gamma-aminobutyric acid (GABA) A receptor, delta	1	uniqDRlyp/lyp_119	1	0	0	0	0	0	0	0	0	0	5.18	5.33	5.50	5.49	5.27	5.73	5.50	5.46	5.23	5.38	5.45	5.57	5.38	5.34	5.55	5.47	5.22	5.60	5.53	5.52
1396163_at	Igtp	interferon gamma induced GTPase	1	uniqDRlyp/lyp_119	1	0	0	0	0	0	0	0	0	0	3.65	3.56	3.88	5.15	3.81	3.62	3.62	3.75	3.45	3.61	3.68	3.73	3.44	3.77	3.82	3.75	3.53	3.46	3.52	3.67
1397304_at	Igtp	interferon gamma induced GTPase	1	uniqDRlyp/lyp_119	1	0	0	0	0	0	0	0	0	0	5.62	5.71	5.83	8.51	5.56	5.60	5.64	6.07	5.61	5.89	5.69	5.80	5.82	5.95	5.85	5.86	5.73	5.61	5.86	5.75
1397703_at	Tmem243	transmembrane protein 243, mitochondrial	1	uniqDRlyp/lyp_119	1	0	0	0	0	0	0	0	0	0	5.88	5.96	6.02	6.53	6.03	6.07	6.04	6.33	6.11	6.18	6.14	6.33	6.06	6.13	6.23	6.47	6.05	6.22	6.13	6.02
1377534_at	Stk32c	serine/threonine kinase 32C	0	uniqF+/+_122	0	0	0	1	uniqDR+/+_89	0	1	0	0	0	5.63	5.40	5.20	5.69	5.33	5.49	5.32	4.86	6.37	6.75	6.18	6.27	6.03	6.19	6.44	5.69	6.84	7.07	7.60	7.46
1367874_at	LOC1009117:	rho-related GTP-binding protein RhoQ-like /// ras homolog family member Q	0	uniqF+/+_122	0	0	0	1	0	0	0	0	0	0	7.73	7.88	7.96	7.96	7.61	7.74	7.91	7.95	7.83	8.08	8.14	8.28	7.79	8.18	8.28	8.30	7.75	8.06	8.20	8.31
1368064_a_at	Ddc	dopa decarboxylase (aromatic L-amino acid decarboxylase)	0	uniqF+/+_122	0	0	0	1	0	0	0	0	0	0	9.68	9.92	10.06	10.05	9.87	9.95	9.90	10.04	9.42	9.81	10.01	10.12	9.08	9.59	9.93	9.99	9.40	9.84	9.99	10.14
1368245_at	Upb1	ureidopropionase, beta	0	uniqF+/+_122	0	0	0	1	0	0	0	0	0	0	8.02	8.30	8.41	8.36	8.23	8.45	8.33	8.34	7.75	8.09	8.17	8.24	7.76	8.14	8.37	8.25	7.71	8.10	8.24	8.19
1368260_at	Aurkb	aurora kinase B	0	uniqF+/+_122	0	0	0	1	0	0	0	0	0	0	4.45	5.26	4.97	5.21	4.51	5.43	5.06	5.50	4.84	5.92	5.78	5.15	4.57	5.82	5.73	5.22	4.79	5.70	5.64	5.65
1368261_at	Nrxn3	neurexin 3	0	uniqF+/+_122	0	0	0	1	0	0	0	0	0	0	4.98	5.28	5.68	5.66	5.18	5.38	5.50	5.71	5.21	5.53	5.93	6.02	5.23	5.43	5.73	5.97	5.16	5.61	5.76	6.14
1368328_at	Gys2	glycogen synthase 2	0	uniqF+/+_122	0	0	0	1	0	0	0	0	0	0	3.17	2.95	3.75	3.31	2.96	2.89	2.98	2.84	2.62	2.85	2.71	3.18	3.42	2.56	3.18	2.93	2.61	2.90	3.13	3.27
1368359_a_at	Vgf	VGF nerve growth factor inducible	0	uniqF+/+_122	0	0	0	1	0	0	0	0	0	0	3.90	4.06	4.34	4.17	4.16	4.38	4.39	3.93	4.74	4.78	4.76	4.87	4.56	4.64	4.74	4.79	4.40	4.85	4.71	5.21
1368382_at	S100a3	S100 calcium binding protein A3	0	uniqF+/+_122	0	0	0	1	0	0	0	0	0	0	4.93	5.28	5.23	5.29	5.04	5.22	5.18	5.47	4.74	5.12	4.75	5.06	4.68	4.83	5.06	5.28	4.47	4.83	4.86	5.02
1368564_at	Slc17a6	solute carrier family 17 (sodium-dependent inorganic phosphate cotransporter), member 6	0	uniqF+/+_122	0	0	0	1	0	0	0	0	0	0	4.73	4.69	4.70	4.95	4.61	4.74	4.86	4.82	4.54	4.64	4.82	4.92	4.69	4.50	4.52	4.71	4.48	4.65	4.98	4.78
1368888_a_at	Rtn4	reticulon 4	0	uniqF+/+_122	0	0	0	1	0	0	0	0	0	0	9.56	9.64	9.70	9.83	9.46	9.60	9.68	9.65	9.54	9.70	9.72	9.72	9.47	9.63	9.75	9.76	9.42	9.59	9.65	9.84
1369070_at	LOC1009097(	peroxisome assembly protein 12-like /// peroxisomal biogenesis factor 12	0	uniqF+/+_122	0	0	0	1	0	0	0	0	0	0	6.86	7.08	7.23	7.18	6.92	7.05	7.17	7.18	6.95	7.11	7.16	7.35	7.00	7.06	7.30	7.26	6.91	7.00	7.34	7.51
1369194_a_at	Cdkn2a	cyclin-dependent kinase inhibitor 2A	0	uniqF+/+_122	0	0	0	1	0	0	0	0	0	0	3.54	3.66	3.83	3.97	3.74	3.38	3.90	3.93	3.47	3.84	3.78	3.96	3.43	3.81	3.96	4.17	3.45	3.73	4.06	3.94
1369293_at	Rtn4r	reticulon 4 receptor	0	uniqF+/+_122	0	0	0	1	0	0	0	0	0	0	4.10	4.15	4.13	4.12	4.06	4.29	4.21	4.09	4.49	4.15	4.36	4.27	3.81	4.23	4.38	4.20	4.12	4.28	4.25	4.26
1369404_a_at	Nrxn1	neurexin 1	0	uniqF+/+_122	0	0	0	1	0	0	0	0	0	0	6.76	6.98	7.12	7.17	6.80	6.86	6.98	7.06	6.85	7.13	7.16	7.23	6.77	6.98	7.11	7.37	6.80	7.12	7.16	7.42
1369509_a_at	A1bg	alpha-1-B glycoprotein	0	uniqF+/+_122	0	0	0	1	0	0	0	0	0	0	2.38	2.65	2.63	2.60	2.51	2.59	2.55	2.66	2.65	2.63	2.99	3.00	2.52	2.89	3.07	3.12	2.51	2.82	2.92	3.30
1370030_at	Gclm	glutamate cysteine ligase, modifier subunit	0	uniqF+/+_122	0	0	0	1	0	0	0	0	0	0	7.45	7.37	7.55	7.75	7.31	7.49	7.49	7.69	7.39	7.40	7.47	7.72	7.43	7.33	7.60	7.64	7.33	7.49	7.56	7.83
1370067_at	Me1	malic enzyme 1, NADP(+)-dependent, cytosolic	0	uniqF+/+_122	0	0	0	1	0	0	0	0	0	0	7.58	7.61	7.78	7.67	7.68	7.77	7.67	7.70	7.59	7.67	7.84	8.05	7.37	7.71	7.94	7.97	7.59	7.83	7.94	8.28
1370344_at	Hspa4	heat shock protein 4	0	uniqF+/+_122	0	0	0	1	0	0	0	0	0	0	7.24	7.19	6.92	8.10	7.09	7.11	7.12	7.35	7.19	7.30	7.21	7.44	6.83	7.25	7.24	7.32	7.21	7.26	7.97	7.83
1370444_at	Cacna1b	calcium channel, voltage-dependent, N type, alpha 1B subunit	0	uniqF+/+_122	0	0	0	1	0	0	0	0	0	0	5.70	6.13	6.24	6.10	5.71	6.23	6.15	5.93	5.94	6.23	6.26	6.28	5.88	6.11	6.14	6.22	5.78	6.25	6.38	6.41
1370478_at	Myo16	myosin XVI	0	uniqF+/+_122	0	0	0	1	0	0	0	0	0	0	4.63	4.55	4.73	4.83	4.74	4.83	4.73	4.82	4.40	4.58	4.92	5.05	4.55	4.75	4.70	5.07	4.31	4.71	5.10	5.25
1370870_at	Me1	malic enzyme 1, NADP(+)-dependent, cytosolic	0	uniqF+/+_122	0	0	0	1	0	0	0	0	0	0	8.42	8.41	8.49	8.45	8.56	8.50	8.37	8.42	8.59	8.40	8.52	8.65	8.63	8.33	8.56	8.63	8.52	8.57	8.60	8.86
1371007_at	Epha5	EPH receptor A5	0	uniqF+/+_122	0	0	0	1	0	0	0	0	0	0	4.33	4.41	4.45	4.41	4.43	4.31	4.58	4.42	4.34	4.61	4.83	4.75	4.84	4.57	4.73	4.80	4.46	4.35	4.76	5.12
1371175_a_at	Cacna1b /// L	calcium channel, voltage-dependent, N type, alpha 1B subunit /// voltage-dependent N-type	0	uniqF+/+_122	0	0	0	1	0	0	0	0	0	0	4.15	4.15	4.28	4.27	4.13	4.33	4.30	4.31	4.19	4.28	4.36	4.38	4.09	4.46	4.37	4.33	4.04	4.33	4.41	4.48
1371574_at	Ghitm	growth hormone inducible transmembrane protein	0	uniqF+/+_122	0	0	0	1	0	0	0	0	0	0	9.05	9.44	9.41	9.48	9.06	9.30	9.41	9.54	9.26	9.59	9.64	9.73	9.07	9.82	9.88	9.81	9.24	9.59	9.67	9.81
1371708_at	Car6	carbonic anhydrase 6	0	uniqF+/+_122	0	0	0	1	0	0	0	0	0	0	3.69	3.55	3.50	3.75	3.57	3.57	3.61	3.71	3.65	3.68	3.58	3.66	3.74	3.52	3.83	3.67	3.26	3.79	3.92	3.79
1371875_at	Manba	mannosidase, beta A, lysosomal	0	uniqF+/+_122	0	0	0	1	0	0	0	0	0	0	9.52	9.72	9.85	9.74	9.45	9.74	9.75	9.68	9.01	9.12	9.27	9.36	9.03	9.32	9.45	9.40	8.97	9.29	9.34	9.55
1371963_at	Pcca	propionyl CoA carboxylase, alpha polypeptide	0	uniqF+/+_122	0	0	0	1	0	0	0	0	0	0	7.03	7.29	7.43	7.31	7.11	7.25	7.24	7.32	7.05	7.23	7.38	7.50	6.97	7.27	7.33	7.47	6.98	7.20	7.40	7.57
1372048_at	Podxl2	podocalyxin-like 2	0	uniqF+/+_122	0	0	0	1	0	0	0	0	0	0	6.73	6.87	7.01	6.90	6.58	6.85	7.11	6.77	6.49	6.85	6.93	6.97	6.42	6.95	6.91	6.90	6.53	6.85	7.04	6.93
1372440_at	Serpine2	serpin peptidase inhibitor, clade E (nexin, plasminogen activator inhibitor type 1), member 2	0	uniqF+/+_122	0	0	0	1	0	0	0	0	0	0	6.95	7.34	7.31	7.21	7.05	7.33	7.18	7.54	6.52	6.47	6.78	6.92	6.57	6.73	6.89	7.04	6.59	6.82	7.10	7.16
1372601_at	Atf5	activating transcription factor 5	0	uniqF+/+_122	0	0	0	1	0	0	0	0	0	0	9.31	9.86	9.73	9.84	9.15	9.81	10.01	9.54	9.39	9.83	9.79	9.72	9.34	10.12	10.01	9.90	9.47	10.04	10.10	10.07
1372629_at	Coro2b	coronin, actin binding protein, 2B	0	uniqF+/+_122	0	0	0	1	0	0	0	0	0	0	7.42	7.54	7.67	7.63	7.39	7.59	7.61	7.54	7.50	7.66	7.82	7.91	7.56	7.64	7.62	7.82	7.42	7.69	7.96	7.91
1372665_at	Psat1	phosphoserine aminotransferase 1	0	uniqF+/+_122	0	0	0	1	0	0	0	0	0	0	6.91	7.53	7.23	7.31	7.12	7.38	7.33	7.59	7.22	7.47	7.60	7.51	7.01	7.91	7.74	7.46	7.31	7.82	7.74	7.82
1372736_at	Agl	amylo-alpha-1, 6-glucosidase, 4-alpha-glucanotransferase	0	uniqF+/+_122	0	0	0	1	0	0	0	0	0	0	7.29	7.61	7.68	7.58	7.39	7.65	7.55	7.58	7.23	7.58	7.56	7.58	7.15	7.51	7.63	7.63	7.10	7.60	7.64	7.69
1373008_x_at	Rtn4r	reticulon 4 receptor	0	uniqF+/+_122	0	0	0	1	0	0	0	0	0	0	4.97	4.87	4.93	4.92	4.86	4.97	5.04	4.77	5.54	5.34	5.48	5.57	5.14	5.32	5.45	5.47	5.42	5.27	5.48	5.96
1373199_at	Gla	galactosidase, alpha	0	uniqF+/+_122	0	0	0	1	0	0	0	0	0	0	7.46	7.51	7.66	7.70	7.68	7.54	7.84	7.81	7.70	7.80	7.93	8.06	7.71	7.82	7.96	8.08	7.68	7.83	7.99	8.22
1373427_at	Rragd	Ras-related GTP binding D	0	uniqF+/+_122	0	0	0	1	0	0	0	0	0	0	6.86	7.31	7.37	7.23	6.83	7.17	7.37	7.14	7.01	7.45	7.43	7.40	6.79	7.57	7.61	7.39	6.94	7.37	7.43	7.56
1374004_at	Prepl	prolyl endopeptidase-like	0	uniqF+/+_122	0	0	0	1	0	0	0	0	0	0	8.07	8.28	8.51	8.43	8.03	8.25	8.36	8.43	8.21	8.43	8.58	8.66	8.09	8.48	8.60	8.70	8.23	8.45	8.61	8.77
1374041_at	Ap5s1	adaptor-related protein complex 5, sigma 1 subunit	0	uniqF+/+_122	0	0	0	1	0	0	0	0	0	0	5.42	5.39	5.54	5.58	5.22	5.28	5.53	5.46	5.32	5.38	5.53	5.69	5.34	5.60	5.58	5.61	5.25	5.45	5.60	5.78
1374081_at	Casc4	cancer susceptibility candidate 4	0	uniqF+/+_122	0	0	0	1	0	0	0	0	0	0	9.57	9.73	9.92	9.90	9.66	9.70	9.76	9.89	9.95	10.08	10.18	10.36	10.01	10.02	10.20	10.33	9.78	10.01	10.05	10.33
1374108_at	Lrp12	low density lipoprotein receptor-related protein 12	0	uniqF+/+_122	0	0	0	1	0	0	0	0	0	0	7.37	7.46	7.63	7.50	7.47	7.42	7.38	7.55	7.48	7.65	7.80	7.90	7.69	7.52	7.75	7.92	7.45	7.69	7.91	8.04
1374235_at	Rcan2	regulator of calcineurin 2	0	uniqF+/+_122	0	0	0	1	0	0	0	0	0	0	6.79	6.88	7.07	7.03	6.78	6.80	6.93	7.01	6.76	6.90	7.05	7.11	6.53	7.08	7.02	7.18	6.75	7.01	7.14	7.26
1374633_at	Pm20d2	peptidase M20 domain containing 2	0	uniqF+/+_122	0	0	0	1	0	0	0	0	0	0	6.57	6.86	7.11	6.98	6.71	6.86	6.87	7.03	6.61	6.90	7.07	7.22	6.85	6.96	7.18	7.29	6.53	6.95	7.16	7.35
1374767_at	Npepo	aminopeptidase O	0	uniqF+/+_122	0	0	0	1	0	0	0	0	0	0	8.03	8.17	8.30	8.37	8.37	8.32	8.18	8.31	8.53	8.40	8.56	8.86	8.29	8.44	8.61	8.72	8.33	8.54	8.55	8.84
1374885_at	Slc12a6	solute carrier family 12, member 6	0	uniqF+/+_122	0	0	0	1	0	0	0	0	0	0	7.49	7.70	7.70	7.85	7.55	7.68	7.64	7.78	7.78	7.95	8.06	8.22	7.94	7.94	8.13	8.23	7.66	8.08	8.29	8.43
1374953_at	Tstd3	thiosulfate sulfurtransferase (rhodanese)-like domain containing 3	0	uniqF+/+_122	0	0	0	1	0	0	0	0	0	0	6.83	7.05	7.32	7.31	6.87	7.06	7.05	7.24	6.93	7.09	7.24	7.42	6.77	7.07	7.42	7.50	6.96	7.18	7.27	7.52
1375317_at	Ddc	dopa decarboxylase (aromatic L-amino acid decarboxylase)	0	uniqF+/+_122	0	0	0	1	0	0	0	0	0	0	6.75	6.93	6.80	6.83	7.05	6.99	6.85	6.69	6.51	6.62	6.70	6.76	6.34	6.33	6.59	6.77	6.44	6.67	6.78	6.78
1375398_at	---	---	0	uniqF+/+_122	0	0	0	1	0	0	0	0	0	0	3.74	3.80	3.93	3.81	3.71	3.70	3.74	3.69	3.71	3.78	3.66	3.85	3.66	3.86	3.80	3.76	3.72	3.86	3.53	3.98
1376142_at	Cnnm1	cyclin M1	0	uniqF+/+_122	0	0	0	1	0	0	0	0	0	0	4.04	3.90	4.10	4.05	4.36	4.32	4.09	3.79	4.00	4.08	3.99	4.11	4.03	4.00	4.10	3.94	3.86	4.03	3.82	4.07
1376168_at	Mmgt1	membrane magnesium transporter 1	0	uniqF+/+_122	0	0	0	1	0	0	0	0	0	0	6.66	6.80	6.92	6.86	6.58	6.71	6.84	6.92	6.70	7.02	7.10	7.09	6.77	6.98	7.19	7.07	6.73	7.02	7.20	7.33
1376258_at	---	---	0	uniqF+/+_122	0	0	0	1	0	0	0	0	0	0	3.73	3.84	3.81	4.14	3.92	4.20	4.13	4.08	3.84	4.01	4.01	4.07	3.90	3.83	4.08	3.98	3.66	3.95	3.89	4.18
1376418_a_at	Iars	isoleucyl-tRNA synthetase	0	uniqF+/+_122	0	0	0	1	0	0	0	0	0	0	9.09	9.57	9.65	9.67	9.14	9.58	9.57	9.59	9.46	9.82	9.80	9.73	9.19	9.88	9.95	9.84	9.50	10.00	9.94	10.05
1376456_at	Nmnat2	nicotinamide nucleotide adenylyltransferase 2	0	uniqF+/+_122	0	0	0	1	0	0	0	0	0	0	4.98	5.06	4.80	5.05	4.89	5.07	5.14	5.02	5.00	4.91	5.30	5.22	4.99	5.12	5.15	5.36	4.85	5.21	5.25	5.44
1376974_at	Ttc7a	tetratricopeptide repeat domain 7A	0	uniqF+/+_122	0	0	0	1	0	0	0	0	0	0	6.90	6.83	6.92	7.17	6.87	6.87	6.97	7.13	6.81	6.87	7.05	7.16	6.96	6.96	7.02	7.14	6.76	7.02	7.14	7.30
1377288_at	Hsf4	heat shock transcription factor 4	0	uniqF+/+_122	0	0	0	1	0	0	0	0	0	0	4.41	4.47	4.47	4.72	4.57	4.62	4.68	4.53	4.52	4.55	4.57	4.91	4.51	4.59	4.62	4.58	4.26	4.56	4.61	4.82
1377855_at	Ccdc41 /// LC	coiled-coil domain containing 41 /// coiled-coil domain-containing protein 41-like	0	uniqF+/+_122	0	0	0	1	0	0	0	0	0	0	6.86	7.09	6.96	7.02	6.81	7.08	6.95	6.94	7.04	7.27	7.39	7.37	6.83	7.28	7.26	7.34	7.04	7.43	7.40	7.52
1377935_at	Cldnd1	claudin domain containing 1	0	uniqF+/+_122	0	0	0	1	0	0	0	0	0	0	2.88	3.07	3.07	3.22	3.13	2.91	3.08	3.05	2.90	2.99	3.06	3.05	3.08	2.97	3.05	3.20	2.93	2.94	3.50	3.95
1378349_at	LOC1003629:	zinc finger protein 648	0	uniqF+/+_122	0	0	0	1	0	0	0	0	0	0	3.07	3.01	3.17	3.21	2.95	3.05	3.14	2.99	2.99	3.05	3.09	3.12	3.00	2.94	3.17	2.94	2.75	3.12	3.27	3.12
1378400_at	Fam122b ///	family with sequence similarity 122B /// protein FAM122B-like	0	uniqF+/+_122	0	0	0	1	0	0	0	0	0	0	4.60	4.69	4.87	4.68	4.64	4.58	4.88	4.83	4.42	4.83	4.83	4.87	4.67	4.78	4.90	4.66	4.41	4.64	4.89	4.91
1379246_at	RGD1308742	similar to Complement C5 precursor	0	uniqF+/+_122	0	0	0	1	0	0	0	0	0	0	4.53	4.20	4.24	4.36	4.55	4.22	4.22	4.31	4.69	4.39	4.60	4.87	5.22	4.90	4.76	4.71	4.57	4.52	4.75	5.13
1379333_at	Armcx6	armadillo repeat containing, X-linked 6	0	uniqF+/+_122	0	0	0	1	0	0	0	0	0	0	6.94	7.18	7.20	7.22	7.01	7.20	7.29	7.10	7.01	7.24	7.33	7.47	6.91	7.26	7.43	7.43	6.97	7.29	7.49	7.51
1380483_at	Pde8b	phosphodiesterase 8B	0	uniqF+/+_122	0	0	0	1	0	0	0	0	0	0	2.53	2.56	2.43	2.57	2.51	2.75	2.71	2.65	2.55	2.52	2.64	2.70	2.42	2.52	2.52	2.68	2.27	2.50	2.44	2.77
1381676_at	Nrxn3	neurexin 3	0	uniqF+/+_122	0	0	0	1	0	0	0	0	0	0	2.19	2.29	2.14	2.16	2.12	2.13	2.23	2.03	2.24	2.16	2.21	2.31	2.08	1.98	2.14	2.20	2.18	2.25	2.16	2.24
1382063_at	Gla	galactosidase, alpha	0	uniqF+/+_122	0	0	0	1	0	0	0	0	0	0	7.41	7.62	7.79	7.86	7.67	7.43	7.86	7.91	7.72	7.75	7.90	8.31	7.61	7.77	7.96	8.13	7.77	7.81	7.96	8.27
1382176_at	Klhl11	kelch-like family member 11	0	uniqF+/+_122	0	0	0	1	0	0	0	0	0	0	4.31	4.21	4.24	4.24	4.05	4.22	4.21	4.19	4.09	4.20	4.16	4.39	4.21	4.23	4.15	4.30	3.94	4.34	4.23	4.49

Affymetrix Probeset ID	Gene Symbol	Gene Name	unique BB on P7 n=822	Figure 1B P7 Venn n=1526	DRlyp/lyp STEM P7 n=712	DR+/+ STEM P7 n=936	Flyp/lyp STEM P7 n=520	F+/+ STEM P7 n=465	Figure 1B P0 Venn n=1071	DRlyp/lyp STEM P0 n=442	DR+/+ STEM P0 n=437	Flyp/lyp STEM P0 n=643	F+/+ STEM P0 n=632	Fig1D n=25	Mean log2 intensity DRlyp/lyp 20	Mean log2 intensity DRlyp/lyp 30	Mean log2 intensity DRlyp/lyp 40	Mean log2 intensity DRlyp/lyp 50	Mean log2 intensity DR+/+ 20	Mean log2 intensity DR+/+ 30	Mean log2 intensity DR+/+ 40	Mean log2 intensity DR+/+ 50	Mean log2 intensity Flyp/lyp 20	Mean log2 intensity Flyp/lyp 30	Mean log2 intensity Flyp/lyp 40	Mean log2 intensity Flyp/lyp 50	Mean log2 intensity Flyp20	Mean log2 intensity Flyp30	Mean log2 intensity Flyp40	Mean log2 intensity Flyp50	Mean log2 intensity F+/+ 20	Mean log2 intensity F+/+ 30	Mean log2 intensity F+/+ 40	Mean log2 intensity F+/+ 50
1382222_at	Tmem163	transmembrane protein 163	0	uniqF+/+_122	0	0	0	1	0	0	0	0	0	0	6.66	6.99	7.15	7.21	6.65	7.11	7.04	7.22	6.93	7.36	7.55	7.63	6.95	7.29	7.48	7.63	7.00	7.40	7.71	7.81
1382325_at	Gcat	glycine C-acetyltransferase	0	uniqF+/+_122	0	0	0	1	0	0	0	0	0	0	7.01	7.35	7.20	7.30	7.32	7.22	7.15	7.36	7.14	7.10	7.28	7.39	7.04	7.44	7.31	7.33	7.00	7.49	7.51	7.61
1382479_at	Lrrc4c	leucine rich repeat containing 4C	0	uniqF+/+_122	0	0	0	1	0	0	0	0	0	0	3.44	3.19	3.50	3.47	3.32	3.28	3.31	3.45	3.06	3.41	3.22	3.60	3.11	3.28	3.44	3.51	2.98	3.26	3.64	3.53
1382748_at	Mut	methylmalonyl CoA mutase	0	uniqF+/+_122	0	0	0	1	0	0	0	0	0	0	7.11	7.21	7.39	7.52	6.95	7.25	7.14	7.41	7.14	7.36	7.46	7.61	6.94	7.33	7.49	7.77	7.12	7.45	7.51	7.64
1382814_at	Tenm3	teneurin transmembrane protein 3	0	uniqF+/+_122	0	0	0	1	0	0	0	0	0	0	6.99	7.28	7.24	7.25	6.91	7.05	7.24	7.31	7.36	7.58	7.66	7.78	7.46	7.67	7.67	7.79	7.35	7.58	7.76	7.97
1383434_at	---	---	0	uniqF+/+_122	0	0	0	1	0	0	0	0	0	0	7.70	7.98	7.99	7.93	7.89	7.93	7.91	8.11	7.76	7.85	8.06	8.23	7.76	8.29	8.07	8.13	7.75	8.18	8.27	8.34
1383567_at	Colec11	collectin sub-family member 11	0	uniqF+/+_122	0	0	0	1	0	0	0	0	0	0	4.65	4.80	4.61	4.99	4.79	4.74	4.90	4.82	5.28	5.40	5.30	5.66	5.52	5.58	5.56	5.55	5.09	5.24	5.34	5.61
1383843_at	---	---	0	uniqF+/+_122	0	0	0	1	0	0	0	0	0	0	5.75	5.81	5.86	6.06	5.86	5.76	5.89	5.93	5.84	5.74	5.88	6.08	6.25	5.83	5.93	6.12	5.77	5.80	6.06	6.31
1384289_at	Rell2	RELT-like 2	0	uniqF+/+_122	0	0	0	1	0	0	0	0	0	0	5.78	5.97	6.04	6.06	5.76	6.10	6.03	6.01	5.95	6.18	6.26	6.38	5.97	6.21	6.28	6.38	5.94	6.30	6.42	6.57
1384368_at	Slc25a16	solute carrier family 25 (mitochondrial carrier, Graves disease autoantigen), member 16	0	uniqF+/+_122	0	0	0	1	0	0	0	0	0	0	7.22	7.20	7.33	7.27	7.12	7.10	7.21	7.34	7.23	7.30	7.37	7.48	7.40	7.23	7.38	7.53	7.16	7.28	7.45	7.68
1384428_at	Tmem181	transmembrane protein 181	0	uniqF+/+_122	0	0	0	1	0	0	0	0	0	0	6.91	6.91	7.00	7.06	6.91	6.90	7.07	6.96	6.77	6.88	6.98	7.03	6.60	6.98	7.06	7.13	6.62	6.96	7.04	7.22
1385690_at	Mut	methylmalonyl CoA mutase	0	uniqF+/+_122	0	0	0	1	0	0	0	0	0	0	6.19	6.31	6.42	6.40	6.25	6.23	6.26	6.36	6.29	6.45	6.62	6.78	6.07	6.37	6.71	6.64	6.19	6.51	6.64	6.74
1385794_at	MGC94891	hypothetical protein LOC681210	0	uniqF+/+_122	0	0	0	1	0	0	0	0	0	0	2.67	2.64	2.68	2.75	2.60	2.69	2.85	2.55	2.28	2.52	2.69	2.65	2.61	2.64	2.53	2.65	2.55	2.44	2.71	2.98
1386571_at	Tom1l1	target of myb1 (chicken)-like 1	0	uniqF+/+_122	0	0	0	1	0	0	0	0	0	0	5.12	5.26	5.40	5.16	5.02	5.10	5.26	5.32	4.90	5.26	5.34	5.11	4.91	5.11	5.27	5.25	4.79	4.96	5.27	5.41
1386583_at	Iars	isoleucyl-tRNA synthetase	0	uniqF+/+_122	0	0	0	1	0	0	0	0	0	0	4.42	4.79	4.71	4.73	4.31	4.55	4.75	4.72	4.70	4.83	4.74	4.81	4.64	4.96	4.83	4.93	4.55	4.84	4.79	5.13
1386917_at	Pc	pyruvate carboxylase	0	uniqF+/+_122	0	0	0	1	0	0	0	0	0	0	6.23	6.43	6.51	6.67	6.14	6.33	6.63	6.54	6.17	6.35	6.52	6.60	6.23	6.33	6.56	6.80	6.20	6.36	6.72	6.67
1386967_at	LOC1009117	rho-related GTP-binding protein RhoQ-like /// ras homolog family member Q	0	uniqF+/+_122	0	0	0	1	0	0	0	0	0	0	8.57	8.78	8.99	8.91	8.52	8.76	8.88	8.89	8.60	8.88	8.94	9.06	8.67	8.88	9.03	9.14	8.54	8.87	8.92	9.10
1387058_at	Pctp	phosphatidylcholine transfer protein	0	uniqF+/+_122	0	0	0	1	0	0	0	0	0	0	6.60	6.78	7.04	6.94	6.71	6.76	6.86	7.01	6.75	6.89	7.13	7.23	7.00	6.92	7.19	7.14	6.73	6.79	7.06	7.26
1387086_at	Camlg	calcium modulating ligand	0	uniqF+/+_122	0	0	0	1	0	0	0	0	0	0	7.47	7.51	7.61	7.59	7.42	7.49	7.60	7.53	7.46	7.61	7.62	7.85	7.58	7.63	7.71	7.78	7.44	7.56	7.77	7.95
1387141_at	Dpysl5 /// LO	dihydropyrimidinase-like 5 /// dihydropyrimidinase-related protein 5-like /// dihydropyrimidi	0	uniqF+/+_122	0	0	0	1	0	0	0	0	0	0	5.54	5.64	5.83	5.82	5.41	5.73	5.67	5.67	5.52	5.76	5.74	6.01	5.48	5.71	5.63	5.91	5.45	5.73	5.97	5.91
1387301_at	Fgf1	fibroblast growth factor 1 (acidic)	0	uniqF+/+_122	0	0	0	1	0	0	0	0	0	0	4.74	4.93	4.79	4.93	4.85	4.90	4.79	4.85	4.78	4.67	4.92	4.78	4.72	4.91	4.77	4.70	4.45	4.78	4.82	5.01
1387596_at	F2rl1	coagulation factor II (thrombin) receptor-like 1	0	uniqF+/+_122	0	0	0	1	0	0	0	0	0	0	4.85	5.05	5.28	5.00	4.96	5.00	5.18	5.07	4.99	5.15	5.25	5.39	4.76	5.09	5.40	5.32	4.68	5.27	5.20	5.22
1387868_at	Lbp	lipopolysaccharide binding protein	0	uniqF+/+_122	0	0	0	1	0	0	0	0	0	0	3.34	3.39	3.19	3.36	2.96	3.18	3.38	3.41	3.38	3.07	3.27	3.49	3.03	3.33	3.37	3.51	3.15	3.40	3.76	3.52
1387925_at	Asns	asparagine synthetase (glutamine-hydrolyzing)	0	uniqF+/+_122	0	0	0	1	0	0	0	0	0	0	9.39	9.81	9.79	9.73	9.36	9.85	9.78	9.62	9.58	9.87	9.94	9.86	9.29	10.10	10.06	9.91	9.60	10.03	10.08	10.10
1388010_at	Parpbp	PARP1 binding protein	0	uniqF+/+_122	0	0	0	1	0	0	0	0	0	0	4.59	4.97	4.78	4.99	4.65	4.95	5.00	4.98	4.68	5.34	5.25	4.76	4.68	4.83	5.13	4.74	4.65	5.15	5.18	5.21
1388020_a_at	Pde1c	phosphodiesterase 1C	0	uniqF+/+_122	0	0	0	1	0	0	0	0	0	0	3.75	3.81	3.84	3.92	3.84	3.95	3.90	3.75	3.90	3.68	3.89	4.23	3.69	3.99	3.94	4.06	3.91	4.04	4.22	4.50
1388027_a_at	Rtn4	reticulon 4	0	uniqF+/+_122	0	0	0	1	0	0	0	0	0	0	8.44	8.33	8.49	8.20	8.25	8.01	8.49	8.13	8.23	8.43	8.49	8.42	7.70	8.38	8.51	8.31	8.10	8.26	8.69	8.86
1388040_a_at	Myo16	myosin XVI	0	uniqF+/+_122	0	0	0	1	0	0	0	0	0	0	3.60	3.68	3.61	3.72	3.64	3.80	3.77	3.76	3.65	3.72	3.75	3.86	3.65	3.81	3.75	3.77	3.61	3.69	3.73	3.76
1388060_at	Syt12	synaptotagmin XII	0	uniqF+/+_122	0	0	0	1	0	0	0	0	0	0	6.88	7.03	7.24	6.75	6.75	7.07	6.87	6.80	6.94	7.01	7.07	7.05	6.43	7.09	7.19	6.87	6.51	6.90	7.00	7.26
1388189_at	Grm3	glutamate receptor, metabotropic 3	0	uniqF+/+_122	0	0	0	1	0	0	0	0	0	0	2.57	2.47	2.70	2.49	2.57	2.69	2.68	2.64	2.53	2.63	2.52	2.88	2.49	2.75	2.58	2.68	2.32	2.70	2.91	2.73
1388219_at	Htr5b	5-hydroxytryptamine (serotonin) receptor 5B	0	uniqF+/+_122	0	0	0	1	0	0	0	0	0	0	4.61	5.08	5.01	4.73	4.67	4.88	5.04	4.70	4.93	5.19	5.28	5.20	5.18	5.44	5.55	5.29	4.99	5.24	5.49	5.51
1388395_at	G0s2	G0/G1switch 2	0	uniqF+/+_122	0	0	0	1	0	0	0	0	0	0	5.69	5.78	5.98	5.90	5.67	5.77	5.96	6.01	5.60	5.64	5.73	5.84	5.46	5.85	5.95	5.92	5.59	5.86	6.08	6.16
1388501_at	MGC125239	hypothetical protein LOC686179	0	uniqF+/+_122	0	0	0	1	0	0	0	0	0	0	6.18	6.22	6.36	6.49	6.31	6.20	6.26	6.34	6.12	6.22	6.40	6.52	6.38	6.25	6.38	6.59	6.12	6.24	6.46	6.67
1388972_at	Rtn4r	reticulon 4 receptor	0	uniqF+/+_122	0	0	0	1	0	0	0	0	0	0	5.85	5.80	5.83	5.76	5.44	5.70	5.87	5.64	6.25	6.15	6.31	6.39	6.18	6.20	6.22	6.19	6.27	6.17	6.22	6.78
1389066_at	Rcan2	regulator of calcineurin 2	0	uniqF+/+_122	0	0	0	1	0	0	0	0	0	0	8.67	8.77	8.92	8.85	8.75	8.73	8.82	8.84	8.53	8.64	8.82	8.89	8.70	8.64	8.80	8.90	8.48	8.69	8.92	9.09
1389573_at	Chac1	ChaC, cation transport regulator homolog 1 (E. coli)	0	uniqF+/+_122	0	0	0	1	0	0	0	0	0	0	7.76	9.10	8.65	8.76	8.00	9.11	9.12	8.46	8.01	8.79	8.88	8.43	8.14	8.70	8.76	8.56	8.16	9.16	9.03	9.00
1389594_at	Abhd12	abhydrolase domain containing 12	0	uniqF+/+_122	0	0	0	1	0	0	0	0	0	0	6.30	6.46	6.62	6.68	6.27	6.44	6.52	6.52	6.35	6.56	6.75	6.80	6.13	6.62	6.74	6.74	6.28	6.64	6.71	6.88
1389735_at	Rps6ka6	ribosomal protein S6 kinase polypeptide 6	0	uniqF+/+_122	0	0	0	1	0	0	0	0	0	0	5.39	5.73	5.81	5.65	5.24	5.58	5.56	5.57	5.58	5.90	5.98	5.95	5.41	5.64	5.84	5.91	5.48	5.83	5.92	6.19
1389877_at	Foxc1	forkhead box C1	0	uniqF+/+_122	0	0	0	1	0	0	0	0	0	0	3.12	2.94	3.04	3.02	2.97	2.98	2.92	3.13	3.03	2.89	3.16	3.22	2.83	3.22	3.03	3.03	2.80	3.05	2.94	3.32
1389918_at	LOC1003602	palladin	0	uniqF+/+_122	0	0	0	1	0	0	0	0	0	0	7.20	7.34	7.49	7.44	7.35	7.29	7.32	7.72	7.11	7.25	7.44	7.58	7.20	7.13	7.44	7.61	7.13	7.26	7.48	7.66
1390232_at	RGD1311946	similar to RIKEN cDNA 1810055G02	0	uniqF+/+_122	0	0	0	1	0	0	0	0	0	0	4.20	4.26	4.36	4.53	4.18	4.09	4.37	4.54	4.28	4.27	4.45	4.73	4.39	4.50	4.70	4.63	4.20	4.44	4.50	4.71
1390272_at	Dph5	DPH5 homolog (S. cerevisiae)	0	uniqF+/+_122	0	0	0	1	0	0	0	0	0	0	5.50	5.78	5.80	5.88	5.56	5.61	5.84	5.96	5.37	5.41	5.47	5.48	5.51	5.46	5.74	5.47	5.36	5.19	5.49	5.70
1390407_at	Cldnd1	claudin domain containing 1	0	uniqF+/+_122	0	0	0	1	0	0	0	0	0	0	8.72	8.66	8.68	8.58	8.66	8.73	8.67	8.52	8.63	8.69	8.74	8.68	8.55	8.77	8.73	8.64	8.72	8.80	8.77	8.76
1390623_at	Gins3	GINS complex subunit 3 (Psf3 homolog)	0	uniqF+/+_122	0	0	0	1	0	0	0	0	0	0	5.68	5.90	5.96	5.97	5.63	5.86	5.91	6.06	5.65	5.84	6.00	6.08	5.66	5.77	6.08	6.00	5.55	5.76	6.00	6.10
1390805_at	Ccdc167	coiled-coil domain containing 167	0	uniqF+/+_122	0	0	0	1	0	0	0	0	0	0	6.68	6.95	6.94	7.04	6.77	6.82	6.88	7.11	6.89	7.05	7.22	7.35	6.30	6.94	6.93	7.01	6.87	7.40	7.29	7.44
1390815_at	---	---	0	uniqF+/+_122	0	0	0	1	0	0	0	0	0	0	4.25	4.22	4.69	4.62	4.39	4.42	4.65	4.45	4.66	4.68	4.86	5.14	4.60	4.59	4.81	4.91	4.50	4.82	5.12	5.44
1390875_a_at	Ccdc41 /// LC	coiled-coil domain containing 41 /// coiled-coil domain-containing protein 41-like	0	uniqF+/+_122	0	0	0	1	0	0	0	0	0	0	6.08	6.34	6.38	6.42	6.15	6.37	6.31	6.40	6.21	6.63	6.79	6.80	6.20	6.54	6.77	6.89	6.16	6.67	6.83	6.98
1391200_at	Spry3	sprouty homolog 3 (Drosophila)	0	uniqF+/+_122	0	0	0	1	0	0	0	0	0	0	2.63	2.90	2.99	2.74	2.79	2.78	2.86	2.93	2.67	3.03	2.93	3.07	2.78	2.78	2.82	2.95	2.52	2.89	3.07	3.19
1391919_at	Tcerg1l	transcription elongation regulator 1-like	0	uniqF+/+_122	0	0	0	1	0	0	0	0	0	0	4.60	4.59	4.45	4.34	4.54	4.51	4.46	4.60	4.55	4.69	4.83	5.02	4.51	4.58	4.95	4.72	4.50	4.87	4.94	5.43
1392815_at	Arap2	ArfGAP with RhoGAP domain, ankyrin repeat and PH domain 2	0	uniqF+/+_122	0	0	0	1	0	0	0	0	0	0	7.75	7.94	7.89	8.09	7.74	7.74	7.96	7.99	7.86	7.86	7.99	8.02	7.98	7.81	8.08	8.06	7.71	7.76	8.05	8.22
1393152_at	Mut	methylmalonyl CoA mutase	0	uniqF+/+_122	0	0	0	1	0	0	0	0	0	0	7.33	7.49	7.49	7.65	7.38	7.38	7.56	7.59	7.38	7.59	7.72	7.90	7.25	7.55	7.72	7.93	7.32	7.61	7.89	8.05
1393373_at	Opn3	opsin 3	0	uniqF+/+_122	0	0	0	1	0	0	0	0	0	0	4.86	5.05	5.24	5.12	5.28	5.12	5.00	5.49	5.56	5.57	5.54	5.64	5.30	5.52	5.86	5.84	5.28	5.67	5.61	6.15
1393831_at	---	---	0	uniqF+/+_122	0	0	0	1	0	0	0	0	0	0	2.97	2.95	2.85	2.86	3.08	3.11	2.93	3.02	3.52	4.26	4.24	3.86	3.74	4.14	4.59	4.23	3.60	4.44	4.51	4.49
1395325_s_at	Mmgt1	membrane magnesium transporter 1	0	uniqF+/+_122	0	0	0	1	0	0	0	0	0	0	8.45	8.62	8.68	8.63	8.45	8.66	8.58	8.65	8.46	8.72	8.91	8.81	8.32	8.82	8.90	8.77	8.56	8.80	8.80	8.96
1395414_at	Agl	amylo-alpha-1, 6-glucosidase, 4-alpha-glucanotransferase	0	uniqF+/+_122	0	0	0	1	0	0	0	0	0	0	5.10	5.42	5.27	5.38	5.03	5.12	5.31	5.41	5.13	5.36	5.35	5.49	4.83	5.31	5.53	5.57	5.02	5.25	5.82	5.71
1396182_at	Pm20d2	peptidase M20 domain containing 2	0	uniqF+/+_122	0	0	0	1	0	0	0	0	0	0	3.00	3.07	3.10	3.11	3.12	3.19	3.06	3.00	3.18	2.97	3.23	3.29	3.12	3.24	3.15	3.15	2.95	3.22	3.21	3.29
1399115_at	Agt4a /// Atg	autophagy related 4A, cysteine peptidase /// autophagy related 4A, cysteine peptidase-like 1	0	uniqF+/+_122	0	0	0	1	0	0	0	0	0	0	6.42	6.44	6.55	6.66	6.27	6.33	6.61	6.57	6.42	6.56	6.71	6.74	6.44	6.53	6.55	6.71	6.53	6.39	6.61	6.94
1387930_at	Reg3a	regenerating islet-derived 3 alpha	0	uniqFlyp/lyp_183	0	0	1	0	DRlyp/lypnDR+/+_48	1	1	0	0	0	10.97	7.58	6.85	4.57	9.36	6.31	5.45	6.28	8.05	9.28	9.82	9.86	11.80	11.19	9.68	8.54	3.97	6.64	4.80	3.39
1368238_at	Reg3b	regenerating islet-derived 3 beta	0	uniqFlyp/lyp_183	0	0	1	0	DRlyp/lypnF+/+_28	1	0	0	1	0	11.48	7.99	7.53	5.20	10.34	6.66	6.92	7.45	8.34	9.17	9.76	9.76	11.83	11.28	9.38	8.34	5.48	6.50	4.94	3.51
1367728_at	Tsn	translin	0	uniqFlyp/lyp_183	0	0	1	0	0	0	0	0	0	0	6.97	7.07	7.28	7.31	7.04	7.40	7.22	7.14	6.55	6.87	7.18	7.00	5.89	6.90	7.16	7.06	6.77	7.03	6.54	6.83
1367809_at	Prl4a1	prolactin family 4, subfamily a, member 1	0	uniqFlyp/lyp_183	0	0	1	0	0	0	0	0	0	0	5.15	5.00	5.06	5.15	4.93	5.00	5.01	5.10	4.84	5.02	5.27	5.42	5.02	5.08	5.02	5.25	5.02	5.00	5.31	5.29
1367885_at	Pxmp2	peroxisomal membrane protein 2	0	uniqFlyp/lyp_183	0	0	1	0	0	0	0	0	0	0	6.18	6.50	6.55	6.57	6.38	6.52	6.69	6.54	6.35	6.55	6.60	6.86	6.52	6.54	6.62	6.76	6.44	6.51	6.69	6.83
1367932_at	Hmgcs1	3-hydroxy-3-methylglutaryl-CoA synthase 1 (soluble)	0	uniqFlyp/lyp_183	0	0	1	0	0	0	0	0	0	0	8.19	7.67	7.75	7.84	8.11	7.81	7.61	8.30	7.68	8.09	8.20	8.07	7.81	7.46	7.31	7.70	7.71	7.97	7.91	7.47
1368092_at	Fah	fumarylacetoacetate hydrolase	0	uniqFlyp/lyp_183	0	0	1	0	0	0	0	0	0	0	6.25	6.45	6.45	6.55	6.03	6.40	6.49	6.43	6.19	6.65	6.63	6.72	6.23	6.56	6.55	6.72	6.36	6.54	6.78	6.80
1368254_a_at	Sphk1	sphingosine kinase 1	0	uniqFlyp/lyp_183	0	0	1	0	0	0	0	0	0	0	5.03	4.81	4.99	5.14	5.20	4.86	5.37	5.22	4.49	4.78	4.94	5.08	4.71	4.69	4.76	4.95	4.71	4.84	4.96	4.89
1368275_at	Msmo1	methylsterol monooxygenase 1	0	uniqFlyp/lyp_183	0	0	1	0	0	0	0	0	0	0	8.77	8.61	8.84	8.98	8.87	8.62	8.84	9.11	8.52	8.74	8.97	9.07	8.79	8.43	8.61	8.75	8.55	8.53	8.72	8.71
1368293_at	Cpz	carboxypeptidase Z	0	uniqFlyp/lyp_183	0	0	1	0	0	0	0	0	0	0	5.37	5.11	5.05	5.13	5.20	5.34	5.40	5.07	4.78	5.08	5.03	5.46	4.97	5.23	5.21	5.00	5.07	5.35	5.33	5.31
1368417_at	Syt5	synaptotagmin V	0	uniqFlyp/lyp_183	0	0	1	0	0	0	0	0	0	0	5.31	5.12	5.14	5.23	5.23	5.08	5.18	5.29	5.21	4.98	5.39	5.73	5.10	5.17	5.30	5.54	5.24	5.29	5.58	5.66
1368453_at	Fads2	fatty acid desaturase 2	0	uniqFlyp/lyp_183	0	0	1	0	0	0	0	0	0	0	5.87	6.02	6.02	6.02	5.96	5.98	5.98	6.04	5.65	5.81	5.90	6.22	5.62	5.98	5.90	6.00	5.61	5.89	6.01	5.98
1368460_at	Slc2a5	solute carrier family 2 (facilitated glucose/fructose transporter), member 5	0	uniqFlyp/lyp_183	0	0	1	0	0	0	0	0	0	0	5.95	5.87	6.09	5.96	6.00	5.88	6.03	5.96	5.64	5.84	5.95	6.20	5.85	5.88	5.90	6.12	5.64	5.70	5.93	5.80
1368483_a_at	Slit1	slit homolog 1 (Drosophila)	0	uniqFlyp/lyp_183	0	0	1	0	0	0	0	0	0	0	4.76	4.83	4.83	4.73	4.67	4.65	4.78	4.88	4.79	5.02	5.29	5.26	5.04	5.12	5.08	5.33	5.09	4.98	5.15	5.28
1368500_a_at	Rgs9	regulator of G-protein signaling 9	0	uniqFlyp/lyp_183	0	0	1	0	0	0	0	0	0	0	6.23	6.25	6.29	6.43	6.19	6.25	6.45	6.41	6.10	6.28	6.51	6.75	6.33	6.25	6.39	6.57	6.18	6.28	6.47	6.53
1368548_at	Slc12a1	solute carrier family 12 (sodium/potassium/chloride transporters), member 1	0	uniqFlyp/lyp_183	0	0	1	0	0	0	0	0	0	0	3.91	3.69	3.72	3.46	4.19	3.59	3.63	3.81	2.95	2.73	3.22	3.41	3.32	3.08	3.15	3.15	3.63	3.48	4.20	3.91
1368695_at	C4bpb	complement component 4 binding protein, beta	0	uniqFlyp/lyp_183	0	0	1	0	0	0	0	0	0	0	4.50	4.63	4.57	4.64	4.65	4.59	4.75	4.80	4.42	4.52	4.82	5.03	4.63	4.72	4.91	4.82	4.53	4.59	4.78	4.89
1368766_at	Mmp7	matrix metallopeptidase 7	0	uniqFlyp/lyp_183	0	0	1	0	0	0	0	0	0	0	2.58	2.41	2.45	2.92	2.40	2.62	3.70	2.51	2.51	2.42	2.47	2.92	2.48	2.53	2.64	3.18	2.47	2.47	2.61	2.63
1368788_at	Chad	chondroadherin	0	uniqFlyp/lyp_183	0	0	1	0	0	0	0	0	0	0	2.85	2.67	2.74	2.88	2.58	2.77	2.89	2.93	2.50	2.71	2.72	3.10	2.61	2.63	2.71	2.79	2.53	2.61	2.82	2.97
1368857_at	Ugt8	UDP glycosyltransferase 8	0	uniqFlyp/lyp_183	0	0	1	0	0	0	0	0	0	0	2.00	1.96	1.88	1.92	1.84	1.93	1.97	1.92	1.91	1.86	1.92	1.99	1.92	2.01	1.96	1.93	1.96	1.95	1.93	2.05
1368858_at	Ugt8	UDP glycosyltransferase 8	0	uniqFlyp/lyp_183	0	0	1	0	0	0	0	0	0	0	6.00	6.32	6.64	6.58	6.05	6.25	6.54	6.50	6.22	6.65	6.90	7.18	6.18	6.70	7.07	7.09	6.36	6.97	7.09	7.24
1368972_at	Ntrk2	neurotrophic tyrosine kinase, receptor, type 2	0	uniqFlyp/lyp_183	0	0	1	0	0	0	0	0	0	0	4.20	4.64	4.59	4.37	4.31	4.50	4.46	4.43	3.58	3.76	3.90	4.16	4.07	3.69	3.90	4.15	3.77	3.64	3.93	3.88
1368999_a_at	Begain	brain-enriched guanylate kinase-associated	0	uniqFlyp/lyp_183	0	0	1	0	0	0	0	0	0	0	5.82	5.81	5.85	5.98	5.90	5.87	6.07	5.96	5.61	5.81	5.84	6.24	5.59	5.96	5.90	5.95	5.72	5.85	5.94	6.08
1369063_at	Anp32a	acidic (leucine-rich) nuclear phosphoprotein 32 family, member A	0	uniqFlyp/lyp_183	0	0	1	0	0	0	0	0	0	0	7.78	7.81	8.55	6.50	7.90	8.50	8.21	6.83	7.77	7.92	8.35	8.01	8.05	8.57	8.23	6.61	8.09	8.17	6.87	7.39
1369067_at	Nr4a3	nuclear receptor subfamily 4, group A, member 3	0	uniqFlyp/lyp_183	0	0	1	0	0	0	0	0	0	0	3.89	4.14	4.34	4.22	4.27	3.74	5.34	5.13	4.02	5.09	5.46	5.08	3.87	4.61	4.86	5.37	4.05	5.38	5.46	4.33
1369073_at	Nr1h4	nuclear receptor subfamily 1, group H, member 4	0	uniqFlyp/lyp_183	0	0	1	0	0	0	0	0	0	0	7.70	7.69	7.90	7.79	7.71	7.67	7.76	7.68	7.51	7.69	7.86	8.09	7.64	7.75	7.76	7.93	7.59	7.77	7.94	7.94
1369076_at	Tmem33	transmembrane protein 33	0	uniqFlyp/lyp_183	0	0	1	0	0	0	0	0	0	0	7.20	7.03	7.07	7.64	7.01	7.09	7.28	7.39	7.08	7.34	7.40	7.60	6.59	7.54	7.53	7.69	7.13	7.42	7.59	7.54
1369161_at	Abcb4	ATP-binding cassette, subfamily B (MDR/TAP), member 4	0	uniqFlyp/lyp_183	0	0	1	0	0	0	0	0	0	0	5.42	5.71	5.83	5.64	5.70	5.65	5.72	5.59	5.52	5.78	5.87	6.04	5.74	5.85	5.92	5.95	5.74	5.72	5.83	5.98
1369217_at	Nr4a3	nuclear receptor subfamily 4, group A, member 3	0	uniqFlyp/lyp_183	0	0	1	0	0	0	0	0	0	0	5.29	5.24	5.13	5.04	5.48	5.14	5.30	5.01	5.34	5.20	5.37	5.04	5.19	4.89	5.48	5.31	5.18	5.25	5.61	4.99
1369284_at	Barhl2	BarH-like homeobox 2	0	uniqFlyp/lyp_183	0	0	1	0	0	0	0	0	0	0	2.91	3.04	3.17	3.12	3.07	3.08	3.09	2.98	2.79	3.06	3.00	3.30	3.10	3.05	2.91	2.92	2.93	3.16	3.11	3.11
1369407_at	Tnfrsf11b	tumor necrosis factor receptor superfamily, member 11b	0	uniqFlyp/lyp_183	0	0	1	0	0	0	0	0	0	0	9.10	9.08	9.29	9.37	9.19	8.83	9.09	9.37	8.23	8.14	8.34	8.67	8.89	8.06	8.49	8.44	8.38	7.90	8.39	8.30
1369526_at	Acadsb	acyl-CoA dehydrogenase, short/branched chain	0	uniqFlyp/lyp_183	0	0	1	0	0	0	0	0	0	0	3.69	3.81	3.90	3.60	3.57	3.74	3.64	3.57	3.19	3.41	3.73	3.50	2.47	3.42	3.47	3.40	3.13	3.15	3.34	3.22
1369539_at	St3gal3	ST3 beta-galactoside alpha-2,3-sialyltransferase 3	0	uniqFlyp/lyp_183	0	0	1	0	0	0	0	0	0	0	3.83	3.97	4.03	4.08	3.91	3.95	4.17	3.82	3.97	4.10	4.12	4.34	3.53	4.10	4.14	4.13	3.95	4.20	4.13	4.18
1369567_at	Taar1	trace-amine-associated receptor 1	0	uniqFlyp/lyp_183	0	0	1	0	0	0	0	0	0	0	5.12	5.16	5.32	5.24	5.06	5.12	5.30	5.14	4.78	5.09	5.19	5.58	5.27	5.07	5.28	5.19	4.67	4.79	4.95	5.06
1369624_at	Prlh	prolactin releasing hormone	0	uniqFlyp/lyp_183	0	0	1	0	0	0	0	0	0	0	4.99	4.98	5.00	5.07	5.08	5.05	5.12	5.16	4.81	4.97	5.04	5.39	5.06	5.07	5.12	5.22	4.95	5.07	5.08	5.26
1369659_at	Cga	glycoprotein hormones, alpha polypeptide	0	uniqFlyp/lyp_183	0	0	1	0	0	0	0	0	0	0	3.68	3.39	3.51	3.58	3.52	3.58	3.30	3.33	3.45	3.35	3.53	3.88	3.45	3.51	3.74	3.52	3.78	3.63	3.61	3.65
1369754_a_at	Cast	calpastatin	0	uniqFlyp/lyp_183	0	0	1	0	0	0	0	0	0	0	6.29	6.43	6.47	6.47	6.36	6.26	6.39	6.78	5.99	6.25	6.32	6.57	5.86	6.22	6.50	6.48	6.18	6.26	6.35	6.48
1369787_at	Cckar	cholecystokinin A receptor	0	uniqFlyp/lyp_183	0	0	1	0	0	0	0	0	0	0	9.80	9.50	9.72	10.09	9.91	9.50	9.62	10.11	9.47	9.39	9.89	10.27	9.89	9.51	9.82	10.15	9.41	9.31	9.77	9.91
1369957_at	Rgs5	regulator of G-protein signaling 5	0	uniqFlyp/lyp_183	0	0	1	0	0	0	0	0	0	0	8.79	8.87	8.98	8.51	8.54	8.96	8.95	8.76	8.20	8.45	8.72	8.92	7.75	8.59	8.87	8.87	8.43	8.60	8.48	8.73
1370153_at	Gdf15	growth differentiation factor 15	0	uniqFlyp/lyp_183	0	0	1	0	0	0	0	0	0	0	4.51	5.04	4.99	4.38	4.25	5.25	5.56	4.64	4.55	5.03	5.17	5.38	4.36	5.99	5.30	5.01	4.63	5.68	5.06	5.19
1370177_at	PVR	poliovirus receptor	0	uniqFlyp/lyp_183	0	0	1	0	0	0	0	0	0	0	3.96	4.11	4.27	4.37	4.14	4.04	4.21	4.49	4.36	4.43	4.57	4.98	4.42	4.60	4.81	4.93	4.61	4.53	4.76	4.92
1370222_at	Pitx3	paired-like homeodomain 3	0	uniqFlyp/lyp_183	0	0	1	0	0	0	0	0	0	0	2.79	2.80	2.84	2.81	2.70	2.80	2.93	2.88	2.49	2.68	2.89	3.02	2.81	2.82	2.81	2.94	2.72	2.75	2.88	3.12
1370229_at	Ndrg4	NDRG family member 4	0	uniqFlyp/lyp_183	0	0	1	0	0	0	0	0	0	0	9.51	9.48	9.64	9.83	9.50	9.44	9.70	9.66	9.53	9.70	9.82	10.06	9.61	9.62	9.75	10.03	9.60	9.66	9.94	10.07
1370362_at	Ptprn	protein tyrosine phosphatase, receptor type, N	0	uniqFlyp/lyp_183	0	0	1	0	0	0	0	0	0	0	8.73	8.79	8.83	9.06	8.83	8.74	8.96	8.94	8.83	8.94	9.06	9.37	8.77	8.81	9.07	9.39	8.93	8.97	9.25	9.21
1370526_at	Itgae	integrin, alpha E	0	uniqFlyp/lyp_183	0	0	1	0	0	0	0	0	0	0	6.25	6.49	6.61	6.70	6.19	6.32	6.56	6.69	6.01	6.56	6.56	6.64	6.14	6.36	6.57	6.65	6.22	6.27	6.61	6.60
1370606_at	P2ry1	purinergic receptor P2Y, G-protein coupled, 1	0	uniqFlyp/lyp_183	0	0	1	0	0	0	0	0	0	0	9.17	9.14	9.43	9.31	8.97	8.97	9.08	9.37	9.33	9.72	9.76	9.87	9.49	9.57	9.53	9.72	9.29	9.69	9.83	9.62
1370649_at	Bdkrb2	bradykinin receptor B2	0	uniqFlyp/lyp_183	0	0	1	0	0	0	0	0	0	0	3.65	3.82	4.04	3.97	3.80	3.89	4.19	3.92	3.67	3.87	3.93	4.23	3.70	3.88	4.11	3.96	4.02	3.98	4.01	3.99
1370650_s_at	Bdkrb2	bradykinin receptor B2	0	uniqFlyp/lyp_183	0	0	1	0	0	0	0	0	0	0	4.05	4.01	4.14	4.06	3.92	3.95	4.14	4.05	3.80	4.14	4.09	4.30	3.95	4.07	4.10	4.17	3.99	4.16	4.08	4.26
1370652_at	Ntrk2	neurotrophic tyrosine kinase, receptor, type 2	0	uniqFlyp/lyp_183	0	0	1	0	0	0	0	0	0	0	4.44	4.51	4.90	4.61	4.62	4.28	4.40	4.23	3.70	3.71	4.06	4.15	4.27	3.72	3.71	4.41	3.95	4.15	4.31	3.99
1370687_a_at	Ntrk2	neurotrophic tyrosine kinase, receptor, type 2	0	uniqFlyp/lyp_183	0	0	1	0	0	0	0	0	0	0	8.05	8.25	8.33	8.47	7.97	8.16	8.31	8.40	7.09	7.39	7.72	7.84	7.27	7.77	7.71	7.82	7.09	7.46	7.58	7.79
1370814_at	Dhrs4	dehydrogenase/reductase (SDR family) member 4	0	uniqFlyp/lyp_183	0	0	1	0	0	0	0	0	0	0	7.88	8.30	8.41	8.25	7.92	8.29	8.31	8.33	7.71	8.10	8.34	8.47	7.35	8.17	8.43	8.52	7.85	8.34	8.25	8.52
1370815_at	Nefh	neurofilament, heavy polypeptide	0	uniqFlyp/lyp_183	0	0	1	0	0	0	0	0	0	0	6.33	6.31	6.30	6.51	6.35	6.19	6.32	6.47	6.45	6.33	6.67	6.84	6.57	6.50	6.58	6.73	6.53	6.52	6.64	6.83
1371120_s_at	Bdkrb2	bradykinin receptor B2	0	uniqFlyp/lyp_183	0	0	1	0	0	0	0	0	0	0	3.39	3.43	3.35	3.39	3.31	3.36	3.49	3.51	3.28	3.28	3.24	3.43	3.48	3.55	3.39	3.35	3.36	3.38	3.34	3.53
1371157_at	Apob	apolipoprotein B	0	uniqFlyp/lyp_183	0	0	1	0	0	0	0	0	0	0	3.74	3.85	3.91	3.81	3.72	3.74	3.89	3.79	3.83	3.73	3.90	3.98	3.70	3.87	3.90	3.81	3.81	3.94	3.95	3.86
1371173_a_at	Cast	calpastatin	0	uniqFlyp/lyp_183	0	0	1	0	0	0	0	0	0	0	5.13	5.25	5.25	5.25	5.18	5.20	4.97	5.15	4.92	4.90	5.34	5.40	4.15	5.03	5.38	5.14	5.05	5.11	4.90	5.09
1371700_at	LOC1009117	microfibril-associated glycoprotein 4-like /// microfibrillar-associated protein 4	0	uniqFlyp/lyp_183	0	0	1	0	0	0	0	0	0	0	5.66	5.78	5.87	5.37	6.06	6.02	5.70	5.70	4.64	4.82	4.71	5.17	4.68	4.64	5.19	5.19	4.81	5.22	5.17	5.11
1371932_at	Tmem192	transmembrane protein 192	0	uniqFlyp/lyp_183	0	0	1	0	0	0	0	0	0	0	7.51	7.76	7.99	7.94	7.49	7.79	7.85	7.85	7.40	7.92	7.88	7.93	7.54	7.78	7.95	7.96	7.49	7.75	7.90	7.93
1372213_at	LOC500300	similar to hypothetical protein MGC6835	0	uniqFlyp/lyp_183	0	0	1	0	0	0	0	0	0	0	7.65	8.35	8.37	8.03	8.11	8.23	8.26	8.23	7.62	8.02	8.12	8.06	7.83	8.25	7.86	8.08	7.66	8.39	8.25	7.60
1372229_at	Pdk3	pyruvate dehydrogenase kinase, isozyme 3	0	uniqFlyp/lyp_183	0	0	1	0	0	0	0	0	0	0	6.38	6.54	6.70	6.82	6.39	6.53	6.76	6.92	6.62	6.85	7.07	7.18	6.63	6.89	6.96	7.19	6.73	6.85	7.06	7.27
1372387_at	RGD1311874	hypothetical LOC300751	0	uniqFlyp/lyp_183	0	0	1	0	0	0	0	0	0	0	4.55	4.71	4.70	4.53	4.37	4.63	4.60	4.62	4.08	4.38	4.62	4.44	4.17	4.51	4.49	4.36	4.52	4.18	4.69	4.63

Affymetrix Probeset ID	Gene Symbol	Gene Name	unique BB on P7 n=822	Figure 1B P7 Venn n=1526	DRlyp/lyp STEM P7 n=712	DR+/+ STEM P7 n=936	Flyp/lyp STEM P7 n=520	F+/+ STEM P7 n=465	Figure 1B P0 Venn n=1071	DRlyp/lyp STEM P0 n=442	DR+/+ STEM P0 n=437	Flyp/lyp STEM P0 n=643	F+/+ STEM P0 n=632	Fig1D n=25	Mean log2 intensity DRlyp/lyp 20	Mean log2 intensity DRlyp/lyp 30	Mean log2 intensity DRlyp/lyp 40	Mean log2 intensity DRlyp/lyp 50	Mean log2 intensity DR+/+ 20	Mean log2 intensity DR+/+ 30	Mean log2 intensity DR+/+ 40	Mean log2 intensity DR+/+ 50	Mean log2 intensity Flyp/lyp 20	Mean log2 intensity Flyp/lyp 30	Mean log2 intensity Flyp/lyp 40	Mean log2 intensity Flyp/lyp 50	Mean log2 intensity Flyp20	Mean log2 intensity Flyp30	Mean log2 intensity Flyp40	Mean log2 intensity Flyp50	Mean log2 intensity F+/+ 20	Mean log2 intensity F+/+ 30	Mean log2 intensity F+/+ 40	Mean log2 intensity F+/+ 50
1372434_at	---	---	0	uniqFlyp/lyp_183	0	0	1	0	0	0	0	0	0	0	5.81	6.06	5.99	6.08	5.99	6.19	6.02	6.07	5.90	6.08	6.08	6.43	5.97	6.03	6.12	6.12	5.95	6.12	6.31	6.31
1372499_at	Ankrd24 /// L	ankyrin repeat domain 24 /// ankyrin repeat domain-containing protein 24-like	0	uniqFlyp/lyp_183	0	0	1	0	0	0	0	0	0	0	6.96	7.35	7.42	7.36	6.98	7.25	7.34	7.24	6.95	7.41	7.40	7.48	6.77	7.42	7.52	7.56	7.04	7.37	7.39	7.53
1372510_at	Srxn1	sulfiredoxin 1	0	uniqFlyp/lyp_183	0	0	1	0	0	0	0	0	0	0	6.40	6.45	6.71	6.68	6.43	6.63	6.74	6.74	6.34	6.71	6.90	7.14	6.40	6.74	6.95	7.14	6.52	6.90	7.01	7.24
1372931_at	Praf2	PRA1 domain family, member 2	0	uniqFlyp/lyp_183	0	0	1	0	0	0	0	0	0	0	4.96	5.21	5.21	5.28	5.04	5.27	5.26	5.30	4.83	5.21	5.28	5.45	4.67	5.20	5.23	5.41	5.11	5.23	5.53	5.49
1373043_at	Sdf2l1	stromal cell-derived factor 2-like 1	0	uniqFlyp/lyp_183	0	0	1	0	0	0	0	0	0	0	8.69	8.71	8.78	9.02	9.21	8.87	8.75	9.29	8.74	8.95	9.16	9.35	8.85	8.97	9.05	9.19	8.88	9.08	9.25	9.37
1373064_at	Dnajc15	DnaJ (Hsp40) homolog, subfamily C, member 15	0	uniqFlyp/lyp_183	0	0	1	0	0	0	0	0	0	0	7.43	7.69	7.79	7.89	7.48	7.56	7.80	7.95	7.40	7.68	7.77	7.92	7.55	7.75	7.84	7.93	7.41	7.64	7.72	7.79
1373198_at	Dnajc22	DnaJ (Hsp40) homolog, subfamily C, member 22	0	uniqFlyp/lyp_183	0	0	1	0	0	0	0	0	0	0	6.17	6.34	6.41	6.38	6.40	6.37	6.41	6.47	6.09	6.35	6.39	6.64	6.23	6.27	6.47	6.40	6.40	6.56	6.67	6.73
1373329_at	Tmprss2	transmembrane protease, serine 2	0	uniqFlyp/lyp_183	0	0	1	0	0	0	0	0	0	0	6.00	6.47	6.64	6.79	6.36	6.44	6.70	6.59	5.87	6.33	6.53	6.66	6.03	6.24	6.37	6.60	6.03	6.39	6.71	6.66
1373333_at	MGC109340	similar to Microsomal signal peptidase 23 kDa subunit (SPase 22 kDa subunit) (SPC22/23)	0	uniqFlyp/lyp_183	0	0	1	0	0	0	0	0	0	0	7.35	7.53	7.66	7.72	7.42	7.56	7.61	7.78	7.42	7.84	7.97	7.92	7.64	7.82	7.99	7.95	7.46	7.64	7.77	7.95
1373466_at	Cast	calpastatin	0	uniqFlyp/lyp_183	0	0	1	0	0	0	0	0	0	0	8.24	8.43	8.68	8.77	8.15	8.34	8.54	8.87	7.73	8.15	8.39	8.66	7.93	8.22	8.49	8.73	7.82	8.26	8.51	8.82
1373607_at	---	---	0	uniqFlyp/lyp_183	0	0	1	0	0	0	0	0	0	0	6.97	7.25	7.35	7.40	6.99	7.24	7.30	7.30	6.88	7.26	7.39	7.53	6.96	7.16	7.40	7.45	6.97	7.31	7.48	7.54
1374043_at	Gramd3	GRAM domain containing 3	0	uniqFlyp/lyp_183	0	0	1	0	0	0	0	0	0	0	9.75	9.69	9.92	10.05	9.73	9.75	9.84	10.01	9.69	9.81	10.03	10.24	9.87	9.92	9.96	10.15	9.70	9.92	10.08	10.16
1375085_at	Slc25a35	solute carrier family 25, member 35	0	uniqFlyp/lyp_183	0	0	1	0	0	0	0	0	0	0	3.52	3.62	3.61	3.79	3.47	3.42	3.71	3.73	3.49	3.80	3.70	4.14	3.62	3.77	3.63	3.82	3.59	3.68	3.70	3.96
1375198_at	Rbm24	RNA binding motif protein 24	0	uniqFlyp/lyp_183	0	0	1	0	0	0	0	0	0	0	3.53	3.47	3.48	3.49	3.34	3.35	3.55	3.42	3.21	3.24	3.33	3.80	3.29	3.50	3.47	3.56	3.32	3.42	3.59	3.67
1375305_at	---	---	0	uniqFlyp/lyp_183	0	0	1	0	0	0	0	0	0	0	10.40	10.61	10.62	10.62	10.64	10.64	10.56	10.41	9.63	10.18	10.29	10.40	9.38	9.89	10.10	10.45	9.94	10.18	10.34	10.34
1375726_at	Lmo7	LIM domain 7	0	uniqFlyp/lyp_183	0	0	1	0	0	0	0	0	0	0	6.77	6.86	6.73	6.92	6.77	6.74	6.93	6.88	6.67	6.75	6.95	7.24	6.98	7.02	7.05	7.25	6.93	6.90	7.09	7.41
1375799_at	Tmem121	transmembrane protein 121	0	uniqFlyp/lyp_183	0	0	1	0	0	0	0	0	0	0	4.83	4.75	4.83	4.95	4.64	4.67	4.91	4.74	4.41	4.75	4.93	5.00	4.49	4.82	4.80	4.94	4.56	4.76	4.93	4.94
1376188_at	---	---	0	uniqFlyp/lyp_183	0	0	1	0	0	0	0	0	0	0	4.57	4.66	4.50	4.60	4.57	4.70	4.87	4.66	4.25	4.54	4.64	4.81	4.45	4.62	4.65	4.69	4.54	4.58	4.73	4.71
1376425_at	---	---	0	uniqFlyp/lyp_183	0	0	1	0	0	0	0	0	0	0	6.18	6.37	6.48	6.45	6.34	6.31	6.38	6.43	5.71	6.08	6.11	6.25	5.84	6.07	6.22	6.40	5.80	6.18	6.10	6.17
1376746_at	Ldhd	lactate dehydrogenase D	0	uniqFlyp/lyp_183	0	0	1	0	0	0	0	0	0	0	4.15	4.12	4.25	4.22	4.09	4.15	4.30	4.17	4.08	4.17	4.27	4.63	4.02	4.15	4.20	4.22	3.97	4.20	4.34	4.35
1376770_at	Efhd1	EF-hand domain family, member D1	0	uniqFlyp/lyp_183	0	0	1	0	0	0	0	0	0	0	6.22	6.15	6.11	6.42	6.35	6.06	6.40	6.52	5.61	5.55	5.79	6.20	6.41	5.60	5.93	5.97	5.94	5.37	5.79	5.79
1376995_at	Foxd4	forkhead box D4	0	uniqFlyp/lyp_183	0	0	1	0	0	0	0	0	0	0	4.15	4.20	4.22	4.20	4.09	4.16	4.25	4.14	3.86	4.06	4.34	4.42	4.07	4.21	4.27	4.26	4.20	4.27	4.21	4.30
1377615_at	Tmem130	transmembrane protein 130	0	uniqFlyp/lyp_183	0	0	1	0	0	0	0	0	0	0	10.77	10.81	11.02	10.97	10.76	10.84	10.95	10.86	10.73	10.90	11.09	11.23	10.98	11.02	11.17	11.25	10.84	11.01	11.19	11.30
1377860_at	Ttc39b	tetratricopeptide repeat domain 39B	0	uniqFlyp/lyp_183	0	0	1	0	0	0	0	0	0	0	5.80	6.00	6.12	6.13	5.76	5.94	6.04	6.00	5.97	6.23	6.47	6.62	6.09	6.37	6.51	6.43	6.03	6.30	6.45	6.56
1377918_at	Stoml1	stomatin (EPB72)-like 1	0	uniqFlyp/lyp_183	0	0	1	0	0	0	0	0	0	0	5.66	5.92	5.87	5.86	5.50	6.10	5.98	5.81	5.65	5.92	5.91	6.16	5.46	5.96	5.93	6.22	5.51	5.92	5.56	5.86
1378180_at	Dctd	dCMP deaminase	0	uniqFlyp/lyp_183	0	0	1	0	0	0	0	0	0	0	7.76	7.87	7.78	7.76	7.65	7.75	7.71	7.66	7.57	8.05	8.08	8.12	7.80	8.18	8.04	8.13	7.85	8.15	8.22	8.08
1378963_at	---	---	0	uniqFlyp/lyp_183	0	0	1	0	0	0	0	0	0	0	2.62	2.60	2.75	2.62	2.53	2.71	2.61	2.61	2.58	2.59	2.43	2.69	2.48	2.59	2.50	2.77	2.71	2.49	2.47	2.69
1378997_at	Ephb6	Eph receptor B6	0	uniqFlyp/lyp_183	0	0	1	0	0	0	0	0	0	0	4.26	4.52	4.53	4.60	4.09	4.40	4.53	4.50	4.33	4.56	4.56	4.85	4.23	4.53	4.70	4.70	4.40	4.56	4.72	4.79
1379676_a_at	Dnase1l1	deoxyribonuclease 1-like 1	0	uniqFlyp/lyp_183	0	0	1	0	0	0	0	0	0	0	6.55	6.56	6.70	6.94	6.48	6.70	6.73	6.86	6.46	6.61	6.88	6.96	6.52	6.70	6.85	6.97	6.57	6.73	6.78	6.95
1379724_at	Phldb2	pleckstrin homology-like domain, family B, member 2	0	uniqFlyp/lyp_183	0	0	1	0	0	0	0	0	0	0	8.40	8.31	8.41	8.48	8.48	8.09	8.23	8.41	8.19	8.18	8.31	8.70	8.09	8.24	8.33	8.54	8.13	8.35	8.62	8.55
1379790_at	Dll4	delta-like 4 (Drosophila)	0	uniqFlyp/lyp_183	0	0	1	0	0	0	0	0	0	0	5.40	5.76	5.81	5.75	5.65	5.90	5.83	5.85	5.65	5.64	5.96	6.02	5.69	5.92	5.84	6.05	5.56	5.92	5.78	6.16
1380228_at	Rimkla	ribosomal modification protein rimK-like family member A	0	uniqFlyp/lyp_183	0	0	1	0	0	0	0	0	0	0	2.95	2.83	3.02	3.15	2.90	3.08	3.12	2.97	3.12	3.30	3.21	3.40	3.22	3.08	3.06	3.44	3.20	3.34	3.43	3.45
1380305_at	Lrrc73	leucine rich repeat containing 73	0	uniqFlyp/lyp_183	0	0	1	0	0	0	0	0	0	0	6.31	6.16	6.33	6.34	6.30	6.09	6.23	6.24	6.00	6.23	6.43	6.72	6.49	6.36	6.33	6.51	6.27	6.34	6.58	6.64
1380474_at	Loxl2	lysyl oxidase-like 2	0	uniqFlyp/lyp_183	0	0	1	0	0	0	0	0	0	0	5.32	5.64	5.77	5.82	5.29	5.54	5.59	5.66	5.34	5.90	6.06	6.20	5.01	5.86	5.95	6.14	5.29	6.00	5.87	6.22
1381067_at	Srxn1	sulfiredoxin 1	0	uniqFlyp/lyp_183	0	0	1	0	0	0	0	0	0	0	2.79	2.95	2.86	2.77	2.80	2.71	2.76	2.75	2.81	2.91	2.85	3.03	2.74	2.81	2.78	3.02	2.82	2.79	2.99	2.87
1381190_at	Lmo7	LIM domain 7	0	uniqFlyp/lyp_183	0	0	1	0	0	0	0	0	0	0	7.02	7.13	7.25	7.31	7.19	7.09	7.32	7.32	6.89	7.04	7.37	7.54	7.12	7.35	7.39	7.78	7.09	7.27	7.32	7.78
1381798_at	Lmo7	LIM domain 7	0	uniqFlyp/lyp_183	0	0	1	0	0	0	0	0	0	0	4.99	5.00	4.99	5.11	4.96	4.85	4.99	4.90	4.72	4.91	5.38	5.67	5.19	5.14	4.99	5.70	4.96	5.28	5.60	5.41
1381821_at	Mdga2	MAM domain containing glycosylphosphatidylinositol anchor 2	0	uniqFlyp/lyp_183	0	0	1	0	0	0	0	0	0	0	3.97	4.06	3.99	3.87	3.65	3.84	3.82	3.77	3.47	3.94	4.15	4.44	3.59	3.95	3.88	4.15	3.65	3.75	3.89	4.02
1381904_at	Vwa7	von Willebrand factor A domain containing 7	0	uniqFlyp/lyp_183	0	0	1	0	0	0	0	0	0	0	3.54	3.75	3.81	3.74	3.76	3.83	3.91	3.79	3.62	3.83	3.83	4.13	3.71	3.77	3.69	3.82	3.68	3.74	3.84	3.90
1381975_at	---	---	0	uniqFlyp/lyp_183	0	0	1	0	0	0	0	0	0	0	8.77	9.01	9.19	9.25	8.84	9.02	9.11	9.20	8.62	8.85	8.93	9.15	8.70	8.81	9.05	9.11	8.58	8.85	8.89	8.97
1382061_at	Ldhd	lactate dehydrogenase D	0	uniqFlyp/lyp_183	0	0	1	0	0	0	0	0	0	0	5.86	6.04	6.19	6.15	5.79	6.09	5.91	5.90	5.56	6.08	6.04	6.07	5.55	6.01	6.13	6.00	5.66	5.98	5.99	6.06
1382941_at	Lrrc32	leucine rich repeat containing 32	0	uniqFlyp/lyp_183	0	0	1	0	0	0	0	0	0	0	4.83	4.74	4.69	4.69	4.60	4.85	4.73	4.93	4.32	4.24	4.60	4.79	4.78	4.64	4.40	4.53	4.37	4.63	4.56	4.45
1383395_at	Agmat	agmatine ureohydrolase (agmatinase)	0	uniqFlyp/lyp_183	0	0	1	0	0	0	0	0	0	0	4.99	5.07	5.05	5.41	5.04	5.24	5.22	5.33	4.89	5.02	5.10	5.49	4.89	5.13	5.26	5.54	5.02	5.05	5.29	5.37
1383554_at	Rnf128	ring finger protein 128, E3 ubiquitin protein ligase	0	uniqFlyp/lyp_183	0	0	1	0	0	0	0	0	0	0	5.03	5.19	5.20	5.17	5.15	4.94	5.21	5.05	4.85	4.81	5.06	5.50	4.68	5.03	5.21	5.12	4.83	5.16	5.16	5.28
1383653_at	Snx11	sorting nexin 11	0	uniqFlyp/lyp_183	0	0	1	0	0	0	0	0	0	0	7.01	7.07	7.15	7.26	6.99	7.04	7.23	7.27	6.93	7.09	7.35	7.48	7.22	7.18	7.17	7.40	7.00	7.06	7.23	7.34
1383915_at	LOC686120	hypothetical protein LOC686120	0	uniqFlyp/lyp_183	0	0	1	0	0	0	0	0	0	0	4.93	4.89	5.01	4.98	4.79	4.82	5.01	5.02	5.37	5.53	5.65	5.90	5.41	5.59	5.82	5.95	5.33	5.41	5.59	5.59
1384023_at	LOC1009094	spindlin-2-like /// spindlin family, member 2A	0	uniqFlyp/lyp_183	0	0	1	0	0	0	0	0	0	0	4.91	4.98	5.23	5.04	4.89	4.71	4.92	5.04	4.61	4.99	5.13	5.51	5.40	5.16	5.10	5.28	4.89	4.91	5.04	5.09
1384177_at	Gramd3	GRAM domain containing 3	0	uniqFlyp/lyp_183	0	0	1	0	0	0	0	0	0	0	6.67	6.67	6.94	6.91	6.70	6.70	6.93	7.00	6.74	6.81	7.10	7.30	6.46	6.97	6.89	7.07	6.73	6.90	7.06	7.12
1384285_at	March3	membrane-associated ring finger (C3HC4) 3, E3 ubiquitin protein ligase	0	uniqFlyp/lyp_183	0	0	1	0	0	0	0	0	0	0	6.49	6.55	6.77	6.76	6.38	6.42	6.74	6.67	6.18	6.42	6.55	6.89	6.54	6.49	6.64	6.84	6.45	6.56	6.70	6.87
1384315_at	Pdk3	pyruvate dehydrogenase kinase, isozyme 3	0	uniqFlyp/lyp_183	0	0	1	0	0	0	0	0	0	0	5.47	5.64	5.65	5.69	5.39	5.38	5.55	5.81	5.61	5.76	6.01	6.14	5.33	5.84	5.94	6.03	5.60	5.85	5.91	6.05
1384331_at	Srxn1	sulfiredoxin 1	0	uniqFlyp/lyp_183	0	0	1	0	0	0	0	0	0	0	4.51	4.60	4.79	4.34	4.64	4.75	4.87	4.67	4.47	4.62	4.86	5.04	4.34	4.81	4.95	4.91	4.67	4.84	4.79	4.79
1384645_at	Tcaim	T cell activation inhibitor, mitochondrial	0	uniqFlyp/lyp_183	0	0	1	0	0	0	0	0	0	0	3.21	3.06	3.35	3.17	3.16	3.16	3.31	3.17	3.15	3.48	3.57	3.65	3.27	3.33	3.40	3.49	3.42	3.39	3.41	3.55
1384950_at	Pi4k2b	phosphatidylinositol 4-kinase type 2 beta	0	uniqFlyp/lyp_183	0	0	1	0	0	0	0	0	0	0	4.63	4.67	4.88	4.87	4.46	4.60	4.75	4.77	4.02	4.58	4.57	4.72	4.09	4.51	4.74	4.49	4.28	4.52	4.63	4.70
1385139_at	Fam221a	family with sequence similarity 221, member A	0	uniqFlyp/lyp_183	0	0	1	0	0	0	0	0	0	0	4.08	4.09	4.29	4.18	4.12	4.38	4.52	4.33	3.84	4.24	4.17	4.45	4.40	4.28	4.17	4.25	3.93	4.18	4.23	4.22
1385297_at	Recql4	RecQ protein-like 4	0	uniqFlyp/lyp_183	0	0	1	0	0	0	0	0	0	0	4.12	4.13	3.97	4.13	3.89	4.08	4.12	4.17	3.86	4.05	4.14	4.37	4.09	4.10	4.04	4.08	3.95	4.19	4.11	4.24
1385349_at	Cetn4	centrin 4	0	uniqFlyp/lyp_183	0	0	1	0	0	0	0	0	0	0	5.12	5.61	5.67	5.54	5.14	5.43	5.59	5.40	5.27	5.72	5.83	5.84	5.27	5.71	5.75	5.83	5.45	5.73	5.81	5.83
1385488_at	Dhrs4	dehydrogenase/reductase (SDR family) member 4	0	uniqFlyp/lyp_183	0	0	1	0	0	0	0	0	0	0	4.43	4.35	4.47	4.53	4.47	4.58	4.58	4.49	4.41	4.35	4.55	4.80	4.34	4.35	4.68	4.69	4.43	4.43	4.65	4.59
1385562_at	Jph3	junctophilin 3	0	uniqFlyp/lyp_183	0	0	1	0	0	0	0	0	0	0	4.43	4.46	4.37	4.30	4.62	4.57	4.49	4.22	3.78	4.13	4.16	4.37	3.69	4.24	4.25	4.20	3.98	4.08	4.07	4.32
1385623_at	Tulp2	tubby-like protein 2	0	uniqFlyp/lyp_183	0	0	1	0	0	0	0	0	0	0	3.72	3.65	3.73	3.69	3.66	3.68	3.76	3.62	3.44	3.65	3.83	3.95	3.66	3.67	3.80	3.86	3.68	3.74	3.75	3.88
1385810_at	Lrrc51	leucine rich repeat containing 51	0	uniqFlyp/lyp_183	0	0	1	0	0	0	0	0	0	0	2.17	2.44	2.57	2.43	2.32	2.59	2.51	2.45	2.30	2.63	2.47	2.71	2.19	2.40	2.59	2.55	2.36	2.57	2.66	2.52
1387029_at	Cfh	complement factor H	0	uniqFlyp/lyp_183	0	0	1	0	0	0	0	0	0	0	5.03	5.35	5.40	5.29	4.98	5.29	5.45	5.40	4.66	4.85	4.70	5.31	4.56	4.78	5.33	5.29	4.49	5.04	5.19	4.95
1387125_at	S100a9	S100 calcium binding protein A9	0	uniqFlyp/lyp_183	0	0	1	0	0	0	0	0	0	0	5.19	3.82	4.23	4.45	4.81	3.99	4.16	4.31	4.14	4.60	4.59	4.93	4.50	4.55	4.64	4.82	4.02	3.97	4.19	4.21
1387163_at	Hcrt	hypocretin	0	uniqFlyp/lyp_183	0	0	1	0	0	0	0	0	0	0	4.79	5.03	4.82	5.04	4.89	5.10	5.17	5.02	4.87	5.10	5.15	5.46	4.91	5.10	5.13	5.20	4.96	5.13	5.20	5.22
1387172_a_at	Tgfb2	transforming growth factor, beta 2	0	uniqFlyp/lyp_183	0	0	1	0	0	0	0	0	0	0	6.34	6.68	6.76	6.68	6.61	6.57	6.66	6.62	5.97	6.21	6.35	6.44	6.35	6.33	6.41	6.42	6.09	6.39	6.45	6.46
1387211_at	Barhl1	BarH-like homeobox 1	0	uniqFlyp/lyp_183	0	0	1	0	0	0	0	0	0	0	4.53	4.51	4.66	4.63	4.46	4.58	4.84	4.73	4.33	4.51	4.77	4.88	4.57	4.48	4.58	4.57	4.48	4.48	4.62	4.53
1387251_at	Msmb	microseminoprotein, beta	0	uniqFlyp/lyp_183	0	0	1	0	0	0	0	0	0	0	2.42	2.30	2.45	2.48	2.28	2.24	2.54	2.51	2.13	2.45	2.43	2.71	2.34	2.43	2.46	2.41	2.32	2.38	2.51	2.54
1387404_at	Slc8a3	solute carrier family 8 (sodium/calcium exchanger), member 3	0	uniqFlyp/lyp_183	0	0	1	0	0	0	0	0	0	0	4.64	4.60	4.57	4.73	4.59	4.59	4.65	4.60	4.47	4.54	4.77	4.98	4.55	4.81	4.75	5.09	4.77	4.47	4.75	4.66
1387422_at	Pglyrp1	peptidoglycan recognition protein 1	0	uniqFlyp/lyp_183	0	0	1	0	0	0	0	0	0	0	4.18	4.26	4.48	4.38	4.27	4.39	4.57	4.30	4.09	4.10	4.16	4.60	4.14	4.32	4.28	4.45	4.22	4.08	4.27	4.21
1387426_at	Slc25a21	solute carrier family 25 (mitochondrial oxoadipate carrier), member 21	0	uniqFlyp/lyp_183	0	0	1	0	0	0	0	0	0	0	3.10	3.18	3.26	3.25	3.12	3.18	3.35	3.23	2.98	3.13	3.17	3.53	3.08	3.05	3.22	3.32	3.15	3.02	3.18	3.29
1387530_a_at	Fosl2	fos-like antigen 2	0	uniqFlyp/lyp_183	0	0	1	0	0	0	0	0	0	0	3.81	3.78	3.97	4.00	3.70	3.98	3.97	3.93	3.67	3.90	3.82	4.18	3.56	3.94	3.95	4.00	3.81	3.92	4.16	4.05
1387545_at	Mtr	5-methyltetrahydrofolate-homocysteine methyltransferase	0	uniqFlyp/lyp_183	0	0	1	0	0	0	0	0	0	0	3.91	4.02	3.72	3.88	4.02	3.81	3.95	3.96	3.74	3.82	4.10	4.26	3.86	3.97	3.71	4.11	3.88	3.92	4.13	4.15
1387607_at	Npffr2	neuropeptide FF receptor 2	0	uniqFlyp/lyp_183	0	0	1	0	0	0	0	0	0	0	3.48	3.40	3.55	3.56	3.41	3.62	3.70	3.40	3.30	3.54	3.79	4.06	3.55	3.57	3.69	3.80	3.66	3.65	3.91	4.07
1387700_at	Cckar	cholecystokinin A receptor	0	uniqFlyp/lyp_183	0	0	1	0	0	0	0	0	0	0	6.10	5.95	6.32	6.33	6.33	5.93	6.06	6.36	5.89	5.86	6.27	6.72	5.71	5.92	6.20	6.53	5.99	5.82	5.71	6.01
1387708_at	Adra2a	adrenoceptor alpha 2A	0	uniqFlyp/lyp_183	0	0	1	0	0	0	0	0	0	0	4.90	5.15	5.09	5.34	4.99	5.18	5.15	5.28	5.20	5.39	5.47	5.70	4.86	5.23	5.34	5.62	5.17	5.53	5.70	5.47
1387787_at	Mylpf	myosin light chain, phosphorylatable, fast skeletal muscle	0	uniqFlyp/lyp_183	0	0	1	0	0	0	0	0	0	0	3.14	3.17	3.23	3.04	3.20	3.17	3.12	3.38	2.92	3.12	3.48	3.43	2.89	3.22	3.22	3.40	2.94	3.09	3.00	3.38
1387816_at	Igfals	insulin-like growth factor binding protein, acid labile subunit	0	uniqFlyp/lyp_183	0	0	1	0	0	0	0	0	0	0	3.15	3.06	3.32	3.18	3.09	3.28	3.24	3.25	3.18	3.13	3.27	3.71	3.21	3.23	3.41	3.38	3.24	3.24	3.54	3.57
1387831_at	Xcl1	chemokine (C motif) ligand 1	0	uniqFlyp/lyp_183	0	0	1	0	0	0	0	0	0	0	4.07	3.85	3.95	4.78	3.96	4.01	4.18	4.24	3.81	4.04	4.07	4.34	4.06	4.19	4.33	4.75	4.21	4.16	4.19	4.37
1387833_at	Tmprss2	transmembrane protease, serine 2	0	uniqFlyp/lyp_183	0	0	1	0	0	0	0	0	0	0	5.01	5.14	5.28	5.12	5.03	5.12	5.24	4.85	4.76	5.09	5.16	5.36	4.77	5.08	4.97	5.23	5.05	5.14	5.19	5.14
1387970_at	Slc38a5	solute carrier family 38, member 5	0	uniqFlyp/lyp_183	0	0	1	0	0	0	0	0	0	0	10.78	10.56	10.66	10.75	10.93	10.72	10.63	10.80	10.61	10.60	10.81	11.11	10.39	10.54	10.75	10.98	10.77	10.76	10.94	11.10
1388011_a_at	Tgfb2	transforming growth factor, beta 2	0	uniqFlyp/lyp_183	0	0	1	0	0	0	0	0	0	0	6.76	6.72	6.83	6.79	6.59	6.95	6.86	6.68	6.44	6.50	6.81	6.95	6.16	6.81	6.74	6.69	6.48	6.85	6.64	6.70
1388190_at	Apob	apolipoprotein B	0	uniqFlyp/lyp_183	0	0	1	0	0	0	0	0	0	0	5.02	5.50	4.88	4.67	5.68	5.32	4.80	5.25	5.91	6.16	6.35	7.19	6.73	6.49	6.82	6.84	6.54	6.35	6.65	6.93
1388968_at	Mpped1	metallophosphoesterase domain containing 1	0	uniqFlyp/lyp_183	0	0	1	0	0	0	0	0	0	0	3.77	3.81	3.91	3.99	3.56	3.79	3.90	3.92	3.57	3.84	3.89	4.12	3.81	3.70	3.88	4.08	3.81	3.91	3.91	3.99
1389221_at	Mmd2	monocyte to macrophage differentiation-associated 2	0	uniqFlyp/lyp_183	0	0	1	0	0	0	0	0	0	0	6.36	6.54	6.90	6.58	6.30	6.52	6.70	6.50	5.51	5.81	5.89	6.09	5.66	5.95	6.00	5.94	5.76	5.56	5.84	5.57
1389811_at	Rasgef1c	RasGEF domain family, member 1C	0	uniqFlyp/lyp_183	0	0	1	0	0	0	0	0	0	0	4.79	5.39	5.63	5.25	4.77	5.55	5.23	5.02	3.98	4.35	4.55	4.42	4.20	4.17	4.51	4.57	4.01	4.40	4.44	4.18
1390667_at	Lrrc51	leucine rich repeat containing 51	0	uniqFlyp/lyp_183	0	0	1	0	0	0	0	0	0	0	3.78	4.06	4.06	4.17	3.92	4.19	4.07	4.00	3.74	4.11	4.27	4.39	3.60	4.26	4.20	4.09	3.62	4.27	4.28	4.29
1390846_at	Col16a1	collagen, type XVI, alpha 1	0	uniqFlyp/lyp_183	0	0	1	0	0	0	0	0	0	0	7.36	7.29	7.38	7.72	7.31	7.17	7.46	7.59	7.68	7.87	8.24	8.46	7.88	7.90	7.98	8.29	7.97	7.96	8.26	8.39
1391450_at	Loxl2	lysyl oxidase-like 2	0	uniqFlyp/lyp_183	0	0	1	0	0	0	0	0	0	0	7.43	7.62	7.57	7.61	7.74	7.71	7.46	7.50	7.51	7.66	8.16	7.90	6.96	7.71	8.11	7.78	7.59	8.13	7.63	7.84
1391648_at	Dscr6	Down syndrome critical region gene 6	0	uniqFlyp/lyp_183	0	0	1	0	0	0	0	0	0	0	8.13	8.33	8.55	8.45	8.23	8.36	8.29	8.53	8.06	8.26	8.33	8.57	8.12	8.10	8.49	8.57	8.13	8.22	8.35	8.55
1391869_at	---	---	0	uniqFlyp/lyp_183	0	0	1	0	0	0	0	0	0	0	6.59	6.72	6.73	6.65	6.58	6.69	6.95	6.71	6.47	6.58	6.75	7.02	6.35	6.66	6.75	6.80	6.54	6.65	6.87	6.85
1391933_at	Chtf8	CTF8, chromosome transmission fidelity factor 8 homolog (S. cerevisiae)	0	uniqFlyp/lyp_183	0	0	1	0	0	0	0	0	0	0	4.12	4.21	4.33	4.13	4.16	4.23	4.38	4.16	3.94	3.85	4.21	4.40	4.59	4.13	4.02	4.18	3.95	4.24	4.25	4.18
1392045_at	Slc35g2	solute carrier family 35, member G2	0	uniqFlyp/lyp_183	0	0	1	0	0	0	0	0	0	0	8.75	8.88	9.13	9.09	8.73	8.81	8.98	9.04	8.87	8.99	9.19	9.39	9.12	9.00	9.16	9.29	8.97	8.92	9.22	9.28
1392319_at	Lrrc10b	leucine rich repeat containing 10B	0	uniqFlyp/lyp_183	0	0	1	0	0	0	0	0	0	0	4.30	4.47	4.52	4.50	4.34	4.36	4.72	4.46	4.36	4.58	4.86	5.00	4.32	4.77	4.75	4.89	4.55	4.63	4.75	4.81
1392382_at	---	---	0	uniqFlyp/lyp_183	0	0	1	0	0	0	0	0	0	0	6.95	7.22	7.25	7.19	6.99	6.99	7.06	7.14	6.43	6.76	6.82	7.08	6.61	6.79	7.03	7.11	6.49	6.95	6.94	7.06
1392529_at	Spry4	sprouty homolog 4 (Drosophila)	0	uniqFlyp/lyp_183	0	0	1	0	0	0	0	0	0	0	6.73	6.87	6.91	6.82	6.78	6.90	6.99	6.84	6.44	6.79	6.98	6.85	6.74	6.91	6.80	7.02	6.77	6.95	7.07	7.00
1392701_at	Gmppb	GDP-mannose pyrophosphorylase B	0	uniqFlyp/lyp_183	0	0	1	0	0	0	0	0	0	0	6.17	5.96	6.10	6.33	6.18	6.07	6.17	6.21	6.45	6.09	6.30	6.62	6.38	6.28	6.23	6.53	6.54	6.34	6.22	6.57
1392823_at	Snhg11	small nucleolar RNA host gene 11 (non-protein coding)	0	uniqFlyp/lyp_183	0	0	1	0	0	0	0	0	0	0	10.13	10.18	10.16	10.25	10.22	10.06	10.12	9.94	9.64	10.02	10.02	10.26	9.49	9.78	9.87	10.27	9.86	9.91	10.17	10.18
1392832_at	---	---	0	uniqFlyp/lyp_183	0	0	1	0	0	0	0	0	0	0	2.62	2.94	2.93	2.73	2.64	2.69	2.66	2.87	2.49	2.71	2.83	3.01	2.67	2.59	3.05	2.72	2.70	2.89	2.90	2.84
1393389_at	Nr4a3	nuclear receptor subfamily 4, group A, member 3	0	uniqFlyp/lyp_183	0	0	1	0	0	0	0	0	0	0	4.50	4.43	4.48	4.78	4.52	4.59	5.16	5.02	4.32	4.78	5.27	5.09	4.16	4.78	4.73	5.15	4.44	5.28	5.31	4.97
1393452_at	Car9	carbonic anhydrase 9	0	uniqFlyp/lyp_183	0	0	1	0	0	0	0	0	0	0	3.92	4.06	4.20	4.29	4.10	4.18	4.34	4.16	4.01	4.08	4.33	4.67	3.97	4.18	4.22	4.30	3.93	4.25	4.36	4.36
1393713_at	Gmds	GDP-mannose 4, 6-dehydratase	0	uniqFlyp/lyp_183	0	0	1	0	0	0	0	0	0	0	7.03	7.10	7.11	7.17	7.01	7.01	7.02	7.19	6.94	7.22	7.27	7.48	7.09	7.28	7.19	7.31	7.01	7.18	7.37	7.45
1393904_at	Lin28a	lin-28 homolog A (C. elegans)	0	uniqFlyp/lyp_183	0	0	1	0	0	0	0	0	0	0	3.82	3.62	3.83	3.76	3.70	3.91	3.96	3.90	3.62	4.02	4.12	4.19	4.07	4.00	3.97	4.07	3.86	3.69	4.00	4.12
1394367_at	Dll4	delta-like 4 (Drosophila)	0	uniqFlyp/lyp_183	0	0	1	0	0	0	0	0	0	0	5.15	5.35	5.24	5.42	5.14	5.59	5.36	5.45	4.95	5.19	5.39	5.60	5.28	5.30	5.55	5.53	5.20	5.30	5.41	5.31
1394609_at	Ablim2	actin binding LIM protein family, member 2	0	uniqFlyp/lyp_183	0	0	1	0	0	0	0	0	0	0	3.56	3.86	4.00	3.68	3.67	3.80	3.98	3.68	3.66	3.99	4.10	4.42	3.88	3.99	4.10	4.02	3.89	4.15	4.12	4.22
1395249_at	Snhg11	small nucleolar RNA host gene 11 (non-protein coding)	0	uniqFlyp/lyp_183	0	0	1	0	0	0	0	0	0	0	9.39	9.46	9.49	9.53	9.44	9.37	9.33	9.35	8.65	9.07	9.01	9.21	8.63	8.74	8.90	9.41	8.84	9.04	9.10	9.27
1395329_at	Gmppb	GDP-mannose pyrophosphorylase B	0	uniqFlyp/lyp_183	0	0	1	0	0	0	0	0	0	0	5.22	5.00	5.28	5.31	5.14	4.99	5.30	5.28	5.34	5.25	5.53	5.94	5.17	5.27	5.39	5.66	5.56	5.36	5.54	5.69
1395403_at	Stac3	SH3 and cysteine rich domain 3	0	uniqFlyp/lyp_183	0	0	1	0	0	0	0	0	0	0	4.40	4.51	4.56	4.68	4.41	4.35	4.64	4.61	4.33	4.55	4.69	4.85	4.28	4.28	4.49	4.75	4.37	4.46	4.60	4.79
1395730_at	Gars	glycyl-tRNA synthetase	0	uniqFlyp/lyp_183	0	0	1	0	0	0	0	0	0	0	6.35	6.59	6.70	6.55	6.37	6.54	6.58	6.57	6.36	6.72	6.86	6.89	5.58	6.87	6.82	6.69	6.51	6.70	6.46	6.49
1395879_at	Ttc39b	tetratricopeptide repeat domain 39B	0	uniqFlyp/lyp_183	0	0	1	0	0	0	0	0	0	0	3.05	3.27	3.32	3.05	2.99	3.31	3.23	3.29	3.12	3.36	3.37	3.65	2.96	3.47	3.53	3.14	3.31	3.17	3.39	3.50
1395947_at	---	---	0	uniqFlyp/lyp_183	0	0	1	0	0	0	0	0	0	0	4.29	4.47	4.43	4.23	4.32	4.45	4.45	4.43	4.01	4.35	4.51	4.60	4.44	4.40	4.60	4.48	4.18	4.30	4.47	4.65
1396278_at	Snx11	sorting nexin 11	0	uniqFlyp/lyp_183	0	0	1	0	0	0	0	0	0	0	6.19	6.22	6.41	6.44	6.30	6.20	6.39	6.48	6.18	6.29	6.46	6.73	6.28	6.41	6.53	6.69	6.28	6.34	6.38	6.54
1396492_at	Nfam1	NFAT activating protein with ITAM motif 1	0	uniqFlyp/lyp_183	0	0	1	0	0	0	0	0	0	0	5.81	5.86	5.82	6.00	5.85	6.05	5.98	5.96	5.60	5.70	5.99	6.11	5.47	5.90	5.87	5.92	5.69	5.88	6.03	6.05
1396788_at	LOC1003655	rCG41957-like	0	uniqFlyp/lyp_183	0	0	1	0	0	0	0	0	0	0	4.31	4.11	4.17	4.39	4.00	4.15	4.53	4.18	3.89	4.09	4.33	4.58	4.02	4.26	4.36	4.25	4.03	4.17	4.52	4.50
1396789_x_at	LOC1003655	rCG41957-like	0	uniqFlyp/lyp_183	0	0	1	0	0	0	0	0	0	0	3.40	3.42	3.39	3.57	3.37	3.56	3.76	3.45	3.14	3.43	3.61	3.73	3.25	3.40	3.47	3.52	3.36	3.50	3.57	3.82
1396924_at	Mpped1	metallophosphoesterase domain containing 1	0	uniqFlyp/lyp_183	0	0	1	0	0	0	0	0	0	0	4.56	4.18	4.48	4.43	4.24	4.63	4.33	4.42	4.14	4.38	4.50	4.82	4.38	4.38	4.63	4.65	4.32	4.56	4.63	4.66
1396951_at	LOC1003621	hypothetical protein LOC100362109	0	uniqFlyp/lyp_183	0	0	1	0	0	0	0	0	0	0	3.93	3.96	3.98	4.18	4.09	4.06	4.26	4.05	4.01	4.25	4.16	4.56	4.00	4.04	4.13	4.36	4.10	4.12	4.09	4.32
1397180_at	Styxl1	serine/threonine/tyrosine interacting-like 1	0	uniqFlyp/lyp_183	0	0	1	0	0	0	0	0	0	0	5.51	5.80	5.97	5.83	5.62	5.85	5.91	5.59	5.31	5.53	5.70	5.84	5.56	5.45	5.51	5.66	5.48	5.44	5.61	5.69
1397241_at	Rimkla	ribosomal modification protein rimK-like family member A	0	uniqFlyp/lyp_183	0	0	1	0	0	0	0	0	0	0	3.52	3.95	4.00	4.06	3.90	4.14	4.21	4.15	3.66	3.96	4.07	4.48	3.81	3.91	4.02	4.27	3.60	3.82	4.15	4.12

Affymetrix Probeset ID	Gene Symbol	Gene Name	unique BB on P7 n=822	Figure 1B P7 Venn n=1526	DRlyp/lyp STEM P7 n=712	DR+/+ STEM P7 n=936	Flyp/lyp STEM P7 n=520	F+/+ STEM P7 n=465	Figure 1B P0 Venn n=1071	DRlyp/lyp STEM P0 n=442	DR+/+ STEM P0 n=437	Flyp/lyp STEM P0 n=643	F+/+ STEM P0 n=632	Fig1D n=25	Mean log2 intensity DRlyp/lyp 20	Mean log2 intensity DRlyp/lyp 30	Mean log2 intensity DRlyp/lyp 40	Mean log2 intensity DRlyp/lyp 50	Mean log2 intensity DR+/+ 20	Mean log2 intensity DR+/+ 30	Mean log2 intensity DR+/+ 40	Mean log2 intensity DR+/+ 50	Mean log2 intensity Flyp/lyp 20	Mean log2 intensity Flyp/lyp 30	Mean log2 intensity Flyp/lyp 40	Mean log2 intensity Flyp/lyp 50	Mean log2 intensity Flyp20	Mean log2 intensity Flyp30	Mean log2 intensity Flyp40	Mean log2 intensity Flyp50	Mean log2 intensity F+/+ 20	Mean log2 intensity F+/+ 30	Mean log2 intensity F+/+ 40	Mean log2 intensity F+/+ 50
1397246_at	Ntrk2	neurotrophic tyrosine kinase, receptor, type 2	0	uniqFlyp/lyp_183	0	0	1	0	0	0	0	0	0	0	8.48	8.63	8.73	8.92	8.50	8.41	8.66	8.76	7.73	7.78	8.10	8.41	8.47	8.05	8.14	8.41	7.68	7.80	8.32	8.38
1397492_at	Lmo7	LIM domain 7	0	uniqFlyp/lyp_183	0	0	1	0	0	0	0	0	0	0	3.84	3.73	3.91	3.84	3.88	3.82	3.94	3.81	3.78	3.88	4.10	4.22	3.82	3.97	3.83	4.20	3.72	4.06	4.15	4.13
1397636_at	Fam214b	family with sequence similarity 214, member B	0	uniqFlyp/lyp_183	0	0	1	0	0	0	0	0	0	0	4.92	4.93	4.79	4.92	4.97	4.99	5.15	4.89	4.75	5.14	5.19	5.36	4.86	4.98	5.01	5.07	4.72	5.11	5.12	5.11
1397733_at	Ogfod2	2-oxoglutarate and iron-dependent oxygenase domain containing 2	0	uniqFlyp/lyp_183	0	0	1	0	0	0	0	0	0	0	2.99	3.11	2.79	2.92	2.89	3.16	3.26	2.75	2.87	2.96	3.23	3.41	2.81	2.86	3.02	3.14	3.07	3.08	3.10	3.04
1397780_at	Armc2	armadillo repeat containing 2	0	uniqFlyp/lyp_183	0	0	1	0	0	0	0	0	0	0	4.10	3.95	4.03	4.08	4.04	4.13	4.17	3.90	3.80	3.99	4.10	4.36	3.95	4.03	4.07	4.18	3.94	4.04	4.10	4.05
1398664_at	Gramd3	GRAM domain containing 3	0	uniqFlyp/lyp_183	0	0	1	0	0	0	0	0	0	0	8.27	8.26	8.61	8.53	8.24	8.15	8.53	8.57	8.27	8.37	8.75	8.98	7.75	8.67	8.63	8.85	8.30	8.45	8.56	8.66
1368266_at	Arg1	arginase, liver	0	0	0	0	0	0	BBnF+/+_19	1	1	0	1	0	9.85	9.43	9.44	9.09	9.76	9.46	9.29	9.15	9.07	8.89	8.79	8.58	9.05	9.04	8.91	8.56	9.24	9.03	8.90	8.61
1368278_at	Lgals2	lectin, galactoside-binding, soluble, 2	0	0	0	0	0	0	BBnF+/+_19	1	1	0	1	0	6.44	6.25	6.15	5.81	6.48	6.16	6.21	5.92	5.66	5.61	5.42	5.53	5.88	5.77	5.71	5.46	5.85	5.72	5.61	5.35
1368629_at	Reg1a	regenerating islet-derived 1 alpha	0	0	0	0	0	0	BBnF+/+_19	1	1	0	1	0	12.17	11.63	8.88	8.33	11.70	11.42	8.99	9.71	11.88	11.49	11.66	11.48	12.92	12.80	11.99	11.00	10.13	11.51	9.86	9.02
1369041_at	Nlgn1	neuroligin 1	0	0	0	0	0	0	BBnF+/+_19	1	1	0	1	0	5.54	5.61	5.42	4.98	5.61	5.57	5.17	4.85	4.99	5.16	4.89	4.71	5.06	5.41	4.92	4.64	5.24	5.04	5.03	4.49
1370658_a_at	St18	suppression of tumorigenicity 18	0	0	0	0	0	0	BBnF+/+_19	1	1	0	1	0	8.79	8.37	8.37	7.87	8.55	8.55	8.27	7.50	8.85	8.67	8.65	8.79	8.19	8.68	8.57	8.36	8.86	8.88	8.62	7.88
1371369_at	Col6a2	collagen, type VI, alpha 2	0	0	0	0	0	0	BBnF+/+_19	1	1	0	1	0	5.20	4.94	4.61	4.75	5.26	4.74	4.72	4.59	5.12	4.82	4.68	4.75	5.02	4.64	4.56	4.56	5.24	4.91	4.89	4.53
1379226_at	Aim1l	absent in melanoma 1-like	0	0	0	0	0	0	BBnF+/+_19	1	1	0	1	0	5.06	4.76	4.84	4.46	5.19	4.91	4.74	4.54	4.97	4.99	4.88	4.57	5.21	4.63	4.57	4.61	5.23	4.92	4.81	4.68
1379409_at	Atxn7	ataxin 7	0	0	0	0	0	0	BBnF+/+_19	1	1	0	1	0	4.96	4.29	4.05	3.81	4.27	4.95	3.94	3.69	5.13	4.31	4.61	4.20	4.00	4.19	4.40	4.01	4.65	4.98	4.26	3.98
1379578_at	Zbtb20	zinc finger and BTB domain containing 20	0	0	0	0	0	0	BBnF+/+_19	1	1	0	1	0	10.00	9.78	9.67	9.60	9.96	9.76	9.63	9.55	9.90	9.74	9.65	9.50	9.74	9.62	9.55	9.53	9.88	9.67	9.54	9.41
1382083_at	Coch	cochlin	0	0	0	0	0	0	BBnF+/+_19	1	1	0	1	0	5.95	6.03	5.71	5.37	5.88	5.79	5.62	5.07	4.70	4.29	4.83	3.93	5.02	4.37	3.82	3.85	4.90	4.26	3.98	3.69
1382368_at	Trove2	TROVE domain family, member 2	0	0	0	0	0	0	BBnF+/+_19	1	1	0	1	0	5.70	5.52	5.48	5.12	5.44	5.83	4.99	4.98	6.50	6.27	6.50	6.03	5.12	6.27	6.72	5.87	6.33	6.58	5.64	5.57
1382524_at	Zbtb20	zinc finger and BTB domain containing 20	0	0	0	0	0	0	BBnF+/+_19	1	1	0	1	0	6.51	6.32	6.14	6.00	6.61	6.03	5.95	6.17	6.61	6.24	6.19	6.13	5.58	6.28	6.14	6.01	6.59	6.33	5.98	5.88
1387803_at	Ppp2r2b	protein phosphatase 2, regulatory subunit B, beta	0	0	0	0	0	0	BBnF+/+_19	1	1	0	1	0	7.59	7.22	7.26	6.86	7.66	7.45	6.95	6.73	6.63	7.01	6.64	6.44	6.52	6.77	6.52	6.35	6.89	6.90	6.75	6.36
1389350_at	Apoh	apolipoprotein H (beta-2-glycoprotein I)	0	0	0	0	0	0	BBnF+/+_19	1	1	0	1	0	7.73	7.46	6.96	6.70	6.83	7.09	6.74	6.37	6.35	6.65	6.34	6.34	5.85	6.17	6.18	5.81	6.51	6.23	6.13	5.77
1389444_at	Zdhhc20	zinc finger, DHHC-type containing 20	0	0	0	0	0	0	BBnF+/+_19	1	1	0	1	0	9.03	8.69	8.73	8.37	8.64	9.20	8.46	8.34	9.27	9.00	9.04	8.83	9.15	9.07	9.11	8.55	9.02	9.19	8.66	8.64
1392206_at	Slc35f4	solute carrier family 35, member F4	0	0	0	0	0	0	BBnF+/+_19	1	1	0	1	0	6.86	6.91	6.66	6.21	6.64	6.76	6.55	6.04	5.56	5.42	5.32	5.26	5.69	5.45	5.40	5.09	5.59	5.32	5.41	5.08
1393891_at	Col8a1	collagen, type VIII, alpha 1	0	0	0	0	0	0	BBnF+/+_19	1	1	0	1	0	6.75	6.65	6.37	6.21	6.90	6.74	6.33	6.22	6.77	6.44	6.35	6.27	6.26	6.37	6.37	6.12	6.84	6.62	6.25	6.16
1394849_at	Zbtb20	zinc finger and BTB domain containing 20	0	0	0	0	0	0	BBnF+/+_19	1	1	0	1	0	7.09	6.75	6.43	6.22	6.90	6.82	6.30	6.11	6.99	6.74	6.68	6.17	5.85	6.70	6.63	6.23	7.02	6.86	6.03	6.28
1394972_at	Trove2	TROVE domain family, member 2	0	0	0	0	0	0	BBnF+/+_19	1	1	0	1	0	5.56	5.60	5.31	4.95	5.54	5.66	5.29	4.98	6.07	5.94	6.43	5.85	4.83	6.20	6.44	5.66	6.07	6.22	5.37	5.26
1367985_at	Alas2	aminolevulinate, delta-, synthase 2	0	0	0	0	0	0	BBnFlyp/lyp_29	1	1	1	0	0	4.52	4.41	4.09	3.99	4.31	4.63	4.14	3.99	4.76	4.17	4.08	4.04	3.85	4.31	3.97	3.99	4.34	4.23	4.36	4.15
1368239_at	Lrp3	low density lipoprotein receptor-related protein 3	0	0	0	0	0	0	BBnFlyp/lyp_29	1	1	1	0	0	7.07	6.78	6.79	6.66	6.97	6.87	6.70	6.56	7.05	6.90	6.78	6.72	7.00	6.84	6.92	6.65	6.95	6.82	6.67	6.74
1368374_a_at	Ggt1	gamma-glutamyltransferase 1	0	0	0	0	0	0	BBnFlyp/lyp_29	1	1	1	0	0	6.26	6.32	5.49	5.52	6.50	5.91	5.78	5.71	6.98	6.08	5.94	5.78	6.98	7.20	6.09	5.62	6.46	6.90	5.97	6.31
1368378_at	Aldh1l1	aldehyde dehydrogenase 1 family, member L1	0	0	0	0	0	0	BBnFlyp/lyp_29	1	1	1	0	0	4.57	4.42	3.98	4.10	4.97	4.51	4.28	4.13	5.37	4.47	4.21	4.28	5.20	5.25	4.26	4.15	4.79	4.99	4.40	4.70
1368403_at	Rbl2	retinoblastoma-like 2	0	0	0	0	0	0	BBnFlyp/lyp_29	1	1	1	0	0	8.68	8.23	8.14	7.93	8.68	8.34	7.99	8.09	8.51	8.09	7.94	7.77	8.58	8.16	8.15	7.73	8.43	8.21	8.04	7.95
1368658_at	Cntf	ciliary neurotrophic factor	0	0	0	0	0	0	BBnFlyp/lyp_29	1	1	1	0	0	5.87	5.67	5.58	5.19	5.70	5.89	5.44	5.38	6.16	6.10	6.05	5.65	6.51	5.85	6.02	5.72	6.16	5.98	6.01	5.94
1368900_at	Thbd	thrombomodulin	0	0	0	0	0	0	BBnFlyp/lyp_29	1	1	1	0	0	2.97	2.91	2.95	3.05	3.09	3.15	3.34	2.98	3.03	2.88	3.08	3.04	2.86	3.03	3.06	3.05	2.90	2.99	3.00	3.02
1368901_at	Thbd	thrombomodulin	0	0	0	0	0	0	BBnFlyp/lyp_29	1	1	1	0	0	4.27	3.67	3.63	3.44	4.20	3.67	3.67	3.62	4.30	3.44	3.56	3.40	4.19	3.65	3.50	3.31	3.98	3.47	3.57	3.57
1369178_a_at	P2rx1	purinergic receptor P2X, ligand-gated ion channel, 1	0	0	0	0	0	0	BBnFlyp/lyp_29	1	1	1	0	0	5.29	5.08	4.10	3.91	5.40	4.69	4.31	4.61	6.17	5.05	4.70	4.57	6.19	6.50	4.95	4.17	5.66	5.87	4.95	5.35
1373093_at	Errfi1	ERBB receptor feedback inhibitor 1	0	0	0	0	0	0	BBnFlyp/lyp_29	1	1	1	0	0	10.65	10.64	10.17	9.78	10.68	10.49	10.01	9.87	10.86	10.80	10.53	10.19	10.76	10.54	10.17	10.21	10.47	10.97	10.66	9.89
1375951_at	Thbd	thrombomodulin	0	0	0	0	0	0	BBnFlyp/lyp_29	1	1	1	0	0	6.48	5.69	5.69	5.70	6.54	5.56	5.78	6.07	6.28	5.47	5.32	5.25	6.46	5.44	5.49	5.33	6.36	5.81	5.59	5.61
1376460_at	---	---	0	0	0	0	0	0	BBnFlyp/lyp_29	1	1	1	0	0	4.09	3.94	3.81	3.40	4.18	4.07	3.72	3.54	4.15	3.88	3.60	3.45	4.26	3.87	3.74	3.53	3.86	3.84	3.80	3.60
1377095_at	Rbfox3	RNA binding protein, fox-1 homolog (C. elegans) 3	0	0	0	0	0	0	BBnFlyp/lyp_29	1	1	1	0	0	6.29	6.04	6.00	5.47	6.22	5.98	5.75	5.45	6.61	6.19	5.81	5.71	6.55	5.98	5.62	5.35	6.17	6.01	5.86	5.58
1377623_at	Mex3d	mex-3 homolog D (C. elegans)	0	0	0	0	0	0	BBnFlyp/lyp_29	1	1	1	0	0	7.35	7.04	6.86	6.74	7.23	7.06	6.99	6.72	7.38	7.11	6.84	6.76	7.54	6.97	6.84	6.80	7.20	6.92	6.72	6.81
1378126_at	Dennd2a	DENN/MADD domain containing 2A	0	0	0	0	0	0	BBnFlyp/lyp_29	1	1	1	0	0	7.39	7.02	6.89	6.64	7.32	6.93	6.90	6.49	6.82	6.32	6.16	5.92	7.16	6.40	6.19	6.03	7.25	6.88	6.95	6.70
1378136_at	Lrp3	low density lipoprotein receptor-related protein 3	0	0	0	0	0	0	BBnFlyp/lyp_29	1	1	1	0	0	6.18	5.78	5.74	5.47	6.13	5.80	5.63	5.40	6.33	5.88	5.75	5.55	6.22	5.88	5.78	5.34	6.08	5.79	5.59	5.65
1379281_at	Sostdc1	sclerostin domain containing 1	0	0	0	0	0	0	BBnFlyp/lyp_29	1	1	1	0	0	5.43	5.33	4.63	4.55	5.53	5.31	4.61	4.92	5.41	5.05	4.61	4.40	5.70	5.37	4.78	4.31	4.96	5.16	4.60	4.71
1383311_at	Mtmr11	myotubularin related protein 11	0	0	0	0	0	0	BBnFlyp/lyp_29	1	1	1	0	0	6.83	6.72	6.68	6.33	6.92	6.70	6.52	6.13	7.08	6.83	6.71	6.50	7.09	6.77	6.54	6.57	7.06	6.68	6.76	6.58
1383887_at	Lamp5	lysosomal-associated membrane protein family, member 5	0	0	0	0	0	0	BBnFlyp/lyp_29	1	1	1	0	0	8.18	7.70	7.78	7.62	8.18	7.67	7.57	7.62	8.10	7.87	7.67	7.56	8.15	7.32	7.43	7.38	7.90	7.41	7.63	7.37
1384210_at	Micalcl	MICAL C-terminal like	0	0	0	0	0	0	BBnFlyp/lyp_29	1	1	1	0	0	3.61	3.31	3.25	2.77	3.65	3.27	3.09	2.88	3.32	2.94	2.61	2.84	3.33	2.95	2.82	2.89	3.25	2.93	2.79	2.77
1384305_at	Slc45a1	solute carrier family 45, member 1	0	0	0	0	0	0	BBnFlyp/lyp_29	1	1	1	0	0	5.41	5.23	5.09	4.68	5.73	5.38	5.13	4.89	5.80	5.50	5.40	5.13	6.25	5.60	5.26	5.19	5.64	5.36	5.41	5.24
1385148_at	P2rx1	purinergic receptor P2X, ligand-gated ion channel, 1	0	0	0	0	0	0	BBnFlyp/lyp_29	1	1	1	0	0	6.10	5.89	5.36	5.45	6.28	5.73	5.45	5.62	6.45	5.61	5.56	5.54	6.92	6.38	5.59	5.38	6.37	6.07	5.75	5.79
1387672_at	Gnmt	glycine N-methyltransferase	0	0	0	0	0	0	BBnFlyp/lyp_29	1	1	1	0	0	6.36	6.19	5.82	5.50	6.46	6.09	5.79	5.64	6.93	6.09	5.90	5.84	6.87	6.70	5.76	5.56	6.40	6.47	5.88	6.06
1390404_at	Lama2	laminin, alpha 2	0	0	0	0	0	0	BBnFlyp/lyp_29	1	1	1	0	0	6.67	6.33	6.07	5.73	6.92	6.45	6.10	6.04	6.73	6.26	6.11	5.98	6.99	6.26	6.26	6.05	6.67	6.34	6.22	6.18
1390591_at	Slc17a3	solute carrier family 17 (sodium phosphate), member 3	0	0	0	0	0	0	BBnFlyp/lyp_29	1	1	1	0	0	3.86	3.50	3.19	2.77	4.12	3.38	3.02	3.05	3.30	2.84	2.84	2.64	3.70	2.90	2.83	2.71	3.05	2.79	2.92	2.64
1392267_at	Dennd2a	DENN/MADD domain containing 2A	0	0	0	0	0	0	BBnFlyp/lyp_29	1	1	1	0	0	6.38	6.23	6.13	5.96	6.39	6.24	6.06	5.89	6.06	5.76	5.76	5.75	6.12	5.75	5.61	5.75	6.40	6.25	6.06	6.04
1393383_at	Arhgdig	Rho GDP dissociation inhibitor (GDI) gamma	0	0	0	0	0	0	BBnFlyp/lyp_29	1	1	1	0	0	6.41	6.51	5.88	5.85	6.84	6.31	5.92	6.24	6.86	6.29	6.20	5.86	6.81	6.78	6.05	5.81	6.48	6.56	6.17	6.24
1393874_at	Aqp12a	aquaporin 12A	0	0	0	0	0	0	BBnFlyp/lyp_29	1	1	1	0	0	7.02	7.24	6.38	6.40	7.60	7.07	6.56	6.85	7.91	6.90	6.87	6.83	7.68	7.94	6.96	6.56	7.33	7.67	7.00	7.36
1395774_at	Gpr153	G protein-coupled receptor 153	0	0	0	0	0	0	BBnFlyp/lyp_29	1	1	1	0	0	4.89	4.42	4.43	4.16	4.80	4.64	4.35	4.07	4.65	4.47	4.39	4.02	4.47	4.47	4.35	4.03	4.59	4.52	4.48	4.20
1367553_x_at	Hbb	hemoglobin, beta	0	0	0	0	0	0	Common_171	1	1	1	1	0	10.84	11.01	10.24	9.71	10.74	11.18	10.06	9.75	11.22	10.48	10.01	8.91	9.71	10.90	10.01	8.97	10.73	10.71	10.10	9.23
1367601_at	Cited2	Cbp/p300-interacting transactivator, with Glu/Asp-rich carboxy-terminal domain, 2	0	0	0	0	0	0	Common_171	1	1	1	1	0	9.37	9.44	9.19	8.78	9.61	9.46	9.02	8.87	9.51	9.31	9.01	8.59	9.49	8.91	8.86	8.57	9.29	9.23	8.96	8.29
1367602_at	Cited2	Cbp/p300-interacting transactivator, with Glu/Asp-rich carboxy-terminal domain, 2	0	0	0	0	0	0	Common_171	1	1	1	1	0	10.10	10.14	9.91	9.52	10.37	10.19	9.76	9.61	10.13	9.90	9.71	9.28	10.34	9.53	9.55	9.16	10.00	9.92	9.84	9.08
1367647_at	Serpina1	serpin peptidase inhibitor, clade A (alpha-1 antiproteinase, antitrypsin), member 1	0	0	0	0	0	0	Common_171	1	1	1	1	0	8.30	7.70	7.47	7.12	8.20	7.64	7.35	7.03	8.23	7.77	7.55	7.34	8.09	7.69	7.62	7.16	8.32	7.71	7.48	7.24
1367648_at	Igfbp2	insulin-like growth factor binding protein 2	0	0	0	0	0	0	Common_171	1	1	1	1	0	7.67	6.66	6.27	5.43	7.43	6.72	6.43	5.66	8.23	6.92	6.22	5.91	7.59	7.03	6.21	5.64	8.10	6.98	6.19	5.83
1367650_at	Tinagl1	tubulointerstitial nephritis antigen-like 1	0	0	0	0	0	0	Common_171	1	1	1	1	0	8.62	8.22	8.02	7.76	8.59	8.11	8.11	7.79	8.95	8.55	8.18	7.86	8.81	8.58	8.12	7.86	8.86	8.46	8.06	7.78
1367689_a_at	Cd36	CD36 molecule (thrombospondin receptor)	0	0	0	0	0	0	Common_171	1	1	1	1	0	8.54	8.21	8.04	7.61	8.40	7.97	7.99	7.86	8.85	8.51	8.17	7.70	8.53	8.46	8.21	7.73	8.64	8.13	7.63	7.36
1367802_at	Sgk1	serum/glucocorticoid regulated kinase 1	0	0	0	0	0	0	Common_171	1	1	1	1	0	9.30	9.03	8.93	8.76	9.35	8.99	8.96	8.77	9.16	8.81	8.68	8.55	9.36	8.85	8.66	8.59	9.44	9.32	9.16	8.85
1367835_at	LOC10091128	proSAAS-like /// proprotein convertase subtilisin/kexin type 1 inhibitor	0	0	0	0	0	0	Common_171	1	1	1	1	0	10.73	10.48	10.39	10.22	10.81	10.59	10.33	10.10	10.68	10.27	10.22	10.02	10.56	10.35	10.19	10.00	10.54	10.29	10.14	10.02
1368023_at	Qsox1	quiescin Q6 sulfhydryl oxidase 1	0	0	0	0	0	0	Common_171	1	1	1	1	0	7.45	7.23	7.11	6.61	7.28	7.38	7.00	6.61	7.45	7.15	7.00	6.45	7.30	7.38	6.95	6.54	7.49	7.30	6.64	6.33
1368024_at	Qsox1	quiescin Q6 sulfhydryl oxidase 1	0	0	0	0	0	0	Common_171	1	1	1	1	0	6.81	6.78	6.59	6.14	6.64	6.70	6.56	6.29	6.63	6.59	6.34	6.10	6.06	6.77	6.27	5.85	6.79	6.58	5.72	5.63
1368160_at	Igfbp1	insulin-like growth factor binding protein 1	0	0	0	0	0	0	Common_171	1	1	1	1	0	9.36	8.81	8.54	7.79	9.34	8.70	8.57	7.67	9.64	8.93	8.55	7.85	9.39	9.06	8.61	7.77	9.40	8.94	8.46	7.66
1368171_at	Lox	lysyl oxidase	0	0	0	0	0	0	Common_171	1	1	1	1	0	7.03	6.68	6.45	6.11	7.13	6.48	6.47	6.39	6.37	5.85	5.30	5.54	6.57	5.95	5.85	5.62	6.28	5.86	5.94	5.57
1368172_a_at	Lox	lysyl oxidase	0	0	0	0	0	0	Common_171	1	1	1	1	0	7.11	6.56	6.38	6.27	6.88	6.57	6.38	6.50	6.21	5.64	5.20	5.23	5.87	5.69	5.82	5.75	6.12	5.75	5.47	5.44
1368250_at	Tekt1	tektin 1	0	0	0	0	0	0	Common_171	1	1	1	1	0	5.69	5.28	5.12	4.72	5.69	5.28	4.94	4.75	6.23	5.79	5.44	5.32	6.19	5.50	5.38	5.09	6.15	5.52	5.50	5.09
1368253_at	Gamt	guanidinoacetate N-methyltransferase	0	0	0	0	0	0	Common_171	1	1	1	1	0	7.07	6.90	6.67	6.50	7.41	6.95	6.53	6.79	7.75	7.01	6.92	6.70	7.78	7.42	6.95	6.64	7.61	7.27	6.84	6.97
1368281_at	Dpep1	dipeptidase 1 (renal)	0	0	0	0	0	0	Common_171	1	1	1	1	0	6.97	6.66	6.29	5.99	6.80	6.62	6.34	6.28	6.83	6.25	6.19	6.11	6.93	6.80	6.19	6.07	6.66	6.60	6.40	6.27
1368282_at	Dpep1	dipeptidase 1 (renal)	0	0	0	0	0	0	Common_171	1	1	1	1	0	5.36	5.11	5.15	4.91	5.40	5.19	4.97	4.90	5.43	4.81	4.64	4.51	5.46	4.95	4.80	4.76	5.37	5.07	4.96	4.62
1368320_at	Ncam1	neural cell adhesion molecule 1	0	0	0	0	0	0	Common_171	1	1	1	1	0	7.47	7.23	7.09	6.85	7.35	7.24	6.91	6.73	7.45	7.20	7.00	6.68	7.51	7.02	7.01	6.70	7.41	7.11	6.82	6.74
1368524_at	Kcnc1	potassium voltage gated channel, Shaw-related subfamily, member 1	0	0	0	0	0	0	Common_171	1	1	1	1	0	4.48	4.44	4.06	3.59	4.45	4.03	3.99	3.94	4.84	4.51	4.24	3.94	4.51	4.30	3.87	3.84	4.86	4.17	3.92	3.82
1368539_at	Scn9a	sodium channel, voltage-gated, type IX, alpha	0	0	0	0	0	0	Common_171	1	1	1	1	0	8.51	8.39	8.04	7.84	8.25	8.30	7.95	7.78	8.39	8.10	7.51	6.74	8.34	7.77	7.51	7.13	8.41	7.66	7.29	7.03
1368540_at	Tpbg	trophoblast glycoprotein	0	0	0	0	0	0	Common_171	1	1	1	1	0	4.77	4.29	3.95	3.53	4.40	4.07	3.85	3.60	4.35	3.44	3.06	3.03	4.62	3.37	3.40	3.15	4.34	3.55	3.42	2.93
1368577_at	Gjb6	gap junction protein, beta 6	0	0	0	0	0	0	Common_171	1	1	1	1	0	5.53	5.55	4.54	3.77	4.99	5.04	4.62	3.18	7.19	7.00	5.73	4.77	5.74	5.92	5.47	4.74	6.97	5.77	5.43	4.08
1368662_at	Rnf39	ring finger protein 39	0	0	0	0	0	0	Common_171	1	1	1	1	0	6.92	6.71	6.44	6.14	6.99	6.68	6.71	6.28	7.46	6.88	6.85	6.27	7.35	6.89	6.76	6.35	7.34	6.72	6.60	6.30
1368691_at	Gria3	glutamate receptor, ionotropic, AMPA 3	0	0	0	0	0	0	Common_171	1	1	1	1	0	9.03	8.54	8.31	7.95	9.17	8.43	8.11	7.77	9.14	8.52	8.26	7.60	8.72	8.27	7.90	7.56	9.12	8.26	7.91	7.55
1368700_at	Plcl1	phospholipase C-like 1	0	0	0	0	0	0	Common_171	1	1	1	1	0	7.98	7.73	7.55	7.16	8.00	7.73	7.40	7.27	8.05	7.63	7.46	6.99	8.12	7.70	7.51	7.05	8.05	7.70	7.26	7.16
1368778_at	Slc6a6	solute carrier family 6 (neurotransmitter transporter, taurine), member 6	0	0	0	0	0	0	Common_171	1	1	1	1	0	9.34	8.85	8.76	8.62	9.29	9.03	8.58	8.58	9.39	8.70	8.88	8.50	8.63	8.80	8.82	8.57	9.16	8.99	9.05	8.63
1368912_at	Trh	thyrotropin releasing hormone	0	0	0	0	0	0	Common_171	1	1	1	1	0	11.60	11.04	11.14	10.83	11.56	11.12	11.09	10.68	11.57	11.30	11.31	10.95	11.80	11.08	11.12	10.96	11.45	11.13	11.05	10.69
1368964_at	Lrrn3	leucine rich repeat neuronal 3	0	0	0	0	0	0	Common_171	1	1	1	1	0	7.43	7.32	7.47	6.73	7.37	7.25	7.14	6.77	7.42	7.16	7.09	6.48	7.83	7.26	6.95	6.40	7.36	7.13	7.03	6.65
1369150_at	Pdk4	pyruvate dehydrogenase kinase, isozyme 4	0	0	0	0	0	0	Common_171	1	1	1	1	0	7.44	7.33	7.06	6.67	7.55	7.20	6.93	6.83	7.30	6.80	6.75	6.51	6.38	6.96	6.64	6.35	7.33	6.98	6.57	6.21
1369291_at	Agtr1a	angiotensin II receptor, type 1a	0	0	0	0	0	0	Common_171	1	1	1	1	0	5.76	5.38	5.19	4.97	5.78	5.35	5.22	5.04	5.77	5.38	5.25	5.04	5.84	5.53	5.26	5.16	5.84	5.46	5.32	5.17
1369692_at	Tnr	tenascin R	0	0	0	0	0	0	Common_171	1	1	1	1	0	5.79	5.27	5.06	4.66	5.60	5.19	4.79	4.62	5.67	5.22	4.74	4.50	5.60	5.04	4.77	4.41	5.81	5.02	4.90	4.52
1369696_at	LOC10090965	ras-related GTP-binding protein B-like /// Ras-related GTP binding B	0	0	0	0	0	0	Common_171	1	1	1	1	0	9.63	9.35	9.30	8.91	9.69	9.44	9.09	8.93	9.41	9.16	9.00	8.78	9.37	9.05	9.07	8.76	9.43	9.12	9.01	8.72
1369725_at	Adap2	ArfGAP with dual PH domains 2	0	0	0	0	0	0	Common_171	1	1	1	1	0	7.09	6.80	6.58	6.61	7.04	6.63	6.45	6.53	7.25	6.91	6.75	6.60	7.28	6.94	6.79	6.56	7.33	6.82	6.88	6.51
1369837_at	Gulo	gulonolactone (L-) oxidase	0	0	0	0	0	0	Common_171	1	1	1	1	0	6.34	6.07	5.79	5.40	6.52	6.09	5.71	5.31	6.56	5.99	5.71	5.30	6.24	5.82	5.68	5.24	6.49	5.84	5.67	5.34
1369949_at	Bcam	basal cell adhesion molecule (Lutheran blood group)	0	0	0	0	0	0	Common_171	1	1	1	1	0	7.72	7.05	6.95	6.74	7.68	7.18	7.02	6.71	8.02	7.52	7.11	6.74	7.93	7.38	7.05	6.66	7.90	7.35	7.04	6.78
1369999_a_at	Nnat	neuronatin	0	0	0	0	0	0	Common_171	1	1	1	1	0	10.42	10.01	9.74	9.53	10.49	9.85	9.68	9.23	11.32	10.50	10.05	9.77	11.12	10.09	9.87	9.63	11.15	10.18	9.97	9.41
1370062_at	Higd1a	HIG1 hypoxia inducible domain family, member 1A	0	0	0	0	0	0	Common_171	1	1	1	1	0	9.01	8.85	8.82	8.71	9.13	9.01	8.77	8.91	9.05	8.90	8.86	8.45	9.11	8.93	8.77	8.45	9.13	8.89	8.70	8.39
1370239_at	Hba1 /// Hba	hemoglobin, alpha 1 /// hemoglobin, alpha 2	0	0	0	0	0	0	Common_171	1	1	1	1	0	11.96	11.96	11.37	10.86	11.82	12.19	11.23	10.84	12.18	11.57	11.16	9.97	11.05	11.64	11.12	10.21	11.86	11.62	11.26	10.40
1370240_x_at	Hba1 /// Hba	hemoglobin, alpha 1 /// hemoglobin, alpha 2	0	0	0	0	0	0	Common_171	1	1	1	1	0	12.12	12.17	11.62	11.13	12.06	12.44	11.43	11.05	12.39	11.81	11.38	10.27	11.24	11.97	11.36	10.44	12.02	11.92	11.51	10.65
1370379_at	Prss8	protease, serine, 8	0	0	0	0	0	0	Common_171	1	1	1	1	0	5.59	5.29	5.01	4.90	5.69	5.38	5.38	5.03	5.29	4.79	4.61	4.40	5.27	4.89	4.80	4.52	5.27	4.96	4.60	4.68
1370438_at	Nos1ap	nitric oxide synthase 1 (neuronal) adaptor protein	0	0	0	0	0	0	Common_171	1	1	1	1	0	6.26	5.91	5.83	5.63	6.22	5.94	5.86	5.56	6.29	5.85	5.76	5.64	5.99	5.84	5.63	5.49	6.04	5.94	5.99	5.53
1370491_a_at	Hdc	histidine decarboxylase	0	0	0	0	0	0	Common_171	1	1	1	1	0	7.71	7.12	6.48	6.21	7.32	6.81	6.76	5.87	8.14	7.06	6.72	6.15	7.50	7.02	6.38	6.01	8.23	6.82	6.65	6.46
1370555_at	Nalcn	sodium leak channel, non-selective	0	0	0	0	0	0	Common_171	1	1	1	1	0	8.54	8.35	8.13	7.82	8.37	8.40	8.09	7.75	8.21	8.05	7.93	7.35	8.27	8.13	7.84	7.45	8.19	8.04	7.79	7.59
1370707_at	Fev	FEV (ETS oncogene family)	0	0	0	0	0	0	Common_171	1	1	1	1	0	7.66	7.31	6.98	6.72	7.55	7.36	7.08	6.69	7.44	7.19	7.02	6.56	7.41	7.21	6.96	6.52	7.50	7.15	7.00	6.64
1370895_at	Col5a2	collagen, type V, alpha 2	0	0	0	0	0	0	Common_171	1	1	1	1	0	8.11	7.87	7.61	7.34	8.14	7.92	7.59	7.64	7.82	7.51	7.35	7.14	8.11	7.57	7.45	7.33	7.86	7.65	7.46	7.38
1371021_at	Arsb	arylsulfatase B	0	0	0	0	0	0	Common_171	1	1	1	1	0	7.21	7.22	7.15	6.68	7.13	7.20	6.95	6.64	7.33	7.16	6.99	6.53	7.02	7.27	7.07	6.51	7.34	7.20	6.88	6.41
1371102_x_at	LOC10013487	beta globin minor gene /// beta-globin	0	0	0	0	0	0	Common_171	1	1	1	1	0	9.60	9.68	8.92	8.35	9.54	9.88	8.69	8.43	9.95	9.00	8.52	7.31	8.26	9.38	8.46	7.33	9.26	9.19	8.59	7.65
1371245_a_at	LOC10013487	beta globin minor gene /// beta-globin	0	0	0	0	0	0	Common_171	1	1	1	1	0	9.88	9.90	9.26	8.72	9.90	10.26	9.16	8.76	8.59	7.38	7.00	5.84	6.94	7.57	6.99	5.91	7.60	7.50	7.03	6.12
1371762_at	Rbp4	retinol binding protein 4, plasma	0	0	0	0	0	0	Common_171	1	1	1	1	0	9.14	8.89	8.62	8.20	9.06	9.02	8.40	8.16	10.07	9.68	9.35	8.89	10.02	9.73	9.42	8.86	10.21	9.66	9.23	8.77
1371815_at	Mfap2	microfibrillar-associated protein 2	0	0	0	0	0	0	Common_171	1	1	1	1	0	6.56	5.48	5.06	4.98	6.57	5.56	5.16	5.03	6.17	5.33	5.24	4.89	6.61	5.14	5.15	5.20	6.36	5.36	5.27	5.19
1371849_at	Nt5dc2	5'-nucleotidase domain containing 2	0	0	0	0	0	0	Common_171	1	1	1	1	0	7.84	7.61	7.45	7.16	7.78	7.59	7.38	7.10	7.93	7.63	7.37	6.93	7.63	7.52	7.30	6.87	7.99	7.61	7.22	6.95
1372248_at	Sesn1	sestrin 1	0	0	0	0	0	0	Common_171	1	1	1	1	0	8.72	8.58	8.24	7.89	8.65	8.47	8.07	8.04	8.94	8.61	8.31	7.87	8.84	8.67	8.11	7.79	8.93	8.65	8.20	7.83
1372299_at	Cdkn1c	cyclin-dependent kinase inhibitor 1C	0	0	0	0	0	0	Common_171	1	1	1	1	0	7.33	6.66	6.51	6.01	7.38	6.66	6.49	6.12	7.25	6.79	6.39	6.00	7.55	6.70	6.39	5.85	7.08	6.63	6.21	5.80
1372410_at	C1qtnf6	C1q and tumor necrosis factor related protein 6	0	0	0	0	0	0	Common_171	1	1	1	1	0	8.00	7.71	7.46	7.29	7.96	7.59	7.31	7.26	8.27	7.80	7.58	7.35	8.29	7.73	7.35	7.30	8.09	7.63	7.53	7.27
1372439_at	Col4a1	collagen, type IV, alpha 1	0	0	0	0	0	0	Common_171	1	1	1	1	0	8.62	8.33	7.92	7.91	8.74	8.38	7.94	7.87	8.71	8.26	8.13	8.07	8.86	8.37	8.24	8.03	8.77	8.44	8.35	8.22
1372592_at	Hdac6 /// LOC	histone deacetylase 6 /// histone deacetylase 6-like	0	0	0	0	0	0	Common_171	1	1	1	1	0	8.39	8.17	7.92	7.67	8.43	8.24	7.82	7.68	8.56	8.19	7.83	7.61	8.52	8.05	7.86	7.53	8.38	8.18	7.93	7.49
1372621_at	Eva1b	eva-1 homolog B	0	0	0	0	0	0	Common_171	1	1	1	1	0	6.32	6.08	6.02	5.76	6.41	6.16	5.89	6.06	6.56	6.36	6.17	5.94	6.71	6.29	6.20	6.00	6.54	6.13	6.11	5.94
1372637_at	LOC683751	similar to trophinin isoform 1	0	0	0	0	0	0	Common_171	1	1	1	1	0	6.82	6.76	6.68	6.24	6.89	6.78	6.55	6.18	7.23	7.02	6.77	6.41	7.30	6.99	6.81	6.43	7.16	6.86	6.66	6.40
1373245_at	Col4a1	collagen, type IV, alpha 1	0	0	0	0	0	0	Common_171	1	1	1	1	0	8.80	8.57	8.24	8.16	8.86	8.61	8.21	8.17	8.85	8.49	8.40	8.25	8.89	8.61	8.47	8.29	8.88	8.69	8.52	8.41
1373282_at	Slc25a33	solute carrier family 25 (pyrimidine nucleotide carrier), member 33	0	0	0	0	0	0	Common_171	1	1	1	1	0	6.39	6.06	5.72	5.31	6.43	6.02	5.67	5.67	6.11	5.66	5.40	5.17	6.09	5.66	5.33	5.08	6.05	5.65	5.44	4.99
1373463_at	Col5a2	collagen, type V, alpha 2	0	0	0	0	0	0	Common_171	1	1	1	1	0	7.88	7.32	6.90	6.55	7.94	7.49	6.73	7.01	7.48	7.00	6.68	6.53	7.90	7.01	6.92	6.67	7.53	7.14	6.85	6.65
1373773_at	Gpm6a	glycoprotein m6a	0	0	0	0	0	0	Common_171	1	1	1	1	0	6.02	6.05	6.01	5.54	6.11	5.88	5.53	5.53	5.99	5.93	5.64	5.28	5.74	5.68	5.63	5.24	6.05	5.79	5.59	5.20

Affymetrix Probeset ID	Gene Symbol	Gene Name	unique BB on P7 n=822	Figure 1B P7 Venn n=1526	DRlyp/lyp STEM P7 n=712	DR+/+ STEM P7 n=936	Flyp/lyp STEM P7 n=520	F+/+ STEM P7 n=465	Figure 1B P0 Venn n=1071	DRlyp/lyp STEM P0 n=442	DR+/+ STEM P0 n=437	Flyp/lyp STEM P0 n=643	F+/+ STEM P0 n=632	Fig1D n=25	Mean log2 intensity DRlyp/lyp 20	Mean log2 intensity DRlyp/lyp 30	Mean log2 intensity DRlyp/lyp 40	Mean log2 intensity DRlyp/lyp 50	Mean log2 intensity DR+/+ 20	Mean log2 intensity DR+/+ 30	Mean log2 intensity DR+/+ 40	Mean log2 intensity DR+/+ 50	Mean log2 intensity Flyp/lyp 20	Mean log2 intensity Flyp/lyp 30	Mean log2 intensity Flyp/lyp 40	Mean log2 intensity Flyp/lyp 50	Mean log2 intensity Flyp20	Mean log2 intensity Flyp30	Mean log2 intensity Flyp40	Mean log2 intensity Flyp50	Mean log2 intensity F+/+ 20	Mean log2 intensity F+/+ 30	Mean log2 intensity F+/+ 40	Mean log2 intensity F+/+ 50
1373860_at	Sox4	SRY (sex determining region Y)-box 4	0	0	0	0	0	0	Common_171	1	1	1	1	0	8.01	7.51	7.05	6.97	7.92	7.46	7.14	7.03	8.35	7.83	7.52	7.20	7.75	7.64	7.48	7.07	7.83	7.57	7.28	7.12
1374105_at	Higd1a	HIG1 hypoxia inducible domain family, member 1A	0	0	0	0	0	0	Common_171	1	1	1	1	0	7.30	7.03	6.96	6.59	7.40	7.34	6.74	6.93	7.25	6.92	6.95	6.44	7.48	6.96	6.97	6.44	7.29	7.08	6.87	6.54
1374211_at	RGD1566084	similar to Hypothetical protein LOC73072	0	0	0	0	0	0	Common_171	1	1	1	1	0	6.21	6.04	5.94	5.64	6.21	6.02	5.88	5.57	6.26	6.02	5.81	5.52	6.24	5.76	5.81	5.62	6.14	5.84	5.89	5.53
1374591_at	Ptprd	protein tyrosine phosphatase, receptor type, D	0	0	0	0	0	0	Common_171	1	1	1	1	0	7.48	7.15	6.92	6.75	7.50	7.35	6.78	6.75	6.35	6.00	5.84	5.40	6.54	6.02	5.64	5.47	6.60	6.09	5.88	5.74
1375026_at	Calml4	calmodulin-like 4	0	0	0	0	0	0	Common_171	1	1	1	1	0	7.25	7.23	7.13	6.73	7.39	7.34	6.91	6.64	7.13	7.07	6.77	6.58	6.87	6.69	6.76	6.45	7.11	6.89	6.71	6.32
1375123_at	Sox4	SRY (sex determining region Y)-box 4	0	0	0	0	0	0	Common_171	1	1	1	1	0	6.97	6.78	6.50	6.21	7.19	6.76	6.50	6.51	7.19	7.01	6.89	6.56	5.53	6.92	6.71	6.42	6.90	6.74	6.09	6.09
1375257_at	Sox4	SRY (sex determining region Y)-box 4	0	0	0	0	0	0	Common_171	1	1	1	1	0	4.91	4.89	4.97	5.02	5.00	5.11	5.10	4.86	4.90	4.90	5.13	5.21	4.80	5.00	5.03	5.16	4.88	5.04	5.11	5.17
1375412_at	Arsb	arylsulfatase B	0	0	0	0	0	0	Common_171	1	1	1	1	0	9.55	9.33	9.21	8.87	9.50	9.37	9.13	8.84	9.46	9.24	9.13	8.63	9.71	9.28	9.05	8.68	9.57	9.22	9.06	8.80
1375420_at	Tp53i11	tumor protein p53 inducible protein 11	0	0	0	0	0	0	Common_171	1	1	1	1	0	6.47	6.28	6.17	5.79	6.42	6.46	6.16	5.89	6.30	6.06	5.89	5.61	6.33	6.04	5.82	5.64	6.19	6.02	5.92	5.60
1375519_at	LOC287167	globin, alpha	0	0	0	0	0	0	Common_171	1	1	1	1	0	8.02	8.33	7.62	7.17	7.80	8.44	7.57	7.21	7.42	6.81	6.67	6.49	6.57	7.05	6.72	6.30	7.00	7.07	6.62	6.39
1375767_at	Plekhg3	pleckstrin homology domain containing, family G (with RhoGef domain) member 3	0	0	0	0	0	0	Common_171	1	1	1	1	0	8.21	7.98	7.74	7.62	8.24	7.84	7.82	7.55	8.25	7.88	7.76	7.51	8.41	7.86	7.59	7.59	8.18	7.74	7.79	7.56
1376619_at	Ptprd	protein tyrosine phosphatase, receptor type, D	0	0	0	0	0	0	Common_171	1	1	1	1	0	7.10	6.80	6.54	6.18	7.13	6.87	6.38	6.44	6.23	5.92	5.59	5.38	6.34	5.88	5.69	5.23	6.42	5.89	5.70	5.52
1376892_at	Gria3	glutamate receptor, ionotropic, AMPA 3	0	0	0	0	0	0	Common_171	1	1	1	1	0	9.90	9.46	9.17	8.87	10.03	9.47	9.02	8.74	10.10	9.44	9.21	8.59	9.96	9.19	8.91	8.68	9.89	9.22	9.06	8.68
1377367_at	Kazald1	Kazal-type serine peptidase inhibitor domain 1	0	0	0	0	0	0	Common_171	1	1	1	1	0	5.79	5.18	4.87	4.82	5.94	5.28	4.72	4.94	5.86	5.11	5.02	4.77	5.87	5.19	4.92	4.77	5.80	5.09	5.19	4.81
1377651_at	---	---	0	0	0	0	0	0	Common_171	1	1	1	1	0	4.59	4.22	4.05	3.69	4.28	4.45	4.03	3.90	7.24	7.26	7.19	6.60	6.27	7.07	7.12	6.66	7.24	7.20	6.55	6.46
1377666_at	Chdh	choline dehydrogenase	0	0	0	0	0	0	Common_171	1	1	1	1	0	5.45	5.04	4.91	4.70	5.46	5.08	4.92	4.58	5.53	5.13	4.84	4.60	5.31	5.11	4.80	4.31	5.49	5.15	4.99	4.66
1378298_at	Igsf21	immunoglobin superfamily, member 21	0	0	0	0	0	0	Common_171	1	1	1	1	0	6.79	6.48	6.41	6.11	6.68	6.66	6.25	5.99	6.63	6.34	6.24	5.99	6.66	6.64	6.22	5.89	6.64	6.54	6.26	5.98
1378904_at	Slc7a14	solute carrier family 7 (orphan transporter), member 14	0	0	0	0	0	0	Common_171	1	1	1	1	0	7.14	6.97	6.86	6.56	7.11	7.04	6.71	6.37	6.59	6.49	6.23	5.97	6.95	6.51	6.27	5.94	6.96	6.39	6.52	6.03
1379340_at	Lamc2	laminin, gamma 2	0	0	0	0	0	0	Common_171	1	1	1	1	0	5.87	5.00	4.80	4.81	5.84	4.80	5.15	4.92	6.33	5.48	5.25	4.43	6.67	5.50	5.20	5.02	6.55	5.16	4.86	4.54
1379397_at	Rora	RAR-related orphan receptor A	0	0	0	0	0	0	Common_171	1	1	1	1	0	8.30	8.12	8.09	7.50	8.14	8.23	7.85	7.54	8.45	8.09	8.15	7.80	7.92	8.26	8.12	7.57	8.27	8.15	7.66	7.65
1379566_at	Rbm11	RNA binding motif protein 11	0	0	0	0	0	0	Common_171	1	1	1	1	0	5.37	5.27	5.11	4.81	5.28	5.25	4.98	4.76	5.10	5.24	4.77	4.46	5.50	4.90	4.82	4.63	5.28	4.85	4.88	4.51
1379747_at	Prss35	protease, serine, 35	0	0	0	0	0	0	Common_171	1	1	1	1	0	3.15	3.22	2.85	2.67	3.15	3.18	2.84	2.54	3.79	3.68	3.50	2.85	3.65	3.20	3.10	2.93	3.60	3.13	3.02	2.79
1379824_at	Tox	thymocyte selection-associated high mobility group box	0	0	0	0	0	0	Common_171	1	1	1	1	0	9.02	8.80	8.48	8.40	9.04	8.81	8.42	8.25	8.99	8.62	8.34	7.93	9.00	8.55	8.37	8.18	9.05	8.55	8.29	8.18
1379889_at	Lamc2	laminin, gamma 2	0	0	0	0	0	0	Common_171	1	1	1	1	0	2.66	2.50	2.54	2.67	2.61	2.94	2.62	2.44	2.94	2.83	2.70	2.50	2.77	2.44	2.52	2.53	3.22	2.75	2.72	2.49
1380682_at	Mex3b	mex3 homolog B (C. elegans)	0	0	0	0	0	0	Common_171	1	1	1	1	0	5.63	5.46	5.09	5.00	5.69	5.50	5.16	5.19	5.82	5.46	5.30	4.89	5.80	5.27	5.47	5.08	5.74	5.11	5.07	5.12
1380683_at	Ptprd	protein tyrosine phosphatase, receptor type, D	0	0	0	0	0	0	Common_171	1	1	1	1	0	4.70	4.54	4.61	4.30	4.64	4.85	4.31	4.24	3.59	3.62	3.65	3.80	3.46	3.63	3.74	3.66	3.43	3.54	3.60	3.82
1380866_at	LOC365985	similar to adenylate kinase 5 isoform 1	0	0	0	0	0	0	Common_171	1	1	1	1	0	5.95	5.52	5.22	4.82	6.28	5.25	5.14	4.72	6.02	5.19	4.88	4.57	5.78	5.10	4.97	4.58	5.73	4.96	4.58	4.70
1380962_at	---	---	0	0	0	0	0	0	Common_171	1	1	1	1	0	9.34	9.08	8.81	8.50	9.26	9.15	8.76	8.45	9.40	9.07	9.00	8.62	9.44	9.06	8.98	8.59	9.47	9.14	9.00	8.81
1380981_at	---	---	0	0	0	0	0	0	Common_171	1	1	1	1	0	6.75	6.68	6.56	6.10	6.76	6.63	6.25	6.00	6.29	6.10	5.79	5.75	6.03	5.69	5.69	5.79	6.30	5.76	5.65	5.64
1381006_at	Hgfac	HGF activator	0	0	0	0	0	0	Common_171	1	1	1	1	0	8.77	8.63	8.49	8.10	8.74	8.69	8.36	8.08	8.89	8.54	8.38	8.03	8.67	8.64	8.43	8.01	8.81	8.63	8.35	8.18
1381955_at	Fam183b	family with sequence similarity 183, member B	0	0	0	0	0	0	Common_171	1	1	1	1	0	6.31	6.09	6.07	5.76	6.28	6.09	5.92	5.81	6.59	6.65	6.51	6.01	6.41	6.55	6.34	6.19	6.71	6.34	6.13	5.97
1382081_at	Scara3	scavenger receptor class A, member 3	0	0	0	0	0	0	Common_171	1	1	1	1	0	5.88	5.40	5.19	4.74	5.78	5.30	5.18	4.78	5.98	5.60	5.17	4.86	6.41	5.46	5.22	4.93	5.98	5.45	5.28	4.77
1382209_at	Ccdc28b	coiled coil domain containing 28B	0	0	0	0	0	0	Common_171	1	1	1	1	0	6.49	6.24	6.17	5.79	6.50	6.05	5.86	5.95	6.58	6.30	5.92	5.86	6.53	6.03	6.03	5.90	6.55	5.90	6.02	5.77
1382218_at	RGD1305807	hypothetical LOC298077	0	0	0	0	0	0	Common_171	1	1	1	1	0	7.12	6.74	6.48	6.06	7.31	6.76	6.34	6.02	6.87	6.45	6.10	5.61	7.09	6.31	5.96	5.60	6.85	6.21	6.14	5.40
1382593_at	Etv1	ets variant 1	0	0	0	0	0	0	Common_171	1	1	1	1	0	8.15	8.06	7.70	7.37	7.94	7.87	7.70	7.41	8.03	7.83	7.67	6.93	7.93	7.90	7.56	6.92	8.14	7.81	7.43	7.18
1383078_at	Efnb3	ephrin B3	0	0	0	0	0	0	Common_171	1	1	1	1	0	8.04	7.91	7.89	7.26	8.18	7.98	7.61	7.19	7.56	7.46	7.29	6.81	7.76	7.27	7.05	6.66	7.57	7.06	7.03	6.55
1383137_at	Sox4	SRY (sex determining region Y)-box 4	0	0	0	0	0	0	Common_171	1	1	1	1	0	10.69	10.17	9.87	9.76	10.58	10.23	9.91	9.85	10.96	10.60	10.26	10.08	10.79	10.37	10.26	9.95	10.62	10.30	10.11	10.02
1383283_at	Ocrl	oculocerebrorenal syndrome of Lowe	0	0	0	0	0	0	Common_171	1	1	1	1	0	9.01	8.78	8.64	8.27	9.02	8.90	8.42	8.30	8.96	8.65	8.31	7.89	8.92	8.49	8.32	8.01	9.04	8.51	8.27	8.07
1383418_at	Adam11	ADAM metallopeptidase domain 11	0	0	0	0	0	0	Common_171	1	1	1	1	0	4.70	4.49	4.26	4.20	4.79	4.46	4.17	4.28	5.10	4.84	4.62	4.30	5.52	4.71	4.43	4.11	5.16	4.58	4.67	4.31
1383895_at	Dynlrb2	dynein light chain roadblock-type 2	0	0	0	0	0	0	Common_171	1	1	1	1	0	5.42	5.26	5.14	4.85	5.27	5.39	5.26	4.81	6.26	6.24	5.59	4.96	6.00	5.97	5.57	4.94	6.20	5.68	5.42	4.81
1383989_at	Sox4	SRY (sex determining region Y)-box 4	0	0	0	0	0	0	Common_171	1	1	1	1	0	6.55	6.26	6.08	5.83	6.55	6.43	5.96	5.90	6.94	6.43	6.40	6.03	6.07	6.13	6.28	5.88	6.52	6.35	5.98	5.81
1384000_at	Sox4	SRY (sex determining region Y)-box 4	0	0	0	0	0	0	Common_171	1	1	1	1	0	7.32	6.82	6.52	6.26	7.16	6.77	6.31	6.34	7.63	7.23	6.73	6.64	7.30	6.91	6.82	6.39	7.16	6.87	6.79	6.69
1384240_at	Agtr1a	angiotensin II receptor, type 1a	0	0	0	0	0	0	Common_171	1	1	1	1	0	7.35	6.88	6.68	6.07	7.50	6.95	6.54	6.40	7.69	7.20	6.87	6.62	7.89	7.11	7.04	6.78	7.67	7.28	7.06	6.81
1384436_at	LOC10036158	anoctamin 2-like	0	0	0	0	0	0	Common_171	1	1	1	1	0	4.96	4.74	4.67	4.43	4.92	4.76	4.62	4.41	5.01	4.72	4.54	4.34	5.29	4.40	4.49	4.34	5.16	4.76	4.53	4.31
1384437_at	Smarca1	SWI/SNF related, matrix associated, actin dependent regulator of chromatin, subfamily a, me	0	0	0	0	0	0	Common_171	1	1	1	1	0	8.25	7.98	7.61	7.41	7.73	7.92	7.63	7.38	8.15	7.94	7.61	7.11	8.00	7.88	7.62	7.20	8.22	7.84	7.47	7.16
1384509_s_at	Pcdh17	protocadherin 17	0	0	0	0	0	0	Common_171	1	1	1	1	0	5.67	5.30	4.94	4.19	5.91	5.30	4.58	4.74	5.67	4.86	4.70	4.96	6.14	4.80	4.78	4.63	5.79	5.19	5.13	4.75
1384804_at	Dnmt3a	DNA (cytosine-5-)-methyltransferase 3 alpha	0	0	0	0	0	0	Common_171	1	1	1	1	0	8.89	8.44	8.28	8.00	8.80	8.54	8.20	8.08	8.97	8.37	8.32	7.94	8.65	8.22	8.33	7.96	8.88	8.42	8.02	7.96
1385073_at	---	---	0	0	0	0	0	0	Common_171	1	1	1	1	0	10.16	9.91	9.70	9.40	10.16	9.99	9.67	9.39	10.34	10.08	9.88	9.41	10.53	9.96	9.86	9.59	10.31	9.88	9.61	9.28
1385089_at	Grhl1	grainyhead-like 1 (Drosophila)	0	0	0	0	0	0	Common_171	1	1	1	1	0	6.40	6.01	5.73	5.03	6.14	5.66	5.29	5.55	6.58	6.36	5.93	5.48	6.54	6.05	5.47	5.21	6.20	6.09	5.84	5.33
1385756_at	Itih1	inter-alpha trypsin inhibitor, heavy chain 1	0	0	0	0	0	0	Common_171	1	1	1	1	0	7.05	6.58	6.59	6.33	7.13	6.71	6.47	6.28	7.15	6.67	6.32	6.26	6.89	6.50	6.49	6.12	6.99	6.48	6.34	6.10
1385881_at	Gatsl2	GATS protein-like 2	0	0	0	0	0	0	Common_171	1	1	1	1	0	9.55	9.19	8.91	8.71	9.60	9.21	8.95	8.49	9.84	9.41	9.17	8.64	10.04	9.28	8.99	8.57	9.81	9.20	8.89	8.98
1386538_at	RGD1310495	similar to KIAA1919 protein	0	0	0	0	0	0	Common_171	1	1	1	1	0	5.64	5.32	4.81	4.36	5.41	5.19	4.78	4.40	5.43	4.73	4.53	3.98	4.56	4.90	4.53	4.11	5.30	4.76	4.15	3.75
1386901_at	Cd36 /// LOC	CD36 molecule (thrombospondin receptor) /// scavenger receptor class B, member 2-like ///	0	0	0	0	0	0	Common_171	1	1	1	1	0	9.66	9.34	9.20	9.00	9.58	9.27	9.24	9.09	9.94	9.64	9.29	8.97	10.04	9.55	9.38	9.14	9.75	9.26	9.04	8.78
1386965_at	Lpl	lipoprotein lipase	0	0	0	0	0	0	Common_171	1	1	1	1	0	9.49	9.16	8.95	8.29	9.44	9.11	8.88	8.53	9.13	8.79	8.47	7.95	9.26	8.89	8.66	8.08	9.04	8.84	8.51	8.20
1387111_at	Ddah1	dimethylarginine dimethylaminohydrolase 1	0	0	0	0	0	0	Common_171	1	1	1	1	0	7.21	6.90	6.69	6.37	7.19	6.96	6.83	6.48	7.32	7.06	6.75	6.37	7.54	7.29	6.77	6.30	7.36	7.07	6.73	6.87
1387178_a_at	Cbs	cystathionine beta synthase	0	0	0	0	0	0	Common_171	1	1	1	1	0	5.13	5.28	4.37	4.59	5.56	5.02	4.58	4.81	5.81	4.92	4.81	4.73	5.47	5.81	4.79	4.73	5.41	5.62	5.13	4.99
1387195_at	St14	suppression of tumorigenicity 14 (colon carcinoma)	0	0	0	0	0	0	Common_171	1	1	1	1	0	7.15	7.00	6.89	6.56	7.23	6.99	6.82	6.63	6.89	6.60	6.34	5.95	7.14	6.46	6.46	6.11	6.86	6.51	6.27	5.91
1387249_at	Bik	BCL2-interacting killer (apoptosis-inducing)	0	0	0	0	0	0	Common_171	1	1	1	1	0	6.05	5.77	5.54	5.25	6.15	6.01	5.64	5.55	6.24	5.80	5.53	5.34	6.59	5.76	5.68	5.25	6.37	5.76	5.41	5.21
1387254_at	Ghrl	ghrelin/obestatin prepropeptide	0	0	0	0	0	0	Common_171	1	1	1	1	0	9.44	9.10	8.66	8.29	9.45	9.08	8.62	8.32	8.43	7.23	7.33	6.96	8.52	7.60	7.45	6.67	8.00	7.49	6.96	6.71
1387666_at	Gpr85	G protein-coupled receptor 85	0	0	0	0	0	0	Common_171	1	1	1	1	0	7.22	6.85	6.68	6.46	7.18	6.76	6.66	6.40	6.87	6.53	6.45	5.98	7.10	6.58	6.43	6.00	7.04	6.43	6.28	6.24
1387677_at	Pou3f4	POU class 3 homeobox 4	0	0	0	0	0	0	Common_171	1	1	1	1	0	5.87	5.43	5.23	4.94	5.88	5.98	5.10	5.02	5.74	4.93	4.98	4.49	4.77	5.05	5.25	4.45	5.70	4.99	4.22	4.36
1387702_at	Galr1	galanin receptor 1	0	0	0	0	0	0	Common_171	1	1	1	1	0	5.41	5.27	5.14	4.29	5.45	5.59	4.88	4.38	5.98	5.59	5.64	5.13	5.28	5.63	5.70	4.87	5.85	5.81	5.31	4.93
1387710_at	Oxtr	oxytocin receptor	0	0	0	0	0	0	Common_171	1	1	1	1	0	4.31	3.82	3.57	3.26	4.05	4.08	3.64	3.26	4.15	3.89	4.03	3.44	3.05	3.95	3.89	3.15	4.22	4.10	3.38	3.32
1387725_at	Gulo	gulonolactone (L-) oxidase	0	0	0	0	0	0	Common_171	1	1	1	1	0	6.05	5.79	5.55	5.06	6.15	5.88	5.53	5.15	6.14	5.60	5.39	5.25	5.47	5.53	5.41	4.93	5.97	5.68	5.14	5.07
1387729_at	Ggt5	gamma-glutamyltransferase 5	0	0	0	0	0	0	Common_171	1	1	1	1	0	6.78	6.28	6.23	6.03	6.62	6.12	6.40	6.03	6.54	6.37	6.33	6.01	6.03	6.60	6.23	5.91	6.84	6.55	6.18	5.99
1387811_at	Agt	angiotensinogen (serpin peptidase inhibitor, clade A, member 8)	0	0	0	0	0	0	Common_171	1	1	1	1	0	7.01	6.78	6.53	6.19	6.97	6.77	6.26	6.01	6.63	6.34	6.19	5.88	6.36	6.32	6.14	5.63	6.73	6.30	6.07	5.63
1387947_at	Mafb	v-maf musculoaponeurotic fibrosarcoma oncogene homolog B (avian)	0	0	0	0	0	0	Common_171	1	1	1	1	0	9.44	9.07	8.64	8.45	9.45	9.16	8.66	8.42	9.55	8.95	8.58	7.88	9.54	8.77	8.57	8.13	9.38	8.73	8.23	7.88
1388223_at	Gnat3	guanine nucleotide binding protein, alpha transducing 3	0	0	0	0	0	0	Common_171	1	1	1	1	0	7.22	6.66	5.99	5.43	7.16	6.70	6.06	5.70	6.94	6.15	5.51	5.18	6.93	5.92	5.61	5.20	6.79	5.62	5.49	5.28
1388547_at	Cldn4	claudin 4	0	0	0	0	0	0	Common_171	1	1	1	1	0	7.31	7.03	6.86	6.54	7.17	7.03	6.94	6.45	6.71	6.52	6.36	6.04	6.60	6.71	6.45	6.07	6.79	6.49	6.21	6.14
1388608_x_at	Hba1 /// Hba	hemoglobin, alpha 1 /// hemoglobin, alpha 2	0	0	0	0	0	0	Common_171	1	1	1	1	0	11.65	11.78	11.06	10.55	11.54	11.99	10.87	10.57	12.01	11.30	10.89	9.78	10.70	11.51	10.80	9.90	11.55	11.52	10.96	10.10
1388902_at	Loxl1	lysyl oxidase-like 1	0	0	0	0	0	0	Common_171	1	1	1	1	0	7.72	7.42	7.24	6.98	7.95	7.63	7.11	7.06	7.46	7.03	6.51	6.46	7.69	6.99	6.98	6.71	7.38	6.96	6.80	6.47
1389474_at	Mylip	myosin regulatory light chain interacting protein	0	0	0	0	0	0	Common_171	1	1	1	1	0	8.03	7.72	7.42	7.39	8.11	7.67	7.56	7.38	7.63	7.00	6.84	6.71	7.84	7.12	7.22	6.84	7.66	6.94	6.90	6.82
1389503_at	Upk3bl	uroplakin 3B-like	0	0	0	0	0	0	Common_171	1	1	1	1	0	6.41	6.19	5.91	5.41	6.41	6.10	5.79	5.63	7.00	6.62	6.42	5.92	6.64	6.58	6.23	5.76	7.01	6.46	6.11	5.71
1389597_at	Pgbd5	piggyBac transposable element derived 5	0	0	0	0	0	0	Common_171	1	1	1	1	0	8.72	8.35	8.23	8.13	8.56	8.43	8.29	7.81	8.75	8.39	8.12	7.82	8.72	8.38	8.16	7.82	8.58	8.24	8.09	7.90
1389781_at	Elmod1	ELMO/CED-12 domain containing 1	0	0	0	0	0	0	Common_171	1	1	1	1	0	6.82	6.51	6.13	5.76	6.81	6.49	6.06	5.75	6.90	6.54	5.84	5.28	7.02	6.45	5.99	5.20	6.89	6.30	5.75	5.36
1389971_at	Sgce	sarcoglycan, epsilon	0	0	0	0	0	0	Common_171	1	1	1	1	0	6.97	6.57	6.23	5.79	6.89	6.62	6.24	6.02	6.73	6.35	6.11	5.46	6.75	6.23	5.92	5.57	6.86	6.17	6.00	5.76
1390051_at	RGD1562533	similar to mKIAA0774 protein	0	0	0	0	0	0	Common_171	1	1	1	1	0	5.08	4.84	4.49	4.26	5.36	4.81	4.50	4.29	4.89	4.50	4.37	4.19	5.03	4.49	4.09	3.95	4.86	4.21	4.34	4.30
1390518_at	Emid1	EMI domain containing 1	0	0	0	0	0	0	Common_171	1	1	1	1	0	5.47	5.29	5.17	4.97	5.55	5.29	5.30	4.94	5.55	5.16	5.06	5.01	5.71	5.25	5.32	5.02	5.63	5.38	5.34	5.11
1390569_at	Cndp1	carnosine dipeptidase 1 (metallopeptidase M20 family)	0	0	0	0	0	0	Common_171	1	1	1	1	0	4.28	3.63	3.43	3.31	4.43	3.53	3.42	3.42	5.08	3.97	3.59	3.21	5.68	3.53	3.46	3.26	4.82	3.54	3.43	3.16
1391184_at	Rfx2	regulatory factor X, 2 (influences HLA class II expression)	0	0	0	0	0	0	Common_171	1	1	1	1	0	6.65	6.46	6.20	6.10	6.58	6.40	6.13	5.77	6.51	6.32	6.20	6.00	6.43	6.18	6.05	5.93	6.48	6.31	6.32	5.82
1391187_at	Ppl	periplakin	0	0	0	0	0	0	Common_171	1	1	1	1	0	7.50	7.44	7.37	6.86	7.55	7.41	7.23	6.89	7.18	7.02	6.70	6.48	7.30	6.88	6.75	6.51	7.12	6.81	6.65	6.41
1391241_at	Etv1	ets variant 1	0	0	0	0	0	0	Common_171	1	1	1	1	0	8.05	7.97	7.63	7.17	7.97	7.83	7.47	7.22	8.42	8.05	7.80	6.96	8.16	8.10	7.75	7.22	8.46	8.08	7.59	7.22
1391269_at	Pim2	pim-2 oncogene	0	0	0	0	0	0	Common_171	1	1	1	1	0	8.74	8.46	8.37	8.14	8.65	8.58	8.26	8.13	9.18	8.91	8.65	8.60	9.20	8.68	8.57	8.48	9.11	8.70	8.59	8.42
1391457_a_at	Irx2	iroquois homeobox 2	0	0	0	0	0	0	Common_171	1	1	1	1	0	7.22	6.78	6.46	6.08	7.25	6.88	6.45	6.09	7.14	6.58	6.25	5.68	7.24	6.48	6.34	5.63	7.05	6.40	6.08	5.85
1391857_at	Mslnl	mesothelin-like	0	0	0	0	0	0	Common_171	1	1	1	1	0	8.16	8.04	7.75	7.34	8.14	7.97	7.67	7.29	7.50	7.36	6.98	6.67	7.49	7.48	7.14	6.82	7.33	7.17	6.91	6.54
1392299_at	Nos1ap	nitric oxide synthase 1 (neuronal) adaptor protein	0	0	0	0	0	0	Common_171	1	1	1	1	0	7.62	7.47	7.22	6.99	7.67	7.45	7.23	6.97	7.55	7.03	7.10	6.89	6.87	7.19	6.94	6.77	7.38	7.34	7.16	6.81
1392477_at	Etv1	ets variant 1	0	0	0	0	0	0	Common_171	1	1	1	1	0	10.89	10.77	10.38	10.13	10.79	10.79	10.33	10.04	10.78	10.57	10.31	9.56	10.71	10.51	10.13	9.67	10.85	10.58	10.09	9.74
1392555_at	Etv1	ets variant 1	0	0	0	0	0	0	Common_171	1	1	1	1	0	5.18	5.07	4.78	4.66	5.16	5.14	4.76	4.59	5.14	4.96	4.72	4.23	5.43	4.88	4.58	4.20	5.28	4.93	4.66	4.39
1392678_a_at	Dnmt3a	DNA (cytosine-5-)-methyltransferase 3 alpha	0	0	0	0	0	0	Common_171	1	1	1	1	0	5.82	5.52	5.43	5.33	5.99	5.64	5.45	5.27	6.28	5.73	5.80	5.56	5.79	5.65	5.73	5.37	6.21	5.87	5.48	5.55
1393015_at	RGD1310587	similar to hypothetical protein FLJ14146	0	0	0	0	0	0	Common_171	1	1	1	1	0	7.77	7.37	7.04	6.86	7.87	7.35	7.11	7.25	7.20	6.89	6.73	6.53	7.63	6.99	6.68	6.62	7.55	7.32	7.08	6.92
1393083_at	Tox	thymocyte selection-associated high mobility group box	0	0	0	0	0	0	Common_171	1	1	1	1	0	5.24	5.12	4.90	4.85	5.17	5.00	4.93	4.82	5.16	5.03	4.93	4.70	5.64	4.90	4.92	4.79	5.39	4.86	5.05	4.89
1393386_at	Fam183b	family with sequence similarity 183, member B	0	0	0	0	0	0	Common_171	1	1	1	1	0	5.98	5.58	5.68	5.43	5.93	5.51	5.38	5.28	6.19	6.30	6.07	5.55	6.11	6.09	5.89	5.63	6.19	5.96	5.67	5.59
1393454_at	Pcdh17	protocadherin 17	0	0	0	0	0	0	Common_171	1	1	1	1	0	4.60	4.16	3.89	3.27	4.90	4.32	3.58	3.60	4.54	3.85	3.59	3.80	4.97	3.76	3.72	3.62	4.74	4.14	4.09	3.79
1393814_at	Zan	zonadhesin	0	0	0	0	0	0	Common_171	1	1	1	1	0	4.69	4.31	4.15	3.94	4.91	4.23	4.22	4.12	5.14	4.81	4.47	4.27	5.07	4.71	4.36	4.30	5.05	4.53	4.64	4.15
1394571_at	Fam227a	family with sequence similarity 227, member A	0	0	0	0	0	0	Common_171	1	1	1	1	0	6.19	5.74	5.76	5.50	6.41	5.64	5.53	5.06	6.90	6.12	5.66	5.65	7.00	5.52	5.47	5.62	6.60	5.58	5.68	5.33
1394717_at	---	---	0	0	0	0	0	0	Common_171	1	1	1	1	0	5.83	5.57	5.49	5.04	5.66	5.60	5.29	4.75	6.13	5.67	5.50	5.65	5.37	5.67	5.65	5.14	6.03	5.80	5.28	5.12
1395148_at	---	---	0	0	0	0	0	0	Common_171	1	1	1	1	0	4.75	4.42	4.46	3.97	4.90	4.58	4.34	3.93	4.26	3.77	3.97	3.92	4.43	3.82	3.78	3.67	4.29	4.02	4.03	3.74
1395809_at	Sesn1	sestrin 1	0	0	0	0	0	0	Common_171	1	1	1	1	0	6.42	6.44	6.15	5.62	6.46	6.30	6.03	5.97	6.81	6.28	6.20	5.96	5.47	6.43	6.17	5.57	6.68	6.32	5.77	5.72
1396208_at	Ggt5	gamma-glutamyltransferase 5	0	0	0	0	0	0	Common_171	1	1	1	1	0	6.92	6.27	6.32	5.71	6.79	6.33	6.09	5.83	6.80	6.50	6.23	5.79	6.63	6.67	6.39	5.74	7.07	6.58	6.30	5.91
1396209_at	Mcart1l	mitochondrial carrier triple repeat 1-like	0	0	0	0	0	0	Common_171	1	1	1	1	0	9.16	8.92	8.64	8.43	9.20	9.05	8.51	8.39	9.20	8.92	8.66	8.44	9.23	8.79	8.69	8.43	9.12	8.94	8.74	8.44
1396896_at	Fstl4	follistatin-like 4	0	0	0	0	0	0	Common_171	1	1	1	1	0	7.61	7.27	7.24	6.83	7.61	7.38	7.13	6.65	7.96	7.58	7.19	6.58	8.04	7.45	7.09	6.54	7.71	7.25	6.94	6.38
1397015_at	Etv1	ets variant 1	0	0	0	0	0	0	Common_171	1	1	1	1	0	7.94	7.88	7.64	7.46	7.89	7.88	7.60	7.28	7.97	7.91	7.69	7.04	7.78	7.82	7.61	7.13	8.24	7.84	7.54	7.23
1397233_at	Dclk3	doublecortin-like kinase 3	0	0	0	0	0	0	Common_171	1	1	1	1	0	6.40	6.10	5.80	5.32	6.30	5.95	5.91	5.10	6.09	5.91	5.53	5.19	6.22	5.99	5.55	5.13	6.26	5.76	5.55	5.35
1398255_at	Slc15a2	solute carrier family 15 (H+/peptide transporter), member 2	0	0	0	0	0	0	Common_171	1	1	1	1	0	6.10	5.68	5.18	4.93	5.86	5.56	5.44	4.98	6.79	6.17	5.57	5.08	6.67	5.98	5.51	5.05	6.97	5.69	5.24	5.30
1398295_at	Slc29a1	solute carrier family 29 (nucleoside transporters), member 1	0	0	0	0	0	0	Common_171	1	1	1	1	0	7.32	7.26	7.08	6.76	7.51	7.34	7.07	6.76	6.69	6.35	6.14	5.83	6.96	6.38	6.17	5.90	6.77	6.31	6.13	5.96
1398318_at	Muc1	mucin 1, cell surface associated	0	0	0	0	0	0	Common_171	1	1	1	1	0	5.63	5.39	4.44	4.61	6.05	5.21	5.03	4.92	5.94	5.12	4.76	4.91	6.04	5.70	4.82	4.94	5.60	5.82	5.36	5.17
1398646_at	Rgs22	regulator of G-protein signaling 22	0	0	0	0	0	0	Common_171	1	1	1	1	0	8.74	8.78	8.50	8.15	8.83	8.73	8.39	8.02	6.74	6.54	6.25	6.13	6.41	6.52	6.25	6.03	6.50	6.35	6.12	5.89
1398659_at	Vwc2	von Willebrand factor C domain containing 2	0	0	0	0	0	0	Common_171	1	1	1	1	0	7.59	7.29	7.20	6.85	7.55	7.42	7.17	6.83	7.55	7.42	7.09	6.54	7.39	7.36	7.12	6.60	7.45	7.31	7.00	6.92
1369691_at	Scn3a	sodium channel, voltage-gated, type III, alpha	0	0	0	0	0	0	DR+/+nF+/+_27	0	1	0	1	0	9.01	8.60	8.72	8.60	9.01	8.67	8.43	8.50	9.72	9.54	9.48	9.56	9.70	9.54	9.46	9.44	9.74	9.49	9.31	9.15
1369711_at	Agtr2	angiotensin II receptor, type 2	0	0	0	0	0	0	DR+/+nF+/+_27	0	1	0	1	0	3.96	3.99	4.01	3.92	4.19	3.99	4.05	3.92	4.01	3.91	3.93	4.10	4.08	3.98	3.98	3.83	4.10	4.00	4.09	4.06
1372365_at	Rin2	Ras and Rab interactor 2	0	0	0	0	0	0	DR+/+nF+/+_27	0	1	0	1	0	8.80	8.82	8.71	8.64	8.94	8.71	8.65	8.59	8.74	8.64	8.46	8.54	8.81	8.53	8.53	8.46	8.84	8.63	8.59	8.49
1375602_at	Rin2	Ras and Rab interactor 2	0	0	0	0	0	0	DR+/+nF+/+_27	0	1	0	1	0	5.36	5.42	5.14	4.95	5.76	5.25	5.11	4.85	5.59	5.23	4.99	5.06	5.62	4.96	5.04	5.00	5.55	5.24	5.23	4.87
1376175_at	Gbas	glioblastoma amplified sequence	0	0	0	0	0	0	DR+/+nF+/+_27	0	1	0	1	0	6.81	6.57	6.57	6.45	6.67	6.76	6.36	6.26	6.65	6.62	6.81	6.50	5.45	6.61	6.69	6.41	6.70	6.69	6.19	6.02
1376359_at	Ms4a8	membrane-spanning 4-domains, subfamily A	0	0	0	0	0	0	DR+/+nF+/+_27	0	1	0	1	0	5.65	5.63	5.15	5.25	5.79	5.70	5.59	5.14	5.88	5.58	5.50	5.44	5.85	5.31	5.50	5.38	5.97	5.69	5.77	5.21
1376762_at	Plekha1	pleckstrin homology domain containing, family A (phosphoinositide binding specific) membe	0	0	0	0	0	0	DR+/+nF+/+_27	0	1	0	1	0	9.14	9.00	8.89	8.68	9.27	9.15	8.77	8.56	9.16	8.92	8.85	8.49	9.23	8.99	8.79	8.53	9.12	8.99	8.73	8.64
1377090_at	Dock6	dedicator of cytokinesis 6	0	0	0	0	0	0	DR+/+nF+/+_27	0	1	0	1	0	6.81	6.66	6.48	6.37	6.93	6.63	6.50	6.25	6.48	6.30	6.01	6.03	6.95	6.14	6.03	6.07	6.63	6.24	6.16	6.03
1378155_at	Prrc2c	proline-rich coiled-coil 2C	0	0	0	0	0	0	DR+/+nF+/+_27	0	1	0	1	0	8.29	8.25	8.02	8.08	8.59	8.24	7.89	8.21	8.52	8.03	8.05	7.81	7.82	8.12	8.08	7.90	8.39	8.16	8.07	7.83

Affymetrix Probeset ID	Gene Symbol	Gene Name	unique BB on P7 n=822	Figure 1B P7 Venn n=1526	DRlyp/lyp STEM P7 n=712	DR+/+ STEM P7 n=936	Flyp/lyp STEM P7 n=520	F+/+ STEM P7 n=465	Figure 1B P0 Venn n=1071	DRlyp/lyp STEM P0 n=442	DR+/+ STEM P0 n=437	Flyp/lyp STEM P0 n=643	F+/+ STEM P0 n=632	Fig1D n=25	Mean log2 intensity DRlyp/lyp 20	Mean log2 intensity DRlyp/lyp 30	Mean log2 intensity DRlyp/lyp 40	Mean log2 intensity DRlyp/lyp 50	Mean log2 intensity DR+/+ 20	Mean log2 intensity DR+/+ 30	Mean log2 intensity DR+/+ 40	Mean log2 intensity DR+/+ 50	Mean log2 intensity Flyp/lyp 20	Mean log2 intensity Flyp/lyp 30	Mean log2 intensity Flyp/lyp 40	Mean log2 intensity Flyp/lyp 50	Mean log2 intensity Flyp20	Mean log2 intensity Flyp30	Mean log2 intensity Flyp40	Mean log2 intensity Flyp50	Mean log2 intensity F+/+ 20	Mean log2 intensity F+/+ 30	Mean log2 intensity F+/+ 40	Mean log2 intensity F+/+ 50
1378527_at	LOC303590	similar to cactin CG1676-PA	0	0	0	0	0	0	DR+/+nF+/+_27	0	1	0	1	0	5.63	5.48	5.52	5.18	6.05	5.58	5.43	5.12	5.76	5.48	5.42	5.36	6.23	5.35	5.30	5.29	5.93	5.29	5.37	5.15
1379232_at	LOC1009118	TBC1 domain family member 12-like /// TBC1 domain family member 12-like /// hypothetica	0	0	0	0	0	0	DR+/+nF+/+_27	0	1	0	1	0	6.35	6.50	6.34	6.29	6.66	6.19	6.14	5.99	6.30	6.25	6.30	6.07	5.51	5.92	6.34	6.06	6.25	6.30	5.93	5.73
1380435_at	Shoc2	soc-2 (suppressor of clear) homolog (C. elegans)	0	0	0	0	0	0	DR+/+nF+/+_27	0	1	0	1	0	6.55	6.32	6.34	6.18	6.44	6.60	6.38	6.03	6.39	6.25	6.29	6.03	5.61	6.22	6.20	5.87	6.38	6.29	5.90	5.79
1381537_at	Klc3	kinesin light chain 3	0	0	0	0	0	0	DR+/+nF+/+_27	0	1	0	1	0	6.17	5.91	5.93	5.70	6.00	6.05	5.98	5.47	5.91	5.78	5.65	5.54	6.00	5.68	5.75	5.63	6.10	5.73	5.58	5.42
1387223_at	Aadat	aminoadipate aminotransferase	0	0	0	0	0	0	DR+/+nF+/+_27	0	1	0	1	0	4.12	4.21	4.09	4.04	4.38	4.16	3.95	3.82	4.33	4.10	3.86	3.85	4.54	4.09	4.02	3.83	4.27	4.14	3.92	3.76
1387312_a_at	Gck	glucokinase	0	0	0	0	0	0	DR+/+nF+/+_27	0	1	0	1	0	7.89	7.80	7.74	7.61	7.95	7.85	7.71	7.45	7.99	7.79	7.72	7.59	7.79	7.70	7.69	7.51	7.98	7.76	7.69	7.45
1388068_at	Klc3	kinesin light chain 3	0	0	0	0	0	0	DR+/+nF+/+_27	0	1	0	1	0	5.92	5.76	5.82	5.40	5.83	6.05	5.64	5.40	5.78	5.63	5.49	5.27	5.76	5.67	5.45	5.16	5.60	5.60	5.26	5.07
1390138_at	Ccdc8	coiled-coil domain containing 8	0	0	0	0	0	0	DR+/+nF+/+_27	0	1	0	1	0	7.44	7.29	7.07	7.05	7.40	7.35	7.21	6.83	7.63	7.52	7.34	7.31	7.60	7.36	7.30	7.30	7.71	7.48	7.39	7.19
1392230_at	---	---	0	0	0	0	0	0	DR+/+nF+/+_27	0	1	0	1	0	4.51	4.50	4.43	4.26	4.34	4.52	4.38	4.04	4.43	4.48	4.30	4.21	4.63	4.30	4.17	4.03	4.55	4.22	4.21	3.91
1392510_at	Fam180a	family with sequence similarity 180, member A	0	0	0	0	0	0	DR+/+nF+/+_27	0	1	0	1	0	5.21	4.93	4.85	4.81	5.52	4.97	4.95	4.83	4.98	4.82	4.54	4.55	5.11	4.64	4.66	4.81	5.52	4.80	4.85	4.81
1392738_at	Prrc2c	proline-rich coiled-coil 2C	0	0	0	0	0	0	DR+/+nF+/+_27	0	1	0	1	0	6.22	5.88	5.67	5.68	6.12	6.14	5.57	5.61	6.41	5.87	6.06	5.83	5.32	6.01	5.98	5.74	6.28	6.09	5.67	5.72
1394964_at	LOC1009104	protein PRRC2C-like	0	0	0	0	0	0	DR+/+nF+/+_27	0	1	0	1	0	7.13	6.74	6.63	6.45	6.75	6.88	6.41	6.18	7.34	6.69	7.07	6.33	5.81	6.64	7.07	6.04	7.24	7.18	6.40	6.46
1395336_at	Spats2l	spermatogenesis associated, serine-rich 2-like	0	0	0	0	0	0	DR+/+nF+/+_27	0	1	0	1	0	7.24	7.37	7.19	7.08	7.27	7.38	7.05	6.84	6.96	7.07	7.12	6.77	6.39	7.03	7.03	6.61	7.00	7.14	6.76	6.60
1396479_at	Zbtb7c	zinc finger and BTB domain containing 7C	0	0	0	0	0	0	DR+/+nF+/+_27	0	1	0	1	0	4.88	4.59	4.61	4.58	4.80	4.78	4.67	4.28	4.71	4.47	4.40	4.40	4.91	4.37	4.36	4.23	4.69	4.43	4.35	4.18
1397487_at	Xpnpep3	X-prolyl aminopeptidase (aminopeptidase P) 3, putative	0	0	0	0	0	0	DR+/+nF+/+_27	0	1	0	1	0	5.51	5.53	5.63	5.59	5.74	5.60	5.61	5.21	5.32	5.43	5.47	5.32	5.31	5.42	5.63	5.18	5.72	5.39	5.21	5.29
1397828_at	Sphkap	SPHK1 interactor, AKAP domain containing	0	0	0	0	0	0	DR+/+nF+/+_27	0	1	0	1	0	8.53	8.28	8.29	8.13	8.57	8.33	8.02	8.03	8.33	8.06	7.92	7.89	8.49	7.92	7.88	7.99	8.24	8.38	8.20	7.71
1398288_at	Agtr2	angiotensin II receptor, type 2	0	0	0	0	0	0	DR+/+nF+/+_27	0	1	0	1	0	3.30	2.90	2.68	2.52	3.36	2.88	2.55	2.43	2.88	2.63	2.53	2.41	3.60	2.60	2.61	2.32	3.44	2.70	2.75	2.45
1398633_at	---	---	0	0	0	0	0	0	DR+/+nF+/+_27	0	1	0	1	0	5.56	5.54	5.24	5.27	5.73	5.42	5.21	5.18	5.60	5.35	5.54	5.39	5.19	5.43	5.35	5.25	5.60	5.44	5.33	5.34
1367714_at	Eif2b2	eukaryotic translation initiation factor 2B, subunit 2 beta	0	0	0	0	0	0	DR+/+nFIS_32	0	1	1	1	0	8.05	7.82	7.80	7.57	8.17	7.83	7.68	7.54	8.15	7.79	7.69	7.56	8.33	7.80	7.80	7.47	8.14	7.80	7.66	7.55
1368686_at	Ambp	alpha-1-microglobulin/bikunin precursor	0	0	0	0	0	0	DR+/+nFIS_32	0	1	1	1	0	8.58	8.64	8.48	8.25	8.88	8.58	8.33	8.01	8.65	8.43	8.13	7.99	9.03	8.24	8.03	8.06	8.58	8.26	8.28	7.96
1369004_at	Rab26	RAB26, member RAS oncogene family	0	0	0	0	0	0	DR+/+nFIS_32	0	1	1	1	0	6.32	6.25	6.08	5.96	6.48	6.08	5.98	6.01	6.44	6.00	5.81	5.64	6.62	6.20	5.68	5.66	6.23	6.00	5.82	5.68
1369017_at	Kcnh6	potassium voltage-gated channel, subfamily H (eag-related), member 6	0	0	0	0	0	0	DR+/+nFIS_32	0	1	1	1	0	8.25	8.11	7.97	7.87	8.31	8.13	7.91	7.74	8.19	8.02	7.82	7.59	8.21	7.80	7.78	7.67	8.14	7.92	7.89	7.56
1369766_at	Ptger2	prostaglandin E receptor 2 (subtype EP2)	0	0	0	0	0	0	DR+/+nFIS_32	0	1	1	1	0	4.86	5.03	4.69	4.57	5.14	5.15	4.91	4.77	4.88	4.79	4.75	4.41	4.76	4.75	4.64	4.28	5.09	4.65	4.52	4.44
1370248_at	Fxyd6	FXYD domain-containing ion transport regulator 6	0	0	0	0	0	0	DR+/+nFIS_32	0	1	1	1	0	9.80	9.59	9.63	9.30	9.79	9.76	9.51	9.26	9.91	9.74	9.50	9.21	9.82	9.67	9.58	9.32	9.92	9.66	9.37	9.11
1370282_at	Csrp2	cysteine and glycine-rich protein 2	0	0	0	0	0	0	DR+/+nFIS_32	0	1	1	1	0	8.69	8.88	8.59	8.39	8.82	8.91	8.54	8.39	8.69	8.72	8.49	8.07	8.31	8.59	8.59	8.27	8.69	8.68	8.43	8.06
1370512_at	Tnrc6b	trinucleotide repeat containing 6B	0	0	0	0	0	0	DR+/+nFIS_32	0	1	1	1	0	7.27	7.12	6.90	6.97	7.29	6.99	6.91	6.85	7.28	7.02	6.91	6.79	7.32	6.89	6.86	6.86	7.19	6.95	6.99	6.79
1370881_at	Tst	thiosulfate sulfurtransferase	0	0	0	0	0	0	DR+/+nFIS_32	0	1	1	1	0	5.34	5.20	4.92	4.96	5.53	5.00	5.00	5.05	5.72	5.02	4.90	4.82	5.79	5.65	4.99	4.90	5.45	5.32	4.80	4.89
1376982_at	Tnrc6b	trinucleotide repeat containing 6B	0	0	0	0	0	0	DR+/+nFIS_32	0	1	1	1	0	8.93	8.68	8.52	8.47	8.95	8.68	8.44	8.33	8.95	8.55	8.44	8.27	8.93	8.49	8.42	8.28	8.87	8.56	8.45	8.32
1377014_at	Nim1	serine/threonine-protein kinase NIM1	0	0	0	0	0	0	DR+/+nFIS_32	0	1	1	1	0	3.34	3.29	3.19	3.05	3.65	3.18	3.07	2.94	3.50	3.36	3.38	2.91	3.76	3.38	3.38	3.07	3.79	3.40	3.29	3.12
1377177_at	Irx1	iroquois homeobox 1	0	0	0	0	0	0	DR+/+nFIS_32	0	1	1	1	0	2.90	2.94	2.85	3.02	2.91	3.08	3.03	3.03	2.97	2.73	3.01	3.00	3.10	3.14	3.06	3.18	2.81	3.09	2.86	3.18
1377761_at	Gfpt2	glutamine-fructose-6-phosphate transaminase 2	0	0	0	0	0	0	DR+/+nFIS_32	0	1	1	1	0	9.66	9.52	9.36	9.18	9.72	9.60	9.36	9.17	9.70	9.23	9.16	8.89	9.69	9.30	9.23	8.91	9.57	9.22	9.10	8.93
1378101_at	Ppp1r17	protein phosphatase 1, regulatory subunit 17	0	0	0	0	0	0	DR+/+nFIS_32	0	1	1	1	0	3.40	3.11	3.01	3.16	3.49	3.27	3.24	2.93	4.91	3.96	3.80	3.29	4.62	3.89	3.53	3.32	6.50	5.32	4.59	4.70
1378303_at	Tnrc6b	trinucleotide repeat containing 6B	0	0	0	0	0	0	DR+/+nFIS_32	0	1	1	1	0	8.40	7.73	7.62	7.60	8.11	7.93	7.45	7.59	8.42	8.07	7.73	7.79	7.32	7.88	7.68	7.89	8.26	7.77	7.45	7.26
1383217_at	---	---	0	0	0	0	0	0	DR+/+nFIS_32	0	1	1	1	0	4.95	4.88	4.79	4.59	5.24	4.92	4.67	4.56	5.24	5.19	4.76	4.63	5.53	5.11	4.79	4.58	5.55	4.94	4.98	4.59
1383590_at	Csgalnact1	chondroitin sulfate N-acetylgalactosaminyltransferase 1	0	0	0	0	0	0	DR+/+nFIS_32	0	1	1	1	0	6.23	6.18	5.97	5.76	6.34	5.98	5.71	5.82	6.14	5.89	5.79	5.53	6.14	5.80	5.79	5.62	6.06	5.86	5.75	5.44
1383840_at	Csgalnact1	chondroitin sulfate N-acetylgalactosaminyltransferase 1	0	0	0	0	0	0	DR+/+nFIS_32	0	1	1	1	0	5.21	5.12	4.94	4.83	5.36	5.03	4.78	4.81	5.08	4.97	4.88	4.65	5.31	4.97	4.92	4.55	5.29	5.05	4.94	4.63
1384308_at	Meis1	Meis homeobox 1	0	0	0	0	0	0	DR+/+nFIS_32	0	1	1	1	0	9.51	9.46	9.38	9.22	9.69	9.42	9.27	9.16	9.30	9.03	8.97	8.77	9.35	8.95	8.92	8.78	9.25	9.02	8.88	8.70
1384312_at	Irx1	iroquois homeobox 1	0	0	0	0	0	0	DR+/+nFIS_32	0	1	1	1	0	8.81	8.39	8.12	7.79	8.99	8.61	8.08	7.60	8.51	8.06	7.71	6.90	8.73	7.86	7.59	7.04	8.50	7.85	7.33	7.03
1385434_at	---	---	0	0	0	0	0	0	DR+/+nFIS_32	0	1	1	1	0	6.42	6.24	6.11	5.80	6.48	6.50	6.06	5.87	6.23	6.01	5.76	5.51	6.43	5.92	5.87	5.43	6.24	5.79	5.70	5.56
1386059_at	Hist2h2be	histone cluster 2, H2be	0	0	0	0	0	0	DR+/+nFIS_32	0	1	1	1	0	7.30	7.28	7.11	6.82	7.43	7.42	6.96	6.91	7.61	7.61	7.40	7.05	7.83	7.40	7.36	7.03	7.65	7.51	7.21	7.04
1389636_at	Camta1	calmodulin binding transcription activator 1	0	0	0	0	0	0	DR+/+nFIS_32	0	1	1	1	0	6.84	6.90	7.01	7.02	7.00	6.89	6.95	7.09	7.21	7.07	7.15	7.01	7.20	7.07	7.07	7.15	7.06	7.11	7.02	6.97
1390000_at	Kdm6b	lysine (K)-specific demethylase 6B	0	0	0	0	0	0	DR+/+nFIS_32	0	1	1	1	0	8.07	7.85	7.66	7.71	8.08	8.04	7.78	7.39	8.21	7.99	8.08	7.48	7.96	7.95	7.96	7.55	8.17	8.14	7.90	7.65
1391030_at	Camta1	calmodulin binding transcription activator 1	0	0	0	0	0	0	DR+/+nFIS_32	0	1	1	1	0	6.25	6.10	6.07	5.87	6.46	6.08	6.10	5.66	6.38	6.25	5.87	5.58	6.59	5.99	5.69	5.60	6.26	5.77	5.65	5.77
1391075_at	Rgs17	regulator of G-protein signaling 17	0	0	0	0	0	0	DR+/+nFIS_32	0	1	1	1	0	8.67	8.55	8.44	8.16	8.62	8.73	8.43	7.98	9.14	8.65	8.66	8.52	8.25	8.77	8.71	8.39	9.29	9.09	8.83	8.83
1392939_at	Slc41a3	solute carrier family 41, member 3	0	0	0	0	0	0	DR+/+nFIS_32	0	1	1	1	0	5.21	5.01	4.97	4.75	5.38	5.11	4.89	4.68	5.33	5.08	4.89	4.80	5.80	5.00	4.67	4.61	5.21	4.86	4.68	4.60
1393907_at	Pyy	peptide YY (mapped)	0	0	0	0	0	0	DR+/+nFIS_32	0	1	1	1	0	12.40	12.45	12.20	12.05	12.44	12.39	12.10	11.85	12.11	11.95	11.80	11.40	12.17	11.93	11.84	11.41	12.41	11.93	11.70	11.15
1394280_at	Rgs17	regulator of G-protein signaling 17	0	0	0	0	0	0	DR+/+nFIS_32	0	1	1	1	0	7.53	7.37	7.21	7.08	7.40	7.28	7.30	6.86	7.67	7.45	7.35	7.28	7.15	7.55	7.45	6.98	7.73	7.63	7.43	7.28
1394600_at	Rgs17	regulator of G-protein signaling 17	0	0	0	0	0	0	DR+/+nFIS_32	0	1	1	1	0	7.76	7.68	7.60	7.33	7.67	7.69	7.53	7.26	7.73	7.59	7.41	7.18	7.71	7.47	7.54	7.12	7.82	7.57	7.41	7.22
1397536_at	Camta1	calmodulin binding transcription activator 1	0	0	0	0	0	0	DR+/+nFIS_32	0	1	1	1	0	6.48	6.44	6.45	6.15	6.70	6.48	6.42	6.14	6.75	6.66	6.12	5.66	6.69	6.19	5.91	6.02	6.69	6.16	5.78	5.80
1398483_at	Rgs17	regulator of G-protein signaling 17	0	0	0	0	0	0	DR+/+nFIS_32	0	1	1	1	0	8.22	8.00	7.85	7.71	8.05	8.20	7.80	7.45	8.53	8.01	7.96	7.99	7.74	8.18	7.98	7.78	8.50	8.31	8.02	7.87
1367682_at	Mdk	midkine	0	0	0	0	0	0	DR+/+nFlyp/lyp_22	0	1	1	0	0	5.41	5.08	5.09	5.09	5.53	5.23	5.06	5.00	5.38	5.11	4.95	4.76	5.60	4.96	4.97	4.93	5.37	5.10	4.99	4.88
1370061_at	Rab3b	RAB3B, member RAS oncogene family	0	0	0	0	0	0	DR+/+nFlyp/lyp_22	0	1	1	0	0	9.49	9.46	9.39	9.19	9.57	9.38	9.36	9.00	9.56	9.62	9.44	9.10	9.33	9.46	9.35	9.13	9.45	9.33	9.22	8.99
1370264_at	Syne1	spectrin repeat containing, nuclear envelope 1	0	0	0	0	0	0	DR+/+nFlyp/lyp_22	0	1	1	0	0	9.14	9.08	9.03	9.04	9.17	9.05	8.97	8.86	9.16	8.99	8.97	8.79	9.24	8.87	8.89	8.95	8.99	8.92	8.95	8.87
1371558_at	Nisch	nischarin	0	0	0	0	0	0	DR+/+nFlyp/lyp_22	0	1	1	0	0	10.16	9.96	9.83	9.83	10.17	9.98	9.87	9.63	10.23	9.97	9.85	9.68	10.24	9.89	9.70	9.65	10.15	9.96	9.91	9.78
1372989_at	Zdhhc14	zinc finger, DHHC-type containing 14	0	0	0	0	0	0	DR+/+nFlyp/lyp_22	0	1	1	0	0	9.46	9.36	9.27	9.10	9.39	9.45	9.21	8.91	9.40	9.20	9.08	8.72	9.27	9.27	9.05	8.80	9.31	9.22	9.11	8.81
1377453_at	RGD1309108	similar to hypothetical protein FLJ23554	0	0	0	0	0	0	DR+/+nFlyp/lyp_22	0	1	1	0	0	4.15	4.08	4.00	3.77	4.31	4.06	3.95	3.59	4.11	4.18	3.82	3.43	4.61	3.90	3.73	3.49	4.13	3.97	3.66	3.77
1378886_x_at	Syne1	spectrin repeat containing, nuclear envelope 1	0	0	0	0	0	0	DR+/+nFlyp/lyp_22	0	1	1	0	0	5.51	5.48	5.38	5.00	5.64	5.53	5.16	4.75	5.56	5.47	5.11	4.76	5.84	5.24	5.13	5.07	5.48	5.24	5.10	4.80
1379452_at	Gas2	growth arrest-specific 2	0	0	0	0	0	0	DR+/+nFlyp/lyp_22	0	1	1	0	0	6.93	6.74	6.80	6.54	7.02	6.69	6.68	6.44	7.12	6.68	6.42	6.13	6.89	6.42	6.21	6.13	6.99	6.15	6.22	6.31
1379674_at	Cbfa2t3	core-binding factor, runt domain, alpha subunit 2; translocated to, 3	0	0	0	0	0	0	DR+/+nFlyp/lyp_22	0	1	1	0	0	6.13	6.00	5.72	5.86	6.21	5.83	5.61	5.75	6.29	5.75	5.75	5.77	5.44	5.62	5.73	5.67	6.06	5.75	5.65	5.58
1379873_at	---	---	0	0	0	0	0	0	DR+/+nFlyp/lyp_22	0	1	1	0	0	4.50	4.57	4.46	4.46	4.70	4.62	4.27	4.36	4.95	4.69	4.34	4.57	4.38	4.65	4.73	4.68	4.79	4.60	4.59	4.58
1382088_at	Ryr2	ryanodine receptor 2, cardiac	0	0	0	0	0	0	DR+/+nFlyp/lyp_22	0	1	1	0	0	6.78	6.74	6.71	6.60	6.83	6.94	6.63	6.61	5.93	5.75	5.37	5.03	6.23	5.71	5.17	4.95	5.76	5.55	5.36	5.16
1382775_at	Ryr2	ryanodine receptor 2, cardiac	0	0	0	0	0	0	DR+/+nFlyp/lyp_22	0	1	1	0	0	5.80	5.77	5.68	5.52	5.90	6.11	5.55	5.27	5.25	5.16	5.11	4.91	4.84	5.00	5.10	4.64	5.22	5.14	4.91	4.88
1384116_at	Vps37d	vacuolar protein sorting 37 homolog D (S. cerevisiae)	0	0	0	0	0	0	DR+/+nFlyp/lyp_22	0	1	1	0	0	7.22	7.08	6.97	7.00	7.29	7.11	6.89	6.71	7.40	7.10	6.88	6.80	7.48	6.89	6.95	6.78	7.27	6.84	6.98	6.72
1384342_at	Nisch	nischarin	0	0	0	0	0	0	DR+/+nFlyp/lyp_22	0	1	1	0	0	4.98	4.80	4.78	4.97	4.81	4.71	4.89	4.88	5.20	5.01	4.85	4.99	4.58	5.05	4.77	4.92	5.02	4.93	4.87	4.77
1385262_at	---	---	0	0	0	0	0	0	DR+/+nFlyp/lyp_22	0	1	1	0	0	4.91	4.77	4.79	4.57	4.95	4.74	4.27	4.47	4.23	3.81	3.70	3.86	3.45	3.90	3.86	3.67	4.06	3.93	3.67	4.02
1389801_at	Fgfr4	fibroblast growth factor receptor 4	0	0	0	0	0	0	DR+/+nFlyp/lyp_22	0	1	1	0	0	6.57	6.58	6.54	6.21	6.62	6.52	6.40	6.11	6.55	6.34	6.16	6.00	6.54	6.15	6.21	6.20	6.33	6.16	6.25	6.19
1391037_at	Vwa5b2	von Willebrand factor A domain containing 5B2	0	0	0	0	0	0	DR+/+nFlyp/lyp_22	0	1	1	0	0	9.39	9.24	9.11	9.02	9.42	9.24	9.10	8.82	9.39	9.17	9.00	8.87	9.44	9.02	8.91	8.85	9.39	9.12	9.19	8.91
1392640_at	Cry1	cryptochrome 1 (photolyase-like)	0	0	0	0	0	0	DR+/+nFlyp/lyp_22	0	1	1	0	0	6.76	6.43	6.36	6.50	6.87	6.68	6.35	6.34	6.77	6.66	6.21	6.13	7.29	6.74	6.28	6.41	6.81	6.49	6.41	6.35
1394957_at	Nisch	nischarin	0	0	0	0	0	0	DR+/+nFlyp/lyp_22	0	1	1	0	0	7.69	7.47	7.31	7.22	7.62	7.42	7.38	7.06	7.81	7.45	7.31	7.25	7.36	7.35	7.16	7.10	7.67	7.44	7.28	7.05
1398263_at	Syne1	spectrin repeat containing, nuclear envelope 1	0	0	0	0	0	0	DR+/+nFlyp/lyp_22	0	1	1	0	0	6.42	6.28	6.36	6.36	6.40	6.42	6.28	6.25	6.47	6.24	6.38	6.34	6.62	6.40	6.27	6.17	6.41	6.27	6.40	6.38
1398499_at	Syne1	spectrin repeat containing, nuclear envelope 1	0	0	0	0	0	0	DR+/+nFlyp/lyp_22	0	1	1	0	0	5.25	5.13	5.07	4.64	5.40	5.14	4.79	4.51	5.30	5.14	4.71	4.43	5.54	4.92	4.74	4.70	5.09	4.85	4.64	4.59
1398615_at	Rap1gap2	RAP1 GTPase activating protein 2	0	0	0	0	0	0	DR+/+nFlyp/lyp_22	0	1	1	0	0	11.49	11.23	11.24	11.07	11.53	11.35	11.16	10.97	11.50	11.19	11.16	10.89	11.48	11.19	11.16	10.93	11.35	11.17	10.98	11.04
1368288_at	Gc	group specific component	0	0	0	0	0	0	DRlyp/lypnDR+/+_48	1	1	0	0	0	12.36	12.23	12.19	12.00	12.36	12.36	12.03	11.95	12.46	12.34	12.31	12.06	12.34	12.23	12.30	12.10	12.48	12.27	12.18	12.13
1368289_at	Gc	group specific component	0	0	0	0	0	0	DRlyp/lypnDR+/+_48	1	1	0	0	0	6.49	6.11	5.87	5.63	6.55	6.05	5.79	5.75	7.49	7.37	7.39	7.24	7.31	7.36	7.38	7.26	7.61	7.38	7.43	7.48
1368300_at	Adora2a	adenosine A2a receptor	0	0	0	0	0	0	DRlyp/lypnDR+/+_48	1	1	0	0	0	5.96	5.70	5.45	5.09	5.96	5.71	5.54	5.21	5.44	5.37	5.04	4.92	5.84	5.29	5.10	4.92	5.41	5.24	5.23	5.05
1368301_at	Adora2a	adenosine A2a receptor	0	0	0	0	0	0	DRlyp/lypnDR+/+_48	1	1	0	0	0	5.03	4.86	4.68	4.60	5.04	4.98	4.87	4.68	4.75	4.65	4.61	4.60	4.62	4.69	4.65	4.81	4.68	4.82	4.52	4.71
1368432_a_at	Ros1	c-ros oncogene 1 , receptor tyrosine kinase	0	0	0	0	0	0	DRlyp/lypnDR+/+_48	1	1	0	0	0	7.24	7.07	6.61	6.62	7.21	6.88	6.71	6.60	7.20	7.14	7.11	7.04	7.69	7.24	7.18	6.94	7.49	6.96	7.14	7.05
1368471_at	Guca2a	guanylate cyclase activator 2a (guanylin)	0	0	0	0	0	0	DRlyp/lypnDR+/+_48	1	1	0	0	0	4.25	3.98	3.85	3.73	4.28	4.18	3.96	3.67	3.84	3.86	3.84	3.75	3.92	3.64	4.11	3.78	3.74	3.84	3.59	3.54
1368608_at	Cyp2f4	cytochrome P450, family 2, subfamily f, polypeptide 4	0	0	0	0	0	0	DRlyp/lypnDR+/+_48	1	1	0	0	0	5.38	5.56	5.36	4.82	5.62	5.40	5.23	5.08	5.33	5.31	5.02	4.86	5.14	4.91	4.83	4.81	5.12	4.92	4.84	4.74
1369079_at	Nxph1	neurexophilin 1	0	0	0	0	0	0	DRlyp/lypnDR+/+_48	1	1	0	0	0	5.30	4.26	4.28	4.17	5.08	4.52	4.42	4.33	4.13	4.17	4.01	3.78	4.36	4.10	4.10	3.99	4.38	3.93	4.07	4.00
1369187_a_at	Ptger3	prostaglandin E receptor 3 (subtype EP3)	0	0	0	0	0	0	DRlyp/lypnDR+/+_48	1	1	0	0	0	5.49	5.34	5.05	5.21	5.48	5.38	5.38	5.07	5.42	5.31	5.30	5.43	5.59	5.87	5.39	5.10	5.43	5.50	5.25	5.42
1369286_at	Proc	protein C	0	0	0	0	0	0	DRlyp/lypnDR+/+_48	1	1	0	0	0	6.01	5.62	5.38	5.21	6.25	5.69	5.37	5.14	5.81	5.67	5.57	5.67	5.61	5.65	5.52	5.57	6.04	5.59	5.60	5.73
1369773_at	Bmp3	bone morphogenetic protein 3	0	0	0	0	0	0	DRlyp/lypnDR+/+_48	1	1	0	0	0	6.14	6.00	5.71	5.02	6.10	6.09	5.59	4.98	3.11	3.33	3.44	3.31	3.23	3.35	3.17	3.49	3.49	3.44	3.40	3.47
1369897_s_at	Gnas	GNAS complex locus	0	0	0	0	0	0	DRlyp/lypnDR+/+_48	1	1	0	0	0	12.91	12.73	12.75	12.67	12.96	12.89	12.73	12.63	13.07	13.01	13.10	13.01	13.01	12.89	13.00	13.00	13.05	13.01	13.10	13.06
1369898_a_at	Gip	gastric inhibitory polypeptide	0	0	0	0	0	0	DRlyp/lypnDR+/+_48	1	1	0	0	0	4.36	4.09	3.78	3.67	4.74	4.08	3.79	3.86	3.74	3.90	3.76	3.82	3.87	3.61	3.73	3.89	3.47	3.85	3.69	3.63
1369955_at	Col5a1	collagen, type V, alpha 1	0	0	0	0	0	0	DRlyp/lypnDR+/+_48	1	1	0	0	0	5.92	5.55	5.28	5.13	5.94	5.65	5.29	5.26	5.58	5.34	5.10	5.19	5.81	5.47	5.34	5.24	5.77	5.37	5.27	5.20
1370864_at	Col1a1	collagen, type I, alpha 1	0	0	0	0	0	0	DRlyp/lypnDR+/+_48	1	1	0	0	0	6.73	6.60	5.97	6.13	6.94	6.55	6.06	6.38	5.92	5.84	5.77	5.67	6.45	5.87	5.95	5.95	6.31	6.10	6.34	5.89
1371092_at	LOC286960	preprotrypsinogen IV	0	0	0	0	0	0	DRlyp/lypnDR+/+_48	1	1	0	0	0	8.73	8.65	8.37	7.56	9.54	7.73	6.41	6.10	8.81	8.89	9.40	8.33	9.56	10.41	9.71	8.72	7.44	8.59	8.08	7.16
1372091_at	Mid1ip1	MID1 interacting protein 1	0	0	0	0	0	0	DRlyp/lypnDR+/+_48	1	1	0	0	0	8.38	8.10	7.98	7.81	8.42	8.16	7.95	7.85	8.55	8.39	8.34	8.15	8.56	8.16	8.16	8.02	8.50	8.32	8.42	8.10
1372294_at	Pxdn	peroxidasin homolog (Drosophila)	0	0	0	0	0	0	DRlyp/lypnDR+/+_48	1	1	0	0	0	7.25	7.09	6.68	6.58	7.31	7.15	6.69	6.76	6.79	6.60	6.47	6.40	7.06	6.67	6.63	6.58	6.92	6.75	6.56	6.57
1373217_at	Ehbp1	EH domain binding protein 1	0	0	0	0	0	0	DRlyp/lypnDR+/+_48	1	1	0	0	0	8.07	8.01	7.83	7.55	8.14	7.95	7.73	7.61	8.06	7.90	7.77	7.61	8.24	7.76	7.72	7.54	8.02	7.83	7.81	7.63
1374496_at	Tril	TLR4 interactor with leucine-rich repeats	0	0	0	0	0	0	DRlyp/lypnDR+/+_48	1	1	0	0	0	7.76	7.61	7.34	7.07	7.89	7.62	7.23	7.23	7.47	7.21	6.99	7.12	7.86	7.21	7.23	7.09	7.50	7.06	7.14	7.08
1374643_at	Fat4	FAT atypical cadherin 4	0	0	0	0	0	0	DRlyp/lypnDR+/+_48	1	1	0	0	0	5.34	5.05	4.80	4.76	5.51	5.10	4.82	4.83	4.92	4.50	4.44	4.51	5.33	4.54	4.79	4.69	5.05	5.02	4.94	4.85
1375729_at	Epha4	Eph receptor A4	0	0	0	0	0	0	DRlyp/lypnDR+/+_48	1	1	0	0	0	6.25	6.36	6.16	5.65	6.50	6.11	5.85	5.38	5.69	5.40	5.33	5.31	5.76	5.36	5.34	5.10	5.69	5.69	5.58	5.42
1375862_at	Pxdn	peroxidasin homolog (Drosophila)	0	0	0	0	0	0	DRlyp/lypnDR+/+_48	1	1	0	0	0	5.98	5.65	5.24	5.29	5.74	5.57	5.35	5.31	5.48	5.07	5.00	5.09	5.41	5.39	5.27	5.11	5.56	5.28	5.12	5.07
1376099_at	Col5a1	collagen, type V, alpha 1	0	0	0	0	0	0	DRlyp/lypnDR+/+_48	1	1	0	0	0	6.39	6.12	5.81	5.71	6.57	6.15	5.97	6.05	6.36	6.01	5.92	5.83	6.37	6.15	5.98	6.00	6.36	6.10	6.03	5.99
1376977_at	Ptger3	prostaglandin E receptor 3 (subtype EP3)	0	0	0	0	0	0	DRlyp/lypnDR+/+_48	1	1	0	0	0	6.79	6.08	4.95	4.72	6.57	5.81	4.84	5.49	6.28	5.99	5.58	5.62	7.24	7.30	5.87	5.13	6.04	6.55	5.94	6.20
1377033_at	Serpinf2	serpin peptidase inhibitor, clade F (alpha-2 antiplasmin, pigment epithelium derived factor),	0	0	0	0	0	0	DRlyp/lypnDR+/+_48	1	1	0	0	0	5.99	5.62	5.46	5.49	6.01	5.62	5.70	5.41	6.02	5.69	5.53	5.60	5.86	5.51	5.47	5.56	6.08	5.76	5.69	5.73
1379469_at	Tbl1x	transducin (beta)-like 1 X-linked	0	0	0	0	0	0	DRlyp/lypnDR+/+_48	1	1	0	0	0	6.65	6.43	6.31	6.00	6.58	6.55	6.05	6.03	6.64	6.15	6.58	6.24	6.22	6.34	6.58	5.74	6.69	6.60	6.02	6.14
1379970_at	Epha4	Eph receptor A4	0	0	0	0	0	0	DRlyp/lypnDR+/+_48	1	1	0	0	0	4.10	4.11	3.99	3.62	3.89	3.86	3.86	3.46	3.53	3.55	3.60	3.71	3.73	3.58	3.59	3.54	3.79	3.77	3.63	3.57
1382908_at	Epha4	Eph receptor A4	0	0	0	0	0	0	DRlyp/lypnDR+/+_48	1	1	0	0	0	3.82	3.73	3.73	3.49	3.82	3.73	3.61	3.26	3.37	3.38	3.49	3.66	3.20	3.38	3.42	3.16	3.53	3.48	3.32	3.44
1385215_at	Runx1t1	runt-related transcription factor 1; translocated to, 1 (cyclin D-related)	0	0	0	0	0	0	DRlyp/lypnDR+/+_48	1	1	0	0	0	3.82	3.85	3.48	3.59	4.01	3.75	3.51	3.43	4.60	4.42	4.47	4.35	4.44	4.05	4.02	4.29	4.66	4.25	4.37	4.57
1385519_at	Runx1t1	runt-related transcription factor 1; translocated to, 1 (cyclin D-related)	0	0	0	0	0	0	DRlyp/lypnDR+/+_48	1	1	0	0	0	5.34	5.22	4.96	4.67	5.48	5.00	4.88	4.68	5.63	5.31	5.32	5.44	5.30	5.17	4.97	5.31	5.66	5.20	5.25	5.33
1385799_at	Nxph1	neurexophilin 1	0	0	0	0	0	0	DRlyp/lypnDR+/+_48	1	1	0	0	0	3.64	2.48	2.37	2.17	3.33	2.70	2.48	2.25	2.37	2.06	2.06	1.95	2.78	2.21	2.00	1.93	2.32	2.09	2.14	1.91
1385954_at	LOC1001253	hypothetical protein LOC100125362	0	0	0	0	0	0	DRlyp/lypnDR+/+_48	1	1	0	0	0	3.11	3.18	3.08	2.65	3.06	3.18	2.76	2.57	3.22	3.14	2.99	2.76	3.15	3.20	2.86	2.72	3.05	3.01	2.86	2.59
1386697_at	Nxph1	neurexophilin 1	0	0	0	0	0	0	DRlyp/lypnDR+/+_48	1	1	0	0	0	4.86	3.86	3.78	3.66	4.83	3.93	3.81	3.87	3.75	3.66	3.58	3.36	4.00	3.68	3.49	3.21	3.81	3.27	3.22	3.27
1386912_at	Pcolce	procollagen C-endopeptidase enhancer	0	0	0	0	0	0	DRlyp/lypnDR+/+_48	1	1	0	0	0	7.06	6.65	6.50	6.59	7.12	6.82	6.46	6.53	7.54	7.28	7.22	7.32	7.61	7.20	7.22	7.18	7.67	7.27	7.27	7.44
1387034_at	Pah	phenylalanine hydroxylase	0	0	0	0	0	0	DRlyp/lypnDR+/+_48	1	1	0	0	0	8.12	7.89	7.70	7.59	8.33	7.94	7.69	7.55	7.80	7.61	7.72	7.66	7.49	7.95	7.67	7.43	7.74	7.78	7.55	7.65
1387906_a_at	Gnas	GNAS complex locus	0	0	0	0	0	0	DRlyp/lypnDR+/+_48	1	1	0	0	0	11.08	10.57	10.39	9.89	11.22	10.73	10.40	10.03	11.44	11.12	11.15	11.00	11.66	10.87	10.91	10.85	11.49	11.15	11.15	11.05
1388116_at	Col1a1	collagen, type I, alpha 1	0	0	0	0	0	0	DRlyp/lypnDR+/+_48	1	1	0	0	0	6.01	5.75	5.72	5.60	6.22	6.21	5.82	5.91	5.26	5.44	5.19	5.27	5.40	5.19	5.52	5.57	5.62	5.54	5.64	5.43
1388385_at	Cryba2	crystallin, beta A2	0	0	0	0	0	0	DRlyp/lypnDR+/+_48	1	1	0	0	0	9.00	8.74	8.73	8.48	9.07	8.87	8.65	8.55	8.72	8.57	8.57	8.51	8.84	8.57	8.49	8.39	8.77	8.54	8.46	8.35
1388478_at	Tbl1x	transducin (beta)-like 1 X-linked	0	0	0	0	0	0	DRlyp/lypnDR+/+_48	1	1	0	0	0	8.63	8.59	8.59	8.56	8.52	8.56	8.61	8.49	8.51	8.47	8.48	8.49	8.99	8.56	8.42	8.48	8.59	8.47	8.59	8.60
1388494_at	Col4a2	collagen, type IV, alpha 2	0	0	0	0	0	0	DRlyp/lypnDR+/+_48	1	1	0	0	0	7.45	7.24	6.87	6.94	7.52	7.29	7.05	6.90	7.13	6.76	6.74	6.71	7.48	6.85	6.81	6.71	7.15	6.96	7.03	6.98
1388618_at	Nid2	nidogen 2 (osteonidogen)	0	0	0	0	0	0	DRlyp/lypnDR+/+_48	1	1	0	0	0	7.49	7.26	6.98	6.92	7.63	7.31	6.95	7.01	7.36	7.03	7.04	7.07	7.68	7.26	7.28	7.05	7.51	7.42	7.41	7.36
1390638_at	Epha4	Eph receptor A4	0	0	0	0	0	0	DRlyp/lypnDR+/+_48	1	1	0	0	0	5.35	5.35	5.43	4.55	5.53	5.09	4.92	4.45	4.56	4.21	4.06	4.49	4.79	4.17	4.54	4.02	4.74	4.65	4.84	4.38
1392496_at	LOC312273	Trypsin V-A	0	0	0	0	0	0	DRlyp/lypnDR+/+_48	1	1	0	0	0	11.03	11.11	9.96	10.01	9.79	8.29	7.53	7.42	11.46	11.12	11.39	10.46	12.10	12.37	11.61	10.53	10.27	11.15	10.62	10.06
1393491_at	Tbl1x	transducin (beta)-like 1 X-linked	0	0	0	0	0	0	DRlyp/lypnDR+/+_48	1	1	0	0	0	6.87	6.57	6.61	6.20	6.60	6.80	6.30	5.89	6.95	6.67	6.93	6.72	6.43	6.92	6.90	6.31	6.99	6.80	6.51	6.54

Affymetrix Probeset ID	Gene Symbol	Gene Name	unique BB on P7 n=822	Figure 1B P7 Venn n=1526	DRlyp/lyp STEM P7 n=712	DR+/+ STEM P7 n=936	Flyp/lyp STEM P7 n=520	F+/+ STEM P7 n=465	Figure 1B P0 Venn n=1071	DRlyp/lyp STEM P0 n=442	DR+/+ STEM P0 n=437	Flyp/lyp STEM P0 n=643	F+/+ STEM P0 n=632	Fig1D n=25	Mean log2 intensity DRlyp/lyp 20	Mean log2 intensity DRlyp/lyp 30	Mean log2 intensity DRlyp/lyp 40	Mean log2 intensity DRlyp/lyp 50	Mean log2 intensity DR+/+ 20	Mean log2 intensity DR+/+ 30	Mean log2 intensity DR+/+ 40	Mean log2 intensity DR+/+ 50	Mean log2 intensity Flyp/lyp 20	Mean log2 intensity Flyp/lyp 30	Mean log2 intensity Flyp/lyp 40	Mean log2 intensity Flyp/lyp 50	Mean log2 intensity Flyp20	Mean log2 intensity Flyp30	Mean log2 intensity Flyp40	Mean log2 intensity Flyp50	Mean log2 intensity F+/+ 20	Mean log2 intensity F+/+ 30	Mean log2 intensity F+/+ 40	Mean log2 intensity F+/+ 50
1393901_at	Runx1t1	runt-related transcription factor 1; translocated to, 1 (cyclin D-related)	0	0	0	0	0	0	DRlyp/lypnDR+/+_48	1	1	0	0	0	4.95	4.67	4.60	4.28	4.92	4.84	4.45	4.36	6.14	5.55	5.52	5.79	5.72	5.42	5.35	5.58	5.97	5.53	5.63	5.71
1395076_at	Runx1t1	runt-related transcription factor 1; translocated to, 1 (cyclin D-related)	0	0	0	0	0	0	DRlyp/lypnDR+/+_48	1	1	0	0	0	5.24	4.90	4.78	4.61	5.08	4.85	4.70	4.51	5.92	5.69	5.54	5.71	5.70	5.30	5.47	5.50	5.91	5.49	5.60	5.73
1367598_at	Ttr	transthyretin	0	0	0	0	0	0	DRlyp/lypnF+/+_28	1	0	0	1	0	7.17	6.66	6.65	6.61	7.07	6.50	7.06	6.64	7.16	7.11	6.79	6.82	7.28	6.90	6.73	6.84	7.47	6.80	6.85	6.54
1367805_at	Gls	glutaminase	0	0	0	0	0	0	DRlyp/lypnF+/+_28	1	0	0	1	0	7.66	7.80	7.88	7.68	7.75	7.80	7.72	7.71	7.71	7.71	7.79	7.71	7.90	7.71	7.75	7.74	7.75	7.81	7.89	7.94
1367806_at	Gls	glutaminase	0	0	0	0	0	0	DRlyp/lypnF+/+_28	1	0	0	1	0	6.86	7.09	6.90	6.34	7.13	7.36	6.71	6.45	7.26	6.77	6.95	6.98	6.34	7.19	7.02	6.60	7.21	7.42	6.31	6.55
1368770_at	Gcnt1	glucosaminyl (N-acetyl) transferase 1, core 2	0	0	0	0	0	0	DRlyp/lypnF+/+_28	1	0	0	1	0	5.82	5.41	5.19	5.39	5.96	5.34	5.23	5.88	4.68	4.40	4.21	4.33	4.83	4.47	4.42	4.37	4.91	4.57	4.52	4.38
1369007_at	Nr4a2	nuclear receptor subfamily 4, group A, member 2	0	0	0	0	0	0	DRlyp/lypnF+/+_28	1	0	0	1	0	8.90	8.56	8.44	8.23	9.05	8.36	8.75	8.02	8.56	8.20	8.34	7.78	8.53	7.76	8.08	8.05	8.58	8.26	8.21	7.63
1369115_at	Adrb2	adrenoceptor beta 2, surface	0	0	0	0	0	0	DRlyp/lypnF+/+_28	1	0	0	1	0	4.31	4.08	4.22	3.56	4.01	3.99	3.98	4.01	3.88	3.60	3.86	3.50	3.95	4.08	3.71	3.40	3.80	3.91	3.85	3.25
1369248_a_at	Xiap	X-linked inhibitor of apoptosis	0	0	0	0	0	0	DRlyp/lypnF+/+_28	1	0	0	1	0	5.21	4.95	4.87	4.70	5.02	5.16	4.71	4.74	5.20	4.87	5.22	4.87	3.84	5.16	5.25	4.60	5.15	5.08	4.58	4.68
1370209_at	Klf9	Kruppel-like factor 9	0	0	0	0	0	0	DRlyp/lypnF+/+_28	1	0	0	1	0	11.39	11.15	11.05	10.63	11.35	11.17	11.00	10.86	11.58	11.52	11.38	11.05	11.59	11.41	11.11	10.86	11.35	11.45	11.23	10.86
1370312_at	Spon1	spondin 1, extracellular matrix protein	0	0	0	0	0	0	DRlyp/lypnF+/+_28	1	0	0	1	0	7.83	7.86	7.78	7.35	7.83	7.69	7.45	7.41	6.65	6.39	5.60	6.06	6.81	5.90	6.21	6.14	6.65	6.06	5.94	5.56
1370770_s_at	Kitlg	KIT ligand	0	0	0	0	0	0	DRlyp/lypnF+/+_28	1	0	0	1	0	3.98	3.80	3.70	3.47	4.11	3.88	3.71	3.65	3.80	3.66	3.44	3.62	3.57	3.54	3.82	3.37	3.89	3.85	3.35	3.31
1371032_at	Nid1	nidogen 1	0	0	0	0	0	0	DRlyp/lypnF+/+_28	1	0	0	1	0	5.70	5.60	5.50	5.42	5.64	5.55	5.58	5.48	5.83	5.70	5.69	5.63	5.71	5.78	5.67	5.52	5.81	5.74	5.64	5.51
1371517_at	Grb10	growth factor receptor bound protein 10	0	0	0	0	0	0	DRlyp/lypnF+/+_28	1	0	0	1	0	6.43	6.14	5.69	5.39	6.50	5.97	5.73	5.43	6.37	5.94	5.55	5.23	6.65	6.20	5.75	5.39	6.56	6.00	5.65	5.71
1371518_at	Nid1	nidogen 1	0	0	0	0	0	0	DRlyp/lypnF+/+_28	1	0	0	1	0	7.63	7.34	6.90	6.86	7.69	7.38	7.02	7.00	7.48	7.01	6.93	6.77	7.79	7.13	7.03	6.81	7.63	7.24	7.06	6.84
1382068_at	---	---	0	0	0	0	0	0	DRlyp/lypnF+/+_28	1	0	0	1	0	7.95	7.75	7.63	7.36	7.86	7.82	7.50	7.48	8.03	7.76	8.02	7.60	6.81	7.78	7.93	7.44	8.01	7.99	7.59	7.48
1382521_at	Gls	glutaminase	0	0	0	0	0	0	DRlyp/lypnF+/+_28	1	0	0	1	0	10.36	10.16	10.09	9.92	10.39	10.34	9.96	9.97	10.51	10.25	10.11	9.87	10.62	10.12	10.10	9.89	10.44	10.25	10.03	9.89
1382616_at	Gls	glutaminase	0	0	0	0	0	0	DRlyp/lypnF+/+_28	1	0	0	1	0	6.99	6.82	6.62	6.28	7.06	6.79	6.41	6.57	7.10	6.70	6.68	6.42	6.37	6.68	6.77	6.43	7.09	6.74	6.36	6.18
1382891_at	Usp27x	ubiquitin specific peptidase 27, X-linked	0	0	0	0	0	0	DRlyp/lypnF+/+_28	1	0	0	1	0	5.16	5.11	4.97	4.48	5.15	4.96	4.91	4.87	5.47	5.33	5.20	4.97	5.46	5.16	5.06	5.01	5.42	5.30	5.10	4.85
1384789_at	Tekt4	tektin 4	0	0	0	0	0	0	DRlyp/lypnF+/+_28	1	0	0	1	0	4.34	4.17	4.23	3.79	4.11	4.28	4.14	3.83	4.58	4.59	4.41	4.16	4.24	4.54	4.18	4.22	4.65	4.38	4.09	4.03
1386932_at	Gls	glutaminase	0	0	0	0	0	0	DRlyp/lypnF+/+_28	1	0	0	1	0	5.00	5.11	5.21	4.91	4.95	5.09	5.08	4.83	5.26	5.16	5.10	5.24	4.53	5.22	4.95	4.99	5.28	5.19	4.87	4.91
1387410_at	Nr4a2	nuclear receptor subfamily 4, group A, member 2	0	0	0	0	0	0	DRlyp/lypnF+/+_28	1	0	0	1	0	8.37	8.00	7.89	7.68	8.48	7.77	8.13	7.41	7.62	7.17	7.47	7.00	7.32	6.93	7.15	7.15	7.68	7.32	7.33	6.64
1387882_at	Klf9	Kruppel-like factor 9	0	0	0	0	0	0	DRlyp/lypnF+/+_28	1	0	0	1	0	6.64	6.60	6.74	5.94	6.67	6.49	6.64	6.35	6.84	6.89	6.83	6.59	5.69	6.90	6.52	6.18	6.74	6.71	6.38	5.96
1388096_at	Kitlg	KIT ligand	0	0	0	0	0	0	DRlyp/lypnF+/+_28	1	0	0	1	0	2.48	2.18	2.26	2.10	2.44	2.45	2.20	2.14	2.29	2.19	2.24	2.18	2.31	2.07	2.22	1.98	2.40	2.49	2.22	2.21
1388856_at	Kitlg	KIT ligand	0	0	0	0	0	0	DRlyp/lypnF+/+_28	1	0	0	1	0	7.53	7.44	7.25	6.94	7.59	7.38	7.24	7.07	7.17	6.93	6.86	6.68	7.37	6.96	6.95	6.58	7.38	7.22	7.11	6.87
1394610_at	Grb10	growth factor receptor bound protein 10	0	0	0	0	0	0	DRlyp/lypnF+/+_28	1	0	0	1	0	2.65	2.75	2.72	2.60	2.87	2.75	2.76	2.44	2.71	2.69	2.65	2.65	2.67	2.65	2.54	2.57	2.66	2.66	2.60	2.79
1396214_at	Kitlg	KIT ligand	0	0	0	0	0	0	DRlyp/lypnF+/+_28	1	0	0	1	0	5.99	5.59	5.55	5.11	5.97	5.48	5.35	5.31	5.49	5.18	5.11	5.18	5.27	5.23	5.35	4.91	5.79	5.52	5.10	5.19
1397211_at	Grb10	growth factor receptor bound protein 10	0	0	0	0	0	0	DRlyp/lypnF+/+_28	1	0	0	1	0	4.82	4.52	4.21	4.15	4.74	4.44	4.51	4.25	4.48	4.51	4.18	4.33	4.60	4.63	4.18	4.14	4.94	4.28	4.41	4.21
1398250_at	Acot1	acyl-CoA thioesterase 1	0	0	0	0	0	0	DRlyp/lypnF+/+_28	1	0	0	1	0	3.06	2.75	2.64	2.52	2.92	3.02	2.79	2.55	2.84	2.76	2.53	2.56	3.00	2.69	2.73	2.50	3.03	2.72	2.55	2.45
1368104_at	Tspan2	tetraspanin 2	0	0	0	0	0	0	DRlyp/lypnFIS_41	1	0	1	1	0	8.03	7.87	7.79	7.53	8.08	7.82	7.63	7.68	7.43	7.32	7.14	6.84	7.80	7.11	7.13	6.72	7.72	7.23	7.09	6.81
1368105_at	Tspan2	tetraspanin 2	0	0	0	0	0	0	DRlyp/lypnFIS_41	1	0	1	1	0	7.30	7.12	6.89	6.69	7.22	7.03	6.82	6.90	6.63	6.49	6.34	6.15	7.02	6.40	6.27	5.95	6.83	6.36	6.37	6.22
1368395_at	Gpc3	glypican 3	0	0	0	0	0	0	DRlyp/lypnFIS_41	1	0	1	1	0	5.79	5.77	5.48	5.03	5.67	5.71	5.44	5.30	5.55	5.38	4.97	4.90	6.20	5.52	5.24	5.29	5.79	5.22	5.38	4.77
1368671_at	Srpx	sushi-repeat-containing protein, X-linked	0	0	0	0	0	0	DRlyp/lypnFIS_41	1	0	1	1	0	6.83	6.18	5.96	6.04	6.73	6.20	6.04	6.25	6.35	5.88	5.69	5.59	6.65	5.93	5.81	5.61	6.43	5.88	5.62	5.41
1369158_at	Casr	calcium-sensing receptor	0	0	0	0	0	0	DRlyp/lypnFIS_41	1	0	1	1	0	8.12	8.13	7.96	7.44	8.20	8.12	7.78	7.83	7.67	7.44	7.10	6.90	8.06	7.70	7.44	6.95	7.47	7.50	7.23	6.85
1369218_at	Met	met proto-oncogene	0	0	0	0	0	0	DRlyp/lypnFIS_41	1	0	1	1	0	3.68	3.34	3.31	3.08	3.55	3.30	3.30	3.18	3.57	3.19	3.31	3.01	3.15	3.38	3.08	2.97	3.47	3.27	2.87	2.94
1369841_at	Hspa2	heat shock protein 2	0	0	0	0	0	0	DRlyp/lypnFIS_41	1	0	1	1	0	5.81	5.55	5.42	5.15	5.64	5.49	5.44	5.26	6.04	5.67	5.21	4.69	6.00	5.49	5.24	4.85	5.91	5.09	4.79	4.34
1369871_at	Areg	amphiregulin	0	0	0	0	0	0	DRlyp/lypnFIS_41	1	0	1	1	0	4.55	4.17	4.04	3.52	4.13	3.95	4.01	3.91	5.13	4.99	4.75	4.06	4.62	4.77	4.36	3.83	4.83	4.65	4.41	3.86
1369944_at	LOC1009111	MARCKS-related protein-like /// MARCKS-related protein-like /// MARCKS-like 1	0	0	0	0	0	0	DRlyp/lypnFIS_41	1	0	1	1	0	7.91	7.19	6.85	7.17	7.99	7.27	7.02	7.46	7.90	7.13	6.65	6.37	8.06	6.90	6.70	6.72	7.81	6.83	6.78	6.51
1370037_at	Fbn2	fibrillin 2	0	0	0	0	0	0	DRlyp/lypnFIS_41	1	0	1	1	0	4.88	4.38	4.15	4.08	4.99	3.82	3.89	4.25	4.88	4.79	4.63	4.29	5.17	4.68	4.38	4.71	5.23	4.57	4.61	4.33
1370155_at	Col1a2 /// LO	collagen, type I, alpha 2 /// collagen alpha-2(I) chain-like	0	0	0	0	0	0	DRlyp/lypnFIS_41	1	0	1	1	0	8.11	7.97	7.45	7.40	8.09	7.91	7.40	7.75	7.32	7.12	6.87	6.81	7.71	7.16	7.29	7.07	7.57	7.23	7.36	7.23
1370268_at	Kcna5	potassium voltage-gated channel, shaker-related subfamily, member 5	0	0	0	0	0	0	DRlyp/lypnFIS_41	1	0	1	1	0	7.39	7.35	7.12	6.75	7.15	7.17	6.96	6.84	7.15	7.34	7.16	6.77	7.18	7.33	6.66	6.52	7.12	7.38	7.10	6.68
1371202_a_at	Nfib	nuclear factor I/B	0	0	0	0	0	0	DRlyp/lypnFIS_41	1	0	1	1	0	7.72	7.48	7.19	7.10	7.78	7.24	7.29	7.29	7.43	7.10	6.90	6.92	7.63	7.25	6.97	6.91	7.65	7.21	7.23	6.87
1372156_at	Tmem97	transmembrane protein 97	0	0	0	0	0	0	DRlyp/lypnFIS_41	1	0	1	1	0	8.14	7.62	6.68	6.80	8.30	7.33	6.66	7.23	8.27	7.18	7.10	7.06	8.49	8.02	7.11	6.83	7.76	7.76	7.22	7.30
1372643_at	Epb41l2	erythrocyte membrane protein band 4.1-like 2	0	0	0	0	0	0	DRlyp/lypnFIS_41	1	0	1	1	0	8.25	8.02	7.72	7.69	8.25	8.00	7.77	7.86	8.27	7.95	7.84	7.42	8.19	8.05	7.82	7.48	8.21	8.05	7.85	7.69
1372818_at	Colec12	collectin sub-family member 12	0	0	0	0	0	0	DRlyp/lypnFIS_41	1	0	1	1	0	6.36	6.17	6.01	5.80	6.46	6.29	6.02	6.39	5.69	5.74	5.32	5.14	5.86	5.64	5.95	5.85	5.95	5.80	5.61	5.41
1373177_x_at	LOC679608	hypothetical protein LOC679608	0	0	0	0	0	0	DRlyp/lypnFIS_41	1	0	1	1	0	5.34	5.13	5.07	4.80	5.20	5.06	4.78	4.92	5.91	5.71	5.22	5.05	6.05	5.24	4.99	5.23	5.92	5.48	5.56	5.36
1374446_at	Tiparp	TCDD-inducible poly(ADP-ribose) polymerase	0	0	0	0	0	0	DRlyp/lypnFIS_41	1	0	1	1	0	7.37	7.47	7.27	6.94	7.78	7.35	8.05	7.68	7.84	7.66	7.57	7.08	7.75	7.09	7.63	7.31	7.62	7.31	7.17	7.09
1376748_at	Arx	aristaless related homeobox	0	0	0	0	0	0	DRlyp/lypnFIS_41	1	0	1	1	0	10.12	9.91	9.65	9.31	10.11	9.94	9.51	9.14	9.96	9.51	9.27	8.59	10.00	9.60	9.22	8.80	9.97	9.46	9.13	8.86
1376973_at	Sdcbp2	syndecan binding protein (syntenin) 2	0	0	0	0	0	0	DRlyp/lypnFIS_41	1	0	1	1	0	5.46	5.09	5.04	4.87	5.34	5.14	5.24	5.04	5.78	5.41	5.27	5.38	5.44	5.31	5.18	5.02	5.82	5.30	5.31	5.21
1377632_at	Timp4	tissue inhibitor of metalloproteinase 4	0	0	0	0	0	0	DRlyp/lypnFIS_41	1	0	1	1	0	4.97	4.52	4.41	4.22	4.60	4.40	4.71	4.44	5.13	4.94	4.49	4.49	5.36	5.02	4.82	4.46	5.15	4.72	4.54	4.47
1378133_at	Slc7a11	solute carrier family 7 (anionic amino acid transporter light chain, xc- system), member 11	0	0	0	0	0	0	DRlyp/lypnFIS_41	1	0	1	1	0	6.38	6.34	5.80	5.87	6.20	6.07	5.70	6.22	6.62	6.34	6.04	5.70	6.43	6.64	5.87	5.50	6.53	6.37	6.07	5.90
1379894_at	RGD1310110	similar to 3632451O06Rik protein	0	0	0	0	0	0	DRlyp/lypnFIS_41	1	0	1	1	0	5.62	5.42	5.18	5.02	5.35	5.35	5.11	5.18	5.99	5.65	5.22	4.72	5.69	5.34	5.12	4.73	5.88	5.13	4.98	4.86
1383824_at	Ldb2	LIM domain binding 2	0	0	0	0	0	0	DRlyp/lypnFIS_41	1	0	1	1	0	5.26	5.06	4.79	4.55	5.02	4.97	4.79	4.96	4.58	4.74	4.31	4.21	4.97	4.60	4.38	4.26	4.86	4.64	4.42	4.31
1384202_at	Tesc	tescalcin	0	0	0	0	0	0	DRlyp/lypnFIS_41	1	0	1	1	0	5.07	4.91	4.52	4.60	5.42	4.73	4.45	5.43	5.47	4.68	4.34	4.51	5.48	5.03	4.49	4.40	5.17	4.91	4.45	4.68
1385014_at	Nudt10	nudix (nucleoside diphosphate linked moiety X)-type motif 10	0	0	0	0	0	0	DRlyp/lypnFIS_41	1	0	1	1	0	9.48	9.16	9.11	8.81	9.33	9.34	8.92	8.92	9.38	9.09	8.77	8.70	9.25	9.00	8.92	8.71	9.33	8.96	8.82	8.69
1386979_at	Serinc5	serine incorporator 5	0	0	0	0	0	0	DRlyp/lypnFIS_41	1	0	1	1	0	7.37	7.17	6.83	6.54	7.20	7.06	6.79	6.72	7.34	7.04	6.70	6.20	7.43	6.94	6.65	6.29	7.33	6.95	6.58	6.48
1386987_at	Il6r	interleukin 6 receptor	0	0	0	0	0	0	DRlyp/lypnFIS_41	1	0	1	1	0	9.37	9.15	8.87	8.81	9.12	9.01	9.00	8.75	9.36	9.18	8.85	8.46	9.54	9.17	8.79	8.56	9.37	8.97	8.76	8.61
1387028_a_at	Id1	inhibitor of DNA binding 1	0	0	0	0	0	0	DRlyp/lypnFIS_41	1	0	1	1	0	8.12	7.91	7.61	7.79	7.96	7.74	8.02	8.00	8.00	7.88	7.52	7.37	8.20	7.99	7.67	7.34	8.10	7.87	7.56	7.66
1387854_at	Col1a2 /// LO	collagen, type I, alpha 2 /// collagen alpha-2(I) chain-like	0	0	0	0	0	0	DRlyp/lypnFIS_41	1	0	1	1	0	7.83	7.51	6.99	6.87	7.98	7.71	6.91	7.31	7.05	6.70	6.59	6.31	7.53	6.74	6.81	6.67	7.36	6.93	6.76	6.63
1388167_at	Nfib	nuclear factor I/B	0	0	0	0	0	0	DRlyp/lypnFIS_41	1	0	1	1	0	7.06	6.68	6.56	6.39	6.91	6.58	6.38	6.43	6.73	6.37	6.13	6.12	6.79	6.45	6.21	6.19	6.93	6.32	6.41	6.19
1388250_at	Nfib	nuclear factor I/B	0	0	0	0	0	0	DRlyp/lypnFIS_41	1	0	1	1	0	3.78	3.63	3.63	3.84	3.59	3.84	3.78	3.59	3.54	3.71	3.90	3.82	3.74	3.66	3.82	3.73	3.74	3.66	3.87	3.76
1390045_at	Arx	aristaless related homeobox	0	0	0	0	0	0	DRlyp/lypnFIS_41	1	0	1	1	0	4.44	4.34	4.29	3.99	4.25	4.27	4.31	4.31	4.41	4.22	4.13	4.11	4.11	4.37	4.23	3.97	4.32	4.37	4.26	4.29
1390396_at	Eif4e3	eukaryotic translation initiation factor 4E family member 3	0	0	0	0	0	0	DRlyp/lypnFIS_41	1	0	1	1	0	7.15	6.94	6.87	6.79	7.08	6.93	6.74	6.75	7.13	6.92	6.78	6.27	7.15	6.99	6.89	6.76	7.35	7.18	7.04	6.84
1391563_at	Mum1l1	melanoma associated antigen (mutated) 1-like 1	0	0	0	0	0	0	DRlyp/lypnFIS_41	1	0	1	1	0	7.54	7.35	7.24	7.04	7.25	7.27	7.18	7.01	7.72	7.52	7.31	6.73	7.91	7.63	6.93	6.63	7.80	7.31	7.12	6.76
1391935_at	Eif4e3	eukaryotic translation initiation factor 4E family member 3	0	0	0	0	0	0	DRlyp/lypnFIS_41	1	0	1	1	0	7.05	6.87	6.63	6.40	6.98	6.91	6.46	6.42	7.06	6.81	6.56	5.88	6.93	6.83	6.55	5.98	6.97	6.78	5.88	5.62
1392512_at	Hist3h2ba	histone cluster 3, H2ba	0	0	0	0	0	0	DRlyp/lypnFIS_41	1	0	1	1	0	6.71	6.51	6.39	5.99	6.63	6.61	6.33	6.14	6.73	6.77	6.56	6.21	6.71	6.64	6.51	6.18	6.76	6.65	6.33	6.05
1393641_at	Blnk	B-cell linker	0	0	0	0	0	0	DRlyp/lypnFIS_41	1	0	1	1	0	8.57	8.07	7.84	7.97	8.64	8.16	7.77	8.30	8.37	7.79	7.66	7.18	8.36	7.78	7.58	7.11	8.34	7.92	7.74	7.13
1393659_at	Tram1l1	translocation associated membrane protein 1-like 1	0	0	0	0	0	0	DRlyp/lypnFIS_41	1	0	1	1	0	4.62	4.66	4.46	4.14	4.59	4.61	4.34	4.17	4.71	4.76	4.58	4.19	4.92	4.84	4.52	4.11	4.77	4.38	4.43	4.13
1394200_at	Hspa2	heat shock protein 2	0	0	0	0	0	0	DRlyp/lypnFIS_41	1	0	1	1	0	5.24	4.97	4.73	4.35	5.09	4.82	4.71	4.72	5.64	5.19	4.76	4.21	5.98	4.86	4.72	4.21	5.46	4.69	4.39	3.92
1396086_at	Tmem97	transmembrane protein 97	0	0	0	0	0	0	DRlyp/lypnFIS_41	1	0	1	1	0	7.11	6.70	5.87	5.95	7.22	6.24	5.96	6.49	7.38	6.39	6.12	6.38	7.48	7.27	6.29	6.09	6.86	6.73	6.31	6.32
1367627_at	Gatm	glycine amidinotransferase (L-arginine:glycine amidinotransferase)	0	0	0	0	0	0	DRlyp/lypnFlyp/lyp_52	1	0	1	0	0	6.70	6.76	5.97	5.75	6.78	6.50	5.95	6.38	6.98	5.94	5.96	5.83	7.02	7.99	6.40	5.56	6.32	7.29	6.27	6.69
1367838_at	Cth	cystathionase (cystathionine gamma-lyase)	0	0	0	0	0	0	DRlyp/lypnFlyp/lyp_52	1	0	1	0	0	5.62	5.18	4.02	3.56	5.25	4.70	4.03	4.83	6.50	4.47	4.67	4.16	6.27	6.61	4.45	3.60	5.51	5.49	4.55	5.13
1367851_at	Ptgds	prostaglandin D2 synthase (brain)	0	0	0	0	0	0	DRlyp/lypnFlyp/lyp_52	1	0	1	0	0	6.90	6.77	6.78	6.32	6.97	6.96	6.65	6.50	7.65	7.25	6.88	7.11	7.44	7.08	6.89	6.89	7.57	6.69	6.80	7.18
1368113_at	Tff2	trefoil factor 2	0	0	0	0	0	0	DRlyp/lypnFlyp/lyp_52	1	0	1	0	0	8.73	9.18	6.82	6.92	9.38	8.67	7.18	8.09	9.31	7.85	7.78	7.11	8.58	9.55	7.72	7.10	8.36	9.38	7.78	8.32
1368196_at	Clps	colipase, pancreatic	0	0	0	0	0	0	DRlyp/lypnFlyp/lyp_52	1	0	1	0	0	12.02	12.12	10.65	10.84	12.20	11.84	10.92	11.77	12.46	11.75	11.60	11.32	12.30	12.72	11.68	10.93	12.05	12.49	11.72	12.20
1368342_at	Ampd3	adenosine monophosphate deaminase 3	0	0	0	0	0	0	DRlyp/lypnFlyp/lyp_52	1	0	1	0	0	6.11	5.94	5.80	5.52	6.07	5.94	5.87	5.76	5.71	5.48	5.07	4.94	5.75	5.23	5.21	4.95	5.43	5.15	5.06	4.94
1368367_at	Cuzd1	CUB and zona pellucida-like domains 1	0	0	0	0	0	0	DRlyp/lypnFlyp/lyp_52	1	0	1	0	0	6.63	6.33	4.88	5.32	6.44	6.20	5.13	6.03	9.26	7.93	8.11	7.53	8.96	9.51	8.10	7.44	8.44	9.12	7.85	8.63
1368396_at	Cel	carboxyl ester lipase	0	0	0	0	0	0	DRlyp/lypnFlyp/lyp_52	1	0	1	0	0	12.10	12.35	10.84	10.88	12.48	12.05	11.16	11.86	12.39	11.47	11.58	11.16	12.06	12.77	11.74	11.07	11.87	12.48	11.55	12.20
1368532_at	Pnliprp1	pancreatic lipase-related protein 1	0	0	0	0	0	0	DRlyp/lypnFlyp/lyp_52	1	0	1	0	0	12.28	12.21	10.95	11.04	12.43	11.96	11.11	11.93	12.55	11.75	11.57	11.39	12.50	12.82	11.83	11.08	12.28	12.46	11.83	12.40
1368586_at	Zg16	zymogen granule protein 16	0	0	0	0	0	0	DRlyp/lypnFlyp/lyp_52	1	0	1	0	0	11.52	11.71	10.11	10.02	11.67	11.40	10.36	11.19	12.20	11.48	11.24	10.94	11.80	12.51	11.39	10.61	11.60	12.26	11.27	11.76
1369302_at	Gper	G protein-coupled estrogen receptor 1	0	0	0	0	0	0	DRlyp/lypnFlyp/lyp_52	1	0	1	0	0	4.31	4.01	3.76	3.57	4.07	4.05	3.64	3.60	4.03	3.65	3.49	3.54	3.83	3.81	3.71	3.43	4.14	3.75	3.60	3.77
1369503_at	Amy2a3	amylase 2a3	0	0	0	0	0	0	DRlyp/lypnFlyp/lyp_52	1	0	1	0	0	12.80	12.72	12.22	12.20	12.74	12.68	12.22	12.68	12.90	12.56	12.34	12.28	12.76	12.81	12.39	12.22	12.72	12.89	12.47	12.72
1369657_at	Cpa1	carboxypeptidase A1 (pancreatic)	0	0	0	0	0	0	DRlyp/lypnFlyp/lyp_52	1	0	1	0	0	11.77	12.07	10.70	10.62	12.04	11.81	10.94	11.70	12.17	11.60	11.62	11.26	11.88	12.75	11.81	10.96	11.75	12.48	11.59	12.14
1369748_at	Serpini2	serpin peptidase inhibitor, clade I (pancpin), member 2	0	0	0	0	0	0	DRlyp/lypnFlyp/lyp_52	1	0	1	0	0	8.22	8.60	6.86	6.79	8.55	8.16	7.03	8.06	8.43	7.46	7.31	7.08	8.44	9.32	7.70	7.08	7.92	8.66	7.49	8.23
1369769_at	Kcne1	potassium voltage-gated channel, Isk-related family, member 1	0	0	0	0	0	0	DRlyp/lypnFlyp/lyp_52	1	0	1	0	0	5.38	5.73	4.59	4.79	5.82	5.39	4.90	5.39	5.87	5.36	5.18	5.24	5.70	6.71	5.34	5.04	5.66	6.25	5.60	6.07
1369803_at	Ptf1a	pancreas specific transcription factor, 1a	0	0	0	0	0	0	DRlyp/lypnFlyp/lyp_52	1	0	1	0	0	4.95	5.23	4.14	4.12	5.44	4.75	4.53	4.80	5.60	4.75	4.72	4.36	6.01	5.99	5.06	4.20	5.27	5.34	4.76	5.26
1369953_a_at	Cd24	CD24 molecule	0	0	0	0	0	0	DRlyp/lypnFlyp/lyp_52	1	0	1	0	0	9.01	8.74	8.61	8.41	9.01	8.73	8.63	8.67	8.96	9.00	8.76	8.45	8.76	8.68	8.70	8.54	8.86	8.81	8.52	8.39
1370084_at	Cpb1	carboxypeptidase B1 (tissue)	0	0	0	0	0	0	DRlyp/lypnFlyp/lyp_52	1	0	1	0	0	13.07	13.16	12.18	12.15	13.21	13.07	12.36	12.85	13.29	12.84	12.85	12.51	13.09	13.47	12.99	12.35	12.96	13.31	12.77	13.06
1370107_at	Ctrl	chymotrypsin-like	0	0	0	0	0	0	DRlyp/lypnFlyp/lyp_52	1	0	1	0	0	12.29	12.65	11.23	11.40	12.48	12.40	11.51	12.28	12.83	12.19	12.03	11.78	12.47	13.18	12.30	11.50	12.38	13.01	12.23	12.64
1370126_at	LOC1009120	anionic trypsin-2-like /// protease, serine, 2	0	0	0	0	0	0	DRlyp/lypnFlyp/lyp_52	1	0	1	0	0	12.33	12.54	10.86	10.56	12.56	12.34	11.11	11.78	12.79	12.12	11.94	11.28	12.30	13.07	12.16	11.14	12.56	13.01	12.23	12.40
1370257_at	Pla2g1b	phospholipase A2, group IB, pancreas	0	0	0	0	0	0	DRlyp/lypnFlyp/lyp_52	1	0	1	0	0	11.77	11.96	10.64	10.81	11.95	11.72	10.93	11.77	12.31	11.66	11.58	11.43	12.13	12.66	11.86	11.40	11.63	12.33	11.51	12.20
1370359_at	Amy1a	amylase, alpha 1A (salivary)	0	0	0	0	0	0	DRlyp/lypnFlyp/lyp_52	1	0	1	0	0	4.69	4.75	3.25	3.10	4.64	4.27	3.64	4.28	4.88	3.81	3.51	3.32	3.74	5.49	3.50	3.38	4.19	4.78	3.54	4.79
1370837_at	Sycn	syncollin	0	0	0	0	0	0	DRlyp/lypnFlyp/lyp_52	1	0	1	0	0	11.38	11.82	10.55	10.78	11.47	11.29	10.79	11.65	11.91	11.63	11.42	11.23	11.85	12.65	11.64	10.93	11.48	12.35	11.62	12.18
1370959_at	Col3a1	collagen, type III, alpha 1	0	0	0	0	0	0	DRlyp/lypnFlyp/lyp_52	1	0	1	0	0	9.97	9.83	9.27	9.25	9.96	9.85	9.23	9.73	9.33	9.07	8.85	8.76	9.75	9.20	9.24	9.09	9.55	9.36	9.24	9.06
1371462_at	Igfbp4	insulin-like growth factor binding protein 4	0	0	0	0	0	0	DRlyp/lypnFlyp/lyp_52	1	0	1	0	0	6.81	6.38	6.26	6.33	6.57	6.38	6.44	6.14	6.61	6.33	6.26	5.92	6.62	6.41	6.07	5.82	6.47	6.24	6.15	6.00
1376972_at	Slc40a1	solute carrier family 40 (iron-regulated transporter), member 1	0	0	0	0	0	0	DRlyp/lypnFlyp/lyp_52	1	0	1	0	0	3.88	3.79	3.54	3.40	3.81	3.49	3.50	3.55	4.19	3.80	3.89	3.68	4.36	3.90	3.72	3.75	4.19	3.79	3.91	4.01
1377635_at	Fmo2	flavin containing monooxygenase 2	0	0	0	0	0	0	DRlyp/lypnFlyp/lyp_52	1	0	1	0	0	5.47	5.03	4.79	4.73	5.22	4.51	5.24	4.66	5.19	4.81	4.43	4.21	5.25	4.71	4.48	4.69	5.21	4.40	4.48	4.78
1377758_at	Hsd17b13	hydroxysteroid (17-beta) dehydrogenase 13	0	0	0	0	0	0	DRlyp/lypnFlyp/lyp_52	1	0	1	0	0	4.72	4.44	3.33	3.45	4.80	3.94	3.59	4.05	5.53	4.24	4.29	3.95	5.62	5.67	4.11	3.74	4.97	4.94	4.12	4.53
1378896_at	LOC1009117	zinc transporter 2-like /// solute carrier family 30 (zinc transporter), member 2	0	0	0	0	0	0	DRlyp/lypnFlyp/lyp_52	1	0	1	0	0	4.21	4.07	3.69	3.76	4.22	3.93	3.90	4.00	4.68	4.17	3.83	4.03	5.45	4.54	3.96	3.89	4.32	4.11	4.09	4.12
1381776_at	Tmed6	transmembrane emp24 protein transport domain containing 6	0	0	0	0	0	0	DRlyp/lypnFlyp/lyp_52	1	0	1	0	0	6.17	6.32	4.93	4.77	6.42	5.94	5.17	5.90	6.43	5.47	5.42	5.15	6.34	7.37	5.78	5.10	5.90	6.53	5.64	6.29
1382532_at	Cela3b	chymotrypsin-like elastase family, member 3B	0	0	0	0	0	0	DRlyp/lypnFlyp/lyp_52	1	0	1	0	0	12.20	12.25	11.08	11.02	12.32	12.05	11.23	11.76	12.68	12.13	12.14	11.70	12.66	13.03	12.24	11.37	12.27	12.83	12.14	12.43
1384063_at	Cthrc1	collagen triple helix repeat containing 1	0	0	0	0	0	0	DRlyp/lypnFlyp/lyp_52	1	0	1	0	0	4.62	4.46	4.33	4.09	4.63	4.44	4.20	4.39	5.10	4.66	4.73	4.55	5.18	4.77	4.79	4.74	4.95	4.74	4.68	4.70
1384778_at	Ctrc	chymotrypsin C (caldecrin)	0	0	0	0	0	0	DRlyp/lypnFlyp/lyp_52	1	0	1	0	0	12.08	12.44	11.40	11.57	12.29	12.20	11.69	12.35	12.55	12.14	12.21	11.93	12.08	12.99	12.38	11.71	12.08	12.82	12.23	12.69
1387130_at	LOC1009118	solute carrier family 40 member 1-like /// solute carrier family 40 (iron-regulated transporte	0	0	0	0	0	0	DRlyp/lypnFlyp/lyp_52	1	0	1	0	0	7.42	7.23	6.97	6.75	7.25	6.96	6.90	7.11	7.82	7.49	7.35	7.00	7.52	7.41	7.25	7.10	7.85	7.19	7.21	7.23
1387471_at	Cela2a	chymotrypsin-like elastase family, member 2A	0	0	0	0	0	0	DRlyp/lypnFlyp/lyp_52	1	0	1	0	0	12.64	12.83	11.96	12.06	12.73	12.65	12.18	12.66	12.90	12.61	12.55	12.28	12.72	13.19	12.71	12.12	12.62	13.02	12.64	12.85
1387516_at	Pnliprp2	pancreatic lipase-related protein 2	0	0	0	0	0	0	DRlyp/lypnFlyp/lyp_52	1	0	1	0	0	11.61	11.43	9.79	9.71	11.65	11.01	9.98	10.82	12.11	11.15	10.90	10.48	12.07	12.27	11.03	10.14	11.70	11.98	11.06	11.58
1387819_at	Cela1	chymotrypsin-like elastase family, member 1	0	0	0	0	0	0	DRlyp/lypnFlyp/lyp_52	1	0	1	0	0	12.44	12.77	11.69	11.83	12.53	12.60	11.93	12.54	12.81	12.36	12.31	12.01	12.34	13.12	12.46	11.82	12.32	12.98	12.38	12.71
1387891_at	Prdx4	peroxiredoxin 4	0	0	0	0	0	0	DRlyp/lypnFlyp/lyp_52	1	0	1	0	0	9.59	9.62	9.11	8.98	9.83	9.47	9.10	9.40	9.90	9.29	9.15	9.01	9.78	10.11	9.33	8.94	9.55	9.76	9.15	9.45
1387967_at	LOC1009115	serine protease inhibitor Kazal-type 3-like /// serine peptidase inhibitor, Kazal type 1	0	0	0	0	0	0	DRlyp/lypnFlyp/lyp_52	1	0	1	0	0	8.60	9.06	7.63	7.66	8.73	8.48	7.83	8.92	9.45	9.02	8.91	8.51	9.52	10.61	9.09	8.33	8.76	9.89	8.93	9.51
1388186_at	Prss3	protease, serine, 3	0	0	0	0	0	0	DRlyp/lypnFlyp/lyp_52	1	0	1	0	0	13.00	13.23	12.30	12.46	13.16	13.09	12.51	13.01	13.15	12.76	12.85	12.64	12.87	13.37	12.94	12.53	12.76	13.25	12.77	13.13
1388422_at	Lims2	LIM and senescent cell antigen like domains 2	0	0	0	0	0	0	DRlyp/lypnFlyp/lyp_52	1	0	1	0	0	6.70	6.39	6.19	6.08	6.64	6.32	6.38	6.29	6.61	6.27	6.05	5.85	6.52	6.36	6.12	5.83	6.66	6.41	6.21	6.20
1390326_at	Ang1	angiogenin, ribonuclease A family, member 1	0	0	0	0	0	0	DRlyp/lypnFlyp/lyp_52	1	0	1	0	0	4.89	4.86	4.35	4.33	5.01	4.63	4.52	4.61	5.55	5.29	5.21	4.96	5.62	6.52	5.35	4.74	5.45	6.16	5.36	5.96
1390412_at	LOC1009118	solute carrier family 40 member 1-like /// solute carrier family 40 (iron-regulated transporte	0	0	0	0	0	0	DRlyp/lypnFlyp/lyp_52	1	0	1	0	0	7.63	7.41	7.20	7.01	7.63	7.32	7.23	7.17	8.07	7.68	7.62	7.35	8.08	7.62	7.46	7.42	7.98	7.43	7.41	7.54
1390783_at	Abca8a	ATP-binding cassette, subfamily A (ABC1), member 8a	0	0	0	0	0	0	DRlyp/lypnFlyp/lyp_52	1	0	1	0	0	7.22	7.46	7.17	6.85	7.08	7.28	7.15	6.92	6.63	7.01	6.45	6.31	6.78	6.92	6.70	6.53	6.84	6.94	6.90	6.52
1391352_at	Erp27	endoplasmic reticulum protein 27	0	0	0	0	0	0	DRlyp/lypnFlyp/lyp_52	1	0	1	0	0	7.97	8.12	6.54	6.52	8.41	7.60	6.66	7.53	8.32	7.50	7.27	6.99	8.61	9.07	7.46	6.66	7.80	8.46	7.47	8.08
1391581_at	---	---	0	0	0	0	0	0	DRlyp/lypnFlyp/lyp_52	1	0	1	0	0	7.13	6.74	6.65	6.67	6.72	6.77	6.56	6.38	7.25	7.00	6.97	6.85	6.65	7.17	7.04	6.94	7.14	6.99	6.81	6.75
1392482_at	Grpel2	GrpE-like 2, mitochondrial	0	0	0	0	0	0	DRlyp/lypnFlyp/lyp_52	1	0	1	0	0	6.35	6.18	5.82	5.98	6.44	6.06	6.01	6.06	6.11	5.92	5.83	5.59	6.32	5.91	5.78	5.75	6.03	5.75	5.71	5.70

Affymetrix Probeset ID	Gene Symbol	Gene Name	unique BB on P7 n=822	Figure 1B P7 Venn n=1526	DRlyp/lyp STEM P7 n=712	DR+/+ STEM P7 n=936	Flyp/lyp STEM P7 n=520	F+/+ STEM P7 n=465	Figure 1B P0 Venn n=1071	DRlyp/lyp STEM P0 n=442	DR+/+ STEM P0 n=437	Flyp/lyp STEM P0 n=643	F+/+ STEM P0 n=632	Fig1D n=25	Mean log2 intensity DRlyp/lyp 20	Mean log2 intensity DRlyp/lyp 30	Mean log2 intensity DRlyp/lyp 40	Mean log2 intensity DRlyp/lyp 50	Mean log2 intensity DR+/+ 20	Mean log2 intensity DR+/+ 30	Mean log2 intensity DR+/+ 40	Mean log2 intensity DR+/+ 50	Mean log2 intensity Flyp/lyp 20	Mean log2 intensity Flyp/lyp 30	Mean log2 intensity Flyp/lyp 40	Mean log2 intensity Flyp/lyp 50	Mean log2 intensity Flyp20	Mean log2 intensity Flyp30	Mean log2 intensity Flyp40	Mean log2 intensity Flyp50	Mean log2 intensity F+/+ 20	Mean log2 intensity F+/+ 30	Mean log2 intensity F+/+ 40	Mean log2 intensity F+/+ 50
1392798_at	Pdia2	protein disulfide isomerase family A, member 2	0	0	0	0	0	0	DRlyp/lypnFlyp/lyp_52	1	0	1	0	0	7.60	7.66	6.06	6.24	8.03	7.23	6.34	7.21	8.24	7.11	6.91	6.66	8.14	8.69	7.11	6.54	7.69	7.23	7.15	7.70
1392864_at	Arhgap5	Rho GTPase activating protein 5	0	0	0	0	0	0	DRlyp/lypnFlyp/lyp_52	1	0	1	0	0	9.65	9.45	9.25	8.98	9.42	9.31	9.04	8.88	9.83	9.47	9.53	8.92	8.67	9.28	9.51	8.98	9.72	9.48	9.14	9.20
1393026_at	Rnase1l2	ribonuclease, RNase A family, 1-like 2 (pancreatic)	0	0	0	0	0	0	DRlyp/lypnFlyp/lyp_52	1	0	1	0	0	12.48	12.86	11.73	11.92	12.84	12.25	11.85	12.65	12.73	12.11	12.06	11.93	12.45	13.03	12.35	11.72	12.39	12.89	12.27	12.73
1393397_at	Cpa2	carboxypeptidase A2 (pancreatic)	0	0	0	0	0	0	DRlyp/lypnFlyp/lyp_52	1	0	1	0	0	12.52	12.75	11.69	11.76	12.71	12.56	11.87	12.57	12.76	12.40	12.33	12.04	12.61	13.16	12.55	11.92	12.48	13.05	12.43	12.82
1398264_at	LOC1009117(	zinc transporter 2-like /// solute carrier family 30 (zinc transporter), member 2	0	0	0	0	0	0	DRlyp/lypnFlyp/lyp_52	1	0	1	0	0	4.14	3.85	3.47	3.60	4.09	3.90	3.53	3.78	4.57	3.76	3.77	3.83	4.93	4.65	3.83	3.87	4.28	4.28	3.82	3.99
1367585_a_at	Atp1a1	ATPase, Na+/K+ transporting, alpha 1 polypeptide	0	0	0	0	0	0	Flyp/lypnF+/+_151	0	0	1	1	0	10.48	10.22	10.15	10.19	10.47	10.20	10.16	10.05	10.45	10.13	10.09	10.00	10.61	10.18	10.02	9.96	10.42	10.12	10.04	9.92
1367676_at	Hmgb2 /// LC	high mobility group box 2 /// high mobility group protein B2-like /// high mobility group prot	0	0	0	0	0	0	Flyp/lypnF+/+_151	0	0	1	1	0	8.01	8.06	7.67	8.23	8.09	8.12	7.66	9.03	8.21	8.17	7.91	7.34	8.03	7.78	7.87	7.78	8.15	8.00	7.63	7.48
1367733_at	Ca2	carbonic anhydrase 2	0	0	0	0	0	0	Flyp/lypnF+/+_151	0	0	1	1	0	6.04	5.87	5.77	5.77	5.99	6.00	5.74	5.92	6.57	6.19	6.06	5.70	6.64	6.14	6.00	5.89	6.47	5.83	5.83	5.57
1367745_at	Dgkz	diacylglycerol kinase zeta	0	0	0	0	0	0	Flyp/lypnF+/+_151	0	0	1	1	0	6.70	6.62	6.51	6.40	6.62	6.58	6.53	6.35	6.43	6.21	6.12	5.83	6.53	6.27	5.91	5.92	6.36	6.02	5.82	5.73
1367816_at	Hopx	HOP homeobox	0	0	0	0	0	0	Flyp/lypnF+/+_151	0	0	1	1	0	7.73	7.76	7.54	7.61	7.60	7.51	7.57	7.58	8.19	8.09	7.68	7.21	8.89	8.27	7.73	7.51	8.34	7.82	7.48	7.19
1367880_at	Lamb2	laminin, beta 2	0	0	0	0	0	0	Flyp/lypnF+/+_151	0	0	1	1	0	7.15	6.94	6.73	6.73	7.17	6.82	6.95	6.94	6.80	6.55	6.40	6.28	7.28	6.67	6.46	6.25	7.05	6.64	6.62	6.37
1367939_at	Rbp1	retinol binding protein 1, cellular	0	0	0	0	0	0	Flyp/lypnF+/+_151	0	0	1	1	0	9.74	9.69	9.67	9.47	9.77	9.64	9.53	9.42	9.40	9.27	9.11	8.86	9.21	9.19	9.15	8.83	9.23	9.16	9.01	8.74
1367944_at	Chst10	carbohydrate sulfotransferase 10	0	0	0	0	0	0	Flyp/lypnF+/+_151	0	0	1	1	0	6.25	5.95	5.96	5.87	6.01	5.85	5.96	5.81	6.47	6.52	6.10	5.80	6.82	6.28	6.14	5.82	6.44	5.90	5.78	5.59
1367986_at	Ptgfrn	prostaglandin F2 receptor inhibitor	0	0	0	0	0	0	Flyp/lypnF+/+_151	0	0	1	1	0	4.83	4.79	4.51	4.59	4.90	4.72	4.56	4.60	4.81	4.65	4.42	4.24	5.15	4.69	4.51	4.32	5.03	4.70	4.68	4.36
1368077_at	Fbp1	fructose-1,6-bisphosphatase 1	0	0	0	0	0	0	Flyp/lypnF+/+_151	0	0	1	1	0	5.11	5.25	5.00	5.60	5.31	5.27	5.31	5.14	5.56	5.18	4.98	4.94	5.70	5.65	5.18	5.05	5.56	5.40	5.01	5.14
1368162_at	Cst6	cystatin E/M	0	0	0	0	0	0	Flyp/lypnF+/+_151	0	0	1	1	0	9.71	9.60	9.52	9.35	9.75	9.59	9.40	9.33	10.10	9.76	9.55	9.52	10.13	9.69	9.63	9.52	10.15	9.74	9.62	9.60
1368174_at	Egln3	EGL nine homolog 3 (C. elegans)	0	0	0	0	0	0	Flyp/lypnF+/+_151	0	0	1	1	0	4.87	4.95	4.74	4.48	4.93	4.57	4.62	4.82	4.65	4.84	4.44	4.24	5.04	4.54	4.53	4.25	4.81	4.42	4.38	4.04
1368321_at	Egr1	early growth response 1	0	0	0	0	0	0	Flyp/lypnF+/+_151	0	0	1	1	0	12.62	12.62	12.47	12.27	12.73	12.61	12.68	12.28	12.57	12.51	12.46	12.06	12.60	12.09	12.25	12.21	12.50	12.35	12.35	11.84
1368502_at	Gast	gastrin	0	0	0	0	0	0	Flyp/lypnF+/+_151	0	0	1	1	0	6.67	6.87	6.71	6.45	6.80	6.92	6.51	6.46	6.21	6.18	5.67	5.42	6.45	5.81	5.67	5.50	6.36	5.78	5.47	5.17
1368536_at	Enpp2	ectonucleotide pyrophosphatase/phosphodiesterase 2	0	0	0	0	0	0	Flyp/lypnF+/+_151	0	0	1	1	0	8.46	8.31	8.30	8.43	8.56	8.40	8.13	8.79	8.15	7.70	7.58	7.08	8.05	7.66	7.64	7.49	8.15	7.76	7.35	7.31
1368642_at	Cdh2	cadherin 2	0	0	0	0	0	0	Flyp/lypnF+/+_151	0	0	1	1	0	8.72	8.51	8.49	8.24	8.52	8.33	8.33	8.10	8.87	8.63	8.50	8.28	8.99	8.52	8.32	8.31	8.75	8.35	8.20	8.19
1368699_at	Dscam	Down syndrome cell adhesion molecule	0	0	0	0	0	0	Flyp/lypnF+/+_151	0	0	1	1	0	5.72	5.57	5.44	5.28	5.70	5.68	5.45	5.43	6.19	5.90	5.90	5.60	5.91	5.84	5.72	5.69	5.99	5.76	5.78	5.43
1368840_at	Tmem176b	transmembrane protein 176B	0	0	0	0	0	0	Flyp/lypnF+/+_151	0	0	1	1	0	8.33	8.23	8.13	8.57	8.73	8.61	8.46	8.98	8.41	8.37	7.97	7.82	8.42	8.30	8.40	8.21	8.66	8.57	8.32	8.09
1369116_a_at	Calca	calcitonin-related polypeptide alpha	0	0	0	0	0	0	Flyp/lypnF+/+_151	0	0	1	1	0	5.81	5.72	5.73	5.28	5.69	5.78	5.73	5.67	6.05	5.92	5.86	5.72	5.95	5.77	5.67	5.72	5.93	5.86	5.84	5.75
1369117_at	Calca	calcitonin-related polypeptide alpha	0	0	0	0	0	0	Flyp/lypnF+/+_151	0	0	1	1	0	5.97	5.83	5.88	5.69	5.95	5.90	5.76	5.59	6.25	6.33	5.88	5.55	6.16	5.90	5.72	5.38	6.16	6.00	5.72	5.38
1369151_at	Dlk1	delta-like 1 homolog (Drosophila)	0	0	0	0	0	0	Flyp/lypnF+/+_151	0	0	1	1	0	3.93	3.76	3.57	3.62	4.16	4.03	3.80	3.84	4.88	3.96	3.94	3.55	5.00	3.88	3.93	3.62	4.81	4.12	4.05	3.88
1369373_at	Fgfr3	fibroblast growth factor receptor 3	0	0	0	0	0	0	Flyp/lypnF+/+_151	0	0	1	1	0	4.35	4.07	4.14	4.03	4.20	4.02	4.24	4.28	4.17	4.09	3.60	3.89	4.23	3.92	4.09	4.05	4.27	3.96	3.94	3.85
1369638_at	Eef2k	eukaryotic elongation factor-2 kinase	0	0	0	0	0	0	Flyp/lypnF+/+_151	0	0	1	1	0	7.26	7.06	6.88	6.72	7.25	6.89	6.97	6.70	7.54	7.21	6.75	6.59	7.52	7.12	6.77	6.72	7.48	6.92	6.74	6.62
1369973_at	Xdh	xanthine dehydrogenase	0	0	0	0	0	0	Flyp/lypnF+/+_151	0	0	1	1	0	5.97	5.67	5.78	6.53	5.71	5.44	5.51	5.68	6.13	5.79	5.53	5.38	6.49	5.89	5.75	5.79	6.03	5.69	5.46	5.55
1370019_at	Sult1a1	sulfotransferase family, cytosolic, 1A, phenol-preferring, member 1	0	0	0	0	0	0	Flyp/lypnF+/+_151	0	0	1	1	0	6.72	6.31	6.28	6.35	6.44	6.07	6.65	6.67	7.06	6.45	5.90	5.63	7.32	6.42	6.05	5.71	7.06	6.03	5.60	5.66
1370023_at	Gja4	gap junction protein, alpha 4	0	0	0	0	0	0	Flyp/lypnF+/+_151	0	0	1	1	0	7.86	7.81	7.53	7.40	7.96	7.74	7.56	7.57	7.65	7.37	7.22	7.08	7.82	7.47	7.22	7.05	7.66	7.57	7.18	7.05
1370201_at	Calb1	calbindin 1	0	0	0	0	0	0	Flyp/lypnF+/+_151	0	0	1	1	0	7.72	7.78	7.69	7.42	7.55	7.52	7.65	7.29	7.25	7.38	7.31	6.61	7.51	7.36	7.02	6.58	7.48	7.03	6.95	6.45
1370281_at	Fabp5	fatty acid binding protein 5, epidermal	0	0	0	0	0	0	Flyp/lypnF+/+_151	0	0	1	1	0	7.68	7.23	7.06	7.60	7.84	7.13	7.12	7.86	8.16	7.97	7.32	7.06	8.26	7.60	7.44	7.13	8.03	7.70	7.44	6.98
1370299_at	Aldob	aldolase B, fructose-bisphosphate	0	0	0	0	0	0	Flyp/lypnF+/+_151	0	0	1	1	0	5.30	5.35	5.05	5.07	5.49	4.99	5.50	5.10	5.81	4.83	5.00	4.95	5.52	5.41	4.68	5.05	5.85	5.17	4.84	5.15
1370334_at	Plekhb1	pleckstrin homology domain containing, family B (evectins) member 1	0	0	0	0	0	0	Flyp/lypnF+/+_151	0	0	1	1	0	7.78	7.88	7.89	7.62	7.80	7.92	7.82	7.50	7.35	7.26	6.75	6.58	7.35	7.28	7.13	6.76	7.45	7.11	6.85	6.37
1370442_at	Tmsbl1	thymosin beta-like protein 1	0	0	0	0	0	0	Flyp/lypnF+/+_151	0	0	1	1	0	11.54	11.43	11.33	11.26	11.52	11.63	11.09	11.32	11.52	11.33	11.17	10.99	11.53	11.18	11.10	11.10	11.59	11.37	11.12	11.02
1370746_at	Prkacb	protein kinase, cAMP dependent, catalytic, beta	0	0	0	0	0	0	Flyp/lypnF+/+_151	0	0	1	1	0	4.91	4.97	5.02	4.40	4.93	4.80	4.49	4.56	5.36	4.83	4.85	4.82	5.36	5.19	5.09	4.49	5.22	4.95	4.15	4.14
1370775_a_at	Calca	calcitonin-related polypeptide alpha	0	0	0	0	0	0	Flyp/lypnF+/+_151	0	0	1	1	0	4.01	4.01	3.70	3.61	4.31	3.82	3.86	3.58	5.80	5.25	4.30	4.26	5.42	4.13	4.06	3.99	4.96	4.57	4.25	3.87
1370834_at	Hs3st1	heparan sulfate (glucosamine) 3-O-sulfotransferase 1	0	0	0	0	0	0	Flyp/lypnF+/+_151	0	0	1	1	0	5.42	5.30	4.99	5.13	5.50	4.94	5.34	5.45	5.05	4.66	4.34	4.40	5.13	4.71	4.87	4.67	5.16	4.65	4.66	4.53
1370862_at	Apoe	apolipoprotein E	0	0	0	0	0	0	Flyp/lypnF+/+_151	0	0	1	1	0	8.67	8.21	8.09	8.69	8.64	8.07	8.11	9.11	9.17	8.73	8.12	7.92	9.05	8.27	8.39	8.60	8.98	8.15	7.85	7.57
1370973_at	Scn7a	sodium channel, voltage-gated, type VII, alpha	0	0	0	0	0	0	Flyp/lypnF+/+_151	0	0	1	1	0	10.68	10.57	10.40	10.29	10.55	10.39	10.34	10.11	10.59	10.32	10.16	9.92	10.49	10.22	10.12	9.93	10.58	10.14	10.00	9.90
1371108_a_at	Atp1a1	ATPase, Na+/K+ transporting, alpha 1 polypeptide	0	0	0	0	0	0	Flyp/lypnF+/+_151	0	0	1	1	0	8.62	8.29	8.13	8.10	8.49	8.12	8.19	8.15	8.62	8.22	7.94	8.00	7.54	8.22	8.04	8.00	8.57	8.11	7.86	7.42
1371131_a_at	Txnip	thioredoxin interacting protein	0	0	0	0	0	0	Flyp/lypnF+/+_151	0	0	1	1	0	10.43	10.33	10.05	10.10	10.41	10.21	10.24	10.24	10.28	9.90	9.72	9.61	10.62	10.17	9.83	9.70	10.30	10.01	9.82	9.65
1371347_at	Tmem50b	transmembrane protein 50B	0	0	0	0	0	0	Flyp/lypnF+/+_151	0	0	1	1	0	9.33	9.04	9.06	8.87	9.31	8.94	8.99	8.83	9.55	9.33	9.13	8.67	9.42	9.08	9.01	8.83	9.39	8.96	8.76	8.78
1371471_at	Gltscr2	glioma tumor suppressor candidate region gene 2	0	0	0	0	0	0	Flyp/lypnF+/+_151	0	0	1	1	0	10.06	10.05	10.02	9.88	10.02	10.01	9.98	9.89	10.12	10.06	9.81	9.48	10.21	10.12	9.85	9.65	10.13	9.93	9.77	9.50
1371491_at	Notch1	notch 1	0	0	0	0	0	0	Flyp/lypnF+/+_151	0	0	1	1	0	7.49	7.38	7.27	7.11	7.49	7.33	7.16	7.10	7.35	7.12	6.90	6.58	7.34	7.05	6.92	6.66	7.29	6.83	6.81	6.65
1371530_at	Krt8	keratin 8	0	0	0	0	0	0	Flyp/lypnF+/+_151	0	0	1	1	0	10.76	10.72	10.62	10.48	10.82	10.78	10.73	10.42	10.93	10.71	10.47	10.35	10.74	10.52	10.61	10.38	10.80	10.60	10.44	10.12
1371583_at	Rbm3	RNA binding motif (RNP1, RRM) protein 3	0	0	0	0	0	0	Flyp/lypnF+/+_151	0	0	1	1	0	10.49	10.24	10.29	10.15	10.48	10.29	10.21	10.44	10.98	10.30	10.08	10.02	11.18	10.21	10.06	10.11	10.86	10.13	10.02	9.89
1371697_at	LOC1009116:	patatin-like phospholipase domain-containing protein 2-like /// patatin-like phospholipase do	0	0	0	0	0	0	Flyp/lypnF+/+_151	0	0	1	1	0	7.17	7.02	7.00	7.06	7.12	6.95	6.99	6.84	7.81	7.60	7.31	7.19	7.71	7.38	7.29	7.23	7.74	7.35	7.34	7.19
1371703_at	Ahnak	AHNAK nucleoprotein	0	0	0	0	0	0	Flyp/lypnF+/+_151	0	0	1	1	0	8.41	8.16	8.00	8.20	8.38	8.10	8.25	8.38	8.41	8.05	7.87	7.82	8.67	8.11	8.02	7.96	8.47	8.14	8.04	7.92
1371953_at	Ccng2	cyclin G2	0	0	0	0	0	0	Flyp/lypnF+/+_151	0	0	1	1	0	7.93	7.78	7.68	7.81	7.83	7.73	7.68	7.89	8.16	8.02	7.81	7.59	8.15	7.82	7.88	7.78	8.14	7.77	7.53	7.61
1372162_at	Acss1	acyl-CoA synthetase short-chain family member 1	0	0	0	0	0	0	Flyp/lypnF+/+_151	0	0	1	1	0	6.11	5.97	5.47	5.93	6.00	5.64	5.80	6.35	5.97	5.67	5.42	5.29	6.26	5.75	5.41	5.32	5.95	5.74	5.70	5.37
1372254_at	Serping1	serpin peptidase inhibitor, clade G (C1 inhibitor), member 1	0	0	0	0	0	0	Flyp/lypnF+/+_151	0	0	1	1	0	9.41	9.29	9.23	10.18	9.28	9.26	9.14	9.02	9.56	9.47	9.15	9.04	9.75	9.30	9.28	9.17	9.45	9.16	9.07	8.93
1372520_at	Mcl1	myeloid cell leukemia sequence 1	0	0	0	0	0	0	Flyp/lypnF+/+_151	0	0	1	1	0	9.35	9.38	9.16	9.13	9.40	9.37	9.43	9.43	9.53	9.53	9.32	9.02	9.43	9.18	9.26	9.06	9.36	9.33	9.06	8.79
1373036_at	LOC1003606:	IQ motif containing GTPase activating protein 2	0	0	0	0	0	0	Flyp/lypnF+/+_151	0	0	1	1	0	4.82	4.88	4.48	4.76	4.86	4.63	4.75	5.06	5.53	4.99	4.83	4.85	5.83	5.22	5.04	4.83	5.30	5.03	4.79	4.77
1373040_at	Eif3f	eukaryotic translation initiation factor 3, subunit F	0	0	0	0	0	0	Flyp/lypnF+/+_151	0	0	1	1	0	10.34	10.30	10.27	10.12	10.30	10.36	10.19	10.14	10.43	10.39	10.12	9.80	10.47	10.33	10.19	9.92	10.38	10.24	10.03	9.87
1373128_at	Rcn3	reticulocalbin 3, EF-hand calcium binding domain	0	0	0	0	0	0	Flyp/lypnF+/+_151	0	0	1	1	0	6.83	6.80	6.60	6.47	6.84	6.64	6.67	6.54	7.20	7.05	6.90	6.62	7.21	7.11	6.94	6.73	7.40	7.07	7.02	6.65
1373345_at	Amigo2	adhesion molecule with Ig like domain 2	0	0	0	0	0	0	Flyp/lypnF+/+_151	0	0	1	1	0	9.27	9.49	9.39	9.29	9.15	9.42	9.35	9.06	8.84	8.84	8.62	8.13	8.93	8.81	8.71	8.32	8.83	8.66	8.59	8.12
1373403_at	LOC1009101(	uncharacterized LOC100910163 /// similar to Protein C8orf4 (Thyroid cancer protein 1) (TC-1	0	0	0	0	0	0	Flyp/lypnF+/+_151	0	0	1	1	0	8.10	7.99	7.70	7.68	8.28	7.82	7.89	7.81	8.03	7.64	7.36	7.33	8.09	7.71	7.50	7.29	7.98	7.88	7.61	7.37
1373436_at	RGD1306739	similar to RIKEN cDNA 1700040L02	0	0	0	0	0	0	Flyp/lypnF+/+_151	0	0	1	1	0	6.50	6.63	6.75	6.52	6.54	6.51	6.66	6.77	6.74	6.57	6.20	6.27	6.69	6.50	6.56	6.36	6.67	6.35	6.33	6.11
1373804_at	Foxp1	forkhead box P1	0	0	0	0	0	0	Flyp/lypnF+/+_151	0	0	1	1	0	8.04	7.93	7.66	7.83	8.08	7.79	7.73	7.81	8.12	7.80	7.74	7.30	7.58	7.62	7.66	7.44	8.11	7.71	7.36	7.06
1374165_at	Rasa4	RAS p21 protein activator 4	0	0	0	0	0	0	Flyp/lypnF+/+_151	0	0	1	1	0	7.79	7.67	7.58	7.61	7.68	7.61	7.53	7.40	7.74	7.50	7.30	7.10	7.76	7.39	7.30	7.14	7.68	7.39	7.22	7.10
1374204_at	Wsb1	WD repeat and SOCS box-containing 1	0	0	0	0	0	0	Flyp/lypnF+/+_151	0	0	1	1	0	8.49	8.40	8.18	8.10	8.37	7.82	8.16	8.32	8.53	8.28	8.15	7.82	8.55	8.15	8.02	7.91	8.38	8.25	8.08	7.75
1374320_at	F5	coagulation factor V (proaccelerin, labile factor)	0	0	0	0	0	0	Flyp/lypnF+/+_151	0	0	1	1	0	5.94	6.10	6.22	5.80	6.05	5.94	6.04	5.84	6.12	5.94	5.91	5.56	6.38	6.08	6.10	5.71	6.19	5.96	5.82	5.53
1374359_at	Ccne2	cyclin E2	0	0	0	0	0	0	Flyp/lypnF+/+_151	0	0	1	1	0	5.98	5.99	5.90	6.01	6.05	6.05	5.87	6.04	5.97	5.85	5.91	5.92	6.10	5.81	5.97	6.02	6.08	5.72	5.82	5.89
1374784_at	Prtfdc1	phosphoribosyl transferase domain containing 1	0	0	0	0	0	0	Flyp/lypnF+/+_151	0	0	1	1	0	7.72	7.81	7.80	7.49	7.76	7.74	7.73	7.50	7.66	7.59	7.38	7.16	7.71	7.53	7.33	7.12	7.65	7.31	7.24	7.01
1374794_at	Kif15	kinesin family member 15	0	0	0	0	0	0	Flyp/lypnF+/+_151	0	0	1	1	0	5.92	6.32	6.06	5.96	6.15	6.16	5.85	6.29	6.90	6.76	6.49	6.07	6.73	6.37	6.51	6.08	6.65	6.30	6.13	5.99
1374884_at	Ppm1d	protein phosphatase, Mg2+/Mn2+ dependent, 1D	0	0	0	0	0	0	Flyp/lypnF+/+_151	0	0	1	1	0	8.23	8.17	8.10	7.96	8.34	8.22	8.12	7.95	8.31	8.17	8.09	7.74	8.24	8.09	8.14	7.84	8.26	8.07	7.97	7.76
1375215_x_at	Pgpep1	pyroglutamyl-peptidase I	0	0	0	0	0	0	Flyp/lypnF+/+_151	0	0	1	1	0	8.17	7.97	8.02	7.79	8.11	8.09	7.70	7.87	8.34	8.05	7.86	7.77	8.13	8.11	7.93	7.95	8.31	8.08	7.61	7.73
1375377_at	Igsf3	immunoglobulin superfamily, member 3	0	0	0	0	0	0	Flyp/lypnF+/+_151	0	0	1	1	0	6.40	6.26	6.16	5.92	6.40	6.10	6.07	6.01	7.38	7.03	6.80	6.64	7.27	6.96	6.72	6.66	7.12	6.73	6.61	6.69
1375378_at	---	---	0	0	0	0	0	0	Flyp/lypnF+/+_151	0	0	1	1	0	7.81	7.47	7.36	7.45	7.63	7.56	7.33	7.46	7.88	7.44	7.20	7.14	7.41	7.56	7.32	7.26	7.85	7.56	6.97	6.94
1376066_at	Rnd3	Rho family GTPase 3	0	0	0	0	0	0	Flyp/lypnF+/+_151	0	0	1	1	0	7.07	7.12	7.02	6.92	7.25	7.10	7.23	7.16	6.67	6.55	6.36	5.95	6.68	6.38	6.57	6.37	6.81	6.29	6.23	6.27
1376080_at	Rcor1	REST corepressor 1	0	0	0	0	0	0	Flyp/lypnF+/+_151	0	0	1	1	0	5.40	5.10	4.82	4.78	5.14	5.24	4.83	4.74	5.49	5.03	5.17	4.67	4.32	5.10	5.19	4.59	5.30	5.23	4.76	4.47
1376117_at	Slc44a4	solute carrier family 44, member 4	0	0	0	0	0	0	Flyp/lypnF+/+_151	0	0	1	1	0	5.57	5.57	5.54	5.28	5.68	5.39	5.36	5.19	5.49	5.06	4.99	5.00	5.67	4.93	4.82	4.70	5.38	4.94	4.88	4.57
1376788_at	Dapk1	death associated protein kinase 1	0	0	0	0	0	0	Flyp/lypnF+/+_151	0	0	1	1	0	7.62	7.48	7.27	7.38	7.52	7.20	7.28	7.26	7.75	7.53	7.36	7.18	8.06	7.40	7.18	7.16	7.74	7.26	7.30	6.94
1377307_at	Fam89a	family with sequence similarity 89, member A	0	0	0	0	0	0	Flyp/lypnF+/+_151	0	0	1	1	0	4.69	4.64	4.38	4.63	4.68	4.69	4.60	4.18	4.79	4.59	4.30	4.26	4.65	4.23	4.32	4.41	4.81	4.43	4.20	4.07
1377663_at	Rnd3	Rho family GTPase 3	0	0	0	0	0	0	Flyp/lypnF+/+_151	0	0	1	1	0	6.79	6.84	6.74	6.46	7.07	6.73	6.80	6.77	6.38	6.21	6.15	5.54	5.96	6.10	6.36	5.91	6.62	6.04	5.86	5.92
1377867_at	RGD1562284	similar to Glutaminyl-peptide cyclotransferase precursor (QC)	0	0	0	0	0	0	Flyp/lypnF+/+_151	0	0	1	1	0	6.04	6.47	6.52	6.14	6.23	6.10	6.15	6.10	4.87	4.67	4.66	4.32	4.95	4.53	4.77	4.22	4.97	4.63	4.45	4.29
1378163_at	Grem2	gremlin 2	0	0	0	0	0	0	Flyp/lypnF+/+_151	0	0	1	1	0	6.13	6.07	6.07	5.69	5.88	6.28	6.03	5.94	7.28	7.06	6.94	6.41	7.03	7.13	6.98	6.18	7.05	6.99	6.83	6.68
1378339_at	Fam124a	family with sequence similarity 124A	0	0	0	0	0	0	Flyp/lypnF+/+_151	0	0	1	1	0	4.29	4.33	4.08	4.01	4.28	4.21	4.33	3.96	4.72	4.48	4.32	3.86	5.05	4.35	4.23	4.16	4.93	4.29	4.23	4.17
1378374_at	RGD1560137	similar to expressed sequence AU021034	0	0	0	0	0	0	Flyp/lypnF+/+_151	0	0	1	1	0	3.06	3.07	3.01	3.22	3.12	3.06	2.96	3.14	3.99	3.41	3.19	3.13	4.18	3.23	3.18	2.99	3.93	3.18	3.21	3.21
1378914_a_at	LOC308990	hypothetical protein LOC308990	0	0	0	0	0	0	Flyp/lypnF+/+_151	0	0	1	1	0	5.91	5.70	5.56	5.77	5.88	5.81	5.55	6.24	5.63	5.50	5.21	5.01	6.09	5.34	5.37	5.14	5.72	5.29	5.24	4.91
1378915_x_at	LOC308990	hypothetical protein LOC308990	0	0	0	0	0	0	Flyp/lypnF+/+_151	0	0	1	1	0	5.07	5.31	5.15	5.39	5.48	5.32	5.19	5.65	5.26	5.00	4.73	4.69	5.32	5.02	4.93	4.93	5.18	4.91	4.90	4.55
1379194_at	Klhl42	kelch-like family, member 42	0	0	0	0	0	0	Flyp/lypnF+/+_151	0	0	1	1	0	5.72	5.67	5.54	5.53	5.73	5.40	5.55	5.27	5.97	5.66	5.57	5.14	4.42	5.41	5.57	5.32	5.79	5.54	4.80	4.47
1379615_at	Klhl42	kelch-like family, member 42	0	0	0	0	0	0	Flyp/lypnF+/+_151	0	0	1	1	0	7.13	7.05	6.87	6.86	7.07	7.00	6.87	6.79	7.27	7.00	7.01	6.69	6.35	6.91	7.00	6.70	7.04	6.99	6.46	6.25
1379651_at	Foxp1	forkhead box P1	0	0	0	0	0	0	Flyp/lypnF+/+_151	0	0	1	1	0	7.19	7.26	7.14	7.30	7.29	7.04	7.24	7.40	7.32	7.20	7.03	6.84	7.19	7.07	7.10	6.91	7.41	6.99	6.91	6.58
1379868_at	Pbxip1	pre-B-cell leukemia homeobox interacting protein 1	0	0	0	0	0	0	Flyp/lypnF+/+_151	0	0	1	1	0	6.98	6.69	6.49	6.52	6.87	6.79	6.54	6.51	6.91	6.61	6.38	6.30	7.06	6.49	6.39	6.25	6.93	6.42	6.40	6.14
1379911_at	Dapk1	death associated protein kinase 1	0	0	0	0	0	0	Flyp/lypnF+/+_151	0	0	1	1	0	7.02	6.85	6.72	6.61	6.92	6.75	6.76	6.56	7.10	6.80	6.74	6.52	6.85	6.86	6.61	6.48	7.05	6.76	6.65	6.38
1380403_at	Rwdd2a	RWD domain containing 2A	0	0	0	0	0	0	Flyp/lypnF+/+_151	0	0	1	1	0	3.71	3.55	3.69	3.39	3.53	3.62	3.49	3.24	3.66	3.49	3.20	3.06	4.06	3.46	3.39	3.14	3.62	3.33	3.27	2.84
1380799_at	Grem2	gremlin 2	0	0	0	0	0	0	Flyp/lypnF+/+_151	0	0	1	1	0	3.03	3.16	3.24	2.82	2.96	3.11	2.97	2.91	4.23	3.81	3.76	3.45	4.02	4.00	3.80	3.29	4.38	3.97	3.72	3.52
1381677_at	Elavl2	ELAV (embryonic lethal, abnormal vision, Drosophila)-like 2 (Hu antigen B)	0	0	0	0	0	0	Flyp/lypnF+/+_151	0	0	1	1	0	3.97	3.98	3.99	3.94	3.90	3.89	3.88	3.83	4.01	3.99	4.01	4.01	3.94	3.95	3.81	3.82	3.99	3.95	3.87	3.95
1381850_at	Ppp1r12a	protein phosphatase 1, regulatory subunit 12A	0	0	0	0	0	0	Flyp/lypnF+/+_151	0	0	1	1	0	7.62	7.43	7.28	7.56	7.70	7.21	7.15	7.75	7.63	7.32	7.30	6.94	6.54	7.13	7.27	7.24	7.56	7.28	7.11	6.77
1382186_a_at	Gpatch4	G patch domain containing 4	0	0	0	0	0	0	Flyp/lypnF+/+_151	0	0	1	1	0	5.12	5.06	5.08	4.99	5.08	4.89	5.11	5.12	5.45	5.13	5.06	4.82	5.53	5.21	4.93	4.77	5.59	4.79	4.98	4.59
1382187_at	Gpatch4	G patch domain containing 4	0	0	0	0	0	0	Flyp/lypnF+/+_151	0	0	1	1	0	4.97	5.30	5.36	5.33	5.22	5.20	5.33	5.30	5.51	5.33	5.26	5.22	5.22	5.31	5.20	5.12	5.37	5.22	5.15	5.24
1382188_at	Gpatch4	G patch domain containing 4	0	0	0	0	0	0	Flyp/lypnF+/+_151	0	0	1	1	0	5.47	5.46	5.29	5.14	5.38	5.35	5.39	5.37	5.67	5.46	5.13	5.24	5.46	5.49	5.27	5.22	5.71	5.35	5.27	5.36
1382307_at	Ppp1r12a	protein phosphatase 1, regulatory subunit 12A	0	0	0	0	0	0	Flyp/lypnF+/+_151	0	0	1	1	0	7.91	7.82	7.82	7.86	7.94	7.75	7.80	7.93	7.82	7.75	7.59	7.52	7.29	7.54	7.66	7.62	7.84	7.71	7.63	7.34
1382489_at	Phip	pleckstrin homology domain interacting protein	0	0	0	0	0	0	Flyp/lypnF+/+_151	0	0	1	1	0	8.84	8.67	8.52	8.39	8.81	8.75	8.37	8.38	8.96	8.60	8.37	8.04	8.88	8.42	8.36	8.14	8.83	8.49	8.25	8.19
1382927_at	LOC308990	hypothetical protein LOC308990	0	0	0	0	0	0	Flyp/lypnF+/+_151	0	0	1	1	0	5.31	5.30	5.31	5.33	5.39	5.40	5.43	5.34	5.32	5.29	5.35	5.45	5.21	5.36	5.30	5.32	5.42	5.39	5.26	5.35
1383287_at	Fubp1	far upstream element (FUSE) binding protein 1	0	0	0	0	0	0	Flyp/lypnF+/+_151	0	0	1	1	0	8.55	8.28	8.20	8.19	8.44	8.12	8.13	8.30	8.53	8.14	8.12	8.01	8.53	8.19	8.14	7.91	8.50	8.13	8.10	7.98
1383422_at	Bend5	BEN domain containing 5	0	0	0	0	0	0	Flyp/lypnF+/+_151	0	0	1	1	0	5.55	5.52	5.43	5.41	5.58	5.40	5.32	5.67	5.87	5.75	5.47	5.25	5.83	5.40	5.51	5.40	5.79	5.39	5.24	5.24
1383736_at	Elavl2	ELAV (embryonic lethal, abnormal vision, Drosophila)-like 2 (Hu antigen B)	0	0	0	0	0	0	Flyp/lypnF+/+_151	0	0	1	1	0	4.91	5.39	5.35	5.18	5.48	5.15	5.05	5.18	5.64	5.95	5.12	5.05	5.64	5.41	5.02	5.00	5.48	5.20	5.19	5.02
1383768_at	Elavl2	ELAV (embryonic lethal, abnormal vision, Drosophila)-like 2 (Hu antigen B)	0	0	0	0	0	0	Flyp/lypnF+/+_151	0	0	1	1	0	5.30	5.21	5.01	4.97	5.09	4.76	4.85	4.80	5.63	5.82	5.00	4.75	5.54	5.12	4.64	4.75	5.67	4.80	4.80	4.83
1384056_at	---	---	0	0	0	0	0	0	Flyp/lypnF+/+_151	0	0	1	1	0	7.76	7.87	7.87	7.75	7.75	7.78	7.78	7.79	7.71	7.45	7.21	7.10	7.61	7.32	7.51	7.39	7.72	7.10	7.25	6.81
1384283_at	Asb9	ankyrin repeat and SOCS box-containing 9	0	0	0	0	0	0	Flyp/lypnF+/+_151	0	0	1	1	0	8.28	7.38	7.22	7.56	8.23	7.02	7.27	7.28	8.36	7.86	7.72	7.87	9.23	7.66	7.50	7.66	8.58	7.46	7.63	7.34
1384799_at	RGD1561931	similar to KIAA2022 protein	0	0	0	0	0	0	Flyp/lypnF+/+_151	0	0	1	1	0	2.78	2.92	2.69	2.64	3.06	3.05	2.55	2.76	3.13	2.69	2.78	2.45	3.03	3.09	2.66	2.29	3.06	2.86	2.30	2.56
1384943_at	RGD1563888	similar to DNA segment, Chr 16, ERATO Doi 472, expressed	0	0	0	0	0	0	Flyp/lypnF+/+_151	0	0	1	1	0	7.06	7.12	6.95	6.91	7.25	6.94	7.04	7.17	6.85	6.75	6.60	6.41	6.44	6.61	6.58	6.47	6.84	6.52	6.51	6.40
1386062_at	---	---	0	0	0	0	0	0	Flyp/lypnF+/+_151	0	0	1	1	0	2.37	2.45	2.33	2.35	2.45	2.52	2.38	2.25	2.31	2.28	2.32	2.20	2.32	2.33	2.39	2.17	2.30	2.13	2.41	2.20
1386899_at	Ctsh	cathepsin H	0	0	0	0	0	0	Flyp/lypnF+/+_151	0	0	1	1	0	9.02	8.79	8.73	8.58	8.95	8.78	8.61	8.77	9.86	9.48	9.19	8.75	9.80	9.59	9.26	8.79	9.86	9.47	9.16	8.80
1386922_at	Ca2	carbonic anhydrase 2	0	0	0	0	0	0	Flyp/lypnF+/+_151	0	0	1	1	0	5.27	5.11	5.06	4.96	5.28	5.21	4.95	5.01	5.68	5.17	4.96	5.06	5.55	5.40	4.99	4.75	5.51	5.06	5.03	4.73
1386985_at	Gstm1	glutathione S-transferase mu 1	0	0	0	0	0	0	Flyp/lypnF+/+_151	0	0	1	1	0	6.79	6.47	6.39	6.29	6.82	6.62	6.46	6.33	6.17	5.84	5.78	5.54	6.22	5.83	5.62	5.56	6.11	5.86	5.65	5.56
1386994_at	Btg2	BTG family, member 2	0	0	0	0	0	0	Flyp/lypnF+/+_151	0	0	1	1	0	10.88	10.90	10.59	10.37	11.06	10.85	11.07	10.48	10.88	10.76	10.76	10.17	10.78	10.37	10.55	10.40	10.62	10.55	10.45	9.96
1386995_at	Btg2	BTG family, member 2	0	0	0	0	0	0	Flyp/lypnF+/+_151	0	0	1	1	0	11.66	11.68	11.53	11.21	11.80	11.69	11.90	11.28	11.64	11.57	11.69	11.12	11.33	11.24	11.48	11.22	11.49	11.41	11.11	10.63
1387121_a_at	Ndrg2	NDRG family member 2	0	0	0	0	0	0	Flyp/lypnF+/+_151	0	0	1	1	0	6.67	6.52	6.38	6.38	6.63	6.38	6.50	6.54	6.64	6.53	6.18	5.97	6.93	6.41	6.31	6.08	6.76	6.25	6.09	5.89
1387219_at	Adm	adrenomedullin	0	0	0	0	0	0	Flyp/lypnF+/+_151	0	0	1	1	0	4.65	4.43	4.44	4.36	4.68	4.24	4.19	4.04	4.25	4.19	3.79	3.73	4.25	3.67	3.78	3.83	4.27	3.96	3.96	3.62
1387259_at	Cdh2	cadherin 2	0	0	0	0	0	0	Flyp/lypnF+/+_151	0	0	1	1	0	8.35	8.29	8.17	8.10	8.28	8.07	8.12	7.93	8.40	8.34	8.28	7.94	8.17	8.09	7.94	7.91	8.33	7.99	7.94	7.80
1387260_at	Klf4	Kruppel-like factor 4 (gut)	0	0	0	0	0	0	Flyp/lypnF+/+_151	0	0	1	1	0	8.77	9.02	8.88	8.95	9.09	8.72	9.64	9.36	8.91	8.76	8.58	8.35	8.85	8.68	8.60	8.44	8.81	8.85	8.59	8.34
1387291_at	Itih3	inter-alpha trypsin inhibitor, heavy chain 3	0	0	0	0	0	0	Flyp/lypnF+/+_151	0	0	1	1	0	5.36	5.00	5.06	4.97	5.22	5.17	4.98	4.95	6.19	5.77	5.32	5.17	6.25	5.56	5.33	5.19	6.40	5.29	5.25	5.07

Affymetrix Probeset ID	Gene Symbol	Gene Name	unique BB on P7 n=822	Figure 1B P7 Venn n=1526	DRlyp/lyp STEM P7 n=712	DR+/+ STEM P7 n=936	Flyp/lyp STEM P7 n=520	F+/+ STEM P7 n=465	Figure 1B P0 Venn n=1071	DRlyp/lyp STEM P0 n=442	DR+/+ STEM P0 n=437	Flyp/lyp STEM P0 n=643	F+/+ STEM P0 n=632	Fig1D n=25	Mean log2 intensity DRlyp/lyp 20	Mean log2 intensity DRlyp/lyp 30	Mean log2 intensity DRlyp/lyp 40	Mean log2 intensity DRlyp/lyp 50	Mean log2 intensity DR+/+ 20	Mean log2 intensity DR+/+ 30	Mean log2 intensity DR+/+ 40	Mean log2 intensity DR+/+ 50	Mean log2 intensity Flyp/lyp 20	Mean log2 intensity Flyp/lyp 30	Mean log2 intensity Flyp/lyp 40	Mean log2 intensity Flyp/lyp 50	Mean log2 intensity Flyp20	Mean log2 intensity Flyp30	Mean log2 intensity Flyp40	Mean log2 intensity Flyp50	Mean log2 intensity F+/+ 20	Mean log2 intensity F+/+ 30	Mean log2 intensity F+/+ 40	Mean log2 intensity F+/+ 50
1387408_at	Siah2	siah E3 ubiquitin protein ligase 2	0	0	0	0	0	0	Flyp/lypnF+/+_151	0	0	1	1	0	6.05	6.34	6.10	5.86	6.32	6.24	6.24	6.01	6.47	6.31	6.27	5.83	6.64	6.01	6.21	6.05	6.38	6.22	6.06	5.69
1387477_at	Kcnk12	potassium channel, subfamily K, member 12	0	0	0	0	0	0	Flyp/lypnF+/+_151	0	0	1	1	0	6.47	6.46	6.24	6.05	6.34	6.47	6.34	5.98	6.55	6.29	6.30	5.76	6.36	6.36	6.12	5.85	6.46	6.23	6.06	5.81
1387658_at	Eef2k	eukaryotic elongation factor-2 kinase	0	0	0	0	0	0	Flyp/lypnF+/+_151	0	0	1	1	0	5.42	5.40	5.11	5.19	5.36	5.06	5.41	5.12	5.57	5.27	5.10	4.81	5.70	5.41	4.95	5.09	5.78	5.20	4.84	5.07
1387796_at	Alox15	arachidonate 15-lipoxygenase	0	0	0	0	0	0	Flyp/lypnF+/+_151	0	0	1	1	0	4.46	4.36	4.08	4.04	4.50	4.43	4.13	4.09	4.72	4.20	4.04	4.07	4.22	4.32	4.17	3.84	4.53	4.29	3.99	4.00
1387883_a_at	Tmsb4x	thymosin beta 4, X-linked	0	0	0	0	0	0	Flyp/lypnF+/+_151	0	0	1	1	0	11.63	11.45	11.39	11.76	11.71	11.62	11.41	12.15	11.63	11.54	11.34	11.03	11.59	11.42	11.36	11.50	11.64	11.46	11.05	10.99
1387907_at	Itpr1	inositol 1,4,5-trisphosphate receptor, type 1	0	0	0	0	0	0	Flyp/lypnF+/+_151	0	0	1	1	0	9.59	9.42	9.33	9.15	9.60	9.50	9.20	9.13	9.34	9.16	9.02	8.73	9.28	9.14	9.01	8.71	9.32	9.12	8.97	8.78
1388074_at	Krt20	keratin 20	0	0	0	0	0	0	Flyp/lypnF+/+_151	0	0	1	1	0	4.28	4.00	3.70	3.93	4.21	4.07	4.04	4.03	4.60	4.39	4.08	4.07	4.63	4.61	4.33	4.15	4.71	4.25	4.08	3.98
1388102_at	Ptgr1	prostaglandin reductase 1	0	0	0	0	0	0	Flyp/lypnF+/+_151	0	0	1	1	0	6.71	6.58	6.62	6.36	6.67	6.61	6.54	6.19	6.60	6.43	6.10	5.77	6.48	6.27	6.14	5.84	6.76	6.28	6.17	5.78
1388155_at	Krt18	keratin 18	0	0	0	0	0	0	Flyp/lypnF+/+_151	0	0	1	1	0	10.76	10.60	10.58	10.57	10.83	10.69	10.65	10.37	10.70	10.45	10.20	10.06	10.53	10.26	10.40	10.21	10.65	10.32	10.11	10.02
1388158_at	Ddx39b	DEAD (Asp-Glu-Ala-Asp) box polypeptide 39B	0	0	0	0	0	0	Flyp/lypnF+/+_151	0	0	1	1	0	8.01	7.91	7.77	7.85	8.20	7.86	7.78	7.76	7.97	7.77	7.69	7.44	8.08	7.61	7.53	7.60	8.06	7.64	7.74	7.54
1388363_at	Raly	RNA binding protein, autoantigenic (hnRNP-associated with lethal yellow homolog (mouse))	0	0	0	0	0	0	Flyp/lypnF+/+_151	0	0	1	1	0	7.58	7.33	7.04	7.26	7.42	7.20	7.16	7.24	7.77	7.48	7.14	6.97	7.85	7.21	7.13	7.15	7.69	7.23	7.00	6.82
1388493_at	Vopp1	vesicular, overexpressed in cancer, prosurvival protein 1	0	0	0	0	0	0	Flyp/lypnF+/+_151	0	0	1	1	0	7.81	7.74	7.64	7.63	7.77	7.78	7.58	7.81	7.78	7.88	7.58	7.25	7.75	7.74	7.59	7.33	7.67	7.66	7.39	7.07
1388506_at	Dsp	desmoplakin	0	0	0	0	0	0	Flyp/lypnF+/+_151	0	0	1	1	0	9.56	9.60	9.59	9.51	9.60	9.63	9.58	9.45	8.56	8.38	8.07	7.79	8.64	8.37	8.39	8.00	8.49	8.29	8.28	7.70
1389449_at	Fitm1	fat storage-inducing transmembrane protein 1	0	0	0	0	0	0	Flyp/lypnF+/+_151	0	0	1	1	0	3.35	3.42	3.34	3.18	3.49	3.42	3.40	3.33	4.71	4.24	3.87	3.99	5.10	4.17	3.98	3.82	4.44	4.04	4.14	3.90
1389911_at	Metrnl	meteorin, glial cell differentiation regulator-like	0	0	0	0	0	0	Flyp/lypnF+/+_151	0	0	1	1	0	5.01	4.99	4.88	5.04	4.99	4.95	5.02	5.03	5.69	5.32	5.01	5.13	5.69	5.22	5.05	4.95	5.75	5.22	5.09	5.21
1389999_at	---	---	0	0	0	0	0	0	Flyp/lypnF+/+_151	0	0	1	1	0	6.59	6.33	5.93	5.91	6.50	6.15	5.94	5.90	6.64	6.27	5.87	5.54	6.44	6.32	5.48	5.49	6.63	6.18	6.05	5.50
1390426_at	Notch1	notch 1	0	0	0	0	0	0	Flyp/lypnF+/+_151	0	0	1	1	0	6.79	6.60	6.44	6.39	6.67	6.61	6.42	6.33	6.59	6.52	6.11	6.00	6.95	6.35	6.14	5.99	6.59	6.27	6.11	5.89
1390443_at	RGD1563888	similar to DNA segment, Chr 16, ERATO Doi 472, expressed	0	0	0	0	0	0	Flyp/lypnF+/+_151	0	0	1	1	0	8.72	8.54	8.28	8.21	8.70	8.64	8.24	8.18	8.40	8.22	7.96	7.75	8.04	7.89	8.00	7.76	8.27	8.03	7.78	7.55
1390786_at	Arhgef2	rho/rac guanine nucleotide exchange factor (GEF) 2	0	0	0	0	0	0	Flyp/lypnF+/+_151	0	0	1	1	0	7.82	7.59	7.47	7.35	7.86	7.65	7.50	7.46	7.74	7.38	7.32	7.12	7.87	7.48	7.35	7.23	7.71	7.42	7.41	7.17
1390925_a_at	Igsf3	immunoglobulin superfamily, member 3	0	0	0	0	0	0	Flyp/lypnF+/+_151	0	0	1	1	0	5.31	5.20	5.09	4.92	5.26	5.36	5.19	5.00	5.97	5.72	5.54	5.46	5.86	5.63	5.77	5.56	6.06	5.43	5.44	5.58
1390952_at	Rcor1	REST corepressor 1	0	0	0	0	0	0	Flyp/lypnF+/+_151	0	0	1	1	0	7.38	7.24	7.20	7.12	7.41	7.18	7.13	7.21	7.36	7.14	7.01	6.87	7.56	7.07	7.03	6.91	7.31	7.16	7.14	7.02
1391218_at	Fam76b	family with sequence similarity 76, member B	0	0	0	0	0	0	Flyp/lypnF+/+_151	0	0	1	1	0	7.90	7.78	7.70	7.46	7.94	7.80	7.51	7.62	7.90	7.68	7.57	7.35	7.98	7.45	7.56	7.50	7.87	7.58	7.53	7.31
1391297_at	Rcor1	REST corepressor 1	0	0	0	0	0	0	Flyp/lypnF+/+_151	0	0	1	1	0	7.04	6.87	6.71	6.73	7.12	6.88	6.57	6.84	7.12	6.64	6.69	6.46	6.23	6.67	6.59	6.38	6.89	6.78	6.32	6.11
1391356_at	Iws1	IWS1 homolog (S. cerevisiae)	0	0	0	0	0	0	Flyp/lypnF+/+_151	0	0	1	1	0	8.03	7.75	7.81	7.74	8.08	7.77	7.69	7.96	8.44	8.07	8.00	7.89	8.25	8.09	7.90	7.83	8.37	7.97	7.78	7.74
1391524_at	Phip	pleckstrin homology domain interacting protein	0	0	0	0	0	0	Flyp/lypnF+/+_151	0	0	1	1	0	8.62	8.49	8.31	8.26	8.56	8.35	8.22	8.17	8.33	7.92	7.97	7.53	7.59	7.72	7.96	7.60	8.20	7.93	7.42	7.39
1391681_at	Ccne2	cyclin E2	0	0	0	0	0	0	Flyp/lypnF+/+_151	0	0	1	1	0	5.24	5.11	4.91	4.61	5.27	5.18	4.64	4.38	5.51	5.06	5.12	4.34	4.76	4.91	5.02	4.40	5.31	5.08	4.61	4.21
1392648_at	Mrc1	mannose receptor, C type 1	0	0	0	0	0	0	Flyp/lypnF+/+_151	0	0	1	1	0	4.82	4.28	3.73	5.04	4.87	4.09	3.98	5.10	4.54	3.63	3.76	3.78	4.91	4.04	4.16	4.50	4.54	3.90	3.94	3.41
1393128_at	Gpatch4	G patch domain containing 4	0	0	0	0	0	0	Flyp/lypnF+/+_151	0	0	1	1	0	5.93	5.87	5.75	5.76	5.75	5.76	5.67	5.97	6.35	6.10	5.92	5.54	6.23	6.07	5.76	5.71	6.44	5.77	5.79	5.75
1393435_at	---	---	0	0	0	0	0	0	Flyp/lypnF+/+_151	0	0	1	1	0	6.01	6.12	5.92	6.01	6.04	5.92	6.02	5.64	6.72	6.55	6.28	6.11	6.81	6.18	6.14	6.11	6.93	6.12	6.12	5.80
1393757_at	N4bp2l1	NEDD4 binding protein 2-like 1	0	0	0	0	0	0	Flyp/lypnF+/+_151	0	0	1	1	0	6.21	6.44	6.04	6.16	6.20	6.19	6.18	6.10	6.54	6.31	6.11	5.77	6.27	6.42	6.13	5.90	6.31	6.23	5.97	5.68
1393944_at	Zfp521	zinc finger protein 521	0	0	0	0	0	0	Flyp/lypnF+/+_151	0	0	1	1	0	5.46	5.41	5.21	5.03	5.54	5.31	5.19	5.39	5.84	5.64	5.63	5.21	6.22	5.66	5.40	5.15	5.74	5.50	5.45	5.08
1394077_at	Rnd3	Rho family GTPase 3	0	0	0	0	0	0	Flyp/lypnF+/+_151	0	0	1	1	0	4.79	4.86	4.77	4.59	4.93	4.64	5.01	4.98	4.61	4.48	4.39	4.25	4.45	4.09	4.49	4.13	4.71	4.16	4.24	4.17
1394253_at	---	---	0	0	0	0	0	0	Flyp/lypnF+/+_151	0	0	1	1	0	3.46	3.32	3.52	3.64	3.61	3.67	3.52	3.54	3.52	3.51	3.56	3.57	3.42	3.54	3.54	3.64	3.53	3.71	3.49	3.68
1394691_at	Foxp1	forkhead box P1	0	0	0	0	0	0	Flyp/lypnF+/+_151	0	0	1	1	0	5.37	5.23	5.21	5.06	5.39	5.31	5.16	5.16	5.65	5.39	5.23	5.08	5.20	5.35	5.15	5.21	5.45	5.34	5.27	4.94
1395590_at	---	---	0	0	0	0	0	0	Flyp/lypnF+/+_151	0	0	1	1	0	7.09	7.01	6.94	6.98	7.08	7.03	6.91	6.86	7.28	6.96	6.87	6.77	7.05	6.94	6.66	6.61	7.50	7.12	6.97	6.75
1398833_at	Mbtps1	membrane-bound transcription factor peptidase, site 1	0	0	0	0	0	0	Flyp/lypnF+/+_151	0	0	1	1	0	8.90	8.76	8.61	8.67	8.88	8.68	8.64	8.59	9.01	8.69	8.52	8.49	9.01	8.70	8.63	8.55	8.89	8.70	8.69	8.36
1399167_a_at	Gab1	GRB2-associated binding protein 1	0	0	0	0	0	0	Flyp/lypnF+/+_151	0	0	1	1	0	9.82	9.63	9.56	9.48	9.80	9.57	9.54	9.37	9.88	9.56	9.49	9.24	9.96	9.54	9.46	9.39	9.76	9.33	9.21	9.35
1367644_at	Adcy6	adenylate cyclase 6	0	0	0	0	0	0	uniqDR+/+_89	0	1	0	0	0	9.34	9.19	9.12	8.94	9.41	9.20	9.09	8.85	9.26	9.12	9.05	8.79	9.46	9.05	8.96	8.95	9.25	8.94	8.85	8.78
1367918_at	Fez1	fasciculation and elongation protein zeta 1 (zygin I)	0	0	0	0	0	0	uniqDR+/+_89	0	1	0	0	0	7.23	7.16	7.27	7.24	7.33	7.25	7.13	6.79	6.87	7.06	6.93	7.15	7.11	7.05	7.04	7.02	6.94	6.84	6.81	7.00
1367952_at	Lrp2	low density lipoprotein receptor-related protein 2	0	0	0	0	0	0	uniqDR+/+_89	0	1	0	0	0	4.50	4.52	4.40	4.38	4.62	4.66	4.36	4.15	4.55	4.45	4.55	4.49	4.62	4.53	4.53	4.41	4.49	4.62	4.52	4.71
1368284_at	Plvap	plasmalemma vesicle associated protein	0	0	0	0	0	0	uniqDR+/+_89	0	1	0	0	0	10.46	10.28	10.15	10.01	10.57	10.26	10.10	9.99	10.31	10.00	9.88	9.83	10.66	10.14	10.03	9.76	10.36	10.19	10.09	9.99
1368316_at	Aqp8	aquaporin 8	0	0	0	0	0	0	uniqDR+/+_89	0	1	0	0	0	5.24	5.18	4.89	4.79	5.48	4.95	4.77	4.85	5.54	5.28	4.98	5.20	6.44	6.35	5.23	4.88	5.59	5.85	5.49	5.66
1368553_at	Acvrl1	activin A receptor type II-like 1	0	0	0	0	0	0	uniqDR+/+_89	0	1	0	0	0	6.00	5.88	5.67	5.67	6.20	5.65	5.65	5.63	5.83	5.66	5.33	5.36	6.06	5.58	5.47	5.46	5.87	5.64	5.70	5.67
1368736_at	Tsx	testis specific X-linked gene	0	0	0	0	0	0	uniqDR+/+_89	0	1	0	0	0	4.82	4.81	4.89	4.58	4.89	4.66	4.78	4.24	4.81	4.58	4.42	4.38	5.06	4.33	4.64	4.48	4.83	4.34	4.45	4.47
1368782_at	Sstr2	somatostatin receptor 2	0	0	0	0	0	0	uniqDR+/+_89	0	1	0	0	0	6.38	6.22	5.87	5.60	6.44	6.20	5.94	5.52	6.15	5.77	5.63	5.12	6.23	5.92	5.89	5.25	6.25	5.97	5.79	5.63
1368902_at	Pak3	p21 protein (Cdc42/Rac)-activated kinase 3	0	0	0	0	0	0	uniqDR+/+_89	0	1	0	0	0	6.95	6.73	6.66	6.49	7.14	7.04	6.70	6.52	6.73	6.87	6.88	6.58	6.77	6.88	6.88	6.61	6.77	7.17	6.97	6.69
1369433_at	Cep78	centrosomal protein 78	0	0	0	0	0	0	uniqDR+/+_89	0	1	0	0	0	5.92	5.82	5.70	5.46	6.12	5.85	5.64	5.58	5.74	5.50	5.40	5.40	5.79	5.44	5.56	5.32	5.66	5.54	5.36	5.47
1369632_a_at	Abcc8	ATP-binding cassette, subfamily C (CFTR/MRP), member 8	0	0	0	0	0	0	uniqDR+/+_89	0	1	0	0	0	8.98	8.86	8.76	8.76	9.02	8.91	8.74	8.50	8.96	8.82	8.69	8.53	8.99	8.75	8.61	8.57	8.98	8.78	8.81	8.55
1369861_at	Gabra6	gamma-aminobutyric acid (GABA) A receptor, alpha 6	0	0	0	0	0	0	uniqDR+/+_89	0	1	0	0	0	3.45	3.30	3.26	3.31	3.72	3.51	3.59	3.18	3.41	3.37	3.23	3.38	3.37	3.40	3.46	3.14	3.30	3.34	3.33	3.55
1370043_at	Alcam	activated leukocyte cell adhesion molecule	0	0	0	0	0	0	uniqDR+/+_89	0	1	0	0	0	10.11	9.90	9.79	9.65	10.15	9.89	9.73	9.61	10.38	10.14	10.10	9.91	10.24	10.01	10.03	9.75	10.28	10.06	9.89	9.92
1370145_at	---	---	0	0	0	0	0	0	uniqDR+/+_89	0	1	0	0	0	5.34	5.36	5.38	5.13	5.51	5.36	5.27	4.95	5.36	5.37	5.34	5.21	4.97	5.42	5.46	4.89	5.33	5.32	5.10	5.34
1370823_at	Bambi	BMP and activin membrane-bound inhibitor, homolog (Xenopus laevis)	0	0	0	0	0	0	uniqDR+/+_89	0	1	0	0	0	9.10	9.06	8.97	8.75	9.25	8.96	8.82	8.73	9.14	8.80	8.74	8.70	9.15	8.70	8.76	8.64	8.82	8.79	8.73	8.46
1370932_at	Lrp4	low density lipoprotein receptor-related protein 4	0	0	0	0	0	0	uniqDR+/+_89	0	1	0	0	0	4.38	4.45	4.23	4.17	4.62	4.67	4.09	4.22	4.11	3.78	3.59	3.96	4.25	3.73	4.05	3.95	4.16	3.88	4.06	4.05
1370995_at	Pou2f1	POU class 2 homeobox 1	0	0	0	0	0	0	uniqDR+/+_89	0	1	0	0	0	5.36	5.36	5.01	4.98	5.34	5.32	5.21	4.81	6.77	6.66	6.55	6.39	6.69	6.64	6.39	6.35	6.62	6.48	6.53	6.37
1371056_at	Neo1	neogenin 1	0	0	0	0	0	0	uniqDR+/+_89	0	1	0	0	0	7.04	6.90	6.73	6.68	7.01	6.73	6.79	6.66	7.08	6.90	6.66	6.64	7.23	6.81	6.67	6.68	7.06	6.84	6.84	6.45
1371174_s_at	Sstr2	somatostatin receptor 2	0	0	0	0	0	0	uniqDR+/+_89	0	1	0	0	0	5.40	5.40	5.46	5.51	5.62	5.82	5.74	5.48	5.38	5.46	5.41	5.56	5.33	5.40	5.49	5.43	5.37	5.49	5.51	5.61
1372390_at	LOC10036300	rCG32052-like	0	0	0	0	0	0	uniqDR+/+_89	0	1	0	0	0	11.25	11.01	10.92	10.92	11.22	11.09	10.93	10.83	11.31	11.22	11.17	11.14	11.32	11.04	11.07	11.13	11.32	11.24	11.22	11.11
1372470_at	Arvcf	armadillo repeat gene deleted in velo-cardio-facial syndrome	0	0	0	0	0	0	uniqDR+/+_89	0	1	0	0	0	6.98	6.91	6.69	6.61	7.09	6.91	6.65	6.49	7.38	7.06	7.06	6.96	7.33	7.02	7.04	7.03	7.39	7.14	7.03	7.00
1372994_at	Fance	Fanconi anemia, complementation group E	0	0	0	0	0	0	uniqDR+/+_89	0	1	0	0	0	6.12	6.09	5.95	5.83	6.22	6.09	5.93	5.71	6.16	6.10	6.08	5.84	6.42	6.10	6.03	6.01	6.14	6.08	6.05	6.04
1373118_at	Camsap3	calmodulin regulated spectrin-associated protein family, member 3	0	0	0	0	0	0	uniqDR+/+_89	0	1	0	0	0	6.15	6.22	6.16	6.07	6.43	6.24	6.16	5.88	6.20	6.19	6.13	6.02	6.41	6.06	6.18	6.06	6.15	6.06	6.10	6.13
1373596_at	Vstm4	V-set and transmembrane domain containing 4	0	0	0	0	0	0	uniqDR+/+_89	0	1	0	0	0	5.52	5.36	5.17	5.37	5.68	5.43	5.14	5.32	5.39	5.23	5.12	5.05	5.77	5.14	5.25	5.17	5.51	5.36	5.35	5.40
1373885_at	Cbx5	chromobox homolog 5	0	0	0	0	0	0	uniqDR+/+_89	0	1	0	0	0	6.98	6.98	7.12	6.71	6.86	7.27	6.69	6.61	7.04	6.98	7.18	6.80	6.49	6.92	7.21	6.75	7.07	7.05	6.64	6.74
1373945_at	Celf5	CUGBP, Elav-like family member 5	0	0	0	0	0	0	uniqDR+/+_89	0	1	0	0	0	6.66	6.75	6.65	6.36	6.82	6.62	6.51	6.27	6.92	6.97	6.81	6.57	6.91	6.72	6.45	6.30	6.90	6.73	6.80	6.43
1374349_at	Ctdspl	CTD (carboxy-terminal domain, RNA polymerase II, polypeptide A) small phosphatase-like	0	0	0	0	0	0	uniqDR+/+_89	0	1	0	0	0	8.28	8.14	8.01	7.86	8.38	8.21	8.05	7.80	8.00	7.85	7.77	7.57	8.17	7.91	7.72	7.63	8.13	7.83	7.74	7.73
1374616_at	Pdgfrl	platelet-derived growth factor receptor-like	0	0	0	0	0	0	uniqDR+/+_89	0	1	0	0	0	9.58	9.54	9.42	9.42	9.74	9.56	9.43	9.21	7.45	7.32	7.26	7.04	7.57	7.31	7.15	7.10	7.38	7.15	7.18	7.03
1375251_at	Ctdspl	CTD (carboxy-terminal domain, RNA polymerase II, polypeptide A) small phosphatase-like	0	0	0	0	0	0	uniqDR+/+_89	0	1	0	0	0	4.59	4.46	4.32	4.39	4.45	4.43	4.54	4.36	4.23	4.32	4.52	4.52	4.45	4.37	4.43	4.44	4.38	4.29	4.44	4.41
1375307_at	Cbx6	chromobox homolog 6	0	0	0	0	0	0	uniqDR+/+_89	0	1	0	0	0	8.51	8.29	8.19	8.07	8.50	8.31	8.12	7.96	8.40	8.20	8.19	8.04	8.45	8.20	8.08	8.03	8.34	8.22	8.23	8.03
1375511_at	Neo1	neogenin 1	0	0	0	0	0	0	uniqDR+/+_89	0	1	0	0	0	2.22	2.20	2.27	2.28	2.25	2.41	2.41	2.24	2.27	2.18	2.27	2.29	2.26	2.39	2.31	2.49	2.27	2.36	2.36	2.35
1376786_a_at	---	---	0	0	0	0	0	0	uniqDR+/+_89	0	1	0	0	0	10.47	10.50	10.57	10.29	10.46	10.61	10.47	10.03	10.36	10.39	10.37	10.12	10.43	10.41	10.29	10.04	10.32	10.34	10.26	10.16
1376858_at	LOC686809	similar to protein 7 transactivated by hepatitis B virus X antigen	0	0	0	0	0	0	uniqDR+/+_89	0	1	0	0	0	7.18	7.03	7.05	6.89	7.25	6.91	6.93	6.73	6.88	6.73	6.63	6.76	7.40	6.86	6.83	6.75	7.03	6.92	6.96	7.12
1377990_at	---	---	0	0	0	0	0	0	uniqDR+/+_89	0	1	0	0	0	4.98	5.00	5.08	4.68	5.21	5.25	4.88	4.62	5.28	4.94	4.88	4.85	4.88	5.19	4.98	4.90	5.37	5.03	5.06	5.21
1378269_at	Dnal1	dynein, axonemal, light chain 1	0	0	0	0	0	0	uniqDR+/+_89	0	1	0	0	0	7.09	6.86	6.93	6.74	6.90	7.14	6.79	6.48	7.20	7.01	7.07	6.97	7.08	7.18	7.12	6.91	7.17	7.08	6.82	7.03
1378369_at	Dact1	dishevelled-binding antagonist of beta-catenin 1	0	0	0	0	0	0	uniqDR+/+_89	0	1	0	0	0	7.42	7.41	7.17	6.96	7.46	7.24	7.14	6.83	7.33	7.33	7.33	7.03	7.23	7.27	7.16	7.11	7.36	7.30	7.28	7.13
1379033_at	Ctdspl	CTD (carboxy-terminal domain, RNA polymerase II, polypeptide A) small phosphatase-like	0	0	0	0	0	0	uniqDR+/+_89	0	1	0	0	0	2.99	2.97	2.76	2.89	2.98	3.01	2.99	2.73	2.90	2.85	2.88	2.70	2.91	2.86	2.76	3.03	2.99	3.08	3.01	2.70
1379238_at	Ctdspl	CTD (carboxy-terminal domain, RNA polymerase II, polypeptide A) small phosphatase-like	0	0	0	0	0	0	uniqDR+/+_89	0	1	0	0	0	6.65	6.45	6.38	6.07	6.58	6.33	6.14	5.82	6.30	6.01	5.74	5.47	6.36	5.97	5.82	5.56	6.30	5.87	5.67	5.54
1379434_at	Fgd1	FYVE, RhoGEF and PH domain containing 1	0	0	0	0	0	0	uniqDR+/+_89	0	1	0	0	0	6.04	6.07	6.20	5.61	6.03	6.18	6.05	5.48	6.11	6.09	6.14	5.90	6.04	6.06	6.06	5.96	5.92	5.98	5.80	5.89
1380144_at	---	---	0	0	0	0	0	0	uniqDR+/+_89	0	1	0	0	0	6.53	6.08	6.00	5.72	6.24	6.89	5.98	5.63	6.56	6.19	6.86	6.11	4.86	6.55	6.56	5.66	6.23	6.71	5.78	5.72
1380241_a_at	Lrp5	low density lipoprotein receptor-related protein 5	0	0	0	0	0	0	uniqDR+/+_89	0	1	0	0	0	4.05	4.06	4.11	4.04	4.29	4.09	4.15	3.81	4.10	4.18	4.20	3.89	4.03	4.13	4.00	3.93	4.15	4.23	4.04	4.10
1380242_at	Lrp5	low density lipoprotein receptor-related protein 5	0	0	0	0	0	0	uniqDR+/+_89	0	1	0	0	0	3.96	4.29	4.12	3.90	4.10	4.27	4.06	3.59	4.25	3.90	3.84	4.07	3.93	3.99	3.98	3.99	3.80	4.25	3.80	4.03
1380817_at	Prex2	phosphatidylinositol-3,4,5-trisphosphate-dependent Rac exchange factor 2	0	0	0	0	0	0	uniqDR+/+_89	0	1	0	0	0	2.90	2.81	2.97	2.85	3.17	2.80	2.84	2.63	2.89	2.71	2.46	2.67	2.92	2.63	2.67	2.71	2.98	2.78	2.87	2.66
1381467_at	---	---	0	0	0	0	0	0	uniqDR+/+_89	0	1	0	0	0	7.49	7.61	7.79	7.14	7.50	7.81	7.64	7.09	7.50	7.46	7.52	7.21	7.58	7.64	7.54	7.13	7.43	7.44	7.28	7.20
1382118_at	LOC10036300	rCG32052-like	0	0	0	0	0	0	uniqDR+/+_89	0	1	0	0	0	11.44	11.18	11.13	11.09	11.54	11.27	11.06	10.96	11.41	11.24	11.29	11.22	11.01	11.18	11.18	11.17	11.50	11.34	11.23	11.23
1382617_at	Cuedc1	CUE domain containing 1	0	0	0	0	0	0	uniqDR+/+_89	0	1	0	0	0	5.74	5.54	5.57	5.30	5.58	5.51	5.37	5.07	5.57	5.44	5.44	5.35	5.62	5.60	5.28	5.11	5.58	5.43	5.59	5.49
1382937_at	Spdef	SAM pointed domain containing ets transcription factor	0	0	0	0	0	0	uniqDR+/+_89	0	1	0	0	0	3.92	3.87	3.81	3.87	4.05	4.15	4.01	3.58	4.03	3.81	3.95	3.85	4.25	3.99	3.82	3.69	4.10	4.00	3.78	4.09
1383286_at	Plek2	pleckstrin 2	0	0	0	0	0	0	uniqDR+/+_89	0	1	0	0	0	5.44	5.50	5.21	5.10	5.54	5.53	5.26	4.89	4.97	4.94	4.89	4.65	5.46	4.88	4.81	4.69	4.99	4.97	4.78	4.82
1383500_at	Rrbp1	ribosome binding protein 1	0	0	0	0	0	0	uniqDR+/+_89	0	1	0	0	0	7.84	7.53	7.07	7.08	7.90	7.36	7.02	7.36	7.89	7.40	7.40	7.51	7.78	8.06	7.36	7.31	7.66	7.85	7.49	7.57
1384048_at	RGD1310712	similar to EMSY protein	0	0	0	0	0	0	uniqDR+/+_89	0	1	0	0	0	7.10	6.98	6.88	6.65	7.13	6.95	6.78	6.65	7.20	6.89	6.82	6.69	7.07	6.88	6.82	6.63	7.20	6.82	6.73	6.69
1384448_at	RGD1565844	similar to RIKEN cDNA 1700045I19	0	0	0	0	0	0	uniqDR+/+_89	0	1	0	0	0	6.78	6.71	6.65	6.35	6.97	6.71	6.66	6.36	6.64	6.52	6.50	6.32	6.56	6.43	6.49	6.21	6.66	6.39	6.43	6.24
1384675_at	Apeg3	antisense paternally expressed gene 3	0	0	0	0	0	0	uniqDR+/+_89	0	1	0	0	0	7.24	6.98	6.93	6.84	7.28	7.33	6.73	6.72	7.12	6.96	6.96	6.87	6.88	6.93	6.91	6.81	7.10	7.02	6.83	7.07
1385274_at	Pcdhb19	protocadherin beta 19	0	0	0	0	0	0	uniqDR+/+_89	0	1	0	0	0	5.57	5.28	5.34	5.24	5.64	5.19	5.20	4.99	5.07	4.90	4.93	5.10	5.17	4.97	5.08	4.95	4.96	4.88	4.76	5.00
1385555_at	Fam101a	family with sequence similarity 101, member A	0	0	0	0	0	0	uniqDR+/+_89	0	1	0	0	0	6.00	5.86	5.81	5.75	6.19	5.92	5.83	5.67	5.67	5.55	5.49	5.48	5.70	5.56	5.50	5.19	5.66	5.50	5.36	5.58
1386876_at	Adcy6	adenylate cyclase 6	0	0	0	0	0	0	uniqDR+/+_89	0	1	0	0	0	4.92	4.86	4.71	4.69	5.03	5.07	4.87	4.51	4.89	4.80	4.92	4.73	4.56	4.95	4.77	4.79	4.93	4.76	4.65	4.67
1387274_at	Dlx5	distal-less homeobox 5	0	0	0	0	0	0	uniqDR+/+_89	0	1	0	0	0	3.70	3.49	3.22	3.31	3.80	3.55	3.29	3.32	3.66	3.39	3.57	3.36	3.98	3.54	3.51	3.59	3.69	3.73	3.42	3.46
1387503_at	Cpn1	carboxypeptidase N, polypeptide 1	0	0	0	0	0	0	uniqDR+/+_89	0	1	0	0	0	9.39	9.26	9.14	8.96	9.38	9.22	9.06	8.81	9.05	8.98	8.92	8.73	8.98	9.01	8.89	8.70	9.05	8.91	8.97	8.77
1387547_a_at	Eltd1	EGF, latrophilin and seven transmembrane domain containing 1	0	0	0	0	0	0	uniqDR+/+_89	0	1	0	0	0	6.74	6.48	6.30	6.41	6.80	6.43	6.29	6.40	6.79	6.55	6.48	6.42	6.91	6.62	6.41	6.17	6.94	6.54	6.59	6.56
1388006_at	Muc13	mucin 13, cell surface associated	0	0	0	0	0	0	uniqDR+/+_89	0	1	0	0	0	5.47	5.82	5.78	5.52	5.69	5.85	5.69	5.35	4.92	5.19	4.77	4.88	5.14	5.24	5.00	4.89	5.14	5.11	5.16	4.72
1388299_at	Dnal1	dynein, axonemal, light chain 1	0	0	0	0	0	0	uniqDR+/+_89	0	1	0	0	0	4.76	4.30	4.40	4.33	4.55	4.54	4.20	4.03	4.75	4.64	4.63	4.42	4.15	4.67	4.59	4.23	4.46	4.60	4.28	4.36
1388413_at	Rrbp1	ribosome binding protein 1	0	0	0	0	0	0	uniqDR+/+_89	0	1	0	0	0	9.63	9.51	9.50	9.60	9.66	9.49	9.48	9.50	9.71	9.58	9.60	9.66	9.82	9.63	9.56	9.61	9.66	9.65	9.71	9.56
1389405_at	Chd9	chromodomain helicase DNA binding protein 9	0	0	0	0	0	0	uniqDR+/+_89	0	1	0	0	0	9.51	9.54	9.44	9.45	9.64	9.53	9.30	9.30	9.47	9.36	9.38	9.22	9.47	9.24	9.37	9.36	9.38	9.39	9.33	9.26
1389894_at	Dlc1	deleted in liver cancer 1	0	0	0	0	0	0	uniqDR+/+_89	0	1	0	0	0	4.48	4.40	4.30	4.02	4.42	4.53	4.20	3.91	4.48	4.41	4.24	4.26	4.74	4.39	4.24	4.57	4.71	4.65	4.58	4.53
1390123_at	Tmem45b	transmembrane protein 45b	0	0	0	0	0	0	uniqDR+/+_89	0	1	0	0	0	4.64	4.40	4.35	4.38	4.74	4.82	4.42	4.30	5.16	4.85	5.08	4.97	5.12	5.02	4.90	4.77	5.15	4.99	5.03	5.05
1390219_at	Wdr19	WD repeat domain 19	0	0	0	0	0	0	uniqDR+/+_89	0	1	0	0	0	5.99	5.86	5.72	5.59	5.91	5.86	5.62	5.39	6.01	5.88	5.68	5.64	6.16	5.83	5.73	5.64	5.97	5.92	5.82	5.63
1390468_at	---	---	0	0	0	0	0	0	uniqDR+/+_89	0	1	0	0	0	8.94	8.96	9.09	8.66	8.97	9.12	8.92	8.40	8.87	8.88	8.83	8.61	9.01	8.85	8.76	8.45	8.77	8.79	8.71	8.65
1390520_at	Dnajb3	DnaJ (Hsp40) homolog, subfamily B, member 3	0	0	0	0	0	0	uniqDR+/+_89	0	1	0	0	0	5.33	5.30	5.29	5.11	5.30	5.31	5.27	4.79	5.02	5.31	5.24	5.10	5.20	5.18	5.00	5.08	5.18	5.24	5.18	4.86
1390533_at	LOC691920	similar to kinesin-like motor protein C20orf23	0	0	0	0	0	0	uniqDR+/+_89	0	1	0	0	0	4.19	4.16	3.90	3.85	4.25	3.91	3.72	3.89	4.26	4.30	4.37	4.30	4.43	4.20	4.16	4.15	4.21	4.36	4.26	4.19
1390655_at	LOC10036300	rCG32052-like	0	0	0	0	0	0	uniqDR+/+_89	0	1	0	0	0	8.70	8.18	7.82	7.84	8.58	8.08	7.71	7.72	8.77	8.28	8.31	8.15	7.42	8.16	8.13	7.94	8.77	8.46	8.09	7.94
1391607_at	Sesn2	sestrin 2	0	0	0	0	0	0	uniqDR+/+_89	0	1	0	0	0	8.97	9.20	8.92	8.89	9.00	9.18	8.98	8.67	8.94	8.91	8.86	8.66	9.03	8.84	8.90	8.74	8.95	8.95	8.87	8.73
1391635_at	Ctdspl	CTD (carboxy-terminal domain, RNA polymerase II, polypeptide A) small phosphatase-like	0	0	0	0	0	0	uniqDR+/+_89	0	1	0	0	0	7.82	7.72	7.39	7.36	7.80	7.49	7.46	7.25	6.03	5.85	5.65	5.59	5.23	5.73	5.58	5.70	5.87	5.73	5.80	5.69
1391855_at	LOC690085	similar to B-cell CLL/lymphoma 7A	0	0	0	0	0	0	uniqDR+/+_89	0	1	0	0	0	5.89	5.76	5.71	5.70	5.94	5.83	5.79	5.43	5.95	5.75	5.79	5.56	5.82	5.77	5.83	5.50	5.81	5.75	5.51	5.54
1392002_at	Pcdhb22	protocadherin beta 22	0	0	0	0	0	0	uniqDR+/+_89	0	1	0	0	0	3.35	3.32	3.11	3.38	3.60	3.41	3.07	3.06	3.14	2.96	2.95	3.11	3.32	2.93	2.93	2.91	2.99	3.07	3.02	3.15
1392520_at	Dlc1	deleted in liver cancer 1	0	0	0	0	0	0	uniqDR+/+_89	0	1	0	0	0	4.79	4.54	4.61	4.43	4.94	4.60	4.53	4.33	4.89	4.65	4.49	4.49	4.67	4.71	4.73	4.54	4.95	4.79	4.60	4.46
1392550_at	Dact1	dishevelled-binding antagonist of beta-catenin 1	0	0	0	0	0	0	uniqDR+/+_89	0	1	0	0	0	6.97	6.88	6.59	6.49	7.02	6.90	6.70	6.49	6.92	6.92	6.79	6.54	6.97	6.72	6.65	6.61	6.83	6.85	6.86	6.48
1392552_at	Gatad2b	GATA zinc finger domain containing 2B	0	0	0	0	0	0	uniqDR+/+_89	0	1	0	0	0	5.46	5.30	5.28	5.05	5.58	5.69	5.22	5.08	5.86	5.31	5.50	5.04	5.10	5.38	5.44	5.15	5.50	5.52	5.29	5.39
1392770_at	Neo1	neogenin 1	0	0	0	0	0	0	uniqDR+/+_89	0	1	0	0	0	8.96	8.77	8.64	8.63	8.95	8.80	8.67	8.52	8.94	8.73	8.62	8.45	9.22	8.67	8.61	8.53	8.86	8.72	8.72	8.44
1392795_at	---	---	0	0	0	0	0	0	uniqDR+/+_89	0	1	0	0	0	3.57	3.55	3.62	3.41	4.11	3.57	3.63	3.50	3.35	3.36	3.31	3.17	3.49	3.05	3.25	3.23	3.39	2.99	3.06	3.17
1393234_at	LOC687105	hypothetical protein LOC687105	0	0	0	0	0	0	uniqDR+/+_89	0	1	0	0	0	6.81	6.74	6.63	6.70	6.90	6.85	6.63	6.37	6.70	6.71	6.65	6.46	6.86	6.53	6.60	6.43	6.68	6.64	6.65	6.56
1393629_at	Hlx	H2.0-like homeobox	0	0	0	0	0	0	uniqDR+/+_89	0	1	0	0	0	6.80	6.79	6.66	6.65	7.13	6.79	6.60	6.46	6.48	6.41	6.25	6.40	6.98	6.25	6.56	6.43	6.72	6.44	6.35	6.48

Affymetrix Probeset ID	Gene Symbol	Gene Name	unique BB on P7 n=822	Figure 1B P7 Venn n=1526	DRlyp/lyp STEM P7 n=712	DR+/+ STEM P7 n=936	Flyp/lyp STEM P7 n=520	F+/+ STEM P7 n=465	Figure 1B P0 Venn n=1071	DRlyp/lyp STEM P0 n=442	DR+/+ STEM P0 n=437	Flyp/lyp STEM P0 n=643	F+/+ STEM P0 n=632	Fig1D n=25	Mean log2 intensity DRlyp/lyp 20	Mean log2 intensity DRlyp/lyp 30	Mean log2 intensity DRlyp/lyp 40	Mean log2 intensity DRlyp/lyp 50	Mean log2 intensity DR+/+ 20	Mean log2 intensity DR+/+ 30	Mean log2 intensity DR+/+ 40	Mean log2 intensity DR+/+ 50	Mean log2 intensity Flyp/lyp 20	Mean log2 intensity Flyp/lyp 30	Mean log2 intensity Flyp/lyp 40	Mean log2 intensity Flyp/lyp 50	Mean log2 intensity Flyp20	Mean log2 intensity Flyp30	Mean log2 intensity Flyp40	Mean log2 intensity Flyp50	Mean log2 intensity F+/+ 20	Mean log2 intensity F+/+ 30	Mean log2 intensity F+/+ 40	Mean log2 intensity F+/+ 50
1393808_at	Fa2h	fatty acid 2-hydroxylase	0	0	0	0	0	0	uniqDR+/+_89	0	1	0	0	0	3.75	3.74	3.63	3.65	3.89	4.08	3.82	3.51	3.95	3.85	3.80	3.66	3.80	3.76	3.72	3.77	3.97	3.88	3.74	3.74
1395318_at	Chd9	chromodomain helicase DNA binding protein 9	0	0	0	0	0	0	uniqDR+/+_89	0	1	0	0	0	7.35	7.19	6.98	6.77	7.24	7.53	6.83	6.66	7.27	7.00	7.43	6.97	6.51	7.03	7.38	6.62	7.34	7.30	6.78	6.84
1397484_at	Dnal1	dynein, axonemal, light chain 1	0	0	0	0	0	0	uniqDR+/+_89	0	1	0	0	0	3.66	3.39	3.58	3.43	3.32	3.48	3.22	3.19	3.45	3.54	3.38	3.40	3.12	3.50	3.46	3.45	3.49	3.50	3.43	3.39
1397533_at	Mob1b	MOB kinase activator 1B	0	0	0	0	0	0	uniqDR+/+_89	0	1	0	0	0	3.20	3.34	3.04	3.03	3.28	3.27	3.02	3.17	3.21	3.08	3.30	3.41	2.98	3.35	3.42	3.06	3.12	3.13	2.96	3.51
1398020_at	Mir23b	microRNA mir-23b	0	0	0	0	0	0	uniqDR+/+_89	0	1	0	0	0	7.24	7.10	6.89	6.88	7.22	7.25	7.14	6.71	7.71	7.54	7.48	7.25	7.45	7.28	7.53	7.50	7.56	7.30	7.48	7.25
1398435_at	Slc22a15	solute carrier family 22, member 15	0	0	0	0	0	0	uniqDR+/+_89	0	1	0	0	0	6.13	6.08	6.00	5.86	6.21	6.04	5.94	5.69	6.14	6.05	5.90	5.85	6.27	5.97	5.91	5.72	6.08	6.17	6.43	5.98
1398486_at	LOC683007	similar to melanoma inhibitory activity 3	0	0	0	0	0	0	uniqDR+/+_89	0	1	0	0	0	9.53	9.35	9.29	9.16	9.35	9.63	9.13	9.07	9.25	9.07	9.50	9.24	8.18	9.12	9.46	9.01	9.21	9.45	8.82	8.98
1367858_at	Mmp11	matrix metallopeptidase 11	0	0	0	0	0	0	uniqDRlyp/lyp_54	1	0	0	0	0	4.27	3.99	3.84	3.76	3.95	3.98	3.99	3.81	3.94	3.75	3.82	3.76	4.34	3.99	3.83	3.83	3.99	3.86	3.93	3.90
1367961_at	Klk1	kallikrein 1	0	0	0	0	0	0	uniqDRlyp/lyp_54	1	0	0	0	0	8.58	8.85	7.89	8.02	8.60	8.32	7.98	8.80	9.21	8.67	8.88	8.97	8.96	10.16	8.94	8.80	8.73	9.56	8.93	9.77
1368335_at	Apoa1	apolipoprotein A-I	0	0	0	0	0	0	uniqDRlyp/lyp_54	1	0	0	0	0	2.96	3.42	2.85	2.67	3.04	3.05	2.78	2.78	2.97	2.79	2.78	2.85	2.77	2.98	2.91	2.86	2.94	3.16	2.98	2.83
1368494_at	S100a8	S100 calcium binding protein A8	0	0	0	0	0	0	uniqDRlyp/lyp_54	1	0	0	0	0	3.29	2.78	2.66	2.77	2.92	2.60	2.76	2.72	2.72	3.00	2.84	3.12	2.95	2.91	2.77	3.01	2.75	2.81	2.78	2.74
1368533_at	Heph	hephaestin	0	0	0	0	0	0	uniqDRlyp/lyp_54	1	0	0	0	0	5.36	5.33	5.00	4.83	5.30	5.20	5.30	5.18	5.41	5.19	5.13	5.23	5.55	5.27	5.41	5.27	5.71	5.30	5.23	5.27
1368554_at	Pnlip	pancreatic lipase	0	0	0	0	0	0	uniqDRlyp/lyp_54	1	0	0	0	0	12.79	12.92	12.02	12.04	12.91	12.76	12.24	12.76	12.73	12.62	12.78	12.51	12.39	13.33	12.94	12.39	12.49	13.20	12.75	13.21
1368829_at	Fbn1	fibrillin 1	0	0	0	0	0	0	uniqDRlyp/lyp_54	1	0	0	0	0	7.08	6.84	6.48	6.50	7.15	6.81	6.37	6.73	6.44	6.14	5.92	5.94	6.88	6.08	6.19	6.17	6.62	6.28	6.21	6.01
1369030_at	Prss1	protease, serine, 1 (trypsin 1)	0	0	0	0	0	0	uniqDRlyp/lyp_54	1	0	0	0	0	13.22	13.43	12.88	12.93	13.37	13.37	13.02	13.38	13.45	13.31	13.29	13.15	13.22	13.73	13.37	13.06	13.28	13.64	13.39	13.58
1369853_at	Neurog3	neurogenin 3	0	0	0	0	0	0	uniqDRlyp/lyp_54	1	0	0	0	0	4.99	4.88	4.75	4.48	4.98	4.94	4.62	4.51	4.92	4.56	4.48	4.50	4.82	4.74	4.62	4.49	4.87	4.72	4.62	4.65
1369929_at	Psap	prosaposin	0	0	0	0	0	0	uniqDRlyp/lyp_54	1	0	0	0	0	10.23	10.22	10.30	9.68	9.91	10.27	10.25	9.84	10.13	10.12	10.20	10.23	9.93	10.27	10.17	10.31	10.09	10.18	9.85	9.81
1369951_at	Ctrb1	chymotrypsinogen B1	0	0	0	0	0	0	uniqDRlyp/lyp_54	1	0	0	0	0	13.21	13.20	12.48	12.47	13.19	13.08	12.55	12.93	13.42	13.24	13.20	13.05	13.42	13.50	13.26	12.90	13.24	13.41	13.15	13.20
1371006_at	Jag2	jagged 2	0	0	0	0	0	0	uniqDRlyp/lyp_54	1	0	0	0	0	4.66	4.49	4.19	4.12	4.48	4.43	4.33	4.21	4.31	4.30	4.23	4.15	4.31	4.36	4.40	4.19	4.52	4.29	4.23	4.25
1371303_at	Tdh	L-threonine dehydrogenase	0	0	0	0	0	0	uniqDRlyp/lyp_54	1	0	0	0	0	5.41	5.93	4.79	4.90	5.77	5.32	4.75	5.54	4.61	4.59	4.40	4.85	5.67	5.55	4.44	4.29	4.74	5.24	4.97	5.06
1371695_at	Tpr	translocated promoter region, nuclear basket protein	0	0	0	0	0	0	uniqDRlyp/lyp_54	1	0	0	0	0	8.14	8.07	8.08	7.95	7.99	8.08	7.91	8.09	8.19	8.10	8.02	7.93	8.13	8.02	8.09	7.98	8.13	8.06	7.99	8.01
1372296_at	Sh3bgr	SH3 domain binding glutamic acid-rich protein	0	0	0	0	0	0	uniqDRlyp/lyp_54	1	0	0	0	0	6.34	6.17	6.05	5.81	6.43	6.24	6.09	6.01	6.31	6.27	6.28	6.20	6.42	6.38	6.17	5.98	6.45	6.37	6.26	6.11
1372492_at	Cldn10	claudin 10	0	0	0	0	0	0	uniqDRlyp/lyp_54	1	0	0	0	0	4.10	4.03	3.36	3.52	4.11	3.85	3.76	4.07	3.99	3.92	3.76	3.96	4.50	4.61	3.46	3.58	4.07	4.21	3.85	4.20
1372923_at	---	---	0	0	0	0	0	0	uniqDRlyp/lyp_54	1	0	0	0	0	5.40	5.20	5.07	4.59	5.33	5.33	5.01	5.13	7.54	7.61	7.61	7.62	7.70	7.70	7.63	7.58	7.53	7.57	7.52	7.60
1373567_at	RGD1561530	similar to Tle6 protein	0	0	0	0	0	0	uniqDRlyp/lyp_54	1	0	0	0	0	4.68	4.69	4.69	4.13	4.41	4.57	4.60	4.39	4.38	4.45	4.37	4.37	4.51	4.43	4.41	4.40	4.54	4.40	4.46	4.43
1373723_at	Gpihbp1	glycosylphosphatidylinositol anchored high density lipoprotein binding protein 1	0	0	0	0	0	0	uniqDRlyp/lyp_54	1	0	0	0	0	7.22	7.01	6.94	6.60	7.11	6.96	6.91	6.74	7.46	7.43	7.41	7.40	7.86	7.57	7.37	7.40	7.54	7.15	7.17	7.24
1373882_at	---	---	0	0	0	0	0	0	uniqDRlyp/lyp_54	1	0	0	0	0	6.06	5.65	5.51	5.19	5.98	5.82	5.53	5.35	5.49	5.37	4.73	4.86	5.44	5.41	5.21	5.07	5.40	5.20	5.35	4.82
1374142_at	Prr15	proline rich 15	0	0	0	0	0	0	uniqDRlyp/lyp_54	1	0	0	0	0	4.82	4.74	4.36	4.28	4.54	4.62	4.42	4.22	3.75	3.90	3.61	3.48	4.03	3.91	3.74	3.60	4.00	3.88	3.88	3.60
1376279_at	Pop1	processing of precursor 1, ribonuclease P/MRP subunit (S. cerevisiae)	0	0	0	0	0	0	uniqDRlyp/lyp_54	1	0	0	0	0	6.23	6.19	5.93	5.68	6.19	5.96	5.86	5.80	6.00	5.88	5.58	5.60	5.94	5.66	5.54	5.36	5.84	5.60	5.70	5.35
1376725_at	Lrp6	low density lipoprotein receptor-related protein 6	0	0	0	0	0	0	uniqDRlyp/lyp_54	1	0	0	0	0	7.69	7.44	7.25	7.34	7.61	7.51	7.31	7.11	7.55	7.26	7.46	7.16	6.89	7.33	7.41	7.00	7.49	7.56	7.06	6.97
1376831_at	Fam64a	family with sequence similarity 64, member A	0	0	0	0	0	0	uniqDRlyp/lyp_54	1	0	0	0	0	4.20	4.14	3.55	3.72	3.97	3.94	3.69	3.72	3.85	4.06	3.90	3.52	4.02	3.76	3.93	3.58	4.03	3.85	3.84	3.88
1377940_at	Fam101b	family with sequence similarity 101, member B	0	0	0	0	0	0	uniqDRlyp/lyp_54	1	0	0	0	0	7.38	7.20	7.04	6.84	7.25	7.15	7.11	7.16	7.14	6.95	6.85	6.75	7.54	7.03	6.87	6.81	7.25	6.99	6.94	6.81
1378168_at	Fam101b	family with sequence similarity 101, member B	0	0	0	0	0	0	uniqDRlyp/lyp_54	1	0	0	0	0	6.82	6.67	6.67	6.33	6.83	6.79	6.63	6.65	6.69	6.55	6.41	6.43	6.73	6.57	6.53	6.36	6.65	6.52	6.53	6.42
1379257_at	Epb41l4a	erythrocyte membrane protein band 4.1 like 4A	0	0	0	0	0	0	uniqDRlyp/lyp_54	1	0	0	0	0	8.12	7.95	7.85	7.59	8.22	8.09	7.75	7.73	7.88	7.73	7.67	7.50	8.01	7.78	7.60	7.43	7.71	7.73	7.66	7.48
1379361_at	Pex11a	peroxisomal biogenesis factor 11 alpha	0	0	0	0	0	0	uniqDRlyp/lyp_54	1	0	0	0	0	6.40	6.19	6.07	5.82	6.21	5.95	6.09	5.95	5.64	5.77	5.64	5.73	5.63	5.70	5.68	5.67	5.79	5.72	5.55	5.77
1379380_at	Spry1	sprouty homolog 1, antagonist of FGF signaling (Drosophila)	0	0	0	0	0	0	uniqDRlyp/lyp_54	1	0	0	0	0	7.30	7.14	6.99	6.72	7.33	7.08	7.04	6.83	7.04	6.87	6.86	6.70	7.24	6.73	6.89	6.82	7.15	7.00	7.09	6.72
1379488_at	Tp53rk	TP53 regulating kinase	0	0	0	0	0	0	uniqDRlyp/lyp_54	1	0	0	0	0	6.50	6.36	6.21	6.00	6.47	6.36	6.30	6.32	6.17	6.20	6.19	5.92	6.27	6.11	6.22	6.08	6.23	5.98	6.11	5.85
1380250_at	Sned1	sushi, nidogen and EGF-like domains 1	0	0	0	0	0	0	uniqDRlyp/lyp_54	1	0	0	0	0	5.49	5.03	5.10	4.90	5.54	5.06	5.19	5.19	5.32	4.88	4.89	5.16	5.54	5.16	5.08	5.25	5.36	4.94	5.05	5.17
1380581_at	Zfp867	zinc finger protein 867	0	0	0	0	0	0	uniqDRlyp/lyp_54	1	0	0	0	0	4.64	4.60	4.45	4.02	4.47	4.25	4.17	4.21	4.45	4.61	4.59	4.26	4.55	4.55	4.56	4.45	4.51	4.49	4.62	4.39
1380688_at	Galnt18	UDP-N-acetyl-alpha-D-galactosamine:polypeptide N-acetylgalactosaminyltransferase 18	0	0	0	0	0	0	uniqDRlyp/lyp_54	1	0	0	0	0	7.18	6.85	6.84	6.67	7.10	6.94	6.72	6.66	6.74	6.62	6.59	6.51	6.95	6.63	6.47	6.42	6.70	6.44	6.37	6.30
1382130_at	Pcdh19	protocadherin 19	0	0	0	0	0	0	uniqDRlyp/lyp_54	1	0	0	0	0	7.07	6.85	6.60	6.56	7.03	6.71	6.61	6.62	7.00	6.80	6.64	6.55	7.22	6.77	6.68	6.66	7.06	6.89	6.76	6.69
1382939_at	Tpr	translocated promoter region, nuclear basket protein	0	0	0	0	0	0	uniqDRlyp/lyp_54	1	0	0	0	0	8.75	8.42	8.35	8.12	8.59	8.72	8.06	8.08	8.74	8.45	8.69	8.39	8.10	8.52	8.67	8.18	8.67	8.76	8.27	8.29
1382981_at	Ahi1	Abelson helper integration site 1	0	0	0	0	0	0	uniqDRlyp/lyp_54	1	0	0	0	0	7.87	7.77	7.82	7.00	7.45	7.82	7.47	7.22	8.05	7.72	7.81	7.68	7.19	7.64	7.79	7.24	7.93	7.77	7.66	7.54
1383481_at	LOC1003628	autism susceptibility candidate 2-like	0	0	0	0	0	0	uniqDRlyp/lyp_54	1	0	0	0	0	6.51	6.22	6.12	5.83	6.39	6.24	6.00	5.89	6.18	5.79	5.96	5.61	5.95	5.96	5.79	5.67	6.03	5.82	5.91	5.61
1384526_at	Tex15	testis expressed 15	0	0	0	0	0	0	uniqDRlyp/lyp_54	1	0	0	0	0	3.84	3.64	3.50	3.27	3.72	3.53	3.43	3.32	3.66	3.64	3.62	3.77	4.33	3.90	3.59	3.73	3.58	3.96	3.80	3.87
1385687_at	Fam25c	family with sequence similarity 25, member C	0	0	0	0	0	0	uniqDRlyp/lyp_54	1	0	0	0	0	5.06	5.41	4.78	4.80	5.46	5.17	5.05	5.01	5.24	5.05	4.94	5.11	5.06	5.58	4.92	5.00	5.09	5.43	4.87	5.51
1386933_at	Gp2	glycoprotein 2 (zymogen granule membrane)	0	0	0	0	0	0	uniqDRlyp/lyp_54	1	0	0	0	0	9.89	10.15	8.85	8.99	9.74	9.63	9.20	10.02	10.27	9.92	9.97	9.93	10.30	11.34	10.13	9.71	8.93	10.47	9.68	10.83
1387351_at	Fbn1	fibrillin 1	0	0	0	0	0	0	uniqDRlyp/lyp_54	1	0	0	0	0	6.60	6.36	6.03	6.08	6.66	6.46	6.06	6.39	5.99	5.85	5.57	5.81	6.11	5.78	5.85	5.80	6.23	5.97	5.95	5.92
1387709_at	Figf	c-fos induced growth factor	0	0	0	0	0	0	uniqDRlyp/lyp_54	1	0	0	0	0	3.65	3.52	3.32	3.46	3.55	3.49	3.38	3.33	3.35	3.33	3.43	3.28	3.25	3.24	3.56	3.32	3.46	3.43	3.37	3.44
1387740_at	Pex11a	peroxisomal biogenesis factor 11 alpha	0	0	0	0	0	0	uniqDRlyp/lyp_54	1	0	0	0	0	3.13	2.91	2.81	2.66	3.03	2.85	2.89	2.82	2.67	2.53	2.59	2.55	2.42	2.63	2.72	2.49	2.67	2.69	2.36	2.40
1387822_at	Gna11	guanine nucleotide binding protein, alpha 11	0	0	0	0	0	0	uniqDRlyp/lyp_54	1	0	0	0	0	6.92	6.81	6.89	6.37	6.41	6.98	6.79	6.32	6.84	6.83	6.97	6.84	6.42	7.02	6.92	6.69	6.85	6.86	6.15	6.48
1388536_at	Prrc2a	proline-rich coiled-coil 2A	0	0	0	0	0	0	uniqDRlyp/lyp_54	1	0	0	0	0	6.75	6.65	6.52	6.04	6.54	6.53	6.60	6.18	6.50	6.55	6.64	6.50	6.42	6.73	6.50	6.55	6.60	6.59	6.22	6.29
1390063_at	Mfap3	microfibrillar-associated protein 3	0	0	0	0	0	0	uniqDRlyp/lyp_54	1	0	0	0	0	7.67	7.41	7.49	7.14	7.56	7.52	7.16	7.11	8.28	8.19	8.04	7.94	8.56	8.12	7.95	7.85	8.26	8.17	8.07	7.89
1390114_at	Mpzl1	myelin protein zero-like 1	0	0	0	0	0	0	uniqDRlyp/lyp_54	1	0	0	0	0	7.52	7.34	7.32	6.92	7.50	7.40	7.12	7.00	7.50	7.43	7.35	7.14	7.65	7.40	7.37	7.12	7.57	7.40	7.36	7.12
1390403_at	Fam43a	family with sequence similarity 43, member A	0	0	0	0	0	0	uniqDRlyp/lyp_54	1	0	0	0	0	8.59	8.40	8.22	7.93	8.47	8.52	8.10	8.02	8.58	8.54	8.50	8.36	8.75	8.52	8.24	8.13	8.44	8.72	8.59	8.13
1393672_at	Hmcn1	hemicentin 1	0	0	0	0	0	0	uniqDRlyp/lyp_54	1	0	0	0	0	3.91	3.69	3.68	3.38	3.84	3.65	3.53	3.76	2.87	3.00	2.76	3.04	3.26	3.11	3.12	3.22	3.45	3.24	3.17	3.02
1393750_at	G2e3	G2/M-phase specific E3 ubiquitin protein ligase	0	0	0	0	0	0	uniqDRlyp/lyp_54	1	0	0	0	0	3.73	3.59	3.45	3.13	3.77	3.34	3.21	3.46	3.91	3.88	3.47	3.72	3.46	3.50	3.85	3.51	3.73	3.69	3.52	3.77
1393754_at	Tbc1d1	TBC1 (tre-2/USP6, BUB2, cdc16) domain family, member 1	0	0	0	0	0	0	uniqDRlyp/lyp_54	1	0	0	0	0	4.57	4.32	4.23	3.97	4.46	4.32	4.28	4.18	4.54	4.32	4.42	4.22	4.31	4.38	4.34	4.28	4.59	4.29	4.37	4.27
1393809_at	Traf6	TNF receptor-associated factor 6, E3 ubiquitin protein ligase	0	0	0	0	0	0	uniqDRlyp/lyp_54	1	0	0	0	0	7.99	7.25	7.51	6.36	6.56	7.87	7.16	6.20	7.77	7.47	8.01	7.36	7.16	7.71	7.94	6.78	7.79	7.89	7.59	7.64
1394814_at	Tpr	translocated promoter region, nuclear basket protein	0	0	0	0	0	0	uniqDRlyp/lyp_54	1	0	0	0	0	6.17	6.14	6.02	5.56	6.22	6.27	5.49	6.00	6.36	6.08	6.29	6.18	5.11	5.93	6.25	5.85	6.25	6.25	5.57	6.09
1395413_at	---	---	0	0	0	0	0	0	uniqDRlyp/lyp_54	1	0	0	0	0	6.63	6.30	6.17	5.96	6.30	6.50	6.01	5.96	6.73	6.22	6.41	6.39	6.02	6.59	6.45	6.09	6.58	6.48	6.08	6.14
1367531_at	Eif4h	eukaryotic translation initiation factor 4H	0	0	0	0	0	0	uniqF+/+_163	0	0	0	1	0	10.30	10.12	10.01	9.92	10.30	10.16	9.98	9.86	10.26	10.06	10.01	9.77	10.37	10.11	10.01	9.79	10.27	10.13	9.95	9.81
1367579_a_at	LOC1009094	tubulin alpha-1C chain-like /// tubulin alpha-1 chain-like /// tubulin, alpha 1A /// tubulin, alp	0	0	0	0	0	0	uniqF+/+_163	0	0	0	1	0	10.87	10.68	10.53	10.72	10.90	10.70	10.74	10.88	10.86	10.75	10.63	10.43	10.85	10.51	10.50	10.45	10.91	10.54	10.52	10.32
1367632_at	Glul	glutamate-ammonia ligase	0	0	0	0	0	0	uniqF+/+_163	0	0	0	1	0	10.08	9.96	9.83	9.71	9.88	9.83	9.92	9.60	10.59	10.39	10.26	10.05	10.55	10.49	10.17	10.05	10.55	10.37	10.20	10.10
1367633_at	Glul	glutamate-ammonia ligase	0	0	0	0	0	0	uniqF+/+_163	0	0	0	1	0	8.67	8.67	8.52	8.21	8.50	8.52	8.63	8.31	9.24	9.10	9.03	8.83	7.97	9.28	8.97	8.72	9.24	8.97	8.28	8.21
1367725_at	Pim3	pim-3 oncogene	0	0	0	0	0	0	uniqF+/+_163	0	0	0	1	0	9.37	9.37	9.23	8.99	9.40	9.21	9.39	9.09	9.61	9.68	9.54	9.19	9.53	9.57	9.13	9.02	9.47	9.55	9.51	8.97
1367869_at	Oxr1	oxidation resistance 1	0	0	0	0	0	0	uniqF+/+_163	0	0	0	1	0	8.48	8.45	8.32	8.35	8.49	8.42	8.24	8.29	8.89	8.73	8.64	8.53	9.08	8.61	8.70	8.46	8.95	8.69	8.59	8.40
1367890_at	Casp2	caspase 2	0	0	0	0	0	0	uniqF+/+_163	0	0	0	1	0	8.21	8.01	7.95	7.98	8.23	8.01	8.00	7.98	8.15	7.93	7.79	7.70	8.38	7.88	7.90	7.76	8.17	7.86	7.90	7.75
1367891_a_at	Casp2	caspase 2	0	0	0	0	0	0	uniqF+/+_163	0	0	0	1	0	4.85	4.90	4.71	4.51	4.66	4.64	4.62	4.66	5.06	4.67	4.43	4.66	4.21	4.76	4.72	4.37	4.81	4.64	4.26	4.14
1368052_at	Tspan8	tetraspanin 8	0	0	0	0	0	0	uniqF+/+_163	0	0	0	1	0	6.75	6.56	6.57	6.68	6.81	6.51	7.45	6.87	6.34	6.82	6.02	6.31	6.39	6.17	6.15	6.61	6.67	6.33	6.30	6.16
1368146_at	Dusp1	dual specificity phosphatase 1	0	0	0	0	0	0	uniqF+/+_163	0	0	0	1	0	10.91	11.09	11.03	11.04	11.04	10.85	11.65	11.27	10.89	10.74	10.86	10.57	10.83	10.73	10.73	10.73	10.75	10.61	10.69	10.45
1368147_at	Dusp1	dual specificity phosphatase 1	0	0	0	0	0	0	uniqF+/+_163	0	0	0	1	0	7.56	7.86	7.83	7.54	7.94	7.58	8.66	8.26	7.69	7.47	7.57	7.18	6.49	7.34	7.51	7.28	7.40	7.27	7.03	6.70
1368187_at	Gpnmb	glycoprotein (transmembrane) nmb	0	0	0	0	0	0	uniqF+/+_163	0	0	0	1	0	4.85	3.89	4.40	6.03	4.54	4.18	4.37	5.64	4.42	4.59	4.19	4.78	4.68	4.35	4.53	4.59	4.51	4.13	3.96	3.85
1368209_at	Pdzk1ip1	PDZK1 interacting protein 1	0	0	0	0	0	0	uniqF+/+_163	0	0	0	1	0	6.25	6.36	6.13	5.88	6.33	6.28	6.14	5.87	5.98	5.86	5.71	5.57	6.10	5.93	5.74	5.53	6.37	5.94	5.96	5.79
1368549_at	Hbp1	HMG-box transcription factor 1	0	0	0	0	0	0	uniqF+/+_163	0	0	0	1	0	8.15	8.02	7.85	7.92	8.22	7.94	7.93	7.72	8.29	8.02	7.87	7.80	8.25	7.96	8.08	7.87	8.33	7.90	7.82	7.88
1368752_at	Tacr3	tachykinin receptor 3	0	0	0	0	0	0	uniqF+/+_163	0	0	0	1	0	5.55	5.35	5.45	5.09	5.36	5.55	5.32	5.14	6.45	6.29	6.30	5.97	5.72	6.25	6.42	5.57	6.17	6.47	5.92	5.43
1368811_at	Lmnb1	lamin B1	0	0	0	0	0	0	uniqF+/+_163	0	0	0	1	0	4.41	4.53	4.48	4.23	4.44	4.49	4.33	4.85	4.51	4.60	4.62	4.24	4.27	4.59	4.53	4.44	4.56	4.57	3.76	3.94
1368812_at	Lmnb1	lamin B1	0	0	0	0	0	0	uniqF+/+_163	0	0	0	1	0	3.31	3.07	3.25	3.22	3.19	3.27	3.48	3.39	3.04	3.28	3.29	3.20	3.09	3.30	3.16	3.51	3.20	3.33	3.34	3.31
1368824_at	Cald1	caldesmon 1	0	0	0	0	0	0	uniqF+/+_163	0	0	0	1	0	8.13	8.04	7.96	7.90	8.32	8.06	7.95	8.21	8.23	7.93	7.89	7.89	7.71	8.04	8.01	7.78	8.33	8.05	7.93	7.82
1368866_at	Ago2	argonaute RISC catalytic component 2	0	0	0	0	0	0	uniqF+/+_163	0	0	0	1	0	5.91	5.92	5.88	5.96	6.03	5.88	5.87	5.72	5.98	5.84	5.99	5.75	5.05	5.78	5.88	5.73	5.86	6.12	5.51	5.53
1368867_at	Ago2	argonaute RISC catalytic component 2	0	0	0	0	0	0	uniqF+/+_163	0	0	0	1	0	7.17	7.28	7.29	7.35	7.27	7.21	7.23	7.24	7.29	7.11	7.42	6.99	6.16	7.08	7.36	7.03	7.27	7.38	6.83	6.97
1368944_at	Dlg1	discs, large homolog 1 (Drosophila)	0	0	0	0	0	0	uniqF+/+_163	0	0	0	1	0	7.14	6.97	7.00	6.74	7.16	6.90	6.80	6.80	7.11	6.95	6.77	6.83	6.91	6.85	6.95	6.70	7.01	6.91	6.84	6.46
1368971_a_at	Synj2	synaptojanin 2	0	0	0	0	0	0	uniqF+/+_163	0	0	0	1	0	4.25	4.24	4.24	4.12	4.38	3.98	4.08	4.11	4.02	4.03	3.94	4.25	4.27	4.15	4.10	4.07	4.42	4.18	4.15	3.76
1369164_a_at	Trpc4	transient receptor potential cation channel, subfamily C, member 4	0	0	0	0	0	0	uniqF+/+_163	0	0	0	1	0	3.98	3.82	3.70	3.66	3.84	3.84	3.90	3.60	3.88	3.97	3.79	3.49	3.86	3.74	3.66	3.57	4.04	3.90	3.61	3.45
1369182_at	F3	coagulation factor III (thromboplastin, tissue factor)	0	0	0	0	0	0	uniqF+/+_163	0	0	0	1	0	9.20	9.10	9.23	8.72	9.25	9.10	8.81	9.17	7.26	7.35	7.22	6.86	7.84	7.51	7.39	7.19	7.26	7.31	7.14	6.52
1369196_at	Ppy	pancreatic polypeptide	0	0	0	0	0	0	uniqF+/+_163	0	0	0	1	0	13.58	13.76	13.69	13.67	13.51	13.74	13.64	13.50	12.83	13.13	12.92	12.78	12.79	12.96	13.12	12.80	13.04	12.97	12.88	12.38
1369462_at	Gad2	glutamate decarboxylase 2	0	0	0	0	0	0	uniqF+/+_163	0	0	0	1	0	7.60	7.29	7.30	7.31	7.09	7.86	7.19	6.88	7.44	7.36	7.55	7.42	5.52	7.22	7.76	7.26	7.18	7.47	6.96	6.48
1369633_at	Cxcl12	chemokine (C-X-C motif) ligand 12	0	0	0	0	0	0	uniqF+/+_163	0	0	0	1	0	7.65	7.53	7.41	7.91	7.61	7.52	7.63	8.29	7.26	7.01	7.13	6.99	7.37	7.21	7.39	7.97	7.35	7.21	7.04	6.89
1369660_at	Defb1	defensin beta 1	0	0	0	0	0	0	uniqF+/+_163	0	0	0	1	0	7.30	7.33	7.33	7.38	7.29	7.24	7.30	7.14	6.86	7.03	6.67	6.88	7.31	6.95	6.85	6.91	6.93	6.56	6.59	6.18
1369768_at	Hist1h1d	histone cluster 1, H1d	0	0	0	0	0	0	uniqF+/+_163	0	0	0	1	0	5.75	5.71	5.67	5.55	5.97	5.73	5.81	5.70	5.77	5.69	5.99	5.48	5.09	5.78	5.81	5.55	5.72	5.89	5.23	5.08
1369943_at	Tgm2	transglutaminase 2, C polypeptide	0	0	0	0	0	0	uniqF+/+_163	0	0	0	1	0	8.05	7.75	7.56	8.05	8.04	7.76	7.76	8.18	7.66	7.40	7.15	7.12	7.84	7.35	7.40	7.36	7.86	7.39	7.33	7.15
1369963_at	Pafah1b3	platelet-activating factor acetylhydrolase 1b, catalytic subunit 3	0	0	0	0	0	0	uniqF+/+_163	0	0	0	1	0	6.53	6.74	6.71	6.41	6.55	6.70	6.64	6.29	7.14	7.16	6.85	6.74	7.30	7.10	7.02	6.66	7.10	6.90	6.89	6.56
1370091_at	Gnaq	guanine nucleotide binding protein (G protein), q polypeptide	0	0	0	0	0	0	uniqF+/+_163	0	0	0	1	0	5.06	5.08	4.89	4.80	5.08	4.81	4.79	4.67	5.23	4.95	4.88	5.10	4.55	5.15	5.33	4.98	5.21	4.89	4.77	4.65
1370114_a_at	Pik3r1	phosphoinositide-3-kinase, regulatory subunit 1 (alpha)	0	0	0	0	0	0	uniqF+/+_163	0	0	0	1	0	6.31	6.19	6.13	5.92	6.47	6.09	6.09	6.09	6.19	6.06	5.95	5.72	5.76	6.11	6.03	5.78	6.34	6.08	5.83	5.73
1370131_at	Cav1	caveolin 1, caveolae protein	0	0	0	0	0	0	uniqF+/+_163	0	0	0	1	0	6.59	6.33	6.27	5.92	6.54	6.20	6.24	6.55	6.24	6.15	6.09	6.07	5.34	6.28	6.22	5.87	6.41	6.24	5.79	5.50
1370218_at	Ldhb	lactate dehydrogenase B	0	0	0	0	0	0	uniqF+/+_163	0	0	0	1	0	8.90	9.04	9.02	8.76	9.21	9.09	8.95	8.80	8.78	8.68	8.59	8.34	9.02	8.41	8.56	8.39	8.76	8.70	8.56	8.18
1370256_at	Fzd1	frizzled family receptor 1	0	0	0	0	0	0	uniqF+/+_163	0	0	0	1	0	3.68	3.64	3.63	3.70	3.89	3.81	3.64	3.93	3.56	3.34	3.40	3.38	3.67	3.45	3.41	3.50	3.72	3.55	3.37	3.18
1370548_at	Slc16a10	solute carrier family 16, member 10 (aromatic amino acid transporter)	0	0	0	0	0	0	uniqF+/+_163	0	0	0	1	0	8.48	8.29	8.29	8.23	8.37	8.15	8.27	8.12	8.34	8.24	8.13	8.04	8.43	8.33	8.07	7.99	8.39	8.30	8.12	7.74
1370550_at	Lsamp	limbic system-associated membrane protein	0	0	0	0	0	0	uniqF+/+_163	0	0	0	1	0	4.84	4.47	4.38	4.52	4.66	4.69	4.63	4.55	4.57	4.55	4.60	4.40	4.65	4.57	4.32	4.49	4.91	4.56	4.51	4.40
1370570_at	Nrp1	neuropilin 1	0	0	0	0	0	0	uniqF+/+_163	0	0	0	1	0	7.61	7.55	7.43	7.16	7.19	7.69	7.21	6.90	7.42	7.24	7.60	6.83	6.20	7.51	7.63	6.94	7.55	7.61	6.51	6.23
1370717_at	Synrg	synergin, gamma	0	0	0	0	0	0	uniqF+/+_163	0	0	0	1	0	4.30	4.24	4.26	4.19	4.28	4.08	4.24	4.23	4.54	4.33	4.25	4.38	3.87	4.36	4.49	4.10	4.42	4.43	4.10	3.87
1370813_at	Gstm5 /// LO	glutathione S-transferase, mu 5 /// glutathione S-transferase Mu 5-like	0	0	0	0	0	0	uniqF+/+_163	0	0	0	1	0	7.24	7.03	7.09	6.75	7.17	6.94	7.00	6.76	7.02	7.02	6.92	6.68	7.27	6.93	6.89	6.69	7.26	6.70	6.76	6.48
1370941_at	Pdgfra	platelet derived growth factor receptor, alpha polypeptide	0	0	0	0	0	0	uniqF+/+_163	0	0	0	1	0	6.91	6.73	6.81	6.75	7.03	6.68	6.69	6.89	6.08	5.80	5.48	6.21	6.63	5.94	6.16	6.26	6.33	5.99	6.03	5.82
1370993_at	Lamc1	laminin, gamma 1	0	0	0	0	0	0	uniqF+/+_163	0	0	0	1	0	5.82	5.65	5.51	5.29	5.62	5.58	5.51	5.34	5.96	5.58	5.42	5.53	5.91	5.73	5.55	5.40	6.05	5.62	5.45	5.41
1371185_at	Itga6	integrin, alpha 6	0	0	0	0	0	0	uniqF+/+_163	0	0	0	1	0	3.79	3.86	4.00	3.86	3.85	4.03	3.99	3.88	3.81	3.96	3.88	4.02	3.90	4.00	3.87	3.88	4.00	4.02	4.16	4.11
1371186_at	Itga6	integrin, alpha 6	0	0	0	0	0	0	uniqF+/+_163	0	0	0	1	0	4.23	4.19	4.13	3.95	4.22	4.36	3.81	3.94	4.07	4.03	3.86	3.78	3.87	3.97	4.07	3.79	4.24	4.12	3.73	3.68
1371237_a_at	LOC1003627	hypothetical protein LOC100362769 /// metallothionein 1a /// transthyretin	0	0	0	0	0	0	uniqF+/+_163	0	0	0	1	0	11.36	11.87	11.51	11.22	11.46	11.85	11.79	11.13	11.42	11.89	11.54	10.71	11.40	11.66	11.63	11.15	11.26	11.49	11.19	10.43
1371322_at	Lamc1	laminin, gamma 1	0	0	0	0	0	0	uniqF+/+_163	0	0	0	1	0	8.66	8.54	8.30	8.28	8.67	8.54	8.34	8.37	8.71	8.42	8.36	8.27	8.94	8.48	8.45	8.32	8.83	8.51	8.40	8.27
1371425_at	Srrm1	serine/arginine repetitive matrix 1	0	0	0	0	0	0	uniqF+/+_163	0	0	0	1	0	9.16	9.12	9.10	9.03	9.10	9.05	9.08	9.04	9.12	9.10	9.06	9.01	9.41	9.11	8.94	8.95	9.10	9.01	9.06	8.93
1371625_at	Pygb	phosphorylase, glycogen; brain	0	0	0	0	0	0	uniqF+/+_163	0	0	0	1	0	6.42	6.32	6.27	6.31	6.49	6.19	6.33	6.35	6.06	6.22	6.10	6.01	6.34	6.09	6.16	6.05	6.26	6.13	5.90	5.73
1371792_at	LOC1003653	nuclear LIM interactor-interacting factor 2-like	0	0	0	0	0	0	uniqF+/+_163	0	0	0	1	0	10.10	10.04	9.95	9.89	10.22	10.16	9.91	9.73	10.04	9.94	9.77	9.54	10.16	9.79	9.83	9.69	10.09	9.89	9.71	9.56
1371836_at	Rab5c	RAB5C, member RAS oncogene family	0	0	0	0	0	0	uniqF+/+_163	0	0	0	1	0	8.16	8.12	8.50	7.75	8.31	8.49	8.54	7.93	8.27	8.31	8.40	8.42	8.26	8.47	8.41	8.11	8.36	8.27	7.83	7.90
1371838_at	Srsf2	serine/arginine-rich splicing factor 2	0	0	0	0	0	0	uniqF+/+_163	0	0	0	1	0	10.46	10.28	10.16	10.35	10.51	10.33	10.26	10.34	10.53	10.27	10.22	10.09	10.62	10.19	10.08	10.17	10.48	10.21	10.18	10.08
1371839_at	Srsf2	serine/arginine-rich splicing factor 2	0	0	0	0	0	0	uniqF+/+_163	0	0	0	1	0	8.74	8.53	8.42	8.60	8.82	8.50	8.51	8.70	8.81	8.58	8.59	8.43	8.68	8.44	8.43	8.46	8.76	8.48	8.35	8.15
1371884_at	Ttc3	tetratricopeptide repeat domain 3	0	0	0	0	0	0	uniqF+/+_163	0	0	0	1	0	10.20	10.18	10.14	10.05	10.21	10.14	10.07	9.94	10.26	10.16	10.13	10.00	10.34	10.03	10.10	10.03	10.28	10.09	10.16	10.01
1371913_at	Tgfbi	transforming growth factor, beta induced	0	0	0	0	0	0	uniqF+/+_163	0	0	0	1	0	7.48	7.33	7.30	8.02	7.56	7.23	7.27	7.77	7.94	7.61	7.71	7.66	8.36	7.73	7.89	7.84	8.13	7.49	7.50	7.25
1372111_at	Cav1	caveolin 1, caveolae protein	0	0	0	0	0	0	uniqF+/+_163	0	0	0	1	0	9.09	8.89	8.71	8.63	9.08	8.83	8.78	9.03	8.79	8.71	8.56	8.54	8.94	8.77	8.65	8.51	8.90	8.85	8.71	8.53

Affymetrix Probeset ID	Gene Symbol	Gene Name	unique BB on P7 n=822	Figure 1B P7 Venn n=1526	DRlyp/lyp STEM P7 n=712	DR+/+ STEM P7 n=936	Flyp/lyp STEM P7 n=520	F+/+ STEM P7 n=465	Figure 1B P0 Venn n=1071	DRlyp/lyp STEM P0 n=442	DR+/+ STEM P0 n=437	Flyp/lyp STEM P0 n=643	F+/+ STEM P0 n=632	Fig1D n=25	Mean log2 intensity DRlyp/lyp 20	Mean log2 intensity DRlyp/lyp 30	Mean log2 intensity DRlyp/lyp 40	Mean log2 intensity DRlyp/lyp 50	Mean log2 intensity DR+/+ 20	Mean log2 intensity DR+/+ 30	Mean log2 intensity DR+/+ 40	Mean log2 intensity DR+/+ 50	Mean log2 intensity Flyp/lyp 20	Mean log2 intensity Flyp/lyp 30	Mean log2 intensity Flyp/lyp 40	Mean log2 intensity Flyp/lyp 50	Mean log2 intensity Flyp20	Mean log2 intensity Flyp30	Mean log2 intensity Flyp40	Mean log2 intensity Flyp50	Mean log2 intensity F+/+ 20	Mean log2 intensity F+/+ 30	Mean log2 intensity F+/+ 40	Mean log2 intensity F+/+ 50
1372423_at	Perp	PERP, TP53 apoptosis effector	0	0	0	0	0	0	uniqF+/+_163	0	0	0	1	0	8.18	8.35	8.34	8.06	8.31	8.40	8.28	8.08	7.91	7.96	7.57	7.39	8.03	7.82	7.85	7.53	7.86	7.82	7.62	7.37
1372443_at	Lrp11	low density lipoprotein receptor-related protein 11	0	0	0	0	0	0	uniqF+/+_163	0	0	0	1	0	11.16	11.09	11.20	11.18	11.22	11.14	11.13	11.09	11.14	11.11	11.13	11.26	11.41	11.00	11.19	11.16	11.14	11.08	11.22	11.23
1372599_at	Mgst2	microsomal glutathione S-transferase 2	0	0	0	0	0	0	uniqF+/+_163	0	0	0	1	0	6.69	6.92	6.74	6.91	6.77	6.71	6.96	7.05	6.90	6.82	6.71	6.62	7.30	7.01	6.80	6.74	7.09	6.62	6.58	6.67
1372778_at	Slc39a1	solute carrier family 39 (zinc transporter), member 1	0	0	0	0	0	0	uniqF+/+_163	0	0	0	1	0	7.47	7.63	7.37	7.89	7.45	7.73	7.70	7.82	7.58	7.64	7.58	7.77	7.48	7.70	7.53	7.73	7.73	7.68	7.40	7.13
1373149_at	Ypel3	yippee-like 3 (Drosophila)	0	0	0	0	0	0	uniqF+/+_163	0	0	0	1	0	8.99	9.05	8.92	8.77	9.07	9.12	8.90	8.74	9.10	9.10	8.91	8.60	8.98	9.00	8.90	8.67	8.98	8.99	8.83	8.46
1373745_at	Gtf2h4	general transcription factor II H, polypeptide 4	0	0	0	0	0	0	uniqF+/+_163	0	0	0	1	0	6.99	7.08	7.07	7.00	7.05	7.00	6.97	6.93	6.84	6.96	6.86	6.83	6.70	6.66	6.69	6.54	6.97	6.83	6.46	6.63
1373755_at	Lrrc39	leucine rich repeat containing 39	0	0	0	0	0	0	uniqF+/+_163	0	0	0	1	0	7.23	7.26	7.23	7.17	7.04	6.90	7.10	7.01	7.67	7.56	7.23	7.17	8.18	7.59	7.53	7.25	7.73	7.15	7.14	6.94
1373897_at	---	---	0	0	0	0	0	0	uniqF+/+_163	0	0	0	1	0	7.53	7.60	7.62	7.39	7.52	7.76	7.54	7.95	7.26	7.59	7.49	7.22	7.24	7.28	7.45	7.42	7.30	7.31	6.78	6.73
1374017_at	Trim72	tripartite motif-containing 72	0	0	0	0	0	0	uniqF+/+_163	0	0	0	1	0	6.17	6.26	6.43	6.53	6.22	6.04	6.35	6.44	6.66	6.45	6.53	6.91	7.68	6.61	6.51	6.78	7.07	6.64	6.68	6.56
1374111_at	Pnn	pinin, desmosome associated protein	0	0	0	0	0	0	uniqF+/+_163	0	0	0	1	0	8.34	8.28	8.23	8.14	7.95	8.21	8.21	7.96	7.93	7.99	7.92	7.85	7.99	7.88	7.82	7.96	7.95	7.89	7.58	7.73
1374124_at	Jmjd8	jumonji domain containing 8	0	0	0	0	0	0	uniqF+/+_163	0	0	0	1	0	7.95	7.87	7.76	7.61	7.95	7.76	7.68	7.55	7.96	7.78	7.69	7.68	7.89	7.83	7.70	7.59	8.00	7.72	7.54	7.49
1374635_at	---	---	0	0	0	0	0	0	uniqF+/+_163	0	0	0	1	0	5.18	4.79	4.74	4.76	5.17	4.76	4.83	4.76	5.77	5.62	5.04	4.91	6.33	5.22	5.04	4.78	6.03	5.07	5.09	4.56
1374722_at	C1qtnf1	C1q and tumor necrosis factor related protein 1	0	0	0	0	0	0	uniqF+/+_163	0	0	0	1	0	4.87	4.77	4.84	4.78	4.93	4.88	4.81	4.80	5.04	4.88	4.84	4.72	4.98	5.01	4.79	4.76	5.32	4.90	4.86	4.73
1374863_at	Rbp7	retinol binding protein 7, cellular	0	0	0	0	0	0	uniqF+/+_163	0	0	0	1	0	4.46	4.29	4.57	4.19	4.23	4.14	4.19	4.37	4.62	4.46	4.32	4.29	5.16	4.39	4.37	4.41	4.68	3.87	3.99	3.89
1375030_at	B3galt5	UDP-Gal:betaGlcNAc beta 1,3-galactosyltransferase, polypeptide 5	0	0	0	0	0	0	uniqF+/+_163	0	0	0	1	0	3.13	3.27	3.13	3.35	3.07	3.22	3.42	3.33	3.91	3.51	3.58	3.63	4.02	3.75	3.60	3.82	4.11	3.69	3.66	3.50
1375193_at	Lrp11	low density lipoprotein receptor-related protein 11	0	0	0	0	0	0	uniqF+/+_163	0	0	0	1	0	6.15	5.99	5.95	5.97	6.02	6.04	5.93	5.93	5.93	5.75	6.20	5.99	4.95	5.91	6.09	6.02	6.06	6.05	5.38	5.23
1375533_at	Vgll4	vestigial like 4 (Drosophila)	0	0	0	0	0	0	uniqF+/+_163	0	0	0	1	0	6.32	6.24	6.13	6.06	6.32	6.13	6.08	6.01	6.08	5.98	5.89	5.82	6.36	5.88	5.82	5.73	6.15	5.81	5.77	5.61
1375921_at	Rcn1	reticulocalbin 1, EF-hand calcium binding domain	0	0	0	0	0	0	uniqF+/+_163	0	0	0	1	0	4.94	4.99	5.05	4.83	5.00	5.10	4.76	4.84	5.17	5.00	4.78	4.45	5.39	4.94	4.90	4.59	5.08	4.79	4.69	4.52
1377660_at	LOC1009110	5-hydroxyisourate hydrolase-like /// similar to transthyretin (4L369)	0	0	0	0	0	0	uniqF+/+_163	0	0	0	1	0	5.89	5.62	5.62	5.71	5.83	5.82	5.87	5.82	6.36	6.33	5.88	5.95	6.15	6.02	6.25	5.71	6.69	6.43	6.23	6.02
1377833_at	Rif1	RAP1 interacting factor homolog (yeast)	0	0	0	0	0	0	uniqF+/+_163	0	0	0	1	0	7.43	7.24	7.44	7.33	7.48	7.22	7.30	7.28	7.30	7.28	7.21	7.14	7.45	7.12	7.24	7.11	7.25	7.10	7.20	7.17
1377914_at	Srrm1	serine/arginine repetitive matrix 1	0	0	0	0	0	0	uniqF+/+_163	0	0	0	1	0	8.47	8.41	8.29	8.41	8.56	8.32	8.21	8.38	8.51	8.34	8.34	8.18	8.14	8.21	8.27	8.25	8.53	8.34	8.15	7.96
1378112_at	Kat6a	K(lysine) acetyltransferase 6A	0	0	0	0	0	0	uniqF+/+_163	0	0	0	1	0	8.50	8.41	8.38	8.42	8.51	8.46	8.39	8.28	8.43	8.39	8.30	8.15	8.66	8.38	8.29	8.22	8.39	8.34	8.32	8.24
1378484_at	Rasl12	RAS-like, family 12	0	0	0	0	0	0	uniqF+/+_163	0	0	0	1	0	6.02	5.84	5.58	5.60	6.08	5.83	5.80	5.84	5.82	5.46	5.37	5.44	6.19	5.57	5.72	5.48	5.91	5.51	5.39	5.49
1379108_at	---	---	0	0	0	0	0	0	uniqF+/+_163	0	0	0	1	0	5.17	5.13	5.22	4.98	5.16	5.07	4.89	4.84	4.64	4.69	4.63	4.22	4.71	4.54	4.49	4.36	4.77	4.39	4.47	4.16
1379527_at	Epc1	enhancer of polycomb homolog 1 (Drosophila)	0	0	0	0	0	0	uniqF+/+_163	0	0	0	1	0	6.59	6.61	6.47	6.40	6.74	6.56	6.63	6.59	6.48	6.48	6.42	6.19	6.15	6.45	6.40	6.26	6.55	6.28	6.20	6.04
1379603_at	Cbl	Cbl proto-oncogene, E3 ubiquitin protein ligase	0	0	0	0	0	0	uniqF+/+_163	0	0	0	1	0	7.51	7.65	7.52	7.73	7.53	7.34	7.61	7.59	7.60	7.43	7.58	7.18	7.50	7.43	7.43	7.46	7.73	7.48	7.51	7.30
1379604_at	Apol3	apolipoprotein L, 3	0	0	0	0	0	0	uniqF+/+_163	0	0	0	1	0	5.75	5.60	5.47	6.13	5.75	5.70	5.76	6.19	5.44	5.46	5.19	5.12	5.58	5.31	5.23	5.39	5.59	5.42	5.08	5.21
1380001_at	Pnn	pinin, desmosome associated protein	0	0	0	0	0	0	uniqF+/+_163	0	0	0	1	0	7.60	7.44	7.48	7.12	7.36	7.63	7.33	7.06	7.32	7.17	7.29	7.23	5.67	7.06	7.12	7.21	7.19	7.22	6.14	6.28
1380069_at	---	---	0	0	0	0	0	0	uniqF+/+_163	0	0	0	1	0	7.07	7.09	6.94	7.02	7.14	6.72	6.95	6.94	7.25	6.75	6.83	6.82	6.54	6.78	6.83	6.91	7.24	6.83	6.86	6.55
1380577_at	Abcg2	ATP-binding cassette, subfamily G (WHITE), member 2	0	0	0	0	0	0	uniqF+/+_163	0	0	0	1	0	3.20	3.26	2.85	2.99	3.21	3.28	3.14	3.21	3.05	3.00	3.00	3.31	3.15	3.24	3.10	3.05	3.89	3.49	3.26	3.40
1380733_at	Qrich1	glutamine-rich 1	0	0	0	0	0	0	uniqF+/+_163	0	0	0	1	0	7.79	8.07	8.06	8.18	8.30	7.95	8.04	7.94	8.26	8.08	8.15	7.91	7.33	7.97	8.09	8.10	8.18	8.12	7.53	7.32
1381543_at	Slc38a7	solute carrier family 38, member 7	0	0	0	0	0	0	uniqF+/+_163	0	0	0	1	0	6.30	6.37	6.23	6.40	6.18	6.57	6.38	6.15	6.42	6.45	6.49	6.09	6.07	6.37	6.54	6.23	6.34	6.57	5.81	5.70
1382059_at	Fbxo30	F-box protein 30	0	0	0	0	0	0	uniqF+/+_163	0	0	0	1	0	5.83	5.73	5.71	5.46	5.72	5.56	5.53	5.52	6.00	5.96	5.72	5.61	6.25	5.83	5.61	5.67	6.11	5.85	5.51	5.68
1382558_at	Tcf7l1	transcription factor 7-like 1 (T-cell specific, HMG-box)	0	0	0	0	0	0	uniqF+/+_163	0	0	0	1	0	4.64	4.43	4.28	4.24	4.65	4.52	4.47	4.38	4.49	4.25	4.06	4.09	4.82	4.37	4.26	3.96	4.77	4.25	4.21	4.08
1382584_at	LOC681371	hypothetical protein LOC681371	0	0	0	0	0	0	uniqF+/+_163	0	0	0	1	0	6.97	6.67	6.64	6.94	6.81	6.65	6.54	6.63	7.12	6.89	6.71	6.74	5.75	6.68	6.65	6.84	7.08	6.54	6.09	6.25
1382767_at	Rcn1	reticulocalbin 1, EF-hand calcium binding domain	0	0	0	0	0	0	uniqF+/+_163	0	0	0	1	0	4.46	4.46	4.26	4.07	4.34	4.18	4.12	4.34	4.44	4.38	4.51	4.49	4.56	4.23	4.26	4.30	4.58	4.55	4.71	4.46
1382859_at	Rif1	RAP1 interacting factor homolog (yeast)	0	0	0	0	0	0	uniqF+/+_163	0	0	0	1	0	4.31	4.23	4.32	3.93	4.52	4.13	4.05	4.25	4.47	4.27	4.38	4.35	3.65	4.06	4.44	4.14	4.76	4.15	3.93	4.12
1383336_at	Pnn	pinin, desmosome associated protein	0	0	0	0	0	0	uniqF+/+_163	0	0	0	1	0	8.46	8.38	8.34	8.41	8.15	8.25	8.27	8.28	8.31	8.31	8.15	8.22	8.32	8.16	8.14	8.43	8.35	8.22	8.08	8.14
1383382_at	LOC681740	similar to jumonji protein	0	0	0	0	0	0	uniqF+/+_163	0	0	0	1	0	8.02	7.92	7.95	7.72	8.16	7.98	7.80	7.69	8.26	8.06	8.03	7.86	8.24	8.08	7.88	7.91	8.24	8.06	7.95	7.87
1383391_a_at	C2 /// Cfb	complement component 2 /// complement factor B	0	0	0	0	0	0	uniqF+/+_163	0	0	0	1	0	5.88	5.54	5.57	6.51	5.58	5.44	5.47	5.42	5.99	5.79	5.61	5.37	5.80	5.36	5.38	5.29	5.96	5.39	5.45	5.37
1383392_at	C2 /// Cfb	complement component 2 /// complement factor B	0	0	0	0	0	0	uniqF+/+_163	0	0	0	1	0	5.93	5.78	5.68	6.40	5.90	5.85	5.77	5.79	5.97	5.98	5.74	5.78	5.79	5.68	5.70	5.69	5.86	5.71	5.67	5.64
1384069_at	Lrp11	low density lipoprotein receptor-related protein 11	0	0	0	0	0	0	uniqF+/+_163	0	0	0	1	0	8.21	8.07	8.02	8.11	8.12	7.94	8.02	8.14	8.16	8.07	8.08	8.18	7.90	8.03	8.18	8.22	8.17	8.05	7.94	7.71
1384390_at	LOC681740	similar to jumonji protein	0	0	0	0	0	0	uniqF+/+_163	0	0	0	1	0	6.79	6.58	6.43	6.49	6.74	6.49	6.54	6.50	7.04	6.85	6.73	6.66	7.00	6.82	6.69	6.68	7.09	6.75	6.83	6.52
1384707_at	Scara5	scavenger receptor class A, member 5 (putative)	0	0	0	0	0	0	uniqF+/+_163	0	0	0	1	0	4.83	4.33	4.49	4.30	4.77	4.24	4.38	4.32	4.34	4.29	4.02	4.07	4.96	4.15	4.33	4.44	4.84	4.13	4.09	4.18
1384742_at	Atrx	alpha thalassemia/mental retardation syndrome X-linked	0	0	0	0	0	0	uniqF+/+_163	0	0	0	1	0	4.39	4.25	4.51	4.31	4.51	4.42	4.33	4.68	4.36	4.22	4.43	4.19	4.58	4.38	4.19	4.25	4.56	4.38	4.56	4.20
1384763_at	RGD1306626	similar to RIKEN cDNA 4930500J03	0	0	0	0	0	0	uniqF+/+_163	0	0	0	1	0	3.38	3.37	3.23	2.88	3.26	3.17	2.91	2.94	3.56	3.57	3.35	3.14	3.78	3.49	2.99	2.90	3.67	3.17	3.34	2.90
1384885_at	Tekt2	tektin 2 (testicular)	0	0	0	0	0	0	uniqF+/+_163	0	0	0	1	0	5.58	5.59	5.45	5.29	5.62	5.52	5.48	5.18	5.40	5.66	5.45	5.12	5.64	5.41	5.38	4.93	5.52	5.30	5.23	4.75
1385209_at	Kat6a	K(lysine) acetyltransferase 6A	0	0	0	0	0	0	uniqF+/+_163	0	0	0	1	0	5.59	5.63	5.29	5.22	5.66	5.69	5.33	5.49	5.63	5.47	5.72	5.38	4.70	5.42	5.52	5.24	5.67	5.57	4.89	4.86
1386870_at	Glul	glutamate-ammonia ligase	0	0	0	0	0	0	uniqF+/+_163	0	0	0	1	0	9.39	9.31	9.14	8.98	9.23	9.20	9.30	8.96	9.90	9.79	9.68	9.41	9.16	9.86	9.61	9.41	9.91	9.66	9.20	9.00
1386914_at	Gmpr	guanosine monophosphate reductase	0	0	0	0	0	0	uniqF+/+_163	0	0	0	1	0	7.04	7.13	7.17	6.76	7.09	7.05	7.00	6.78	6.71	6.60	6.57	6.24	6.75	6.74	6.59	6.08	6.82	6.45	6.45	6.30
1386998_at	Aldoc	aldolase C, fructose-bisphosphate	0	0	0	0	0	0	uniqF+/+_163	0	0	0	1	0	6.38	6.30	6.32	6.24	6.49	6.47	6.29	6.20	6.51	6.61	6.27	6.12	6.61	6.18	6.17	6.04	6.48	6.08	6.01	5.95
1387095_at	Gnaz	guanine nucleotide binding protein (G protein), alpha z polypeptide	0	0	0	0	0	0	uniqF+/+_163	0	0	0	1	0	6.19	6.05	5.99	5.85	5.96	5.89	6.02	5.73	6.09	6.05	6.06	6.01	5.91	6.10	6.10	5.86	6.29	6.13	5.97	5.76
1387106_at	Sh3bp4	SH3-domain binding protein 4	0	0	0	0	0	0	uniqF+/+_163	0	0	0	1	0	3.83	3.83	3.73	3.68	3.97	3.75	3.75	3.67	4.17	4.00	3.90	3.87	4.53	3.95	3.93	3.87	4.42	3.93	4.03	3.63
1387135_at	Adam15	ADAM metallopeptidase domain 15	0	0	0	0	0	0	uniqF+/+_163	0	0	0	1	0	8.01	7.99	7.91	7.68	7.95	7.90	7.83	7.59	7.98	7.93	7.70	7.48	7.95	7.90	7.84	7.52	8.06	7.83	7.67	7.45
1387498_a_at	Fgfr1	Fibroblast growth factor receptor 1	0	0	0	0	0	0	uniqF+/+_163	0	0	0	1	0	5.58	5.40	5.31	5.31	5.55	5.53	5.30	5.10	5.37	5.20	5.12	5.14	5.34	5.28	5.08	5.12	5.36	5.23	5.27	4.73
1387655_at	Cxcl12	chemokine (C-X-C motif) ligand 12	0	0	0	0	0	0	uniqF+/+_163	0	0	0	1	0	4.71	4.35	4.41	4.40	5.66	4.38	4.34	4.81	4.63	4.23	4.40	4.37	4.28	4.04	4.38	4.50	4.62	4.18	4.19	3.90
1387776_at	Tgm2	transglutaminase 2, C polypeptide	0	0	0	0	0	0	uniqF+/+_163	0	0	0	1	0	6.03	5.93	5.66	5.90	6.23	5.87	6.01	6.12	5.94	5.70	5.81	5.77	5.53	5.92	5.86	5.66	6.09	5.87	5.72	5.56
1387788_at	Junb	jun B proto-oncogene	0	0	0	0	0	0	uniqF+/+_163	0	0	0	1	0	9.10	9.25	9.08	8.83	9.27	9.24	9.57	8.97	9.08	9.20	9.20	8.70	8.75	8.79	8.93	8.79	8.97	8.94	8.90	8.38
1388122_at	Gstp1	glutathione S-transferase pi 1	0	0	0	0	0	0	uniqF+/+_163	0	0	0	1	0	9.31	9.34	9.38	9.17	9.39	9.38	9.36	9.42	9.19	9.18	8.94	8.92	9.46	9.21	9.15	8.90	9.20	9.00	8.89	8.68
1388267_a_at	Mt1a	metallothionein 1a	0	0	0	0	0	0	uniqF+/+_163	0	0	0	1	0	5.70	5.88	5.71	5.71	5.72	5.96	5.66	5.46	5.86	6.02	5.78	5.45	5.80	5.92	5.87	5.64	5.64	5.86	5.67	5.31
1388430_at	Ptov1	prostate tumor overexpressed 1	0	0	0	0	0	0	uniqF+/+_163	0	0	0	1	0	7.71	7.54	7.43	7.39	7.62	7.49	7.46	7.27	7.76	7.56	7.45	7.34	7.92	7.51	7.39	7.35	7.77	7.58	7.62	7.23
1388583_at	Cxcl12	chemokine (C-X-C motif) ligand 12	0	0	0	0	0	0	uniqF+/+_163	0	0	0	1	0	9.98	9.22	8.98	8.78	10.21	9.17	8.79	8.82	9.89	9.17	9.07	8.90	10.02	9.34	9.19	8.72	9.91	9.58	9.29	8.91
1388827_at	LOC685069 /	similar to H2A histone family, member V isoform 1 /// similar to H2A histone family, membe	0	0	0	0	0	0	uniqF+/+_163	0	0	0	1	0	9.83	9.76	9.61	9.67	9.88	9.80	9.60	9.81	9.84	9.79	9.70	9.39	9.76	9.69	9.69	9.51	9.79	9.70	9.51	9.29
1389177_at	Perp	PERP, TP53 apoptosis effector	0	0	0	0	0	0	uniqF+/+_163	0	0	0	1	0	7.95	8.16	8.13	7.83	8.14	8.18	8.09	7.85	7.73	7.71	7.44	7.32	7.56	7.67	7.69	7.35	7.70	7.58	7.33	7.08
1389354_at	Gipc2	GIPC PDZ domain containing family, member 2	0	0	0	0	0	0	uniqF+/+_163	0	0	0	1	0	6.28	6.16	6.11	6.13	6.34	6.34	6.29	6.02	6.13	6.17	5.96	5.78	6.19	6.04	6.05	5.68	6.21	6.13	5.93	5.68
1389787_at	Ptk7	protein tyrosine kinase 7	0	0	0	0	0	0	uniqF+/+_163	0	0	0	1	0	6.60	6.53	6.56	6.21	6.55	6.72	6.64	6.25	6.60	6.64	6.59	6.43	7.02	6.72	6.63	6.57	6.80	6.56	6.18	6.15
1389868_at	Ddx6	DEAD (Asp-Glu-Ala-Asp) box helicase 6	0	0	0	0	0	0	uniqF+/+_163	0	0	0	1	0	10.70	10.68	10.49	10.67	10.89	10.52	10.47	10.55	10.79	10.59	10.53	10.35	10.14	10.32	10.56	10.47	10.67	10.53	10.46	10.38
1389957_at	Ttc3	tetratricopeptide repeat domain 3	0	0	0	0	0	0	uniqF+/+_163	0	0	0	1	0	10.17	10.04	9.95	9.86	10.11	10.07	9.85	9.64	10.17	10.03	9.82	9.74	9.87	9.93	9.88	9.74	10.19	9.97	9.86	9.60
1389984_at	LOC681740	similar to jumonji protein	0	0	0	0	0	0	uniqF+/+_163	0	0	0	1	0	8.53	8.41	8.30	8.25	8.64	8.40	8.23	8.18	8.86	8.63	8.56	8.38	8.94	8.57	8.46	8.34	8.89	8.63	8.56	8.33
1389995_at	Srrm1	serine/arginine repetitive matrix 1	0	0	0	0	0	0	uniqF+/+_163	0	0	0	1	0	6.55	6.40	6.29	6.07	6.37	6.48	6.14	6.06	6.66	6.26	6.31	6.14	6.14	6.41	6.22	6.18	6.51	6.47	5.90	5.91
1390283_at	Tcf7l1	transcription factor 7-like 1 (T-cell specific, HMG-box)	0	0	0	0	0	0	uniqF+/+_163	0	0	0	1	0	5.30	5.06	5.14	5.01	5.19	5.20	5.19	4.99	5.34	5.00	5.10	5.14	5.54	5.19	5.10	4.91	5.42	5.31	5.18	5.00
1390445_at	LOC688133	similar to mbt domain containing 1	0	0	0	0	0	0	uniqF+/+_163	0	0	0	1	0	8.57	8.51	8.41	8.25	8.66	8.46	8.36	8.20	8.60	8.46	8.35	8.09	8.70	8.35	8.30	8.14	8.62	8.35	8.28	8.07
1390937_at	Ppp1r36	protein phosphatase 1, regulatory subunit 36	0	0	0	0	0	0	uniqF+/+_163	0	0	0	1	0	7.23	7.33	7.30	7.04	7.33	7.32	7.17	7.10	6.93	6.91	6.71	6.50	7.00	6.75	6.56	6.46	6.96	6.63	6.55	6.36
1391068_at	Sfta2	surfactant associated 2	0	0	0	0	0	0	uniqF+/+_163	0	0	0	1	0	2.60	2.58	2.59	2.58	2.53	2.53	2.78	2.58	2.61	2.66	2.38	2.64	2.74	2.40	2.44	2.57	2.94	2.46	2.46	2.42
1391701_at	Kat6a	K(lysine) acetyltransferase 6A	0	0	0	0	0	0	uniqF+/+_163	0	0	0	1	0	6.89	6.80	6.58	6.40	6.50	6.84	6.63	6.06	6.99	6.62	6.72	6.24	6.62	6.75	6.62	6.20	6.82	6.78	6.22	6.22
1392204_at	Zfp90	zinc finger protein 90	0	0	0	0	0	0	uniqF+/+_163	0	0	0	1	0	6.85	6.80	6.72	6.54	6.87	6.91	6.60	6.44	6.77	6.70	6.57	6.36	6.94	6.38	6.69	6.41	6.84	6.48	6.41	6.32
1392854_at	---	---	0	0	0	0	0	0	uniqF+/+_163	0	0	0	1	0	6.33	6.11	5.64	5.43	5.59	6.75	6.25	5.10	7.04	5.57	6.36	5.50	4.98	5.73	5.76	5.25	5.85	5.75	5.23	4.96
1393198_at	Pcmtd1	protein-L-isoaspartate (D-aspartate) O-methyltransferase domain containing 1	0	0	0	0	0	0	uniqF+/+_163	0	0	0	1	0	5.98	5.92	5.74	5.69	5.86	5.46	5.46	5.83	5.76	5.79	5.88	5.48	5.15	5.79	5.94	5.65	5.86	5.78	5.23	5.33
1393219_at	C2 /// Cfb	complement component 2 /// complement factor B	0	0	0	0	0	0	uniqF+/+_163	0	0	0	1	0	5.15	5.04	4.85	5.68	5.12	5.07	5.01	4.81	5.09	5.17	4.76	4.81	4.85	4.69	4.69	4.72	5.04	4.80	4.51	4.63
1393281_at	Cav1	caveolin 1, caveolae protein	0	0	0	0	0	0	uniqF+/+_163	0	0	0	1	0	7.32	7.16	7.06	6.88	7.35	7.11	7.13	7.22	7.10	6.98	6.96	6.96	6.95	7.04	6.98	6.78	7.30	7.05	6.90	6.64
1393558_at	Itga6	integrin, alpha 6	0	0	0	0	0	0	uniqF+/+_163	0	0	0	1	0	4.55	4.32	4.00	3.92	4.41	4.19	4.00	4.10	4.24	3.92	3.90	3.70	4.06	4.28	4.15	3.71	4.47	4.02	3.60	3.81
1393643_at	Rcn1	reticulocalbin 1, EF-hand calcium binding domain	0	0	0	0	0	0	uniqF+/+_163	0	0	0	1	0	7.51	7.60	7.54	7.51	7.57	7.47	7.30	7.54	7.41	7.43	7.07	6.94	7.57	7.42	7.35	7.08	7.43	7.29	7.02	6.83
1393798_at	Atrx	alpha thalassemia/mental retardation syndrome X-linked	0	0	0	0	0	0	uniqF+/+_163	0	0	0	1	0	7.04	6.90	6.69	6.61	6.88	6.78	6.59	6.53	7.14	6.90	6.91	6.61	6.02	6.70	6.91	6.42	7.06	6.95	6.42	6.33
1393813_at	Ttc3	tetratricopeptide repeat domain 3	0	0	0	0	0	0	uniqF+/+_163	0	0	0	1	0	8.29	7.90	7.87	7.76	7.87	7.91	7.60	7.60	8.30	8.06	7.90	8.00	7.44	8.01	7.85	7.81	8.29	7.96	7.84	7.68
1394935_at	Wasf2	WAS protein family, member 2	0	0	0	0	0	0	uniqF+/+_163	0	0	0	1	0	7.08	6.99	6.68	6.86	6.97	6.77	6.59	6.93	7.14	6.72	6.76	6.67	6.52	6.70	6.67	6.65	7.06	6.67	6.54	6.59
1395221_at	Snx13	sorting nexin 13	0	0	0	0	0	0	uniqF+/+_163	0	0	0	1	0	3.22	3.44	3.67	3.41	3.62	3.37	3.47	3.43	4.13	3.97	4.03	3.81	3.91	3.71	4.03	3.89	4.29	3.99	3.61	3.59
1395264_at	Rif1	RAP1 interacting factor homolog (yeast)	0	0	0	0	0	0	uniqF+/+_163	0	0	0	1	0	6.92	6.76	6.51	6.42	6.88	6.33	6.22	6.52	6.70	6.57	6.59	6.46	5.95	6.26	6.68	6.41	6.83	6.39	6.25	6.38
1395418_at	Zfp131	zinc finger protein 131	0	0	0	0	0	0	uniqF+/+_163	0	0	0	1	0	6.26	6.29	6.30	6.08	6.41	6.06	6.23	6.12	6.41	6.21	6.23	6.05	5.89	6.28	6.31	6.11	6.58	6.19	6.07	5.94
1396105_at	LOC688133	similar to mbt domain containing 1	0	0	0	0	0	0	uniqF+/+_163	0	0	0	1	0	6.95	6.97	6.88	6.77	7.01	6.85	6.77	6.78	7.06	6.96	6.85	6.66	7.13	6.78	6.90	6.65	7.04	6.75	6.73	6.52
1396140_at	Lrrc39	leucine rich repeat containing 39	0	0	0	0	0	0	uniqF+/+_163	0	0	0	1	0	5.95	6.06	6.05	5.86	5.82	5.72	5.98	5.71	6.53	6.44	6.20	6.14	7.04	6.50	6.34	6.18	6.53	6.11	6.08	5.76
1396463_at	Pnn	pinin, desmosome associated protein	0	0	0	0	0	0	uniqF+/+_163	0	0	0	1	0	6.54	6.42	6.48	6.09	6.26	6.52	6.22	6.23	6.38	6.26	6.26	6.14	5.42	6.06	6.03	6.24	6.36	6.23	5.52	5.68
1396737_at	Smchd1	structural maintenance of chromosomes flexible hinge domain containing 1	0	0	0	0	0	0	uniqF+/+_163	0	0	0	1	0	4.10	3.88	3.89	3.82	3.76	3.80	3.55	4.04	3.88	4.02	4.05	3.97	3.16	4.25	4.02	3.84	4.31	4.14	3.62	3.23
1397172_at	Ago3	argonaute RISC catalytic component 3	0	0	0	0	0	0	uniqF+/+_163	0	0	0	1	0	2.84	2.85	2.91	2.83	2.81	2.90	2.82	2.72	3.90	3.46	3.43	3.59	2.82	3.23	3.51	3.48	3.76	3.48	3.13	2.97
1397367_at	---	---	0	0	0	0	0	0	uniqF+/+_163	0	0	0	1	0	2.79	2.81	2.91	2.95	2.94	2.93	3.04	3.00	2.95	2.89	2.76	3.16	2.86	3.01	2.73	2.74	2.82	2.80	2.91	2.95
1397523_at	Lrp11	low density lipoprotein receptor-related protein 11	0	0	0	0	0	0	uniqF+/+_163	0	0	0	1	0	8.21	8.11	7.97	8.12	8.14	7.99	7.88	8.07	8.00	7.91	8.04	8.07	7.01	7.92	8.14	8.04	8.12	8.06	7.59	7.20
1397607_at	Crebrf	CREB3 regulatory factor	0	0	0	0	0	0	uniqF+/+_163	0	0	0	1	0	4.74	4.68	4.61	4.58	4.68	4.65	4.49	4.47	4.70	4.47	4.78	4.71	3.86	4.75	4.76	4.45	4.60	4.69	4.09	4.18
1397675_at	Eif4h	eukaryotic translation initiation factor 4H	0	0	0	0	0	0	uniqF+/+_163	0	0	0	1	0	5.86	5.93	5.88	5.57	5.88	5.85	5.73	5.57	5.89	5.83	5.76	5.51	4.83	5.88	5.81	5.68	5.99	5.68	5.19	5.21
1398134_at	Zfp90	zinc finger protein 90	0	0	0	0	0	0	uniqF+/+_163	0	0	0	1	0	3.86	3.76	3.81	3.31	3.90	3.70	3.82	3.59	3.77	3.85	3.47	3.50	3.46	3.36	3.66	3.26	3.84	3.50	3.37	3.31
1398719_at	Cdyl2	chromodomain protein, Y-like 2	0	0	0	0	0	0	uniqF+/+_163	0	0	0	1	0	5.56	5.51	5.54	5.50	5.55	5.47	5.45	5.51	5.58	5.61	5.73	5.30	5.85	5.53	5.46	5.24	5.75	5.50	5.48	5.15
1399099_at	Hnrnpul1	heterogeneous nuclear ribonucleoprotein U-like 1	0	0	0	0	0	0	uniqF+/+_163	0	0	0	1	0	6.53	6.46	6.35	6.29	6.48	6.48	6.32	6.18	6.49	6.11	6.06	6.07	6.33	6.19	6.01	6.07	6.31	6.22	5.89	5.77
1367540_at	Zfyve26	zinc finger, FYVE domain containing 26	0	0	0	0	0	0	uniqFlyp/lyp_145	0	0	1	0	0	7.11	7.08	6.96	6.99	7.16	7.00	6.93	6.93	7.31	7.11	6.99	6.76	7.38	6.94	7.12	6.93	7.17	7.00	7.04	6.96
1367798_at	Ahcy	adenosylhomocysteinase	0	0	0	0	0	0	uniqFlyp/lyp_145	0	0	1	0	0	8.83	8.64	8.45	8.48	8.74	8.40	8.56	8.54	9.19	9.03	8.72	8.48	9.25	9.10	8.58	8.56	9.00	8.78	8.62	8.76
1368240_a_at	Prkcb	protein kinase C, beta	0	0	0	0	0	0	uniqFlyp/lyp_145	0	0	1	0	0	5.21	4.96	4.68	4.85	5.23	4.83	4.70	5.39	5.70	5.38	5.28	4.97	5.08	5.07	4.97	4.90	5.43	5.15	5.06	4.80
1368305_at	Casp6	caspase 6	0	0	0	0	0	0	uniqFlyp/lyp_145	0	0	1	0	0	6.98	6.80	6.50	6.62	6.96	6.79	6.63	6.70	6.68	6.34	6.18	6.14	6.68	6.43	6.36	6.14	6.72	6.26	6.23	6.23
1368325_at	Egf /// LOC10	epidermal growth factor /// pro-epidermal growth factor-like	0	0	0	0	0	0	uniqFlyp/lyp_145	0	0	1	0	0	6.47	6.37	6.32	6.19	6.48	6.12	6.17	6.13	7.01	6.61	6.51	6.42	7.03	6.55	6.38	6.34	6.90	6.55	6.57	6.60
1368431_at	Hpn	hepsin	0	0	0	0	0	0	uniqFlyp/lyp_145	0	0	1	0	0	5.44	5.38	5.03	5.15	5.37	5.12	5.12	5.30	5.59	5.15	5.08	5.12	5.67	5.75	5.27	5.21	5.57	5.53	5.25	5.48
1368446_at	Spink3	serine peptidase inhibitor, Kazal type 3	0	0	0	0	0	0	uniqFlyp/lyp_145	0	0	1	0	0	4.05	4.02	3.70	3.86	4.00	4.13	4.07	3.88	4.49	4.20	4.03	4.06	4.43	4.57	4.28	4.16	4.06	4.31	4.09	4.46
1368447_x_at	Spink3	serine peptidase inhibitor, Kazal type 3	0	0	0	0	0	0	uniqFlyp/lyp_145	0	0	1	0	0	7.14	7.59	6.15	6.20	7.21	7.00	6.41	7.36	7.89	7.39	7.33	6.88	7.83	8.98	7.46	6.71	7.31	8.40	7.32	7.81
1368598_at	Sstr3	somatostatin receptor 3	0	0	0	0	0	0	uniqFlyp/lyp_145	0	0	1	0	0	8.94	8.91	8.59	8.74	8.74	8.72	8.75	8.58	9.14	8.98	8.78	8.60	9.10	8.98	8.70	8.53	8.95	8.81	8.85	8.62
1369047_at	Sult1d1	sulfotransferase family 1D, member 1	0	0	0	0	0	0	uniqFlyp/lyp_145	0	0	1	0	0	3.29	3.48	3.32	3.11	3.23	3.34	3.08	2.92	3.38	3.53	3.05	3.01	3.21	3.22	3.31	3.05	3.16	3.26	3.10	2.85
1369074_at	Slc38a4	solute carrier family 38, member 4	0	0	0	0	0	0	uniqFlyp/lyp_145	0	0	1	0	0	10.45	10.67	10.51	10.41	10.89	10.25	10.47	10.13	10.60	10.42	10.22	9.86	9.73	10.08	10.18	10.38	10.48	10.28	10.36	9.99
1369170_at	Khsrp	KH-type splicing regulatory protein	0	0	0	0	0	0	uniqFlyp/lyp_145	0	0	1	0	0	2.83	2.96	2.90	3.03	2.84	2.91	3.17	2.88	2.90	2.89	2.93	3.00	2.83	2.91	2.86	2.88	3.04	2.96	2.99	2.80
1370247_a_at	Pmp22	peripheral myelin protein 22	0	0	0	0	0	0	uniqFlyp/lyp_145	0	0	1	0	0	6.90	6.77	6.63	6.52	6.92	6.76	6.67	6.83	6.41	6.68	5.91	5.83	6.56	6.40	6.20	5.87	6.64	6.17	6.02	5.85
1370254_at	Clic5	chloride intracellular channel 5	0	0	0	0	0	0	uniqFlyp/lyp_145	0	0	1	0	0	5.43	5.04	5.25	5.12	5.26	5.01	5.09	4.90	5.46	5.11	5.05	4.90	5.59	5.08	4.92	4.86	5.32	5.12	5.10	5.25
1370286_at	Slc38a2	solute carrier family 38, member 2	0	0	0	0	0	0	uniqFlyp/lyp_145	0	0	1	0	0	9.82	9.88	9.61	9.44	9.91	9.81	9.57	9.47	9.98	9.83	9.68	9.36	9.86	9.54	9.63	9.45	9.79	9.80	9.56	9.42
1370375_at	Gls2	glutaminase 2 (liver, mitochondrial)	0	0	0	0	0	0	uniqFlyp/lyp_145	0	0	1	0	0	6.38	6.47	6.03	6.11	6.48	6.29	6.08	6.12	7.32	6.47	6.63	6.40	7.22	7.59	6.59	6.37	6.81	7.10	6.51	6.93
1370504_a_at	Pmp22	peripheral myelin protein 22	0	0	0	0	0	0	uniqFlyp/lyp_145	0	0	1	0	0	5.93	5.89	5.85	5.89	5.94	5.90	5.91	5.99	5.80	5.98	5.74	5.83	5.99	5.81	5.76	5.74	5.95	5.74	5.75	5.75

Affymetrix Probeset ID	Gene Symbol	Gene Name	unique BB on P7 n=822	Figure 1B P7 Venn n=1526	DRlyp/lyp STEM P7 n=712	DR+/+ STEM P7 n=936	Flyp/lyp STEM P7 n=520	F+/+ STEM P7 n=465	Figure 1B P0 Venn n=1071	DRlyp/lyp STEM P0 n=442	DR+/+ STEM P0 n=437	Flyp/lyp STEM P0 n=643	F+/+ STEM P0 n=632	Fig1D n=25	Mean log2 intensity DRlyp/lyp 20	Mean log2 intensity DRlyp/lyp 30	Mean log2 intensity DRlyp/lyp 40	Mean log2 intensity DRlyp/lyp 50	Mean log2 intensity DR+/+ 20	Mean log2 intensity DR+/+ 30	Mean log2 intensity DR+/+ 40	Mean log2 intensity DR+/+ 50	Mean log2 intensity Flyp/lyp 20	Mean log2 intensity Flyp/lyp 30	Mean log2 intensity Flyp/lyp 40	Mean log2 intensity Flyp/lyp 50	Mean log2 intensity Flyp20	Mean log2 intensity Flyp30	Mean log2 intensity Flyp40	Mean log2 intensity Flyp50	Mean log2 intensity F+/+ 20	Mean log2 intensity F+/+ 30	Mean log2 intensity F+/+ 40	Mean log2 intensity F+/+ 50
1370585_a_at	Prkcb	protein kinase C, beta	0	0	0	0	0	0	uniqFlyp/lyp_145	0	0	1	0	0	8.16	7.89	7.68	7.99	8.22	7.93	7.71	8.52	8.69	8.40	8.27	7.90	8.43	8.17	8.00	8.04	8.43	8.33	8.07	8.06
1370733_at	Lipogenin	Lipogenin	0	0	0	0	0	0	uniqFlyp/lyp_145	0	0	1	0	0	4.99	4.60	4.64	4.79	4.93	4.48	4.66	4.65	4.94	4.61	4.63	4.33	4.82	4.93	4.44	4.61	5.07	4.66	4.70	4.59
1370805_at	Cited1 /// RG	Cbp/p300-interacting transactivator with Glu/Asp-rich carboxy-terminal domain 1 /// similar	0	0	0	0	0	0	uniqFlyp/lyp_145	0	0	1	0	0	5.33	5.57	5.50	5.35	5.46	5.56	5.65	5.48	5.77	5.83	5.82	5.32	5.93	5.93	5.77	5.45	5.76	5.88	5.71	5.60
1370919_at	Hnrnpm	heterogeneous nuclear ribonucleoprotein M	0	0	0	0	0	0	uniqFlyp/lyp_145	0	0	1	0	0	8.18	7.95	7.78	8.02	8.12	7.91	7.74	7.99	8.24	8.00	7.92	7.69	8.00	7.82	7.82	7.76	8.09	7.94	7.82	7.78
1371027_at	Cblb	Cbl proto-oncogene, E3 ubiquitin protein ligase B	0	0	0	0	0	0	uniqFlyp/lyp_145	0	0	1	0	0	7.36	7.18	7.13	7.34	7.22	7.19	7.14	7.42	7.81	7.50	7.51	7.23	7.82	7.64	7.40	7.37	7.66	7.43	7.32	7.30
1371166_at	Nos3	nitric oxide synthase 3, endothelial cell	0	0	0	0	0	0	uniqFlyp/lyp_145	0	0	1	0	0	4.80	4.61	4.42	4.42	4.64	4.30	4.65	4.31	4.68	4.30	4.24	4.13	4.73	4.62	4.27	3.96	4.72	4.30	4.35	4.30
1371374_at	Maf1	MAF1 homolog (S. cerevisiae)	0	0	0	0	0	0	uniqFlyp/lyp_145	0	0	1	0	0	8.13	8.09	7.97	7.79	8.08	7.99	7.92	7.79	8.33	8.12	7.94	7.79	8.30	8.12	7.94	7.83	8.16	8.06	7.97	7.71
1371377_at	LOC1003608	ribosomal protein S19-like /// ribosomal protein S19-like /// 40S ribosomal protein S19-like /	0	0	0	0	0	0	uniqFlyp/lyp_145	0	0	1	0	0	11.71	11.73	11.72	11.69	11.73	11.81	11.65	11.94	11.65	11.60	11.38	11.13	11.60	11.60	11.49	11.34	11.62	11.51	11.25	11.20
1371713_at	Abl1 /// LOC1	c-abl oncogene 1, non-receptor tyrosine kinase /// tyrosine-protein kinase ABL1-like	0	0	0	0	0	0	uniqFlyp/lyp_145	0	0	1	0	0	7.10	6.85	6.73	6.69	7.06	6.89	6.75	6.63	7.14	6.88	6.64	6.59	7.02	6.74	6.71	6.68	7.07	6.75	6.62	6.67
1371744_at	LOC679203	similar to Negative elongation factor D (NELF-D) (TH1-like protein)	0	0	0	0	0	0	uniqFlyp/lyp_145	0	0	1	0	0	7.88	7.69	7.59	7.51	7.77	7.65	7.47	7.38	7.80	7.66	7.52	7.30	7.81	7.52	7.47	7.33	7.84	7.63	7.55	7.39
1371970_at	Fam111a	family with sequence similarity 111, member A	0	0	0	0	0	0	uniqFlyp/lyp_145	0	0	1	0	0	10.93	10.68	10.88	11.35	11.00	10.80	10.92	11.23	4.62	4.33	4.31	3.88	3.81	3.99	4.20	4.10	4.21	4.29	3.85	4.06
1372015_at	Tacc1	transforming, acidic coiled-coil containing protein 1	0	0	0	0	0	0	uniqFlyp/lyp_145	0	0	1	0	0	7.92	7.88	7.79	7.60	7.98	7.84	7.76	7.70	7.91	7.69	7.62	7.40	8.17	7.80	7.70	7.45	7.83	7.72	7.81	7.62
1372038_at	Mknk2	MAP kinase-interacting serine/threonine kinase 2	0	0	0	0	0	0	uniqFlyp/lyp_145	0	0	1	0	0	6.31	6.23	6.00	6.21	6.47	6.21	6.01	6.42	6.71	6.43	6.21	5.96	6.78	6.20	6.25	6.23	6.61	6.30	6.19	6.12
1372039_at	Nkiras2	NFKB inhibitor interacting Ras-like 2	0	0	0	0	0	0	uniqFlyp/lyp_145	0	0	1	0	0	5.88	5.83	5.61	5.56	5.76	5.80	5.60	5.49	6.15	5.91	5.74	5.52	6.07	5.98	5.88	5.75	6.08	5.86	5.84	5.72
1372108_at	Ptcd3	Pentatricopeptide repeat domain 3	0	0	0	0	0	0	uniqFlyp/lyp_145	0	0	1	0	0	10.02	10.12	10.05	10.04	9.97	10.01	10.12	9.62	10.04	9.94	9.61	9.45	9.47	9.30	9.37	9.57	9.15	9.11	8.90	8.68
1372468_at	Cd97	CD97 molecule	0	0	0	0	0	0	uniqFlyp/lyp_145	0	0	1	0	0	6.30	5.98	5.84	6.07	6.40	5.90	5.87	6.49	6.32	5.80	5.82	5.48	6.40	5.90	5.72	5.75	6.09	5.94	5.71	5.65
1372475_at	Pink1	PTEN induced putative kinase 1	0	0	0	0	0	0	uniqFlyp/lyp_145	0	0	1	0	0	7.54	7.60	7.54	7.20	7.59	7.53	7.47	7.20	7.76	7.57	7.46	7.23	7.73	7.70	7.50	7.31	7.63	7.60	7.40	7.28
1373010_at	Krt12	keratin 12	0	0	0	0	0	0	uniqFlyp/lyp_145	0	0	1	0	0	4.10	4.04	3.86	3.69	4.03	4.07	3.78	3.77	4.19	4.34	3.83	3.79	4.00	4.29	4.04	3.88	4.30	3.97	4.03	4.13
1373223_at	---	---	0	0	0	0	0	0	uniqFlyp/lyp_145	0	0	1	0	0	7.17	7.36	7.31	7.02	7.13	7.27	7.17	7.01	7.98	8.04	7.90	7.70	8.05	7.83	7.78	7.67	7.85	7.79	7.68	7.61
1373243_at	Pmvk	phosphomevalonate kinase	0	0	0	0	0	0	uniqFlyp/lyp_145	0	0	1	0	0	7.17	7.22	6.99	7.04	7.26	7.09	7.16	7.11	7.33	7.29	7.03	6.83	7.35	7.20	7.01	6.92	7.16	7.05	6.95	6.80
1373288_at	St5	suppression of tumorigenicity 5	0	0	0	0	0	0	uniqFlyp/lyp_145	0	0	1	0	0	7.33	7.29	7.07	7.17	7.33	7.07	7.23	7.01	6.62	6.36	6.17	6.11	6.80	6.31	6.19	6.20	6.60	6.29	6.40	6.17
1373386_at	Gjb2	gap junction protein, beta 2	0	0	0	0	0	0	uniqFlyp/lyp_145	0	0	1	0	0	4.91	4.76	4.24	4.52	4.83	4.51	4.47	4.80	5.31	4.72	4.57	4.46	5.70	5.72	4.58	4.20	4.81	5.53	4.75	4.94
1373748_at	Pdzrn3	PDZ domain containing RING finger 3	0	0	0	0	0	0	uniqFlyp/lyp_145	0	0	1	0	0	5.51	5.75	5.45	5.30	5.80	5.59	5.46	5.73	5.84	5.93	5.67	5.29	5.81	5.95	5.65	5.49	5.78	5.94	5.79	5.48
1373757_at	Trafd1	TRAF type zinc finger domain containing 1	0	0	0	0	0	0	uniqFlyp/lyp_145	0	0	1	0	0	8.03	7.94	7.79	8.09	8.12	8.01	7.83	7.66	8.22	8.02	7.90	7.72	8.43	7.94	7.88	7.93	8.20	7.98	8.02	7.88
1373943_at	Stk4	serine/threonine kinase 4	0	0	0	0	0	0	uniqFlyp/lyp_145	0	0	1	0	0	6.30	6.16	6.00	6.32	6.33	6.23	6.11	6.38	6.26	6.13	6.12	6.15	6.43	6.22	6.13	6.25	6.26	6.32	6.29	6.22
1374024_at	Gpatch8	G patch domain containing 8	0	0	0	0	0	0	uniqFlyp/lyp_145	0	0	1	0	0	8.18	8.03	7.90	7.88	8.10	8.09	7.95	7.82	8.42	8.13	7.98	7.90	8.37	8.07	7.99	8.05	8.18	8.08	7.97	7.87
1374025_at	Nmnat3	nicotinamide nucleotide adenylyltransferase 3	0	0	0	0	0	0	uniqFlyp/lyp_145	0	0	1	0	0	4.98	4.97	4.84	4.84	5.09	4.89	4.78	4.63	4.98	4.77	4.57	4.32	4.78	4.85	4.64	4.45	4.80	4.68	4.58	4.56
1374033_at	Psmb10	proteasome (prosome, macropain) subunit, beta type 10	0	0	0	0	0	0	uniqFlyp/lyp_145	0	0	1	0	0	7.31	7.22	7.12	8.16	7.49	7.17	7.04	7.68	7.50	7.10	6.92	6.96	7.62	7.27	7.07	6.92	7.36	7.22	7.08	6.98
1374225_at	LOC500700	similar to chromosome 14 open reading frame 145	0	0	0	0	0	0	uniqFlyp/lyp_145	0	0	1	0	0	6.35	6.40	6.29	6.26	6.59	6.41	6.27	6.26	6.91	6.91	6.91	6.37	7.01	6.79	6.65	6.51	7.01	6.67	6.59	6.52
1374565_at	Nek6	NIMA-related kinase 6	0	0	0	0	0	0	uniqFlyp/lyp_145	0	0	1	0	0	4.33	4.17	4.24	4.26	4.44	4.39	4.18	4.40	4.39	4.31	3.95	3.81	4.60	4.23	4.00	3.92	4.24	4.01	4.08	3.82
1374574_at	Fam214a	family with sequence similarity 214, member A	0	0	0	0	0	0	uniqFlyp/lyp_145	0	0	1	0	0	6.67	6.58	6.45	6.47	6.53	6.24	6.36	6.33	7.76	7.50	7.20	7.14	7.74	7.27	7.22	7.28	7.48	7.27	7.27	7.16
1374693_at	Parp16	poly (ADP-ribose) polymerase family, member 16	0	0	0	0	0	0	uniqFlyp/lyp_145	0	0	1	0	0	5.61	5.73	5.37	5.31	5.71	5.69	5.36	5.37	5.44	5.29	5.16	4.67	5.63	5.28	5.10	4.96	5.45	5.21	4.97	5.02
1374738_at	Cwc27	CWC27 spliceosome-associated protein homolog (S. cerevisiae)	0	0	0	0	0	0	uniqFlyp/lyp_145	0	0	1	0	0	6.02	5.94	5.72	5.81	6.02	6.08	5.79	5.96	6.53	6.38	6.18	5.85	5.94	6.17	6.29	5.77	6.28	6.06	5.93	5.96
1375426_a_at	Khsrp	KH-type splicing regulatory protein	0	0	0	0	0	0	uniqFlyp/lyp_145	0	0	1	0	0	8.51	8.26	8.16	8.06	8.10	8.44	7.97	7.89	8.57	7.96	8.09	7.92	8.24	8.23	8.05	7.85	8.32	8.29	7.76	7.82
1375453_at	Gpatch8	G patch domain containing 8	0	0	0	0	0	0	uniqFlyp/lyp_145	0	0	1	0	0	8.68	8.69	8.53	8.38	8.80	8.57	8.36	8.34	8.92	8.66	8.53	8.27	8.68	8.45	8.54	8.35	8.76	8.50	8.49	8.44
1375703_at	Kmt2e	lysine (K)-specific methyltransferase 2E	0	0	0	0	0	0	uniqFlyp/lyp_145	0	0	1	0	0	8.61	8.45	8.36	8.25	8.67	8.51	8.28	8.27	8.71	8.48	8.38	8.22	8.67	8.37	8.37	8.18	8.62	8.43	8.23	8.04
1376150_at	S1pr3	sphingosine-1-phosphate receptor 3	0	0	0	0	0	0	uniqFlyp/lyp_145	0	0	1	0	0	5.71	5.70	5.60	6.07	5.83	5.60	5.63	6.34	5.93	5.44	5.58	5.33	6.06	5.49	5.72	5.93	5.63	5.62	5.61	5.72
1376287_at	Capn13	calpain 13	0	0	0	0	0	0	uniqFlyp/lyp_145	0	0	1	0	0	4.77	4.19	3.79	4.65	4.92	4.13	4.02	4.96	5.00	4.38	4.22	4.04	4.87	4.12	4.31	4.70	4.81	3.78	4.02	3.87
1376525_at	Khsrp	KH-type splicing regulatory protein	0	0	0	0	0	0	uniqFlyp/lyp_145	0	0	1	0	0	6.05	6.10	5.88	5.74	6.17	5.89	5.98	5.73	6.12	6.06	5.79	5.61	6.32	5.90	5.66	5.66	6.12	6.02	5.91	5.82
1376767_at	Gpr116	G protein-coupled receptor 116	0	0	0	0	0	0	uniqFlyp/lyp_145	0	0	1	0	0	6.51	6.41	6.31	6.32	6.56	6.27	6.41	6.30	6.73	6.71	6.51	6.39	6.89	6.68	6.61	6.30	6.85	6.50	6.58	6.40
1376868_at	Cobll1	cordon-bleu WH2 repeat protein-like 1	0	0	0	0	0	0	uniqFlyp/lyp_145	0	0	1	0	0	8.84	8.93	8.77	8.88	8.62	8.72	8.85	8.77	9.01	9.17	8.93	8.61	9.05	9.05	9.05	8.98	9.18	8.90	8.78	9.07
1376987_at	Lrrc49	leucine rich repeat containing 49	0	0	0	0	0	0	uniqFlyp/lyp_145	0	0	1	0	0	6.40	6.25	6.38	6.02	6.47	6.34	6.22	6.12	6.43	6.23	6.26	5.87	6.54	6.23	6.17	5.95	6.24	6.14	5.92	5.80
1377025_at	Kif3a	kinesin family member 3a	0	0	0	0	0	0	uniqFlyp/lyp_145	0	0	1	0	0	5.02	5.09	4.99	4.82	4.95	5.03	4.99	4.88	5.08	5.19	5.03	4.82	4.72	4.95	5.10	4.85	4.98	4.96	4.90	4.78
1377034_at	Serpinb1a	serine (or cysteine) proteinase inhibitor, clade B, member 1a	0	0	0	0	0	0	uniqFlyp/lyp_145	0	0	1	0	0	7.34	7.02	6.43	7.90	7.40	6.70	6.97	8.10	7.50	6.97	6.72	6.76	8.03	8.17	7.31	7.19	7.20	7.45	6.74	7.36
1377351_at	Susd3	sushi domain containing 3	0	0	0	0	0	0	uniqFlyp/lyp_145	0	0	1	0	0	4.50	4.69	4.25	4.71	4.73	4.50	4.39	5.33	5.05	4.49	4.44	4.41	5.14	5.19	4.44	4.53	4.79	4.54	4.45	4.78
1377582_at	Tox3	TOX high mobility group box family member 3	0	0	0	0	0	0	uniqFlyp/lyp_145	0	0	1	0	0	2.98	2.95	3.04	3.04	2.77	2.91	3.08	2.92	2.87	2.84	3.10	3.30	3.04	2.95	2.86	3.09	3.02	3.06	3.05	3.06
1377644_at	RGD1308706	similar to RIKEN cDNA 4921524J17	0	0	0	0	0	0	uniqFlyp/lyp_145	0	0	1	0	0	8.53	8.49	8.40	8.26	8.59	8.43	8.35	8.29	8.77	8.50	8.33	8.12	8.58	8.42	8.38	8.23	8.76	8.44	8.30	8.30
1377846_a_at	LOC1003603	mutS homolog 6 (E. coli)	0	0	0	0	0	0	uniqFlyp/lyp_145	0	0	1	0	0	7.34	7.27	7.21	7.02	7.27	7.24	7.04	7.16	7.39	7.20	7.11	6.79	7.50	7.10	7.07	6.87	7.33	7.13	7.07	7.10
1378310_at	Nmb	neuromedin B	0	0	0	0	0	0	uniqFlyp/lyp_145	0	0	1	0	0	5.46	5.30	5.21	5.21	5.64	5.19	5.53	5.24	5.74	5.35	5.29	4.97	5.75	5.29	5.17	5.08	5.51	5.35	5.20	5.01
1378332_at	Klf3	Kruppel-like factor 3 (basic)	0	0	0	0	0	0	uniqFlyp/lyp_145	0	0	1	0	0	7.43	7.26	7.16	7.05	7.45	7.30	7.04	7.10	7.52	7.28	7.08	6.86	7.73	7.23	7.16	6.96	7.49	7.18	7.13	7.09
1378509_at	Kif3a	kinesin family member 3a	0	0	0	0	0	0	uniqFlyp/lyp_145	0	0	1	0	0	5.94	5.94	5.90	5.75	5.87	5.59	5.87	5.68	5.73	5.92	5.64	5.24	5.43	5.49	5.71	5.49	5.65	5.59	5.80	5.68
1378642_at	Plk1s1	polo-like kinase 1 substrate 1	0	0	0	0	0	0	uniqFlyp/lyp_145	0	0	1	0	0	7.12	7.13	6.99	6.85	7.08	7.11	6.94	6.81	7.24	7.02	6.96	6.67	7.13	6.94	7.01	6.79	7.05	6.84	6.87	6.81
1378899_at	Slc35d3	solute carrier family 35, member D3	0	0	0	0	0	0	uniqFlyp/lyp_145	0	0	1	0	0	10.00	10.12	9.99	9.88	9.98	10.13	10.02	9.78	10.29	10.14	10.01	9.73	10.13	10.02	10.01	9.79	10.15	10.10	9.87	9.77
1378962_at	Cep95	centrosomal protein 95kDa	0	0	0	0	0	0	uniqFlyp/lyp_145	0	0	1	0	0	6.68	6.60	6.41	6.42	6.60	6.35	6.37	6.25	6.60	6.47	6.27	5.97	6.84	6.36	6.33	6.24	6.54	6.32	6.33	6.12
1378995_at	Fam214a	family with sequence similarity 214, member A	0	0	0	0	0	0	uniqFlyp/lyp_145	0	0	1	0	0	4.94	4.98	4.99	5.02	5.08	5.27	5.15	5.03	5.04	4.91	5.03	5.11	5.09	5.09	5.14	5.08	5.09	5.18	5.05	5.18
1379751_at	Tox3	TOX high mobility group box family member 3	0	0	0	0	0	0	uniqFlyp/lyp_145	0	0	1	0	0	8.16	8.18	7.88	7.73	8.13	8.17	8.00	7.55	8.61	8.28	7.97	7.64	8.33	8.06	8.03	7.76	8.33	8.05	7.81	7.64
1380346_at	Serpinb1a	serine (or cysteine) proteinase inhibitor, clade B, member 1a	0	0	0	0	0	0	uniqFlyp/lyp_145	0	0	1	0	0	5.83	5.67	5.34	6.05	5.72	5.67	5.84	6.19	5.95	5.54	5.59	5.63	6.10	6.33	5.82	5.76	5.76	5.95	5.45	5.85
1380493_at	Tet3	tet methylcytosine dioxygenase 3	0	0	0	0	0	0	uniqFlyp/lyp_145	0	0	1	0	0	5.73	5.51	5.46	5.41	5.82	5.69	5.58	5.61	5.85	5.53	5.54	5.27	5.70	5.57	5.60	5.23	5.69	5.77	5.80	5.60
1380611_at	Fkbp5	FK506 binding protein 5	0	0	0	0	0	0	uniqFlyp/lyp_145	0	0	1	0	0	6.92	6.92	6.64	6.75	6.69	6.76	7.02	6.72	7.55	7.43	7.12	6.60	6.97	7.58	7.25	7.04	7.74	6.97	6.24	6.87
1382553_at	Rfx3	regulatory factor X, 3 (influences HLA class II expression)	0	0	0	0	0	0	uniqFlyp/lyp_145	0	0	1	0	0	3.01	2.79	2.86	2.58	2.99	2.86	2.84	2.68	3.06	2.93	2.63	2.51	3.03	2.68	2.57	2.73	2.89	2.90	2.67	2.83
1382579_at	Tox3	TOX high mobility group box family member 3	0	0	0	0	0	0	uniqFlyp/lyp_145	0	0	1	0	0	10.62	10.53	10.31	10.18	10.56	10.58	10.30	10.12	10.91	10.63	10.47	10.14	10.72	10.49	10.46	10.29	10.72	10.50	10.29	10.33
1382588_at	Ldlrad4	low density lipoprotein receptor class A domain containing 4	0	0	0	0	0	0	uniqFlyp/lyp_145	0	0	1	0	0	4.43	4.59	4.39	4.21	4.56	4.17	4.20	4.57	4.87	4.37	4.41	4.41	4.54	4.47	4.58	4.61	4.58	4.38	4.45	4.79
1382899_at	LOC1003607	SLC2A4 regulator-like	0	0	0	0	0	0	uniqFlyp/lyp_145	0	0	1	0	0	6.99	6.91	6.63	6.85	7.04	6.78	6.68	6.54	7.10	6.85	6.66	6.53	6.90	6.70	6.56	6.58	6.99	6.79	6.71	6.56
1383606_at	Tc2n	tandem C2 domains, nuclear	0	0	0	0	0	0	uniqFlyp/lyp_145	0	0	1	0	0	6.66	6.71	6.51	6.33	6.65	6.58	6.55	6.60	6.97	6.47	6.37	6.41	7.00	7.01	6.56	6.23	6.64	6.75	6.44	6.67
1383654_a_at	Fn3k	fructosamine 3 kinase	0	0	0	0	0	0	uniqFlyp/lyp_145	0	0	1	0	0	7.89	7.93	7.83	7.73	7.91	7.96	7.83	7.67	7.85	7.67	7.39	7.32	7.87	7.66	7.59	7.51	7.80	7.57	7.46	7.55
1383726_at	Rgs7bp	regulator of G-protein signaling 7 binding protein	0	0	0	0	0	0	uniqFlyp/lyp_145	0	0	1	0	0	2.69	2.57	2.61	2.70	2.62	2.53	2.66	2.72	3.12	2.68	2.50	2.65	2.74	2.76	2.60	2.59	2.94	2.78	2.62	2.63
1384125_at	Kmt2e	lysine (K)-specific methyltransferase 2E	0	0	0	0	0	0	uniqFlyp/lyp_145	0	0	1	0	0	10.23	10.10	10.02	9.97	10.26	9.99	9.94	9.96	10.26	10.01	9.88	9.72	10.31	9.91	9.93	9.82	10.14	9.94	9.82	9.69
1384173_at	Skap1	src kinase associated phosphoprotein 1	0	0	0	0	0	0	uniqFlyp/lyp_145	0	0	1	0	0	5.59	5.43	5.18	5.21	5.70	5.49	5.43	5.58	5.64	5.54	5.36	5.02	5.95	5.40	5.41	5.20	5.63	5.46	5.29	5.16
1384993_at	RGD1309847	similar to peptidylglycine alpha-amidating monooxygenase COOH-terminal interactor; peptid	0	0	0	0	0	0	uniqFlyp/lyp_145	0	0	1	0	0	6.08	6.17	6.05	6.08	6.51	6.25	6.24	6.11	5.90	5.67	5.48	5.27	5.69	5.67	5.50	5.48	5.80	5.50	5.40	5.31
1385211_at	LOC1003615	rCG31991-like	0	0	0	0	0	0	uniqFlyp/lyp_145	0	0	1	0	0	8.71	8.72	8.44	8.47	8.72	8.57	8.50	8.30	9.22	9.06	8.82	8.48	8.77	8.76	8.78	8.55	8.99	8.69	8.55	8.63
1385422_at	Kmt2e	lysine (K)-specific methyltransferase 2E	0	0	0	0	0	0	uniqFlyp/lyp_145	0	0	1	0	0	9.37	9.21	9.15	8.97	9.41	9.27	9.07	9.02	9.39	9.14	8.98	8.80	9.42	9.02	9.07	8.90	9.30	9.07	8.93	8.77
1385472_at	Zbtb8a	zinc finger and BTB domain containing 8a	0	0	0	0	0	0	uniqFlyp/lyp_145	0	0	1	0	0	7.34	7.12	7.05	6.97	7.35	7.01	7.05	6.92	7.66	7.24	7.14	6.98	7.52	7.31	7.34	7.00	7.59	7.09	6.91	7.10
1386065_at	Sowahc	sosondowah ankyrin repeat domain family member C	0	0	0	0	0	0	uniqFlyp/lyp_145	0	0	1	0	0	7.85	7.96	7.78	7.75	7.78	7.86	7.94	7.89	8.15	8.01	7.83	7.53	8.04	7.71	7.84	7.70	7.87	7.87	7.68	7.45
1386857_at	RGD1565395	similar to stathmin 1/oncoprotein 18) /// stathmin 1	0	0	0	0	0	0	uniqFlyp/lyp_145	0	0	1	0	0	8.74	8.98	8.48	8.63	8.77	8.95	8.51	8.95	8.76	8.99	8.80	8.20	8.59	8.68	8.70	8.34	8.77	8.78	8.44	8.33
1386903_at	S100b	S100 calcium binding protein B	0	0	0	0	0	0	uniqFlyp/lyp_145	0	0	1	0	0	4.75	4.38	4.36	4.48	4.79	4.52	4.31	4.84	4.67	4.34	4.02	4.02	4.58	4.12	4.15	4.01	4.86	4.28	4.14	4.34
1387088_at	Gal	galanin/GMAP prepropeptide	0	0	0	0	0	0	uniqFlyp/lyp_145	0	0	1	0	0	4.16	3.91	3.82	3.84	4.12	4.12	4.09	3.77	4.99	4.37	4.34	4.44	4.57	4.28	4.18	4.23	4.15	4.48	4.20	4.23
1387099_at	Npr2	natriuretic peptide receptor B/guanylate cyclase B (atrionatriuretic peptide receptor B)	0	0	0	0	0	0	uniqFlyp/lyp_145	0	0	1	0	0	6.54	6.54	6.30	6.27	6.43	6.52	6.38	6.19	6.75	6.67	6.51	6.18	6.97	6.58	6.38	6.23	6.63	6.41	6.42	6.14
1387182_at	Gpr37	G protein-coupled receptor 37	0	0	0	0	0	0	uniqFlyp/lyp_145	0	0	1	0	0	3.48	3.30	3.27	3.23	3.47	3.45	3.38	2.98	3.55	3.63	3.17	3.08	3.73	3.29	3.08	3.17	3.69	3.15	3.29	3.32
1387184_at	Axin2	axin 2	0	0	0	0	0	0	uniqFlyp/lyp_145	0	0	1	0	0	4.95	4.85	4.62	4.76	5.11	4.81	4.71	4.76	5.18	4.99	4.98	4.63	4.74	4.96	5.06	4.83	5.07	4.89	4.73	4.73
1387193_a_at	Spink3	serine peptidase inhibitor, Kazal type 3	0	0	0	0	0	0	uniqFlyp/lyp_145	0	0	1	0	0	8.17	8.64	7.53	7.73	8.17	8.09	7.65	8.37	8.55	8.19	8.23	7.81	8.72	9.92	8.30	7.60	8.03	9.31	8.27	8.78
1387270_at	Hhex	hematopoietically expressed homeobox	0	0	0	0	0	0	uniqFlyp/lyp_145	0	0	1	0	0	7.37	7.29	7.28	7.39	7.55	7.41	7.44	7.44	7.46	7.23	7.15	6.88	7.65	7.21	7.57	7.26	7.68	6.97	7.01	7.21
1387546_at	Cdk12	cyclin-dependent kinase 12	0	0	0	0	0	0	uniqFlyp/lyp_145	0	0	1	0	0	6.13	6.05	5.89	5.90	5.98	5.83	5.82	5.68	6.34	5.83	6.02	5.49	5.35	5.88	5.81	5.58	6.16	6.04	5.78	6.04
1387808_at	Slc7a7	solute carrier family 7 (amino acid transporter light chain, y+L system), member 7	0	0	0	0	0	0	uniqFlyp/lyp_145	0	0	1	0	0	4.85	5.00	4.79	4.97	5.24	4.77	4.63	5.47	4.78	4.63	4.23	4.34	4.96	4.73	4.59	4.35	4.61	4.62	4.51	4.32
1388108_at	Elovl6 /// LOC	ELOVL fatty acid elongase 6 /// elongation of very long chain fatty acids protein 6-like	0	0	0	0	0	0	uniqFlyp/lyp_145	0	0	1	0	0	8.65	8.40	8.35	8.27	8.45	8.23	8.51	8.08	9.32	9.02	8.94	8.46	9.18	8.83	8.79	8.62	9.05	8.53	8.43	8.68
1388109_at	Gpr116	G protein-coupled receptor 116	0	0	0	0	0	0	uniqFlyp/lyp_145	0	0	1	0	0	7.19	7.03	6.86	6.83	7.21	6.94	6.86	6.85	7.51	7.33	7.13	6.84	7.30	7.37	7.21	6.86	7.51	7.36	7.19	7.18
1388471_at	Tcp11l2	t-complex 11, testis-specific-like 2	0	0	0	0	0	0	uniqFlyp/lyp_145	0	0	1	0	0	9.24	9.14	9.05	8.99	9.31	9.11	9.11	9.00	9.49	9.18	9.11	8.91	9.55	9.22	9.26	9.02	9.37	9.19	9.01	8.97
1388730_at	Cdc42ep4	CDC42 effector protein (Rho GTPase binding) 4	0	0	0	0	0	0	uniqFlyp/lyp_145	0	0	1	0	0	7.67	7.47	7.50	7.32	7.71	7.63	7.47	7.36	7.71	7.57	7.45	7.15	7.89	7.57	7.41	7.29	7.63	7.44	7.36	7.31
1388799_at	Klhl7	kelch-like family member 7	0	0	0	0	0	0	uniqFlyp/lyp_145	0	0	1	0	0	9.88	9.88	9.79	9.66	9.92	9.91	9.67	9.61	9.90	9.72	9.55	9.35	9.65	9.61	9.70	9.44	9.75	9.63	9.45	9.48
1388901_at	Fkbp5	FK506 binding protein 5	0	0	0	0	0	0	uniqFlyp/lyp_145	0	0	1	0	0	8.83	8.71	8.44	8.44	8.57	8.63	8.69	8.27	9.34	9.06	8.71	8.10	9.37	9.21	8.94	8.69	9.43	8.62	8.16	8.74
1389479_at	Klf3	Kruppel-like factor 3 (basic)	0	0	0	0	0	0	uniqFlyp/lyp_145	0	0	1	0	0	7.73	7.63	7.46	7.50	7.70	7.61	7.44	7.51	7.76	7.60	7.43	7.24	8.14	7.58	7.56	7.45	7.82	7.50	7.41	7.49
1389517_at	LOC681186	hypothetical protein LOC681186	0	0	0	0	0	0	uniqFlyp/lyp_145	0	0	1	0	0	4.53	4.42	4.39	4.41	4.52	4.46	4.46	4.42	5.00	4.47	4.54	4.43	4.90	4.41	4.44	4.48	4.65	4.47	4.41	4.35
1389728_at	Meis2	Meis homeobox 2	0	0	0	0	0	0	uniqFlyp/lyp_145	0	0	1	0	0	8.40	8.41	8.28	8.00	8.41	8.31	8.11	7.95	8.34	8.13	7.97	7.75	8.25	8.03	7.95	7.80	8.22	8.08	8.03	7.76
1389738_at	Ung	uracil-DNA glycosylase	0	0	0	0	0	0	uniqFlyp/lyp_145	0	0	1	0	0	7.08	7.06	6.86	7.00	7.22	6.98	6.99	7.10	7.06	6.82	6.77	6.45	7.05	6.88	6.82	6.67	7.02	6.86	6.70	6.82
1390042_at	Tmem140	transmembrane protein 140	0	0	0	0	0	0	uniqFlyp/lyp_145	0	0	1	0	0	7.76	7.74	7.60	8.00	7.77	7.70	7.63	7.47	7.77	7.57	7.16	7.23	7.79	7.46	7.44	7.15	7.59	7.29	7.18	7.12
1390107_at	LOC1009118	synaptotagmin-like protein 2-like /// synaptotagmin-like 2	0	0	0	0	0	0	uniqFlyp/lyp_145	0	0	1	0	0	6.25	6.35	6.20	6.25	6.36	6.39	6.25	6.17	5.88	5.91	5.60	5.41	5.86	5.91	5.80	5.51	5.82	5.80	5.70	5.53
1390519_at	Gltscr1	glioma tumor suppressor candidate region gene 1	0	0	0	0	0	0	uniqFlyp/lyp_145	0	0	1	0	0	5.33	5.11	4.98	5.05	5.25	5.05	5.02	4.98	5.26	5.00	4.94	4.71	5.44	5.02	4.91	5.05	5.15	4.95	5.09	5.16
1390650_at	Nup85	nucleoporin 85	0	0	0	0	0	0	uniqFlyp/lyp_145	0	0	1	0	0	6.31	6.31	6.13	6.07	6.28	6.09	6.07	6.17	6.42	6.23	6.03	5.90	6.43	6.22	6.16	5.94	6.34	5.99	6.03	5.91
1390796_at	Gpatch8	G patch domain containing 8	0	0	0	0	0	0	uniqFlyp/lyp_145	0	0	1	0	0	7.67	7.61	7.47	7.40	7.59	7.42	7.44	7.27	7.68	7.51	7.36	7.15	7.70	7.32	7.29	7.34	7.62	7.19	7.37	7.29
1390814_at	Peli1	pellino E3 ubiquitin protein ligase 1	0	0	0	0	0	0	uniqFlyp/lyp_145	0	0	1	0	0	8.34	8.27	8.11	8.05	8.28	8.21	8.10	8.09	8.38	8.08	8.00	7.75	8.39	8.11	8.03	7.84	8.26	8.11	7.89	7.81
1390820_at	Zcchc11	zinc finger, CCHC domain containing 11	0	0	0	0	0	0	uniqFlyp/lyp_145	0	0	1	0	0	8.32	8.12	7.95	8.04	8.16	8.06	7.94	7.96	8.40	8.18	7.99	7.87	8.17	8.10	7.96	8.00	8.27	8.04	7.92	7.83
1390832_at	Tmcc3	transmembrane and coiled-coil domain family 3	0	0	0	0	0	0	uniqFlyp/lyp_145	0	0	1	0	0	9.07	9.00	8.87	8.65	9.11	9.00	8.85	8.67	9.12	8.83	8.70	8.44	9.09	8.75	8.80	8.47	9.13	8.77	8.67	8.59
1391714_at	Plag1	pleiomorphic adenoma gene 1	0	0	0	0	0	0	uniqFlyp/lyp_145	0	0	1	0	0	7.85	7.90	7.80	7.56	8.03	7.94	7.69	7.53	8.18	8.06	7.84	7.61	8.33	7.81	7.76	7.77	8.10	7.86	7.83	7.63
1392231_at	Stk4	serine/threonine kinase 4	0	0	0	0	0	0	uniqFlyp/lyp_145	0	0	1	0	0	5.43	5.76	5.47	5.65	5.39	5.37	5.39	5.72	6.08	5.47	5.61	5.17	4.83	5.40	5.56	5.19	5.62	5.42	5.15	5.01
1392467_at	Impa2	inositol (myo)-1(or 4)-monophosphatase 2	0	0	0	0	0	0	uniqFlyp/lyp_145	0	0	1	0	0	5.11	5.11	4.95	4.80	4.97	5.12	4.89	5.17	4.91	4.71	4.63	4.33	4.96	5.01	4.60	4.61	5.09	4.69	4.65	4.72
1392564_at	Kmt2e	lysine (K)-specific methyltransferase 2E	0	0	0	0	0	0	uniqFlyp/lyp_145	0	0	1	0	0	6.95	6.85	6.68	6.68	6.88	6.69	6.72	6.64	7.14	6.79	6.62	6.64	7.36	6.85	6.69	6.70	7.05	6.75	6.77	6.72
1393098_at	Llgl2	lethal giant larvae homolog 2 (Drosophila)	0	0	0	0	0	0	uniqFlyp/lyp_145	0	0	1	0	0	8.36	8.33	8.17	8.16	8.37	8.25	8.29	8.04	8.65	8.43	8.32	8.13	8.64	8.38	8.30	8.19	8.52	8.27	8.37	8.35
1393147_at	Clca5	chloride channel calcium activated 5	0	0	0	0	0	0	uniqFlyp/lyp_145	0	0	1	0	0	6.15	5.90	5.71	5.66	6.12	6.15	5.90	5.85	6.52	5.82	5.85	5.85	5.91	6.71	5.77	5.68	6.23	6.52	5.99	6.22
1393150_at	Klf3	Kruppel-like factor 3 (basic)	0	0	0	0	0	0	uniqFlyp/lyp_145	0	0	1	0	0	7.20	7.18	7.00	6.98	7.22	7.10	6.99	6.89	7.28	7.07	6.86	6.68	7.36	7.03	6.93	6.92	7.25	6.97	7.04	6.86
1393174_at	Fam171b	family with sequence similarity 171, member B	0	0	0	0	0	0	uniqFlyp/lyp_145	0	0	1	0	0	4.41	4.27	3.88	3.90	4.04	4.01	3.65	3.65	5.10	4.80	4.64	4.37	4.22	4.68	4.26	4.51	4.72	4.26	4.16	4.00
1393352_at	Fam214a	family with sequence similarity 214, member A	0	0	0	0	0	0	uniqFlyp/lyp_145	0	0	1	0	0	8.28	8.23	8.13	8.14	8.39	8.27	8.19	8.08	8.82	8.54	8.28	8.16	8.71	8.43	8.35	8.23	8.70	8.41	8.24	8.23
1393822_at	Tmcc3	transmembrane and coiled-coil domain family 3	0	0	0	0	0	0	uniqFlyp/lyp_145	0	0	1	0	0	5.64	5.50	5.33	5.21	5.69	5.36	5.26	5.25	5.62	5.32	5.28	5.09	5.75	5.34	5.42	5.10	5.78	5.43	5.44	5.41
1394042_at	LOC681371	hypothetical protein LOC681371	0	0	0	0	0	0	uniqFlyp/lyp_145	0	0	1	0	0	6.40	6.38	6.30	6.19	6.65	6.37	6.39	6.27	6.70	6.45	6.36	6.01	6.77	6.28	6.34	6.26	6.51	6.21	6.12	6.06
1394401_at	Elovl6 /// LOC	ELOVL fatty acid elongase 6 /// elongation of very long chain fatty acids protein 6-like	0	0	0	0	0	0	uniqFlyp/lyp_145	0	0	1	0	0	8.68	8.45	8.27	8.13	8.57	8.19	8.39	8.08	9.23	8.85	8.71	8.07	9.03	8.56	8.60	8.13	8.88	8.42	7.92	8.31
1394550_at	LOC691221	similar to CG1998-PA	0	0	0	0	0	0	uniqFlyp/lyp_145	0	0	1	0	0	5.78	5.75	5.59	5.62	5.72	5.56	5.68	5.48	5.31	5.32	4.93	4.81	5.30	5.06	5.07	4.96	5.31	4.98	4.99	4.81
1395460_at	Caskin2	cask-interacting protein 2	0	0	0	0	0	0	uniqFlyp/lyp_145	0	0	1	0	0	6.35	6.06	6.11	6.02	6.30	6.23	6.17	6.02	6.16	5.96	5.72	5.62	6.48	5.97	5.85	5.75	6.28	5.93	5.95	5.87
1396320_at	Cdk12	cyclin-dependent kinase 12	0	0	0	0	0	0	uniqFlyp/lyp_145	0	0	1	0	0	5.59	5.22	5.27	5.54	5.25	4.94	5.12	5.14	5.93	5.50	5.34	5.63	5.66	5.53	5.19	5.62	5.78	5.34	5.47	5.61
1397189_at	Prkcb	protein kinase C, beta	0	0	0	0	0	0	uniqFlyp/lyp_145	0	0	1	0	0	3.03	3.02	2.90	3.23	3.28	2.97	2.88	3.13	3.71	3.22	3.32	3.17	3.14	3.11	3.04	3.23	3.32	3.24	3.24	3.22

Affymetrix Probeset ID	Gene Symbol	Gene Name	unique BB on P7 n=822	Figure 1B P7 Venn n=1526	DRlyp/lyp STEM P7 n=712	DR+/+ STEM P7 n=936	Flyp/lyp STEM P7 n=520	F+/+ STEM P7 n=465	Figure 1B P0 Venn n=1071	DRlyp/lyp STEM P0 n=442	DR+/+ STEM P0 n=437	Flyp/lyp STEM P0 n=643	F+/+ STEM P0 n=632	Fig1D n=25	Mean log2 intensity DRlyp/lyp 20	Mean log2 intensity DRlyp/lyp 30	Mean log2 intensity DRlyp/lyp 40	Mean log2 intensity DRlyp/lyp 50	Mean log2 intensity DR+/+ 20	Mean log2 intensity DR+/+ 30	Mean log2 intensity DR+/+ 40	Mean log2 intensity DR+/+ 50	Mean log2 intensity Flyp/lyp 20	Mean log2 intensity Flyp/lyp 30	Mean log2 intensity Flyp/lyp 40	Mean log2 intensity Flyp/lyp 50	Mean log2 intensity Flyp20	Mean log2 intensity Flyp30	Mean log2 intensity Flyp40	Mean log2 intensity Flyp50	Mean log2 intensity F+/+ 20	Mean log2 intensity F+/+ 30	Mean log2 intensity F+/+ 40	Mean log2 intensity F+/+ 50
1397882_at	Zbtb8a	zinc finger and BTB domain containing 8a	0	0	0	0	0	0	uniqFlyp/lyp_145	0	0	1	0	0	5.84	5.82	5.85	5.60	5.88	5.69	5.71	5.52	6.19	5.94	5.82	5.70	6.02	5.81	5.88	5.77	6.15	5.63	5.70	5.93
1398365_at	Tppp3	tubulin polymerization-promoting protein family member 3	0	0	0	0	0	0	uniqFlyp/lyp_145	0	0	1	0	0	6.74	6.65	6.66	6.51	6.93	6.71	6.70	6.69	6.80	7.05	6.47	6.52	6.80	6.64	6.54	6.41	6.90	6.57	6.43	6.41
1398575_at	Zbtb5	zinc finger and BTB domain containing 5	0	0	0	0	0	0	uniqFlyp/lyp_145	0	0	1	0	0	7.32	7.10	7.06	6.88	7.27	7.14	6.96	6.80	7.27	7.05	6.98	6.75	7.35	6.97	6.95	6.98	7.22	6.90	6.88	6.88

Data are available as a sortable spreadsheet upon request

[1] Data are expressed in a binar fashion as meeting (1) or not meeting (0) the query defined in the header.
