# Supplementary material for: Modulation of the diet and gastrointestinal microbiota normalizes systemic inflammation and β-cell chemokine expression associated with autoimmune diabetes susceptibility
Source: PLoS One. 2018 Jan 2;13(1):e0190351. doi: 10.1371/journal.pone.0190351 (PMC5749787; doi:10.1371/journal.pone.0190351)
Supplement: S4 Table — (PDF) [file pone.0190351.s004.pdf]

**S4 Table. Plasma Induced Transcription Data [1]**

| Affymetrix Probeset ID | Gene Symbol | Gene Title | Figure 6 B Venn n =2021 | Mean BN log2 intensity autologous n=8 | Mean log2 intensity day 30 DR +/+ ND n=4 | Mean log2 intensity day 30 DR+/+ HCD n=4 | Mean log2 intensity day 30 DR+/+ B/S n=4 | Mean log2 intensity day 30 F+/+ ND n=4 | DR+/+ HCD vs DR+/+ ND n=380 1=yes 0=no | Log2 ratio DR+/+ HCD vs DR+/+ ND | Fold-Change DR+/+ HCD vs DR+/+ ND | RP-FDR 4DR30 HCD vs 4DR30 Reg | p value DR+/+ HCD vs DR+/+ ND | DR+/+ B/S vs DR+/+ ND n=1,330 1=yes 0=no | Log2 ratio DR+/+ B/S vs DR+/+ ND | Fold-Change DR+/+ B/S vs DR+/+ ND | RP-FDR 4DR30 Antib vs 4DR30 Reg | p value DR+/+ B/S vs DR+/+ ND | DR+/+ ND vs F+/+ ND n=398 1=yes 0=no | Log2 ratio DR+/+ ND vs F+/+ ND | Fold-Change DR+/+ ND vs F+/+ ND | RP-FDR 4RT1uu 30Reg vs 4DR30 Reg | p value DR+/+ ND vs F+/+ ND | DR+/+ Longitudinal n=228 1=yes 0=no | Figure 6F |
|---|---|---|---|---|---|---|---|---|---|---|---|---|---|---|---|---|---|---|---|---|---|---|---|---|---|
| 1368490_at | Cd14 | CD14 molecule | Any2_257 | -0.12 | -0.63 | -0.38 | 0.81 | 0.35 | 0 | 0.24973 | 1.18898 | 2.62192 | 0.00438 | 1 | 1.44082 | 2.71474 | 0 | 0 | 1 | 0.9772 | 1.96865 | 0 | 0 | 1 | 1 |
| 1367973_at | Ccl2 | chemokine (C-C motif) ligand 2 | Any2_257 | 0.00 | -0.36 | -0.07 | 1.03 | 0.40 | 1 | 0.28277 | 1.21653 | 2.14529 | 0.02414 | 1 | 1.38484 | 2.61143 | 0 | 0 | 1 | 0.75644 | 1.68932 | 0.00579 | 3.5E-06 | 1 | 2 |
| 1371913_at | Tgfbi | transforming growth factor, beta induced | Any2_257 | 0.04 | -0.19 | -0.10 | 1.57 | 0.73 | 0 | 0.08727 | 1.06236 | 1.52394 | 0.15157 | 1 | 1.75865 | 3.3838 | 0 | 0 | 1 | 0.91865 | 1.89034 | 0 | 0 | 1 | 3 |
| 1382108_at | Mafb | musculoaponeurotic fibrosarcoma oncogene homol | Any2_257 | 0.32 | -0.14 | -0.06 | 1.38 | 0.80 | 0 | 0.08248 | 1.05884 | 1.44159 | 0.17587 | 1 | 1.52318 | 2.87423 | 0 | 0 | 1 | 0.943 | 1.92252 | 0 | 0 | 0 | 4 |
| 1393795_at | Zeb2 | zinc finger E-box binding homeobox 2 | Any2_257 | -0.20 | -0.50 | -0.09 | 0.21 | -0.50 | 1 | 0.41973 | 1.33768 | 1.4975 | 0.00019 | 1 | 0.71224 | 1.63835 | 0.02426 | 3.7E-05 | 0 | 0.00358 | 1.00248 | 1.41382 | 0.17876 | 0 | 5 |
| 1377064_at | Dusp6 | dual specificity phosphatase 6 | Any2_257 | 0.00 | -0.41 | -0.49 | 0.25 | -0.13 | 0 | -0.0859 | -1.0614 | 1.16649 | 0.30078 | 1 | 0.65325 | 1.57271 | 0.03083 | 4.8E-05 | 1 | 0.27469 | 1.20974 | 2.3556 | 0.02962 | 1 | 6 |
| 1377353_a_at | Tnfsf13 | tumor necrosis factor (ligand) superfamily, member | Any2_257 | -0.01 | -0.31 | -0.53 | 0.16 | 0.00 | 0 | -0.2158 | -1.1613 | 3.24315 | 0.01888 | 1 | 0.46869 | 1.38385 | 0.5609 | 0.00299 | 1 | 0.30822 | 1.23818 | 2.26093 | 0.0149 | 1 | 7 |
| 1368412_a_at | Ptpro | protein tyrosine phosphatase, receptor type, O | Any2_257 | -0.18 | -0.32 | -0.33 | 0.25 | 0.21 | 0 | -0.0044 | -1.0031 | 1.04009 | 0.40799 | 1 | 0.57459 | 1.48926 | 0.12038 | 0.00031 | 1 | 0.53147 | 1.44541 | 0.17343 | 0.0002 | 1 | 8 |
| 1371079_at | Fcgr2b | Fc fragment of IgG, low affinity IIb, receptor (CD32) | Any2_257 | -0.14 | -0.30 | -0.34 | 0.19 | -0.18 | 0 | -0.0427 | -1.03 | 1.33135 | 0.22706 | 1 | 0.49221 | 1.4066 | 0.34345 | 0.00125 | 0 | 0.11782 | 1.08509 | 1.3441 | 0.20249 | 1 | 9 |
| 1387260_at | Klf4 | Kruppel-like factor 4 (gut) | Any2_257 | 0.24 | 0.15 | 0.25 | 0.71 | 0.37 | 0 | 0.0916 | 1.06555 | 1.3241 | 0.22063 | 1 | 0.55747 | 1.47168 | 0.14185 | 0.00042 | 0 | 0.21385 | 1.15978 | 2.2084 | 0.04609 | 1 | 10 |
| 1386879_at | Lgals3 | lectin, galactoside-binding, soluble, 3 | Any2_257 | -0.06 | -0.18 | -0.22 | 1.03 | 0.16 | 0 | -0.0385 | -1.0271 | 1.00399 | 0.42079 | 1 | 1.20582 | 2.30668 | 0 | 0 | 1 | 0.33757 | 1.26363 | 1.6982 | 0.00546 | 1 | 11 |
| 1387599_a_at | Nqo1 | NAD(P)H dehydrogenase, quinone 1 | Any2_257 | -0.10 | -0.10 | -0.15 | 0.72 | 0.17 | 0 | -0.0518 | -1.0366 | 1.32934 | 0.22783 | 1 | 0.81634 | 1.76093 | 0.005 | 5.1E-06 | 1 | 0.26851 | 1.20456 | 2.16122 | 0.01029 | 1 | 12 |
| 1387029_at | Cfh | complement factor H | Any2_257 | -0.24 | -0.44 | -0.41 | 0.97 | 0.53 | 0 | 0.02337 | 1.01633 | 1.70435 | 0.1021 | 1 | 1.40973 | 2.65688 | 0 | 0 | 1 | 0.96497 | 1.95203 | 0 | 0 | 1 | 13 |
| 1367816_at | Hopx | HOP homeobox | Any2_257 | -0.11 | -0.51 | -0.43 | 0.39 | -0.08 | 0 | 0.07769 | 1.05533 | 1.53152 | 0.14789 | 1 | 0.9063 | 1.87424 | 0.00042 | 3.2E-07 | 1 | 0.43563 | 1.3525 | 0.72545 | 0.00128 | 1 | 14 |
| 1387391_at | Cdkn1a | cyclin-dependent kinase inhibitor 1A | Any2_257 | 0.12 | -0.24 | 0.17 | 0.47 | 0.26 | 1 | 0.40983 | 1.32853 | 3.07741 | 0.00267 | 1 | 0.70732 | 1.63276 | 0.02111 | 3.1E-05 | 1 | 0.49691 | 1.41118 | 0.35732 | 0.00047 | 0 | 15 |
| 1398256_at | Il1b | interleukin 1 beta | Any2_257 | -0.19 | -0.85 | -0.40 | -0.60 | -0.18 | 1 | 0.45396 | 1.36979 | 0.87 | 5.6E-05 | 0 | 0.2553 | 1.19359 | 0.78105 | 0.00691 | 1 | 0.67555 | 1.59721 | 0.01077 | 9E-06 | 0 | 16 |
| 1368147_at | Dusp1 | dual specificity phosphatase 1 | Any2_257 | -0.04 | -0.23 | 0.04 | 0.13 | -0.09 | 1 | 0.26848 | 1.20453 | 2.08329 | 0.02686 | 1 | 0.35898 | 1.28252 | 0.71979 | 0.00558 | 0 | 0.13975 | 1.10172 | 1.70919 | 0.10217 | 0 | 17 |
| 1372986_at | Jdp2 | Jun dimerization protein 2 | Any2_257 | 0.14 | 0.03 | 0.14 | 0.45 | 0.31 | 0 | 0.11464 | 1.08271 | 1.26382 | 0.24814 | 1 | 0.42548 | 1.34302 | 0.68493 | 0.00478 | 1 | 0.28859 | 1.22145 | 2.38127 | 0.02481 | 0 | 18 |
| 1374732_at | Hexim2 | hexamethylene bis-acetamide inducible 2 | Any2_257 | 0.09 | -0.32 | -0.03 | 0.05 | -0.04 | 1 | 0.28889 | 1.2217 | 2.21548 | 0.0156 | 1 | 0.37014 | 1.29248 | 0.83649 | 0.00804 | 1 | 0.28225 | 1.21609 | 2.28185 | 0.0182 | 0 | 0 |
| 1367612_at | Mgst1 | microsomal glutathione S-transferase 1 | Any2_257 | -0.08 | -0.38 | -0.39 | -0.11 | -0.27 | 0 | -0.0049 | -1.0034 | 1.52912 | 0.14957 | 1 | 0.26992 | 1.20574 | 1.12945 | 0.02106 | 0 | 0.11298 | 1.08146 | 1.89038 | 0.0755 | 1 | 0 |
| 1367677_at | Prdx5 | peroxiredoxin 5 | Any2_257 | -0.10 | -0.34 | -0.36 | 0.22 | -0.04 | 0 | -0.0257 | -1.018 | 1.02657 | 0.39998 | 1 | 0.56052 | 1.4748 | 0.10676 | 0.00025 | 1 | 0.30261 | 1.23337 | 2.17462 | 0.01014 | 1 | 0 |
| 1367709_at | Cd63 | Cd63 molecule | Any2_257 | -0.11 | -0.46 | -0.38 | 0.30 | 0.07 | 0 | 0.0811 | 1.05782 | 1.23446 | 0.26107 | 1 | 0.75185 | 1.68395 | 0.00842 | 1E-05 | 1 | 0.52192 | 1.43587 | 0.25179 | 0.00032 | 1 | 0 |
| 1367850_at | Fcgr2a /// LO | Fc fragment of IgG, low affinity IIa, receptor /// Low | Any2_257 | -0.07 | -0.60 | -0.24 | 0.34 | 0.12 | 1 | 0.35781 | 1.28148 | 2.65902 | 0.00351 | 1 | 0.94048 | 1.91917 | 0.00043 | 3.2E-07 | 1 | 0.72098 | 1.6483 | 0.005 | 3.5E-06 | 0 | 0 |
| 1367979_s_at | Cyp51 | cytochrome P450, family 51 | Any2_257 | -0.08 | -0.30 | -0.24 | 0.03 | -0.11 | 0 | 0.05753 | 1.04068 | 1.26428 | 0.24799 | 1 | 0.32665 | 1.2541 | 1.20241 | 0.02726 | 0 | 0.19092 | 1.14149 | 1.62875 | 0.12114 | 1 | 0 |
| 1367998_at | Slpi | secretory leukocyte peptidase inhibitor | Any2_257 | -0.01 | -1.16 | -1.32 | -0.68 | -0.35 | 0 | -0.1648 | -1.121 | 3.16861 | 0.02272 | 1 | 0.47616 | 1.39103 | 0.17302 | 0.00053 | 1 | 0.80651 | 1.74897 | 0.0025 | 9.6E-07 | 0 | 0 |
| 1368187_at | Gpnmb | glycoprotein (transmembrane) nmb | Any2_257 | -0.16 | -0.30 | -0.19 | 1.11 | 0.53 | 0 | 0.11106 | 1.08002 | 1.85229 | 0.06802 | 1 | 1.41294 | 2.6628 | 0 | 0 | 1 | 0.83422 | 1.78289 | 0.00556 | 3.2E-06 | 1 | 0 |
| 1368202_a_at | Dab2 | disabled 2, mitogen-responsive phosphoprotein | Any2_257 | -0.12 | -0.38 | -0.22 | 0.51 | 0.12 | 0 | 0.16001 | 1.11729 | 1.47154 | 0.16798 | 1 | 0.88776 | 1.8503 | 0.00333 | 2.9E-06 | 1 | 0.49509 | 1.40941 | 0.41711 | 0.0006 | 1 | 0 |
| 1368275_at | Msmo1 | methylsterol monooxygenase 1 | Any2_257 | -0.11 | -0.36 | -0.43 | 0.00 | -0.13 | 0 | -0.0691 | -1.0491 | 1.06596 | 0.36364 | 1 | 0.35791 | 1.28157 | 1.01961 | 0.01587 | 0 | 0.233 | 1.17528 | 2.01976 | 0.06293 | 1 | 0 |
| 1368368_a_at | Lsr | lipolysis stimulated lipoprotein receptor | Any2_257 | -0.09 | 0.28 | 0.03 | -0.01 | -0.05 | 0 | -0.2431 | -1.1836 | 2.9593 | 0.03131 | 1 | -0.2889 | -1.2217 | 1.75907 | 0.02427 | 1 | -0.3269 | -1.2543 | 3.66222 | 0.00318 | 0 | 0 |
| 1368554_at | Pnlip | pancreatic lipase | Any2_257 | 0.02 | -0.01 | -0.02 | -0.39 | -0.29 | 0 | -0.0095 | -1.0066 | 1.07526 | 0.35716 | 1 | -0.3828 | -1.3039 | 3.28 | 0.00485 | 1 | -0.2802 | -1.2144 | 3.00869 | 0.01693 | 0 | 0 |
| 1368742_at | C5ar1 | complement component 5a receptor 1 | Any2_257 | -0.09 | -0.03 | -0.10 | 0.59 | 0.23 | 0 | -0.0683 | -1.0485 | 1.44069 | 0.19026 | 1 | 0.6199 | 1.53677 | 0.05339 | 0.00011 | 0 | 0.2582 | 1.19599 | 2.35618 | 0.03076 | 1 | 0 |
| 1368760_at | Cxcl2 | chemokine (C-X-C motif) ligand 2 | Any2_257 | -0.03 | -0.72 | -0.56 | -0.26 | 0.02 | 0 | 0.16492 | 1.12111 | 2.41237 | 0.00752 | 1 | 0.46702 | 1.38226 | 0.12494 | 0.00034 | 1 | 0.74383 | 1.67461 | 0.00588 | 3.2E-06 | 0 | 0 |
| 1368778_at | Slc6a6 | solute carrier family 6 (neurotransmitter transporte | Any2_257 | 0.18 | 0.55 | 0.56 | 0.91 | 0.62 | 0 | 0.01505 | 1.01049 | 1.34493 | 0.2142 | 1 | 0.36093 | 1.28425 | 0.71432 | 0.00526 | 0 | 0.071 | 1.05044 | 1.71161 | 0.10204 | 1 | 0 |
| 1368878_at | Idi1 | isopentenyl-diphosphate delta isomerase 1 | Any2_257 | -0.14 | -0.29 | -0.48 | 0.02 | -0.08 | 0 | -0.1899 | -1.1407 | 1.71828 | 0.13365 | 1 | 0.30471 | 1.23517 | 1.22181 | 0.03163 | 0 | 0.20431 | 1.15213 | 1.68532 | 0.10611 | 1 | 0 |
| 1368916_at | Asl | argininosuccinate lyase | Any2_257 | 0.03 | -0.40 | -0.35 | 0.00 | -0.10 | 0 | 0.04805 | 1.03387 | 1.2269 | 0.26496 | 1 | 0.39925 | 1.31882 | 0.63625 | 0.00426 | 1 | 0.29822 | 1.22963 | 2.24823 | 0.01511 | 0 | 0 |
| 1369003_at | Dedd | death effector domain-containing | Any2_257 | -0.21 | -0.25 | 0.05 | 0.08 | -0.14 | 1 | 0.30102 | 1.23201 | 2.2924 | 0.01231 | 1 | 0.33231 | 1.25903 | 0.84854 | 0.0084 | 0 | 0.10549 | 1.07586 | 1.8672 | 0.07901 | 0 | 0 |
| 1369036_at | Grik2 | glutamate receptor, ionotropic, kainate 2 | Any2_257 | -0.17 | -0.38 | -0.11 | -0.09 | -0.31 | 1 | 0.26989 | 1.20572 | 2.28857 | 0.01906 | 1 | 0.28753 | 1.22055 | 1.16183 | 0.02331 | 0 | 0.07024 | 1.04989 | 1.28205 | 0.23168 | 0 | 0 |
| 1369132_at | Slc18a2 | solute carrier family 18 (vesicular monoamine), mer | Any2_257 | -0.04 | 0.01 | -0.32 | -0.28 | -0.11 | 1 | -0.3372 | -1.2633 | 3.47455 | 0.00983 | 1 | -0.289 | -1.2218 | 1.66083 | 0.02889 | 0 | -0.1181 | -1.0853 | 1.33281 | 0.18013 | 0 | 0 |
| 1369368_at | Trpc5 | transient receptor potential cation channel, subfam | Any2_257 | -0.08 | 0.17 | 0.05 | -0.13 | -0.14 | 0 | -0.1175 | -1.0849 | 1.49565 | 0.17674 | 1 | -0.2962 | -1.2279 | 1.91866 | 0.01888 | 1 | -0.3135 | -1.2427 | 2.97223 | 0.01242 | 0 | 0 |
| 1369410_at | --- | --- | Any2_257 | -0.21 | -0.98 | -0.70 | -0.40 | -0.74 | 1 | 0.27859 | 1.21301 | 2.8175 | 0.00217 | 1 | 0.58156 | 1.49647 | 0.05068 | 9.6E-05 | 0 | 0.24026 | 1.18121 | 1.28362 | 0.00285 | 0 | 0 |
| 1369517_at | Cyth1 | cytohesin 1 | Any2_257 | 0.08 | -0.04 | 0.06 | 0.24 | 0.21 | 0 | 0.10678 | 1.07682 | 1.45861 | 0.17218 | 1 | 0.28333 | 1.217 | 1.20391 | 0.02869 | 0 | 0.25609 | 1.19423 | 2.39306 | 0.02739 | 1 | 0 |
| 1369574_at | Tas2r13 | taste receptor, type 2, member 13 | Any2_257 | -0.01 | 0.14 | -0.10 | -0.19 | -0.14 | 0 | -0.241 | -1.1818 | 2.55372 | 0.04853 | 1 | -0.326 | -1.2535 | 2.02842 | 0.01689 | 1 | -0.2748 | -1.2098 | 2.57813 | 0.02785 | 0 | 0 |
| 1369718_at | Ssr3 | signal sequence receptor, gamma | Any2_257 | -0.24 | -0.50 | -0.15 | 0.00 | -0.32 | 1 | 0.34743 | 1.2723 | 2.59208 | 0.00442 | 1 | 0.4921 | 1.4065 | 0.42051 | 0.00184 | 0 | 0.18087 | 1.13356 | 1.91959 | 0.0703 | 0 | 0 |
| 1370000_at | Nucb2 | nucleobindin 2 | Any2_257 | -0.06 | -0.17 | -0.47 | 0.07 | -0.44 | 1 | -0.2926 | -1.2249 | 3.09321 | 0.02785 | 0 | 0.24144 | 1.18217 | 1.27318 | 0.0861 | 1 | -0.2683 | -1.2044 | 2.8982 | 0.01295 | 0 | 0 |
| 1370083_at | Ccr1 | chemokine (C-C motif) receptor 1 | Any2_257 | 0.02 | -0.69 | -0.44 | 1.18 | 0.35 | 0 | 0.2493 | 1.18863 | 2.29631 | 0.0178 | 1 | 1.87054 | 3.65669 | 0 | 0 | 1 | 1.04382 | 2.06168 | 0 | 0 | 1 | 0 |
| 1370154_at | Lyz2 | lysozyme 2 | Any2_257 | -0.14 | -0.77 | -0.72 | 0.20 | 0.03 | 0 | 0.05059 | 1.03569 | 1.88086 | 0.06217 | 1 | 0.96605 | 1.95348 | 0.00045 | 3.2E-07 | 1 | 0.7972 | 1.73772 | 0.00286 | 1.3E-06 | 1 | 0 |
| 1370234_at | Fn1 | fibronectin 1 | Any2_257 | -0.08 | -0.45 | -0.40 | 1.50 | 0.89 | 0 | 0.05385 | 1.03803 | 1.40802 | 0.19056 | 1 | 1.95519 | 3.87768 | 0 | 0 | 1 | 1.34399 | 2.53853 | 0 | 0 | 1 | 0 |
| 1370244_at | Ctsl1 | cathepsin L1 | Any2_257 | -0.09 | -0.52 | -0.58 | 0.11 | -0.18 | 0 | -0.0573 | -1.0405 | 1.2919 | 0.24161 | 1 | 0.6276 | 1.545 | 0.05107 | 9.2E-05 | 1 | 0.33627 | 1.26249 | 2.14375 | 0.01048 | 1 | 0 |
| 1370281_at | Fabp5 | fatty acid binding protein 5, epidermal | Any2_257 | -0.15 | -0.62 | -0.63 | -0.02 | -0.34 | 0 | -0.0036 | -1.0025 | 1.36093 | 0.2083 | 1 | 0.60804 | 1.52418 | 0.0514 | 9.4E-05 | 1 | 0.28415 | 1.21769 | 2.24472 | 0.01393 | 1 | 0 |
| 1370283_at | Hspa5 | heat shock protein 5 | Any2_257 | -0.04 | -0.15 | -0.14 | 0.19 | 0.17 | 0 | 0.0079 | 1.00549 | 0.89074 | 0.62442 | 1 | 0.34202 | 1.26753 | 1.09318 | 0.01947 | 1 | 0.31784 | 1.24646 | 2.34764 | 0.01917 | 1 | 0 |
| 1370348_at | Ninj1 | ninjurin 1 | Any2_257 | 0.04 | -0.51 | -0.32 | 0.05 | -0.04 | 0 | 0.18783 | 1.13905 | 1.86112 | 0.06481 | 1 | 0.55782 | 1.47205 | 0.12049 | 0.00031 | 1 | 0.46418 | 1.37953 | 0.46936 | 0.00071 | 0 | 0 |
| 1370382_at | RT1-Db1 | RT1 class II, locus Db1 | Any2_257 | -0.19 | 0.17 | 0.22 | -0.20 | -0.04 | 0 | 0.04906 | 1.03459 | 0.97322 | 0.48087 | 1 | -0.3717 | -1.2939 | 2.40868 | 0.00999 | 0 | -0.2022 | -1.1504 | 1.7436 | 0.08292 | 1 | 0 |
| 1370388_at | Slc9a5 | solute carrier family 9, subfamily A (NHE5, cation pr | Any2_257 | -0.08 | 0.22 | -0.09 | -0.11 | -0.16 | 1 | -0.3189 | -1.2474 | 3.28132 | 0.01361 | 1 | -0.3302 | -1.2572 | 1.76125 | 0.02441 | 1 | -0.3883 | -1.3089 | 3.06824 | 0.00503 | 0 | 0 |
| 1370442_at | Tmsbl1 | thymosin beta-like protein 1 | Any2_257 | -0.10 | -0.67 | -0.65 | -0.40 | -0.40 | 0 | 0.02247 | 1.0157 | 1.92782 | 0.05585 | 1 | 0.27328 | 1.20855 | 1.0479 | 0.01671 | 1 | 0.27237 | 1.20779 | 1.89566 | 0.00646 | 0 | 0 |
| 1370493_a_at | Lilrb3l /// LO | leukocyte immunoglobulin-like receptor, subfamily | Any2_257 | 0.16 | -0.08 | 0.08 | 0.77 | 0.41 | 0 | 0.15851 | 1.11613 | 1.52559 | 0.15144 | 1 | 0.84775 | 1.79969 | 0.00516 | 5.1E-06 | 1 | 0.489 | 1.40347 | 0.42159 | 0.0006 | 0 | 0 |
| 1370559_at | Kcnc2 | potassium voltage gated channel, Shaw-related sub | Any2_257 | 0.02 | 0.18 | -0.02 | -0.18 | -0.09 | 0 | -0.2022 | -1.1504 | 3.06824 | 0.02861 | 1 | -0.3633 | -1.2864 | 2.22609 | 0.0121 | 1 | -0.268 | -1.2041 | 2.55912 | 0.02806 | 0 | 0 |
| 1370576_at | Dync1li1 | dynein cytoplasmic 1 light intermediate chain 1 | Any2_257 | -0.13 | -0.33 | 0.12 | 0.22 | -0.03 | 1 | 0.45257 | 1.36847 | 2.39143 | 0.00108 | 1 | 0.55683 | 1.47104 | 0.13189 | 0.00038 | 1 | 0.2987 | 1.23004 | 2.1449 | 0.01055 | 0 | 0 |
| 1370647_at | Impg2 | interphotoreceptor matrix proteoglycan 2 | Any2_257 | 0.22 | 0.29 | 0.06 | -0.25 | -0.05 | 0 | -0.2267 | -1.1701 | 2.75542 | 0.03774 | 1 | -0.5333 | -1.4472 | 3.55 | 0.00023 | 1 | -0.3417 | -1.2672 | 3.19093 | 0.00441 | 0 | 0 |
| 1370711_a_at | Nupl1 | nucleoporin like 1 | Any2_257 | -0.20 | -0.18 | 0.09 | 0.23 | -0.19 | 1 | 0.26938 | 1.20529 | 2.10374 | 0.02638 | 1 | 0.41106 | 1.32966 | 0.81913 | 0.00761 | 0 | -0.0047 | -1.0033 | 0.94891 | 0.51386 | 0 | 0 |
| 1370745_at | --- | --- | Any2_257 | -0.12 | -0.42 | -0.01 | -0.11 | -0.37 | 1 | 0.40726 | 1.32617 | 2.62611 | 0.00152 | 1 | 0.31088 | 1.24047 | 0.92797 | 0.01015 | 0 | 0.05227 | 1.03689 | 1.53977 | 0.14096 | 0 | 0 |
| 1370870_at | Me1 | malic enzyme 1, NADP(+)-dependent, cytosolic | Any2_257 | -0.01 | -1.05 | -0.66 | -0.48 | -0.75 | 1 | 0.39251 | 1.31268 | 2.0125 | 0.00078 | 1 | 0.57457 | 1.48923 | 0.03224 | 5.1E-05 | 1 | 0.30182 | 1.23269 | 1.476 | 0.00332 | 0 | 0 |
| 1370902_at | Akr1b8 | aldo-keto reductase family 1, member B8 | Any2_257 | 0.17 | 0.02 | 0.15 | 1.01 | 0.49 | 0 | 0.13003 | 1.09432 | 1.95226 | 0.04589 | 1 | 0.99518 | 1.99333 | 0.00048 | 3.2E-07 | 1 | 0.47146 | 1.38651 | 0.11419 | 0.00011 | 1 | 0 |
| 1371014_at | Plcb1 | phospholipase C, beta 1 (phosphoinositide-specific) | Any2_257 | 0.07 | -0.15 | -0.26 | 0.42 | 0.39 | 0 | -0.1164 | -1.084 | 1.56825 | 0.16152 | 1 | 0.5658 | 1.48021 | 0.12064 | 0.0003 | 1 | 0.53561 | 1.44955 | 0.13212 | 0.00014 | 1 | 0 |
| 1371199_at | Rapgef1 | Rap guanine nucleotide exchange factor (GEF) 1 | Any2_257 | 0.00 | 0.20 | -0.05 | -0.23 | -0.06 | 0 | -0.2481 | -1.1876 | 2.60494 | 0.04439 | 1 | -0.4278 | -1.3452 | 4.01714 | 0.00181 | 1 | -0.265 | -1.2016 | 2.63064 | 0.02656 | 0 | 0 |
| 1371232_a_at | Vcan | versican | Any2_257 | -0.12 | -0.37 | -0.64 | 1.02 | 0.42 | 1 | -0.2641 | -1.2009 | 3.445 | 0.00377 | 1 | 1.38998 | 2.62075 | 0 | 0 | 1 | 0.78633 | 1.72468 | 0.0055 | 3.5E-06 | 1 | 0 |
| 1371480_at | Cks1b | CDC28 protein kinase regulatory subunit 1B | Any2_257 | -0.02 | -0.26 | 0.01 | 0.07 | -0.19 | 1 | 0.2728 | 1.20815 | 2.05392 | 0.03976 | 1 | 0.33331 | 1.2599 | 0.96593 | 0.01258 | 0 | 0.07275 | 1.05172 | 1.02316 | 0.405 | 0 | 0 |
| 1371703_at | Ahnak | AHNAK nucleoprotein | Any2_257 | 0.11 | 0.57 | 0.71 | 0.85 | 0.71 | 0 | 0.13651 | 1.09924 | 1.71568 | 0.09682 | 1 | 0.27636 | 1.21114 | 1.17318 | 0.0249 | 0 | 0.13546 | 1.09845 | 1.80987 | 0.08555 | 1 | 0 |
| 1372031_at | --- | --- | Any2_257 | 0.16 | -0.04 | 0.11 | 0.49 | 0.28 | 0 | 0.14536 | 1.10601 | 1.81109 | 0.0714 | 1 | 0.5221 | 1.43605 | 0.20188 | 0.00066 | 1 | 0.32049 | 1.24876 | 1.81863 | 0.00596 | 0 | 0 |
| 1372064_at | Cxcl16 | chemokine (C-X-C motif) ligand 16 | Any2_257 | -0.09 | -0.25 | -0.22 | 0.10 | -0.08 | 0 | 0.02906 | 1.02034 | 0.95919 | 0.49642 | 1 | 0.34978 | 1.27437 | 0.96346 | 0.0118 | 0 | 0.17595 | 1.12971 | 1.65734 | 0.11511 | 1 | 0 |
| 1372176_at | Prkca | protein kinase C, alpha | Any2_257 | 0.38 | 0.43 | 0.11 | 0.24 | 0.15 | 1 | -0.3247 | -1.2524 | 3.27797 | 0.01507 | 0 | -0.1892 | -1.1401 | 1.08127 | 0.15319 | 1 | -0.2854 | -1.2188 | 2.9817 | 0.01409 | 0 | 0 |

| Affymetrix Probeset ID | Gene Symbol | Gene Title | Figure 6 B Venn n =2021 | Mean BN log2 intensity autologous n=8 | Mean log2 intensity day 30 DR +/+ ND n=4 | Mean log2 intensity day 30 DR+/+ HCD n=4 | Mean log2 intensity day 30 DR+/+ B/S n=4 | Mean log2 intensity day 30 F+/+ ND n=4 | DR+/+ HCD vs DR+/+ ND n=380 1=yes 0=no | Log2 ratio DR+/+ HCD vs DR+/+ ND | Fold-Change DR+/+ HCD vs DR+/+ ND | RP-FDR 4DR30 HCD vs 4DR30 Reg | p value DR+/+ HCD vs DR+/+ ND | DR+/+ B/S vs DR+/+ ND n=1,330 1=yes 0=no | Log2 ratio DR+/+ B/S vs DR+/+ ND | Fold-Change DR+/+ B/S vs DR+/+ ND | RP-FDR 4DR30 Antib vs 4DR30 Reg | p value DR+/+ B/S vs DR+/+ ND | DR+/+ ND vs F+/+ ND n=398 1=yes 0=no | Log2 ratio DR+/+ ND vs F+/+ ND | Fold-Change DR+/+ ND vs F+/+ ND | RP-FDR 4RT1uu 30Reg vs 4DR30 Reg | p value DR+/+ ND vs F+/+ ND | DR+/+ Longitudinal n=228 1=yes 0=no | Figure 6F |
|---|---|---|---|---|---|---|---|---|---|---|---|---|---|---|---|---|---|---|---|---|---|---|---|---|---|
| 1372665_at | Psat1 | phosphoserine aminotransferase 1 | Any2_257 | -0.17 | -0.16 | -0.18 | 0.19 | -0.12 | 0 | -0.0118 | -1.0082 | 1.19454 | 0.28585 | 1 | 0.35358 | 1.27772 | 0.9657 | 0.01192 | 0 | 0.04744 | 1.03343 | 1.17475 | 0.28516 | 1 | 0 |
| 1373035_at | --- | --- | Any2_257 | 0.15 | -0.25 | -0.20 | 0.06 | 0.15 | 0 | 0.05234 | 1.03694 | 1.57837 | 0.13546 | 1 | 0.30497 | 1.23539 | 1.0108 | 0.01544 | 1 | 0.39701 | 1.31677 | 0.64423 | 0.00108 | 0 | 0 |
| 1373206_at | Fndc3b | fibronectin type III domain containing 3B | Any2_257 | 0.03 | -0.19 | -0.17 | 0.32 | 0.28 | 0 | 0.01991 | 1.0139 | 0.89287 | 0.61837 | 1 | 0.51477 | 1.42877 | 0.27849 | 0.00095 | 1 | 0.47025 | 1.38535 | 0.45804 | 0.00068 | 0 | 0 |
| 1373889_at | LOC363715 /// | similar to CLM3 /// similar to dendritic cell-derived i | Any2_257 | -0.01 | -0.34 | -0.43 | 0.50 | 0.14 | 0 | -0.0894 | -1.0639 | 1.86351 | 0.11565 | 1 | 0.83631 | 1.78547 | 0.005 | 4.8E-06 | 1 | 0.48187 | 1.39655 | 0.40535 | 0.00056 | 1 | 0 |
| 1374061_at | Cd302 | CD302 molecule | Any2_257 | -0.22 | -0.38 | -0.35 | 0.32 | 0.13 | 0 | 0.03475 | 1.02438 | 1.51623 | 0.15368 | 1 | 0.7058 | 1.63104 | 0.02114 | 3E-05 | 1 | 0.51163 | 1.42566 | 0.20611 | 0.00024 | 1 | 0 |
| 1374273_at | --- | --- | Any2_257 | -0.05 | -0.13 | 0.01 | 0.28 | 0.17 | 0 | 0.13493 | 1.09804 | 1.66975 | 0.11399 | 1 | 0.41147 | 1.33004 | 0.81771 | 0.0077 | 1 | 0.29597 | 1.22771 | 2.30479 | 0.01593 | 0 | 0 |
| 1374334_at | Igha /// LOC3 | immunoglobulin heavy chain, alpha /// similar to im | Any2_257 | -0.07 | -0.22 | -0.87 | -0.46 | -0.21 | 1 | -0.653 | -1.5725 | 0.15 | 4.8E-06 | 0 | -0.2422 | -1.1828 | 2.7575 | 0.00638 | 0 | 0.01165 | 1.00811 | 1.47285 | 0.13597 | 1 | 0 |
| 1374529_at | --- | --- | Any2_257 | 0.16 | -1.31 | -1.13 | 0.36 | 0.09 | 0 | 0.18769 | 1.13894 | 2.20219 | 0.02195 | 1 | 1.67301 | 3.1888 | 0 | 0 | 1 | 1.40183 | 2.64236 | 0 | 0 | 0 | 0 |
| 1374719_at | Uhrf1bp1 | UHRF1 binding protein 1 | Any2_257 | 0.07 | 0.03 | 0.29 | 0.31 | 0.29 | 1 | 0.26406 | 1.20085 | 1.97796 | 0.04738 | 1 | 0.27712 | 1.21177 | 1.26525 | 0.04012 | 1 | 0.26467 | 1.20136 | 2.32948 | 0.03453 | 0 | 0 |
| 1374976_a_at | Soat1 | sterol O-acyltransferase 1 | Any2_257 | -0.07 | -0.56 | -0.55 | 0.06 | -0.15 | 0 | 0.00991 | 1.00689 | 1.10464 | 0.34646 | 1 | 0.62311 | 1.54019 | 0.07179 | 0.00015 | 1 | 0.41367 | 1.33207 | 1.18098 | 0.00232 | 0 | 0 |
| 1375010_at | Cd68 | Cd68 molecule | Any2_257 | 0.01 | -0.16 | 0.03 | 0.01 | 0.12 | 0 | 0.1855 | 1.13721 | 1.63022 | 0.12277 | 0 | 0.16162 | 1.11854 | 1.24239 | 0.11573 | 1 | 0.27997 | 1.21417 | 2.37297 | 0.02388 | 1 | 0 |
| 1375043_at | --- | --- | Any2_257 | -0.03 | -0.97 | -0.90 | 0.44 | -0.18 | 0 | 0.07093 | 1.05039 | 2.42101 | 0.00771 | 1 | 1.41859 | 2.67325 | 0 | 0 | 1 | 0.7993 | 1.74026 | 0.00308 | 1.3E-06 | 1 | 0 |
| 1375104_at | Ppp1r14b | protein phosphatase 1, regulatory (inhibitor) subuni | Any2_257 | 0.05 | -0.19 | 0.28 | 0.01 | 0.14 | 1 | 0.46545 | 1.38074 | 2.06429 | 0.00046 | 0 | 0.199 | 1.1479 | 1.28573 | 0.05805 | 1 | 0.32869 | 1.25587 | 1.6944 | 0.00458 | 0 | 0 |
| 1375191_at | LOC1009122( | uncharacterized LOC100912205 | Any2_257 | -0.19 | -0.40 | -0.06 | -0.04 | -0.37 | 1 | 0.33962 | 1.26542 | 2.41468 | 0.00481 | 1 | 0.36701 | 1.28968 | 0.72406 | 0.00591 | 0 | 0.0295 | 1.02066 | 1.47143 | 0.15827 | 0 | 0 |
| 1375234_at | Ncoa5 | nuclear receptor coactivator 5 | Any2_257 | -0.22 | -0.42 | -0.07 | -0.21 | -0.13 | 1 | 0.35133 | 1.27573 | 2.3159 | 0.01072 | 0 | 0.21555 | 1.16115 | 1.27327 | 0.04692 | 1 | 0.29442 | 1.22639 | 2.23356 | 0.01393 | 0 | 0 |
| 1375292_at | --- | --- | Any2_257 | 0.06 | 0.02 | -0.29 | -0.25 | -0.09 | 1 | -0.3078 | -1.2378 | 3.34174 | 0.013 | 1 | -0.2676 | -1.2038 | 1.43548 | 0.04874 | 0 | -0.1074 | -1.0773 | 1.12348 | 0.29793 | 0 | 0 |
| 1375385_at | --- | --- | Any2_257 | -0.07 | -0.16 | 0.22 | 0.29 | -0.03 | 1 | 0.37519 | 1.29701 | 2.94533 | 0.00284 | 1 | 0.45353 | 1.36939 | 0.48503 | 0.00229 | 0 | 0.1288 | 1.09339 | 1.69353 | 0.10483 | 0 | 0 |
| 1375532_at | Id2 | inhibitor of DNA binding 2 | Any2_257 | -0.10 | -0.38 | -0.10 | 0.26 | -0.19 | 1 | 0.28024 | 1.2144 | 2.19549 | 0.02174 | 1 | 0.63764 | 1.55578 | 0.04782 | 8.5E-05 | 0 | 0.18746 | 1.13875 | 1.99574 | 0.06372 | 0 | 0 |
| 1375612_at | Hnrnpa1 | heterogeneous nuclear ribonucleoprotein A1 | Any2_257 | -0.12 | -0.56 | -0.23 | -0.26 | -0.31 | 1 | 0.33014 | 1.25714 | 2.33164 | 0.00825 | 1 | 0.29392 | 1.22597 | 1.0161 | 0.01547 | 0 | 0.24557 | 1.18556 | 2.23189 | 0.01292 | 0 | 0 |
| 1375796_at | Igtp /// Irgm2 | interferon gamma induced GTPase /// immunity-rel | Any2_257 | 0.19 | 0.19 | 0.39 | 0.57 | 0.23 | 0 | 0.20499 | 1.15268 | 2.31021 | 0.01077 | 1 | 0.37887 | 1.30032 | 0.55344 | 0.00279 | 0 | 0.04417 | 1.03109 | 2.17557 | 0.04736 | 1 | 0 |
| 1375917_at | Lilrb4 | leukocyte immunoglobulin-like receptor, subfamily | Any2_257 | -0.18 | -0.67 | -0.73 | 0.06 | 0.00 | 0 | -0.0585 | -1.0414 | 2.0241 | 0.09086 | 1 | 0.73142 | 1.66027 | 0.01405 | 1.9E-05 | 1 | 0.67193 | 1.59321 | 0.01679 | 1.5E-05 | 0 | 0 |
| 1376089_at | --- | --- | Any2_257 | -0.03 | 0.06 | -0.01 | 0.72 | 0.58 | 0 | -0.0684 | -1.0485 | 1.59663 | 0.15505 | 1 | 0.66623 | 1.58692 | 0.04451 | 7.3E-05 | 1 | 0.52406 | 1.43799 | 0.20676 | 0.00025 | 0 | 0 |
| 1376175_at | Gbas | glioblastoma amplified sequence | Any2_257 | -0.26 | -0.44 | -0.11 | -0.06 | -0.46 | 1 | 0.33804 | 1.26404 | 2.48 | 0.00455 | 1 | 0.38142 | 1.30262 | 0.76112 | 0.00656 | 0 | -0.0154 | -1.0107 | 1.46778 | 0.13772 | 1 | 0 |
| 1376283_at | --- | --- | Any2_257 | 0.08 | 0.16 | 0.18 | -0.15 | -0.11 | 0 | 0.01404 | 1.00978 | 0.91986 | 0.52519 | 1 | -0.3109 | -1.2405 | 1.70028 | 0.02739 | 1 | -0.2742 | -1.2093 | 2.22672 | 0.03823 | 0 | 0 |
| 1376310_at | --- | --- | Any2_257 | 0.12 | 0.27 | -0.06 | -0.04 | 0.13 | 1 | -0.3316 | -1.2584 | 3.38297 | 0.0099 | 1 | -0.3152 | -1.2442 | 1.74955 | 0.02475 | 0 | -0.1475 | -1.1076 | 1.70755 | 0.09109 | 0 | 0 |
| 1376317_at | Orc6 | origin recognition complex, subunit 6 | Any2_257 | -0.33 | -0.41 | -0.15 | -0.05 | -0.35 | 1 | 0.26892 | 1.20491 | 2.05137 | 0.04004 | 1 | 0.36219 | 1.28538 | 1.00855 | 0.01547 | 0 | 0.06653 | 1.04719 | 1.01399 | 0.4166 | 0 | 0 |
| 1376688_a_at | LOC1009104S | paired immunoglobulin-like type 2 receptor alpha-li | Any2_257 | -0.11 | -0.68 | -0.43 | 0.19 | 0.09 | 0 | 0.2517 | 1.19061 | 2.22372 | 0.01652 | 1 | 0.87625 | 1.83559 | 0.00414 | 3.9E-06 | 1 | 0.76983 | 1.70507 | 0.00583 | 4.5E-06 | 1 | 0 |
| 1376900_at | Aptx | aprataxin | Any2_257 | -0.25 | -0.05 | -0.20 | -0.33 | -0.32 | 0 | -0.1493 | -1.109 | 1.42932 | 0.1923 | 1 | -0.2808 | -1.2148 | 1.6175 | 0.03303 | 1 | -0.2699 | -1.2057 | 2.52199 | 0.02855 | 0 | 0 |
| 1376998_a_at | Arhgef40 | Rho guanine nucleotide exchange factor (GEF) 40 | Any2_257 | 0.05 | 0.12 | -0.21 | -0.24 | -0.02 | 1 | -0.3293 | -1.2564 | 3.61542 | 0.00558 | 1 | -0.3615 | -1.2847 | 2.74974 | 0.0069 | 0 | -0.1466 | -1.107 | 1.48228 | 0.13246 | 0 | 0 |
| 1377016_at | Creld2 | cysteine-rich with EGF-like domains 2 | Any2_257 | -0.03 | -0.19 | -0.06 | 0.27 | 0.09 | 0 | 0.12685 | 1.09191 | 1.416 | 0.1839 | 1 | 0.4558 | 1.37154 | 0.56795 | 0.00294 | 1 | 0.27979 | 1.21402 | 2.37136 | 0.02814 | 0 | 0 |
| 1377058_at | --- | --- | Any2_257 | -0.17 | -0.09 | 0.13 | 0.32 | -0.08 | 0 | 0.22373 | 1.16775 | 2.06209 | 0.03388 | 1 | 0.41053 | 1.32917 | 0.60855 | 0.00391 | 0 | 0.00999 | 1.00695 | 1.19471 | 0.27295 | 1 | 0 |
| 1377314_at | Gfpt1 | glutamine fructose-6-phosphate transaminase 1 | Any2_257 | 0.04 | 0.16 | 0.24 | 0.49 | 0.27 | 0 | 0.08089 | 1.05767 | 1.2091 | 0.27635 | 1 | 0.32142 | 1.24956 | 0.9514 | 0.01138 | 0 | 0.11092 | 1.07992 | 1.28371 | 0.23058 | 1 | 0 |
| 1377741_at | Phf20l1 | PHD finger protein 20-like 1 | Any2_257 | -0.16 | 0.12 | -0.15 | 0.11 | -0.26 | 1 | -0.2725 | -1.2079 | 3.29109 | 0.01556 | 0 | -0.0119 | -1.0083 | 1.02 | 0.23677 | 1 | -0.3836 | -1.3046 | 4.37286 | 0.00197 | 0 | 0 |
| 1378377_at | Dapp1 | dual adaptor of phosphotyrosine and 3-phosphoino | Any2_257 | -0.08 | -0.50 | -0.37 | -0.16 | -0.19 | 0 | 0.12402 | 1.08977 | 1.70613 | 0.10188 | 1 | 0.33338 | 1.25996 | 0.83815 | 0.00803 | 1 | 0.30895 | 1.2388 | 2.1549 | 0.01046 | 0 | 0 |
| 1378443_at | Slamf9 | SLAM family member 9 | Any2_257 | 0.05 | -0.01 | 0.01 | 0.37 | 0.28 | 0 | 0.02447 | 1.01711 | 1.00859 | 0.43601 | 1 | 0.38166 | 1.30284 | 0.72246 | 0.00558 | 1 | 0.29441 | 1.22639 | 2.24318 | 0.01291 | 0 | 0 |
| 1378510_at | --- | --- | Any2_257 | -0.02 | -0.04 | -0.34 | -0.35 | -0.21 | 1 | -0.2973 | -1.2288 | 3.42183 | 0.01144 | 1 | -0.3053 | -1.2356 | 2.39029 | 0.01053 | 0 | -0.1692 | -1.1244 | 1.8697 | 0.06679 | 0 | 0 |
| 1378925_at | Crem | cAMP responsive element modulator | Any2_257 | -0.10 | -0.95 | -0.84 | -0.50 | -0.68 | 0 | 0.10303 | 1.07403 | 1.49269 | 0.17377 | 1 | 0.44325 | 1.35966 | 0.57013 | 0.00293 | 1 | 0.26345 | 1.20035 | 2.36025 | 0.02429 | 0 | 0 |
| 1379161_at | --- | --- | Any2_257 | 0.02 | 0.20 | -0.08 | 0.02 | -0.10 | 1 | -0.2758 | -1.2107 | 3.09722 | 0.0244 | 0 | -0.1787 | -1.1318 | 1.10997 | 0.1378 | 1 | -0.301 | -1.232 | 2.96926 | 0.01795 | 0 | 0 |
| 1379181_at | --- | --- | Any2_257 | -0.21 | -0.25 | 0.06 | 0.05 | -0.17 | 1 | 0.30883 | 1.2387 | 2.52731 | 0.00544 | 1 | 0.29959 | 1.2308 | 1.13835 | 0.02171 | 0 | 0.07332 | 1.05214 | 1.53699 | 0.14313 | 0 | 0 |
| 1379293_at | Gzma | granzyme A | Any2_257 | -0.04 | -0.69 | -0.44 | -0.42 | -0.36 | 0 | 0.24478 | 1.18491 | 2.31631 | 0.01169 | 1 | 0.26578 | 1.20229 | 1.17362 | 0.02536 | 1 | 0.32667 | 1.25412 | 1.5572 | 0.00376 | 0 | 0 |
| 1379458_at | Klhl14 | kelch-like family member 14 | Any2_257 | -0.06 | -0.02 | -0.07 | 0.31 | -0.02 | 0 | -0.0543 | -1.0384 | 1.13612 | 0.31728 | 1 | 0.3285 | 1.25571 | 1.14401 | 0.02192 | 0 | 0.00399 | 1.00277 | 1.0701 | 0.33859 | 1 | 0 |
| 1379482_at | Tm6sf1 | transmembrane 6 superfamily member 1 | Any2_257 | 0.13 | -0.38 | -0.33 | 0.22 | -0.08 | 0 | 0.04636 | 1.03266 | 2.13927 | 0.02442 | 1 | 0.59729 | 1.51287 | 0.12289 | 0.00033 | 1 | 0.29921 | 1.23047 | 2.09522 | 0.00775 | 0 | 0 |
| 1379497_at | --- | --- | Any2_257 | -0.07 | -0.81 | -0.91 | -0.20 | -0.05 | 0 | -0.0929 | -1.0665 | 3.10328 | 0.02345 | 1 | 0.61237 | 1.52877 | 0.05365 | 0.00011 | 1 | 0.76449 | 1.69877 | 0.00524 | 3.5E-06 | 0 | 0 |
| 1379677_at | Tnfsf13 | tumor necrosis factor (ligand) superfamily, member | Any2_257 | 0.04 | -0.10 | -0.26 | 0.32 | 0.16 | 0 | -0.1564 | -1.1145 | 1.78553 | 0.12488 | 1 | 0.42385 | 1.3415 | 0.57409 | 0.00334 | 0 | 0.25838 | 1.19614 | 2.28499 | 0.03828 | 1 | 0 |
| 1379682_at | --- | --- | Any2_257 | -0.05 | -0.41 | -0.13 | -0.27 | -0.04 | 1 | 0.27494 | 1.20994 | 2.05237 | 0.03564 | 0 | 0.13814 | 1.10049 | 1.13918 | 0.22447 | 1 | 0.36768 | 1.29027 | 1.71827 | 0.00541 | 0 | 0 |
| 1379763_at | --- | --- | Any2_257 | -0.15 | -0.06 | 0.23 | 0.32 | -0.06 | 1 | 0.2968 | 1.22842 | 2.25841 | 0.02012 | 1 | 0.38113 | 1.30236 | 0.82963 | 0.00787 | 0 | 0.00114 | 1.00079 | 1.33279 | 0.18111 | 0 | 0 |
| 1379830_at | --- | --- | Any2_257 | -0.24 | 0.12 | -0.22 | 0.01 | -0.14 | 1 | -0.3439 | -1.2692 | 3.39306 | 0.01069 | 0 | -0.1121 | -1.0808 | 1.05504 | 0.176 | 1 | -0.2634 | -1.2003 | 2.44808 | 0.02999 | 0 | 0 |
| 1379861_at | Dcakd | dephospho-CoA kinase domain containing | Any2_257 | 0.01 | 0.02 | 0.17 | -0.30 | -0.27 | 0 | 0.15284 | 1.11176 | 1.35156 | 0.21174 | 1 | -0.3155 | -1.2445 | 1.79692 | 0.02213 | 1 | -0.294 | -1.226 | 3.04812 | 0.01666 | 0 | 0 |
| 1379996_at | --- | --- | Any2_257 | -0.29 | -0.65 | -0.38 | -0.19 | -0.53 | 1 | 0.26889 | 1.20488 | 2.5588 | 0.00617 | 1 | 0.45435 | 1.37017 | 0.4246 | 0.00187 | 0 | 0.1196 | 1.08644 | 2.37029 | 0.03155 | 0 | 0 |
| 1380084_at | --- | --- | Any2_257 | 0.04 | 0.04 | 0.48 | 0.73 | 0.02 | 1 | 0.43693 | 1.35372 | 2.488 | 0.0012 | 1 | 0.69476 | 1.61862 | 0.05138 | 9.6E-05 | 0 | -0.0161 | -1.0112 | 1.00508 | 0.4235 | 0 | 0 |
| 1380334_at | Rbm47 | RNA binding motif protein 47 | Any2_257 | 0.02 | -0.12 | -0.15 | 0.47 | 0.34 | 0 | -0.0327 | -1.0229 | 1.03434 | 0.393 | 1 | 0.58924 | 1.50445 | 0.13057 | 0.00037 | 1 | 0.45535 | 1.37111 | 0.71519 | 0.00124 | 0 | 0 |
| 1380529_at | --- | --- | Any2_257 | -0.42 | -0.23 | -0.56 | -0.14 | -0.62 | 1 | -0.326 | -1.2535 | 3.36162 | 0.012 | 0 | 0.09239 | 1.06613 | 1.10726 | 0.26269 | 1 | -0.3866 | -1.3073 | 3.78692 | 0.00317 | 0 | 0 |
| 1380531_at | Slc9a9 | solute carrier family 9, subfamily A (NHE9, cation pr | Any2_257 | 0.08 | -0.32 | 0.00 | 0.05 | -0.08 | 1 | 0.32047 | 1.24874 | 2.37439 | 0.00939 | 1 | 0.3727 | 1.29477 | 0.91941 | 0.00949 | 0 | 0.24388 | 1.18418 | 2.27749 | 0.03793 | 0 | 0 |
| 1380651_at | Rnf6 | ring finger protein (C3H2C3 type) 6 | Any2_257 | 0.04 | 0.00 | 0.33 | 0.37 | 0.05 | 1 | 0.33058 | 1.25752 | 2.25765 | 0.01423 | 1 | 0.36164 | 1.28489 | 0.92598 | 0.00971 | 0 | 0.04629 | 1.03261 | 1.17807 | 0.28388 | 0 | 0 |
| 1381044_at | --- | --- | Any2_257 | -0.15 | 0.02 | -0.12 | -0.35 | -0.25 | 0 | -0.1346 | -1.0978 | 2.04984 | 0.08806 | 1 | -0.3719 | -1.2941 | 3.10294 | 0.00509 | 1 | -0.2709 | -1.2065 | 3.02923 | 0.01393 | 0 | 0 |
| 1381133_at | --- | --- | Any2_257 | -0.13 | -0.45 | -0.33 | 0.37 | -0.12 | 0 | 0.12125 | 1.08767 | 1.67816 | 0.11165 | 1 | 0.82388 | 1.77016 | 0.00346 | 2.9E-06 | 1 | 0.33069 | 1.25761 | 2.19078 | 0.01176 | 0 | 0 |
| 1381274_at | --- | --- | Any2_257 | -0.15 | -0.02 | -0.29 | -0.06 | -0.32 | 1 | -0.2708 | -1.2065 | 3.5493 | 0.00982 | 0 | -0.0406 | -1.0286 | 1.11996 | 0.13364 | 1 | -0.308 | -1.238 | 3.28094 | 0.00559 | 0 | 0 |
| 1381311_at | Emr1 | EGF-like module containing, mucin-like, hormone re | Any2_257 | -0.10 | -0.48 | -0.48 | -0.78 | -0.05 | 0 | -0.0048 | -1.0033 | 1.75614 | 0.08905 | 1 | -0.3029 | -1.2336 | 2.93633 | 0.00567 | 1 | 0.4242 | 1.34183 | 0.46735 | 0.00074 | 0 | 0 |
| 1381628_at | --- | --- | Any2_257 | -0.02 | 0.19 | 0.52 | 0.51 | 0.44 | 1 | 0.33516 | 1.26152 | 2.955 | 0.00209 | 1 | 0.32361 | 1.25146 | 0.92395 | 0.00977 | 0 | 0.25115 | 1.19015 | 2.17304 | 0.01104 | 0 | 0 |
| 1381662_at | --- | --- | Any2_257 | 0.15 | 0.24 | -0.02 | 0.08 | -0.08 | 1 | -0.2668 | -1.2032 | 3.21585 | 0.01696 | 0 | -0.1602 | -1.1174 | 1.39799 | 0.05322 | 1 | -0.3261 | -1.2536 | 3.03018 | 0.01091 | 0 | 0 |
| 1381679_at | --- | --- | Any2_257 | -0.01 | -0.03 | -0.11 | -0.35 | -0.33 | 0 | -0.0827 | -1.059 | 1.17438 | 0.29704 | 1 | -0.3166 | -1.2454 | 1.77742 | 0.02418 | 1 | -0.2998 | -1.231 | 3.2312 | 0.00956 | 0 | 0 |
| 1381786_at | --- | --- | Any2_257 | -0.08 | 0.02 | 0.35 | 0.39 | 0.05 | 1 | 0.32913 | 1.25626 | 2.24365 | 0.00679 | 1 | 0.36924 | 1.29167 | 0.79877 | 0.00729 | 0 | 0.02775 | 1.01942 | 1.66477 | 0.11097 | 0 | 0 |
| 1381993_at | Clic2 | chloride intracellular channel 2 | Any2_257 | -0.17 | -0.49 | -0.20 | 0.07 | -0.24 | 1 | 0.28698 | 1.22008 | 2.10805 | 0.0261 | 1 | 0.56232 | 1.47664 | 0.15106 | 0.00046 | 1 | 0.25362 | 1.1922 | 2.28111 | 0.03579 | 0 | 0 |
| 1382078_at | Tlr8 | toll-like receptor 8 | Any2_257 | -0.01 | -0.25 | -0.14 | 0.33 | 0.09 | 0 | 0.11277 | 1.0813 | 1.59097 | 0.13224 | 1 | 0.58478 | 1.49981 | 0.11213 | 0.00027 | 1 | 0.34098 | 1.26661 | 2.08662 | 0.00892 | 0 | 0 |
| 1382105_at | Gnb5 | guanine nucleotide binding protein (G protein), beta | Any2_257 | 0.10 | -0.30 | -0.20 | 0.16 | 0.00 | 0 | 0.09806 | 1.07034 | 1.46903 | 0.16883 | 1 | 0.45809 | 1.37372 | 0.45317 | 0.00211 | 1 | 0.30166 | 1.23256 | 2.20736 | 0.01157 | 0 | 0 |
| 1382266_at | Gpr146 | G protein-coupled receptor 146 | Any2_257 | 0.02 | 0.01 | -0.11 | -0.26 | -0.29 | 0 | -0.1224 | -1.0885 | 2.1646 | 0.07649 | 1 | -0.2775 | -1.2121 | 2.08801 | 0.01383 | 1 | -0.3028 | -1.2335 | 3.33143 | 0.006 | 0 | 0 |
| 1382531_at | Tlr7 | toll-like receptor 7 | Any2_257 | -0.24 | -0.40 | -0.31 | -0.07 | -0.07 | 0 | 0.08246 | 1.05882 | 1.26688 | 0.2463 | 1 | 0.33002 | 1.25703 | 1.12082 | 0.02058 | 1 | 0.32729 | 1.25466 | 2.19921 | 0.01167 | 1 | 0 |
| 1382601_at | --- | --- | Any2_257 | 0.16 | -0.14 | -0.30 | 0.41 | 0.25 | 0 | -0.1613 | -1.1183 | 2.00569 | 0.09571 | 1 | 0.5489 | 1.46297 | 0.12143 | 0.0003 | 1 | 0.38527 | 1.3061 | 1.74978 | 0.00512 | 0 | 0 |
| 1382692_at | Clec7a | C-type lectin domain family 7, member A | Any2_257 | -0.19 | -0.58 | -0.48 | 0.17 | -0.17 | 0 | 0.09802 | 1.07031 | 2.02104 | 0.0412 | 1 | 0.74379 | 1.67457 | 0.0075 | 8.7E-06 | 1 | 0.40226 | 1.32158 | 0.64 | 0.00105 | 1 | 0 |
| 1382760_at | Slc25a32 | solute carrier family 25 (mitochondrial folate carrier | Any2_257 | -0.25 | -0.52 | -0.24 | -0.21 | -0.48 | 1 | 0.28186 | 1.21576 | 2.12549 | 0.02365 | 1 | 0.31537 | 1.24433 | 1.18223 | 0.02612 | 0 | 0.04408 | 1.03103 | 1.06756 | 0.3648 | 0 | 0 |
| 1382775_at | Ryr2 | ryanodine receptor 2, cardiac | Any2_257 | 0.08 | 0.32 | 0.05 | -0.05 | -0.05 | 1 | -0.2676 | -1.2038 | 3.311 | 0.01491 | 1 | -0.3735 | -1.2955 | 2.75108 | 0.00655 | 1 | -0.368 | -1.2905 | 2.95268 | 0.00674 | 0 | 0 |

| Affymetrix Probeset ID | Gene Symbol | Gene Title | Figure 6 B Venn n =2021 | Mean BN log2 intensity autologous n=8 | Mean log2 intensity day 30 DR +/+ ND n=4 | Mean log2 intensity day 30 DR+/+ HCD n=4 | Mean log2 intensity day 30 DR+/+ B/S n=4 | Mean log2 intensity day 30 F+/+ ND n=4 | DR+/+ HCD vs DR+/+ ND n=380 1=yes 0=no | Log2 ratio DR+/+ HCD vs DR+/+ ND | Fold-Change DR+/+ HCD vs DR+/+ ND | RP-FDR 4DR30 HCD vs 4DR30 Reg | p value DR+/+ HCD vs DR+/+ ND | DR+/+ B/S vs DR+/+ ND n=1,330 1=yes 0=no | Log2 ratio DR+/+ B/S vs DR+/+ ND | Fold-Change DR+/+ B/S vs DR+/+ ND | RP-FDR 4DR30 Antib vs 4DR30 Reg | p value DR+/+ B/S vs DR+/+ ND | DR+/+ ND vs F+/+ ND n=398 1=yes 0=no | Log2 ratio DR+/+ ND vs F+/+ ND | Fold-Change DR+/+ ND vs F+/+ ND | RP-FDR 4RT1uu 30Reg vs 4DR30 Reg | p value DR+/+ ND vs F+/+ ND | DR+/+ Longitudinal n=228 1=yes 0=no | Figure 6F |
|---|---|---|---|---|---|---|---|---|---|---|---|---|---|---|---|---|---|---|---|---|---|---|---|---|---|
| 1382847_at | Ash1l | ash1 (absent, small, or homeotic)-like (Drosophila) | Any2_257 | -0.06 | 0.03 | 0.33 | 0.36 | 0.09 | 1 | 0.30899 | 1.23884 | 2.4065 | 0.00464 | 1 | 0.33427 | 1.26074 | 0.90697 | 0.00924 | 0 | 0.06227 | 1.04411 | 1.43053 | 0.17402 | 0 | 0 |
| 1382982_at | --- | --- | Any2_257 | -0.10 | -0.10 | 0.21 | 0.22 | 0.05 | 1 | 0.3102 | 1.23988 | 2.64283 | 0.00391 | 1 | 0.31747 | 1.24614 | 0.94917 | 0.01099 | 0 | 0.14787 | 1.10793 | 2.36994 | 0.02416 | 0 | 0 |
| 1382998_at | --- | --- | Any2_257 | -0.16 | 0.16 | 0.45 | 0.57 | 0.04 | 1 | 0.29538 | 1.22721 | 2.976 | 0.00239 | 1 | 0.41184 | 1.33038 | 0.56356 | 0.00315 | 0 | -0.1214 | -1.0878 | 2.19884 | 0.03974 | 0 | 0 |
| 1383022_at | --- | --- | Any2_257 | -0.24 | -0.28 | -0.14 | 0.03 | 0.10 | 0 | 0.13382 | 1.0972 | 1.74386 | 0.09112 | 1 | 0.30298 | 1.23369 | 1.24681 | 0.03416 | 1 | 0.38084 | 1.3021 | 1.74138 | 0.00526 | 0 | 0 |
| 1383420_at | Lace1 | lactation elevated 1 | Any2_257 | -0.14 | -0.59 | -0.32 | -0.30 | -0.46 | 1 | 0.27236 | 1.20778 | 2.2726 | 0.01966 | 1 | 0.29365 | 1.22574 | 1.22554 | 0.03157 | 0 | 0.1291 | 1.09361 | 1.9348 | 0.06943 | 0 | 0 |
| 1383585_s_at | --- | --- | Any2_257 | -0.21 | -0.35 | -0.25 | 0.06 | 0.15 | 0 | 0.10429 | 1.07497 | 2.08126 | 0.03346 | 1 | 0.40959 | 1.32831 | 0.6866 | 0.00475 | 1 | 0.50125 | 1.41544 | 0.37619 | 0.00051 | 0 | 0 |
| 1383798_at | Fam151a | family with sequence similarity 151, member A | Any2_257 | 0.23 | 0.22 | 0.14 | -0.15 | -0.18 | 0 | -0.0828 | -1.0591 | 1.40704 | 0.2012 | 1 | -0.3736 | -1.2956 | 2.58464 | 0.00931 | 1 | -0.4066 | -1.3256 | 3.71647 | 0.00203 | 0 | 0 |
| 1384110_at | --- | --- | Any2_257 | -0.08 | -0.42 | -0.48 | -0.06 | -0.11 | 0 | -0.0654 | -1.0464 | 1.29125 | 0.24173 | 1 | 0.36141 | 1.28468 | 0.96155 | 0.01234 | 1 | 0.31175 | 1.24122 | 2.25245 | 0.01507 | 0 | 0 |
| 1384380_at | --- | --- | Any2_257 | -0.10 | -0.07 | 0.20 | 0.23 | -0.06 | 1 | 0.26794 | 1.20409 | 2.29504 | 0.01815 | 1 | 0.29626 | 1.22796 | 1.1689 | 0.0236 | 0 | 0.01098 | 1.00764 | 1.10698 | 0.33043 | 0 | 0 |
| 1384432_at | Zscan12 | zinc finger and SCAN domain containing 12 | Any2_257 | -0.17 | -0.60 | -0.29 | -0.24 | -0.43 | 1 | 0.31211 | 1.24152 | 2.44568 | 0.00692 | 1 | 0.35722 | 1.28096 | 0.94608 | 0.01101 | 0 | 0.16751 | 1.12312 | 2.09331 | 0.05324 | 0 | 0 |
| 1384453_at | --- | --- | Any2_257 | -0.08 | 0.00 | 0.12 | 0.28 | 0.30 | 0 | 0.12408 | 1.08981 | 1.57722 | 0.13627 | 1 | 0.27783 | 1.21237 | 1.20195 | 0.02821 | 1 | 0.30001 | 1.23116 | 2.16312 | 0.0096 | 0 | 0 |
| 1384791_at | B3gnt2 | UDP-GlcNAc:betaGal beta-1,3-N-acetylglucosaminyl | Any2_257 | 0.07 | -0.09 | 0.07 | 0.19 | -0.06 | 0 | 0.15872 | 1.1163 | 1.72086 | 0.0954 | 1 | 0.28078 | 1.21485 | 1.24428 | 0.03525 | 0 | 0.03686 | 1.02588 | 1.10081 | 0.33436 | 1 | 0 |
| 1384887_at | LOC1009115( | uncharacterized LOC100911506 | Any2_257 | 0.02 | 0.21 | 0.00 | -0.16 | -0.17 | 0 | -0.2089 | -1.1558 | 3.09504 | 0.02349 | 1 | -0.3669 | -1.2895 | 3.55727 | 0.00377 | 1 | -0.3769 | -1.2986 | 4.05158 | 0.00248 | 0 | 0 |
| 1384948_at | --- | --- | Any2_257 | -0.06 | -0.10 | 0.20 | 0.22 | 0.04 | 1 | 0.2984 | 1.22978 | 2.56907 | 0.00446 | 1 | 0.31705 | 1.24578 | 0.92833 | 0.01018 | 0 | 0.14175 | 1.10324 | 2.29534 | 0.04177 | 0 | 0 |
| 1384951_at | Col20a1 | collagen, type XX, alpha 1 | Any2_257 | 0.12 | 0.23 | 0.26 | -0.14 | -0.04 | 0 | 0.02781 | 1.01947 | 0.94396 | 0.51786 | 1 | -0.3646 | -1.2875 | 2.11869 | 0.01349 | 1 | -0.2712 | -1.2068 | 2.3376 | 0.03262 | 0 | 0 |
| 1385006_at | Atrx | alpha thalassemia/mental retardation syndrome X-l | Any2_257 | -0.06 | -0.19 | 0.14 | 0.25 | -0.39 | 1 | 0.33574 | 1.26202 | 2.132 | 0.00034 | 1 | 0.43966 | 1.35628 | 0.12464 | 0.00034 | 0 | -0.2001 | -1.1488 | 3.93696 | 0.00291 | 0 | 0 |
| 1385091_at | Med13 | mediator complex subunit 13 | Any2_257 | 0.13 | 0.06 | 0.55 | 0.58 | 0.08 | 1 | 0.48941 | 1.40387 | 0.77333 | 7.5E-05 | 1 | 0.516 | 1.42999 | 0.12113 | 0.00031 | 0 | 0.02062 | 1.01439 | 1.91099 | 0.0711 | 0 | 0 |
| 1385108_at | --- | --- | Any2_257 | -0.08 | -0.30 | 0.13 | 0.07 | -0.16 | 1 | 0.43186 | 1.34897 | 2.06636 | 0.00073 | 1 | 0.3733 | 1.29531 | 0.89869 | 0.00907 | 0 | 0.14222 | 1.1036 | 2.31358 | 0.03995 | 0 | 0 |
| 1385347_at | --- | --- | Any2_257 | 0.16 | -0.07 | 0.21 | 0.24 | 0.02 | 1 | 0.27903 | 1.21338 | 2.09196 | 0.02852 | 1 | 0.31178 | 1.24123 | 1.20161 | 0.02956 | 0 | 0.09103 | 1.06513 | 1.23237 | 0.25508 | 0 | 0 |
| 1385706_at | --- | --- | Any2_257 | -0.22 | -0.45 | -0.17 | 0.04 | -0.44 | 1 | 0.28471 | 1.21817 | 2.4421 | 0.00785 | 1 | 0.49714 | 1.41141 | 0.22343 | 0.00073 | 0 | 0.01824 | 1.01272 | 1.74694 | 0.0942 | 0 | 0 |
| 1385828_at | LOC686393 | similar to Protease-associated domain-containing pı | Any2_257 | 0.02 | -0.21 | 0.05 | 0.07 | 0.00 | 1 | 0.26869 | 1.20472 | 2.08837 | 0.0321 | 1 | 0.28712 | 1.2202 | 1.28007 | 0.044 | 0 | 0.21653 | 1.16193 | 2.36885 | 0.03054 | 0 | 0 |
| 1386064_at | Dyrk2 | dual-specificity tyrosine-(Y)-phosphorylation regulat | Any2_257 | -0.21 | -0.21 | 0.14 | 0.24 | -0.04 | 1 | 0.35045 | 1.27496 | 2.29904 | 0.00843 | 1 | 0.45239 | 1.3683 | 0.5682 | 0.00325 | 0 | 0.1707 | 1.1256 | 1.67226 | 0.10932 | 0 | 0 |
| 1386065_at | Sowahc | sosondowah ankyrin repeat domain family member | Any2_257 | 0.01 | -0.04 | 0.17 | 0.62 | 0.37 | 0 | 0.20459 | 1.15236 | 1.72887 | 0.09462 | 1 | 0.65199 | 1.57133 | 0.05833 | 0.00012 | 1 | 0.40247 | 1.32177 | 1.56351 | 0.00387 | 1 | 0 |
| 1386478_at | LOC10090969 | mitochondrial carrier triple repeat protein 1-like /// | Any2_257 | -0.02 | 0.19 | 0.50 | 0.32 | 0.47 | 1 | 0.30332 | 1.23398 | 2.62289 | 0.0032 | 0 | 0.12933 | 1.09379 | 1.27399 | 0.04711 | 1 | 0.27646 | 1.21122 | 1.74301 | 0.00521 | 0 | 0 |
| 1386621_at | --- | --- | Any2_257 | -0.20 | -0.23 | 0.08 | 0.26 | -0.02 | 1 | 0.30786 | 1.23787 | 2.2573 | 0.02098 | 1 | 0.49203 | 1.40643 | 0.42135 | 0.0018 | 0 | 0.21265 | 1.15881 | 2.06599 | 0.0572 | 0 | 0 |
| 1386836_at | Zfp574 | zinc finger protein 574 | Any2_257 | 0.10 | 0.63 | 0.22 | 0.33 | 0.68 | 1 | -0.4125 | -1.331 | 2.9775 | 0.00077 | 1 | -0.2981 | -1.2295 | 3.1196 | 0.00502 | 0 | 0.05578 | 1.03942 | 1.77674 | 0.09055 | 0 | 0 |
| 1386862_at | Anxa5 | annexin A5 | Any2_257 | -0.06 | -0.24 | -0.34 | 0.23 | 0.03 | 0 | -0.097 | -1.0695 | 1.7051 | 0.13553 | 1 | 0.47471 | 1.38964 | 0.37424 | 0.00142 | 1 | 0.26575 | 1.20226 | 2.37883 | 0.02409 | 1 | 0 |
| 1387017_at | Sqle | squalene epoxidase | Any2_257 | 0.02 | 0.06 | -0.28 | 0.18 | 0.19 | 1 | -0.3469 | -1.2718 | 3.37571 | 0.01444 | 0 | 0.11212 | 1.08082 | 1.11936 | 0.24987 | 0 | 0.12523 | 1.09068 | 1.50322 | 0.14926 | 1 | 0 |
| 1387024_at | Dusp6 | dual specificity phosphatase 6 | Any2_257 | 0.00 | -0.32 | -0.37 | 0.10 | -0.26 | 0 | -0.0556 | -1.0393 | 1.055 | 0.37147 | 1 | 0.41479 | 1.3331 | 0.61792 | 0.00401 | 0 | 0.0596 | 1.04218 | 1.0762 | 0.35457 | 1 | 0 |
| 1387033_at | LOC10090961 | mitochondrial brown fat uncoupling protein 1-like / | Any2_257 | 0.11 | 0.24 | -0.01 | -0.13 | -0.05 | 1 | -0.2487 | -1.1881 | 2.71712 | 0.03827 | 1 | -0.3706 | -1.2929 | 3.42171 | 0.00451 | 1 | -0.2823 | -1.2161 | 3.0035 | 0.01738 | 0 | 0 |
| 1387087_at | Cebpb | CCAAT/enhancer binding protein (C/EBP), beta | Any2_257 | 0.03 | -0.32 | -0.29 | 0.02 | 0.10 | 0 | 0.02191 | 1.0153 | 0.95331 | 0.50524 | 1 | 0.33739 | 1.26347 | 1.07059 | 0.01852 | 1 | 0.41569 | 1.33393 | 1.28279 | 0.0028 | 0 | 0 |
| 1387269_s_at | Plaur | plasminogen activator, urokinase receptor | Any2_257 | -0.04 | -0.20 | -0.15 | 0.46 | 0.24 | 0 | 0.04755 | 1.03351 | 1.1267 | 0.33078 | 1 | 0.6529 | 1.57233 | 0.04808 | 8E-05 | 1 | 0.43812 | 1.35484 | 0.79586 | 0.00148 | 0 | 0 |
| 1387617_at | Tpm3 | tropomyosin 3 | Any2_257 | -0.16 | -0.92 | -0.56 | -0.49 | -0.68 | 1 | 0.36038 | 1.28377 | 1.924 | 0.00062 | 1 | 0.43717 | 1.35395 | 0.24596 | 0.00082 | 0 | 0.24687 | 1.18663 | 1.20302 | 0.00244 | 0 | 0 |
| 1387687_at | Igsf6 | immunoglobulin superfamily, member 6 | Any2_257 | -0.20 | -0.63 | -0.58 | 0.25 | 0.19 | 0 | 0.05006 | 1.03531 | 1.64567 | 0.11732 | 1 | 0.88544 | 1.84733 | 0.00357 | 3.2E-06 | 1 | 0.81842 | 1.76347 | 0.00333 | 1.6E-06 | 1 | 0 |
| 1387799_at | Fxyd2 | FXYD domain-containing ion transport regulator 2 | Any2_257 | -0.01 | -0.22 | -0.29 | 0.42 | 0.00 | 0 | -0.0732 | -1.052 | 1.3972 | 0.20406 | 1 | 0.63477 | 1.55269 | 0.04849 | 8.3E-05 | 0 | 0.21992 | 1.16467 | 2.28317 | 0.03752 | 1 | 0 |
| 1387934_at | Bcan /// LOC1 | brevican /// brevican core protein-like | Any2_257 | 0.04 | 0.10 | 0.05 | -0.20 | -0.23 | 0 | -0.0546 | -1.0386 | 1.14241 | 0.31382 | 1 | -0.3026 | -1.2334 | 1.73413 | 0.02649 | 1 | -0.3375 | -1.2636 | 3.28158 | 0.00601 | 0 | 0 |
| 1387947_at | Mafb | v-maf musculoaponeurotic fibrosarcoma oncogene | Any2_257 | 0.08 | 0.03 | 0.11 | 0.77 | 0.46 | 0 | 0.07979 | 1.05686 | 1.56989 | 0.13806 | 1 | 0.73957 | 1.66968 | 0.01073 | 1.4E-05 | 1 | 0.42223 | 1.34 | 0.72482 | 0.00131 | 1 | 0 |
| 1388046_at | Itgam | integrin, alpha M | Any2_257 | 0.05 | -0.03 | -0.09 | 0.33 | 0.04 | 0 | -0.0584 | -1.0413 | 1.92977 | 0.10549 | 1 | 0.35924 | 1.28275 | 0.56561 | 0.00298 | 0 | 0.07745 | 1.05515 | 1.7836 | 0.08993 | 1 | 0 |
| 1388054_a_at | Vcan | versican | Any2_257 | -0.05 | -0.48 | -0.44 | 0.90 | 0.17 | 0 | 0.03949 | 1.02775 | 1.32302 | 0.22101 | 1 | 1.37377 | 2.59147 | 0 | 0 | 1 | 0.64412 | 1.56279 | 0.02414 | 2.3E-05 | 1 | 0 |
| 1388123_at | Psbpc2 | prostatic steroid-binding protein C2 | Any2_257 | 0.16 | 0.18 | -0.02 | -0.09 | -0.11 | 0 | -0.1965 | -1.1459 | 2.39611 | 0.06079 | 1 | -0.2698 | -1.2057 | 1.54429 | 0.03824 | 1 | -0.2825 | -1.2163 | 2.37051 | 0.0314 | 0 | 0 |
| 1388142_at | Vcan | versican | Any2_257 | -0.09 | -0.64 | -0.40 | 1.73 | 1.08 | 0 | 0.235 | 1.17691 | 2.25389 | 0.01377 | 1 | 2.37063 | 5.17165 | 0 | 0 | 1 | 1.7232 | 3.30167 | 0 | 0 | 1 | 0 |
| 1388233_at | Cish | cytokine inducible SH2-containing protein | Any2_257 | 0.16 | -0.09 | 0.09 | 0.18 | 0.29 | 0 | 0.17959 | 1.13256 | 1.83528 | 0.06911 | 1 | 0.27438 | 1.20948 | 1.20626 | 0.02855 | 1 | 0.37764 | 1.29921 | 1.54792 | 0.00358 | 0 | 0 |
| 1388265_x_at | Vcan | versican | Any2_257 | -0.04 | -0.27 | -0.22 | 0.97 | 0.42 | 0 | 0.05034 | 1.03551 | 1.95229 | 0.05267 | 1 | 1.24249 | 2.36606 | 0 | 0 | 1 | 0.69312 | 1.61677 | 0.01148 | 1E-05 | 1 | 0 |
| 1388267_a_at | Mt1a | metallothionein 1a | Any2_257 | -0.11 | -0.39 | -0.10 | -0.11 | -0.39 | 1 | 0.29236 | 1.22464 | 2.69794 | 0.00295 | 1 | 0.28276 | 1.21652 | 0.50205 | 0.00244 | 0 | 0.00025 | 1.00017 | 2.46762 | 0.02936 | 0 | 0 |
| 1388272_at | IGH-1A | --- | Any2_257 | 0.06 | -0.02 | -0.29 | 0.07 | -0.25 | 1 | -0.2698 | -1.2057 | 3.21858 | 0.01966 | 0 | 0.08865 | 1.06337 | 1.25299 | 0.10895 | 0 | -0.2329 | -1.1752 | 3.17333 | 0.0049 | 1 | 0 |
| 1388587_at | Ier3 | immediate early response 3 | Any2_257 | -0.12 | -0.55 | -0.46 | 0.40 | 0.01 | 0 | 0.08608 | 1.06148 | 1.14512 | 0.317 | 1 | 0.9493 | 1.93093 | 0 | 0 | 1 | 0.55796 | 1.47219 | 0.13313 | 0.00014 | 1 | 0 |
| 1388745_at | Sema4a | sema domain, immunoglobulin domain (Ig), transm | Any2_257 | 0.02 | 0.19 | 0.20 | 0.56 | 0.47 | 0 | 0.01328 | 1.00924 | 0.92619 | 0.54882 | 1 | 0.37314 | 1.29517 | 0.95819 | 0.01122 | 1 | 0.27951 | 1.21378 | 2.40538 | 0.02676 | 0 | 0 |
| 1388784_at | Csf1r | colony stimulating factor 1 receptor | Any2_257 | -0.16 | -0.31 | -0.20 | 0.49 | 0.11 | 0 | 0.10644 | 1.07657 | 1.4614 | 0.17067 | 1 | 0.79985 | 1.74093 | 0.00545 | 5.8E-06 | 1 | 0.42246 | 1.34021 | 0.90763 | 0.00172 | 1 | 0 |
| 1389006_at | --- | --- | Any2_257 | -0.10 | -0.10 | 0.03 | 1.20 | 0.69 | 0 | 0.12791 | 1.09271 | 1.84866 | 0.06527 | 1 | 1.29622 | 2.45585 | 0 | 0 | 1 | 0.78501 | 1.72311 | 0.00375 | 1.9E-06 | 0 | 0 |
| 1389294_at | Cyfip1 | cytoplasmic FMR1 interacting protein 1 | Any2_257 | -0.19 | -0.25 | -0.18 | 0.10 | -0.02 | 0 | 0.07667 | 1.05458 | 1.61451 | 0.12948 | 1 | 0.35022 | 1.27475 | 0.88341 | 0.00883 | 0 | 0.23414 | 1.1762 | 2.19823 | 0.01159 | 1 | 0 |
| 1389543_at | Masp1 | mannan-binding lectin serine peptidase 1 | Any2_257 | -0.02 | 0.12 | -0.18 | -0.18 | -0.01 | 1 | -0.2942 | -1.2262 | 2.94213 | 0.0315 | 1 | -0.3026 | -1.2333 | 1.83178 | 0.02115 | 0 | -0.1264 | -1.0915 | 1.22209 | 0.23295 | 0 | 0 |
| 1389553_at | Clec4a3 | C-type lectin domain family 4, member A3 | Any2_257 | -0.24 | -0.86 | -0.76 | 0.43 | 0.12 | 0 | 0.1051 | 1.07557 | 1.84721 | 0.06843 | 1 | 1.29204 | 2.44873 | 0 | 0 | 1 | 0.97721 | 1.96865 | 0 | 0 | 1 | 0 |
| 1389986_at | LOC10091241 | uncharacterized LOC100912411 | Any2_257 | -0.45 | -0.30 | 0.00 | 0.03 | -0.50 | 1 | 0.30143 | 1.23236 | 1.91222 | 0.00055 | 1 | 0.33505 | 1.26142 | 0.42871 | 0.00192 | 0 | -0.1949 | -1.1446 | 3.43703 | 0.00409 | 0 | 0 |
| 1390086_at | --- | --- | Any2_257 | -0.05 | -0.19 | 0.14 | 0.12 | 0.05 | 1 | 0.33146 | 1.25829 | 2.3857 | 0.00821 | 1 | 0.31128 | 1.24081 | 1.01556 | 0.01567 | 0 | 0.24454 | 1.18472 | 2.33727 | 0.02232 | 0 | 0 |
| 1390168_a_at | Dnajc24 | DnaJ (Hsp40) homolog, subfamily C, member 24 | Any2_257 | -0.13 | -0.34 | -0.61 | -0.23 | -0.61 | 1 | -0.2753 | -1.2103 | 3.21032 | 0.01951 | 0 | 0.1039 | 1.07467 | 1.05026 | 0.34991 | 1 | -0.2725 | -1.2079 | 2.90814 | 0.01459 | 0 | 0 |
| 1390246_at | --- | --- | Any2_257 | 0.00 | -0.19 | -0.03 | 0.12 | 0.10 | 0 | 0.1631 | 1.11969 | 1.54753 | 0.14396 | 1 | 0.31043 | 1.24008 | 1.22117 | 0.03188 | 1 | 0.29225 | 1.22454 | 2.35374 | 0.01945 | 0 | 0 |
| 1390335_at | --- | --- | Any2_257 | -0.16 | -0.37 | -0.03 | -0.01 | -0.27 | 1 | 0.34156 | 1.26712 | 2.38181 | 0.0072 | 1 | 0.35333 | 1.2775 | 0.94331 | 0.01071 | 0 | 0.09826 | 1.07048 | 1.49423 | 0.15231 | 0 | 0 |
| 1390336_at | Eva1c | eva-1 homolog C | Any2_257 | 0.05 | 0.17 | -0.10 | -0.18 | 0.17 | 1 | -0.2765 | -1.2112 | 3.25604 | 0.01665 | 1 | -0.3543 | -1.2784 | 2.54333 | 0.00957 | 0 | -0.0028 | -1.0019 | 1.25567 | 0.24347 | 0 | 0 |
| 1390383_at | Plin2 | perilipin 2 | Any2_257 | 0.02 | -0.56 | -0.48 | 0.04 | -0.11 | 0 | 0.07376 | 1.05245 | 1.58959 | 0.13295 | 1 | 0.60155 | 1.51735 | 0.05831 | 0.00012 | 1 | 0.45091 | 1.3669 | 0.4646 | 0.00075 | 0 | 0 |
| 1390546_at | --- | --- | Any2_257 | 0.10 | 0.21 | -0.05 | -0.11 | -0.14 | 0 | -0.2625 | -1.1996 | 2.88217 | 0.03244 | 1 | -0.3219 | -1.25 | 2.38529 | 0.01043 | 1 | -0.3456 | -1.2707 | 2.97786 | 0.0067 | 0 | 0 |
| 1390549_at | Adipor2 | adiponectin receptor 2 | Any2_257 | 0.15 | 0.02 | 0.44 | 0.57 | 0.21 | 1 | 0.42127 | 1.3391 | 2.44462 | 0.00102 | 1 | 0.54626 | 1.4603 | 0.16116 | 0.00049 | 0 | 0.19261 | 1.14283 | 2.28513 | 0.03549 | 0 | 0 |
| 1390866_at | --- | --- | Any2_257 | -0.13 | -0.65 | -0.53 | 0.46 | 0.23 | 0 | 0.11249 | 1.08109 | 1.15494 | 0.31047 | 1 | 1.10053 | 2.14434 | 0 | 0 | 1 | 0.87411 | 1.83288 | 0 | 0 | 0 | 0 |
| 1391222_at | N4bp1 | Nedd4 binding protein 1 | Any2_257 | 0.07 | 0.24 | 0.59 | 0.63 | 0.23 | 1 | 0.34811 | 1.27289 | 1.93875 | 0.0005 | 1 | 0.39022 | 1.3106 | 0.39389 | 0.00166 | 0 | -0.016 | -1.0111 | 2.08061 | 0.04576 | 1 | 0 |
| 1391505_x_at | --- | --- | Any2_257 | -0.17 | -0.78 | -0.90 | -0.09 | 0.01 | 0 | -0.1207 | -1.0873 | 3.38299 | 0.01055 | 1 | 0.68566 | 1.60844 | 0.0338 | 5.4E-05 | 1 | 0.78591 | 1.72418 | 0.00522 | 3.9E-06 | 0 | 0 |
| 1391626_at | Mnd1 | meiotic nuclear divisions 1 homolog (S. cerevisiae) | Any2_257 | -0.16 | 0.26 | 0.11 | 0.22 | -0.04 | 0 | -0.1524 | -1.1115 | 2.43417 | 0.05745 | 0 | -0.0347 | -1.0243 | 0.9958 | 0.28194 | 1 | -0.297 | -1.2286 | 3.05488 | 0.00825 | 1 | 0 |
| 1391735_at | --- | --- | Any2_257 | -0.21 | -0.36 | 0.03 | 0.03 | -0.16 | 1 | 0.39133 | 1.3116 | 2.544 | 0.0045 | 1 | 0.39378 | 1.31383 | 0.79259 | 0.00709 | 0 | 0.20915 | 1.156 | 1.83681 | 0.08174 | 0 | 0 |
| 1391757_at | Ptpn4 | protein tyrosine phosphatase, non-receptor type 4 | Any2_257 | -0.41 | -0.75 | -0.40 | 0.04 | -0.67 | 1 | 0.35638 | 1.28021 | 2.67438 | 0.00138 | 1 | 0.79458 | 1.73458 | 0.00865 | 1E-05 | 0 | 0.07923 | 1.05645 | 2.10788 | 0.05239 | 0 | 0 |
| 1391992_at | --- | --- | Any2_257 | 0.06 | -0.34 | -0.05 | 0.24 | -0.01 | 1 | 0.29738 | 1.22891 | 2.09495 | 0.032 | 1 | 0.5834 | 1.49837 | 0.13247 | 0.00038 | 1 | 0.3317 | 1.2585 | 2.28179 | 0.01475 | 0 | 0 |
| 1392026_at | --- | --- | Any2_257 | -0.22 | -0.11 | 0.24 | 0.22 | -0.08 | 1 | 0.35251 | 1.27678 | 2.545 | 0.00606 | 1 | 0.32926 | 1.25637 | 1.05275 | 0.01737 | 0 | 0.03617 | 1.02539 | 0.96154 | 0.47568 | 0 | 0 |
| 1392059_at | --- | --- | Any2_257 | 0.14 | -0.04 | 0.23 | -0.14 | -0.34 | 1 | 0.26926 | 1.20519 | 1.97275 | 0.04777 | 0 | -0.102 | -1.0732 | 1.01698 | 0.2381 | 1 | -0.294 | -1.226 | 3.04186 | 0.01575 | 0 | 0 |
| 1392126_at | --- | --- | Any2_257 | 0.02 | -0.23 | 0.19 | -0.16 | 0.15 | 1 | 0.42284 | 1.34056 | 2.97 | 0.00267 | 0 | 0.07234 | 1.05142 | 1.07151 | 0.30513 | 1 | 0.38341 | 1.30442 | 1.55269 | 0.00389 | 0 | 0 |

| Affymetrix Probeset ID | Gene Symbol | Gene Title | Figure 6 B Venn n =2021 | Mean BN log2 intensity autologous n=8 | Mean log2 intensity day 30 DR +/+ ND n=4 | Mean log2 intensity day 30 DR+/+ HCD n=4 | Mean log2 intensity day 30 DR+/+ B/S n=4 | Mean log2 intensity day 30 F+/+ ND n=4 | DR+/+ HCD vs DR+/+ ND n=380 1=yes 0=no | Log2 ratio DR+/+ HCD vs DR+/+ ND | Fold-Change DR+/+ HCD vs DR+/+ ND | RP-FDR 4DR30 HCD vs 4DR30 Reg | p value DR+/+ HCD vs DR+/+ ND | DR+/+ B/S vs DR+/+ ND n=1,330 1=yes 0=no | Log2 ratio DR+/+ B/S vs DR+/+ ND | Fold-Change DR+/+ B/S vs DR+/+ ND | RP-FDR 4DR30 Antib vs 4DR30 Reg | p value DR+/+ B/S vs DR+/+ ND | DR+/+ ND vs F+/+ ND n=398 1=yes 0=no | Log2 ratio DR+/+ ND vs F+/+ ND | Fold-Change DR+/+ ND vs F+/+ ND | RP-FDR 4RT1uu 30Reg vs 4DR30 Reg | p value DR+/+ ND vs F+/+ ND | DR+/+ Longitudinal n=228 1=yes 0=no | Figure 6F |
|---|---|---|---|---|---|---|---|---|---|---|---|---|---|---|---|---|---|---|---|---|---|---|---|---|---|
| 1392137_at | --- | --- | Any2_257 | -0.12 | 0.20 | -0.07 | -0.21 | -0.10 | 1 | -0.2763 | -1.2111 | 2.64448 | 0.03971 | 1 | -0.4148 | -1.3331 | 3.25311 | 0.00471 | 1 | -0.3061 | -1.2363 | 2.66941 | 0.02489 | 0 | 0 |
| 1392171_at | Chi3l1 | chitinase 3-like 1 (cartilage glycoprotein-39) | Any2_257 | 0.13 | -0.39 | -0.23 | 0.03 | 0.02 | 0 | 0.16378 | 1.12022 | 1.63365 | 0.12423 | 1 | 0.42323 | 1.34093 | 0.56185 | 0.00304 | 1 | 0.40635 | 1.32533 | 1.55257 | 0.00369 | 0 | 0 |
| 1392222_at | --- | --- | Any2_257 | -0.25 | -0.06 | -0.16 | -0.34 | -0.39 | 0 | -0.0984 | -1.0706 | 1.62742 | 0.14977 | 1 | -0.2779 | -1.2124 | 1.87281 | 0.01969 | 1 | -0.3314 | -1.2582 | 2.92077 | 0.00733 | 0 | 0 |
| 1392658_at | Tcea1 | transcription elongation factor A (SII) 1 | Any2_257 | -0.29 | -0.35 | -0.04 | -0.08 | -0.18 | 1 | 0.3123 | 1.24169 | 2.42819 | 0.0082 | 1 | 0.26821 | 1.20431 | 1.18593 | 0.02635 | 0 | 0.17526 | 1.12917 | 2.16468 | 0.04865 | 0 | 0 |
| 1392854_at | --- | --- | Any2_257 | 0.38 | 0.13 | -0.31 | 0.36 | 0.40 | 1 | -0.4386 | -1.3553 | 1.64 | 0.00011 | 0 | 0.23623 | 1.17791 | 3.6175 | 0.00372 | 1 | 0.27224 | 1.20768 | 0.46125 | 0.00071 | 0 | 0 |
| 1392972_at | Trio | triple functional domain (PTPRF interacting) | Any2_257 | -0.10 | -0.12 | 0.26 | 0.21 | 0.04 | 1 | 0.3729 | 1.29496 | 2.50045 | 0.00531 | 1 | 0.32378 | 1.2516 | 1.0072 | 0.0147 | 0 | 0.15646 | 1.11455 | 1.71396 | 0.09997 | 0 | 0 |
| 1393038_at | Fcgr1a | Fc fragment of IgG, high affinity Ia, receptor (CD64) | Any2_257 | -0.15 | -0.15 | -0.26 | 0.47 | 0.07 | 0 | -0.1078 | -1.0776 | 1.46438 | 0.18327 | 1 | 0.62096 | 1.5379 | 0.09471 | 0.00021 | 0 | 0.21992 | 1.16467 | 2.26533 | 0.0145 | 1 | 0 |
| 1393585_at | --- | --- | Any2_257 | -0.02 | 0.07 | 0.35 | 0.46 | 0.44 | 1 | 0.27586 | 1.21071 | 2.24353 | 0.01674 | 1 | 0.38445 | 1.30537 | 0.83645 | 0.0081 | 1 | 0.3658 | 1.28859 | 1.69612 | 0.00464 | 0 | 0 |
| 1393606_at | Gpcpd1 | glycerophosphocholine phosphodiesterase GDE1 ho | Any2_257 | -0.22 | -0.69 | -0.31 | -0.37 | -0.50 | 1 | 0.37419 | 1.29612 | 2.64081 | 0.00314 | 1 | 0.31691 | 1.24566 | 1.07489 | 0.01825 | 0 | 0.18342 | 1.13557 | 1.6173 | 0.12268 | 0 | 0 |
| 1393816_at | Cenpw | centromere protein W | Any2_257 | -0.04 | -0.12 | -0.42 | -0.05 | -0.42 | 1 | -0.2958 | -1.2276 | 3.13361 | 0.02318 | 0 | 0.0687 | 1.04877 | 1.03031 | 0.3905 | 1 | -0.3011 | -1.232 | 2.75344 | 0.02293 | 0 | 0 |
| 1394109_at | --- | --- | Any2_257 | 0.22 | -1.22 | -1.11 | 0.46 | 0.12 | 0 | 0.10711 | 1.07707 | 2.37398 | 0.00824 | 1 | 1.67969 | 3.2036 | 0 | 0 | 1 | 1.34119 | 2.53361 | 0 | 0 | 0 | 0 |
| 1394119_at | --- | --- | Any2_257 | 0.03 | 0.15 | 0.06 | -0.16 | -0.18 | 0 | -0.0908 | -1.065 | 1.75768 | 0.1283 | 1 | -0.3123 | -1.2417 | 2.67012 | 0.00721 | 1 | -0.3302 | -1.2572 | 3.9475 | 0.00203 | 0 | 0 |
| 1394427_at | --- | --- | Any2_257 | -0.14 | 0.15 | 0.36 | 0.59 | 0.41 | 0 | 0.21159 | 1.15796 | 2.3778 | 0.01009 | 1 | 0.44683 | 1.36304 | 0.37454 | 0.00143 | 1 | 0.26445 | 1.20118 | 1.82842 | 0.00594 | 0 | 0 |
| 1394654_at | Zfp451 | zinc finger protein 451 | Any2_257 | 0.11 | -0.13 | 0.41 | 0.53 | 0.03 | 1 | 0.53777 | 1.45173 | 0.9 | 2.9E-05 | 1 | 0.65571 | 1.57539 | 0.05297 | 0.00011 | 0 | 0.15392 | 1.11259 | 2.37608 | 0.02399 | 0 | 0 |
| 1394681_at | Akr1cl | aldo-keto reductase family 1, member C-like | Any2_257 | 0.12 | 0.23 | -0.04 | -0.07 | 0.10 | 1 | -0.2685 | -1.2045 | 2.86051 | 0.03412 | 1 | -0.2934 | -1.2255 | 2.03324 | 0.01674 | 0 | -0.1279 | -1.0927 | 1.37865 | 0.16433 | 0 | 0 |
| 1395505_at | --- | --- | Any2_257 | -0.16 | 0.21 | -0.11 | 0.03 | -0.11 | 1 | -0.3189 | -1.2474 | 3.58 | 0.00771 | 0 | -0.1773 | -1.1308 | 1.32263 | 0.06167 | 1 | -0.3127 | -1.242 | 3.03391 | 0.00624 | 0 | 0 |
| 1395530_at | --- | --- | Any2_257 | -0.11 | -0.15 | 0.12 | 0.32 | 0.00 | 1 | 0.26607 | 1.20253 | 1.99949 | 0.04301 | 1 | 0.46962 | 1.38474 | 0.5944 | 0.00352 | 0 | 0.15159 | 1.11079 | 1.69487 | 0.10507 | 0 | 0 |
| 1395740_at | --- | --- | Any2_257 | -0.04 | -0.28 | 0.05 | 0.06 | -0.25 | 1 | 0.32456 | 1.25228 | 2.26193 | 0.01542 | 1 | 0.33579 | 1.26207 | 1.1346 | 0.02127 | 0 | 0.02649 | 1.01853 | 1.17897 | 0.28258 | 0 | 0 |
| 1395766_at | --- | --- | Any2_257 | -0.03 | -0.05 | 0.25 | 0.26 | 0.05 | 1 | 0.30159 | 1.2325 | 2.28082 | 0.01966 | 1 | 0.31346 | 1.24268 | 0.96626 | 0.01246 | 0 | 0.09947 | 1.07138 | 1.53466 | 0.144 | 0 | 0 |
| 1395900_at | Chtf8 | CTF8, chromosome transmission fidelity factor 8 ho | Any2_257 | -0.19 | -0.49 | -0.27 | -0.06 | -0.10 | 0 | 0.22526 | 1.16899 | 2.19951 | 0.01577 | 1 | 0.42814 | 1.3455 | 0.43669 | 0.00199 | 1 | 0.39167 | 1.31191 | 0.66906 | 0.00114 | 0 | 0 |
| 1396287_at | --- | --- | Any2_257 | 0.09 | 0.17 | 0.56 | 0.66 | 0.41 | 1 | 0.38132 | 1.30254 | 2.85516 | 0.00285 | 1 | 0.48512 | 1.3997 | 0.32766 | 0.00117 | 0 | 0.23724 | 1.17874 | 2.37478 | 0.02062 | 0 | 0 |
| 1396443_at | --- | --- | Any2_257 | -0.05 | -0.05 | -0.34 | -0.32 | -0.31 | 1 | -0.2818 | -1.2157 | 3.40813 | 0.00351 | 1 | -0.2653 | -1.2019 | 2.55913 | 0.00757 | 0 | -0.2578 | -1.1957 | 4.104 | 0.00264 | 0 | 0 |
| 1396545_at | Crem | cAMP responsive element modulator | Any2_257 | 0.04 | -0.42 | -0.09 | 0.03 | -0.15 | 1 | 0.33668 | 1.26285 | 2.513 | 0.00646 | 1 | 0.45442 | 1.37023 | 0.595 | 0.00371 | 1 | 0.2734 | 1.20865 | 2.36397 | 0.02204 | 0 | 0 |
| 1396789_x_at | LOC1003655… | rCG41957-like | Any2_257 | -0.08 | 0.02 | -0.09 | -0.32 | -0.28 | 0 | -0.1014 | -1.0728 | 1.62598 | 0.15005 | 1 | -0.332 | -1.2588 | 2.57473 | 0.00753 | 1 | -0.2988 | -1.2301 | 2.98225 | 0.01745 | 0 | 0 |
| 1396921_at | --- | --- | Any2_257 | -0.23 | -0.35 | 0.07 | 0.47 | -0.02 | 1 | 0.41457 | 1.3329 | 2.77727 | 0.00295 | 1 | 0.81324 | 1.75716 | 0.01026 | 1.3E-05 | 1 | 0.32795 | 1.25523 | 1.89583 | 0.00628 | 0 | 0 |
| 1396988_at | --- | --- | Any2_257 | 0.02 | -0.01 | -0.07 | -0.38 | -0.28 | 0 | -0.0688 | -1.0489 | 1.44468 | 0.18907 | 1 | -0.3693 | -1.2917 | 2.726 | 0.00701 | 1 | -0.2741 | -1.2092 | 2.22786 | 0.03553 | 0 | 0 |
| 1397009_at | --- | --- | Any2_257 | -0.01 | 0.19 | -0.04 | -0.21 | -0.24 | 0 | -0.2293 | -1.1723 | 3.06642 | 0.02534 | 1 | -0.4001 | -1.3196 | 2.79338 | 0.00638 | 1 | -0.4332 | -1.3502 | 4.70889 | 0.00136 | 0 | 0 |
| 1397109_at | --- | --- | Any2_257 | -0.16 | 0.07 | -0.29 | -0.27 | -0.31 | 1 | -0.3619 | -1.2851 | 3.46484 | 0.00345 | 1 | -0.3382 | -1.2642 | 2.02403 | 0.01471 | 1 | -0.3781 | -1.2996 | 4.09545 | 0.0029 | 0 | 0 |
| 1397226_at | --- | --- | Any2_257 | 0.04 | -0.33 | -0.45 | -0.06 | 0.00 | 0 | -0.1218 | -1.0881 | 1.61101 | 0.15199 | 1 | 0.26453 | 1.20125 | 1.0878 | 0.0192 | 1 | 0.32537 | 1.25298 | 1.64663 | 0.00439 | 0 | 0 |
| 1397288_at | Nudt21 | nudix (nucleoside diphosphate linked moiety X)-typ | Any2_257 | -0.01 | -0.40 | -0.07 | 0.04 | -0.09 | 1 | 0.32826 | 1.2555 | 2.40038 | 0.01003 | 1 | 0.4381 | 1.35482 | 0.59908 | 0.00376 | 1 | 0.31689 | 1.24564 | 2.17079 | 0.0097 | 0 | 0 |
| 1397372_at | LOC10091052 | MOB kinase activator 1A-like /// similar to Mob4B p | Any2_257 | 0.05 | -0.24 | -0.18 | 0.06 | -0.32 | 0 | 0.05971 | 1.04226 | 1.82691 | 0.07032 | 1 | 0.29849 | 1.22986 | 1.13199 | 0.02118 | 0 | -0.076 | -1.0541 | 1.23734 | 0.22492 | 1 | 0 |
| 1397481_at | --- | --- | Any2_257 | -0.11 | 0.03 | 0.35 | 0.33 | 0.17 | 1 | 0.3126 | 1.24194 | 2.62353 | 0.00143 | 1 | 0.292 | 1.22434 | 0.92173 | 0.00957 | 0 | 0.14101 | 1.10267 | 2.31173 | 0.01762 | 0 | 0 |
| 1397607_at | Crebrf | CREB3 regulatory factor | Any2_257 | -0.32 | -0.49 | -0.29 | -0.15 | -0.40 | 0 | 0.19841 | 1.14744 | 2.30158 | 0.01029 | 1 | 0.33293 | 1.25957 | 0.71716 | 0.0056 | 0 | 0.08952 | 1.06401 | 1.44344 | 0.16835 | 1 | 0 |
| 1397766_at | --- | --- | Any2_257 | 0.01 | -0.16 | 0.10 | 0.63 | 0.38 | 0 | 0.26163 | 1.19883 | 1.93091 | 0.05669 | 1 | 0.78802 | 1.7267 | 0.00588 | 6.4E-06 | 1 | 0.53777 | 1.45173 | 0.25275 | 0.00033 | 0 | 0 |
| 1397919_at | Ccdc37 | coiled-coil domain containing 37 | Any2_257 | 0.01 | 0.03 | 0.06 | -0.33 | -0.30 | 0 | 0.0266 | 1.01861 | 1.17269 | 0.29922 | 1 | -0.3614 | -1.2847 | 3.01111 | 0.00523 | 1 | -0.3335 | -1.26 | 3.56371 | 0.00401 | 0 | 0 |
| 1398150_at | --- | --- | Any2_257 | 0.14 | 0.27 | 0.13 | -0.20 | -0.02 | 0 | -0.1355 | -1.0985 | 2.00148 | 0.09641 | 1 | -0.4692 | -1.3843 | 4.61 | 0.00163 | 1 | -0.2873 | -1.2204 | 3.02751 | 0.01684 | 0 | 0 |
| 1398246_s_at | Fcgr2a /// LO | Fc fragment of IgG, low affinity IIa, receptor /// low | Any2_257 | -0.05 | -0.51 | -0.54 | 0.21 | 0.12 | 0 | -0.0248 | -1.0173 | 1.20317 | 0.28003 | 1 | 0.72129 | 1.64865 | 0.01721 | 2.4E-05 | 1 | 0.63588 | 1.55388 | 0.02667 | 2.6E-05 | 0 | 0 |
| 1398258_at | Apod | apolipoprotein D | Any2_257 | 0.02 | 0.19 | -0.14 | -0.21 | -0.06 | 1 | -0.3326 | -1.2593 | 3.4133 | 0.01163 | 1 | -0.404 | -1.3231 | 3.70053 | 0.00226 | 0 | -0.248 | -1.1876 | 2.82892 | 0.0211 | 0 | 0 |
| 1398486_at | LOC683007 | similar to melanoma inhibitory activity 3 | Any2_257 | -0.22 | -0.15 | -0.02 | 0.14 | -0.03 | 0 | 0.12595 | 1.09123 | 2.2596 | 0.0202 | 1 | 0.2899 | 1.22256 | 0.72224 | 0.00569 | 0 | 0.1216 | 1.08794 | 2.35804 | 0.02206 | 1 | 0 |
| 1398587_at | --- | --- | Any2_257 | -0.25 | 0.01 | -0.31 | -0.09 | -0.26 | 1 | -0.3131 | -1.2423 | 3.65333 | 0.00916 | 0 | -0.0965 | -1.0692 | 1.02807 | 0.22588 | 1 | -0.2664 | -1.2028 | 3.0309 | 0.01618 | 0 | 0 |
| 1398589_at | LOC100910… | paired immunoglobulin-like type 2 receptor alpha-li | Any2_257 | 0.19 | -0.01 | -0.06 | 0.35 | 0.31 | 0 | -0.0541 | -1.0382 | 1.62168 | 0.15049 | 1 | 0.35539 | 1.27933 | 0.81676 | 0.00754 | 1 | 0.31851 | 1.24704 | 2.15237 | 0.0108 | 0 | 0 |
| 1398593_at | LOC298139 | similar to RIKEN cDNA 2310003M01 | Any2_257 | -0.05 | 0.09 | 0.03 | -0.25 | -0.18 | 0 | -0.0607 | -1.043 | 1.62582 | 0.15009 | 1 | -0.3435 | -1.2688 | 2.16574 | 0.01226 | 1 | -0.2705 | -1.2062 | 2.41109 | 0.03062 | 0 | 0 |
| 1398664_at | Gramd3 | GRAM domain containing 3 | Any2_257 | -0.12 | -0.96 | -0.83 | -0.45 | -0.60 | 0 | 0.13209 | 1.09588 | 2.54819 | 0.0059 | 1 | 0.51008 | 1.42413 | 0.08725 | 0.00019 | 1 | 0.36402 | 1.28701 | 0.25211 | 0.00031 | 0 | 0 |
| 1398669_at | LOC690734 | hypothetical protein LOC690734 | Any2_257 | -0.11 | -0.17 | 0.10 | 0.12 | -0.20 | 1 | 0.26613 | 1.20258 | 2.25452 | 0.01434 | 1 | 0.28589 | 1.21909 | 1.04816 | 0.01668 | 0 | -0.0262 | -1.0183 | 1.03569 | 0.37822 | 0 | 0 |
| 1398703_at | --- | --- | Any2_257 | -0.05 | -0.01 | 0.34 | 0.33 | 0.10 | 1 | 0.35593 | 1.27981 | 2.35937 | 0.00956 | 1 | 0.34731 | 1.27219 | 1.08015 | 0.01889 | 0 | 0.1163 | 1.08395 | 1.25018 | 0.24606 | 0 | 0 |
| 1367512_at | Chmp5 | charged multivesicular body protein 5 | uniqAntib_1098 | -0.07 | -0.31 | -0.34 | -0.04 | -0.32 | 0 | -0.0281 | -1.0197 | 1.08191 | 0.35151 | 1 | 0.27071 | 1.2064 | 1.25585 | 0.03695 | 0 | -0.0107 | -1.0075 | 0.98421 | 0.45807 | 0 | 0 |
| 1367571_a_at | Igf2 | insulin-like growth factor 2 | uniqAntib_1098 | -0.02 | 0.00 | -0.06 | -0.32 | 0.01 | 0 | -0.0629 | -1.0445 | 1.01133 | 0.41369 | 1 | -0.3194 | -1.2478 | 1.63572 | 0.03145 | 0 | 0.0057 | 1.00396 | 0.9164 | 0.56527 | 0 | 0 |
| 1367661_at | S100a6 | S100 calcium binding protein A6 | uniqAntib_1098 | 0.00 | -0.30 | -0.22 | 0.28 | -0.13 | 0 | 0.08302 | 1.05923 | 1.26989 | 0.24468 | 1 | 0.58488 | 1.49992 | 0.07735 | 0.00017 | 0 | 0.16781 | 1.12335 | 1.71392 | 0.09975 | 0 | 0 |
| 1367671_at | Pcna | proliferating cell nuclear antigen | uniqAntib_1098 | -0.08 | -0.35 | -0.38 | -0.07 | -0.31 | 0 | -0.0394 | -1.0277 | 1.09296 | 0.34456 | 1 | 0.27692 | 1.2116 | 1.26251 | 0.038 | 0 | 0.03887 | 1.02731 | 1.04285 | 0.38188 | 0 | 0 |
| 1367683_at | Kpna2 /// LO | karyopherin alpha 2 /// karyopherin alpha 2-like | uniqAntib_1098 | -0.08 | -0.25 | -0.24 | 0.08 | -0.19 | 0 | 0.01279 | 1.0089 | 1.09102 | 0.35626 | 1 | 0.33495 | 1.26133 | 1.07539 | 0.01874 | 0 | 0.06029 | 1.04268 | 1.11021 | 0.32708 | 0 | 0 |
| 1367684_at | Crybb2 | crystallin, beta B2 | uniqAntib_1098 | -0.02 | 0.02 | -0.13 | -0.32 | -0.06 | 0 | -0.1544 | -1.113 | 2.30774 | 0.06723 | 1 | -0.345 | -1.2701 | 2.10279 | 0.01359 | 0 | -0.086 | -1.0614 | 1.23049 | 0.22881 | 0 | 0 |
| 1367705_at | Glrx | glutaredoxin (thioltransferase) | uniqAntib_1098 | -0.07 | -0.55 | -0.56 | -0.27 | -0.43 | 0 | -0.0134 | -1.0093 | 0.96598 | 0.48869 | 1 | 0.27766 | 1.21223 | 1.2765 | 0.04109 | 0 | 0.12028 | 1.08694 | 1.2832 | 0.23074 | 0 | 0 |
| 1367728_at | Tsn | translin | uniqAntib_1098 | -0.13 | -0.12 | 0.03 | 0.23 | 0.01 | 0 | 0.15577 | 1.11402 | 2.00036 | 0.0429 | 1 | 0.34794 | 1.27274 | 0.78886 | 0.0071 | 0 | 0.12896 | 1.0935 | 1.78116 | 0.09038 | 0 | 0 |
| 1367729_at | Oat | ornithine aminotransferase | uniqAntib_1098 | -0.04 | -0.34 | -0.40 | 0.04 | -0.19 | 0 | -0.0571 | -1.0404 | 0.99974 | 0.42511 | 1 | 0.37643 | 1.29812 | 0.9413 | 0.01075 | 0 | 0.15591 | 1.11413 | 1.46171 | 0.16258 | 0 | 0 |
| 1367754_s_at | Asl | argininosuccinate lyase | uniqAntib_1098 | -0.04 | -0.35 | -0.36 | 0.12 | -0.21 | 0 | -0.014 | -1.0098 | 1.35258 | 0.21129 | 1 | 0.4631 | 1.3785 | 0.33777 | 0.00122 | 0 | 0.14013 | 1.102 | 1.71278 | 0.102 | 0 | 0 |
| 1367758_at | Afp | alpha-fetoprotein | uniqAntib_1098 | 0.11 | 0.03 | 0.00 | -0.24 | -0.05 | 0 | -0.0346 | -1.0243 | 1.22442 | 0.26816 | 1 | -0.2709 | -1.2065 | 1.5716 | 0.03406 | 0 | -0.0819 | -1.0584 | 1.46317 | 0.14044 | 0 | 0 |
| 1367759_at | H1f0 | H1 histone family, member 0 | uniqAntib_1098 | 0.15 | 0.19 | 0.09 | -0.14 | 0.00 | 0 | -0.0983 | -1.0705 | 1.12321 | 0.32495 | 1 | -0.3326 | -1.2593 | 1.7531 | 0.02475 | 0 | -0.1917 | -1.1421 | 1.83231 | 0.07459 | 0 | 0 |
| 1367787_at | Ica1 | islet cell autoantigen 1 | uniqAntib_1098 | 0.03 | -0.02 | -0.15 | -0.38 | -0.06 | 0 | -0.1287 | -1.0933 | 1.49307 | 0.17706 | 1 | -0.3606 | -1.284 | 3.79955 | 0.00269 | 0 | -0.0431 | -1.0303 | 1.031 | 0.38437 | 0 | 0 |
| 1367808_at | Timm8b | translocase of inner mitochondrial membrane 8 hor | uniqAntib_1098 | -0.06 | -0.43 | -0.37 | -0.16 | -0.28 | 0 | 0.05571 | 1.03937 | 1.49103 | 0.16018 | 1 | 0.2684 | 1.20447 | 1.2231 | 0.03201 | 0 | 0.1428 | 1.10405 | 1.52405 | 0.1457 | 0 | 0 |
| 1367835_at | LOC10091128 | proSAAS-like /// proprotein convertase subtilisin/ke | uniqAntib_1098 | -0.06 | 0.05 | -0.06 | -0.22 | 0.07 | 0 | -0.1075 | -1.0774 | 1.86641 | 0.11631 | 1 | -0.269 | -1.205 | 1.56326 | 0.03549 | 0 | 0.02125 | 1.01484 | 1.05889 | 0.37168 | 0 | 0 |
| 1367846_at | S100a4 | S100 calcium-binding protein A4 | uniqAntib_1098 | -0.06 | -0.96 | -0.92 | -0.23 | -0.72 | 0 | 0.04634 | 1.03264 | 1.95 | 0.04966 | 1 | 0.72876 | 1.65721 | 0.00743 | 8.4E-06 | 0 | 0.24198 | 1.18261 | 2.0504 | 0.00818 | 0 | 0 |
| 1367904_at | Resp18 | regulated endocrine-specific protein 18 | uniqAntib_1098 | -0.09 | 0.11 | 0.13 | -0.18 | 0.05 | 0 | 0.02771 | 1.0194 | 1.01482 | 0.42967 | 1 | -0.2908 | -1.2233 | 1.51734 | 0.03962 | 0 | -0.0584 | -1.0413 | 1.02332 | 0.39259 | 0 | 0 |
| 1367908_at | Gcsh | glycine cleavage system protein H (aminomethyl ca | uniqAntib_1098 | 0.06 | -0.32 | -0.20 | 0.04 | -0.27 | 0 | 0.12144 | 1.08782 | 1.78724 | 0.07465 | 1 | 0.36428 | 1.28723 | 0.96003 | 0.01161 | 0 | 0.04975 | 1.03509 | 1.08858 | 0.34318 | 0 | 0 |
| 1367913_at | Cygb | cytoglobin | uniqAntib_1098 | -0.03 | -0.06 | -0.07 | -0.33 | 0.04 | 0 | -0.0123 | -1.0085 | 0.94041 | 0.48779 | 1 | -0.2791 | -1.2135 | 1.7968 | 0.02328 | 0 | 0.09536 | 1.06833 | 1.38156 | 0.18943 | 0 | 0 |
| 1367929_at | Cd59 | CD59 molecule, complement regulatory protein | uniqAntib_1098 | 0.03 | -0.30 | -0.16 | 0.08 | -0.21 | 0 | 0.14354 | 1.10461 | 1.78721 | 0.07477 | 1 | 0.38105 | 1.30229 | 0.90067 | 0.00906 | 0 | 0.09392 | 1.06726 | 1.45016 | 0.16638 | 0 | 0 |
| 1367949_at | Penk | proenkephalin | uniqAntib_1098 | 0.00 | 0.15 | 0.01 | -0.19 | 0.07 | 0 | -0.137 | -1.0996 | 1.60411 | 0.15392 | 1 | -0.3409 | -1.2665 | 1.91688 | 0.01874 | 0 | -0.0838 | -1.0598 | 1.04521 | 0.36853 | 0 | 0 |
| 1367986_at | Ptgfrn | prostaglandin F2 receptor inhibitor | uniqAntib_1098 | 0.41 | 0.71 | 0.68 | 0.98 | 0.81 | 0 | -0.0292 | -1.0204 | 2.97154 | 0.02981 | 1 | 0.26912 | 1.20507 | 0.56047 | 0.00306 | 0 | 0.09539 | 1.06835 | 2.2296 | 0.01247 | 0 | 0 |
| 1367988_at | Cyp2c23 | cytochrome P450, family 2, subfamily c, polypeptide | uniqAntib_1098 | -0.07 | 0.00 | -0.02 | -0.27 | -0.20 | 0 | -0.0204 | -1.0142 | 1.0428 | 0.38478 | 1 | -0.2726 | -1.2079 | 1.52511 | 0.03909 | 0 | -0.2016 | -1.15 | 1.70795 | 0.09117 | 0 | 0 |
| 1367989_at | Slc2a4 | solute carrier family 2 (facilitated glucose transporte | uniqAntib_1098 | -0.12 | -0.70 | -0.94 | -0.98 | -0.69 | 0 | -0.2359 | -1.1777 | 2.94682 | 0.00208 | 1 | -0.276 | -1.2108 | 3.647 | 0.00117 | 0 | 0.00766 | 1.00533 | 2.05052 | 0.04721 | 0 | 0 |
| 1368024_at | Qsox1 | quiescin Q6 sulfhydryl oxidase 1 | uniqAntib_1098 | 0.00 | 0.03 | -0.06 | -0.30 | -0.06 | 0 | -0.0837 | -1.0597 | 1.2091 | 0.27767 | 1 | -0.324 | -1.2518 | 1.9257 | 0.01845 | 0 | -0.0892 | -1.0638 | 1.24961 | 0.2208 | 0 | 0 |
| 1368046_at | Slc31a1 | solute carrier family 31 (copper transporters), mem | uniqAntib_1098 | -0.03 | -0.01 | -0.05 | 0.29 | 0.10 | 0 | -0.0432 | -1.0304 | 1.15197 | 0.30715 | 1 | 0.30044 | 1.23152 | 1.20545 | 0.02895 | 0 | 0.10871 | 1.07827 | 1.29013 | 0.22717 | 0 | 0 |

| Affymetrix Probeset ID | Gene Symbol | Gene Title | Figure 6 B Venn n =2021 | Mean BN log2 intensity autologous n=8 | Mean log2 intensity day 30 DR +/+ ND n=4 | Mean log2 intensity day 30 DR/+ HCD n=4 | Mean log2 intensity day 30 B/S n=4 | Mean log2 intensity day 30 F+/+ ND n=4 | DR+/+ HCD vs DR+/+ ND n=380 1=yes 0=no | Log2 ratio DR+/+ HCD vs DR+/+ ND | Fold-Change DR+/+ HCD vs DR+/+ ND | RP-FDR 4DR30 HCD vs 4DR30 Reg | p value DR+/+ HCD vs DR+/+ ND | DR+/+ B/S vs DR+/+ ND n=1,330 1=yes 0=no | Log2 ratio DR+/+ B/S vs DR+/+ ND | Fold-Change DR+/+ B/S vs DR+/+ ND | RP-FDR 4DR30 Antib vs 4DR30 Reg | p value DR+/+ B/S vs DR+/+ ND | DR+/+ ND vs F+/+ ND n=398 1=yes 0=no | Log2 ratio DR+/+ ND vs F+/+ ND | Fold-Change DR+/+ ND vs F+/+ ND | RP-FDR 4RT1uu 30Reg vs 4DR30 Reg | p value DR+/+ ND vs F+/+ ND | DR+/+ Longitudinal n=228 1=yes 0=no | Figure 6F |
|---|---|---|---|---|---|---|---|---|---|---|---|---|---|---|---|---|---|---|---|---|---|---|---|---|---|
| 1368052_at | Tspan8 | tetraspanin 8 | uniqAntib_1098 | -0.12 | -0.66 | -0.70 | -0.21 | -0.58 | 0 | -0.0418 | -1.0294 | 1.19551 | 0.28443 | 1 | 0.45298 | 1.36886 | 0.44622 | 0.00205 | 0 | 0.079 | 1.05629 | 1.58612 | 0.13 | 0 | 0 |
| 1368055_a_at | Lmna | lamin A/C | uniqAntib_1098 | 0.07 | -0.06 | 0.09 | 0.24 | 0.05 | 0 | 0.14545 | 1.10608 | 1.85298 | 0.06685 | 1 | 0.30529 | 1.23567 | 1.13786 | 0.02173 | 0 | 0.11264 | 1.0812 | 1.99264 | 0.06382 | 0 | 0 |
| 1368080_at | LOC683573 /// | similar to response gene to complement 32 /// regu | uniqAntib_1098 | -0.10 | -0.11 | -0.11 | 0.22 | -0.10 | 0 | -0.0035 | -1.0024 | 1.03104 | 0.4166 | 1 | 0.32854 | 1.25574 | 1.17156 | 0.02516 | 0 | 0.01222 | 1.0085 | 0.89811 | 0.62575 | 0 | 0 |
| 1368144_at | Rgs2 | regulator of G-protein signaling 2 | uniqAntib_1098 | -0.06 | -0.51 | -0.58 | -0.20 | -0.39 | 0 | -0.0727 | -1.0517 | 1.27925 | 0.24611 | 1 | 0.30236 | 1.23316 | 1.18682 | 0.02458 | 0 | 0.11827 | 1.08543 | 1.40995 | 0.1813 | 0 | 0 |
| 1368208_at | Nat8b | N-acetyltransferase 8B | uniqAntib_1098 | -0.16 | -0.04 | -0.14 | -0.34 | -0.09 | 0 | -0.1062 | -1.0764 | 1.50959 | 0.17499 | 1 | -0.3051 | -1.2355 | 2.1473 | 0.01305 | 0 | -0.0568 | -1.0402 | 1.21972 | 0.23462 | 0 | 0 |
| 1368209_at | Pdzk1ip1 | PDZK1 interacting protein 1 | uniqAntib_1098 | -0.19 | -0.20 | -0.23 | -0.56 | -0.31 | 0 | -0.0295 | -1.0207 | 1.00632 | 0.41791 | 1 | -0.3581 | -1.2817 | 2.01384 | 0.01483 | 0 | -0.1127 | -1.0813 | 1.20214 | 0.24364 | 0 | 0 |
| 1368222_at | Nr3c1 | nuclear receptor subfamily 3, group C, member 1 | uniqAntib_1098 | 0.10 | -0.15 | 0.05 | 0.15 | -0.07 | 0 | 0.19838 | 1.14741 | 1.69541 | 0.10516 | 1 | 0.29994 | 1.23109 | 1.20569 | 0.02939 | 0 | 0.0798 | 1.05687 | 1.29851 | 0.22322 | 0 | 0 |
| 1368261_at | Nrxn3 | neurexin 3 | uniqAntib_1098 | -0.09 | -0.11 | -0.14 | -0.41 | -0.14 | 0 | -0.0216 | -1.0151 | 1.04524 | 0.38164 | 1 | -0.3009 | -1.2319 | 2.1382 | 0.01334 | 0 | -0.0238 | -1.0166 | 1.01087 | 0.4132 | 0 | 0 |
| 1368345_at | Map6 | microtubule-associated protein 6 | uniqAntib_1098 | 0.02 | 0.19 | -0.03 | -0.13 | 0.04 | 0 | -0.2238 | -1.1678 | 2.56619 | 0.04654 | 1 | -0.3183 | -1.2469 | 1.86798 | 0.01994 | 0 | -0.1501 | -1.1096 | 1.61676 | 0.10278 | 0 | 0 |
| 1368364_at | Smcp | sperm mitochondria-associated cysteine-rich protei | uniqAntib_1098 | 0.12 | 0.19 | -0.01 | -0.09 | 0.02 | 0 | -0.1929 | -1.143 | 2.03114 | 0.08941 | 1 | -0.2788 | -1.2131 | 1.43859 | 0.04788 | 0 | -0.1664 | -1.1222 | 1.40065 | 0.15899 | 0 | 0 |
| 1368430_at | Lgmn | legumain | uniqAntib_1098 | -0.03 | -0.58 | -0.56 | -0.26 | -0.45 | 0 | 0.02507 | 1.01753 | 1.02599 | 0.42087 | 1 | 0.32369 | 1.25153 | 1.12508 | 0.02087 | 0 | 0.13676 | 1.09943 | 1.4135 | 0.17881 | 0 | 0 |
| 1368438_at | Pde10a | phosphodiesterase 10A | uniqAntib_1098 | -0.01 | 0.13 | -0.10 | -0.22 | -0.08 | 0 | -0.2284 | -1.1715 | 2.13559 | 0.07925 | 1 | -0.3475 | -1.2724 | 1.90735 | 0.0192 | 0 | -0.2105 | -1.1571 | 1.70477 | 0.09204 | 0 | 0 |
| 1368448_at | Ltbp2 | latent transforming growth factor beta binding prot | uniqAntib_1098 | -0.02 | -0.02 | -0.07 | -0.32 | -0.16 | 0 | -0.0551 | -1.039 | 1.03175 | 0.39626 | 1 | -0.3004 | -1.2315 | 1.61806 | 0.03184 | 0 | -0.146 | -1.1065 | 1.34024 | 0.17777 | 0 | 0 |
| 1368454_at | Prl7d1 | prolactin family 7, subfamily d, member 1 | uniqAntib_1098 | -0.01 | 0.08 | -0.04 | -0.34 | -0.09 | 0 | -0.1196 | -1.0864 | 1.26684 | 0.25134 | 1 | -0.4223 | -1.3401 | 3.29233 | 0.00455 | 0 | -0.1772 | -1.1307 | 1.6272 | 0.10025 | 0 | 0 |
| 1368466_a_at | Odf2 | outer dense fiber of sperm tails 2 | uniqAntib_1098 | 0.09 | -0.09 | 0.08 | 0.20 | 0.15 | 0 | 0.17496 | 1.12893 | 2.21843 | 0.02047 | 1 | 0.28907 | 1.22185 | 1.03864 | 0.01647 | 0 | 0.24162 | 1.18232 | 2.34237 | 0.01973 | 0 | 0 |
| 1368468_at | Cyp11a1 | cytochrome P450, family 11, subfamily a, polypeptic | uniqAntib_1098 | 0.09 | 0.14 | -0.06 | -0.17 | -0.11 | 0 | -0.1925 | -1.1428 | 2.50305 | 0.05167 | 1 | -0.3103 | -1.24 | 2.06301 | 0.01453 | 0 | -0.249 | -1.1884 | 2.93935 | 0.01456 | 0 | 0 |
| 1368472_at | Celsr3 | cadherin, EGF LAG seven-pass G-type receptor 3 | uniqAntib_1098 | 0.05 | -0.01 | -0.05 | -0.28 | -0.11 | 0 | -0.0484 | -1.0341 | 1.38071 | 0.21066 | 1 | -0.2723 | -1.2078 | 1.56265 | 0.03522 | 0 | -0.1021 | -1.0734 | 1.41916 | 0.15237 | 0 | 0 |
| 1368481_at | Gipr | gastric inhibitory polypeptide receptor | uniqAntib_1098 | 0.13 | 0.17 | 0.04 | -0.12 | 0.16 | 0 | -0.1338 | -1.0972 | 1.5077 | 0.17535 | 1 | -0.2906 | -1.2231 | 1.56607 | 0.03691 | 0 | -0.0054 | -1.0038 | 0.91563 | 0.59553 | 0 | 0 |
| 1368484_at | Abcb9 | ATP-binding cassette, subfamily B (MDR/TAP), mem | uniqAntib_1098 | 0.04 | 0.07 | 0.08 | -0.21 | -0.17 | 0 | 0.00861 | 1.00598 | 1.20396 | 0.27971 | 1 | -0.2798 | -1.214 | 1.82608 | 0.02126 | 0 | -0.2387 | -1.1799 | 2.66949 | 0.02515 | 0 | 0 |
| 1368536_at | Enpp2 | ectonucleotide pyrophosphatase/phosphodiesteras | uniqAntib_1098 | -0.05 | -0.26 | -0.16 | 0.03 | -0.12 | 0 | 0.09754 | 1.06995 | 1.9878 | 0.04417 | 1 | 0.2824 | 1.21622 | 1.07486 | 0.0187 | 0 | 0.13977 | 1.10173 | 1.94476 | 0.06916 | 0 | 0 |
| 1368558_s_at | Aif1 | allograft inflammatory factor 1 | uniqAntib_1098 | -0.34 | -0.42 | -0.21 | 0.00 | -0.22 | 0 | 0.21495 | 1.16067 | 2.1579 | 0.02283 | 1 | 0.41849 | 1.33652 | 0.56377 | 0.00303 | 0 | 0.20583 | 1.15335 | 2.36619 | 0.02275 | 0 | 0 |
| 1368594_at | Pla2g2c | phospholipase A2, group IIC | uniqAntib_1098 | -0.09 | 0.03 | 0.03 | -0.35 | -0.21 | 0 | -0.0002 | -1.0001 | 1.1706 | 0.29834 | 1 | -0.38 | -1.3013 | 2.75421 | 0.00673 | 0 | -0.2413 | -1.1821 | 2.36706 | 0.03128 | 0 | 0 |
| 1368600_at | Slc26a1 | solute carrier family 26 (sulfate transporter), memb | uniqAntib_1098 | -0.07 | 0.08 | -0.03 | -0.20 | -0.09 | 0 | -0.1086 | -1.0782 | 1.13431 | 0.31849 | 1 | -0.2752 | -1.2102 | 1.57301 | 0.03399 | 0 | -0.1679 | -1.1234 | 1.44716 | 0.14337 | 0 | 0 |
| 1368691_at | Gria3 | glutamate receptor, ionotropic, AMPA 3 | uniqAntib_1098 | 0.30 | 0.31 | 0.16 | -0.03 | 0.05 | 0 | -0.1456 | -1.1062 | 1.91404 | 0.10795 | 1 | -0.3377 | -1.2638 | 2.28611 | 0.01154 | 0 | -0.2605 | -1.1979 | 2.89995 | 0.02005 | 0 | 0 |
| 1368722_at | Lta | lymphotoxin alpha (TNF superfamily, member 1) | uniqAntib_1098 | 0.07 | -0.35 | -0.32 | -0.08 | -0.35 | 0 | 0.03289 | 1.02306 | 1.25623 | 0.25251 | 1 | 0.27644 | 1.2112 | 1.18447 | 0.02582 | 0 | 0.00399 | 1.00277 | 1.10204 | 0.31457 | 0 | 0 |
| 1368741_at | C9 | complement component 9 | uniqAntib_1098 | -0.04 | -0.04 | -0.10 | -0.36 | -0.08 | 0 | -0.0677 | -1.048 | 1.38856 | 0.2078 | 1 | -0.3213 | -1.2494 | 2.1917 | 0.01283 | 0 | -0.0494 | -1.0348 | 1.22449 | 0.23136 | 0 | 0 |
| 1368762_at | Ubd | ubiquitin D | uniqAntib_1098 | 0.01 | -1.15 | -1.11 | -0.27 | -1.29 | 0 | 0.03901 | 1.02741 | 1.45375 | 0.17357 | 1 | 0.87845 | 1.8384 | 0.002 | 1.6E-06 | 0 | -0.1369 | -1.0995 | 1.87892 | 0.06356 | 0 | 0 |
| 1368870_at | Id2 | inhibitor of DNA binding 2 | uniqAntib_1098 | 0.06 | -0.12 | -0.03 | 0.24 | -0.04 | 0 | 0.09856 | 1.0707 | 1.09098 | 0.35635 | 1 | 0.36844 | 1.29095 | 0.97155 | 0.0129 | 0 | 0.08134 | 1.058 | 1.0416 | 0.38313 | 0 | 0 |
| 1368902_at | Pak3 | p21 protein (Cdc42/Rac)-activated kinase 3 | uniqAntib_1098 | -0.10 | -0.20 | -0.25 | -0.49 | -0.37 | 0 | -0.0427 | -1.0301 | 1.45491 | 0.18718 | 1 | -0.2859 | -1.2192 | 2.58692 | 0.0089 | 0 | -0.1714 | -1.1262 | 2.39474 | 0.03103 | 0 | 0 |
| 1368970_at | Cdh23 | cadherin-related 23 | uniqAntib_1098 | 0.23 | 0.25 | 0.27 | -0.04 | 0.17 | 0 | 0.02265 | 1.01582 | 1.07345 | 0.37327 | 1 | -0.2944 | -1.2264 | 1.70337 | 0.02711 | 0 | -0.0814 | -1.0581 | 1.20566 | 0.24168 | 0 | 0 |
| 1369029_at | Plscr1 | phospholipid scramblase 1 | uniqAntib_1098 | 0.10 | -0.11 | 0.00 | 0.28 | -0.15 | 0 | 0.11691 | 1.08441 | 1.35138 | 0.21201 | 1 | 0.38986 | 1.31027 | 0.71473 | 0.00549 | 0 | -0.0364 | -1.0255 | 1.0371 | 0.37537 | 0 | 0 |
| 1369048_at | Gabrd | gamma-aminobutyric acid (GABA) A receptor, delta | uniqAntib_1098 | 0.07 | 0.10 | -0.01 | -0.22 | 0.07 | 0 | -0.1104 | -1.0796 | 1.24508 | 0.25991 | 1 | -0.3166 | -1.2454 | 1.70708 | 0.02673 | 0 | -0.0333 | -1.0233 | 0.95318 | 0.50783 | 0 | 0 |
| 1369067_at | Nr4a3 | nuclear receptor subfamily 4, group A, member 3 | uniqAntib_1098 | 0.11 | -0.01 | -0.02 | 0.41 | 0.02 | 0 | -0.0081 | -1.0057 | 1.09599 | 0.34329 | 1 | 0.41619 | 1.3344 | 0.68991 | 0.00484 | 0 | 0.02706 | 1.01893 | 1.43201 | 0.17392 | 0 | 0 |
| 1369068_at | Cul5 | cullin 5 | uniqAntib_1098 | -0.02 | -0.05 | -0.04 | 0.32 | -0.08 | 0 | 0.00868 | 1.00604 | 1.42258 | 0.18188 | 1 | 0.36101 | 1.28433 | 1.04543 | 0.01677 | 0 | -0.0373 | -1.0262 | 0.94878 | 0.51407 | 0 | 0 |
| 1369076_at | Tmem33 | transmembrane protein 33 | uniqAntib_1098 | -0.22 | -0.33 | -0.37 | -0.06 | -0.39 | 0 | -0.0346 | -1.0243 | 1.17094 | 0.29824 | 1 | 0.27148 | 1.20704 | 1.28212 | 0.04795 | 0 | -0.0581 | -1.0411 | 1.18901 | 0.25417 | 0 | 0 |
| 1369168_a_at | Clock | clock circadian regulator | uniqAntib_1098 | 0.09 | 0.14 | 0.12 | -0.19 | 0.21 | 0 | -0.0108 | -1.0075 | 1.14831 | 0.31419 | 1 | -0.3296 | -1.2567 | 2.58202 | 0.00946 | 0 | 0.07879 | 1.05614 | 1.28332 | 0.22989 | 0 | 0 |
| 1369171_at | Mst1 | Macrophage stimulating 1 (hepatocyte growth factc | uniqAntib_1098 | -0.12 | 0.08 | -0.05 | -0.24 | -0.09 | 0 | -0.1246 | -1.0902 | 1.94728 | 0.10313 | 1 | -0.3209 | -1.2491 | 1.79056 | 0.02349 | 0 | -0.1668 | -1.1226 | 2.02305 | 0.05035 | 0 | 0 |
| 1369218_at | Met | met proto-oncogene | uniqAntib_1098 | -0.04 | 0.07 | 0.08 | -0.20 | -0.06 | 0 | 0.00827 | 1.00575 | 0.99065 | 0.45664 | 1 | -0.2781 | -1.2126 | 1.50915 | 0.04154 | 0 | -0.1368 | -1.0995 | 1.33402 | 0.17995 | 0 | 0 |
| 1369237_at | Slc6a7 | solute carrier family 6 (neurotransmitter transporte | uniqAntib_1098 | 0.12 | 0.17 | 0.19 | -0.24 | 0.08 | 0 | 0.01815 | 1.01266 | 0.97957 | 0.44129 | 1 | -0.4112 | -1.3298 | 3.61345 | 0.00337 | 0 | -0.0934 | -1.0669 | 1.15625 | 0.27353 | 0 | 0 |
| 1369248_a_at | Xiap | X-linked inhibitor of apoptosis | uniqAntib_1098 | -0.20 | -0.13 | -0.01 | 0.27 | -0.21 | 0 | 0.1169 | 1.0844 | 2.26403 | 0.01733 | 1 | 0.39138 | 1.31164 | 0.37413 | 0.00146 | 0 | -0.089 | -1.0636 | 2.4399 | 0.03013 | 0 | 0 |
| 1369291_at | Agtr1a | angiotensin II receptor, type 1a | uniqAntib_1098 | -0.10 | 0.02 | -0.16 | -0.31 | -0.17 | 0 | -0.1788 | -1.1319 | 1.65469 | 0.14472 | 1 | -0.335 | -1.2614 | 1.80219 | 0.02144 | 0 | -0.1918 | -1.1422 | 1.52251 | 0.11828 | 0 | 0 |
| 1369328_at | Acacb | acetyl-CoA carboxylase beta | uniqAntib_1098 | 0.02 | -0.10 | -0.24 | -0.37 | -0.14 | 0 | -0.1387 | -1.1009 | 1.60268 | 0.1545 | 1 | -0.277 | -1.2117 | 1.84601 | 0.02036 | 0 | -0.0433 | -1.0305 | 1.15925 | 0.27193 | 0 | 0 |
| 1369331_a_at | Unc13b | unc-13 homolog B (C. elegans) | uniqAntib_1098 | 0.07 | -0.02 | -0.07 | -0.33 | 0.03 | 0 | -0.0431 | -1.0304 | 1.02748 | 0.39951 | 1 | -0.3088 | -1.2387 | 1.69604 | 0.02716 | 0 | 0.05312 | 1.03751 | 0.9769 | 0.45702 | 0 | 0 |
| 1369344_at | Wdr7 | WD repeat domain 7 | uniqAntib_1098 | 0.13 | 0.22 | 0.08 | -0.23 | 0.00 | 0 | -0.1394 | -1.1015 | 1.86637 | 0.11667 | 1 | -0.4453 | -1.3616 | 3.816 | 0.00184 | 0 | -0.2174 | -1.1626 | 1.87802 | 0.06661 | 0 | 0 |
| 1369348_at | Trpm8 | transient receptor potential cation channel, subfam | uniqAntib_1098 | -0.06 | 0.01 | -0.08 | -0.27 | -0.17 | 0 | -0.0939 | -1.0672 | 1.66711 | 0.14249 | 1 | -0.2877 | -1.2207 | 2.25926 | 0.01184 | 0 | -0.1804 | -1.1332 | 2.34781 | 0.0342 | 0 | 0 |
| 1369416_at | Hcn3 | hyperpolarization-activated cyclic nucleotide-gated | uniqAntib_1098 | 0.02 | 0.00 | 0.03 | -0.32 | -0.20 | 0 | 0.03352 | 1.02351 | 0.92855 | 0.54362 | 1 | -0.3159 | -1.2448 | 1.74679 | 0.02589 | 0 | -0.1956 | -1.1452 | 1.63313 | 0.09925 | 0 | 0 |
| 1369437_at | Slco4a1 | solute carrier organic anion transporter family, men | uniqAntib_1098 | 0.03 | 0.07 | 0.04 | -0.20 | 0.04 | 0 | -0.0276 | -1.0193 | 1.6402 | 0.14794 | 1 | -0.2652 | -1.2018 | 1.48285 | 0.04329 | 0 | -0.0289 | -1.0202 | 1.09126 | 0.32293 | 0 | 0 |
| 1369462_at | Gad2 | glutamate decarboxylase 2 | uniqAntib_1098 | 0.03 | 0.02 | 0.16 | -0.29 | -0.07 | 0 | 0.13881 | 1.101 | 1.67727 | 0.11261 | 1 | -0.3064 | -1.2367 | 2.46398 | 0.00975 | 0 | -0.093 | -1.0666 | 1.48583 | 0.13191 | 0 | 0 |
| 1369537_at | Mchr1 | melanin-concentrating hormone receptor 1 | uniqAntib_1098 | 0.12 | 0.04 | -0.07 | -0.25 | -0.10 | 0 | -0.1081 | -1.0778 | 1.41842 | 0.19503 | 1 | -0.2869 | -1.22 | 1.47133 | 0.04419 | 0 | -0.1435 | -1.1046 | 1.42457 | 0.14975 | 0 | 0 |
| 1369546_at | Bbox1 | butyrobetaine (gamma), 2-oxoglutarate dioxygenas | uniqAntib_1098 | 0.02 | 0.09 | 0.03 | -0.20 | 0.11 | 0 | -0.0615 | -1.0435 | 1.14435 | 0.31163 | 1 | -0.2856 | -1.2189 | 1.5851 | 0.03389 | 0 | 0.02716 | 1.019 | 1.03485 | 0.38817 | 0 | 0 |
| 1369581_at | Pemt | phosphatidylethanolamine N-methyltransferase | uniqAntib_1098 | 0.02 | 0.03 | -0.04 | -0.31 | -0.04 | 0 | -0.0717 | -1.0509 | 1.12791 | 0.32337 | 1 | -0.3408 | -1.2664 | 2.04736 | 0.01672 | 0 | -0.0737 | -1.0524 | 1.24094 | 0.22354 | 0 | 0 |
| 1369610_at | Lin7c | lin-7 homolog C (C. elegans) | uniqAntib_1098 | -0.12 | -0.33 | -0.31 | -0.04 | -0.34 | 0 | 0.01717 | 1.01197 | 1.04789 | 0.39872 | 1 | 0.29019 | 1.2228 | 1.22488 | 0.03238 | 0 | -0.0135 | -1.0094 | 0.9844 | 0.4579 | 0 | 0 |
| 1369622_at | Prok2 | prokineticin 2 | uniqAntib_1098 | -0.18 | 0.00 | -0.15 | -0.32 | -0.22 | 0 | -0.1452 | -1.1059 | 2.50209 | 0.05149 | 1 | -0.3139 | -1.2431 | 1.69222 | 0.02764 | 0 | -0.2118 | -1.1581 | 1.91443 | 0.06033 | 0 | 0 |
| 1369702_at | Ensa | endosulfine alpha | uniqAntib_1098 | 0.02 | -0.07 | 0.08 | 0.29 | 0.01 | 0 | 0.15539 | 1.11372 | 1.52726 | 0.14969 | 1 | 0.36742 | 1.29005 | 0.97186 | 0.01294 | 0 | 0.08538 | 1.06097 | 1.11689 | 0.32118 | 0 | 0 |
| 1369712_at | Stk3 | serine/threonine kinase 3 | uniqAntib_1098 | 0.07 | 0.03 | -0.06 | 0.30 | 0.15 | 0 | -0.0947 | -1.0679 | 1.40286 | 0.20205 | 1 | 0.27094 | 1.20659 | 1.27844 | 0.04168 | 0 | 0.12199 | 1.08823 | 1.71209 | 0.10069 | 0 | 0 |
| 1369737_at | Crem | cAMP responsive element modulator | uniqAntib_1098 | -0.13 | -1.06 | -1.15 | -0.63 | -0.83 | 0 | -0.0847 | -1.0605 | 1.31371 | 0.23394 | 1 | 0.43112 | 1.34828 | 0.59337 | 0.00363 | 0 | 0.22651 | 1.17 | 2.22454 | 0.04549 | 0 | 0 |
| 1369745_at | Grin2b | glutamate receptor, ionotropic, N-methyl D-asparta | uniqAntib_1098 | 0.10 | 0.06 | 0.03 | -0.23 | -0.15 | 0 | -0.0333 | -1.0234 | 1.05366 | 0.37235 | 1 | -0.2879 | -1.2209 | 1.57334 | 0.0342 | 0 | -0.2099 | -1.1566 | 1.98098 | 0.05223 | 0 | 0 |
| 1369788_s_at | Jun | jun proto-oncogene | uniqAntib_1098 | 0.12 | 0.40 | 0.34 | 0.72 | 0.29 | 0 | -0.0524 | -1.037 | 1.11165 | 0.33129 | 1 | 0.32378 | 1.2516 | 1.13874 | 0.02157 | 0 | -0.1044 | -1.075 | 1.2056 | 0.2421 | 0 | 0 |
| 1369816_at | Rab3a | RAB3A, member RAS oncogene family | uniqAntib_1098 | 0.17 | 0.18 | 0.15 | -0.11 | 0.03 | 0 | -0.0292 | -1.0204 | 1.18555 | 0.28957 | 1 | -0.2931 | -1.2253 | 1.56251 | 0.03703 | 0 | -0.1532 | -1.112 | 1.61072 | 0.10348 | 0 | 0 |
| 1369823_at | Adam7 | ADAM metallopeptidase domain 7 | uniqAntib_1098 | -0.05 | -0.04 | -0.16 | -0.31 | -0.14 | 0 | -0.1198 | -1.0866 | 1.26499 | 0.25199 | 1 | -0.2742 | -1.2093 | 1.65701 | 0.02941 | 0 | -0.1037 | -1.0745 | 1.65687 | 0.09707 | 0 | 0 |
| 1369828_at | Csf2rb | colony stimulating factor 2 receptor, beta, low-affin | uniqAntib_1098 | 0.22 | 0.18 | 0.11 | -0.09 | 0.11 | 0 | -0.0656 | -1.0466 | 1.1932 | 0.28542 | 1 | -0.2676 | -1.2038 | 1.50591 | 0.04193 | 0 | -0.0647 | -1.0459 | 1.22469 | 0.23104 | 0 | 0 |
| 1369860_a_at | Htr2c | 5-hydroxytryptamine (serotonin) receptor 2C, G pro | uniqAntib_1098 | 0.02 | 0.02 | -0.01 | -0.25 | 0.00 | 0 | -0.0377 | -1.0265 | 1.18781 | 0.28795 | 1 | -0.2713 | -1.2069 | 1.85027 | 0.02005 | 0 | -0.0239 | -1.0167 | 1.04713 | 0.36772 | 0 | 0 |
| 1369886_a_at | Cabp1 | calcium binding protein 1 | uniqAntib_1098 | 0.10 | 0.15 | -0.08 | -0.14 | 0.15 | 0 | -0.2257 | -1.1693 | 2.56192 | 0.04712 | 1 | -0.2892 | -1.222 | 1.73599 | 0.02478 | 0 | 0.00619 | 1.0043 | 1.03432 | 0.3887 | 0 | 0 |
| 1369907_at | Alpi | alkaline phosphatase, intestinal | uniqAntib_1098 | 0.04 | 0.08 | -0.03 | -0.23 | -0.06 | 0 | -0.1132 | -1.0816 | 1.34503 | 0.22252 | 1 | -0.3055 | -1.2359 | 1.5728 | 0.03464 | 0 | -0.139 | -1.1012 | 1.22418 | 0.2317 | 0 | 0 |
| 1369940_at | Taldo1 | transaldolase 1 | uniqAntib_1098 | -0.03 | -0.33 | -0.29 | -0.02 | -0.19 | 0 | 0.03425 | 1.02402 | 1.04971 | 0.39516 | 1 | 0.30823 | 1.23819 | 1.20476 | 0.02716 | 0 | 0.13527 | 1.0983 | 1.40809 | 0.18197 | 0 | 0 |
| 1370007_at | Pdia4 | protein disulfide isomerase family A, member 4 | uniqAntib_1098 | -0.04 | -0.27 | -0.36 | 0.09 | -0.18 | 0 | -0.0816 | -1.0582 | 1.16118 | 0.30292 | 1 | 0.36269 | 1.28582 | 0.94322 | 0.01074 | 0 | 0.09809 | 1.07036 | 1.20108 | 0.27023 | 0 | 0 |
| 1370009_at | Apoc3 | apolipoprotein C-III | uniqAntib_1098 | 0.17 | 0.24 | 0.07 | -0.08 | 0.01 | 0 | -0.1734 | -1.1277 | 2.27005 | 0.06891 | 1 | -0.319 | -1.2475 | 1.65247 | 0.02928 | 0 | -0.2318 | -1.1743 | 1.93031 | 0.05959 | 0 | 0 |
| 1370010_at | Lamp2 | lysosomal-associated membrane protein 2 | uniqAntib_1098 | -0.11 | -0.48 | -0.43 | -0.18 | -0.35 | 0 | 0.04846 | 1.03416 | 1.35688 | 0.20943 | 1 | 0.29898 | 1.23028 | 1.17747 | 0.02559 | 0 | 0.12921 | 1.09369 | 1.46682 | 0.16079 | 0 | 0 |
| 1370024_at | Fabp7 | fatty acid binding protein 7, brain | uniqAntib_1098 | 0.02 | -0.03 | -0.13 | -0.33 | -0.08 | 0 | -0.0973 | -1.0697 | 1.41233 | 0.19914 | 1 | -0.2991 | -1.2304 | 1.93522 | 0.01811 | 0 | -0.0524 | -1.037 | 1.20399 | 0.24255 | 0 | 0 |
| 1370027_a_at | A1i3 /// Cpan | alpha-1-inhibitor III /// C3 and PZP-like, alpha-2-ma | uniqAntib_1098 | -0.10 | 0.06 | -0.10 | -0.43 | -0.04 | 0 | -0.1612 | -1.1182 | 2.18677 | 0.07608 | 1 | -0.4839 | -1.3985 | 3.865 | 0.00075 | 0 | -0.0986 | -1.0707 | 1.44002 | 0.14604 | 0 | 0 |
| 1370043_at | Alcam | activated leukocyte cell adhesion molecule | uniqAntib_1098 | 0.00 | -0.24 | -0.26 | 0.28 | -0.16 | 0 | -0.0212 | -1.0148 | 1.05838 | 0.36759 | 1 | 0.52459 | 1.43852 | 0.2978 | 0.00104 | 0 | 0.08032 | 1.05725 | 1.34402 | 0.20295 | 0 | 0 |

| Affymetrix Probeset ID | Gene Symbol | Gene Title | Figure 6 B Venn n =2021 | Mean BN log2 intensity autologous n=8 | Mean log2 intensity day 30 DR +/+ ND n=4 | Mean log2 intensity day 30 DR+/+ HCD n=4 | Mean log2 intensity day 30 DR+/+ B/S n=4 | Mean log2 intensity day 30 F+/+ ND n=4 | DR+/+ HCD vs DR+/+ ND n=380 1=yes 0=no | Log2 ratio DR+/+ HCD vs DR+/+ ND | Fold-Change DR+/+ HCD vs DR+/+ ND | RP-FDR 4DR30 HCD vs 4DR30 Reg | p value DR+/+ HCD vs DR+/+ ND | DR+/+ B/S vs DR+/+ ND n=1,330 1=yes 0=no | Log2 ratio DR+/+ B/S vs DR+/+ ND | Fold-Change DR+/+ B/S vs DR+/+ ND | RP-FDR 4DR30 Antib vs 4DR30 Reg | p value DR+/+ B/S vs DR+/+ ND | DR+/+ ND vs F+/+ ND n=398 1=yes 0=no | Log2 ratio DR+/+ ND vs F+/+ ND | Fold-Change DR+/+ ND vs F+/+ ND | RP-FDR 4RT1uu 30Reg vs 4DR30 Reg | p value DR+/+ ND vs F+/+ ND | DR+/+ Longitudinal n=228 1=yes 0=no | Figure 6F |
|---|---|---|---|---|---|---|---|---|---|---|---|---|---|---|---|---|---|---|---|---|---|---|---|---|---|
| 1370073_at | Dnajc3 | DnaJ (Hsp40) homolog, subfamily C, member 3 | uniqAntib_1098 | -0.04 | -0.44 | -0.43 | -0.18 | -0.38 | 0 | 0.01723 | 1.01201 | 1.03993 | 0.40803 | 1 | 0.26616 | 1.2026 | 1.2604 | 0.0381 | 0 | 0.05829 | 1.04123 | 1.20394 | 0.26937 | 0 | 0 |
| 1370080_at | Hmox1 | heme oxygenase (decycling) 1 | uniqAntib_1098 | -0.08 | -0.11 | -0.13 | 0.23 | 0.14 | 0 | -0.0182 | -1.0127 | 1.07152 | 0.35985 | 1 | 0.34144 | 1.26702 | 1.0123 | 0.0154 | 0 | 0.2482 | 1.18772 | 2.29992 | 0.03483 | 0 | 0 |
| 1370084_at | Cpb1 | carboxypeptidase B1 (tissue) | uniqAntib_1098 | 0.01 | 0.05 | -0.08 | -0.33 | -0.16 | 0 | -0.1294 | -1.0939 | 1.40011 | 0.20336 | 1 | -0.3717 | -1.2939 | 2.48298 | 0.0083 | 0 | -0.2073 | -1.1545 | 1.85031 | 0.06789 | 0 | 0 |
| 1370101_at | Crx | cone-rod homeobox | uniqAntib_1098 | 0.14 | 0.15 | -0.07 | -0.19 | -0.09 | 0 | -0.2176 | -1.1628 | 2.0907 | 0.08397 | 1 | -0.3358 | -1.2621 | 2.09751 | 0.01383 | 0 | -0.2418 | -1.1824 | 2.23116 | 0.03594 | 0 | 0 |
| 1370143_at | Smo | smoothened, frizzled family receptor | uniqAntib_1098 | 0.14 | 0.14 | 0.10 | -0.13 | 0.08 | 0 | -0.0405 | -1.0284 | 1.31966 | 0.23207 | 1 | -0.2682 | -1.2043 | 1.84246 | 0.0202 | 0 | -0.0559 | -1.0395 | 1.16176 | 0.27114 | 0 | 0 |
| 1370145_at | --- | --- | uniqAntib_1098 | 0.07 | -0.22 | -0.04 | 0.16 | -0.21 | 0 | 0.17363 | 1.12789 | 1.8803 | 0.06179 | 1 | 0.37515 | 1.29697 | 0.96509 | 0.01232 | 0 | 0.00977 | 1.00679 | 1.37218 | 0.19189 | 0 | 0 |
| 1370192_at | Stx12 | syntaxin 12 | uniqAntib_1098 | -0.12 | -0.27 | -0.24 | 0.05 | -0.16 | 0 | 0.03027 | 1.0212 | 1.41652 | 0.1868 | 1 | 0.32381 | 1.25163 | 1.18373 | 0.02611 | 0 | 0.1109 | 1.07991 | 1.267 | 0.23756 | 0 | 0 |
| 1370226_at | Cstb | cystatin B (stefin B) | uniqAntib_1098 | -0.05 | -0.29 | -0.30 | 0.12 | -0.19 | 0 | -0.0182 | -1.0127 | 0.97052 | 0.45107 | 1 | 0.40514 | 1.32421 | 0.71148 | 0.00526 | 0 | 0.09867 | 1.07078 | 1.20714 | 0.26605 | 0 | 0 |
| 1370245_at | Ctsl1 | cathepsin L1 | uniqAntib_1098 | 0.04 | -0.28 | -0.23 | 0.13 | -0.13 | 0 | 0.04876 | 1.03437 | 1.1048 | 0.34662 | 1 | 0.41257 | 1.33105 | 0.77975 | 0.00692 | 0 | 0.15094 | 1.11029 | 1.47283 | 0.1578 | 0 | 0 |
| 1370252_at | Avpi1 | arginine vasopressin-induced 1 | uniqAntib_1098 | -0.11 | -0.16 | -0.15 | 0.14 | -0.05 | 0 | 0.00858 | 1.00596 | 0.88589 | 0.64062 | 1 | 0.30229 | 1.2331 | 1.26257 | 0.03873 | 0 | 0.1099 | 1.07915 | 1.15044 | 0.29835 | 0 | 0 |
| 1370286_at | Slc38a2 | solute carrier family 38, member 2 | uniqAntib_1098 | -0.03 | 0.04 | 0.07 | 0.38 | 0.03 | 0 | 0.02923 | 1.02047 | 0.92204 | 0.55677 | 1 | 0.33698 | 1.26311 | 1.16937 | 0.02373 | 0 | -0.0143 | -1.0099 | 0.91831 | 0.58331 | 0 | 0 |
| 1370291_at | Pdlim3 | PDZ and LIM domain 3 | uniqAntib_1098 | 0.00 | 0.03 | -0.02 | -0.27 | -0.08 | 0 | -0.0448 | -1.0316 | 1.35803 | 0.21803 | 1 | -0.2953 | -1.2272 | 1.75843 | 0.02443 | 0 | -0.1091 | -1.0786 | 1.36593 | 0.16778 | 0 | 0 |
| 1370407_at | Pcyox1 | prenylcysteine oxidase 1 | uniqAntib_1098 | -0.08 | 0.03 | 0.14 | 0.36 | 0.17 | 0 | 0.11086 | 1.07987 | 1.22143 | 0.26861 | 1 | 0.33239 | 1.2591 | 1.17286 | 0.02504 | 0 | 0.14121 | 1.10283 | 1.34155 | 0.20422 | 0 | 0 |
| 1370408_at | Smim3 | small integral membrane protein 3 | uniqAntib_1098 | -0.06 | -0.66 | -0.56 | -0.37 | -0.40 | 0 | 0.10372 | 1.07454 | 1.34449 | 0.21383 | 1 | 0.29352 | 1.22563 | 1.17849 | 0.02437 | 0 | 0.25448 | 1.19291 | 2.37291 | 0.03121 | 0 | 0 |
| 1370410_at | Igsf1 | immunoglobulin superfamily, member 1 | uniqAntib_1098 | -0.02 | 0.01 | 0.10 | -0.29 | 0.01 | 0 | 0.09763 | 1.07001 | 1.22576 | 0.23769 | 1 | -0.2938 | -1.2259 | 1.64374 | 0.0314 | 0 | 0.00365 | 1.00254 | 0.9208 | 0.55614 | 0 | 0 |
| 1370435_a_at | Nudt6 | nudix (nucleoside diphosphate linked moiety X)-typ | uniqAntib_1098 | -0.20 | -0.37 | -0.45 | -0.65 | -0.35 | 0 | -0.0778 | -1.0554 | 1.43861 | 0.19077 | 1 | -0.2745 | -1.2096 | 1.78308 | 0.02368 | 0 | 0.02051 | 1.01432 | 1.0305 | 0.39584 | 0 | 0 |
| 1370453_at | Tex101 | testis expressed 101 | uniqAntib_1098 | 0.08 | 0.05 | -0.14 | -0.22 | -0.16 | 0 | -0.1962 | -1.1457 | 2.08866 | 0.08261 | 1 | -0.2752 | -1.2101 | 1.44962 | 0.04582 | 0 | -0.2113 | -1.1577 | 1.83599 | 0.07002 | 0 | 0 |
| 1370487_a_at | Kalrn | kalirin, RhoGEF kinase | uniqAntib_1098 | 0.05 | 0.04 | 0.07 | -0.25 | -0.10 | 0 | 0.02948 | 1.02065 | 0.97905 | 0.47339 | 1 | -0.2869 | -1.2201 | 1.69412 | 0.02762 | 0 | -0.1424 | -1.1037 | 1.55887 | 0.11093 | 0 | 0 |
| 1370488_a_at | Ptprs | protein tyrosine phosphatase, receptor type, S | uniqAntib_1098 | 0.20 | 0.04 | 0.28 | 0.31 | -0.16 | 0 | 0.24075 | 1.1816 | 2.09153 | 0.03208 | 1 | 0.2658 | 1.20231 | 1.16738 | 0.02346 | 0 | -0.2044 | -1.1522 | 2.75205 | 0.02203 | 0 | 0 |
| 1370491_a_at | Hdc | histidine decarboxylase | uniqAntib_1098 | 0.11 | -0.40 | -0.26 | -0.05 | -0.18 | 0 | 0.13878 | 1.10098 | 1.52183 | 0.15189 | 1 | 0.35384 | 1.27796 | 1.0043 | 0.01456 | 0 | 0.2192 | 1.16409 | 1.97857 | 0.06496 | 0 | 0 |
| 1370506_at | Coq7 | coenzyme Q7 homolog, ubiquinone (yeast) | uniqAntib_1098 | -0.12 | -0.28 | -0.22 | 0.00 | -0.21 | 0 | 0.05912 | 1.04183 | 1.14842 | 0.31418 | 1 | 0.27354 | 1.20877 | 1.24473 | 0.03422 | 0 | 0.06999 | 1.04971 | 1.18606 | 0.27875 | 0 | 0 |
| 1370588_a_at | Slc8a1 | solute carrier family 8 (sodium/calcium exchanger), | uniqAntib_1098 | -0.04 | -0.02 | -0.07 | -0.28 | -0.02 | 0 | -0.0506 | -1.0357 | 0.97167 | 0.44995 | 1 | -0.2655 | -1.2021 | 1.45852 | 0.04516 | 0 | -0.0073 | -1.0051 | 0.93054 | 0.5502 | 0 | 0 |
| 1370620_at | Ccl22 | chemokine (C-C motif) ligand 22 | uniqAntib_1098 | 0.28 | 0.21 | 0.19 | -0.12 | 0.01 | 0 | -0.0136 | -1.0095 | 1.42257 | 0.19359 | 1 | -0.3294 | -1.2565 | 2.42583 | 0.00991 | 0 | -0.1931 | -1.1432 | 2.12023 | 0.04431 | 0 | 0 |
| 1370675_at | Trpv1 | transient receptor potential cation channel, subfam | uniqAntib_1098 | -0.12 | 0.04 | -0.06 | -0.28 | -0.01 | 0 | -0.1043 | -1.075 | 1.65741 | 0.14299 | 1 | -0.3229 | -1.2508 | 2.4424 | 0.00982 | 0 | -0.0521 | -1.0368 | 1.21053 | 0.23935 | 0 | 0 |
| 1370682_at | Lilrb3l | leukocyte immunoglobulin-like receptor, subfamily | uniqAntib_1098 | -0.06 | -0.05 | -0.10 | 0.38 | 0.14 | 0 | -0.0506 | -1.0357 | 1.03838 | 0.39015 | 1 | 0.43107 | 1.34824 | 0.62108 | 0.00407 | 0 | 0.18718 | 1.13853 | 1.95096 | 0.06901 | 0 | 0 |
| 1370704_at | Kcnj6 | potassium inwardly-rectifying channel, subfamily J, | uniqAntib_1098 | 0.03 | 0.21 | 0.10 | -0.07 | -0.02 | 0 | -0.111 | -1.08 | 1.27907 | 0.24616 | 1 | -0.282 | -1.2158 | 1.53071 | 0.03874 | 0 | -0.2342 | -1.1763 | 2.06079 | 0.04685 | 0 | 0 |
| 1370733_at | Lipogenin | Lipogenin | uniqAntib_1098 | 0.00 | 0.18 | 0.24 | -0.09 | 0.13 | 0 | 0.06105 | 1.04322 | 1.63015 | 0.12266 | 1 | -0.2691 | -1.2051 | 2.04643 | 0.01454 | 0 | -0.0513 | -1.0362 | 1.62954 | 0.0993 | 0 | 0 |
| 1370770_s_at | Kitlg | KIT ligand | uniqAntib_1098 | -0.06 | -0.04 | -0.20 | -0.39 | -0.18 | 0 | -0.165 | -1.1212 | 1.66145 | 0.14286 | 1 | -0.3549 | -1.2789 | 2.00214 | 0.01564 | 0 | -0.1365 | -1.0993 | 1.38039 | 0.16361 | 0 | 0 |
| 1370805_at | Cited1 /// RG | Cbp/p300-interacting transactivator with Glu/Asp-ri | uniqAntib_1098 | 0.03 | 0.08 | -0.03 | -0.29 | -0.15 | 0 | -0.1066 | -1.0767 | 1.21077 | 0.27627 | 1 | -0.3663 | -1.289 | 2.41701 | 0.01041 | 0 | -0.2305 | -1.1733 | 2.03809 | 0.04876 | 0 | 0 |
| 1370813_at | Gstm5 /// LO | glutathione S-transferase, mu 5 /// glutathione S-tra | uniqAntib_1098 | 0.03 | -0.34 | -0.32 | 0.02 | -0.17 | 0 | 0.01972 | 1.01376 | 1.328 | 0.21957 | 1 | 0.36095 | 1.28427 | 0.7559 | 0.00634 | 0 | 0.17191 | 1.12655 | 2.18804 | 0.047 | 0 | 0 |
| 1370837_at | Sycn | syncollin | uniqAntib_1098 | 0.01 | 0.06 | -0.13 | -0.23 | -0.14 | 0 | -0.1884 | -1.1395 | 2.61301 | 0.04243 | 1 | -0.2907 | -1.2233 | 1.67532 | 0.02817 | 0 | -0.208 | -1.1551 | 1.85162 | 0.06782 | 0 | 0 |
| 1370912_at | Hspa1a | heat shock 70kD protein 1A | uniqAntib_1098 | 0.21 | 0.17 | 0.12 | -0.20 | 0.17 | 0 | -0.0516 | -1.0364 | 0.93314 | 0.49998 | 1 | -0.3693 | -1.2917 | 2.31116 | 0.01152 | 0 | -0.0043 | -1.003 | 0.95917 | 0.49681 | 0 | 0 |
| 1370930_at | Kif1c | kinesin family member 1C | uniqAntib_1098 | 0.15 | 0.10 | 0.12 | 0.38 | 0.35 | 0 | 0.0203 | 1.01417 | 1.21696 | 0.3022 | 1 | 0.28328 | 1.21696 | 1.202 | 0.02729 | 0 | 0.2525 | 1.19127 | 2.35434 | 0.01711 | 0 | 0 |
| 1370948_a_at | Marcks | myristoylated alanine rich protein kinase C substrat | uniqAntib_1098 | -0.12 | -0.40 | -0.27 | -0.08 | -0.26 | 0 | 0.12885 | 1.09342 | 1.63134 | 0.12222 | 1 | 0.32614 | 1.25366 | 1.17422 | 0.0253 | 0 | 0.1419 | 1.10336 | 1.30081 | 0.22194 | 0 | 0 |
| 1370954_at | P4ha1 | prolyl 4-hydroxylase, alpha polypeptide I | uniqAntib_1098 | -0.14 | -0.21 | -0.31 | 0.08 | -0.38 | 0 | -0.0998 | -1.0716 | 1.39148 | 0.20649 | 1 | 0.2958 | 1.22756 | 1.25968 | 0.03787 | 0 | -0.1656 | -1.1216 | 1.90563 | 0.06164 | 0 | 0 |
| 1371033_at | LOC688090 // | similar to RT1 class II histocompatibility antigen, B-1 | uniqAntib_1098 | 0.10 | 0.97 | 0.85 | 0.68 | 0.88 | 0 | -0.1224 | -1.0885 | 2.65138 | 0.03956 | 1 | -0.291 | -1.2235 | 2.88169 | 0.00602 | 0 | -0.0862 | -1.0615 | 1.97245 | 0.05391 | 0 | 0 |
| 1371048_at | Foxe1 | forkhead box E1 (thyroid transcription factor 2) | uniqAntib_1098 | 0.01 | 0.00 | 0.00 | -0.29 | -0.06 | 0 | -0.0028 | -1.002 | 0.87562 | 0.63489 | 1 | -0.2908 | -1.2233 | 1.52782 | 0.03876 | 0 | -0.0596 | -1.0422 | 1.05123 | 0.36182 | 0 | 0 |
| 1371081_at | Rapgef4 | Rap guanine nucleotide exchange factor (GEF) 4 | uniqAntib_1098 | -0.22 | -0.05 | 0.03 | -0.41 | -0.11 | 0 | 0.08004 | 1.05705 | 1.16679 | 0.30315 | 1 | -0.3607 | -1.284 | 3.20604 | 0.00495 | 0 | -0.0514 | -1.0363 | 1.07946 | 0.33235 | 0 | 0 |
| 1371090_at | Scamp2 | secretory carrier membrane protein 2 | uniqAntib_1098 | 0.09 | 0.14 | 0.30 | 0.46 | 0.28 | 0 | 0.15521 | 1.11359 | 1.62999 | 0.12238 | 1 | 0.32296 | 1.2509 | 1.12142 | 0.02052 | 0 | 0.14289 | 1.10411 | 1.57391 | 0.13148 | 0 | 0 |
| 1371098_a_at | Masp2 | mannan-binding lectin serine peptidase 2 | uniqAntib_1098 | 0.03 | -0.04 | -0.13 | -0.33 | -0.02 | 0 | -0.0922 | -1.066 | 1.38964 | 0.2072 | 1 | -0.2912 | -1.2236 | 2.19601 | 0.03357 | 0 | 0.01294 | 1.00901 | 0.92104 | 0.55519 | 0 | 0 |
| 1371113_a_at | Tfrc | transferrin receptor | uniqAntib_1098 | -0.07 | -0.29 | -0.34 | 0.06 | -0.04 | 0 | -0.0438 | -1.0308 | 1.93807 | 0.10345 | 1 | 0.34812 | 1.2729 | 0.72787 | 0.00604 | 0 | 0.25382 | 1.19236 | 2.03582 | 0.00799 | 0 | 0 |
| 1371173_a_at | Cast | calpastatin | uniqAntib_1098 | -0.12 | -0.33 | -0.19 | -0.05 | -0.24 | 0 | 0.1441 | 1.10504 | 1.622 | 0.12856 | 1 | 0.2815 | 1.21546 | 1.20635 | 0.02851 | 0 | 0.09947 | 1.07138 | 1.47868 | 0.15743 | 0 | 0 |
| 1371228_at | Csf2 | colony stimulating factor 2 (granulocyte-macrophag | uniqAntib_1098 | 0.06 | 0.18 | -0.02 | -0.22 | 0.14 | 0 | -0.1967 | -1.1461 | 2.35938 | 0.06449 | 1 | -0.3943 | -1.3143 | 2.70272 | 0.00704 | 0 | -0.0392 | -1.0275 | 1.07557 | 0.33506 | 0 | 0 |
| 1371248_at | Sprr1a | small proline-rich protein 1A | uniqAntib_1098 | -0.02 | 0.11 | 0.13 | -0.20 | -0.05 | 0 | 0.02037 | 1.01422 | 1.16471 | 0.30587 | 1 | -0.3055 | -1.2358 | 2.00878 | 0.01486 | 0 | -0.1604 | -1.1176 | 1.74056 | 0.08362 | 0 | 0 |
| 1371261_at | Ptpn2 | protein tyrosine phosphatase, non-receptor type 2 | uniqAntib_1098 | -0.12 | -0.06 | -0.03 | 0.28 | 0.05 | 0 | 0.03 | 1.02101 | 0.98588 | 0.46433 | 1 | 0.33732 | 1.26341 | 1.12282 | 0.02051 | 0 | 0.10436 | 1.07501 | 1.40958 | 0.18167 | 0 | 0 |
| 1371283_at | --- | --- | uniqAntib_1098 | 0.10 | -0.01 | -0.09 | -0.32 | -0.09 | 0 | -0.0806 | -1.0574 | 1.25726 | 0.25469 | 1 | -0.3076 | -1.2377 | 1.97833 | 0.01788 | 0 | -0.0756 | -1.0538 | 1.05554 | 0.35594 | 0 | 0 |
| 1371332_at | --- | --- | uniqAntib_1098 | -0.10 | -0.38 | -0.45 | -0.98 | -0.53 | 0 | -0.0662 | -1.0469 | 0.98968 | 0.43347 | 1 | -0.5974 | -1.513 | 3.27 | 0.00011 | 0 | -0.1533 | -1.1121 | 1.30455 | 0.19128 | 0 | 0 |
| 1371350_at | Mat2a | methionine adenosyltransferase II, alpha | uniqAntib_1098 | -0.05 | -0.10 | -0.07 | 0.20 | -0.06 | 0 | 0.03185 | 1.02233 | 0.94604 | 0.5152 | 1 | 0.30544 | 1.2358 | 1.24037 | 0.0337 | 0 | 0.04284 | 1.03014 | 0.94938 | 0.4929 | 0 | 0 |
| 1371351_at | Rpo1-3 | RNA polymerase 1-3 | uniqAntib_1098 | -0.05 | -0.22 | -0.18 | 0.10 | -0.15 | 0 | 0.03632 | 1.0255 | 1.07419 | 0.37163 | 1 | 0.31252 | 1.24187 | 1.20433 | 0.02916 | 0 | 0.06846 | 1.0486 | 1.03158 | 0.3939 | 0 | 0 |
| 1371369_at | Col6a2 | collagen, type VI, alpha 2 | uniqAntib_1098 | -0.02 | 0.12 | 0.05 | -0.16 | -0.07 | 0 | -0.0686 | -1.0487 | 1.26533 | 0.25197 | 1 | -0.2761 | -1.2109 | 1.57691 | 0.03463 | 0 | -0.1876 | -1.1389 | 2.35326 | 0.0339 | 0 | 0 |
| 1371460_at | Cox14 | COX14 cytochrome c oxidase assembly | uniqAntib_1098 | 0.00 | -0.28 | -0.30 | -0.02 | -0.14 | 0 | -0.0132 | -1.0092 | 1.18946 | 0.28907 | 1 | 0.26706 | 1.20335 | 1.25594 | 0.03627 | 0 | 0.14593 | 1.10644 | 1.66522 | 0.11191 | 0 | 0 |
| 1371484_at | LOC690349 | hypothetical protein LOC690349 | uniqAntib_1098 | -0.04 | -0.37 | -0.37 | -0.08 | -0.29 | 0 | 0.00066 | 1.00046 | 1.07497 | 0.37062 | 1 | 0.2928 | 1.22501 | 1.20115 | 0.02777 | 0 | 0.07942 | 1.05659 | 1.38275 | 0.18834 | 0 | 0 |
| 1371523_at | Commd1 | copper metabolism (Murr1) domain containing 1 | uniqAntib_1098 | -0.08 | -0.33 | -0.21 | -0.04 | -0.30 | 0 | 0.11566 | 1.08347 | 1.44878 | 0.17479 | 1 | 0.28744 | 1.22048 | 1.27561 | 0.04126 | 0 | 0.03435 | 1.02409 | 1.27082 | 0.23529 | 0 | 0 |
| 1371566_at | Fbxl22 | F-box and leucine-rich repeat protein 22 | uniqAntib_1098 | -0.09 | -0.02 | 0.15 | 0.30 | 0.21 | 0 | 0.16671 | 1.1225 | 1.86737 | 0.06449 | 1 | 0.32063 | 1.24888 | 0.92883 | 0.01021 | 0 | 0.22395 | 1.16793 | 2.27651 | 0.03623 | 0 | 0 |
| 1371643_at | Ccnd1 | cyclin D1 | uniqAntib_1098 | 0.23 | 0.09 | 0.17 | -0.23 | 0.05 | 0 | 0.08082 | 1.05762 | 1.34254 | 0.21507 | 1 | -0.3273 | -1.2547 | 2.23647 | 0.01201 | 0 | -0.042 | -1.0295 | 1.09982 | 0.31786 | 0 | 0 |
| 1371746_at | Srl | sarcalumenin | uniqAntib_1098 | 0.11 | 0.23 | 0.00 | -0.07 | 0.03 | 0 | -0.2273 | -1.1707 | 2.31501 | 0.06677 | 1 | -0.2944 | -1.2264 | 1.49763 | 0.04233 | 0 | -0.1963 | -1.1458 | 1.69838 | 0.09306 | 0 | 0 |
| 1371757_s_at | LOC10036521 | keratin 6A-like /// similar to keratin complex 2, basi | uniqAntib_1098 | 0.14 | 0.07 | 0.04 | -0.21 | 0.03 | 0 | -0.0319 | -1.0223 | 1.36338 | 0.21613 | 1 | -0.2858 | -1.2191 | 1.5799 | 0.03394 | 0 | -0.0452 | -1.0318 | 1.20079 | 0.2443 | 0 | 0 |
| 1371779_at | Snx6 | sorting nexin 6 | uniqAntib_1098 | -0.07 | -0.19 | -0.16 | 0.08 | -0.13 | 0 | 0.03222 | 1.02259 | 1.31123 | 0.22469 | 1 | 0.26895 | 1.20493 | 1.28402 | 0.04282 | 0 | 0.06361 | 1.04508 | 1.12843 | 0.31354 | 0 | 0 |
| 1371862_at | Rrm1 | ribonucleotide reductase M1 | uniqAntib_1098 | -0.18 | -0.34 | -0.39 | -0.06 | -0.27 | 0 | -0.0479 | -1.0338 | 1.18743 | 0.34828 | 1 | 0.27983 | 1.21405 | 1.26935 | 0.04645 | 0 | 0.0755 | 1.05373 | 1.09582 | 0.33781 | 0 | 0 |
| 1371875_at | Manba | mannosidase, beta A, lysosomal | uniqAntib_1098 | -0.09 | -0.87 | -0.84 | -0.58 | -0.77 | 0 | 0.02748 | 1.01923 | 1.24664 | 0.25539 | 1 | 0.29383 | 1.22589 | 1.245 | 0.03467 | 0 | 0.10404 | 1.07478 | 1.3257 | 0.21114 | 0 | 0 |
| 1371885_at | LOC10091177 | tubulin-folding cofactor B-like /// tubulin folding cof | uniqAntib_1098 | -0.05 | -0.22 | -0.14 | 0.06 | -0.14 | 0 | 0.08176 | 1.0583 | 1.13213 | 0.32727 | 1 | 0.28695 | 1.22006 | 1.24586 | 0.03465 | 0 | 0.08521 | 1.06084 | 1.09359 | 0.33966 | 0 | 0 |
| 1371899_at | Prkra | protein kinase, interferon inducible double strandec | uniqAntib_1098 | -0.16 | -0.20 | -0.22 | 0.16 | -0.17 | 0 | -0.0164 | -1.0114 | 0.97815 | 0.44358 | 1 | 0.36324 | 1.28631 | 0.96301 | 0.01223 | 0 | 0.02925 | 1.02048 | 1.04522 | 0.38005 | 0 | 0 |
| 1371921_at | Ctnna1 | catenin (cadherin-associated protein), alpha 1, 102k | uniqAntib_1098 | 0.03 | -0.41 | -0.34 | -0.06 | -0.30 | 0 | 0.07785 | 1.05544 | 1.29129 | 0.23547 | 1 | 0.35568 | 1.27959 | 0.93009 | 0.01014 | 0 | 0.11742 | 1.08479 | 1.46606 | 0.16085 | 0 | 0 |
| 1372195_at | Tnnc2 | troponin C type 2 (fast) | uniqAntib_1098 | -0.09 | -0.24 | -0.05 | 0.12 | -0.13 | 0 | 0.18805 | 1.13923 | 1.63181 | 0.12315 | 1 | 0.35895 | 1.28249 | 1.0436 | 0.01658 | 0 | 0.11356 | 1.08189 | 1.41053 | 0.17956 | 0 | 0 |
| 1372305_at | Copz2 | coatomer protein complex, subunit zeta 2 | uniqAntib_1098 | -0.01 | -0.06 | 0.03 | -0.40 | -0.16 | 0 | 0.09138 | 1.06539 | 1.24016 | 0.25861 | 1 | -0.3351 | -1.2615 | 2.18511 | 0.01293 | 0 | -0.1026 | -1.0737 | 1.25233 | 0.21798 | 0 | 0 |
| 1372316_at | Pkdcc | protein kinase domain containing, cytoplasmic | uniqAntib_1098 | -0.07 | 0.00 | -0.01 | -0.33 | -0.15 | 0 | -0.0012 | -1.0008 | 0.94366 | 0.48347 | 1 | -0.3263 | -1.2538 | 1.86147 | 0.01999 | 0 | -0.1469 | -1.1072 | 1.32311 | 0.18316 | 0 | 0 |
| 1372368_at | --- | --- | uniqAntib_1098 | -0.10 | -0.29 | -0.30 | 0.08 | -0.26 | 0 | -0.0054 | -1.0038 | 0.94777 | 0.51269 | 1 | 0.36754 | 1.29015 | 1.02097 | 0.01596 | 0 | 0.03096 | 1.02169 | 0.94238 | 0.50829 | 0 | 0 |
| 1372373_at | Wdr13 | WD repeat domain 13 | uniqAntib_1098 | -0.04 | -0.10 | 0.03 | 0.21 | 0.03 | 0 | 0.12705 | 1.09206 | 1.299 | 0.23074 | 1 | 0.3134 | 1.24263 | 1.20386 | 0.02837 | 0 | 0.1264 | 1.09157 | 1.30997 | 0.21702 | 0 | 0 |
| 1372469_at | LOC686310 | similar to Ephrin type-B receptor 4 precursor (Tyrosi | uniqAntib_1098 | -0.01 | -0.04 | -0.20 | -0.40 | -0.22 | 0 | -0.1621 | -1.1189 | 1.42208 | 0.19361 | 1 | -0.3624 | -1.2856 | 2.00163 | 0.01583 | 0 | -0.1866 | -1.1381 | 1.53643 | 0.11422 | 0 | 0 |
| 1372473_at | Tjp1 | tight junction protein 1 | uniqAntib_1098 | 0.15 | 0.18 | 0.33 | 0.51 | 0.33 | 0 | 0.15587 | 1.1141 | 1.48111 | 0.16359 | 1 | 0.33258 | 1.25926 | 0.97954 | 0.0137 | 0 | 0.15598 | 1.11418 | 1.6808 | 0.10685 | 0 | 0 |
| 1372521_at | Rnd2 | Rho family GTPase 2 | uniqAntib_1098 | 0.01 | -0.05 | -0.13 | -0.37 | -0.01 | 0 | -0.0875 | -1.0625 | 1.27902 | 0.24623 | 1 | -0.3226 | -1.2506 | 1.80024 | 0.02171 | 0 | 0.04077 | 1.02867 | 0.99662 | 0.43382 | 0 | 0 |

| Affymetrix Probeset ID | Gene Symbol | Gene Title | Figure 6 B Venn n =2021 | Mean BN log2 intensity autologous n=8 | Mean log2 intensity day 30 DR +/+ ND n=4 | Mean log2 intensity day 30 DR+/+ HCD n=4 | Mean log2 intensity day 30 B/S n=4 | Mean log2 intensity day 30 F+/+ ND n=4 | DR+/+ HCD vs DR+/+ ND n=380 1=yes 0=no | Log2 ratio DR+/+ HCD vs DR+/+ ND | Fold-Change DR+/+ HCD vs DR+/+ ND | RP-FDR 4DR30 HCD vs 4DR30 Reg | p value DR+/+ HCD vs DR+/+ ND | DR+/+ B/S vs DR+/+ ND n=1,330 1=yes 0=no | Log2 ratio DR+/+ B/S vs DR+/+ ND | Fold-Change DR+/+ B/S vs DR+/+ ND | RP-FDR 4DR30 Antib vs 4DR30 Reg | p value DR+/+ B/S vs DR+/+ ND | DR+/+ ND vs F+/+ ND n=398 1=yes 0=no | Log2 ratio DR+/+ ND vs F+/+ ND | Fold-Change DR+/+ ND vs F+/+ ND | RP-FDR 4RT1uu 30Reg vs 4DR30 Reg | p value DR+/+ ND vs F+/+ ND | DR+/+ Longitudinal n=228 1=yes 0=no | Figure 6F |
|---|---|---|---|---|---|---|---|---|---|---|---|---|---|---|---|---|---|---|---|---|---|---|---|---|---|
| 1372542_at | --- | --- | uniqAntib_1098 | 0.00 | 0.00 | 0.01 | 0.40 | 0.20 | 0 | 0.01677 | 1.01169 | 0.91572 | 0.56888 | 1 | 0.39872 | 1.31834 | 0.83765 | 0.00813 | 0 | 0.20022 | 1.14888 | 1.73751 | 0.09638 | 0 | 0 |
| 1372543_at | Pbdc1 | polysaccharide biosynthesis domain containing 1 | uniqAntib_1098 | -0.02 | -0.29 | -0.31 | -0.01 | -0.24 | 0 | -0.0233 | -1.0163 | 1.04569 | 0.38083 | 1 | 0.27829 | 1.21275 | 1.25531 | 0.03617 | 0 | 0.04309 | 1.03032 | 0.99998 | 0.42898 | 0 | 0 |
| 1372562_at | MGC94207 | similar to RIKEN cDNA C030006K11 | uniqAntib_1098 | 0.03 | 0.17 | 0.14 | -0.13 | 0.13 | 0 | -0.0333 | -1.0233 | 0.95745 | 0.46612 | 1 | -0.2941 | -1.2261 | 1.56236 | 0.03617 | 0 | -0.0427 | -1.03 | 0.95363 | 0.50685 | 0 | 0 |
| 1372597_at | Mrpl14 | mitochondrial ribosomal protein L14 | uniqAntib_1098 | -0.12 | -0.48 | -0.43 | -0.21 | -0.38 | 0 | 0.05192 | 1.03664 | 1.20158 | 0.28093 | 1 | 0.2724 | 1.20782 | 1.17664 | 0.02554 | 0 | 0.10826 | 1.07793 | 1.71519 | 0.10187 | 0 | 0 |
| 1372716_at | --- | --- | uniqAntib_1098 | 0.00 | -0.07 | -0.02 | 0.21 | 0.01 | 0 | 0.04776 | 1.03366 | 1.12023 | 0.33431 | 1 | 0.27756 | 1.21214 | 1.27983 | 0.04453 | 0 | 0.07258 | 1.0516 | 1.16561 | 0.2919 | 0 | 0 |
| 1372849_at | --- | --- | uniqAntib_1098 | -0.11 | -0.23 | -0.31 | 0.06 | -0.14 | 0 | -0.0827 | -1.059 | 1.27138 | 0.24909 | 1 | 0.29184 | 1.2242 | 1.27758 | 0.0417 | 0 | 0.09375 | 1.06714 | 1.27339 | 0.23483 | 0 | 0 |
| 1372914_at | Ltbr | lymphotoxin beta receptor (TNFR superfamily, mem | uniqAntib_1098 | -0.03 | -0.09 | -0.10 | 0.18 | 0.10 | 0 | -0.0083 | -1.0058 | 1.18376 | 0.29131 | 1 | 0.2713 | 1.20689 | 1.25802 | 0.03713 | 0 | 0.18373 | 1.13581 | 1.88829 | 0.07651 | 0 | 0 |
| 1372923_at | --- | --- | uniqAntib_1098 | -0.07 | 0.09 | -0.03 | -0.18 | 0.07 | 0 | -0.1148 | -1.0828 | 1.31188 | 0.2345 | 1 | -0.2676 | -1.2038 | 1.51444 | 0.04018 | 0 | -0.0159 | -1.0111 | 0.99433 | 0.43953 | 0 | 0 |
| 1372936_at | Pcp2 | Purkinje cell protein 2 | uniqAntib_1098 | -0.06 | -0.10 | -0.15 | -0.36 | -0.17 | 0 | -0.0516 | -1.0364 | 1.22955 | 0.26462 | 1 | -0.2692 | -1.2051 | 1.51356 | 0.0403 | 0 | -0.078 | -1.0556 | 1.01317 | 0.40769 | 0 | 0 |
| 1372940_at | --- | --- | uniqAntib_1098 | 0.14 | 0.02 | -0.13 | 0.29 | 0.10 | 0 | -0.1528 | -1.1117 | 1.59089 | 0.15556 | 1 | 0.27083 | 1.2065 | 1.27732 | 0.04834 | 0 | 0.08527 | 1.06089 | 1.32642 | 0.20976 | 0 | 0 |
| 1372992_at | --- | --- | uniqAntib_1098 | 0.13 | 0.18 | 0.14 | -0.10 | 0.05 | 0 | -0.0418 | -1.0294 | 1.06758 | 0.36261 | 1 | -0.2793 | -1.2136 | 1.5562 | 0.03738 | 0 | -0.1298 | -1.0941 | 1.35098 | 0.17433 | 0 | 0 |
| 1373088_at | Cystm1 | cysteine-rich transmembrane module containing 1 | uniqAntib_1098 | 0.01 | -0.51 | -0.57 | -0.08 | -0.33 | 0 | -0.0567 | -1.0401 | 1.29086 | 0.24282 | 1 | 0.43484 | 1.35176 | 0.6052 | 0.00385 | 0 | 0.17975 | 1.13269 | 1.9121 | 0.07095 | 0 | 0 |
| 1373108_at | LOC10091067 | protein phosphatase 1 regulatory subunit 3C-like //, | uniqAntib_1098 | 0.04 | -0.01 | 0.01 | -0.32 | 0.00 | 0 | 0.01243 | 1.00865 | 0.91138 | 0.54127 | 1 | -0.3136 | -1.2428 | 1.69718 | 0.0269 | 0 | 0.00724 | 1.00503 | 0.95819 | 0.47954 | 0 | 0 |
| 1373114_at | --- | --- | uniqAntib_1098 | 0.09 | 0.63 | 0.65 | 0.91 | 0.57 | 0 | 0.01913 | 1.01335 | 1.26451 | 0.24762 | 1 | 0.28017 | 1.21433 | 1.12245 | 0.02036 | 0 | -0.0522 | -1.0369 | 1.36488 | 0.16871 | 0 | 0 |
| 1373148_at | Cpxm2 | carboxypeptidase X (M14 family), member 2 | uniqAntib_1098 | 0.07 | 0.25 | 0.10 | -0.08 | 0.21 | 0 | -0.1514 | -1.1107 | 1.75759 | 0.12823 | 1 | -0.3373 | -1.2634 | 2.1995 | 0.01266 | 0 | -0.0461 | -1.0324 | 1.0393 | 0.37316 | 0 | 0 |
| 1373238_at | Tada1 | transcriptional adaptor 1 | uniqAntib_1098 | -0.10 | -0.25 | -0.21 | 0.11 | -0.22 | 0 | 0.03547 | 1.02489 | 1.33617 | 0.21702 | 1 | 0.35434 | 1.2784 | 1.0103 | 0.01527 | 0 | 0.0321 | 1.0225 | 0.98145 | 0.45158 | 0 | 0 |
| 1373248_at | Vsig10 | V-set and immunoglobulin domain containing 10 | uniqAntib_1098 | 0.02 | 0.25 | 0.14 | -0.01 | 0.00 | 0 | -0.1139 | -1.0822 | 1.53986 | 0.16696 | 1 | -0.2647 | -1.2014 | 1.56385 | 0.03616 | 0 | -0.2565 | -1.1946 | 2.89089 | 0.01878 | 0 | 0 |
| 1373267_at | Sh3yl1 | SH3 domain containing, Ysc84-like 1 (S. cerevisiae) | uniqAntib_1098 | 0.13 | -0.30 | -0.24 | 0.05 | -0.23 | 0 | 0.05896 | 1.04171 | 1.83578 | 0.07001 | 1 | 0.35738 | 1.28109 | 0.78804 | 0.00712 | 0 | 0.07154 | 1.05084 | 2.27788 | 0.0419 | 0 | 0 |
| 1373303_at | Scaf11 | SR-related CTD-associated factor 11 | uniqAntib_1098 | -0.02 | 0.03 | 0.24 | 0.43 | -0.05 | 0 | 0.20364 | 1.1516 | 2.82844 | 0.00291 | 1 | 0.39969 | 1.31922 | 0.25295 | 0.00085 | 0 | -0.0861 | -1.0615 | 2.8267 | 0.02118 | 0 | 0 |
| 1373317_at | Calml5 | calmodulin-like 5 | uniqAntib_1098 | -0.03 | 0.02 | -0.07 | -0.32 | -0.12 | 0 | -0.0908 | -1.0649 | 1.13593 | 0.31749 | 1 | -0.3421 | -1.2676 | 1.8219 | 0.02127 | 0 | -0.1401 | -1.102 | 1.27735 | 0.20607 | 0 | 0 |
| 1373387_at | Slirp | SRA stem-loop interacting RNA binding protein | uniqAntib_1098 | -0.07 | -0.52 | -0.54 | -0.23 | -0.44 | 0 | -0.0152 | -1.0106 | 1.36469 | 0.20629 | 1 | 0.28818 | 1.2211 | 1.14536 | 0.02261 | 0 | 0.08172 | 1.05828 | 1.5794 | 0.13113 | 0 | 0 |
| 1373416_at | Fndc3b | fibronectin type III domain containing 3B | uniqAntib_1098 | 0.17 | 0.09 | 0.01 | 0.49 | 0.20 | 0 | -0.0792 | -1.0564 | 1.11134 | 0.33138 | 1 | 0.40551 | 1.32456 | 0.83966 | 0.00802 | 0 | 0.11458 | 1.08266 | 1.30597 | 0.219 | 0 | 0 |
| 1373432_at | Marcks | myristoylated alanine rich protein kinase C substrat | uniqAntib_1098 | 0.01 | -0.31 | -0.22 | 0.00 | -0.15 | 0 | 0.087 | 1.06216 | 1.3651 | 0.20666 | 1 | 0.30506 | 1.23547 | 1.23149 | 0.03279 | 0 | 0.15882 | 1.11637 | 1.38213 | 0.18906 | 0 | 0 |
| 1373433_at | Hmgn5 /// Hr | high mobility group nucleosome binding domain 5 / | uniqAntib_1098 | -0.19 | -0.17 | -0.21 | 0.21 | -0.19 | 0 | -0.0433 | -1.0305 | 1.41759 | 0.19537 | 1 | 0.38163 | 1.30281 | 0.72265 | 0.00579 | 0 | -0.0206 | -1.0144 | 1.16855 | 0.26697 | 0 | 0 |
| 1373447_at | Hn1l | hematological and neurological expressed 1-like | uniqAntib_1098 | -0.05 | -0.11 | -0.16 | 0.18 | -0.05 | 0 | -0.0455 | -1.032 | 1.00277 | 0.42227 | 1 | 0.29524 | 1.22709 | 1.24084 | 0.03368 | 0 | 0.06935 | 1.04924 | 1.11297 | 0.3241 | 0 | 0 |
| 1373465_at | Pqlc1 | PQ loop repeat containing 1 | uniqAntib_1098 | 0.08 | -0.09 | -0.10 | 0.21 | 0.12 | 0 | -0.0176 | -1.0123 | 1.0314 | 0.39636 | 1 | 0.29413 | 1.22615 | 1.25779 | 0.03717 | 0 | 0.20633 | 1.15375 | 1.92812 | 0.06994 | 0 | 0 |
| 1373527_at | Eml3 | echinoderm microtubule associated protein like 3 | uniqAntib_1098 | -0.05 | 0.17 | 0.21 | 0.45 | 0.34 | 0 | 0.03676 | 1.02581 | 1.21667 | 0.2721 | 1 | 0.27533 | 1.21027 | 1.27862 | 0.04424 | 0 | 0.16391 | 1.12032 | 1.59846 | 0.12665 | 0 | 0 |
| 1373538_at | Usp1 | ubiquitin specific peptidase 1 | uniqAntib_1098 | -0.03 | -0.07 | -0.13 | 0.27 | -0.16 | 0 | -0.0572 | -1.0405 | 2.14969 | 0.07742 | 1 | 0.33914 | 1.265 | 0.53929 | 0.00269 | 0 | -0.0895 | -1.064 | 2.66095 | 0.02439 | 0 | 0 |
| 1373547_at | RGD1308147 | similar to expressed sequence AW209491 | uniqAntib_1098 | -0.13 | -0.40 | -0.40 | -0.12 | -0.23 | 0 | -0.0043 | -1.003 | 1.33195 | 0.21843 | 1 | 0.28198 | 1.21586 | 1.28013 | 0.04763 | 0 | 0.17156 | 1.12627 | 1.69963 | 0.10286 | 0 | 0 |
| 1373567_at | RGD1561530 | similar to Tle6 protein | uniqAntib_1098 | 0.05 | 0.09 | 0.08 | -0.19 | -0.13 | 0 | -0.0067 | -1.0046 | 1.31206 | 0.23512 | 1 | -0.2779 | -1.2124 | 1.5672 | 0.03689 | 0 | -0.2207 | -1.1653 | 1.84817 | 0.069 | 0 | 0 |
| 1373574_at | Cnpy4 | canopy 4 homolog (zebrafish) | uniqAntib_1098 | 0.02 | -0.22 | -0.13 | 0.22 | -0.01 | 0 | 0.09137 | 1.06538 | 1.13972 | 0.32247 | 1 | 0.43984 | 1.35645 | 0.59577 | 0.00362 | 0 | 0.21488 | 1.16061 | 2.15241 | 0.04997 | 0 | 0 |
| 1373587_at | Gnat1 | guanine nucleotide binding protein (G protein), alph | uniqAntib_1098 | 0.12 | 0.07 | -0.02 | -0.31 | -0.02 | 0 | -0.0898 | -1.0642 | 1.19165 | 0.2865 | 1 | -0.3794 | -1.3008 | 2.35444 | 0.01075 | 0 | -0.0943 | -1.0675 | 1.08206 | 0.32995 | 0 | 0 |
| 1373632_at | Taf9 | TAF9 RNA polymerase II, TATA box binding protein ( | uniqAntib_1098 | 0.00 | -0.27 | -0.18 | 0.11 | -0.18 | 0 | 0.08856 | 1.06331 | 1.30089 | 0.23015 | 1 | 0.38627 | 1.30701 | 0.77004 | 0.00676 | 0 | 0.09374 | 1.06713 | 1.36906 | 0.19295 | 0 | 0 |
| 1373642_at | Paqr6 | progestin and adipoQ receptor family member VI | uniqAntib_1098 | 0.00 | -0.01 | -0.02 | -0.27 | -0.01 | 0 | -0.0164 | -1.0114 | 1.51424 | 0.17397 | 1 | -0.2648 | -1.2015 | 2.05491 | 0.01454 | 0 | -0.0063 | -1.0044 | 1.12661 | 0.29416 | 0 | 0 |
| 1373703_at | --- | --- | uniqAntib_1098 | 0.12 | 0.53 | 0.49 | 0.24 | 0.55 | 0 | -0.0405 | -1.0285 | 1.2127 | 0.27483 | 1 | -0.287 | -1.2201 | 1.92622 | 0.01833 | 0 | 0.01836 | 1.01281 | 1.1285 | 0.31352 | 0 | 0 |
| 1373743_at | Rwdd2b | RWD domain containing 2B | uniqAntib_1098 | -0.02 | 0.07 | 0.17 | 0.44 | 0.11 | 0 | 0.09144 | 1.06543 | 1.23235 | 0.26272 | 1 | 0.36311 | 1.2862 | 1.00972 | 0.01532 | 0 | 0.03751 | 1.02634 | 0.97289 | 0.46212 | 0 | 0 |
| 1373765_at | LOC1009101 | GPI ethanolamine phosphate transferase 2-like | uniqAntib_1098 | -0.04 | 0.36 | 0.49 | 0.09 | 0.28 | 0 | 0.12225 | 1.08843 | 1.4629 | 0.17052 | 1 | -0.277 | -1.2116 | 2.3954 | 0.01071 | 0 | -0.0808 | -1.0576 | 1.5276 | 0.11588 | 0 | 0 |
| 1373780_at | Tspan1 | tetraspanin 1 | uniqAntib_1098 | 0.02 | 0.17 | 0.01 | -0.18 | -0.02 | 0 | -0.159 | -1.1165 | 1.65462 | 0.14456 | 1 | -0.3466 | -1.2716 | 1.90825 | 0.01927 | 0 | -0.183 | -1.1353 | 1.42052 | 0.15183 | 0 | 0 |
| 1373784_at | Cct8 | chaperonin containing Tcp1, subunit 8 (theta) | uniqAntib_1098 | -0.06 | -0.32 | -0.21 | -0.05 | -0.30 | 0 | 0.10593 | 1.07619 | 1.79864 | 0.07293 | 1 | 0.27129 | 1.20689 | 1.21513 | 0.03075 | 0 | 0.02307 | 1.01612 | 1.16861 | 0.28938 | 0 | 0 |
| 1373799_at | --- | --- | uniqAntib_1098 | 0.01 | 0.04 | -0.11 | -0.23 | -0.11 | 0 | -0.1501 | -1.1096 | 1.51413 | 0.17411 | 1 | -0.2681 | -1.2042 | 1.52687 | 0.03879 | 0 | -0.1424 | -1.1037 | 1.28555 | 0.20098 | 0 | 0 |
| 1373807_at | Vegfa | vascular endothelial growth factor A | uniqAntib_1098 | 0.02 | -0.10 | -0.22 | 0.24 | -0.12 | 0 | -0.1187 | -1.0858 | 1.31695 | 0.23257 | 1 | 0.33651 | 1.26269 | 1.03104 | 0.01621 | 0 | -0.0229 | -1.016 | 1.21693 | 0.23576 | 0 | 0 |
| 1373823_at | Cks2 | CDC28 protein kinase regulatory subunit 2 | uniqAntib_1098 | -0.04 | -0.26 | -0.29 | 0.20 | -0.17 | 0 | -0.0285 | -1.02 | 1.4218 | 0.19366 | 1 | 0.46132 | 1.3768 | 0.42703 | 0.00189 | 0 | 0.09347 | 1.06693 | 1.56382 | 0.13487 | 0 | 0 |
| 1373885_at | Cbx5 | chromobox homolog 5 | uniqAntib_1098 | -0.05 | -0.13 | 0.04 | 0.27 | -0.02 | 0 | 0.16829 | 1.12372 | 2.09553 | 0.03012 | 1 | 0.40307 | 1.32232 | 0.76408 | 0.00651 | 0 | 0.11625 | 1.08391 | 1.4132 | 0.17886 | 0 | 0 |
| 1373909_at | LOC689176 | similar to transmembrane protein 64 | uniqAntib_1098 | -0.02 | 0.01 | 0.07 | 0.30 | 0.13 | 0 | 0.05666 | 1.04005 | 1.14054 | 0.32167 | 1 | 0.29027 | 1.22287 | 1.23086 | 0.03281 | 0 | 0.11713 | 1.08457 | 1.46679 | 0.1606 | 0 | 0 |
| 1373913_at | Pnpt1 | polyribonucleotide nucleotidyltransferase 1 | uniqAntib_1098 | -0.13 | -0.38 | -0.35 | -0.12 | -0.27 | 0 | 0.03525 | 1.02474 | 1.28802 | 0.23686 | 1 | 0.26312 | 1.20007 | 1.26612 | 0.03994 | 0 | 0.11823 | 1.0854 | 1.55456 | 0.13777 | 0 | 0 |
| 1373959_at | Ppp2r1b | protein phosphatase 2, regulatory subunit A, beta | uniqAntib_1098 | -0.01 | -0.14 | -0.12 | 0.14 | -0.11 | 0 | 0.02291 | 1.01601 | 1.01628 | 0.42803 | 1 | 0.27549 | 1.21041 | 1.27008 | 0.0466 | 0 | 0.03314 | 1.02323 | 1.05482 | 0.37395 | 0 | 0 |
| 1373986_at | Fnip2 | folliculin interacting protein 2 | uniqAntib_1098 | 0.16 | 0.28 | 0.43 | 0.63 | 0.43 | 0 | 0.14935 | 1.10907 | 1.60372 | 0.13005 | 1 | 0.35195 | 1.27629 | 0.97 | 0.0131 | 0 | 0.15412 | 1.11275 | 1.69631 | 0.10293 | 0 | 0 |
| 1374015_at | --- | --- | uniqAntib_1098 | -0.07 | 0.09 | -0.14 | -0.23 | 0.06 | 0 | -0.2252 | -1.1689 | 2.54085 | 0.04902 | 1 | -0.316 | -1.2449 | 1.82977 | 0.02059 | 0 | -0.0299 | -1.021 | 1.04189 | 0.3701 | 0 | 0 |
| 1374043_at | Gramd3 | GRAM domain containing 3 | uniqAntib_1098 | -0.08 | -0.68 | -0.50 | -0.34 | -0.48 | 0 | 0.17852 | 1.13172 | 1.83639 | 0.0698 | 1 | 0.33593 | 1.26219 | 1.07625 | 0.01834 | 0 | 0.19894 | 1.14786 | 1.89392 | 0.07393 | 0 | 0 |
| 1374099_at | Nrap | nebulin-related anchoring protein | uniqAntib_1098 | 0.01 | 0.02 | 0.04 | -0.30 | -0.08 | 0 | 0.01591 | 1.01109 | 1.14793 | 0.31482 | 1 | -0.3174 | -1.2461 | 1.92359 | 0.01825 | 0 | -0.0982 | -1.0704 | 1.28416 | 0.20122 | 0 | 0 |
| 1374287_at | Pald1 | phosphatase domain containing, paladin 1 | uniqAntib_1098 | -0.03 | 0.01 | 0.01 | -0.33 | -0.13 | 0 | 0.00496 | 1.00344 | 0.95125 | 0.47427 | 1 | -0.3354 | -1.2618 | 1.82858 | 0.02064 | 0 | -0.1376 | -1.1001 | 1.33723 | 0.17866 | 0 | 0 |
| 1374317_at | Pik3r6 | phosphoinositide-3-kinase, regulatory subunit 6 | uniqAntib_1098 | -0.10 | -0.09 | -0.07 | 0.28 | 0.11 | 0 | 0.02555 | 1.01787 | 1.02906 | 0.41812 | 1 | 0.37369 | 1.29566 | 0.96825 | 0.01333 | 0 | 0.20544 | 1.15304 | 2.07474 | 0.06011 | 0 | 0 |
| 1374344_at | Lppr3 | lipid phosphate phosphatase-related protein type 3 | uniqAntib_1098 | 0.06 | 0.01 | 0.05 | -0.28 | -0.17 | 0 | 0.04514 | 1.03178 | 1.00996 | 0.43433 | 1 | -0.2896 | -1.2223 | 1.56413 | 0.03662 | 0 | -0.1728 | -1.1272 | 1.44017 | 0.14458 | 0 | 0 |
| 1374427_at | --- | --- | uniqAntib_1098 | -0.21 | -0.13 | -0.14 | -0.41 | -0.25 | 0 | -0.009 | -1.0063 | 0.97778 | 0.44379 | 1 | -0.2761 | -1.2109 | 1.62929 | 0.03159 | 0 | -0.1175 | -1.0848 | 1.4958 | 0.12659 | 0 | 0 |
| 1374432_at | --- | --- | uniqAntib_1098 | 0.06 | 0.00 | -0.09 | 0.31 | -0.01 | 0 | -0.0887 | -1.0634 | 1.31931 | 0.2321 | 1 | 0.31755 | 1.24621 | 1.16959 | 0.02366 | 0 | -0.0016 | -1.0011 | 1.07364 | 0.35649 | 0 | 0 |
| 1374480_at | Daam1 | dishevelled associated activator of morphogenesis 1 | uniqAntib_1098 | 0.00 | -0.12 | -0.13 | 0.14 | 0.05 | 0 | -0.001 | -1.0007 | 1.11607 | 0.33957 | 1 | 0.26732 | 1.20357 | 1.27954 | 0.04456 | 0 | 0.1779 | 1.13123 | 1.79119 | 0.08893 | 0 | 0 |
| 1374503_at | Pbx3 | pre-B-cell leukemia homeobox 3 | uniqAntib_1098 | -0.05 | -0.11 | 0.01 | 0.28 | -0.06 | 0 | 0.11873 | 1.08578 | 1.23955 | 0.25876 | 1 | 0.39524 | 1.31516 | 0.92812 | 0.00985 | 0 | 0.05394 | 1.0381 | 0.9897 | 0.44172 | 0 | 0 |
| 1374515_at | Spryd7 | SPRY domain containing 7 | uniqAntib_1098 | -0.07 | -0.43 | -0.37 | -0.11 | -0.37 | 0 | 0.0658 | 1.04666 | 1.75676 | 0.08903 | 1 | 0.32026 | 1.24856 | 0.96681 | 0.01265 | 0 | 0.06069 | 1.04296 | 1.13594 | 0.3092 | 0 | 0 |
| 1374530_at | --- | --- | uniqAntib_1098 | 0.02 | 0.03 | -0.17 | -0.24 | -0.09 | 0 | -0.1958 | -1.1454 | 1.95282 | 0.10223 | 1 | -0.2751 | -1.2101 | 1.56722 | 0.03467 | 0 | -0.1166 | -1.0842 | 1.20237 | 0.24361 | 0 | 0 |
| 1374591_at | Ptprd | protein tyrosine phosphatase, receptor type, D | uniqAntib_1098 | 0.08 | 0.09 | -0.06 | -0.24 | -0.15 | 0 | -0.1583 | -1.116 | 1.90324 | 0.11004 | 1 | -0.339 | -1.2648 | 2.07782 | 0.0141 | 0 | -0.2474 | -1.1871 | 2.36128 | 0.03333 | 0 | 0 |
| 1374610_at | --- | --- | uniqAntib_1098 | 0.15 | -0.11 | -0.04 | 0.63 | 0.14 | 0 | 0.06612 | 1.0469 | 1.71647 | 0.09736 | 1 | 0.73523 | 1.66466 | 0.02435 | 3.6E-05 | 0 | 0.24504 | 1.18512 | 2.31968 | 0.01872 | 0 | 0 |
| 1374685_at | --- | --- | uniqAntib_1098 | -0.21 | -0.29 | -0.17 | 0.22 | -0.22 | 0 | 0.1191 | 1.08606 | 1.56491 | 0.1445 | 1 | 0.50111 | 1.4153 | 0.37436 | 0.00141 | 0 | 0.06576 | 1.04664 | 1.15579 | 0.29658 | 0 | 0 |
| 1374686_at | Slc30a7 | solute carrier family 30 (zinc transporter), member 7 | uniqAntib_1098 | -0.15 | -0.41 | -0.47 | 0.01 | -0.48 | 0 | -0.058 | -1.041 | 1.67949 | 0.13912 | 1 | 0.41989 | 1.33783 | 0.72614 | 0.00586 | 0 | -0.07 | -1.0497 | 1.53006 | 0.11503 | 0 | 0 |
| 1374700_at | --- | --- | uniqAntib_1098 | -0.03 | 0.02 | 0.14 | 0.30 | 0.26 | 0 | 0.11936 | 1.08625 | 1.76402 | 0.07924 | 1 | 0.28441 | 1.21791 | 1.14411 | 0.02229 | 0 | 0.24496 | 1.18506 | 2.36876 | 0.02453 | 0 | 0 |
| 1374712_at | Psmd11 | proteasome (prosome, macropain) 26S subunit, nor | uniqAntib_1098 | -0.04 | -0.10 | 0.00 | 0.20 | -0.11 | 0 | 0.10406 | 1.07479 | 1.56147 | 0.14024 | 1 | 0.30426 | 1.23479 | 1.24196 | 0.03399 | 0 | -0.0098 | -1.0068 | 0.99197 | 0.44487 | 0 | 0 |
| 1374726_at | Fndc1 | fibronectin type III domain containing 1 | uniqAntib_1098 | 0.13 | 0.26 | 0.19 | -0.04 | 0.32 | 0 | -0.0697 | -1.0495 | 1.26228 | 0.25218 | 1 | -0.3065 | -1.2367 | 1.6371 | 0.03143 | 0 | 0.05988 | 1.04238 | 1.12253 | 0.31717 | 0 | 0 |
| 1374730_at | Tyrobp | Tyro protein tyrosine kinase binding protein | uniqAntib_1098 | -0.05 | -0.25 | -0.34 | 0.09 | -0.07 | 0 | -0.085 | -1.0607 | 1.13455 | 0.31849 | 1 | 0.34322 | 1.26859 | 1.0137 | 0.01561 | 0 | 0.17891 | 1.13203 | 1.49295 | 0.15237 | 0 | 0 |
| 1374742_at | --- | --- | uniqAntib_1098 | 0.08 | -0.13 | -0.22 | -0.44 | -0.17 | 0 | -0.0873 | -1.0624 | 1.51898 | 0.17247 | 1 | -0.3019 | -1.2327 | 1.95927 | 0.01802 | 0 | -0.04 | -1.0281 | 1.12074 | 0.29897 | 0 | 0 |
| 1374752_at | Mdfic | MyoD family inhibitor domain containing | uniqAntib_1098 | -0.02 | 0.09 | 0.07 | 0.39 | 0.18 | 0 | -0.0253 | -1.0177 | 1.04178 | 0.38614 | 1 | 0.29705 | 1.22863 | 1.25487 | 0.03636 | 0 | 0.08222 | 1.05864 | 1.15029 | 0.29849 | 0 | 0 |
| 1374789_at | Dcun1d2 | DCN1, defective in cullin neddylation 1, domain con | uniqAntib_1098 | -0.15 | -0.19 | -0.17 | 0.17 | -0.07 | 0 | 0.02214 | 1.01546 | 1.09823 | 0.35081 | 1 | 0.36253 | 1.28567 | 0.96962 | 0.013 | 0 | 0.12104 | 1.08752 | 1.3611 | 0.19577 | 0 | 0 |
| 1374829_at | Srf | serum response factor (c-fos serum response eleme | uniqAntib_1098 | -0.04 | 0.09 | -0.14 | -0.18 | 0.05 | 0 | -0.2292 | -1.1722 | 2.9759 | 0.03129 | 1 | -0.2773 | -1.2119 | 1.43487 | 0.04734 | 0 | -0.0397 | -1.0279 | 0.97764 | 0.46664 | 0 | 0 |

| Affymetrix Probeset ID | Gene Symbol | Gene Title | Figure 6 B Venn n =2021 | Mean BN log2 intensity autologous n=8 | Mean log2 intensity day 30 DR +/+ ND n=4 | Mean log2 intensity day 30 DR+/+ HCD n=4 | Mean log2 intensity day 30 B/S n=4 | Mean log2 intensity day 30 F+/+ ND n=4 | DR+/+ HCD vs DR+/+ ND n=380 1=yes 0=no | Log2 ratio DR+/+ HCD vs DR+/+ ND | Fold-Change DR+/+ HCD vs DR+/+ ND | RP-FDR 4DR30 HCD vs 4DR30 Reg | p value DR+/+ HCD vs DR+/+ ND | DR+/+ B/S vs DR+/+ ND n=1,330 1=yes 0=no | Log2 ratio DR+/+ B/S vs DR+/+ ND | Fold-Change DR+/+ B/S vs DR+/+ ND | RP-FDR 4DR30 Antib vs 4DR30 Reg | p value DR+/+ B/S vs DR+/+ ND | DR+/+ ND vs F+/+ ND n=398 1=yes 0=no | Log2 ratio DR+/+ ND vs F+/+ ND | Fold-Change DR+/+ ND vs F+/+ ND | RP-FDR 4RT1uu 30Reg vs 4DR30 Reg | p value DR+/+ ND vs F+/+ ND | DR+/+ Longitudinal n=228 1=yes 0=no | Figure 6F |
|---|---|---|---|---|---|---|---|---|---|---|---|---|---|---|---|---|---|---|---|---|---|---|---|---|---|
| 1374854_at | Pinx1 | PIN2/TERF1 interacting, telomerase inhibitor 1 | uniqAntib_1098 | 0.01 | -0.09 | -0.17 | -0.38 | -0.23 | 0 | -0.0798 | -1.0569 | 1.85963 | 0.1175 | 1 | -0.289 | -1.2218 | 2.40126 | 0.01042 | 0 | -0.1409 | -1.1026 | 2.0395 | 0.04892 | 0 | 0 |
| 1374873_at | --- | --- | uniqAntib_1098 | -0.03 | 0.12 | -0.05 | -0.15 | -0.03 | 0 | -0.1639 | -1.1203 | 2.32797 | 0.06565 | 1 | -0.2733 | -1.2085 | 1.7455 | 0.02531 | 0 | -0.1521 | -1.1112 | 1.76203 | 0.08114 | 0 | 0 |
| 1374901_at | --- | --- | uniqAntib_1098 | 0.03 | 0.08 | -0.07 | -0.23 | 0.13 | 0 | -0.1536 | -1.1123 | 2.05749 | 0.08766 | 1 | -0.3129 | -1.2422 | 1.91202 | 0.01918 | 0 | 0.04592 | 1.03234 | 1.10545 | 0.33118 | 0 | 0 |
| 1374906_at | Rnf113a1 | ring finger protein 113A1 | uniqAntib_1098 | -0.07 | -0.29 | -0.33 | 0.04 | -0.21 | 0 | -0.0448 | -1.0316 | 1.40782 | 0.20108 | 1 | 0.33342 | 1.26 | 0.99338 | 0.01418 | 0 | 0.08024 | 1.05719 | 1.67213 | 0.10791 | 0 | 0 |
| 1374929_at | Ccdc126 /// L | coiled-coil domain containing 126 /// coiled-coil dor | uniqAntib_1098 | -0.17 | -0.17 | -0.27 | 0.16 | -0.20 | 0 | -0.098 | -1.0703 | 1.10928 | 0.33265 | 1 | 0.33344 | 1.26001 | 1.18094 | 0.02472 | 0 | -0.0318 | -1.0223 | 0.9639 | 0.48953 | 0 | 0 |
| 1374963_s_at | --- | --- | uniqAntib_1098 | 0.13 | 0.37 | 0.23 | 0.81 | 0.24 | 0 | -0.139 | -1.1011 | 1.77527 | 0.1265 | 1 | 0.43781 | 1.35455 | 0.71664 | 0.00548 | 0 | -0.1318 | -1.0957 | 1.31932 | 0.18433 | 0 | 0 |
| 1374966_at | Dcx | doublecortin | uniqAntib_1098 | 0.17 | 0.11 | 0.25 | -0.16 | 0.04 | 0 | 0.14264 | 1.10392 | 1.50678 | 0.15509 | 1 | -0.2677 | -1.2039 | 1.77957 | 0.02415 | 0 | -0.0652 | -1.0462 | 1.03307 | 0.38049 | 0 | 0 |
| 1374969_at | --- | --- | uniqAntib_1098 | 0.11 | 0.08 | 0.04 | -0.19 | 0.00 | 0 | -0.0411 | -1.0289 | 0.9552 | 0.46766 | 1 | -0.275 | -1.21 | 1.84222 | 0.02026 | 0 | -0.0818 | -1.0584 | 1.15669 | 0.27337 | 0 | 0 |
| 1374971_at | --- | --- | uniqAntib_1098 | 0.17 | -0.16 | -0.04 | 0.14 | -0.04 | 0 | 0.11181 | 1.08058 | 1.08267 | 0.36342 | 1 | 0.29831 | 1.22971 | 1.23153 | 0.03307 | 0 | 0.11516 | 1.0831 | 1.1092 | 0.32796 | 0 | 0 |
| 1374977_at | --- | --- | uniqAntib_1098 | -0.10 | -0.19 | -0.21 | -0.52 | -0.31 | 0 | -0.0194 | -1.0135 | 1.07296 | 0.35926 | 1 | -0.3254 | -1.253 | 1.82634 | 0.0212 | 0 | -0.1154 | -1.0833 | 1.33347 | 0.18 | 0 | 0 |
| 1375003_at | Serpinb6 | serpin peptidase inhibitor, clade B (ovalbumin), mer | uniqAntib_1098 | -0.09 | -1.25 | -1.32 | -0.96 | -1.00 | 0 | -0.0688 | -1.0489 | 1.82986 | 0.11962 | 1 | 0.28639 | 1.21959 | 0.96931 | 0.0135 | 0 | 0.2422 | 1.1828 | 2.01429 | 0.00725 | 0 | 0 |
| 1375036_at | LOC10091162 | uncharacterized LOC100911627 | uniqAntib_1098 | -0.05 | -0.13 | -0.15 | 0.24 | -0.13 | 0 | -0.0209 | -1.0146 | 1.04507 | 0.40299 | 1 | 0.37473 | 1.2966 | 0.93715 | 0.01058 | 0 | 0.00462 | 1.00321 | 0.962 | 0.47399 | 0 | 0 |
| 1375056_at | Ociad2 | OCIA domain containing 2 | uniqAntib_1098 | -0.05 | -0.05 | 0.03 | 0.40 | -0.02 | 0 | 0.07402 | 1.05265 | 1.2759 | 0.24181 | 1 | 0.44603 | 1.36229 | 0.57097 | 0.00321 | 0 | 0.0238 | 1.01663 | 1.3039 | 0.22004 | 0 | 0 |
| 1375127_at | Cox5a | cytochrome c oxidase, subunit Va | uniqAntib_1098 | 0.00 | -0.35 | -0.17 | 0.07 | -0.17 | 0 | 0.17303 | 1.12743 | 2.00653 | 0.04168 | 1 | 0.41296 | 1.33141 | 0.60016 | 0.00357 | 0 | 0.17404 | 1.12821 | 2.16935 | 0.04855 | 0 | 0 |
| 1375170_at | S100a11 | S100 calcium binding protein A11 | uniqAntib_1098 | 0.04 | -0.19 | -0.22 | 0.23 | 0.02 | 0 | -0.0362 | -1.0254 | 0.91841 | 0.52626 | 1 | 0.41343 | 1.33185 | 0.68352 | 0.00475 | 0 | 0.2066 | 1.15396 | 1.68203 | 0.10633 | 0 | 0 |
| 1375200_at | Irf2bpl | interferon regulatory factor 2 binding protein-like | uniqAntib_1098 | -0.04 | 0.05 | -0.11 | -0.25 | -0.01 | 0 | -0.169 | -1.1243 | 2.0892 | 0.08256 | 1 | -0.3027 | -1.2334 | 1.55562 | 0.03732 | 0 | -0.0646 | -1.0458 | 0.99778 | 0.43487 | 0 | 0 |
| 1375221_at | Tmx4 | thioredoxin-related transmembrane protein 4 | uniqAntib_1098 | -0.10 | -0.28 | -0.26 | 0.07 | -0.27 | 0 | 0.01714 | 1.01195 | 1.10063 | 0.34917 | 1 | 0.3528 | 1.27704 | 0.97099 | 0.01321 | 0 | 0.00501 | 1.00348 | 0.99439 | 0.43975 | 0 | 0 |
| 1375253_at | Nfe2l1 | nuclear factor, erythroid derived 2,-like 1 | uniqAntib_1098 | -0.04 | 0.12 | -0.06 | -0.24 | -0.07 | 0 | -0.1791 | -1.1322 | 1.93648 | 0.10467 | 1 | -0.3665 | -1.2892 | 2.26677 | 0.01174 | 0 | -0.1946 | -1.1444 | 1.5405 | 0.11339 | 0 | 0 |
| 1375288_at | Gcn1l1 | GCN1 general control of amino-acid synthesis 1-like | uniqAntib_1098 | 0.11 | 0.22 | 0.11 | -0.05 | 0.03 | 0 | -0.1089 | -1.0784 | 1.74486 | 0.13011 | 1 | -0.2653 | -1.2019 | 1.5581 | 0.03748 | 0 | -0.1858 | -1.1375 | 1.7674 | 0.08059 | 0 | 0 |
| 1375290_at | Rbm24 | RNA binding motif protein 24 | uniqAntib_1098 | 0.00 | -0.01 | -0.21 | -0.29 | 0.01 | 0 | -0.1953 | -1.1449 | 2.8821 | 0.03355 | 1 | -0.2706 | -1.2063 | 1.57721 | 0.03454 | 0 | 0.02188 | 1.01528 | 1.22632 | 0.25781 | 0 | 0 |
| 1375309_at | --- | --- | uniqAntib_1098 | -0.24 | 0.01 | 0.08 | 0.29 | -0.04 | 0 | 0.06706 | 1.04758 | 1.1678 | 0.3027 | 1 | 0.27309 | 1.20839 | 1.26603 | 0.04604 | 0 | -0.0585 | -1.0414 | 1.04931 | 0.3643 | 0 | 0 |
| 1375316_at | --- | --- | uniqAntib_1098 | 0.00 | -0.02 | -0.06 | -0.34 | -0.09 | 0 | -0.0354 | -1.0249 | 1.4318 | 0.19208 | 1 | -0.3132 | -1.2425 | 2.07977 | 0.01451 | 0 | -0.0693 | -1.0492 | 1.19511 | 0.24833 | 0 | 0 |
| 1375343_at | --- | --- | uniqAntib_1098 | -0.04 | -0.29 | -0.26 | 0.17 | -0.18 | 0 | 0.03512 | 1.02464 | 0.93903 | 0.52587 | 1 | 0.45958 | 1.37514 | 0.62296 | 0.00413 | 0 | 0.11052 | 1.07962 | 1.24818 | 0.24752 | 0 | 0 |
| 1375361_at | Snx18 | sorting nexin 18 | uniqAntib_1098 | -0.04 | 0.11 | -0.08 | -0.30 | -0.14 | 0 | -0.1876 | -1.1388 | 2.24702 | 0.07095 | 1 | -0.4022 | -1.3216 | 3.70941 | 0.00406 | 0 | -0.2406 | -1.1815 | 2.93989 | 0.01796 | 0 | 0 |
| 1375382_at | Mdc1 | mediator of DNA damage checkpoint 1 | uniqAntib_1098 | -0.07 | 0.34 | 0.35 | 0.00 | 0.41 | 0 | 0.01378 | 1.00959 | 1.11729 | 0.33847 | 1 | -0.3393 | -1.2651 | 2.19659 | 0.01222 | 0 | 0.07343 | 1.05221 | 1.28958 | 0.22745 | 0 | 0 |
| 1375431_at | RGD1306941 | similar to CG31122-PA | uniqAntib_1098 | -0.15 | -0.31 | -0.30 | -0.03 | -0.19 | 0 | 0.01339 | 1.00932 | 1.68791 | 0.10649 | 1 | 0.2746 | 1.20966 | 1.25736 | 0.03671 | 0 | 0.11547 | 1.08332 | 1.511 | 0.14766 | 0 | 0 |
| 1375449_at | --- | --- | uniqAntib_1098 | -0.12 | 0.01 | 0.16 | 0.38 | 0.00 | 0 | 0.15828 | 1.11596 | 1.75935 | 0.08039 | 1 | 0.3743 | 1.29621 | 0.95102 | 0.0111 | 0 | -0.0009 | -1.0006 | 0.95449 | 0.50626 | 0 | 0 |
| 1375465_at | LOC10091149 | homeobox protein OTX2-like /// orthodenticle hom | uniqAntib_1098 | -0.01 | -0.29 | -0.10 | 0.06 | -0.17 | 0 | 0.19448 | 1.14431 | 1.75876 | 0.08636 | 1 | 0.34734 | 1.27221 | 0.79032 | 0.00709 | 0 | 0.1177 | 1.085 | 1.21587 | 0.26316 | 0 | 0 |
| 1375477_at | Fem1b | fem-1 homolog b (C. elegans) | uniqAntib_1098 | -0.03 | -0.01 | -0.02 | -0.33 | -0.19 | 0 | -0.0167 | -1.0117 | 1.21216 | 0.2751 | 1 | -0.3233 | -1.2512 | 1.97479 | 0.01791 | 0 | -0.1793 | -1.1323 | 1.97189 | 0.05428 | 0 | 0 |
| 1375535_at | LOC10091149 | uncharacterized LOC100911498 | uniqAntib_1098 | -0.07 | -0.12 | -0.24 | -0.42 | -0.29 | 0 | -0.1187 | -1.0857 | 1.63812 | 0.14833 | 1 | -0.3005 | -1.2316 | 1.80339 | 0.02157 | 0 | -0.1647 | -1.1209 | 1.86696 | 0.06688 | 0 | 0 |
| 1375589_at | Tmem8a | transmembrane protein 8A | uniqAntib_1098 | -0.06 | -0.04 | -0.17 | -0.35 | -0.07 | 0 | -0.1304 | -1.0946 | 1.51088 | 0.17461 | 1 | -0.3106 | -1.2402 | 1.79383 | 0.02348 | 0 | -0.0324 | -1.0227 | 0.9734 | 0.47504 | 0 | 0 |
| 1375590_at | Zc3h11a | zinc finger CCCH-type containing 11A | uniqAntib_1098 | 0.09 | -0.15 | 0.08 | 0.19 | -0.01 | 0 | 0.22865 | 1.17174 | 1.9996 | 0.04231 | 1 | 0.34192 | 1.26744 | 1.00847 | 0.01482 | 0 | 0.14449 | 1.10534 | 1.43641 | 0.17334 | 0 | 0 |
| 1375616_at | Apba2 | amyloid beta (A4) precursor protein-binding, family | uniqAntib_1098 | 0.02 | -0.02 | -0.10 | -0.30 | -0.08 | 0 | -0.0801 | -1.0571 | 1.16014 | 0.30362 | 1 | -0.2817 | -1.2156 | 1.64926 | 0.03134 | 0 | -0.0581 | -1.0411 | 1.15017 | 0.27664 | 0 | 0 |
| 1375629_at | Lamp1 | lysosomal-associated membrane protein 1 | uniqAntib_1098 | 0.12 | 0.20 | 0.10 | -0.09 | 0.13 | 0 | -0.1051 | -1.0756 | 1.32648 | 0.22862 | 1 | -0.2878 | -1.2208 | 1.59853 | 0.03213 | 0 | -0.0709 | -1.0503 | 1.14814 | 0.279 | 0 | 0 |
| 1375631_at | Plekhb2 | pleckstrin homology domain containing, family B (e | uniqAntib_1098 | 0.06 | -0.14 | -0.10 | 0.15 | 0.02 | 0 | 0.0329 | 1.02307 | 0.986 | 0.46312 | 1 | 0.28362 | 1.21725 | 1.2775 | 0.0449 | 0 | 0.15618 | 1.11433 | 1.50544 | 0.1489 | 0 | 0 |
| 1375652_at | Ssr3 | signal sequence receptor, gamma | uniqAntib_1098 | -0.11 | -0.33 | -0.15 | -0.02 | -0.24 | 0 | 0.17658 | 1.1302 | 1.90589 | 0.0592 | 1 | 0.30402 | 1.23458 | 1.2073 | 0.03001 | 0 | 0.08695 | 1.06212 | 1.13386 | 0.31082 | 0 | 0 |
| 1375672_at | --- | --- | uniqAntib_1098 | -0.02 | -0.18 | -0.03 | 0.13 | -0.06 | 0 | 0.14652 | 1.1069 | 1.36473 | 0.20669 | 1 | 0.30649 | 1.2367 | 1.21644 | 0.03063 | 0 | 0.11913 | 1.08608 | 1.47675 | 0.15756 | 0 | 0 |
| 1375676_at | Lin7c | lin-7 homolog C (C. elegans) | uniqAntib_1098 | -0.42 | -0.73 | -0.76 | -0.11 | -0.80 | 0 | -0.0359 | -1.0252 | 3.09152 | 0.02803 | 1 | 0.61143 | 1.52777 | 0.04833 | 8.4E-05 | 0 | -0.072 | -1.0512 | 2.57379 | 0.02797 | 0 | 0 |
| 1375721_at | --- | --- | uniqAntib_1098 | -0.05 | 0.38 | 0.43 | 0.64 | 0.47 | 0 | 0.04959 | 1.03497 | 1.86415 | 0.06474 | 1 | 0.26646 | 1.20285 | 0.92222 | 0.00949 | 0 | 0.09349 | 1.06695 | 2.2837 | 0.04186 | 0 | 0 |
| 1375724_at | Glcci1 | glucocorticoid induced transcript 1 | uniqAntib_1098 | -0.20 | 0.01 | 0.27 | 0.35 | 0.09 | 0 | 0.25644 | 1.19453 | 2.22954 | 0.02036 | 1 | 0.33705 | 1.26317 | 0.96304 | 0.01232 | 0 | 0.0803 | 1.05724 | 1.48614 | 0.15397 | 0 | 0 |
| 1375755_at | B4galt1 | UDP-Gal:betaGlcNAc beta 1,4- galactosyltransferase | uniqAntib_1098 | 0.04 | 0.09 | 0.03 | -0.29 | 0.03 | 0 | -0.0608 | -1.043 | 1.25781 | 0.25469 | 1 | -0.382 | -1.3031 | 2.95746 | 0.00561 | 0 | -0.0573 | -1.0405 | 1.25244 | 0.21796 | 0 | 0 |
| 1375787_at | RGD1304878 | similar to 2410024A21Rik protein | uniqAntib_1098 | 0.11 | 0.11 | -0.06 | -0.23 | 0.02 | 0 | -0.1701 | -1.1251 | 1.9582 | 0.10207 | 1 | -0.3384 | -1.2643 | 1.84153 | 0.02007 | 0 | -0.0949 | -1.068 | 1.10891 | 0.30879 | 0 | 0 |
| 1375788_at | Rpl7 | ribosomal protein L7 | uniqAntib_1098 | -0.04 | -0.35 | -0.17 | -0.07 | -0.30 | 0 | 0.185 | 1.13682 | 2.13603 | 0.02425 | 1 | 0.27984 | 1.21406 | 1.20141 | 0.02766 | 0 | 0.04812 | 1.03392 | 1.51813 | 0.14679 | 0 | 0 |
| 1375884_at | Msantd4 | Myb/SANT-like DNA-binding domain containing 4 w | uniqAntib_1098 | -0.17 | -0.39 | -0.37 | -0.02 | -0.24 | 0 | 0.01823 | 1.01271 | 1.25322 | 0.25396 | 1 | 0.37396 | 1.29591 | 0.9317 | 0.01007 | 0 | 0.15351 | 1.11227 | 1.57032 | 0.13214 | 0 | 0 |
| 1375894_at | Lztfl1 | leucine zipper transcription factor-like 1 | uniqAntib_1098 | -0.03 | -0.26 | -0.36 | 0.04 | -0.24 | 0 | -0.1057 | -1.076 | 1.35259 | 0.21968 | 1 | 0.30106 | 1.23205 | 1.24825 | 0.0346 | 0 | 0.01977 | 1.0138 | 0.98637 | 0.44461 | 0 | 0 |
| 1375906_at | LOC10035983 | rCG38975-like | uniqAntib_1098 | -0.08 | -0.46 | -0.47 | -0.17 | -0.46 | 0 | -0.0129 | -1.009 | 0.8982 | 0.56669 | 1 | 0.29357 | 1.22567 | 1.19901 | 0.02768 | 0 | -8E-05 | -1.0001 | 0.94851 | 0.51429 | 0 | 0 |
| 1375951_at | Thbd | thrombomodulin | uniqAntib_1098 | 0.19 | -0.11 | -0.05 | 0.45 | 0.13 | 0 | 0.06555 | 1.04648 | 1.00218 | 0.44323 | 1 | 0.55903 | 1.47328 | 0.14065 | 0.00042 | 0 | 0.23795 | 1.17932 | 2.06443 | 0.05722 | 0 | 0 |
| 1376010_at | Prpf4b | PRP4 pre-mRNA processing factor 4 homolog B (yea | uniqAntib_1098 | -0.14 | -0.18 | -0.15 | 0.12 | -0.13 | 0 | 0.02619 | 1.01832 | 1.03686 | 0.41022 | 1 | 0.29477 | 1.22669 | 1.25662 | 0.03612 | 0 | 0.05006 | 1.0353 | 1.06731 | 0.36513 | 0 | 0 |
| 1376076_at | --- | --- | uniqAntib_1098 | 0.00 | -0.33 | -0.37 | 0.01 | -0.36 | 0 | -0.0413 | -1.029 | 1.67 | 0.14096 | 1 | 0.34025 | 1.26598 | 0.83653 | 0.00807 | 0 | -0.033 | -1.0231 | 1.2601 | 0.2143 | 0 | 0 |
| 1376111_at | --- | --- | uniqAntib_1098 | 0.08 | 0.23 | 0.01 | -0.08 | 0.03 | 0 | -0.2198 | -1.1646 | 2.98074 | 0.02981 | 1 | -0.3121 | -1.2415 | 3.04382 | 0.00538 | 0 | -0.2034 | -1.1514 | 2.35667 | 0.03418 | 0 | 0 |
| 1376121_at | --- | --- | uniqAntib_1098 | -0.11 | 0.30 | 0.28 | 0.57 | 0.31 | 0 | -0.0261 | -1.0182 | 1.05428 | 0.37172 | 1 | 0.26399 | 1.20079 | 1.26824 | 0.03939 | 0 | 0.01134 | 1.00789 | 1.06743 | 0.3651 | 0 | 0 |
| 1376124_at | Iqub | IQ motif and ubiquitin domain containing | uniqAntib_1098 | -0.14 | -0.08 | -0.01 | 0.21 | 0.02 | 0 | 0.06692 | 1.04748 | 1.05176 | 0.39156 | 1 | 0.28461 | 1.21808 | 1.27208 | 0.04577 | 0 | 0.09539 | 1.06835 | 1.18881 | 0.27577 | 0 | 0 |
| 1376152_at | Mical2 | microtubule associated monooxygenase, calponin a | uniqAntib_1098 | 0.11 | 0.21 | 0.08 | -0.18 | 0.04 | 0 | -0.1318 | -1.0957 | 1.53172 | 0.16899 | 1 | -0.384 | -1.305 | 2.81382 | 0.00615 | 0 | -0.1661 | -1.1221 | 1.69789 | 0.09309 | 0 | 0 |
| 1376158_at | --- | --- | uniqAntib_1098 | 0.08 | 0.14 | 0.04 | -0.18 | -0.08 | 0 | -0.1027 | -1.0738 | 1.17376 | 0.29726 | 1 | -0.3252 | -1.2529 | 1.7977 | 0.02162 | 0 | -0.2206 | -1.1652 | 1.8068 | 0.07553 | 0 | 0 |
| 1376237_at | --- | --- | uniqAntib_1098 | -0.12 | -0.29 | -0.18 | 0.00 | -0.14 | 0 | 0.10667 | 1.07674 | 1.38476 | 0.19975 | 1 | 0.29171 | 1.22409 | 1.21157 | 0.03086 | 0 | 0.15031 | 1.10981 | 1.53448 | 0.14393 | 0 | 0 |
| 1376428_at | --- | --- | uniqAntib_1098 | 0.19 | 0.08 | 0.23 | -0.21 | 0.02 | 0 | 0.14961 | 1.10927 | 1.6157 | 0.12947 | 1 | -0.2903 | -1.2229 | 1.7939 | 0.02336 | 0 | -0.0582 | -1.0411 | 1.10213 | 0.31442 | 0 | 0 |
| 1376448_at | --- | --- | uniqAntib_1098 | 0.00 | 0.12 | 0.15 | 0.48 | 0.26 | 0 | 0.02109 | 1.01473 | 1.33664 | 0.21675 | 1 | 0.35211 | 1.27643 | 0.95829 | 0.01152 | 0 | 0.13877 | 1.10096 | 1.81084 | 0.08664 | 0 | 0 |
| 1376472_at | --- | --- | uniqAntib_1098 | -0.03 | 0.04 | 0.07 | 0.35 | 0.13 | 0 | 0.02091 | 1.0146 | 1.18014 | 0.29527 | 1 | 0.30597 | 1.23625 | 1.01898 | 0.01579 | 0 | 0.08751 | 1.06254 | 1.45536 | 0.1651 | 0 | 0 |
| 1376507_a_at | Lemd2 | LEM domain containing 2 | uniqAntib_1098 | -0.06 | -0.11 | -0.10 | -0.44 | -0.17 | 0 | 0.01122 | 1.00781 | 1.00468 | 0.44114 | 1 | -0.3292 | -1.2563 | 2.89097 | 0.00576 | 0 | -0.0594 | -1.042 | 1.22428 | 0.23148 | 0 | 0 |
| 1376560_at | --- | --- | uniqAntib_1098 | 0.04 | 0.02 | 0.18 | 0.34 | 0.17 | 0 | 0.1654 | 1.12147 | 1.8353 | 0.06917 | 1 | 0.32134 | 1.24949 | 0.9739 | 0.01372 | 0 | 0.15485 | 1.1133 | 1.89141 | 0.07231 | 0 | 0 |
| 1376562_at | Tnik | TRAF2 and NCK interacting kinase | uniqAntib_1098 | 0.07 | 0.19 | 0.22 | 0.51 | 0.32 | 0 | 0.02912 | 1.02039 | 1.17005 | 0.30204 | 1 | 0.31926 | 1.24769 | 1.1412 | 0.02202 | 0 | 0.12303 | 1.08902 | 1.39598 | 0.18422 | 0 | 0 |
| 1376575_at | Twsg1 | twisted gastrulation homolog 1 (Drosophila) | uniqAntib_1098 | -0.12 | -0.16 | -0.27 | 0.14 | -0.11 | 0 | -0.1113 | -1.0802 | 1.14253 | 0.31364 | 1 | 0.29811 | 1.22953 | 1.25241 | 0.0356 | 0 | 0.04892 | 1.03449 | 1.19592 | 0.27272 | 0 | 0 |
| 1376604_at | Synm | synemin, intermediate filament protein | uniqAntib_1098 | 0.02 | 0.09 | -0.13 | -0.24 | -0.16 | 0 | -0.2228 | -1.167 | 3.16584 | 0.02229 | 1 | -0.3303 | -1.2573 | 2.1718 | 0.01243 | 0 | -0.2463 | -1.1861 | 2.89964 | 0.01837 | 0 | 0 |
| 1376669_at | --- | --- | uniqAntib_1098 | 0.15 | -0.32 | -0.12 | 0.26 | -0.27 | 0 | 0.20165 | 1.15001 | 2.27136 | 0.01994 | 1 | 0.57391 | 1.48855 | 0.11711 | 0.00029 | 0 | 0.05215 | 1.03681 | 1.34098 | 0.2043 | 0 | 0 |
| 1376675_at | Cd300a | Cd300a molecule | uniqAntib_1098 | 0.09 | -0.32 | -0.19 | -0.02 | -0.25 | 0 | 0.12996 | 1.09427 | 1.73949 | 0.0919 | 1 | 0.29906 | 1.23034 | 0.96436 | 0.01188 | 0 | 0.07128 | 1.05065 | 1.46577 | 0.16133 | 0 | 0 |
| 1376689_at | Ikbip | IKBKB interacting protein | uniqAntib_1098 | -0.14 | -0.26 | -0.23 | 0.01 | -0.09 | 0 | 0.03331 | 1.02336 | 1.71623 | 0.09746 | 1 | 0.26927 | 1.2052 | 1.27099 | 0.04075 | 0 | 0.17569 | 1.12951 | 1.88917 | 0.07684 | 0 | 0 |
| 1376693_at | --- | --- | uniqAntib_1098 | -0.04 | -0.21 | -0.20 | 0.07 | -0.17 | 0 | 0.00912 | 1.00634 | 1.19718 | 0.28333 | 1 | 0.27713 | 1.21178 | 1.27228 | 0.04553 | 0 | 0.04039 | 1.02839 | 1.04944 | 0.3766 | 0 | 0 |
| 1376700_at | Lima1 | LIM domain and actin binding 1 | uniqAntib_1098 | 0.00 | 0.01 | 0.00 | -0.28 | -0.17 | 0 | -0.0125 | -1.0087 | 1.1012 | 0.33901 | 1 | -0.2919 | -1.2242 | 2.02678 | 0.01701 | 0 | -0.1812 | -1.1339 | 1.83756 | 0.07368 | 0 | 0 |
| 1376709_at | Slc39a8 | solute carrier family 39 (zinc transporter), member 8 | uniqAntib_1098 | -0.06 | -0.08 | -0.16 | 0.26 | 0.09 | 0 | -0.0819 | -1.0584 | 1.21739 | 0.2714 | 1 | 0.33896 | 1.26485 | 1.01951 | 0.0159 | 0 | 0.16519 | 1.12131 | 1.69537 | 0.10396 | 0 | 0 |
| 1376728_at | --- | --- | uniqAntib_1098 | -0.03 | -0.31 | -0.42 | 0.04 | -0.15 | 0 | -0.1109 | -1.0799 | 1.72714 | 0.1329 | 1 | 0.35327 | 1.27745 | 0.83743 | 0.00816 | 0 | 0.16475 | 1.12097 | 2.15739 | 0.04946 | 0 | 0 |
| 1376770_at | Efhd1 | EF-hand domain family, member D1 | uniqAntib_1098 | 0.04 | 0.01 | 0.00 | -0.27 | -0.07 | 0 | -0.0134 | -1.0093 | 1.64292 | 0.14744 | 1 | -0.2779 | -1.2124 | 1.82992 | 0.02101 | 0 | -0.0795 | -1.0567 | 1.31825 | 0.18541 | 0 | 0 |

| Affymetrix Probeset ID | Gene Symbol | Gene Title | Figure 6 B Venn n =2021 | Mean BN log2 intensity autologous n=8 | Mean log2 intensity day 30 DR +/+ ND n=4 | Mean log2 intensity day 30 DR+/+ HCD n=4 | Mean log2 intensity day 30 B/S n=4 | Mean log2 intensity day 30 F+/+ ND n=4 | DR+/+ HCD vs DR+/+ ND n=380 1=yes 0=no | Log2 ratio DR+/+ HCD vs DR+/+ ND | Fold-Change DR+/+ HCD vs DR+/+ ND | RP-FDR 4DR30 HCD vs 4DR30 Reg | p value DR+/+ HCD vs DR+/+ ND | DR+/+ B/S vs DR+/+ ND n=1,330 1=yes 0=no | Log2 ratio DR+/+ B/S vs DR+/+ ND | Fold-Change DR+/+ B/S vs DR+/+ ND | RP-FDR 4DR30 Antib vs 4DR30 Reg | p value DR+/+ B/S vs DR+/+ ND | DR+/+ ND vs F+/+ ND n=398 1=yes 0=no | Log2 ratio DR+/+ ND vs F+/+ ND | Fold-Change DR+/+ ND vs F+/+ ND | RP-FDR 4RT1uu 30Reg vs 4DR30 Reg | p value DR+/+ ND vs F+/+ ND | DR+/+ Longitudinal n=228 1=yes 0=no | Figure 6F |
|---|---|---|---|---|---|---|---|---|---|---|---|---|---|---|---|---|---|---|---|---|---|---|---|---|---|
| 1376776_at | Ccdc17 | coiled-coil domain containing 17 | uniqAntib_1098 | -0.11 | -0.03 | -0.11 | -0.36 | -0.25 | 0 | -0.081 | -1.0577 | 1.14906 | 0.30915 | 1 | -0.3274 | -1.2547 | 1.80566 | 0.02154 | 0 | -0.2182 | -1.1632 | 1.8996 | 0.06279 | 0 | 0 |
| 1376823_at | Fndc3a | fibronectin type III domain containing 3a | uniqAntib_1098 | 0.08 | 0.16 | 0.15 | -0.13 | 0.04 | 0 | -0.0126 | -1.0088 | 0.88798 | 0.59179 | 1 | -0.2882 | -1.2211 | 1.4452 | 0.04601 | 0 | -0.1138 | -1.0821 | 1.35167 | 0.17468 | 0 | 0 |
| 1376842_at | --- | --- | uniqAntib_1098 | -0.06 | -0.18 | -0.33 | 0.21 | -0.25 | 0 | -0.145 | -1.1057 | 1.38639 | 0.20828 | 1 | 0.39205 | 1.31226 | 0.93846 | 0.01056 | 0 | -0.0715 | -1.0508 | 1.40454 | 0.1578 | 0 | 0 |
| 1376851_at | Ubr2 | ubiquitin protein ligase E3 component n-recognin 2 | uniqAntib_1098 | -0.03 | 0.05 | -0.14 | -0.27 | -0.13 | 0 | -0.1967 | -1.146 | 1.73603 | 0.13191 | 1 | -0.3264 | -1.2539 | 1.72626 | 0.02653 | 0 | -0.1816 | -1.1342 | 1.54239 | 0.11313 | 0 | 0 |
| 1376877_at | Cdcp1 | CUB domain containing protein 1 | uniqAntib_1098 | -0.01 | 0.20 | 0.21 | -0.16 | 0.10 | 0 | 0.01028 | 1.00715 | 0.89336 | 0.57551 | 1 | -0.3558 | -1.2797 | 1.91799 | 0.01906 | 0 | -0.0987 | -1.0708 | 1.08044 | 0.33185 | 0 | 0 |
| 1376928_at | --- | --- | uniqAntib_1098 | -0.09 | -0.08 | -0.10 | -0.40 | -0.14 | 0 | -0.0197 | -1.0138 | 1.08806 | 0.34808 | 1 | -0.3219 | -1.25 | 2.36894 | 0.01074 | 0 | -0.0661 | -1.0468 | 1.00685 | 0.41968 | 0 | 0 |
| 1376931_at | Phf20 | PHD finger protein 20 | uniqAntib_1098 | -0.02 | 0.12 | 0.25 | 0.39 | 0.27 | 0 | 0.13349 | 1.09694 | 1.70863 | 0.10175 | 1 | 0.26768 | 1.20387 | 1.2037 | 0.02717 | 0 | 0.15263 | 1.1116 | 1.82154 | 0.08399 | 0 | 0 |
| 1376959_at | Sprr3 | small proline-rich protein 3 | uniqAntib_1098 | -0.01 | -0.01 | -0.04 | -0.27 | -0.03 | 0 | -0.0332 | -1.0232 | 1.3752 | 0.21235 | 1 | -0.2672 | -1.2035 | 1.65761 | 0.02964 | 0 | -0.02 | -1.014 | 1.06893 | 0.34111 | 0 | 0 |
| 1376983_at | LOC680200 | similar to zinc finger protein 455 | uniqAntib_1098 | -0.10 | -0.41 | -0.49 | -0.06 | -0.23 | 0 | -0.0746 | -1.053 | 1.28969 | 0.24302 | 1 | 0.35456 | 1.27859 | 1.00692 | 0.01483 | 0 | 0.1839 | 1.13595 | 1.88955 | 0.0765 | 0 | 0 |
| 1377011_at | Fry | furry homolog (Drosophila) | uniqAntib_1098 | -0.05 | 0.00 | -0.12 | 0.31 | 0.06 | 0 | -0.1201 | -1.0868 | 1.33817 | 0.2241 | 1 | 0.31393 | 1.24309 | 1.20372 | 0.0281 | 0 | 0.0634 | 1.04493 | 1.12712 | 0.31444 | 0 | 0 |
| 1377055_at | LOC10036281 | hypothetical protein LOC100362814 | uniqAntib_1098 | 0.19 | -0.03 | 0.11 | -0.31 | -0.05 | 0 | 0.14557 | 1.10617 | 1.6911 | 0.1056 | 1 | -0.2727 | -1.2081 | 2.00025 | 0.01531 | 0 | -0.017 | -1.0119 | 1.51676 | 0.12013 | 0 | 0 |
| 1377063_at | --- | --- | uniqAntib_1098 | 0.01 | 0.35 | 0.24 | 0.07 | 0.39 | 0 | -0.1037 | -1.0746 | 1.28135 | 0.24594 | 1 | -0.2763 | -1.2111 | 1.50571 | 0.04207 | 0 | 0.04541 | 1.03198 | 0.96038 | 0.47681 | 0 | 0 |
| 1377105_at | Zfp91 | zinc finger protein 91 | uniqAntib_1098 | -0.04 | 0.03 | 0.23 | 0.30 | 0.03 | 0 | 0.19588 | 1.14542 | 2.093 | 0.0278 | 1 | 0.26605 | 1.20251 | 1.17845 | 0.0255 | 0 | -0.0034 | -1.0023 | 1.10159 | 0.31515 | 0 | 0 |
| 1377111_at | --- | --- | uniqAntib_1098 | -0.07 | -0.29 | -0.10 | 0.28 | -0.25 | 0 | 0.19108 | 1.14162 | 2.50452 | 0.00676 | 1 | 0.5696 | 1.48411 | 0.13044 | 0.00038 | 0 | 0.03437 | 1.02411 | 1.36703 | 0.19346 | 0 | 0 |
| 1377114_at | --- | --- | uniqAntib_1098 | -0.24 | -1.08 | -1.02 | -0.73 | -1.27 | 0 | 0.05694 | 1.04026 | 1.99267 | 0.04242 | 1 | 0.34583 | 1.27088 | 0.64336 | 0.00437 | 0 | -0.1934 | -1.1435 | 2.99217 | 0.00799 | 0 | 0 |
| 1377115_at | --- | --- | uniqAntib_1098 | 0.04 | 0.05 | 0.11 | -0.26 | -0.04 | 0 | 0.05947 | 1.04208 | 1.01948 | 0.42505 | 1 | -0.3135 | -1.2427 | 1.79142 | 0.02229 | 0 | -0.0925 | -1.0662 | 1.1203 | 0.29928 | 0 | 0 |
| 1377178_at | --- | --- | uniqAntib_1098 | -0.04 | 0.01 | -0.09 | -0.28 | 0.07 | 0 | -0.1038 | -1.0746 | 1.22494 | 0.26737 | 1 | -0.2888 | -1.2216 | 1.56569 | 0.03469 | 0 | 0.0633 | 1.04485 | 0.99436 | 0.43546 | 0 | 0 |
| 1377203_at | --- | --- | uniqAntib_1098 | 0.12 | 0.23 | 0.13 | -0.07 | 0.06 | 0 | -0.1012 | -1.0726 | 2.00194 | 0.0965 | 1 | -0.3014 | -1.2324 | 2.60387 | 0.00888 | 0 | -0.1686 | -1.124 | 2.14326 | 0.04039 | 0 | 0 |
| 1377255_at | --- | --- | uniqAntib_1098 | -0.03 | 0.04 | 0.03 | -0.28 | -0.11 | 0 | -0.0126 | -1.0088 | 0.94822 | 0.47626 | 1 | -0.3186 | -1.2472 | 1.65092 | 0.03015 | 0 | -0.1518 | -1.111 | 1.40297 | 0.15623 | 0 | 0 |
| 1377276_at | Cdk5r2 | cyclin-dependent kinase 5, regulatory subunit 2 (p3! | uniqAntib_1098 | -0.04 | -0.01 | -0.09 | -0.42 | -0.17 | 0 | -0.0787 | -1.0561 | 1.71613 | 0.13426 | 1 | -0.4085 | -1.3273 | 3.72 | 0.00191 | 0 | -0.161 | -1.1181 | 1.87582 | 0.06641 | 0 | 0 |
| 1377324_at | LOC689600 /// | hypothetical protein LOC689600 /// RGD1564599 | uniqAntib_1098 | 0.05 | 0.10 | 0.07 | -0.20 | -0.07 | 0 | -0.0228 | -1.0159 | 1.00717 | 0.41671 | 1 | -0.2954 | -1.2272 | 1.63015 | 0.0315 | 0 | -0.165 | -1.1212 | 1.4056 | 0.15742 | 0 | 0 |
| 1377328_at | Mitf | microphthalmia-associated transcription factor | uniqAntib_1098 | 0.04 | -0.01 | 0.17 | 0.27 | 0.17 | 0 | 0.18652 | 1.13802 | 1.70611 | 0.09902 | 1 | 0.27904 | 1.21339 | 1.23096 | 0.03293 | 0 | 0.18855 | 1.13962 | 1.72429 | 0.0983 | 0 | 0 |
| 1377337_at | --- | --- | uniqAntib_1098 | 0.01 | 0.24 | 0.15 | 0.54 | 0.15 | 0 | -0.094 | -1.0673 | 1.21867 | 0.27101 | 1 | 0.30103 | 1.23203 | 1.12606 | 0.02075 | 0 | -0.0901 | -1.0645 | 1.25941 | 0.21451 | 0 | 0 |
| 1377395_at | --- | --- | uniqAntib_1098 | 0.02 | 0.09 | 0.00 | -0.26 | -0.07 | 0 | -0.0895 | -1.064 | 1.47083 | 0.18176 | 1 | -0.3477 | -1.2725 | 1.98812 | 0.01566 | 0 | -0.1599 | -1.1172 | 1.48545 | 0.13193 | 0 | 0 |
| 1377542_at | --- | --- | uniqAntib_1098 | -0.14 | 0.22 | 0.48 | 0.65 | 0.44 | 0 | 0.25741 | 1.19533 | 1.95963 | 0.05066 | 1 | 0.42832 | 1.34566 | 0.70413 | 0.00505 | 0 | 0.21246 | 1.15866 | 1.8662 | 0.07963 | 0 | 0 |
| 1377632_at | Timp4 | tissue inhibitor of metalloproteinase 4 | uniqAntib_1098 | -0.12 | 0.00 | -0.16 | -0.27 | -0.04 | 0 | -0.1566 | -1.1147 | 1.79612 | 0.12221 | 1 | -0.2713 | -1.2069 | 1.48439 | 0.04305 | 0 | -0.0388 | -1.0273 | 1.04205 | 0.37043 | 0 | 0 |
| 1377650_at | --- | --- | uniqAntib_1098 | -0.10 | -0.42 | -0.25 | 0.31 | -0.18 | 0 | 0.1691 | 1.12436 | 1.52663 | 0.15124 | 1 | 0.72781 | 1.65612 | 0.01 | 1.3E-05 | 0 | 0.2388 | 1.18001 | 2.12278 | 0.05085 | 0 | 0 |
| 1377671_at | --- | --- | uniqAntib_1098 | 0.22 | 0.10 | 0.20 | 0.40 | 0.31 | 0 | 0.09133 | 1.06535 | 1.29151 | 0.2351 | 1 | 0.29991 | 1.23107 | 1.1991 | 0.02745 | 0 | 0.20934 | 1.15616 | 1.96054 | 0.06645 | 0 | 0 |
| 1377739_at | Gng12 | guanine nucleotide binding protein (G protein), gam | uniqAntib_1098 | -0.11 | -0.41 | -0.20 | 0.03 | -0.27 | 0 | 0.2092 | 1.15605 | 1.85157 | 0.06644 | 1 | 0.43885 | 1.35553 | 0.67254 | 0.00461 | 0 | 0.13731 | 1.09985 | 1.41 | 0.1814 | 0 | 0 |
| 1377763_at | --- | --- | uniqAntib_1098 | -0.11 | -0.18 | -0.08 | 0.12 | -0.11 | 0 | 0.1041 | 1.07482 | 1.24956 | 0.25478 | 1 | 0.30467 | 1.23513 | 1.2472 | 0.03421 | 0 | 0.07113 | 1.05054 | 1.16526 | 0.29245 | 0 | 0 |
| 1377768_at | Kctd1 | potassium channel tetramerisation domain containi | uniqAntib_1098 | 0.11 | 0.14 | -0.05 | -0.29 | -0.07 | 0 | -0.1976 | -1.1468 | 2.10831 | 0.08149 | 1 | -0.432 | -1.3491 | 3.56833 | 0.00207 | 0 | -0.2114 | -1.1578 | 1.89925 | 0.06229 | 0 | 0 |
| 1377780_at | Tspan9 | tetraspanin 9 | uniqAntib_1098 | 0.07 | 0.01 | -0.05 | -0.26 | -0.12 | 0 | -0.0585 | -1.0414 | 1.14933 | 0.309 | 1 | -0.2741 | -1.2093 | 1.68479 | 0.02768 | 0 | -0.1255 | -1.0909 | 1.43026 | 0.14749 | 0 | 0 |
| 1377813_at | Col22a1 | collagen, type XXII, alpha 1 | uniqAntib_1098 | -0.20 | -0.05 | -0.19 | -0.34 | -0.17 | 0 | -0.1344 | -1.0976 | 1.62822 | 0.14974 | 1 | -0.2836 | -1.2172 | 1.65717 | 0.02995 | 0 | -0.1163 | -1.0839 | 1.35156 | 0.1741 | 0 | 0 |
| 1377856_at | Zfyve27 | zinc finger, FYVE domain containing 27 | uniqAntib_1098 | 0.00 | 0.12 | 0.01 | -0.18 | 0.08 | 0 | -0.1132 | -1.0816 | 1.2433 | 0.2605 | 1 | -0.304 | -1.2345 | 1.89596 | 0.01933 | 0 | -0.0421 | -1.0296 | 1.02001 | 0.3986 | 0 | 0 |
| 1377885_at | LOC685203 | hypothetical protein LOC685203 | uniqAntib_1098 | -0.40 | -0.19 | -0.23 | -0.54 | -0.28 | 0 | -0.0431 | -1.0303 | 1.41256 | 0.19908 | 1 | -0.3551 | -1.2791 | 3.04429 | 0.00548 | 0 | -0.0932 | -1.0667 | 1.79116 | 0.07896 | 0 | 0 |
| 1377899_at | RGD1304982 | similar to RIKEN cDNA 2810025M15 | uniqAntib_1098 | -0.03 | -0.04 | -0.13 | -0.32 | -0.29 | 0 | -0.0857 | -1.0612 | 1.6146 | 0.15093 | 1 | -0.2832 | -1.2169 | 1.65274 | 0.02971 | 0 | -0.2486 | -1.1881 | 2.90306 | 0.0183 | 0 | 0 |
| 1377901_at | Fbxw8 | F-box and WD repeat domain containing 8 | uniqAntib_1098 | 0.04 | 0.04 | -0.01 | -0.31 | -0.16 | 0 | -0.0461 | -1.0325 | 1.00001 | 0.42445 | 1 | -0.3471 | -1.272 | 2.2708 | 0.01183 | 0 | -0.2009 | -1.1494 | 1.66699 | 0.09654 | 0 | 0 |
| 1377913_at | --- | --- | uniqAntib_1098 | -0.26 | -0.21 | -0.18 | 0.10 | -0.12 | 0 | 0.03335 | 1.02339 | 1.24325 | 0.25685 | 1 | 0.31214 | 1.24155 | 1.07479 | 0.01818 | 0 | 0.08952 | 1.06402 | 1.8898 | 0.07535 | 0 | 0 |
| 1377924_at | --- | --- | uniqAntib_1098 | -0.15 | -0.15 | -0.24 | 0.14 | -0.17 | 0 | -0.0866 | -1.0618 | 1.06649 | 0.36331 | 1 | 0.29071 | 1.22324 | 1.27341 | 0.04086 | 0 | -0.0146 | -1.0102 | 1.01988 | 0.39833 | 0 | 0 |
| 1377943_at | --- | --- | uniqAntib_1098 | 0.06 | -0.63 | -0.69 | -0.31 | -0.56 | 0 | -0.0599 | -1.0424 | 1.1796 | 0.29445 | 1 | 0.31954 | 1.24794 | 1.2003 | 0.02736 | 0 | 0.06647 | 1.04715 | 1.1757 | 0.28479 | 0 | 0 |
| 1377994_at | --- | --- | uniqAntib_1098 | 0.08 | 0.08 | 0.16 | 0.61 | 0.26 | 0 | 0.08406 | 1.06 | 1.19272 | 0.28684 | 1 | 0.52847 | 1.4424 | 0.18939 | 0.0006 | 0 | 0.18243 | 1.13479 | 1.66432 | 0.11169 | 0 | 0 |
| 1378056_at | Gmnn | geminin | uniqAntib_1098 | -0.11 | -0.26 | -0.39 | 0.08 | -0.20 | 0 | -0.1365 | -1.0992 | 2.01257 | 0.09209 | 1 | 0.33606 | 1.26231 | 1.01056 | 0.01505 | 0 | 0.05442 | 1.03844 | 1.1655 | 0.29191 | 0 | 0 |
| 1378059_at | --- | --- | uniqAntib_1098 | -0.09 | 0.05 | -0.03 | -0.23 | -0.12 | 0 | -0.0814 | -1.058 | 1.21823 | 0.27107 | 1 | -0.2784 | -1.2129 | 2.00222 | 0.01738 | 0 | -0.1691 | -1.1244 | 1.57621 | 0.10831 | 0 | 0 |
| 1378075_at | Tm9sf4 | transmembrane 9 superfamily protein member 4 | uniqAntib_1098 | 0.01 | -0.17 | -0.08 | 0.10 | 0.01 | 0 | 0.09262 | 1.0663 | 1.23143 | 0.26352 | 1 | 0.2749 | 1.20991 | 1.28081 | 0.04769 | 0 | 0.18879 | 1.13981 | 1.81622 | 0.08462 | 0 | 0 |
| 1378107_at | --- | --- | uniqAntib_1098 | 0.01 | -1.28 | -1.06 | -0.95 | -1.20 | 0 | 0.21784 | 1.16299 | 2.65444 | 0.00384 | 1 | 0.33385 | 1.26037 | 0.58917 | 0.00364 | 0 | 0.08027 | 1.05721 | 1.74533 | 0.09569 | 0 | 0 |
| 1378108_at | --- | --- | uniqAntib_1098 | 0.18 | 0.12 | 0.07 | -0.17 | -0.05 | 0 | -0.0462 | -1.0326 | 1.04709 | 0.37969 | 1 | -0.2817 | -1.2156 | 1.44072 | 0.04651 | 0 | -0.1648 | -1.121 | 1.40462 | 0.15442 | 0 | 0 |
| 1378136_at | Lrp3 | low density lipoprotein receptor-related protein 3 | uniqAntib_1098 | -0.03 | -0.08 | -0.14 | -0.42 | -0.11 | 0 | -0.0581 | -1.0411 | 1.41041 | 0.20005 | 1 | -0.3397 | -1.2655 | 2.53634 | 0.00758 | 0 | -0.0262 | -1.0183 | 1.31558 | 0.18651 | 0 | 0 |
| 1378141_at | --- | --- | uniqAntib_1098 | 0.04 | 0.20 | 0.12 | -0.13 | 0.12 | 0 | -0.0793 | -1.0565 | 1.07918 | 0.35284 | 1 | -0.3255 | -1.2531 | 1.64936 | 0.0305 | 0 | -0.077 | -1.0548 | 1.09553 | 0.32004 | 0 | 0 |
| 1378160_at | Frrs1 | ferric-chelate reductase 1 | uniqAntib_1098 | 0.00 | 0.02 | -0.03 | -0.27 | -0.01 | 0 | -0.0573 | -1.0405 | 1.10376 | 0.33625 | 1 | -0.2972 | -1.2288 | 1.73769 | 0.02492 | 0 | -0.0318 | -1.0223 | 0.96828 | 0.48347 | 0 | 0 |
| 1378175_at | --- | --- | uniqAntib_1098 | -0.14 | -0.19 | -0.15 | 0.17 | -0.17 | 0 | 0.03695 | 1.02595 | 1.21422 | 0.27311 | 1 | 0.36144 | 1.28471 | 1.00622 | 0.01472 | 0 | 0.02112 | 1.01475 | 0.95508 | 0.48419 | 0 | 0 |
| 1378194_a_at | Rufy3 | RUN and FYVE domain containing 3 | uniqAntib_1098 | -0.10 | 0.00 | 0.24 | 0.36 | 0.07 | 0 | 0.23555 | 1.17736 | 2.2096 | 0.02139 | 1 | 0.35835 | 1.28196 | 1.00144 | 0.01449 | 0 | 0.07191 | 1.05111 | 1.07943 | 0.35098 | 0 | 0 |
| 1378241_at | --- | --- | uniqAntib_1098 | 0.06 | 0.12 | -0.01 | -0.21 | -0.11 | 0 | -0.1368 | -1.0995 | 1.51189 | 0.17448 | 1 | -0.3348 | -1.2612 | 2.13561 | 0.01346 | 0 | -0.2322 | -1.1746 | 1.90917 | 0.06127 | 0 | 0 |
| 1378254_at | RGD1561609 | similar to TBC1 domain family member 4 | uniqAntib_1098 | -0.16 | -0.11 | 0.11 | 0.22 | -0.01 | 0 | 0.2259 | 1.16951 | 1.9254 | 0.05733 | 1 | 0.33043 | 1.25739 | 1.17648 | 0.02429 | 0 | 0.09696 | 1.06952 | 1.27457 | 0.23418 | 0 | 0 |
| 1378272_at | --- | --- | uniqAntib_1098 | 0.03 | 0.17 | 0.01 | -0.11 | 0.04 | 0 | -0.1652 | -1.1213 | 2.06101 | 0.08741 | 1 | -0.2782 | -1.2127 | 1.7036 | 0.02679 | 0 | -0.1263 | -1.0915 | 1.52741 | 0.11532 | 0 | 0 |
| 1378279_at | Gabarap | GABA(A) receptor-associated protein | uniqAntib_1098 | 0.09 | 0.22 | 0.19 | 0.60 | 0.40 | 0 | -0.0317 | -1.0222 | 0.93565 | 0.49414 | 1 | 0.38513 | 1.30597 | 0.91705 | 0.0095 | 0 | 0.17669 | 1.13029 | 1.50007 | 0.15102 | 0 | 0 |
| 1378282_at | Csnk2a2 | casein kinase 2, alpha prime polypeptide | uniqAntib_1098 | 0.09 | 0.35 | 0.52 | 0.62 | 0.37 | 0 | 0.16739 | 1.12303 | 1.93545 | 0.05508 | 1 | 0.26421 | 1.20098 | 1.2062 | 0.02847 | 0 | 0.01789 | 1.01247 | 1.26956 | 0.23571 | 0 | 0 |
| 1378384_at | Ints6 | integrator complex subunit 6 | uniqAntib_1098 | 0.04 | -0.24 | -0.05 | 0.12 | -0.06 | 0 | 0.18991 | 1.14069 | 2.05953 | 0.03543 | 1 | 0.35682 | 1.2806 | 0.9284 | 0.01027 | 0 | 0.17123 | 1.12602 | 1.8882 | 0.07693 | 0 | 0 |
| 1378474_at | --- | --- | uniqAntib_1098 | -0.13 | -0.27 | -0.38 | 0.08 | -0.32 | 0 | -0.1097 | -1.079 | 1.35163 | 0.22114 | 1 | 0.34497 | 1.27013 | 1.13954 | 0.0221 | 0 | -0.0572 | -1.0405 | 1.01963 | 0.39915 | 0 | 0 |
| 1378475_at | Appbp2 | amyloid beta precursor protein (cytoplasmic tail) bi | uniqAntib_1098 | -0.15 | -0.11 | -0.12 | 0.18 | -0.10 | 0 | -0.0038 | -1.0026 | 0.87321 | 0.6553 | 1 | 0.29179 | 1.22416 | 1.28236 | 0.04342 | 0 | 0.01607 | 1.0112 | 0.91937 | 0.55873 | 0 | 0 |
| 1378506_at | Pik3c2a | phosphatidylinositol-4-phosphate 3-kinase, catalyti | uniqAntib_1098 | -0.09 | -0.16 | -0.11 | 0.13 | -0.05 | 0 | 0.04911 | 1.03463 | 1.01838 | 0.4261 | 1 | 0.2913 | 1.22374 | 1.27718 | 0.04472 | 0 | 0.10762 | 1.07745 | 1.26056 | 0.23931 | 0 | 0 |
| 1378515_at | --- | --- | uniqAntib_1098 | -0.19 | -0.01 | 0.10 | 0.36 | -0.08 | 0 | 0.10306 | 1.07405 | 1.41586 | 0.18429 | 1 | 0.3645 | 1.28744 | 0.96138 | 0.01169 | 0 | -0.0689 | -1.0489 | 1.37623 | 0.16498 | 0 | 0 |
| 1378518_at | --- | --- | uniqAntib_1098 | -0.31 | -0.15 | 0.06 | 0.17 | -0.39 | 0 | 0.21098 | 1.15748 | 1.88715 | 0.06159 | 1 | 0.32082 | 1.24904 | 1.17786 | 0.02473 | 0 | -0.238 | -1.1794 | 3.0852 | 0.00496 | 0 | 0 |
| 1378533_at | Notch4 | notch 4 | uniqAntib_1098 | 0.08 | 0.20 | 0.03 | -0.35 | 0.20 | 0 | -0.1655 | -1.1216 | 2.07329 | 0.0862 | 1 | -0.5497 | -1.4638 | 2.9825 | 0.00038 | 0 | -0.0012 | -1.0008 | 0.95797 | 0.49884 | 0 | 0 |
| 1378561_at | --- | --- | uniqAntib_1098 | 0.07 | 0.04 | 0.12 | 0.35 | 0.15 | 0 | 0.08043 | 1.05734 | 1.39449 | 0.19515 | 1 | 0.30708 | 1.2372 | 1.18351 | 0.02458 | 0 | 0.10818 | 1.07787 | 1.50741 | 0.14827 | 0 | 0 |
| 1378578_at | --- | --- | uniqAntib_1098 | -0.23 | -0.11 | -0.15 | 0.20 | -0.12 | 0 | -0.0468 | -1.033 | 1.00594 | 0.41885 | 1 | 0.30373 | 1.23433 | 1.26896 | 0.03934 | 0 | -0.0145 | -1.0101 | 1.02236 | 0.3939 | 0 | 0 |
| 1378596_at | --- | --- | uniqAntib_1098 | -0.02 | 0.37 | 0.13 | 0.09 | 0.11 | 0 | -0.2371 | -1.1787 | 2.79542 | 0.03667 | 1 | -0.2761 | -1.2109 | 1.56279 | 0.03472 | 0 | -0.2526 | -1.1914 | 3.00262 | 0.014 | 0 | 0 |
| 1378603_at | --- | --- | uniqAntib_1098 | 0.17 | 0.17 | 0.03 | -0.11 | 0.12 | 0 | -0.142 | -1.1034 | 1.6548 | 0.14409 | 1 | -0.2827 | -1.2165 | 1.50542 | 0.04197 | 0 | -0.0466 | -1.0329 | 1.10307 | 0.31366 | 0 | 0 |
| 1378634_at | Hdac8 /// LO( | histone deacetylase 8 /// histone deacetylase 8-like | uniqAntib_1098 | -0.06 | -0.19 | -0.08 | 0.12 | -0.08 | 0 | 0.113 | 1.08147 | 1.67802 | 0.11229 | 1 | 0.309 | 1.23884 | 1.04466 | 0.01686 | 0 | 0.10722 | 1.07715 | 1.55568 | 0.13736 | 0 | 0 |
| 1378635_at | --- | --- | uniqAntib_1098 | -0.10 | 0.20 | 0.16 | -0.11 | 0.11 | 0 | -0.0346 | -1.0243 | 0.94809 | 0.47631 | 1 | -0.3015 | -1.2325 | 1.6607 | 0.029 | 0 | -0.0811 | -1.0578 | 1.15061 | 0.2766 | 0 | 0 |
| 1378661_x_at | LOC10036195 | hypothetical protein LOC100361951 /// late cornifie | uniqAntib_1098 | 0.00 | -0.06 | -0.05 | -0.36 | -0.18 | 0 | 0.00491 | 1.00341 | 0.96526 | 0.45738 | 1 | -0.3051 | -1.2355 | 1.59487 | 0.03359 | 0 | -0.1244 | -1.0901 | 1.23788 | 0.2247 | 0 | 0 |
| 1378694_at | Plxnb1 | plexin B1 | uniqAntib_1098 | 0.10 | 0.10 | 0.05 | -0.19 | 0.04 | 0 | -0.0557 | -1.0394 | 1.15415 | 0.30621 | 1 | -0.2953 | -1.2271 | 1.73507 | 0.02499 | 0 | -0.0664 | -1.0471 | 1.17795 | 0.2617 | 0 | 0 |

| Affymetrix Probeset ID | Gene Symbol | Gene Title | Figure 6 B Venn n =2021 | Mean BN log2 intensity autologous n=8 | Mean log2 intensity day 30 DR +/+ ND n=4 | Mean log2 intensity day 30 DR+/+ HCD n=4 | Mean log2 intensity day 30 DR+/+ B/S n=4 | Mean log2 intensity day 30 F+/+ ND n=4 | DR+/+ HCD vs DR+/+ ND n=380 1=yes 0=no | Log2 ratio DR+/+ HCD vs DR+/+ ND | Fold-Change DR+/+ HCD vs DR+/+ ND | RP-FDR 4DR30 HCD vs 4DR30 Reg | p value DR+/+ HCD vs DR+/+ ND | DR+/+ B/S vs DR+/+ ND n=1,330 1=yes 0=no | Log2 ratio DR+/+ B/S vs DR+/+ ND | Fold-Change DR+/+ B/S vs DR+/+ ND | RP-FDR 4DR30 Antib vs 4DR30 Reg | p value DR+/+ B/S vs DR+/+ ND | DR+/+ ND vs F+/+ ND n=398 1=yes 0=no | Log2 ratio DR+/+ ND vs F+/+ ND | Fold-Change DR+/+ ND vs F+/+ ND | RP-FDR 4RT1uu 30Reg vs 4DR30 Reg | p value DR+/+ ND vs F+/+ ND | DR+/+ Longitudinal n=228 1=yes 0=no | Figure 6F |
|---|---|---|---|---|---|---|---|---|---|---|---|---|---|---|---|---|---|---|---|---|---|---|---|---|---|
| 1378706_at | --- | --- | uniqAntib_1098 | 0.10 | 0.16 | 0.17 | -0.17 | 0.01 | 0 | 0.01634 | 1.01139 | 1.03102 | 0.4164 | 1 | -0.3295 | -1.2565 | 2.63213 | 0.0147 | 0 | -0.1433 | -1.1045 | 1.51234 | 0.12294 | 0 | 0 |
| 1378714_at | --- | --- | uniqAntib_1098 | -0.02 | 0.10 | 0.05 | -0.28 | 0.09 | 0 | -0.0519 | -1.0366 | 0.94801 | 0.47673 | 1 | -0.3816 | -1.3028 | 2.62685 | 0.00752 | 0 | -0.0133 | -1.0093 | 0.94427 | 0.52076 | 0 | 0 |
| 1378785_at | --- | --- | uniqAntib_1098 | 0.05 | 0.16 | 0.11 | -0.17 | 0.00 | 0 | -0.0516 | -1.0364 | 0.98631 | 0.43577 | 1 | -0.3358 | -1.2621 | 1.98527 | 0.01781 | 0 | -0.1631 | -1.1197 | 1.36336 | 0.17071 | 0 | 0 |
| 1378788_at | Zfp474 | zinc finger protein 474 | uniqAntib_1098 | -0.04 | 0.03 | -0.19 | -0.30 | -0.15 | 0 | -0.2248 | -1.1687 | 2.99542 | 0.02967 | 1 | -0.328 | -1.2552 | 2.07161 | 0.01452 | 0 | -0.1802 | -1.133 | 2.2249 | 0.03706 | 0 | 0 |
| 1378806_at | --- | --- | uniqAntib_1098 | -0.04 | 0.23 | 0.25 | 0.61 | 0.20 | 0 | 0.0193 | 1.01347 | 1.2822 | 0.23822 | 1 | 0.38277 | 1.30385 | 0.95186 | 0.01136 | 0 | -0.0272 | -1.019 | 1.06507 | 0.34573 | 0 | 0 |
| 1378861_at | --- | --- | uniqAntib_1098 | -0.16 | 0.19 | 0.28 | 0.52 | 0.35 | 0 | 0.09379 | 1.06717 | 1.3393 | 0.21597 | 1 | 0.33498 | 1.26136 | 1.13225 | 0.02115 | 0 | 0.16703 | 1.12274 | 1.81115 | 0.08654 | 0 | 0 |
| 1378903_at | --- | --- | uniqAntib_1098 | -0.17 | -0.03 | -0.08 | -0.34 | -0.10 | 0 | -0.0543 | -1.0384 | 1.35702 | 0.21809 | 1 | -0.3059 | -1.2362 | 2.20281 | 0.01211 | 0 | -0.0677 | -1.048 | 1.44474 | 0.14355 | 0 | 0 |
| 1378931_at | --- | --- | uniqAntib_1098 | 0.09 | 0.13 | 0.00 | -0.21 | -0.11 | 0 | -0.1324 | -1.0961 | 1.18291 | 0.29307 | 1 | -0.3445 | -1.2697 | 1.83686 | 0.0205 | 0 | -0.2473 | -1.1869 | 1.96277 | 0.05447 | 0 | 0 |
| 1378936_at | Cstf2 | cleavage stimulation factor, 3' pre-RNA subunit 2 | uniqAntib_1098 | -0.18 | -0.29 | -0.35 | 0.02 | -0.34 | 0 | -0.0524 | -1.037 | 1.07766 | 0.35366 | 1 | 0.31391 | 1.24307 | 1.11288 | 0.02011 | 0 | -0.0455 | -1.032 | 1.40518 | 0.15494 | 0 | 0 |
| 1378943_at | --- | --- | uniqAntib_1098 | 0.01 | 0.09 | -0.08 | -0.18 | -0.08 | 0 | -0.1704 | -1.1254 | 1.9849 | 0.09925 | 1 | -0.2731 | -1.2084 | 1.46982 | 0.04429 | 0 | -0.1715 | -1.1262 | 1.80278 | 0.07704 | 0 | 0 |
| 1378989_at | Aifm2 | apoptosis-inducing factor, mitochondrion-associate | uniqAntib_1098 | 0.08 | 0.14 | 0.02 | -0.14 | -0.06 | 0 | -0.1173 | -1.0847 | 1.56948 | 0.16049 | 1 | -0.2758 | -1.2107 | 1.47652 | 0.04377 | 0 | -0.1927 | -1.1429 | 1.70307 | 0.09211 | 0 | 0 |
| 1379010_at | Srsf3 | serine/arginine-rich splicing factor 3 | uniqAntib_1098 | -0.18 | -0.30 | -0.22 | 0.12 | -0.25 | 0 | 0.076 | 1.05409 | 1.84917 | 0.06553 | 1 | 0.41838 | 1.33643 | 0.68832 | 0.00474 | 0 | 0.04244 | 1.02985 | 1.64417 | 0.11753 | 0 | 0 |
| 1379016_at | --- | --- | uniqAntib_1098 | -0.01 | 0.12 | -0.08 | -0.24 | -0.08 | 0 | -0.1982 | -1.1473 | 1.75253 | 0.1286 | 1 | -0.3506 | -1.2751 | 1.85752 | 0.02001 | 0 | -0.1903 | -1.141 | 1.43939 | 0.14579 | 0 | 0 |
| 1379022_at | Adamts8 | ADAM metallopeptidase with thrombospondin type | uniqAntib_1098 | -0.03 | 0.15 | 0.06 | -0.20 | -0.09 | 0 | -0.0848 | -1.0605 | 1.38044 | 0.21067 | 1 | -0.3438 | -1.2691 | 1.8828 | 0.01943 | 0 | -0.2351 | -1.177 | 2.07077 | 0.04648 | 0 | 0 |
| 1379058_at | --- | --- | uniqAntib_1098 | -0.14 | -0.16 | -0.24 | 0.16 | -0.11 | 0 | -0.0841 | -1.06 | 1.07613 | 0.35576 | 1 | 0.32181 | 1.2499 | 1.14307 | 0.02194 | 0 | 0.05191 | 1.03663 | 1.07236 | 0.35813 | 0 | 0 |
| 1379090_at | --- | --- | uniqAntib_1098 | -0.08 | 0.04 | 0.10 | -0.24 | -0.09 | 0 | 0.05925 | 1.04193 | 0.96104 | 0.49425 | 1 | -0.2769 | -1.2116 | 1.58544 | 0.03365 | 0 | -0.1278 | -1.0926 | 1.2085 | 0.24015 | 0 | 0 |
| 1379110_at | --- | --- | uniqAntib_1098 | 0.12 | 0.26 | 0.21 | -0.02 | 0.17 | 0 | -0.0527 | -1.0372 | 1.69564 | 0.13746 | 1 | -0.2804 | -1.2146 | 2.07472 | 0.01428 | 0 | -0.0928 | -1.0665 | 1.55287 | 0.1118 | 0 | 0 |
| 1379132_at | --- | --- | uniqAntib_1098 | -0.30 | -0.12 | -0.15 | 0.19 | -0.31 | 0 | -0.0284 | -1.0198 | 1.96593 | 0.10102 | 1 | 0.30532 | 1.23569 | 0.84456 | 0.00834 | 0 | -0.196 | -1.1455 | 2.6235 | 0.02624 | 0 | 0 |
| 1379179_at | Nhsl2 | NHS-like 2 | uniqAntib_1098 | -0.13 | 0.00 | -0.04 | -0.36 | -0.12 | 0 | -0.0431 | -1.0304 | 1.09333 | 0.3445 | 1 | -0.3659 | -1.2887 | 2.3018 | 0.0111 | 0 | -0.1275 | -1.0924 | 1.36182 | 0.17139 | 0 | 0 |
| 1379187_at | --- | --- | uniqAntib_1098 | 0.01 | 0.05 | -0.09 | -0.28 | -0.08 | 0 | -0.1403 | -1.1021 | 1.41764 | 0.19747 | 1 | -0.3344 | -1.2609 | 1.92 | 0.01834 | 0 | -0.1253 | -1.0908 | 1.21243 | 0.23906 | 0 | 0 |
| 1379193_at | --- | --- | uniqAntib_1098 | 0.07 | 0.10 | 0.03 | -0.26 | -0.10 | 0 | -0.0744 | -1.0529 | 1.63241 | 0.14902 | 1 | -0.3633 | -1.2863 | 3 | 0.0056 | 0 | -0.2025 | -1.1507 | 2.25021 | 0.03517 | 0 | 0 |
| 1379249_at | Wtap | Wilms tumor 1 associated protein | uniqAntib_1098 | -0.26 | -0.37 | -0.17 | -0.08 | -0.35 | 0 | 0.1907 | 1.14131 | 2.2683 | 0.01284 | 1 | 0.28984 | 1.22251 | 0.90082 | 0.00915 | 0 | 0.01969 | 1.01374 | 1.85237 | 0.08047 | 0 | 0 |
| 1379275_at | Snx10 | sorting nexin 10 | uniqAntib_1098 | -0.11 | -0.16 | -0.29 | 0.12 | -0.07 | 0 | -0.1346 | -1.0978 | 1.29669 | 0.24079 | 1 | 0.27571 | 1.21059 | 1.25688 | 0.0369 | 0 | 0.09268 | 1.06635 | 1.31707 | 0.214 | 0 | 0 |
| 1379276_at | --- | --- | uniqAntib_1098 | 0.02 | 0.24 | 0.11 | 0.58 | 0.15 | 0 | -0.129 | -1.0935 | 1.38092 | 0.21065 | 1 | 0.34404 | 1.26931 | 1.0763 | 0.01817 | 0 | -0.0904 | -1.0647 | 1.19725 | 0.24712 | 0 | 0 |
| 1379285_at | Rtp4 | receptor (chemosensory) transporter protein 4 | uniqAntib_1098 | -0.07 | -0.26 | -0.34 | 0.01 | -0.10 | 0 | -0.0827 | -1.059 | 1.71036 | 0.13496 | 1 | 0.26503 | 1.20166 | 1.26609 | 0.0401 | 0 | 0.15328 | 1.11209 | 1.96032 | 0.06808 | 0 | 0 |
| 1379381_at | Ciita | class II, major histocompatibility complex, transacti | uniqAntib_1098 | 0.00 | 1.05 | 1.02 | 0.77 | 0.81 | 0 | -0.0321 | -1.0225 | 1.01384 | 0.41213 | 1 | -0.2833 | -1.217 | 1.67168 | 0.02822 | 0 | -0.2407 | -1.1815 | 2.18439 | 0.03997 | 0 | 0 |
| 1379433_at | Pik3c2a | phosphatidylinositol-4-phosphate 3-kinase, catalyti | uniqAntib_1098 | -0.17 | -0.31 | -0.22 | -0.03 | -0.19 | 0 | 0.08259 | 1.05892 | 1.45299 | 0.17441 | 1 | 0.2781 | 1.2126 | 1.27239 | 0.04967 | 0 | 0.11431 | 1.08246 | 1.38689 | 0.18668 | 0 | 0 |
| 1379485_at | Eif3a | eukaryotic translation initiation factor 3, subunit A | uniqAntib_1098 | -0.08 | -0.20 | -0.17 | 0.10 | -0.37 | 0 | 0.03082 | 1.02159 | 2.47087 | 0.00548 | 1 | 0.30479 | 1.23524 | 0.37927 | 0.0015 | 0 | -0.1652 | -1.1213 | 3.60069 | 0.00336 | 0 | 0 |
| 1379488_at | Tp53rk | TP53 regulating kinase | uniqAntib_1098 | -0.08 | -0.20 | -0.19 | 0.09 | -0.15 | 0 | 0.00524 | 1.00364 | 1.02387 | 0.42201 | 1 | 0.28767 | 1.22067 | 1.28001 | 0.04412 | 0 | 0.04946 | 1.03488 | 1.0478 | 0.3781 | 0 | 0 |
| 1379509_at | --- | --- | uniqAntib_1098 | -0.12 | 0.11 | -0.05 | 0.43 | 0.11 | 0 | -0.1629 | -1.1196 | 2.98729 | 0.03122 | 1 | 0.31703 | 1.24576 | 1.03517 | 0.00968 | 0 | -0.0052 | -1.0036 | 1.74286 | 0.09516 | 0 | 0 |
| 1379540_at | --- | --- | uniqAntib_1098 | -0.01 | 0.12 | 0.29 | 0.43 | 0.22 | 0 | 0.1682 | 1.12365 | 1.68248 | 0.10847 | 1 | 0.30432 | 1.23484 | 1.12713 | 0.02098 | 0 | 0.09529 | 1.06828 | 1.3808 | 0.18977 | 0 | 0 |
| 1379548_at | --- | --- | uniqAntib_1098 | -0.16 | -0.26 | -0.01 | 0.05 | -0.13 | 0 | 0.25175 | 1.19065 | 1.96034 | 0.04892 | 1 | 0.30997 | 1.23968 | 1.20494 | 0.02712 | 0 | 0.13121 | 1.09521 | 2.24502 | 0.04519 | 0 | 0 |
| 1379561_at | Atad1 /// LOC | ATPase family, AAA domain containing 1 /// ATPase | uniqAntib_1098 | -0.16 | -0.43 | -0.44 | -0.10 | -0.31 | 0 | -0.0058 | -1.004 | 1.29861 | 0.23125 | 1 | 0.3293 | 1.2564 | 1.07488 | 0.01828 | 0 | 0.11905 | 1.08602 | 1.50033 | 0.14946 | 0 | 0 |
| 1379606_at | Rab30 | RAB30, member RAS oncogene family | uniqAntib_1098 | -0.06 | -0.42 | -0.48 | -0.11 | -0.32 | 0 | -0.0552 | -1.039 | 0.98443 | 0.43725 | 1 | 0.31684 | 1.2456 | 1.22284 | 0.03244 | 0 | 0.10699 | 1.07698 | 1.16089 | 0.29426 | 0 | 0 |
| 1379608_at | RGD1560010 | RGD1560010 | uniqAntib_1098 | -0.21 | -0.08 | -0.08 | 0.20 | -0.07 | 0 | 0.00714 | 1.00496 | 0.97671 | 0.47568 | 1 | 0.28554 | 1.21887 | 1.25376 | 0.03584 | 0 | 0.01806 | 1.0126 | 1.00909 | 0.41994 | 0 | 0 |
| 1379620_at | --- | --- | uniqAntib_1098 | 0.06 | -0.01 | 0.17 | -0.27 | 0.02 | 0 | 0.17878 | 1.13192 | 1.77793 | 0.07632 | 1 | -0.2687 | -1.2047 | 1.56553 | 0.0366 | 0 | 0.02926 | 1.02049 | 1.1879 | 0.27712 | 0 | 0 |
| 1379624_at | --- | --- | uniqAntib_1098 | -0.03 | 0.17 | 0.23 | 0.45 | 0.43 | 0 | 0.05775 | 1.04084 | 1.09683 | 0.35191 | 1 | 0.27712 | 1.21175 | 1.26464 | 0.03851 | 0 | 0.25594 | 1.19411 | 2.33653 | 0.03419 | 0 | 0 |
| 1379627_at | Tm9sf3 | transmembrane 9 superfamily member 3 | uniqAntib_1098 | -0.01 | -0.03 | 0.11 | 0.33 | 0.10 | 0 | 0.14779 | 1.10787 | 1.46996 | 0.16818 | 1 | 0.36658 | 1.28929 | 0.97252 | 0.01317 | 0 | 0.13308 | 1.09663 | 1.26654 | 0.2376 | 0 | 0 |
| 1379636_at | Rmdn2 | regulator of microtubule dynamics 2 | uniqAntib_1098 | -0.21 | -0.26 | -0.21 | 0.04 | -0.22 | 0 | 0.05187 | 1.03661 | 1.06961 | 0.37678 | 1 | 0.30268 | 1.23343 | 1.20602 | 0.02905 | 0 | 0.04722 | 1.03327 | 1.2315 | 0.25561 | 0 | 0 |
| 1379698_at | --- | --- | uniqAntib_1098 | -0.05 | -0.21 | -0.01 | 0.21 | -0.04 | 0 | 0.19414 | 1.14404 | 1.62567 | 0.1264 | 1 | 0.41532 | 1.33359 | 0.70755 | 0.0053 | 0 | 0.16545 | 1.12151 | 1.41648 | 0.17827 | 0 | 0 |
| 1379722_at | Large | like-glycosyltransferase | uniqAntib_1098 | -0.09 | -0.09 | -0.03 | 0.18 | -0.13 | 0 | 0.06846 | 1.04859 | 1.15027 | 0.31254 | 1 | 0.27754 | 1.21212 | 1.14402 | 0.0224 | 0 | -0.0358 | -1.0252 | 1.12866 | 0.29183 | 0 | 0 |
| 1379760_at | Tnfaip8l2 | tumor necrosis factor, alpha-induced protein 8-like | uniqAntib_1098 | -0.03 | -0.14 | -0.05 | 0.12 | -0.03 | 0 | 0.09907 | 1.07109 | 1.23871 | 0.25926 | 1 | 0.26839 | 1.20446 | 1.26749 | 0.04667 | 0 | 0.11017 | 1.07936 | 1.25288 | 0.24462 | 0 | 0 |
| 1379817_at | --- | --- | uniqAntib_1098 | -0.09 | 0.18 | 0.24 | 0.48 | 0.17 | 0 | 0.06514 | 1.04619 | 1.19906 | 0.28289 | 1 | 0.30726 | 1.23736 | 1.23385 | 0.03317 | 0 | -0.0046 | -1.0032 | 1.02381 | 0.40434 | 0 | 0 |
| 1379829_at | Mycbp | c-myc binding protein | uniqAntib_1098 | -0.22 | -0.31 | -0.27 | -0.01 | -0.25 | 0 | 0.03531 | 1.02478 | 1.19825 | 0.28324 | 1 | 0.30185 | 1.23272 | 1.23431 | 0.03342 | 0 | 0.06144 | 1.04351 | 1.00914 | 0.42012 | 0 | 0 |
| 1379850_at | Psmc6 | proteasome (prosome, macropain) 26S subunit, ATF | uniqAntib_1098 | -0.26 | -0.41 | -0.44 | -0.13 | -0.41 | 0 | -0.0386 | -1.0271 | 1.07505 | 0.35706 | 1 | 0.27594 | 1.21078 | 1.27297 | 0.04568 | 0 | -0.0002 | -1.0001 | 1.03542 | 0.38721 | 0 | 0 |
| 1379909_at | Gkap1 | G kinase anchoring protein 1 | uniqAntib_1098 | -0.03 | 0.12 | 0.16 | 0.51 | 0.24 | 0 | 0.04038 | 1.02839 | 1.20787 | 0.2784 | 1 | 0.38963 | 1.31005 | 0.71781 | 0.00559 | 0 | 0.12262 | 1.08871 | 1.59327 | 0.12813 | 0 | 0 |
| 1379917_at | Ap3s1 | adaptor-related protein complex 3, sigma 1 subunit | uniqAntib_1098 | -0.17 | 0.01 | -0.06 | 0.32 | -0.02 | 0 | -0.0675 | -1.0479 | 1.11288 | 0.33094 | 1 | 0.31498 | 1.24399 | 1.20351 | 0.02926 | 0 | -0.0253 | -1.0177 | 1.0298 | 0.38567 | 0 | 0 |
| 1379939_a_at | RGD1306063 | similar to HT021 | uniqAntib_1098 | -0.16 | -0.32 | -0.36 | -0.65 | -0.44 | 0 | -0.0424 | -1.0299 | 1.04219 | 0.38559 | 1 | -0.332 | -1.2587 | 1.94062 | 0.0181 | 0 | -0.1201 | -1.0868 | 1.44972 | 0.14274 | 0 | 0 |
| 1379945_at | --- | --- | uniqAntib_1098 | 0.12 | -0.15 | -0.18 | 0.22 | -0.05 | 0 | -0.0257 | -1.018 | 1.69977 | 0.13637 | 1 | 0.3762 | 1.29792 | 0.74923 | 0.00624 | 0 | 0.10618 | 1.07637 | 1.96638 | 0.06601 | 0 | 0 |
| 1379971_at | Zc3h6 | zinc finger CCCH type containing 6 | uniqAntib_1098 | 0.02 | 0.30 | 0.23 | 0.03 | 0.15 | 0 | -0.0688 | -1.0488 | 1.74096 | 0.13178 | 1 | -0.2748 | -1.2098 | 2.61409 | 0.0074 | 0 | -0.1477 | -1.1078 | 2.22348 | 0.03847 | 0 | 0 |
| 1379993_at | Oaz3 | ornithine decarboxylase antizyme 3 | uniqAntib_1098 | -0.04 | 0.03 | -0.08 | -0.31 | -0.13 | 0 | -0.1112 | -1.0801 | 1.43241 | 0.19202 | 1 | -0.3344 | -1.2609 | 1.91498 | 0.01853 | 0 | -0.1588 | -1.1164 | 1.49282 | 0.12855 | 0 | 0 |
| 1380045_at | Pdp2 | pyruvate dehyrogenase phosphatase catalytic subu | uniqAntib_1098 | 0.16 | 0.10 | 0.31 | 0.40 | 0.29 | 0 | 0.20763 | 1.15479 | 1.76028 | 0.08422 | 1 | 0.29268 | 1.22491 | 1.2342 | 0.03338 | 0 | 0.18956 | 1.14041 | 2.09257 | 0.05309 | 0 | 0 |
| 1380082_at | --- | --- | uniqAntib_1098 | 0.03 | 0.19 | 0.33 | 0.52 | 0.36 | 0 | 0.14083 | 1.10254 | 1.46381 | 0.16983 | 1 | 0.32808 | 1.25534 | 1.06085 | 0.0177 | 0 | 0.16483 | 1.12103 | 1.73096 | 0.09718 | 0 | 0 |
| 1380126_at | --- | --- | uniqAntib_1098 | 0.15 | 0.24 | 0.09 | -0.05 | 0.16 | 0 | -0.1589 | -1.1165 | 2.1011 | 0.08168 | 1 | -0.2925 | -1.2248 | 2.07298 | 0.01433 | 0 | -0.0841 | -1.06 | 1.12585 | 0.29501 | 0 | 0 |
| 1380133_at | Osr2 | odd-skipped related 2 (Drosophila) | uniqAntib_1098 | 0.08 | 0.11 | 0.21 | -0.19 | 0.08 | 0 | 0.10359 | 1.07445 | 1.60889 | 0.12985 | 1 | -0.2953 | -1.2271 | 1.6587 | 0.02912 | 0 | -0.0281 | -1.0197 | 1.01891 | 0.39961 | 0 | 0 |
| 1380144_at | --- | --- | uniqAntib_1098 | -0.14 | -0.19 | -0.05 | 0.19 | -0.17 | 0 | 0.13686 | 1.09951 | 1.97538 | 0.04719 | 1 | 0.37626 | 1.29797 | 0.62202 | 0.00406 | 0 | 0.02487 | 1.01739 | 1.1611 | 0.29349 | 0 | 0 |
| 1380200_at | --- | --- | uniqAntib_1098 | 0.07 | 0.09 | 0.18 | 0.52 | 0.13 | 0 | 0.09313 | 1.06668 | 1.45985 | 0.17171 | 1 | 0.4326 | 1.34966 | 0.70694 | 0.00534 | 0 | 0.04659 | 1.03282 | 0.98349 | 0.4484 | 0 | 0 |
| 1380202_at | Zfp821 | zinc finger protein 821 | uniqAntib_1098 | -0.13 | 0.00 | 0.03 | 0.33 | 0.05 | 0 | 0.02562 | 1.01792 | 1.04125 | 0.40654 | 1 | 0.32598 | 1.25352 | 1.16928 | 0.02403 | 0 | 0.04248 | 1.02988 | 1.06388 | 0.36779 | 0 | 0 |
| 1380230_at | --- | --- | uniqAntib_1098 | -0.13 | -0.25 | -0.06 | 0.04 | -0.24 | 0 | 0.19066 | 1.14129 | 1.67575 | 0.11289 | 1 | 0.28697 | 1.22008 | 1.08268 | 0.01908 | 0 | 0.00883 | 1.00614 | 0.99168 | 0.43855 | 0 | 0 |
| 1380242_at | Lrp5 | low density lipoprotein receptor-related protein 5 | uniqAntib_1098 | -0.05 | -0.10 | -0.30 | -0.37 | -0.11 | 0 | -0.1981 | -1.1472 | 2.66632 | 0.0403 | 1 | -0.2707 | -1.2064 | 1.766 | 0.02425 | 0 | -0.0097 | -1.0068 | 1.26208 | 0.21346 | 0 | 0 |
| 1380300_at | --- | --- | uniqAntib_1098 | -0.07 | -0.06 | -0.10 | -0.40 | -0.24 | 0 | -0.0406 | -1.0286 | 1.13651 | 0.31666 | 1 | -0.3416 | -1.2671 | 2.12781 | 0.01314 | 0 | -0.1787 | -1.1319 | 1.83897 | 0.07226 | 0 | 0 |
| 1380337_at | LOC688390 | hypothetical protein LOC688390 | uniqAntib_1098 | -0.01 | 0.02 | 0.02 | -0.25 | -0.06 | 0 | 0.00082 | 1.00057 | 1.06578 | 0.38003 | 1 | -0.2707 | -1.2064 | 1.77877 | 0.02368 | 0 | -0.0797 | -1.0568 | 1.30552 | 0.19113 | 0 | 0 |
| 1380349_at | --- | --- | uniqAntib_1098 | -0.06 | -0.22 | -0.17 | 0.06 | -0.13 | 0 | 0.04614 | 1.0325 | 1.06606 | 0.37941 | 1 | 0.27778 | 1.21233 | 1.21408 | 0.03084 | 0 | 0.09309 | 1.06665 | 1.12249 | 0.31738 | 0 | 0 |
| 1380365_at | --- | --- | uniqAntib_1098 | 0.10 | -0.04 | 0.07 | 0.32 | 0.15 | 0 | 0.11245 | 1.08106 | 1.35114 | 0.21215 | 1 | 0.36214 | 1.28533 | 1.00942 | 0.01509 | 0 | 0.18702 | 1.13841 | 1.79806 | 0.08794 | 0 | 0 |
| 1380390_at | --- | --- | uniqAntib_1098 | -0.18 | -0.16 | -0.26 | -0.44 | -0.33 | 0 | -0.0971 | -1.0696 | 1.63027 | 0.14951 | 1 | -0.2746 | -1.2096 | 2.35903 | 0.01092 | 0 | -0.1657 | -1.1217 | 2.2513 | 0.03511 | 0 | 0 |
| 1380414_at | Bcl3 | B-cell CLL/lymphoma 3 | uniqAntib_1098 | 0.00 | 0.15 | 0.05 | -0.22 | 0.14 | 0 | -0.0999 | -1.0717 | 1.26679 | 0.25137 | 1 | -0.3627 | -1.2858 | 2.27935 | 0.01121 | 0 | -0.0109 | -1.0076 | 0.97532 | 0.47231 | 0 | 0 |
| 1380430_at | --- | --- | uniqAntib_1098 | -0.18 | -0.13 | -0.01 | 0.14 | 0.05 | 0 | 0.12061 | 1.08719 | 1.72156 | 0.09588 | 1 | 0.26909 | 1.20505 | 1.27633 | 0.0495 | 0 | 0.17905 | 1.13214 | 1.96301 | 0.06786 | 0 | 0 |
| 1380449_at | --- | --- | uniqAntib_1098 | 0.23 | 0.71 | 0.64 | 0.32 | 0.65 | 0 | -0.0745 | -1.053 | 2.38665 | 0.06216 | 1 | -0.3976 | -1.3173 | 3.34 | 0.00054 | 0 | -0.0628 | -1.0445 | 2.60966 | 0.02694 | 0 | 0 |
| 1380461_at | --- | --- | uniqAntib_1098 | 0.00 | 0.05 | 0.01 | -0.27 | 0.00 | 0 | -0.0419 | -1.0295 | 1.41467 | 0.20117 | 1 | -0.3181 | -1.2467 | 1.84642 | 0.02007 | 0 | -0.0512 | -1.0361 | 1.07005 | 0.33885 | 0 | 0 |
| 1380464_at | Ints6 | integrator complex subunit 6 | uniqAntib_1098 | -0.13 | 0.02 | 0.11 | 0.36 | 0.04 | 0 | 0.09294 | 1.06654 | 2.10324 | 0.02631 | 1 | 0.34908 | 1.27375 | 0.38216 | 0.00154 | 0 | 0.0245 | 1.01713 | 1.89085 | 0.07284 | 0 | 0 |
| 1380467_at | Slc16a1 | solute carrier family 16, member 1 (monocarboxylic | uniqAntib_1098 | -0.06 | -0.09 | -0.08 | -0.37 | -0.15 | 0 | 0.00968 | 1.00673 | 0.94943 | 0.51023 | 1 | -0.2724 | -1.2078 | 1.56312 | 0.03604 | 0 | -0.0589 | -1.0417 | 1.10087 | 0.31717 | 0 | 0 |

| Affymetrix Probeset ID | Gene Symbol | Gene Title | Figure 6 B Venn n =2021 | Mean BN log2 intensity autologous n=8 | Mean log2 intensity day 30 DR +/+ ND n=4 | Mean log2 intensity day 30 DR+/+ HCD n=4 | Mean log2 intensity day 30 B/S n=4 | Mean log2 intensity day 30 F+/+ ND n=4 | DR+/+ HCD vs DR+/+ ND n=380 1=yes 0=no | Log2 ratio DR+/+ HCD vs DR+/+ ND | Fold-Change DR+/+ HCD vs DR+/+ ND | RP-FDR 4DR30 HCD vs 4DR30 Reg | p value DR+/+ HCD vs DR+/+ ND | DR+/+ B/S vs DR+/+ ND n=1,330 1=yes 0=no | Log2 ratio DR+/+ B/S vs DR+/+ ND | Fold-Change DR+/+ B/S vs DR+/+ ND | RP-FDR 4DR30 Antib vs 4DR30 Reg | p value DR+/+ B/S vs DR+/+ ND | DR+/+ ND vs F+/+ ND n=398 1=yes 0=no | Log2 ratio DR+/+ ND vs F+/+ ND | Fold-Change DR+/+ ND vs F+/+ ND | RP-FDR 4RT1uu 30Reg vs 4DR30 Reg | p value DR+/+ ND vs F+/+ ND | DR+/+ Longitudinal n=228 1=yes 0=no | Figure 6F |
|---|---|---|---|---|---|---|---|---|---|---|---|---|---|---|---|---|---|---|---|---|---|---|---|---|---|
| 1380524_at | Krt86 | keratin 86 | uniqAntib_1098 | 0.01 | -0.08 | -0.05 | -0.35 | -0.28 | 0 | 0.02467 | 1.01725 | 0.97328 | 0.48018 | 1 | -0.2666 | -1.203 | 1.43042 | 0.04972 | 0 | -0.1997 | -1.1485 | 1.73943 | 0.08334 | 0 | 0 |
| 1380538_at | LOC1009106 | uncharacterized LOC100910668 /// uncharacterized | uniqAntib_1098 | 0.06 | -0.13 | -0.17 | -0.46 | -0.22 | 0 | -0.0373 | -1.0262 | 1.90153 | 0.11024 | 1 | -0.3286 | -1.2558 | 3.22319 | 0.00487 | 0 | -0.0926 | -1.0663 | 1.58553 | 0.10605 | 0 | 0 |
| 1380545_at | Asap1 | ArfGAP with SH3 domain, ankyrin repeat and PH do | uniqAntib_1098 | -0.01 | -0.12 | -0.03 | 0.20 | -0.36 | 0 | 0.09594 | 1.06876 | 2.25915 | 0.01373 | 1 | 0.32887 | 1.25603 | 0.38107 | 0.00149 | 0 | -0.2307 | -1.1734 | 4.12867 | 0.00199 | 0 | 0 |
| 1380552_at | --- | --- | uniqAntib_1098 | -0.12 | -0.36 | -0.30 | -0.01 | -0.33 | 0 | 0.05689 | 1.04022 | 1.50337 | 0.15585 | 1 | 0.34394 | 1.26922 | 0.98304 | 0.01394 | 0 | 0.02425 | 1.01695 | 1.09921 | 0.33649 | 0 | 0 |
| 1380564_at | --- | --- | uniqAntib_1098 | 0.12 | 0.03 | -0.01 | -0.27 | -0.15 | 0 | -0.0439 | -1.0309 | 1.31234 | 0.23425 | 1 | -0.3051 | -1.2355 | 1.74186 | 0.02644 | 0 | -0.1801 | -1.1329 | 1.87243 | 0.06677 | 0 | 0 |
| 1380571_at | --- | --- | uniqAntib_1098 | -0.02 | -0.08 | -0.08 | -0.36 | -0.09 | 0 | 0.0075 | 1.00521 | 1.21444 | 0.27363 | 1 | -0.2759 | -1.2107 | 2.28899 | 0.0117 | 0 | -0.006 | -1.0042 | 1.1898 | 0.25342 | 0 | 0 |
| 1380582_at | Csf1 | colony stimulating factor 1 (macrophage) | uniqAntib_1098 | 0.07 | -0.02 | 0.00 | -0.34 | -0.20 | 0 | 0.01796 | 1.01253 | 0.94731 | 0.51348 | 1 | -0.3155 | -1.2444 | 1.88556 | 0.01952 | 0 | -0.18 | -1.1329 | 1.80231 | 0.0769 | 0 | 0 |
| 1380607_at | --- | --- | uniqAntib_1098 | 0.00 | 0.03 | 0.01 | -0.25 | 0.02 | 0 | -0.0217 | -1.0151 | 0.97737 | 0.44442 | 1 | -0.2848 | -1.2182 | 1.47141 | 0.044 | 0 | -0.0103 | -1.0071 | 0.93619 | 0.53921 | 0 | 0 |
| 1380612_at | --- | --- | uniqAntib_1098 | 0.00 | 0.03 | 0.19 | 0.31 | -0.01 | 0 | 0.16039 | 1.11759 | 2.09066 | 0.02944 | 1 | 0.28458 | 1.21806 | 1.0107 | 0.01524 | 0 | -0.0365 | -1.0256 | 1.3775 | 0.16438 | 0 | 0 |
| 1380616_at | St8sia4 | ST8 alpha-N-acetyl-neuraminide alpha-2,8-sialyltrar | uniqAntib_1098 | 0.00 | -0.28 | -0.32 | 0.02 | -0.23 | 0 | -0.0395 | -1.0278 | 1.07431 | 0.35802 | 1 | 0.29102 | 1.22351 | 1.20355 | 0.02887 | 0 | 0.04993 | 1.03522 | 0.98295 | 0.44971 | 0 | 0 |
| 1380619_at | RGD1305537 | similar to RIKEN cDNA 3110001I22 | uniqAntib_1098 | -0.14 | -0.44 | -0.41 | -0.07 | -0.38 | 0 | 0.029 | 1.02031 | 1.39636 | 0.19478 | 1 | 0.36667 | 1.28938 | 0.95302 | 0.01128 | 0 | 0.05383 | 1.03801 | 1.15213 | 0.29767 | 0 | 0 |
| 1380621_at | Fes | feline sarcoma oncogene | uniqAntib_1098 | 0.04 | -0.10 | 0.05 | 0.16 | 0.07 | 0 | 0.15526 | 1.11362 | 1.76105 | 0.08783 | 1 | 0.26369 | 1.20054 | 1.16209 | 0.0238 | 0 | 0.17581 | 1.1296 | 1.74448 | 0.09553 | 0 | 0 |
| 1380632_at | --- | --- | uniqAntib_1098 | 0.09 | 0.14 | -0.06 | -0.15 | -0.01 | 0 | -0.1963 | -1.1457 | 2.40318 | 0.06058 | 1 | -0.2929 | -1.2251 | 1.79313 | 0.02341 | 0 | -0.1474 | -1.1075 | 1.49617 | 0.12542 | 0 | 0 |
| 1380644_at | --- | --- | uniqAntib_1098 | -0.18 | -0.02 | 0.10 | 0.43 | -0.09 | 0 | 0.12383 | 1.08963 | 2.14076 | 0.02457 | 1 | 0.4516 | 1.36756 | 0.38427 | 0.00153 | 0 | -0.0665 | -1.0472 | 1.74941 | 0.0823 | 0 | 0 |
| 1380678_at | --- | --- | uniqAntib_1098 | 0.21 | 0.19 | 0.06 | -0.08 | 0.08 | 0 | -0.1312 | -1.0952 | 1.74209 | 0.13142 | 1 | -0.2703 | -1.2061 | 1.47998 | 0.04312 | 0 | -0.1128 | -1.0813 | 1.24956 | 0.22083 | 0 | 0 |
| 1380695_at | --- | --- | uniqAntib_1098 | -0.01 | -0.14 | -0.06 | 0.27 | 0.12 | 0 | 0.07536 | 1.05362 | 1.58948 | 0.13258 | 1 | 0.40724 | 1.32614 | 0.72152 | 0.00566 | 0 | 0.2593 | 1.1969 | 2.27951 | 0.0376 | 0 | 0 |
| 1380699_at | --- | --- | uniqAntib_1098 | 0.03 | -0.07 | -0.26 | 0.54 | -0.05 | 0 | -0.1873 | -1.1386 | 1.91559 | 0.10749 | 1 | 0.6155 | 1.53208 | 0.09634 | 0.00022 | 0 | 0.02316 | 1.01618 | 1.45029 | 0.16635 | 0 | 0 |
| 1380711_at | --- | --- | uniqAntib_1098 | -0.08 | 0.11 | 0.03 | -0.18 | 0.06 | 0 | -0.0823 | -1.0587 | 1.13663 | 0.31651 | 1 | -0.2864 | -1.2196 | 1.6123 | 0.03199 | 0 | -0.0463 | -1.0326 | 1.11829 | 0.30195 | 0 | 0 |
| 1380763_at | --- | --- | uniqAntib_1098 | -0.28 | 0.01 | 0.18 | 0.49 | 0.05 | 0 | 0.16957 | 1.12473 | 1.97542 | 0.04478 | 1 | 0.48362 | 1.39825 | 0.37992 | 0.00154 | 0 | 0.04387 | 1.03088 | 1.66761 | 0.11068 | 0 | 0 |
| 1380775_at | Kif20b | kinesin family member 20B | uniqAntib_1098 | -0.31 | -0.15 | -0.22 | 0.20 | -0.18 | 0 | -0.0717 | -1.051 | 1.16376 | 0.30176 | 1 | 0.35088 | 1.27534 | 1.03126 | 0.01631 | 0 | -0.0355 | -1.0249 | 0.9979 | 0.43463 | 0 | 0 |
| 1380811_at | Ptpn4 | protein tyrosine phosphatase, non-receptor type 4 | uniqAntib_1098 | -0.04 | -0.32 | -0.24 | 0.01 | -0.22 | 0 | 0.07905 | 1.05632 | 1.84989 | 0.06656 | 1 | 0.33115 | 1.25802 | 1.11403 | 0.0201 | 0 | 0.0995 | 1.07141 | 1.41678 | 0.17808 | 0 | 0 |
| 1380823_at | --- | --- | uniqAntib_1098 | -0.10 | -0.04 | 0.10 | 0.23 | 0.05 | 0 | 0.13561 | 1.09856 | 1.76123 | 0.08387 | 1 | 0.27201 | 1.20749 | 1.16784 | 0.02403 | 0 | 0.08524 | 1.06087 | 1.83833 | 0.08157 | 0 | 0 |
| 1380851_at | Sema3f | sema domain, immunoglobulin domain (Ig), short b | uniqAntib_1098 | 0.10 | 0.09 | 0.15 | -0.17 | -0.11 | 0 | 0.06117 | 1.04331 | 1.00335 | 0.44246 | 1 | -0.2636 | -1.2005 | 1.43982 | 0.0469 | 0 | -0.1971 | -1.1464 | 1.80366 | 0.07586 | 0 | 0 |
| 1380898_at | --- | --- | uniqAntib_1098 | -0.07 | 0.05 | 0.24 | -0.32 | 0.06 | 0 | 0.19095 | 1.14152 | 2.08967 | 0.02869 | 1 | -0.3736 | -1.2956 | 3.54 | 0.00091 | 0 | 0.00531 | 1.00369 | 1.44193 | 0.14383 | 0 | 0 |
| 1380912_at | --- | --- | uniqAntib_1098 | 0.07 | -0.09 | -0.04 | 0.18 | -0.13 | 0 | 0.04218 | 1.02967 | 1.00705 | 0.43777 | 1 | 0.26428 | 1.20103 | 1.25226 | 0.03749 | 0 | -0.046 | -1.0324 | 0.99558 | 0.43788 | 0 | 0 |
| 1380919_at | --- | --- | uniqAntib_1098 | -0.04 | -0.01 | -0.08 | -0.34 | -0.09 | 0 | -0.0674 | -1.0478 | 1.18184 | 0.29349 | 1 | -0.3282 | -1.2555 | 2.0165 | 0.01517 | 0 | -0.0784 | -1.0558 | 1.10505 | 0.31191 | 0 | 0 |
| 1380970_at | --- | --- | uniqAntib_1098 | 0.15 | 0.12 | 0.21 | -0.22 | 0.03 | 0 | 0.09181 | 1.06571 | 1.16047 | 0.30834 | 1 | -0.3419 | -1.2674 | 1.86332 | 0.01983 | 0 | -0.092 | -1.0658 | 1.08471 | 0.32821 | 0 | 0 |
| 1381036_at | --- | --- | uniqAntib_1098 | -0.38 | -0.26 | -0.12 | 0.05 | -0.33 | 0 | 0.13631 | 1.09909 | 2.28783 | 0.01935 | 1 | 0.30988 | 1.2396 | 0.96677 | 0.01271 | 0 | -0.0717 | -1.051 | 1.47836 | 0.13382 | 0 | 0 |
| 1381038_at | --- | --- | uniqAntib_1098 | 0.03 | 0.01 | -0.04 | -0.29 | -0.02 | 0 | -0.056 | -1.0396 | 1.08466 | 0.3494 | 1 | -0.3075 | -1.2376 | 2.40165 | 0.01027 | 0 | -0.0369 | -1.0259 | 1.02051 | 0.39735 | 0 | 0 |
| 1381053_at | --- | --- | uniqAntib_1098 | -0.03 | -0.13 | -0.04 | 0.17 | -0.11 | 0 | 0.09627 | 1.06901 | 1.55439 | 0.14135 | 1 | 0.30182 | 1.2327 | 1.21181 | 0.03098 | 0 | 0.01931 | 1.01348 | 1.10499 | 0.33144 | 0 | 0 |
| 1381088_at | --- | --- | uniqAntib_1098 | -0.05 | -0.12 | -0.19 | -0.41 | -0.21 | 0 | -0.071 | -1.0504 | 1.07626 | 0.35601 | 1 | -0.2849 | -1.2183 | 1.486 | 0.04257 | 0 | -0.0902 | -1.0645 | 1.14915 | 0.2781 | 0 | 0 |
| 1381090_at | --- | --- | uniqAntib_1098 | 0.07 | 0.07 | 0.01 | -0.20 | 0.02 | 0 | -0.0562 | -1.0397 | 1.20746 | 0.27889 | 1 | -0.2667 | -1.203 | 1.60439 | 0.03209 | 0 | -0.0465 | -1.0327 | 1.00284 | 0.42701 | 0 | 0 |
| 1381099_at | --- | --- | uniqAntib_1098 | -0.07 | -0.20 | -0.10 | 0.11 | -0.23 | 0 | 0.09463 | 1.06779 | 2.02807 | 0.04115 | 1 | 0.30988 | 1.2396 | 0.91997 | 0.00944 | 0 | -0.0333 | -1.0234 | 1.48668 | 0.13084 | 0 | 0 |
| 1381102_at | --- | --- | uniqAntib_1098 | -0.16 | -0.04 | -0.22 | -0.32 | -0.27 | 0 | -0.1753 | -1.1292 | 2.35336 | 0.06455 | 1 | -0.2755 | -1.2104 | 2.25 | 0.01194 | 0 | -0.2212 | -1.1657 | 1.84406 | 0.06902 | 0 | 0 |
| 1381112_at | --- | --- | uniqAntib_1098 | 0.13 | -0.02 | -0.09 | 0.28 | 0.07 | 0 | -0.0685 | -1.0486 | 1.11426 | 0.32999 | 1 | 0.29624 | 1.22794 | 1.28143 | 0.04327 | 0 | 0.09002 | 1.06438 | 1.16546 | 0.29055 | 0 | 0 |
| 1381173_at | --- | --- | uniqAntib_1098 | -0.12 | -0.28 | -0.24 | 0.21 | -0.14 | 0 | 0.04497 | 1.03166 | 1.11025 | 0.34358 | 1 | 0.48871 | 1.40319 | 0.39523 | 0.00168 | 0 | 0.14273 | 1.10399 | 1.59287 | 0.12774 | 0 | 0 |
| 1381196_at | LOC10091141 | transmembrane 6 superfamily member 2-like /// tra | uniqAntib_1098 | 0.15 | 0.06 | 0.08 | -0.25 | -0.03 | 0 | 0.02814 | 1.0197 | 0.88904 | 0.62987 | 1 | -0.3069 | -1.237 | 1.56147 | 0.03495 | 0 | -0.0859 | -1.0614 | 1.01173 | 0.40955 | 0 | 0 |
| 1381292_at | --- | --- | uniqAntib_1098 | 0.18 | 0.20 | 0.07 | -0.09 | 0.14 | 0 | -0.1374 | -1.0999 | 1.6417 | 0.1467 | 1 | -0.2924 | -1.2247 | 2.58477 | 0.00906 | 0 | -0.0664 | -1.0471 | 1.21373 | 0.23764 | 0 | 0 |
| 1381328_at | Cr2 | complement component (3d/Epstein Barr virus) rec | uniqAntib_1098 | -0.20 | 0.37 | 0.33 | 0.09 | 0.15 | 0 | -0.0384 | -1.027 | 1.63896 | 0.14809 | 1 | -0.2846 | -1.2181 | 1.5394 | 0.03871 | 0 | -0.2218 | -1.1662 | 1.9609 | 0.05511 | 0 | 0 |
| 1381342_at | --- | --- | uniqAntib_1098 | -0.02 | 0.03 | -0.09 | -0.24 | -0.22 | 0 | -0.1163 | -1.084 | 1.55781 | 0.1632 | 1 | -0.2645 | -1.2012 | 1.57525 | 0.03394 | 0 | -0.2497 | -1.189 | 2.80791 | 0.02158 | 0 | 0 |
| 1381358_at | Aak1 | AP2 associated kinase 1 | uniqAntib_1098 | -0.11 | -0.02 | -0.19 | -0.39 | -0.14 | 0 | -0.1707 | -1.1256 | 2.34989 | 0.06468 | 1 | -0.3756 | -1.2974 | 2.48127 | 0.00814 | 0 | -0.1182 | -1.0854 | 1.12679 | 0.29316 | 0 | 0 |
| 1381387_at | --- | --- | uniqAntib_1098 | -0.02 | 0.02 | 0.05 | -0.31 | 0.06 | 0 | 0.03189 | 1.02235 | 1.12959 | 0.32923 | 1 | -0.3306 | -1.2576 | 1.99566 | 0.01566 | 0 | 0.03536 | 1.02482 | 1.28037 | 0.23266 | 0 | 0 |
| 1381415_at | --- | --- | uniqAntib_1098 | 0.05 | 0.01 | -0.13 | -0.36 | -0.14 | 0 | -0.1349 | -1.098 | 1.69422 | 0.13761 | 1 | -0.3677 | -1.2903 | 3.18204 | 0.00501 | 0 | -0.1431 | -1.1043 | 1.70684 | 0.09127 | 0 | 0 |
| 1381505_at | LOC10091157 | uncharacterized LOC100911574 | uniqAntib_1098 | -0.12 | -0.20 | 0.02 | -0.48 | -0.31 | 0 | 0.22054 | 1.16517 | 2.04107 | 0.04063 | 1 | -0.2733 | -1.2086 | 1.51436 | 0.03978 | 0 | -0.1079 | -1.0777 | 1.43803 | 0.14644 | 0 | 0 |
| 1381584_at | --- | --- | uniqAntib_1098 | 0.01 | -0.27 | -0.16 | 0.03 | -0.08 | 0 | 0.11071 | 1.07976 | 1.55569 | 0.14112 | 1 | 0.30181 | 1.23269 | 0.97123 | 0.01349 | 0 | 0.19299 | 1.14313 | 2.17023 | 0.04836 | 0 | 0 |
| 1381814_at | Srgap2 | SLIT-ROBO Rho GTPase activating protein 2 | uniqAntib_1098 | 0.10 | 0.17 | 0.08 | 0.52 | 0.29 | 0 | -0.0843 | -1.0602 | 1.62812 | 0.14968 | 1 | 0.34958 | 1.27419 | 0.94346 | 0.01053 | 0 | 0.11632 | 1.08397 | 1.45514 | 0.16498 | 0 | 0 |
| 1381815_at | --- | --- | uniqAntib_1098 | -0.10 | -0.06 | -0.09 | -0.45 | -0.16 | 0 | -0.0333 | -1.0233 | 1.20407 | 0.27962 | 1 | -0.394 | -1.3141 | 3.70419 | 0.00369 | 0 | -0.1045 | -1.0751 | 1.49902 | 0.12484 | 0 | 0 |
| 1381817_at | --- | --- | uniqAntib_1098 | -0.25 | -0.11 | 0.06 | 0.25 | -0.14 | 0 | 0.16996 | 1.12503 | 1.64316 | 0.11835 | 1 | 0.36418 | 1.28715 | 1.00731 | 0.01467 | 0 | -0.0276 | -1.0193 | 0.97197 | 0.477 | 0 | 0 |
| 1381901_at | Tmem212 | transmembrane protein 212 | uniqAntib_1098 | 0.05 | -0.02 | -0.09 | -0.30 | 0.05 | 0 | -0.0662 | -1.047 | 1.06149 | 0.36597 | 1 | -0.2709 | -1.2066 | 1.66284 | 0.02882 | 0 | 0.06959 | 1.04942 | 1.20191 | 0.27011 | 0 | 0 |
| 1381938_at | Usp13 | ubiquitin specific peptidase 13 | uniqAntib_1098 | 0.02 | 0.01 | 0.09 | -0.30 | -0.13 | 0 | 0.07945 | 1.05662 | 1.59414 | 0.13174 | 1 | -0.306 | -1.2363 | 2.41086 | 0.00992 | 0 | -0.1372 | -1.0998 | 2.11757 | 0.04406 | 0 | 0 |
| 1381958_at | Topbp1 | topoisomerase (DNA) II binding protein 1 | uniqAntib_1098 | -0.35 | -0.35 | -0.31 | 0.09 | -0.33 | 0 | 0.03899 | 1.02739 | 1.87233 | 0.0643 | 1 | 0.43123 | 1.34838 | 0.29398 | 0.00102 | 0 | 0.01778 | 1.0124 | 2.26601 | 0.04328 | 0 | 0 |
| 1381990_at | Galnt1 | UDP-N-acetyl-alpha-D-galactosamine:polypeptide N | uniqAntib_1098 | 0.09 | -0.14 | 0.08 | 0.21 | -0.18 | 0 | 0.21182 | 1.15815 | 1.86633 | 0.06451 | 1 | 0.34184 | 1.26737 | 0.9614 | 0.01218 | 0 | -0.041 | -1.0288 | 1.25475 | 0.21767 | 0 | 0 |
| 1382027_at | --- | --- | uniqAntib_1098 | -0.12 | -0.86 | -0.64 | -0.58 | -0.84 | 0 | 0.21587 | 1.1614 | 2.06145 | 0.03381 | 1 | 0.2751 | 1.21008 | 1.02152 | 0.016 | 0 | 0.01569 | 1.01093 | 1.06143 | 0.3697 | 0 | 0 |
| 1382048_at | Atp6v1a | ATPase, H+ transporting, lysosomal V1 subunit A | uniqAntib_1098 | -0.15 | -0.21 | -0.23 | 0.18 | -0.19 | 0 | -0.0243 | -1.017 | 1.35356 | 0.20987 | 1 | 0.38948 | 1.30992 | 0.69911 | 0.00506 | 0 | 0.02182 | 1.01524 | 1.12579 | 0.31577 | 0 | 0 |
| 1382067_at | Ubxn2a | UBX domain protein 2A | uniqAntib_1098 | -0.05 | -0.28 | -0.19 | -0.01 | -0.22 | 0 | 0.08667 | 1.06192 | 1.39234 | 0.19534 | 1 | 0.27154 | 1.20709 | 1.27749 | 0.04888 | 0 | 0.05256 | 1.0371 | 1.12055 | 0.31881 | 0 | 0 |
| 1382115_at | Tctn3 | tectonic family member 3 | uniqAntib_1098 | -0.06 | 0.10 | 0.00 | -0.22 | -0.13 | 0 | -0.1018 | -1.0731 | 1.42655 | 0.19257 | 1 | -0.3226 | -1.2506 | 1.87261 | 0.01981 | 0 | -0.2269 | -1.1703 | 2.21642 | 0.03685 | 0 | 0 |
| 1382129_at | Chmp1b | charged multivesicular body protein 1B | uniqAntib_1098 | -0.21 | -0.48 | -0.58 | -0.21 | -0.55 | 0 | -0.1019 | -1.0732 | 2.78735 | 0.03684 | 1 | 0.26812 | 1.20424 | 1.04569 | 0.01695 | 0 | -0.0743 | -1.0528 | 1.9043 | 0.06142 | 0 | 0 |
| 1382160_at | --- | --- | uniqAntib_1098 | -0.07 | 0.27 | 0.49 | 0.58 | 0.40 | 0 | 0.21862 | 1.16362 | 1.9899 | 0.04447 | 1 | 0.30862 | 1.23852 | 1.15613 | 0.02294 | 0 | 0.13282 | 1.09643 | 1.53789 | 0.14217 | 0 | 0 |
| 1382166_at | Srprb | signal recognition particle receptor, B subunit | uniqAntib_1098 | 0.17 | 0.02 | 0.08 | 0.28 | 0.14 | 0 | 0.06588 | 1.04672 | 1.25675 | 0.25192 | 1 | 0.26441 | 1.20114 | 1.26961 | 0.04058 | 0 | 0.12712 | 1.09211 | 1.43867 | 0.17001 | 0 | 0 |
| 1382171_at | Tsc22d2 | TSC22 domain family, member 2 | uniqAntib_1098 | -0.01 | -0.01 | 0.06 | 0.43 | 0.21 | 0 | 0.06959 | 1.04942 | 1.51273 | 0.1539 | 1 | 0.44419 | 1.36055 | 0.57478 | 0.00333 | 0 | 0.22321 | 1.16733 | 2.37029 | 0.03148 | 0 | 0 |
| 1382173_at | --- | --- | uniqAntib_1098 | -0.10 | -0.09 | 0.01 | -0.39 | -0.16 | 0 | 0.10322 | 1.07417 | 1.40555 | 0.19213 | 1 | -0.2931 | -1.2253 | 2.00543 | 0.01735 | 0 | -0.0614 | -1.0435 | 1.03963 | 0.37288 | 0 | 0 |
| 1382193_at | Cisd2 | CDGSH iron sulfur domain 2 | uniqAntib_1098 | -0.10 | -0.06 | 0.04 | 0.32 | 0.10 | 0 | 0.10489 | 1.07541 | 2.0548 | 0.03522 | 1 | 0.38058 | 1.30186 | 0.56632 | 0.00297 | 0 | 0.16256 | 1.11927 | 2.3401 | 0.02227 | 0 | 0 |
| 1382244_at | LOC10091238 | uncharacterized LOC100912382 | uniqAntib_1098 | -0.04 | 0.17 | -0.06 | -0.12 | 0.04 | 0 | -0.2308 | -1.1735 | 2.91612 | 0.03235 | 1 | -0.2861 | -1.2193 | 1.58186 | 0.03393 | 0 | -0.1269 | -1.092 | 1.39888 | 0.1591 | 0 | 0 |
| 1382252_at | --- | --- | uniqAntib_1098 | -0.03 | -0.07 | -0.08 | 0.23 | 0.08 | 0 | -0.013 | -1.0091 | 0.89602 | 0.57079 | 1 | 0.29207 | 1.2244 | 1.28081 | 0.04308 | 0 | 0.14491 | 1.10566 | 1.38557 | 0.18775 | 0 | 0 |
| 1382272_at | Agtrap | angiotensin II receptor-associated protein | uniqAntib_1098 | 0.15 | 0.22 | 0.39 | 0.56 | 0.37 | 0 | 0.17223 | 1.1268 | 1.85121 | 0.0681 | 1 | 0.3478 | 1.27262 | 0.93983 | 0.01064 | 0 | 0.15255 | 1.11153 | 1.84067 | 0.08097 | 0 | 0 |
| 1382278_at | LOC10091244 | uncharacterized LOC100912446 | uniqAntib_1098 | 0.07 | 0.29 | 0.40 | 0.85 | 0.50 | 0 | 0.11672 | 1.08427 | 1.54553 | 0.14407 | 1 | 0.56538 | 1.47977 | 0.17979 | 0.00056 | 0 | 0.21381 | 1.15975 | 2.30787 | 0.04119 | 0 | 0 |
| 1382294_at | --- | --- | uniqAntib_1098 | -0.10 | -0.21 | 0.05 | 0.17 | -0.03 | 0 | 0.25606 | 1.19421 | 2.08832 | 0.0272 | 1 | 0.38059 | 1.30187 | 0.846 | 0.0083 | 0 | 0.17459 | 1.12864 | 1.866 | 0.0792 | 0 | 0 |
| 1382309_at | --- | --- | uniqAntib_1098 | 0.31 | 0.93 | 0.92 | 1.20 | 1.06 | 0 | -0.0122 | -1.0085 | 3.09173 | 0.02764 | 1 | 0.26509 | 1.20171 | 0.56178 | 0.00305 | 0 | 0.13301 | 1.09658 | 1.70121 | 0.00542 | 0 | 0 |
| 1382311_at | Tifa | TRAF-interacting protein with forkhead-associated c | uniqAntib_1098 | -0.08 | -0.15 | -0.10 | 0.31 | -0.12 | 0 | 0.05772 | 1.04082 | 1.15587 | 0.31016 | 1 | 0.45918 | 1.37476 | 0.5664 | 0.00313 | 0 | 0.03055 | 1.0214 | 0.95529 | 0.48334 | 0 | 0 |
| 1382355_at | Galnt4 | UDP-N-acetyl-alpha-D-galactosamine:polypeptide N | uniqAntib_1098 | 0.07 | 0.06 | 0.01 | -0.25 | -0.02 | 0 | -0.0439 | -1.0309 | 0.92782 | 0.50951 | 1 | -0.3073 | -1.2373 | 1.62245 | 0.03297 | 0 | -0.0769 | -1.0548 | 1.05436 | 0.35856 | 0 | 0 |
| 1382363_at | Mpp5 | membrane protein, palmitoylated 5 (MAGUK p55 su | uniqAntib_1098 | 0.05 | -0.02 | -0.01 | 0.37 | -0.15 | 0 | 0.01043 | 1.00726 | 1.48374 | 0.16126 | 1 | 0.38403 | 1.30498 | 0.61289 | 0.00396 | 0 | -0.1293 | -1.0938 | 1.79919 | 0.07602 | 0 | 0 |

| Affymetrix Probeset ID | Gene Symbol | Gene Title | Figure 6 B Venn n =2021 | Mean BN log2 intensity autologous n=8 | Mean log2 intensity day 30 DR +/+ ND n=4 | Mean log2 intensity day 30 DR+/+ HCD n=4 | Mean log2 intensity day 30 B/S n=4 | Mean log2 intensity day 30 F+/+ ND n=4 | DR+/+ HCD vs DR+/+ ND n=380 1=yes 0=no | Log2 ratio DR+/+ HCD vs DR+/+ ND | Fold-Change DR+/+ HCD vs DR+/+ ND | RP-FDR 4DR30 HCD vs 4DR30 Reg | p value DR+/+ HCD vs DR+/+ ND | DR+/+ B/S vs DR+/+ ND n=1,330 1=yes 0=no | Log2 ratio DR+/+ B/S vs DR+/+ ND | Fold-Change DR+/+ B/S vs DR+/+ ND | RP-FDR 4DR30 Antib vs 4DR30 Reg | p value DR+/+ B/S vs DR+/+ ND | DR+/+ ND vs F+/+ ND n=398 1=yes 0=no | Log2 ratio DR+/+ ND vs F+/+ ND | Fold-Change DR+/+ ND vs F+/+ ND | RP-FDR 4RT1uu 30Reg vs 4DR30 Reg | p value DR+/+ ND vs F+/+ ND | DR+/+ Longitudinal n=228 1=yes 0=no | Figure 6F |
|---|---|---|---|---|---|---|---|---|---|---|---|---|---|---|---|---|---|---|---|---|---|---|---|---|---|
| 1382365_at | Nlk | nemo like kinase | uniqAntib_1098 | -0.13 | -0.17 | -0.14 | 0.20 | -0.12 | 0 | 0.02944 | 1.02062 | 1.07848 | 0.36676 | 1 | 0.3687 | 1.29119 | 0.94812 | 0.01101 | 0 | 0.04586 | 1.0323 | 1.15052 | 0.29803 | 0 | 0 |
| 1382431_at | Abca1 | ATP-binding cassette, subfamily A (ABC1), member | uniqAntib_1098 | 0.04 | -0.28 | -0.09 | 0.14 | -0.25 | 0 | 0.18816 | 1.13931 | 1.95356 | 0.05289 | 1 | 0.41446 | 1.3328 | 0.59878 | 0.00362 | 0 | 0.03184 | 1.02232 | 1.30004 | 0.22206 | 0 | 0 |
| 1382436_at | Chmp1b /// R | charged multivesicular body protein 1B /// similar t | uniqAntib_1098 | -0.16 | -0.51 | -0.54 | -0.10 | -0.45 | 0 | -0.0275 | -1.0193 | 1.3281 | 0.22818 | 1 | 0.41401 | 1.33238 | 0.71848 | 0.00548 | 0 | 0.05725 | 1.04048 | 1.91949 | 0.07055 | 0 | 0 |
| 1382446_at | Atp5s | ATP synthase, H+ transporting, mitochondrial Fo co | uniqAntib_1098 | -0.03 | -0.31 | -0.39 | -0.04 | -0.31 | 0 | -0.0799 | -1.057 | 1.47682 | 0.18003 | 1 | 0.26495 | 1.20159 | 1.27123 | 0.04582 | 0 | -0.0004 | -1.0003 | 0.93553 | 0.54046 | 0 | 0 |
| 1382497_at | --- | --- | uniqAntib_1098 | -0.09 | -0.16 | 0.01 | 0.14 | -0.09 | 0 | 0.16762 | 1.12321 | 1.68746 | 0.10809 | 1 | 0.29505 | 1.22693 | 1.1672 | 0.02376 | 0 | 0.06476 | 1.04591 | 1.14907 | 0.29969 | 0 | 0 |
| 1382527_at | Plcxd2 | phosphatidylinositol-specific phospholipase C, X do | uniqAntib_1098 | 0.00 | -0.04 | -0.01 | 0.29 | 0.19 | 0 | 0.03305 | 1.02317 | 1.19645 | 0.28508 | 1 | 0.33326 | 1.25985 | 0.9382 | 0.01005 | 0 | 0.23253 | 1.17489 | 2.37257 | 0.02853 | 0 | 0 |
| 1382532_at | Cela3b | chymotrypsin-like elastase family, member 3B | uniqAntib_1098 | 0.06 | 0.20 | 0.12 | -0.17 | 0.12 | 0 | -0.085 | -1.0607 | 1.20848 | 0.27815 | 1 | -0.3786 | -1.3001 | 2.34441 | 0.01093 | 0 | -0.0853 | -1.0609 | 1.03896 | 0.37407 | 0 | 0 |
| 1382541_at | Alk | anaplastic lymphoma receptor tyrosine kinase | uniqAntib_1098 | -0.06 | -0.16 | -0.17 | -0.44 | -0.21 | 0 | -0.0105 | -1.0073 | 0.93588 | 0.52898 | 1 | -0.2841 | -1.2177 | 1.65121 | 0.03026 | 0 | -0.054 | -1.0381 | 1.13613 | 0.28634 | 0 | 0 |
| 1382547_at | --- | --- | uniqAntib_1098 | 0.13 | 0.13 | 0.06 | -0.16 | -0.07 | 0 | -0.068 | -1.0483 | 1.17362 | 0.29726 | 1 | -0.29 | -1.2226 | 1.70831 | 0.0267 | 0 | -0.2061 | -1.1536 | 1.84136 | 0.06916 | 0 | 0 |
| 1382552_at | Dip2c | DIP2 disco-interacting protein 2 homolog C (Drosop | uniqAntib_1098 | -0.03 | -0.01 | -0.20 | -0.31 | -0.16 | 0 | -0.1872 | -1.1385 | 1.87783 | 0.11249 | 1 | -0.3039 | -1.2344 | 1.75186 | 0.02631 | 0 | -0.1503 | -1.1098 | 1.42937 | 0.14809 | 0 | 0 |
| 1382575_at | --- | --- | uniqAntib_1098 | -0.02 | -0.30 | -0.24 | 0.12 | -0.28 | 0 | 0.05416 | 1.03825 | 1.76459 | 0.08154 | 1 | 0.41331 | 1.33174 | 0.73169 | 0.006 | 0 | 0.01673 | 1.01166 | 1.07753 | 0.35244 | 0 | 0 |
| 1382578_at | --- | --- | uniqAntib_1098 | -0.01 | -0.04 | 0.07 | 0.28 | 0.10 | 0 | 0.11156 | 1.08039 | 1.41505 | 0.18496 | 1 | 0.32606 | 1.25359 | 1.01038 | 0.0154 | 0 | 0.14303 | 1.10422 | 1.66075 | 0.11444 | 0 | 0 |
| 1382584_at | LOC681371 | hypothetical protein LOC681371 | uniqAntib_1098 | -0.20 | 0.02 | -0.06 | 0.30 | -0.20 | 0 | -0.0815 | -1.0581 | 1.65104 | 0.14541 | 1 | 0.28183 | 1.21573 | 1.07582 | 0.01847 | 0 | -0.2254 | -1.1691 | 2.35818 | 0.032 | 0 | 0 |
| 1382592_at | Spdl1 | spindle apparatus coiled-coil protein 1 | uniqAntib_1098 | -0.01 | -0.08 | -0.08 | 0.20 | -0.09 | 0 | -0.0041 | -1.0028 | 1.07977 | 0.36529 | 1 | 0.28314 | 1.21684 | 1.27491 | 0.04477 | 0 | -0.0067 | -1.0046 | 0.96273 | 0.49169 | 0 | 0 |
| 1382594_at | Man2a1 | mannosidase, alpha, class 2A, member 1 | uniqAntib_1098 | 0.01 | 0.26 | 0.03 | -0.01 | 0.09 | 0 | -0.2358 | -1.1775 | 3.19206 | 0.02145 | 1 | -0.2705 | -1.2062 | 1.73662 | 0.02496 | 0 | -0.1768 | -1.1304 | 1.88117 | 0.06376 | 0 | 0 |
| 1382605_at | --- | --- | uniqAntib_1098 | -0.18 | -0.40 | -0.23 | -0.13 | -0.27 | 0 | 0.16799 | 1.12349 | 1.85189 | 0.06515 | 1 | 0.26637 | 1.20278 | 1.24667 | 0.035 | 0 | 0.13006 | 1.09434 | 1.66922 | 0.10831 | 0 | 0 |
| 1382651_at | --- | --- | uniqAntib_1098 | 0.04 | 0.28 | 0.31 | 0.63 | 0.36 | 0 | 0.02988 | 1.02092 | 1.66945 | 0.11429 | 1 | 0.35736 | 1.28108 | 0.76307 | 0.00655 | 0 | 0.08164 | 1.05822 | 2.04855 | 0.06179 | 0 | 0 |
| 1382653_at | Lin52 | lin-52 homolog (C. elegans) | uniqAntib_1098 | -0.14 | -0.15 | -0.16 | 0.14 | -0.22 | 0 | -0.0052 | -1.0036 | 0.96725 | 0.48657 | 1 | 0.29209 | 1.22441 | 1.2812 | 0.04396 | 0 | -0.0641 | -1.0454 | 1.08516 | 0.32786 | 0 | 0 |
| 1382654_at | --- | --- | uniqAntib_1098 | -0.05 | 0.00 | 0.22 | 0.31 | -0.05 | 0 | 0.21966 | 1.16446 | 1.85825 | 0.06495 | 1 | 0.30245 | 1.23324 | 1.20548 | 0.02837 | 0 | -0.0527 | -1.0372 | 1.13734 | 0.28548 | 0 | 0 |
| 1382665_at | --- | --- | uniqAntib_1098 | -0.23 | -0.11 | -0.07 | -0.42 | -0.05 | 0 | 0.04566 | 1.03215 | 0.99605 | 0.4517 | 1 | -0.3041 | -1.2347 | 1.75522 | 0.02455 | 0 | 0.06633 | 1.04705 | 1.45489 | 0.16528 | 0 | 0 |
| 1382680_at | --- | --- | uniqAntib_1098 | 0.01 | -0.15 | -0.22 | 0.34 | -0.10 | 0 | -0.0698 | -1.0496 | 1.25885 | 0.25368 | 1 | 0.48831 | 1.4028 | 0.4197 | 0.00181 | 0 | 0.05061 | 1.0357 | 1.1108 | 0.32561 | 0 | 0 |
| 1382681_at | --- | --- | uniqAntib_1098 | -0.37 | 0.16 | 0.42 | 0.65 | 0.32 | 0 | 0.25573 | 1.19394 | 2.27782 | 0.01846 | 1 | 0.49138 | 1.40579 | 0.46623 | 0.00219 | 0 | 0.15889 | 1.11643 | 1.72743 | 0.09759 | 0 | 0 |
| 1382702_at | --- | --- | uniqAntib_1098 | 0.01 | -0.72 | -0.78 | -0.41 | -0.85 | 0 | -0.0511 | -1.0361 | 1.08543 | 0.34902 | 1 | 0.31589 | 1.24478 | 1.15427 | 0.02294 | 0 | -0.1208 | -1.0874 | 1.30651 | 0.19006 | 0 | 0 |
| 1382716_at | --- | --- | uniqAntib_1098 | 0.01 | 0.08 | -0.13 | -0.21 | -0.15 | 0 | -0.2109 | -1.1574 | 3.02739 | 0.02911 | 1 | -0.2936 | -1.2257 | 2.45444 | 0.00979 | 0 | -0.2335 | -1.1757 | 2.75881 | 0.02244 | 0 | 0 |
| 1382746_s_at | --- | --- | uniqAntib_1098 | -0.19 | -0.11 | -0.10 | 0.29 | 0.07 | 0 | 0.00782 | 1.00543 | 0.97608 | 0.476 | 1 | 0.4056 | 1.32464 | 0.76397 | 0.00668 | 0 | 0.17981 | 1.13273 | 1.65842 | 0.11412 | 0 | 0 |
| 1382757_at | Foxl2 | forkhead box L2 | uniqAntib_1098 | 0.06 | 0.08 | 0.04 | -0.19 | -0.01 | 0 | -0.0437 | -1.0307 | 0.94124 | 0.48634 | 1 | -0.2712 | -1.2068 | 1.45684 | 0.04521 | 0 | -0.0857 | -1.0612 | 1.01439 | 0.40391 | 0 | 0 |
| 1382789_at | --- | --- | uniqAntib_1098 | 0.09 | 0.46 | 0.58 | 0.77 | 0.63 | 0 | 0.12487 | 1.09041 | 1.96307 | 0.0452 | 1 | 0.31149 | 1.24099 | 0.76124 | 0.00651 | 0 | 0.16747 | 1.12309 | 2.34714 | 0.02136 | 0 | 0 |
| 1382792_at | --- | --- | uniqAntib_1098 | 0.03 | 0.22 | 0.37 | 0.49 | 0.26 | 0 | 0.15363 | 1.11237 | 1.53097 | 0.14833 | 1 | 0.27397 | 1.20913 | 1.27983 | 0.04239 | 0 | 0.04081 | 1.02869 | 1.08679 | 0.34506 | 0 | 0 |
| 1382812_at | RGD1310429 | similar to Protein Njmu-R1 | uniqAntib_1098 | -0.17 | -0.10 | -0.09 | -0.46 | -0.01 | 0 | 0.0139 | 1.00968 | 0.88386 | 0.60425 | 1 | -0.3604 | -1.2838 | 2.51326 | 0.00768 | 0 | 0.08785 | 1.06278 | 1.09942 | 0.33595 | 0 | 0 |
| 1382816_at | --- | --- | uniqAntib_1098 | -0.13 | 0.17 | -0.01 | -0.13 | 0.00 | 0 | -0.1728 | -1.1272 | 1.90828 | 0.10928 | 1 | -0.2927 | -1.2249 | 1.59271 | 0.0336 | 0 | -0.1628 | -1.1195 | 1.32276 | 0.18319 | 0 | 0 |
| 1382839_at | --- | --- | uniqAntib_1098 | -0.19 | -0.35 | -0.14 | 0.16 | -0.25 | 0 | 0.21419 | 1.16005 | 2.09441 | 0.02674 | 1 | 0.51735 | 1.43132 | 0.37435 | 0.00138 | 0 | 0.10608 | 1.0763 | 1.43219 | 0.17389 | 0 | 0 |
| 1382845_at | --- | --- | uniqAntib_1098 | -0.06 | -0.09 | -0.15 | -0.36 | -0.19 | 0 | -0.0563 | -1.0398 | 1.06839 | 0.36158 | 1 | -0.2659 | -1.2024 | 1.47961 | 0.04353 | 0 | -0.0921 | -1.0659 | 1.21606 | 0.23622 | 0 | 0 |
| 1382856_at | Xrcc6bp1 | XRCC6 binding protein 1 | uniqAntib_1098 | 0.02 | 0.14 | 0.22 | 0.56 | 0.32 | 0 | 0.08868 | 1.06339 | 1.22287 | 0.26837 | 1 | 0.42197 | 1.33976 | 0.71239 | 0.00518 | 0 | 0.1872 | 1.13855 | 1.78235 | 0.09009 | 0 | 0 |
| 1382904_at | Nsrp1 | nuclear speckle splicing regulatory protein 1 | uniqAntib_1098 | 0.00 | -0.41 | -0.20 | -0.11 | -0.36 | 0 | 0.20831 | 1.15533 | 2.2075 | 0.02215 | 1 | 0.2903 | 1.22289 | 1.27708 | 0.04102 | 0 | 0.04575 | 1.03222 | 1.13586 | 0.30921 | 0 | 0 |
| 1382995_at | --- | --- | uniqAntib_1098 | -0.17 | 0.03 | -0.11 | 0.42 | 0.11 | 0 | -0.1404 | -1.1022 | 1.73464 | 0.13203 | 1 | 0.39332 | 1.31342 | 0.7611 | 0.00646 | 0 | 0.08152 | 1.05814 | 1.3269 | 0.20954 | 0 | 0 |
| 1383005_at | --- | --- | uniqAntib_1098 | 0.07 | -0.12 | -0.11 | 0.18 | -0.10 | 0 | 0.01162 | 1.00809 | 1.24865 | 0.25496 | 1 | 0.2983 | 1.22969 | 1.2481 | 0.03439 | 0 | 0.02157 | 1.01506 | 1.15706 | 0.29593 | 0 | 0 |
| 1383020_at | Cntrob | centrobin, centrosomal BRCA2 interacting protein | uniqAntib_1098 | 0.05 | 0.43 | 0.28 | 0.08 | 0.31 | 0 | -0.1467 | -1.107 | 1.9146 | 0.10829 | 1 | -0.3434 | -1.2688 | 3.97077 | 0.00332 | 0 | -0.1168 | -1.0844 | 1.57487 | 0.10837 | 0 | 0 |
| 1383034_at | Rybp | RING1 and YY1 binding protein | uniqAntib_1098 | -0.03 | -0.09 | 0.17 | 0.26 | -0.07 | 0 | 0.2593 | 1.19689 | 2.15991 | 0.02334 | 1 | 0.34541 | 1.27051 | 0.9727 | 0.01298 | 0 | 0.01552 | 1.01082 | 1.17831 | 0.28273 | 0 | 0 |
| 1383051_at | --- | --- | uniqAntib_1098 | 0.02 | -0.08 | 0.07 | 0.22 | 0.09 | 0 | 0.15097 | 1.11031 | 1.31692 | 0.22168 | 1 | 0.30569 | 1.23601 | 1.26207 | 0.03876 | 0 | 0.17023 | 1.12524 | 1.45621 | 0.16473 | 0 | 0 |
| 1383059_a_at | Gkap1 | G kinase anchoring protein 1 | uniqAntib_1098 | 0.12 | 0.49 | 0.31 | 0.83 | 0.38 | 0 | -0.1811 | -1.1337 | 2.45058 | 0.05603 | 1 | 0.33176 | 1.25855 | 0.81437 | 0.00749 | 0 | -0.1107 | -1.0797 | 1.60354 | 0.104 | 0 | 0 |
| 1383060_at | Gkap1 | G kinase anchoring protein 1 | uniqAntib_1098 | -0.07 | 0.06 | 0.09 | 0.34 | 0.03 | 0 | 0.02624 | 1.01836 | 0.9863 | 0.46377 | 1 | 0.28017 | 1.21434 | 1.28153 | 0.04261 | 0 | -0.0262 | -1.0183 | 0.98179 | 0.46266 | 0 | 0 |
| 1383069_at | Rbms3 | RNA binding motif, single stranded interacting prote | uniqAntib_1098 | -0.06 | -0.30 | -0.30 | -0.02 | -0.15 | 0 | 0.00283 | 1.00196 | 1.11899 | 0.33607 | 1 | 0.27943 | 1.21372 | 1.20528 | 0.02856 | 0 | 0.14821 | 1.10819 | 1.66839 | 0.10869 | 0 | 0 |
| 1383080_at | --- | --- | uniqAntib_1098 | -0.13 | -0.43 | -0.43 | -0.14 | -0.37 | 0 | -0.0022 | -1.0015 | 1.31576 | 0.22237 | 1 | 0.29133 | 1.22377 | 1.18533 | 0.02588 | 0 | 0.05887 | 1.04165 | 1.29306 | 0.22544 | 0 | 0 |
| 1383091_at | Appbp2 | amyloid beta precursor protein (cytoplasmic tail) bi | uniqAntib_1098 | -0.09 | -0.22 | -0.12 | 0.16 | -0.15 | 0 | 0.10283 | 1.07388 | 1.48007 | 0.16481 | 1 | 0.38678 | 1.30747 | 0.9536 | 0.01131 | 0 | 0.07627 | 1.05429 | 1.11136 | 0.32527 | 0 | 0 |
| 1383097_at | RGD1311783 | similar to RIKEN cDNA 2010012O05 | uniqAntib_1098 | 0.01 | -0.01 | 0.10 | 0.26 | 0.04 | 0 | 0.11382 | 1.08209 | 1.51199 | 0.15393 | 1 | 0.26642 | 1.20282 | 1.25574 | 0.03622 | 0 | 0.04729 | 1.03332 | 1.18251 | 0.28088 | 0 | 0 |
| 1383112_at | Trps1 | trichorhinophalangeal syndrome I | uniqAntib_1098 | 0.08 | -0.06 | 0.06 | 0.20 | -0.01 | 0 | 0.128 | 1.09278 | 1.96037 | 0.04923 | 1 | 0.26765 | 1.20385 | 1.27178 | 0.0456 | 0 | 0.05061 | 1.0357 | 1.40885 | 0.18193 | 0 | 0 |
| 1383146_at | RGD1562629 | similar to neurobeachin | uniqAntib_1098 | -0.03 | -0.59 | -0.61 | -0.26 | -0.53 | 0 | -0.0247 | -1.0173 | 1.77047 | 0.07754 | 1 | 0.32328 | 1.25117 | 0.6051 | 0.00381 | 0 | 0.05433 | 1.03838 | 1.80686 | 0.08512 | 0 | 0 |
| 1383161_a_at | --- | --- | uniqAntib_1098 | -0.26 | -0.86 | -0.88 | -0.55 | -0.64 | 0 | -0.0187 | -1.013 | 1.75592 | 0.08944 | 1 | 0.31287 | 1.24217 | 0.77604 | 0.00665 | 0 | 0.22175 | 1.16614 | 2.26586 | 0.01355 | 0 | 0 |
| 1383189_at | --- | --- | uniqAntib_1098 | -0.02 | 0.13 | -0.05 | -0.16 | -0.04 | 0 | -0.1827 | -1.135 | 1.75063 | 0.12975 | 1 | -0.2938 | -1.2259 | 1.56607 | 0.03641 | 0 | -0.1748 | -1.1288 | 1.52239 | 0.1194 | 0 | 0 |
| 1383276_at | Lancl2 | LanC lantibiotic synthetase component C-like 2 (bac | uniqAntib_1098 | 0.02 | -0.35 | -0.30 | 0.11 | -0.14 | 0 | 0.05438 | 1.03842 | 1.2575 | 0.25143 | 1 | 0.46615 | 1.38142 | 0.49333 | 0.00238 | 0 | 0.21656 | 1.16196 | 2.10882 | 0.05235 | 0 | 0 |
| 1383330_at | --- | --- | uniqAntib_1098 | -0.19 | -0.47 | -0.45 | -0.14 | -0.41 | 0 | 0.02327 | 1.01626 | 1.47007 | 0.16831 | 1 | 0.33201 | 1.25876 | 0.43525 | 0.00197 | 0 | 0.06251 | 1.04428 | 1.72147 | 0.09853 | 0 | 0 |
| 1383367_at | Samd14 | sterile alpha motif domain containing 14 | uniqAntib_1098 | 0.09 | 0.01 | -0.06 | -0.27 | -0.17 | 0 | -0.0732 | -1.0521 | 1.43892 | 0.19058 | 1 | -0.2841 | -1.2177 | 1.55898 | 0.03725 | 0 | -0.1797 | -1.1327 | 1.61965 | 0.10156 | 0 | 0 |
| 1383418_at | Adam11 | ADAM metallopeptidase domain 11 | uniqAntib_1098 | 0.08 | 0.18 | -0.03 | -0.17 | 0.03 | 0 | -0.2072 | -1.1544 | 2.06587 | 0.08729 | 1 | -0.3469 | -1.2718 | 2.02907 | 0.01683 | 0 | -0.1461 | -1.1066 | 1.3391 | 0.17814 | 0 | 0 |
| 1383440_at | Accs | 1-aminocyclopropane-1-carboxylate synthase homo | uniqAntib_1098 | -0.17 | 0.01 | -0.10 | -0.28 | -0.08 | 0 | -0.1131 | -1.0816 | 1.23522 | 0.26314 | 1 | -0.291 | -1.2235 | 1.44309 | 0.04636 | 0 | -0.0883 | -1.0631 | 1.1761 | 0.26212 | 0 | 0 |
| 1383445_at | --- | --- | uniqAntib_1098 | -0.03 | 0.01 | -0.15 | 0.28 | 0.08 | 0 | -0.154 | -1.1126 | 2.05977 | 0.08756 | 1 | 0.27599 | 1.21083 | 1.27609 | 0.04112 | 0 | 0.07749 | 1.05518 | 1.10763 | 0.32945 | 0 | 0 |
| 1383510_at | LOC1009121( | uncharacterized LOC100912169 | uniqAntib_1098 | 0.00 | -0.20 | -0.10 | 0.14 | -0.17 | 0 | 0.09824 | 1.07047 | 1.54155 | 0.14588 | 1 | 0.33477 | 1.26118 | 0.94888 | 0.01086 | 0 | 0.02919 | 1.02044 | 1.12443 | 0.31666 | 0 | 0 |
| 1383524_at | --- | --- | uniqAntib_1098 | -0.06 | 0.17 | 0.02 | -0.13 | -0.08 | 0 | -0.1466 | -1.1069 | 1.85136 | 0.11787 | 1 | -0.2992 | -1.2305 | 1.80312 | 0.02325 | 0 | -0.2436 | -1.184 | 2.34996 | 0.03393 | 0 | 0 |
| 1383553_at | Nlrx1 | NLR family member X1 | uniqAntib_1098 | 0.07 | 0.18 | 0.05 | -0.19 | -0.07 | 0 | -0.1244 | -1.09 | 1.36009 | 0.21732 | 1 | -0.3704 | -1.2927 | 2.60177 | 0.00945 | 0 | -0.2433 | -1.1837 | 2.33661 | 0.03253 | 0 | 0 |
| 1383582_at | Tmem54 | transmembrane protein 54 | uniqAntib_1098 | 0.08 | 0.16 | 0.17 | -0.21 | 0.14 | 0 | 0.01065 | 1.00741 | 1.07607 | 0.35615 | 1 | -0.369 | -1.2915 | 2.91 | 0.0059 | 0 | -0.0176 | -1.0122 | 1.14025 | 0.28331 | 0 | 0 |
| 1383601_at | Bclaf1 /// LOC | BCL2-associated transcription factor 1 /// protein FA | uniqAntib_1098 | -0.05 | 0.18 | 0.14 | 0.66 | 0.24 | 0 | -0.0394 | -1.0277 | 1.01087 | 0.41392 | 1 | 0.4807 | 1.39542 | 0.4943 | 0.00237 | 0 | 0.05752 | 1.04068 | 1.01377 | 0.41699 | 0 | 0 |
| 1383648_x_at | Crb3 | crumbs homolog 3 (Drosophila) | uniqAntib_1098 | 0.08 | 0.08 | 0.07 | -0.23 | -0.15 | 0 | -0.0112 | -1.0078 | 0.94256 | 0.52054 | 1 | -0.3042 | -1.2348 | 1.51799 | 0.03954 | 0 | -0.2287 | -1.1718 | 1.83916 | 0.0715 | 0 | 0 |
| 1383693_at | --- | --- | uniqAntib_1098 | 0.17 | 0.16 | 0.25 | -0.13 | -0.01 | 0 | 0.08758 | 1.06258 | 1.13656 | 0.32506 | 1 | -0.291 | -1.2235 | 1.70196 | 0.02731 | 0 | -0.17 | -1.1251 | 1.48586 | 0.13096 | 0 | 0 |
| 1383697_at | --- | --- | uniqAntib_1098 | -0.06 | -0.29 | -0.17 | 0.14 | -0.33 | 0 | 0.11324 | 1.08166 | 1.42398 | 0.18183 | 1 | 0.42571 | 1.34323 | 0.71229 | 0.0052 | 0 | -0.0446 | -1.0314 | 1.0147 | 0.40351 | 0 | 0 |
| 1383744_at | --- | --- | uniqAntib_1098 | -0.10 | -0.20 | -0.16 | 0.40 | -0.05 | 0 | 0.04504 | 1.03172 | 1.1414 | 0.31993 | 1 | 0.60327 | 1.51915 | 0.10356 | 0.00024 | 0 | 0.15488 | 1.11333 | 1.51011 | 0.14776 | 0 | 0 |
| 1383761_at | --- | --- | uniqAntib_1098 | -0.35 | -0.59 | -0.34 | -0.28 | -0.44 | 0 | 0.24506 | 1.18514 | 2.27776 | 0.01179 | 1 | 0.30656 | 1.23675 | 0.95887 | 0.0115 | 0 | 0.14947 | 1.10916 | 2.36218 | 0.02826 | 0 | 0 |
| 1383834_at | Agap3 | ArfGAP with GTPase domain, ankyrin repeat and PH | uniqAntib_1098 | 0.11 | 0.30 | 0.43 | 0.59 | 0.39 | 0 | 0.12915 | 1.09365 | 1.31454 | 0.22246 | 1 | 0.28919 | 1.22195 | 1.27091 | 0.0407 | 0 | 0.09067 | 1.06487 | 1.17738 | 0.2844 | 0 | 0 |
| 1383852_at | --- | --- | uniqAntib_1098 | 0.21 | 0.73 | 0.51 | 0.44 | 0.54 | 0 | -0.2207 | -1.1653 | 3.19903 | 0.01697 | 1 | -0.2846 | -1.2181 | 2.2943 | 0.01166 | 0 | -0.1832 | -1.1354 | 2.23049 | 0.03794 | 0 | 0 |
| 1383924_at | LOC688765 | hypothetical protein LOC688765 | uniqAntib_1098 | 0.09 | 0.07 | -0.02 | -0.25 | -0.15 | 0 | -0.0986 | -1.0707 | 1.24619 | 0.25959 | 1 | -0.3224 | -1.2504 | 1.622 | 0.03182 | 0 | -0.2254 | -1.1691 | 1.94826 | 0.05713 | 0 | 0 |
| 1383956_at | RGD1565709 | similar to ovostatin-2 | uniqAntib_1098 | 0.03 | -0.42 | -0.36 | 0.03 | -0.37 | 0 | 0.05527 | 1.03905 | 1.20386 | 0.27984 | 1 | 0.44357 | 1.35997 | 0.54058 | 0.00271 | 0 | 0.04297 | 1.03023 | 1.18598 | 0.27892 | 0 | 0 |
| 1383966_at | --- | --- | uniqAntib_1098 | 0.08 | 0.10 | 0.08 | -0.19 | -0.10 | 0 | -0.0129 | -1.009 | 1.02972 | 0.39829 | 1 | -0.2838 | -1.2174 | 1.54561 | 0.03777 | 0 | -0.1974 | -1.1467 | 1.54517 | 0.11274 | 0 | 0 |
| 1384000_at | Sox4 | SRY (sex determining region Y)-box 4 | uniqAntib_1098 | -0.19 | -0.42 | -0.44 | -0.12 | -0.45 | 0 | -0.0148 | -1.0103 | 1.48421 | 0.17873 | 1 | 0.30449 | 1.23498 | 0.93733 | 0.0104 | 0 | -0.0274 | -1.0192 | 1.48708 | 0.13002 | 0 | 0 |

| Affymetrix Probeset ID | Gene Symbol | Gene Title | Figure 6 B Venn n =2021 | Mean BN log2 intensity autologous n=8 | Mean log2 intensity day 30 DR +/+ ND n=4 | Mean log2 intensity day 30 DR+/+ HCD n=4 | Mean log2 intensity day 30 B/S n=4 | Mean log2 intensity day 30 F+/+ ND n=4 | DR+/+ HCD vs DR+/+ ND n=380 1=yes 0=no | Log2 ratio DR+/+ HCD vs DR+/+ ND | Fold-Change DR+/+ HCD vs DR+/+ ND | RP-FDR 4DR30 HCD vs 4DR30 Reg | p value DR+/+ HCD vs DR+/+ ND | DR+/+ B/S vs DR+/+ ND n=1,330 1=yes 0=no | Log2 ratio DR+/+ B/S vs DR+/+ ND | Fold-Change DR+/+ B/S vs DR+/+ ND | RP-FDR 4DR30 Antib vs 4DR30 Reg | p value DR+/+ B/S vs DR+/+ ND | DR+/+ ND vs F+/+ ND n=398 1=yes 0=no | Log2 ratio DR+/+ ND vs F+/+ ND | Fold-Change DR+/+ ND vs F+/+ ND | RP-FDR 4RT1uu 30Reg vs 4DR30 Reg | p value DR+/+ ND vs F+/+ ND | DR+/+ Longitudinal n=228 1=yes 0=no | Figure 6F |
|---|---|---|---|---|---|---|---|---|---|---|---|---|---|---|---|---|---|---|---|---|---|---|---|---|---|
| 1384059_at | --- | --- | uniqAntib_1098 | -0.18 | -0.06 | -0.06 | 0.22 | -0.07 | 0 | 0.0058 | 1.00403 | 1.056 | 0.38547 | 1 | 0.28191 | 1.2158 | 1.26572 | 0.03846 | 0 | -0.0103 | -1.0072 | 0.98747 | 0.45298 | 0 | 0 |
| 1384083_at | B3gntl1 | UDP-GlcNAc:betaGal beta-1,3-N-acetylglucosaminyl | uniqAntib_1098 | 0.07 | 0.18 | 0.02 | -0.13 | -0.05 | 0 | -0.1647 | -1.121 | 1.885 | 0.11147 | 1 | -0.3059 | -1.2362 | 1.67487 | 0.02833 | 0 | -0.2322 | -1.1746 | 1.97475 | 0.05359 | 0 | 0 |
| 1384204_at | --- | --- | uniqAntib_1098 | 0.03 | 0.03 | 0.06 | 0.35 | 0.08 | 0 | 0.02845 | 1.01991 | 1.16566 | 0.30409 | 1 | 0.3193 | 1.24772 | 1.18299 | 0.02465 | 0 | 0.04428 | 1.03117 | 0.98933 | 0.44197 | 0 | 0 |
| 1384216_at | --- | --- | uniqAntib_1098 | -0.07 | -0.06 | -0.01 | 0.27 | 0.13 | 0 | 0.04812 | 1.03392 | 0.99076 | 0.45653 | 1 | 0.33008 | 1.25708 | 1.18214 | 0.02486 | 0 | 0.18633 | 1.13787 | 1.57109 | 0.13337 | 0 | 0 |
| 1384256_at | Larp7 | La ribonucleoprotein domain family, member 7 | uniqAntib_1098 | -0.15 | -0.28 | -0.15 | 0.10 | -0.21 | 0 | 0.1249 | 1.09043 | 1.43389 | 0.17973 | 1 | 0.37392 | 1.29587 | 0.96649 | 0.01187 | 0 | 0.06593 | 1.04676 | 1.11587 | 0.32196 | 0 | 0 |
| 1384268_at | Ccnyl1 | cyclin Y-like 1 | uniqAntib_1098 | 0.02 | 0.30 | 0.38 | 0.57 | 0.37 | 0 | 0.08705 | 1.06219 | 1.18219 | 0.29389 | 1 | 0.27928 | 1.21359 | 1.26019 | 0.03683 | 0 | 0.07788 | 1.05546 | 1.1552 | 0.29672 | 0 | 0 |
| 1384281_at | --- | --- | uniqAntib_1098 | -0.05 | 0.03 | -0.02 | 0.47 | 0.25 | 0 | -0.0473 | -1.0334 | 0.97922 | 0.44161 | 1 | 0.44335 | 1.35976 | 0.59099 | 0.00363 | 0 | 0.22257 | 1.16681 | 2.21555 | 0.04581 | 0 | 0 |
| 1384323_at | Psmc6 | proteasome (prosome, macropain) 26S subunit, ATF | uniqAntib_1098 | -0.21 | -0.40 | -0.44 | -0.12 | -0.35 | 0 | -0.0404 | -1.0284 | 1.51727 | 0.17359 | 1 | 0.27429 | 1.2094 | 1.27823 | 0.04464 | 0 | 0.04999 | 1.03526 | 1.29438 | 0.22467 | 0 | 0 |
| 1384329_at | Prss22 | protease, serine, 22 | uniqAntib_1098 | 0.15 | 0.07 | -0.01 | -0.20 | -0.06 | 0 | -0.077 | -1.0548 | 1.21733 | 0.27166 | 1 | -0.2718 | -1.2074 | 1.89376 | 0.01943 | 0 | -0.128 | -1.0928 | 1.49873 | 0.12491 | 0 | 0 |
| 1384417_at | Apoc4 | apolipoprotein C-IV | uniqAntib_1098 | -0.09 | 0.02 | -0.02 | -0.24 | -0.02 | 0 | -0.0445 | -1.0313 | 1.37374 | 0.21296 | 1 | -0.2645 | -1.2012 | 2.39094 | 0.01061 | 0 | -0.0408 | -1.0287 | 1.5964 | 0.10503 | 0 | 0 |
| 1384422_at | --- | --- | uniqAntib_1098 | 0.02 | 0.31 | 0.35 | 0.78 | 0.33 | 0 | 0.03639 | 1.02555 | 0.93195 | 0.53417 | 1 | 0.46504 | 1.38035 | 0.57056 | 0.00325 | 0 | 0.01941 | 1.01354 | 0.90284 | 0.60771 | 0 | 0 |
| 1384423_at | LOC10036325 | 5'-nucleotidase, cytosolic II | uniqAntib_1098 | -0.06 | 0.05 | 0.02 | 0.35 | 0.03 | 0 | -0.0351 | -1.0246 | 1.05142 | 0.37487 | 1 | 0.30008 | 1.23121 | 1.2478 | 0.03443 | 0 | -0.0175 | -1.0122 | 1.08109 | 0.33091 | 0 | 0 |
| 1384434_at | --- | --- | uniqAntib_1098 | 0.03 | 0.08 | -0.07 | -0.18 | -0.06 | 0 | -0.1502 | -1.1098 | 1.58222 | 0.15746 | 1 | -0.2671 | -1.2034 | 1.72975 | 0.02653 | 0 | -0.1402 | -1.102 | 1.6491 | 0.09752 | 0 | 0 |
| 1384468_at | --- | --- | uniqAntib_1098 | -0.40 | -1.39 | -1.27 | -1.06 | -1.28 | 0 | 0.1215 | 1.08786 | 2.25103 | 0.01549 | 1 | 0.33116 | 1.25802 | 0.56556 | 0.00311 | 0 | 0.1084 | 1.07803 | 2.37866 | 0.03136 | 0 | 0 |
| 1384501_at | --- | --- | uniqAntib_1098 | 0.08 | -0.17 | -0.15 | 0.16 | 0.04 | 0 | 0.01593 | 1.0111 | 1.09915 | 0.35043 | 1 | 0.32947 | 1.25656 | 1.14171 | 0.02195 | 0 | 0.20972 | 1.15646 | 2.04862 | 0.06166 | 0 | 0 |
| 1384515_at | Hey2 | hairy/enhancer-of-split related with YRPW motif 2 | uniqAntib_1098 | -0.20 | -0.53 | -0.28 | -0.13 | -0.38 | 0 | 0.25038 | 1.18952 | 2.04148 | 0.04031 | 1 | 0.3983 | 1.31795 | 0.72464 | 0.00578 | 0 | 0.14772 | 1.10782 | 1.69367 | 0.10473 | 0 | 0 |
| 1384521_at | --- | --- | uniqAntib_1098 | -0.25 | -0.10 | -0.22 | -0.46 | -0.13 | 0 | -0.1209 | -1.0874 | 2.09152 | 0.08393 | 1 | -0.3576 | -1.2813 | 2.78029 | 0.00626 | 0 | -0.0336 | -1.0236 | 1.07405 | 0.33656 | 0 | 0 |
| 1384531_at | Crb3 | crumbs homolog 3 (Drosophila) | uniqAntib_1098 | 0.19 | -0.01 | 0.03 | -0.29 | -0.07 | 0 | 0.04087 | 1.02873 | 0.95517 | 0.50312 | 1 | -0.2775 | -1.2121 | 1.54459 | 0.03819 | 0 | -0.0655 | -1.0465 | 1.02259 | 0.39386 | 0 | 0 |
| 1384561_at | Muc5b | mucin 5B, oligomeric mucus/gel-forming | uniqAntib_1098 | 0.03 | 0.01 | 0.02 | -0.28 | -0.07 | 0 | 0.0117 | 1.00814 | 0.89282 | 0.57748 | 1 | -0.2826 | -1.2163 | 1.45105 | 0.0454 | 0 | -0.0715 | -1.0508 | 1.04961 | 0.3639 | 0 | 0 |
| 1384565_at | Ndc1 | NDC1 transmembrane nucleoporin | uniqAntib_1098 | -0.08 | -0.10 | -0.05 | 0.18 | -0.02 | 0 | 0.05272 | 1.03722 | 1.13066 | 0.32848 | 1 | 0.28427 | 1.21779 | 1.264 | 0.03853 | 0 | 0.07782 | 1.05542 | 1.07953 | 0.35098 | 0 | 0 |
| 1384588_at | Naa40 | N(alpha)-acetyltransferase 40, NatD catalytic subun | uniqAntib_1098 | 0.09 | 0.28 | 0.33 | 0.57 | 0.38 | 0 | 0.04795 | 1.0338 | 1.06534 | 0.38031 | 1 | 0.28905 | 1.22183 | 1.26663 | 0.04032 | 0 | 0.09494 | 1.06802 | 1.19397 | 0.2742 | 0 | 0 |
| 1384598_at | --- | --- | uniqAntib_1098 | -0.28 | -0.51 | -0.39 | -0.23 | -0.46 | 0 | 0.11326 | 1.08167 | 1.8754 | 0.06428 | 1 | 0.27672 | 1.21144 | 1.21632 | 0.03074 | 0 | 0.05162 | 1.03643 | 1.21131 | 0.26494 | 0 | 0 |
| 1384625_at | Gtsf1l | gametocyte specific factor 1-like | uniqAntib_1098 | 0.11 | 0.21 | 0.26 | -0.09 | 0.08 | 0 | 0.05419 | 1.03827 | 1.02669 | 0.41997 | 1 | -0.2976 | -1.2291 | 1.54079 | 0.03835 | 0 | -0.1303 | -1.0945 | 1.37623 | 0.16533 | 0 | 0 |
| 1384681_at | Frmd4b | FERM domain containing 4B | uniqAntib_1098 | -0.04 | -0.23 | -0.02 | 0.13 | -0.01 | 0 | 0.20262 | 1.15078 | 1.95823 | 0.04584 | 1 | 0.35628 | 1.28012 | 0.95262 | 0.01133 | 0 | 0.21262 | 1.15879 | 2.2125 | 0.04603 | 0 | 0 |
| 1384692_at | --- | --- | uniqAntib_1098 | -0.13 | -0.34 | -0.28 | 0.08 | -0.21 | 0 | 0.06697 | 1.04751 | 1.64213 | 0.1178 | 1 | 0.42731 | 1.34473 | 0.60731 | 0.00385 | 0 | 0.13462 | 1.09781 | 2.07841 | 0.05601 | 0 | 0 |
| 1384743_at | --- | --- | uniqAntib_1098 | -0.23 | -1.46 | -1.65 | -0.98 | -1.56 | 0 | -0.1873 | -1.1386 | 3.50128 | 0.00439 | 1 | 0.48197 | 1.39665 | 0.10347 | 0.00024 | 0 | -0.0912 | -1.0652 | 2.99205 | 0.01405 | 0 | 0 |
| 1384804_at | Dnmt3a | DNA (cytosine-5-)-methyltransferase 3 alpha | uniqAntib_1098 | -0.07 | 0.05 | 0.29 | 0.37 | 0.18 | 0 | 0.23639 | 1.17804 | 1.96463 | 0.04814 | 1 | 0.32163 | 1.24974 | 1.10559 | 0.01991 | 0 | 0.13139 | 1.09535 | 1.47903 | 0.15742 | 0 | 0 |
| 1384808_at | Stox2 | storkhead box 2 | uniqAntib_1098 | -0.23 | -0.37 | -0.19 | -0.07 | -0.35 | 0 | 0.18076 | 1.13348 | 1.90229 | 0.05946 | 1 | 0.30719 | 1.2373 | 1.20313 | 0.02956 | 0 | 0.02283 | 1.01595 | 1.00857 | 0.42047 | 0 | 0 |
| 1384817_at | Shc3 | SHC (Src homology 2 domain containing) transformi | uniqAntib_1098 | -0.12 | -0.12 | -0.29 | -0.45 | -0.26 | 0 | -0.167 | -1.1228 | 2.53333 | 0.04961 | 1 | -0.3276 | -1.2549 | 2.35385 | 0.01082 | 0 | -0.1392 | -1.1013 | 1.52445 | 0.11902 | 0 | 0 |
| 1384844_at | Ankrd13b | ankyrin repeat domain 13B | uniqAntib_1098 | 0.08 | 0.19 | 0.08 | -0.15 | 0.03 | 0 | -0.1111 | -1.08 | 1.41023 | 0.20066 | 1 | -0.3339 | -1.2604 | 1.73586 | 0.02506 | 0 | -0.1584 | -1.1161 | 1.3636 | 0.17109 | 0 | 0 |
| 1384881_at | Zdhhc19 | zinc finger, DHHC-type containing 19 | uniqAntib_1098 | 0.00 | 0.16 | 0.13 | -0.10 | 0.05 | 0 | -0.03 | -1.021 | 1.12162 | 0.3255 | 1 | -0.2688 | -1.2048 | 1.56073 | 0.03709 | 0 | -0.1114 | -1.0802 | 1.4376 | 0.14631 | 0 | 0 |
| 1384882_at | Hectd2 | HECT domain containing 2 | uniqAntib_1098 | 0.03 | 0.09 | 0.12 | 0.36 | 0.24 | 0 | 0.03251 | 1.02279 | 1.35155 | 0.21087 | 1 | 0.27275 | 1.20811 | 1.20505 | 0.02864 | 0 | 0.15346 | 1.11223 | 1.9628 | 0.06734 | 0 | 0 |
| 1384920_at | --- | --- | uniqAntib_1098 | -0.21 | -0.10 | 0.12 | 0.17 | -0.07 | 0 | 0.22037 | 1.16503 | 2.06171 | 0.03964 | 1 | 0.26876 | 1.20477 | 1.26657 | 0.04008 | 0 | 0.03135 | 1.02197 | 1.13571 | 0.30902 | 0 | 0 |
| 1384927_at | Tubb4a | tubulin, beta 4A class IVa | uniqAntib_1098 | 0.05 | 0.18 | 0.09 | -0.10 | -0.02 | 0 | -0.0896 | -1.0641 | 1.16875 | 0.29964 | 1 | -0.2777 | -1.2123 | 1.43847 | 0.0482 | 0 | -0.1986 | -1.1476 | 1.58052 | 0.10683 | 0 | 0 |
| 1384946_at | Tlr1 | toll-like receptor 1 | uniqAntib_1098 | -0.18 | -0.73 | -0.81 | -0.17 | -0.53 | 0 | -0.0739 | -1.0526 | 1.24322 | 0.26033 | 1 | 0.5636 | 1.47795 | 0.12988 | 0.00036 | 0 | 0.20052 | 1.14911 | 1.94317 | 0.06917 | 0 | 0 |
| 1384949_at | --- | --- | uniqAntib_1098 | 0.06 | -0.23 | -0.14 | 0.04 | -0.21 | 0 | 0.08879 | 1.06348 | 1.24439 | 0.25629 | 1 | 0.27577 | 1.21064 | 1.27695 | 0.04533 | 0 | 0.01959 | 1.01367 | 0.9591 | 0.47883 | 0 | 0 |
| 1384963_at | --- | --- | uniqAntib_1098 | 0.26 | 0.47 | 0.38 | 0.76 | 0.61 | 0 | -0.0897 | -1.0642 | 1.42341 | 0.19859 | 1 | 0.29095 | 1.22344 | 1.13511 | 0.02135 | 0 | 0.14076 | 1.10248 | 1.65701 | 0.11514 | 0 | 0 |
| 1384974_at | Fam219a | family with sequence similarity 219, member A | uniqAntib_1098 | -0.02 | 0.13 | -0.02 | -0.21 | 0.08 | 0 | -0.1488 | -1.1087 | 2.16343 | 0.07659 | 1 | -0.3339 | -1.2604 | 2.57773 | 0.00912 | 0 | -0.0526 | -1.0371 | 1.19474 | 0.25017 | 0 | 0 |
| 1384989_at | Plekhn1 | pleckstrin homology domain containing, family N m | uniqAntib_1098 | -0.15 | -0.08 | -0.13 | -0.37 | -0.13 | 0 | -0.0464 | -1.0327 | 1.13003 | 0.3222 | 1 | -0.2863 | -1.2195 | 1.44619 | 0.04594 | 0 | -0.0487 | -1.0343 | 1.03111 | 0.38424 | 0 | 0 |
| 1384995_at | Jrk | jerky homolog (mouse) | uniqAntib_1098 | -0.15 | 0.01 | 0.13 | -0.39 | -0.05 | 0 | 0.11923 | 1.08615 | 1.19279 | 0.28751 | 1 | -0.4046 | -1.3237 | 3.01561 | 0.00553 | 0 | -0.0621 | -1.044 | 1.34041 | 0.17766 | 0 | 0 |
| 1385030_at | --- | --- | uniqAntib_1098 | -0.02 | 0.05 | 0.06 | 0.35 | 0.23 | 0 | 0.01423 | 1.00991 | 0.91069 | 0.58005 | 1 | 0.30323 | 1.2339 | 1.24792 | 0.03499 | 0 | 0.18317 | 1.13537 | 1.63007 | 0.12061 | 0 | 0 |
| 1385035_at | Usp12 | ubiquitin specific peptidase 12 | uniqAntib_1098 | 0.00 | -0.07 | -0.07 | 0.23 | 0.10 | 0 | 0.00254 | 1.00176 | 1.29773 | 0.2321 | 1 | 0.3062 | 1.23645 | 1.17584 | 0.02488 | 0 | 0.17239 | 1.12693 | 2.0771 | 0.05697 | 0 | 0 |
| 1385064_at | Lao1 | L-amino acid oxidase 1 | uniqAntib_1098 | -0.08 | 0.04 | 0.04 | -0.30 | -0.05 | 0 | 0.00066 | 1.00046 | 0.96795 | 0.45439 | 1 | -0.3421 | -1.2676 | 2.11105 | 0.01358 | 0 | -0.0927 | -1.0663 | 1.22658 | 0.23053 | 0 | 0 |
| 1385070_at | --- | --- | uniqAntib_1098 | -0.09 | -0.08 | -0.09 | 0.19 | -0.07 | 0 | -0.009 | -1.0063 | 1.22459 | 0.26808 | 1 | 0.27211 | 1.20757 | 1.25274 | 0.03569 | 0 | 0.00896 | 1.00623 | 1.18608 | 0.27849 | 0 | 0 |
| 1385076_at | LOC10090987 | zinc finger BED domain-containing protein 6-like | uniqAntib_1098 | -0.05 | -0.25 | -0.02 | 0.08 | -0.22 | 0 | 0.2299 | 1.17275 | 2.41438 | 0.00497 | 1 | 0.32291 | 1.25085 | 0.71919 | 0.00546 | 0 | 0.02083 | 1.01454 | 1.89265 | 0.07492 | 0 | 0 |
| 1385093_at | RGD1565033 | similar to hypothetical protein LOC284018 isoform I | uniqAntib_1098 | 0.13 | 0.21 | 0.09 | -0.06 | -0.04 | 0 | -0.1221 | -1.0883 | 1.37639 | 0.21178 | 1 | -0.2686 | -1.2046 | 1.6825 | 0.02775 | 0 | -0.2529 | -1.1916 | 1.9794 | 0.05238 | 0 | 0 |
| 1385117_at | --- | --- | uniqAntib_1098 | 0.01 | 0.10 | 0.21 | -0.21 | -0.08 | 0 | 0.10849 | 1.0781 | 1.37843 | 0.20323 | 1 | -0.3084 | -1.2383 | 1.61685 | 0.03281 | 0 | -0.1812 | -1.1338 | 1.54256 | 0.11334 | 0 | 0 |
| 1385158_at | Chmp1b | charged multivesicular body protein 1B | uniqAntib_1098 | -0.16 | -0.42 | -0.41 | -0.15 | -0.45 | 0 | 0.00784 | 1.00545 | 1.29087 | 0.23598 | 1 | 0.26862 | 1.20466 | 1.25984 | 0.03804 | 0 | -0.0257 | -1.018 | 1.15289 | 0.27511 | 0 | 0 |
| 1385208_at | RGD1305014 | similar to RIKEN cDNA 2310057M21 | uniqAntib_1098 | -0.13 | -0.34 | -0.26 | -0.06 | -0.26 | 0 | 0.07575 | 1.05391 | 0.99677 | 0.45023 | 1 | 0.27925 | 1.21357 | 1.27748 | 0.04174 | 0 | 0.07818 | 1.05569 | 1.02435 | 0.40399 | 0 | 0 |
| 1385229_at | Pcdh20 | protocadherin 20 | uniqAntib_1098 | -0.10 | -0.28 | -0.33 | -0.01 | -0.09 | 0 | -0.0457 | -1.0322 | 1.07273 | 0.35932 | 1 | 0.27465 | 1.2097 | 1.20199 | 0.02694 | 0 | 0.18989 | 1.14068 | 2.08433 | 0.05509 | 0 | 0 |
| 1385290_at | Tmco5a | transmembrane and coiled-coil domains 5A | uniqAntib_1098 | 0.10 | 0.08 | 0.00 | -0.23 | -0.07 | 0 | -0.0877 | -1.0627 | 1.51763 | 0.17295 | 1 | -0.3145 | -1.2436 | 2.03331 | 0.0168 | 0 | -0.1559 | -1.1141 | 1.52896 | 0.11509 | 0 | 0 |
| 1385314_at | --- | --- | uniqAntib_1098 | -0.21 | -0.11 | 0.15 | 0.20 | 0.10 | 0 | 0.25287 | 1.19158 | 1.93892 | 0.05474 | 1 | 0.30341 | 1.23406 | 1.21427 | 0.03046 | 0 | 0.20036 | 1.14898 | 1.77676 | 0.0921 | 0 | 0 |
| 1385423_at | Immt | inner membrane protein, mitochondrial | uniqAntib_1098 | 0.10 | -0.14 | -0.16 | 0.16 | -0.16 | 0 | -0.0211 | -1.0147 | 0.96404 | 0.45897 | 1 | 0.29344 | 1.22556 | 1.26685 | 0.03866 | 0 | -0.0199 | -1.0139 | 0.97902 | 0.46466 | 0 | 0 |
| 1385426_at | Ccdc109b | coiled-coil domain containing 109B | uniqAntib_1098 | -0.29 | -0.87 | -0.72 | -0.39 | -0.76 | 0 | 0.14349 | 1.10457 | 1.77488 | 0.07676 | 1 | 0.48136 | 1.39606 | 0.39209 | 0.00163 | 0 | 0.1087 | 1.07826 | 1.7443 | 0.09591 | 0 | 0 |
| 1385428_at | Xpo4 | exportin 4 | uniqAntib_1098 | -0.05 | -0.29 | -0.15 | 0.05 | -0.20 | 0 | 0.13415 | 1.09744 | 1.62556 | 0.12838 | 1 | 0.33187 | 1.25864 | 1.05562 | 0.01721 | 0 | 0.0825 | 1.05885 | 1.37822 | 0.19021 | 0 | 0 |
| 1385429_at | --- | --- | uniqAntib_1098 | 0.19 | 0.19 | 0.35 | 0.56 | 0.36 | 0 | 0.15334 | 1.11214 | 1.3895 | 0.19637 | 1 | 0.36463 | 1.28755 | 1.00387 | 0.01459 | 0 | 0.16819 | 1.12365 | 1.56084 | 0.13546 | 0 | 0 |
| 1385494_at | Tbc1d23 | TBC1 domain family, member 23 | uniqAntib_1098 | 0.00 | -0.31 | -0.33 | 0.03 | -0.26 | 0 | -0.0267 | -1.0186 | 1.19976 | 0.28197 | 1 | 0.33609 | 1.26233 | 1.06781 | 0.01785 | 0 | 0.04473 | 1.03149 | 1.02593 | 0.40121 | 0 | 0 |
| 1385505_at | --- | --- | uniqAntib_1098 | 0.18 | 0.46 | 0.60 | 1.07 | 0.54 | 0 | 0.13694 | 1.09957 | 1.7586 | 0.08494 | 1 | 0.60596 | 1.52199 | 0.05 | 9.8E-05 | 0 | 0.08052 | 1.0574 | 2.09175 | 0.05314 | 0 | 0 |
| 1385507_at | --- | --- | uniqAntib_1098 | 0.09 | 0.03 | 0.04 | -0.27 | -0.18 | 0 | 0.00161 | 1.00112 | 0.91153 | 0.54069 | 1 | -0.3016 | -1.2325 | 1.45291 | 0.04532 | 0 | -0.2105 | -1.1571 | 1.70398 | 0.09205 | 0 | 0 |
| 1385527_at | Usp7 | ubiquitin specific peptidase 7 (herpes virus-associat | uniqAntib_1098 | -0.05 | 0.26 | 0.51 | 0.60 | 0.31 | 0 | 0.24961 | 1.18888 | 2.30635 | 0.01483 | 1 | 0.33646 | 1.26266 | 1.0095 | 0.01493 | 0 | 0.04267 | 1.03002 | 1.29595 | 0.22365 | 0 | 0 |
| 1385551_at | RGD1562652 | similar to class I histocompatibility antigen alpha ch | uniqAntib_1098 | 0.09 | 0.17 | 0.02 | -0.13 | 0.09 | 0 | -0.1524 | -1.1114 | 1.63116 | 0.14927 | 1 | -0.3014 | -1.2323 | 1.51497 | 0.0397 | 0 | -0.0789 | -1.0562 | 1.08308 | 0.32883 | 0 | 0 |
| 1385558_at | Esyt3 | extended synaptotagmin-like protein 3 | uniqAntib_1098 | 0.09 | 0.23 | 0.06 | -0.10 | 0.16 | 0 | -0.1692 | -1.1244 | 2.0891 | 0.08431 | 1 | -0.3296 | -1.2566 | 2.85149 | 0.00614 | 0 | -0.0705 | -1.0501 | 1.48501 | 0.13175 | 0 | 0 |
| 1385570_at | Tmem52 | transmembrane protein 52 | uniqAntib_1098 | 0.22 | 0.16 | 0.05 | -0.13 | 0.10 | 0 | -0.1142 | -1.0824 | 2.62868 | 0.04218 | 1 | -0.2889 | -1.2217 | 3.32143 | 0.00075 | 0 | -0.0615 | -1.0436 | 1.76475 | 0.08103 | 0 | 0 |
| 1385588_at | Cabp4 | calcium binding protein 4 | uniqAntib_1098 | 0.05 | 0.27 | 0.08 | -0.04 | 0.09 | 0 | -0.1978 | -1.147 | 2.58344 | 0.04635 | 1 | -0.3169 | -1.2457 | 2.04714 | 0.01659 | 0 | -0.1839 | -1.136 | 1.87734 | 0.06345 | 0 | 0 |
| 1385600_at | --- | --- | uniqAntib_1098 | -0.08 | -0.06 | -0.29 | -0.40 | -0.23 | 0 | -0.2314 | -1.174 | 2.56058 | 0.04718 | 1 | -0.3425 | -1.268 | 1.88262 | 0.01961 | 0 | -0.1634 | -1.1199 | 1.6214 | 0.10088 | 0 | 0 |
| 1385625_at | Efemp2 | EGF-containing fibulin-like extracellular matrix prote | uniqAntib_1098 | -0.05 | -0.08 | 0.05 | -0.35 | -0.10 | 0 | 0.12588 | 1.09117 | 1.56759 | 0.13842 | 1 | -0.2674 | -1.2036 | 1.72198 | 0.02658 | 0 | -0.0168 | -1.0117 | 1.10203 | 0.31467 | 0 | 0 |
| 1385626_at | MGC95152 | similar to B230212L03Rik protein | uniqAntib_1098 | -0.09 | 0.02 | 0.26 | 0.31 | 0.14 | 0 | 0.24766 | 1.18728 | 2.30811 | 0.01885 | 1 | 0.29551 | 1.22732 | 1.05297 | 0.0173 | 0 | 0.12816 | 1.0929 | 1.84796 | 0.08058 | 0 | 0 |
| 1385687_at | Fam25c | family with sequence similarity 25, member C | uniqAntib_1098 | 0.22 | 0.04 | 0.06 | -0.24 | 0.07 | 0 | 0.02385 | 1.01667 | 1.05225 | 0.38924 | 1 | -0.2818 | -1.2157 | 1.65026 | 0.0303 | 0 | 0.03497 | 1.02453 | 0.94479 | 0.50294 | 0 | 0 |
| 1385720_at | Leng1 | leukocyte receptor cluster (LRC) member 1 | uniqAntib_1098 | 0.14 | 0.17 | 0.23 | 0.52 | 0.43 | 0 | 0.05953 | 1.04213 | 1.23101 | 0.26398 | 1 | 0.3507 | 1.27518 | 0.95845 | 0.01156 | 0 | 0.26071 | 1.19806 | 2.31987 | 0.03931 | 0 | 0 |
| 1385760_at | --- | --- | uniqAntib_1098 | 0.00 | 0.19 | 0.15 | -0.08 | 0.07 | 0 | -0.041 | -1.0288 | 1.2603 | 0.25349 | 1 | -0.267 | -1.2033 | 1.46558 | 0.04449 | 0 | -0.1162 | -1.0838 | 1.25134 | 0.21925 | 0 | 0 |
| 1385781_at | Ercc6l | excision repair cross-complementing rodent repair ( | uniqAntib_1098 | -0.05 | -0.20 | -0.24 | 0.07 | -0.26 | 0 | -0.0407 | -1.0286 | 1.01724 | 0.40861 | 1 | 0.26951 | 1.2054 | 1.20573 | 0.02954 | 0 | -0.0595 | -1.0421 | 1.10834 | 0.30924 | 0 | 0 |

| Affymetrix Probeset ID | Gene Symbol | Gene Title | Figure 6 B Venn n =2021 | Mean BN log2 intensity autologous n=8 | Mean log2 intensity day 30 DR +/+ ND n=4 | Mean log2 intensity day 30 DR+/+ HCD n=4 | Mean log2 intensity day 30 B/S n=4 | Mean log2 intensity day 30 F+/+ ND n=4 | DR+/+ HCD vs DR+/+ ND n=380 1=yes 0=no | Log2 ratio DR+/+ HCD vs DR+/+ ND | Fold-Change DR+/+ HCD vs DR+/+ ND | RP-FDR 4DR30 HCD vs 4DR30 Reg | p value DR+/+ HCD vs DR+/+ ND | DR+/+ B/S vs DR+/+ ND n=1,330 1=yes 0=no | Log2 ratio DR+/+ B/S vs DR+/+ ND | Fold-Change DR+/+ B/S vs DR+/+ ND | RP-FDR 4DR30 Antib vs 4DR30 Reg | p value DR+/+ B/S vs DR+/+ ND | DR+/+ ND vs F+/+ ND n=398 1=yes 0=no | Log2 ratio DR+/+ ND vs F+/+ ND | Fold-Change DR+/+ ND vs F+/+ ND | RP-FDR 4RT1uu 30Reg vs 4DR30 Reg | p value DR+/+ ND vs F+/+ ND | DR+/+ Longitudinal n=228 1=yes 0=no | Figure 6F |
|---|---|---|---|---|---|---|---|---|---|---|---|---|---|---|---|---|---|---|---|---|---|---|---|---|---|
| 1385785_at | Rhox4g | reproductive homeobox 4G | uniqAntib_1098 | 0.00 | 0.02 | -0.06 | -0.27 | 0.00 | 0 | -0.0757 | -1.0539 | 1.2095 | 0.27757 | 1 | -0.2848 | -1.2183 | 1.48422 | 0.04295 | 0 | -0.0153 | -1.0106 | 0.91437 | 0.60274 | 0 | 0 |
| 1385796_at | --- | --- | uniqAntib_1098 | 0.12 | -0.04 | -0.05 | -0.30 | -0.25 | 0 | -0.0145 | -1.0101 | 0.97749 | 0.44441 | 1 | -0.2639 | -1.2007 | 2.27838 | 0.01172 | 0 | -0.2163 | -1.1618 | 2.21237 | 0.03664 | 0 | 0 |
| 1385809_at | Fastkd5 | FAST kinase domains 5 | uniqAntib_1098 | -0.14 | -0.31 | -0.27 | 0.00 | -0.28 | 0 | 0.04292 | 1.0302 | 1.76448 | 0.07983 | 1 | 0.31093 | 1.24051 | 1.16631 | 0.02348 | 0 | 0.03785 | 1.02658 | 1.18712 | 0.27725 | 0 | 0 |
| 1385862_at | Armc9 | armadillo repeat containing 9 | uniqAntib_1098 | -0.23 | 0.18 | -0.08 | -0.09 | 0.19 | 0 | -0.2587 | -1.1964 | 3.18155 | 0.02251 | 1 | -0.2703 | -1.2061 | 1.56069 | 0.03513 | 0 | 0.00582 | 1.00404 | 1.01368 | 0.41732 | 0 | 0 |
| 1385892_at | --- | --- | uniqAntib_1098 | -0.05 | 0.16 | 0.23 | 0.50 | 0.14 | 0 | 0.06602 | 1.04683 | 1.14248 | 0.31954 | 1 | 0.33373 | 1.26027 | 1.14 | 0.02207 | 0 | -0.027 | -1.0189 | 1.0272 | 0.38807 | 0 | 0 |
| 1385903_at | Zswim6 | zinc finger, SWIM-type containing 6 | uniqAntib_1098 | -0.05 | 0.15 | 0.35 | 0.60 | 0.38 | 0 | 0.1985 | 1.1475 | 1.67046 | 0.11398 | 1 | 0.44474 | 1.36107 | 0.71 | 0.0053 | 0 | 0.22532 | 1.16904 | 1.92927 | 0.0701 | 0 | 0 |
| 1385931_at | Hook3 | hook homolog 3 (Drosophila) | uniqAntib_1098 | -0.19 | -0.45 | -0.35 | -0.15 | -0.34 | 0 | 0.10455 | 1.07516 | 2.3104 | 0.01493 | 1 | 0.29852 | 1.22988 | 0.70658 | 0.00504 | 0 | 0.10798 | 1.07772 | 2.31157 | 0.0411 | 0 | 0 |
| 1385965_at | Ankhd1 | ankyrin repeat and KH domain containing 1 | uniqAntib_1098 | -0.18 | -0.19 | -0.02 | 0.22 | -0.21 | 0 | 0.17644 | 1.13009 | 2.09033 | 0.03267 | 1 | 0.41763 | 1.33573 | 0.68732 | 0.00486 | 0 | -0.0212 | -1.0148 | 1.21666 | 0.23587 | 0 | 0 |
| 1386043_at | --- | --- | uniqAntib_1098 | 0.25 | 0.21 | 0.05 | -0.12 | 0.00 | 0 | -0.167 | -1.1227 | 1.56358 | 0.16255 | 1 | -0.3347 | -1.2611 | 1.79229 | 0.02236 | 0 | -0.2081 | -1.1552 | 2.03746 | 0.04855 | 0 | 0 |
| 1386071_at | Srsf10 | serine/arginine-rich splicing factor 10 | uniqAntib_1098 | -0.08 | -0.09 | -0.15 | 0.27 | -0.03 | 0 | -0.0584 | -1.0413 | 1.67802 | 0.13953 | 1 | 0.36497 | 1.28785 | 0.97484 | 0.0136 | 0 | 0.06544 | 1.0464 | 1.1631 | 0.29288 | 0 | 0 |
| 1386258_at | Rnpc3 | RNA-binding region (RNP1, RRM) containing 3 | uniqAntib_1098 | -0.25 | -0.51 | -0.26 | -0.19 | -0.35 | 0 | 0.25503 | 1.19336 | 2.28957 | 0.01524 | 1 | 0.31669 | 1.24547 | 0.96785 | 0.01329 | 0 | 0.16532 | 1.12142 | 2.36776 | 0.03266 | 0 | 0 |
| 1386266_at | --- | --- | uniqAntib_1098 | -0.13 | -0.27 | -0.16 | -0.01 | -0.23 | 0 | 0.1107 | 1.07975 | 1.33031 | 0.2188 | 1 | 0.26581 | 1.20231 | 1.27758 | 0.04531 | 0 | 0.03993 | 1.02807 | 1.12984 | 0.31273 | 0 | 0 |
| 1386321_s_at | Trib3 | tribbles homolog 3 (Drosophila) | uniqAntib_1098 | 0.06 | 0.07 | 0.13 | 0.35 | 0.24 | 0 | 0.05666 | 1.04005 | 1.03826 | 0.40914 | 1 | 0.28149 | 1.21545 | 1.26143 | 0.03894 | 0 | 0.17143 | 1.12618 | 1.60476 | 0.12555 | 0 | 0 |
| 1386358_at | --- | --- | uniqAntib_1098 | -0.14 | -0.20 | -0.21 | -0.51 | -0.32 | 0 | -0.0077 | -1.0053 | 1.09457 | 0.34366 | 1 | -0.3043 | -1.2348 | 2.07213 | 0.01439 | 0 | -0.12 | -1.0867 | 1.40322 | 0.15598 | 0 | 0 |
| 1386401_at | --- | --- | uniqAntib_1098 | 0.04 | 0.15 | -0.10 | -0.14 | 0.04 | 0 | -0.2515 | -1.1904 | 2.60498 | 0.04557 | 1 | -0.2889 | -1.2217 | 1.43319 | 0.04862 | 0 | -0.1154 | -1.0833 | 1.28805 | 0.19914 | 0 | 0 |
| 1386403_at | --- | --- | uniqAntib_1098 | -0.17 | 0.02 | -0.17 | -0.28 | -0.09 | 0 | -0.1895 | -1.1403 | 2.06712 | 0.08727 | 1 | -0.2967 | -1.2283 | 1.50794 | 0.04189 | 0 | -0.1036 | -1.0744 | 1.1016 | 0.31526 | 0 | 0 |
| 1386477_at | --- | --- | uniqAntib_1098 | -0.18 | -0.20 | -0.19 | 0.06 | -0.26 | 0 | 0.01822 | 1.01271 | 1.08346 | 0.36261 | 1 | 0.26358 | 1.20046 | 1.26572 | 0.03838 | 0 | -0.0564 | -1.0399 | 1.11469 | 0.30556 | 0 | 0 |
| 1386658_at | Gpr45 | G protein-coupled receptor 45 | uniqAntib_1098 | 0.13 | -0.03 | -0.07 | -0.31 | 0.03 | 0 | -0.0424 | -1.0299 | 0.99157 | 0.43168 | 1 | -0.2811 | -1.2151 | 1.47835 | 0.04373 | 0 | 0.05975 | 1.04228 | 0.99021 | 0.44074 | 0 | 0 |
| 1386811_at | Ptplad2 | protein tyrosine phosphatase-like A domain contain | uniqAntib_1098 | 0.00 | -0.81 | -0.82 | -0.52 | -0.78 | 0 | -0.0037 | -1.0026 | 1.99662 | 0.04372 | 1 | 0.29493 | 1.22682 | 0.80078 | 0.00729 | 0 | 0.03548 | 1.0249 | 1.47755 | 0.15555 | 0 | 0 |
| 1386857_at | RGD1565395 | similar to stathmin 1/oncoprotein 18) /// stathmin 1 | uniqAntib_1098 | -0.05 | -0.24 | -0.15 | 0.05 | -0.10 | 0 | 0.09166 | 1.0656 | 1.22037 | 0.26927 | 1 | 0.29246 | 1.22473 | 1.26263 | 0.03975 | 0 | 0.13983 | 1.10178 | 1.51508 | 0.14698 | 0 | 0 |
| 1386873_at | Tnni1 | troponin I type 1 (skeletal, slow) | uniqAntib_1098 | 0.00 | 0.12 | 0.16 | -0.20 | 0.12 | 0 | 0.04272 | 1.03005 | 1.34923 | 0.21228 | 1 | -0.3181 | -1.2467 | 1.91473 | 0.01915 | 0 | 4.7E-05 | 1.00003 | 1.05703 | 0.35505 | 0 | 0 |
| 1386901_at | Cd36 /// LOC | CD36 molecule (thrombospondin receptor) /// scav | uniqAntib_1098 | -0.04 | -0.41 | -0.35 | -0.14 | -0.38 | 0 | 0.05516 | 1.03898 | 1.30862 | 0.22634 | 1 | 0.26737 | 1.20361 | 1.27111 | 0.04067 | 0 | 0.02083 | 1.01455 | 1.08852 | 0.34337 | 0 | 0 |
| 1386908_at | Glrx | glutaredoxin (thioltransferase) | uniqAntib_1098 | -0.18 | -0.64 | -0.58 | -0.27 | -0.45 | 0 | 0.05531 | 1.03908 | 1.37492 | 0.20377 | 1 | 0.3713 | 1.29352 | 0.81997 | 0.00765 | 0 | 0.18545 | 1.13717 | 1.87577 | 0.07739 | 0 | 0 |
| 1386940_at | Timp2 | TIMP metallopeptidase inhibitor 2 | uniqAntib_1098 | 0.00 | -0.27 | -0.35 | 0.03 | -0.10 | 0 | -0.0808 | -1.0576 | 1.8288 | 0.11985 | 1 | 0.30311 | 1.2338 | 0.95485 | 0.01127 | 0 | 0.17613 | 1.12985 | 2.27866 | 0.0422 | 0 | 0 |
| 1386967_at | LOC10091171 | rho-related GTP-binding protein RhoQ-like /// ras h | uniqAntib_1098 | 0.04 | 0.12 | 0.29 | 0.42 | 0.34 | 0 | 0.17122 | 1.12601 | 1.76386 | 0.07884 | 1 | 0.30678 | 1.23695 | 1.08246 | 0.01897 | 0 | 0.21923 | 1.16411 | 2.22397 | 0.04562 | 0 | 0 |
| 1387010_s_at | Scn1b | sodium channel, voltage-gated, type I, beta | uniqAntib_1098 | -0.11 | -0.05 | -0.25 | -0.32 | -0.29 | 0 | -0.1939 | -1.1438 | 2.10036 | 0.0824 | 1 | -0.2687 | -1.2047 | 1.50386 | 0.04226 | 0 | -0.2433 | -1.1837 | 2.49953 | 0.02877 | 0 | 0 |
| 1387057_at | Slc7a8 | solute carrier family 7 (amino acid transporter light | uniqAntib_1098 | 0.06 | 0.11 | 0.08 | -0.21 | 0.14 | 0 | -0.0329 | -1.0231 | 0.92054 | 0.52256 | 1 | -0.3174 | -1.2461 | 1.65975 | 0.02989 | 0 | 0.03344 | 1.02345 | 0.9167 | 0.56448 | 0 | 0 |
| 1387074_at | Rgs2 | regulator of G-protein signaling 2 | uniqAntib_1098 | -0.02 | -0.46 | -0.44 | -0.18 | -0.31 | 0 | 0.01622 | 1.01131 | 1.04647 | 0.40063 | 1 | 0.27986 | 1.21407 | 1.27692 | 0.04176 | 0 | 0.15501 | 1.11343 | 1.52153 | 0.14614 | 0 | 0 |
| 1387116_at | Dnajb9 | DnaJ (Hsp40) homolog, subfamily B, member 9 | uniqAntib_1098 | -0.02 | 0.06 | -0.01 | 0.38 | 0.11 | 0 | -0.0651 | -1.0462 | 0.97448 | 0.44677 | 1 | 0.32099 | 1.24919 | 1.1804 | 0.02486 | 0 | 0.05607 | 1.03963 | 0.99284 | 0.43817 | 0 | 0 |
| 1387137_at | Comp | cartilage oligomeric matrix protein | uniqAntib_1098 | -0.02 | 0.15 | 0.14 | -0.13 | -0.03 | 0 | -0.0062 | -1.0043 | 1.1928 | 0.28548 | 1 | -0.2827 | -1.2164 | 1.78824 | 0.02248 | 0 | -0.1776 | -1.131 | 1.93141 | 0.05832 | 0 | 0 |
| 1387150_at | Padi1 | peptidyl arginine deiminase, type I | uniqAntib_1098 | 0.10 | 0.11 | 0.08 | -0.21 | 0.03 | 0 | -0.0243 | -1.017 | 1.18726 | 0.28858 | 1 | -0.312 | -1.2415 | 1.79393 | 0.02204 | 0 | -0.071 | -1.0505 | 1.08505 | 0.3279 | 0 | 0 |
| 1387180_at | Il1r2 | interleukin 1 receptor, type II | uniqAntib_1098 | -0.09 | -0.72 | -0.83 | -0.36 | -0.61 | 0 | -0.1092 | -1.0787 | 2.53283 | 0.05001 | 1 | 0.36088 | 1.28421 | 0.59451 | 0.00369 | 0 | 0.10875 | 1.0783 | 2.37235 | 0.03143 | 0 | 0 |
| 1387206_at | B4galt6 | UDP-Gal:betaGlcNAc beta 1,4-galactosyltransferase | uniqAntib_1098 | 0.04 | 0.05 | -0.09 | 0.40 | 0.29 | 0 | -0.1378 | -1.1003 | 1.36945 | 0.21423 | 1 | 0.35004 | 1.2746 | 0.97052 | 0.01258 | 0 | 0.23736 | 1.17883 | 2.17499 | 0.04749 | 0 | 0 |
| 1387226_at | Ina | internexin neuronal intermediate filament protein, | uniqAntib_1098 | -0.16 | 0.00 | -0.24 | -0.29 | -0.01 | 0 | -0.2459 | -1.1858 | 2.71139 | 0.03827 | 1 | -0.2968 | -1.2284 | 1.75702 | 0.02429 | 0 | -0.014 | -1.0098 | 0.98032 | 0.4636 | 0 | 0 |
| 1387230_at | Slc12a3 | solute carrier family 12 (sodium/chloride transporte | uniqAntib_1098 | 0.04 | 0.06 | 0.18 | -0.21 | 0.23 | 0 | 0.1129 | 1.0814 | 1.16431 | 0.30565 | 1 | -0.2778 | -1.2123 | 1.47282 | 0.0439 | 0 | 0.1634 | 1.11993 | 1.3766 | 0.19047 | 0 | 0 |
| 1387243_at | Cyp1a2 | cytochrome P450, family 1, subfamily a, polypeptide | uniqAntib_1098 | -0.04 | -0.06 | -0.14 | -0.38 | -0.05 | 0 | -0.0759 | -1.054 | 1.27063 | 0.24919 | 1 | -0.3228 | -1.2507 | 1.69982 | 0.02684 | 0 | 0.01138 | 1.00792 | 1.06509 | 0.36684 | 0 | 0 |
| 1387245_at | Lipf | lipase, gastric | uniqAntib_1098 | -0.04 | 0.01 | -0.05 | -0.26 | 0.12 | 0 | -0.0588 | -1.0416 | 1.33672 | 0.22506 | 1 | -0.2725 | -1.2079 | 1.60836 | 0.03336 | 0 | 0.10523 | 1.07567 | 1.4351 | 0.17194 | 0 | 0 |
| 1387274_at | Dlx5 | distal-less homeobox 5 | uniqAntib_1098 | 0.09 | 0.20 | 0.00 | -0.09 | 0.05 | 0 | -0.2039 | -1.1518 | 2.04024 | 0.0885 | 1 | -0.2916 | -1.224 | 1.71447 | 0.02663 | 0 | -0.1462 | -1.1067 | 1.3642 | 0.16989 | 0 | 0 |
| 1387313_at | Myoc | myocilin, trabecular meshwork inducible glucocortic | uniqAntib_1098 | 0.01 | 0.03 | -0.08 | -0.28 | 0.01 | 0 | -0.1105 | -1.0796 | 1.17628 | 0.29563 | 1 | -0.3054 | -1.2358 | 1.54502 | 0.03811 | 0 | -0.0185 | -1.0129 | 0.92807 | 0.55614 | 0 | 0 |
| 1387322_at | Sema6b | sema domain, transmembrane domain (TM), and cy | uniqAntib_1098 | 0.04 | 0.02 | -0.08 | -0.28 | -0.10 | 0 | -0.1 | -1.0718 | 1.07885 | 0.35298 | 1 | -0.299 | -1.2303 | 1.70125 | 0.02713 | 0 | -0.1185 | -1.0856 | 1.1184 | 0.30029 | 0 | 0 |
| 1387334_at | Tpsb2 | tryptase beta 2 | uniqAntib_1098 | 0.02 | 0.00 | -0.07 | -0.33 | -0.16 | 0 | -0.066 | -1.0468 | 1.15971 | 0.30377 | 1 | -0.3281 | -1.2554 | 2.16226 | 0.01231 | 0 | -0.1635 | -1.12 | 1.54996 | 0.11189 | 0 | 0 |
| 1387347_at | Igfbp5 | insulin-like growth factor binding protein 5 | uniqAntib_1098 | 0.13 | -0.05 | -0.09 | -0.38 | -0.24 | 0 | -0.041 | -1.0288 | 2.0196 | 0.0915 | 1 | -0.3311 | -1.258 | 3.56342 | 0.00435 | 0 | -0.1956 | -1.1452 | 2.71318 | 0.02329 | 0 | 0 |
| 1387387_at | Hpca | hippocalcin | uniqAntib_1098 | 0.08 | 0.06 | 0.00 | -0.22 | 0.00 | 0 | -0.0639 | -1.0453 | 1.10824 | 0.33345 | 1 | -0.2818 | -1.2157 | 1.7788 | 0.02385 | 0 | -0.0633 | -1.0449 | 1.04031 | 0.37178 | 0 | 0 |
| 1387428_at | Cacnb1 | calcium channel, voltage-dependent, beta 1 subunit | uniqAntib_1098 | 0.01 | 0.07 | -0.10 | -0.21 | -0.09 | 0 | -0.1696 | -1.1247 | 2.33615 | 0.0652 | 1 | -0.2792 | -1.2135 | 1.50435 | 0.04199 | 0 | -0.1581 | -1.1158 | 1.47632 | 0.13411 | 0 | 0 |
| 1387443_at | Nacc1 | nucleus accumbens associated 1, BEN and BTB (POZ | uniqAntib_1098 | 0.08 | 0.24 | 0.02 | -0.09 | 0.07 | 0 | -0.225 | -1.1687 | 2.39884 | 0.06163 | 1 | -0.3314 | -1.2582 | 2.50325 | 0.00966 | 0 | -0.1696 | -1.1247 | 1.69715 | 0.09321 | 0 | 0 |
| 1387459_at | Pkib | protein kinase (cAMP-dependent, catalytic) inhibito | uniqAntib_1098 | -0.13 | -0.77 | -0.70 | -0.49 | -0.69 | 0 | 0.06936 | 1.04925 | 1.45052 | 0.17458 | 1 | 0.27859 | 1.21301 | 1.25609 | 0.03615 | 0 | 0.08543 | 1.061 | 1.2796 | 0.23272 | 0 | 0 |
| 1387492_at | Slco2a1 | solute carrier organic anion transporter family, men | uniqAntib_1098 | 0.00 | 0.05 | -0.20 | -0.32 | -0.12 | 0 | -0.2519 | -1.1908 | 3.13419 | 0.02308 | 1 | -0.3699 | -1.2922 | 2.57609 | 0.00953 | 0 | -0.1692 | -1.1244 | 1.57466 | 0.10922 | 0 | 0 |
| 1387496_a_at | Cnga3 | cyclic nucleotide gated channel alpha 3 | uniqAntib_1098 | 0.07 | 0.09 | 0.09 | -0.23 | -0.01 | 0 | 0.00249 | 1.00173 | 1.07757 | 0.35367 | 1 | -0.3193 | -1.2478 | 1.74183 | 0.02476 | 0 | -0.0974 | -1.0698 | 1.06454 | 0.34754 | 0 | 0 |
| 1387546_at | Cdk12 | cyclin-dependent kinase 12 | uniqAntib_1098 | -0.14 | 0.15 | 0.24 | 0.52 | 0.17 | 0 | 0.09155 | 1.06551 | 2.08835 | 0.02686 | 1 | 0.36541 | 1.28825 | 0.56858 | 0.00296 | 0 | 0.01555 | 1.01083 | 2.27478 | 0.04191 | 0 | 0 |
| 1387566_at | Pla2g4a | phospholipase A2, group IVA (cytosolic, calcium-dep | uniqAntib_1098 | 0.04 | -0.10 | -0.06 | 0.23 | 0.13 | 0 | 0.04163 | 1.02928 | 1.19995 | 0.28248 | 1 | 0.32795 | 1.25522 | 1.13213 | 0.02137 | 0 | 0.22868 | 1.17176 | 2.27522 | 0.03687 | 0 | 0 |
| 1387572_at | Psd | pleckstrin and Sec7 domain containing | uniqAntib_1098 | 0.08 | 0.02 | 0.14 | -0.34 | -0.04 | 0 | 0.12121 | 1.08764 | 1.62897 | 0.12451 | 1 | -0.3557 | -1.2796 | 2.52186 | 0.00957 | 0 | -0.059 | -1.0417 | 1.23646 | 0.22527 | 0 | 0 |
| 1387589_at | Rims3 | regulating synaptic membrane exocytosis 3 | uniqAntib_1098 | -0.06 | 0.14 | 0.06 | -0.21 | -0.05 | 0 | -0.0829 | -1.0591 | 1.15186 | 0.30731 | 1 | -0.3531 | -1.2773 | 2.78973 | 0.00673 | 0 | -0.1924 | -1.1427 | 2.88459 | 0.02059 | 0 | 0 |
| 1387645_at | Ucn | urocortin | uniqAntib_1098 | 0.06 | 0.13 | 0.12 | -0.15 | 0.06 | 0 | -0.0015 | -1.001 | 0.95804 | 0.46594 | 1 | -0.2769 | -1.2116 | 1.66291 | 0.02871 | 0 | -0.0694 | -1.0493 | 1.02656 | 0.38912 | 0 | 0 |
| 1387673_a_at | Anxa6 | annexin A6 | uniqAntib_1098 | 0.12 | 0.28 | 0.24 | -0.01 | 0.26 | 0 | -0.0376 | -1.0264 | 1.01157 | 0.41358 | 1 | -0.2921 | -1.2245 | 1.52146 | 0.03919 | 0 | -0.0225 | -1.0157 | 0.92271 | 0.57347 | 0 | 0 |
| 1387674_at | Cnr1 | cannabinoid receptor 1 (brain) | uniqAntib_1098 | 0.13 | 0.00 | 0.04 | -0.29 | -0.17 | 0 | 0.04488 | 1.0316 | 1.23394 | 0.26132 | 1 | -0.2894 | -1.2221 | 1.58607 | 0.03386 | 0 | -0.1674 | -1.123 | 1.93793 | 0.05664 | 0 | 0 |
| 1387683_at | Crhr1 | corticotropin releasing hormone receptor 1 | uniqAntib_1098 | 0.10 | 0.08 | -0.04 | -0.22 | 0.02 | 0 | -0.1203 | -1.087 | 1.29785 | 0.24021 | 1 | -0.295 | -1.2269 | 1.48301 | 0.04273 | 0 | -0.0603 | -1.0427 | 1.05809 | 0.35415 | 0 | 0 |
| 1387714_at | Crem | cAMP responsive element modulator | uniqAntib_1098 | -0.07 | -0.66 | -0.68 | -0.35 | -0.57 | 0 | -0.0122 | -1.0085 | 0.9972 | 0.42705 | 1 | 0.31481 | 1.24385 | 1.20043 | 0.02683 | 0 | 0.09315 | 1.0667 | 1.24974 | 0.24614 | 0 | 0 |
| 1387717_at | Plcb2 | phospholipase C, beta 2 | uniqAntib_1098 | 0.01 | 0.16 | 0.11 | -0.19 | -0.04 | 0 | -0.0496 | -1.035 | 1.13596 | 0.31684 | 1 | -0.3435 | -1.2688 | 2.25652 | 0.0119 | 0 | -0.1979 | -1.147 | 1.76621 | 0.08093 | 0 | 0 |
| 1387720_at | Clstn2 | calsyntenin 2 | uniqAntib_1098 | 0.07 | 0.16 | 0.06 | -0.16 | -0.02 | 0 | -0.1028 | -1.0739 | 1.37431 | 0.21278 | 1 | -0.324 | -1.2518 | 2.19628 | 0.01271 | 0 | -0.1803 | -1.1331 | 1.94992 | 0.05637 | 0 | 0 |
| 1387762_s_at | Jund | jun D proto-oncogene | uniqAntib_1098 | 0.16 | 0.16 | 0.37 | 0.44 | 0.33 | 0 | 0.20845 | 1.15544 | 1.70908 | 0.09865 | 1 | 0.27564 | 1.21053 | 1.27686 | 0.04906 | 0 | 0.16756 | 1.12316 | 1.8081 | 0.08494 | 0 | 0 |
| 1387791_at | Ace | angiotensin I converting enzyme (peptidyl-dipeptida | uniqAntib_1098 | -0.01 | -0.03 | -0.03 | -0.32 | -0.10 | 0 | -0.0041 | -1.0029 | 1.04509 | 0.38119 | 1 | -0.295 | -1.2269 | 1.8054 | 0.02322 | 0 | -0.0741 | -1.0527 | 1.1888 | 0.25428 | 0 | 0 |
| 1387820_at | Klk1c7 | kallikrein 1-related peptidase C7 | uniqAntib_1098 | 0.20 | 0.18 | 0.06 | -0.32 | -0.04 | 0 | -0.1173 | -1.0847 | 2.22555 | 0.07285 | 1 | -0.5006 | -1.4148 | 3.93889 | 0.00114 | 0 | -0.2122 | -1.1585 | 1.83914 | 0.07162 | 0 | 0 |
| 1387841_at | Exoc5 | exocyst complex component 5 | uniqAntib_1098 | -0.07 | -0.02 | -0.04 | 0.27 | 0.03 | 0 | -0.0119 | -1.0083 | 0.98797 | 0.46039 | 1 | 0.29325 | 1.2254 | 1.24596 | 0.03518 | 0 | 0.05768 | 1.04079 | 1.10129 | 0.33397 | 0 | 0 |
| 1387896_at | Scp2 | sterol carrier protein 2 | uniqAntib_1098 | -0.07 | -0.36 | -0.31 | -0.08 | -0.35 | 0 | 0.04989 | 1.03518 | 1.33497 | 0.21751 | 1 | 0.28203 | 1.21591 | 1.26738 | 0.03949 | 0 | 0.01054 | 1.00733 | 0.95608 | 0.48153 | 0 | 0 |
| 1387945_at | --- | --- | uniqAntib_1098 | -0.19 | -0.63 | -0.53 | -0.28 | -0.58 | 0 | 0.10144 | 1.07284 | 1.66745 | 0.11506 | 1 | 0.34774 | 1.27256 | 0.96286 | 0.01167 | 0 | 0.05121 | 1.03613 | 1.03549 | 0.38731 | 0 | 0 |
| 1387973_at | Cyp4f4 | cytochrome P450, family 4, subfamily f, polypeptide | uniqAntib_1098 | -0.12 | -0.04 | -0.14 | -0.31 | -0.23 | 0 | -0.0967 | -1.0693 | 1.74063 | 0.13159 | 1 | -0.268 | -1.2041 | 1.79549 | 0.02229 | 0 | -0.1886 | -1.1397 | 2.02023 | 0.05073 | 0 | 0 |
| 1387982_at | Tlr4 | toll-like receptor 4 | uniqAntib_1098 | -0.13 | -0.16 | -0.36 | 0.23 | -0.22 | 0 | -0.1938 | -1.1438 | 2.05303 | 0.08787 | 1 | 0.39334 | 1.31343 | 0.7226 | 0.00572 | 0 | -0.0604 | -1.0427 | 1.183 | 0.2581 | 0 | 0 |
| 1387986_at | Scnn1g | sodium channel, nonvoltage-gated 1, gamma | uniqAntib_1098 | 0.12 | 0.18 | 0.11 | -0.11 | 0.05 | 0 | -0.072 | -1.0512 | 1.31728 | 0.23254 | 1 | -0.2971 | -1.2287 | 1.64621 | 0.03086 | 0 | -0.1326 | -1.0963 | 1.38525 | 0.16227 | 0 | 0 |
| 1388013_at | Cd80 | Cd80 molecule | uniqAntib_1098 | -0.07 | -0.25 | -0.12 | 0.06 | -0.22 | 0 | 0.13509 | 1.09816 | 1.46932 | 0.16843 | 1 | 0.31211 | 1.24152 | 1.11838 | 0.02025 | 0 | 0.02674 | 1.01871 | 1.00264 | 0.42631 | 0 | 0 |
| 1388048_a_at | Inpp4b | inositol polyphosphate-4-phosphatase, type II | uniqAntib_1098 | -0.04 | -0.24 | -0.05 | 0.24 | -0.36 | 0 | 0.18352 | 1.13565 | 2.21434 | 0.01574 | 1 | 0.47601 | 1.39089 | 0.29636 | 0.00102 | 0 | -0.1205 | -1.0871 | 1.90542 | 0.06207 | 0 | 0 |
| 1388080_a_at | Hrh3 | histamine receptor H3 | uniqAntib_1098 | -0.06 | 0.04 | 0.00 | -0.24 | -0.13 | 0 | -0.0373 | -1.0262 | 1.73898 | 0.13185 | 1 | -0.2813 | -1.2153 | 2.74557 | 0.00697 | 0 | -0.1752 | -1.1291 | 2.24967 | 0.03523 | 0 | 0 |

| Affymetrix Probeset ID | Gene Symbol | Gene Title | Figure 6 B Venn n =2021 | Mean BN log2 intensity autologous n=8 | Mean log2 intensity day 30 DR +/+ ND n=4 | Mean log2 intensity day 30 DR+/+ HCD n=4 | Mean log2 intensity day 30 B/S n=4 | Mean log2 intensity day 30 F+/+ ND n=4 | DR+/+ HCD vs DR+/+ ND n=380 1=yes 0=no | Log2 ratio DR+/+ HCD vs DR+/+ ND | Fold-Change DR+/+ HCD vs DR+/+ ND | RP-FDR 4DR30 HCD vs 4DR30 Reg | p value DR+/+ HCD vs DR+/+ ND | DR+/+ B/S vs DR+/+ ND n=1,330 1=yes 0=no | Log2 ratio DR+/+ B/S vs DR+/+ ND | Fold-Change DR+/+ B/S vs DR+/+ ND | RP-FDR 4DR30 Antib vs 4DR30 Reg | p value DR+/+ B/S vs DR+/+ ND | DR+/+ ND vs F+/+ ND n=398 1=yes 0=no | Log2 ratio DR+/+ ND vs F+/+ ND | Fold-Change DR+/+ ND vs F+/+ ND | RP-FDR 4RT1uu 30Reg vs 4DR30 Reg | p value DR+/+ ND vs F+/+ ND | DR+/+ Longitudinal n=228 1=yes 0=no | Figure 6F |
|---|---|---|---|---|---|---|---|---|---|---|---|---|---|---|---|---|---|---|---|---|---|---|---|---|---|
| 1388101_at | Dpysl3 | dihydropyrimidinase-like 3 | uniqAntib_1098 | -0.19 | 0.04 | -0.20 | -0.28 | -0.21 | 0 | -0.2333 | -1.1755 | 2.1725 | 0.07621 | 1 | -0.3185 | -1.247 | 1.70393 | 0.02674 | 0 | -0.2502 | -1.1893 | 2.06836 | 0.04676 | 0 | 0 |
| 1388140_at | Rab13 | RAB13, member RAS oncogene family | uniqAntib_1098 | 0.05 | 0.04 | -0.09 | -0.35 | -0.19 | 0 | -0.1258 | -1.0911 | 1.62264 | 0.15048 | 1 | -0.3943 | -1.3143 | 3.82667 | 0.00369 | 0 | -0.2328 | -1.1751 | 1.92301 | 0.06017 | 0 | 0 |
| 1388177_at | Ddn | dendrin | uniqAntib_1098 | 0.04 | 0.06 | 0.00 | -0.25 | -0.08 | 0 | -0.0607 | -1.043 | 1.04626 | 0.38057 | 1 | -0.3092 | -1.239 | 1.54402 | 0.03828 | 0 | -0.138 | -1.1004 | 1.31157 | 0.18814 | 0 | 0 |
| 1388198_at | --- | --- | uniqAntib_1098 | -0.17 | 0.03 | 0.14 | 0.32 | 0.05 | 0 | 0.11002 | 1.07924 | 1.4396 | 0.17785 | 1 | 0.29788 | 1.22934 | 1.27866 | 0.04227 | 0 | 0.02064 | 1.01441 | 1.0181 | 0.4121 | 0 | 0 |
| 1388216_at | Zfp423 | zinc finger protein 423 | uniqAntib_1098 | -0.08 | -0.02 | -0.04 | -0.32 | -0.07 | 0 | -0.0141 | -1.0098 | 0.9645 | 0.45823 | 1 | -0.2985 | -1.2299 | 1.73831 | 0.02644 | 0 | -0.0475 | -1.0335 | 1.17666 | 0.26194 | 0 | 0 |
| 1388230_at | Ajuba | ajuba LIM protein | uniqAntib_1098 | 0.00 | 0.25 | 0.15 | -0.01 | 0.08 | 0 | -0.101 | -1.0725 | 2.10062 | 0.08193 | 1 | -0.2646 | -1.2013 | 2.0243 | 0.01712 | 0 | -0.1769 | -1.1304 | 2.11203 | 0.04442 | 0 | 0 |
| 1388238_at | Defa24 | defensin, alpha, 24 | uniqAntib_1098 | 0.05 | 0.11 | -0.09 | -0.18 | 0.04 | 0 | -0.2035 | -1.1515 | 1.91883 | 0.10736 | 1 | -0.2887 | -1.2215 | 1.6161 | 0.03186 | 0 | -0.0662 | -1.0469 | 1.01125 | 0.41118 | 0 | 0 |
| 1388241_at | Insl3 | insulin-like 3 | uniqAntib_1098 | 0.11 | 0.13 | -0.06 | -0.23 | -0.02 | 0 | -0.1852 | -1.1369 | 1.88158 | 0.11193 | 1 | -0.3596 | -1.2831 | 2.235 | 0.01207 | 0 | -0.1533 | -1.1121 | 1.34404 | 0.17659 | 0 | 0 |
| 1388257_at | Lrrc7 | leucine rich repeat containing 7 | uniqAntib_1098 | -0.03 | 0.04 | -0.15 | -0.23 | -0.09 | 0 | -0.1901 | -1.1408 | 2.46149 | 0.05406 | 1 | -0.2699 | -1.2057 | 1.83991 | 0.02047 | 0 | -0.1328 | -1.0964 | 1.92395 | 0.06013 | 0 | 0 |
| 1388282_s_at | Klra5 /// LOC | killer cell lectin-like receptor, subfamily A, member | uniqAntib_1098 | -0.01 | -0.34 | -0.20 | 0.01 | -0.23 | 0 | 0.1433 | 1.10443 | 1.9043 | 0.0597 | 1 | 0.35136 | 1.27576 | 0.96601 | 0.01333 | 0 | 0.11749 | 1.08485 | 1.59243 | 0.1276 | 0 | 0 |
| 1388292_at | Kcnj3 | potassium inwardly-rectifying channel, subfamily J, | uniqAntib_1098 | 0.05 | -0.02 | -0.06 | -0.29 | -0.14 | 0 | -0.0471 | -1.0332 | 1.35644 | 0.21843 | 1 | -0.275 | -1.21 | 1.61325 | 0.03304 | 0 | -0.1288 | -1.0934 | 1.40377 | 0.15645 | 0 | 0 |
| 1388395_at | G0s2 | G0/G1switch 2 | uniqAntib_1098 | -0.14 | -0.62 | -0.74 | -0.21 | -0.36 | 0 | -0.1256 | -1.091 | 3.09488 | 0.02428 | 1 | 0.40183 | 1.32119 | 0.35149 | 0.00129 | 0 | 0.25225 | 1.19106 | 1.72505 | 0.00527 | 0 | 0 |
| 1388439_at | Fkbp10 | FK506 binding protein 10 | uniqAntib_1098 | 0.12 | 0.02 | -0.17 | -0.28 | -0.17 | 0 | -0.1988 | -1.1478 | 2.12966 | 0.07957 | 1 | -0.3011 | -1.2321 | 1.66788 | 0.02837 | 0 | -0.1901 | -1.1408 | 1.68185 | 0.09513 | 0 | 0 |
| 1388460_at | Capg | capping protein (actin filament), gelsolin-like | uniqAntib_1098 | -0.01 | -0.07 | -0.12 | 0.24 | 0.09 | 0 | -0.0457 | -1.0322 | 0.92738 | 0.50975 | 1 | 0.3149 | 1.24392 | 1.22386 | 0.03231 | 0 | 0.15803 | 1.11576 | 1.44691 | 0.16698 | 0 | 0 |
| 1388644_at | Mgll | monoglyceride lipase | uniqAntib_1098 | 0.14 | 0.00 | 0.00 | -0.30 | -0.14 | 0 | -0.002 | -1.0014 | 0.89761 | 0.60592 | 1 | -0.3061 | -1.2364 | 1.83606 | 0.02114 | 0 | -0.1437 | -1.1047 | 1.43755 | 0.14635 | 0 | 0 |
| 1388681_at | Sar1b | SAR1 homolog B (S. cerevisiae) | uniqAntib_1098 | -0.09 | -0.41 | -0.43 | -0.14 | -0.33 | 0 | -0.0189 | -1.0132 | 1.14695 | 0.31002 | 1 | 0.27066 | 1.20636 | 1.2535 | 0.03636 | 0 | 0.07796 | 1.05553 | 1.3361 | 0.20747 | 0 | 0 |
| 1388711_at | Il13ra1 /// LO | interleukin 13 receptor, alpha 1 /// interleukin 13 re | uniqAntib_1098 | -0.12 | -0.15 | -0.14 | 0.26 | -0.01 | 0 | 0.01003 | 1.00697 | 1.65778 | 0.1161 | 1 | 0.40827 | 1.32709 | 0.50182 | 0.00248 | 0 | 0.14043 | 1.10224 | 2.37935 | 0.02341 | 0 | 0 |
| 1388728_at | Laptm4b | lysosomal protein transmembrane 4 beta | uniqAntib_1098 | 0.09 | -0.81 | -0.77 | -0.43 | -0.75 | 0 | 0.03506 | 1.0246 | 1.39608 | 0.19483 | 1 | 0.377 | 1.29864 | 0.55886 | 0.00284 | 0 | 0.05823 | 1.04119 | 1.47911 | 0.15738 | 0 | 0 |
| 1388738_at | --- | --- | uniqAntib_1098 | 0.15 | 0.07 | 0.11 | 0.47 | 0.25 | 0 | 0.04567 | 1.03216 | 1.22244 | 0.26843 | 1 | 0.40498 | 1.32407 | 0.72756 | 0.00585 | 0 | 0.18247 | 1.13482 | 1.67219 | 0.10786 | 0 | 0 |
| 1388792_at | Gadd45g | growth arrest and DNA-damage-inducible, gamma | uniqAntib_1098 | 0.06 | 0.14 | 0.33 | -0.21 | 0.30 | 0 | 0.18783 | 1.13905 | 1.60195 | 0.13043 | 1 | -0.3517 | -1.276 | 2.01645 | 0.01718 | 0 | 0.1558 | 1.11404 | 1.57091 | 0.13199 | 0 | 0 |
| 1388799_at | Klhl7 | kelch-like family member 7 | uniqAntib_1098 | -0.13 | 0.07 | 0.07 | 0.47 | 0.06 | 0 | -0.0017 | -1.0012 | 1.23315 | 0.26147 | 1 | 0.39812 | 1.31779 | 0.83807 | 0.00798 | 0 | -0.009 | -1.0063 | 1.09182 | 0.32275 | 0 | 0 |
| 1388831_at | Slc9a3r2 | solute carrier family 9 (sodium/hydrogen exchanger | uniqAntib_1098 | -0.07 | 0.06 | -0.18 | -0.40 | -0.19 | 0 | -0.24 | -1.181 | 2.50018 | 0.05258 | 1 | -0.4604 | -1.3759 | 4.29583 | 0.00166 | 0 | -0.2479 | -1.1875 | 2.34911 | 0.03241 | 0 | 0 |
| 1388846_at | Bcl2l12 | BCL2-like 12 (proline rich) | uniqAntib_1098 | 0.01 | -0.01 | 0.07 | 0.29 | 0.12 | 0 | 0.0787 | 1.05607 | 1.06206 | 0.3827 | 1 | 0.29854 | 1.2299 | 1.25309 | 0.03747 | 0 | 0.12616 | 1.09138 | 1.35636 | 0.19762 | 0 | 0 |
| 1388853_at | Mrpl54 | mitochondrial ribosomal protein L54 | uniqAntib_1098 | -0.04 | -0.39 | -0.31 | -0.11 | -0.27 | 0 | 0.07635 | 1.05435 | 1.17982 | 0.29538 | 1 | 0.28078 | 1.21485 | 1.20226 | 0.02826 | 0 | 0.12237 | 1.08852 | 1.31236 | 0.2156 | 0 | 0 |
| 1388876_at | Pln | phospholamban | uniqAntib_1098 | -0.06 | -0.02 | -0.28 | -0.40 | -0.11 | 0 | -0.259 | -1.1966 | 3.35464 | 0.01208 | 1 | -0.3726 | -1.2946 | 4.086 | 0.00328 | 0 | -0.0864 | -1.0617 | 1.83366 | 0.07064 | 0 | 0 |
| 1388882_at | Fkbp3 | FK506 binding protein 3 | uniqAntib_1098 | -0.07 | -0.43 | -0.40 | -0.12 | -0.29 | 0 | 0.0228 | 1.01593 | 1.38235 | 0.20123 | 1 | 0.30694 | 1.23708 | 1.1037 | 0.01984 | 0 | 0.13049 | 1.09466 | 1.70143 | 0.10264 | 0 | 0 |
| 1388969_at | Acsf3 | acyl-CoA synthetase family member 3 | uniqAntib_1098 | 0.03 | 0.03 | 0.03 | -0.26 | -0.05 | 0 | 0.00117 | 1.00081 | 0.88054 | 0.61221 | 1 | -0.2898 | -1.2224 | 1.43735 | 0.04858 | 0 | -0.0844 | -1.0602 | 1.01436 | 0.404 | 0 | 0 |
| 1388996_at | Hdhd2 | haloacid dehalogenase-like hydrolase domain conta | uniqAntib_1098 | -0.11 | -0.26 | -0.36 | 0.01 | -0.25 | 0 | -0.1038 | -1.0746 | 1.55481 | 0.16359 | 1 | 0.26886 | 1.20486 | 1.27598 | 0.04119 | 0 | 0.01307 | 1.0091 | 1.01958 | 0.41135 | 0 | 0 |
| 1389045_at | --- | --- | uniqAntib_1098 | 0.16 | 0.17 | 0.05 | -0.11 | -0.02 | 0 | -0.1278 | -1.0926 | 1.35142 | 0.22023 | 1 | -0.2826 | -1.2164 | 1.57612 | 0.03446 | 0 | -0.1905 | -1.1412 | 1.61944 | 0.10102 | 0 | 0 |
| 1389071_at | Ehbp1l1 | EH domain binding protein 1-like 1 | uniqAntib_1098 | 0.04 | 0.21 | 0.24 | 0.51 | 0.37 | 0 | 0.02678 | 1.01874 | 1.22784 | 0.26453 | 1 | 0.29865 | 1.22999 | 1.18417 | 0.02593 | 0 | 0.15497 | 1.1134 | 1.66655 | 0.11071 | 0 | 0 |
| 1389110_at | LOC10036001 | mitochondrial ribosomal protein S6-like | uniqAntib_1098 | -0.05 | -0.26 | -0.24 | 0.23 | -0.15 | 0 | 0.01943 | 1.01356 | 1.05261 | 0.38812 | 1 | 0.48574 | 1.4003 | 0.39246 | 0.00164 | 0 | 0.11258 | 1.08116 | 1.23503 | 0.25349 | 0 | 0 |
| 1389114_at | Thnsl2 | threonine synthase-like 2 (S. cerevisiae) | uniqAntib_1098 | 0.10 | 0.17 | 0.04 | -0.18 | 0.04 | 0 | -0.1372 | -1.0998 | 1.45026 | 0.1884 | 1 | -0.3547 | -1.2787 | 2.18908 | 0.01225 | 0 | -0.1336 | -1.0971 | 1.42859 | 0.14884 | 0 | 0 |
| 1389239_at | --- | --- | uniqAntib_1098 | 0.12 | 0.03 | 0.09 | -0.30 | 0.08 | 0 | 0.06771 | 1.04805 | 1.1925 | 0.28724 | 1 | -0.3231 | -1.251 | 1.63381 | 0.03147 | 0 | 0.04934 | 1.03479 | 0.96176 | 0.4753 | 0 | 0 |
| 1389267_at | --- | --- | uniqAntib_1098 | -0.04 | -0.24 | -0.17 | 0.08 | 0.02 | 0 | 0.07295 | 1.05186 | 1.67754 | 0.11166 | 1 | 0.31894 | 1.24741 | 1.00964 | 0.01513 | 0 | 0.26017 | 1.19762 | 2.28945 | 0.01472 | 0 | 0 |
| 1389295_at | Olfml2b | olfactomedin-like 2B | uniqAntib_1098 | 0.18 | 0.03 | 0.05 | -0.29 | 0.07 | 0 | 0.02786 | 1.0195 | 1.04389 | 0.40438 | 1 | -0.3116 | -1.2411 | 1.55479 | 0.0377 | 0 | 0.04216 | 1.02965 | 1.17762 | 0.28415 | 0 | 0 |
| 1389326_at | Rfc3 | replication factor C (activator 1) 3 | uniqAntib_1098 | -0.06 | -0.74 | -0.67 | -0.27 | -0.51 | 0 | 0.06974 | 1.04953 | 1.13954 | 0.32253 | 1 | 0.46542 | 1.38072 | 0.43743 | 0.00197 | 0 | 0.22971 | 1.1726 | 2.17571 | 0.04729 | 0 | 0 |
| 1389418_at | --- | --- | uniqAntib_1098 | 0.19 | 0.47 | 0.48 | 0.76 | 0.58 | 0 | 0.00931 | 1.00647 | 1.4102 | 0.18954 | 1 | 0.28846 | 1.22133 | 0.9292 | 0.0101 | 0 | 0.11033 | 1.07947 | 2.08705 | 0.05503 | 0 | 0 |
| 1389433_at | Mkks | McKusick-Kaufman syndrome | uniqAntib_1098 | -0.08 | -0.28 | -0.28 | 0.05 | -0.17 | 0 | -0.0035 | -1.0024 | 1.01845 | 0.40693 | 1 | 0.33514 | 1.2615 | 0.96314 | 0.01214 | 0 | 0.11214 | 1.08083 | 1.61153 | 0.12421 | 0 | 0 |
| 1389447_at | P4htm | prolyl 4-hydroxylase, transmembrane | uniqAntib_1098 | 0.03 | -0.02 | -0.15 | -0.33 | -0.05 | 0 | -0.1283 | -1.093 | 1.52345 | 0.17072 | 1 | -0.3115 | -1.241 | 1.58825 | 0.0336 | 0 | -0.0358 | -1.0251 | 0.95269 | 0.50853 | 0 | 0 |
| 1389463_at | Prkar1b | protein kinase, cAMP dependent regulatory, type I, | uniqAntib_1098 | -0.04 | 0.06 | -0.03 | -0.33 | -0.13 | 0 | -0.0924 | -1.0662 | 1.36971 | 0.21405 | 1 | -0.3881 | -1.3087 | 2.63779 | 0.00729 | 0 | -0.1965 | -1.1459 | 1.95608 | 0.05466 | 0 | 0 |
| 1389525_at | Rnf149 | ring finger protein 149 | uniqAntib_1098 | -0.10 | 0.06 | 0.15 | 0.35 | 0.11 | 0 | 0.09038 | 1.06465 | 1.40367 | 0.19273 | 1 | 0.28678 | 1.21992 | 1.2016 | 0.02701 | 0 | 0.04654 | 1.03278 | 1.16519 | 0.29116 | 0 | 0 |
| 1389528_s_at | Jun | jun proto-oncogene | uniqAntib_1098 | -0.02 | 0.50 | 0.59 | 0.89 | 0.32 | 0 | 0.09084 | 1.06499 | 1.39224 | 0.19577 | 1 | 0.38714 | 1.30779 | 0.76424 | 0.00666 | 0 | -0.1873 | -1.1386 | 2.08281 | 0.04574 | 0 | 0 |
| 1389592_at | --- | --- | uniqAntib_1098 | 0.07 | 0.16 | 0.02 | -0.13 | 0.14 | 0 | -0.1466 | -1.1069 | 2.04482 | 0.08824 | 1 | -0.2869 | -1.22 | 1.56056 | 0.03508 | 0 | -0.0205 | -1.0143 | 1.12481 | 0.29557 | 0 | 0 |
| 1389666_at | Rom1 | retinal outer segment membrane protein 1 | uniqAntib_1098 | -0.13 | -0.21 | -0.17 | 0.09 | -0.20 | 0 | 0.03519 | 1.02469 | 0.99604 | 0.45137 | 1 | 0.29554 | 1.22734 | 1.24635 | 0.03535 | 0 | 0.01044 | 1.00726 | 0.95716 | 0.48069 | 0 | 0 |
| 1389668_at | Spc25 | SPC25, NDC80 kinetochore complex component, ho | uniqAntib_1098 | -0.19 | -0.29 | -0.47 | 0.04 | -0.27 | 0 | -0.181 | -1.1337 | 2.08273 | 0.08452 | 1 | 0.32388 | 1.25169 | 1.1229 | 0.0204 | 0 | 0.01457 | 1.01015 | 0.97276 | 0.46218 | 0 | 0 |
| 1389711_at | LOC679082 | similar to Rho GTPase activating protein 19 | uniqAntib_1098 | 0.13 | 0.09 | -0.09 | -0.20 | 0.08 | 0 | -0.1868 | -1.1382 | 1.8051 | 0.12154 | 1 | -0.2915 | -1.2239 | 1.69927 | 0.02688 | 0 | -0.0102 | -1.0071 | 0.91851 | 0.5829 | 0 | 0 |
| 1389865_at | Toe1 | target of EGR1, member 1 (nuclear) | uniqAntib_1098 | 0.02 | -0.33 | -0.28 | -0.03 | -0.13 | 0 | 0.04629 | 1.03261 | 1.38622 | 0.19782 | 1 | 0.29288 | 1.22508 | 1.13939 | 0.0218 | 0 | 0.19385 | 1.14381 | 2.06146 | 0.06059 | 0 | 0 |
| 1389868_at | Ddx6 | DEAD (Asp-Glu-Ala-Asp) box helicase 6 | uniqAntib_1098 | -0.09 | 0.02 | 0.12 | 0.33 | -0.02 | 0 | 0.10829 | 1.07795 | 2.06529 | 0.0348 | 1 | 0.31675 | 1.24552 | 0.7017 | 0.00505 | 0 | -0.0369 | -1.0259 | 1.74379 | 0.08271 | 0 | 0 |
| 1389876_at | Camk2n1 | calcium/calmodulin-dependent protein kinase II inh | uniqAntib_1098 | -0.02 | -0.12 | -0.01 | 0.24 | -0.11 | 0 | 0.10859 | 1.07817 | 1.14805 | 0.31449 | 1 | 0.36304 | 1.28613 | 0.98912 | 0.01406 | 0 | 0.00882 | 1.00614 | 0.95956 | 0.478 | 0 | 0 |
| 1389884_at | Rps6ka4 | ribosomal protein S6 kinase, polypeptide 4 | uniqAntib_1098 | -0.07 | -0.03 | -0.12 | -0.30 | -0.22 | 0 | -0.0882 | -1.0631 | 1.64111 | 0.14681 | 1 | -0.2682 | -1.2043 | 1.48337 | 0.04283 | 0 | -0.1899 | -1.1407 | 1.73781 | 0.08762 | 0 | 0 |
| 1389908_at | --- | --- | uniqAntib_1098 | -0.08 | 0.32 | 0.10 | 0.04 | 0.11 | 0 | -0.2182 | -1.1633 | 2.76066 | 0.03773 | 1 | -0.2864 | -1.2196 | 1.97018 | 0.01799 | 0 | -0.209 | -1.1559 | 2.35242 | 0.03343 | 0 | 0 |
| 1389936_at | LOC687035 | similar to Tripartite motif protein 56 | uniqAntib_1098 | -0.03 | 0.00 | 0.12 | -0.34 | -0.10 | 0 | 0.12094 | 1.08744 | 1.32568 | 0.21987 | 1 | -0.3453 | -1.2704 | 2.12898 | 0.01349 | 0 | -0.1035 | -1.0744 | 1.2826 | 0.20205 | 0 | 0 |
| 1389989_at | Atrx | alpha thalassemia/mental retardation syndrome X-l | uniqAntib_1098 | 0.20 | 0.07 | 0.09 | 0.46 | 0.09 | 0 | 0.02018 | 1.01409 | 2.13524 | 0.0254 | 1 | 0.39039 | 1.31075 | 0.37509 | 0.0014 | 0 | 0.01493 | 1.0104 | 2.32408 | 0.02107 | 0 | 0 |
| 1390001_at | Rhobtb2 | Rho-related BTB domain containing 2 | uniqAntib_1098 | 0.00 | 0.01 | -0.05 | -0.27 | 0.02 | 0 | -0.0655 | -1.0465 | 1.26722 | 0.25133 | 1 | -0.2772 | -1.2119 | 1.44785 | 0.0459 | 0 | 0.00639 | 1.00444 | 0.9675 | 0.46837 | 0 | 0 |
| 1390006_at | Rbm15b | RNA binding motif protein 15B | uniqAntib_1098 | -0.22 | -0.25 | -0.09 | -0.61 | -0.33 | 0 | 0.15621 | 1.11436 | 1.25373 | 0.25353 | 1 | -0.3625 | -1.2857 | 2.0025 | 0.01545 | 0 | -0.0806 | -1.0574 | 1.01766 | 0.402 | 0 | 0 |
| 1390017_at | Hapln2 | hyaluronan and proteoglycan link protein 2 | uniqAntib_1098 | 0.07 | 0.14 | 0.04 | -0.14 | 0.05 | 0 | -0.1025 | -1.0736 | 1.29368 | 0.24144 | 1 | -0.2801 | -1.2142 | 1.46394 | 0.04491 | 0 | -0.0932 | -1.0667 | 1.21288 | 0.23786 | 0 | 0 |
| 1390140_at | Lemd3 | LEM domain containing 3 | uniqAntib_1098 | 0.16 | 0.19 | 0.04 | -0.12 | 0.11 | 0 | -0.154 | -1.1126 | 1.6101 | 0.15294 | 1 | -0.3137 | -1.2429 | 1.59678 | 0.03353 | 0 | -0.0834 | -1.0595 | 1.05357 | 0.35948 | 0 | 0 |
| 1390150_at | --- | --- | uniqAntib_1098 | -0.10 | 0.03 | -0.04 | -0.24 | -0.08 | 0 | -0.0717 | -1.0509 | 1.22919 | 0.2649 | 1 | -0.2704 | -1.2061 | 1.78831 | 0.02409 | 0 | -0.108 | -1.0777 | 1.36468 | 0.16965 | 0 | 0 |
| 1390159_at | Rasgrp3 | RAS guanyl releasing protein 3 (calcium and DAG-re | uniqAntib_1098 | -0.03 | -0.07 | 0.03 | 0.21 | -0.03 | 0 | 0.10063 | 1.07224 | 1.7845 | 0.07488 | 1 | 0.28646 | 1.21964 | 1.18282 | 0.02468 | 0 | 0.04129 | 1.02904 | 1.31991 | 0.21302 | 0 | 0 |
| 1390199_at | --- | --- | uniqAntib_1098 | 0.02 | 0.11 | 0.17 | 0.54 | 0.20 | 0 | 0.05825 | 1.0412 | 1.16675 | 0.30318 | 1 | 0.43211 | 1.3492 | 0.68881 | 0.00485 | 0 | 0.08743 | 1.06247 | 1.23342 | 0.25458 | 0 | 0 |
| 1390263_at | Gnas | GNAS complex locus | uniqAntib_1098 | 0.01 | 0.17 | 0.43 | 0.54 | 0.05 | 0 | 0.25897 | 1.19663 | 2.27754 | 0.01311 | 1 | 0.36159 | 1.28484 | 0.72341 | 0.00586 | 0 | -0.1235 | -1.0894 | 1.81755 | 0.07522 | 0 | 0 |
| 1390297_at | Zc3h14 | zinc finger CCCH type containing 14 | uniqAntib_1098 | -0.01 | 0.05 | 0.00 | 0.33 | 0.09 | 0 | -0.0564 | -1.0399 | 1.40766 | 0.20097 | 1 | 0.27816 | 1.21265 | 1.21371 | 0.03079 | 0 | 0.04002 | 1.02813 | 1.54752 | 0.13953 | 0 | 0 |
| 1390304_at | --- | --- | uniqAntib_1098 | -0.01 | -0.23 | -0.07 | -0.52 | -0.04 | 0 | 0.16142 | 1.11839 | 1.556 | 0.1411 | 1 | -0.2935 | -1.2256 | 2.55759 | 0.00954 | 0 | 0.18997 | 1.14074 | 2.32322 | 0.03967 | 0 | 0 |
| 1390355_at | --- | --- | uniqAntib_1098 | -0.04 | 0.19 | 0.07 | -0.20 | -0.04 | 0 | -0.1188 | -1.0859 | 1.28757 | 0.24419 | 1 | -0.3885 | -1.3091 | 2.44365 | 0.0099 | 0 | -0.233 | -1.1753 | 1.96226 | 0.05508 | 0 | 0 |
| 1390386_at | Casp3 | caspase 3 | uniqAntib_1098 | -0.09 | -0.19 | -0.22 | 0.13 | -0.14 | 0 | -0.0324 | -1.0227 | 0.93622 | 0.49365 | 1 | 0.32168 | 1.24978 | 1.20535 | 0.02725 | 0 | 0.05206 | 1.03674 | 1.06462 | 0.36719 | 0 | 0 |
| 1390398_at | LOC1009125( | uncharacterized LOC100912503 | uniqAntib_1098 | -0.04 | -0.07 | 0.03 | -0.33 | -0.25 | 0 | 0.09861 | 1.07074 | 1.26166 | 0.24901 | 1 | -0.2674 | -1.2037 | 1.56121 | 0.03534 | 0 | -0.1877 | -1.1389 | 1.83689 | 0.07241 | 0 | 0 |
| 1390406_at | Arhgap18 | Rho GTPase activating protein 18 | uniqAntib_1098 | 0.06 | -0.32 | -0.35 | 0.06 | -0.17 | 0 | -0.0285 | -1.0199 | 1.30658 | 0.23742 | 1 | 0.38066 | 1.30193 | 0.62054 | 0.00409 | 0 | 0.14773 | 1.10782 | 2.27892 | 0.03803 | 0 | 0 |
| 1390470_at | Ccnt2 | cyclin T2 | uniqAntib_1098 | -0.11 | -0.40 | -0.14 | -0.11 | -0.18 | 0 | 0.26041 | 1.19782 | 2.22073 | 0.01557 | 1 | 0.29579 | 1.22756 | 1.20759 | 0.02998 | 0 | 0.21992 | 1.16467 | 2.31603 | 0.03992 | 0 | 0 |
| 1390481_a_at | Ube2t | ubiquitin-conjugating enzyme E2T (putative) | uniqAntib_1098 | 0.09 | 0.10 | -0.05 | -0.21 | -0.01 | 0 | -0.1532 | -1.112 | 1.76398 | 0.1274 | 1 | -0.307 | -1.2372 | 1.79358 | 0.02192 | 0 | -0.1091 | -1.0785 | 1.35119 | 0.17484 | 0 | 0 |
| 1390483_at | Slc25a29 | solute carrier family 25 (mitochondrial carnitine/acy | uniqAntib_1098 | 0.12 | 0.22 | 0.20 | -0.10 | 0.15 | 0 | -0.0227 | -1.0159 | 1.07619 | 0.35567 | 1 | -0.3207 | -1.2489 | 1.67194 | 0.0286 | 0 | -0.0661 | -1.0469 | 1.02424 | 0.391 | 0 | 0 |
| 1390512_at | --- | --- | uniqAntib_1098 | -0.07 | 0.08 | 0.01 | -0.25 | 0.00 | 0 | -0.0725 | -1.0515 | 1.1315 | 0.32083 | 1 | -0.3287 | -1.2559 | 1.76923 | 0.02424 | 0 | -0.0805 | -1.0574 | 1.01449 | 0.40356 | 0 | 0 |

| Affymetrix Probeset ID | Gene Symbol | Gene Title | Figure 6 B Venn n =2021 | Mean BN log2 intensity autologous n=8 | Mean log2 intensity day 30 DR +/+ ND n=4 | Mean log2 intensity day 30 DR/+/+ HCD n=4 | Mean log2 intensity day 30 DR+/+ B/S n=4 | Mean log2 intensity day 30 F+/+ ND n=4 | DR+/+ HCD vs DR+/+ ND n=380 1=yes 0=no | Log2 ratio DR+/+ HCD vs DR+/+ ND | Fold-Change DR+/+ HCD vs DR+/+ ND | RP-FDR 4DR30 HCD vs 4DR30 Reg | p value DR+/+ HCD vs DR+/+ ND | DR+/+ B/S vs DR+/+ ND n=1,330 1=yes 0=no | Log2 ratio DR+/+ B/S vs DR+/+ ND | Fold-Change DR+/+ B/S vs DR+/+ ND | RP-FDR 4DR30 Antib vs 4DR30 Reg | p value DR+/+ B/S vs DR+/+ ND | DR+/+ ND vs F+/+ ND n=398 1=yes 0=no | Log2 ratio DR+/+ ND vs F+/+ ND | Fold-Change DR+/+ ND vs F+/+ ND | RP-FDR 4RT1uu 30Reg vs 4DR30 Reg | p value DR+/+ ND vs F+/+ ND | DR+/+ Longitudinal n=228 1=yes 0=no | Figure 6F |
|---|---|---|---|---|---|---|---|---|---|---|---|---|---|---|---|---|---|---|---|---|---|---|---|---|---|
| 1390571_at | Gpr4 | G protein-coupled receptor 4 | uniqAntib_1098 | 0.05 | -0.07 | -0.05 | -0.40 | -0.19 | 0 | 0.01953 | 1.01363 | 1.05593 | 0.38554 | 1 | -0.3256 | -1.2531 | 2.21429 | 0.0121 | 0 | -0.1141 | -1.0823 | 1.51701 | 0.12029 | 0 | 0 |
| 1390589_at | --- | --- | uniqAntib_1098 | -0.03 | -0.11 | -0.06 | -0.39 | -0.01 | 0 | 0.05161 | 1.03642 | 1.16894 | 0.3025 | 1 | -0.2792 | -1.2135 | 1.7416 | 0.02492 | 0 | 0.09588 | 1.06872 | 1.38881 | 0.18622 | 0 | 0 |
| 1390610_at | --- | --- | uniqAntib_1098 | -0.12 | 0.05 | -0.17 | -0.30 | -0.12 | 0 | -0.2189 | -1.1638 | 2.378 | 0.06255 | 1 | -0.3489 | -1.2736 | 2.00447 | 0.01528 | 0 | -0.1726 | -1.1271 | 1.46436 | 0.14023 | 0 | 0 |
| 1390649_at | Slc30a3 | solute carrier family 30 (zinc transporter), member 3 | uniqAntib_1098 | -0.02 | 0.13 | 0.02 | -0.18 | -0.04 | 0 | -0.1083 | -1.078 | 1.11966 | 0.32644 | 1 | -0.308 | -1.238 | 1.56388 | 0.0347 | 0 | -0.1708 | -1.1257 | 1.447 | 0.14349 | 0 | 0 |
| 1390741_at | Zfr2 | zinc finger RNA binding protein 2 | uniqAntib_1098 | -0.02 | -0.04 | 0.04 | -0.36 | -0.13 | 0 | 0.0761 | 1.05416 | 1.02174 | 0.42343 | 1 | -0.3247 | -1.2524 | 2.02251 | 0.01476 | 0 | -0.0925 | -1.0662 | 1.23943 | 0.22398 | 0 | 0 |
| 1390767_at | Ssr1 | signal sequence receptor, alpha | uniqAntib_1098 | -0.09 | -0.39 | -0.32 | 0.00 | -0.31 | 0 | 0.07178 | 1.05101 | 1.92965 | 0.05696 | 1 | 0.38737 | 1.30801 | 0.96331 | 0.01227 | 0 | 0.07593 | 1.05404 | 1.15044 | 0.29846 | 0 | 0 |
| 1390813_at | Msi2 | musashi RNA-binding protein 2 | uniqAntib_1098 | 0.18 | 0.40 | 0.50 | 0.79 | 0.41 | 0 | 0.0996 | 1.07147 | 1.84188 | 0.06864 | 1 | 0.38759 | 1.3082 | 0.56315 | 0.00299 | 0 | 0.00784 | 1.00545 | 1.59202 | 0.12783 | 0 | 0 |
| 1390839_at | Pqlc3 | PQ loop repeat containing 3 | uniqAntib_1098 | -0.13 | -0.40 | -0.13 | 0.01 | -0.32 | 0 | 0.26241 | 1.19948 | 2.28196 | 0.01834 | 1 | 0.40556 | 1.32461 | 0.59866 | 0.0036 | 0 | 0.08123 | 1.05792 | 1.28938 | 0.22766 | 0 | 0 |
| 1390858_at | Vcpip1 | valosin containing protein (p97)/p47 complex intera | uniqAntib_1098 | -0.28 | -0.03 | 0.12 | 0.24 | -0.10 | 0 | 0.15125 | 1.11053 | 1.6435 | 0.11748 | 1 | 0.27263 | 1.20801 | 1.21312 | 0.03097 | 0 | -0.0616 | -1.0436 | 1.13776 | 0.28474 | 0 | 0 |
| 1390876_at | Ppip5k1 | diphosphoinositol pentakisphosphate kinase 1 | uniqAntib_1098 | 0.02 | 0.48 | 0.33 | 0.18 | 0.50 | 0 | -0.1436 | -1.1047 | 1.9357 | 0.10444 | 1 | -0.297 | -1.2286 | 2.07765 | 0.01423 | 0 | 0.02648 | 1.01852 | 1.07578 | 0.35502 | 0 | 0 |
| 1391048_at | --- | --- | uniqAntib_1098 | -0.08 | 0.00 | 0.25 | 0.27 | 0.04 | 0 | 0.24809 | 1.18763 | 2.29209 | 0.01518 | 1 | 0.26602 | 1.20249 | 1.2298 | 0.03298 | 0 | 0.03504 | 1.02459 | 1.37915 | 0.18994 | 0 | 0 |
| 1391055_at | --- | --- | uniqAntib_1098 | -0.07 | 0.12 | 0.11 | -0.19 | -0.11 | 0 | -0.005 | -1.0035 | 1.05214 | 0.37384 | 1 | -0.303 | -1.2337 | 2.38351 | 0.01004 | 0 | -0.2272 | -1.1705 | 2.46655 | 0.02919 | 0 | 0 |
| 1391063_at | Kif23 | kinesin family member 23 | uniqAntib_1098 | -0.06 | -0.04 | -0.07 | 0.28 | -0.03 | 0 | -0.0308 | -1.0216 | 1.03497 | 0.3922 | 1 | 0.31559 | 1.24452 | 1.25484 | 0.03724 | 0 | 0.0054 | 1.00375 | 0.91125 | 0.61695 | 0 | 0 |
| 1391085_at | --- | --- | uniqAntib_1098 | -0.19 | -0.16 | -0.16 | 0.13 | -0.13 | 0 | -0.001 | -1.0007 | 0.98683 | 0.46116 | 1 | 0.28229 | 1.21612 | 1.28164 | 0.04356 | 0 | 0.02385 | 1.01667 | 0.93043 | 0.52946 | 0 | 0 |
| 1391110_at | --- | --- | uniqAntib_1098 | -0.06 | -0.03 | -0.05 | -0.33 | -0.15 | 0 | -0.018 | -1.0125 | 0.93346 | 0.49928 | 1 | -0.2976 | -1.2291 | 1.54126 | 0.03846 | 0 | -0.119 | -1.086 | 1.1384 | 0.28465 | 0 | 0 |
| 1391133_at | Il17rc | interleukin 17 receptor C | uniqAntib_1098 | -0.01 | -0.15 | 0.03 | -0.41 | -0.14 | 0 | 0.17696 | 1.1305 | 1.48195 | 0.16216 | 1 | -0.2643 | -1.2011 | 1.46786 | 0.04441 | 0 | 0.01191 | 1.00829 | 0.94608 | 0.50062 | 0 | 0 |
| 1391141_at | --- | --- | uniqAntib_1098 | -0.09 | -0.08 | -0.19 | -0.38 | -0.15 | 0 | -0.116 | -1.0837 | 1.57616 | 0.15884 | 1 | -0.3007 | -1.2317 | 1.65823 | 0.03007 | 0 | -0.0707 | -1.0502 | 1.07518 | 0.3356 | 0 | 0 |
| 1391156_at | Podnl1 | podocan-like 1 | uniqAntib_1098 | -0.01 | 0.28 | 0.16 | 0.02 | 0.04 | 0 | -0.1198 | -1.0866 | 1.93457 | 0.10445 | 1 | -0.2639 | -1.2007 | 1.461 | 0.04454 | 0 | -0.2397 | -1.1807 | 2.10062 | 0.04492 | 0 | 0 |
| 1391178_at | --- | --- | uniqAntib_1098 | 0.05 | 0.02 | -0.06 | -0.26 | -0.01 | 0 | -0.0781 | -1.0556 | 1.41081 | 0.19979 | 1 | -0.2816 | -1.2155 | 1.50284 | 0.04103 | 0 | -0.0299 | -1.0209 | 0.96745 | 0.48427 | 0 | 0 |
| 1391193_at | --- | --- | uniqAntib_1098 | -0.12 | 0.03 | 0.06 | 0.30 | -0.04 | 0 | 0.0293 | 1.02051 | 1.09589 | 0.35235 | 1 | 0.2694 | 1.20531 | 1.26524 | 0.03959 | 0 | -0.0612 | -1.0434 | 1.06444 | 0.34655 | 0 | 0 |
| 1391224_at | Cnbd2 | cyclic nucleotide binding domain containing 2 | uniqAntib_1098 | 0.01 | 0.04 | 0.18 | 0.31 | -0.02 | 0 | 0.13285 | 1.09646 | 1.30997 | 0.2251 | 1 | 0.26997 | 1.20578 | 1.27166 | 0.04968 | 0 | -0.0674 | -1.0478 | 1.06823 | 0.34167 | 0 | 0 |
| 1391258_at | --- | --- | uniqAntib_1098 | -0.09 | 0.02 | 0.02 | -0.31 | -0.10 | 0 | 0.00365 | 1.00253 | 0.93993 | 0.4895 | 1 | -0.3233 | -1.2512 | 1.64428 | 0.03067 | 0 | -0.1112 | -1.0801 | 1.15047 | 0.27745 | 0 | 0 |
| 1391266_at | --- | --- | uniqAntib_1098 | -0.09 | 0.44 | 0.30 | 0.16 | 0.49 | 0 | -0.1454 | -1.1061 | 1.88119 | 0.11197 | 1 | -0.2846 | -1.2181 | 1.62467 | 0.03176 | 0 | 0.05045 | 1.03559 | 1.13697 | 0.30842 | 0 | 0 |
| 1391277_at | S100a5 | S100 calcium binding protein A5 | uniqAntib_1098 | 0.21 | 0.22 | 0.16 | -0.07 | 0.22 | 0 | -0.0608 | -1.043 | 1.12869 | 0.32297 | 1 | -0.2949 | -1.2268 | 1.74316 | 0.02522 | 0 | 0.00039 | 1.00027 | 0.94539 | 0.51941 | 0 | 0 |
| 1391289_at | LOC100911432 | uncharacterized LOC100911432 | uniqAntib_1098 | -0.02 | -0.01 | -0.02 | -0.30 | -0.10 | 0 | -0.0124 | -1.0086 | 0.98476 | 0.43689 | 1 | -0.2939 | -1.2259 | 1.5672 | 0.03659 | 0 | -0.0931 | -1.0666 | 1.17192 | 0.26522 | 0 | 0 |
| 1391333_at | --- | --- | uniqAntib_1098 | -0.01 | 0.10 | 0.29 | 0.42 | 0.19 | 0 | 0.19325 | 1.14333 | 1.75933 | 0.07886 | 1 | 0.3241 | 1.25188 | 1.13323 | 0.02135 | 0 | 0.09056 | 1.06478 | 1.32921 | 0.20858 | 0 | 0 |
| 1391341_at | Plxdc1 | plexin domain containing 1 | uniqAntib_1098 | -0.08 | -0.03 | -0.09 | -0.38 | -0.02 | 0 | -0.0663 | -1.0471 | 1.07532 | 0.35736 | 1 | -0.3517 | -1.276 | 1.92799 | 0.01816 | 0 | 0.00709 | 1.00493 | 0.90228 | 0.61139 | 0 | 0 |
| 1391415_at | Pno1 | partner of NOB1 homolog (S. cerevisiae) | uniqAntib_1098 | -0.05 | -0.18 | -0.03 | 0.16 | -0.12 | 0 | 0.15795 | 1.1157 | 1.72694 | 0.09479 | 1 | 0.34418 | 1.26943 | 1.07518 | 0.01808 | 0 | 0.064 | 1.04536 | 1.32804 | 0.20942 | 0 | 0 |
| 1391438_at | Evi5 | ecotropic viral integration site 5 | uniqAntib_1098 | -0.03 | -0.01 | 0.05 | 0.33 | 0.01 | 0 | 0.06105 | 1.04323 | 1.0172 | 0.42763 | 1 | 0.34074 | 1.26641 | 1.05724 | 0.01751 | 0 | 0.02602 | 1.0182 | 0.92558 | 0.54296 | 0 | 0 |
| 1391466_at | Nucks1 | nuclear casein kinase and cyclin-dependent kinase s | uniqAntib_1098 | -0.13 | -0.16 | -0.24 | 0.15 | -0.14 | 0 | -0.0737 | -1.0524 | 1.25437 | 0.25568 | 1 | 0.31778 | 1.24641 | 1.16621 | 0.02378 | 0 | 0.02352 | 1.01644 | 1.01836 | 0.41197 | 0 | 0 |
| 1391524_at | Phip | pleckstrin homology domain interacting protein | uniqAntib_1098 | -0.19 | -0.28 | -0.11 | 0.02 | -0.26 | 0 | 0.16875 | 1.12408 | 2.37242 | 0.00915 | 1 | 0.2984 | 1.22978 | 0.78765 | 0.00702 | 0 | 0.01783 | 1.01243 | 1.78661 | 0.08939 | 0 | 0 |
| 1391529_at | Gpx4 | glutathione peroxidase 4 | uniqAntib_1098 | 0.08 | 0.04 | -0.04 | -0.32 | -0.07 | 0 | -0.088 | -1.0629 | 1.20662 | 0.27901 | 1 | -0.3645 | -1.2875 | 2.47836 | 0.00972 | 0 | -0.1102 | -1.0793 | 1.28801 | 0.19921 | 0 | 0 |
| 1391561_at | --- | --- | uniqAntib_1098 | -0.41 | -0.21 | -0.04 | 0.16 | -0.06 | 0 | 0.17019 | 1.12521 | 2.13625 | 0.02494 | 1 | 0.37617 | 1.29789 | 0.71152 | 0.00529 | 0 | 0.15786 | 1.11563 | 1.8743 | 0.07781 | 0 | 0 |
| 1391586_at | --- | --- | uniqAntib_1098 | -0.09 | 0.24 | 0.11 | -0.10 | 0.17 | 0 | -0.1282 | -1.0929 | 1.31975 | 0.23166 | 1 | -0.3348 | -1.2612 | 2.02281 | 0.01691 | 0 | -0.0726 | -1.0516 | 1.19486 | 0.24905 | 0 | 0 |
| 1391588_at | --- | --- | uniqAntib_1098 | -0.09 | -0.05 | -0.13 | -0.35 | -0.12 | 0 | -0.0787 | -1.056 | 0.99915 | 0.42538 | 1 | -0.2929 | -1.2251 | 1.65704 | 0.02989 | 0 | -0.0641 | -1.0454 | 0.97608 | 0.46907 | 0 | 0 |
| 1391594_at | Samd8 | sterile alpha motif domain containing 8 | uniqAntib_1098 | -0.11 | 0.20 | 0.24 | 0.46 | 0.08 | 0 | 0.03907 | 1.02745 | 1.98367 | 0.04452 | 1 | 0.26409 | 1.20088 | 0.81729 | 0.00767 | 0 | -0.1184 | -1.0855 | 2.89798 | 0.02031 | 0 | 0 |
| 1391600_at | --- | --- | uniqAntib_1098 | -0.05 | 0.19 | 0.16 | 0.56 | 0.12 | 0 | -0.0335 | -1.0235 | 1.31192 | 0.23442 | 1 | 0.36724 | 1.28988 | 0.71404 | 0.00523 | 0 | -0.0715 | -1.0508 | 1.41519 | 0.15281 | 0 | 0 |
| 1391636_at | --- | --- | uniqAntib_1098 | -0.05 | 0.12 | 0.17 | 0.58 | 0.14 | 0 | 0.04807 | 1.03388 | 1.71038 | 0.09801 | 1 | 0.4594 | 1.37497 | 0.57011 | 0.00323 | 0 | 0.02233 | 1.0156 | 1.03264 | 0.39185 | 0 | 0 |
| 1391650_at | Dido1 | death inducer-obliterator 1 | uniqAntib_1098 | -0.22 | -0.18 | 0.08 | 0.21 | 0.05 | 0 | 0.25289 | 1.19159 | 2.159 | 0.02298 | 1 | 0.38534 | 1.30617 | 0.70914 | 0.00504 | 0 | 0.22808 | 1.17128 | 2.36439 | 0.02425 | 0 | 0 |
| 1391663_at | --- | --- | uniqAntib_1098 | -0.29 | -0.59 | -0.55 | -0.30 | -0.50 | 0 | 0.03829 | 1.0269 | 1.48332 | 0.16145 | 1 | 0.29053 | 1.22309 | 0.92641 | 0.01022 | 0 | 0.08828 | 1.0631 | 2.08719 | 0.0547 | 0 | 0 |
| 1391674_at | --- | --- | uniqAntib_1098 | -0.05 | 0.16 | 0.19 | 0.44 | 0.19 | 0 | 0.03538 | 1.02483 | 1.05798 | 0.38494 | 1 | 0.27994 | 1.21415 | 1.25572 | 0.03594 | 0 | 0.0336 | 1.02356 | 1.0025 | 0.42561 | 0 | 0 |
| 1391676_at | --- | --- | uniqAntib_1098 | -0.22 | -0.19 | -0.15 | 0.18 | -0.36 | 0 | 0.04552 | 1.03205 | 1.36636 | 0.20571 | 1 | 0.37541 | 1.29721 | 0.76118 | 0.00644 | 0 | -0.1695 | -1.1246 | 1.94592 | 0.05719 | 0 | 0 |
| 1391700_at | --- | --- | uniqAntib_1098 | -0.07 | -0.11 | -0.07 | -0.41 | -0.15 | 0 | 0.04083 | 1.0287 | 1.18077 | 0.29505 | 1 | -0.2997 | -1.2309 | 1.88809 | 0.01943 | 0 | -0.0485 | -1.0342 | 1.16588 | 0.26816 | 0 | 0 |
| 1391746_at | --- | --- | uniqAntib_1098 | 0.07 | 0.02 | 0.00 | -0.24 | -0.19 | 0 | -0.0193 | -1.0135 | 1.25346 | 0.25594 | 1 | -0.2642 | -1.201 | 1.92694 | 0.01822 | 0 | -0.2058 | -1.1533 | 2.30528 | 0.03469 | 0 | 0 |
| 1391767_at | RGD1310495 | similar to KIAA1919 protein | uniqAntib_1098 | 0.02 | 0.05 | -0.10 | -0.23 | -0.02 | 0 | -0.1469 | -1.1072 | 1.90279 | 0.10995 | 1 | -0.2804 | -1.2145 | 1.54194 | 0.03857 | 0 | -0.0635 | -1.045 | 1.16794 | 0.26732 | 0 | 0 |
| 1391771_at | Atp2a2 | ATPase, Ca++ transporting, cardiac muscle, slow twi | uniqAntib_1098 | -0.02 | -0.07 | 0.20 | 0.24 | 0.03 | 0 | 0.26157 | 1.19878 | 2.10038 | 0.02668 | 1 | 0.30937 | 1.23916 | 1.16787 | 0.02366 | 0 | 0.09256 | 1.06626 | 1.41578 | 0.17718 | 0 | 0 |
| 1391773_at | Srp54 | signal recognition particle 54A | uniqAntib_1098 | 0.02 | -0.11 | 0.04 | 0.17 | 0.00 | 0 | 0.14955 | 1.10922 | 1.43889 | 0.17827 | 1 | 0.28373 | 1.21734 | 1.27202 | 0.04569 | 0 | 0.11061 | 1.07968 | 1.22827 | 0.25708 | 0 | 0 |
| 1391788_at | --- | --- | uniqAntib_1098 | -0.10 | 0.03 | 0.04 | 0.30 | 0.01 | 0 | 0.0125 | 1.0087 | 1.04258 | 0.38543 | 1 | 0.27234 | 1.20777 | 1.22078 | 0.03184 | 0 | -0.0221 | -1.0154 | 1.02975 | 0.38592 | 0 | 0 |
| 1391791_at | Acer2 | alkaline ceramidase 2 | uniqAntib_1098 | 0.06 | 0.16 | -0.02 | -0.14 | -0.04 | 0 | -0.1799 | -1.1328 | 1.54348 | 0.16631 | 1 | -0.3018 | -1.2327 | 1.51551 | 0.03962 | 0 | -0.2071 | -1.1544 | 1.58453 | 0.10623 | 0 | 0 |
| 1391810_at | Rbm20 | RNA binding motif protein 20 | uniqAntib_1098 | 0.00 | 0.01 | -0.08 | -0.28 | -0.02 | 0 | -0.0924 | -1.0661 | 1.45108 | 0.18827 | 1 | -0.2872 | -1.2202 | 1.57759 | 0.03394 | 0 | -0.0281 | -1.0197 | 1.00838 | 0.41711 | 0 | 0 |
| 1391828_at | --- | --- | uniqAntib_1098 | -0.03 | 0.51 | 0.56 | 0.85 | 0.48 | 0 | 0.05278 | 1.03726 | 1.49412 | 0.15941 | 1 | 0.34654 | 1.27151 | 0.75854 | 0.00634 | 0 | -0.023 | -1.0161 | 1.28583 | 0.20003 | 0 | 0 |
| 1391860_at | --- | --- | uniqAntib_1098 | 0.10 | 0.12 | -0.03 | -0.22 | 0.23 | 0 | -0.1483 | -1.1083 | 1.91587 | 0.10855 | 1 | -0.3451 | -1.2703 | 2.08135 | 0.01385 | 0 | 0.11257 | 1.08115 | 1.28313 | 0.23068 | 0 | 0 |
| 1391869_at | --- | --- | uniqAntib_1098 | -0.03 | -0.02 | 0.01 | -0.30 | -0.13 | 0 | 0.03005 | 1.02104 | 0.92014 | 0.55977 | 1 | -0.2865 | -1.2196 | 1.50684 | 0.04191 | 0 | -0.1149 | -1.0829 | 1.18447 | 0.25659 | 0 | 0 |
| 1391870_at | --- | --- | uniqAntib_1098 | 0.13 | 0.07 | -0.06 | -0.20 | 0.07 | 0 | -0.1259 | -1.0912 | 1.449 | 0.18852 | 1 | -0.2719 | -1.2074 | 1.64491 | 0.03115 | 0 | 0.00318 | 1.00221 | 0.96759 | 0.46822 | 0 | 0 |
| 1391883_at | Wwox | WW domain-containing oxidoreductase | uniqAntib_1098 | -0.08 | -0.17 | -0.12 | -0.46 | -0.07 | 0 | 0.05159 | 1.03641 | 1.01963 | 0.42465 | 1 | -0.288 | -1.2209 | 1.74827 | 0.02637 | 0 | 0.09899 | 1.07102 | 1.36549 | 0.19363 | 0 | 0 |
| 1391889_at | --- | --- | uniqAntib_1098 | -0.13 | -0.27 | -0.38 | 0.00 | -0.35 | 0 | -0.1097 | -1.079 | 1.77076 | 0.12715 | 1 | 0.26651 | 1.20289 | 1.13983 | 0.02203 | 0 | -0.077 | -1.0548 | 1.38482 | 0.16218 | 0 | 0 |
| 1391890_at | Bcas3 | breast carcinoma amplified sequence 3 | uniqAntib_1098 | 0.01 | 0.17 | 0.10 | -0.11 | 0.01 | 0 | -0.0739 | -1.0526 | 1.61932 | 0.15064 | 1 | -0.281 | -1.2151 | 1.84131 | 0.02037 | 0 | -0.1694 | -1.1246 | 1.93721 | 0.05656 | 0 | 0 |
| 1391907_at | Nags | N-acetylglutamate synthase | uniqAntib_1098 | -0.02 | -0.13 | -0.03 | -0.44 | -0.05 | 0 | 0.09966 | 1.07152 | 1.23454 | 0.26113 | 1 | -0.3136 | -1.2428 | 2.32252 | 0.01098 | 0 | 0.07799 | 1.05555 | 1.30517 | 0.21954 | 0 | 0 |
| 1391958_at | --- | --- | uniqAntib_1098 | 0.07 | 0.47 | 0.48 | 0.86 | 0.56 | 0 | 0.01685 | 1.01175 | 1.26416 | 0.24792 | 1 | 0.39412 | 1.31414 | 0.902 | 0.00914 | 0 | 0.09634 | 1.06906 | 1.44389 | 0.16821 | 0 | 0 |
| 1392040_at | Sass6 | spindle assembly 6 homolog (C. elegans) | uniqAntib_1098 | -0.06 | -0.26 | -0.13 | 0.08 | -0.21 | 0 | 0.13678 | 1.09945 | 1.97321 | 0.04511 | 1 | 0.34529 | 1.2704 | 0.89256 | 0.00895 | 0 | 0.05028 | 1.03547 | 1.60917 | 0.12512 | 0 | 0 |
| 1392087_at | --- | --- | uniqAntib_1098 | -0.12 | -0.16 | 0.08 | 0.11 | -0.01 | 0 | 0.24254 | 1.18307 | 1.94942 | 0.04996 | 1 | 0.2728 | 1.20815 | 1.24017 | 0.03374 | 0 | 0.15065 | 1.11007 | 1.42551 | 0.17528 | 0 | 0 |
| 1392088_at | Pde8b | phosphodiesterase 8B | uniqAntib_1098 | 0.12 | 0.13 | -0.04 | -0.22 | 0.04 | 0 | -0.1723 | -1.1268 | 1.70148 | 0.13601 | 1 | -0.3498 | -1.2744 | 2.00906 | 0.01718 | 0 | -0.0911 | -1.0652 | 1.12065 | 0.29891 | 0 | 0 |
| 1392121_at | --- | --- | uniqAntib_1098 | -0.16 | -0.11 | -0.06 | -0.47 | -0.22 | 0 | 0.05123 | 1.03615 | 1.16882 | 0.30251 | 1 | -0.3642 | -1.2872 | 2.59919 | 0.00928 | 0 | -0.1088 | -1.0783 | 1.44088 | 0.14409 | 0 | 0 |
| 1392155_at | --- | --- | uniqAntib_1098 | -0.12 | -0.36 | -0.26 | 0.05 | -0.40 | 0 | 0.09955 | 1.07144 | 1.93318 | 0.05632 | 1 | 0.41424 | 1.3326 | 0.59753 | 0.00357 | 0 | -0.0341 | -1.0239 | 1.37712 | 0.1646 | 0 | 0 |
| 1392183_at | --- | --- | uniqAntib_1098 | -0.21 | -0.32 | -0.14 | 0.14 | -0.15 | 0 | 0.17584 | 1.12962 | 2.09602 | 0.0308 | 1 | 0.46097 | 1.37647 | 0.4983 | 0.00245 | 0 | 0.17168 | 1.12637 | 2.10749 | 0.05266 | 0 | 0 |
| 1392214_at | --- | --- | uniqAntib_1098 | -0.04 | -0.02 | -0.08 | 0.37 | 0.07 | 0 | -0.0661 | -1.0469 | 1.37786 | 0.21151 | 1 | 0.38929 | 1.30975 | 0.94336 | 0.01056 | 0 | 0.08309 | 1.05929 | 1.52453 | 0.14579 | 0 | 0 |
| 1392235_at | --- | --- | uniqAntib_1098 | 0.09 | 0.12 | 0.12 | -0.15 | -0.01 | 0 | -0.0072 | -1.005 | 1.13423 | 0.31898 | 1 | -0.2702 | -1.206 | 1.50621 | 0.04093 | 0 | -0.1353 | -1.0984 | 1.41334 | 0.15288 | 0 | 0 |
| 1392259_at | Hectd1 | HECT domain containing E3 ubiquitin protein ligase | uniqAntib_1098 | -0.08 | 0.01 | 0.17 | 0.28 | 0.14 | 0 | 0.15515 | 1.11354 | 2.00266 | 0.04212 | 1 | 0.27205 | 1.20752 | 0.97764 | 0.01383 | 0 | 0.12454 | 1.09016 | 2.05045 | 0.06132 | 0 | 0 |
| 1392300_at | --- | --- | uniqAntib_1098 | 0.04 | 0.14 | 0.07 | -0.21 | -0.01 | 0 | -0.0683 | -1.0485 | 1.09636 | 0.34291 | 1 | -0.3443 | -1.2695 | 1.99068 | 0.0178 | 0 | -0.1487 | -1.1086 | 1.23012 | 0.22843 | 0 | 0 |
| 1392313_at | Lrfn4 | leucine rich repeat and fibronectin type III domain c | uniqAntib_1098 | -0.08 | -0.05 | -0.03 | -0.35 | -0.17 | 0 | 0.01846 | 1.01288 | 1.13173 | 0.32046 | 1 | -0.3087 | -1.2386 | 1.84353 | 0.02015 | 0 | -0.1216 | -1.088 | 1.44922 | 0.14283 | 0 | 0 |
| 1392343_at | --- | --- | uniqAntib_1098 | 0.05 | -0.23 | -0.21 | 0.11 | -0.23 | 0 | 0.02017 | 1.01408 | 0.88133 | 0.65856 | 1 | 0.34779 | 1.27261 | 1.0205 | 0.01585 | 0 | 0.00243 | 1.00169 | 0.96117 | 0.49491 | 0 | 0 |

| Affymetrix Probeset ID | Gene Symbol | Gene Title | Figure 6 B Venn n =2021 | Mean BN log2 intensity autologous n=8 | Mean log2 intensity day 30 DR +/+ ND n=4 | Mean log2 intensity day 30 DR+/+ HCD n=4 | Mean log2 intensity day 30 B/S n=4 | Mean log2 intensity day 30 F+/+ ND n=4 | DR+/+ HCD vs DR+/+ ND n=380 1=yes 0=no | Log2 ratio DR+/+ HCD vs DR+/+ ND | Fold-Change DR+/+ HCD vs DR+/+ ND | RP-FDR 4DR30 HCD vs 4DR30 Reg | p value DR+/+ HCD vs DR+/+ ND | DR+/+ B/S vs DR+/+ ND n=1,330 1=yes 0=no | Log2 ratio DR+/+ B/S vs DR+/+ ND | Fold-Change DR+/+ B/S vs DR+/+ ND | RP-FDR 4DR30 Antib vs 4DR30 Reg | p value DR+/+ B/S vs DR+/+ ND | DR+/+ ND vs F+/+ ND n=398 1=yes 0=no | Log2 ratio DR+/+ ND vs F+/+ ND | Fold-Change DR+/+ ND vs F+/+ ND | RP-FDR 4RT1uu 30Reg vs 4DR30 Reg | p value DR+/+ ND vs F+/+ ND | DR+/+ Longitudinal n=228 1=yes 0=no | Figure 6F |
|---|---|---|---|---|---|---|---|---|---|---|---|---|---|---|---|---|---|---|---|---|---|---|---|---|---|
| 1392345_at | --- | --- | uniqAntib_1098 | 0.05 | 0.10 | -0.01 | -0.30 | 0.10 | 0 | -0.1136 | -1.0819 | 1.28298 | 0.24509 | 1 | -0.3994 | -1.319 | 2.48917 | 0.00968 | 0 | 0.00445 | 1.00309 | 0.89506 | 0.65281 | 0 | 0 |
| 1392359_at | --- | --- | uniqAntib_1098 | -0.21 | 0.05 | -0.05 | 0.40 | -0.01 | 0 | -0.101 | -1.0725 | 1.94661 | 0.10315 | 1 | 0.34961 | 1.27422 | 0.96786 | 0.01251 | 0 | -0.0698 | -1.0496 | 1.28542 | 0.20026 | 0 | 0 |
| 1392371_at | --- | --- | uniqAntib_1098 | -0.04 | 0.08 | 0.00 | -0.27 | -0.15 | 0 | -0.0819 | -1.0584 | 1.60445 | 0.1538 | 1 | -0.3469 | -1.2718 | 2.12 | 0.01357 | 0 | -0.2314 | -1.1739 | 1.97869 | 0.05357 | 0 | 0 |
| 1392373_at | --- | --- | uniqAntib_1098 | -0.02 | -0.04 | -0.09 | 0.26 | -0.04 | 0 | -0.0446 | -1.0314 | 1.6322 | 0.14911 | 1 | 0.30111 | 1.23209 | 0.93424 | 0.01006 | 0 | 0.00126 | 1.00087 | 1.21901 | 0.26251 | 0 | 0 |
| 1392378_at | Klhdc3 | kelch domain containing 3 | uniqAntib_1098 | -0.04 | 0.19 | 0.32 | 0.47 | 0.37 | 0 | 0.13051 | 1.09468 | 1.64321 | 0.11751 | 1 | 0.28176 | 1.21568 | 1.20302 | 0.02766 | 0 | 0.17522 | 1.12914 | 2.03004 | 0.06234 | 0 | 0 |
| 1392406_at | Ipp | intracisternal A particle-promoted polypeptide | uniqAntib_1098 | -0.04 | -0.06 | -0.02 | 0.21 | -0.15 | 0 | 0.03937 | 1.02766 | 1.38899 | 0.19688 | 1 | 0.26811 | 1.20423 | 1.27687 | 0.04106 | 0 | -0.0956 | -1.0685 | 1.69797 | 0.09315 | 0 | 0 |
| 1392418_at | Mapk8 | mitogen-activated protein kinase 8 | uniqAntib_1098 | 0.05 | 0.07 | 0.06 | 0.36 | 0.12 | 0 | -0.0105 | -1.0073 | 1.05456 | 0.37155 | 1 | 0.28392 | 1.2175 | 1.26692 | 0.04591 | 0 | 0.04382 | 1.03084 | 0.96997 | 0.46467 | 0 | 0 |
| 1392425_x_at | Luc7l | LUC7-like (S. cerevisiae) | uniqAntib_1098 | -0.13 | -0.11 | -0.14 | 0.23 | -0.08 | 0 | -0.0362 | -1.0254 | 1.03175 | 0.39609 | 1 | 0.33875 | 1.26466 | 1.01268 | 0.01504 | 0 | 0.02844 | 1.01991 | 0.98331 | 0.44848 | 0 | 0 |
| 1392430_at | --- | --- | uniqAntib_1098 | -0.09 | -0.03 | -0.08 | -0.34 | -0.12 | 0 | -0.0447 | -1.0315 | 1.47052 | 0.182 | 1 | -0.3107 | -1.2403 | 2.66012 | 0.00727 | 0 | -0.0914 | -1.0654 | 1.60245 | 0.10409 | 0 | 0 |
| 1392529_at | Spry4 | sprouty homolog 4 (Drosophila) | uniqAntib_1098 | 0.02 | 0.18 | -0.02 | -0.10 | -0.05 | 0 | -0.2018 | -1.1501 | 1.93381 | 0.10509 | 1 | -0.284 | -1.2175 | 1.46394 | 0.04453 | 0 | -0.2319 | -1.1743 | 1.75274 | 0.08195 | 0 | 0 |
| 1392568_at | RGD1562272 | similar to TAF11 RNA polymerase II, TATA box bindi | uniqAntib_1098 | 0.07 | 0.08 | -0.05 | -0.19 | -0.11 | 0 | -0.1389 | -1.101 | 1.96888 | 0.10085 | 1 | -0.272 | -1.2075 | 2.04063 | 0.0166 | 0 | -0.1918 | -1.1422 | 2.11578 | 0.04436 | 0 | 0 |
| 1392645_at | --- | --- | uniqAntib_1098 | -0.05 | 0.04 | -0.05 | -0.24 | -0.18 | 0 | -0.0916 | -1.0655 | 1.18601 | 0.2903 | 1 | -0.2814 | -1.2154 | 1.61152 | 0.03311 | 0 | -0.222 | -1.1663 | 1.74358 | 0.08668 | 0 | 0 |
| 1392653_at | LOC1009103 | uncharacterized LOC100910396 | uniqAntib_1098 | -0.05 | 0.28 | 0.32 | 0.60 | 0.23 | 0 | 0.03217 | 1.02255 | 1.05153 | 0.39097 | 1 | 0.31248 | 1.24184 | 1.18472 | 0.02625 | 0 | -0.057 | -1.0403 | 1.1737 | 0.26464 | 0 | 0 |
| 1392662_at | Cisd2 | CDGSH iron sulfur domain 2 | uniqAntib_1098 | -0.11 | -0.22 | -0.13 | 0.08 | -0.26 | 0 | 0.0874 | 1.06246 | 1.28867 | 0.23682 | 1 | 0.29706 | 1.22864 | 1.20859 | 0.03012 | 0 | -0.044 | -1.031 | 1.08725 | 0.32601 | 0 | 0 |
| 1392675_at | --- | --- | uniqAntib_1098 | -0.06 | -0.35 | -0.12 | 0.09 | -0.32 | 0 | 0.23092 | 1.17359 | 2.33581 | 0.01021 | 1 | 0.44183 | 1.35833 | 0.42104 | 0.00183 | 0 | 0.03046 | 1.02134 | 1.7144 | 0.10105 | 0 | 0 |
| 1392705_at | Slc9a6 | solute carrier family 9, subfamily A (NHE6, cation pr | uniqAntib_1098 | -0.08 | -0.13 | -0.16 | 0.18 | -0.06 | 0 | -0.0265 | -1.0185 | 1.08044 | 0.35235 | 1 | 0.31104 | 1.2406 | 1.0468 | 0.01683 | 0 | 0.07037 | 1.04999 | 1.22335 | 0.25971 | 0 | 0 |
| 1392752_at | LOC1003629 | CG3918-like /// CG3918-like /// SREK1-interacting p | uniqAntib_1098 | -0.16 | -0.33 | -0.48 | -0.03 | -0.50 | 0 | -0.1461 | -1.1066 | 1.94521 | 0.10321 | 1 | 0.303 | 1.23371 | 1.14055 | 0.02197 | 0 | -0.1718 | -1.1265 | 1.60202 | 0.10447 | 0 | 0 |
| 1392794_at | --- | --- | uniqAntib_1098 | -0.14 | -0.25 | -0.20 | 0.21 | -0.09 | 0 | 0.04791 | 1.03377 | 1.10744 | 0.34503 | 1 | 0.46158 | 1.37705 | 0.38756 | 0.00158 | 0 | 0.15915 | 1.11663 | 1.53763 | 0.14101 | 0 | 0 |
| 1392860_at | --- | --- | uniqAntib_1098 | -0.29 | 0.03 | 0.24 | 0.41 | -0.07 | 0 | 0.20461 | 1.15237 | 2.09648 | 0.03101 | 1 | 0.38119 | 1.30242 | 0.49888 | 0.00244 | 0 | -0.1031 | -1.0741 | 1.70629 | 0.09179 | 0 | 0 |
| 1392864_at | Arhgap5 | Rho GTPase activating protein 5 | uniqAntib_1098 | -0.06 | -0.28 | -0.24 | 0.03 | -0.32 | 0 | 0.03448 | 1.02419 | 1.77296 | 0.07742 | 1 | 0.30745 | 1.23752 | 0.84578 | 0.00832 | 0 | -0.038 | -1.0267 | 1.44653 | 0.1435 | 0 | 0 |
| 1392887_at | --- | --- | uniqAntib_1098 | -0.08 | -0.11 | -0.08 | 0.18 | -0.06 | 0 | 0.03179 | 1.02228 | 1.23825 | 0.2594 | 1 | 0.29467 | 1.2266 | 1.26356 | 0.03872 | 0 | 0.04806 | 1.03388 | 0.96534 | 0.47021 | 0 | 0 |
| 1392999_at | --- | --- | uniqAntib_1098 | 0.15 | -0.07 | 0.17 | 0.31 | -0.15 | 0 | 0.24066 | 1.18153 | 1.94764 | 0.04973 | 1 | 0.38665 | 1.30735 | 0.7735 | 0.00682 | 0 | -0.0802 | -1.0572 | 1.05029 | 0.36255 | 0 | 0 |
| 1393091_at | Mier3 | mesoderm induction early response 1, family memb | uniqAntib_1098 | -0.11 | -0.27 | -0.23 | 0.04 | -0.26 | 0 | 0.04567 | 1.03217 | 1.05392 | 0.38708 | 1 | 0.30978 | 1.23952 | 1.12566 | 0.02078 | 0 | 0.01454 | 1.01013 | 1.02492 | 0.40303 | 0 | 0 |
| 1393113_at | Arhgap1 | Rho GTPase activating protein 1 | uniqAntib_1098 | 0.04 | 0.06 | 0.03 | 0.33 | 0.03 | 0 | -0.0289 | -1.0203 | 0.96104 | 0.46132 | 1 | 0.26306 | 1.20002 | 1.22039 | 0.03202 | 0 | -0.0286 | -1.02 | 0.92588 | 0.56061 | 0 | 0 |
| 1393159_at | --- | --- | uniqAntib_1098 | -0.12 | -0.27 | -0.21 | 0.01 | -0.27 | 0 | 0.05868 | 1.04151 | 1.35999 | 0.20851 | 1 | 0.27523 | 1.21018 | 1.25079 | 0.03571 | 0 | -0.0042 | -1.0029 | 1.06591 | 0.34532 | 0 | 0 |
| 1393238_at | --- | --- | uniqAntib_1098 | -0.02 | 0.03 | 0.16 | 0.32 | 0.13 | 0 | 0.13323 | 1.09675 | 1.19241 | 0.28692 | 1 | 0.29858 | 1.22993 | 1.24592 | 0.03506 | 0 | 0.10548 | 1.07585 | 1.13696 | 0.30794 | 0 | 0 |
| 1393261_at | --- | --- | uniqAntib_1098 | -0.07 | 0.04 | -0.05 | -0.27 | -0.11 | 0 | -0.094 | -1.0673 | 1.57919 | 0.15797 | 1 | -0.3179 | -1.2465 | 1.98353 | 0.01588 | 0 | -0.151 | -1.1104 | 1.54517 | 0.11244 | 0 | 0 |
| 1393265_at | --- | --- | uniqAntib_1098 | -0.04 | -0.19 | -0.07 | 0.10 | 0.00 | 0 | 0.119 | 1.08598 | 1.7615 | 0.08406 | 1 | 0.28993 | 1.22258 | 1.17221 | 0.0251 | 0 | 0.18825 | 1.13938 | 2.04886 | 0.06173 | 0 | 0 |
| 1393293_at | Chfr | checkpoint with forkhead and ring finger domains, E | uniqAntib_1098 | -0.09 | -0.14 | -0.14 | 0.13 | -0.09 | 0 | 0.00578 | 1.00402 | 0.91034 | 0.5817 | 1 | 0.27025 | 1.20601 | 1.28037 | 0.04817 | 0 | 0.05482 | 1.03873 | 1.03871 | 0.38517 | 0 | 0 |
| 1393321_at | --- | --- | uniqAntib_1098 | -0.03 | -0.18 | -0.16 | 0.09 | -0.07 | 0 | 0.02209 | 1.01543 | 0.9602 | 0.49488 | 1 | 0.26935 | 1.20527 | 1.27676 | 0.04943 | 0 | 0.10903 | 1.0785 | 1.22618 | 0.25743 | 0 | 0 |
| 1393342_at | --- | --- | uniqAntib_1098 | 0.00 | 0.19 | 0.16 | -0.10 | 0.03 | 0 | -0.0286 | -1.02 | 1.10239 | 0.33828 | 1 | -0.2936 | -1.2257 | 1.64584 | 0.0308 | 0 | -0.1642 | -1.1206 | 1.53364 | 0.11485 | 0 | 0 |
| 1393356_at | --- | --- | uniqAntib_1098 | 0.02 | -1.32 | -1.29 | -0.99 | -1.20 | 0 | 0.02528 | 1.01768 | 2.26025 | 0.01737 | 1 | 0.32421 | 1.25198 | 0.45417 | 0.0021 | 0 | 0.11231 | 1.08096 | 2.37573 | 0.02452 | 0 | 0 |
| 1393379_a_at | Blk | B lymphoid tyrosine kinase | uniqAntib_1098 | -0.04 | 0.42 | 0.48 | 0.72 | 0.42 | 0 | 0.06588 | 1.04672 | 1.16108 | 0.30801 | 1 | 0.30733 | 1.23741 | 1.18245 | 0.02593 | 0 | 0.00399 | 1.00277 | 0.98251 | 0.46091 | 0 | 0 |
| 1393383_at | Arhgdig | Rho GDP dissociation inhibitor (GDI) gamma | uniqAntib_1098 | 0.03 | -0.01 | -0.05 | -0.41 | -0.15 | 0 | -0.0486 | -1.0342 | 1.15822 | 0.30409 | 1 | -0.3999 | -1.3194 | 2.64126 | 0.00739 | 0 | -0.1398 | -1.1017 | 1.29341 | 0.19705 | 0 | 0 |
| 1393414_at | LOC689176 | similar to transmembrane protein 64 | uniqAntib_1098 | -0.15 | -0.11 | -0.09 | 0.20 | -0.03 | 0 | 0.01827 | 1.01274 | 1.39553 | 0.19507 | 1 | 0.31453 | 1.2436 | 0.99264 | 0.01427 | 0 | 0.08077 | 1.05758 | 1.53783 | 0.14128 | 0 | 0 |
| 1393427_s_at | Egfl7 | EGF-like-domain, multiple 7 | uniqAntib_1098 | -0.10 | 0.05 | 0.03 | -0.25 | 0.01 | 0 | -0.02 | -1.014 | 1.3101 | 0.23642 | 1 | -0.3017 | -1.2326 | 1.71877 | 0.02658 | 0 | -0.0452 | -1.0318 | 1.08978 | 0.32344 | 0 | 0 |
| 1393428_at | --- | --- | uniqAntib_1098 | -0.06 | -0.18 | 0.02 | 0.15 | 0.04 | 0 | 0.19236 | 1.14263 | 1.77922 | 0.07603 | 1 | 0.32589 | 1.25344 | 1.18125 | 0.02469 | 0 | 0.22023 | 1.16492 | 2.07233 | 0.05871 | 0 | 0 |
| 1393550_at | Crem | cAMP responsive element modulator | uniqAntib_1098 | -0.11 | -0.87 | -0.90 | -0.47 | -0.71 | 0 | -0.0293 | -1.0205 | 1.0783 | 0.35339 | 1 | 0.39579 | 1.31567 | 0.72308 | 0.00574 | 0 | 0.16081 | 1.11792 | 1.74279 | 0.09504 | 0 | 0 |
| 1393565_at | RGD1563441 | similar to RIKEN cDNA A030009H04 | uniqAntib_1098 | -0.01 | -0.05 | 0.01 | -0.31 | -0.16 | 0 | 0.05654 | 1.03997 | 1.02003 | 0.42443 | 1 | -0.2657 | -1.2022 | 1.51484 | 0.03985 | 0 | -0.1189 | -1.0859 | 1.34857 | 0.17536 | 0 | 0 |
| 1393584_at | Tnfrsf21 | tumor necrosis factor receptor superfamily, membe | uniqAntib_1098 | 0.19 | 0.18 | 0.26 | 0.48 | 0.30 | 0 | 0.0803 | 1.05724 | 1.41566 | 0.1855 | 1 | 0.30541 | 1.23577 | 1.24801 | 0.03463 | 0 | 0.12914 | 1.09364 | 1.34365 | 0.20324 | 0 | 0 |
| 1393603_at | Camp | cathelicidin antimicrobial peptide | uniqAntib_1098 | 0.08 | 0.04 | -0.09 | -0.26 | -0.01 | 0 | -0.1336 | -1.097 | 1.4168 | 0.19585 | 1 | -0.3028 | -1.2335 | 1.64672 | 0.03055 | 0 | -0.046 | -1.0324 | 1.00167 | 0.4298 | 0 | 0 |
| 1393649_at | --- | --- | uniqAntib_1098 | -0.06 | 0.30 | 0.54 | 0.58 | 0.36 | 0 | 0.24186 | 1.18251 | 2.0541 | 0.03514 | 1 | 0.28478 | 1.21822 | 1.15695 | 0.02292 | 0 | 0.05859 | 1.04145 | 1.45827 | 0.16318 | 0 | 0 |
| 1393653_at | --- | --- | uniqAntib_1098 | 0.01 | -0.11 | 0.01 | 0.55 | 0.10 | 0 | 0.12559 | 1.09095 | 1.19777 | 0.28328 | 1 | 0.66913 | 1.59011 | 0.0505 | 9.7E-05 | 0 | 0.2098 | 1.15653 | 1.804 | 0.08684 | 0 | 0 |
| 1393655_at | Rad54l | RAD54 like (S. cerevisiae) | uniqAntib_1098 | -0.06 | 0.07 | 0.05 | -0.25 | 0.03 | 0 | -0.0181 | -1.0127 | 1.60906 | 0.1531 | 1 | -0.3271 | -1.2545 | 2.59989 | 0.00752 | 0 | -0.038 | -1.0267 | 1.82464 | 0.07498 | 0 | 0 |
| 1393668_at | Pex11g | peroxisomal biogenesis factor 11 gamma | uniqAntib_1098 | 0.22 | 0.07 | 0.02 | -0.33 | 0.04 | 0 | -0.0575 | -1.0407 | 1.49701 | 0.17637 | 1 | -0.4024 | -1.3217 | 3.77 | 0.00206 | 0 | -0.033 | -1.0232 | 1.47382 | 0.1363 | 0 | 0 |
| 1393686_at | --- | --- | uniqAntib_1098 | -0.06 | 0.23 | 0.38 | 0.59 | 0.30 | 0 | 0.15042 | 1.10989 | 1.50613 | 0.15561 | 1 | 0.36506 | 1.28794 | 0.97113 | 0.01299 | 0 | 0.07393 | 1.05258 | 1.25683 | 0.24232 | 0 | 0 |
| 1393702_at | Xrn2 | 5'-3' exoribonuclease 2 | uniqAntib_1098 | -0.05 | -0.29 | -0.12 | -0.01 | -0.30 | 0 | 0.16166 | 1.11857 | 2.19974 | 0.0215 | 1 | 0.27163 | 1.20717 | 1.01208 | 0.0152 | 0 | -0.0154 | -1.0107 | 1.12894 | 0.29176 | 0 | 0 |
| 1393727_at | --- | --- | uniqAntib_1098 | -0.04 | -0.15 | -0.08 | 0.15 | -0.15 | 0 | 0.07455 | 1.05303 | 1.18281 | 0.29351 | 1 | 0.30108 | 1.23206 | 1.21208 | 0.03036 | 0 | 8.7E-05 | 1.00006 | 0.98932 | 0.45055 | 0 | 0 |
| 1393798_at | Atrx | alpha thalassemia/mental retardation syndrome X-l | uniqAntib_1098 | 0.00 | 0.03 | 0.21 | 0.44 | -0.02 | 0 | 0.17895 | 1.13206 | 2.95379 | 0.00275 | 1 | 0.41176 | 1.33031 | 0.1993 | 0.00064 | 0 | -0.0514 | -1.0363 | 3.01241 | 0.01685 | 0 | 0 |
| 1393809_at | Traf6 | TNF receptor-associated factor 6, E3 ubiquitin prote | uniqAntib_1098 | 0.10 | -0.03 | 0.09 | 0.24 | 0.06 | 0 | 0.12623 | 1.09144 | 2.28131 | 0.01907 | 1 | 0.26959 | 1.20546 | 0.95095 | 0.01095 | 0 | 0.08947 | 1.06398 | 2.28503 | 0.03564 | 0 | 0 |
| 1393818_s_at | Fam71e1 | family with sequence similarity 71, member E1 | uniqAntib_1098 | -0.08 | 0.06 | -0.03 | -0.28 | -0.03 | 0 | -0.0842 | -1.0601 | 1.43585 | 0.19133 | 1 | -0.3386 | -1.2645 | 1.89877 | 0.01929 | 0 | -0.0907 | -1.0649 | 1.40809 | 0.15399 | 0 | 0 |
| 1393845_a_at | LOC10090961 | transmembrane channel-like protein 4-like /// trans | uniqAntib_1098 | 0.05 | -0.11 | -0.11 | -0.47 | -0.19 | 0 | -0.0025 | -1.0017 | 1.53429 | 0.16853 | 1 | -0.3587 | -1.2823 | 4.06462 | 0.0017 | 0 | -0.0731 | -1.052 | 1.89257 | 0.06286 | 0 | 0 |
| 1393889_x_at | --- | --- | uniqAntib_1098 | -0.19 | 0.10 | 0.09 | -0.21 | -0.09 | 0 | -0.0055 | -1.0038 | 0.98485 | 0.43686 | 1 | -0.3113 | -1.2408 | 1.74968 | 0.02605 | 0 | -0.1862 | -1.1377 | 1.5792 | 0.10826 | 0 | 0 |
| 1393932_at | Samd8 | sterile alpha motif domain containing 8 | uniqAntib_1098 | 0.16 | 0.12 | 0.21 | 0.41 | 0.23 | 0 | 0.09184 | 1.06573 | 1.42837 | 0.18018 | 1 | 0.29148 | 1.22389 | 1.20271 | 0.02699 | 0 | 0.1083 | 1.07796 | 1.34416 | 0.20319 | 0 | 0 |
| 1393971_at | --- | --- | uniqAntib_1098 | 0.11 | 0.07 | 0.17 | -0.21 | 0.08 | 0 | 0.10133 | 1.07276 | 1.51824 | 0.15305 | 1 | -0.2833 | -1.217 | 1.50384 | 0.04096 | 0 | 0.01133 | 1.00788 | 1.03234 | 0.39267 | 0 | 0 |
| 1394009_at | --- | --- | uniqAntib_1098 | -0.19 | 0.07 | 0.09 | 0.36 | 0.27 | 0 | 0.01521 | 1.0106 | 0.90673 | 0.5856 | 1 | 0.28622 | 1.21944 | 1.26478 | 0.03965 | 0 | 0.19158 | 1.14202 | 2.06764 | 0.05784 | 0 | 0 |
| 1394029_at | LOC10091247 | vacuolar ATPase assembly integral membrane prote | uniqAntib_1098 | -0.12 | 0.00 | 0.08 | 0.33 | -0.03 | 0 | 0.08686 | 1.06205 | 1.30405 | 0.22924 | 1 | 0.33691 | 1.26305 | 1.091 | 0.01958 | 0 | -0.0254 | -1.0177 | 1.06771 | 0.34436 | 0 | 0 |
| 1394037_at | LOC10091221 | uncharacterized LOC100912219 | uniqAntib_1098 | -0.07 | 0.05 | -0.02 | -0.23 | -0.13 | 0 | -0.0682 | -1.0484 | 1.1167 | 0.32852 | 1 | -0.2853 | -1.2186 | 1.64506 | 0.03137 | 0 | -0.1806 | -1.1334 | 1.51227 | 0.12235 | 0 | 0 |
| 1394042_at | LOC681371 | hypothetical protein LOC681371 | uniqAntib_1098 | -0.15 | 0.01 | -0.01 | 0.37 | 0.06 | 0 | -0.0192 | -1.0134 | 0.94134 | 0.48612 | 1 | 0.35538 | 1.27933 | 0.99424 | 0.01426 | 0 | 0.05267 | 1.03719 | 1.13699 | 0.30802 | 0 | 0 |
| 1394068_x_at | Klf2 | Kruppel-like factor 2 (lung) | uniqAntib_1098 | 0.16 | 0.43 | 0.35 | 0.10 | 0.31 | 0 | -0.0781 | -1.0556 | 1.79334 | 0.12398 | 1 | -0.3302 | -1.2572 | 1.82652 | 0.02073 | 0 | -0.1197 | -1.0865 | 1.38871 | 0.16111 | 0 | 0 |
| 1394105_at | Samd4b | sterile alpha motif domain containing 4B | uniqAntib_1098 | 0.03 | -0.11 | -0.10 | -0.38 | -0.17 | 0 | 0.01478 | 1.0103 | 1.10234 | 0.33784 | 1 | -0.2682 | -1.2043 | 1.6113 | 0.03306 | 0 | -0.0586 | -1.0415 | 1.37222 | 0.16617 | 0 | 0 |
| 1394156_at | Igsf3 | immunoglobulin superfamily, member 3 | uniqAntib_1098 | 0.01 | 0.02 | 0.22 | -0.27 | -0.11 | 0 | 0.20112 | 1.14959 | 1.95995 | 0.04865 | 1 | -0.2893 | -1.222 | 1.7915 | 0.0227 | 0 | -0.1286 | -1.0933 | 1.67779 | 0.09571 | 0 | 0 |
| 1394184_at | LOC10091011 | uncharacterized LOC100910119 | uniqAntib_1098 | -0.19 | 0.52 | 0.45 | 0.17 | 0.37 | 0 | -0.062 | -1.0439 | 1.20205 | 0.28089 | 1 | -0.351 | -1.2754 | 2.54113 | 0.00793 | 0 | -0.1467 | -1.107 | 1.65069 | 0.0974 | 0 | 0 |
| 1394196_at | --- | --- | uniqAntib_1098 | 0.00 | -0.13 | 0.05 | 0.25 | -0.13 | 0 | 0.1798 | 1.13273 | 1.4988 | 0.15668 | 1 | 0.37193 | 1.29408 | 0.9676 | 0.01207 | 0 | 0.00069 | 1.00048 | 0.93923 | 0.53091 | 0 | 0 |
| 1394216_at | L3hypdh | L-3-hydroxyproline dehydratase (trans-) | uniqAntib_1098 | 0.05 | -0.01 | 0.00 | -0.31 | -0.04 | 0 | 0.00759 | 1.00528 | 0.91461 | 0.53385 | 1 | -0.3021 | -1.2329 | 1.57142 | 0.03421 | 0 | -0.0312 | -1.0218 | 0.98955 | 0.44961 | 0 | 0 |
| 1394347_at | Golga4 | golgin A4 | uniqAntib_1098 | -0.10 | 0.12 | 0.28 | 0.40 | -0.02 | 0 | 0.16323 | 1.11979 | 2.63657 | 0.00297 | 1 | 0.28379 | 1.21739 | 0.56428 | 0.00314 | 0 | -0.1419 | -1.1034 | 2.91775 | 0.01295 | 0 | 0 |
| 1394367_at | Dll4 | delta-like 4 (Drosophila) | uniqAntib_1098 | -0.02 | 0.12 | -0.09 | -0.19 | 0.04 | 0 | -0.203 | -1.1511 | 3.17326 | 0.02255 | 1 | -0.3045 | -1.235 | 2.4948 | 0.00802 | 0 | -0.08 | -1.057 | 1.57395 | 0.10937 | 0 | 0 |
| 1394411_at | RGD1560608 | similar to novel protein | uniqAntib_1098 | 0.10 | 0.03 | -0.02 | -0.25 | 0.03 | 0 | -0.051 | -1.036 | 1.14277 | 0.31297 | 1 | -0.279 | -1.2133 | 1.44024 | 0.04636 | 0 | 0.00232 | 1.00161 | 0.92354 | 0.54951 | 0 | 0 |
| 1394419_at | Arhgap11a | Rho GTPase activating protein 11A | uniqAntib_1098 | -0.05 | 0.00 | 0.07 | 0.30 | 0.07 | 0 | 0.06949 | 1.04934 | 1.11007 | 0.34367 | 1 | 0.29969 | 1.23088 | 1.19238 | 0.02653 | 0 | 0.07244 | 1.05149 | 1.05826 | 0.372 | 0 | 0 |
| 1394428_at | --- | --- | uniqAntib_1098 | -0.18 | -0.07 | -0.23 | -0.33 | -0.23 | 0 | -0.1661 | -1.122 | 1.55539 | 0.16355 | 1 | -0.265 | -1.2016 | 1.44436 | 0.04603 | 0 | -0.1666 | -1.1224 | 1.95881 | 0.05524 | 0 | 0 |
| 1394436_at | Spag9 | sperm associated antigen 9 | uniqAntib_1098 | -0.10 | -0.07 | 0.11 | 0.24 | 0.04 | 0 | 0.1879 | 1.1391 | 1.9374 | 0.05326 | 1 | 0.31694 | 1.24569 | 1.07445 | 0.01848 | 0 | 0.11449 | 1.08259 | 1.46942 | 0.16008 | 0 | 0 |

| Affymetrix Probeset ID | Gene Symbol | Gene Title | Figure 6 B Venn n =2021 | Mean BN log2 intensity autologous n=8 | Mean log2 intensity day 30 DR +/+ ND n=4 | Mean log2 intensity day 30 DR+/+ HCD n=4 | Mean log2 intensity day 30 DR+/+ B/S n=4 | Mean log2 intensity day 30 F+/+ ND n=4 | DR+/+ HCD vs DR+/+ ND n=380 1=yes 0=no | Log2 ratio DR+/+ HCD vs DR+/+ ND | Fold-Change DR+/+ HCD vs DR+/+ ND | RP-FDR 4DR30 HCD vs 4DR30 Reg | p value DR+/+ HCD vs DR+/+ ND | DR+/+ B/S vs DR+/+ ND n=1,330 1=yes 0=no | Log2 ratio DR+/+ B/S vs DR+/+ ND | Fold-Change DR+/+ B/S vs DR+/+ ND | RP-FDR 4DR30 Antib vs 4DR30 Reg | p value DR+/+ B/S vs DR+/+ ND | DR+/+ ND vs F+/+ ND n=398 1=yes 0=no | Log2 ratio DR+/+ ND vs F+/+ ND | Fold-Change DR+/+ ND vs F+/+ ND | RP-FDR 4RT1uu 30Reg vs 4DR30 Reg | p value DR+/+ ND vs F+/+ ND | DR+/+ Longitudinal n=228 1=yes 0=no | Figure 6F |
|---|---|---|---|---|---|---|---|---|---|---|---|---|---|---|---|---|---|---|---|---|---|---|---|---|---|
| 1394439_at | Mau2 | Mau2 chromatid cohesion factor homolog (C. elegа | uniqAntib_1098 | 0.08 | 0.10 | 0.18 | -0.19 | 0.01 | 0 | 0.08264 | 1.05896 | 1.07034 | 0.3757 | 1 | -0.2931 | -1.2253 | 1.58336 | 0.03365 | 0 | -0.0864 | -1.0617 | 1.05112 | 0.36196 | 0 | 0 |
| 1394547_at | --- | --- | uniqAntib_1098 | -0.01 | -0.13 | 0.07 | 0.17 | -0.01 | 0 | 0.19884 | 1.14778 | 1.61326 | 0.12958 | 1 | 0.29881 | 1.23013 | 1.28083 | 0.04263 | 0 | 0.12152 | 1.08788 | 1.37585 | 0.1905 | 0 | 0 |
| 1394550_at | LOC691221 | similar to CG1998-PA | uniqAntib_1098 | 0.02 | 0.08 | -0.07 | -0.22 | 0.00 | 0 | -0.1425 | -1.1038 | 1.4182 | 0.19573 | 1 | -0.2991 | -1.2304 | 1.64156 | 0.03141 | 0 | -0.0734 | -1.0522 | 1.1149 | 0.30526 | 0 | 0 |
| 1394564_at | --- | --- | uniqAntib_1098 | -0.07 | -0.14 | -0.36 | -0.44 | -0.13 | 0 | -0.2165 | -1.1619 | 2.65931 | 0.0419 | 1 | -0.2981 | -1.2295 | 1.93065 | 0.01813 | 0 | 0.00739 | 1.00513 | 1.13169 | 0.31172 | 0 | 0 |
| 1394608_at | --- | --- | uniqAntib_1098 | 0.05 | 0.08 | -0.06 | -0.21 | -0.09 | 0 | -0.1419 | -1.1034 | 1.93499 | 0.1054 | 1 | -0.2991 | -1.2304 | 1.65299 | 0.03024 | 0 | -0.1772 | -1.1306 | 1.40283 | 0.15599 | 0 | 0 |
| 1394616_at | Mesdc2 | mesoderm development candidate 2 | uniqAntib_1098 | 0.01 | -0.07 | -0.02 | 0.21 | -0.01 | 0 | 0.05152 | 1.03635 | 1.25353 | 0.25374 | 1 | 0.28494 | 1.21836 | 1.20334 | 0.02871 | 0 | 0.0652 | 1.04623 | 1.14269 | 0.3027 | 0 | 0 |
| 1394669_at | --- | --- | uniqAntib_1098 | 0.00 | 0.04 | -0.05 | -0.29 | -0.03 | 0 | -0.0821 | -1.0586 | 1.79339 | 0.12347 | 1 | -0.3292 | -1.2563 | 2.08822 | 0.01397 | 0 | -0.0693 | -1.0492 | 1.74323 | 0.08582 | 0 | 0 |
| 1394692_at | Mbd4 | methyl-CpG binding domain protein 4 | uniqAntib_1098 | -0.09 | -0.24 | -0.01 | -0.51 | -0.27 | 0 | 0.23181 | 1.17431 | 2.0918 | 0.02993 | 1 | -0.2774 | -1.212 | 1.7814 | 0.02412 | 0 | -0.0368 | -1.0259 | 1.43377 | 0.14707 | 0 | 0 |
| 1394709_at | --- | --- | uniqAntib_1098 | -0.12 | -0.25 | -0.05 | 0.07 | 0.01 | 0 | 0.19743 | 1.14666 | 1.75962 | 0.08448 | 1 | 0.32702 | 1.25442 | 1.15617 | 0.02309 | 0 | 0.25753 | 1.19543 | 2.30211 | 0.03472 | 0 | 0 |
| 1394740_at | --- | --- | uniqAntib_1098 | -0.05 | -0.02 | -0.10 | -0.33 | -0.25 | 0 | -0.0763 | -1.0543 | 1.32236 | 0.23106 | 1 | -0.3063 | -1.2365 | 1.52944 | 0.03875 | 0 | -0.2327 | -1.1751 | 1.97947 | 0.05207 | 0 | 0 |
| 1394764_at | Lmtk2 | lemur tyrosine kinase 2 | uniqAntib_1098 | -0.13 | -0.09 | -0.20 | -0.36 | -0.20 | 0 | -0.1139 | -1.0821 | 1.26066 | 0.25319 | 1 | -0.2696 | -1.2055 | 1.5082 | 0.04035 | 0 | -0.1058 | -1.0761 | 1.281 | 0.20307 | 0 | 0 |
| 1394766_at | --- | --- | uniqAntib_1098 | -0.01 | -0.34 | -0.24 | -0.02 | -0.14 | 0 | 0.09696 | 1.06951 | 1.41124 | 0.18869 | 1 | 0.3203 | 1.24859 | 0.96792 | 0.0127 | 0 | 0.1916 | 1.14203 | 1.96243 | 0.06626 | 0 | 0 |
| 1394788_at | --- | --- | uniqAntib_1098 | -0.05 | -0.19 | -0.21 | -0.46 | -0.33 | 0 | -0.0242 | -1.0169 | 1.63432 | 0.14883 | 1 | -0.2684 | -1.2045 | 1.74715 | 0.0264 | 0 | -0.1368 | -1.0995 | 1.95094 | 0.05596 | 0 | 0 |
| 1394836_at | --- | --- | uniqAntib_1098 | 0.23 | 0.16 | 0.13 | -0.13 | 0.16 | 0 | -0.0251 | -1.0175 | 1.36357 | 0.21612 | 1 | -0.2886 | -1.2215 | 2.01037 | 0.01752 | 0 | 0.00018 | 1.00012 | 1.22981 | 0.25649 | 0 | 0 |
| 1394855_at | --- | --- | uniqAntib_1098 | -0.02 | 0.25 | 0.28 | 0.64 | 0.20 | 0 | 0.03148 | 1.02206 | 1.11389 | 0.34037 | 1 | 0.39474 | 1.3147 | 0.76818 | 0.00664 | 0 | -0.0541 | -1.0382 | 1.25071 | 0.22051 | 0 | 0 |
| 1394905_at | Kmt2d /// Ml | lysine (K)-specific methyltransferase 2D /// myeloid | uniqAntib_1098 | 0.10 | 0.03 | -0.02 | -0.28 | -0.01 | 0 | -0.0427 | -1.03 | 1.02605 | 0.40021 | 1 | -0.306 | -1.2362 | 1.6459 | 0.03101 | 0 | -0.0335 | -1.0235 | 0.98182 | 0.46175 | 0 | 0 |
| 1394914_at | Pdlim5 | PDZ and LIM domain 5 | uniqAntib_1098 | 0.03 | 0.02 | -0.06 | -0.26 | -0.03 | 0 | -0.0809 | -1.0577 | 1.3899 | 0.20702 | 1 | -0.2739 | -1.2091 | 1.56284 | 0.03598 | 0 | -0.0456 | -1.0321 | 1.00671 | 0.41988 | 0 | 0 |
| 1394925_at | RGD1307235 | similar to RIKEN cDNA 2310035C23 | uniqAntib_1098 | -0.12 | 0.11 | 0.30 | 0.47 | 0.12 | 0 | 0.18966 | 1.1405 | 1.89313 | 0.06057 | 1 | 0.35106 | 1.2755 | 0.72984 | 0.00601 | 0 | 0.00991 | 1.00689 | 1.07588 | 0.35498 | 0 | 0 |
| 1394934_at | Zfp870 | zinc finger protein 870 | uniqAntib_1098 | -0.12 | 0.07 | -0.07 | -0.23 | -0.16 | 0 | -0.14 | -1.1019 | 1.69349 | 0.13793 | 1 | -0.2939 | -1.226 | 1.76189 | 0.02425 | 0 | -0.2282 | -1.1714 | 1.95621 | 0.05473 | 0 | 0 |
| 1394954_at | Slc31a2 | solute carrier family 31 (copper transporters), mem | uniqAntib_1098 | 0.04 | -0.22 | -0.18 | 0.12 | -0.05 | 0 | 0.03679 | 1.02583 | 0.99995 | 0.44604 | 1 | 0.33747 | 1.26354 | 0.941 | 0.01056 | 0 | 0.16129 | 1.11828 | 1.61739 | 0.12248 | 0 | 0 |
| 1394964_at | LOC1009104 | protein PRRC2C-like | uniqAntib_1098 | 0.03 | 0.05 | 0.27 | 0.43 | 0.15 | 0 | 0.21706 | 1.16236 | 2.91368 | 0.00178 | 1 | 0.37594 | 1.29769 | 0.2367 | 0.00078 | 0 | 0.10022 | 1.07194 | 1.73333 | 0.00485 | 0 | 0 |
| 1395040_at | RGD1561777 | similar to Na+ dependent glucose transporter 1 | uniqAntib_1098 | -0.02 | 0.15 | -0.06 | -0.17 | -0.09 | 0 | -0.2116 | -1.1579 | 2.35823 | 0.06438 | 1 | -0.3169 | -1.2456 | 1.98852 | 0.01599 | 0 | -0.2407 | -1.1816 | 2.23224 | 0.03553 | 0 | 0 |
| 1395054_at | Usp29 | ubiquitin specific peptidase 29 | uniqAntib_1098 | 0.03 | -0.03 | -0.14 | -0.39 | -0.26 | 0 | -0.1024 | -1.0736 | 1.34776 | 0.22224 | 1 | -0.3577 | -1.2814 | 2.22044 | 0.01481 | 0 | -0.2232 | -1.1673 | 1.84403 | 0.06843 | 0 | 0 |
| 1395057_at | --- | --- | uniqAntib_1098 | 0.09 | 0.07 | -0.04 | -0.30 | -0.14 | 0 | -0.1088 | -1.0783 | 1.29683 | 0.24053 | 1 | -0.3698 | -1.2922 | 2.17777 | 0.01225 | 0 | -0.2032 | -1.1512 | 1.68429 | 0.09418 | 0 | 0 |
| 1395060_at | --- | --- | uniqAntib_1098 | -0.22 | -0.05 | 0.12 | 0.23 | 0.13 | 0 | 0.17173 | 1.12641 | 2.08766 | 0.03162 | 1 | 0.28625 | 1.21946 | 1.18383 | 0.02627 | 0 | 0.1835 | 1.13564 | 1.71728 | 0.09951 | 0 | 0 |
| 1395068_at | --- | --- | uniqAntib_1098 | 0.08 | 0.07 | -0.06 | -0.29 | 0.03 | 0 | -0.1265 | -1.0917 | 1.62527 | 0.15041 | 1 | -0.3563 | -1.2802 | 2.09619 | 0.01362 | 0 | -0.0399 | -1.028 | 1.00872 | 0.41661 | 0 | 0 |
| 1395069_at | --- | --- | uniqAntib_1098 | 0.10 | -0.02 | 0.13 | 0.25 | 0.15 | 0 | 0.14439 | 1.10526 | 1.47448 | 0.16599 | 1 | 0.26965 | 1.20551 | 1.22013 | 0.03217 | 0 | 0.16351 | 1.12001 | 1.89507 | 0.07325 | 0 | 0 |
| 1395071_at | Zbtb6 | zinc finger and BTB domain containing 6 | uniqAntib_1098 | -0.08 | -0.31 | -0.31 | 0.02 | -0.33 | 0 | 0.00675 | 1.00469 | 1.67853 | 0.11156 | 1 | 0.33689 | 1.26303 | 1.05457 | 0.01723 | 0 | -0.0157 | -1.011 | 1.25464 | 0.21753 | 0 | 0 |
| 1395083_at | --- | --- | uniqAntib_1098 | -0.33 | -0.10 | -0.16 | 0.19 | -0.16 | 0 | -0.0609 | -1.0431 | 1.16865 | 0.2995 | 1 | 0.29203 | 1.22436 | 1.20007 | 0.0279 | 0 | -0.0602 | -1.0426 | 1.06339 | 0.34836 | 0 | 0 |
| 1395098_at | Blzf1 | basic leucine zipper nuclear factor 1 | uniqAntib_1098 | -0.22 | -0.32 | -0.24 | -0.03 | -0.27 | 0 | 0.07817 | 1.05568 | 1.3533 | 0.20988 | 1 | 0.28527 | 1.21864 | 1.27705 | 0.04119 | 0 | 0.04624 | 1.03257 | 1.11426 | 0.323 | 0 | 0 |
| 1395146_at | Adipor2 | adiponectin receptor 2 | uniqAntib_1098 | 0.00 | 0.16 | -0.02 | 0.42 | 0.06 | 0 | -0.1833 | -1.1355 | 2.55946 | 0.04839 | 1 | 0.26469 | 1.20137 | 1.00978 | 0.0149 | 0 | -0.0961 | -1.0689 | 1.83441 | 0.0745 | 0 | 0 |
| 1395162_at | --- | --- | uniqAntib_1098 | -0.02 | 0.00 | -0.12 | -0.27 | -0.24 | 0 | -0.1214 | -1.0878 | 1.27789 | 0.24659 | 1 | -0.2652 | -1.2018 | 1.45795 | 0.04519 | 0 | -0.2349 | -1.1768 | 1.84497 | 0.06811 | 0 | 0 |
| 1395166_at | Ehd2 | EH-domain containing 2 | uniqAntib_1098 | 0.04 | 0.05 | 0.00 | -0.24 | 0.03 | 0 | -0.0514 | -1.0363 | 0.94079 | 0.48656 | 1 | -0.2886 | -1.2214 | 1.47421 | 0.04385 | 0 | -0.0181 | -1.0126 | 0.91609 | 0.5928 | 0 | 0 |
| 1395183_at | --- | --- | uniqAntib_1098 | -0.02 | -0.34 | -0.32 | -0.05 | -0.38 | 0 | 0.0187 | 1.01305 | 0.94919 | 0.51069 | 1 | 0.2919 | 1.22425 | 1.27929 | 0.04192 | 0 | -0.0431 | -1.0303 | 0.96897 | 0.48064 | 0 | 0 |
| 1395184_at | Clec12a | C-type lectin domain family 12, member A | uniqAntib_1098 | -0.28 | -0.49 | -0.32 | -0.14 | -0.36 | 0 | 0.16653 | 1.12236 | 1.76181 | 0.08577 | 1 | 0.35039 | 1.27491 | 0.95584 | 0.01122 | 0 | 0.13017 | 1.09442 | 1.81207 | 0.08653 | 0 | 0 |
| 1395209_at | --- | --- | uniqAntib_1098 | 0.10 | 0.13 | 0.36 | 0.39 | 0.22 | 0 | 0.23414 | 1.1762 | 2.09302 | 0.03298 | 1 | 0.26703 | 1.20333 | 1.26548 | 0.03963 | 0 | 0.08986 | 1.06427 | 1.43834 | 0.17127 | 0 | 0 |
| 1395342_at | Ckap4 | cytoskeleton-associated protein 4 | uniqAntib_1098 | 0.08 | 0.13 | 0.03 | -0.17 | 0.05 | 0 | -0.0966 | -1.0692 | 1.67167 | 0.14008 | 1 | -0.3005 | -1.2315 | 2.57565 | 0.00894 | 0 | -0.0837 | -1.0597 | 1.41718 | 0.15252 | 0 | 0 |
| 1395343_at | Tm9sf3 | transmembrane 9 superfamily member 3 | uniqAntib_1098 | -0.18 | -0.13 | -0.22 | 0.15 | -0.19 | 0 | -0.0869 | -1.0621 | 1.55837 | 0.16316 | 1 | 0.28009 | 1.21427 | 1.05006 | 0.01709 | 0 | -0.0628 | -1.0445 | 1.3108 | 0.18858 | 0 | 0 |
| 1395360_at | Usp30 | ubiquitin specific peptidase 30 | uniqAntib_1098 | 0.02 | 0.06 | -0.02 | -0.23 | 0.10 | 0 | -0.0821 | -1.0586 | 1.53885 | 0.1677 | 1 | -0.2912 | -1.2237 | 1.51703 | 0.0401 | 0 | 0.04212 | 1.02963 | 1.02406 | 0.40371 | 0 | 0 |
| 1395418_at | Zfp131 | zinc finger protein 131 | uniqAntib_1098 | -0.17 | -0.24 | -0.27 | 0.09 | -0.30 | 0 | -0.0297 | -1.0208 | 1.49212 | 0.17738 | 1 | 0.3361 | 1.26234 | 0.96776 | 0.01211 | 0 | -0.0582 | -1.0412 | 1.36443 | 0.1697 | 0 | 0 |
| 1395419_at | Kmt2a | lysine (K)-specific methyltransferase 2A | uniqAntib_1098 | 0.06 | -0.15 | 0.09 | 0.30 | 0.10 | 0 | 0.24165 | 1.18234 | 1.93714 | 0.05394 | 1 | 0.44949 | 1.36555 | 0.60854 | 0.00389 | 0 | 0.25678 | 1.19481 | 2.33825 | 0.03436 | 0 | 0 |
| 1395488_at | Msh3 | mutS homolog 3 (E. coli) | uniqAntib_1098 | -0.11 | -0.17 | -0.11 | 0.12 | -0.12 | 0 | 0.06224 | 1.04409 | 1.017 | 0.42777 | 1 | 0.29047 | 1.22304 | 1.20325 | 0.02929 | 0 | 0.0518 | 1.03656 | 1.1007 | 0.33436 | 0 | 0 |
| 1395490_at | --- | --- | uniqAntib_1098 | -0.09 | -0.20 | -0.27 | 0.12 | -0.24 | 0 | -0.0751 | -1.0534 | 1.5463 | 0.16567 | 1 | 0.31947 | 1.24787 | 0.79071 | 0.00717 | 0 | -0.0447 | -1.0315 | 1.03741 | 0.37575 | 0 | 0 |
| 1395585_at | --- | --- | uniqAntib_1098 | -0.26 | -0.20 | -0.23 | 0.07 | -0.13 | 0 | -0.023 | -1.0161 | 1.19656 | 0.28384 | 1 | 0.2723 | 1.20773 | 1.27045 | 0.04641 | 0 | 0.07754 | 1.05522 | 1.30778 | 0.21842 | 0 | 0 |
| 1395690_at | --- | --- | uniqAntib_1098 | 0.16 | 0.18 | 0.10 | -0.13 | -0.09 | 0 | -0.0777 | -1.0554 | 1.31678 | 0.23258 | 1 | -0.3095 | -1.2393 | 2.0078 | 0.01556 | 0 | -0.262 | -1.1991 | 2.21973 | 0.03662 | 0 | 0 |
| 1395706_at | Lingo1 | leucine rich repeat and Ig domain containing 1 | uniqAntib_1098 | -0.13 | -0.04 | -0.10 | -0.33 | -0.22 | 0 | -0.0627 | -1.0444 | 1.01168 | 0.41357 | 1 | -0.2865 | -1.2197 | 1.44979 | 0.04545 | 0 | -0.1849 | -1.1367 | 1.48815 | 0.12949 | 0 | 0 |
| 1395747_at | --- | --- | uniqAntib_1098 | 0.00 | -0.11 | 0.00 | 0.17 | -0.12 | 0 | 0.11728 | 1.08469 | 1.87143 | 0.06252 | 1 | 0.28124 | 1.21524 | 0.9516 | 0.01092 | 0 | -0.0075 | -1.0052 | 1.17982 | 0.26063 | 0 | 0 |
| 1395776_at | --- | --- | uniqAntib_1098 | -0.25 | -0.23 | -0.30 | 0.05 | -0.20 | 0 | -0.073 | -1.0519 | 1.17655 | 0.29551 | 1 | 0.28144 | 1.21541 | 1.27851 | 0.04218 | 0 | 0.03495 | 1.02452 | 1.23725 | 0.25295 | 0 | 0 |
| 1395819_at | --- | --- | uniqAntib_1098 | 0.01 | -0.29 | -0.17 | 0.05 | -0.28 | 0 | 0.12282 | 1.08886 | 1.63482 | 0.12064 | 1 | 0.34475 | 1.26993 | 0.96963 | 0.01344 | 0 | 0.01127 | 1.00785 | 0.93286 | 0.52392 | 0 | 0 |
| 1395850_at | Pus3 | pseudouridylate synthase 3 | uniqAntib_1098 | -0.04 | -0.24 | -0.30 | 0.08 | -0.11 | 0 | -0.0671 | -1.0476 | 1.32942 | 0.2278 | 1 | 0.31502 | 1.24403 | 1.1731 | 0.02531 | 0 | 0.12671 | 1.0918 | 1.85104 | 0.08047 | 0 | 0 |
| 1395864_at | --- | --- | uniqAntib_1098 | -0.03 | -0.29 | -0.04 | 0.02 | -0.05 | 0 | 0.24518 | 1.18524 | 1.93996 | 0.0551 | 1 | 0.30439 | 1.2349 | 1.04606 | 0.01699 | 0 | 0.23471 | 1.17667 | 2.26978 | 0.04313 | 0 | 0 |
| 1395870_at | Trmt2a | tRNA methyltransferase 2 homolog A (S. cerevisiae) | uniqAntib_1098 | -0.13 | -0.15 | -0.04 | 0.19 | 0.10 | 0 | 0.10986 | 1.07912 | 1.70032 | 0.10273 | 1 | 0.3331 | 1.25971 | 1.1233 | 0.02048 | 0 | 0.24652 | 1.18634 | 2.29234 | 0.04061 | 0 | 0 |
| 1395911_at | --- | --- | uniqAntib_1098 | -0.14 | -0.09 | -0.15 | -0.39 | -0.20 | 0 | -0.062 | -1.0439 | 0.99547 | 0.4289 | 1 | -0.3028 | -1.2335 | 1.56349 | 0.037 | 0 | -0.11 | -1.0792 | 1.13706 | 0.28559 | 0 | 0 |
| 1395934_at | LOC684934 | similar to coiled-coil domain containing 9 | uniqAntib_1098 | 0.07 | 0.04 | -0.11 | -0.34 | -0.18 | 0 | -0.147 | -1.1073 | 2.50364 | 0.05257 | 1 | -0.3738 | -1.2958 | 3.71036 | 0.00334 | 0 | -0.2211 | -1.1656 | 2.86858 | 0.02075 | 0 | 0 |
| 1395944_at | Mis18a | MIS18 kinetochore protein homolog A (S. pombe) | uniqAntib_1098 | -0.10 | -0.17 | -0.02 | 0.25 | -0.12 | 0 | 0.15274 | 1.11168 | 1.54502 | 0.14412 | 1 | 0.42064 | 1.33852 | 0.64217 | 0.00438 | 0 | 0.0496 | 1.03498 | 1.01772 | 0.41237 | 0 | 0 |
| 1395963_at | --- | --- | uniqAntib_1098 | 0.01 | 0.26 | 0.31 | 0.76 | 0.44 | 0 | 0.05568 | 1.03935 | 1.02008 | 0.42435 | 1 | 0.50117 | 1.41536 | 0.37242 | 0.00144 | 0 | 0.18587 | 1.1375 | 1.56503 | 0.13482 | 0 | 0 |
| 1395989_at | Gfm2 | G elongation factor, mitochondrial 2 | uniqAntib_1098 | 0.10 | 0.08 | -0.03 | -0.19 | 0.05 | 0 | -0.1061 | -1.0763 | 1.55903 | 0.16293 | 1 | -0.2681 | -1.2042 | 1.60585 | 0.03248 | 0 | -0.0309 | -1.0217 | 1.0549 | 0.35746 | 0 | 0 |
| 1396068_at | --- | --- | uniqAntib_1098 | 0.08 | -0.08 | -0.10 | 0.30 | -0.04 | 0 | -0.0182 | -1.0127 | 0.96592 | 0.45667 | 1 | 0.38752 | 1.30814 | 0.86094 | 0.00855 | 0 | 0.03828 | 1.02689 | 1.02271 | 0.40716 | 0 | 0 |
| 1396088_at | --- | --- | uniqAntib_1098 | -0.02 | -0.07 | -0.17 | -0.37 | -0.15 | 0 | -0.1037 | -1.0745 | 1.3998 | 0.20349 | 1 | -0.304 | -1.2345 | 1.60183 | 0.03209 | 0 | -0.0842 | -1.0601 | 1.1632 | 0.27009 | 0 | 0 |
| 1396202_at | --- | --- | uniqAntib_1098 | 0.15 | -0.08 | 0.14 | 0.23 | 0.08 | 0 | 0.21837 | 1.16342 | 2.26028 | 0.01548 | 1 | 0.30949 | 1.23927 | 0.93596 | 0.01005 | 0 | 0.15393 | 1.11259 | 1.80106 | 0.08774 | 0 | 0 |
| 1396210_at | --- | --- | uniqAntib_1098 | -0.02 | 0.13 | -0.13 | -0.15 | -0.10 | 0 | -0.2593 | -1.1969 | 2.63 | 0.04228 | 1 | -0.2781 | -1.2126 | 1.51643 | 0.03969 | 0 | -0.2307 | -1.1734 | 2.37022 | 0.03178 | 0 | 0 |
| 1396236_at | --- | --- | uniqAntib_1098 | -0.01 | 0.08 | 0.18 | 0.43 | 0.26 | 0 | 0.09909 | 1.0711 | 1.67801 | 0.10937 | 1 | 0.34496 | 1.27011 | 0.95955 | 0.01169 | 0 | 0.1753 | 1.1292 | 2.38487 | 0.02316 | 0 | 0 |
| 1396246_at | --- | --- | uniqAntib_1098 | 0.10 | 0.08 | 0.28 | -0.20 | 0.03 | 0 | 0.20204 | 1.15033 | 1.77863 | 0.07629 | 1 | -0.2789 | -1.2132 | 1.6156 | 0.03304 | 0 | -0.0457 | -1.0322 | 1.08949 | 0.32346 | 0 | 0 |
| 1396363_at | --- | --- | uniqAntib_1098 | -0.13 | -0.23 | -0.40 | 0.06 | -0.24 | 0 | -0.1763 | -1.13 | 2.37868 | 0.06364 | 1 | 0.2844 | 1.2179 | 1.27952 | 0.04365 | 0 | -0.0143 | -1.01 | 0.93196 | 0.54697 | 0 | 0 |
| 1396367_at | --- | --- | uniqAntib_1098 | 0.13 | 0.21 | 0.15 | -0.12 | 0.08 | 0 | -0.0617 | -1.0437 | 1.0479 | 0.3787 | 1 | -0.336 | -1.2622 | 1.98948 | 0.01587 | 0 | -0.1361 | -1.0989 | 1.46597 | 0.13972 | 0 | 0 |
| 1396375_at | --- | --- | uniqAntib_1098 | 0.15 | 0.06 | 0.02 | -0.22 | -0.06 | 0 | -0.0422 | -1.0297 | 1.20386 | 0.27965 | 1 | -0.2842 | -1.2178 | 1.65771 | 0.02953 | 0 | -0.1239 | -1.0897 | 1.43594 | 0.14665 | 0 | 0 |
| 1396435_at | --- | --- | uniqAntib_1098 | -0.02 | -0.01 | -0.19 | -0.36 | -0.20 | 0 | -0.1869 | -1.1383 | 2.22342 | 0.07307 | 1 | -0.351 | -1.2755 | 2.18197 | 0.01284 | 0 | -0.1957 | -1.1453 | 1.8311 | 0.07472 | 0 | 0 |
| 1396441_at | --- | --- | uniqAntib_1098 | -0.21 | 0.30 | 0.14 | 0.00 | 0.28 | 0 | -0.1517 | -1.1109 | 2.13019 | 0.07939 | 1 | -0.2974 | -1.2289 | 2.0128 | 0.01527 | 0 | -0.0121 | -1.0084 | 1.15038 | 0.27762 | 0 | 0 |
| 1396466_at | --- | --- | uniqAntib_1098 | 0.04 | -0.04 | 0.04 | -0.32 | -0.05 | 0 | 0.08365 | 1.0597 | 1.0348 | 0.41393 | 1 | -0.28 | -1.2142 | 1.43746 | 0.04844 | 0 | -0.0025 | -1.0017 | 1.01286 | 0.41773 | 0 | 0 |
| 1396470_at | Lmo4 | LIM domain only 4 | uniqAntib_1098 | -0.15 | 0.03 | 0.11 | 0.31 | 0.01 | 0 | 0.08198 | 1.05847 | 1.16528 | 0.30418 | 1 | 0.28679 | 1.21992 | 1.27961 | 0.04382 | 0 | -0.0173 | -1.012 | 0.92962 | 0.55336 | 0 | 0 |
| 1396498_at | --- | --- | uniqAntib_1098 | -0.02 | -0.06 | -0.23 | -0.34 | -0.20 | 0 | -0.175 | -1.1289 | 1.65475 | 0.14335 | 1 | -0.2772 | -1.2118 | 1.44167 | 0.04636 | 0 | -0.1372 | -1.0998 | 1.23261 | 0.22731 | 0 | 0 |

| Affymetrix Probeset ID | Gene Symbol | Gene Title | Figure 6 B Venn n =2021 | Mean BN log2 intensity autologous n=8 | Mean log2 intensity day 30 DR +/+ ND n=4 | Mean log2 intensity day 30 DR+/+ HCD n=4 | Mean log2 intensity day 30 B/S n=4 | Mean log2 intensity day 30 F+/+ ND n=4 | DR+/+ HCD vs DR+/+ ND n=380 1=yes 0=no | Log2 ratio DR+/+ HCD vs DR+/+ ND | Fold-Change DR+/+ HCD vs DR+/+ ND | RP-FDR 4DR30 HCD vs 4DR30 Reg | p value DR+/+ HCD vs DR+/+ ND | DR+/+ B/S vs DR+/+ ND n=1,330 1=yes 0=no | Log2 ratio DR+/+ B/S vs DR+/+ ND | Fold-Change DR+/+ B/S vs DR+/+ ND | RP-FDR 4DR30 Antib vs 4DR30 Reg | p value DR+/+ B/S vs DR+/+ ND | DR+/+ ND vs F+/+ ND n=398 1=yes 0=no | Log2 ratio DR+/+ ND vs F+/+ ND | Fold-Change DR+/+ ND vs F+/+ ND | RP-FDR 4RT1uu 30Reg vs 4DR30 Reg | p value DR+/+ ND vs F+/+ ND | DR+/+ Longitudinal n=228 1=yes 0=no | Figure 6F |
|---|---|---|---|---|---|---|---|---|---|---|---|---|---|---|---|---|---|---|---|---|---|---|---|---|---|
| 1396516_at | --- | --- | uniqAntib_1098 | -0.08 | -0.07 | 0.13 | 0.24 | 0.11 | 0 | 0.20339 | 1.1514 | 2.39076 | 0.01007 | 1 | 0.31318 | 1.24244 | 0.8829 | 0.0088 | 0 | 0.17533 | 1.12923 | 2.36754 | 0.02543 | 0 | 0 |
| 1396544_at | Rspo3 | R-spondin 3 | uniqAntib_1098 | -0.02 | 0.11 | 0.10 | -0.16 | 0.01 | 0 | -0.0096 | -1.0066 | 0.92444 | 0.51464 | 1 | -0.2746 | -1.2097 | 1.56932 | 0.03689 | 0 | -0.0992 | -1.0712 | 1.31226 | 0.1876 | 0 | 0 |
| 1396567_at | --- | --- | uniqAntib_1098 | -0.13 | -0.19 | 0.03 | -0.51 | -0.22 | 0 | 0.22487 | 1.16867 | 1.89135 | 0.06118 | 1 | -0.3196 | -1.248 | 2.37242 | 0.01007 | 0 | -0.0271 | -1.019 | 1.09995 | 0.31762 | 0 | 0 |
| 1396595_at | --- | --- | uniqAntib_1098 | -0.11 | -0.06 | -0.21 | -0.36 | -0.18 | 0 | -0.1535 | -1.1123 | 2.29258 | 0.0676 | 1 | -0.2972 | -1.2287 | 1.92318 | 0.01886 | 0 | -0.1165 | -1.0841 | 1.64589 | 0.09807 | 0 | 0 |
| 1396606_at | --- | --- | uniqAntib_1098 | -0.01 | -0.02 | -0.05 | 0.26 | -0.21 | 0 | -0.0313 | -1.0219 | 2.2638 | 0.07003 | 1 | 0.28015 | 1.21432 | 0.639 | 0.00431 | 0 | -0.1855 | -1.1372 | 2.03967 | 0.04762 | 0 | 0 |
| 1396616_at | --- | --- | uniqAntib_1098 | 0.00 | -0.11 | -0.09 | -0.39 | -0.29 | 0 | 0.02288 | 1.01598 | 0.99076 | 0.45637 | 1 | -0.2761 | -1.2109 | 1.46438 | 0.04502 | 0 | -0.1783 | -1.1315 | 1.45441 | 0.14156 | 0 | 0 |
| 1396629_at | Ormdl3 | ORM1-like 3 (S. cerevisiae) | uniqAntib_1098 | 0.18 | 0.00 | 0.04 | -0.32 | -0.11 | 0 | 0.03797 | 1.02667 | 0.9278 | 0.54578 | 1 | -0.321 | -1.2492 | 1.75371 | 0.02628 | 0 | -0.1165 | -1.0841 | 1.1921 | 0.25169 | 0 | 0 |
| 1396659_at | --- | --- | uniqAntib_1098 | 0.03 | 0.20 | -0.03 | -0.08 | 0.01 | 0 | -0.237 | -1.1785 | 2.08581 | 0.08444 | 1 | -0.2808 | -1.2148 | 1.43554 | 0.04861 | 0 | -0.1885 | -1.1395 | 1.58037 | 0.10667 | 0 | 0 |
| 1396669_at | --- | --- | uniqAntib_1098 | 0.01 | 0.02 | -0.02 | -0.25 | -0.06 | 0 | -0.0428 | -1.0301 | 0.92585 | 0.51242 | 1 | -0.2699 | -1.2057 | 1.49472 | 0.04244 | 0 | -0.084 | -1.06 | 1.09932 | 0.318 | 0 | 0 |
| 1396670_at | --- | --- | uniqAntib_1098 | 0.09 | -0.05 | -0.05 | -0.32 | -0.13 | 0 | -0.0005 | -1.0003 | 0.89254 | 0.57963 | 1 | -0.2702 | -1.206 | 1.5751 | 0.03464 | 0 | -0.0828 | -1.0591 | 1.49643 | 0.1254 | 0 | 0 |
| 1396679_at | --- | --- | uniqAntib_1098 | 0.02 | -0.03 | -0.06 | -0.34 | -0.05 | 0 | -0.0209 | -1.0146 | 1.0428 | 0.38491 | 1 | -0.3104 | -1.2401 | 1.99534 | 0.01585 | 0 | -0.011 | -1.0076 | 1.02628 | 0.38937 | 0 | 0 |
| 1396712_at | --- | --- | uniqAntib_1098 | -0.04 | -0.10 | 0.00 | -0.41 | -0.23 | 0 | 0.09408 | 1.06739 | 1.48295 | 0.16194 | 1 | -0.31 | -1.2397 | 2.13103 | 0.01336 | 0 | -0.1386 | -1.1009 | 1.87365 | 0.06543 | 0 | 0 |
| 1396717_at | --- | --- | uniqAntib_1098 | 0.14 | 0.15 | 0.40 | 0.48 | 0.26 | 0 | 0.25501 | 1.19335 | 2.2167 | 0.02138 | 1 | 0.33333 | 1.25992 | 0.99682 | 0.01439 | 0 | 0.11397 | 1.0822 | 1.66343 | 0.112 | 0 | 0 |
| 1396720_at | --- | --- | uniqAntib_1098 | 0.06 | -0.16 | -0.04 | -0.44 | -0.12 | 0 | 0.1195 | 1.08636 | 1.37449 | 0.20379 | 1 | -0.276 | -1.2108 | 1.79811 | 0.02174 | 0 | 0.04111 | 1.02891 | 1.24105 | 0.25153 | 0 | 0 |
| 1396724_at | --- | --- | uniqAntib_1098 | 0.26 | 0.26 | 0.28 | -0.07 | 0.03 | 0 | 0.02101 | 1.01467 | 1.13576 | 0.31689 | 1 | -0.3286 | -1.2558 | 2.14968 | 0.01313 | 0 | -0.2286 | -1.1717 | 2.32877 | 0.0346 | 0 | 0 |
| 1396813_at | --- | --- | uniqAntib_1098 | -0.04 | 0.12 | -0.12 | -0.18 | -0.05 | 0 | -0.2412 | -1.182 | 2.53088 | 0.04907 | 1 | -0.3049 | -1.2353 | 1.59913 | 0.03353 | 0 | -0.1701 | -1.1251 | 1.46761 | 0.1395 | 0 | 0 |
| 1396829_at | --- | --- | uniqAntib_1098 | -0.09 | 0.04 | -0.10 | -0.27 | -0.11 | 0 | -0.1466 | -1.107 | 1.54308 | 0.16647 | 1 | -0.319 | -1.2474 | 1.70231 | 0.02682 | 0 | -0.1518 | -1.111 | 1.36712 | 0.16766 | 0 | 0 |
| 1396863_at | --- | --- | uniqAntib_1098 | -0.13 | 0.06 | -0.16 | -0.24 | -0.07 | 0 | -0.2223 | -1.1666 | 2.64609 | 0.04203 | 1 | -0.2966 | -1.2283 | 1.77897 | 0.0238 | 0 | -0.1338 | -1.0972 | 1.18463 | 0.25655 | 0 | 0 |
| 1396865_at | --- | --- | uniqAntib_1098 | 0.06 | 0.17 | 0.13 | -0.19 | 0.10 | 0 | -0.043 | -1.0302 | 1.49695 | 0.17646 | 1 | -0.3582 | -1.2819 | 2.20773 | 0.01221 | 0 | -0.072 | -1.0512 | 1.20847 | 0.24023 | 0 | 0 |
| 1396884_at | --- | --- | uniqAntib_1098 | -0.16 | 0.17 | 0.43 | 0.78 | 0.22 | 0 | 0.25286 | 1.19157 | 2.29224 | 0.01216 | 1 | 0.60704 | 1.52313 | 0.13318 | 0.00038 | 0 | 0.04497 | 1.03166 | 1.09335 | 0.34035 | 0 | 0 |
| 1396930_x_at | --- | --- | uniqAntib_1098 | 0.10 | 0.02 | 0.09 | -0.25 | 0.07 | 0 | 0.06631 | 1.04703 | 1.04108 | 0.40674 | 1 | -0.2647 | -1.2014 | 1.42893 | 0.0493 | 0 | 0.04984 | 1.03515 | 1.0139 | 0.41689 | 0 | 0 |
| 1396935_at | --- | --- | uniqAntib_1098 | 0.04 | 0.12 | 0.08 | -0.18 | 0.00 | 0 | -0.0347 | -1.0244 | 0.94881 | 0.47582 | 1 | -0.2997 | -1.2309 | 1.83244 | 0.02056 | 0 | -0.1116 | -1.0804 | 1.32456 | 0.18293 | 0 | 0 |
| 1396975_at | --- | --- | uniqAntib_1098 | -0.06 | 0.11 | 0.15 | 0.41 | 0.29 | 0 | 0.04031 | 1.02834 | 1.76515 | 0.07952 | 1 | 0.3002 | 1.23132 | 1.07207 | 0.01851 | 0 | 0.17324 | 1.12759 | 1.71028 | 0.10037 | 0 | 0 |
| 1397036_at | --- | --- | uniqAntib_1098 | -0.01 | 0.14 | 0.07 | -0.25 | 0.11 | 0 | -0.0674 | -1.0479 | 2.01884 | 0.09374 | 1 | -0.3859 | -1.3066 | 3.5845 | 0.00231 | 0 | -0.0321 | -1.0225 | 1.23099 | 0.22784 | 0 | 0 |
| 1397037_at | Prrt2 | proline-rich transmembrane protein 2 | uniqAntib_1098 | 0.09 | 0.15 | 0.17 | -0.14 | 0.26 | 0 | 0.01872 | 1.01306 | 1.18309 | 0.29259 | 1 | -0.2889 | -1.2217 | 1.50764 | 0.04169 | 0 | 0.11264 | 1.0812 | 1.2428 | 0.25021 | 0 | 0 |
| 1397049_at | --- | --- | uniqAntib_1098 | -0.22 | 0.01 | -0.12 | -0.26 | -0.20 | 0 | -0.1286 | -1.0933 | 1.72795 | 0.13285 | 1 | -0.2676 | -1.2038 | 1.64454 | 0.03088 | 0 | -0.2101 | -1.1568 | 1.78408 | 0.07928 | 0 | 0 |
| 1397085_at | --- | --- | uniqAntib_1098 | 0.15 | 0.12 | -0.02 | -0.17 | -0.07 | 0 | -0.1371 | -1.0997 | 2.0395 | 0.08867 | 1 | -0.287 | -1.2201 | 1.90393 | 0.01855 | 0 | -0.1873 | -1.1386 | 1.96767 | 0.05429 | 0 | 0 |
| 1397138_at | --- | --- | uniqAntib_1098 | -0.10 | 0.08 | -0.06 | -0.18 | -0.10 | 0 | -0.1461 | -1.1066 | 2.34086 | 0.06488 | 1 | -0.2688 | -1.2048 | 1.79085 | 0.02246 | 0 | -0.1819 | -1.1344 | 1.97996 | 0.05291 | 0 | 0 |
| 1397175_at | --- | --- | uniqAntib_1098 | 0.07 | 0.10 | -0.05 | -0.23 | -0.06 | 0 | -0.1524 | -1.1114 | 2.22714 | 0.0734 | 1 | -0.3285 | -1.2557 | 1.79374 | 0.02296 | 0 | -0.1563 | -1.1144 | 1.55684 | 0.11129 | 0 | 0 |
| 1397179_at | Appl1 | adaptor protein, phosphotyrosine interaction, PH dc | uniqAntib_1098 | 0.13 | 0.10 | 0.07 | 0.37 | 0.11 | 0 | -0.0267 | -1.0187 | 1.32706 | 0.22834 | 1 | 0.27031 | 1.20607 | 1.14246 | 0.02252 | 0 | 0.00645 | 1.00448 | 1.4394 | 0.16935 | 0 | 0 |
| 1397180_at | Styxl1 | serine/threonine/tyrosine interacting-like 1 | uniqAntib_1098 | 0.06 | -0.16 | -0.24 | -0.49 | -0.30 | 0 | -0.0802 | -1.0572 | 1.05277 | 0.37336 | 1 | -0.3246 | -1.2523 | 1.88025 | 0.01965 | 0 | -0.1405 | -1.1023 | 1.27797 | 0.20518 | 0 | 0 |
| 1397286_at | --- | --- | uniqAntib_1098 | -0.15 | -0.48 | -0.24 | 0.03 | -0.40 | 0 | 0.23331 | 1.17553 | 1.79882 | 0.07277 | 1 | 0.5054 | 1.41952 | 0.3875 | 0.00159 | 0 | 0.07355 | 1.05231 | 1.26961 | 0.23556 | 0 | 0 |
| 1397303_at | --- | --- | uniqAntib_1098 | -0.04 | 0.24 | 0.37 | 0.66 | 0.23 | 0 | 0.13015 | 1.09441 | 1.66279 | 0.11538 | 1 | 0.42067 | 1.33855 | 0.57429 | 0.00336 | 0 | -0.0101 | -1.0071 | 1.47817 | 0.13385 | 0 | 0 |
| 1397404_at | --- | --- | uniqAntib_1098 | 0.03 | -0.17 | -0.05 | -0.44 | -0.15 | 0 | 0.11823 | 1.0854 | 1.7421 | 0.09181 | 1 | -0.2678 | -1.204 | 1.73502 | 0.02544 | 0 | 0.01645 | 1.01147 | 1.10127 | 0.33425 | 0 | 0 |
| 1397425_at | --- | --- | uniqAntib_1098 | 0.02 | 0.10 | -0.04 | 0.41 | 0.08 | 0 | -0.1367 | -1.0994 | 2.0533 | 0.08775 | 1 | 0.31141 | 1.24092 | 1.03065 | 0.01624 | 0 | -0.0239 | -1.0167 | 1.23274 | 0.22701 | 0 | 0 |
| 1397474_at | --- | --- | uniqAntib_1098 | 0.03 | -0.17 | 0.06 | 0.12 | -0.06 | 0 | 0.23013 | 1.17294 | 1.78328 | 0.07437 | 1 | 0.29171 | 1.22409 | 1.25179 | 0.03562 | 0 | 0.1126 | 1.08118 | 1.27865 | 0.23308 | 0 | 0 |
| 1397485_at | Casp7 | caspase 7 | uniqAntib_1098 | -0.01 | -0.12 | -0.28 | -0.39 | -0.27 | 0 | -0.154 | -1.1126 | 1.41496 | 0.19824 | 1 | -0.2666 | -1.203 | 1.55599 | 0.03753 | 0 | -0.1485 | -1.1084 | 1.32961 | 0.18213 | 0 | 0 |
| 1397491_at | Agpat3 | 1-acylglycerol-3-phosphate O-acyltransferase 3 | uniqAntib_1098 | 0.12 | 0.04 | 0.11 | -0.22 | -0.03 | 0 | 0.07177 | 1.05101 | 1.2898 | 0.23653 | 1 | -0.266 | -1.2025 | 1.65915 | 0.0295 | 0 | -0.0694 | -1.0493 | 1.30445 | 0.1914 | 0 | 0 |
| 1397525_at | Alkbh5 | alkB, alkylation repair homolog 5 (E. coli) | uniqAntib_1098 | 0.17 | 0.50 | 0.69 | 0.77 | 0.70 | 0 | 0.19036 | 1.14105 | 2.49284 | 0.00649 | 1 | 0.26991 | 1.20574 | 0.92776 | 0.00987 | 0 | 0.20315 | 1.15121 | 2.17958 | 0.01002 | 0 | 0 |
| 1397535_at | Mpp5 | membrane protein, palmitoylated 5 (MAGUK p55 su | uniqAntib_1098 | -0.09 | -0.09 | -0.06 | 0.26 | 0.00 | 0 | 0.03074 | 1.02154 | 1.01193 | 0.4329 | 1 | 0.35216 | 1.27647 | 1.04495 | 0.01683 | 0 | 0.09407 | 1.06738 | 1.35225 | 0.19893 | 0 | 0 |
| 1397573_at | LOC10091238 | uncharacterized LOC100912383 | uniqAntib_1098 | 0.02 | 0.13 | 0.11 | -0.21 | 0.10 | 0 | -0.0238 | -1.0166 | 0.92672 | 0.51153 | 1 | -0.3402 | -1.2659 | 1.94712 | 0.01803 | 0 | -0.0324 | -1.0227 | 0.94949 | 0.5125 | 0 | 0 |
| 1397579_x_at | Apc2 | adenomatosis polyposis coli 2 | uniqAntib_1098 | -0.07 | 0.05 | 0.02 | -0.28 | 0.05 | 0 | -0.0331 | -1.0232 | 0.93257 | 0.50126 | 1 | -0.337 | -1.2631 | 1.80656 | 0.02144 | 0 | -0.0069 | -1.0048 | 0.93803 | 0.534 | 0 | 0 |
| 1397632_at | Slitrk1 | SLIT and NTRK-like family, member 1 | uniqAntib_1098 | -0.03 | 0.07 | -0.04 | -0.23 | -0.05 | 0 | -0.1065 | -1.0766 | 1.21051 | 0.27683 | 1 | -0.295 | -1.2269 | 1.6142 | 0.03187 | 0 | -0.1208 | -1.0874 | 1.22489 | 0.23104 | 0 | 0 |
| 1397666_at | --- | --- | uniqAntib_1098 | 0.05 | -0.21 | -0.12 | 0.11 | -0.30 | 0 | 0.09001 | 1.06438 | 1.54037 | 0.14612 | 1 | 0.3207 | 1.24894 | 1.02838 | 0.01614 | 0 | -0.085 | -1.0607 | 1.35818 | 0.17203 | 0 | 0 |
| 1397675_at | Eif4h | eukaryotic translation initiation factor 4H | uniqAntib_1098 | 0.20 | 0.24 | 0.49 | 0.53 | 0.34 | 0 | 0.25694 | 1.19494 | 2.27874 | 0.01282 | 1 | 0.29412 | 1.22614 | 0.99183 | 0.01413 | 0 | 0.10267 | 1.07376 | 1.89535 | 0.0749 | 0 | 0 |
| 1397745_at | --- | --- | uniqAntib_1098 | -0.33 | -0.07 | 0.02 | 0.26 | 0.04 | 0 | 0.0893 | 1.06385 | 1.24567 | 0.25551 | 1 | 0.3269 | 1.25431 | 1.20364 | 0.02818 | 0 | 0.10507 | 1.07554 | 1.1273 | 0.31453 | 0 | 0 |
| 1397798_at | --- | --- | uniqAntib_1098 | 0.11 | -0.06 | -0.12 | 0.28 | 0.11 | 0 | -0.0645 | -1.0457 | 1.65234 | 0.14494 | 1 | 0.3417 | 1.26725 | 0.6334 | 0.00426 | 0 | 0.16789 | 1.12341 | 2.08844 | 0.0544 | 0 | 0 |
| 1397932_at | --- | --- | uniqAntib_1098 | 0.02 | 0.11 | 0.10 | -0.18 | 0.05 | 0 | -0.0071 | -1.0049 | 1.30994 | 0.23622 | 1 | -0.2913 | -1.2237 | 2.17532 | 0.01301 | 0 | -0.0565 | -1.0399 | 1.19452 | 0.24982 | 0 | 0 |
| 1397936_at | Foxr1 | forkhead box R1 | uniqAntib_1098 | -0.30 | -0.11 | -0.23 | -0.39 | -0.24 | 0 | -0.1156 | -1.0835 | 1.53803 | 0.1679 | 1 | -0.2804 | -1.2145 | 1.50611 | 0.04223 | 0 | -0.1334 | -1.0969 | 1.42271 | 0.15106 | 0 | 0 |
| 1397977_at | --- | --- | uniqAntib_1098 | -0.16 | -0.24 | 0.02 | 0.14 | -0.16 | 0 | 0.25421 | 1.19269 | 1.86384 | 0.06275 | 1 | 0.3745 | 1.29639 | 0.96456 | 0.01259 | 0 | 0.08082 | 1.05762 | 1.13669 | 0.30871 | 0 | 0 |
| 1397998_at | LOC10036545 | rCG26795-like | uniqAntib_1098 | 0.08 | 0.03 | 0.01 | -0.30 | -0.06 | 0 | -0.0195 | -1.0136 | 0.95987 | 0.46214 | 1 | -0.3326 | -1.2593 | 1.81052 | 0.02137 | 0 | -0.095 | -1.0681 | 1.18432 | 0.2566 | 0 | 0 |
| 1398095_at | --- | --- | uniqAntib_1098 | 0.14 | 0.10 | 0.05 | -0.22 | 0.00 | 0 | -0.0513 | -1.0362 | 1.13317 | 0.31985 | 1 | -0.3195 | -1.2479 | 1.78595 | 0.02366 | 0 | -0.1089 | -1.0784 | 1.26745 | 0.21115 | 0 | 0 |
| 1398166_at | --- | --- | uniqAntib_1098 | 0.05 | 0.12 | 0.15 | -0.18 | -0.04 | 0 | 0.03018 | 1.02114 | 1.10086 | 0.34907 | 1 | -0.3008 | -1.2319 | 1.56513 | 0.03563 | 0 | -0.1584 | -1.1161 | 1.59117 | 0.1054 | 0 | 0 |
| 1398209_at | --- | --- | uniqAntib_1098 | -0.11 | 0.01 | 0.18 | 0.28 | 0.20 | 0 | 0.16898 | 1.12427 | 1.69469 | 0.10436 | 1 | 0.26715 | 1.20343 | 1.2789 | 0.04186 | 0 | 0.19252 | 1.14276 | 2.07028 | 0.05872 | 0 | 0 |
| 1398218_at | --- | --- | uniqAntib_1098 | 0.16 | -0.03 | 0.17 | 0.24 | 0.08 | 0 | 0.19809 | 1.14717 | 2.07224 | 0.03358 | 1 | 0.26566 | 1.20219 | 1.20694 | 0.02915 | 0 | 0.11137 | 1.08026 | 1.46599 | 0.16122 | 0 | 0 |
| 1398237_at | --- | --- | uniqAntib_1098 | 0.01 | 0.01 | 0.04 | -0.26 | -0.19 | 0 | 0.0268 | 1.01875 | 1.00723 | 0.43759 | 1 | -0.2701 | -1.2059 | 1.47278 | 0.04418 | 0 | -0.1944 | -1.1443 | 1.83989 | 0.07283 | 0 | 0 |
| 1398304_at | Fzd2 | frizzled family receptor 2 | uniqAntib_1098 | 0.06 | 0.07 | 0.20 | -0.21 | -0.19 | 0 | 0.13236 | 1.09609 | 1.93292 | 0.05668 | 1 | -0.2813 | -1.2153 | 3.11846 | 0.00521 | 0 | -0.2615 | -1.1988 | 3.10339 | 0.00619 | 0 | 0 |
| 1398365_at | Tppp3 | tubulin polymerization-promoting protein family me | uniqAntib_1098 | 0.15 | -0.05 | -0.04 | -0.37 | 0.00 | 0 | 0.01433 | 1.00998 | 1.18564 | 0.34617 | 1 | -0.322 | -1.2501 | 2.77696 | 0.00616 | 0 | 0.05212 | 1.03679 | 1.25315 | 0.24504 | 0 | 0 |
| 1398409_at | St13 | suppression of tumorigenicity 13 | uniqAntib_1098 | 0.05 | 0.21 | 0.10 | 0.48 | 0.19 | 0 | -0.1061 | -1.0763 | 1.22885 | 0.26526 | 1 | 0.26891 | 1.2049 | 1.26449 | 0.03867 | 0 | -0.0133 | -1.0093 | 0.94748 | 0.51632 | 0 | 0 |
| 1398420_at | Smurf2 | SMAD specific E3 ubiquitin protein ligase 2 | uniqAntib_1098 | -0.07 | -0.03 | 0.09 | 0.27 | 0.09 | 0 | 0.12541 | 1.09082 | 1.75924 | 0.08016 | 1 | 0.30712 | 1.23724 | 0.97342 | 0.0129 | 0 | 0.12692 | 1.09196 | 1.69788 | 0.1033 | 0 | 0 |
| 1398429_at | Pdhx | pyruvate dehydrogenase complex, component X | uniqAntib_1098 | -0.12 | -0.34 | -0.40 | 0.05 | -0.27 | 0 | -0.0672 | -1.0477 | 1.01051 | 0.41432 | 1 | 0.38763 | 1.30824 | 0.9233 | 0.00971 | 0 | 0.06537 | 1.04635 | 1.06055 | 0.37011 | 0 | 0 |
| 1398439_a_at | Orc6 | origin recognition complex, subunit 6 | uniqAntib_1098 | 0.07 | -0.03 | 0.01 | 0.28 | 0.08 | 0 | 0.03964 | 1.02786 | 1.48184 | 0.16382 | 1 | 0.31043 | 1.24008 | 0.97106 | 0.01324 | 0 | 0.10214 | 1.07336 | 2.08568 | 0.05352 | 0 | 0 |
| 1398460_at | RGD1311723 | similar to KIAA1731 protein | uniqAntib_1098 | -0.18 | -0.05 | -0.06 | 0.22 | 0.09 | 0 | -0.0124 | -1.0086 | 1.11739 | 0.33681 | 1 | 0.26653 | 1.20291 | 1.28079 | 0.04806 | 0 | 0.13856 | 1.1008 | 1.47336 | 0.15791 | 0 | 0 |
| 1398522_at | --- | --- | uniqAntib_1098 | 0.05 | -0.05 | 0.12 | 0.23 | 0.09 | 0 | 0.16711 | 1.12281 | 2.06407 | 0.03942 | 1 | 0.27676 | 1.21147 | 1.0427 | 0.01686 | 0 | 0.13462 | 1.09781 | 2.12054 | 0.05141 | 0 | 0 |
| 1398553_at | Tmed5 | transmembrane emp24 protein transport domain cc | uniqAntib_1098 | -0.27 | -0.54 | -0.37 | 0.01 | -0.52 | 0 | 0.1755 | 1.12936 | 2.09 | 0.02675 | 1 | 0.55424 | 1.4684 | 0.12244 | 0.00032 | 0 | 0.01946 | 1.01358 | 1.54742 | 0.13912 | 0 | 0 |
| 1398600_at | --- | --- | uniqAntib_1098 | -0.04 | 0.04 | -0.08 | 0.36 | 0.04 | 0 | -0.1137 | -1.082 | 1.46677 | 0.18309 | 1 | 0.32455 | 1.25227 | 1.07454 | 0.01811 | 0 | -0.0007 | -1.0005 | 1.05241 | 0.36061 | 0 | 0 |
| 1398604_at | Osbp2 | oxysterol binding protein 2 | uniqAntib_1098 | 0.03 | -0.11 | -0.03 | -0.39 | -0.11 | 0 | 0.07952 | 1.05667 | 1.14946 | 0.31284 | 1 | -0.283 | -1.2167 | 1.60416 | 0.03337 | 0 | -0.0006 | -1.0004 | 1.09749 | 0.33713 | 0 | 0 |
| 1398613_at | --- | --- | uniqAntib_1098 | 0.00 | 0.06 | -0.07 | 0.36 | 0.23 | 0 | -0.1259 | -1.0912 | 2.4906 | 0.05342 | 1 | 0.30575 | 1.23606 | 1.01026 | 0.01498 | 0 | 0.17455 | 1.12861 | 2.28099 | 0.0355 | 0 | 0 |
| 1398630_at | Hmgb1 | high mobility group box 1 | uniqAntib_1098 | 0.01 | 0.02 | -0.10 | -0.34 | -0.16 | 0 | -0.116 | -1.0837 | 1.51719 | 0.17309 | 1 | -0.3552 | -1.2792 | 2.53904 | 0.00767 | 0 | -0.1742 | -1.1284 | 1.53105 | 0.11496 | 0 | 0 |
| 1398656_at | --- | --- | uniqAntib_1098 | 0.03 | -0.01 | -0.01 | -0.30 | -0.15 | 0 | -0.0031 | -1.0021 | 0.90832 | 0.54568 | 1 | -0.2912 | -1.2236 | 1.57085 | 0.03465 | 0 | -0.1432 | -1.1044 | 1.44868 | 0.14296 | 0 | 0 |
| 1398668_at | --- | --- | uniqAntib_1098 | -0.11 | 0.07 | -0.05 | -0.20 | -0.12 | 0 | -0.1115 | -1.0803 | 1.35228 | 0.21985 | 1 | -0.2664 | -1.2028 | 1.42795 | 0.04936 | 0 | -0.1828 | -1.1351 | 1.64594 | 0.09844 | 0 | 0 |

| Affymetrix Probeset ID | Gene Symbol | Gene Title | Figure 6 B Venn n =2021 | Mean BN log2 intensity autologous n=8 | Mean log2 intensity day 30 DR +/+ ND n=4 | Mean log2 intensity day 30 DR+/+ HCD n=4 | Mean log2 intensity day 30 DR+/+ B/S n=4 | Mean log2 intensity day 30 F+/+ ND n=4 | DR+/+ HCD vs DR+/+ ND n=380 1=yes 0=no | Log2 ratio DR+/+ HCD vs DR+/+ ND | Fold-Change DR+/+ HCD vs DR+/+ ND | RP-FDR 4DR30 HCD vs 4DR30 Reg | p value DR+/+ HCD vs DR+/+ ND | DR+/+ B/S vs DR+/+ ND n=1,330 1=yes 0=no | Log2 ratio DR+/+ B/S vs DR+/+ ND | Fold-Change DR+/+ B/S vs DR+/+ ND | RP-FDR 4DR30 Antib vs 4DR30 Reg | p value DR+/+ B/S vs DR+/+ ND | DR+/+ ND vs F+/+ ND n=398 1=yes 0=no | Log2 ratio DR+/+ ND vs F+/+ ND | Fold-Change DR+/+ ND vs F+/+ ND | RP-FDR 4RT1uu 30Reg vs 4DR30 Reg | p value DR+/+ ND vs F+/+ ND | DR+/+ Longitudinal n=228 1=yes 0=no | Figure 6F |
|---|---|---|---|---|---|---|---|---|---|---|---|---|---|---|---|---|---|---|---|---|---|---|---|---|---|
| 1398714_at | Usf2 | upstream transcription factor 2, c-fos interacting | uniqAntib_1098 | 0.13 | 0.27 | 0.16 | -0.07 | 0.05 | 0 | -0.1087 | -1.0783 | 1.35599 | 0.21862 | 1 | -0.3473 | -1.2722 | 2.08505 | 0.01408 | 0 | -0.2257 | -1.1694 | 2.04192 | 0.0476 | 0 | 0 |
| 1398808_at | Impa1 | Inositol (myo)-1(or 4)-monophosphatase 1 | uniqAntib_1098 | -0.06 | -0.27 | -0.19 | 0.01 | -0.23 | 0 | 0.0791 | 1.05636 | 1.33278 | 0.21839 | 1 | 0.27271 | 1.20807 | 1.26321 | 0.03867 | 0 | 0.04033 | 1.02835 | 1.0249 | 0.40292 | 0 | 0 |
| 1398826_s_at | Nr2f6 | nuclear receptor subfamily 2, group F, member 6 | uniqAntib_1098 | 0.00 | -0.01 | 0.12 | 0.29 | 0.18 | 0 | 0.13079 | 1.09489 | 1.24007 | 0.25847 | 1 | 0.30367 | 1.23428 | 1.20507 | 0.02906 | 0 | 0.19368 | 1.14368 | 1.80563 | 0.08512 | 0 | 0 |
| 1398866_at | Magi3 | membrane associated guanylate kinase, WW and PI | uniqAntib_1098 | -0.01 | 0.32 | 0.43 | 0.72 | 0.34 | 0 | 0.10714 | 1.07709 | 1.7392 | 0.09362 | 1 | 0.4007 | 1.32015 | 0.56604 | 0.00289 | 0 | 0.023 | 1.01607 | 1.30567 | 0.21878 | 0 | 0 |
| 1399024_at | Scyl1 | SCY1-like 1 (S. cerevisiae) | uniqAntib_1098 | -0.17 | -0.30 | -0.14 | -0.02 | -0.17 | 0 | 0.15702 | 1.11498 | 1.51571 | 0.15328 | 1 | 0.28284 | 1.21659 | 1.25622 | 0.03741 | 0 | 0.12868 | 1.09329 | 1.3135 | 0.21502 | 0 | 0 |
| 1399037_at | Ttc19 | tetratricopeptide repeat domain 19 | uniqAntib_1098 | -0.06 | -0.19 | -0.14 | 0.14 | -0.11 | 0 | 0.05434 | 1.03838 | 1.0781 | 0.36695 | 1 | 0.32662 | 1.25407 | 1.15851 | 0.02306 | 0 | 0.08304 | 1.05925 | 1.21368 | 0.26366 | 0 | 0 |
| 1399061_at | --- | --- | uniqAntib_1098 | -0.01 | -0.04 | -0.03 | 0.26 | 0.06 | 0 | 0.00858 | 1.00596 | 0.94567 | 0.51585 | 1 | 0.30048 | 1.23155 | 1.2558 | 0.03634 | 0 | 0.09951 | 1.07141 | 1.26802 | 0.23596 | 0 | 0 |
| 1367743_at | Pfkl | phosphofructokinase, liver | uniqHCD_268 | 0.01 | 0.32 | -0.03 | 0.14 | 0.19 | 1 | -0.3577 | -1.2814 | 3.53679 | 0.00318 | 0 | -0.1868 | -1.1383 | 1.29235 | 0.07044 | 0 | -0.1297 | -1.094 | 1.74332 | 0.08577 | 0 | 0 |
| 1367755_at | Cdo1 | cysteine dioxygenase, type I | uniqHCD_268 | -0.21 | -0.23 | 0.04 | -0.28 | -0.08 | 1 | 0.27058 | 1.20629 | 2.2548 | 0.02023 | 0 | -0.0476 | -1.0336 | 0.98432 | 0.30496 | 0 | 0.14768 | 1.10779 | 2.0783 | 0.05674 | 0 | 0 |
| 1368169_at | Pick1 | protein interacting with PRKCA 1 | uniqHCD_268 | 0.10 | 0.11 | 0.40 | 0.14 | 0.21 | 1 | 0.28337 | 1.21703 | 2.32717 | 0.01137 | 0 | 0.02392 | 1.01672 | 1.12805 | 0.2348 | 0 | 0.09408 | 1.06738 | 1.52985 | 0.14477 | 0 | 0 |
| 1368282_at | Dpep1 | dipeptidase 1 (renal) | uniqHCD_268 | -0.02 | -0.13 | 0.16 | -0.08 | -0.19 | 1 | 0.28253 | 1.21632 | 2.24039 | 0.02032 | 0 | 0.04415 | 1.03108 | 1.09359 | 0.27734 | 0 | -0.0626 | -1.0443 | 1.32106 | 0.18359 | 0 | 0 |
| 1368408_at | Grk5 | G protein-coupled receptor kinase 5 | uniqHCD_268 | 0.07 | 0.09 | -0.46 | 0.07 | -0.05 | 1 | -0.546 | -1.4601 | 1.3675 | 0.00018 | 0 | -0.0172 | -1.012 | 1.02779 | 0.22497 | 0 | -0.1348 | -1.098 | 1.874 | 0.0655 | 0 | 0 |
| 1368449_at | Adap1 | ArfGAP with dual PH domains 1 | uniqHCD_268 | -0.02 | -0.10 | 0.22 | -0.02 | -0.02 | 1 | 0.32799 | 1.25527 | 2.2678 | 0.01327 | 0 | 0.08715 | 1.06227 | 1.12213 | 0.24269 | 0 | 0.08625 | 1.0616 | 1.41488 | 0.17739 | 0 | 0 |
| 1368529_at | Cd99l2 /// LO | CD99 molecule-like 2 /// CD99 antigen-like protein | uniqHCD_268 | 0.06 | 0.27 | -0.01 | 0.15 | 0.05 | 1 | -0.2756 | -1.2105 | 2.8529 | 0.03413 | 0 | -0.1169 | -1.0844 | 1.07 | 0.16088 | 0 | -0.2204 | -1.1651 | 1.93801 | 0.05758 | 0 | 0 |
| 1368555_at | Cd37 | CD37 molecule | uniqHCD_268 | -0.04 | -0.04 | -0.38 | -0.01 | -0.04 | 1 | -0.3443 | -1.2695 | 3.70972 | 0.00859 | 0 | 0.02989 | 1.02094 | 0.99218 | 0.4986 | 0 | -0.0096 | -1.0067 | 0.91045 | 0.62012 | 0 | 0 |
| 1368688_at | Ntsr2 | neurotensin receptor 2 | uniqHCD_268 | -0.01 | -0.06 | 0.23 | 0.02 | 0.00 | 1 | 0.28765 | 1.22065 | 2.1383 | 0.02551 | 0 | 0.07806 | 1.0556 | 1.09763 | 0.27212 | 0 | 0.06358 | 1.04506 | 1.16831 | 0.28946 | 0 | 0 |
| 1368772_at | Slc4a3 | solute carrier family 4 (anion exchanger), member 3 | uniqHCD_268 | 0.25 | 0.18 | -0.12 | 0.00 | 0.02 | 1 | -0.3079 | -1.2379 | 3.15867 | 0.02214 | 0 | -0.1815 | -1.134 | 1.09316 | 0.14377 | 0 | -0.1612 | -1.1182 | 1.67734 | 0.09584 | 0 | 0 |
| 1368800_at | Cd40lg | CD40 ligand | uniqHCD_268 | -0.04 | 0.14 | -0.13 | 0.02 | 0.28 | 1 | -0.2719 | -1.2074 | 2.75077 | 0.03786 | 0 | -0.1213 | -1.0877 | 1.25137 | 0.0777 | 0 | 0.13541 | 1.0984 | 1.83808 | 0.0821 | 0 | 0 |
| 1368882_at | St6galnac3 | ST6 (alpha-N-acetyl-neuraminyl-2,3-beta-galactosyl | uniqHCD_268 | -0.16 | 0.12 | -0.16 | 0.03 | 0.00 | 1 | -0.2817 | -1.2157 | 3.3585 | 0.0122 | 0 | -0.0961 | -1.0689 | 1.27779 | 0.0733 | 0 | -0.1246 | -1.0902 | 1.67771 | 0.09581 | 0 | 0 |
| 1368975_at | Cd38 | CD38 molecule | uniqHCD_268 | -0.06 | -0.01 | -0.37 | -0.10 | -0.07 | 1 | -0.3611 | -1.2844 | 3.648 | 0.00645 | 0 | -0.0954 | -1.0684 | 0.97877 | 0.31853 | 0 | -0.0598 | -1.0423 | 1.16929 | 0.26601 | 0 | 0 |
| 1368976_at | Cd38 | CD38 molecule | uniqHCD_268 | 0.05 | 0.00 | -0.34 | -0.19 | 0.00 | 1 | -0.3426 | -1.2681 | 3.33741 | 0.01449 | 0 | -0.1916 | -1.142 | 1.08188 | 0.1515 | 0 | -0.0046 | -1.0032 | 0.91957 | 0.57911 | 0 | 0 |
| 1369027_at | A4galt | alpha 1,4-galactosyltransferase | uniqHCD_268 | 0.03 | 0.02 | -0.26 | -0.10 | -0.01 | 1 | -0.2806 | -1.2147 | 3.04793 | 0.02607 | 0 | -0.1208 | -1.0874 | 1.03515 | 0.20694 | 0 | -0.0244 | -1.017 | 1.41938 | 0.15235 | 0 | 0 |
| 1369055_at | Lpar3 | lysophosphatidic acid receptor 3 | uniqHCD_268 | 0.06 | -0.01 | 0.27 | -0.08 | -0.02 | 1 | 0.27879 | 1.21318 | 1.98665 | 0.04421 | 0 | -0.0672 | -1.0477 | 0.9663 | 0.40278 | 0 | -0.0039 | -1.0027 | 1.02094 | 0.39647 | 0 | 0 |
| 1369151_at | Dlk1 | delta-like 1 homolog (Drosophila) | uniqHCD_268 | 0.04 | -0.13 | 0.14 | -0.19 | -0.08 | 1 | 0.26941 | 1.20532 | 2.00204 | 0.04352 | 0 | -0.0552 | -1.039 | 0.96328 | 0.4452 | 0 | 0.05521 | 1.03901 | 1.11835 | 0.32045 | 0 | 0 |
| 1369193_at | Cdkn2b | cyclin-dependent kinase inhibitor 2B (p15, inhibits C | uniqHCD_268 | 0.10 | -0.07 | 0.22 | -0.09 | -0.11 | 1 | 0.28328 | 1.21696 | 2.12726 | 0.02374 | 0 | -0.0272 | -1.019 | 1.03574 | 0.20572 | 0 | -0.0478 | -1.0337 | 1.25125 | 0.22004 | 0 | 0 |
| 1369227_at | Chm | choroideremia (Rab escort protein 1) | uniqHCD_268 | -0.04 | -0.23 | -0.60 | -0.16 | -0.38 | 1 | -0.364 | -1.287 | 3.5893 | 0.00496 | 0 | 0.06788 | 1.04817 | 1.09224 | 0.28125 | 0 | -0.1481 | -1.1081 | 1.74164 | 0.08356 | 0 | 0 |
| 1369388_at | Musk | muscle, skeletal, receptor tyrosine kinase | uniqHCD_268 | 0.05 | -0.01 | -0.29 | -0.09 | -0.14 | 1 | -0.2785 | -1.2129 | 2.60586 | 0.04273 | 0 | -0.083 | -1.0592 | 0.96775 | 0.37354 | 0 | -0.1295 | -1.0939 | 1.25266 | 0.21795 | 0 | 0 |
| 1369545_at | Egr3 | early growth response 3 | uniqHCD_268 | 0.10 | 0.22 | -0.05 | 0.06 | -0.01 | 1 | -0.2707 | -1.2064 | 3.26386 | 0.01333 | 0 | -0.1664 | -1.1222 | 1.18824 | 0.09457 | 0 | -0.2378 | -1.1792 | 2.65834 | 0.02522 | 0 | 0 |
| 1369585_at | Tore | trispanning orphan | uniqHCD_268 | 0.12 | -0.03 | 0.25 | 0.00 | 0.15 | 1 | 0.27867 | 1.21307 | 2.08212 | 0.03133 | 0 | 0.02871 | 1.0201 | 1.03435 | 0.38016 | 0 | 0.18278 | 1.13507 | 1.66197 | 0.11313 | 0 | 0 |
| 1369817_at | Hand2 | heart and neural crest derivatives expressed 2 | uniqHCD_268 | 0.07 | -0.20 | 0.15 | -0.17 | -0.09 | 1 | 0.34539 | 1.27049 | 2.2875 | 0.01353 | 0 | 0.03425 | 1.02402 | 1.06273 | 0.29972 | 0 | 0.10728 | 1.07719 | 1.34127 | 0.20383 | 0 | 0 |
| 1369837_at | Gulo | gulonolactone (L-) oxidase | uniqHCD_268 | 0.08 | -0.19 | 0.13 | -0.20 | -0.15 | 1 | 0.31699 | 1.24573 | 2.22369 | 0.02131 | 0 | -0.0121 | -1.0084 | 0.96303 | 0.51367 | 0 | 0.03908 | 1.02746 | 0.99744 | 0.43315 | 0 | 0 |
| 1370167_at | Sdc2 | syndecan 2 | uniqHCD_268 | 0.04 | -0.01 | -0.30 | -0.17 | -0.04 | 1 | -0.2926 | -1.2249 | 3.06652 | 0.02692 | 0 | -0.1613 | -1.1183 | 1.12211 | 0.12932 | 0 | -0.0305 | -1.0213 | 1.04911 | 0.3645 | 0 | 0 |
| 1370266_at | Parva | parvin, alpha | uniqHCD_268 | 0.08 | 0.19 | -0.21 | 0.09 | 0.15 | 1 | -0.3979 | -1.3176 | 2.70889 | 0.00157 | 0 | -0.1016 | -1.0729 | 1.0847 | 0.14893 | 0 | -0.0328 | -1.023 | 1.11879 | 0.3 | 0 | 0 |
| 1370377_at | Cyp2d1 /// Cy | cytochrome P450, family 2, subfamily d, polypeptide | uniqHCD_268 | -0.08 | -0.28 | -0.01 | -0.20 | -0.20 | 1 | 0.26858 | 1.20462 | 2.21958 | 0.02041 | 0 | 0.0828 | 1.05907 | 1.11725 | 0.25069 | 0 | 0.07966 | 1.05677 | 1.68286 | 0.10622 | 0 | 0 |
| 1370394_at | IgG-2a | gamma-2a immunoglobulin heavy chain | uniqHCD_268 | -0.10 | -0.16 | -0.55 | -0.15 | -0.23 | 1 | -0.3887 | -1.3092 | 3.60778 | 0.00104 | 0 | 0.01692 | 1.01179 | 1.31434 | 0.06238 | 0 | -0.0637 | -1.0451 | 1.47484 | 0.13492 | 0 | 0 |
| 1370462_at | Hmmr | hyaluronan mediated motility receptor (RHAMM) | uniqHCD_268 | 0.16 | 0.09 | -0.32 | -0.04 | -0.12 | 1 | -0.4123 | -1.3308 | 2.87696 | 0.00213 | 0 | -0.1337 | -1.0971 | 1.18079 | 0.09902 | 0 | -0.2081 | -1.1552 | 2.27212 | 0.03485 | 0 | 0 |
| 1370656_a_at | Homer1 | homer homolog 1 (Drosophila) | uniqHCD_268 | -0.01 | -0.28 | -0.01 | -0.17 | -0.12 | 1 | 0.26839 | 1.20446 | 2.23016 | 0.02247 | 0 | 0.10726 | 1.07718 | 1.14229 | 0.22248 | 0 | 0.1577 | 1.11551 | 1.91921 | 0.07066 | 0 | 0 |
| 1370680_at | Stau2 | staufen, RNA binding protein, homolog 2 (Drosophil | uniqHCD_268 | 0.01 | -0.33 | -0.62 | -0.31 | -0.53 | 1 | -0.2857 | -1.219 | 3.27868 | 0.00401 | 0 | 0.01464 | 1.0102 | 1.15136 | 0.1117 | 0 | -0.1975 | -1.1467 | 2.93887 | 0.01257 | 0 | 0 |
| 1370799_at | Slc9a1 | solute carrier family 9, subfamily A (NHE1, cation pr | uniqHCD_268 | -0.06 | -0.16 | 0.13 | -0.21 | -0.17 | 1 | 0.29365 | 1.22574 | 2.13791 | 0.02461 | 0 | -0.0449 | -1.0316 | 0.99308 | 0.28526 | 0 | -0.0085 | -1.0059 | 1.00076 | 0.43044 | 0 | 0 |
| 1370831_at | Mgll | monoglyceride lipase | uniqHCD_268 | 0.21 | -0.20 | 0.07 | -0.19 | -0.05 | 1 | 0.26758 | 1.20379 | 2.39172 | 0.00715 | 0 | 0.00966 | 1.00672 | 1.19323 | 0.16702 | 0 | 0.15097 | 1.11031 | 2.34051 | 0.01919 | 0 | 0 |
| 1370991_at | Cml3 | camello-like 3 | uniqHCD_268 | 0.07 | -0.13 | 0.16 | 0.00 | -0.06 | 1 | 0.28447 | 1.21797 | 2.08227 | 0.03308 | 0 | 0.12278 | 1.08883 | 1.13124 | 0.22906 | 0 | 0.06846 | 1.0486 | 1.12709 | 0.31469 | 0 | 0 |
| 1371950_at | Pdzd11 | PDZ domain containing 11 | uniqHCD_268 | -0.12 | -0.18 | -0.54 | -0.30 | -0.35 | 1 | -0.354 | -1.2781 | 3.2968 | 0.01325 | 0 | -0.1183 | -1.0855 | 1.03216 | 0.21052 | 0 | -0.1668 | -1.1226 | 1.57158 | 0.10976 | 0 | 0 |
| 1372170_at | Acy1 | aminoacylase 1 | uniqHCD_268 | -0.02 | 0.05 | -0.24 | 0.29 | 0.01 | 1 | -0.2884 | -1.2213 | 2.65967 | 0.03908 | 0 | 0.24437 | 1.18458 | 1.29112 | 0.07066 | 0 | -0.0436 | -1.0307 | 0.94705 | 0.51718 | 0 | 0 |
| 1372219_at | Tpm2 | tropomyosin 2, beta | uniqHCD_268 | -0.03 | -0.25 | -0.57 | -0.42 | -0.39 | 1 | -0.3178 | -1.2465 | 3.0575 | 0.00197 | 0 | -0.1746 | -1.1286 | 3.22886 | 0.00457 | 0 | -0.1383 | -1.1006 | 2.9912 | 0.01202 | 0 | 0 |
| 1372809_at | LOC290595 | hypothetical gene supported by AF152002 | uniqHCD_268 | 0.16 | -0.02 | 0.26 | 0.06 | 0.13 | 1 | 0.27969 | 1.21394 | 1.96494 | 0.04518 | 0 | 0.07768 | 1.05532 | 1.10206 | 0.26713 | 0 | 0.14661 | 1.10697 | 1.63493 | 0.12018 | 0 | 0 |
| 1372887_at | Scarf2 | scavenger receptor class F, member 2 | uniqHCD_268 | -0.02 | -0.20 | 0.10 | -0.16 | -0.07 | 1 | 0.30453 | 1.23501 | 2.15704 | 0.02317 | 0 | 0.0358 | 1.02513 | 1.02468 | 0.40521 | 0 | 0.13446 | 1.09768 | 1.48871 | 0.15294 | 0 | 0 |
| 1373656_at | Ppp1r3d | protein phosphatase 1, regulatory subunit 3D | uniqHCD_268 | 0.11 | 0.10 | 0.37 | 0.31 | 0.35 | 1 | 0.26831 | 1.2044 | 2.26057 | 0.01403 | 0 | 0.21717 | 1.16245 | 1.25756 | 0.03668 | 0 | 0.2491 | 1.18847 | 2.34682 | 0.0197 | 0 | 0 |
| 1373671_at | Pet100 | PET100 homolog (S. cerevisiae) | uniqHCD_268 | 0.12 | -0.16 | 0.12 | 0.00 | -0.13 | 1 | 0.27861 | 1.21303 | 2.26428 | 0.0201 | 0 | 0.15641 | 1.11451 | 1.22475 | 0.12807 | 0 | 0.02563 | 1.01793 | 0.97346 | 0.46114 | 0 | 0 |
| 1373686_at | Serpina6 | serpin peptidase inhibitor, clade A (alpha-1 antiprot | uniqHCD_268 | 0.16 | -0.07 | 0.19 | -0.10 | -0.02 | 1 | 0.26524 | 1.20184 | 2.09446 | 0.02943 | 0 | -0.0266 | -1.0186 | 0.96535 | 0.41303 | 0 | 0.05035 | 1.03551 | 1.25383 | 0.24452 | 0 | 0 |
| 1374025_at | Nmnat3 | nicotinamide nucleotide adenylyltransferase 3 | uniqHCD_268 | 0.16 | 0.19 | -0.13 | 0.15 | -0.01 | 1 | -0.3272 | -1.2546 | 3.19825 | 0.01707 | 0 | -0.0408 | -1.0287 | 0.96879 | 0.36896 | 0 | -0.1986 | -1.1476 | 1.60972 | 0.10352 | 0 | 0 |
| 1374206_at | Enkd1 | enkurin domain containing 1 | uniqHCD_268 | -0.04 | -0.20 | 0.07 | -0.18 | -0.03 | 1 | 0.27149 | 1.20705 | 2.0672 | 0.03849 | 0 | 0.01602 | 1.01117 | 0.97327 | 0.60507 | 0 | 0.16381 | 1.12024 | 1.44403 | 0.16818 | 0 | 0 |
| 1374218_at | LOC1009128 | uncharacterized LOC100912843 | uniqHCD_268 | -0.10 | -0.27 | 0.01 | -0.20 | -0.12 | 1 | 0.27562 | 1.21051 | 1.95799 | 0.04539 | 0 | 0.07087 | 1.05035 | 1.12426 | 0.23889 | 0 | 0.15273 | 1.11167 | 1.49085 | 0.15278 | 0 | 0 |
| 1374787_at | --- | --- | uniqHCD_268 | -0.01 | -0.17 | 0.16 | -0.15 | -0.10 | 1 | 0.32135 | 1.2495 | 2.33755 | 0.01135 | 0 | 0.01501 | 1.01046 | 1.04014 | 0.36784 | 0 | 0.06269 | 1.04441 | 1.41641 | 0.1779 | 0 | 0 |
| 1375395_at | Zfp324 | zinc finger protein 324 | uniqHCD_268 | -0.06 | -0.07 | 0.20 | -0.07 | -0.07 | 1 | 0.26722 | 1.20349 | 2.05709 | 0.03975 | 0 | -0.0021 | -1.0015 | 1.02835 | 0.39442 | 0 | -0.0046 | -1.0032 | 1.06398 | 0.36765 | 0 | 0 |
| 1375453_at | Gpatch8 | G patch domain containing 8 | uniqHCD_268 | 0.11 | 0.41 | 0.68 | 0.58 | 0.54 | 1 | 0.27613 | 1.21094 | 2.12778 | 0.0247 | 0 | 0.17217 | 1.12675 | 1.23493 | 0.12219 | 0 | 0.13703 | 1.09964 | 1.6034 | 0.1258 | 0 | 0 |
| 1375675_at | Mbnl2 | muscleblind-like splicing regulator 2 | uniqHCD_268 | -0.15 | -0.23 | 0.04 | -0.04 | 0.00 | 1 | 0.26931 | 1.20523 | 2.05539 | 0.03999 | 0 | 0.1934 | 1.14345 | 1.2964 | 0.0737 | 0 | 0.22375 | 1.16777 | 2.0838 | 0.05354 | 0 | 0 |
| 1376270_at | --- | --- | uniqHCD_268 | 0.13 | -0.12 | 0.16 | -0.11 | 0.02 | 1 | 0.27717 | 1.21181 | 2.00878 | 0.0414 | 0 | 0.00612 | 1.00425 | 0.98762 | 0.51256 | 0 | 0.13097 | 1.09503 | 1.72782 | 0.09745 | 0 | 0 |
| 1376384_at | Dusp19 | dual specificity phosphatase 19 | uniqHCD_268 | 0.08 | 0.06 | -0.21 | -0.04 | -0.10 | 1 | -0.2706 | -1.2063 | 3.3179 | 0.01323 | 0 | -0.1045 | -1.0751 | 1.13791 | 0.12148 | 0 | -0.1665 | -1.1224 | 2.00559 | 0.05133 | 0 | 0 |
| 1376438_at | --- | --- | uniqHCD_268 | -0.21 | -0.22 | 0.08 | -0.08 | 0.03 | 1 | 0.29355 | 1.22565 | 2.25565 | 0.02118 | 0 | 0.13208 | 1.09587 | 1.20931 | 0.14283 | 0 | 0.24605 | 1.18596 | 2.26424 | 0.04332 | 0 | 0 |
| 1376783_at | --- | --- | uniqHCD_268 | 0.02 | -0.12 | 0.25 | -0.08 | 0.03 | 1 | 0.36401 | 1.287 | 2.3594 | 0.00888 | 0 | 0.03216 | 1.02254 | 1.00069 | 0.47455 | 0 | 0.14698 | 1.10725 | 1.45575 | 0.16524 | 0 | 0 |
| 1376789_at | --- | --- | uniqHCD_268 | -0.05 | 0.07 | -0.24 | -0.18 | 0.03 | 1 | -0.3138 | -1.243 | 3.61588 | 0.0093 | 0 | -0.2536 | -1.1921 | 1.70229 | 0.02753 | 0 | -0.0423 | -1.0298 | 1.28032 | 0.20395 | 0 | 0 |
| 1376970_at | Ybey | ybeY metallopeptidase | uniqHCD_268 | 0.11 | -0.07 | -0.35 | -0.33 | -0.28 | 1 | -0.2873 | -1.2204 | 3.37556 | 0.0127 | 0 | -0.262 | -1.1992 | 2.04049 | 0.0147 | 0 | -0.2124 | -1.1586 | 2.15682 | 0.04099 | 0 | 0 |
| 1377046_at | Ankrd6 | ankyrin repeat domain 6 | uniqHCD_268 | 0.21 | -0.05 | 0.33 | 0.09 | 0.06 | 1 | 0.37483 | 1.29668 | 2.32333 | 0.01165 | 0 | 0.13661 | 1.09932 | 1.21234 | 0.14642 | 0 | 0.10603 | 1.07626 | 1.26288 | 0.23882 | 0 | 0 |
| 1377133_at | --- | --- | uniqHCD_268 | -0.18 | -0.06 | -0.34 | -0.05 | -0.16 | 1 | -0.2755 | -1.2104 | 3.08151 | 0.02487 | 0 | 0.01475 | 1.01028 | 1.07299 | 0.30241 | 0 | -0.0973 | -1.0698 | 1.62455 | 0.10035 | 0 | 0 |
| 1377598_at | Coq2 | coenzyme Q2 4-hydroxybenzoate polyprenyltransfe | uniqHCD_268 | -0.12 | 0.28 | -0.14 | 0.18 | 0.12 | 1 | -0.4126 | -1.3311 | 3.56207 | 0.00332 | 0 | -0.0948 | -1.0679 | 1.0023 | 0.26331 | 0 | -0.1527 | -1.1117 | 1.4932 | 0.12882 | 0 | 0 |
| 1377877_at | --- | --- | uniqHCD_268 | -0.14 | -0.30 | -0.02 | -0.22 | -0.15 | 1 | 0.27822 | 1.2127 | 2.07637 | 0.03752 | 0 | 0.08332 | 1.05946 | 1.25847 | 0.09542 | 0 | 0.15402 | 1.11267 | 1.77399 | 0.09247 | 0 | 0 |
| 1377914_at | Srrm1 | serine/arginine repetitive matrix 1 | uniqHCD_268 | 0.19 | 0.25 | 0.56 | 0.47 | 0.23 | 1 | 0.30059 | 1.23165 | 2.40906 | 0.00744 | 0 | 0.21271 | 1.15886 | 1.23238 | 0.03277 | 0 | -0.0253 | -1.0177 | 1.35472 | 0.17259 | 0 | 0 |
| 1377921_at | Etnk2 | ethanolamine kinase 2 | uniqHCD_268 | 0.04 | -0.13 | 0.14 | -0.09 | 0.09 | 1 | 0.27456 | 1.20963 | 2.20399 | 0.02218 | 0 | 0.04027 | 1.02831 | 1.01614 | 0.4296 | 0 | 0.22068 | 1.16528 | 1.90574 | 0.07127 | 0 | 0 |
| 1378114_at | --- | --- | uniqHCD_268 | -0.12 | -0.06 | 0.23 | 0.10 | 0.13 | 1 | 0.285 | 1.21841 | 2.08099 | 0.02697 | 0 | 0.16032 | 1.11753 | 1.16796 | 0.19736 | 0 | 0.18811 | 1.13927 | 1.6651 | 0.11164 | 0 | 0 |
| 1378233_at | --- | --- | uniqHCD_268 | 0.10 | -0.11 | 0.22 | 0.12 | 0.05 | 1 | 0.32716 | 1.25454 | 2.36647 | 0.01012 | 0 | 0.22182 | 1.1662 | 1.28653 | 0.07546 | 0 | 0.1596 | 1.11697 | 1.51285 | 0.14725 | 0 | 0 |
| 1378313_at | --- | --- | uniqHCD_268 | 0.02 | 0.00 | 0.27 | 0.19 | 0.08 | 1 | 0.26442 | 1.20116 | 2.09638 | 0.02791 | 0 | 0.18575 | 1.13741 | 1.28592 | 0.07732 | 0 | 0.07796 | 1.05553 | 1.06197 | 0.36904 | 0 | 0 |

| Affymetrix Probeset ID | Gene Symbol | Gene Title | Figure 6 B Venn n =2021 | Mean BN log2 intensity autologous n=8 | Mean log2 intensity day 30 DR+/+ ND n=4 | Mean log2 intensity day 30 DR+/+ HCD n=4 | Mean log2 intensity day 30 DR+/+ B/S n=4 | Mean log2 intensity day 30 F+/+ ND n=4 | DR+/+ HCD vs DR+/+ ND n=380 1=yes 0=no | Log2 ratio DR+/+ HCD vs DR+/+ ND | Fold-Change DR+/+ HCD vs DR+/+ ND | RP-FDR 4DR30 HCD vs 4DR30 Reg | p value DR+/+ HCD vs DR+/+ ND | DR+/+ B/S vs DR+/+ ND n=1,330 1=yes 0=no | Log2 ratio DR+/+ B/S vs DR+/+ ND | Fold-Change DR+/+ B/S vs DR+/+ ND | RP-FDR 4DR30 Antib vs 4DR30 Reg | p value DR+/+ B/S vs DR+/+ ND | DR+/+ ND vs F+/+ ND n=398 1=yes 0=no | Log2 ratio DR+/+ ND vs F+/+ ND | Fold-Change DR+/+ ND vs F+/+ ND | RP-FDR 4RT1uu 30Reg vs 4DR30 Reg | p value DR+/+ ND vs F+/+ ND | DR+/+ Longitudinal n=228 1=yes 0=no | Figure 6F |
|---|---|---|---|---|---|---|---|---|---|---|---|---|---|---|---|---|---|---|---|---|---|---|---|---|---|
| 1378512_at | --- | --- | uniqHCD_268 | -0.06 | -0.32 | -0.04 | -0.11 | -0.19 | 1 | 0.27376 | 1.20896 | 2.06931 | 0.03859 | 0 | 0.20158 | 1.14996 | 1.23369 | 0.12242 | 0 | 0.12506 | 1.09056 | 1.20508 | 0.26826 | 0 | 0 |
| 1378520_at | Bcl11b | B-cell CLL/lymphoma 11B (zinc finger protein) | uniqHCD_268 | 0.02 | 0.70 | 0.39 | 0.44 | 0.64 | 1 | -0.3125 | -1.2419 | 3.24556 | 0.00282 | 0 | -0.2617 | -1.1989 | 4.21292 | 0.00325 | 0 | -0.0655 | -1.0464 | 2.7058 | 0.02401 | 0 | 0 |
| 1378537_at | --- | --- | uniqHCD_268 | 0.12 | 0.16 | 0.45 | 0.16 | 0.13 | 1 | 0.28624 | 1.21946 | 1.99719 | 0.04271 | 0 | -0.0037 | -1.0026 | 0.96342 | 0.47005 | 0 | -0.0285 | -1.0199 | 0.93215 | 0.54597 | 0 | 0 |
| 1378612_at | --- | --- | uniqHCD_268 | 0.05 | -0.20 | 0.11 | -0.11 | -0.24 | 1 | 0.3114 | 1.24091 | 2.14335 | 0.02529 | 0 | 0.09646 | 1.06915 | 1.0777 | 0.29553 | 0 | -0.038 | -1.0267 | 0.9914 | 0.44643 | 0 | 0 |
| 1378690_at | Ly6al | lymphocyte antigen 6 complex, locus A-like | uniqHCD_268 | 0.21 | 0.23 | -0.04 | 0.05 | 0.00 | 1 | -0.2669 | -1.2033 | 2.72632 | 0.02206 | 0 | -0.1746 | -1.1286 | 1.20914 | 0.08682 | 0 | -0.2296 | -1.1725 | 2.47444 | 0.02904 | 0 | 0 |
| 1378819_at | Aspdh | aspartate dehydrogenase domain containing | uniqHCD_268 | 0.03 | -0.17 | 0.15 | -0.12 | 0.00 | 1 | 0.31991 | 1.24825 | 2.30663 | 0.01476 | 0 | 0.04411 | 1.03105 | 1.12805 | 0.23215 | 0 | 0.1621 | 1.11891 | 1.96212 | 0.06808 | 0 | 0 |
| 1378847_x_at | H2afx | H2A histone family, member X | uniqHCD_268 | 0.00 | 0.19 | -0.11 | 0.21 | 0.01 | 1 | -0.3052 | -1.2356 | 3.36605 | 0.01234 | 0 | 0.02048 | 1.0143 | 1.07177 | 0.3061 | 0 | -0.1822 | -1.1346 | 1.65375 | 0.09721 | 0 | 0 |
| 1378978_a_at | Prr12 | proline rich 12 | uniqHCD_268 | 0.33 | 0.95 | 0.63 | 0.97 | 0.92 | 1 | -0.3165 | -1.2453 | 2.72632 | 0.00167 | 0 | 0.0295 | 1.02066 | 1.61387 | 0.03269 | 0 | -0.0263 | -1.0184 | 2.69029 | 0.02405 | 0 | 0 |
| 1379088_x_at | --- | --- | uniqHCD_268 | -0.18 | 0.30 | -0.02 | 0.23 | 0.23 | 1 | -0.3148 | -1.2438 | 3.21093 | 0.02106 | 0 | -0.0623 | -1.0441 | 1.13666 | 0.12281 | 0 | -0.0663 | -1.047 | 1.19045 | 0.25287 | 0 | 0 |
| 1379139_x_at | --- | --- | uniqHCD_268 | -0.02 | -0.22 | 0.09 | -0.11 | -0.25 | 1 | 0.31054 | 1.24017 | 2.19718 | 0.01604 | 0 | 0.10454 | 1.07515 | 1.26201 | 0.10088 | 0 | -0.0363 | -1.0255 | 1.0761 | 0.33377 | 0 | 0 |
| 1379180_at | --- | --- | uniqHCD_268 | 0.03 | -0.11 | 0.23 | -0.04 | -0.16 | 1 | 0.3371 | 1.26322 | 2.31471 | 0.01042 | 0 | 0.06866 | 1.04874 | 1.1459 | 0.21839 | 0 | -0.0473 | -1.0333 | 1.26709 | 0.21121 | 0 | 0 |
| 1379226_at | Aim1l | absent in melanoma 1-like | uniqHCD_268 | 0.15 | 0.05 | 0.35 | 0.14 | 0.15 | 1 | 0.29551 | 1.22732 | 2.13612 | 0.02445 | 0 | 0.08533 | 1.06093 | 1.03261 | 0.3843 | 0 | 0.09641 | 1.06911 | 1.21979 | 0.26209 | 0 | 0 |
| 1379272_at | Paip1 | poly(A) binding protein interacting protein 1 | uniqHCD_268 | -0.02 | 0.25 | -0.09 | 0.11 | 0.05 | 1 | -0.34 | -1.2657 | 3.42011 | 0.0099 | 0 | -0.1341 | -1.0974 | 1.04194 | 0.19734 | 0 | -0.1976 | -1.1468 | 1.79456 | 0.07836 | 0 | 0 |
| 1379372_at | --- | --- | uniqHCD_268 | 0.05 | 0.20 | -0.08 | 0.24 | 0.11 | 1 | -0.2752 | -1.2102 | 3.24005 | 0.01938 | 0 | 0.04394 | 1.03093 | 1.0704 | 0.30746 | 0 | -0.0917 | -1.0656 | 1.27173 | 0.20929 | 0 | 0 |
| 1379522_at | --- | --- | uniqHCD_268 | 0.03 | -0.03 | 0.27 | -0.05 | 0.03 | 1 | 0.29746 | 1.22898 | 2.04639 | 0.04007 | 0 | -0.0218 | -1.0152 | 0.96278 | 0.48063 | 0 | 0.061 | 1.04319 | 1.25226 | 0.24563 | 0 | 0 |
| 1379609_at | --- | --- | uniqHCD_268 | 0.02 | -0.14 | 0.14 | -0.26 | -0.31 | 1 | 0.28653 | 1.21971 | 2.08771 | 0.03108 | 0 | -0.1191 | -1.086 | 1.01245 | 0.24749 | 0 | -0.1626 | -1.1193 | 1.84123 | 0.07341 | 0 | 0 |
| 1379856_at | Arid1b | AT rich interactive domain 1B (Swi1 like) | uniqHCD_268 | 0.07 | -0.10 | 0.27 | 0.13 | 0.13 | 1 | 0.36319 | 1.28627 | 2.50706 | 0.00548 | 0 | 0.2241 | 1.16805 | 1.29379 | 0.06769 | 0 | 0.2252 | 1.16894 | 2.28226 | 0.03501 | 0 | 0 |
| 1379880_at | --- | --- | uniqHCD_268 | 0.03 | -0.11 | 0.19 | -0.16 | -0.06 | 1 | 0.29336 | 1.22549 | 2.09641 | 0.03229 | 0 | -0.0551 | -1.039 | 0.96603 | 0.40326 | 0 | 0.05007 | 1.03531 | 0.93907 | 0.51207 | 0 | 0 |
| 1379922_at | --- | --- | uniqHCD_268 | -0.19 | 0.22 | -0.05 | 0.10 | 0.14 | 1 | -0.2681 | -1.2042 | 2.60936 | 0.04439 | 0 | -0.1226 | -1.0887 | 1.00403 | 0.2606 | 0 | -0.0813 | -1.058 | 1.07396 | 0.33646 | 0 | 0 |
| 1379956_at | --- | --- | uniqHCD_268 | 0.01 | 0.03 | 0.34 | 0.16 | 0.14 | 1 | 0.31581 | 1.24471 | 2.1347 | 0.02553 | 0 | 0.13539 | 1.09839 | 1.16238 | 0.20441 | 0 | 0.11199 | 1.08072 | 1.31115 | 0.21658 | 0 | 0 |
| 1380052_at | --- | --- | uniqHCD_268 | -0.23 | 0.07 | -0.28 | -0.17 | -0.14 | 1 | -0.3543 | -1.2784 | 3.60265 | 0.00962 | 0 | -0.2432 | -1.1836 | 1.19785 | 0.0919 | 0 | -0.2192 | -1.1641 | 1.62044 | 0.10093 | 0 | 0 |
| 1380105_at | --- | --- | uniqHCD_268 | 0.12 | -0.06 | 0.24 | 0.01 | 0.08 | 1 | 0.29837 | 1.22975 | 2.13726 | 0.02536 | 0 | 0.06901 | 1.049 | 1.0796 | 0.29237 | 0 | 0.13456 | 1.09775 | 1.41782 | 0.17657 | 0 | 0 |
| 1380263_at | Fam193a | family with sequence similarity 193, member A | uniqHCD_268 | 0.05 | 0.64 | 0.38 | 0.57 | 0.52 | 1 | -0.2677 | -1.2039 | 2.82941 | 0.00155 | 0 | -0.0746 | -1.0531 | 2.49156 | 0.00769 | 0 | -0.1282 | -1.093 | 3.52389 | 0.00408 | 0 | 0 |
| 1380268_at | --- | --- | uniqHCD_268 | 0.07 | -0.01 | 0.28 | 0.07 | 0.07 | 1 | 0.29015 | 1.22277 | 2.08718 | 0.02852 | 0 | 0.07667 | 1.05458 | 1.09334 | 0.27788 | 0 | 0.08166 | 1.05823 | 1.18655 | 0.27837 | 0 | 0 |
| 1380316_at | Mcat | malonyl CoA:ACP acyltransferase (mitochondrial) | uniqHCD_268 | 0.16 | -0.19 | 0.19 | -0.03 | 0.04 | 1 | 0.37715 | 1.29878 | 2.41593 | 0.00707 | 0 | 0.1614 | 1.11837 | 1.29054 | 0.07084 | 0 | 0.22898 | 1.17201 | 2.35537 | 0.02174 | 0 | 0 |
| 1380433_at | --- | --- | uniqHCD_268 | 0.06 | -0.13 | 0.14 | -0.01 | 0.03 | 1 | 0.26376 | 1.20061 | 2.05834 | 0.03951 | 0 | 0.11529 | 1.08319 | 1.1792 | 0.18242 | 0 | 0.15929 | 1.11674 | 1.62895 | 0.12094 | 0 | 0 |
| 1380438_at | --- | --- | uniqHCD_268 | 0.02 | -0.10 | 0.17 | -0.05 | -0.03 | 1 | 0.26357 | 1.20044 | 2.29142 | 0.0182 | 0 | 0.0435 | 1.03061 | 1.1871 | 0.17326 | 0 | 0.06451 | 1.04573 | 1.5866 | 0.12989 | 0 | 0 |
| 1380492_at | Ncs1 | neuronal calcium sensor 1 | uniqHCD_268 | -0.07 | -0.23 | 0.05 | -0.16 | -0.22 | 1 | 0.27923 | 1.21355 | 2.0898 | 0.02681 | 0 | 0.06961 | 1.04943 | 1.1657 | 0.1984 | 0 | 0.01287 | 1.00896 | 1.07425 | 0.356 | 0 | 0 |
| 1380522_at | --- | --- | uniqHCD_268 | -0.06 | 0.11 | -0.16 | -0.11 | 0.01 | 1 | -0.2719 | -1.2074 | 2.6108 | 0.04508 | 0 | -0.216 | -1.1615 | 1.21252 | 0.08613 | 0 | -0.0976 | -1.07 | 1.23912 | 0.224 | 0 | 0 |
| 1380634_at | --- | --- | uniqHCD_268 | 0.04 | -0.10 | -0.37 | -0.16 | -0.31 | 1 | -0.2655 | -1.202 | 3.13877 | 0.02301 | 0 | -0.0628 | -1.0445 | 0.99448 | 0.28448 | 0 | -0.2128 | -1.159 | 2.14544 | 0.04208 | 0 | 0 |
| 1380708_at | --- | --- | uniqHCD_268 | 0.25 | 0.16 | 0.48 | 0.15 | 0.31 | 1 | 0.32298 | 1.25092 | 2.36534 | 0.00897 | 0 | -0.0134 | -1.0093 | 0.96809 | 0.37486 | 0 | 0.1529 | 1.1118 | 1.66824 | 0.10841 | 0 | 0 |
| 1380728_at | --- | --- | uniqHCD_268 | -0.14 | 0.02 | -0.30 | -0.01 | -0.05 | 1 | -0.322 | -1.2501 | 3.28461 | 0.01352 | 0 | -0.0289 | -1.0202 | 1.08958 | 0.14571 | 0 | -0.0746 | -1.053 | 1.50257 | 0.12427 | 0 | 0 |
| 1380764_at | Rrp1b | ribosomal RNA processing 1 homolog B (S. cerevisia | uniqHCD_268 | 0.08 | 0.24 | -0.09 | 0.08 | 0.15 | 1 | -0.3268 | -1.2543 | 3.24308 | 0.01523 | 0 | -0.1591 | -1.1166 | 1.07107 | 0.16039 | 0 | -0.0905 | -1.0647 | 1.13377 | 0.28797 | 0 | 0 |
| 1381015_at | --- | --- | uniqHCD_268 | 0.07 | 0.12 | -0.17 | 0.03 | 0.14 | 1 | -0.2892 | -1.222 | 3.16086 | 0.02256 | 0 | -0.0863 | -1.0616 | 0.96689 | 0.39215 | 0 | 0.0247 | 1.01726 | 1.1869 | 0.27781 | 0 | 0 |
| 1381071_at | --- | --- | uniqHCD_268 | -0.18 | -0.10 | -0.38 | -0.15 | -0.29 | 1 | -0.2713 | -1.2069 | 2.68498 | 0.03868 | 0 | -0.0431 | -1.0304 | 1.00096 | 0.26567 | 0 | -0.186 | -1.1376 | 1.79759 | 0.0763 | 0 | 0 |
| 1381100_at | --- | --- | uniqHCD_268 | 0.00 | -0.26 | 0.01 | -0.23 | -0.32 | 1 | 0.26961 | 1.20548 | 2.08388 | 0.03123 | 0 | 0.03274 | 1.02295 | 1.01802 | 0.42719 | 0 | -0.0596 | -1.0422 | 1.10144 | 0.31539 | 0 | 0 |
| 1381105_at | --- | --- | uniqHCD_268 | -0.05 | -0.06 | 0.21 | 0.06 | -0.03 | 1 | 0.27861 | 1.21303 | 2.18398 | 0.0224 | 0 | 0.12648 | 1.09163 | 1.20798 | 0.1485 | 0 | 0.03789 | 1.02661 | 0.99999 | 0.42876 | 0 | 0 |
| 1381144_at | Impad1 /// L( | inositol monophosphatase domain containing 1 /// | uniqHCD_268 | -0.19 | -0.03 | 0.28 | 0.17 | -0.04 | 1 | 0.30956 | 1.23933 | 2.30852 | 0.01804 | 0 | 0.19852 | 1.14752 | 1.29817 | 0.06742 | 0 | -0.0076 | -1.0053 | 0.96695 | 0.48598 | 0 | 0 |
| 1381240_at | --- | --- | uniqHCD_268 | 0.05 | 0.05 | -0.27 | -0.08 | -0.11 | 1 | -0.3234 | -1.2513 | 3.59484 | 0.00717 | 0 | -0.1323 | -1.096 | 1.1773 | 0.10411 | 0 | -0.1608 | -1.1179 | 1.79254 | 0.07885 | 0 | 0 |
| 1381321_at | --- | --- | uniqHCD_268 | -0.21 | 0.30 | 0.01 | 0.07 | 0.34 | 1 | -0.2872 | -1.2203 | 3.36042 | 0.01027 | 0 | -0.231 | -1.1737 | 1.59955 | 0.03209 | 0 | 0.03795 | 1.02665 | 1.28427 | 0.22948 | 0 | 0 |
| 1381390_at | Cnst | consortin, connexin sorting protein | uniqHCD_268 | 0.11 | -0.10 | 0.18 | 0.00 | -0.03 | 1 | 0.28232 | 1.21615 | 2.11747 | 0.02581 | 0 | 0.10798 | 1.07772 | 1.08506 | 0.28896 | 0 | 0.07489 | 1.05328 | 1.06629 | 0.36574 | 0 | 0 |
| 1381726_at | --- | --- | uniqHCD_268 | 0.13 | 0.14 | 0.49 | 0.04 | 0.15 | 1 | 0.34974 | 1.27433 | 2.32279 | 0.00829 | 0 | -0.0921 | -1.0659 | 1.01479 | 0.24454 | 0 | 0.01269 | 1.00883 | 0.98219 | 0.45056 | 0 | 0 |
| 1381730_at | --- | --- | uniqHCD_268 | -0.02 | -0.24 | 0.05 | -0.47 | -0.20 | 1 | 0.29466 | 1.22659 | 2.33116 | 0.01102 | 0 | -0.2252 | -1.169 | 1.66989 | 0.02862 | 0 | 0.04334 | 1.0305 | 1.47372 | 0.15818 | 0 | 0 |
| 1381829_at | Zfp318 | zinc finger protein 318 | uniqHCD_268 | 0.10 | 0.13 | 0.43 | 0.39 | 0.28 | 1 | 0.30399 | 1.23456 | 2.42847 | 0.00461 | 0 | 0.26027 | 1.1977 | 1.00987 | 0.01507 | 0 | 0.15001 | 1.10958 | 2.30697 | 0.0181 | 0 | 0 |
| 1381841_at | --- | --- | uniqHCD_268 | -0.11 | 0.04 | -0.22 | -0.06 | -0.14 | 1 | -0.2632 | -1.2002 | 2.5541 | 0.04722 | 0 | -0.0988 | -1.0709 | 0.96856 | 0.36806 | 0 | -0.1789 | -1.132 | 1.47459 | 0.13561 | 0 | 0 |
| 1381873_at | --- | --- | uniqHCD_268 | -0.04 | -0.19 | 0.08 | -0.11 | -0.19 | 1 | 0.27616 | 1.21097 | 2.20046 | 0.02144 | 0 | 0.08567 | 1.06118 | 1.25242 | 0.10902 | 0 | 0.00015 | 1.0001 | 1.06603 | 0.36609 | 0 | 0 |
| 1382087_at | --- | --- | uniqHCD_268 | 0.13 | -0.32 | -0.03 | -0.21 | -0.14 | 1 | 0.2854 | 1.21875 | 2.58579 | 0.00632 | 0 | 0.10709 | 1.07705 | 1.23448 | 0.12171 | 0 | 0.17698 | 1.13051 | 2.2136 | 0.04584 | 0 | 0 |
| 1382229_at | --- | --- | uniqHCD_268 | -0.02 | -0.02 | -0.32 | -0.04 | -0.01 | 1 | -0.2991 | -1.2303 | 3.04707 | 0.02538 | 0 | -0.0206 | -1.0144 | 0.96138 | 0.55326 | 0 | 0.01181 | 1.00822 | 0.89748 | 0.63429 | 0 | 0 |
| 1382273_at | Bcl2l14 | Bcl2-like 14 (apoptosis facilitator) | uniqHCD_268 | -0.01 | 0.06 | -0.25 | -0.16 | -0.01 | 1 | -0.3071 | -1.2372 | 3.59595 | 0.00971 | 0 | -0.2196 | -1.1644 | 1.32188 | 0.06095 | 0 | -0.0702 | -1.0499 | 1.20319 | 0.24343 | 0 | 0 |
| 1382499_at | --- | --- | uniqHCD_268 | 0.05 | -0.13 | 0.14 | -0.29 | -0.29 | 1 | 0.26776 | 1.20394 | 2.05748 | 0.03989 | 0 | -0.1582 | -1.1159 | 1.07717 | 0.15687 | 0 | -0.1582 | -1.1159 | 1.51997 | 0.1197 | 0 | 0 |
| 1382504_at | Chd7 | chromodomain helicase DNA binding protein 7 | uniqHCD_268 | 0.01 | 0.19 | -0.08 | -0.04 | 0.09 | 1 | -0.2705 | -1.2062 | 2.85473 | 0.03608 | 0 | -0.2376 | -1.179 | 1.21759 | 0.08426 | 0 | -0.0997 | -1.0716 | 1.18392 | 0.25689 | 0 | 0 |
| 1382534_at | --- | --- | uniqHCD_268 | 0.18 | -0.17 | 0.16 | -0.17 | 0.07 | 1 | 0.33479 | 1.2612 | 2.27017 | 0.01292 | 0 | 0.00272 | 1.00189 | 0.98887 | 0.50911 | 0 | 0.23503 | 1.17693 | 2.30963 | 0.0401 | 0 | 0 |
| 1382641_at | --- | --- | uniqHCD_268 | -0.04 | -0.05 | 0.24 | -0.14 | -0.12 | 1 | 0.29494 | 1.22684 | 2.30111 | 0.01805 | 0 | -0.0868 | -1.062 | 0.98883 | 0.29459 | 0 | -0.07 | -1.0497 | 1.04717 | 0.36767 | 0 | 0 |
| 1382691_at | Sf3b1 | splicing factor 3b, subunit 1 | uniqHCD_268 | -0.20 | -0.43 | -0.14 | -0.25 | -0.38 | 1 | 0.28532 | 1.21868 | 3.11308 | 0.0026 | 0 | 0.1848 | 1.13666 | 1.28279 | 0.07817 | 0 | 0.05096 | 1.03596 | 1.4762 | 0.15693 | 0 | 0 |
| 1382718_at | Nfx1 | nuclear transcription factor, X-box binding 1 | uniqHCD_268 | -0.01 | -0.25 | 0.02 | -0.12 | -0.27 | 1 | 0.27415 | 1.20929 | 1.97482 | 0.04756 | 0 | 0.12709 | 1.09209 | 1.10888 | 0.26115 | 0 | -0.0226 | -1.0158 | 0.94586 | 0.51829 | 0 | 0 |
| 1382794_at | --- | --- | uniqHCD_268 | 0.17 | 0.30 | 0.58 | 0.30 | 0.47 | 1 | 0.27214 | 1.2076 | 2.37623 | 0.00932 | 0 | 0.00106 | 1.00074 | 1.06425 | 0.16676 | 0 | 0.16568 | 1.12169 | 2.39029 | 0.02606 | 0 | 0 |
| 1382819_at | Tyrp1 | tyrosinase-related protein 1 | uniqHCD_268 | -0.12 | -0.02 | -0.34 | -0.23 | -0.20 | 1 | -0.3176 | -1.2462 | 3.58171 | 0.00472 | 0 | -0.2069 | -1.1542 | 1.70214 | 0.02737 | 0 | -0.1829 | -1.1351 | 2.26443 | 0.03502 | 0 | 0 |
| 1382895_at | --- | --- | uniqHCD_268 | -0.03 | -0.21 | 0.11 | -0.20 | -0.10 | 1 | 0.32644 | 1.25391 | 2.19435 | 0.02223 | 0 | 0.01591 | 1.01109 | 1.0225 | 0.40914 | 0 | 0.11826 | 1.08542 | 1.43581 | 0.17346 | 0 | 0 |
| 1383163_at | Igj | immunoglobulin joining chain | uniqHCD_268 | 0.01 | 0.27 | -0.22 | 0.03 | 0.22 | 1 | -0.497 | -1.4113 | 1.436 | 0.00023 | 0 | -0.2422 | -1.1828 | 3.635 | 0.00421 | 0 | -0.0489 | -1.0345 | 1.40369 | 0.15811 | 0 | 0 |
| 1383268_at | --- | --- | uniqHCD_268 | -0.03 | -0.15 | 0.12 | 0.01 | -0.07 | 1 | 0.2702 | 1.20598 | 2.11916 | 0.02603 | 0 | 0.15942 | 1.11684 | 1.21902 | 0.13351 | 0 | 0.07137 | 1.05071 | 1.16586 | 0.29046 | 0 | 0 |
| 1383411_at | Gpr115 | G protein-coupled receptor 115 | uniqHCD_268 | -0.07 | 0.05 | -0.23 | -0.18 | -0.03 | 1 | -0.2875 | -1.2205 | 3.14804 | 0.02278 | 0 | -0.2286 | -1.1717 | 1.22484 | 0.08279 | 0 | -0.0817 | -1.0583 | 1.12834 | 0.29222 | 0 | 0 |
| 1383435_at | Scn3b | sodium channel, voltage-gated, type III, beta | uniqHCD_268 | 0.08 | 0.18 | -0.10 | 0.00 | 0.00 | 1 | -0.273 | -1.2083 | 3.20625 | 0.01897 | 0 | -0.1802 | -1.133 | 1.2232 | 0.08338 | 0 | -0.1808 | -1.1335 | 1.84294 | 0.06904 | 0 | 0 |
| 1383536_at | Dhfr | dihydrofolate reductase | uniqHCD_268 | -0.30 | -0.03 | -0.40 | -0.03 | -0.12 | 1 | -0.3722 | -1.2943 | 3.57347 | 0.00563 | 0 | -0.0014 | -1.0009 | 0.97431 | 0.33481 | 0 | -0.0944 | -1.0676 | 1.52325 | 0.11902 | 0 | 0 |
| 1383590_at | Csgalnact1 | chondroitin sulfate N-acetylgalactosaminyltransfera | uniqHCD_268 | -0.07 | -0.40 | -0.71 | -0.30 | -0.45 | 1 | -0.3074 | -1.2375 | 3.7644 | 0.00908 | 0 | 0.09896 | 1.071 | 1.27248 | 0.08482 | 0 | -0.0518 | -1.0366 | 1.22044 | 0.23444 | 0 | 0 |
| 1383599_at | --- | --- | uniqHCD_268 | -0.08 | 0.06 | -0.24 | 0.09 | -0.13 | 1 | -0.3011 | -1.2321 | 3.09803 | 0.02779 | 0 | 0.03127 | 1.02191 | 1.06237 | 0.3206 | 0 | -0.1831 | -1.1353 | 1.75132 | 0.08171 | 0 | 0 |
| 1383642_at | --- | --- | uniqHCD_268 | 0.03 | 0.06 | -0.30 | 0.10 | -0.17 | 1 | -0.362 | -1.2852 | 3.57875 | 0.0046 | 0 | 0.04039 | 1.02839 | 1.04025 | 0.36755 | 0 | -0.2269 | -1.1703 | 2.80108 | 0.02162 | 0 | 0 |
| 1383678_at | Oscp1 | organic solute carrier partner 1 | uniqHCD_268 | 0.07 | -0.01 | 0.27 | -0.14 | -0.03 | 1 | 0.28449 | 1.21798 | 2.13859 | 0.02338 | 0 | -0.123 | -1.089 | 1.00177 | 0.2625 | 0 | -0.0115 | -1.008 | 0.9578 | 0.49946 | 0 | 0 |
| 1383844_at | Arhgef15 | Rho guanine nucleotide exchange factor (GEF) 15 | uniqHCD_268 | 0.06 | -0.29 | 0.05 | -0.04 | -0.04 | 1 | 0.337 | 1.26312 | 2.2791 | 0.01217 | 0 | 0.25322 | 1.19187 | 1.27952 | 0.04777 | 0 | 0.25471 | 1.1931 | 2.38595 | 0.02747 | 0 | 0 |
| 1384297_at | --- | --- | uniqHCD_268 | 0.18 | -0.07 | 0.22 | -0.17 | 0.03 | 1 | 0.2935 | 1.22561 | 2.07064 | 0.03848 | 0 | -0.097 | -1.0695 | 0.96729 | 0.38904 | 0 | 0.10201 | 1.07327 | 1.0708 | 0.36033 | 0 | 0 |
| 1384480_at | Pcnxl4 | pecanex-like 4 (Drosophila) | uniqHCD_268 | -0.08 | -0.26 | 0.01 | -0.10 | -0.12 | 1 | 0.2688 | 1.20481 | 1.99928 | 0.04397 | 0 | 0.15793 | 1.11569 | 1.19951 | 0.15567 | 0 | 0.14011 | 1.10199 | 1.6794 | 0.10655 | 0 | 0 |
| 1384602_at | Gpr107 | G protein-coupled receptor 107 | uniqHCD_268 | -0.03 | -0.13 | 0.20 | -0.05 | -0.16 | 1 | 0.33059 | 1.25753 | 2.54676 | 0.00581 | 0 | 0.08405 | 1.05999 | 1.23526 | 0.12031 | 0 | -0.0273 | -1.0191 | 1.23255 | 0.22734 | 0 | 0 |

| Affymetrix Probeset ID | Gene Symbol | Gene Title | Figure 6 B Venn n =2021 | Mean BN log2 intensity autologous n=8 | Mean log2 intensity day 30 DR +/+ ND n=4 | Mean log2 intensity day 30 DR/+/+ HCD n=4 | Mean log2 intensity day 30 DR/+/+ B/S n=4 | Mean log2 intensity day 30 F+/+ ND n=4 | DR+/+ HCD vs DR+/+ ND n=380 1=yes 0=no | Log2 ratio DR+/+ HCD vs DR+/+ ND | Fold-Change DR+/+ HCD vs DR+/+ ND | RP-FDR 4DR30 HCD vs 4DR30 Reg | p value DR+/+ HCD vs DR+/+ ND | DR+/+ B/S vs DR+/+ ND n=1,330 1=yes 0=no | Log2 ratio DR+/+ B/S vs DR+/+ ND | Fold-Change DR+/+ B/S vs DR+/+ ND | RP-FDR 4DR30 Antib vs 4DR30 Reg | p value DR+/+ B/S vs DR+/+ ND | DR+/+ ND vs F+/+ ND n=398 1=yes 0=no | Log2 ratio DR+/+ ND vs F+/+ ND | Fold-Change DR+/+ ND vs F+/+ ND | RP-FDR 4RT1uu 30Reg vs 4DR30 Reg | p value DR+/+ ND vs F+/+ ND | DR+/+ Longitudinal n=228 1=yes 0=no | Figure 6F |
|---|---|---|---|---|---|---|---|---|---|---|---|---|---|---|---|---|---|---|---|---|---|---|---|---|---|
| 1384629_at | --- | --- | uniqHCD_268 | 0.02 | -0.15 | 0.19 | -0.18 | -0.16 | 1 | 0.33323 | 1.25983 | 2.24313 | 0.02121 | 0 | -0.0383 | -1.0269 | 0.96318 | 0.46426 | 0 | -0.0142 | -1.0099 | 0.91822 | 0.58381 | 0 | 0 |
| 1384649_at | --- | --- | uniqHCD_268 | 0.00 | 0.01 | -0.26 | 0.01 | -0.07 | 1 | -0.2757 | -1.2106 | 3.23848 | 0.02051 | 0 | -0.0028 | -1.002 | 0.98915 | 0.50891 | 0 | -0.0833 | -1.0594 | 1.22911 | 0.22923 | 0 | 0 |
| 1384815_at | --- | --- | uniqHCD_268 | 0.03 | 0.49 | 0.03 | 0.66 | 0.38 | 1 | -0.4573 | -1.373 | 3.684 | 0.00118 | 0 | 0.16416 | 1.12051 | 1.21768 | 0.03125 | 0 | -0.111 | -1.08 | 2.17483 | 0.04 | 0 | 0 |
| 1384902_at | --- | --- | uniqHCD_268 | -0.12 | -0.37 | -0.10 | -0.33 | -0.37 | 1 | 0.27189 | 1.20739 | 2.11122 | 0.0262 | 0 | 0.04181 | 1.0294 | 1.09456 | 0.27706 | 0 | 0.00515 | 1.00358 | 1.08531 | 0.34633 | 0 | 0 |
| 1384923_at | RGD1307461 | similar to RIKEN cDNA 6430571L13 gene; similar to | uniqHCD_268 | 0.16 | -0.04 | 0.27 | -0.10 | 0.03 | 1 | 0.30774 | 1.23777 | 2.29312 | 0.01512 | 0 | -0.0571 | -1.0403 | 0.9691 | 0.36737 | 0 | 0.07226 | 1.05136 | 1.18538 | 0.27901 | 0 | 0 |
| 1385069_at | Acvr1 | activin A receptor, type I | uniqHCD_268 | -0.07 | 0.10 | -0.21 | -0.01 | -0.16 | 1 | -0.3117 | -1.2412 | 3.67942 | 0.00615 | 0 | -0.1022 | -1.0734 | 1.02227 | 0.23184 | 0 | -0.2547 | -1.1931 | 2.79556 | 0.02184 | 0 | 0 |
| 1385155_at | Lrrfip1 | leucine rich repeat (in FLII) interacting protein 1 | uniqHCD_268 | 0.13 | 0.04 | 0.31 | 0.09 | 0.10 | 1 | 0.26769 | 1.20388 | 1.97106 | 0.04494 | 0 | 0.05061 | 1.0357 | 0.99346 | 0.49681 | 0 | 0.06143 | 1.0435 | 1.10304 | 0.33234 | 0 | 0 |
| 1385160_at | Stab2 | stabilin 2 | uniqHCD_268 | 0.04 | 0.14 | -0.15 | -0.12 | 0.09 | 1 | -0.2813 | -1.2153 | 3.21323 | 0.02077 | 0 | -0.2562 | -1.1944 | 1.41127 | 0.05128 | 0 | -0.0454 | -1.0319 | 1.0702 | 0.33896 | 0 | 0 |
| 1385417_at | Zfr2 | zinc finger RNA binding protein 2 | uniqHCD_268 | 0.10 | -0.20 | 0.11 | -0.18 | 0.00 | 1 | 0.30464 | 1.23511 | 2.20237 | 0.01615 | 0 | 0.01635 | 1.0114 | 1.18694 | 0.17309 | 0 | 0.19792 | 1.14705 | 2.27681 | 0.03646 | 0 | 0 |
| 1385672_at | --- | --- | uniqHCD_268 | 0.03 | 0.25 | -0.06 | 0.17 | 0.07 | 1 | -0.3032 | -1.2339 | 3.07692 | 0.02859 | 0 | -0.0746 | -1.0531 | 0.97515 | 0.32915 | 0 | -0.1764 | -1.1301 | 1.48998 | 0.12912 | 0 | 0 |
| 1385754_s_at | Lrrc4 | leucine rich repeat containing 4 | uniqHCD_268 | 0.03 | 0.00 | -0.28 | -0.12 | 0.00 | 1 | -0.2709 | -1.2066 | 2.62081 | 0.04357 | 0 | -0.1112 | -1.0801 | 0.98362 | 0.30572 | 0 | 0.00386 | 1.00268 | 0.92099 | 0.55545 | 0 | 0 |
| 1385764_at | Slc25a41 | solute carrier family 25, member 41 | uniqHCD_268 | 0.20 | 0.00 | 0.40 | -0.02 | 0.10 | 1 | 0.39295 | 1.31307 | 2.6655 | 0.00343 | 0 | -0.0217 | -1.0152 | 0.98917 | 0.29352 | 0 | 0.09786 | 1.07018 | 1.4979 | 0.1496 | 0 | 0 |
| 1385769_at | Odf3 | outer dense fiber of sperm tails 3 | uniqHCD_268 | -0.10 | 0.01 | -0.26 | -0.12 | -0.07 | 1 | -0.2681 | -1.2042 | 2.93068 | 0.03166 | 0 | -0.1222 | -1.0884 | 0.97925 | 0.31347 | 0 | -0.0726 | -1.0516 | 1.15075 | 0.27648 | 0 | 0 |
| 1385853_at | Baz1a | bromodomain adjacent to zinc finger domain, 1A | uniqHCD_268 | -0.06 | 0.32 | 0.03 | 0.38 | 0.35 | 1 | -0.2877 | -1.2207 | 3.04375 | 0.02662 | 0 | 0.05443 | 1.03845 | 1.18082 | 0.18032 | 0 | 0.02665 | 1.01865 | 1.56166 | 0.13493 | 0 | 0 |
| 1386018_at | --- | --- | uniqHCD_268 | 0.02 | 0.14 | -0.17 | 0.01 | 0.06 | 1 | -0.3104 | -1.2401 | 3.01092 | 0.02953 | 0 | -0.1217 | -1.088 | 1.07664 | 0.15731 | 0 | -0.0797 | -1.0568 | 1.14063 | 0.28319 | 0 | 0 |
| 1386141_at | LOC10091063 | zinc finger CW-type PWWP domain protein 1-like // | uniqHCD_268 | 0.16 | -0.07 | 0.20 | -0.08 | -0.05 | 1 | 0.27483 | 1.20985 | 1.99561 | 0.04254 | 0 | -0.011 | -1.0076 | 0.98304 | 0.53588 | 0 | 0.01708 | 1.01191 | 0.99421 | 0.43587 | 0 | 0 |
| 1386479_at | --- | --- | uniqHCD_268 | -0.19 | 0.06 | -0.21 | -0.04 | -0.12 | 1 | -0.2693 | -1.2052 | 3.22859 | 0.01692 | 0 | -0.1062 | -1.0764 | 1.0007 | 0.26624 | 0 | -0.181 | -1.1336 | 1.66945 | 0.09636 | 0 | 0 |
| 1386520_at | Irx3 | iroquois homeobox 3 | uniqHCD_268 | 0.15 | -0.16 | 0.16 | -0.14 | -0.10 | 1 | 0.31235 | 1.24173 | 2.28863 | 0.01177 | 0 | 0.01389 | 1.00967 | 1.19736 | 0.15763 | 0 | 0.05174 | 1.03651 | 1.50593 | 0.1489 | 0 | 0 |
| 1386569_at | --- | --- | uniqHCD_268 | 0.01 | 0.12 | -0.18 | 0.04 | -0.01 | 1 | -0.2958 | -1.2275 | 3.09506 | 0.02478 | 0 | -0.0762 | -1.0543 | 0.9661 | 0.3794 | 0 | -0.128 | -1.0928 | 1.36275 | 0.1702 | 0 | 0 |
| 1386623_at | --- | --- | uniqHCD_268 | -0.14 | -0.30 | 0.05 | -0.33 | -0.17 | 1 | 0.34778 | 1.2726 | 2.71487 | 0.0034 | 0 | -0.0369 | -1.0259 | 1.04043 | 0.19929 | 0 | 0.12355 | 1.08941 | 1.6357 | 0.11966 | 0 | 0 |
| 1387146_a_at | Ednrb | endothelin receptor type B | uniqHCD_268 | -0.12 | -0.30 | -0.02 | -0.32 | -0.08 | 1 | 0.27402 | 1.20918 | 2.05708 | 0.03552 | 0 | -0.0213 | -1.0149 | 0.9633 | 0.53092 | 0 | 0.21498 | 1.16069 | 1.89511 | 0.07373 | 0 | 0 |
| 1387343_at | Cebpd | CCAAT/enhancer binding protein (C/EBP), delta | uniqHCD_268 | 0.04 | 0.31 | 0.03 | 0.06 | 0.16 | 1 | -0.2771 | -1.2118 | 3.05539 | 0.02535 | 0 | -0.2516 | -1.1905 | 1.29191 | 0.06838 | 0 | -0.1536 | -1.1124 | 1.75323 | 0.08158 | 0 | 0 |
| 1387349_at | Shox2 | short stature homeobox 2 | uniqHCD_268 | -0.08 | -0.12 | -0.39 | -0.17 | -0.36 | 1 | -0.2702 | -1.206 | 2.90372 | 0.0324 | 0 | -0.0542 | -1.0383 | 0.96666 | 0.40238 | 0 | -0.2394 | -1.1805 | 2.28192 | 0.03478 | 0 | 0 |
| 1387493_at | Akap5 | A kinase (PRKA) anchor protein 5 | uniqHCD_268 | -0.01 | 0.03 | -0.28 | -0.14 | -0.03 | 1 | -0.3082 | -1.2382 | 2.92693 | 0.03153 | 0 | -0.1735 | -1.1278 | 1.05488 | 0.17567 | 0 | -0.0614 | -1.0435 | 1.18838 | 0.25438 | 0 | 0 |
| 1387507_at | Il1rap | interleukin 1 receptor accessory protein | uniqHCD_268 | -0.12 | -0.21 | 0.09 | -0.10 | 0.03 | 1 | 0.29797 | 1.22941 | 2.27087 | 0.02008 | 0 | 0.11196 | 1.0807 | 1.23333 | 0.12187 | 0 | 0.2377 | 1.17911 | 2.26874 | 0.03597 | 0 | 0 |
| 1387631_at | Hpgd | hydroxyprostaglandin dehydrogenase 15 (NAD) | uniqHCD_268 | -0.13 | 0.04 | -0.23 | -0.19 | -0.13 | 1 | -0.2678 | -1.204 | 2.66835 | 0.0417 | 0 | -0.2282 | -1.1714 | 1.16058 | 0.10867 | 0 | -0.1682 | -1.1237 | 1.64976 | 0.0975 | 0 | 0 |
| 1387642_at | Il23a | Interleukin 23, alpha subunit p19 | uniqHCD_268 | 0.06 | -0.09 | -0.41 | -0.23 | -0.17 | 1 | -0.3202 | -1.2485 | 3.3674 | 0.01083 | 0 | -0.1392 | -1.1013 | 1.04235 | 0.19618 | 0 | -0.0806 | -1.0574 | 1.15225 | 0.27596 | 0 | 0 |
| 1387701_at | Hgf | hepatocyte growth factor | uniqHCD_268 | 0.00 | -0.12 | 0.16 | -0.09 | -0.06 | 1 | 0.2788 | 1.21318 | 2.098 | 0.03265 | 0 | 0.03246 | 1.02275 | 1.06789 | 0.31464 | 0 | 0.05589 | 1.0395 | 1.02067 | 0.41048 | 0 | 0 |
| 1387882_at | Klf9 | Kruppel-like factor 9 | uniqHCD_268 | 0.18 | -0.04 | 0.26 | -0.09 | 0.21 | 1 | 0.30576 | 1.23607 | 2.12208 | 0.02559 | 0 | -0.0476 | -1.0336 | 0.96802 | 0.37381 | 0 | 0.24903 | 1.18841 | 2.2366 | 0.04524 | 0 | 0 |
| 1387933_s_at | Podxl | podocalyxin-like | uniqHCD_268 | 0.16 | -0.15 | 0.19 | 0.06 | -0.03 | 1 | 0.34146 | 1.26704 | 2.32119 | 0.01067 | 0 | 0.21034 | 1.15696 | 1.26602 | 0.09436 | 0 | 0.11356 | 1.0819 | 1.14617 | 0.30181 | 0 | 0 |
| 1388239_at | Per3 | period circadian clock 3 | uniqHCD_268 | 0.05 | -0.09 | 0.24 | -0.04 | -0.02 | 1 | 0.33008 | 1.25708 | 2.23895 | 0.02124 | 0 | 0.05189 | 1.03662 | 1.01206 | 0.44155 | 0 | 0.0753 | 1.05358 | 1.01342 | 0.41734 | 0 | 0 |
| 1388276_at | LOC679994 | similar to histone 1, H2ai | uniqHCD_268 | -0.04 | -0.33 | -0.60 | -0.45 | -0.57 | 1 | -0.2782 | -1.2127 | 3.3071 | 0.01872 | 0 | -0.123 | -1.089 | 1.1853 | 0.09612 | 0 | -0.2469 | -1.1867 | 3.06455 | 0.00867 | 0 | 0 |
| 1388795_at | --- | --- | uniqHCD_268 | -0.04 | 0.07 | -0.25 | -0.11 | -0.01 | 1 | -0.325 | -1.2527 | 3.24696 | 0.01681 | 0 | -0.1833 | -1.1355 | 1.24687 | 0.07926 | 0 | -0.083 | -1.0592 | 1.09026 | 0.32337 | 0 | 0 |
| 1389586_at | --- | --- | uniqHCD_268 | 0.02 | 0.10 | -0.28 | 0.12 | -0.09 | 1 | -0.3825 | -1.3036 | 3.63586 | 0.00678 | 0 | 0.02377 | 1.01662 | 1.1002 | 0.26965 | 0 | -0.1862 | -1.1378 | 1.52794 | 0.11516 | 0 | 0 |
| 1389792_at | Solh | small optic lobes homolog (Drosophila) | uniqHCD_268 | 0.11 | 0.11 | 0.48 | 0.33 | 0.35 | 1 | 0.36996 | 1.29232 | 2.44038 | 0.00816 | 0 | 0.21339 | 1.15941 | 1.29261 | 0.07507 | 0 | 0.23593 | 1.17767 | 2.08226 | 0.05591 | 0 | 0 |
| 1389800_at | Pwwp2b | PWWP domain containing 2B | uniqHCD_268 | -0.24 | -0.13 | -0.40 | -0.25 | -0.28 | 1 | -0.2665 | -1.2029 | 3.3651 | 0.01039 | 0 | -0.1241 | -1.0898 | 1.09804 | 0.14229 | 0 | -0.1458 | -1.1064 | 1.52805 | 0.11615 | 0 | 0 |
| 1389818_at | --- | --- | uniqHCD_268 | 0.13 | 0.42 | 0.73 | 0.52 | 0.45 | 1 | 0.31023 | 1.2399 | 2.39217 | 0.00708 | 0 | 0.09885 | 1.07092 | 1.26913 | 0.0868 | 0 | 0.0276 | 1.01932 | 1.49934 | 0.15105 | 0 | 0 |
| 1389850_at | Arid1a | AT rich interactive domain 1A (SWI-like) | uniqHCD_268 | 0.18 | 0.21 | 0.54 | 0.42 | 0.42 | 1 | 0.33519 | 1.26155 | 2.33437 | 0.01013 | 0 | 0.21502 | 1.16072 | 1.27977 | 0.04774 | 0 | 0.21144 | 1.15784 | 2.27899 | 0.04228 | 0 | 0 |
| 1390043_at | LOC685179 | similar to SWI/SNF-related matrix-associated actin-( | uniqHCD_268 | -0.03 | -0.18 | 0.16 | -0.07 | -0.10 | 1 | 0.33958 | 1.26539 | 2.45843 | 0.00704 | 0 | 0.10682 | 1.07685 | 1.16699 | 0.19614 | 0 | 0.08229 | 1.0587 | 1.55341 | 0.13746 | 0 | 0 |
| 1390097_at | Tspyl4 | TSPY-like 4 | uniqHCD_268 | -0.05 | 0.14 | -0.32 | 0.02 | -0.04 | 1 | -0.468 | -1.3832 | 3.04381 | 0.00206 | 0 | -0.1294 | -1.0938 | 1.0813 | 0.15288 | 0 | -0.1819 | -1.1344 | 1.51696 | 0.12102 | 0 | 0 |
| 1390184_at | --- | --- | uniqHCD_268 | 0.02 | 0.05 | 0.32 | 0.23 | 0.16 | 1 | 0.27372 | 1.20892 | 2.25491 | 0.0211 | 0 | 0.18488 | 1.13673 | 1.28017 | 0.0792 | 0 | 0.11614 | 1.08383 | 1.63319 | 0.12037 | 0 | 0 |
| 1390329_at | --- | --- | uniqHCD_268 | -0.16 | -0.31 | -0.02 | -0.20 | -0.32 | 1 | 0.28942 | 1.22215 | 2.32032 | 0.01149 | 0 | 0.11498 | 1.08296 | 1.12195 | 0.242 | 0 | -0.0071 | -1.0049 | 0.96626 | 0.48653 | 0 | 0 |
| 1390425_at | --- | --- | uniqHCD_268 | -0.11 | -0.09 | -0.40 | -0.03 | -0.20 | 1 | -0.3107 | -1.2403 | 3.51046 | 0.00982 | 0 | 0.06206 | 1.04395 | 1.10415 | 0.26444 | 0 | -0.1112 | -1.0801 | 1.61396 | 0.10312 | 0 | 0 |
| 1390484_at | --- | --- | uniqHCD_268 | -0.42 | -0.51 | -0.25 | -0.57 | -0.50 | 1 | 0.26612 | 1.20257 | 2.28906 | 0.01259 | 0 | -0.0571 | -1.0404 | 1.3025 | 0.06345 | 0 | 0.01643 | 1.01145 | 1.48515 | 0.15444 | 0 | 0 |
| 1390624_at | Prrt4 | proline-rich transmembrane protein 4 | uniqHCD_268 | 0.14 | -0.17 | 0.13 | -0.23 | -0.01 | 1 | 0.29438 | 1.22636 | 2.25005 | 0.01382 | 0 | -0.0654 | -1.0464 | 0.97522 | 0.32895 | 0 | 0.15985 | 1.11717 | 2.3208 | 0.03463 | 0 | 0 |
| 1390685_at | --- | --- | uniqHCD_268 | 0.12 | 0.25 | -0.04 | 0.05 | 0.11 | 1 | -0.2944 | -1.2264 | 3.08577 | 0.02818 | 0 | -0.2036 | -1.1515 | 1.20701 | 0.0895 | 0 | -0.1471 | -1.1074 | 1.28379 | 0.20149 | 0 | 0 |
| 1390722_at | --- | --- | uniqHCD_268 | -0.05 | 0.25 | -0.10 | 0.22 | 0.27 | 1 | -0.3456 | -1.2707 | 3.66275 | 0.00813 | 0 | -0.0323 | -1.0226 | 1.06273 | 0.17055 | 0 | 0.01808 | 1.01261 | 1.29886 | 0.22228 | 0 | 0 |
| 1390734_at | --- | --- | uniqHCD_268 | 0.01 | 0.22 | -0.06 | 0.14 | 0.17 | 1 | -0.2752 | -1.2102 | 3.03059 | 0.02621 | 0 | -0.0767 | -1.0546 | 0.99687 | 0.27807 | 0 | -0.0436 | -1.0307 | 1.04931 | 0.36423 | 0 | 0 |
| 1390744_at | --- | --- | uniqHCD_268 | 0.04 | -0.18 | 0.11 | -0.08 | -0.03 | 1 | 0.28569 | 1.21899 | 2.07027 | 0.03468 | 0 | 0.09755 | 1.06995 | 1.11124 | 0.25749 | 0 | 0.14291 | 1.10413 | 1.67039 | 0.10973 | 0 | 0 |
| 1390753_at | --- | --- | uniqHCD_268 | 0.01 | 0.14 | 0.42 | 0.02 | 0.16 | 1 | 0.28133 | 1.21532 | 2.08807 | 0.03303 | 0 | -0.122 | -1.0882 | 1.21235 | 0.08491 | 0 | 0.02267 | 1.01584 | 1.08517 | 0.34625 | 0 | 0 |
| 1390757_at | Cdk11b /// R( | cyclin-dependent kinase 11B /// similar to cell divisi | uniqHCD_268 | -0.06 | 0.58 | 0.23 | 0.36 | 0.34 | 1 | -0.3449 | -1.27 | 1.42 | 0.00014 | 0 | -0.2181 | -1.1632 | 2.70667 | 0.00026 | 0 | -0.2348 | -1.1768 | 2.29667 | 0.00022 | 0 | 0 |
| 1391087_at | Lrrc56 | leucine rich repeat containing 56 | uniqHCD_268 | 0.07 | 0.06 | 0.33 | -0.15 | 0.09 | 1 | 0.26548 | 1.20203 | 2.09089 | 0.02958 | 0 | -0.2145 | -1.1603 | 1.362 | 0.05658 | 0 | 0.02431 | 1.01699 | 1.02312 | 0.40505 | 0 | 0 |
| 1391103_at | --- | --- | uniqHCD_268 | -0.09 | -0.07 | -0.35 | -0.28 | -0.13 | 1 | -0.2852 | -1.2186 | 3.42874 | 0.01136 | 0 | -0.2104 | -1.157 | 1.32122 | 0.06096 | 0 | -0.0681 | -1.0484 | 1.2566 | 0.21561 | 0 | 0 |
| 1391119_at | --- | --- | uniqHCD_268 | -0.05 | 0.04 | -0.37 | -0.21 | -0.19 | 1 | -0.4055 | -1.3245 | 3.55568 | 0.00503 | 0 | -0.2446 | -1.1847 | 1.26385 | 0.07604 | 0 | -0.2283 | -1.1715 | 2.12433 | 0.04358 | 0 | 0 |
| 1391151_at | --- | --- | uniqHCD_268 | 0.18 | -0.10 | 0.23 | -0.09 | -0.04 | 1 | 0.33599 | 1.26224 | 2.27265 | 0.01542 | 0 | 0.00817 | 1.00568 | 1.00082 | 0.47474 | 0 | 0.06485 | 1.04598 | 1.11242 | 0.3248 | 0 | 0 |
| 1391564_at | Zfp846 | zinc finger protein 846 | uniqHCD_268 | 0.03 | 0.06 | -0.25 | -0.06 | 0.08 | 1 | -0.3026 | -1.2334 | 3.34792 | 0.01292 | 0 | -0.1132 | -1.0816 | 1.0275 | 0.22546 | 0 | 0.02435 | 1.01702 | 1.20623 | 0.26755 | 0 | 0 |
| 1391596_at | --- | --- | uniqHCD_268 | 0.23 | 0.20 | -0.07 | 0.27 | -0.04 | 1 | -0.2747 | -1.2098 | 3.55941 | 0.00973 | 0 | 0.06116 | 1.04331 | 1.03005 | 0.39077 | 0 | -0.2418 | -1.1824 | 1.93852 | 0.05741 | 0 | 0 |
| 1391861_at | Txlnb | taxilin beta | uniqHCD_268 | 0.07 | 0.04 | -0.29 | -0.12 | -0.06 | 1 | -0.324 | -1.2518 | 3.39897 | 0.01268 | 0 | -0.1596 | -1.117 | 1.09837 | 0.14205 | 0 | -0.0956 | -1.0685 | 1.23902 | 0.22411 | 0 | 0 |
| 1391894_at | --- | --- | uniqHCD_268 | -0.12 | -0.02 | 0.33 | 0.20 | 0.19 | 1 | 0.35844 | 1.28203 | 2.34652 | 0.01064 | 0 | 0.22131 | 1.16579 | 1.28853 | 0.07147 | 0 | 0.21228 | 1.15852 | 1.96716 | 0.06553 | 0 | 0 |
| 1392023_at | --- | --- | uniqHCD_268 | -0.04 | -0.09 | 0.23 | 0.00 | 0.10 | 1 | 0.3202 | 1.24851 | 2.21813 | 0.02133 | 0 | 0.08431 | 1.06018 | 1.13308 | 0.22757 | 0 | 0.18887 | 1.13987 | 1.80122 | 0.08717 | 0 | 0 |
| 1392179_at | --- | --- | uniqHCD_268 | -0.01 | -0.17 | 0.10 | -0.12 | -0.06 | 1 | 0.27467 | 1.20972 | 2.09387 | 0.03239 | 0 | 0.05266 | 1.03717 | 1.07185 | 0.30564 | 0 | 0.1095 | 1.07886 | 1.28701 | 0.22865 | 0 | 0 |
| 1392317_at | --- | --- | uniqHCD_268 | -0.16 | 0.17 | -0.11 | 0.24 | -0.07 | 1 | -0.2828 | -1.2165 | 2.58197 | 0.04624 | 0 | 0.07411 | 1.05271 | 1.10369 | 0.26468 | 0 | -0.2424 | -1.183 | 1.93488 | 0.05823 | 0 | 0 |
| 1392478_at | Rab6b | RAB6B, member RAS oncogene family | uniqHCD_268 | 0.05 | 0.08 | -0.21 | -0.08 | -0.09 | 1 | -0.2879 | -1.2209 | 3.43257 | 0.01159 | 0 | -0.1558 | -1.1141 | 1.15193 | 0.11075 | 0 | -0.1733 | -1.1277 | 1.8777 | 0.0634 | 0 | 0 |
| 1392572_at | Msi2 | musashi RNA-binding protein 2 | uniqHCD_268 | -0.13 | -0.26 | 0.05 | -0.14 | -0.06 | 1 | 0.30448 | 1.23498 | 2.07814 | 0.03308 | 0 | 0.11078 | 1.07981 | 1.05233 | 0.34315 | 0 | 0.19669 | 1.14606 | 1.6115 | 0.12369 | 0 | 0 |
| 1392667_at | --- | --- | uniqHCD_268 | -0.02 | 0.00 | -0.30 | -0.12 | 0.10 | 1 | -0.3083 | -1.2383 | 3.27962 | 0.01645 | 0 | -0.124 | -1.0898 | 0.97471 | 0.32348 | 0 | 0.09357 | 1.06701 | 1.28353 | 0.23067 | 0 | 0 |
| 1392793_at | LOC10091199 | uncharacterized LOC100911990 | uniqHCD_268 | -0.01 | -0.27 | 0.04 | -0.32 | -0.16 | 1 | 0.3118 | 1.24126 | 2.26208 | 0.01746 | 0 | -0.0522 | -1.0368 | 1.00081 | 0.26543 | 0 | 0.11125 | 1.08017 | 1.4126 | 0.17915 | 0 | 0 |
| 1392853_at | --- | --- | uniqHCD_268 | -0.01 | 0.00 | 0.27 | 0.05 | 0.01 | 1 | 0.26856 | 1.20461 | 2.07978 | 0.02688 | 0 | 0.05388 | 1.03805 | 1.12757 | 0.23299 | 0 | 0.01428 | 1.00995 | 1.02262 | 0.40883 | 0 | 0 |
| 1392898_at | --- | --- | uniqHCD_268 | -0.13 | 0.03 | -0.24 | -0.06 | -0.12 | 1 | -0.2672 | -1.2035 | 3.0664 | 0.02495 | 0 | -0.0922 | -1.066 | 1.06994 | 0.1606 | 0 | -0.1501 | -1.1096 | 1.80187 | 0.07706 | 0 | 0 |
| 1392925_at | ST7 | suppression of tumorigenicity 7 | uniqHCD_268 | 0.11 | -0.15 | -0.50 | -0.04 | -0.17 | 1 | -0.3524 | -1.2766 | 3.34992 | 0.01271 | 0 | 0.11095 | 1.07994 | 1.16665 | 0.19804 | 0 | -0.0247 | -1.0173 | 1.0755 | 0.33515 | 0 | 0 |
| 1392927_at | Paqr4 | progestin and adipoQ receptor family member IV | uniqHCD_268 | 0.00 | -0.28 | 0.06 | -0.22 | -0.10 | 1 | 0.33502 | 1.26139 | 2.3311 | 0.01162 | 0 | 0.05419 | 1.03828 | 1.16728 | 0.19612 | 0 | 0.17454 | 1.12861 | 1.78104 | 0.09043 | 0 | 0 |
| 1392948_at | --- | --- | uniqHCD_268 | -0.18 | -0.15 | 0.12 | -0.15 | -0.16 | 1 | 0.27337 | 1.20863 | 2.08352 | 0.0272 | 0 | -0.0037 | -1.0026 | 0.96291 | 0.53668 | 0 | -0.0109 | -1.0076 | 0.94014 | 0.52813 | 0 | 0 |

| Affymetrix Probeset ID | Gene Symbol | Gene Title | Figure 6 B Venn n =2021 | Mean BN log2 intensity autologous n=8 | Mean log2 intensity day 30 DR +/+ ND n=4 | Mean log2 intensity day 30 DR+/+ HCD n=4 | Mean log2 intensity day 30 DR+/+ B/S n=4 | Mean log2 intensity day 30 F+/+ ND n=4 | DR+/+ HCD vs DR+/+ ND n=380 1=yes 0=no | Log2 ratio DR+/+ HCD vs DR+/+ ND | Fold-Change DR+/+ HCD vs DR+/+ ND | RP-FDR 4DR30 HCD vs 4DR30 Reg | p value DR+/+ HCD vs DR+/+ ND | DR+/+ B/S vs DR+/+ ND n=1,330 1=yes 0=no | Log2 ratio DR+/+ B/S vs DR+/+ ND | Fold-Change DR+/+ B/S vs DR+/+ ND | RP-FDR 4DR30 Antib vs 4DR30 Reg | p value DR+/+ B/S vs DR+/+ ND | DR+/+ ND vs F+/+ ND n=398 1=yes 0=no | Log2 ratio DR+/+ ND vs F+/+ ND | Fold-Change DR+/+ ND vs F+/+ ND | RP-FDR 4RT1uu 30Reg vs 4DR30 Reg | p value DR+/+ ND vs F+/+ ND | DR+/+ Longitudinal n=228 1=yes 0=no | Figure 6F |
|---|---|---|---|---|---|---|---|---|---|---|---|---|---|---|---|---|---|---|---|---|---|---|---|---|---|
| 1393068_at | --- | --- | uniqHCD_268 | 0.12 | 0.35 | 0.02 | 0.27 | 0.44 | 1 | -0.3352 | -1.2615 | 3.56143 | 0.00721 | 0 | -0.0861 | -1.0615 | 1.03361 | 0.20959 | 0 | 0.08722 | 1.06232 | 1.22047 | 0.26141 | 0 | 0 |
| 1393190_at | --- | --- | uniqHCD_268 | 0.01 | -0.11 | 0.17 | -0.16 | 0.08 | 1 | 0.27837 | 1.21282 | 2.29508 | 0.01934 | 0 | -0.0537 | -1.038 | 0.96794 | 0.39045 | 0 | 0.1872 | 1.13855 | 2.01407 | 0.06327 | 0 | 0 |
| 1393268_at | --- | --- | uniqHCD_268 | -0.07 | 0.08 | -0.22 | 0.07 | -0.01 | 1 | -0.2965 | -1.2281 | 3.66189 | 0.00624 | 0 | -0.0074 | -1.0052 | 1.0751 | 0.29924 | 0 | -0.0894 | -1.0639 | 1.7835 | 0.07943 | 0 | 0 |
| 1393284_at | --- | --- | uniqHCD_268 | 0.13 | -0.22 | 0.06 | -0.05 | -0.04 | 1 | 0.27334 | 1.2086 | 2.28621 | 0.02972 | 0 | 0.16322 | 1.11979 | 1.21881 | 0.13552 | 0 | 0.18174 | 1.13425 | 1.7243 | 0.09781 | 0 | 0 |
| 1393433_at | Egflam | EGF-like, fibronectin type III and laminin G domains | uniqHCD_268 | 0.05 | 0.19 | -0.09 | -0.08 | -0.07 | 1 | -0.279 | -1.2133 | 2.75723 | 0.03741 | 0 | -0.267 | -1.2033 | 1.39981 | 0.05316 | 0 | -0.2593 | -1.1969 | 2.41703 | 0.03062 | 0 | 0 |
| 1393528_at | Synpo2 | synaptopodin 2 | uniqHCD_268 | -0.22 | -0.06 | -0.35 | -0.29 | -0.25 | 1 | -0.2923 | -1.2246 | 3.07623 | 0.02493 | 0 | -0.2288 | -1.1719 | 1.18154 | 0.097 | 0 | -0.1866 | -1.1381 | 1.45223 | 0.14182 | 0 | 0 |
| 1393543_at | Zfp846 | zinc finger protein 846 | uniqHCD_268 | -0.25 | -0.07 | -0.35 | -0.17 | -0.18 | 1 | -0.2762 | -1.211 | 3.20139 | 0.02079 | 0 | -0.0965 | -1.0692 | 0.99552 | 0.28202 | 0 | -0.1038 | -1.0746 | 1.26176 | 0.21382 | 0 | 0 |
| 1393673_at | Sdr39u1 | short chain dehydrogenase/reductase family 39U, n | uniqHCD_268 | 0.00 | -0.12 | 0.15 | -0.11 | -0.18 | 1 | 0.26583 | 1.20233 | 2.09568 | 0.03059 | 0 | 0.00735 | 1.0051 | 1.00231 | 0.46446 | 0 | -0.0592 | -1.0419 | 0.98719 | 0.45308 | 0 | 0 |
| 1393719_at | --- | --- | uniqHCD_268 | -0.03 | 0.36 | 0.02 | 0.33 | 0.33 | 1 | -0.347 | -1.2719 | 3.69091 | 0.00914 | 0 | -0.0375 | -1.0263 | 1.04611 | 0.19086 | 0 | -0.0299 | -1.0209 | 1.04867 | 0.36624 | 0 | 0 |
| 1393797_at | --- | --- | uniqHCD_268 | -0.04 | -0.07 | 0.22 | -0.07 | -0.14 | 1 | 0.28728 | 1.22034 | 2.25081 | 0.01701 | 0 | 0.00496 | 1.00344 | 1.01479 | 0.43109 | 0 | -0.0675 | -1.0479 | 1.20865 | 0.2403 | 0 | 0 |
| 1393804_at | Cspp1 | centrosome and spindle pole associated protein 1 | uniqHCD_268 | -0.03 | 0.17 | 0.47 | 0.34 | -0.02 | 1 | 0.29549 | 1.2273 | 2.927 | 0.00188 | 0 | 0.16578 | 1.12177 | 1.26138 | 0.0389 | 0 | -0.1899 | -1.1407 | 2.85449 | 0.02084 | 0 | 0 |
| 1393813_at | Ttc3 | tetratricopeptide repeat domain 3 | uniqHCD_268 | -0.18 | -0.03 | -0.33 | -0.04 | -0.26 | 1 | -0.3069 | -1.237 | 3.07584 | 0.02403 | 0 | -0.0085 | -1.0059 | 0.9834 | 0.30616 | 0 | -0.2319 | -1.1744 | 2.23586 | 0.03789 | 0 | 0 |
| 1394079_at | Tbl1xr1 | transducin (beta)-like 1 X-linked receptor 1 | uniqHCD_268 | 0.03 | 0.37 | 0.11 | 0.36 | 0.13 | 1 | -0.268 | -1.2041 | 3.747 | 0.00843 | 0 | -0.0166 | -1.0116 | 1.64333 | 0.0306 | 0 | -0.2433 | -1.1837 | 3.34895 | 0.00409 | 0 | 0 |
| 1394424_at | Ccdc104 | coiled-coil domain containing 104 | uniqHCD_268 | 0.06 | -0.10 | -0.38 | -0.14 | -0.16 | 1 | -0.282 | -1.2159 | 2.82975 | 0.03612 | 0 | -0.0449 | -1.0316 | 0.96375 | 0.48589 | 0 | -0.0657 | -1.0466 | 1.21368 | 0.23767 | 0 | 0 |
| 1394814_at | Tpr | translocated promoter region, nuclear basket prote | uniqHCD_268 | 0.00 | -0.40 | -0.11 | -0.18 | -0.31 | 1 | 0.28629 | 1.2195 | 2.35833 | 0.00045 | 0 | 0.22139 | 1.16585 | 0.57039 | 0.00328 | 0 | 0.08764 | 1.06263 | 1.58132 | 0.00386 | 0 | 0 |
| 1394849_at | Zbtb20 | zinc finger and BTB domain containing 20 | uniqHCD_268 | -0.15 | 0.23 | 0.50 | 0.43 | 0.24 | 1 | 0.27262 | 1.208 | 2.40357 | 0.00757 | 0 | 0.20109 | 1.14956 | 1.24843 | 0.03448 | 0 | 0.00843 | 1.00586 | 1.6278 | 0.12123 | 0 | 0 |
| 1394986_at | Fam120b | family with sequence similarity 120B | uniqHCD_268 | 0.11 | 0.17 | -0.10 | 0.17 | 0.02 | 1 | -0.2761 | -1.2109 | 3.07832 | 0.02356 | 0 | 0.00151 | 1.00105 | 0.96677 | 0.38258 | 0 | -0.1537 | -1.1124 | 1.9141 | 0.06038 | 0 | 0 |
| 1395299_at | Krt24 | keratin 24 | uniqHCD_268 | -0.05 | -0.25 | 0.06 | -0.35 | -0.14 | 1 | 0.3136 | 1.24281 | 2.27597 | 0.01852 | 0 | -0.0977 | -1.0701 | 0.999 | 0.27584 | 0 | 0.11277 | 1.0813 | 1.67429 | 0.10708 | 0 | 0 |
| 1395427_at | --- | --- | uniqHCD_268 | 0.07 | -0.11 | -0.40 | -0.15 | -0.17 | 1 | -0.289 | -1.2218 | 3.04985 | 0.02579 | 0 | -0.0405 | -1.0284 | 0.96353 | 0.48373 | 0 | -0.0604 | -1.0427 | 1.10101 | 0.31668 | 0 | 0 |
| 1395434_at | Traf3 | Tnf receptor-associated factor 3 | uniqHCD_268 | 0.08 | 0.05 | 0.39 | 0.20 | 0.18 | 1 | 0.33719 | 1.26329 | 2.27643 | 0.01537 | 0 | 0.14592 | 1.10643 | 1.12631 | 0.23722 | 0 | 0.1254 | 1.09081 | 1.1945 | 0.27309 | 0 | 0 |
| 1395650_at | --- | --- | uniqHCD_268 | -0.06 | 0.08 | 0.40 | 0.09 | 0.11 | 1 | 0.31466 | 1.24372 | 2.21827 | 0.01569 | 0 | 0.01296 | 1.00902 | 0.97409 | 0.3432 | 0 | 0.0282 | 1.01974 | 1.04467 | 0.38043 | 0 | 0 |
| 1395669_at | --- | --- | uniqHCD_268 | -0.25 | -0.18 | -0.47 | -0.37 | -0.40 | 1 | -0.2881 | -1.2211 | 3.01242 | 0.02925 | 0 | -0.1848 | -1.1366 | 1.0841 | 0.14965 | 0 | -0.2157 | -1.1612 | 1.74238 | 0.08712 | 0 | 0 |
| 1395684_at | --- | --- | uniqHCD_268 | -0.03 | -0.22 | 0.06 | -0.16 | -0.07 | 1 | 0.28452 | 1.218 | 2.06539 | 0.03487 | 0 | 0.06572 | 1.04661 | 1.15831 | 0.20902 | 0 | 0.15697 | 1.11494 | 1.57697 | 0.13128 | 0 | 0 |
| 1395687_at | Atf7ip | activating transcription factor 7 interacting protein | uniqHCD_268 | -0.03 | 0.19 | -0.09 | 0.16 | 0.23 | 1 | -0.2752 | -1.2101 | 2.65763 | 0.04179 | 0 | -0.0248 | -1.0173 | 0.96164 | 0.55236 | 0 | 0.04144 | 1.02914 | 1.00387 | 0.42451 | 0 | 0 |
| 1395715_at | --- | --- | uniqHCD_268 | -0.14 | -0.29 | -0.02 | -0.35 | -0.10 | 1 | 0.27134 | 1.20693 | 2.11881 | 0.02575 | 0 | -0.0568 | -1.0402 | 0.96303 | 0.46605 | 0 | 0.18838 | 1.13949 | 1.75442 | 0.09399 | 0 | 0 |
| 1395816_at | Fam13c | family with sequence similarity 13, member C | uniqHCD_268 | 0.23 | -0.04 | 0.25 | 0.04 | -0.04 | 1 | 0.28472 | 1.21817 | 2.09264 | 0.02799 | 0 | 0.07991 | 1.05695 | 1.05677 | 0.33148 | 0 | -0.0026 | -1.0018 | 0.97913 | 0.45448 | 0 | 0 |
| 1395854_at | --- | --- | uniqHCD_268 | -0.08 | 0.17 | -0.12 | 0.14 | -0.09 | 1 | -0.2941 | -1.2261 | 2.82077 | 0.03637 | 0 | -0.036 | -1.0253 | 0.9685 | 0.37019 | 0 | -0.2589 | -1.1965 | 2.61874 | 0.02669 | 0 | 0 |
| 1395867_at | --- | --- | uniqHCD_268 | -0.12 | -0.16 | 0.11 | -0.03 | -0.02 | 1 | 0.27005 | 1.20585 | 2.03053 | 0.04074 | 0 | 0.12651 | 1.09165 | 1.10134 | 0.2683 | 0 | 0.13532 | 1.09834 | 1.2201 | 0.26204 | 0 | 0 |
| 1396241_at | --- | --- | uniqHCD_268 | 0.01 | -0.12 | 0.15 | -0.04 | -0.10 | 1 | 0.26778 | 1.20395 | 2.07704 | 0.03814 | 0 | 0.07777 | 1.05539 | 1.04467 | 0.35832 | 0 | 0.01843 | 1.01285 | 0.96482 | 0.47054 | 0 | 0 |
| 1396257_at | --- | --- | uniqHCD_268 | -0.20 | -0.45 | -0.14 | -0.23 | -0.42 | 1 | 0.31196 | 1.24139 | 2.10873 | 0.02624 | 0 | 0.22256 | 1.1668 | 1.25756 | 0.10578 | 0 | 0.02843 | 1.0199 | 0.9344 | 0.5201 | 0 | 0 |
| 1396321_at | --- | --- | uniqHCD_268 | 0.02 | 0.02 | -0.29 | -0.06 | -0.22 | 1 | -0.3074 | -1.2375 | 3.37111 | 0.01073 | 0 | -0.0763 | -1.0543 | 1.0132 | 0.24552 | 0 | -0.2355 | -1.1773 | 2.3999 | 0.03102 | 0 | 0 |
| 1396373_at | --- | --- | uniqHCD_268 | -0.24 | 0.18 | -0.14 | 0.17 | 0.05 | 1 | -0.3274 | -1.2547 | 3.27477 | 0.01569 | 0 | -0.0094 | -1.0065 | 0.99033 | 0.29246 | 0 | -0.1309 | -1.095 | 1.58233 | 0.10634 | 0 | 0 |
| 1396519_at | --- | --- | uniqHCD_268 | -0.16 | 0.02 | -0.31 | -0.18 | -0.20 | 1 | -0.3238 | -1.2517 | 3.58235 | 0.00587 | 0 | -0.2003 | -1.1489 | 1.64711 | 0.03114 | 0 | -0.221 | -1.1655 | 2.21611 | 0.03919 | 0 | 0 |
| 1396746_at | --- | --- | uniqHCD_268 | -0.02 | -0.06 | 0.24 | -0.09 | -0.11 | 1 | 0.30195 | 1.23281 | 2.5225 | 0.00454 | 0 | -0.0337 | -1.0237 | 0.99943 | 0.27416 | 0 | -0.0499 | -1.0352 | 1.19598 | 0.24778 | 0 | 0 |
| 1396821_at | --- | --- | uniqHCD_268 | 0.13 | -0.07 | 0.29 | -0.11 | 0.01 | 1 | 0.3526 | 1.27686 | 2.63941 | 0.00433 | 0 | -0.0383 | -1.0269 | 1.09747 | 0.14261 | 0 | 0.08052 | 1.0574 | 1.6612 | 0.11298 | 0 | 0 |
| 1396855_at | --- | --- | uniqHCD_268 | -0.07 | -0.29 | -0.02 | -0.29 | -0.21 | 1 | 0.26576 | 1.20227 | 2.08783 | 0.02967 | 0 | -0.0079 | -1.0055 | 0.98051 | 0.5509 | 0 | 0.07538 | 1.05364 | 1.04515 | 0.3801 | 0 | 0 |
| 1396873_at | --- | --- | uniqHCD_268 | -0.16 | 0.14 | 0.51 | 0.31 | 0.24 | 1 | 0.36662 | 1.28933 | 2.45931 | 0.00459 | 0 | 0.17265 | 1.12713 | 1.28075 | 0.07915 | 0 | 0.10471 | 1.07528 | 1.56872 | 0.13367 | 0 | 0 |
| 1396919_at | Wnk1 | WNK lysine deficient protein kinase 1 | uniqHCD_268 | -0.05 | -0.17 | 0.15 | 0.03 | -0.10 | 1 | 0.3224 | 1.25041 | 2.09103 | 0.02804 | 0 | 0.2001 | 1.14878 | 1.21048 | 0.14199 | 0 | 0.07006 | 1.04976 | 1.10015 | 0.33515 | 0 | 0 |
| 1396995_at | --- | --- | uniqHCD_268 | 0.00 | 0.12 | -0.16 | -0.03 | -0.03 | 1 | -0.2752 | -1.2102 | 2.80864 | 0.03649 | 0 | -0.1409 | -1.1026 | 1.04301 | 0.19496 | 0 | -0.1472 | -1.1074 | 1.25458 | 0.21663 | 0 | 0 |
| 1397047_at | --- | --- | uniqHCD_268 | -0.13 | 0.17 | -0.09 | 0.17 | 0.03 | 1 | -0.269 | -1.2049 | 2.53729 | 0.04936 | 0 | 0.00098 | 1.00068 | 0.97044 | 0.6372 | 0 | -0.1426 | -1.1039 | 1.39811 | 0.15924 | 0 | 0 |
| 1397164_at | LOC10091244 | uncharacterized LOC100912446 | uniqHCD_268 | 0.11 | 0.12 | 0.38 | 0.36 | 0.35 | 1 | 0.26458 | 1.20129 | 2.08254 | 0.03777 | 0 | 0.24277 | 1.18326 | 1.28552 | 0.06006 | 0 | 0.23002 | 1.17285 | 1.99774 | 0.06372 | 0 | 0 |
| 1397171_at | Rnft2 | ring finger protein, transmembrane 2 | uniqHCD_268 | -0.16 | -0.14 | -0.50 | -0.31 | -0.37 | 1 | -0.3612 | -1.2845 | 3.62204 | 0.00629 | 0 | -0.1643 | -1.1206 | 1.27652 | 0.07421 | 0 | -0.2239 | -1.1679 | 2.02774 | 0.04929 | 0 | 0 |
| 1397196_at | --- | --- | uniqHCD_268 | 0.07 | -0.13 | 0.19 | -0.07 | -0.01 | 1 | 0.32126 | 1.24942 | 2.26815 | 0.01182 | 0 | 0.06053 | 1.04285 | 1.0674 | 0.31642 | 0 | 0.12216 | 1.08836 | 1.37081 | 0.19223 | 0 | 0 |
| 1397242_at | Klhl38 | kelch-like family member 38 | uniqHCD_268 | 0.16 | 0.16 | -0.12 | 0.10 | 0.03 | 1 | -0.2788 | -1.2132 | 3.22745 | 0.02034 | 0 | -0.0578 | -1.0409 | 0.99034 | 0.29281 | 0 | -0.1318 | -1.0957 | 1.33064 | 0.1818 | 0 | 0 |
| 1397257_at | --- | --- | uniqHCD_268 | -0.22 | -0.23 | -0.55 | -0.25 | -0.41 | 1 | -0.3126 | -1.242 | 3.06893 | 0.02388 | 0 | -0.0208 | -1.0145 | 0.96339 | 0.4441 | 0 | -0.1794 | -1.1324 | 1.58087 | 0.107 | 0 | 0 |
| 1397259_at | Fam126b | family with sequence similarity 126, member B | uniqHCD_268 | -0.23 | 0.03 | 0.33 | 0.14 | 0.02 | 1 | 0.30422 | 1.23475 | 2.4914 | 0.00689 | 0 | 0.11553 | 1.08338 | 1.21119 | 0.14379 | 0 | -0.0095 | -1.0066 | 1.24511 | 0.22228 | 0 | 0 |
| 1397392_at | --- | --- | uniqHCD_268 | -0.01 | -0.38 | -0.04 | -0.45 | -0.31 | 1 | 0.34331 | 1.26867 | 2.33831 | 0.01113 | 0 | -0.0707 | -1.0502 | 1.03199 | 0.21473 | 0 | 0.07246 | 1.05151 | 1.52016 | 0.14664 | 0 | 0 |
| 1397423_at | --- | --- | uniqHCD_268 | -0.04 | -0.21 | 0.13 | 0.02 | -0.07 | 1 | 0.34306 | 1.26845 | 2.28724 | 0.0128 | 0 | 0.23417 | 1.17623 | 1.27048 | 0.09294 | 0 | 0.14434 | 1.10523 | 1.55713 | 0.13584 | 0 | 0 |
| 1397472_at | --- | --- | uniqHCD_268 | 0.13 | -0.01 | 0.25 | -0.17 | 0.18 | 1 | 0.26751 | 1.20373 | 1.95607 | 0.04642 | 0 | -0.1598 | -1.1171 | 1.08371 | 0.15141 | 0 | 0.19292 | 1.14308 | 1.76457 | 0.09362 | 0 | 0 |
| 1397654_at | --- | --- | uniqHCD_268 | -0.18 | 0.03 | -0.23 | -0.15 | -0.14 | 1 | -0.2641 | -1.2009 | 2.88808 | 0.03297 | 0 | -0.182 | -1.1344 | 1.0805 | 0.15385 | 0 | -0.1732 | -1.1276 | 1.41567 | 0.15259 | 0 | 0 |
| 1397710_at | --- | --- | uniqHCD_268 | -0.12 | -0.25 | 0.04 | -0.01 | -0.07 | 1 | 0.2876 | 1.22061 | 2.23536 | 0.01675 | 0 | 0.24121 | 1.18199 | 1.27685 | 0.05202 | 0 | 0.18336 | 1.13553 | 1.67707 | 0.10699 | 0 | 0 |
| 1397715_at | --- | --- | uniqHCD_268 | -0.08 | 0.02 | 0.30 | 0.28 | 0.19 | 1 | 0.27413 | 1.20926 | 2.24372 | 0.01551 | 0 | 0.25478 | 1.19316 | 1.27542 | 0.05172 | 0 | 0.16799 | 1.12349 | 1.53221 | 0.14426 | 0 | 0 |
| 1397854_at | Dpf3 | D4, zinc and double PHD fingers, family 3 | uniqHCD_268 | 0.09 | -0.09 | 0.18 | 0.04 | 0.09 | 1 | 0.27062 | 1.20632 | 2.06013 | 0.03551 | 0 | 0.13133 | 1.0953 | 1.16253 | 0.20515 | 0 | 0.18217 | 1.13459 | 1.68163 | 0.10647 | 0 | 0 |
| 1397915_at | --- | --- | uniqHCD_268 | -0.19 | 0.06 | 0.34 | 0.24 | 0.31 | 1 | 0.28674 | 1.21988 | 2.10381 | 0.02625 | 0 | 0.18754 | 1.13882 | 1.2121 | 0.14156 | 0 | 0.25593 | 1.1941 | 2.35981 | 0.02034 | 0 | 0 |
| 1397945_at | Wisp3 | WNT1 inducible signaling pathway protein 3 | uniqHCD_268 | 0.04 | -0.07 | 0.22 | 0.03 | -0.04 | 1 | 0.28423 | 1.21776 | 2.06776 | 0.03693 | 0 | 0.0934 | 1.06688 | 1.06844 | 0.31257 | 0 | 0.02729 | 1.0191 | 0.97406 | 0.46026 | 0 | 0 |
| 1398048_at | Ints10 | integrator complex subunit 10 | uniqHCD_268 | -0.06 | -0.15 | 0.12 | -0.06 | -0.10 | 1 | 0.26968 | 1.20554 | 2.09385 | 0.03023 | 0 | 0.09321 | 1.06674 | 1.12113 | 0.2446 | 0 | 0.04952 | 1.03492 | 1.11473 | 0.32296 | 0 | 0 |
| 1398084_at | --- | --- | uniqHCD_268 | 0.16 | -0.25 | 0.11 | -0.12 | -0.09 | 1 | 0.36216 | 1.28535 | 2.34315 | 0.01123 | 0 | 0.13457 | 1.09777 | 1.17867 | 0.18454 | 0 | 0.16328 | 1.11983 | 1.49776 | 0.15036 | 0 | 0 |
| 1398086_at | --- | --- | uniqHCD_268 | -0.09 | 0.07 | -0.30 | -0.20 | -0.08 | 1 | -0.3671 | -1.2898 | 3.60193 | 0.0066 | 0 | -0.2707 | -1.2064 | 1.32689 | 0.06042 | 0 | -0.1501 | -1.1096 | 1.46839 | 0.13872 | 0 | 0 |
| 1398290_at | Kcnk13 | potassium channel, subfamily K, member 13 | uniqHCD_268 | -0.04 | -0.23 | 0.15 | -0.17 | -0.14 | 1 | 0.37227 | 1.29439 | 2.3552 | 0.00962 | 0 | 0.05207 | 1.03675 | 0.98771 | 0.51255 | 0 | 0.08999 | 1.06436 | 1.54712 | 0.13954 | 0 | 0 |
| 1398643_at | Dicer1 | dicer 1, ribonuclease type III | uniqHCD_268 | 0.01 | 0.22 | -0.07 | 0.19 | 0.27 | 1 | -0.2871 | -1.2202 | 3.07168 | 0.02874 | 0 | -0.0321 | -1.0225 | 0.97105 | 0.35587 | 0 | 0.04817 | 1.03395 | 1.1521 | 0.2977 | 0 | 0 |
| 1398825_at | Rab11b | RAB11B, member RAS oncogene family | uniqHCD_268 | 0.02 | 0.32 | 0.02 | 0.15 | 0.28 | 1 | -0.2989 | -1.2302 | 3.66411 | 0.0066 | 0 | -0.1673 | -1.123 | 1.29585 | 0.06742 | 0 | -0.0378 | -1.0266 | 1.11353 | 0.3065 | 0 | 0 |
| 1399038_at | Usp19 | ubiquitin specific peptidase 19 | uniqHCD_268 | 0.08 | 0.30 | 0.02 | 0.28 | 0.27 | 1 | -0.2803 | -1.2144 | 2.70675 | 0.0383 | 0 | -0.0161 | -1.0112 | 0.96168 | 0.54997 | 0 | -0.0328 | -1.023 | 0.96268 | 0.49235 | 0 | 0 |
| 1367628_at | Lgals1 | lectin, galactoside-binding, soluble, 1 | uniqPf7DR_155 | -0.05 | -0.22 | -0.12 | -0.09 | -0.26 | 0 | 0.10138 | 1.0728 | 1.24064 | 0.25843 | 0 | 0.13323 | 1.09675 | 1.23182 | 0.12584 | 0 | -0.0387 | -1.0272 | 0.98472 | 0.45733 | 1 | 0 |
| 1367860_a_at | Mmp14 | matrix metallopeptidase 14 (membrane-inserted) | uniqPf7DR_155 | -0.08 | -0.28 | -0.13 | -0.03 | -0.03 | 0 | 0.15055 | 1.11 | 1.76149 | 0.08349 | 0 | 0.2565 | 1.19458 | 1.29065 | 0.05682 | 0 | 0.2506 | 1.18971 | 2.3033 | 0.04133 | 1 | 0 |
| 1367906_at | Acp2 | acid phosphatase 2, lysosomal | uniqPf7DR_155 | -0.02 | -0.17 | -0.19 | -0.04 | -0.19 | 0 | -0.0163 | -1.0114 | 1.07711 | 0.35376 | 0 | 0.13589 | 1.09877 | 1.1974 | 0.16175 | 0 | -0.0216 | -1.0151 | 1.23218 | 0.22774 | 1 | 0 |
| 1367932_at | Hmgcs1 | 3-hydroxy-3-methylglutaryl-CoA synthase 1 (soluble | uniqPf7DR_155 | -0.11 | -0.20 | -0.29 | 0.01 | -0.08 | 0 | -0.0904 | -1.0647 | 1.09256 | 0.34461 | 0 | 0.20559 | 1.15315 | 1.21017 | 0.1432 | 0 | 0.12511 | 1.09059 | 1.20525 | 0.26826 | 1 | 0 |
| 1367938_at | Ugdh | UDP-glucose 6-dehydrogenase | uniqPf7DR_155 | -0.03 | -0.05 | -0.03 | 0.05 | 0.02 | 0 | 0.02291 | 1.01601 | 1.05292 | 0.38783 | 0 | 0.10743 | 1.07731 | 1.05841 | 0.32482 | 0 | 0.07462 | 1.05308 | 1.08836 | 0.34321 | 1 | 0 |
| 1368000_at | C3 | complement component 3 | uniqPf7DR_155 | 0.14 | -0.18 | 0.01 | -0.22 | 0.06 | 0 | 0.18691 | 1.13832 | 1.85561 | 0.06713 | 0 | -0.0332 | -1.0233 | 0.99049 | 0.28986 | 0 | 0.24476 | 1.1849 | 2.26153 | 0.01476 | 1 | 0 |
| 1368321_at | Egr1 | early growth response 1 | uniqPf7DR_155 | 0.12 | 0.37 | 0.36 | 0.50 | 0.29 | 0 | -0.0082 | -1.0057 | 1.67655 | 0.13963 | 0 | 0.13274 | 1.09637 | 1.27693 | 0.0494 | 0 | -0.078 | -1.0555 | 1.74692 | 0.08628 | 1 | 0 |
| 1368331_at | Ctbs | chitobiase, di-N-acetyl- | uniqPf7DR_155 | -0.04 | -0.21 | -0.21 | 0.01 | -0.06 | 0 | 0.00358 | 1.00249 | 1.02394 | 0.422 | 0 | 0.22226 | 1.16656 | 1.24073 | 0.11526 | 0 | 0.1523 | 1.11134 | 1.53775 | 0.14171 | 1 | 0 |
| 1368450_at | Myo5a | myosin VA | uniqPf7DR_155 | -0.04 | -0.13 | 0.01 | 0.04 | -0.21 | 0 | 0.14522 | 1.1059 | 1.67787 | 0.11255 | 0 | 0.17328 | 1.12762 | 1.19972 | 0.15701 | 0 | -0.0787 | -1.0561 | 1.11136 | 0.30808 | 1 | 0 |

| Affymetrix Probeset ID | Gene Symbol | Gene Title | Figure 6 B Venn n =2021 | Mean BN log2 intensity autologous n=8 | Mean log2 intensity day 30 DR +/+ ND n=4 | Mean log2 intensity day 30 DR+/+ HCD n=4 | Mean log2 intensity day 30 DR+/+ B/S n=4 | Mean log2 intensity day 30 F+/+ ND n=4 | DR+/+ HCD vs DR+/+ ND n=380 1=yes 0=no | Log2 ratio DR+/+ HCD vs DR+/+ ND | Fold-Change DR+/+ HCD vs DR+/+ ND | RP-FDR 4DR30 HCD vs 4DR30 Reg | p value DR+/+ HCD vs DR+/+ ND | DR+/+ B/S vs DR+/+ ND n=1,330 1=yes 0=no | Log2 ratio DR+/+ B/S vs DR+/+ ND | Fold-Change DR+/+ B/S vs DR+/+ ND | RP-FDR 4DR30 Antib vs 4DR30 Reg | p value DR+/+ B/S vs DR+/+ ND | DR+/+ ND vs F+/+ ND n=398 1=yes 0=no | Log2 ratio DR+/+ ND vs F+/+ ND | Fold-Change DR+/+ ND vs F+/+ ND | RP-FDR 4RT1uu 30Reg vs 4DR30 Reg | p value DR+/+ ND vs F+/+ ND | DR+/+ Longitudinal n=228 1=yes 0=no | Figure 6F |
|---|---|---|---|---|---|---|---|---|---|---|---|---|---|---|---|---|---|---|---|---|---|---|---|---|---|
| 1368482_at | Bcl2a1 | BCL2-related protein A1 | uniqPf7DR_155 | 0.03 | -0.33 | -0.33 | -0.11 | -0.15 | 0 | -0.0038 | -1.0027 | 1.07738 | 0.36913 | 0 | 0.21968 | 1.16448 | 1.27518 | 0.09058 | 0 | 0.17496 | 1.12894 | 1.82292 | 0.08324 | 1 | 0 |
| 1368511_at | Bhlhe41 | basic helix-loop-helix family, member e41 | uniqPf7DR_155 | 0.02 | 0.16 | 0.12 | 0.08 | 0.05 | 0 | -0.0398 | -1.028 | 1.32317 | 0.23099 | 0 | -0.0811 | -1.0578 | 0.97012 | 0.36504 | 0 | -0.1144 | -1.0825 | 1.36256 | 0.17022 | 1 | 0 |
| 1368530_at | Mmp12 | matrix metallopeptidase 12 | uniqPf7DR_155 | 0.03 | -0.12 | -0.11 | -0.18 | -0.05 | 0 | 0.0118 | 1.00821 | 0.92864 | 0.54343 | 0 | -0.0536 | -1.0379 | 0.96219 | 0.49154 | 0 | 0.07003 | 1.04974 | 1.02842 | 0.39785 | 1 | 0 |
| 1368908_at | Anxa4 | annexin A4 | uniqPf7DR_155 | 0.07 | -0.27 | -0.38 | -0.13 | -0.17 | 0 | -0.1024 | -1.0736 | 1.3939 | 0.20555 | 0 | 0.14181 | 1.10329 | 1.26114 | 0.10515 | 0 | 0.1011 | 1.07259 | 1.48834 | 0.15348 | 1 | 0 |
| 1369103_at | Fyn | FYN oncogene related to SRC, FGR, YES | uniqPf7DR_155 | -0.11 | 0.39 | 0.32 | 0.48 | 0.51 | 0 | -0.0637 | -1.0451 | 1.73393 | 0.13203 | 0 | 0.09128 | 1.06531 | 1.17143 | 0.18781 | 0 | 0.11804 | 1.08526 | 1.62917 | 0.1208 | 1 | 0 |
| 1369138_a_at | Park2 | parkinson protein 2, E3 ubiquitin protein ligase | uniqPf7DR_155 | 0.01 | 0.02 | -0.11 | -0.06 | -0.02 | 0 | -0.129 | -1.0935 | 2.62625 | 0.04231 | 0 | -0.0792 | -1.0564 | 1.13861 | 0.12115 | 0 | -0.0417 | -1.0294 | 1.50384 | 0.12394 | 1 | 0 |
| 1369166_at | Mmp9 | matrix metallopeptidase 9 | uniqPf7DR_155 | 0.05 | -0.30 | -0.11 | -0.22 | -0.28 | 0 | 0.19773 | 1.14689 | 2.10063 | 0.02776 | 0 | 0.07875 | 1.0561 | 1.15795 | 0.20907 | 0 | 0.02028 | 1.01416 | 1.06951 | 0.36168 | 1 | 0 |
| 1369181_at | Cybb | cytochrome b-245, beta polypeptide | uniqPf7DR_155 | -0.02 | 0.10 | 0.02 | 0.19 | 0.13 | 0 | -0.0845 | -1.0603 | 1.09867 | 0.34109 | 0 | 0.08391 | 1.05989 | 1.05258 | 0.34208 | 0 | 0.0295 | 1.02066 | 0.98431 | 0.44751 | 1 | 0 |
| 1369204_at | Hck | hemopoietic cell kinase | uniqPf7DR_155 | 0.10 | 0.09 | 0.04 | 0.14 | 0.31 | 0 | -0.0496 | -1.035 | 1.01404 | 0.41212 | 0 | 0.05528 | 1.03906 | 1.098 | 0.27165 | 0 | 0.22017 | 1.16487 | 2.36192 | 0.02453 | 1 | 0 |
| 1369249_at | Ankh | ankylosis, progressive homolog (mouse) | uniqPf7DR_155 | -0.05 | 0.35 | 0.36 | 0.30 | 0.31 | 0 | 0.0077 | 1.00535 | 0.94299 | 0.51993 | 0 | -0.0518 | -1.0365 | 0.97055 | 0.35793 | 0 | -0.0428 | -1.0301 | 1.0053 | 0.42347 | 1 | 0 |
| 1369262_at | Casp8 | caspase 8 | uniqPf7DR_155 | -0.08 | 0.00 | -0.02 | -0.02 | -0.04 | 0 | -0.0206 | -1.0144 | 0.94998 | 0.47491 | 0 | -0.0211 | -1.0147 | 0.96268 | 0.52039 | 0 | -0.0393 | -1.0276 | 1.06904 | 0.34094 | 1 | 0 |
| 1369497_at | LOC24906 | RoBo-1 | uniqPf7DR_155 | 0.06 | 0.07 | -0.13 | -0.01 | 0.13 | 0 | -0.202 | -1.1503 | 3.26123 | 0.01615 | 0 | -0.0881 | -1.063 | 1.27971 | 0.07378 | 0 | 0.05523 | 1.03902 | 1.98221 | 0.06457 | 1 | 0 |
| 1369554_at | Syngr2 | synaptogyrin 2 | uniqPf7DR_155 | 0.05 | 0.32 | 0.31 | 0.41 | 0.44 | 0 | -0.0053 | -1.0037 | 0.99804 | 0.42609 | 0 | 0.093 | 1.06659 | 1.10159 | 0.26683 | 0 | 0.12276 | 1.08882 | 1.39473 | 0.18455 | 1 | 0 |
| 1369627_at | Sv2b | synaptic vesicle glycoprotein 2b | uniqPf7DR_155 | -0.03 | 0.03 | 0.08 | 0.09 | 0.09 | 0 | 0.05328 | 1.03762 | 1.16955 | 0.30214 | 0 | 0.06408 | 1.04542 | 1.0968 | 0.27255 | 0 | 0.0643 | 1.04558 | 1.20071 | 0.27081 | 1 | 0 |
| 1369628_at | Sv2b | synaptic vesicle glycoprotein 2b | uniqPf7DR_155 | -0.05 | -0.06 | 0.10 | -0.04 | -0.05 | 0 | 0.15666 | 1.1147 | 1.75318 | 0.09037 | 0 | 0.02061 | 1.01439 | 1.0407 | 0.3668 | 0 | 0.01275 | 1.00888 | 1.03229 | 0.39291 | 1 | 0 |
| 1369743_a_at | P2rx4 | purinergic receptor P2X, ligand-gated ion channel 4 | uniqPf7DR_155 | 0.00 | -0.03 | -0.02 | 0.08 | 0.04 | 0 | 0.00061 | 1.00043 | 0.89631 | 0.60971 | 0 | 0.10573 | 1.07604 | 1.05315 | 0.34067 | 0 | 0.06394 | 1.04532 | 0.99383 | 0.43647 | 1 | 0 |
| 1370047_at | Enpp1 | ectonucleotide pyrophosphatase/phosphodiesteras | uniqPf7DR_155 | 0.04 | 0.19 | 0.13 | 0.32 | 0.13 | 0 | -0.0524 | -1.037 | 1.06895 | 0.36129 | 0 | 0.13719 | 1.09976 | 1.16334 | 0.20671 | 0 | -0.0545 | -1.0385 | 1.10054 | 0.31754 | 1 | 0 |
| 1370060_at | Slc25a11 | solute carrier family 25 (mitochondrial carrier; oxog | uniqPf7DR_155 | -0.02 | 0.06 | 0.01 | -0.04 | -0.03 | 0 | -0.0468 | -1.033 | 0.91279 | 0.53883 | 0 | -0.1061 | -1.0763 | 0.96707 | 0.37985 | 0 | -0.0908 | -1.065 | 1.03471 | 0.3792 | 1 | 0 |
| 1370114_a_at | Pik3r1 | phosphoinositide-3-kinase, regulatory subunit 1 (alp | uniqPf7DR_155 | -0.05 | -0.13 | -0.07 | -0.12 | -0.15 | 0 | 0.06474 | 1.0459 | 1.21252 | 0.27534 | 0 | 0.01173 | 1.00817 | 1.01241 | 0.43896 | 0 | -0.0178 | -1.0124 | 0.97951 | 0.46451 | 1 | 0 |
| 1370204_at | Pgap2 | post-GPI attachment to proteins 2 | uniqPf7DR_155 | 0.09 | 0.21 | 0.23 | 0.30 | 0.31 | 0 | 0.01913 | 1.01335 | 1.04852 | 0.39676 | 0 | 0.08902 | 1.06365 | 1.12824 | 0.23248 | 0 | 0.09725 | 1.06973 | 1.53753 | 0.14219 | 1 | 0 |
| 1370344_at | Hspa4 | heat shock protein 4 | uniqPf7DR_155 | -0.10 | 0.18 | 0.18 | 0.21 | 0.21 | 0 | 0.00055 | 1.00038 | 1.25331 | 0.25393 | 0 | 0.03541 | 1.02485 | 1.09997 | 0.26945 | 0 | 0.03068 | 1.0215 | 1.27911 | 0.23329 | 1 | 0 |
| 1370383_s_at | RT1-Db1 | RT1 class II, locus Db1 | uniqPf7DR_155 | -0.08 | 0.20 | 0.19 | 0.11 | 0.21 | 0 | -0.0107 | -1.0075 | 0.87681 | 0.6311 | 0 | -0.0936 | -1.067 | 0.96514 | 0.40875 | 0 | 0.00834 | 1.0058 | 0.8969 | 0.63599 | 1 | 0 |
| 1370882_at | LOC10090963 | class II histocompatibility antigen, M beta 1 chain-lil | uniqPf7DR_155 | 0.01 | 0.49 | 0.47 | 0.43 | 0.40 | 0 | -0.021 | -1.0146 | 0.88871 | 0.59034 | 0 | -0.0633 | -1.0449 | 0.96217 | 0.49168 | 0 | -0.0955 | -1.0684 | 1.06229 | 0.34941 | 1 | 0 |
| 1370955_at | Adam10 | ADAM metallopeptidase domain 10 | uniqPf7DR_155 | -0.12 | -0.07 | 0.07 | -0.01 | -0.15 | 0 | 0.14029 | 1.10213 | 1.99087 | 0.04404 | 0 | 0.05979 | 1.04231 | 1.19767 | 0.16202 | 0 | -0.0795 | -1.0567 | 1.62969 | 0.09936 | 1 | 0 |
| 1371210_s_at | RT1-CE5 /// R | RT1 class I, locus CE5 /// RT1 class Ib, locus EC2 | uniqPf7DR_155 | -0.06 | 0.18 | 0.13 | 0.07 | 0.14 | 0 | -0.0454 | -1.032 | 1.18457 | 0.29112 | 0 | -0.1056 | -1.076 | 1.04229 | 0.1972 | 0 | -0.0411 | -1.0289 | 1.16371 | 0.26976 | 1 | 0 |
| 1371233_at | Vcan | versican | uniqPf7DR_155 | -0.01 | -0.08 | -0.24 | -0.01 | -0.02 | 0 | -0.1569 | -1.1149 | 2.01613 | 0.0918 | 0 | 0.07136 | 1.05071 | 1.13457 | 0.22641 | 0 | 0.06016 | 1.04258 | 1.35381 | 0.19811 | 1 | 0 |
| 1371262_at | IgG-2a /// Igh | gamma-2a immunoglobulin heavy chain /// immuno | uniqPf7DR_155 | 0.07 | 0.20 | 0.16 | 0.15 | 0.16 | 0 | -0.0414 | -1.0291 | 1.10254 | 0.33779 | 0 | -0.0476 | -1.0335 | 1.01059 | 0.25019 | 0 | -0.0435 | -1.0306 | 1.11763 | 0.3022 | 1 | 0 |
| 1371694_at | Dpysl2 | dihydropyrimidinase-like 2 | uniqPf7DR_155 | 0.14 | 0.58 | 0.47 | 0.57 | 0.70 | 0 | -0.1051 | -1.0755 | 2.0921 | 0.08416 | 0 | -0.0065 | -1.0045 | 1.08717 | 0.14815 | 0 | 0.12445 | 1.09009 | 2.04942 | 0.06135 | 1 | 0 |
| 1371998_at | LOC10091052 | MOB kinase activator 1A-like /// similar to Mob4B p | uniqPf7DR_155 | 0.00 | 0.05 | 0.07 | 0.10 | 0.11 | 0 | 0.01914 | 1.01335 | 0.90123 | 0.59799 | 0 | 0.04975 | 1.03508 | 0.98138 | 0.53839 | 0 | 0.06462 | 1.04581 | 1.01735 | 0.41265 | 1 | 0 |
| 1372004_at | Hebp1 | heme binding protein 1 | uniqPf7DR_155 | 0.02 | -0.22 | -0.18 | -0.14 | -0.04 | 0 | 0.04158 | 1.02924 | 0.99794 | 0.4487 | 0 | 0.07851 | 1.05593 | 1.07297 | 0.30199 | 0 | 0.17806 | 1.13137 | 2.03792 | 0.06206 | 1 | 0 |
| 1372374_at | Ca1 | carbonic anhydrase I | uniqPf7DR_155 | -0.05 | -0.40 | -0.47 | -0.19 | -0.32 | 0 | -0.0636 | -1.045 | 1.21097 | 0.2762 | 0 | 0.20975 | 1.15649 | 1.29172 | 0.07518 | 0 | 0.08385 | 1.05984 | 1.45092 | 0.16632 | 1 | 0 |
| 1372779_at | B3gnt2 | UDP-GlcNAc:betaGal beta-1,3-N-acetylglucosaminyl | uniqPf7DR_155 | -0.03 | 0.07 | 0.05 | 0.15 | 0.06 | 0 | -0.0252 | -1.0176 | 0.90406 | 0.55213 | 0 | 0.07412 | 1.05272 | 1.01345 | 0.43534 | 0 | -0.014 | -1.0097 | 0.92315 | 0.57237 | 1 | 0 |
| 1372841_at | Reep6 | receptor accessory protein 6 | uniqPf7DR_155 | 0.02 | 0.61 | 0.63 | 0.47 | 0.76 | 0 | 0.01906 | 1.0133 | 1.30037 | 0.2304 | 0 | -0.1376 | -1.1 | 1.0872 | 0.14833 | 0 | 0.15885 | 1.1164 | 1.81057 | 0.08634 | 1 | 0 |
| 1373198_at | Dnajc22 | DnaJ (Hsp40) homolog, subfamily C, member 22 | uniqPf7DR_155 | 0.01 | -0.11 | 0.09 | -0.20 | 0.10 | 0 | 0.19226 | 1.14255 | 1.86325 | 0.06285 | 0 | -0.0987 | -1.0708 | 1.12083 | 0.13101 | 0 | 0.20998 | 1.15667 | 2.07515 | 0.00867 | 1 | 0 |
| 1373386_at | Gjb2 | gap junction protein, beta 2 | uniqPf7DR_155 | 0.21 | -0.08 | 0.00 | 0.12 | 0.03 | 0 | 0.08574 | 1.06123 | 1.29778 | 0.23202 | 0 | 0.1988 | 1.14774 | 1.2224 | 0.03239 | 0 | 0.11336 | 1.08175 | 1.4727 | 0.15864 | 1 | 0 |
| 1373410_at | Mef2c | myocyte enhancer factor 2C | uniqPf7DR_155 | 0.00 | 0.37 | 0.39 | 0.38 | 0.34 | 0 | 0.02061 | 1.01439 | 1.04585 | 0.40154 | 0 | 0.00742 | 1.00515 | 0.96659 | 0.3938 | 0 | -0.0319 | -1.0223 | 1.04833 | 0.36561 | 1 | 0 |
| 1373871_at | Supt7l | suppressor of Ty 7 (S. cerevisiae)-like | uniqPf7DR_155 | -0.10 | -0.13 | -0.36 | -0.16 | -0.22 | 0 | -0.2251 | -1.1689 | 2.43529 | 0.05716 | 0 | -0.0216 | -1.0151 | 0.9626 | 0.60085 | 0 | -0.0823 | -1.0587 | 1.13973 | 0.28413 | 1 | 0 |
| 1375142_at | Sv2b | synaptic vesicle glycoprotein 2b | uniqPf7DR_155 | 0.12 | 0.08 | -0.07 | 0.10 | 0.06 | 0 | -0.1452 | -1.1059 | 1.53872 | 0.16719 | 0 | 0.02556 | 1.01787 | 1.03729 | 0.37344 | 0 | -0.0163 | -1.0113 | 0.99427 | 0.44088 | 1 | 0 |
| 1376132_at | Gtdc1 | glycosyltransferase-like domain containing 1 | uniqPf7DR_155 | -0.06 | -0.06 | 0.01 | -0.06 | -0.04 | 0 | 0.07487 | 1.05327 | 1.07917 | 0.36648 | 0 | 0.00815 | 1.00566 | 0.97452 | 0.5962 | 0 | 0.02648 | 1.01852 | 0.94414 | 0.50445 | 1 | 0 |
| 1376382_at | B3gnt2 | UDP-GlcNAc:betaGal beta-1,3-N-acetylglucosaminyl | uniqPf7DR_155 | -0.07 | -0.11 | 0.15 | -0.05 | -0.10 | 0 | 0.25659 | 1.19465 | 2.23108 | 0.02131 | 0 | 0.05831 | 1.04125 | 1.18834 | 0.17165 | 0 | 0.01427 | 1.00994 | 1.24605 | 0.24894 | 1 | 0 |
| 1376394_at | Clec9a | C-type lectin domain family 9, member A | uniqPf7DR_155 | 0.02 | -0.07 | -0.11 | 0.09 | 0.09 | 0 | -0.0459 | -1.0323 | 0.96239 | 0.46048 | 0 | 0.15327 | 1.11208 | 1.1766 | 0.18543 | 0 | 0.15611 | 1.11428 | 1.7554 | 0.09393 | 1 | 0 |
| 1376644_at | Med19 | mediator complex subunit 19 | uniqPf7DR_155 | 0.06 | -0.07 | -0.18 | -0.07 | -0.13 | 0 | -0.116 | -1.0837 | 1.50829 | 0.17504 | 0 | -0.0033 | -1.0023 | 0.96885 | 0.36705 | 0 | -0.058 | -1.041 | 1.19026 | 0.25329 | 1 | 0 |
| 1376775_at | Prr5l | proline rich 5 like | uniqPf7DR_155 | 0.01 | 0.09 | 0.01 | 0.27 | -0.02 | 0 | -0.077 | -1.0549 | 1.21153 | 0.27582 | 0 | 0.18454 | 1.13645 | 1.26336 | 0.09831 | 0 | -0.1108 | -1.0798 | 1.36365 | 0.17066 | 1 | 0 |
| 1376827_at | Eml4 | echinoderm microtubule associated protein like 4 | uniqPf7DR_155 | -0.07 | 0.23 | 0.22 | 0.28 | 0.25 | 0 | -0.0103 | -1.0072 | 0.91486 | 0.53308 | 0 | 0.04413 | 1.03106 | 1.01036 | 0.44409 | 0 | 0.02017 | 1.01408 | 0.93869 | 0.51303 | 1 | 0 |
| 1377637_at | B3gnt5 | UDP-GlcNAc:betaGal beta-1,3-N-acetylglucosaminyl | uniqPf7DR_155 | -0.12 | -0.11 | -0.09 | 0.05 | -0.14 | 0 | 0.01995 | 1.01393 | 0.97438 | 0.47821 | 0 | 0.16064 | 1.11779 | 1.13067 | 0.22992 | 0 | -0.0308 | -1.0216 | 1.00774 | 0.41824 | 1 | 0 |
| 1377698_at | Cd40 | CD40 molecule, TNF receptor superfamily member 5 | uniqPf7DR_155 | -0.06 | -0.28 | -0.24 | -0.15 | -0.24 | 0 | 0.03626 | 1.02545 | 0.99805 | 0.44869 | 0 | 0.13042 | 1.09461 | 1.08766 | 0.28591 | 0 | 0.04208 | 1.02959 | 0.96448 | 0.471 | 1 | 0 |
| 1378016_at | Eml4 | echinoderm microtubule associated protein like 4 | uniqPf7DR_155 | -0.08 | 0.23 | 0.20 | 0.23 | 0.21 | 0 | -0.0266 | -1.0186 | 0.91978 | 0.52441 | 0 | 0.00527 | 1.00366 | 0.96093 | 0.58041 | 0 | -0.0219 | -1.0153 | 0.93965 | 0.52963 | 1 | 0 |
| 1378140_at | Arl11 | ADP-ribosylation factor-like 11 | uniqPf7DR_155 | 0.10 | -0.06 | 0.09 | -0.03 | -0.02 | 0 | 0.14705 | 1.1073 | 2.05081 | 0.03568 | 0 | 0.03038 | 1.02128 | 1.21173 | 0.14545 | 0 | 0.03614 | 1.02537 | 1.98559 | 0.06398 | 1 | 0 |
| 1378225_at | --- | --- | uniqPf7DR_155 | 0.04 | -0.06 | 0.02 | -0.26 | -0.08 | 0 | 0.08641 | 1.06173 | 1.12091 | 0.3338 | 0 | -0.2006 | -1.1492 | 1.16407 | 0.10608 | 0 | -0.0184 | -1.0128 | 0.98427 | 0.45807 | 1 | 0 |
| 1379232_at | LOC10091186 | TBC1 domain family member 12-like /// TBC1 doma | uniqPf7DR_155 | -0.06 | 0.07 | 0.22 | 0.25 | 0.03 | 0 | 0.14926 | 1.109 | 2.16979 | 0.02281 | 0 | 0.18228 | 1.13468 | 1.07556 | 0.01823 | 0 | -0.0408 | -1.0287 | 1.75221 | 0.0817 | 1 | 0 |
| 1379258_at | Klhl5 | kelch-like family member 5 | uniqPf7DR_155 | -0.12 | -0.04 | 0.05 | 0.02 | 0.01 | 0 | 0.09527 | 1.06827 | 1.08727 | 0.35993 | 0 | 0.06822 | 1.04842 | 1.01288 | 0.4362 | 0 | 0.05459 | 1.03856 | 1.0228 | 0.40726 | 1 | 0 |
| 1379457_at | Nedd1 | neural precursor cell expressed, developmentally do | uniqPf7DR_155 | -0.21 | -0.12 | -0.16 | -0.06 | -0.15 | 0 | -0.0362 | -1.0254 | 1.31006 | 0.23616 | 0 | 0.06419 | 1.0455 | 1.12246 | 0.24038 | 0 | -0.0299 | -1.0209 | 1.11532 | 0.30401 | 1 | 0 |
| 1379603_at | Cbl | Cbl proto-oncogene, E3 ubiquitin protein ligase | uniqPf7DR_155 | 0.07 | 0.20 | 0.22 | 0.27 | 0.08 | 0 | 0.01044 | 1.00726 | 1.66023 | 0.11563 | 0 | 0.06452 | 1.04574 | 1.29366 | 0.06564 | 0 | -0.1248 | -1.0903 | 2.07003 | 0.04673 | 1 | 0 |
| 1379689_at | Rc3h2 | ring finger and CCCH-type domains 2 | uniqPf7DR_155 | 0.03 | 0.06 | 0.08 | 0.33 | 0.07 | 0 | 0.02463 | 1.01722 | 0.98924 | 0.45962 | 0 | 0.26984 | 1.20568 | 1.27456 | 0.05139 | 0 | 0.01583 | 1.01103 | 0.97578 | 0.45826 | 1 | 0 |
| 1379780_at | Fam65b | family with sequence similarity 65, member B | uniqPf7DR_155 | -0.05 | -0.13 | -0.17 | -0.15 | -0.16 | 0 | -0.0392 | -1.0275 | 0.9337 | 0.49776 | 0 | -0.0214 | -1.015 | 0.96258 | 0.60078 | 0 | -0.0329 | -1.0231 | 0.95621 | 0.5017 | 1 | 0 |
| 1380069_at | --- | --- | uniqPf7DR_155 | 0.01 | 0.21 | 0.11 | 0.35 | 0.11 | 0 | -0.102 | -1.0733 | 1.4174 | 0.1958 | 0 | 0.14082 | 1.10253 | 1.24183 | 0.11596 | 0 | -0.1046 | -1.0752 | 1.44058 | 0.14439 | 1 | 0 |
| 1380435_at | Shoc2 | soc-2 (suppressor of clear) homolog (C. elegans) | uniqPf7DR_155 | 0.07 | 0.16 | 0.30 | 0.22 | 0.25 | 0 | 0.14475 | 1.10554 | 1.85309 | 0.06578 | 0 | 0.06453 | 1.04574 | 1.17132 | 0.18825 | 0 | 0.08658 | 1.06185 | 1.64052 | 0.1188 | 1 | 0 |
| 1380706_at | Fnip2 | folliculin interacting protein 2 | uniqPf7DR_155 | 0.01 | -0.04 | 0.20 | -0.28 | -0.12 | 0 | 0.23738 | 1.17885 | 1.71187 | 0.10117 | 0 | -0.2349 | -1.1768 | 1.20397 | 0.08788 | 0 | -0.0779 | -1.0555 | 1.06107 | 0.35006 | 1 | 0 |
| 1380783_at | Slco5a1 | solute carrier organic anion transporter family, men | uniqPf7DR_155 | -0.05 | -0.10 | 0.06 | 0.15 | -0.07 | 0 | 0.16117 | 1.11819 | 1.76703 | 0.08273 | 0 | 0.24735 | 1.18703 | 1.27811 | 0.04924 | 0 | 0.03456 | 1.02425 | 1.06171 | 0.36949 | 1 | 0 |
| 1380784_at | Klhl5 | kelch-like family member 5 | uniqPf7DR_155 | -0.09 | 0.28 | 0.22 | 0.39 | 0.34 | 0 | -0.0604 | -1.0427 | 1.33193 | 0.22704 | 0 | 0.11276 | 1.08129 | 1.21283 | 0.14636 | 0 | 0.06625 | 1.04699 | 1.44195 | 0.16891 | 1 | 0 |
| 1380909_at | Slc25a24 | solute carrier family 25 (mitochondrial carrier, phos | uniqPf7DR_155 | -0.18 | -0.13 | -0.12 | -0.04 | -0.14 | 0 | 0.008 | 1.00556 | 1.14505 | 0.3169 | 0 | 0.08639 | 1.06171 | 1.09349 | 0.27785 | 0 | -0.0161 | -1.0112 | 1.06207 | 0.34944 | 1 | 0 |
| 1382153_at | Clec4a | C-type lectin domain family 4, member A | uniqPf7DR_155 | 0.01 | -0.10 | -0.06 | 0.01 | -0.15 | 0 | 0.03534 | 1.0248 | 1.12044 | 0.33445 | 0 | 0.10629 | 1.07645 | 1.14942 | 0.21537 | 0 | -0.051 | -1.036 | 1.10156 | 0.31567 | 1 | 0 |
| 1382337_at | Stap1 | signal transducing adaptor family member 1 | uniqPf7DR_155 | -0.16 | 0.25 | 0.22 | 0.15 | 0.03 | 0 | -0.0328 | -1.023 | 1.23672 | 0.26234 | 0 | -0.1036 | -1.0744 | 1.08719 | 0.14844 | 0 | -0.2216 | -1.166 | 1.8153 | 0.07524 | 1 | 0 |
| 1382451_at | Hebp2 | heme binding protein 2 | uniqPf7DR_155 | -0.09 | -0.20 | -0.31 | -0.24 | -0.35 | 0 | -0.1081 | -1.0778 | 1.18677 | 0.28918 | 0 | -0.0411 | -1.0289 | 0.965 | 0.40897 | 0 | -0.1529 | -1.1118 | 1.29175 | 0.19863 | 1 | 0 |
| 1382569_at | Paqr9 | progestin and adipoQ receptor family member IX | uniqPf7DR_155 | 0.27 | 0.22 | 0.31 | 0.27 | 0.26 | 0 | 0.08511 | 1.06077 | 1.95363 | 0.05019 | 0 | 0.04932 | 1.03478 | 1.25647 | 0.10585 | 0 | 0.03502 | 1.02457 | 2.05448 | 0.06111 | 1 | 0 |
| 1382622_at | Cst7 | cystatin F (leukocystatin) | uniqPf7DR_155 | -0.04 | -0.21 | -0.09 | -0.13 | -0.08 | 0 | 0.1222 | 1.08839 | 1.303 | 0.22776 | 0 | 0.07979 | 1.05686 | 1.06107 | 0.3214 | 0 | 0.13549 | 1.09846 | 1.39714 | 0.18375 | 1 | 0 |
| 1382776_at | Mboat1 | membrane bound O-acyltransferase domain contair | uniqPf7DR_155 | 0.15 | 0.00 | -0.10 | 0.13 | 0.06 | 0 | -0.1059 | -1.0762 | 1.16152 | 0.3029 | 0 | 0.12226 | 1.08844 | 1.11001 | 0.2602 | 0 | 0.0556 | 1.03929 | 1.15385 | 0.29712 | 1 | 0 |
| 1382778_at | Dusp6 | dual specificity phosphatase 6 | uniqPf7DR_155 | 0.08 | -0.25 | -0.14 | -0.04 | -0.02 | 0 | 0.10638 | 1.07652 | 1.07054 | 0.37515 | 0 | 0.20894 | 1.15584 | 1.25309 | 0.10605 | 0 | 0.2281 | 1.1713 | 2.29825 | 0.04175 | 1 | 0 |
| 1383085_at | Sh3bgrl | SH3 domain binding glutamic acid-rich protein like | uniqPf7DR_155 | -0.15 | -0.08 | 0.03 | 0.07 | -0.10 | 0 | 0.1176 | 1.08493 | 1.26152 | 0.24907 | 0 | 0.15562 | 1.1139 | 1.14303 | 0.22126 | 0 | -0.0135 | -1.0094 | 0.94642 | 0.51775 | 1 | 0 |
| 1383245_at | Mbip | MAP3K12 binding inhibitory protein 1 | uniqPf7DR_155 | -0.19 | -0.33 | -0.47 | -0.37 | -0.45 | 0 | -0.1395 | -1.1015 | 2.23117 | 0.07217 | 0 | -0.0353 | -1.0248 | 0.97136 | 0.35667 | 0 | -0.1173 | -1.0847 | 1.49326 | 0.12748 | 1 | 0 |
| 1383597_at | Dclre1c | DNA cross-link repair 1C | uniqPf7DR_155 | -0.06 | -0.18 | -0.13 | -0.06 | -0.06 | 0 | 0.05786 | 1.04092 | 1.23233 | 0.26264 | 0 | 0.12859 | 1.09323 | 1.1277 | 0.23632 | 0 | 0.12141 | 1.0878 | 1.33667 | 0.20631 | 1 | 0 |

| Affymetrix Probeset ID | Gene Symbol | Gene Title | Figure 6 B Venn n =2021 | Mean BN log2 intensity autologous n=8 | Mean log2 intensity day 30 DR +/+ ND n=4 | Mean log2 intensity day 30 DR+/+ HCD n=4 | Mean log2 intensity day 30 DR+/+ B/S n=4 | Mean log2 intensity day 30 F+/+ ND n=4 | DR+/+ HCD vs DR+/+ ND n=380 1=yes 0=no | Log2 ratio DR+/+ HCD vs DR+/+ ND | Fold-Change DR+/+ HCD vs DR+/+ ND | RP-FDR 4DR30 HCD vs 4DR30 Reg | p value DR+/+ HCD vs DR+/+ ND | DR+/+ B/S vs DR+/+ ND n=1,330 1=yes 0=no | Log2 ratio DR+/+ B/S vs DR+/+ ND | Fold-Change DR+/+ B/S vs DR+/+ ND | RP-FDR 4DR30 Antib vs 4DR30 Reg | p value DR+/+ B/S vs DR+/+ ND | DR+/+ ND vs F+/+ ND n=398 1=yes 0=no | Log2 ratio DR+/+ ND vs F+/+ ND | Fold-Change DR+/+ ND vs F+/+ ND | RP-FDR 4RT1uu 30Reg vs 4DR30 Reg | p value DR+/+ ND vs F+/+ ND | DR+/+ Longitudinal n=228 1=yes 0=no | Figure 6F |
|---|---|---|---|---|---|---|---|---|---|---|---|---|---|---|---|---|---|---|---|---|---|---|---|---|---|
| 1383653_at | Snx11 | sorting nexin 11 | uniqPf7DR_155 | -0.03 | 0.04 | 0.07 | 0.00 | 0.06 | 0 | 0.03607 | 1.02532 | 0.91368 | 0.57305 | 0 | -0.0367 | -1.0258 | 0.96182 | 0.55422 | 0 | 0.02688 | 1.01881 | 0.90996 | 0.58142 | 1 | 0 |
| 1383786_at | Rab32 | RAB32, member RAS oncogene family | uniqPf7DR_155 | 0.17 | -0.06 | 0.08 | 0.19 | 0.10 | 0 | 0.14292 | 1.10414 | 1.67727 | 0.11245 | 0 | 0.25154 | 1.19048 | 1.28652 | 0.05928 | 0 | 0.16289 | 1.11953 | 1.71442 | 0.10099 | 1 | 0 |
| 1384192_at | Chst1 | carbohydrate (keratan sulfate Gal-6) sulfotransferas | uniqPf7DR_155 | -0.02 | 0.21 | 0.32 | 0.35 | 0.25 | 0 | 0.10913 | 1.07858 | 1.27904 | 0.24138 | 0 | 0.14627 | 1.1067 | 1.18497 | 0.17409 | 0 | 0.047 | 1.03311 | 1.13448 | 0.31041 | 1 | 0 |
| 1384376_at | Dnajb14 | DnaJ (Hsp40) homolog, subfamily B, member 14 | uniqPf7DR_155 | -0.15 | -0.01 | 0.10 | 0.07 | -0.20 | 0 | 0.11647 | 1.08408 | 1.52238 | 0.1517 | 0 | 0.08368 | 1.05971 | 1.14504 | 0.21871 | 0 | -0.1812 | -1.1338 | 2.15218 | 0.04228 | 1 | 0 |
| 1384639_at | Reep6 | receptor accessory protein 6 | uniqPf7DR_155 | 0.08 | 0.42 | 0.19 | 0.37 | 0.43 | 0 | -0.2288 | -1.1719 | 3.07136 | 0.02825 | 0 | -0.0456 | -1.0321 | 1.00164 | 0.26478 | 0 | 0.01277 | 1.00889 | 1.73957 | 0.08323 | 1 | 0 |
| 1385019_at | Bank1 | B-cell scaffold protein with ankyrin repeats 1 | uniqPf7DR_155 | -0.06 | 0.05 | 0.05 | 0.17 | -0.07 | 0 | 0.00356 | 1.00247 | 0.94351 | 0.5191 | 0 | 0.12884 | 1.09341 | 1.10748 | 0.2626 | 0 | -0.1163 | -1.0839 | 1.18874 | 0.25434 | 1 | 0 |
| 1385046_at | Nxpe3 | neurexophilin and PC-esterase domain family, mem | uniqPf7DR_155 | -0.13 | 0.29 | 0.33 | 0.45 | 0.24 | 0 | 0.03772 | 1.02649 | 0.98629 | 0.46373 | 0 | 0.15494 | 1.11338 | 1.19963 | 0.16116 | 0 | -0.055 | -1.0389 | 1.0135 | 0.40668 | 1 | 0 |
| 1385244_at | Adam4 | a disintegrin and metalloprotease domain 4 | uniqPf7DR_155 | -0.11 | 0.28 | 0.24 | 0.11 | 0.30 | 0 | -0.0371 | -1.026 | 1.06032 | 0.36676 | 0 | -0.173 | -1.1274 | 1.14288 | 0.1176 | 0 | 0.01657 | 1.01155 | 0.93247 | 0.52448 | 1 | 0 |
| 1385311_at | Slco5a1 | solute carrier organic anion transporter family, men | uniqPf7DR_155 | 0.00 | -0.08 | -0.03 | -0.02 | -0.04 | 0 | 0.0437 | 1.03075 | 1.42055 | 0.18249 | 0 | 0.05306 | 1.03746 | 1.18747 | 0.17278 | 0 | 0.03452 | 1.02422 | 1.21475 | 0.26354 | 1 | 0 |
| 1385465_at | Siglec5 | sialic acid binding Ig-like lectin 5 | uniqPf7DR_155 | -0.07 | -0.22 | -0.21 | -0.29 | -0.05 | 0 | 0.01197 | 1.00833 | 2.11308 | 0.02562 | 0 | -0.074 | -1.0526 | 2.12254 | 0.01317 | 0 | 0.17276 | 1.12722 | 2.05611 | 0.00833 | 1 | 0 |
| 1385481_at | Dsel | dermatan sulfate epimerase-like | uniqPf7DR_155 | 0.06 | -0.19 | -0.14 | -0.08 | -0.10 | 0 | 0.05248 | 1.03705 | 1.29149 | 0.23493 | 0 | 0.1117 | 1.0805 | 1.13457 | 0.22652 | 0 | 0.09686 | 1.06945 | 1.35056 | 0.19968 | 1 | 0 |
| 1385605_at | Gpr84 | G protein-coupled receptor 84 | uniqPf7DR_155 | -0.03 | -0.06 | 0.00 | -0.11 | -0.09 | 0 | 0.06616 | 1.04693 | 1.05278 | 0.38812 | 0 | -0.0467 | -1.0329 | 0.96308 | 0.46533 | 0 | -0.027 | -1.0189 | 1.02722 | 0.38818 | 1 | 0 |
| 1385702_at | Ifi204 | interferon activated gene 204 | uniqPf7DR_155 | -0.12 | -0.18 | 0.09 | 0.01 | 0.01 | 0 | 0.26667 | 1.20303 | 1.88772 | 0.06149 | 0 | 0.19272 | 1.14292 | 1.23832 | 0.11898 | 0 | 0.19346 | 1.1435 | 1.79376 | 0.08848 | 1 | 0 |
| 1385813_at | Mzb1 | marginal zone B and B1 cell-specific protein | uniqPf7DR_155 | -0.05 | 0.05 | 0.05 | 0.09 | 0.03 | 0 | 0.00186 | 1.00129 | 0.92333 | 0.55592 | 0 | 0.04232 | 1.02977 | 0.99735 | 0.48721 | 0 | -0.0105 | -1.0073 | 0.96243 | 0.49296 | 1 | 0 |
| 1386212_at | Spta1 | spectrin, alpha, erythrocytic 1 (elliptocytosis 2) | uniqPf7DR_155 | 0.01 | -0.15 | -0.20 | -0.03 | -0.27 | 0 | -0.0534 | -1.0377 | 2.5509 | 0.04725 | 0 | 0.11955 | 1.0864 | 1.29238 | 0.0576 | 0 | -0.1248 | -1.0904 | 2.56773 | 0.02799 | 1 | 0 |
| 1386344_at | --- | --- | uniqPf7DR_155 | 0.06 | 0.62 | 0.61 | 0.53 | 0.52 | 0 | -0.0103 | -1.0072 | 0.92588 | 0.51229 | 0 | -0.0881 | -1.063 | 1.06276 | 0.16796 | 0 | -0.0934 | -1.0669 | 1.21182 | 0.23914 | 1 | 0 |
| 1386547_at | Tanc2 | tetratricopeptide repeat, ankyrin repeat and coiled- | uniqPf7DR_155 | 0.09 | 0.11 | 0.15 | 0.24 | 0.06 | 0 | 0.03913 | 1.02749 | 1.22373 | 0.26691 | 0 | 0.12675 | 1.09183 | 1.23543 | 0.12494 | 0 | -0.0558 | -1.0395 | 1.38073 | 0.16298 | 1 | 0 |
| 1386938_at | Anpep | alanyl (membrane) aminopeptidase | uniqPf7DR_155 | 0.12 | -0.09 | -0.12 | 0.14 | 0.12 | 0 | -0.0288 | -1.0202 | 1.14685 | 0.31025 | 0 | 0.22949 | 1.17242 | 1.29219 | 0.05734 | 0 | 0.21027 | 1.1569 | 2.08327 | 0.05527 | 1 | 0 |
| 1386952_a_at | Dync1i2 | dynein cytoplasmic 1 intermediate chain 2 | uniqPf7DR_155 | -0.07 | -0.04 | -0.06 | -0.08 | -0.06 | 0 | -0.0194 | -1.0135 | 0.92736 | 0.51006 | 0 | -0.0372 | -1.0261 | 0.96278 | 0.53809 | 0 | -0.0166 | -1.0116 | 0.95692 | 0.50069 | 1 | 0 |
| 1386953_at | Hsd11b1 | hydroxysteroid 11-beta dehydrogenase 1 | uniqPf7DR_155 | 0.02 | -0.11 | -0.13 | -0.08 | -0.06 | 0 | -0.0173 | -1.0121 | 1.09733 | 0.34234 | 0 | 0.03406 | 1.02389 | 1.04143 | 0.36565 | 0 | 0.05449 | 1.03849 | 1.18479 | 0.27925 | 1 | 0 |
| 1387020_at | Cyp51 | cytochrome P450, family 51 | uniqPf7DR_155 | -0.09 | -0.14 | -0.21 | 0.08 | -0.01 | 0 | -0.0624 | -1.0442 | 1.08214 | 0.35124 | 0 | 0.21987 | 1.16463 | 1.27528 | 0.09054 | 0 | 0.13437 | 1.09761 | 1.233 | 0.25438 | 1 | 0 |
| 1387069_a_at | Tbxa2r | thromboxane A2 receptor | uniqPf7DR_155 | -0.02 | 0.39 | 0.56 | 0.45 | 0.54 | 0 | 0.17228 | 1.12684 | 1.82146 | 0.07105 | 0 | 0.05819 | 1.04116 | 1.1117 | 0.25706 | 0 | 0.1564 | 1.1145 | 1.77532 | 0.09071 | 1 | 0 |
| 1387081_at | Rcn2 | reticulocalbin 2, EF-hand calcium binding domain | uniqPf7DR_155 | 0.03 | 0.05 | 0.05 | 0.16 | 0.03 | 0 | 0.00453 | 1.00314 | 0.91228 | 0.57655 | 0 | 0.11289 | 1.0814 | 1.04801 | 0.35223 | 0 | -0.0161 | -1.0112 | 0.97611 | 0.4704 | 1 | 0 |
| 1387135_at | Adam15 | ADAM metallopeptidase domain 15 | uniqPf7DR_155 | 0.12 | 0.14 | 0.09 | 0.39 | 0.22 | 0 | -0.0508 | -1.0358 | 1.3716 | 0.21369 | 0 | 0.25035 | 1.18949 | 1.26909 | 0.0466 | 0 | 0.08084 | 1.05763 | 1.07206 | 0.35907 | 1 | 0 |
| 1387202_at | Icam1 | intercellular adhesion molecule 1 | uniqPf7DR_155 | 0.12 | 0.26 | 0.21 | 0.48 | 0.45 | 0 | -0.0498 | -1.0351 | 1.07143 | 0.3603 | 0 | 0.21965 | 1.16445 | 1.29143 | 0.07134 | 0 | 0.18155 | 1.1341 | 1.89644 | 0.07476 | 1 | 0 |
| 1387365_at | Nr1h3 | nuclear receptor subfamily 1, group H, member 3 | uniqPf7DR_155 | 0.14 | -0.08 | 0.10 | -0.08 | -0.05 | 0 | 0.18027 | 1.1331 | 1.51604 | 0.15336 | 0 | -0.0062 | -1.0043 | 0.96243 | 0.53923 | 0 | 0.02427 | 1.01696 | 1.14856 | 0.30019 | 1 | 0 |
| 1387367_at | Glg1 | golgi glycoprotein 1 | uniqPf7DR_155 | 0.10 | 0.15 | 0.30 | 0.16 | 0.31 | 0 | 0.15069 | 1.1101 | 1.28222 | 0.23839 | 0 | 0.01011 | 1.00704 | 0.97111 | 0.63074 | 0 | 0.15446 | 1.11301 | 1.39293 | 0.18552 | 1 | 0 |
| 1387432_at | Ptbp3 | polypyrimidine tract binding protein 3 | uniqPf7DR_155 | -0.14 | -0.45 | -0.54 | -0.22 | -0.30 | 0 | -0.0868 | -1.062 | 2.75002 | 0.03776 | 0 | 0.23357 | 1.17574 | 1.05733 | 0.01758 | 0 | 0.1512 | 1.11049 | 2.40316 | 0.02666 | 1 | 0 |
| 1387756_s_at | Hemgn | hemogen | uniqPf7DR_155 | -0.06 | -0.20 | -0.37 | -0.11 | -0.19 | 0 | -0.1726 | -1.1271 | 2.30748 | 0.0673 | 0 | 0.08395 | 1.05992 | 1.12177 | 0.24279 | 0 | 0.00246 | 1.00171 | 1.10862 | 0.32832 | 1 | 0 |
| 1387809_at | Map2k6 | mitogen-activated protein kinase kinase 6 | uniqPf7DR_155 | 0.06 | 0.19 | 0.09 | 0.13 | 0.22 | 0 | -0.1013 | -1.0727 | 1.12453 | 0.3245 | 0 | -0.0647 | -1.0459 | 0.96255 | 0.48922 | 0 | 0.03253 | 1.0228 | 0.9246 | 0.54586 | 1 | 0 |
| 1388161_at | Adam10 | ADAM metallopeptidase domain 10 | uniqPf7DR_155 | 0.04 | 0.08 | 0.08 | 0.09 | 0.13 | 0 | -0.0047 | -1.0033 | 0.88784 | 0.59187 | 0 | 0.00476 | 1.0033 | 0.97885 | 0.56693 | 0 | 0.04686 | 1.03301 | 0.95507 | 0.48385 | 1 | 0 |
| 1388181_at | Igd | immunoglobulin delta heavy chain constant region | uniqPf7DR_155 | 0.07 | 0.50 | 0.35 | 0.28 | 0.48 | 0 | -0.1545 | -1.113 | 1.40297 | 0.2026 | 0 | -0.2224 | -1.1667 | 1.20502 | 0.0878 | 0 | -0.0274 | -1.0192 | 0.9865 | 0.45475 | 1 | 0 |
| 1388277_at | LOC1003617( | lambda-chain C1-region-like /// Ig lambda chain V-V | uniqPf7DR_155 | 0.02 | 0.15 | 0.11 | 0.20 | 0.10 | 0 | -0.0362 | -1.0254 | 0.91617 | 0.53101 | 0 | 0.05204 | 1.03673 | 0.99135 | 0.50069 | 0 | -0.0491 | -1.0346 | 0.97431 | 0.47351 | 1 | 0 |
| 1388365_at | Atp6v0d1 | ATPase, H+ transporting, lysosomal V0 subunit D1 | uniqPf7DR_155 | 0.00 | -0.07 | -0.09 | 0.00 | -0.03 | 0 | -0.025 | -1.0175 | 0.90339 | 0.55579 | 0 | 0.06927 | 1.04918 | 0.99953 | 0.48197 | 0 | 0.03492 | 1.0245 | 0.91786 | 0.56124 | 1 | 0 |
| 1388416_at | Lrp1 | low density lipoprotein receptor-related protein 1 | uniqPf7DR_155 | 0.08 | -0.02 | -0.15 | 0.14 | 0.05 | 0 | -0.1301 | -1.0944 | 1.50821 | 0.17507 | 0 | 0.15604 | 1.11422 | 1.19841 | 0.15977 | 0 | 0.07028 | 1.04992 | 1.47541 | 0.1559 | 1 | 0 |
| 1389074_at | Fchsd2 | FCH and double SH3 domains 2 | uniqPf7DR_155 | -0.02 | 0.67 | 0.77 | 0.65 | 0.64 | 0 | 0.0978 | 1.07014 | 1.06911 | 0.37709 | 0 | -0.0176 | -1.0123 | 0.96319 | 0.47814 | 0 | -0.0236 | -1.0165 | 0.98939 | 0.44988 | 1 | 0 |
| 1389176_at | Inpp5f | inositol polyphosphate-5-phosphatase F | uniqPf7DR_155 | -0.08 | 0.11 | 0.15 | 0.19 | 0.13 | 0 | 0.03698 | 1.02596 | 0.93522 | 0.52978 | 0 | 0.07731 | 1.05505 | 1.01804 | 0.42563 | 0 | 0.01956 | 1.01365 | 0.90914 | 0.58599 | 1 | 0 |
| 1389305_at | Anxa4 | annexin A4 | uniqPf7DR_155 | -0.03 | -0.42 | -0.43 | -0.21 | -0.28 | 0 | -0.0151 | -1.0105 | 1.0102 | 0.41455 | 0 | 0.20677 | 1.1541 | 1.27368 | 0.04964 | 0 | 0.14306 | 1.10424 | 1.66763 | 0.10848 | 1 | 0 |
| 1389441_at | Abp10 | annexin V-binding protein ABP-10 | uniqPf7DR_155 | -0.10 | 0.14 | 0.16 | 0.30 | 0.11 | 0 | 0.01831 | 1.01277 | 1.93028 | 0.05772 | 0 | 0.16128 | 1.11828 | 1.20347 | 0.02821 | 0 | -0.0323 | -1.0227 | 1.79276 | 0.0784 | 1 | 0 |
| 1389911_at | Metrnl | meteorin, glial cell differentiation regulator-like | uniqPf7DR_155 | 0.01 | -0.27 | -0.18 | 0.00 | -0.02 | 0 | 0.0844 | 1.06024 | 1.07173 | 0.37457 | 0 | 0.26712 | 1.2034 | 1.29116 | 0.05717 | 0 | 0.24995 | 1.18917 | 2.37228 | 0.02906 | 1 | 0 |
| 1390859_at | N4bp1 | Nedd4 binding protein 1 | uniqPf7DR_155 | 0.06 | 0.13 | 0.04 | 0.03 | 0.11 | 0 | -0.0886 | -1.0633 | 1.31337 | 0.23354 | 0 | -0.1011 | -1.0726 | 1.01644 | 0.23869 | 0 | -0.0239 | -1.0167 | 0.98026 | 0.4636 | 1 | 0 |
| 1391091_at | Cyld /// RGD1 | cylindromatosis (turban tumor syndrome) /// simila | uniqPf7DR_155 | -0.01 | -0.03 | 0.11 | 0.04 | -0.10 | 0 | 0.13716 | 1.09973 | 1.4502 | 0.17464 | 0 | 0.06554 | 1.04648 | 1.05186 | 0.34398 | 0 | -0.0727 | -1.0517 | 1.19726 | 0.24658 | 1 | 0 |
| 1391312_at | Inpp5f | inositol polyphosphate-5-phosphatase F | uniqPf7DR_155 | 0.24 | 0.40 | 0.53 | 0.64 | 0.50 | 0 | 0.12846 | 1.09313 | 1.34043 | 0.21585 | 0 | 0.24183 | 1.18249 | 1.29409 | 0.06317 | 0 | 0.10126 | 1.07271 | 1.30835 | 0.21826 | 1 | 0 |
| 1391315_at | Fnip2 | folliculin interacting protein 2 | uniqPf7DR_155 | 0.11 | -0.04 | -0.07 | 0.08 | -0.24 | 0 | -0.0279 | -1.0195 | 1.07684 | 0.3544 | 0 | 0.1262 | 1.09141 | 1.12753 | 0.23635 | 0 | -0.1941 | -1.144 | 1.51721 | 0.12036 | 1 | 0 |
| 1391501_at | Ift80 | intraflagellar transport 80 homolog (Chlamydomona | uniqPf7DR_155 | -0.19 | -0.09 | -0.09 | 0.03 | -0.16 | 0 | 0.00384 | 1.00266 | 0.92878 | 0.54265 | 0 | 0.12317 | 1.08913 | 1.10168 | 0.26682 | 0 | -0.0621 | -1.044 | 1.05843 | 0.35334 | 1 | 0 |
| 1391820_at | Tanc2 | tetratricopeptide repeat, ankyrin repeat and coiled- | uniqPf7DR_155 | 0.04 | -0.01 | 0.08 | 0.06 | -0.03 | 0 | 0.09096 | 1.06508 | 1.12348 | 0.33257 | 0 | 0.07273 | 1.05171 | 1.03239 | 0.38469 | 0 | -0.0208 | -1.0145 | 0.93684 | 0.53733 | 1 | 0 |
| 1392539_at | Blvrb /// LOC | biliverdin reductase B (flavin reductase (NADPH)) // | uniqPf7DR_155 | -0.03 | -0.36 | -0.32 | -0.11 | -0.29 | 0 | 0.03976 | 1.02794 | 1.48155 | 0.16383 | 0 | 0.24765 | 1.18727 | 1.20576 | 0.02916 | 0 | 0.06802 | 1.04828 | 1.66586 | 0.11072 | 1 | 0 |
| 1392590_at | Arhgap24 | Rho GTPase activating protein 24 | uniqPf7DR_155 | -0.21 | 0.34 | 0.30 | 0.53 | 0.15 | 0 | -0.0407 | -1.0286 | 1.08028 | 0.35251 | 0 | 0.18921 | 1.14014 | 1.19848 | 0.15847 | 0 | -0.193 | -1.1432 | 1.70807 | 0.0915 | 1 | 0 |
| 1392961_at | LOC10091052 | MOB kinase activator 1A-like /// similar to Mob4B p | uniqPf7DR_155 | 0.08 | -0.14 | 0.00 | 0.10 | -0.10 | 0 | 0.14147 | 1.10303 | 1.68023 | 0.10865 | 0 | 0.23319 | 1.17543 | 1.26907 | 0.04628 | 0 | 0.03739 | 1.02626 | 1.09375 | 0.3396 | 1 | 0 |
| 1393092_at | --- | --- | uniqPf7DR_155 | 0.05 | 0.06 | -0.02 | 0.04 | -0.11 | 0 | -0.0719 | -1.0511 | 1.18979 | 0.28713 | 0 | -0.0164 | -1.0114 | 1.02679 | 0.39921 | 0 | -0.1617 | -1.1186 | 1.59757 | 0.1048 | 1 | 0 |
| 1393198_at | Pcmtd1 | protein-L-isoaspartate (D-aspartate) O-methyltransf | uniqPf7DR_155 | -0.26 | -0.18 | -0.12 | -0.10 | -0.29 | 0 | 0.06234 | 1.04415 | 1.61744 | 0.12924 | 0 | 0.08313 | 1.05932 | 1.19829 | 0.16002 | 0 | -0.1021 | -1.0733 | 1.36958 | 0.16687 | 1 | 0 |
| 1393210_at | LOC10036138 | extracellular matrix protein 2-like | uniqPf7DR_155 | -0.01 | 0.08 | 0.08 | 0.00 | 0.01 | 0 | -0.0016 | -1.0011 | 0.91972 | 0.52526 | 0 | -0.0732 | -1.052 | 0.9729 | 0.32454 | 0 | -0.0703 | -1.05 | 1.18527 | 0.25631 | 1 | 0 |
| 1393439_a_at | Ankh | ankylosis, progressive homolog (mouse) | uniqPf7DR_155 | 0.05 | 0.10 | 0.16 | 0.04 | 0.17 | 0 | 0.06087 | 1.04309 | 0.97409 | 0.4787 | 0 | -0.0587 | -1.0415 | 0.96512 | 0.4111 | 0 | 0.06703 | 1.04756 | 1.03063 | 0.39619 | 1 | 0 |
| 1393811_at | Ercc6l2 | excision repair cross-complementing rodent repair ( | uniqPf7DR_155 | -0.18 | -0.06 | -0.31 | -0.18 | -0.32 | 0 | -0.249 | -1.1884 | 3.33748 | 0.0132 | 0 | -0.121 | -1.0875 | 1.29837 | 0.06584 | 0 | -0.2551 | -1.1934 | 3.08494 | 0.00863 | 1 | 0 |
| 1393836_at | Mitf | microphthalmia-associated transcription factor | uniqPf7DR_155 | -0.02 | 0.10 | 0.10 | 0.06 | 0.09 | 0 | -0.0016 | -1.0011 | 0.92917 | 0.5424 | 0 | -0.0399 | -1.028 | 0.96457 | 0.42476 | 0 | -0.0125 | -1.0087 | 0.93213 | 0.5465 | 1 | 0 |
| 1393926_at | Stap1 | signal transducing adaptor family member 1 | uniqPf7DR_155 | -0.12 | 0.22 | 0.01 | 0.26 | 0.07 | 0 | -0.2154 | -1.161 | 2.00713 | 0.0952 | 0 | 0.03357 | 1.02354 | 0.97684 | 0.57384 | 0 | -0.1487 | -1.1086 | 1.29954 | 0.19318 | 1 | 0 |
| 1393978_at | Stfa2l3 | stefin A2-like 3 | uniqPf7DR_155 | 0.01 | 0.02 | 0.07 | 0.03 | -0.07 | 0 | 0.05016 | 1.03538 | 1.05576 | 0.38579 | 0 | 0.01133 | 1.00788 | 0.98405 | 0.53333 | 0 | -0.0825 | -1.0589 | 1.02073 | 0.39747 | 1 | 0 |
| 1393996_at | Dnajb14 | DnaJ (Hsp40) homolog, subfamily B, member 14 | uniqPf7DR_155 | -0.03 | -0.10 | 0.03 | -0.05 | 0.07 | 0 | 0.13058 | 1.09473 | 1.41832 | 0.18288 | 0 | 0.05064 | 1.03572 | 1.07373 | 0.30003 | 0 | 0.16891 | 1.12421 | 1.7528 | 0.09401 | 1 | 0 |
| 1394001_at | Optc | opticin | uniqPf7DR_155 | 0.17 | 0.05 | 0.07 | -0.09 | -0.05 | 0 | 0.02195 | 1.01533 | 0.94571 | 0.51572 | 0 | -0.1436 | -1.1046 | 1.01394 | 0.24306 | 0 | -0.097 | -1.0695 | 1.10211 | 0.3142 | 1 | 0 |
| 1394318_at | Arpp19 | cAMP-regulated phosphoprotein 19 | uniqPf7DR_155 | 0.07 | -0.21 | -0.20 | -0.08 | -0.24 | 0 | 0.00898 | 1.00625 | 0.92313 | 0.556 | 0 | 0.12833 | 1.09303 | 1.14597 | 0.21833 | 0 | -0.0319 | -1.0223 | 1.07543 | 0.33509 | 1 | 0 |
| 1394626_at | Hmbox1 | homeobox containing 1 | uniqPf7DR_155 | -0.01 | -0.11 | -0.15 | -0.12 | -0.02 | 0 | -0.0455 | -1.0321 | 1.34989 | 0.22146 | 0 | -0.0143 | -1.01 | 1.00679 | 0.25627 | 0 | 0.08111 | 1.05783 | 1.35937 | 0.19679 | 1 | 0 |
| 1394939_at | Ppm1k | protein phosphatase, Mg2+/Mn2+ dependent, 1K | uniqPf7DR_155 | 0.00 | -0.08 | -0.04 | -0.03 | -0.10 | 0 | 0.03784 | 1.02657 | 1.04707 | 0.39965 | 0 | 0.04424 | 1.03114 | 1.0438 | 0.36007 | 0 | -0.0247 | -1.0173 | 0.93845 | 0.53168 | 1 | 0 |
| 1395621_at | Mcart2 | mitochondrial carrier triple repeat 2 | uniqPf7DR_155 | 0.14 | 0.51 | 0.65 | 0.50 | 0.59 | 0 | 0.13995 | 1.10187 | 2.33655 | 0.00872 | 0 | -0.0149 | -1.0104 | 1.44995 | 0.04541 | 0 | 0.08159 | 1.05818 | 2.12307 | 0.05134 | 1 | 0 |
| 1396233_at | Arsk | arylsulfatase family, member K | uniqPf7DR_155 | -0.33 | -0.48 | -0.61 | -0.28 | -0.64 | 0 | -0.1316 | -1.0955 | 1.68774 | 0.13866 | 0 | 0.20065 | 1.14921 | 1.23581 | 0.12506 | 0 | -0.1597 | -1.1171 | 1.88628 | 0.06314 | 1 | 0 |
| 1396278_at | Snx11 | sorting nexin 11 | uniqPf7DR_155 | -0.04 | -0.04 | 0.03 | 0.06 | 0.13 | 0 | 0.06263 | 1.04436 | 1.35772 | 0.20925 | 0 | 0.09687 | 1.06945 | 1.08409 | 0.29055 | 0 | 0.16596 | 1.12191 | 1.6627 | 0.11099 | 1 | 0 |
| 1396737_at | Smchd1 | structural maintenance of chromosomes flexible hir | uniqPf7DR_155 | -0.05 | 0.01 | -0.08 | 0.20 | 0.00 | 0 | -0.0878 | -1.0627 | 2.77081 | 0.03724 | 0 | 0.18822 | 1.13936 | 0.97588 | 0.01371 | 0 | -0.0101 | -1.007 | 2.28346 | 0.03774 | 1 | 0 |
| 1397552_at | Eml4 | echinoderm microtubule associated protein like 4 | uniqPf7DR_155 | -0.06 | 0.12 | 0.11 | 0.12 | 0.16 | 0 | -0.0064 | -1.0045 | 1.12709 | 0.33056 | 0 | -0.0028 | -1.002 | 0.9883 | 0.30019 | 0 | 0.03558 | 1.02497 | 1.27503 | 0.23406 | 1 | 0 |
| 1397824_at | RGD1562407 | similar to WAC | uniqPf7DR_155 | 0.10 | 0.15 | 0.29 | 0.30 | 0.20 | 0 | 0.14102 | 1.10268 | 1.26711 | 0.2461 | 0 | 0.15836 | 1.11602 | 1.14479 | 0.22053 | 0 | 0.05063 | 1.03571 | 0.96054 | 0.4767 | 1 | 0 |
| 1398217_at | Zbtb41 | zinc finger and BTB domain containing 41 | uniqPf7DR_155 | -0.08 | -0.07 | 0.11 | 0.18 | -0.02 | 0 | 0.18124 | 1.13386 | 2.18147 | 0.02245 | 0 | 0.2581 | 1.1959 | 1.18451 | 0.02609 | 0 | 0.05048 | 1.03561 | 1.47153 | 0.1588 | 1 | 0 |
| 1398275_at | Mmp9 | matrix metallopeptidase 9 | uniqPf7DR_155 | 0.16 | -0.28 | -0.11 | -0.09 | -0.10 | 0 | 0.17547 | 1.12933 | 1.67908 | 0.11144 | 0 | 0.18813 | 1.13929 | 1.25181 | 0.10953 | 0 | 0.18602 | 1.13762 | 1.99386 | 0.06373 | 1 | 0 |
| 1398373_at | B3galnt1 | beta-1,3-N-acetylgalactosaminyltransferase 1 (globo | uniqPf7DR_155 | -0.07 | -0.04 | -0.02 | 0.07 | -0.05 | 0 | 0.01429 | 1.00995 | 1.00729 | 0.43733 | 0 | 0.10526 | 1.07569 | 1.19018 | 0.16977 | 0 | -0.012 | -1.0084 | 1.01861 | 0.40074 | 1 | 0 |

| Affymetrix Probeset ID | Gene Symbol | Gene Title | Figure 6 B Venn n =2021 | Mean BN log2 intensity autologous n=8 | Mean log2 intensity day 30 DR +/+ ND n=4 | Mean log2 intensity day 30 DR+/+ HCD n=4 | Mean log2 intensity day 30 DR+/+ B/S n=4 | Mean log2 intensity day 30 F+/+ ND n=4 | DR+/+ HCD vs DR+/+ ND n=380 1=yes 0=no | Log2 ratio DR+/+ HCD vs DR+/+ ND | Fold-Change DR+/+ HCD vs DR+/+ ND | RP-FDR 4DR30 HCD vs 4DR30 Reg | p value DR+/+ HCD vs DR+/+ ND | DR+/+ B/S vs DR+/+ ND n=1,330 1=yes 0=no | Log2 ratio DR+/+ B/S vs DR+/+ ND | Fold-Change DR+/+ B/S vs DR+/+ ND | RP-FDR 4DR30 Antib vs 4DR30 Reg | p value DR+/+ B/S vs DR+/+ ND | DR+/+ ND vs F+/+ ND n=398 1=yes 0=no | Log2 ratio DR+/+ ND vs F+/+ ND | Fold-Change DR+/+ ND vs F+/+ ND | RP-FDR 4RT1uu 30Reg vs 4DR30 Reg | p value DR+/+ ND vs F+/+ ND | DR+/+ Longitudinal n=228 1=yes 0=no | Figure 6F |
|---|---|---|---|---|---|---|---|---|---|---|---|---|---|---|---|---|---|---|---|---|---|---|---|---|---|
| 1398374_at | Lrrc57 | leucine rich repeat containing 57 | uniqPf7DR_155 | -0.02 | 0.04 | 0.00 | 0.06 | 0.00 | 0 | -0.0366 | -1.0257 | 0.99893 | 0.4257 | 0 | 0.02011 | 1.01403 | 1.01622 | 0.4296 | 0 | -0.0385 | -1.0271 | 1.02575 | 0.38954 | 1 | 0 |
| 1398788_at | Pdia3 | protein disulfide isomerase family A, member 3 | uniqPf7DR_155 | -0.24 | 0.28 | 0.17 | 0.24 | 0.32 | 0 | -0.1048 | -1.0754 | 1.80858 | 0.12143 | 0 | -0.0418 | -1.0294 | 0.97526 | 0.3384 | 0 | 0.03852 | 1.02706 | 1.16872 | 0.28779 | 1 | 0 |
| 1398835_at | Actb /// Actg: | actin, beta /// actin, gamma 1 /// actin, gamma 1 pr | uniqPf7DR_155 | -0.01 | -0.17 | -0.19 | -0.11 | -0.11 | 0 | -0.0227 | -1.0159 | 0.87994 | 0.61881 | 0 | 0.05657 | 1.03999 | 0.98687 | 0.51325 | 0 | 0.06132 | 1.04342 | 0.95452 | 0.48471 | 1 | 0 |
| 1398836_s_at | Actb /// Actg: | actin, beta /// actin, gamma 1 /// actin, gamma 1 pr | uniqPf7DR_155 | -0.01 | -0.16 | -0.23 | -0.11 | -0.09 | 0 | -0.0688 | -1.0488 | 0.95192 | 0.47197 | 0 | 0.05204 | 1.03673 | 0.98539 | 0.52114 | 0 | 0.07514 | 1.05347 | 0.98513 | 0.44662 | 1 | 0 |
| 1367881_at | Sirpa | signal-regulatory protein alpha | uniqRT1u_243 | 0.02 | -0.07 | -0.01 | 0.12 | 0.23 | 0 | 0.06405 | 1.0454 | 1.13676 | 0.32466 | 0 | 0.19619 | 1.14567 | 1.26285 | 0.10132 | 1 | 0.30487 | 1.23531 | 2.22346 | 0.01301 | 0 | 0 |
| 1368025_at | Ddit4 | DNA-damage-inducible transcript 4 | uniqRT1u_243 | 0.06 | -0.19 | -0.09 | -0.14 | 0.23 | 0 | 0.10186 | 1.07316 | 1.23234 | 0.26241 | 0 | 0.05093 | 1.03593 | 1.02096 | 0.41755 | 1 | 0.42761 | 1.345 | 1.19781 | 0.00247 | 0 | 0 |
| 1368103_at | Abcg1 | ATP-binding cassette, subfamily G (WHITE), membe | uniqRT1u_243 | 0.13 | 0.77 | 0.90 | 0.71 | 0.19 | 0 | 0.12225 | 1.08843 | 1.67919 | 0.1088 | 0 | -0.0626 | -1.0444 | 1.03157 | 0.21266 | 1 | -0.5821 | -1.497 | 0.485 | 3.1E-05 | 0 | 0 |
| 1368227_at | Slc28a2 | solute carrier family 28 (sodium-coupled nucleoside | uniqRT1u_243 | 0.12 | -0.25 | -0.26 | -0.12 | 0.16 | 0 | -0.0103 | -1.0072 | 0.87607 | 0.63324 | 0 | 0.13062 | 1.09476 | 1.16732 | 0.19571 | 1 | 0.4166 | 1.33478 | 1.26227 | 0.00268 | 0 | 0 |
| 1368285_at | Shbg | sex hormone binding globulin | uniqRT1u_243 | -0.17 | 0.03 | -0.20 | -0.19 | -0.37 | 0 | -0.2376 | -1.179 | 2.6238 | 0.04328 | 0 | -0.2191 | -1.164 | 1.38938 | 0.05428 | 1 | -0.3989 | -1.3185 | 4.62455 | 0.00164 | 0 | 0 |
| 1368540_at | Tpbg | trophoblast glycoprotein | uniqRT1u_243 | 0.13 | 0.19 | 0.12 | 0.16 | -0.20 | 0 | -0.0635 | -1.045 | 1.01979 | 0.40603 | 0 | -0.0276 | -1.0193 | 0.96156 | 0.5477 | 1 | -0.3892 | -1.3096 | 3.13776 | 0.00494 | 0 | 0 |
| 1368584_a_at | Cplx2 | complexin 2 | uniqRT1u_243 | -0.17 | -0.31 | -0.31 | -0.55 | -0.65 | 0 | -0.0028 | -1.0019 | 1.31222 | 0.2241 | 0 | -0.235 | -1.1769 | 1.67958 | 0.02808 | 1 | -0.3425 | -1.268 | 3.53452 | 0.00352 | 0 | 0 |
| 1368599_at | Slc9a2 | solute carrier family 9, subfamily A (NHE2, cation pr | uniqRT1u_243 | -0.04 | 0.02 | -0.05 | -0.23 | -0.25 | 0 | -0.0735 | -1.0522 | 1.10135 | 0.3393 | 0 | -0.2544 | -1.1928 | 1.28966 | 0.06996 | 1 | -0.277 | -1.2117 | 2.33294 | 0.03443 | 0 | 0 |
| 1368607_at | Cyp4a8 | cytochrome P450, family 4, subfamily a, polypeptide | uniqRT1u_243 | 0.04 | 0.18 | -0.05 | 0.16 | -0.13 | 0 | -0.231 | -1.1736 | 2.32067 | 0.06619 | 0 | -0.0128 | -1.0089 | 1.06485 | 0.31888 | 1 | -0.3076 | -1.2376 | 2.98714 | 0.0121 | 0 | 0 |
| 1368629_at | Reg1a | regenerating islet-derived 1 alpha | uniqRT1u_243 | 0.15 | 0.17 | 0.03 | -0.03 | -0.17 | 0 | -0.1421 | -1.1035 | 1.66961 | 0.14098 | 0 | -0.2044 | -1.1522 | 1.35298 | 0.05751 | 1 | -0.3454 | -1.2705 | 2.96529 | 0.00648 | 0 | 0 |
| 1368947_at | Gadd45a | growth arrest and DNA-damage-inducible, alpha | uniqRT1u_243 | 0.05 | -0.38 | -0.41 | -0.29 | -0.06 | 0 | -0.031 | -1.0217 | 1.11949 | 0.32725 | 0 | 0.08854 | 1.0633 | 1.11197 | 0.25519 | 1 | 0.31902 | 1.24748 | 2.22144 | 0.01293 | 0 | 0 |
| 1369063_at | Anp32a | acidic (leucine-rich) nuclear phosphoprotein 32 fam | uniqRT1u_243 | 0.11 | -0.24 | -0.18 | -0.25 | 0.12 | 0 | 0.05976 | 1.04229 | 2.50554 | 0.00524 | 0 | -0.0069 | -1.0048 | 2.6941 | 0.00719 | 1 | 0.36151 | 1.28477 | 0.16706 | 0.00018 | 0 | 0 |
| 1369085_s_at | LOC688682 // | similar to small nuclear ribonucleoparticle-associate | uniqRT1u_243 | 0.07 | -0.07 | 0.10 | -0.04 | 0.23 | 0 | 0.17033 | 1.12531 | 1.55568 | 0.14217 | 0 | 0.03053 | 1.02139 | 1.03499 | 0.37767 | 1 | 0.30736 | 1.23744 | 2.27257 | 0.01476 | 0 | 0 |
| 1369091_at | Chrnb1 | cholinergic receptor, nicotinic, beta 1 (muscle) | uniqRT1u_243 | 0.06 | 0.12 | -0.08 | -0.02 | -0.18 | 0 | -0.2059 | -1.1534 | 2.29418 | 0.06824 | 0 | -0.1457 | -1.1062 | 0.99828 | 0.27738 | 1 | -0.3087 | -1.2386 | 2.63328 | 0.02557 | 0 | 0 |
| 1369153_at | Nphs1 | nephrosis 1, congenital, Finnish type (nephrin) | uniqRT1u_243 | 0.09 | -0.08 | 0.05 | 0.03 | 0.18 | 0 | 0.13274 | 1.09637 | 1.36203 | 0.20733 | 0 | 0.10875 | 1.07829 | 1.08816 | 0.28552 | 1 | 0.26731 | 1.20356 | 2.36856 | 0.02955 | 0 | 0 |
| 1369192_at | Cdkn1b | cyclin-dependent kinase inhibitor 1B | uniqRT1u_243 | 0.01 | -0.17 | -0.42 | -0.23 | -0.64 | 0 | -0.2566 | -1.1947 | 2.98289 | 0.0305 | 0 | -0.0645 | -1.0457 | 1.11014 | 0.13779 | 1 | -0.4764 | -1.3913 | 4.145 | 0.00053 | 0 | 0 |
| 1369214_a_at | Ciita | class II, major histocompatibility complex, transactiv | uniqRT1u_243 | 0.09 | 0.39 | 0.24 | 0.19 | 0.12 | 0 | -0.1449 | -1.1057 | 1.66769 | 0.14227 | 0 | -0.1935 | -1.1436 | 1.06925 | 0.16469 | 1 | -0.2703 | -1.2061 | 2.08985 | 0.0457 | 0 | 0 |
| 1369229_at | Pde5a | phosphodiesterase 5A, cGMP-specific | uniqRT1u_243 | -0.04 | -0.11 | -0.28 | -0.28 | -0.43 | 0 | -0.1693 | -1.1245 | 2.30321 | 0.06747 | 0 | -0.164 | -1.1204 | 1.18137 | 0.09789 | 1 | -0.3201 | -1.2484 | 2.928 | 0.00706 | 0 | 0 |
| 1369660_at | Defb1 | defensin beta 1 | uniqRT1u_243 | -0.23 | -0.08 | -0.22 | -0.20 | -0.37 | 0 | -0.1361 | -1.0989 | 1.61586 | 0.15089 | 0 | -0.1168 | -1.0843 | 1.1278 | 0.12678 | 1 | -0.2851 | -1.2185 | 2.66109 | 0.0243 | 0 | 0 |
| 1369888_at | Gcg | glucagon | uniqRT1u_243 | 0.21 | 0.17 | 0.23 | -0.08 | -0.19 | 0 | 0.06142 | 1.04349 | 1.51463 | 0.15376 | 0 | -0.2496 | -1.1889 | 1.79659 | 0.02201 | 1 | -0.3534 | -1.2776 | 3.92111 | 0.00227 | 0 | 0 |
| 1369891_at | Batf3 | basic leucine zipper transcription factor, ATF-like 3 | uniqRT1u_243 | 0.00 | 0.07 | 0.03 | -0.18 | -0.21 | 0 | -0.0454 | -1.032 | 1.18286 | 0.29321 | 0 | -0.2487 | -1.1882 | 1.28068 | 0.07297 | 1 | -0.2855 | -1.2188 | 2.60401 | 0.02696 | 0 | 0 |
| 1370131_at | Cav1 | caveolin 1, caveolae protein | uniqRT1u_243 | -0.09 | 0.06 | -0.05 | -0.18 | -0.24 | 0 | -0.1091 | -1.0785 | 1.3231 | 0.23089 | 0 | -0.2444 | -1.1846 | 1.36008 | 0.05672 | 1 | -0.2995 | -1.2307 | 2.81692 | 0.0212 | 0 | 0 |
| 1370155_at | Col1a2 /// LO | collagen, type I, alpha 2 /// collagen alpha-2(I) chair | uniqRT1u_243 | -0.06 | -0.08 | 0.13 | -0.25 | 0.22 | 0 | 0.21038 | 1.15699 | 1.97027 | 0.04695 | 0 | -0.1633 | -1.1198 | 1.10255 | 0.14004 | 1 | 0.3014 | 1.23234 | 2.36771 | 0.02193 | 0 | 0 |
| 1370209_at | Klf9 | Kruppel-like factor 9 | uniqRT1u_243 | 0.09 | 0.29 | 0.42 | 0.29 | 0.66 | 0 | 0.13313 | 1.09667 | 1.44179 | 0.17631 | 0 | 0.00681 | 1.00473 | 1.01814 | 0.42642 | 1 | 0.37503 | 1.29687 | 1.63481 | 0.00426 | 0 | 0 |
| 1370397_at | Cyp4a3 | cytochrome P450, family 4, subfamily a, polypeptide | uniqRT1u_243 | -0.02 | 0.03 | -0.09 | -0.15 | -0.24 | 0 | -0.1235 | -1.0894 | 1.28998 | 0.24299 | 0 | -0.1835 | -1.1356 | 1.07089 | 0.1604 | 1 | -0.2739 | -1.2091 | 2.22205 | 0.03658 | 0 | 0 |
| 1370534_at | Acvr1c | activin A receptor, type IC | uniqRT1u_243 | 0.15 | 0.07 | 0.03 | -0.03 | -0.23 | 0 | -0.041 | -1.0289 | 1.15785 | 0.30421 | 0 | -0.0952 | -1.0682 | 1.0506 | 0.18388 | 1 | -0.2974 | -1.2289 | 2.91836 | 0.01314 | 0 | 0 |
| 1370728_at | Il13ra1 /// LO | interleukin 13 receptor, alpha 1 /// interleukin 13 re | uniqRT1u_243 | 0.07 | 0.28 | 0.05 | 0.04 | -0.03 | 0 | -0.2318 | -1.1743 | 2.49645 | 0.05226 | 0 | -0.2399 | -1.1809 | 1.25004 | 0.07866 | 1 | -0.3068 | -1.237 | 2.93843 | 0.01805 | 0 | 0 |
| 1370833_at | Pex5l | peroxisomal biogenesis factor 5-like | uniqRT1u_243 | 0.00 | -0.12 | 0.03 | -0.06 | 0.14 | 0 | 0.15179 | 1.11094 | 1.4112 | 0.18977 | 0 | 0.06367 | 1.04512 | 1.06951 | 0.31003 | 1 | 0.267 | 1.2033 | 2.3598 | 0.03073 | 0 | 0 |
| 1370869_at | Bcat1 | branched chain amino acid transaminase 1, cytosoli | uniqRT1u_243 | 0.06 | 0.04 | -0.06 | 0.28 | 0.35 | 0 | -0.0998 | -1.0716 | 1.45744 | 0.18657 | 0 | 0.23474 | 1.17669 | 1.26589 | 0.04592 | 1 | 0.30641 | 1.23663 | 2.21994 | 0.01235 | 0 | 0 |
| 1370964_at | Ass1 | argininosuccinate synthase 1 | uniqRT1u_243 | 0.12 | -0.14 | -0.23 | -0.10 | 0.20 | 0 | -0.0934 | -1.0669 | 1.74715 | 0.12989 | 0 | 0.03333 | 1.02337 | 1.03172 | 0.38702 | 1 | 0.33173 | 1.25852 | 2.16951 | 0.01005 | 0 | 0 |
| 1371056_at | Neo1 | neogenin 1 | uniqRT1u_243 | 0.15 | -0.12 | -0.03 | -0.24 | 0.15 | 0 | 0.09603 | 1.06883 | 1.25932 | 0.25045 | 0 | -0.1174 | -1.0847 | 1.11964 | 0.13458 | 1 | 0.27363 | 1.20884 | 2.33722 | 0.01729 | 0 | 0 |
| 1371700_at | LOC10091171 | microfibril-associated glycoprotein 4-like /// microfi | uniqRT1u_243 | 0.01 | 0.05 | -0.02 | -0.18 | -0.25 | 0 | -0.0738 | -1.0525 | 1.96257 | 0.10116 | 0 | -0.2303 | -1.1731 | 1.29267 | 0.06954 | 1 | -0.2975 | -1.229 | 2.46704 | 0.02943 | 0 | 0 |
| 1371864_at | --- | --- | uniqRT1u_243 | 0.11 | 0.35 | 0.46 | 0.37 | 0.66 | 0 | 0.114 | 1.08223 | 1.3531 | 0.20989 | 0 | 0.01471 | 1.01025 | 1.02217 | 0.41003 | 1 | 0.30518 | 1.23557 | 2.34486 | 0.01953 | 0 | 0 |
| 1372392_at | --- | --- | uniqRT1u_243 | 0.16 | 0.41 | 0.41 | 0.40 | 0.69 | 0 | 0.00316 | 1.00219 | 1.66844 | 0.11465 | 0 | -0.0007 | -1.0005 | 1.12979 | 0.12523 | 1 | 0.28668 | 1.21983 | 1.50873 | 0.00344 | 0 | 0 |
| 1372499_at | Ankrd24 /// L | ankyrin repeat domain 24 /// ankyrin repeat domai | uniqRT1u_243 | -0.01 | 0.02 | -0.02 | 0.01 | -0.29 | 0 | -0.0408 | -1.0287 | 1.0226 | 0.40343 | 0 | -0.0136 | -1.0095 | 0.96114 | 0.56598 | 1 | -0.3185 | -1.2471 | 3.00273 | 0.01699 | 0 | 0 |
| 1372539_at | --- | --- | uniqRT1u_243 | 0.07 | 0.00 | -0.06 | 0.06 | 0.33 | 0 | -0.0666 | -1.0472 | 1.12926 | 0.32259 | 0 | 0.05991 | 1.0424 | 1.03472 | 0.37833 | 1 | 0.33078 | 1.25769 | 2.24037 | 0.01376 | 0 | 0 |
| 1372968_at | Sbf1 | SET binding factor 1 | uniqRT1u_243 | 0.14 | 0.55 | 0.58 | 0.63 | 0.81 | 0 | 0.03538 | 1.02482 | 1.75123 | 0.09049 | 0 | 0.08691 | 1.06209 | 1.2378 | 0.1198 | 1 | 0.26634 | 1.20275 | 2.0975 | 0.00917 | 0 | 0 |
| 1373310_at | --- | --- | uniqRT1u_243 | 0.13 | -0.11 | 0.09 | 0.05 | 0.17 | 0 | 0.20242 | 1.15063 | 1.69649 | 0.10294 | 0 | 0.15531 | 1.11366 | 1.18746 | 0.17247 | 1 | 0.2848 | 1.21824 | 2.37142 | 0.02532 | 0 | 0 |
| 1373347_at | Acbd3 | acyl-CoA binding domain containing 3 | uniqRT1u_243 | -0.06 | 0.19 | 0.30 | 0.23 | -0.07 | 0 | 0.10889 | 1.0784 | 2.08673 | 0.0312 | 0 | 0.03394 | 1.02381 | 1.29439 | 0.06293 | 1 | -0.2656 | -1.2022 | 3.16386 | 0.01028 | 0 | 0 |
| 1373726_at | --- | --- | uniqRT1u_243 | 0.04 | 0.20 | 0.06 | 0.10 | -0.11 | 0 | -0.1401 | -1.102 | 1.92553 | 0.10643 | 0 | -0.0995 | -1.0714 | 1.00864 | 0.25463 | 1 | -0.3146 | -1.2436 | 3.3395 | 0.0043 | 0 | 0 |
| 1373747_at | Cnih2 | cornichon homolog 2 (Drosophila) | uniqRT1u_243 | 0.11 | -0.14 | -0.16 | -0.29 | -0.40 | 0 | -0.0285 | -1.0199 | 1.15101 | 0.30845 | 0 | -0.1533 | -1.1121 | 1.04486 | 0.19352 | 1 | -0.2683 | -1.2044 | 2.22398 | 0.03647 | 0 | 0 |
| 1373896_at | --- | --- | uniqRT1u_243 | 0.09 | 0.24 | 0.08 | -0.01 | -0.04 | 0 | -0.1618 | -1.1187 | 1.98833 | 0.09884 | 0 | -0.2458 | -1.1857 | 1.44113 | 0.04615 | 1 | -0.2768 | -1.2115 | 2.97604 | 0.0179 | 0 | 0 |
| 1373995_at | --- | --- | uniqRT1u_243 | 0.24 | 0.45 | 0.35 | 0.59 | 0.11 | 0 | -0.1017 | -1.073 | 3.36826 | 0.01181 | 0 | 0.13646 | 1.0992 | 1.292 | 0.06373 | 1 | -0.3376 | -1.2637 | 4.02077 | 0.00168 | 0 | 0 |
| 1374240_at | --- | --- | uniqRT1u_243 | -0.19 | -0.15 | -0.09 | -0.28 | -0.42 | 0 | 0.0606 | 1.0429 | 1.48296 | 0.16175 | 0 | -0.1278 | -1.0926 | 1.19392 | 0.10897 | 1 | -0.2711 | -1.2067 | 3.23824 | 0.00948 | 0 | 0 |
| 1374657_at | Anks4b | ankyrin repeat and sterile alpha motif domain conta | uniqRT1u_243 | -0.17 | 0.07 | -0.03 | -0.16 | -0.20 | 0 | -0.0986 | -1.0708 | 1.2193 | 0.27076 | 0 | -0.2343 | -1.1763 | 1.19392 | 0.09237 | 1 | -0.2734 | -1.2086 | 2.3474 | 0.03246 | 0 | 0 |
| 1374749_at | --- | --- | uniqRT1u_243 | -0.03 | -0.28 | -0.38 | -0.12 | 0.01 | 0 | -0.1038 | -1.0746 | 1.26898 | 0.2507 | 0 | 0.15821 | 1.1159 | 1.18993 | 0.17119 | 1 | 0.28903 | 1.22182 | 2.24316 | 0.01414 | 0 | 0 |
| 1374959_at | Nqo2 | NAD(P)H dehydrogenase, quinone 2 | uniqRT1u_243 | 0.01 | -0.48 | -0.33 | -0.37 | -0.20 | 0 | 0.14713 | 1.10737 | 2.28438 | 0.01829 | 0 | 0.10822 | 1.07789 | 1.19988 | 0.15672 | 1 | 0.27728 | 1.21191 | 2.18509 | 0.01131 | 0 | 0 |
| 1375312_at | --- | --- | uniqRT1u_243 | 0.16 | 0.10 | 0.18 | 0.16 | -0.16 | 0 | 0.08089 | 1.05767 | 1.36515 | 0.20645 | 0 | 0.05768 | 1.04079 | 1.12285 | 0.24072 | 1 | -0.2648 | -1.2015 | 2.74027 | 0.02317 | 0 | 0 |
| 1375476_at | Pygm | phosphorylase, glycogen, muscle | uniqRT1u_243 | -0.08 | -0.29 | -0.07 | -0.06 | 0.03 | 0 | 0.22138 | 1.16585 | 1.9087 | 0.05855 | 0 | 0.23289 | 1.17519 | 1.29631 | 0.07378 | 1 | 0.32648 | 1.25395 | 2.32637 | 0.0175 | 0 | 0 |
| 1375557_at | Otp | orthopedia homeobox | uniqRT1u_243 | -0.08 | 0.07 | -0.03 | -0.19 | -0.24 | 0 | -0.096 | -1.0688 | 1.38893 | 0.20763 | 0 | -0.2626 | -1.1996 | 1.44085 | 0.04684 | 1 | -0.3133 | -1.2425 | 2.92109 | 0.01287 | 0 | 0 |
| 1375571_at | --- | --- | uniqRT1u_243 | 0.20 | -0.16 | -0.03 | -0.25 | 0.17 | 0 | 0.12711 | 1.09211 | 1.1863 | 0.29136 | 0 | -0.0916 | -1.0655 | 0.97466 | 0.3345 | 1 | 0.32718 | 1.25456 | 2.23818 | 0.01223 | 0 | 0 |
| 1375611_at | --- | --- | uniqRT1u_243 | 0.01 | -0.15 | -0.05 | -0.06 | 0.12 | 0 | 0.10257 | 1.07368 | 1.23113 | 0.26342 | 0 | 0.09546 | 1.0684 | 1.07146 | 0.30622 | 1 | 0.2675 | 1.20372 | 2.28362 | 0.03789 | 0 | 0 |
| 1375712_at | Thoc7 | THO complex 7 homolog (Drosophila) | uniqRT1u_243 | 0.07 | -0.03 | -0.13 | -0.04 | -0.29 | 0 | -0.1059 | -1.0762 | 1.77812 | 0.12642 | 0 | -0.0133 | -1.0093 | 1.04165 | 0.19899 | 1 | -0.2692 | -1.2051 | 3.13835 | 0.01039 | 0 | 0 |
| 1375806_at | Tnfaip2 | tumor necrosis factor, alpha-induced protein 2 | uniqRT1u_243 | 0.11 | 0.12 | 0.06 | 0.14 | -0.15 | 0 | -0.0648 | -1.0459 | 1.05682 | 0.36912 | 0 | 0.01569 | 1.01094 | 0.9902 | 0.50457 | 1 | -0.2692 | -1.2052 | 2.21948 | 0.03683 | 0 | 0 |
| 1375834_at | Hrasls5 | HRAS-like suppressor family, member 5 | uniqRT1u_243 | 0.12 | -0.05 | 0.10 | -0.07 | 0.22 | 0 | 0.14992 | 1.10951 | 1.2996 | 0.23055 | 0 | -0.0253 | -1.0177 | 0.96282 | 0.52009 | 1 | 0.26555 | 1.2021 | 2.36337 | 0.02029 | 0 | 0 |
| 1376269_at | Osbp2 | oxysterol binding protein 2 | uniqRT1u_243 | -0.03 | -0.16 | -0.15 | -0.18 | -0.44 | 0 | 0.0154 | 1.01073 | 0.92324 | 0.55595 | 0 | -0.0231 | -1.0162 | 0.96259 | 0.5205 | 1 | -0.2834 | -1.2171 | 2.35184 | 0.03418 | 0 | 0 |
| 1376282_at | --- | --- | uniqRT1u_243 | 0.09 | 0.08 | 0.17 | 0.07 | 0.37 | 0 | 0.09064 | 1.06484 | 1.14985 | 0.31276 | 0 | -0.0154 | -1.0108 | 0.96077 | 0.50802 | 1 | 0.28346 | 1.21711 | 2.38464 | 0.02776 | 0 | 0 |
| 1376390_at | Ms4a11 | membrane-spanning 4-domains, subfamily A, meml | uniqRT1u_243 | -0.25 | -0.45 | -0.49 | -0.30 | -0.09 | 0 | -0.0402 | -1.0282 | 1.27487 | 0.24756 | 0 | 0.14627 | 1.1067 | 1.19097 | 0.1695 | 1 | 0.35715 | 1.28089 | 1.628 | 0.00419 | 0 | 0 |
| 1376539_at | --- | --- | uniqRT1u_243 | -0.05 | -0.16 | 0.04 | -0.14 | 0.11 | 0 | 0.19549 | 1.14511 | 1.93152 | 0.05621 | 0 | 0.01947 | 1.01358 | 1.0428 | 0.36224 | 1 | 0.27045 | 1.20618 | 2.29192 | 0.0157 | 0 | 0 |
| 1376657_at | Cadm1 | cell adhesion molecule 1 | uniqRT1u_243 | 0.03 | 0.00 | -0.08 | -0.15 | -0.27 | 0 | -0.0759 | -1.054 | 1.04359 | 0.38335 | 0 | -0.1484 | -1.1083 | 1.01258 | 0.24781 | 1 | -0.274 | -1.2092 | 2.42992 | 0.03016 | 0 | 0 |
| 1376718_at | Mblac1 | metallo-beta-lactamase domain containing 1 | uniqRT1u_243 | -0.24 | -0.27 | -0.47 | -0.41 | -0.56 | 0 | -0.1989 | -1.1479 | 1.96488 | 0.10147 | 0 | -0.1397 | -1.1017 | 1.02642 | 0.2276 | 1 | -0.2898 | -1.2225 | 2.913 | 0.01901 | 0 | 0 |
| 1376768_at | --- | --- | uniqRT1u_243 | 0.02 | -0.48 | -0.54 | -0.56 | -0.18 | 0 | -0.066 | -1.0468 | 2.37361 | 0.06404 | 0 | -0.0832 | -1.0594 | 1.06278 | 0.16862 | 1 | 0.29233 | 1.22462 | 2.2526 | 0.01391 | 0 | 0 |
| 1376969_at | Slc23a2 | solute carrier family 23 (nucleobase transporters), n | uniqRT1u_243 | 0.11 | -0.62 | -0.53 | -0.38 | -0.27 | 0 | 0.08943 | 1.06395 | 1.0412 | 0.40642 | 0 | 0.24382 | 1.18413 | 1.2782 | 0.08413 | 1 | 0.3462 | 1.2712 | 2.16354 | 0.01023 | 0 | 0 |
| 1377006_at | Cct6a | chaperonin containing Tcp1, subunit 6A (zeta 1) | uniqRT1u_243 | -0.10 | -0.37 | -0.21 | -0.26 | -0.04 | 0 | 0.15666 | 1.11471 | 1.56796 | 0.1389 | 0 | 0.11083 | 1.07985 | 1.16919 | 0.19241 | 1 | 0.32246 | 1.25046 | 2.24689 | 0.01373 | 0 | 0 |
| 1377035_at | Mospd4 | motile sperm domain containing 4 | uniqRT1u_243 | 0.00 | -0.19 | 0.04 | -0.39 | -0.46 | 0 | 0.22742 | 1.17074 | 2.01747 | 0.04126 | 0 | -0.2027 | -1.1509 | 1.16019 | 0.10849 | 1 | -0.2725 | -1.2079 | 3.03365 | 0.01629 | 0 | 0 |
| 1377054_at | Sema4d | sema domain, immunoglobulin domain (Ig), transm | uniqRT1u_243 | 0.16 | 0.24 | 0.26 | 0.40 | 0.53 | 0 | 0.02787 | 1.01951 | 1.13652 | 0.32474 | 0 | 0.15909 | 1.11658 | 1.23764 | 0.12007 | 1 | 0.2886 | 1.22145 | 2.25943 | 0.01526 | 0 | 0 |
| 1377085_at | Car7 | carbonic anhydrase 7 | uniqRT1u_243 | 0.04 | -0.10 | 0.04 | -0.21 | 0.17 | 0 | 0.13392 | 1.09727 | 1.41927 | 0.18278 | 0 | -0.1111 | -1.08 | 1.05157 | 0.18205 | 1 | 0.26673 | 1.20307 | 2.23486 | 0.01272 | 0 | 0 |

| Affymetrix Probeset ID | Gene Symbol | Gene Title | Figure 6 B Venn n =2021 | Mean BN log2 intensity autologous n=8 | Mean log2 intensity day 30 DR +/+ ND n=4 | Mean log2 intensity day 30 DR+/+ HCD n=4 | Mean log2 intensity day 30 DR+/+ B/S n=4 | Mean log2 intensity day 30 F+/+ ND n=4 | DR+/+ HCD vs DR+/+ ND n=380 1=yes 0=no | Log2 ratio DR+/+ HCD vs DR+/+ ND | Fold-Change DR+/+ HCD vs DR+/+ ND | RP-FDR 4DR30 HCD vs 4DR30 Reg | p value DR+/+ HCD vs DR+/+ ND | DR+/+ B/S vs DR+/+ ND n=1,330 1=yes 0=no | Log2 ratio DR+/+ B/S vs DR+/+ ND | Fold-Change DR+/+ B/S vs DR+/+ ND | RP-FDR 4DR30 Antib vs 4DR30 Reg | p value DR+/+ B/S vs DR+/+ ND | DR+/+ ND vs F+/+ ND n=398 1=yes 0=no | Log2 ratio DR+/+ ND vs F+/+ ND | Fold-Change DR+/+ ND vs F+/+ ND | RP-FDR 4RT1uu 30Reg vs 4DR30 Reg | p value DR+/+ ND vs F+/+ ND | DR+/+ Longitudinal n=228 1=yes 0=no | Figure 6F |
|---|---|---|---|---|---|---|---|---|---|---|---|---|---|---|---|---|---|---|---|---|---|---|---|---|---|
| 1377693_at | Frmpd1 | FERM and PDZ domain containing 1 | uniqRT1u_243 | -0.06 | -0.27 | -0.11 | -0.30 | 0.01 | 0 | 0.16365 | 1.12012 | 1.73432 | 0.09375 | 0 | -0.0292 | -1.0204 | 0.9838 | 0.30698 | 1 | 0.28323 | 1.21692 | 2.14484 | 0.01069 | 0 | 0 |
| 1377744_at | Pstpip2 | proline-serine-threonine phosphatase-interacting p | uniqRT1u_243 | -0.02 | -0.22 | -0.14 | -0.07 | 0.25 | 0 | 0.08068 | 1.05752 | 1.21397 | 0.27411 | 0 | 0.14983 | 1.10944 | 1.15243 | 0.21178 | 1 | 0.46836 | 1.38353 | 0.74737 | 0.00137 | 0 | 0 |
| 1377860_at | Ttc39b | tetratricopeptide repeat domain 39B | uniqRT1u_243 | 0.12 | -0.11 | -0.20 | 0.04 | -0.37 | 0 | -0.0919 | -1.0658 | 2.20043 | 0.07507 | 0 | 0.14589 | 1.10641 | 1.26805 | 0.04591 | 1 | -0.2646 | -1.2013 | 3.25238 | 0.00439 | 0 | 0 |
| 1377985_at | Snrnp40 | small nuclear ribonucleoprotein 40 (U5) | uniqRT1u_243 | 0.11 | 0.07 | 0.15 | 0.01 | -0.22 | 0 | 0.07122 | 1.05061 | 1.04804 | 0.39887 | 0 | -0.0681 | -1.0483 | 0.9647 | 0.42445 | 1 | -0.2924 | -1.2246 | 2.70235 | 0.02364 | 0 | 0 |
| 1378032_at | Nfkbiz | nuclear factor of kappa light polypeptide gene enha | uniqRT1u_243 | -0.08 | -0.59 | -0.66 | -0.36 | -0.29 | 0 | -0.0704 | -1.05 | 1.20574 | 0.27927 | 0 | 0.22805 | 1.17125 | 1.29394 | 0.0632 | 1 | 0.29304 | 1.22522 | 2.3596 | 0.02064 | 0 | 0 |
| 1378062_at | --- | --- | uniqRT1u_243 | -0.09 | 0.01 | -0.04 | -0.05 | -0.31 | 0 | -0.0477 | -1.0336 | 1.03608 | 0.39126 | 0 | -0.0618 | -1.0438 | 1.03172 | 0.21282 | 1 | -0.3186 | -1.2471 | 3.05929 | 0.01662 | 0 | 0 |
| 1378247_at | Eaf2 | ELL associated factor 2 | uniqRT1u_243 | -0.20 | -0.94 | -0.74 | -0.85 | -0.65 | 0 | 0.19751 | 1.14671 | 2.53061 | 0.00667 | 0 | 0.08531 | 1.06091 | 1.23501 | 0.12478 | 1 | 0.28592 | 1.21919 | 1.18277 | 0.00247 | 0 | 0 |
| 1378248_at | Apbb2 | amyloid beta (A4) precursor protein-binding, family | uniqRT1u_243 | -0.04 | -0.15 | -0.13 | -0.04 | 0.12 | 0 | 0.01396 | 1.00973 | 0.91311 | 0.57425 | 0 | 0.10421 | 1.07491 | 1.10161 | 0.26606 | 1 | 0.26582 | 1.20232 | 2.33241 | 0.0345 | 0 | 0 |
| 1378271_at | --- | --- | uniqRT1u_243 | 0.18 | -0.10 | 0.10 | 0.01 | 0.18 | 0 | 0.20733 | 1.15455 | 1.62766 | 0.12566 | 0 | 0.11452 | 1.08262 | 1.10259 | 0.26506 | 1 | 0.28528 | 1.21865 | 2.36601 | 0.02062 | 0 | 0 |
| 1378344_at | --- | --- | uniqRT1u_243 | 0.19 | 0.28 | 0.07 | 0.01 | 0.00 | 0 | -0.2049 | -1.1526 | 2.2415 | 0.071 | 0 | -0.2678 | -1.204 | 1.37884 | 0.05471 | 1 | -0.2767 | -1.2114 | 2.95365 | 0.01795 | 0 | 0 |
| 1378379_at | Cox11 /// LOC | cytochrome c oxidase assembly homolog 11 (yeast) | uniqRT1u_243 | 0.03 | 0.09 | 0.09 | 0.07 | -0.20 | 0 | 0.00398 | 1.00276 | 1.02514 | 0.42151 | 0 | -0.0141 | -1.0098 | 0.96776 | 0.39932 | 1 | -0.2831 | -1.2168 | 2.93612 | 0.01218 | 0 | 0 |
| 1378478_at | Stac2 | SH3 and cysteine rich domain 2 | uniqRT1u_243 | -0.03 | -0.05 | -0.15 | -0.12 | -0.33 | 0 | -0.0949 | -1.068 | 1.79635 | 0.1224 | 0 | -0.0694 | -1.0493 | 1.02818 | 0.22634 | 1 | -0.2741 | -1.2093 | 2.95356 | 0.01415 | 0 | 0 |
| 1378683_at | --- | --- | uniqRT1u_243 | -0.02 | -0.04 | 0.11 | 0.14 | 0.25 | 0 | 0.15258 | 1.11155 | 1.54504 | 0.14492 | 0 | 0.18662 | 1.1381 | 1.22599 | 0.13001 | 1 | 0.29198 | 1.22432 | 2.35069 | 0.01965 | 0 | 0 |
| 1378699_at | Pkhd1l1 | polycystic kidney and hepatic disease 1-like 1 | uniqRT1u_243 | -0.07 | 0.09 | -0.05 | -0.17 | -0.19 | 0 | -0.1394 | -1.1014 | 1.57756 | 0.15852 | 0 | -0.2557 | -1.1939 | 1.29685 | 0.06601 | 1 | -0.2791 | -1.2135 | 2.58466 | 0.02784 | 0 | 0 |
| 1378708_at | --- | --- | uniqRT1u_243 | 0.05 | -0.04 | -0.05 | -0.23 | -0.31 | 0 | -0.01 | -1.0069 | 1.07142 | 0.36002 | 0 | -0.182 | -1.1345 | 1.12879 | 0.12707 | 1 | -0.265 | -1.2016 | 2.14525 | 0.04284 | 0 | 0 |
| 1378828_at | --- | --- | uniqRT1u_243 | 0.10 | -0.04 | 0.17 | -0.27 | 0.27 | 0 | 0.20685 | 1.15417 | 1.70302 | 0.09978 | 0 | -0.2293 | -1.1723 | 1.24896 | 0.07835 | 1 | 0.30255 | 1.23333 | 2.38859 | 0.02335 | 0 | 0 |
| 1378856_at | LOC679974 // | similar to transcription elongation factor A (SII)-like | uniqRT1u_243 | 0.00 | 0.02 | 0.01 | -0.18 | -0.33 | 0 | -0.0054 | -1.0038 | 1.19075 | 0.28694 | 0 | -0.2016 | -1.15 | 1.56748 | 0.03654 | 1 | -0.3444 | -1.2696 | 5.01857 | 0.00113 | 0 | 0 |
| 1379118_at | --- | --- | uniqRT1u_243 | 0.01 | 0.05 | -0.02 | -0.16 | -0.28 | 0 | -0.0755 | -1.0537 | 1.32606 | 0.22898 | 0 | -0.2097 | -1.1565 | 1.40458 | 0.05185 | 1 | -0.3387 | -1.2646 | 3.20588 | 0.01 | 0 | 0 |
| 1379134_at | --- | --- | uniqRT1u_243 | -0.15 | -0.10 | -0.14 | -0.19 | -0.41 | 0 | -0.042 | -1.0296 | 1.46054 | 0.1848 | 0 | -0.0908 | -1.0649 | 1.07704 | 0.15678 | 1 | -0.3056 | -1.236 | 3.35073 | 0.00593 | 0 | 0 |
| 1379239_at | Uimc1 | ubiquitin interaction motif containing 1 | uniqRT1u_243 | -0.12 | -0.27 | -0.38 | -0.18 | -0.58 | 0 | -0.1155 | -1.0834 | 2.41182 | 0.05979 | 0 | 0.09018 | 1.0645 | 1.25021 | 0.11192 | 1 | -0.3157 | -1.2446 | 3.05046 | 0.00638 | 0 | 0 |
| 1379321_at | --- | --- | uniqRT1u_243 | 0.13 | -0.05 | 0.01 | -0.06 | 0.23 | 0 | 0.0688 | 1.04884 | 1.28022 | 0.24086 | 0 | -0.007 | -1.0049 | 0.96123 | 0.50588 | 1 | 0.28292 | 1.21665 | 2.35712 | 0.02213 | 0 | 0 |
| 1379343_at | MGC94335 | similar to hypothetical protein FLJ22555 | uniqRT1u_243 | -0.11 | -0.04 | -0.11 | -0.05 | -0.32 | 0 | -0.0711 | -1.0505 | 1.07721 | 0.35435 | 0 | -0.0023 | -1.0016 | 0.96296 | 0.4688 | 1 | -0.2758 | -1.2107 | 2.68117 | 0.02423 | 0 | 0 |
| 1379374_at | Lppr4 | lipid phosphate phosphatase-related protein type 4 | uniqRT1u_243 | -0.04 | 0.04 | -0.01 | -0.11 | -0.25 | 0 | -0.0444 | -1.0313 | 0.96077 | 0.46162 | 0 | -0.1481 | -1.1081 | 1.06929 | 0.16167 | 1 | -0.2828 | -1.2166 | 2.76441 | 0.02258 | 0 | 0 |
| 1379421_at | --- | --- | uniqRT1u_243 | 0.12 | 0.13 | 0.16 | -0.05 | -0.14 | 0 | 0.02712 | 1.01897 | 0.92414 | 0.55432 | 0 | -0.1798 | -1.1327 | 1.14958 | 0.11267 | 1 | -0.268 | -1.2042 | 2.09669 | 0.04517 | 0 | 0 |
| 1379445_at | --- | --- | uniqRT1u_243 | -0.22 | 0.01 | -0.16 | -0.22 | -0.32 | 0 | -0.1707 | -1.1256 | 2.22729 | 0.07284 | 0 | -0.2372 | -1.1787 | 1.44204 | 0.04604 | 1 | -0.3299 | -1.2569 | 2.91472 | 0.00675 | 0 | 0 |
| 1379573_at | LOC1009121 | uncharacterized LOC100912188 | uniqRT1u_243 | -0.02 | -0.31 | -0.26 | -0.21 | 0.02 | 0 | 0.05179 | 1.03655 | 1.85081 | 0.06802 | 0 | 0.10004 | 1.0718 | 1.25094 | 0.10708 | 1 | 0.33295 | 1.25959 | 1.74278 | 0.00504 | 0 | 0 |
| 1379579_at | Fam210a | family with sequence similarity 210, member A | uniqRT1u_243 | -0.04 | -0.01 | -0.12 | 0.04 | -0.30 | 0 | -0.1115 | -1.0803 | 1.28901 | 0.24359 | 0 | 0.05487 | 1.03877 | 1.0498 | 0.34844 | 1 | -0.2878 | -1.2208 | 2.92927 | 0.01808 | 0 | 0 |
| 1379843_at | Vps36 | vacuolar protein sorting 36 homolog (S. cerevisiae) | uniqRT1u_243 | -0.02 | 0.05 | 0.04 | -0.09 | -0.22 | 0 | -0.0136 | -1.0095 | 1.40915 | 0.20014 | 0 | -0.1409 | -1.1026 | 1.20981 | 0.08675 | 1 | -0.2733 | -1.2085 | 3.18602 | 0.01004 | 0 | 0 |
| 1379888_at | LOC1009126 | uncharacterized LOC100912602 | uniqRT1u_243 | -0.02 | 0.02 | -0.22 | -0.16 | -0.25 | 0 | -0.2482 | -1.1877 | 3.04281 | 0.02642 | 0 | -0.1865 | -1.138 | 1.22302 | 0.08322 | 1 | -0.2726 | -1.208 | 2.9001 | 0.01949 | 0 | 0 |
| 1379948_at | --- | --- | uniqRT1u_243 | 0.02 | 0.14 | 0.02 | 0.12 | -0.13 | 0 | -0.1192 | -1.0861 | 1.30611 | 0.23767 | 0 | -0.0143 | -1.01 | 0.96204 | 0.61489 | 1 | -0.2646 | -1.2013 | 2.06623 | 0.04651 | 0 | 0 |
| 1380287_at | Creb5 | cAMP responsive element binding protein 5 | uniqRT1u_243 | 0.08 | 0.09 | -0.05 | -0.07 | -0.20 | 0 | -0.1368 | -1.0995 | 1.33757 | 0.22494 | 0 | -0.1593 | -1.1167 | 1.04452 | 0.19188 | 1 | -0.2916 | -1.224 | 2.58226 | 0.02715 | 0 | 0 |
| 1380352_at | --- | --- | uniqRT1u_243 | 0.14 | -0.21 | -0.08 | -0.07 | 0.11 | 0 | 0.13343 | 1.0969 | 1.23187 | 0.26183 | 0 | 0.13833 | 1.10063 | 1.11042 | 0.25976 | 1 | 0.32111 | 1.24929 | 2.29682 | 0.01581 | 0 | 0 |
| 1380611_at | Fkbp5 | FK506 binding protein 5 | uniqRT1u_243 | 0.03 | -0.27 | -0.37 | -0.41 | 0.08 | 0 | -0.0955 | -1.0684 | 1.1867 | 0.28882 | 0 | -0.1344 | -1.0977 | 1.03051 | 0.21883 | 1 | 0.3546 | 1.27863 | 2.05091 | 0.00798 | 0 | 0 |
| 1380625_at | --- | --- | uniqRT1u_243 | -0.19 | 0.02 | 0.02 | 0.03 | -0.31 | 0 | 0.0071 | 1.00494 | 0.90082 | 0.59911 | 0 | 0.01408 | 1.00981 | 0.98436 | 0.52752 | 1 | -0.3263 | -1.2538 | 3.01175 | 0.01162 | 0 | 0 |
| 1380716_at | Prrt3 | proline-rich transmembrane protein 3 | uniqRT1u_243 | -0.02 | -0.18 | 0.08 | 0.00 | 0.14 | 0 | 0.26163 | 1.19883 | 2.06096 | 0.0395 | 0 | 0.18433 | 1.13629 | 1.25326 | 0.10619 | 1 | 0.32249 | 1.25048 | 2.19809 | 0.01145 | 0 | 0 |
| 1380759_at | --- | --- | uniqRT1u_243 | 0.11 | 0.20 | 0.07 | 0.00 | -0.15 | 0 | -0.138 | -1.1004 | 2.32877 | 0.06552 | 0 | -0.2018 | -1.1502 | 1.29271 | 0.06622 | 1 | -0.3544 | -1.2784 | 3.42513 | 0.0043 | 0 | 0 |
| 1381078_at | --- | --- | uniqRT1u_243 | -0.08 | -0.09 | -0.01 | 0.08 | 0.22 | 0 | 0.07785 | 1.05544 | 1.19747 | 0.28398 | 0 | 0.16601 | 1.12195 | 1.18912 | 0.17153 | 1 | 0.31356 | 1.24277 | 2.29649 | 0.01787 | 0 | 0 |
| 1381104_at | --- | --- | uniqRT1u_243 | 0.01 | 0.09 | -0.01 | -0.11 | -0.19 | 0 | -0.0985 | -1.0706 | 1.21331 | 0.27372 | 0 | -0.2019 | -1.1502 | 1.11807 | 0.13615 | 1 | -0.2743 | -1.2094 | 2.23945 | 0.03543 | 0 | 0 |
| 1381117_at | --- | --- | uniqRT1u_243 | 0.02 | 0.06 | -0.18 | 0.06 | -0.24 | 0 | -0.2306 | -1.1733 | 3.03616 | 0.029 | 0 | 0.00547 | 1.0038 | 1.01787 | 0.42205 | 1 | -0.2981 | -1.2295 | 2.93221 | 0.00726 | 0 | 0 |
| 1381131_at | --- | --- | uniqRT1u_243 | 0.03 | -0.29 | -0.29 | -0.21 | 0.00 | 0 | 0.00635 | 1.00441 | 1.16631 | 0.30374 | 0 | 0.08125 | 1.05793 | 1.03117 | 0.38818 | 1 | 0.29062 | 1.22316 | 2.39403 | 0.02579 | 0 | 0 |
| 1381152_at | Ntmt1 | N-terminal Xaa-Pro-Lys N-methyltransferase 1 | uniqRT1u_243 | 0.13 | 0.18 | -0.03 | 0.03 | -0.15 | 0 | -0.2154 | -1.161 | 2.21281 | 0.07471 | 0 | -0.1514 | -1.1107 | 1.08374 | 0.14901 | 1 | -0.3365 | -1.2627 | 3.1883 | 0.00964 | 0 | 0 |
| 1381182_at | --- | --- | uniqRT1u_243 | 0.14 | 0.01 | 0.06 | -0.02 | -0.27 | 0 | 0.04866 | 1.03431 | 0.97359 | 0.47939 | 0 | -0.0345 | -1.0242 | 0.96121 | 0.56012 | 1 | -0.2858 | -1.2191 | 2.98939 | 0.0174 | 0 | 0 |
| 1381294_at | --- | --- | uniqRT1u_243 | 0.11 | 0.08 | 0.03 | -0.17 | -0.30 | 0 | -0.0496 | -1.0349 | 1.20842 | 0.27853 | 0 | -0.2451 | -1.1852 | 1.37749 | 0.05519 | 1 | -0.3765 | -1.2982 | 3.19089 | 0.00462 | 0 | 0 |
| 1381363_at | --- | --- | uniqRT1u_243 | -0.37 | 0.19 | 0.14 | 0.08 | -0.08 | 0 | -0.0561 | -1.0396 | 1.0431 | 0.38418 | 0 | -0.1174 | -1.0847 | 1.02753 | 0.22236 | 1 | -0.2727 | -1.2081 | 2.74347 | 0.02311 | 0 | 0 |
| 1381368_at | --- | --- | uniqRT1u_243 | -0.04 | 0.19 | 0.02 | 0.10 | -0.14 | 0 | -0.1728 | -1.1272 | 2.46866 | 0.05398 | 0 | -0.0921 | -1.0659 | 1.1484 | 0.11614 | 1 | -0.3343 | -1.2608 | 3.1193 | 0.00863 | 0 | 0 |
| 1381547_at | --- | --- | uniqRT1u_243 | 0.17 | -0.09 | 0.00 | -0.07 | 0.18 | 0 | 0.08926 | 1.06382 | 1.57593 | 0.13647 | 0 | 0.02474 | 1.0173 | 1.01821 | 0.42524 | 1 | 0.27432 | 1.20943 | 2.31402 | 0.01778 | 0 | 0 |
| 1381798_at | Lmo7 | LIM domain 7 | uniqRT1u_243 | -0.17 | 0.22 | 0.10 | 0.13 | -0.07 | 0 | -0.1251 | -1.0906 | 1.49028 | 0.17769 | 0 | -0.0935 | -1.067 | 0.97487 | 0.32269 | 1 | -0.2878 | -1.2207 | 2.98154 | 0.01179 | 0 | 0 |
| 1381996_at | --- | --- | uniqRT1u_243 | -0.06 | 0.00 | -0.06 | -0.13 | -0.36 | 0 | -0.0595 | -1.0421 | 1.05921 | 0.36733 | 0 | -0.1293 | -1.0937 | 1.00061 | 0.26618 | 1 | -0.3529 | -1.2771 | 3.30759 | 0.00574 | 0 | 0 |
| 1382156_at | Vps33a | vacuolar protein sorting 33 homolog A (S. cerevisiae | uniqRT1u_243 | 0.06 | 0.15 | 0.06 | -0.04 | -0.12 | 0 | -0.0947 | -1.0678 | 1.21995 | 0.27075 | 0 | -0.1879 | -1.1391 | 1.06947 | 0.16373 | 1 | -0.268 | -1.2042 | 2.11187 | 0.04462 | 0 | 0 |
| 1382330_at | Asap2 | ArfGAP with SH3 domain, ankyrin repeat and PH do | uniqRT1u_243 | -0.09 | -0.51 | -0.45 | -0.41 | -0.13 | 0 | 0.06192 | 1.04385 | 1.1247 | 0.33214 | 0 | 0.10759 | 1.07742 | 1.11932 | 0.24989 | 1 | 0.38674 | 1.30744 | 1.64098 | 0.00433 | 0 | 0 |
| 1383008_at | Smc4 | structural maintenance of chromosomes 4 | uniqRT1u_243 | -0.24 | 0.12 | -0.04 | -0.12 | -0.18 | 0 | -0.1607 | -1.1179 | 2.07314 | 0.08579 | 0 | -0.2438 | -1.1841 | 1.83094 | 0.02119 | 1 | -0.3067 | -1.2369 | 2.91763 | 0.01267 | 0 | 0 |
| 1383138_at | Ssh3 | slingshot protein phosphatase 3 | uniqRT1u_243 | -0.04 | 0.09 | -0.10 | 0.05 | -0.21 | 0 | -0.1886 | -1.1396 | 1.7202 | 0.13336 | 0 | -0.0448 | -1.0315 | 0.96295 | 0.44904 | 1 | -0.2982 | -1.2296 | 2.6065 | 0.02707 | 0 | 0 |
| 1383165_at | RGD1310209 | similar to KIAA1324 protein | uniqRT1u_243 | -0.13 | -0.34 | -0.12 | -0.31 | 0.00 | 0 | 0.22058 | 1.1652 | 1.71193 | 0.09793 | 0 | 0.03217 | 1.02255 | 1.16429 | 0.20385 | 1 | 0.33913 | 1.265 | 2.06909 | 0.00878 | 0 | 0 |
| 1383295_at | Fam110a | family with sequence similarity 110, member A | uniqRT1u_243 | -0.02 | 0.41 | 0.41 | 0.38 | 0.68 | 0 | -0.0019 | -1.0013 | 1.41049 | 0.19988 | 0 | -0.0326 | -1.0228 | 1.02846 | 0.222 | 1 | 0.26893 | 1.20492 | 2.15773 | 0.01041 | 0 | 0 |
| 1383566_at | LOC10091307 | uncharacterized LOC100913073 | uniqRT1u_243 | 0.22 | 0.03 | 0.21 | 0.25 | 0.29 | 0 | 0.17894 | 1.13205 | 1.67885 | 0.11121 | 0 | 0.22398 | 1.16795 | 1.27868 | 0.04445 | 1 | 0.26407 | 1.20086 | 2.36514 | 0.02814 | 0 | 0 |
| 1383718_at | --- | --- | uniqRT1u_243 | 0.01 | -0.25 | -0.20 | -0.22 | 0.05 | 0 | 0.0503 | 1.03548 | 1.26278 | 0.24858 | 0 | 0.03111 | 1.0218 | 1.06999 | 0.30845 | 1 | 0.2985 | 1.22986 | 2.24197 | 0.01427 | 0 | 0 |
| 1383992_at | --- | --- | uniqRT1u_243 | -0.07 | 0.04 | 0.11 | 0.15 | 0.33 | 0 | 0.07033 | 1.04995 | 1.16487 | 0.30632 | 0 | 0.10984 | 1.07911 | 1.11456 | 0.25313 | 1 | 0.28762 | 1.22062 | 2.34232 | 0.02139 | 0 | 0 |
| 1384004_at | Gpr158 | G protein-coupled receptor 158 | uniqRT1u_243 | 0.10 | 0.22 | 0.01 | 0.00 | -0.07 | 0 | -0.2095 | -1.1563 | 2.09989 | 0.08224 | 0 | -0.2183 | -1.1634 | 1.29797 | 0.06419 | 1 | -0.295 | -1.2269 | 2.64211 | 0.0254 | 0 | 0 |
| 1384014_at | --- | --- | uniqRT1u_243 | 0.33 | 0.18 | 0.16 | 0.06 | -0.12 | 0 | -0.0218 | -1.0152 | 0.97416 | 0.44763 | 0 | -0.1275 | -1.0924 | 0.99283 | 0.28554 | 1 | -0.3062 | -1.2364 | 2.78967 | 0.02198 | 0 | 0 |
| 1384043_at | --- | --- | uniqRT1u_243 | -0.04 | 0.08 | 0.11 | -0.06 | -0.19 | 0 | 0.02965 | 1.02076 | 0.97248 | 0.48166 | 0 | -0.1386 | -1.1008 | 1.05728 | 0.17369 | 1 | -0.2699 | -1.2058 | 2.76943 | 0.022 | 0 | 0 |
| 1384199_at | --- | --- | uniqRT1u_243 | -0.11 | -0.11 | -0.18 | -0.20 | -0.46 | 0 | -0.064 | -1.0454 | 1.39211 | 0.20623 | 0 | -0.0921 | -1.0659 | 0.96694 | 0.38834 | 1 | -0.346 | -1.2711 | 3.19957 | 0.00473 | 0 | 0 |
| 1384229_at | Gnal | guanine nucleotide binding protein, alpha stimulati | uniqRT1u_243 | 0.07 | 0.05 | -0.14 | -0.06 | -0.21 | 0 | -0.1964 | -1.1458 | 2.17564 | 0.0457 | 0 | -0.1106 | -1.0797 | 1.01349 | 0.24517 | 1 | -0.2647 | -1.2014 | 2.62932 | 0.02612 | 0 | 0 |
| 1384240_at | Agtr1a | angiotensin II receptor, type 1a | uniqRT1u_243 | 0.16 | 0.21 | 0.23 | -0.03 | -0.05 | 0 | 0.01903 | 1.01328 | 1.06745 | 0.37811 | 0 | -0.2395 | -1.1806 | 1.52096 | 0.03922 | 1 | -0.2663 | -1.2027 | 2.60467 | 0.02672 | 0 | 0 |
| 1384304_at | --- | --- | uniqRT1u_243 | 0.04 | -0.22 | -0.08 | -0.10 | 0.10 | 0 | 0.14049 | 1.10228 | 1.23979 | 0.25873 | 0 | 0.12362 | 1.08947 | 1.07783 | 0.29407 | 1 | 0.32602 | 1.25355 | 2.21934 | 0.01306 | 0 | 0 |
| 1384309_at | --- | --- | uniqRT1u_243 | 0.17 | 0.27 | 0.28 | 0.21 | -0.03 | 0 | 0.01399 | 1.00974 | 1.45915 | 0.1858 | 0 | -0.0568 | -1.0402 | 1.00404 | 0.26025 | 1 | -0.3003 | -1.2314 | 2.88952 | 0.01923 | 0 | 0 |
| 1384471_at | --- | --- | uniqRT1u_243 | -0.04 | 0.08 | -0.04 | -0.04 | -0.22 | 0 | -0.1143 | -1.0824 | 1.77702 | 0.12617 | 0 | -0.1205 | -1.0871 | 1.02171 | 0.23201 | 1 | -0.2951 | -1.227 | 2.7612 | 0.02291 | 0 | 0 |
| 1384654_at | --- | --- | uniqRT1u_243 | -0.08 | -0.26 | -0.19 | -0.12 | 0.03 | 0 | 0.07286 | 1.0518 | 1.65735 | 0.11612 | 0 | 0.13941 | 1.10145 | 1.28721 | 0.06167 | 1 | 0.2975 | 1.22901 | 1.75913 | 0.0052 | 0 | 0 |
| 1384715_at | --- | --- | uniqRT1u_243 | 0.11 | 0.21 | 0.20 | 0.03 | -0.08 | 0 | -0.0065 | -1.0045 | 0.88991 | 0.58796 | 0 | -0.1754 | -1.1293 | 1.03713 | 0.2028 | 1 | -0.2898 | -1.2225 | 2.4354 | 0.03031 | 0 | 0 |
| 1384925_at | LOC10091221 | uncharacterized LOC100912219 | uniqRT1u_243 | -0.01 | -0.06 | 0.04 | -0.08 | 0.23 | 0 | 0.10612 | 1.07633 | 1.25884 | 0.25028 | 0 | -0.0115 | -1.008 | 1.01217 | 0.43759 | 1 | 0.29249 | 1.22475 | 2.372 | 0.02364 | 0 | 0 |
| 1384936_at | --- | --- | uniqRT1u_243 | 0.06 | 0.05 | -0.01 | -0.05 | -0.37 | 0 | -0.0564 | -1.0399 | 1.0428 | 0.38531 | 0 | -0.0954 | -1.0684 | 1.02441 | 0.23071 | 1 | -0.4146 | -1.3329 | 3.67679 | 0.00331 | 0 | 0 |
| 1385011_at | Smarca4 | SWI/SNF related, matrix associated, actin dependen | uniqRT1u_243 | 0.14 | -0.10 | 0.13 | 0.13 | 0.32 | 0 | 0.22363 | 1.16767 | 2.2691 | 0.01372 | 0 | 0.22523 | 1.16896 | 1.27724 | 0.04933 | 1 | 0.4167 | 1.33487 | 1.19613 | 0.00238 | 0 | 0 |

| Affymetrix Probeset ID | Gene Symbol | Gene Title | Figure 6 B Venn n =2021 | Mean BN log2 intensity autologous n=8 | Mean log2 intensity day 30 DR +/+ ND n=4 | Mean log2 intensity day 30 DR+/+ HCD n=4 | Mean log2 intensity day 30 B/S n=4 | Mean log2 intensity day 30 F+/+ ND n=4 | DR+/+ HCD vs DR+/+ ND n=380 1=yes 0=no | Log2 ratio DR+/+ HCD vs DR+/+ ND | Fold-Change DR+/+ HCD vs DR+/+ ND | RP-FDR 4DR30 HCD vs 4DR30 Reg | p value DR+/+ HCD vs DR+/+ ND | DR+/+ B/S vs DR+/+ ND n=1,330 1=yes 0=no | Log2 ratio DR+/+ B/S vs DR+/+ ND | Fold-Change DR+/+ B/S vs DR+/+ ND | RP-FDR 4DR30 Antib vs 4DR30 Reg | p value DR+/+ B/S vs DR+/+ ND | DR+/+ ND vs F+/+ ND n=398 1=yes 0=no | Log2 ratio DR+/+ ND vs F+/+ ND | Fold-Change DR+/+ ND vs F+/+ ND | RP-FDR 4RT1uu 30Reg vs 4DR30 Reg | p value DR+/+ ND vs F+/+ ND | DR+/+ Longitudinal n=228 1=yes 0=no | Figure 6F |
|---|---|---|---|---|---|---|---|---|---|---|---|---|---|---|---|---|---|---|---|---|---|---|---|---|---|
| 1385051_at | Gbp4 /// LOC | guanylate binding protein 4 /// guanylate-binding p | uniqRT1u_243 | -0.09 | -1.11 | -1.11 | -1.06 | -0.77 | 0 | 0.00489 | 1.0034 | 1.32482 | 0.22029 | 0 | 0.04933 | 1.03478 | 1.02153 | 0.41516 | 1 | 0.34447 | 1.26968 | 1.73485 | 0.00541 | 0 | 0 |
| 1385140_at | Trim9 | tripartite motif-containing 9 | uniqRT1u_243 | -0.04 | -0.27 | -0.23 | -0.10 | -0.01 | 0 | 0.04574 | 1.03221 | 1.12738 | 0.3301 | 0 | 0.17213 | 1.12672 | 1.24092 | 0.11867 | 1 | 0.26548 | 1.20203 | 2.33812 | 0.03398 | 0 | 0 |
| 1385259_at | Ube2k | ubiquitin-conjugating enzyme E2K | uniqRT1u_243 | 0.06 | 0.11 | -0.01 | 0.13 | -0.16 | 0 | -0.1212 | -1.0876 | 2.01662 | 0.09305 | 0 | 0.01813 | 1.01265 | 1.01814 | 0.42645 | 1 | -0.2717 | -1.2072 | 2.89877 | 0.02041 | 0 | 0 |
| 1385483_at | Vsig8 | V-set and immunoglobulin domain containing 8 | uniqRT1u_243 | 0.16 | 0.16 | 0.01 | -0.02 | -0.11 | 0 | -0.1478 | -1.1079 | 1.9784 | 0.09943 | 0 | -0.1863 | -1.1379 | 1.3409 | 0.05799 | 1 | -0.2763 | -1.2111 | 2.75147 | 0.0223 | 0 | 0 |
| 1385486_at | Bnc2 | basonuclin 2 | uniqRT1u_243 | -0.28 | -0.21 | -0.44 | -0.26 | -0.52 | 0 | -0.2298 | -1.1726 | 3.20628 | 0.01969 | 0 | -0.0476 | -1.0335 | 0.97785 | 0.31924 | 1 | -0.3073 | -1.2374 | 3.20687 | 0.01021 | 0 | 0 |
| 1385559_at | RGD1563441 | similar to RIKEN cDNA A030009H04 | uniqRT1u_243 | -0.05 | 0.04 | -0.05 | -0.01 | -0.24 | 0 | -0.0967 | -1.0693 | 1.38252 | 0.20907 | 0 | -0.0504 | -1.0356 | 0.97383 | 0.32382 | 1 | -0.2856 | -1.2189 | 2.89321 | 0.02056 | 0 | 0 |
| 1385990_at | Zfp865 | zinc finger protein 865 | uniqRT1u_243 | 0.07 | 0.24 | 0.14 | 0.22 | -0.07 | 0 | -0.1035 | -1.0744 | 1.62783 | 0.14986 | 0 | -0.0166 | -1.0116 | 0.98407 | 0.30504 | 1 | -0.3097 | -1.2394 | 3.21 | 0.0096 | 0 | 0 |
| 1386188_s_at | --- | --- | uniqRT1u_243 | -0.02 | -0.25 | -0.11 | -0.09 | 0.05 | 0 | 0.14148 | 1.10304 | 1.6161 | 0.1294 | 0 | 0.16179 | 1.11867 | 1.2479 | 0.11203 | 1 | 0.30362 | 1.23424 | 2.21151 | 0.01131 | 0 | 0 |
| 1386859_at | Tkt | transketolase | uniqRT1u_243 | 0.01 | -0.17 | -0.07 | 0.07 | 0.13 | 0 | 0.10101 | 1.07252 | 1.11652 | 0.3392 | 0 | 0.24081 | 1.18165 | 1.27551 | 0.08453 | 1 | 0.30917 | 1.23899 | 2.38346 | 0.02062 | 0 | 0 |
| 1387255_at | Aanat | aralkylamine N-acetyltransferase | uniqRT1u_243 | -0.06 | -0.01 | 0.02 | -0.21 | -0.29 | 0 | 0.02447 | 1.01711 | 0.94601 | 0.51521 | 0 | -0.1995 | -1.1483 | 1.13369 | 0.12427 | 1 | -0.2838 | -1.2174 | 2.24706 | 0.03533 | 0 | 0 |
| 1387420_at | Clic4 | chloride intracellular channel 4 | uniqRT1u_243 | 0.00 | -0.32 | -0.20 | -0.36 | -0.06 | 0 | 0.12384 | 1.08963 | 1.97553 | 0.04466 | 0 | -0.0416 | -1.0293 | 1.00028 | 0.26635 | 1 | 0.26564 | 1.20217 | 2.36461 | 0.02935 | 0 | 0 |
| 1387618_at | Bcl2l10 | BCL2-like 10 (apoptosis facilitator) | uniqRT1u_243 | 0.02 | 0.11 | -0.06 | -0.14 | -0.16 | 0 | -0.1734 | -1.1277 | 2.41307 | 0.06045 | 0 | -0.2459 | -1.1858 | 2.0011 | 0.01757 | 1 | -0.2658 | -1.2023 | 3.00303 | 0.01719 | 0 | 0 |
| 1387700_at | Cckar | cholecystokinin A receptor | uniqRT1u_243 | 0.00 | 0.24 | 0.18 | 0.03 | -0.06 | 0 | -0.0597 | -1.0423 | 1.33795 | 0.22423 | 0 | -0.2064 | -1.1538 | 1.19651 | 0.09211 | 1 | -0.2995 | -1.2307 | 2.99836 | 0.01533 | 0 | 0 |
| 1387748_at | Lep | leptin | uniqRT1u_243 | 0.01 | 0.02 | 0.05 | -0.08 | -0.24 | 0 | 0.02504 | 1.01751 | 1.44614 | 0.17531 | 0 | -0.1081 | -1.0778 | 1.00221 | 0.26242 | 1 | -0.266 | -1.2024 | 2.0966 | 0.04497 | 0 | 0 |
| 1387828_at | Agap2 | ArfGAP with GTPase domain, ankyrin repeat and PH | uniqRT1u_243 | 0.16 | 0.18 | 0.02 | 0.10 | -0.13 | 0 | -0.1562 | -1.1143 | 1.50496 | 0.17562 | 0 | -0.0799 | -1.0569 | 0.98752 | 0.30151 | 1 | -0.3143 | -1.2434 | 2.88 | 0.01954 | 0 | 0 |
| 1387908_at | Rasd1 | RAS, dexamethasone-induced 1 | uniqRT1u_243 | 0.20 | -1.16 | -1.12 | -1.23 | -0.46 | 0 | 0.04203 | 1.02956 | 1.50186 | 0.15657 | 0 | -0.0641 | -1.0454 | 1.29409 | 0.06953 | 1 | 0.70653 | 1.63188 | 0.0084 | 6.8E-06 | 0 | 0 |
| 1388170_at | Kctd1 | potassium channel tetramerisation domain containi | uniqRT1u_243 | 0.27 | 0.07 | 0.32 | -0.07 | 0.38 | 0 | 0.24993 | 1.18915 | 1.93354 | 0.05571 | 0 | -0.1363 | -1.0991 | 1.0192 | 0.23698 | 1 | 0.31407 | 1.24321 | 2.22904 | 0.01276 | 0 | 0 |
| 1388192_at | Rwdd3 | RWD domain containing 3 | uniqRT1u_243 | -0.16 | -0.20 | -0.46 | -0.47 | -0.58 | 0 | -0.259 | -1.1966 | 2.60695 | 0.04258 | 0 | -0.2647 | -1.2014 | 1.32273 | 0.06086 | 1 | -0.3805 | -1.3018 | 3.21386 | 0.00455 | 0 | 0 |
| 1388901_at | Fkbp5 | FK506 binding protein 5 | uniqRT1u_243 | 0.04 | -0.10 | 0.01 | -0.27 | 0.29 | 0 | 0.10767 | 1.07749 | 1.207 | 0.27871 | 0 | -0.168 | -1.1235 | 1.07845 | 0.15609 | 1 | 0.3864 | 1.30713 | 1.74628 | 0.00483 | 0 | 0 |
| 1389474_at | Mylip | myosin regulatory light chain interacting protein | uniqRT1u_243 | 0.08 | 0.65 | 0.68 | 0.75 | 0.18 | 0 | 0.03326 | 1.02332 | 1.0884 | 0.35873 | 0 | 0.10098 | 1.0725 | 1.16573 | 0.20043 | 1 | -0.4651 | -1.3804 | 4.204 | 0.00068 | 0 | 0 |
| 1389565_at | --- | --- | uniqRT1u_243 | 0.16 | 0.19 | 0.06 | 0.12 | 0.50 | 0 | -0.129 | -1.0935 | 2.07631 | 0.08492 | 0 | -0.0755 | -1.0538 | 1.07718 | 0.15455 | 1 | 0.30236 | 1.23316 | 2.16302 | 0.01036 | 0 | 0 |
| 1389585_at | --- | --- | uniqRT1u_243 | 0.10 | 0.08 | -0.03 | -0.03 | -0.20 | 0 | -0.1121 | -1.0808 | 1.41892 | 0.19455 | 0 | -0.1059 | -1.0761 | 0.99957 | 0.27397 | 1 | -0.2847 | -1.2182 | 2.53014 | 0.02848 | 0 | 0 |
| 1389731_at | --- | --- | uniqRT1u_243 | 0.06 | 0.46 | 0.31 | 0.34 | 0.12 | 0 | -0.15 | -1.1096 | 1.64246 | 0.14751 | 0 | -0.1233 | -1.0892 | 1.01152 | 0.24947 | 1 | -0.3483 | -1.273 | 3.1274 | 0.01046 | 0 | 0 |
| 1390014_at | --- | --- | uniqRT1u_243 | 0.00 | 0.07 | 0.10 | -0.04 | -0.27 | 0 | 0.03003 | 1.02103 | 1.49692 | 0.15783 | 0 | -0.1141 | -1.0823 | 1.02795 | 0.22649 | 1 | -0.3418 | -1.2673 | 3.14541 | 0.0086 | 0 | 0 |
| 1390358_at | Cacna2d3 | calcium channel, voltage-dependent, alpha2/delta s | uniqRT1u_243 | 0.03 | -0.14 | 0.11 | -0.02 | 0.13 | 0 | 0.25725 | 1.1952 | 1.89543 | 0.06107 | 0 | 0.12035 | 1.087 | 1.06423 | 0.31925 | 1 | 0.27042 | 1.20616 | 2.26616 | 0.04343 | 0 | 0 |
| 1390503_at | --- | --- | uniqRT1u_243 | 0.22 | 0.00 | 0.26 | 0.17 | 0.33 | 0 | 0.25818 | 1.19597 | 2.23356 | 0.01551 | 0 | 0.162 | 1.11884 | 1.29505 | 0.06301 | 1 | 0.32348 | 1.25135 | 2.2584 | 0.01358 | 0 | 0 |
| 1390640_at | Chtf18 | CTF18, chromosome transmission fidelity factor 18 l | uniqRT1u_243 | 0.08 | 0.26 | 0.01 | 0.02 | -0.06 | 0 | -0.2509 | -1.19 | 2.47539 | 0.05365 | 0 | -0.2412 | -1.182 | 1.38813 | 0.05446 | 1 | -0.3174 | -1.2461 | 3.13551 | 0.01079 | 0 | 0 |
| 1390664_at | Tmem116 | transmembrane protein 116 | uniqRT1u_243 | -0.02 | -0.04 | -0.08 | -0.08 | -0.36 | 0 | -0.0349 | -1.0245 | 1.29837 | 0.24019 | 0 | -0.0378 | -1.0266 | 0.96182 | 0.51083 | 1 | -0.3141 | -1.2432 | 3.01337 | 0.01579 | 0 | 0 |
| 1390673_at | --- | --- | uniqRT1u_243 | 0.02 | -0.04 | 0.04 | -0.11 | 0.23 | 0 | 0.07293 | 1.05185 | 1.18661 | 0.29102 | 0 | -0.0733 | -1.0521 | 0.99064 | 0.28994 | 1 | 0.2687 | 1.20472 | 2.3144 | 0.03468 | 0 | 0 |
| 1390850_at | --- | --- | uniqRT1u_243 | -0.06 | -0.41 | -0.21 | -0.18 | -0.15 | 0 | 0.19994 | 1.14865 | 1.76345 | 0.08154 | 0 | 0.23851 | 1.17978 | 1.28173 | 0.08288 | 1 | 0.26836 | 1.20444 | 2.39262 | 0.02316 | 0 | 0 |
| 1390917_at | --- | --- | uniqRT1u_243 | -0.05 | -0.33 | -0.15 | -0.13 | -0.05 | 0 | 0.18218 | 1.1346 | 1.56567 | 0.1392 | 0 | 0.19726 | 1.14652 | 1.24159 | 0.11778 | 1 | 0.28085 | 1.21491 | 2.36181 | 0.02818 | 0 | 0 |
| 1390936_at | --- | --- | uniqRT1u_243 | 0.20 | 0.39 | 0.18 | 0.18 | 0.11 | 0 | -0.2078 | -1.1549 | 2.32968 | 0.06547 | 0 | -0.2142 | -1.1601 | 1.14298 | 0.11779 | 1 | -0.2835 | -1.2172 | 2.62131 | 0.02579 | 0 | 0 |
| 1391000_at | --- | --- | uniqRT1u_243 | 0.06 | -0.02 | -0.14 | -0.23 | -0.30 | 0 | -0.1143 | -1.0824 | 1.52007 | 0.17151 | 0 | -0.2098 | -1.1566 | 1.43738 | 0.04774 | 1 | -0.2821 | -1.2159 | 2.4643 | 0.02948 | 0 | 0 |
| 1391161_at | Whsc1 | Wolf-Hirschhorn syndrome candidate 1 | uniqRT1u_243 | 0.09 | 0.17 | 0.32 | 0.42 | 0.49 | 0 | 0.15118 | 1.11048 | 1.55341 | 0.14236 | 0 | 0.25329 | 1.19192 | 1.27634 | 0.05204 | 1 | 0.31672 | 1.24549 | 2.35445 | 0.01719 | 0 | 0 |
| 1391187_at | Ppl | periplakin | uniqRT1u_243 | 0.07 | -0.41 | -0.31 | -0.22 | -0.06 | 0 | 0.10097 | 1.0725 | 1.38062 | 0.20204 | 0 | 0.19556 | 1.14517 | 1.24575 | 0.11372 | 1 | 0.35465 | 1.27867 | 2.01881 | 0.00708 | 0 | 0 |
| 1391305_at | --- | --- | uniqRT1u_243 | -0.08 | -0.08 | -0.03 | -0.12 | -0.35 | 0 | 0.04967 | 1.03503 | 1.05587 | 0.38559 | 0 | -0.0429 | -1.0302 | 0.96502 | 0.43061 | 1 | -0.2737 | -1.2089 | 2.34316 | 0.03247 | 0 | 0 |
| 1391358_at | --- | --- | uniqRT1u_243 | 0.02 | -0.18 | -0.10 | -0.01 | 0.09 | 0 | 0.08545 | 1.06102 | 1.42823 | 0.18021 | 0 | 0.17358 | 1.12785 | 1.22031 | 0.132 | 1 | 0.27461 | 1.20967 | 2.37862 | 0.02501 | 0 | 0 |
| 1391411_at | RGD1560248 | similar to formin-like 2 isoform B | uniqRT1u_243 | 0.07 | -0.14 | 0.00 | 0.07 | 0.13 | 0 | 0.14036 | 1.10218 | 1.53416 | 0.14735 | 0 | 0.21951 | 1.16434 | 1.2812 | 0.07836 | 1 | 0.27808 | 1.21258 | 2.29446 | 0.01837 | 0 | 0 |
| 1391462_at | Ebpl | emopamil binding protein-like | uniqRT1u_243 | 0.11 | 0.35 | 0.20 | 0.13 | 0.04 | 0 | -0.1576 | -1.1154 | 2.0157 | 0.09275 | 0 | -0.2248 | -1.1686 | 1.6103 | 0.03335 | 1 | -0.3113 | -1.2408 | 3.04221 | 0.01105 | 0 | 0 |
| 1391934_at | --- | --- | uniqRT1u_243 | 0.06 | -0.05 | 0.02 | -0.05 | 0.22 | 0 | 0.07132 | 1.05068 | 1.01312 | 0.43214 | 0 | 0.00067 | 1.00046 | 0.96154 | 0.61077 | 1 | 0.27566 | 1.21055 | 2.36838 | 0.03016 | 0 | 0 |
| 1392084_at | --- | --- | uniqRT1u_243 | 0.29 | 0.18 | 0.42 | 0.22 | 0.51 | 0 | 0.24477 | 1.1849 | 1.85352 | 0.06508 | 0 | 0.04119 | 1.02897 | 1.02966 | 0.39237 | 1 | 0.33041 | 1.25737 | 2.16153 | 0.01091 | 0 | 0 |
| 1392255_at | --- | --- | uniqRT1u_243 | 0.12 | 0.22 | 0.06 | -0.05 | -0.06 | 0 | -0.1621 | -1.1189 | 1.53505 | 0.16827 | 0 | -0.2644 | -1.2011 | 1.33314 | 0.05911 | 1 | -0.2755 | -1.2105 | 2.26129 | 0.03505 | 0 | 0 |
| 1392311_at | --- | --- | uniqRT1u_243 | -0.08 | 0.29 | 0.50 | 0.32 | 0.56 | 0 | 0.21561 | 1.16119 | 1.76461 | 0.08177 | 0 | 0.03193 | 1.02238 | 1.07201 | 0.30379 | 1 | 0.27036 | 1.20611 | 2.40295 | 0.02728 | 0 | 0 |
| 1392341_at | --- | --- | uniqRT1u_243 | 0.07 | 0.01 | 0.12 | 0.11 | 0.28 | 0 | 0.11332 | 1.08171 | 1.28781 | 0.23687 | 0 | 0.1069 | 1.07691 | 1.14467 | 0.21911 | 1 | 0.27001 | 1.20582 | 2.29682 | 0.03486 | 0 | 0 |
| 1392547_at | MGC105649 | hypothetical LOC302884 | uniqRT1u_243 | -0.04 | -1.03 | -0.99 | -0.87 | -0.69 | 0 | 0.03686 | 1.02588 | 1.3414 | 0.21523 | 0 | 0.15502 | 1.11344 | 1.23568 | 0.12476 | 1 | 0.33427 | 1.26074 | 2.06465 | 0.00757 | 0 | 0 |
| 1393060_at | Adamtsl2 | ADAMTS-like 2 | uniqRT1u_243 | 0.05 | -0.07 | -0.19 | -0.29 | -0.37 | 0 | -0.1181 | -1.0853 | 1.97837 | 0.09981 | 0 | -0.2169 | -1.1623 | 1.402 | 0.0522 | 1 | -0.299 | -1.2303 | 3.02366 | 0.00797 | 0 | 0 |
| 1393185_at | --- | --- | uniqRT1u_243 | -0.14 | 0.06 | -0.19 | 0.30 | 0.35 | 0 | -0.2565 | -1.1945 | 2.8808 | 0.03261 | 0 | 0.24095 | 1.18177 | 1.29134 | 0.06465 | 1 | 0.28866 | 1.22151 | 2.33572 | 0.02273 | 0 | 0 |
| 1393418_at | Tmod2 | tropomodulin 2 | uniqRT1u_243 | -0.22 | -0.06 | -0.22 | -0.26 | -0.36 | 0 | -0.1594 | -1.1168 | 2.27272 | 0.06986 | 0 | -0.1973 | -1.1465 | 1.31106 | 0.06265 | 1 | -0.3004 | -1.2315 | 3.43594 | 0.00354 | 0 | 0 |
| 1393474_at | Sult2b1 | sulfotransferase family, cytosolic, 2B, member 1 | uniqRT1u_243 | 0.24 | 0.30 | 0.10 | 0.06 | 0.01 | 0 | -0.202 | -1.1503 | 2.49493 | 0.05335 | 0 | -0.2322 | -1.1746 | 1.36107 | 0.05668 | 1 | -0.2886 | -1.2215 | 3.07745 | 0.01089 | 0 | 0 |
| 1393518_at | Wfdc5 | WAP four-disulfide core domain 5 | uniqRT1u_243 | 0.02 | 0.25 | 0.00 | 0.02 | -0.07 | 0 | -0.2589 | -1.1966 | 3.30651 | 0.01861 | 0 | -0.2367 | -1.1783 | 1.35212 | 0.05735 | 1 | -0.3197 | -1.2481 | 2.99607 | 0.01175 | 0 | 0 |
| 1393522_at | --- | --- | uniqRT1u_243 | 0.00 | 0.20 | -0.02 | -0.01 | -0.10 | 0 | -0.2235 | -1.1675 | 2.88442 | 0.03524 | 0 | -0.2103 | -1.1569 | 1.22561 | 0.08276 | 1 | -0.3022 | -1.233 | 3.05676 | 0.01091 | 0 | 0 |
| 1393599_at | LOC690020 | similar to killer cell lectin-like receptor, subfamily A, | uniqRT1u_243 | -0.05 | -0.54 | -0.43 | -0.33 | -0.27 | 0 | 0.11521 | 1.08313 | 1.57965 | 0.13511 | 0 | 0.21872 | 1.1637 | 1.27285 | 0.04703 | 1 | 0.27781 | 1.21235 | 2.35243 | 0.01649 | 0 | 0 |
| 1393612_a_at | Depdc7 | DEP domain containing 7 | uniqRT1u_243 | 0.09 | -0.06 | -0.02 | 0.00 | 0.22 | 0 | 0.03843 | 1.027 | 1.04217 | 0.40572 | 0 | 0.05595 | 1.03955 | 1.08419 | 0.29051 | 1 | 0.2728 | 1.20815 | 2.31518 | 0.01883 | 0 | 0 |
| 1393729_at | Neto2 | neuropilin (NRP) and tolloid (TLL)-like 2 | uniqRT1u_243 | 0.21 | 0.25 | 0.25 | 0.12 | -0.07 | 0 | -0.003 | -1.0021 | 1.00585 | 0.41856 | 0 | -0.1368 | -1.0995 | 1.03557 | 0.20579 | 1 | -0.3249 | -1.2526 | 2.92187 | 0.01456 | 0 | 0 |
| 1393734_at | Timm10b | translocase of inner mitochondrial membrane 10 hc | uniqRT1u_243 | -0.12 | -0.06 | -0.19 | -0.13 | -0.35 | 0 | -0.1315 | -1.0954 | 1.67037 | 0.14029 | 0 | -0.0659 | -1.0468 | 0.99521 | 0.28241 | 1 | -0.2913 | -1.2238 | 2.99986 | 0.0136 | 0 | 0 |
| 1393882_at | LOC689618 | similar to Protein C20orf85 homolog | uniqRT1u_243 | -0.05 | -0.07 | -0.09 | -0.29 | -0.34 | 0 | -0.0137 | -1.0095 | 1.05974 | 0.36731 | 0 | -0.2152 | -1.1609 | 1.4847 | 0.04258 | 1 | -0.2663 | -1.2027 | 2.87413 | 0.02061 | 0 | 0 |
| 1394027_at | Nup62cl | nucleoporin 62 C-terminal like | uniqRT1u_243 | -0.03 | -0.06 | -0.28 | -0.29 | -0.39 | 0 | -0.2153 | -1.161 | 2.39484 | 0.06184 | 0 | -0.2282 | -1.1714 | 1.20095 | 0.09148 | 1 | -0.3289 | -1.2561 | 2.92938 | 0.00763 | 0 | 0 |
| 1394186_at | --- | --- | uniqRT1u_243 | -0.13 | -0.09 | -0.19 | -0.33 | -0.37 | 0 | -0.0942 | -1.0675 | 1.39941 | 0.20357 | 0 | -0.2326 | -1.1749 | 1.16829 | 0.10519 | 1 | -0.2778 | -1.2123 | 2.89472 | 0.01973 | 0 | 0 |
| 1394187_at | Lmod3 | leiomodin 3 (fetal) | uniqRT1u_243 | -0.09 | -0.06 | -0.09 | -0.15 | -0.33 | 0 | -0.0371 | -1.0261 | 0.97892 | 0.44286 | 0 | -0.0932 | -1.0667 | 0.97053 | 0.35483 | 1 | -0.2736 | -1.2088 | 3.10422 | 0.01088 | 0 | 0 |
| 1394209_at | --- | --- | uniqRT1u_243 | -0.28 | -0.30 | -0.16 | -0.25 | -0.03 | 0 | 0.13415 | 1.09744 | 1.54935 | 0.14363 | 0 | 0.04431 | 1.03119 | 1.06967 | 0.30973 | 1 | 0.26362 | 1.20048 | 2.30801 | 0.04112 | 0 | 0 |
| 1394270_at | Mks1 | Meckel syndrome, type 1 | uniqRT1u_243 | 0.04 | 0.11 | 0.14 | -0.12 | -0.17 | 0 | 0.02812 | 1.01969 | 1.01822 | 0.42616 | 0 | -0.2288 | -1.1719 | 1.20457 | 0.08781 | 1 | -0.2837 | -1.2173 | 2.40622 | 0.03072 | 0 | 0 |
| 1394354_at | Lppr2 | lipid phosphate phosphatase-related protein type 2 | uniqRT1u_243 | -0.02 | 0.03 | -0.01 | -0.15 | -0.25 | 0 | -0.0394 | -1.0277 | 1.14259 | 0.31343 | 0 | -0.1807 | -1.1334 | 1.16789 | 0.12842 | 1 | -0.2862 | -1.2195 | 2.99878 | 0.01581 | 0 | 0 |
| 1394582_at | --- | --- | uniqRT1u_243 | 0.02 | -0.13 | -0.07 | 0.03 | 0.18 | 0 | 0.06241 | 1.04421 | 1.03522 | 0.41353 | 0 | 0.16054 | 1.1177 | 1.18008 | 0.18153 | 1 | 0.31195 | 1.24139 | 2.32792 | 0.01617 | 0 | 0 |
| 1394595_at | Stim1 | stromal interaction molecule 1 | uniqRT1u_243 | 0.03 | 0.16 | 0.08 | 0.04 | -0.14 | 0 | -0.0795 | -1.0566 | 1.18033 | 0.29395 | 0 | -0.1137 | -1.082 | 1.01475 | 0.2411 | 1 | -0.2937 | -1.2258 | 2.54557 | 0.02808 | 0 | 0 |
| 1394631_at | --- | --- | uniqRT1u_243 | 0.04 | -0.18 | -0.20 | -0.24 | 0.11 | 0 | -0.0155 | -1.0108 | 0.92421 | 0.51659 | 0 | -0.0541 | -1.0382 | 0.99928 | 0.26946 | 1 | 0.28991 | 1.22257 | 2.35399 | 0.02218 | 0 | 0 |
| 1394646_at | --- | --- | uniqRT1u_243 | -0.10 | -0.29 | -0.18 | -0.09 | 0.02 | 0 | 0.11444 | 1.08255 | 1.47475 | 0.16574 | 0 | 0.20357 | 1.15155 | 1.26056 | 0.10052 | 1 | 0.30954 | 1.23932 | 2.08333 | 0.00864 | 0 | 0 |
| 1394690_at | --- | --- | uniqRT1u_243 | -0.12 | -0.23 | -0.16 | -0.10 | 0.05 | 0 | 0.06893 | 1.04894 | 1.28483 | 0.2378 | 0 | 0.12938 | 1.09382 | 1.17209 | 0.18743 | 1 | 0.27472 | 1.20976 | 2.16871 | 0.00976 | 0 | 0 |
| 1394699_at | --- | --- | uniqRT1u_243 | 0.10 | -0.22 | -0.19 | -0.11 | 0.07 | 0 | 0.02437 | 1.01704 | 0.94473 | 0.51685 | 0 | 0.10237 | 1.07354 | 1.10533 | 0.2633 | 1 | 0.28944 | 1.22216 | 2.36812 | 0.02026 | 0 | 0 |
| 1394785_at | --- | --- | uniqRT1u_243 | 0.05 | 0.34 | 0.49 | 0.50 | 0.74 | 0 | 0.14381 | 1.10482 | 1.7677 | 0.07821 | 0 | 0.15855 | 1.11617 | 1.25274 | 0.11013 | 1 | 0.39558 | 1.31547 | 1.26866 | 0.00273 | 0 | 0 |
| 1395109_at | --- | --- | uniqRT1u_243 | -0.04 | -0.23 | -0.15 | -0.28 | 0.09 | 0 | 0.07872 | 1.05608 | 1.06184 | 0.38289 | 0 | -0.0495 | -1.0349 | 0.96551 | 0.40708 | 1 | 0.31192 | 1.24136 | 2.35315 | 0.02066 | 0 | 0 |
| 1395204_at | --- | --- | uniqRT1u_243 | 0.00 | 0.06 | -0.06 | -0.17 | -0.27 | 0 | -0.1245 | -1.0901 | 1.69788 | 0.13731 | 0 | -0.229 | -1.172 | 1.18146 | 0.0995 | 1 | -0.3283 | -1.2555 | 3.01802 | 0.01669 | 0 | 0 |

| Affymetrix Probeset ID | Gene Symbol | Gene Title | Figure 6 B Venn n =2021 | Mean BN log2 intensity autologous n=8 | Mean log2 intensity day 30 DR +/+ ND n=4 | Mean log2 intensity day 30 DR+/+ HCD n=4 | Mean log2 intensity day 30 DR+/+ B/S n=4 | Mean log2 intensity day 30 F+/+ ND n=4 | DR+/+ HCD vs DR+/+ ND n=380 1=yes 0=no | Log2 ratio DR+/+ HCD vs DR+/+ ND | Fold-Change DR+/+ HCD vs DR+/+ ND | RP-FDR 4DR30 HCD vs 4DR30 Reg | p value DR+/+ HCD vs DR+/+ ND | DR+/+ B/S vs DR+/+ ND n=1,330 1=yes 0=no | Log2 ratio DR+/+ B/S vs DR+/+ ND | Fold-Change DR+/+ B/S vs DR+/+ ND | RP-FDR 4DR30 Antib vs 4DR30 Reg | p value DR+/+ B/S vs DR+/+ ND | DR+/+ ND vs F+/+ ND n=398 1=yes 0=no | Log2 ratio DR+/+ ND vs F+/+ ND | Fold-Change DR+/+ ND vs F+/+ ND | RP-FDR 4RT1uu 30Reg vs 4DR30 Reg | p value DR+/+ ND vs F+/+ ND | DR+/+ Longitudinal n=228 1=yes 0=no | Figure 6F |
|---|---|---|---|---|---|---|---|---|---|---|---|---|---|---|---|---|---|---|---|---|---|---|---|---|---|
| 1395205_at | --- | --- | uniqRT1u_243 | -0.10 | 0.19 | 0.21 | 0.04 | -0.09 | 0 | 0.01487 | 1.01036 | 1.2414 | 0.2611 | 0 | -0.1496 | -1.1093 | 1.21265 | 0.08532 | 1 | -0.2875 | -1.2205 | 2.73733 | 0.02209 | 0 | 0 |
| 1395369_at | --- | --- | uniqRT1u_243 | -0.18 | -0.34 | -0.11 | -0.40 | -0.06 | 0 | 0.22933 | 1.17229 | 2.05356 | 0.03526 | 0 | -0.0568 | -1.0402 | 0.97056 | 0.35409 | 1 | 0.2807 | 1.21478 | 2.30311 | 0.01785 | 0 | 0 |
| 1395390_at | --- | --- | uniqRT1u_243 | -0.06 | -0.13 | -0.07 | -0.16 | 0.14 | 0 | 0.05515 | 1.03897 | 0.95327 | 0.50525 | 0 | -0.0331 | -1.0232 | 0.96262 | 0.4908 | 1 | 0.26495 | 1.2016 | 2.36734 | 0.02375 | 0 | 0 |
| 1395446_at | --- | --- | uniqRT1u_243 | 0.05 | -0.45 | -0.28 | -0.32 | -0.16 | 0 | 0.17109 | 1.12591 | 1.39052 | 0.19589 | 0 | 0.12462 | 1.09022 | 1.1158 | 0.25237 | 1 | 0.2871 | 1.22018 | 2.29256 | 0.03487 | 0 | 0 |
| 1395472_at | Lrrc17 | leucine rich repeat containing 17 | uniqRT1u_243 | 0.29 | -0.09 | 0.01 | 0.06 | 0.19 | 0 | 0.10543 | 1.07582 | 1.36333 | 0.20718 | 0 | 0.15159 | 1.1108 | 1.21195 | 0.14435 | 1 | 0.28628 | 1.21949 | 2.33451 | 0.01749 | 0 | 0 |
| 1395679_at | LOC681924 | hypothetical protein LOC681924 | uniqRT1u_243 | 0.08 | 0.19 | 0.00 | 0.06 | -0.16 | 0 | -0.185 | -1.1368 | 3.07653 | 0.0274 | 0 | -0.1282 | -1.0929 | 1.18186 | 0.09873 | 1 | -0.3523 | -1.2766 | 3.90292 | 0.00301 | 0 | 0 |
| 1395769_at | --- | --- | uniqRT1u_243 | 0.10 | -0.16 | 0.02 | 0.05 | 0.11 | 0 | 0.18411 | 1.13612 | 1.7854 | 0.07435 | 0 | 0.2112 | 1.15765 | 1.27841 | 0.08098 | 1 | 0.27376 | 1.20896 | 2.33557 | 0.0344 | 0 | 0 |
| 1395805_at | Prickle1 | prickle homolog 1 (Drosophila) | uniqRT1u_243 | -0.07 | -0.02 | -0.20 | -0.14 | -0.32 | 0 | -0.184 | -1.1361 | 2.31007 | 0.06722 | 0 | -0.129 | -1.0936 | 1.04515 | 0.19159 | 1 | -0.3058 | -1.2361 | 2.98935 | 0.01192 | 0 | 0 |
| 1395909_at | --- | --- | uniqRT1u_243 | 0.23 | 0.21 | 0.13 | 0.29 | 0.51 | 0 | -0.0792 | -1.0565 | 1.33987 | 0.22374 | 0 | 0.07941 | 1.05659 | 1.07211 | 0.30599 | 1 | 0.30344 | 1.23409 | 2.06744 | 0.00831 | 0 | 0 |
| 1395985_at | Spg7 | spastic paraplegia 7 homolog (human) | uniqRT1u_243 | -0.11 | 0.01 | 0.25 | 0.28 | 0.32 | 0 | 0.23621 | 1.17789 | 1.7608 | 0.08442 | 0 | 0.26558 | 1.20212 | 1.28968 | 0.05607 | 1 | 0.31042 | 1.24007 | 2.37386 | 0.02015 | 0 | 0 |
| 1395994_at | --- | --- | uniqRT1u_243 | -0.11 | 0.00 | 0.26 | 0.24 | 0.29 | 0 | 0.2549 | 1.19325 | 2.07393 | 0.03328 | 0 | 0.2357 | 1.17748 | 1.25662 | 0.03693 | 1 | 0.28885 | 1.22167 | 2.34974 | 0.0207 | 0 | 0 |
| 1396026_at | --- | --- | uniqRT1u_243 | 0.14 | -0.04 | 0.20 | 0.19 | 0.24 | 0 | 0.23629 | 1.17796 | 1.8938 | 0.06065 | 0 | 0.22539 | 1.1691 | 1.28089 | 0.0832 | 1 | 0.2738 | 1.20899 | 2.36574 | 0.02944 | 0 | 0 |
| 1396089_at | LOC683522 | similar to Transcriptional enhancer factor TEF-3 (TEA | uniqRT1u_243 | -0.01 | -0.18 | -0.09 | -0.18 | 0.13 | 0 | 0.08701 | 1.06217 | 1.31132 | 0.22445 | 0 | -0.0057 | -1.004 | 1.08408 | 0.28968 | 1 | 0.30198 | 1.23283 | 2.24315 | 0.01421 | 0 | 0 |
| 1396095_at | --- | --- | uniqRT1u_243 | 0.02 | -0.06 | -0.02 | 0.01 | 0.29 | 0 | 0.04209 | 1.02961 | 1.05016 | 0.39475 | 0 | 0.0731 | 1.05197 | 1.05658 | 0.33496 | 1 | 0.35738 | 1.2811 | 1.90327 | 0.00636 | 0 | 0 |
| 1396225_at | --- | --- | uniqRT1u_243 | -0.03 | 0.60 | 0.38 | 0.79 | 0.21 | 0 | -0.2192 | -1.1641 | 1.34857 | 0.0003 | 0 | 0.19577 | 1.14534 | 3.4665 | 0.00446 | 1 | -0.3842 | -1.3052 | 0.56 | 1.8E-05 | 0 | 0 |
| 1396631_at | --- | --- | uniqRT1u_243 | 0.19 | 0.16 | 0.03 | -0.01 | -0.11 | 0 | -0.1368 | -1.0994 | 1.26059 | 0.25241 | 0 | -0.1788 | -1.132 | 1.14454 | 0.11711 | 1 | -0.2736 | -1.2088 | 2.22494 | 0.03835 | 0 | 0 |
| 1396747_at | --- | --- | uniqRT1u_243 | -0.05 | -0.11 | -0.13 | -0.19 | -0.39 | 0 | -0.0245 | -1.0172 | 0.92011 | 0.5235 | 0 | -0.0868 | -1.062 | 0.99045 | 0.28979 | 1 | -0.2843 | -1.2179 | 2.53278 | 0.02842 | 0 | 0 |
| 1396851_at | --- | --- | uniqRT1u_243 | -0.02 | -0.36 | -0.29 | -0.26 | -0.09 | 0 | 0.06787 | 1.04817 | 1.07487 | 0.371 | 0 | 0.09381 | 1.06718 | 1.05603 | 0.33403 | 1 | 0.26384 | 1.20067 | 2.27492 | 0.03628 | 0 | 0 |
| 1397282_at | --- | --- | uniqRT1u_243 | -0.07 | 0.07 | 0.13 | -0.04 | -0.27 | 0 | 0.05919 | 1.04188 | 1.14134 | 0.32047 | 0 | -0.1171 | -1.0845 | 1.03715 | 0.20464 | 1 | -0.3471 | -1.272 | 4.51625 | 0.00116 | 0 | 0 |
| 1397400_at | --- | --- | uniqRT1u_243 | 0.05 | 0.06 | -0.06 | -0.20 | -0.31 | 0 | -0.1236 | -1.0895 | 1.87057 | 0.11404 | 0 | -0.2569 | -1.1949 | 1.41668 | 0.05056 | 1 | -0.3667 | -1.2894 | 2.95363 | 0.0076 | 0 | 0 |
| 1397456_at | Zfp827 | zinc finger protein 827 | uniqRT1u_243 | -0.03 | 0.07 | -0.04 | -0.06 | -0.19 | 0 | -0.1141 | -1.0823 | 1.36909 | 0.21448 | 0 | -0.1267 | -1.0918 | 1.00195 | 0.26467 | 1 | -0.2633 | -1.2002 | 2.47878 | 0.02885 | 0 | 0 |
| 1397542_at | --- | --- | uniqRT1u_243 | -0.11 | -0.26 | -0.17 | -0.11 | 0.06 | 0 | 0.08405 | 1.05999 | 1.13136 | 0.328 | 0 | 0.14663 | 1.10698 | 1.23258 | 0.12287 | 1 | 0.32242 | 1.25043 | 2.0655 | 0.00797 | 0 | 0 |
| 1397672_at | --- | --- | uniqRT1u_243 | -0.07 | 0.25 | 0.13 | 0.05 | -0.02 | 0 | -0.1228 | -1.0889 | 1.35883 | 0.2176 | 0 | -0.2024 | -1.1506 | 1.14874 | 0.11355 | 1 | -0.2725 | -1.2079 | 3.03737 | 0.01113 | 0 | 0 |
| 1397690_at | --- | --- | uniqRT1u_243 | 0.15 | -0.03 | 0.17 | 0.02 | 0.33 | 0 | 0.20192 | 1.15023 | 1.96496 | 0.05181 | 0 | 0.05446 | 1.03847 | 1.12407 | 0.23892 | 1 | 0.36364 | 1.28667 | 1.55562 | 0.00365 | 0 | 0 |
| 1397758_at | --- | --- | uniqRT1u_243 | 0.00 | 0.18 | 0.32 | 0.37 | 0.46 | 0 | 0.14247 | 1.10379 | 1.75439 | 0.09004 | 0 | 0.1912 | 1.14172 | 1.27408 | 0.05133 | 1 | 0.27748 | 1.21208 | 2.2519 | 0.01369 | 0 | 0 |
| 1397774_at | Fam63b | family with sequence similarity 63, member B | uniqRT1u_243 | -0.01 | 0.21 | 0.11 | 0.21 | -0.06 | 0 | -0.0939 | -1.0673 | 1.54093 | 0.16654 | 0 | 0.00637 | 1.00443 | 0.98226 | 0.30836 | 1 | -0.2643 | -1.2011 | 3.0138 | 0.01173 | 0 | 0 |
| 1397923_at | --- | --- | uniqRT1u_243 | 0.21 | 0.26 | 0.14 | 0.10 | -0.01 | 0 | -0.1238 | -1.0896 | 2.23665 | 0.07199 | 0 | -0.1607 | -1.1178 | 1.28471 | 0.07238 | 1 | -0.2703 | -1.2061 | 2.97957 | 0.00661 | 0 | 0 |
| 1398009_at | --- | --- | uniqRT1u_243 | -0.19 | -0.08 | -0.08 | -0.26 | -0.35 | 0 | -0.0081 | -1.0056 | 0.99248 | 0.43042 | 0 | -0.1794 | -1.1324 | 1.07806 | 0.15575 | 1 | -0.2758 | -1.2107 | 2.46768 | 0.02904 | 0 | 0 |
| 1398159_at | Klf5 | Kruppel-like factor 5 | uniqRT1u_243 | 0.07 | 0.13 | -0.08 | -0.04 | -0.14 | 0 | -0.2123 | -1.1586 | 2.09074 | 0.08256 | 0 | -0.1698 | -1.1249 | 1.03714 | 0.20303 | 1 | -0.2631 | -1.2 | 2.09774 | 0.0454 | 0 | 0 |
| 1398502_at | --- | --- | uniqRT1u_243 | 0.20 | 0.06 | 0.13 | -0.02 | 0.37 | 0 | 0.07078 | 1.05029 | 1.15458 | 0.3106 | 0 | -0.078 | -1.0555 | 0.97492 | 0.32966 | 1 | 0.3137 | 1.24289 | 2.31513 | 0.01757 | 0 | 0 |
| 1398507_at | --- | --- | uniqRT1u_243 | -0.09 | -0.87 | -1.03 | -0.70 | -1.13 | 0 | -0.1586 | -1.1162 | 3.14708 | 0.02287 | 0 | 0.16484 | 1.12104 | 1.07618 | 0.01841 | 1 | -0.264 | -1.2008 | 4.589 | 0.00148 | 0 | 0 |
| 1398525_at | --- | --- | uniqRT1u_243 | 0.00 | 0.08 | 0.03 | -0.05 | -0.22 | 0 | -0.0548 | -1.0387 | 1.12129 | 0.32558 | 0 | -0.137 | -1.0996 | 0.99945 | 0.27404 | 1 | -0.299 | -1.2303 | 2.94815 | 0.01431 | 0 | 0 |
| 1398629_at | --- | --- | uniqRT1u_243 | -0.04 | 0.11 | -0.03 | 0.08 | -0.21 | 0 | -0.1473 | -1.1075 | 2.53615 | 0.04958 | 0 | -0.0327 | -1.0229 | 1.05118 | 0.18161 | 1 | -0.3258 | -1.2534 | 4.3425 | 0.00168 | 0 | 0 |
| 1399056_at | Pdss2 | prenyl (decaprenyl) diphosphate synthase, subunit 2 | uniqRT1u_243 | 0.08 | -0.18 | -0.05 | 0.00 | 0.12 | 0 | 0.12943 | 1.09386 | 1.30976 | 0.22545 | 0 | 0.1854 | 1.13713 | 1.23255 | 0.1258 | 1 | 0.30068 | 1.23173 | 2.32915 | 0.02105 | 0 | 0 |

**Data are available as a sortable spreadsheet upon request**

[1] **Data are expressed in a binar fashion as meeting (1) or not meeting (0) the query defined in the header.**
