# Supplementary material for: Modulation of the diet and gastrointestinal microbiota normalizes systemic inflammation and β-cell chemokine expression associated with autoimmune diabetes susceptibility
Source: PLoS One. 2018 Jan 2;13(1):e0190351. doi: 10.1371/journal.pone.0190351 (PMC5749787; doi:10.1371/journal.pone.0190351)
Supplement: S5 Table — (PDF) [file pone.0190351.s005.pdf]

S5 Table: Islet Gene Expression Analysis of DR+/+ND, DR+/+HCD, DR+/+B/S, and F+/+ Islets at 40 days of Age [1]

Affymetrix Probeset ID	Gene Symbol	Gene Title	Figure 7 A Venn n=2289	Present in any 2 analyses n=423 1=yes 0=no	Mean log2 intensity DR+/+ ND	Mean log2 intensity DR+/+ HCD	Mean log2 intensity DR+/+ B/S	Mean log2 intensity F+/+ day 40 ND	DR+/+HCD vs DR+/+ND FDR<10% Log2 ratio >0.5 n=636	Log2 ratio DR+/+HCD vs DR+/+ ND	Fold-Change DR+/+H CD vs DR+/+ ND	FDR DR+/+H CD vs DR+/+ ND	DR+/+B/S vs DR+/+ND Log2 ratio >0.5 n=350	Log2 ratio DR+/+ B/S vs DR+/+ ND	Fold-Change DR+/+B /S vs DR+/+ ND	FDR DR+/+B /S vs DR+/+ ND	F+/+ vs DR+/+N D Log2 ratio >0.5 n=1209	Log2 ratio F+/+ vs DR+/+ ND	Fold-Change F+/+ vs DR+/+ ND	FDR F+/+ vs DR+/+ ND
1398390_at	Cxcl13	chemokine (C-X-C motif) ligand 13	Any2_423	1	5.87	4.20	4.98	4.08	1	-1.678	-3.199	0.000	1	-0.896	-1.861	0.013	1	-1.793	-3.465	0.000
1378015_at	Ccl21	chemokine (C-C motif) ligand 21	Any2_423	1	6.43	4.67	5.09	4.54	1	-1.767	-3.404	0.000	1	-1.340	-2.532	0.000	1	-1.899	-3.729	0.000
1368007_at	Dmbt1 /// LO	deleted in malignant brain tumors 1 /// deleted in m	Any2_423	1	7.77	5.76	5.61	5.87	1	-2.007	-4.019	0.000	1	-2.157	-4.459	0.000	1	-1.893	-3.714	0.000
1373970_at	Il33	interleukin 33	Any2_423	1	6.58	4.83	4.82	4.55	1	-1.752	-3.369	0.000	1	-1.757	-3.380	0.000	1	-2.029	-4.080	0.000
1368337_at	Glycam1	glycosylation dependent cell adhesion molecule 1	Any2_423	1	6.13	5.60	6.58	5.50	1	-0.526	-1.440	0.060	0	0.453	1.369	0.216	1	-0.631	-1.548	0.048
1387319_at	Ccl11	chemokine (C-C motif) ligand 11	Any2_423	1	5.19	4.60	4.99	4.47	1	-0.587	-1.502	0.026	0	-0.200	-1.149	0.736	1	-0.717	-1.643	0.014
1370234_at	Fn1	fibronectin 1	Any2_423	1	7.70	7.12	6.90	6.77	1	-0.579	-1.493	0.033	1	-0.808	-1.751	0.000	1	-0.930	-1.906	0.003
1367577_at	Hspb1	heat shock protein 1	Any2_423	1	8.92	8.55	8.18	8.14	0	-0.371	-1.293	0.307	1	-0.738	-1.668	0.001	1	-0.780	-1.718	0.009
1375170_at	S100a11	S100 calcium binding protein A11	Any2_423	1	8.85	8.55	8.26	7.85	0	-0.293	-1.225	0.457	1	-0.588	-1.503	0.018	1	-0.992	-1.989	0.002
1372013_at	Ifitm1	interferon induced transmembrane protein 1	Any2_423	1	7.40	6.96	6.96	6.54	0	-0.437	-1.354	0.193	0	-0.432	-1.349	0.172	1	-0.853	-1.806	0.006
1367846_at	S100a4	S100 calcium-binding protein A4	Any2_423	1	7.47	6.93	6.94	6.32	1	-0.544	-1.458	0.054	1	-0.535	-1.449	0.043	1	-1.151	-2.221	0.001
1367940_at	Cxcr7	chemokine (C-X-C motif) receptor 7	Any2_423	1	7.26	6.67	6.60	6.13	1	-0.585	-1.500	0.034	1	-0.659	-1.579	0.007	1	-1.127	-2.184	0.001
1367614_at	Anxa1	annexin A1	Any2_423	1	8.71	7.63	7.73	7.43	1	-1.077	-2.109	0.000	1	-0.977	-1.968	0.000	1	-1.280	-2.428	0.000
1368474_at	LOC10091247	vascular cell adhesion protein 1-like /// vascular cell	Any2_423	1	5.04	4.07	4.26	3.51	1	-0.975	-1.966	0.000	1	-0.785	-1.723	0.002	1	-1.539	-2.905	0.000
1367628_at	Lgals1	lectin, galactoside-binding, soluble, 1	Any2_423	1	7.55	6.71	6.54	6.11	1	-0.838	-1.788	0.000	1	-1.015	-2.021	0.000	1	-1.437	-2.707	0.000
1387969_at	Cxcl10	chemokine (C-X-C motif) ligand 10	Any2_423	1	6.67	5.96	5.48	5.37	1	-0.708	-1.633	0.007	1	-1.186	-2.275	0.000	1	-1.299	-2.460	0.000
1370631_at	Reg3g	regenerating islet-derived 3 gamma	Any2_423	1	4.78	4.12	4.40	4.46	1	-0.660	-1.580	0.023	0	-0.375	-1.297	0.235	0	-0.321	-1.249	0.421
1379935_at	Ccl7	chemokine (C-C motif) ligand 7	Any2_423	1	4.68	4.14	4.17	4.42	1	-0.543	-1.457	0.056	1	-0.514	-1.428	0.064	0	-0.261	-1.198	0.616
1369191_at	Il6	interleukin 6	Any2_423	1	3.53	2.89	2.98	3.15	1	-0.635	-1.553	0.020	1	-0.552	-1.466	0.046	0	-0.377	-1.298	0.308
1391925_at	Ccl19	chemokine (C-C motif) ligand 19	Any2_423	1	5.08	4.35	4.44	4.88	1	-0.732	-1.661	0.003	1	-0.641	-1.559	0.007	0	-0.202	-1.151	0.737
1387202_at	Icam1	intercellular adhesion molecule 1	Any2_423	1	5.68	5.07	4.82	5.01	1	-0.613	-1.530	0.026	1	-0.865	-1.822	0.000	1	-0.667	-1.588	0.033
1368527_at	Ptgs2	prostaglandin-endoperoxide synthase 2	Any2_423	1	3.46	3.04	2.81	3.05	0	-0.423	-1.340	0.144	1	-0.652	-1.571	0.013	0	-0.407	-1.326	0.247
1367973_at	Ccl2	chemokine (C-C motif) ligand 2	Any2_423	1	4.93	3.87	3.87	4.04	1	-1.062	-2.087	0.000	1	-1.067	-2.095	0.000	1	-0.895	-1.860	0.008
1367661_at	S100a6	S100 calcium binding protein A6	Any2_423	1	8.27	7.44	7.48	7.56	1	-0.832	-1.780	0.000	1	-0.786	-1.724	0.001	1	-0.707	-1.633	0.018
1368238_at	Reg3b	regenerating islet-derived 3 beta	Any2_423	1	5.17	4.57	4.13	5.11	1	-0.601	-1.516	0.035	1	-1.032	-2.045	0.000	0	-0.058	-1.041	0.977
1369268_at	Atf3	activating transcription factor 3	Any2_423	1	7.92	7.72	6.04	7.40	0	-0.206	-1.154	0.371	1	-1.886	-3.697	0.000	0	-0.524	-1.438	0.146
1387316_at	Cxcl1	chemokine (C-X-C motif) ligand 1 (melanoma growth	Any2_423	1	7.67	6.30	4.88	6.02	1	-1.371	-2.587	0.000	1	-2.791	-6.920	0.000	1	-1.653	-3.145	0.000
1378032_at	Nfkbiz	nuclear factor of kappa light polypeptide gene enha	Any2_423	1	8.19	8.78	7.62	8.70	0	0.589	1.504	0.172	1	-0.574	-1.489	0.059	0	0.503	1.418	0.379
1368147_at	Dusp1	dual specificity phosphatase 1	Any2_423	1	7.43	8.30	6.85	7.79	1	0.869	1.826	0.027	1	-0.585	-1.500	0.040	0	0.358	1.281	0.535
1370912_at	Hspa1a	heat shock 70kD protein 1A	Any2_423	1	10.46	11.34	7.08	8.86	1	0.881	1.841	0.011	1	-3.374	-10.371	0.000	1	-1.600	-3.032	0.000
1367570_at	Tagln	transgelin	Any2_423	1	8.08	7.35	6.84	5.45	1	-0.729	-1.657	0.006	1	-1.235	-2.354	0.000	1	-2.626	-6.174	0.000
1367574_at	Vim	vimentin	Any2_423	1	9.61	9.13	8.90	8.53	0	-0.479	-1.393	0.122	1	-0.706	-1.631	0.002	1	-1.084	-2.119	0.001
1367581_a_at	Spp1	secreted phosphoprotein 1	Any2_423	1	6.22	5.47	5.42	5.65	1	-0.752	-1.684	0.004	1	-0.808	-1.751	0.001	1	-0.572	-1.486	0.067
1367584_at	Anxa2	annexin A2	Any2_423	1	8.65	7.96	7.88	7.67	1	-0.684	-1.607	0.008	1	-0.770	-1.705	0.001	1	-0.981	-1.974	0.002
1367594_at	Bgn	biglycan	Any2_423	1	8.64	7.96	8.18	7.92	1	-0.677	-1.598	0.004	0	-0.454	-1.370	0.113	1	-0.716	-1.643	0.014
1367600_at	Des	desmin	Any2_423	1	6.65	6.30	6.15	6.13	0	-0.353	-1.277	0.371	1	-0.503	-1.417	0.063	1	-0.523	-1.437	0.083
1367655_at	LOC10035949	thymosin, beta 10-like /// thymosin, beta 10-like ///	Any2_423	1	7.89	7.85	7.99	7.35	0	-0.043	-1.030	1.054	0	0.099	1.071	1.023	1	-0.545	-1.460	0.067
1367673_at	Selenbp1	selenium binding protein 1	Any2_423	1	6.57	5.95	6.24	6.18	1	-0.620	-1.537	0.017	0	-0.335	-1.261	0.350	0	-0.392	-1.312	0.259
1367700_at	Fmod	fibromodulin	Any2_423	1	5.66	4.96	4.82	4.91	1	-0.703	-1.627	0.008	1	-0.839	-1.789	0.001	1	-0.746	-1.677	0.016
1367749_at	Lum	lumican	Any2_423	1	8.30	7.61	7.59	6.79	1	-0.688	-1.611	0.005	1	-0.702	-1.627	0.004	1	-1.503	-2.834	0.000
1367774_at	Gsta3	glutathione S-transferase A3	Any2_423	1	7.33	6.93	7.25	6.11	0	-0.399	-1.319	0.230	0	-0.080	-1.057	0.981	1	-1.218	-2.326	0.000
1367785_at	Cnn1	calponin 1, basic, smooth muscle	Any2_423	1	4.83	4.30	4.00	3.84	1	-0.527	-1.441	0.060	1	-0.826	-1.773	0.001	1	-0.990	-1.986	0.002
1367866_at	Fbln5	fibulin 5	Any2_423	1	6.23	5.67	5.82	5.62	1	-0.568	-1.482	0.037	0	-0.410	-1.329	0.205	1	-0.610	-1.526	0.043
1367912_at	Ltbp1	latent transforming growth factor beta binding prot	Any2_423	1	6.95	6.99	6.85	6.36	0	0.045	1.032	1.138	0	-0.095	-1.068	0.837	1	-0.591	-1.507	0.049
1367974_at	Anxa3	annexin A3	Any2_423	1	7.09	6.50	6.51	6.36	1	-0.587	-1.502	0.031	1	-0.578	-1.493	0.029	1	-0.728	-1.656	0.014
1367990_at	Crybb3	crystallin, beta B3	Any2_423	1	6.18	5.49	5.78	5.64	1	-0.685	-1.608	0.004	0	-0.392	-1.312	0.231	1	-0.541	-1.455	0.067
1367998_at	Slpi	secretory leukocyte peptidase inhibitor	Any2_423	1	6.85	5.72	6.09	6.09	1	-1.127	-2.184	0.000	1	-0.758	-1.691	0.004	1	-0.764	-1.699	0.017
1367999_at	Aldh2	aldehyde dehydrogenase 2 family (mitochondrial)	Any2_423	1	6.65	6.18	6.10	6.29	0	-0.468	-1.383	0.126	1	-0.545	-1.459	0.034	0	-0.357	-1.281	0.323
1368000_at	C3	complement component 3	Any2_423	1	4.20	3.82	3.55	3.97	0	-0.381	-1.302	0.216	1	-0.649	-1.568	0.017	0	-0.225	-1.169	0.641
1368003_at	Aldh1a2	aldehyde dehydrogenase 1 family, member A2	Any2_423	1	6.36	5.76	5.79	6.08	1	-0.606	-1.522	0.019	1	-0.579	-1.494	0.015	0	-0.287	-1.221	0.530
1368028_at	Prph	peripherin	Any2_423	1	5.89	5.30	4.99	5.43	1	-0.589	-1.504	0.031	1	-0.899	-1.865	0.000	0	-0.452	-1.368	0.164
1368052_at	Tspan8	tetraspanin 8	Any2_423	1	6.76	6.37	6.19	6.24	0	-0.390	-1.311	0.251	1	-0.575	-1.490	0.019	1	-0.524	-1.438	0.098
1368113_at	Tff2	trefoil factor 2	Any2_423	1	7.01	8.62	5.92	7.91	1	1.611	3.054	0.000	1	-1.084	-2.119	0.000	1	0.907	1.875	0.053
1368128_at	Pla2g2a	phospholipase A2, group IIA (platelets, synovial fluid	Any2_423	1	5.48	4.51	4.54	4.92	1	-0.964	-1.950	0.000	1	-0.934	-1.911	0.000	1	-0.557	-1.471	0.083
1368144_at	Rgs2	regulator of G-protein signaling 2	Any2_423	1	8.36	9.22	8.39	10.59	1	0.858	1.813	0.017	0	0.029	1.020	1.000	1	2.230	4.692	0.001
1368160_at	Igfbp1	insulin-like growth factor binding protein 1	Any2_423	1	8.00	9.24	8.56	8.55	1	1.242	2.366	0.001	1	0.562	1.476	0.091	0	0.545	1.459	0.252
1368168_at	Slc34a2	solute carrier family 34 (sodium phosphate), membe	Any2_423	1	5.38	4.62	4.80	5.04	1	-0.755	-1.688	0.004	1	-0.576	-1.490	0.033	0	-0.333	-1.259	0.390
1368196_at	Clps	colipase, pancreatic	Any2_423	1	10.82	11.54	10.46	12.06	1	0.729	1.657	0.049	0	-0.358	-1.282	0.294	1	1.243	2.366	0.013
1368223_at	Adamts1	ADAM metallopeptidase with thrombospondin type	Any2_423	1	7.63	6.98	6.74	6.94	1	-0.641	-1.559	0.018	1	-0.884	-1.845	0.000	1	-0.684	-1.606	0.027
1368247_at	Hspa1a /// Hs	heat shock 70kD protein 1A /// heat shock 70kD pro	Any2_423	1	10.17	10.48	6.10	8.06	0	0.309	1.239	0.514	1	-4.078	-16.886	0.000	1	-2.118	-4.342	0.000
1368266_at	Arg1	arginase, liver	Any2_423	1	9.61	10.26	9.89	9.05	1	0.647	1.566	0.076	0	0.281	1.215	0.550	1	-0.560	-1.475	0.056
1368290_at	Cyr61	cysteine-rich, angiogenic inducer, 61	Any2_423	1	8.08	8.12	7.55	7.81	0	0.038	1.027	1.143	1	-0.529	-1.443	0.054	0	-0.276	-1.211	0.566
1368302_at	Msx1	msh homeobox 1	Any2_423	1	5.34	4.81	4.82	5.09	1	-0.528	-1.442	0.087	1	-0.515	-1.429	0.083	0	-0.250	-1.189	0.666
1368311_at	Mgmt	O-6-methylguanine-DNA methyltransferase	Any2_423	1	5.08	6.00	5.52	6.84	1	0.916	1.887	0.009	0	0.435	1.352	0.238	1	1.758	3.382	0.006
1368367_at	Cuzd1	CUB and zona pellucida-like domains 1	Any2_423	1	5.83	6.57	5.51	8.10	1	0.738	1.668	0.038	0	-0.320	-1.248	0.381	1	2.273	4.834	0.001
1368396_at	Cel	carboxyl ester lipase	Any2_423	1	11.12	12.29	10.53	11.86	1	1.169	2.248	0.003	1	-0.594	-1.507	0.025	0	0.745	1.676	0.100
1368407_at	Hpse	heparanase	Any2_423	1	5.65	6.10	5.85	4.52	0	0.449	1.365	0.272	0	0.206	1.153	0.728	1	-1.130	-2.188	0.001
1368418_a_at	Cp	ceruloplasmin (ferroxidase)	Any2_423	1	6.49	6.11	6.12	5.87	0	-0.376	-1.298	0.313	0	-0.367	-1.289	0.305	1	-0.615	-1.531	0.037
1368419_at	Cp	ceruloplasmin (ferroxidase)	Any2_423	1	6.19	5.64	5.90	5.51	1	-0.554	-1.468	0.042	0	-0.286	-1.219	0.319	1	-0.675	-1.597	0.022
1368420_at	Cp	ceruloplasmin (ferroxidase)	Any2_423	1	4.12	3.42	3.63	4.19	1	-0.693	-1.616	0.004	0	-0.489	-1.404	0.080	0	0.073	1.052	1.085
1368422_at	Meox2	mesenchyme homeobox 2	Any2_423	1	4.52	4.09	4.14	3.82	0	-0.434	-1.351	0.194	0	-0.375	-1.297	0.290	1	-0.704	-1.629	0.024
1368447_x_at	Spink3	serine peptidase inhibitor, Kazal type 3	Any2_423	1	7.34	8.40	7.00	9.08	1	1.062	2.087	0.003	0	-0.336	-1.262	0.342	1	1.744	3.349	0.006
1368473_at	Gja5	gap junction protein, alpha 5	Any2_423	1	5.77	5.04	5.04	5.00	1	-0.733	-1.662	0.004	1	-0.726	-1.654	0.002	1	-0.767	-1.701	0.012
1368532_at	Pnliprp1	pancreatic lipase-related protein 1	Any2_423	1	10.77	11.88	10.55	12.09	1	1.110	2.158	0.003	0	-0.215	-1.160	0.555	1	1.321	2.499	0.011
1368554_at	Pnlip	pancreatic lipase	Any2_423	1	11.96	13.11	11.54	13.15	1	1.155	2.226	0.003	0	-0.422	-1.340	0.183	1	1.195	2.290	0.014
1368577_at	Gjb6	gap junction protein, beta 6	Any2_423	1	4.25	5.55	4.71	5.47	1	1.298	2.459	0.000	0	0.462	1.377	0.215	1	1.222	2.332	0.013
1368586_at	Zg16	zymogen granule protein 16	Any2_423	1	10.13	10.81	9.70	11.65	1	0.678	1.600	0.075	0	-0.429	-1.346	0.168	1	1.519	2.866	0.007
1368629_at	Reg1a	regenerating islet-derived 1 alpha	Any2_423	1	7.38	8.42	6.50	9.94	1	1.036	2.051	0.004	1	-0.882	-1.843	0.000	1	2.559	5.894	0.000
1368679_a_at	Lyn	v-yes-1 Yamaguchi sarcoma viral related oncogene h	Any2_423	1	5.08	5.29	4.90	5.85	0	0.216	1.161	0.689	0	-0.175	-1.129	0.808	1	0.778	1.714	0.073
1368709_at	Fut1	fucosyltransferase 1	Any2_423	1	6.40	6.75	7.01	7.20	0	0.348	1.272	0.410	1	0.609	1.526	0.055	1	0.797	1.737	0.077
1368732_at	Tap2	transporter 2, ATP-binding cassette, sub-family B (M	Any2_423	1	6.00	5.97	5.88	5.30	0	-0.027	-1.019	1.117	0	-0.113	-1.082	0.895	1	-0.696	-1.620	0.019
1368760_at	Cxcl2	chemokine (C-X-C motif) ligand 2	Any2_423	1	3.84	4.06	3.74	4.77	0	0.212	1.159	0.655	0	-0.100	-1.072	0.725	1	0.923	1.896	0.047
1368771_at	Sulf1	sulfatase 1	Any2_423	1	5.30	4.86	4.78	4.78	0	-0.438	-1.354	0.150	1	-0.520	-1.434	0.044	1	-0.519	-1.433	0.099
1368822_at	Fstl1	follistatin-like 1	Any2_423	1	8.40	7.74	7.81	7.57	1	-0.652	-1.572	0.009	1	-0.586	-1.501	0.017	1	-0.827	-1.773	0.006
1368829_at	Fbn1	fibrillin 1	Any2_423	1	7.54	6.83	7.08	6.53	1	-0.709	-1.635	0.004	0	-0.463	-1.379	0.103	1	-1.011	-2.015	0.002
1368870_at	Id2	inhibitor of DNA binding 2	Any2_423	1	6.63	6.98	6.62	5.69	0	0.347	1.272	0.460	0	-0.008	-1.006	1.056	1	-0.944	-1.924	0.003
1369062_at	Madcam1	mucosal vascular addressin cell adhesion molecule 1	Any2_423	1	5.93	5.16	5.41	5.58	1	-0.774	-1.710	0.001	1	-0.525	-1.439	0.046	0	-0.352	-1.277	0.338
1369067_at	Nr4a3	nuclear receptor subfamily 4, group A, member 3	Any2_423	1	4.56	4.38	3.70	5.59	0	-0.178	-1.131	0.636	1	-0.855	-1.809	0.001	1	1.027	2.038	0.030
1369105_a_at	Pkib	protein kinase (cAMP-dependent, catalytic) inhibito	Any2_423	1	6.41	6.45	6.76	4.60	0	0.045	1.032	1.141	0	0.354	1.278	0.390	1	-1.805	-3.494	0.000
1369241_at	Avpr1b	arginine vasopressin receptor 1B	Any2_423	1	7.17	8.26	7.58	6.43	1	1.087	2.124	0.003	0	0.402	1.321	0.296	1	-0.747	-1.678	0.012
1369425_at	Cdh13	cadherin 13	Any2_423	1	5.50	5.04	5.03	4.89	0	-0.467	-1.382	0.129	0	-0.472	-1.387	0.110	1	-0.613	-1.529	0.044
1369625_at	Aqp1	aquaporin 1	Any2_423	1	5.69	4.38	4.37	4.56	1	-1.303	-2.468	0.000	1	-1.322	-2.500	0.000	1	-1.131	-2.190	0.001
1369657_at	Cpa1	carboxypeptidase A1 (pancreatic)	Any2_423	1	10.84	12.26	10.27	12.12	1	1.415	2.666	0.000	1	-0.576	-1.491	0.034	1	1.276	2.421	0.012
1369748_at	Serpini2	serpin peptidase inhibitor, clade I (pancpin), membe	Any2_423	1	7.08	8.04	6.67	7.86	1	0.962	1.947	0.008	0	-0.406	-1.325	0.210	1	0.786	1.724	0.084
1369823_at	Adam7	ADAM metallopeptidase domain 7	Any2_423	1	5.03	4.34	4.52	4.87	1	-0.690	-1.613	0.004	1	-0.518	-1.431	0.045	0	-0.160	-1.117	0.965
1369923_at	Sebox	SEBOX homeobox	Any2_423	1	5.69	5.08	5.43	4.86	1	-0.606	-1.522	0.016	0	-0.260	-1.198	0.476	1	-0.824	-1.770	0.007
1369943_at	Tgm2	transglutaminase 2, C polypeptide	Any2_423	1	8.74	8.19	8.17	7.93	1	-0.543	-1.457	0.050	1	-0.571	-1.485	0.022	1	-0.805	-1.747	0.007
1369955_at	Col5a1	collagen, type V, alpha 1	Any2_423	1	6.52	5.92	5.96	5.97	1	-0.607	-1.523	0.022	1	-0.567	-1.481	0.021	1	-0.556	-1.470	0.069
1369959_at	Zfp36l1	zinc finger protein 36, C3H type-like 1	Any2_423	1	9.90	9.52	9.59	9.31	0	-0.379	-1.301	0.276	0	-0.309	-1.239	0.425	1	-0.585	-1.500	0.049
1370026_at	Cryab	crystallin, alpha B	Any2_423	1	6.63	6.07	5.80	5.99	1	-0.565	-1.480	0.040	1	-0.836	-1.785	0.000	1	-0.637	-1.555	0.033
1370027_a_at	A1i3 /// Cpan	alpha-1-inhibitor III /// C3 and PZP-like, alpha-2-mac	Any2_423	1	5.73	6.67	6.22	5.15	1	0.941	1.920	0.008	0	0.490	1.405	0.156	1	-0.578	-1.493	0.066
1370047_at	Enpp1	ectonucleotide pyrophosphatase/phosphodiesteras	Any2_423	1	6.04	6.07	5.86	4.81	0	0.028	1.019	1.153	0	-0.185	-1.137	0.742	1	-1.226	-2.339	0.000
1370057_at	Csrp1	cysteine and glycine-rich protein 1	Any2_423	1	7.54	7.44	7.04	6.94	0	-0.100	-1.072	0.863	1	-0.501	-1.415	0.088	1	-0.592	-1.507	0.061
1370058_at	Nefl	neurofilament, light polypeptide	Any2_423	1	4.30	3.52	3.82	3.85	1	-0.779	-1.716	0.001	0	-0.487	-1.401	0.074	0	-0.452	-1.368	0.164
1370126_at	LOC10091208	anionic trypsin-2-like /// protease, serine, 2	Any2_423	1	10.34	11.60	10.14	12.07	1	1.262	2.399	0.001	0	-0.195	-1.145	0.676	1	1.732	3.321	0.006
1370143_at	Smo	smoothened, frizzled family receptor	Any2_423	1	5.49	4.96	4.86	5.17	1	-0.526	-1.440	0.054	1	-0.629	-1.546	0.006	0	-0.315	-1.244	0.419

Affymetrix Probeset ID	Gene Symbol	Gene Title	Figure 7 A Venn n=2289	Present in any 2 analyses n=423 1=yes 0=no	Mean log2 intensity DR+/+ ND	Mean log2 intensity DR+/+ HCD	Mean log2 intensity DR+/+ B/S	Mean log2 intensity F+/+ day 40 ND	DR+/+HCD vs DR+/+ND FDR<10% Log2 ratio >0.5 n=636	Log2 ratio DR+/+HCD vs DR+/+ ND	Fold-Change DR+/+HCD vs DR+/+ ND	FDR DR+/+HCD vs DR+/+ ND	DR+/+B/S vs DR+/+ND Log2 ratio >0.5 n=350	Log2 ratio DR+/+ B/S vs DR+/+ ND	Fold-Change DR+/+B/S vs DR+/+ ND	FDR DR+/+B/S vs DR+/+ ND	F+/+ vs DR+/+ND Log2 ratio >0.5 n=1209	Log2 ratio F+/+ vs DR+/+ ND	Fold-Change F+/+ vs DR+/+ ND	FDR F+/+ vs DR+/+ ND
1370155_at	Col1a2 /// LO	collagen, type I, alpha 2 /// collagen alpha-2(I) chain	Any2_423	1	8.90	8.24	8.34	7.84	1	-0.658	-1.578	0.009	1	-0.554	-1.469	0.030	1	-1.056	-2.080	0.001
1370157_at	Pln	phospholamban	Any2_423	1	6.33	7.02	6.56	5.69	1	0.695	1.619	0.061	0	0.234	1.176	0.638	1	-0.644	-1.563	0.032
1370161_at	Ccdc80	coiled-coil domain containing 80	Any2_423	1	8.57	7.81	7.85	7.30	1	-0.755	-1.688	0.002	1	-0.721	-1.648	0.001	1	-1.266	-2.406	0.000
1370228_at	Srprb /// Tf	signal recognition particle receptor, B subunit /// tra	Any2_423	1	7.19	6.78	6.68	6.67	0	-0.405	-1.324	0.206	1	-0.514	-1.428	0.061	0	-0.517	-1.431	0.102
1370291_at	Pdlim3	PDZ and LIM domain 3	Any2_423	1	6.07	5.75	5.46	5.85	0	-0.324	-1.252	0.421	1	-0.617	-1.534	0.011	0	-0.222	-1.166	0.664
1370563_at	Akr1c14	aldo-keto reductase family 1, member C14	Any2_423	1	4.34	3.83	4.05	4.21	1	-0.506	-1.420	0.080	0	-0.289	-1.222	0.462	0	-0.132	-1.096	1.018
1370658_a_at	St18	suppression of tumorigenicity 18	Any2_423	1	7.72	8.39	7.63	9.32	1	0.664	1.584	0.085	0	-0.094	-1.067	0.965	1	1.603	3.037	0.005
1370694_at	Trib3	tribbles homolog 3 (Drosophila)	Any2_423	1	5.50	6.61	6.41	6.69	1	1.116	2.167	0.002	1	0.913	1.883	0.005	1	1.196	2.291	0.013
1370751_at	LOC257642	rRNA promoter binding protein	Any2_423	1	9.40	8.47	8.76	8.52	1	-0.932	-1.907	0.000	1	-0.636	-1.554	0.008	1	-0.878	-1.837	0.006
1370837_at	Sycn	syncollin	Any2_423	1	10.37	10.94	9.80	11.92	0	0.570	1.485	0.163	1	-0.563	-1.477	0.040	1	1.555	2.938	0.006
1370854_at	Nexn	nexilin (F actin binding protein)	Any2_423	1	5.65	5.25	5.14	5.08	0	-0.405	-1.324	0.239	1	-0.507	-1.421	0.068	1	-0.574	-1.488	0.063
1370864_at	Col1a1	collagen, type I, alpha 1	Any2_423	1	7.68	6.94	7.02	6.70	1	-0.735	-1.665	0.002	1	-0.651	-1.570	0.004	1	-0.977	-1.968	0.002
1370892_at	C4a /// C4b /,	complement component 4A (Rodgers blood group) ,	Any2_423	1	7.15	4.49	4.61	5.19	1	-2.655	-6.300	0.000	1	-2.531	-5.781	0.000	1	-1.960	-3.890	0.000
1370896_a_at	Myh11	myosin, heavy chain 11, smooth muscle	Any2_423	1	8.65	7.90	7.65	6.59	1	-0.750	-1.682	0.004	1	-1.003	-2.003	0.000	1	-2.064	-4.182	0.000
1370956_at	Dcn	decorin	Any2_423	1	8.48	8.46	8.23	7.67	0	-0.016	-1.011	1.122	0	-0.243	-1.183	0.573	1	-0.806	-1.749	0.008
1370959_at	Col3a1	collagen, type III, alpha 1	Any2_423	1	10.63	9.98	10.03	9.96	1	-0.649	-1.568	0.009	1	-0.601	-1.517	0.012	1	-0.675	-1.597	0.021
1370960_at	Igfbp5	insulin-like growth factor binding protein 5	Any2_423	1	6.60	5.22	5.49	4.48	1	-1.383	-2.607	0.000	1	-1.109	-2.157	0.000	1	-2.119	-4.344	0.000
1370961_at	Eci1	enoyl-CoA delta isomerase 1	Any2_423	1	6.73	5.90	6.00	6.38	1	-0.822	-1.768	0.000	1	-0.724	-1.652	0.001	0	-0.349	-1.274	0.387
1370967_at	LOC680329	immunoglobulin lambda-like polypeptide 5-like	Any2_423	1	6.78	6.06	6.03	4.50	1	-0.717	-1.643	0.011	1	-0.746	-1.677	0.006	1	-2.272	-4.830	0.000
1371027_at	Cblb	Cbl proto-oncogene, E3 ubiquitin protein ligase B	Any2_423	1	6.92	7.60	7.05	7.79	1	0.678	1.599	0.064	0	0.130	1.094	0.807	1	0.872	1.830	0.052
1371034_at	Onecut1	one cut homeobox 1	Any2_423	1	4.73	3.45	4.06	3.77	1	-1.282	-2.432	0.000	1	-0.678	-1.600	0.014	1	-0.960	-1.946	0.004
1371237_a_at	LOC1003627(	hypothetical protein LOC100362769 /// metallothio	Any2_423	1	11.32	12.45	12.02	11.70	1	1.134	2.194	0.003	1	0.709	1.635	0.017	0	0.388	1.309	0.508
1371280_at	Krtap1-5	keratin associated protein 1-5	Any2_423	1	4.36	3.77	3.84	3.56	1	-0.589	-1.504	0.024	1	-0.523	-1.437	0.046	1	-0.795	-1.735	0.008
1371331_at	---	---	Any2_423	1	8.24	7.52	7.72	7.29	1	-0.723	-1.650	0.002	1	-0.514	-1.428	0.052	1	-0.943	-1.923	0.003
1371382_at	Flna	filamin A, alpha	Any2_423	1	8.09	7.59	7.40	7.26	1	-0.501	-1.415	0.090	1	-0.684	-1.607	0.002	1	-0.822	-1.768	0.007
1371406_at	---	---	Any2_423	1	8.66	8.13	8.03	7.82	1	-0.532	-1.446	0.062	1	-0.625	-1.543	0.007	1	-0.836	-1.785	0.006
1371447_at	Plac8	placenta-specific 8	Any2_423	1	8.57	7.81	7.48	7.32	1	-0.755	-1.688	0.006	1	-1.088	-2.125	0.000	1	-1.253	-2.383	0.001
1371499_at	Cd9	CD9 molecule	Any2_423	1	7.26	6.81	6.67	6.40	0	-0.448	-1.364	0.160	1	-0.588	-1.503	0.022	1	-0.859	-1.814	0.006
1371500_at	Ltbp4	latent transforming growth factor beta binding prot	Any2_423	1	7.62	7.09	7.12	6.74	1	-0.526	-1.439	0.059	0	-0.497	-1.411	0.065	1	-0.875	-1.834	0.005
1371595_at	LOC1009124(	uncharacterized LOC100912446	Any2_423	1	6.20	5.31	5.25	5.96	1	-0.891	-1.855	0.001	1	-0.956	-1.940	0.000	0	-0.241	-1.181	0.587
1371700_at	LOC1009117:	microfibril-associated glycoprotein 4-like /// microfi	Any2_423	1	7.10	6.46	6.78	6.16	1	-0.636	-1.554	0.010	0	-0.322	-1.250	0.373	1	-0.934	-1.911	0.003
1371703_at	Ahnak	AHNAK nucleoprotein	Any2_423	1	8.80	8.32	8.17	8.22	0	-0.485	-1.400	0.104	1	-0.636	-1.554	0.007	1	-0.584	-1.499	0.046
1371942_at	Gstt3	glutathione S-transferase, theta 3	Any2_423	1	5.45	4.58	4.89	4.88	1	-0.870	-1.828	0.000	1	-0.559	-1.473	0.035	1	-0.568	-1.483	0.065
1371970_at	Fam111a	family with sequence similarity 111, member A	Any2_423	1	12.08	11.49	11.63	4.69	1	-0.587	-1.502	0.030	0	-0.454	-1.370	0.126	1	-7.388	#######	0.000
1372107_at	Fhl1	four and a half LIM domains 1	Any2_423	1	7.66	7.10	6.62	6.78	1	-0.556	-1.471	0.047	1	-1.040	-2.056	0.000	1	-0.880	-1.840	0.005
1372108_at	Ptcd3	Pentatricopeptide repeat domain 3	Any2_423	1	10.04	10.78	10.47	9.48	1	0.741	1.671	0.037	0	0.431	1.348	0.248	1	-0.558	-1.472	0.060
1372111_at	Cav1	caveolin 1, caveolae protein	Any2_423	1	9.42	9.08	8.84	8.75	0	-0.342	-1.267	0.352	1	-0.586	-1.501	0.018	1	-0.668	-1.589	0.025
1372168_s_at	Igfbp6	insulin-like growth factor binding protein 6	Any2_423	1	6.47	5.36	5.49	5.61	1	-1.104	-2.150	0.000	1	-0.977	-1.968	0.000	1	-0.855	-1.809	0.006
1372219_at	Tpm2	tropomyosin 2, beta	Any2_423	1	8.49	7.80	7.48	6.26	1	-0.692	-1.616	0.007	1	-1.010	-2.014	0.000	1	-2.233	-4.702	0.000
1372256_at	Crip1	cysteine-rich protein 1 (intestinal)	Any2_423	1	9.23	8.54	8.39	8.05	1	-0.684	-1.606	0.008	1	-0.837	-1.787	0.000	1	-1.170	-2.251	0.001
1372273_at	Gypc	glycophorin C (Gerbich blood group)	Any2_423	1	5.93	5.57	5.63	5.13	0	-0.364	-1.287	0.302	0	-0.300	-1.231	0.410	1	-0.800	-1.742	0.009
1372301_at	Aebp1	AE binding protein 1	Any2_423	1	6.37	5.76	5.86	5.14	1	-0.613	-1.530	0.023	1	-0.508	-1.422	0.069	1	-1.226	-2.340	0.001
1372397_at	Tiprl	TIP41, TOR signaling pathway regulator-like (S. cerev	Any2_423	1	7.84	8.06	7.95	8.59	0	0.220	1.165	0.747	0	0.118	1.085	0.993	1	0.750	1.681	0.089
1372406_at	Mcm3	minichromosome maintenance complex componen	Any2_423	1	5.19	4.69	4.87	4.47	0	-0.497	-1.412	0.096	0	-0.317	-1.246	0.394	1	-0.719	-1.646	0.014
1372565_at	Htra3	HtrA serine peptidase 3	Any2_423	1	7.10	6.53	6.63	6.60	1	-0.569	-1.483	0.033	0	-0.467	-1.382	0.092	1	-0.501	-1.416	0.100
1372604_at	LOC1009108(	apolipoprotein L3-like	Any2_423	1	5.67	5.14	5.11	5.73	1	-0.534	-1.448	0.059	1	-0.556	-1.470	0.033	0	0.058	1.041	1.062
1372615_at	Aoc3	amine oxidase, copper containing 3	Any2_423	1	8.47	7.96	7.89	6.44	1	-0.510	-1.424	0.077	1	-0.579	-1.494	0.016	1	-2.036	-4.101	0.000
1372616_at	---	---	Any2_423	1	4.05	3.48	3.52	3.80	1	-0.568	-1.482	0.036	1	-0.531	-1.445	0.035	0	-0.252	-1.191	0.582
1372729_at	Procr	protein C receptor, endothelial	Any2_423	1	5.79	5.03	5.26	4.76	1	-0.759	-1.693	0.004	1	-0.524	-1.438	0.064	1	-1.025	-2.034	0.002
1372820_at	LOC691984	similar to Glypican-6 precursor	Any2_423	1	5.01	4.40	4.37	4.58	1	-0.607	-1.524	0.023	1	-0.642	-1.560	0.007	0	-0.432	-1.349	0.190
1373032_at	Mustn1	musculoskeletal, embryonic nuclear protein 1	Any2_423	1	7.29	6.90	6.73	5.83	0	-0.394	-1.314	0.230	1	-0.564	-1.478	0.034	1	-1.464	-2.759	0.000
1373062_at	---	---	Any2_423	1	6.84	6.01	5.88	5.75	1	-0.824	-1.770	0.000	1	-0.959	-1.944	0.000	1	-1.084	-2.120	0.001
1373102_at	Cdh13	cadherin 13	Any2_423	1	6.10	5.76	5.59	5.35	0	-0.337	-1.263	0.377	1	-0.514	-1.428	0.072	1	-0.750	-1.681	0.013
1373266_at	Fam107a	family with sequence similarity 107, member A	Any2_423	1	4.97	4.34	4.20	4.20	1	-0.638	-1.556	0.014	1	-0.777	-1.713	0.001	1	-0.777	-1.714	0.018
1373326_at	---	---	Any2_423	1	6.11	6.83	6.68	6.51	1	0.721	1.648	0.043	1	0.573	1.487	0.079	0	0.404	1.323	0.477
1373490_at	Gmfg	glia maturation factor, gamma	Any2_423	1	6.33	6.12	6.06	5.79	0	-0.202	-1.151	0.726	0	-0.263	-1.200	0.500	1	-0.536	-1.450	0.076
1373686_at	Serpina6	serpin peptidase inhibitor, clade A (alpha-1 antiprot	Any2_423	1	5.22	6.67	6.49	4.75	1	1.445	2.722	0.000	1	1.267	2.407	0.000	0	-0.470	-1.385	0.152
1373908_at	---	---	Any2_423	1	5.91	5.61	5.30	5.26	0	-0.305	-1.236	0.382	1	-0.616	-1.533	0.014	1	-0.655	-1.575	0.028
1373911_at	Postn	periostin, osteoblast specific factor	Any2_423	1	4.96	4.20	4.24	5.46	1	-0.757	-1.690	0.003	1	-0.720	-1.648	0.001	0	0.495	1.409	0.337
1373957_at	Reln	reelin	Any2_423	1	4.58	3.92	4.25	4.31	1	-0.657	-1.576	0.010	0	-0.327	-1.254	0.368	0	-0.266	-1.202	0.587
1374056_at	Rbm24	RNA binding motif protein 24	Any2_423	1	5.15	4.88	4.61	4.56	0	-0.272	-1.208	0.546	1	-0.544	-1.458	0.035	1	-0.597	-1.512	0.044
1374061_at	Cd302	CD302 molecule	Any2_423	1	5.60	5.77	5.77	4.96	0	0.174	1.128	0.849	0	0.171	1.126	0.781	1	-0.632	-1.550	0.052
1374070_at	Gpx2	glutathione peroxidase 2	Any2_423	1	5.83	4.98	5.03	5.51	1	-0.849	-1.801	0.000	1	-0.804	-1.746	0.001	0	-0.319	-1.247	0.422
1374104_at	Cldn5	claudin 5	Any2_423	1	5.84	5.11	5.04	4.85	1	-0.731	-1.659	0.004	1	-0.801	-1.742	0.001	1	-0.984	-1.978	0.002
1374187_at	---	---	Any2_423	1	6.85	7.48	7.27	8.02	1	0.621	1.538	0.099	0	0.411	1.330	0.279	1	1.165	2.243	0.017
1374334_at	Igha /// LOC3	immunoglobulin heavy chain, alpha /// similar to im	Any2_423	1	5.78	5.89	5.14	5.86	0	0.113	1.082	0.919	1	-0.639	-1.557	0.007	0	0.086	1.061	1.072
1374404_at	Jun	jun proto-oncogene	Any2_423	1	8.69	9.21	8.09	7.97	0	0.517	1.431	0.198	1	-0.596	-1.512	0.021	1	-0.723	-1.651	0.018
1374535_at	---	---	Any2_423	1	6.44	5.87	6.08	5.79	1	-0.574	-1.489	0.033	0	-0.368	-1.290	0.284	1	-0.651	-1.570	0.030
1374705_at	Col4a5	collagen, type IV, alpha 5	Any2_423	1	7.36	7.05	7.02	6.80	0	-0.314	-1.243	0.446	0	-0.338	-1.264	0.335	1	-0.563	-1.477	0.063
1374726_at	Fndc1	fibronectin type III domain containing 1	Any2_423	1	5.66	4.84	4.85	4.47	1	-0.822	-1.768	0.000	1	-0.807	-1.750	0.001	1	-1.194	-2.288	0.001
1374779_at	F13a1	coagulation factor XIII, A1 polypeptide	Any2_423	1	4.54	3.88	4.39	4.02	1	-0.665	-1.585	0.009	0	-0.151	-1.111	0.798	1	-0.521	-1.435	0.097
1375001_at	Cytl1	cytokine like 1	Any2_423	1	7.25	6.46	6.38	5.72	1	-0.790	-1.729	0.000	1	-0.863	-1.819	0.000	1	-1.525	-2.878	0.000
1375120_at	Id4	inhibitor of DNA binding 4	Any2_423	1	5.74	5.23	5.35	5.22	1	-0.506	-1.420	0.088	0	-0.393	-1.313	0.238	1	-0.517	-1.431	0.097
1375428_at	Creg1	cellular repressor of E1A-stimulated genes 1	Any2_423	1	5.72	6.35	6.30	6.16	1	0.630	1.547	0.094	1	0.581	1.495	0.072	0	0.439	1.355	0.423
1375622_at	Zfyve20	zinc finger, FYVE domain containing 20	Any2_423	1	5.19	6.11	5.93	5.61	1	0.917	1.889	0.016	1	0.738	1.668	0.016	0	0.418	1.336	0.461
1375680_at	---	---	Any2_423	1	5.79	5.21	5.60	5.22	1	-0.582	-1.497	0.029	0	-0.194	-1.144	0.715	1	-0.571	-1.486	0.052
1375719_s_at	Cdh13	cadherin 13	Any2_423	1	6.47	6.13	6.09	5.73	0	-0.343	-1.269	0.319	0	-0.382	-1.303	0.268	1	-0.737	-1.667	0.013
1375951_at	Thbd	thrombomodulin	Any2_423	1	6.55	6.24	6.00	5.69	0	-0.314	-1.243	0.420	1	-0.550	-1.464	0.032	1	-0.866	-1.823	0.005
1376055_at	Mcm5	minichromosome maintenance complex componen	Any2_423	1	6.84	6.08	6.46	6.27	1	-0.760	-1.694	0.001	0	-0.379	-1.301	0.267	1	-0.569	-1.483	0.053
1376178_at	---	---	Any2_423	1	7.96	7.70	7.19	7.41	0	-0.267	-1.204	0.429	1	-0.771	-1.707	0.001	1	-0.551	-1.465	0.086
1376292_at	---	---	Any2_423	1	4.97	4.42	4.51	4.43	1	-0.558	-1.472	0.047	0	-0.460	-1.376	0.124	1	-0.539	-1.453	0.086
1376319_at	Sema3c	sema domain, immunoglobulin domain (Ig), short ba	Any2_423	1	6.15	5.60	5.49	5.92	1	-0.551	-1.465	0.051	1	-0.661	-1.581	0.007	0	-0.223	-1.167	0.719
1376709_at	Slc39a8	solute carrier family 39 (zinc transporter), member 8	Any2_423	1	4.92	4.16	4.19	4.48	1	-0.759	-1.692	0.004	1	-0.732	-1.660	0.002	0	-0.436	-1.353	0.194
1376749_at	LOC1009108!	mimecan-like /// osteoglycin	Any2_423	1	7.74	7.24	7.26	6.65	1	-0.506	-1.420	0.079	0	-0.488	-1.402	0.085	1	-1.099	-2.141	0.001
1376770_at	Efhd1	EF-hand domain family, member D1	Any2_423	1	6.39	5.74	6.11	5.88	1	-0.657	-1.577	0.010	0	-0.284	-1.217	0.459	1	-0.516	-1.430	0.100
1376860_at	Slc22a23	solute carrier family 22, member 23	Any2_423	1	5.28	6.06	5.61	6.15	1	0.782	1.719	0.025	0	0.331	1.258	0.424	1	0.865	1.821	0.047
1376872_at	Plxnc1	plexin C1	Any2_423	1	7.14	7.48	7.71	7.91	0	0.347	1.272	0.422	1	0.576	1.491	0.078	1	0.778	1.714	0.095
1376876_at	---	---	Any2_423	1	7.74	7.23	7.28	7.18	1	-0.509	-1.423	0.085	0	-0.457	-1.372	0.114	1	-0.552	-1.466	0.063
1376937_at	Filip1l	filamin A interacting protein 1-like	Any2_423	1	6.95	6.71	6.75	6.13	0	-0.238	-1.179	0.562	0	-0.194	-1.144	0.707	1	-0.818	-1.763	0.007
1377018_at	---	---	Any2_423	1	5.87	5.32	5.40	5.56	1	-0.550	-1.464	0.051	0	-0.469	-1.384	0.109	0	-0.314	-1.243	0.447
1377046_at	Ankrd6	ankyrin repeat domain 6	Any2_423	1	5.55	6.22	6.18	5.68	1	0.673	1.595	0.061	1	0.639	1.557	0.042	0	0.136	1.099	1.001
1377114_at	---	---	Any2_423	1	10.11	8.89	9.03	8.61	1	-1.213	-2.317	0.000	1	-1.076	-2.109	0.000	1	-1.491	-2.812	0.000
1377146_at	Vip	vasoactive intestinal peptide	Any2_423	1	5.39	4.45	3.90	3.77	1	-0.937	-1.915	0.000	1	-1.492	-2.813	0.000	1	-1.613	-3.060	0.000
1377223_at	Wt1	Wilms tumor 1	Any2_423	1	5.04	4.29	4.20	4.49	1	-0.748	-1.680	0.003	1	-0.834	-1.782	0.000	1	-0.552	-1.466	0.076
1377240_at	---	---	Any2_423	1	6.05	6.76	6.04	5.55	1	0.701	1.626	0.049	0	-0.013	-1.009	1.101	1	-0.502	-1.416	0.099
1377286_at	Gimap4	GTPase, IMAP family member 4	Any2_423	1	5.84	5.77	5.56	4.88	0	-0.063	-1.044	1.063	0	-0.273	-1.208	0.509	1	-0.958	-1.942	0.003
1377340_at	Tfpi2	tissue factor pathway inhibitor 2	Any2_423	1	4.78	4.12	4.24	4.70	1	-0.655	-1.574	0.008	1	-0.536	-1.449	0.035	0	-0.077	-1.055	1.127
1377466_a_at	Crebzf	CREB/ATF bZIP transcription factor	Any2_423	1	5.28	4.57	4.88	4.60	1	-0.708	-1.634	0.004	0	-0.393	-1.313	0.239	1	-0.670	-1.592	0.043
1377492_at	Rprml	reprimo-like	Any2_423	1	6.72	6.53	6.86	5.71	0	-0.186	-1.138	0.859	0	0.142	1.104	0.908	1	-1.010	-2.014	0.002
1377719_a_at	---	---	Any2_423	1	10.48	9.83	9.84	10.50	1	-0.642	-1.560	0.009	1	-0.637	-1.555	0.006	0	0.027	1.019	1.100
1377720_x_at	---	---	Any2_423	1	10.42	9.64	9.70	10.11	1	-0.777	-1.714	0.001	1	-0.722	-1.649	0.001	0	-0.313	-1.243	0.462
1377795_at	---	---	Any2_423	1	3.43	3.03	2.80	2.73	0	-0.394	-1.314	0.273	1	-0.626	-1.543	0.010	1	-0.696	-1.620	0.020
1377867_at	RGD1562284	similar to Glutaminyl-peptide cyclotransferase precu	Any2_423	1	6.25	6.95	6.38	4.89	1	0.699	1.624	0.048	0	0.133	1.097	0.812	1	-1.362	-2.570	0.000
1377950_at	LOC1009109:	interferon-inducible GTPase 1-like /// similar to inte	Any2_423	1	5.13	4.50	4.87	4.00	1	-0.629	-1.546	0.022	0	-0.258	-1.195	0.528	1	-1.127	-2.184	0.001
1377983_at	Ubp1	upstream binding protein 1 (LBP-1a)	Any2_423	1	5.45	4.78	5.41	4.91	1	-0.675	-1.597	0.004	0	-0.040	-1.028	1.088	1	-0.543	-1.457	0.069
1377993_at	Gng13	guanine nucleotide binding protein (G protein), gam	Any2_423	1	5.23	4.70	4.74	4.48	1	-0.534	-1.447	0.056	0	-0.497	-1.411	0.063	1	-0.758	-1.691	0.010
1378001_at	---	---	Any2_423	1	5.21	4.47	4.46	4.67	1	-0.743	-1.673	0.004	1	-0.756	-1.689	0.003	1	-0.543	-1.457	0.090

Affymetrix Probeset ID	Gene Symbol	Gene Title	Figure 7 A Venn n=2289	Present in any 2 analyses n=423 1=yes 0=no	Mean log2 intensity DR+/+ ND	Mean log2 intensity DR+/+ HCD	Mean log2 intensity DR+/+ B/S	Mean log2 intensity F+/+ day 40 ND	DR+/+HCD vs DR+/+ND FDR<10% Log2 ratio >0.5 n=636	Log2 ratio DR+/+HCD vs DR+/+ ND	Fold-Change DR+/+HCD vs DR+/+ ND	FDR DR+/+HCD vs DR+/+ ND	DR+/+B/S vs DR+/+ND Log2 ratio >0.5 n=350	Log2 ratio DR+/+ B/S vs DR+/+ ND	Fold-Change DR+/+B /S vs DR+/+ ND	FDR DR+/+B /S vs DR+/+ ND	F+/+ vs DR+/+N D Log2 ratio >0.5 n=1209	Log2 ratio F+/+ vs DR+/+ ND	Fold-Change F+/+ vs DR+/+ ND	FDR F+/+ vs DR+/+ ND
1378150_at	---	---	Any2_423	1	5.12	4.78	4.74	4.53	0	-0.339	-1.265	0.415	0	-0.381	-1.302	0.261	1	-0.589	-1.504	0.049
1378260_at	Adh1	alcohol dehydrogenase 1 (class I)	Any2_423	1	5.82	5.30	5.36	5.21	1	-0.514	-1.428	0.068	0	-0.452	-1.368	0.110	1	-0.604	-1.520	0.043
1378367_at	---	---	Any2_423	1	6.82	6.31	6.41	6.16	1	-0.514	-1.428	0.079	0	-0.416	-1.335	0.175	1	-0.664	-1.585	0.027
1378676_at	---	---	Any2_423	1	5.08	4.55	4.51	5.02	1	-0.534	-1.448	0.057	1	-0.565	-1.480	0.016	0	-0.057	-1.040	1.126
1378699_at	Pkhd1l1	polycystic kidney and hepatic disease 1-like 1	Any2_423	1	5.06	4.47	4.36	4.56	1	-0.588	-1.503	0.029	1	-0.700	-1.624	0.003	0	-0.500	-1.414	0.118
1378960_at	---	---	Any2_423	1	4.33	3.79	3.96	3.69	1	-0.541	-1.455	0.055	0	-0.375	-1.297	0.285	1	-0.643	-1.562	0.039
1379021_a_at	---	---	Any2_423	1	4.82	4.32	4.47	4.32	1	-0.504	-1.418	0.094	0	-0.352	-1.277	0.336	1	-0.507	-1.421	0.092
1379151_at	---	---	Any2_423	1	4.91	4.24	4.48	4.27	1	-0.668	-1.589	0.006	0	-0.430	-1.347	0.147	1	-0.631	-1.549	0.034
1379363_at	LOC679818	similar to Maltase-glucoamylase, intestinal	Any2_423	1	4.87	4.20	4.84	4.35	1	-0.671	-1.593	0.008	0	-0.029	-1.021	0.968	1	-0.526	-1.439	0.097
1379376_at	---	---	Any2_423	1	5.88	6.27	6.66	6.76	0	0.385	1.305	0.373	1	0.774	1.711	0.009	1	0.882	1.843	0.049
1379382_at	---	---	Any2_423	1	6.93	7.80	7.25	7.76	1	0.879	1.839	0.011	0	0.324	1.252	0.437	1	0.835	1.783	0.057
1379464_at	---	---	Any2_423	1	6.04	6.89	6.22	4.90	1	0.852	1.805	0.016	0	0.176	1.130	0.743	1	-1.143	-2.209	0.001
1379499_at	Ltb	lymphotoxin beta (TNF superfamily, member 3)	Any2_423	1	5.33	5.44	5.21	4.61	0	0.112	1.081	0.920	0	-0.112	-1.081	0.927	1	-0.720	-1.647	0.016
1379682_at	---	---	Any2_423	1	5.55	5.00	5.23	5.00	1	-0.554	-1.469	0.041	0	-0.322	-1.250	0.284	1	-0.554	-1.468	0.063
1379740_at	---	---	Any2_423	1	4.13	4.26	4.22	3.59	0	0.133	1.096	0.910	0	0.097	1.069	0.921	1	-0.537	-1.451	0.090
1379833_at	Lingo4 /// Ro	leucine rich repeat and Ig domain containing 4 /// R	Any2_423	1	6.21	6.98	6.98	6.12	1	0.776	1.712	0.030	1	0.770	1.705	0.011	0	-0.091	-1.065	1.076
1379895_at	---	---	Any2_423	1	4.89	5.35	5.48	5.79	0	0.456	1.372	0.288	1	0.586	1.501	0.073	1	0.891	1.854	0.053
1379936_at	---	---	Any2_423	1	5.22	4.49	4.71	4.26	1	-0.733	-1.662	0.004	1	-0.509	-1.423	0.070	1	-0.963	-1.950	0.005
1380063_at	Ch25h	cholesterol 25-hydroxylase	Any2_423	1	5.92	5.49	5.24	5.83	0	-0.435	-1.352	0.181	1	-0.682	-1.604	0.005	0	-0.089	-1.064	1.005
1380183_at	LOC10036599	rCG28262-like /// trypsin 4	Any2_423	1	8.75	9.95	8.71	10.99	1	1.194	2.288	0.001	0	-0.045	-1.031	1.078	1	2.233	4.703	0.002
1380210_at	Gimap1	GTPase, IMAP family member 1	Any2_423	1	5.90	5.68	5.75	4.65	0	-0.224	-1.168	0.719	0	-0.155	-1.114	0.688	1	-1.246	-2.372	0.000
1380230_at	---	---	Any2_423	1	7.46	8.56	8.05	8.28	1	1.100	2.144	0.003	1	0.589	1.505	0.072	1	0.819	1.765	0.060
1380425_at	---	---	Any2_423	1	6.95	7.61	7.59	7.08	1	0.654	1.573	0.073	1	0.636	1.554	0.047	0	0.126	1.091	1.004
1380447_a_at	Mrps18c	mitochondrial ribosomal protein S18C	Any2_423	1	6.54	7.26	7.18	6.82	1	0.723	1.651	0.047	1	0.644	1.563	0.041	0	0.286	1.219	0.716
1380537_at	Nckap1l	NCK associated protein 1 like	Any2_423	1	4.88	4.47	4.65	4.35	0	-0.418	-1.336	0.227	0	-0.235	-1.177	0.666	1	-0.538	-1.452	0.072
1380567_at	---	---	Any2_423	1	8.05	9.20	8.60	7.13	1	1.150	2.220	0.003	1	0.556	1.471	0.090	1	-0.919	-1.891	0.003
1380685_at	---	---	Any2_423	1	4.82	5.27	5.52	5.76	0	0.453	1.369	0.252	1	0.700	1.624	0.021	1	0.946	1.927	0.035
1380798_at	Nfkbiz	nuclear factor of kappa light polypeptide gene enha	Any2_423	1	5.90	5.38	5.59	5.73	1	-0.517	-1.431	0.076	0	-0.308	-1.238	0.455	0	-0.168	-1.123	0.939
1381148_at	---	---	Any2_423	1	5.63	4.94	5.33	5.06	1	-0.695	-1.619	0.004	0	-0.300	-1.232	0.406	1	-0.576	-1.491	0.049
1381327_a_at	Cr2	complement component (3d/Epstein Barr virus) rec	Any2_423	1	2.97	2.68	2.78	2.38	0	-0.291	-1.224	0.488	0	-0.193	-1.143	0.726	1	-0.589	-1.504	0.051
1381431_at	---	---	Any2_423	1	3.90	3.34	3.69	3.18	1	-0.561	-1.475	0.040	0	-0.216	-1.162	0.660	1	-0.726	-1.654	0.013
1381435_at	---	---	Any2_423	1	6.04	5.47	5.78	5.28	1	-0.564	-1.479	0.036	0	-0.253	-1.192	0.577	1	-0.760	-1.693	0.034
1381920_at	---	---	Any2_423	1	6.23	4.95	5.27	5.32	1	-1.281	-2.430	0.000	1	-0.951	-1.934	0.000	1	-0.905	-1.873	0.004
1382031_at	---	---	Any2_423	1	4.83	5.69	5.97	4.63	1	0.865	1.821	0.012	1	1.141	2.205	0.000	0	-0.197	-1.146	0.685
1382072_at	Olfml2a	olfactomedin-like 2A	Any2_423	1	4.62	4.34	4.08	4.08	0	-0.283	-1.217	0.491	1	-0.541	-1.455	0.030	1	-0.535	-1.449	0.072
1382083_at	Coch	cochlin	Any2_423	1	5.37	6.39	5.79	4.09	1	1.022	2.030	0.004	0	0.418	1.336	0.245	1	-1.285	-2.438	0.000
1382192_at	Lyve1	lymphatic vessel endothelial hyaluronan receptor 1	Any2_423	1	4.63	3.80	3.67	3.95	1	-0.827	-1.774	0.001	1	-0.956	-1.940	0.000	1	-0.684	-1.606	0.025
1382215_at	RGD1562997	similar to Transcription initiation factor TFIID 105 kD	Any2_423	1	4.26	4.91	4.66	4.89	1	0.654	1.573	0.095	0	0.407	1.326	0.270	0	0.635	1.553	0.204
1382274_at	Rarres1	retinoic acid receptor responder (tazarotene induce	Any2_423	1	6.95	6.17	6.22	6.04	1	-0.787	-1.725	0.000	1	-0.738	-1.667	0.001	1	-0.916	-1.886	0.004
1382278_at	LOC10091244	uncharacterized LOC100912446	Any2_423	1	7.72	6.70	6.08	7.21	1	-1.016	-2.023	0.000	1	-1.638	-3.112	0.000	0	-0.510	-1.424	0.143
1382410_at	Cd209b	CD209b antigen	Any2_423	1	5.75	5.01	5.06	5.10	1	-0.742	-1.672	0.002	1	-0.683	-1.606	0.002	1	-0.643	-1.562	0.028
1382473_at	LOC10091055	histone H2A type 1-like	Any2_423	1	4.82	4.28	4.23	4.24	1	-0.544	-1.458	0.049	1	-0.590	-1.505	0.011	1	-0.581	-1.496	0.048
1382494_at	---	---	Any2_423	1	5.59	4.94	5.04	4.82	1	-0.656	-1.576	0.008	1	-0.550	-1.465	0.030	1	-0.777	-1.713	0.009
1382532_at	Cela3b	chymotrypsin-like elastase family, member 3B	Any2_423	1	11.00	12.18	10.47	12.38	1	1.172	2.253	0.002	1	-0.532	-1.446	0.052	1	1.372	2.587	0.008
1382732_at	Lyve1	lymphatic vessel endothelial hyaluronan receptor 1	Any2_423	1	3.86	3.38	3.32	3.55	0	-0.480	-1.395	0.108	1	-0.540	-1.454	0.045	0	-0.309	-1.239	0.434
1382941_at	Lrrc32	leucine rich repeat containing 32	Any2_423	1	5.85	5.25	5.35	5.26	1	-0.605	-1.521	0.024	1	-0.501	-1.416	0.069	1	-0.595	-1.511	0.043
1383160_at	Chordc1	cysteine and histidine-rich domain (CHORD)-contain	Any2_423	1	8.34	8.27	8.31	7.75	0	-0.071	-1.050	1.072	0	-0.030	-1.021	1.108	1	-0.588	-1.504	0.046
1383161_a_at	---	---	Any2_423	1	10.50	9.84	9.78	10.57	1	-0.660	-1.580	0.008	1	-0.715	-1.641	0.001	0	0.074	1.053	1.015
1383162_at	---	---	Any2_423	1	10.84	10.18	10.20	10.53	1	-0.657	-1.577	0.008	1	-0.633	-1.551	0.007	0	-0.311	-1.240	0.477
1383188_at	---	---	Any2_423	1	5.66	6.08	6.48	5.09	0	0.419	1.337	0.306	1	0.822	1.767	0.010	1	-0.569	-1.483	0.078
1383241_at	C1r	complement component 1, r subcomponent	Any2_423	1	8.10	7.56	7.71	7.05	1	-0.541	-1.455	0.056	0	-0.392	-1.312	0.248	1	-1.043	-2.061	0.001
1383302_at	Dnajb1	DnaJ (Hsp40) homolog, subfamily B, member 1	Any2_423	1	8.44	9.59	7.27	7.93	1	1.157	2.229	0.003	1	-1.169	-2.249	0.000	0	-0.503	-1.418	0.112
1383437_at	Rab17	RAB17, member RAS oncogene family	Any2_423	1	4.19	3.64	3.91	3.91	1	-0.554	-1.468	0.044	0	-0.286	-1.220	0.442	0	-0.283	-1.217	0.521
1383460_at	---	---	Any2_423	1	5.96	4.55	4.75	4.81	1	-1.415	-2.667	0.000	1	-1.211	-2.315	0.000	1	-1.149	-2.217	0.001
1383767_at	---	---	Any2_423	1	5.80	6.25	6.42	6.81	0	0.455	1.371	0.259	1	0.623	1.541	0.046	1	1.012	2.016	0.026
1383867_at	Eif5a2	eukaryotic translation initiation factor 5A2	Any2_423	1	5.04	5.73	5.73	5.91	1	0.698	1.622	0.049	1	0.693	1.617	0.020	1	0.874	1.833	0.051
1383875_at	Upk1b	uroplakin 1B	Any2_423	1	4.36	3.02	2.96	3.40	1	-1.336	-2.525	0.000	1	-1.398	-2.636	0.000	1	-0.960	-1.946	0.003
1383952_at	Mical1	microtubule associated monooxygenase, calponin a	Any2_423	1	4.70	4.46	4.53	4.09	0	-0.243	-1.184	0.519	0	-0.171	-1.126	0.665	1	-0.610	-1.526	0.049
1384184_at	---	---	Any2_423	1	6.76	7.50	7.37	7.44	1	0.740	1.670	0.038	1	0.613	1.529	0.056	0	0.685	1.607	0.122
1384218_at	LOC362795	immunoglobulin G heavy chain	Any2_423	1	5.61	5.38	5.10	5.21	0	-0.227	-1.170	0.633	1	-0.510	-1.425	0.058	0	-0.399	-1.319	0.242
1384245_at	---	---	Any2_423	1	6.66	6.15	6.33	6.12	1	-0.517	-1.431	0.075	0	-0.332	-1.259	0.378	1	-0.547	-1.461	0.065
1384392_at	Cyp26b1	cytochrome P450, family 26, subfamily b, polypepti	Any2_423	1	4.84	3.94	4.37	4.06	1	-0.907	-1.875	0.000	0	-0.476	-1.391	0.094	1	-0.785	-1.723	0.010
1384515_at	Hey2	hairy/enhancer-of-split related with YRPW motif 2	Any2_423	1	6.18	5.63	5.77	5.44	1	-0.554	-1.468	0.043	0	-0.417	-1.335	0.190	1	-0.746	-1.677	0.021
1384667_x_at	Galr2 /// LOC	galanin receptor 2 /// galanin receptor type 2-like	Any2_423	1	4.48	5.20	5.37	4.94	1	0.714	1.640	0.052	1	0.883	1.845	0.006	0	0.457	1.372	0.421
1384778_at	Ctrc	chymotrypsin C (caldecrin)	Any2_423	1	11.46	11.98	10.94	12.47	0	0.522	1.436	0.201	1	-0.520	-1.433	0.073	1	1.011	2.015	0.031
1384816_at	Cxadr	coxsackie virus and adenovirus receptor	Any2_423	1	4.86	5.25	5.40	5.47	0	0.386	1.307	0.333	1	0.545	1.459	0.089	0	0.607	1.524	0.236
1384940_at	Zfp518a	zinc finger protein 518A	Any2_423	1	6.14	6.78	6.69	6.67	1	0.638	1.556	0.095	1	0.554	1.468	0.090	0	0.527	1.441	0.298
1384960_at	Cftr	cystic fibrosis transmembrane conductance regulato	Any2_423	1	4.33	3.79	3.81	4.11	1	-0.543	-1.457	0.052	1	-0.523	-1.437	0.051	0	-0.221	-1.165	0.715
1385025_a_at	Gpr137b	G protein-coupled receptor 137B	Any2_423	1	6.46	7.19	6.65	7.23	1	0.728	1.656	0.043	0	0.191	1.142	0.782	1	0.767	1.702	0.090
1385121_at	Lsm7	LSM7 homolog, U6 small nuclear RNA associated (S.	Any2_423	1	4.69	4.10	4.52	4.15	1	-0.590	-1.506	0.027	0	-0.163	-1.120	0.822	1	-0.537	-1.451	0.078
1385155_at	Lrrfip1	leucine rich repeat (in FLII) interacting protein 1	Any2_423	1	4.96	4.61	4.52	4.41	0	-0.349	-1.274	0.352	0	-0.443	-1.359	0.138	1	-0.558	-1.472	0.085
1385170_at	Fstl5	follistatin-like 5	Any2_423	1	6.89	7.56	7.49	5.93	1	0.667	1.588	0.061	1	0.601	1.516	0.061	1	-0.955	-1.939	0.003
1385171_s_at	Smtnl2	smoothelin-like 2	Any2_423	1	5.57	5.06	5.08	5.03	1	-0.506	-1.420	0.085	0	-0.492	-1.406	0.064	1	-0.537	-1.450	0.078
1385426_at	Ccdc109b	coiled-coil domain containing 109B	Any2_423	1	4.03	4.20	4.12	3.49	0	0.163	1.120	0.901	0	0.086	1.061	1.020	1	-0.540	-1.454	0.076
1385444_at	---	---	Any2_423	1	6.97	6.30	6.59	5.95	1	-0.671	-1.592	0.008	0	-0.377	-1.299	0.268	1	-1.019	-2.026	0.002
1385486_at	Bnc2	basonuclin 2	Any2_423	1	4.18	3.49	3.60	3.66	1	-0.694	-1.618	0.009	1	-0.580	-1.494	0.038	0	-0.520	-1.434	0.119
1385620_at	Hsph1	heat shock 105/110 protein 1	Any2_423	1	7.71	7.83	6.72	6.94	0	0.120	1.087	1.011	1	-0.992	-1.988	0.000	1	-0.774	-1.710	0.009
1386023_at	Lgi1	leucine-rich, glioma inactivated 1	Any2_423	1	4.05	4.90	4.81	3.87	1	0.846	1.797	0.016	1	0.757	1.690	0.009	0	-0.188	-1.139	0.738
1386306_at	---	---	Any2_423	1	6.55	5.96	6.25	5.58	1	-0.591	-1.506	0.026	0	-0.303	-1.233	0.432	1	-0.980	-1.972	0.002
1386321_s_at	Trib3	tribbles homolog 3 (Drosophila)	Any2_423	1	6.51	7.48	7.27	7.25	1	0.964	1.951	0.008	1	0.760	1.694	0.011	0	0.740	1.670	0.101
1386482_at	---	---	Any2_423	1	6.78	7.72	7.16	7.57	1	0.946	1.926	0.010	0	0.382	1.303	0.325	1	0.790	1.729	0.073
1386641_at	LOC690286	similar to hepatic leukemia factor	Any2_423	1	6.63	7.55	7.39	6.95	1	0.917	1.888	0.010	1	0.759	1.692	0.008	0	0.318	1.246	0.664
1386869_at	Actg2	actin, gamma 2, smooth muscle, enteric	Any2_423	1	4.80	4.65	4.03	3.84	0	-0.151	-1.110	0.709	1	-0.765	-1.700	0.001	1	-0.961	-1.946	0.003
1386879_at	Lgals3	lectin, galactoside-binding, soluble, 3	Any2_423	1	7.27	6.84	6.71	6.61	0	-0.432	-1.349	0.178	1	-0.563	-1.477	0.033	1	-0.663	-1.584	0.030
1386890_at	S100a10	S100 calcium binding protein A10	Any2_423	1	8.86	8.38	8.57	8.07	0	-0.482	-1.397	0.110	0	-0.285	-1.218	0.478	1	-0.787	-1.725	0.008
1386933_at	Gp2	glycoprotein 2 (zymogen granule membrane)	Any2_423	1	8.86	9.68	8.23	10.17	1	0.816	1.761	0.022	1	-0.632	-1.549	0.011	1	1.306	2.472	0.011
1386981_at	Slc16a1	solute carrier family 16, member 1 (monocarboxylic	Any2_423	1	4.94	4.69	4.56	4.19	0	-0.247	-1.187	0.643	0	-0.380	-1.302	0.267	1	-0.741	-1.672	0.012
1387022_at	Aldh1a1	aldehyde dehydrogenase 1 family, member A1	Any2_423	1	7.04	6.22	6.65	6.12	1	-0.825	-1.771	0.000	0	-0.393	-1.313	0.207	1	-0.921	-1.893	0.003
1387040_at	Mal	mal, T-cell differentiation protein	Any2_423	1	4.26	3.73	3.94	4.04	1	-0.525	-1.439	0.060	0	-0.322	-1.250	0.379	0	-0.217	-1.162	0.695
1387068_at	Arc	activity-regulated cytoskeleton-associated protein	Any2_423	1	7.33	8.18	7.98	8.29	1	0.858	1.812	0.016	1	0.652	1.571	0.042	1	0.962	1.948	0.036
1387173_at	Cma1	chymase 1, mast cell	Any2_423	1	4.12	3.58	3.75	4.01	1	-0.537	-1.451	0.055	0	-0.365	-1.288	0.298	0	-0.113	-1.081	1.031
1387179_at	Adcy8	adenylate cyclase 8 (brain)	Any2_423	1	4.42	5.07	4.89	5.56	1	0.642	1.561	0.094	0	0.469	1.384	0.193	1	1.136	2.198	0.018
1387193_a_at	Spink3	serine peptidase inhibitor, Kazal type 3	Any2_423	1	7.67	8.60	7.40	8.99	1	0.925	1.899	0.010	0	-0.270	-1.206	0.479	1	1.313	2.485	0.011
1387254_at	Ghrl	ghrelin/obestatin prepropeptide	Any2_423	1	8.67	9.58	9.03	7.26	1	0.902	1.869	0.010	0	0.351	1.275	0.377	1	-1.413	-2.662	0.000
1387276_at	Dclk1	doublecortin-like kinase 1	Any2_423	1	4.16	3.28	3.62	3.52	1	-0.887	-1.849	0.000	1	-0.544	-1.458	0.033	1	-0.641	-1.560	0.033
1387294_at	Sh3bp5	SH3-domain binding protein 5 (BTK-associated)	Any2_423	1	8.34	8.40	8.47	7.80	0	0.059	1.042	1.134	0	0.124	1.090	0.975	1	-0.546	-1.460	0.069
1387348_at	Igfbp5	insulin-like growth factor binding protein 5	Any2_423	1	7.50	6.46	6.54	6.08	1	-1.041	-2.057	0.000	1	-0.960	-1.946	0.000	1	-1.425	-2.686	0.000
1387351_at	Fbn1	fibrillin 1	Any2_423	1	7.08	6.24	6.47	6.15	1	-0.845	-1.797	0.000	1	-0.611	-1.527	0.012	1	-0.938	-1.915	0.003
1387459_at	Pkib	protein kinase (cAMP-dependent, catalytic) inhibito	Any2_423	1	5.65	6.02	6.13	4.06	0	0.374	1.296	0.394	0	0.485	1.399	0.171	1	-1.587	-3.004	0.000
1387472_at	Cd3d	CD3 molecule, delta	Any2_423	1	4.44	4.27	4.28	3.93	0	-0.172	-1.126	0.802	0	-0.155	-1.114	0.799	1	-0.505	-1.419	0.097
1387516_at	Pnliprp2	pancreatic lipase-related protein 2	Any2_423	1	9.80	11.00	9.25	11.40	1	1.195	2.290	0.001	1	-0.554	-1.468	0.038	1	1.596	3.023	0.005
1387625_at	Igfbp6	insulin-like growth factor binding protein 6	Any2_423	1	5.10	4.15	4.34	4.36	1	-0.946	-1.927	0.000	1	-0.756	-1.689	0.002	1	-0.739	-1.669	0.018
1387659_at	Gda	guanine deaminase	Any2_423	1	6.15	5.83	5.79	5.42	0	-0.317	-1.245	0.412	0	-0.356	-1.280	0.316	1	-0.726	-1.654	0.015
1387700_at	Cckar	cholecystokinin A receptor	Any2_423	1	6.92	6.27	6.18	6.48	1	-0.653	-1.572	0.014	1	-0.743	-1.673	0.003	0	-0.441	-1.358	0.184
1387702_at	Galr1	galanin receptor 1	Any2_423	1	4.19	4.88	4.67	5.27	1	0.690	1.613	0.065	0	0.477	1.392	0.155	1	1.075	2.107	0.025
1387749_at	Cd79b	Cd79b molecule, immunoglobulin-associated beta	Any2_423	1	4.61	4.34	4.59	3.94	0	-0.277	-1.212	0.434	0	-0.027	-1.019	1.085	1	-0.675	-1.597	0.026
1387760_a_at	Onecut1	one cut homeobox 1	Any2_423	1	3.92	3.40	3.74	3.65	1	-0.526	-1.440	0.066	0	-0.178	-1.131	0.777	0	-0.272	-1.207	0.575

Affymetrix Probeset ID	Gene Symbol	Gene Title	Figure 7 A Venn n=2289	Present in any 2 analyses n=423 1=yes 0=no	Mean log2 intensity DR+/+ ND	Mean log2 intensity DR+/+ HCD	Mean log2 intensity DR+/+ B/S	Mean log2 intensity F+/+ day 40 ND	DR+/+HCD vs DR+/+ND FDR<10% Log2 ratio >0.5 n=636	Log2 ratio DR+/+HCD vs DR+/+ ND	Fold-Change DR+/+HCD vs DR+/+ ND	FDR DR+/+HCD vs DR+/+ ND	DR+/+B/S vs DR+/+ND Log2 ratio >0.5 n=350	Log2 ratio DR+/+ B/S vs DR+/+ ND	Fold-Change DR+/+B /S vs DR+/+ ND	FDR DR+/+B /S vs DR+/+ ND	F+/+ vs DR+/+N D Log2 ratio >0.5 n=1209	Log2 ratio F+/+ vs DR+/+ ND	Fold-Change F+/+ vs DR+/+ ND	FDR F+/+ vs DR+/+ ND
1387770_at	Ifi27	interferon, alpha-inducible protein 27	Any2_423	1	8.96	8.40	8.73	10.18	1	-0.566	-1.480	0.040	0	-0.237	-1.178	0.603	1	1.220	2.330	0.013
1387854_at	Col1a2 /// LO	collagen, type I, alpha 2 /// collagen alpha-2(I) chain	Any2_423	1	8.00	7.29	7.40	6.60	1	-0.705	-1.630	0.004	1	-0.604	-1.519	0.014	1	-1.402	-2.643	0.000
1387893_at	C1s	complement component 1, s subcomponent	Any2_423	1	7.72	7.03	7.12	6.64	1	-0.682	-1.605	0.008	1	-0.598	-1.514	0.017	1	-1.073	-2.104	0.001
1387902_a_at	LOC500183	similar to NGF-binding Ig light chain	Any2_423	1	7.61	7.17	6.86	5.77	0	-0.438	-1.355	0.113	1	-0.749	-1.681	0.016	1	-1.843	-3.587	0.000
1387967_at	LOC1009115	serine protease inhibitor Kazal-type 3-like /// serine	Any2_423	1	7.53	8.60	7.12	9.21	1	1.066	2.094	0.003	0	-0.416	-1.334	0.198	1	1.675	3.193	0.005
1388111_at	Eln	elastin	Any2_423	1	6.45	5.66	5.73	5.85	1	-0.784	-1.721	0.000	1	-0.718	-1.644	0.001	1	-0.592	-1.507	0.042
1388116_at	Col1a1	collagen, type I, alpha 1	Any2_423	1	6.80	6.16	6.33	6.11	1	-0.640	-1.558	0.009	0	-0.469	-1.384	0.093	1	-0.690	-1.613	0.022
1388138_at	Thbs4	thrombospondin 4	Any2_423	1	7.65	6.94	7.50	5.74	1	-0.701	-1.625	0.004	0	-0.144	-1.105	0.743	1	-1.901	-3.735	0.000
1388145_at	Tnxb	tenascin XB	Any2_423	1	5.33	4.81	4.69	4.59	1	-0.523	-1.437	0.068	1	-0.637	-1.555	0.006	1	-0.741	-1.671	0.012
1388166_at	Igh-6	immunoglobulin heavy chain 6	Any2_423	1	6.47	5.87	5.95	5.38	1	-0.597	-1.513	0.043	1	-0.521	-1.435	0.094	1	-1.089	-2.128	0.003
1388223_at	Gnat3	guanine nucleotide binding protein, alpha transduci	Any2_423	1	6.00	7.08	6.59	5.90	1	1.076	2.108	0.003	1	0.584	1.499	0.072	0	-0.105	-1.076	1.047
1388298_at	Myl9	myosin, light chain 9, regulatory	Any2_423	1	8.34	7.72	7.49	6.98	1	-0.622	-1.539	0.019	1	-0.846	-1.798	0.000	1	-1.354	-2.556	0.000
1388433_at	Krt19	keratin 19	Any2_423	1	8.68	8.65	8.53	5.64	0	-0.034	-1.024	1.077	0	-0.154	-1.113	0.798	1	-3.044	-8.248	0.000
1388460_at	Capg	capping protein (actin filament), gelsolin-like	Any2_423	1	5.25	4.61	4.70	5.04	1	-0.632	-1.550	0.013	1	-0.546	-1.460	0.034	0	-0.209	-1.156	0.705
1388479_at	---	---	Any2_423	1	6.22	5.74	5.61	5.62	0	-0.473	-1.388	0.132	1	-0.604	-1.520	0.011	1	-0.600	-1.515	0.045
1388569_at	Serpinf1	serpin peptidase inhibitor, clade F (alpha-2 antiplasr	Any2_423	1	6.86	6.19	6.20	6.06	1	-0.675	-1.596	0.006	1	-0.659	-1.579	0.003	1	-0.804	-1.746	0.007
1388587_at	Ier3	immediate early response 3	Any2_423	1	8.20	8.12	7.32	7.99	0	-0.083	-1.059	0.836	1	-0.886	-1.848	0.000	0	-0.209	-1.156	0.714
1388611_at	Tcea3	transcription elongation factor A (SII), 3	Any2_423	1	5.90	5.39	5.35	5.19	1	-0.509	-1.423	0.085	1	-0.548	-1.462	0.033	1	-0.708	-1.633	0.018
1388673_at	Lsp1	lymphocyte-specific protein 1	Any2_423	1	4.90	4.36	4.64	4.21	1	-0.546	-1.460	0.051	0	-0.268	-1.204	0.546	1	-0.690	-1.614	0.019
1388722_at	Dnajb1	DnaJ (Hsp40) homolog, subfamily B, member 1	Any2_423	1	9.73	10.28	8.91	8.93	0	0.545	1.459	0.165	1	-0.821	-1.767	0.000	1	-0.803	-1.745	0.008
1388785_at	Dnal4 /// RGI	dynein, axonemal, light chain 4 /// similar to Dynein	Any2_423	1	5.99	6.63	6.55	6.59	1	0.642	1.560	0.084	1	0.562	1.476	0.089	0	0.601	1.517	0.207
1388879_at	Abi3bp	ABI family, member 3 (NESH) binding protein	Any2_423	1	6.49	5.85	5.80	5.23	1	-0.644	-1.563	0.011	1	-0.695	-1.619	0.004	1	-1.261	-2.396	0.000
1388898_at	Hsph1	heat shock 105/110 protein 1	Any2_423	1	9.77	9.69	8.87	8.92	0	-0.086	-1.061	1.079	1	-0.898	-1.864	0.000	1	-0.852	-1.805	0.006
1388955_at	---	---	Any2_423	1	7.08	6.57	6.64	6.37	1	-0.507	-1.421	0.085	0	-0.436	-1.353	0.150	1	-0.712	-1.638	0.014
1388985_at	---	---	Any2_423	1	8.04	7.03	7.27	6.98	1	-1.011	-2.015	0.000	1	-0.766	-1.700	0.001	1	-1.059	-2.084	0.001
1389071_at	Ehbp1l1	EH domain binding protein 1-like 1	Any2_423	1	6.38	6.14	6.06	5.71	0	-0.242	-1.182	0.592	0	-0.323	-1.251	0.335	1	-0.673	-1.594	0.022
1389234_at	Vwf	von Willebrand factor	Any2_423	1	6.42	5.58	5.65	6.09	1	-0.838	-1.788	0.000	1	-0.768	-1.703	0.001	0	-0.332	-1.259	0.402
1389306_at	Matn2	matrilin 2	Any2_423	1	8.07	7.16	7.68	7.40	1	-0.914	-1.884	0.000	0	-0.388	-1.309	0.236	1	-0.670	-1.591	0.023
1389470_at	C2 /// Cfb	complement component 2 /// complement factor B	Any2_423	1	6.62	4.76	4.78	5.17	1	-1.865	-3.642	0.000	1	-1.848	-3.601	0.000	1	-1.453	-2.738	0.000
1389528_s_at	Jun	jun proto-oncogene	Any2_423	1	11.07	11.52	10.45	10.16	0	0.450	1.366	0.288	1	-0.621	-1.538	0.013	1	-0.910	-1.879	0.004
1389533_at	Fbln2	fibulin 2	Any2_423	1	6.80	5.85	5.66	5.87	1	-0.958	-1.943	0.000	1	-1.141	-2.206	0.000	1	-0.936	-1.914	0.003
1389579_at	---	---	Any2_423	1	6.57	7.62	6.51	5.93	1	1.043	2.061	0.004	0	-0.065	-1.046	1.012	1	-0.646	-1.565	0.032
1389581_at	Il33	interleukin 33	Any2_423	1	5.27	4.64	4.65	4.62	1	-0.628	-1.546	0.024	1	-0.620	-1.537	0.020	1	-0.651	-1.571	0.038
1389604_at	Hspa12b	heat shock protein 12B	Any2_423	1	6.30	5.75	5.76	5.85	1	-0.546	-1.460	0.044	1	-0.535	-1.449	0.033	0	-0.447	-1.363	0.165
1389966_at	Col6a3	collagen, type VI, alpha 3	Any2_423	1	7.92	7.25	7.21	6.50	1	-0.665	-1.585	0.008	1	-0.711	-1.637	0.001	1	-1.416	-2.669	0.000
1389998_at	Nr2f2	nuclear receptor subfamily 2, group F, member 2	Any2_423	1	6.03	5.51	5.62	4.98	1	-0.519	-1.433	0.070	0	-0.411	-1.330	0.190	1	-1.047	-2.067	0.001
1390109_at	LOC1009129	uncharacterized LOC100912996	Any2_423	1	9.05	9.88	9.74	9.21	1	0.833	1.782	0.017	1	0.695	1.619	0.019	0	0.162	1.119	0.970
1390112_at	Efemp1	EGF-containing fibulin-like extracellular matrix prote	Any2_423	1	4.48	3.10	3.11	3.17	1	-1.381	-2.605	0.000	1	-1.370	-2.585	0.000	1	-1.314	-2.487	0.000
1390459_at	---	---	Any2_423	1	4.59	4.00	4.07	4.54	1	-0.595	-1.510	0.024	1	-0.521	-1.435	0.050	0	-0.046	-1.032	1.156
1390495_at	---	---	Any2_423	1	5.31	4.77	4.79	4.76	1	-0.544	-1.458	0.046	1	-0.521	-1.435	0.037	1	-0.550	-1.464	0.071
1390707_at	Rgs10	regulator of G-protein signaling 10	Any2_423	1	5.47	5.28	5.33	4.76	0	-0.188	-1.139	0.565	0	-0.135	-1.098	0.819	1	-0.710	-1.636	0.016
1390738_at	Bst2	bone marrow stromal cell antigen 2	Any2_423	1	7.97	7.40	7.83	6.97	1	-0.568	-1.482	0.039	0	-0.138	-1.100	0.890	1	-1.007	-2.010	0.002
1391075_at	Rgs17	regulator of G-protein signaling 17	Any2_423	1	8.17	8.80	8.51	9.06	1	0.632	1.549	0.085	0	0.342	1.268	0.400	1	0.883	1.844	0.042
1391162_at	Dcdc2	doublecortin domain containing 2	Any2_423	1	5.67	5.08	5.27	5.76	1	-0.593	-1.508	0.027	0	-0.405	-1.324	0.213	0	0.084	1.060	1.061
1391264_at	---	---	Any2_423	1	5.80	5.18	5.26	5.09	1	-0.627	-1.544	0.018	1	-0.539	-1.453	0.046	1	-0.708	-1.633	0.023
1391352_at	Erp27	endoplasmic reticulum protein 27	Any2_423	1	6.46	7.57	5.90	7.70	1	1.112	2.162	0.003	1	-0.559	-1.474	0.040	1	1.241	2.364	0.013
1391561_at	---	---	Any2_423	1	4.40	5.03	4.60	5.31	1	0.634	1.552	0.095	0	0.207	1.154	0.682	1	0.916	1.886	0.041
1391910_at	Rftn1	raftlin lipid raft linker 1	Any2_423	1	6.63	6.12	6.24	6.34	1	-0.503	-1.417	0.085	0	-0.382	-1.304	0.258	0	-0.286	-1.219	0.529
1392053_at	Mmrn1	multimerin 1	Any2_423	1	4.68	3.55	3.43	3.24	1	-1.124	-2.180	0.000	1	-1.245	-2.371	0.000	1	-1.437	-2.708	0.000
1392117_at	---	---	Any2_423	1	5.55	4.80	5.24	4.82	1	-0.752	-1.684	0.003	0	-0.312	-1.241	0.357	1	-0.730	-1.659	0.016
1392171_at	Chi3l1	chitinase 3-like 1 (cartilage glycoprotein-39)	Any2_423	1	5.31	4.79	5.04	4.99	1	-0.522	-1.436	0.068	0	-0.270	-1.206	0.528	0	-0.323	-1.251	0.414
1392274_at	---	---	Any2_423	1	5.78	5.20	5.35	4.99	1	-0.580	-1.495	0.041	0	-0.428	-1.345	0.171	1	-0.788	-1.726	0.013
1392349_at	Slc5a3	solute carrier family 5 (sodium/myo-inositol cotrans	Any2_423	1	6.08	6.84	5.99	7.29	1	0.755	1.687	0.038	0	-0.095	-1.068	0.915	1	1.212	2.317	0.013
1392413_at	---	---	Any2_423	1	3.59	4.79	4.34	4.24	1	1.202	2.301	0.001	1	0.751	1.683	0.010	0	0.650	1.569	0.150
1392444_at	Chst5	carbohydrate (N-acetylglucosamine 6-O) sulfotransf	Any2_423	1	6.38	5.94	6.41	4.98	0	-0.435	-1.352	0.181	0	0.034	1.024	1.121	1	-1.397	-2.634	0.000
1392496_at	LOC312273	Trypsin V-A	Any2_423	1	6.48	6.93	7.45	10.58	0	0.456	1.372	0.253	1	0.977	1.968	0.000	1	4.102	17.167	0.000
1392640_at	Cry1	cryptochrome 1 (photolyase-like)	Any2_423	1	6.34	7.02	7.11	6.69	1	0.684	1.607	0.054	1	0.775	1.711	0.011	0	0.356	1.280	0.584
1392880_at	---	---	Any2_423	1	6.84	7.87	7.60	6.15	1	1.031	2.044	0.004	1	0.758	1.691	0.010	1	-0.688	-1.611	0.023
1392886_a_at	Fcrla	Fc receptor-like A	Any2_423	1	6.21	5.28	6.14	6.08	1	-0.929	-1.904	0.002	0	-0.063	-1.044	0.555	0	-0.126	-1.091	0.634
1392965_a_at	LOC10009098	SPARC-related modular calcium-binding protein 2-lil	Any2_423	1	7.27	6.51	6.88	5.58	1	-0.757	-1.690	0.001	0	-0.387	-1.308	0.224	1	-1.692	-3.232	0.000
1393039_a_at	Rab17	RAB17, member RAS oncogene family	Any2_423	1	6.26	5.80	5.89	5.63	0	-0.461	-1.376	0.148	0	-0.363	-1.286	0.303	1	-0.622	-1.539	0.036
1393119_at	---	---	Any2_423	1	6.38	7.24	6.96	7.59	1	0.851	1.804	0.016	1	0.580	1.495	0.073	1	1.210	2.314	0.013
1393210_at	LOC1003613	extracellular matrix protein 2-like	Any2_423	1	4.76	4.97	4.76	4.15	0	0.211	1.157	0.690	0	0.005	1.004	1.126	1	-0.606	-1.522	0.054
1393250_at	---	---	Any2_423	1	5.34	4.65	4.55	4.94	1	-0.695	-1.619	0.004	1	-0.786	-1.724	0.001	0	-0.396	-1.316	0.245
1393260_at	LOC360228	WDNM1 homolog	Any2_423	1	5.95	4.65	4.92	4.84	1	-1.302	-2.466	0.000	1	-1.030	-2.042	0.000	1	-1.111	-2.160	0.001
1393350_at	LOC1009113	uncharacterized LOC100911349	Any2_423	1	4.18	4.82	4.77	4.73	1	0.646	1.565	0.076	1	0.594	1.510	0.070	0	0.551	1.465	0.271
1393401_at	---	---	Any2_423	1	7.13	6.57	6.67	6.29	1	-0.557	-1.471	0.044	0	-0.457	-1.373	0.111	1	-0.840	-1.790	0.007
1393413_at	LOC1009114	neuronal tyrosine-phosphorylated phosphoinositide	Any2_423	1	6.13	6.18	6.25	5.49	0	0.050	1.035	1.134	0	0.115	1.083	0.973	1	-0.640	-1.558	0.060
1393436_at	Scgb1c1	secretoglobin, family 1C, member 1	Any2_423	1	5.43	4.69	4.86	4.87	1	-0.741	-1.671	0.003	1	-0.566	-1.480	0.030	1	-0.554	-1.468	0.069
1393638_at	---	---	Any2_423	1	4.47	3.98	4.09	3.82	0	-0.483	-1.398	0.122	0	-0.379	-1.300	0.290	1	-0.646	-1.565	0.029
1393964_at	LOC1003640	reproductive homeobox 9-like /// reproductive hom	Any2_423	1	4.87	4.27	4.52	4.19	1	-0.597	-1.512	0.022	0	-0.350	-1.274	0.316	1	-0.676	-1.598	0.022
1394022_at	Id4	inhibitor of DNA binding 4	Any2_423	1	5.80	4.85	4.78	4.60	1	-0.951	-1.933	0.000	1	-1.017	-2.024	0.000	1	-1.196	-2.290	0.001
1394264_at	Chst5	carbohydrate (N-acetylglucosamine 6-O) sulfotransf	Any2_423	1	5.55	5.27	5.47	4.96	0	-0.276	-1.211	0.560	0	-0.079	-1.056	1.029	1	-0.582	-1.497	0.047
1394451_at	Anxa1	annexin A1	Any2_423	1	7.88	7.22	6.90	7.04	1	-0.656	-1.576	0.009	1	-0.975	-1.966	0.000	1	-0.832	-1.780	0.007
1394528_at	---	---	Any2_423	1	5.54	6.28	5.69	6.96	1	0.746	1.677	0.040	0	0.148	1.108	0.848	1	1.425	2.685	0.007
1394701_at	---	---	Any2_423	1	5.56	6.04	5.03	7.25	0	0.477	1.392	0.264	1	-0.532	-1.446	0.046	1	1.687	3.220	0.006
1394742_at	---	---	Any2_423	1	7.08	6.52	7.29	6.42	1	-0.553	-1.467	0.043	0	0.211	1.158	0.677	1	-0.657	-1.577	0.025
1394745_at	---	---	Any2_423	1	4.72	5.91	4.92	7.59	1	1.195	2.290	0.001	0	0.203	1.151	0.425	1	2.872	7.318	0.000
1395052_at	---	---	Any2_423	1	6.56	7.20	7.33	5.72	1	0.639	1.557	0.084	1	0.769	1.704	0.013	1	-0.842	-1.793	0.007
1395255_at	---	---	Any2_423	1	4.22	3.21	3.68	4.03	1	-1.009	-2.013	0.000	1	-0.540	-1.454	0.072	0	-0.195	-1.145	0.801
1395471_at	---	---	Any2_423	1	5.90	6.80	5.81	7.10	1	0.900	1.866	0.010	0	-0.092	-1.066	0.850	1	1.202	2.300	0.013
1395557_at	Klf6	Kruppel-like factor 6	Any2_423	1	6.62	7.44	5.85	7.65	1	0.826	1.772	0.028	1	-0.772	-1.708	0.005	1	1.036	2.051	0.039
1395663_at	Mall	mal, T-cell differentiation protein-like	Any2_423	1	4.78	4.08	4.18	4.23	1	-0.701	-1.626	0.004	1	-0.597	-1.513	0.013	1	-0.548	-1.462	0.073
1395753_at	Eln	elastin	Any2_423	1	5.94	5.20	5.35	5.42	1	-0.739	-1.669	0.003	1	-0.590	-1.505	0.016	1	-0.514	-1.428	0.100
1396486_x_at	RGD1564162	similar to Homo sapiens fetal lung specific expressio	Any2_423	1	6.23	5.65	6.02	5.53	1	-0.578	-1.493	0.027	0	-0.210	-1.157	0.683	1	-0.698	-1.622	0.022
1396800_at	---	---	Any2_423	1	4.59	4.07	4.18	3.66	1	-0.522	-1.436	0.070	0	-0.417	-1.335	0.184	1	-0.931	-1.906	0.004
1396916_at	---	---	Any2_423	1	5.53	4.66	4.72	4.65	1	-0.864	-1.820	0.000	1	-0.810	-1.753	0.000	1	-0.882	-1.842	0.005
1396933_s_at	Akr1c14	aldo-keto reductase family 1, member C14	Any2_423	1	4.58	4.01	4.06	4.66	1	-0.571	-1.486	0.034	1	-0.527	-1.440	0.043	0	0.076	1.054	1.062
1396957_at	---	---	Any2_423	1	6.79	6.27	6.41	6.29	1	-0.519	-1.433	0.070	0	-0.377	-1.298	0.283	1	-0.501	-1.415	0.096
1397227_at	Tmem35	transmembrane protein 35	Any2_423	1	7.91	6.75	7.68	6.68	1	-1.152	-2.223	0.000	0	-0.229	-1.172	0.603	1	-1.221	-2.332	0.000
1397356_at	---	---	Any2_423	1	5.66	6.32	6.09	6.43	1	0.666	1.586	0.078	0	0.436	1.353	0.246	1	0.775	1.711	0.091
1397406_at	---	---	Any2_423	1	5.41	6.06	6.02	5.89	1	0.652	1.571	0.095	1	0.611	1.527	0.067	0	0.485	1.400	0.358
1397504_at	Wsb2	WD repeat and SOCS box-containing 2	Any2_423	1	5.81	5.29	5.50	5.26	1	-0.520	-1.434	0.075	0	-0.313	-1.242	0.432	1	-0.553	-1.467	0.071
1397684_at	Dkk2	dickkopf WNT signaling pathway inhibitor 2	Any2_423	1	4.17	4.01	3.99	3.61	0	-0.168	-1.123	0.625	0	-0.185	-1.137	0.636	1	-0.565	-1.480	0.080
1397973_x_at	LOC1003632	Temporarily Assigned Gene name family member (ta	Any2_423	1	5.77	5.08	5.55	4.44	1	-0.688	-1.611	0.006	0	-0.213	-1.159	0.645	1	-1.322	-2.501	0.000
1397999_at	---	---	Any2_423	1	7.65	8.39	8.25	8.96	1	0.740	1.670	0.043	1	0.599	1.515	0.073	1	1.311	2.480	0.011
1398095_at	---	---	Any2_423	1	5.12	4.53	4.79	4.60	1	-0.587	-1.502	0.027	0	-0.331	-1.258	0.381	1	-0.521	-1.435	0.097
1398245_at	Sncg	synuclein, gamma (breast cancer-specific protein 1)	Any2_423	1	7.60	6.88	7.07	6.62	1	-0.718	-1.645	0.004	1	-0.528	-1.441	0.052	1	-0.982	-1.975	0.002
1398345_at	---	---	Any2_423	1	6.72	6.07	6.20	6.18	1	-0.654	-1.574	0.009	1	-0.526	-1.440	0.035	1	-0.543	-1.457	0.076
1398387_at	Fam198b	family with sequence similarity 198, member B	Any2_423	1	6.53	6.01	6.54	4.65	1	-0.511	-1.425	0.072	0	0.010	1.007	1.042	1	-1.872	-3.661	0.000
1398407_at	Tnfaip8	tumor necrosis factor, alpha-induced protein 8	Any2_423	1	3.55	3.33	3.36	3.03	0	-0.223	-1.167	0.637	0	-0.192	-1.143	0.715	1	-0.516	-1.430	0.100
1398564_at	---	---	Any2_423	1	5.97	5.42	5.78	4.90	1	-0.554	-1.469	0.051	0	-0.196	-1.145	0.730	1	-1.076	-2.108	0.001
1398617_at	---	---	Any2_423	1	5.51	6.26	6.23	6.19	1	0.746	1.677	0.035	1	0.721	1.648	0.015	0	0.674	1.596	0.140
1387060_at	Klf6	Kruppel-like factor 6	uniq_BSvsDR_106	0	8.91	9.11	8.32	8.94	0	0.192	1.143	0.784	1	-0.595	-1.510	0.023	0	0.026	1.018	1.093
1367626_at	Ckm	creatine kinase, muscle	uniq_BSvsDR_106	0	5.62	5.28	5.06	5.66	0	-0.337	-1.263	0.414	1	-0.557	-1.471	0.021	0	0.040	1.028	1.006
1367631_at	Ctgf	connective tissue growth factor	uniq_BSvsDR_106	0	8.60	8.23	7.88	8.85	0	-0.373	-1.295	0.315	1	-0.728	-1.656	0.002	0	0.241	1.181	0.823
1367851_at	Ptgds	prostaglandin D2 synthase (brain)	uniq_BSvsDR_106	0	6.56	6.95	7.16	7.20	0	0.389	1.310	0.388	1	0.598	1.513	0.076	0	0.642	1.560	0.176

Affymetrix Probeset ID	Gene Symbol	Gene Title	Figure 7 A Venn n=2289	Present in any 2 analyses n=423 1=yes 0=no	Mean log2 intensity DR+/+ ND	Mean log2 intensity DR+/+ HCD	Mean log2 intensity DR+/+ B/S	Mean log2 intensity F+/+ day 40 ND	DR+/+HCD vs DR+/+ND FDR<10% Log2 ratio >0.5 n=636	Log2 ratio DR+/+HCD vs DR+/+ ND	Fold-Change DR+/+HCD vs DR+/+ ND	FDR DR+/+HCD vs DR+/+ ND	DR+/+B/S vs DR+/+ND Log2 ratio >0.5 n=350	Log2 ratio DR+/+ B/S vs DR+/+ ND	Fold-Change DR+/+B/S vs DR+/+ ND	FDR DR+/+B/S vs DR+/+ ND	F+/+ vs DR+/+ND Log2 ratio >0.5 n=1209	Log2 ratio F+/+ vs DR+/+ ND	Fold-Change F+/+ vs DR+/+ ND	FDR F+/+ vs DR+/+ ND
1368188_at	Hpd	4-hydroxyphenylpyruvate dioxygenase	uniq_BSvsDR_106	0	3.54	3.18	3.00	3.48	0	-0.356	-1.280	0.343	1	-0.540	-1.454	0.028	0	-0.056	-1.040	1.124
1368226_at	Dnph1	2'-deoxynucleoside 5'-phosphate N-hydrolase 1	uniq_BSvsDR_106	0	6.94	7.43	7.51	7.31	0	0.492	1.407	0.193	1	0.572	1.487	0.077	0	0.374	1.296	0.542
1368262_at	Phlpp1	PH domain and leucine rich repeat protein phospha	uniq_BSvsDR_106	0	5.94	6.48	6.50	6.12	0	0.544	1.458	0.171	1	0.557	1.471	0.090	0	0.183	1.136	0.920
1368374_a_at	Ggt1	gamma-glutamyltransferase 1	uniq_BSvsDR_106	0	6.06	6.04	5.53	6.57	0	-0.027	-1.019	1.144	1	-0.536	-1.450	0.040	0	0.506	1.420	0.312
1368821_at	Fstl1	follistatin-like 1	uniq_BSvsDR_106	0	6.93	6.55	6.40	6.43	0	-0.379	-1.300	0.285	1	-0.532	-1.446	0.053	0	-0.500	-1.415	0.118
1369075_a_at	Gmeb2	glucocorticoid modulatory element binding protein	uniq_BSvsDR_106	0	4.22	3.94	3.65	4.08	0	-0.272	-1.207	0.567	1	-0.569	-1.484	0.024	0	-0.140	-1.102	0.990
1369366_at	Cntn5	contactin 5	uniq_BSvsDR_106	0	5.21	4.75	4.69	4.90	0	-0.466	-1.381	0.119	1	-0.522	-1.436	0.054	0	-0.316	-1.245	0.417
1369415_at	Bhlhe40	basic helix-loop-helix family, member e40	uniq_BSvsDR_106	0	5.37	5.79	4.84	5.66	0	0.425	1.343	0.326	1	-0.529	-1.443	0.061	0	0.296	1.228	0.670
1369736_at	Emp1	epithelial membrane protein 1	uniq_BSvsDR_106	0	5.98	5.91	5.32	5.91	0	-0.062	-1.044	0.958	1	-0.653	-1.572	0.010	0	-0.064	-1.045	1.134
1369928_at	Acta1	actin, alpha 1, skeletal muscle	uniq_BSvsDR_106	0	6.55	6.99	7.12	6.76	0	0.443	1.359	0.292	1	0.573	1.488	0.080	0	0.213	1.159	0.855
1369958_at	Rhob	ras homolog family member B	uniq_BSvsDR_106	0	10.05	10.14	9.51	9.98	0	0.089	1.063	1.044	1	-0.541	-1.455	0.047	0	-0.077	-1.055	1.093
1370009_at	Apoc3	apolipoprotein C-III	uniq_BSvsDR_106	0	4.95	4.69	4.41	5.02	0	-0.256	-1.194	0.642	1	-0.540	-1.454	0.032	0	0.066	1.047	1.034
1370034_at	Cdc25b	cell division cycle 25B	uniq_BSvsDR_106	0	5.75	6.02	6.31	6.19	0	0.271	1.206	0.624	1	0.560	1.475	0.092	0	0.446	1.363	0.401
1370131_at	Cav1	caveolin 1, caveolae protein	uniq_BSvsDR_106	0	6.54	6.59	5.87	6.38	0	0.048	1.034	0.944	1	-0.669	-1.590	0.015	0	-0.158	-1.116	0.831
1370540_at	Nr1d2	nuclear receptor subfamily 1, group D, member 2	uniq_BSvsDR_106	0	5.49	5.77	4.85	5.78	0	0.285	1.219	0.563	1	-0.636	-1.554	0.024	0	0.298	1.229	0.696
1370789_a_at	Prlr	prolactin receptor	uniq_BSvsDR_106	0	7.51	7.35	6.94	7.49	0	-0.158	-1.116	0.637	1	-0.571	-1.485	0.047	0	-0.015	-1.011	1.120
1372649_at	Hspb7	heat shock protein family, member 7 (cardiovascula	uniq_BSvsDR_106	0	5.21	5.00	4.69	5.31	0	-0.214	-1.160	0.760	1	-0.527	-1.441	0.035	0	0.098	1.071	1.018
1372963_at	---	---	uniq_BSvsDR_106	0	3.87	4.23	4.52	4.32	0	0.364	1.287	0.426	1	0.645	1.564	0.043	0	0.453	1.369	0.421
1373267_at	Sh3yl1	SH3 domain containing, Ysc84-like 1 (S. cerevisiae)	uniq_BSvsDR_106	0	6.09	6.60	6.71	6.00	0	0.510	1.424	0.192	1	0.618	1.535	0.054	0	-0.088	-1.063	1.092
1373436_at	RGD1306739	similar to RIKEN cDNA 1700040L02	uniq_BSvsDR_106	0	7.02	7.55	7.58	6.79	0	0.533	1.447	0.175	1	0.557	1.472	0.090	0	-0.228	-1.171	0.717
1373767_at	Zfand2a	zinc finger, AN1-type domain 2A	uniq_BSvsDR_106	0	7.45	7.38	6.90	7.14	0	-0.076	-1.054	1.034	1	-0.551	-1.466	0.030	0	-0.312	-1.241	0.451
1374875_at	---	---	uniq_BSvsDR_106	0	4.62	5.08	5.27	5.04	0	0.457	1.373	0.286	1	0.650	1.569	0.037	0	0.418	1.336	0.472
1375529_at	Cbr4	carbonyl reductase 4	uniq_BSvsDR_106	0	6.95	7.39	7.50	6.81	0	0.444	1.361	0.275	1	0.555	1.469	0.090	0	-0.131	-1.095	1.038
1375617_at	---	---	uniq_BSvsDR_106	0	4.57	4.99	5.12	4.29	0	0.427	1.344	0.312	1	0.555	1.469	0.083	0	-0.278	-1.213	0.547
1376128_at	Ntn1	netrin 1	uniq_BSvsDR_106	0	4.62	4.13	4.03	4.29	0	-0.485	-1.400	0.106	1	-0.591	-1.506	0.016	0	-0.334	-1.261	0.377
1376137_at	Plekhb2	pleckstrin homology domain containing, family B (ev	uniq_BSvsDR_106	0	5.31	5.59	4.73	5.50	0	0.276	1.211	0.593	1	-0.576	-1.490	0.029	0	0.194	1.144	0.880
1376454_at	Nek9	NIMA-related kinase 9	uniq_BSvsDR_106	0	5.75	5.75	5.19	6.08	0	0.003	1.002	1.167	1	-0.556	-1.470	0.028	0	0.327	1.255	0.641
1376719_at	Tmem38b	transmembrane protein 38B	uniq_BSvsDR_106	0	4.61	5.23	5.17	5.05	0	0.624	1.541	0.103	1	0.564	1.479	0.083	0	0.437	1.354	0.429
1377034_at	Serpinb1a	serine (or cysteine) proteinase inhibitor, clade B, me	uniq_BSvsDR_106	0	6.45	6.70	5.82	6.77	0	0.250	1.189	0.627	1	-0.627	-1.544	0.010	0	0.317	1.246	0.662
1377160_at	---	---	uniq_BSvsDR_106	0	4.83	5.21	5.39	4.82	0	0.372	1.294	0.410	1	0.556	1.470	0.091	0	-0.015	-1.010	1.145
1377450_s_at	Pvrl4	poliovirus receptor-related 4	uniq_BSvsDR_106	0	4.32	3.91	3.78	4.22	0	-0.412	-1.331	0.227	1	-0.538	-1.452	0.034	0	-0.100	-1.072	0.974
1377596_a_at	Med30	mediator complex subunit 30	uniq_BSvsDR_106	0	6.42	6.83	7.05	7.11	0	0.408	1.327	0.363	1	0.628	1.545	0.047	0	0.687	1.610	0.124
1377729_at	---	---	uniq_BSvsDR_106	0	6.04	6.64	6.71	6.52	0	0.597	1.512	0.108	1	0.669	1.590	0.027	0	0.478	1.393	0.372
1377918_at	Stoml1	stomatin (EPB72)-like 1	uniq_BSvsDR_106	0	5.63	5.82	6.21	5.78	0	0.187	1.139	0.813	1	0.576	1.491	0.074	0	0.147	1.107	0.959
1377963_at	---	---	uniq_BSvsDR_106	0	6.66	6.87	7.22	6.86	0	0.213	1.159	0.734	1	0.559	1.473	0.083	0	0.199	1.148	0.885
1378083_at	---	---	uniq_BSvsDR_106	0	5.32	5.11	4.80	5.06	0	-0.213	-1.159	0.724	1	-0.526	-1.440	0.046	0	-0.260	-1.197	0.606
1378283_at	LOC10091298	uncharacterized LOC100912988	uniq_BSvsDR_106	0	4.35	4.54	4.93	4.32	0	0.185	1.137	0.728	1	0.576	1.490	0.081	0	-0.039	-1.027	1.092
1379025_at	Ubr4	ubiquitin protein ligase E3 component n-recognin 4	uniq_BSvsDR_106	0	6.42	6.48	5.89	6.69	0	0.058	1.041	1.124	1	-0.528	-1.442	0.056	0	0.267	1.203	0.766
1379301_at	---	---	uniq_BSvsDR_106	0	5.37	5.88	5.95	5.75	0	0.514	1.428	0.181	1	0.583	1.498	0.076	0	0.385	1.306	0.509
1379511_at	---	---	uniq_BSvsDR_106	0	4.42	4.96	5.19	4.82	0	0.542	1.456	0.184	1	0.773	1.708	0.011	0	0.404	1.323	0.485
1379754_at	Stau2	staufen, RNA binding protein, homolog 2 (Drosophil	uniq_BSvsDR_106	0	8.02	8.50	8.57	7.93	0	0.476	1.391	0.234	1	0.554	1.468	0.090	0	-0.086	-1.062	1.121
1380582_at	Csf1	colony stimulating factor 1 (macrophage)	uniq_BSvsDR_106	0	8.01	7.55	7.42	7.76	0	-0.456	-1.372	0.148	1	-0.587	-1.503	0.014	0	-0.246	-1.186	0.630
1381036_at	---	---	uniq_BSvsDR_106	0	5.87	6.46	6.46	6.39	0	0.590	1.505	0.132	1	0.595	1.510	0.070	0	0.523	1.437	0.318
1381350_at	---	---	uniq_BSvsDR_106	0	3.59	3.34	3.07	3.17	0	-0.256	-1.194	0.580	1	-0.521	-1.435	0.051	0	-0.421	-1.339	0.199
1381555_at	---	---	uniq_BSvsDR_106	0	5.73	6.14	6.34	5.75	0	0.403	1.322	0.355	1	0.609	1.525	0.057	0	0.017	1.012	1.107
1381919_at	Hps6	Hermansky-Pudlak syndrome 6	uniq_BSvsDR_106	0	6.01	6.44	6.62	6.22	0	0.427	1.345	0.298	1	0.605	1.521	0.065	0	0.209	1.155	0.850
1381940_at	---	---	uniq_BSvsDR_106	0	5.94	6.53	6.58	6.22	0	0.590	1.505	0.122	1	0.641	1.560	0.046	0	0.284	1.217	0.742
1382051_at	Hscb	HscB iron-sulfur cluster co-chaperone homolog (E. c	uniq_BSvsDR_106	0	6.21	6.78	6.87	6.13	0	0.567	1.481	0.163	1	0.661	1.581	0.035	0	-0.083	-1.060	1.068
1382142_at	---	---	uniq_BSvsDR_106	0	5.55	5.96	6.34	5.83	0	0.409	1.328	0.294	1	0.790	1.729	0.011	0	0.283	1.217	0.684
1382458_at	---	---	uniq_BSvsDR_106	0	5.57	5.72	6.13	5.33	0	0.150	1.110	0.799	1	0.565	1.480	0.079	0	-0.237	-1.179	0.680
1382475_at	---	---	uniq_BSvsDR_106	0	5.65	6.25	6.22	5.80	0	0.597	1.512	0.109	1	0.567	1.481	0.083	0	0.151	1.110	0.943
1382485_at	---	---	uniq_BSvsDR_106	0	4.09	4.44	4.68	4.53	0	0.351	1.275	0.398	1	0.586	1.501	0.074	0	0.440	1.357	0.430
1383021_at	Galnt13	UDP-N-acetyl-alpha-D-galactosamine:polypeptide N	uniq_BSvsDR_106	0	5.60	6.13	6.17	5.69	0	0.531	1.445	0.165	1	0.571	1.486	0.083	0	0.087	1.062	1.053
1383148_at	---	---	uniq_BSvsDR_106	0	6.18	6.64	6.74	6.17	0	0.463	1.379	0.256	1	0.559	1.473	0.083	0	-0.011	-1.008	1.161
1383486_at	---	---	uniq_BSvsDR_106	0	4.13	4.07	3.62	4.15	0	-0.062	-1.044	1.008	1	-0.515	-1.429	0.041	0	0.018	1.012	1.069
1383896_at	Zfp639	zinc finger protein 639	uniq_BSvsDR_106	0	5.76	6.35	6.39	6.19	0	0.592	1.507	0.131	1	0.626	1.543	0.055	0	0.429	1.346	0.453
1384120_at	---	---	uniq_BSvsDR_106	0	5.59	6.00	6.18	5.56	0	0.412	1.331	0.342	1	0.589	1.504	0.072	0	-0.031	-1.022	1.084
1384124_at	---	---	uniq_BSvsDR_106	0	7.59	8.18	8.15	8.25	0	0.588	1.503	0.132	1	0.558	1.473	0.092	0	0.656	1.575	0.144
1384225_at	---	---	uniq_BSvsDR_106	0	5.42	5.67	6.01	5.65	0	0.258	1.196	0.634	1	0.597	1.513	0.066	0	0.238	1.179	0.821
1384571_at	---	---	uniq_BSvsDR_106	0	6.20	6.57	6.79	6.35	0	0.367	1.290	0.377	1	0.584	1.499	0.072	0	0.143	1.104	0.999
1385641_at	---	---	uniq_BSvsDR_106	0	7.37	7.10	6.72	7.05	0	-0.278	-1.213	0.461	1	-0.657	-1.577	0.009	0	-0.325	-1.252	0.416
1385719_at	---	---	uniq_BSvsDR_106	0	6.84	7.33	7.42	6.99	0	0.483	1.397	0.244	1	0.575	1.490	0.080	0	0.141	1.102	0.996
1385731_at	---	---	uniq_BSvsDR_106	0	7.82	8.37	8.37	8.51	0	0.555	1.470	0.141	1	0.551	1.465	0.094	0	0.696	1.620	0.121
1385830_at	RGD1561507	similar to hypothetical protein FLJ31606	uniq_BSvsDR_106	0	5.31	5.76	5.95	5.40	0	0.453	1.369	0.299	1	0.648	1.567	0.036	0	0.093	1.067	1.046
1385954_at	LOC1001253(	hypothetical protein LOC100125362	uniq_BSvsDR_106	0	3.89	4.45	4.48	3.83	0	0.564	1.478	0.141	1	0.592	1.507	0.071	0	-0.056	-1.040	1.138
1386069_at	Sp2	Sp2 transcription factor	uniq_BSvsDR_106	0	4.14	4.51	4.75	4.62	0	0.373	1.295	0.366	1	0.609	1.525	0.057	0	0.483	1.398	0.359
1386149_at	---	---	uniq_BSvsDR_106	0	6.50	6.73	7.05	6.91	0	0.231	1.174	0.736	1	0.548	1.462	0.091	0	0.411	1.329	0.474
1386948_at	Nes	nestin	uniq_BSvsDR_106	0	6.30	5.99	5.78	6.25	0	-0.313	-1.242	0.505	1	-0.514	-1.428	0.046	0	-0.051	-1.036	1.145
1387630_at	Elovl5	ELOVL fatty acid elongase 5	uniq_BSvsDR_106	0	5.99	6.11	5.47	5.83	0	0.121	1.088	0.957	1	-0.526	-1.440	0.069	0	-0.164	-1.120	0.880
1387929_at	Pmf31	PMF32 protein	uniq_BSvsDR_106	0	5.91	6.14	6.48	5.98	0	0.234	1.176	0.702	1	0.569	1.484	0.079	0	0.070	1.049	1.075
1388295_s_at	Frs3	fibroblast growth factor receptor substrate 3	uniq_BSvsDR_106	0	5.93	6.29	6.61	6.00	0	0.366	1.289	0.391	1	0.686	1.609	0.020	0	0.074	1.053	1.066
1388395_at	G0s2	G0/G1switch 2	uniq_BSvsDR_106	0	5.94	6.22	6.48	6.14	0	0.279	1.213	0.559	1	0.546	1.460	0.094	0	0.203	1.151	0.904
1388986_at	Klf6	Kruppel-like factor 6	uniq_BSvsDR_106	0	8.04	8.26	7.50	8.12	0	0.216	1.162	0.724	1	-0.542	-1.456	0.051	0	0.084	1.060	1.041
1389423_at	Ddr2	discoidin domain receptor tyrosine kinase 2	uniq_BSvsDR_106	0	5.94	5.57	5.37	5.59	0	-0.377	-1.298	0.240	1	-0.573	-1.488	0.024	0	-0.352	-1.277	0.350
1389720_at	---	---	uniq_BSvsDR_106	0	4.31	3.93	3.80	4.01	0	-0.375	-1.297	0.298	1	-0.511	-1.425	0.064	0	-0.301	-1.232	0.491
1390077_at	Tram1	translocation associated membrane protein 1	uniq_BSvsDR_106	0	4.02	4.13	4.71	4.43	0	0.113	1.081	0.923	1	0.695	1.619	0.020	0	0.407	1.326	0.473
1390581_at	LOC1009116!	uncharacterized LOC100911651	uniq_BSvsDR_106	0	5.16	5.50	5.83	5.33	0	0.336	1.262	0.398	1	0.665	1.586	0.034	0	0.172	1.127	0.867
1391374_at	Tacc3	transforming, acidic coiled-coil containing protein 3	uniq_BSvsDR_106	0	6.03	5.56	5.49	6.27	0	-0.469	-1.384	0.135	1	-0.539	-1.453	0.030	0	0.242	1.182	0.799
1391674_at	---	---	uniq_BSvsDR_106	0	5.10	4.75	4.24	4.68	0	-0.352	-1.276	0.307	1	-0.857	-1.811	0.000	0	-0.414	-1.332	0.230
1391720_at	---	---	uniq_BSvsDR_106	0	4.32	3.95	3.77	4.26	0	-0.370	-1.293	0.269	1	-0.548	-1.462	0.033	0	-0.058	-1.041	1.068
1391826_at	Tp53inp1	tumor protein p53 inducible nuclear protein 1	uniq_BSvsDR_106	0	4.35	4.20	3.82	4.30	0	-0.156	-1.114	0.929	1	-0.530	-1.444	0.034	0	-0.054	-1.038	1.135
1392109_at	---	---	uniq_BSvsDR_106	0	5.48	6.09	6.05	6.12	0	0.608	1.524	0.104	1	0.571	1.485	0.090	0	0.640	1.558	0.153
1392170_at	Rwdd3	RWD domain containing 3	uniq_BSvsDR_106	0	6.51	6.99	7.21	6.37	0	0.489	1.403	0.204	1	0.701	1.626	0.020	0	-0.140	-1.102	0.956
1392883_at	Limch1	LIM and calponin homology domains 1	uniq_BSvsDR_106	0	6.89	6.56	6.34	7.08	0	-0.326	-1.254	0.388	1	-0.550	-1.464	0.046	0	0.193	1.143	0.886
1393026_at	Rnase1l2	ribonuclease, RNase A family, 1-like 2 (pancreatic)	uniq_BSvsDR_106	0	12.37	12.93	11.82	12.76	0	0.561	1.476	0.172	1	-0.553	-1.468	0.046	0	0.389	1.309	0.522
1393063_at	Eif5a2	eukaryotic translation initiation factor 5A2	uniq_BSvsDR_106	0	6.69	7.22	7.41	7.16	0	0.532	1.446	0.172	1	0.720	1.648	0.016	0	0.475	1.390	0.370
1393281_at	Cav1	caveolin 1, caveolae protein	uniq_BSvsDR_106	0	7.90	7.77	7.26	7.59	0	-0.127	-1.092	0.753	1	-0.637	-1.555	0.015	0	-0.313	-1.242	0.461
1393386_at	Fam183b	family with sequence similarity 183, member B	uniq_BSvsDR_106	0	5.73	5.92	6.32	5.94	0	0.193	1.143	0.826	1	0.587	1.502	0.075	0	0.212	1.158	0.886
1393397_at	Cpa2	carboxypeptidase A2 (pancreatic)	uniq_BSvsDR_106	0	11.82	12.33	11.28	12.50	0	0.510	1.424	0.199	1	-0.536	-1.450	0.049	0	0.688	1.611	0.131
1393701_at	Dbr1	debranching enzyme homolog 1 (S. cerevisiae)	uniq_BSvsDR_106	0	5.80	6.29	6.38	5.89	0	0.492	1.407	0.220	1	0.585	1.500	0.073	0	0.089	1.063	1.021
1393800_at	Armcx5	armadillo repeat containing, X-linked 5	uniq_BSvsDR_106	0	7.23	7.71	7.82	7.77	0	0.487	1.401	0.254	1	0.592	1.508	0.065	0	0.540	1.454	0.252
1394107_at	Tbl1xr1	transducin (beta)-like 1 X-linked receptor 1	uniq_BSvsDR_106	0	6.03	6.58	6.59	6.58	0	0.552	1.466	0.159	1	0.556	1.470	0.096	0	0.555	1.469	0.266
1394435_at	Vangl1	VANGL planar cell polarity protein 1	uniq_BSvsDR_106	0	6.90	7.27	7.49	7.00	0	0.364	1.287	0.422	1	0.586	1.501	0.073	0	0.100	1.071	1.058
1395057_at	---	---	uniq_BSvsDR_106	0	5.24	4.79	4.74	4.99	0	-0.457	-1.373	0.127	1	-0.501	-1.415	0.057	0	-0.250	-1.189	0.635
1395117_at	Cdan1	codanin 1	uniq_BSvsDR_106	0	3.94	3.69	3.43	3.63	0	-0.252	-1.191	0.473	1	-0.515	-1.429	0.045	0	-0.317	-1.245	0.435
1395654_at	Ccdc25	coiled-coil domain containing 25	uniq_BSvsDR_106	0	4.96	5.28	5.65	5.11	0	0.327	1.254	0.465	1	0.692	1.615	0.027	0	0.153	1.112	0.990
1396276_at	---	---	uniq_BSvsDR_106	0	5.01	5.17	5.59	4.92	0	0.153	1.112	0.947	1	0.580	1.495	0.076	0	-0.095	-1.068	1.077
1396545_at	Crem	cAMP responsive element modulator	uniq_BSvsDR_106	0	6.14	6.44	6.77	6.47	0	0.293	1.225	0.562	1	0.631	1.549	0.053	0	0.327	1.255	0.649
1397743_at	---	---	uniq_BSvsDR_106	0	4.52	4.67	5.10	4.46	0	0.149	1.109	0.923	1	0.579	1.494	0.077	0	-0.055	-1.039	1.048
1398056_at	---	---	uniq_BSvsDR_106	0	4.50	4.99	5.12	4.62	0	0.490	1.405	0.253	1	0.616	1.532	0.065	0	0.123	1.089	1.010
1398107_at	Ggct	gamma-glutamyl cyclotransferase	uniq_BSvsDR_106	0	6.61	6.23	6.04	6.38	0	-0.373	-1.295	0.324	1	-0.564	-1.478	0.035	0	-0.229	-1.172	0.707
1398983_at	LOC1009100(	39S ribosomal protein L30, mitochondrial-like /// mi	uniq_BSvsDR_106	0	7.47	8.04	8.02	7.65	0	0.563	1.477	0.132	1	0.548	1.462	0.089	0	0.179	1.132	0.948
1367633_at	Glul	glutamate-ammonia ligase	uniq_HCDvsDR_322	0	7.95	8.80	7.82	8.66	1	0.846	1.798	0.017	0	-0.138	-1.101	0.789	0	0.704	1.628	0.116
1367647_at	Serpina1	serpin peptidase inhibitor, clade A (alpha-1 antiprot	uniq_HCDvsDR_322	0	7.30	8.00	7.56	7.51	1	0.697	1.621	0.050	0	0.255	1.194	0.605	0	0.207	1.155	0.901
1367648_at	Igfbp2	insulin-like growth factor binding protein 2	uniq_HCDvsDR_322	0	6.18	7.60	6.45	6.33	1	1.421	2.678	0.000	0	0.271	1.206	0.558	0	0.157	1.115	0.986
1367689_a_at	Cd36	CD36 molecule (thrombospondin receptor)	uniq_HCDvsDR_322	0	7.67	8.54	7.90	7.99	1	0.868	1.825	0.014	0	0.225	1.169	0.587	0	0.322	1.250	0.634
1368079_at	Pdk1	pyruvate dehydrogenase kinase, isozyme 1	uniq_HCDvsDR_322	0	6.07	6.75	6.53	6.41	1	0.680	1.602	0.069	0	0.460	1.375	0.217	0	0.337	1.263	0.609

Affymetrix Probeset ID	Gene Symbol	Gene Title	Figure 7 A Venn n=2289	Present in any 2 analyses n=423 1=yes 0=no	Mean log2 intensity DR+/+ ND	Mean log2 intensity DR+/+ HCD	Mean log2 intensity DR+/+ B/S	Mean log2 intensity F+/+ day 40 ND	DR+/+HCD vs DR+/+ND FDR<10% Log2 ratio >0.5 n=636	Log2 ratio DR+/+HCD vs DR+/+ ND	Fold-Change DR+/+H CD vs DR+/+ ND	FDR DR+/+H CD vs DR+/+ ND	DR+/+B/S vs DR+/+ND Log2 ratio >0.5 n=350	Log2 ratio DR+/+ B/S vs ND	Fold-Change DR+/+B /S vs DR+/+ ND	FDR DR+/+B /S vs DR+/+ ND	F+/+ vs DR+/+N D Log2 ratio >0.5 n=1209	Log2 ratio F+/+ vs DR+/+ ND	Fold-Change F+/+ vs DR+/+ ND	FDR F+/+ vs DR+/+ ND
1368121_at	Akr7a3	aldo-keto reductase family 7, member A3 (aflatoxin	uniq_HCDvsDR_322	0	4.23	3.72	3.92	4.20	1	-0.512	-1.426	0.080	0	-0.311	-1.240	0.412	0	-0.031	-1.022	1.149
1368240_a_at	Prkcb	protein kinase C, beta	uniq_HCDvsDR_322	0	4.87	5.78	4.94	5.58	1	0.910	1.879	0.010	0	0.069	1.049	1.015	0	0.709	1.635	0.106
1368332_at	Gbp2	guanylate binding protein 2, interferon-inducible	uniq_HCDvsDR_322	0	4.40	3.70	4.21	4.05	1	-0.701	-1.625	0.008	0	-0.188	-1.139	0.693	0	-0.351	-1.276	0.365
1368339_at	S100g	S100 calcium binding protein G	uniq_HCDvsDR_322	0	3.47	2.90	3.27	3.27	1	-0.578	-1.493	0.027	0	-0.207	-1.154	0.632	0	-0.203	-1.151	0.723
1368369_at	Pnoc	prepronociceptin	uniq_HCDvsDR_322	0	4.66	4.08	4.46	4.51	1	-0.587	-1.502	0.026	0	-0.209	-1.156	0.730	0	-0.152	-1.111	0.968
1368544_a_at	Nol3	nucleolar protein 3 (apoptosis repressor with CARD	uniq_HCDvsDR_322	0	4.75	4.25	4.48	4.83	1	-0.500	-1.415	0.095	0	-0.273	-1.209	0.548	0	0.076	1.054	1.041
1368671_at	Srpx	sushi-repeat-containing protein, X-linked	uniq_HCDvsDR_322	0	6.73	6.23	6.36	6.46	1	-0.500	-1.415	0.088	0	-0.375	-1.297	0.279	0	-0.271	-1.207	0.561
1368782_at	Sstr2	somatostatin receptor 2	uniq_HCDvsDR_322	0	6.40	7.13	6.78	6.24	1	0.736	1.666	0.039	0	0.389	1.309	0.311	0	-0.152	-1.111	0.924
1368816_at	Mpz	myelin protein zero	uniq_HCDvsDR_322	0	4.55	3.99	4.38	4.30	1	-0.555	-1.469	0.040	0	-0.170	-1.125	0.798	0	-0.248	-1.187	0.649
1368883_at	Nov	nephroblastoma overexpressed	uniq_HCDvsDR_322	0	6.82	7.58	6.75	6.48	1	0.762	1.696	0.033	0	-0.074	-1.053	1.018	0	-0.345	-1.270	0.356
1368885_at	---	---	uniq_HCDvsDR_322	0	6.55	6.03	6.31	6.10	1	-0.519	-1.433	0.074	0	-0.242	-1.182	0.541	0	-0.449	-1.365	0.177
1368924_at	Ghr	growth hormone receptor	uniq_HCDvsDR_322	0	7.34	8.02	7.19	7.79	1	0.678	1.600	0.082	0	-0.151	-1.110	0.725	0	0.457	1.373	0.397
1368932_at	Rock1	Rho-associated coiled-coil containing protein kinase	uniq_HCDvsDR_322	0	5.33	6.05	5.40	5.73	1	0.719	1.647	0.043	0	0.071	1.050	1.001	0	0.398	1.317	0.491
1368964_at	Lrrn3	leucine rich repeat neuronal 3	uniq_HCDvsDR_322	0	6.81	7.80	7.10	6.84	1	0.990	1.986	0.005	0	0.291	1.223	0.518	0	0.037	1.026	1.108
1369001_at	Chrna3	cholinergic receptor, nicotinic, alpha 3 (neuronal)	uniq_HCDvsDR_322	0	3.65	3.12	3.44	3.43	1	-0.538	-1.452	0.057	0	-0.217	-1.162	0.680	0	-0.222	-1.166	0.761
1369041_at	Nlgn1	neuroligin 1	uniq_HCDvsDR_322	0	5.11	5.99	5.33	5.17	1	0.879	1.840	0.011	0	0.219	1.164	0.661	0	0.061	1.043	1.076
1369047_at	Sult1d1	sulfotransferase family 1D, member 1	uniq_HCDvsDR_322	0	3.74	4.44	4.12	3.85	1	0.697	1.621	0.049	0	0.376	1.297	0.342	0	0.103	1.074	0.964
1369074_at	Slc38a4	solute carrier family 38, member 4	uniq_HCDvsDR_322	0	10.05	10.81	10.26	10.58	1	0.758	1.691	0.034	0	0.213	1.159	0.701	0	0.533	1.447	0.275
1369150_at	Pdk4	pyruvate dehydrogenase kinase, isozyme 4	uniq_HCDvsDR_322	0	6.51	7.18	6.58	6.64	1	0.672	1.594	0.084	0	0.069	1.049	0.984	0	0.129	1.094	0.991
1369152_at	Ppp3r1	protein phosphatase 3, regulatory subunit B, alpha	uniq_HCDvsDR_322	0	5.66	6.49	5.58	6.03	1	0.824	1.771	0.020	0	-0.082	-1.059	0.842	0	0.364	1.287	0.569
1369227_at	Chm	choroideremia (Rab escort protein 1)	uniq_HCDvsDR_322	0	5.67	6.31	5.53	6.37	1	0.641	1.560	0.096	0	-0.138	-1.101	0.795	0	0.695	1.619	0.121
1369275_s_at	Cyp2a1 /// Cy	cytochrome P450, family 2, subfamily a, polypeptide	uniq_HCDvsDR_322	0	5.43	4.93	5.25	5.11	1	-0.502	-1.416	0.084	0	-0.182	-1.134	0.797	0	-0.321	-1.249	0.411
1369284_at	Barhl2	BarH-like homeobox 2	uniq_HCDvsDR_322	0	4.50	3.94	4.21	4.40	1	-0.566	-1.480	0.034	0	-0.297	-1.229	0.478	0	-0.106	-1.077	0.996
1369565_at	Il12b	interleukin 12B	uniq_HCDvsDR_322	0	4.58	4.07	4.31	4.63	1	-0.515	-1.429	0.072	0	-0.278	-1.212	0.508	0	0.050	1.036	1.094
1369616_at	Fgf22	fibroblast growth factor 22	uniq_HCDvsDR_322	0	4.89	4.36	4.66	4.61	1	-0.532	-1.446	0.067	0	-0.232	-1.175	0.592	0	-0.288	-1.221	0.457
1369633_at	Cxcl12	chemokine (C-X-C motif) ligand 12	uniq_HCDvsDR_322	0	7.68	7.17	7.32	7.20	1	-0.514	-1.428	0.077	0	-0.361	-1.284	0.315	0	-0.478	-1.393	0.124
1369765_at	Ascl1	achaete-scute complex homolog 1 (Drosophila)	uniq_HCDvsDR_322	0	5.76	6.41	5.99	5.46	1	0.657	1.576	0.082	0	0.238	1.180	0.591	0	-0.295	-1.227	0.519
1369787_at	Cckar	cholecystokinin A receptor	uniq_HCDvsDR_322	0	10.55	9.50	10.16	10.11	1	-1.042	-2.060	0.000	0	-0.389	-1.309	0.246	0	-0.441	-1.358	0.171
1369825_at	Mmp2	matrix metallopeptidase 2	uniq_HCDvsDR_322	0	5.06	4.50	4.66	5.01	1	-0.564	-1.478	0.035	0	-0.397	-1.317	0.220	0	-0.054	-1.038	1.048
1369963_at	Pafah1b3	platelet-activating factor acetylhydrolase 1b, catalyt	uniq_HCDvsDR_322	0	6.42	7.14	6.86	6.90	1	0.728	1.657	0.041	0	0.446	1.362	0.233	0	0.483	1.398	0.349
1370012_at	Ptgis	prostaglandin I2 (prostacyclin) synthase	uniq_HCDvsDR_322	0	6.30	5.79	5.92	5.89	1	-0.506	-1.420	0.087	0	-0.374	-1.296	0.285	0	-0.407	-1.326	0.234
1370065_at	Hpx	hemopexin	uniq_HCDvsDR_322	0	4.07	3.54	3.91	4.18	1	-0.532	-1.446	0.052	0	-0.159	-1.117	0.833	0	0.107	1.077	1.037
1370084_at	Cpb1	carboxypeptidase B1 (tissue)	uniq_HCDvsDR_322	0	12.40	13.41	11.95	13.06	1	1.014	2.020	0.005	0	-0.449	-1.365	0.133	0	0.667	1.588	0.141
1370141_at	LOC689551 /,	similar to myeloid cell leukemia sequence 1 /// myel	uniq_HCDvsDR_322	0	7.21	8.12	7.24	7.78	1	0.913	1.883	0.012	0	0.035	1.025	0.923	0	0.579	1.494	0.239
1370201_at	Calb1	calbindin 1	uniq_HCDvsDR_322	0	7.82	8.52	7.85	7.40	1	0.693	1.616	0.050	0	0.025	1.018	1.138	0	-0.422	-1.339	0.195
1370268_at	Kcna5	potassium voltage-gated channel, shaker-related su	uniq_HCDvsDR_322	0	6.81	7.47	7.18	7.26	1	0.660	1.580	0.072	0	0.367	1.290	0.365	0	0.444	1.361	0.409
1370269_at	Cyp1a1	cytochrome P450, family 1, subfamily a, polypeptide	uniq_HCDvsDR_322	0	5.24	4.64	4.79	4.83	1	-0.602	-1.518	0.018	0	-0.454	-1.370	0.108	0	-0.407	-1.326	0.232
1370271_a_at	Grpca /// Grp	glutamine/glutamic acid-rich protein A /// glutamine	uniq_HCDvsDR_322	0	7.08	6.43	6.63	6.77	1	-0.648	-1.567	0.008	0	-0.450	-1.366	0.119	0	-0.311	-1.240	0.448
1370491_a_at	Hdc	histidine decarboxylase	uniq_HCDvsDR_322	0	6.40	7.25	6.43	6.61	1	0.851	1.804	0.016	0	0.031	1.022	1.050	0	0.217	1.162	0.821
1370555_at	Nalcn	sodium leak channel, non-selective	uniq_HCDvsDR_322	0	8.03	8.64	8.00	7.93	1	0.610	1.526	0.097	0	-0.033	-1.023	1.096	0	-0.100	-1.072	1.093
1370585_a_at	Prkcb	protein kinase C, beta	uniq_HCDvsDR_322	0	7.91	8.58	8.32	8.50	1	0.665	1.586	0.069	0	0.406	1.325	0.296	0	0.586	1.501	0.215
1370781_a_at	Kcnip1	Kv channel-interacting protein 1	uniq_HCDvsDR_322	0	5.61	6.60	6.15	5.48	1	0.984	1.978	0.006	0	0.539	1.453	0.112	0	-0.134	-1.097	0.971
1370972_x_at	RT1-CE5	RT1 class I, locus CE5	uniq_HCDvsDR_322	0	6.92	6.39	6.59	6.43	1	-0.532	-1.446	0.060	0	-0.322	-1.250	0.407	0	-0.492	-1.406	0.105
1371021_at	Arsb	arylsulfatase B	uniq_HCDvsDR_322	0	7.59	8.36	7.68	7.64	1	0.772	1.708	0.028	0	0.096	1.069	1.032	0	0.060	1.042	1.093
1371038_at	Cebpg	CCAAT/enhancer binding protein (C/EBP), gamma	uniq_HCDvsDR_322	0	5.88	6.53	5.85	6.32	1	0.654	1.574	0.078	0	-0.029	-1.020	1.045	0	0.445	1.361	0.426
1371060_at	Trim23	tripartite motif-containing 23	uniq_HCDvsDR_322	0	5.83	6.60	5.86	6.02	1	0.769	1.704	0.044	0	0.035	1.024	0.794	0	0.197	1.146	0.759
1371116_at	LOC10091019	mannan-binding lectin serine protease 2-like /// ma	uniq_HCDvsDR_322	0	5.27	4.76	4.92	5.03	1	-0.513	-1.427	0.081	0	-0.345	-1.271	0.344	0	-0.243	-1.184	0.678
1371119_at	LOC360231	MHC class I RT1.O type 149 processed pseudogene	uniq_HCDvsDR_322	0	7.03	6.49	6.78	6.67	1	-0.539	-1.453	0.054	0	-0.250	-1.189	0.600	0	-0.361	-1.284	0.316
1371332_at	---	---	uniq_HCDvsDR_322	0	10.63	11.27	10.98	10.65	1	0.641	1.560	0.079	0	0.348	1.273	0.393	0	0.021	1.014	1.111
1371349_at	Col6a1	collagen, type VI, alpha 1	uniq_HCDvsDR_322	0	8.27	7.74	7.89	7.83	1	-0.529	-1.443	0.062	0	-0.381	-1.303	0.258	0	-0.444	-1.360	0.173
1371574_at	Ghitm	growth hormone inducible transmembrane protein	uniq_HCDvsDR_322	0	9.59	10.20	9.88	9.99	1	0.614	1.531	0.099	0	0.288	1.221	0.524	0	0.403	1.322	0.479
1371735_at	---	---	uniq_HCDvsDR_322	0	7.86	8.57	8.31	8.26	1	0.711	1.637	0.045	0	0.455	1.371	0.216	0	0.404	1.324	0.481
1371854_at	LOC1009104(	uncharacterized LOC100910406	uniq_HCDvsDR_322	0	8.98	9.72	9.45	9.04	1	0.732	1.661	0.038	0	0.468	1.383	0.186	0	0.057	1.040	1.099
1371898_at	Kcnk5	potassium channel, subfamily K, member 5	uniq_HCDvsDR_322	0	4.82	4.29	4.59	4.35	1	-0.524	-1.438	0.070	0	-0.227	-1.171	0.610	0	-0.467	-1.382	0.137
1371913_at	Tgfbi	transforming growth factor, beta induced	uniq_HCDvsDR_322	0	7.99	7.38	7.59	7.84	1	-0.617	-1.533	0.018	0	-0.408	-1.327	0.199	0	-0.154	-1.112	0.964
1372110_at	---	---	uniq_HCDvsDR_322	0	9.32	8.78	9.14	9.37	1	-0.539	-1.453	0.058	0	-0.178	-1.132	0.784	0	0.053	1.037	1.103
1372535_at	Tceanc2	transcription elongation factor A (SII) N-terminal and	uniq_HCDvsDR_322	0	7.25	7.96	7.72	7.32	1	0.707	1.632	0.045	0	0.466	1.381	0.196	0	0.073	1.052	1.084
1372601_at	Atf5	activating transcription factor 5	uniq_HCDvsDR_322	0	10.08	10.70	10.58	10.25	1	0.618	1.535	0.095	0	0.499	1.413	0.150	0	0.169	1.125	0.962
1372626_at	Tpd52l1	tumor protein D52-like 1	uniq_HCDvsDR_322	0	7.51	6.93	7.28	7.98	1	-0.586	-1.501	0.029	0	-0.231	-1.174	0.636	0	0.463	1.378	0.388
1372631_at	Tk1	thymidine kinase 1, soluble	uniq_HCDvsDR_322	0	6.94	6.42	6.62	6.98	1	-0.512	-1.426	0.076	0	-0.315	-1.244	0.431	0	0.045	1.031	1.095
1372637_at	LOC683751	similar to trophinin isoform 1	uniq_HCDvsDR_322	0	6.47	7.18	6.71	6.83	1	0.713	1.639	0.044	0	0.247	1.187	0.601	0	0.365	1.288	0.559
1372653_at	Fkbp11	FK506 binding protein 11	uniq_HCDvsDR_322	0	9.10	8.49	8.76	9.07	1	-0.605	-1.521	0.023	0	-0.340	-1.266	0.359	0	-0.024	-1.017	1.163
1372665_at	Psat1	phosphoserine aminotransferase 1	uniq_HCDvsDR_322	0	7.21	7.84	7.64	7.66	1	0.629	1.547	0.085	0	0.429	1.346	0.255	0	0.449	1.365	0.395
1372790_at	Mdh1	malate dehydrogenase 1, NAD (soluble)	uniq_HCDvsDR_322	0	10.04	9.41	9.92	9.61	1	-0.628	-1.545	0.013	0	-0.119	-1.086	0.914	0	-0.430	-1.348	0.180
1372920_at	Prodh	proline dehydrogenase (oxidase) 1	uniq_HCDvsDR_322	0	6.86	6.29	6.56	6.35	1	-0.573	-1.488	0.031	0	-0.298	-1.229	0.463	0	-0.511	-1.425	0.117
1372966_at	Mfsd2a	major facilitator superfamily domain containing 2A	uniq_HCDvsDR_322	0	5.81	5.30	5.50	6.14	1	-0.507	-1.421	0.089	0	-0.307	-1.237	0.449	0	0.329	1.256	0.631
1373043_at	Sdf2l1	stromal cell-derived factor 2-like 1	uniq_HCDvsDR_322	0	9.22	8.53	8.82	9.10	1	-0.691	-1.614	0.004	0	-0.402	-1.321	0.221	0	-0.117	-1.085	1.072
1373089_at	Cdh3	cadherin 3	uniq_HCDvsDR_322	0	5.18	4.67	4.85	5.14	1	-0.510	-1.424	0.082	0	-0.327	-1.255	0.395	0	-0.040	-1.028	1.147
1373427_at	Rragd	Ras-related GTP binding D	uniq_HCDvsDR_322	0	7.13	7.82	7.51	7.41	1	0.697	1.622	0.050	0	0.379	1.300	0.345	0	0.286	1.220	0.738
1373700_at	---	---	uniq_HCDvsDR_322	0	6.70	6.13	6.39	6.48	1	-0.566	-1.480	0.037	0	-0.310	-1.240	0.395	0	-0.215	-1.161	0.726
1373759_at	Fosb	FBJ osteosarcoma oncogene B	uniq_HCDvsDR_322	0	9.80	10.67	9.59	10.52	1	0.865	1.821	0.020	0	-0.211	-1.158	0.488	0	0.718	1.645	0.140
1373774_at	Rit2	Ras-like without CAAX 2	uniq_HCDvsDR_322	0	7.23	6.58	7.02	7.73	1	-0.646	-1.565	0.009	0	-0.210	-1.157	0.660	0	0.499	1.413	0.323
1373777_at	---	---	uniq_HCDvsDR_322	0	8.84	9.47	9.26	9.17	1	0.628	1.545	0.095	0	0.413	1.331	0.281	0	0.323	1.251	0.654
1373829_at	Fgfr2	fibroblast growth factor receptor 2	uniq_HCDvsDR_322	0	5.90	5.32	5.69	5.72	1	-0.586	-1.501	0.030	0	-0.214	-1.160	0.682	0	-0.182	-1.134	0.884
1373928_at	Il17re	interleukin 17 receptor E	uniq_HCDvsDR_322	0	5.25	4.70	4.85	4.95	1	-0.550	-1.464	0.049	0	-0.404	-1.323	0.227	0	-0.296	-1.228	0.501
1374012_at	Sypl2	synaptophysin-like 2	uniq_HCDvsDR_322	0	5.23	4.71	4.91	5.33	1	-0.524	-1.438	0.070	0	-0.323	-1.251	0.367	0	0.095	1.068	1.021
1374157_at	Pde4b	phosphodiesterase 4B, cAMP specific	uniq_HCDvsDR_322	0	6.26	5.50	5.96	6.10	1	-0.763	-1.697	0.004	0	-0.298	-1.230	0.479	0	-0.156	-1.114	0.961
1374248_at	Mybpc1	myosin binding protein C, slow type	uniq_HCDvsDR_322	0	4.19	3.65	3.81	3.92	1	-0.543	-1.457	0.044	0	-0.380	-1.301	0.239	0	-0.266	-1.202	0.588
1374446_at	Tiparp	TCDD-inducible poly(ADP-ribose) polymerase	uniq_HCDvsDR_322	0	7.25	7.88	7.28	7.30	1	0.624	1.541	0.096	0	0.027	1.019	1.119	0	0.049	1.034	1.093
1374630_at	Clic3	chloride intracellular channel 3	uniq_HCDvsDR_322	0	4.19	3.65	3.88	3.78	1	-0.540	-1.453	0.055	0	-0.304	-1.234	0.462	0	-0.409	-1.328	0.219
1374709_at	---	---	uniq_HCDvsDR_322	0	6.56	7.33	6.88	6.50	1	0.774	1.710	0.028	0	0.328	1.255	0.425	0	-0.057	-1.040	1.149
1374724_at	Mapk4	mitogen-activated protein kinase 4	uniq_HCDvsDR_322	0	6.93	7.64	7.09	6.77	1	0.708	1.633	0.044	0	0.161	1.118	0.799	0	-0.161	-1.118	0.958
1374966_at	Dcx	doublecortin	uniq_HCDvsDR_322	0	9.02	8.50	8.85	9.07	1	-0.525	-1.439	0.067	0	-0.179	-1.132	0.778	0	0.044	1.031	1.108
1375403_at	---	---	uniq_HCDvsDR_322	0	4.16	3.62	3.79	3.91	1	-0.540	-1.454	0.060	0	-0.367	-1.290	0.289	0	-0.244	-1.184	0.682
1375412_at	Arsb	arylsulfatase B	uniq_HCDvsDR_322	0	9.52	10.14	9.63	9.48	1	0.617	1.534	0.095	0	0.114	1.083	1.000	0	-0.035	-1.025	1.166
1375551_at	Ccdc86	coiled-coil domain containing 86	uniq_HCDvsDR_322	0	9.73	10.44	10.15	9.60	1	0.705	1.630	0.049	0	0.418	1.336	0.269	0	-0.130	-1.095	1.024
1375606_at	---	---	uniq_HCDvsDR_322	0	3.67	3.12	3.31	3.23	1	-0.550	-1.464	0.046	0	-0.356	-1.280	0.281	0	-0.437	-1.354	0.174
1375706_at	LOC1003619:	rCG54286-like	uniq_HCDvsDR_322	0	6.04	5.52	5.62	5.57	1	-0.516	-1.430	0.071	0	-0.414	-1.332	0.176	0	-0.473	-1.388	0.130
1375787_at	RGD1304878	similar to 2410024A21Rik protein	uniq_HCDvsDR_322	0	4.16	3.66	3.91	3.98	1	-0.501	-1.415	0.081	0	-0.248	-1.187	0.500	0	-0.180	-1.133	0.825
1375849_at	Rgma	RGM domain family, member A	uniq_HCDvsDR_322	0	6.07	5.48	5.93	5.58	1	-0.592	-1.507	0.025	0	-0.143	-1.104	0.869	0	-0.485	-1.399	0.128
1375925_at	---	---	uniq_HCDvsDR_322	0	4.63	4.06	4.31	4.22	1	-0.571	-1.486	0.030	0	-0.316	-1.245	0.386	0	-0.407	-1.326	0.232
1376105_at	Col14a1	collagen, type XIV, alpha 1	uniq_HCDvsDR_322	0	7.85	7.32	7.79	8.54	1	-0.530	-1.444	0.065	0	-0.068	-1.048	1.030	0	0.683	1.605	0.124
1376379_a_at	Adprm	ADP-ribose/CDP-alcohol diphosphatase, manganese	uniq_HCDvsDR_322	0	6.21	6.93	6.64	6.27	1	0.724	1.652	0.048	0	0.430	1.347	0.245	0	0.059	1.042	1.069
1376434_at	---	---	uniq_HCDvsDR_322	0	5.58	4.93	5.87	5.57	1	-0.644	-1.562	0.011	0	0.289	1.222	0.454	0	-0.008	-1.005	1.142
1376487_at	Rhcg	Rh family, C glycoprotein	uniq_HCDvsDR_322	0	5.35	4.82	5.30	5.04	1	-0.537	-1.451	0.057	0	-0.051	-1.036	1.077	0	-0.314	-1.243	0.410
1376505_at	---	---	uniq_HCDvsDR_322	0	6.04	5.48	5.73	5.72	1	-0.562	-1.477	0.040	0	-0.310	-1.240	0.441	0	-0.315	-1.244	0.453
1376507_a_at	Lemd2	LEM domain containing 2	uniq_HCDvsDR_322	0	5.04	4.53	4.82	4.66	1	-0.506	-1.420	0.091	0	-0.217	-1.162	0.700	0	-0.378	-1.300	0.275
1376648_at	Mycn	v-myc myelocytomatosis viral related oncogene, neu	uniq_HCDvsDR_322	0	6.14	6.86	6.37	5.78	1	0.715	1.642	0.043	0	0.233	1.175	0.628	0	-0.366	-1.288	0.320
1376734_at	---	---	uniq_HCDvsDR_322	0	6.47	7.41	6.72	6.11	1	0.943	1.922	0.008	0	0.248	1.188	0.621	0	-0.354	-1.278	0.348
1376924_a_at	Palmd	palmdelphin	uniq_HCDvsDR_322	0	7.06	6.49	6.75	6.63	1	-0.570	-1.485	0.035	0	-0.310	-1.239	0.369	0	-0.432	-1.349	0.192
1377059_at	Mapk10 /// M	mitogen activated protein kinase 10 /// mitogen-act	uniq_HCDvsDR_322	0	5.97	5.32	5.54	6.08	1	-0.648	-1.567	0.009	0	-0.436	-1.353	0.150	0	0.104	1.074	1.045
1377064_at	Dusp6	dual specificity phosphatase 6	uniq_HCDvsDR_322	0	8.60	9.58	8.69	8.75	1	0.981	1.974	0.006	0	0.096	1.069	1.041	0	0.155	1.114	0.987
1377213_at	---	---	uniq_HCDvsDR_322	0	9.29	10.01	9.70	9.88	1	0.723	1.650	0.043	0	0.408	1.327	0.279	0	0.587	1.502	0.204
1377232_at	Limch1	LIM and calponin homology domains 1	uniq_HCDvsDR_322	0	9.94	9.28	9.69	10.04	1	-0.655	-1.574	0.008	0	-0.245	-1.185	0.560	0	0.097	1.069	1.069
1377265_at	Hccs	holocytochrome c synthase	uniq_HCDvsDR_322	0	6.25	5.70	5.77	6.25	1	-0.550	-1.464	0.044	0	-0.474	-1.389	0.083	0	0.000	1.000	1.128
1377343_at	---	---	uniq_HCDvsDR_322	0	5.23	4.67	5.05	5.00	1	-0.557	-1.471	0.039	0	-0.177	-1.131	0.771	0	-0.229	-1.172	0.717
1377375_at	Aass	aminoadipate-semialdehyde synthase	uniq_HCDvsDR_322	0	10.26	9.76	10.15	10.05	1	-0.505	-1.419	0.089	0	-0.108	-1.078	0.943	0	-0.215	-1.160	0.784

Affymetrix Probeset ID	Gene Symbol	Gene Title	Figure 7 A Venn n=2289	Present in any 2 analyses n=423 1=yes 0=no	Mean log2 intensity DR+/+ ND	Mean log2 intensity DR+/+ HCD	Mean log2 intensity DR+/+ B/S	Mean log2 intensity F+/+ day 40 ND	DR+/+HCD vs DR+/+ND FDR<10% Log2 ratio >0.5 n=636	Log2 ratio DR+/+HCD vs DR+/+ ND	Fold-Change DR+/+H CD vs DR+/+ ND	FDR DR+/+H CD vs DR+/+ ND	DR+/+B/S vs DR+/+ND Log2 ratio >0.5 n=350	Log2 ratio DR+/+ B/S vs DR+/+ ND	Fold-Change DR+/+B /S vs DR+/+ ND	FDR DR+/+B /S vs DR+/+ ND	F+/+ vs DR+/+N D Log2 ratio >0.5 n=1209	Log2 ratio F+/+ vs DR+/+ ND	Fold-Change F+/+ vs DR+/+ ND	FDR F+/+ vs DR+/+ ND
1377570_at	---	---	uniq_HCDvsDR_322	0	4.57	3.92	4.33	4.34	1	-0.650	-1.569	0.008	0	-0.247	-1.187	0.603	0	-0.233	-1.175	0.679
1377774_at	---	---	uniq_HCDvsDR_322	0	7.00	7.65	7.12	7.28	1	0.653	1.573	0.080	0	0.121	1.088	0.915	0	0.282	1.216	0.736
1377786_at	---	---	uniq_HCDvsDR_322	0	3.48	4.29	3.70	3.53	1	0.807	1.749	0.024	0	0.220	1.165	0.552	0	0.052	1.036	1.061
1378074_at	Pdk4	pyruvate dehydrogenase kinase, isozyme 4	uniq_HCDvsDR_322	0	10.20	11.04	10.73	10.38	1	0.837	1.786	0.017	0	0.533	1.447	0.114	0	0.177	1.131	0.944
1378289_at	Dcaf6	DDB1 and CUL4 associated factor 6	uniq_HCDvsDR_322	0	4.73	4.22	4.56	4.61	1	-0.506	-1.420	0.075	0	-0.165	-1.121	0.784	0	-0.114	-1.082	1.065
1378554_at	---	---	uniq_HCDvsDR_322	0	4.77	4.26	4.51	4.48	1	-0.511	-1.425	0.075	0	-0.261	-1.199	0.546	0	-0.287	-1.220	0.528
1378675_at	Tgm3	transglutaminase 3, E polypeptide	uniq_HCDvsDR_322	0	8.32	7.72	8.27	7.95	1	-0.595	-1.511	0.025	0	-0.048	-1.034	1.085	0	-0.365	-1.288	0.308
1378842_at	Gabarapl1	GABA(A) receptor-associated protein like 1	uniq_HCDvsDR_322	0	7.53	8.24	7.64	7.44	1	0.708	1.633	0.055	0	0.109	1.079	0.860	0	-0.093	-1.066	1.061
1378958_at	Ccdc86	coiled-coil domain containing 86	uniq_HCDvsDR_322	0	9.29	9.99	9.76	9.54	1	0.703	1.628	0.047	0	0.473	1.388	0.187	0	0.247	1.187	0.807
1379055_x_at	---	---	uniq_HCDvsDR_322	0	4.01	4.68	3.98	3.67	1	0.667	1.588	0.076	0	-0.032	-1.022	1.087	0	-0.344	-1.269	0.360
1379133_at	---	---	uniq_HCDvsDR_322	0	4.51	3.95	4.19	4.33	1	-0.558	-1.473	0.041	0	-0.319	-1.248	0.377	0	-0.174	-1.128	0.817
1379238_at	Ctdspl	CTD (carboxy-terminal domain, RNA polymerase II, p	uniq_HCDvsDR_322	0	5.96	6.63	6.42	5.97	1	0.675	1.597	0.082	0	0.468	1.383	0.195	0	0.010	1.007	1.163
1379340_at	Lamc2	laminin, gamma 2	uniq_HCDvsDR_322	0	4.59	5.45	4.61	4.86	1	0.863	1.818	0.015	0	0.021	1.015	1.037	0	0.274	1.209	0.754
1379397_at	Rora	RAR-related orphan receptor A	uniq_HCDvsDR_322	0	7.27	8.03	7.50	7.59	1	0.760	1.694	0.038	0	0.228	1.171	0.643	0	0.325	1.253	0.639
1379430_at	---	---	uniq_HCDvsDR_322	0	3.52	2.94	3.39	3.51	1	-0.582	-1.497	0.029	0	-0.128	-1.092	0.861	0	-0.009	-1.006	1.157
1379601_at	Alkbh5	alkB, alkylation repair homolog 5 (E. coli)	uniq_HCDvsDR_322	0	6.13	6.76	6.39	6.62	1	0.627	1.545	0.091	0	0.257	1.195	0.611	0	0.485	1.400	0.341
1380095_at	Wfdc6a	WAP four-disulfide core domain 6A	uniq_HCDvsDR_322	0	4.74	4.22	4.55	4.88	1	-0.519	-1.433	0.076	0	-0.191	-1.142	0.754	0	0.141	1.102	0.976
1380217_at	---	---	uniq_HCDvsDR_322	0	5.37	4.79	5.12	5.14	1	-0.576	-1.491	0.031	0	-0.244	-1.184	0.595	0	-0.226	-1.169	0.668
1380306_at	---	---	uniq_HCDvsDR_322	0	6.74	7.63	6.46	7.00	1	0.885	1.847	0.011	0	-0.278	-1.213	0.455	0	0.264	1.200	0.747
1380359_at	---	---	uniq_HCDvsDR_322	0	4.75	4.25	4.47	4.66	1	-0.502	-1.416	0.089	0	-0.284	-1.217	0.472	0	-0.088	-1.063	1.010
1380611_at	Fkbp5	FK506 binding protein 5	uniq_HCDvsDR_322	0	6.04	6.70	6.11	6.11	1	0.658	1.578	0.068	0	0.066	1.047	1.094	0	0.075	1.053	1.084
1380779_at	---	---	uniq_HCDvsDR_322	0	5.40	4.88	5.08	5.08	1	-0.524	-1.438	0.070	0	-0.322	-1.250	0.406	0	-0.322	-1.250	0.425
1381089_at	---	---	uniq_HCDvsDR_322	0	3.82	4.60	3.81	3.92	1	0.783	1.721	0.028	0	-0.010	-1.007	1.022	0	0.109	1.078	0.964
1381244_at	---	---	uniq_HCDvsDR_322	0	6.27	5.71	6.01	6.09	1	-0.559	-1.473	0.040	0	-0.259	-1.197	0.550	0	-0.177	-1.131	0.900
1381279_at	Ripk2	receptor-interacting serine-threonine kinase 2	uniq_HCDvsDR_322	0	6.61	7.27	6.90	6.86	1	0.657	1.577	0.084	0	0.290	1.223	0.471	0	0.245	1.185	0.790
1381333_at	---	---	uniq_HCDvsDR_322	0	3.62	4.27	4.12	3.74	1	0.657	1.576	0.075	0	0.505	1.419	0.157	0	0.122	1.088	1.030
1381354_at	---	---	uniq_HCDvsDR_322	0	6.32	5.78	5.95	6.08	1	-0.541	-1.455	0.054	0	-0.373	-1.295	0.290	0	-0.241	-1.182	0.664
1381370_at	---	---	uniq_HCDvsDR_322	0	4.24	5.16	4.42	4.48	1	0.925	1.899	0.010	0	0.184	1.136	0.785	0	0.242	1.182	0.826
1381568_at	Nek4	NIMA-related kinase 4	uniq_HCDvsDR_322	0	4.10	3.53	3.85	3.68	1	-0.564	-1.478	0.036	0	-0.244	-1.184	0.566	0	-0.415	-1.334	0.212
1381583_at	---	---	uniq_HCDvsDR_322	0	5.36	6.04	5.71	5.31	1	0.676	1.598	0.067	0	0.350	1.274	0.394	0	-0.055	-1.039	1.152
1381784_at	Tbc1d9b	TBC1 domain family, member 9B (with GRAM doma	uniq_HCDvsDR_322	0	6.02	5.49	5.82	5.73	1	-0.527	-1.441	0.061	0	-0.199	-1.148	0.758	0	-0.293	-1.226	0.474
1381867_at	Cd99	CD99 antigen	uniq_HCDvsDR_322	0	8.34	7.83	8.09	8.28	1	-0.503	-1.418	0.089	0	-0.243	-1.183	0.635	0	-0.057	-1.040	1.098
1381892_at	Nav2	neuron navigator 2	uniq_HCDvsDR_322	0	4.86	4.28	4.71	4.58	1	-0.578	-1.493	0.031	0	-0.143	-1.104	0.888	0	-0.272	-1.208	0.582
1381931_at	Ablim1	actin-binding LIM protein 1	uniq_HCDvsDR_322	0	5.07	4.53	4.72	4.93	1	-0.530	-1.444	0.058	0	-0.350	-1.275	0.318	0	-0.137	-1.099	0.966
1382171_at	Tsc22d2	TSC22 domain family, member 2	uniq_HCDvsDR_322	0	5.27	5.95	5.69	5.53	1	0.687	1.610	0.061	0	0.428	1.346	0.245	0	0.261	1.198	0.687
1382193_at	Cisd2	CDGSH iron sulfur domain 2	uniq_HCDvsDR_322	0	8.57	9.22	8.67	8.87	1	0.654	1.574	0.078	0	0.101	1.072	0.899	0	0.305	1.235	0.690
1382433_at	Sorcs1	sortilin-related VPS10 domain containing receptor 1	uniq_HCDvsDR_322	0	4.69	4.01	4.66	4.24	1	-0.679	-1.601	0.008	0	-0.036	-1.025	0.972	0	-0.456	-1.371	0.181
1382436_at	Chmp1b /// F	charged multivesicular body protein 1B /// similar to	uniq_HCDvsDR_322	0	5.61	6.28	6.13	5.94	1	0.662	1.582	0.075	0	0.514	1.428	0.132	0	0.329	1.256	0.645
1382443_at	Pabpc4	poly(A) binding protein, cytoplasmic 4	uniq_HCDvsDR_322	0	5.72	6.47	5.62	6.37	1	0.753	1.686	0.042	0	-0.096	-1.069	0.913	0	0.649	1.568	0.160
1382511_at	E2f1	E2F transcription factor 1	uniq_HCDvsDR_322	0	6.63	5.97	6.37	6.61	1	-0.655	-1.574	0.008	0	-0.260	-1.198	0.558	0	-0.016	-1.011	1.161
1382524_at	Zbtb20	zinc finger and BTB domain containing 20	uniq_HCDvsDR_322	0	5.48	6.18	5.60	6.20	1	0.697	1.621	0.075	0	0.117	1.084	0.721	0	0.711	1.637	0.137
1382593_at	Etv1	ets variant 1	uniq_HCDvsDR_322	0	8.07	8.91	8.22	8.02	1	0.843	1.794	0.016	0	0.152	1.111	0.881	0	-0.046	-1.032	1.161
1382623_at	Rhebl1	Ras homolog enriched in brain like 1	uniq_HCDvsDR_322	0	5.87	6.52	6.16	6.20	1	0.656	1.576	0.077	0	0.290	1.222	0.491	0	0.328	1.255	0.643
1382778_at	Dusp6	dual specificity phosphatase 6	uniq_HCDvsDR_322	0	6.59	7.28	6.71	6.99	1	0.695	1.619	0.050	0	0.121	1.088	0.969	0	0.404	1.323	0.477
1382901_at	Gfi1b	growth factor independent 1B transcription represso	uniq_HCDvsDR_322	0	6.20	5.66	5.96	6.30	1	-0.538	-1.452	0.054	0	-0.241	-1.182	0.608	0	0.103	1.074	1.067
1382954_at	---	---	uniq_HCDvsDR_322	0	3.77	4.70	3.80	3.99	1	0.929	1.904	0.010	0	0.023	1.016	1.115	0	0.220	1.165	0.874
1383078_at	Efnb3	ephrin B3	uniq_HCDvsDR_322	0	7.33	8.01	7.66	6.94	1	0.683	1.606	0.053	0	0.329	1.256	0.422	0	-0.383	-1.304	0.265
1383234_at	---	---	uniq_HCDvsDR_322	0	6.80	7.49	7.24	7.13	1	0.687	1.610	0.051	0	0.439	1.356	0.249	0	0.324	1.252	0.651
1383443_at	LOC498685	similar to UPF0308 protein C9orf21	uniq_HCDvsDR_322	0	6.70	7.39	6.83	7.01	1	0.691	1.615	0.078	0	0.127	1.092	0.742	0	0.311	1.241	0.618
1383447_at	Etv5	ets variant 5	uniq_HCDvsDR_322	0	6.13	6.83	6.16	6.57	1	0.705	1.630	0.051	0	0.033	1.023	1.064	0	0.440	1.356	0.427
1383522_at	---	---	uniq_HCDvsDR_322	0	6.86	7.51	7.15	6.84	1	0.649	1.568	0.078	0	0.293	1.225	0.509	0	-0.017	-1.012	1.162
1383531_at	Crebrf	CREB3 regulatory factor	uniq_HCDvsDR_322	0	7.59	8.40	7.97	7.93	1	0.816	1.760	0.023	0	0.384	1.305	0.333	0	0.339	1.265	0.621
1383696_at	Ptpla	protein tyrosine phosphatase-like (proline instead o	uniq_HCDvsDR_322	0	6.12	6.76	6.35	6.30	1	0.636	1.554	0.083	0	0.224	1.168	0.700	0	0.175	1.129	0.959
1383888_at	LOC307495	similar to biliverdin reductase B (flavin reductase (N/	uniq_HCDvsDR_322	0	5.45	4.88	5.06	5.42	1	-0.564	-1.479	0.038	0	-0.387	-1.307	0.257	0	-0.024	-1.017	1.157
1383919_at	---	---	uniq_HCDvsDR_322	0	5.84	6.50	6.23	5.91	1	0.653	1.572	0.075	0	0.389	1.309	0.325	0	0.061	1.043	1.082
1384145_at	RGD1310495	similar to KIAA1919 protein	uniq_HCDvsDR_322	0	5.69	6.38	6.13	5.66	1	0.698	1.622	0.051	0	0.443	1.359	0.228	0	-0.025	-1.017	1.139
1384302_at	Slc6a17	solute carrier family 6 (neurotransmitter transporte	uniq_HCDvsDR_322	0	8.30	8.97	8.59	8.77	1	0.673	1.594	0.076	0	0.296	1.228	0.461	0	0.477	1.392	0.360
1384544_at	Pon3	paraoxonase 3	uniq_HCDvsDR_322	0	5.87	5.30	5.43	5.83	1	-0.571	-1.485	0.034	0	-0.440	-1.357	0.133	0	-0.034	-1.024	1.145
1384603_at	Abca4	ATP-binding cassette, subfamily A (ABC1), member 4	uniq_HCDvsDR_322	0	3.91	3.40	3.63	3.71	1	-0.511	-1.425	0.081	0	-0.284	-1.218	0.479	0	-0.200	-1.149	0.827
1384622_at	Fra10ac1	fragile site, folic acid type, rare, fra(10)(q23.3) or fra	uniq_HCDvsDR_322	0	4.45	3.91	4.24	4.35	1	-0.541	-1.455	0.051	0	-0.217	-1.163	0.665	0	-0.105	-1.075	1.038
1384771_at	Tmem132e	transmembrane protein 132E	uniq_HCDvsDR_322	0	4.01	3.44	3.72	3.67	1	-0.570	-1.485	0.029	0	-0.294	-1.226	0.428	0	-0.344	-1.270	0.371
1384824_at	Pcdh18	protocadherin 18	uniq_HCDvsDR_322	0	6.32	5.76	6.26	5.82	1	-0.557	-1.471	0.040	0	-0.059	-1.041	1.017	0	-0.495	-1.409	0.107
1384882_at	Hectd2	HECT domain containing 2	uniq_HCDvsDR_322	0	5.28	6.13	5.79	5.99	1	0.850	1.803	0.015	0	0.510	1.424	0.151	0	0.712	1.638	0.125
1384930_at	Ccdc13	coiled-coil domain containing 13	uniq_HCDvsDR_322	0	5.89	5.38	5.64	5.66	1	-0.510	-1.424	0.079	0	-0.252	-1.191	0.602	0	-0.231	-1.173	0.696
1385029_at	---	---	uniq_HCDvsDR_322	0	5.25	4.70	4.79	5.29	1	-0.553	-1.467	0.047	0	-0.459	-1.375	0.118	0	0.033	1.023	1.099
1385176_at	---	---	uniq_HCDvsDR_322	0	4.14	3.60	4.01	4.33	1	-0.541	-1.455	0.048	0	-0.132	-1.096	0.845	0	0.190	1.141	0.870
1385180_at	---	---	uniq_HCDvsDR_322	0	4.81	4.19	4.38	4.58	1	-0.623	-1.540	0.016	0	-0.430	-1.347	0.166	0	-0.231	-1.174	0.675
1385182_at	Pkp1	plakophilin 1	uniq_HCDvsDR_322	0	5.85	5.26	5.60	5.93	1	-0.586	-1.501	0.027	0	-0.251	-1.190	0.626	0	0.076	1.054	1.040
1385296_at	Trmt12	tRNA methyltransferase 12 homolog (S. cerevisiae)	uniq_HCDvsDR_322	0	5.29	5.94	5.71	5.34	1	0.654	1.573	0.076	0	0.421	1.339	0.269	0	0.054	1.038	1.090
1385351_at	Sh3d21	SH3 domain containing 21	uniq_HCDvsDR_322	0	4.58	4.08	4.34	4.33	1	-0.503	-1.417	0.093	0	-0.248	-1.188	0.604	0	-0.256	-1.194	0.561
1385483_at	Vsig8	V-set and immunoglobulin domain containing 8	uniq_HCDvsDR_322	0	6.27	5.72	5.93	5.87	1	-0.545	-1.459	0.049	0	-0.339	-1.265	0.347	0	-0.401	-1.321	0.229
1385531_at	Psmb5	proteasome (prosome, macropain) subunit, beta typ	uniq_HCDvsDR_322	0	4.73	4.20	4.54	4.54	1	-0.536	-1.450	0.058	0	-0.197	-1.146	0.739	0	-0.193	-1.143	0.806
1385585_at	LOC690286	similar to hepatic leukemia factor	uniq_HCDvsDR_322	0	6.96	7.75	7.44	7.32	1	0.795	1.735	0.023	0	0.484	1.398	0.179	0	0.362	1.285	0.572
1385687_at	Fam25c	family with sequence similarity 25, member C	uniq_HCDvsDR_322	0	5.64	5.05	5.36	5.53	1	-0.593	-1.509	0.024	0	-0.279	-1.213	0.514	0	-0.116	-1.084	1.054
1385749_at	---	---	uniq_HCDvsDR_322	0	5.30	4.56	4.90	5.33	1	-0.734	-1.664	0.002	0	-0.393	-1.313	0.233	0	0.037	1.026	1.100
1386226_at	Tubgcp4	tubulin, gamma complex associated protein 4	uniq_HCDvsDR_322	0	4.89	4.38	4.41	4.91	1	-0.505	-1.420	0.080	0	-0.474	-1.389	0.089	0	0.021	1.015	1.076
1386538_at	RGD1310495	similar to KIAA1919 protein	uniq_HCDvsDR_322	0	4.47	5.10	4.65	4.31	1	0.629	1.546	0.095	0	0.174	1.128	0.772	0	-0.162	-1.119	0.945
1386675_at	---	---	uniq_HCDvsDR_322	0	3.86	4.70	4.18	4.29	1	0.846	1.797	0.018	0	0.321	1.249	0.424	0	0.437	1.353	0.448
1386785_a_at	---	---	uniq_HCDvsDR_322	0	7.84	8.56	8.08	8.10	1	0.721	1.648	0.050	0	0.235	1.177	0.588	0	0.255	1.193	0.773
1386827_at	LOC363458	similar to procollagen, type IV, alpha 6	uniq_HCDvsDR_322	0	6.00	5.45	5.71	5.95	1	-0.549	-1.463	0.046	0	-0.291	-1.223	0.453	0	-0.044	-1.031	1.052
1386870_at	Glul	glutamate-ammonia ligase	uniq_HCDvsDR_322	0	9.06	9.72	9.05	9.68	1	0.661	1.581	0.082	0	-0.007	-1.005	1.117	0	0.621	1.538	0.174
1386899_at	Ctsh	cathepsin H	uniq_HCDvsDR_322	0	8.47	9.50	8.63	9.10	1	1.028	2.040	0.004	0	0.164	1.120	0.874	0	0.632	1.550	0.165
1386935_at	Nr4a1	nuclear receptor subfamily 4, group A, member 1	uniq_HCDvsDR_322	0	8.41	9.07	8.61	9.11	1	0.661	1.581	0.095	0	0.202	1.150	0.587	0	0.708	1.633	0.128
1386938_at	Anpep	alanyl (membrane) aminopeptidase	uniq_HCDvsDR_322	0	4.97	4.40	4.71	5.30	1	-0.565	-1.479	0.036	0	-0.255	-1.193	0.548	0	0.335	1.261	0.621
1386965_at	Lpl	lipoprotein lipase	uniq_HCDvsDR_322	0	9.49	10.20	9.91	9.44	1	0.712	1.638	0.043	0	0.422	1.340	0.270	0	-0.046	-1.033	1.155
1387024_at	Dusp6	dual specificity phosphatase 6	uniq_HCDvsDR_322	0	8.13	9.12	8.16	8.57	1	0.995	1.994	0.006	0	0.033	1.023	1.097	0	0.436	1.353	0.429
1387033_at	LOC1009096:	mitochondrial brown fat uncoupling protein 1-like /	uniq_HCDvsDR_322	0	4.30	3.65	4.04	4.86	1	-0.642	-1.561	0.011	0	-0.255	-1.193	0.548	0	0.564	1.478	0.243
1387157_at	Pmfbp1	polyamine modulated factor 1 binding protein 1	uniq_HCDvsDR_322	0	4.79	4.29	4.44	4.53	1	-0.501	-1.415	0.087	0	-0.355	-1.279	0.325	0	-0.269	-1.205	0.582
1387201_at	Rnf138	ring finger protein 138, E3 ubiquitin protein ligase	uniq_HCDvsDR_322	0	7.81	8.50	8.05	8.09	1	0.699	1.623	0.060	0	0.245	1.185	0.577	0	0.287	1.220	0.709
1387306_a_at	Egr2	early growth response 2	uniq_HCDvsDR_322	0	6.06	6.98	6.09	6.38	1	0.923	1.896	0.011	0	0.030	1.021	0.877	0	0.324	1.251	0.609
1387452_a_at	Nfyb	nuclear transcription factor-Y beta	uniq_HCDvsDR_322	0	6.17	6.81	6.51	5.80	1	0.648	1.567	0.096	0	0.348	1.273	0.385	0	-0.370	-1.293	0.323
1387546_at	Cdk12	cyclin-dependent kinase 12	uniq_HCDvsDR_322	0	5.50	6.17	5.71	5.97	1	0.671	1.592	0.077	0	0.209	1.156	0.536	0	0.475	1.390	0.385
1387579_at	SIRT2	sirtuin 2	uniq_HCDvsDR_322	0	5.07	4.54	4.69	4.97	1	-0.523	-1.437	0.068	0	-0.375	-1.296	0.281	0	-0.098	-1.070	1.042
1387595_at	Gif	gastric intrinsic factor	uniq_HCDvsDR_322	0	5.10	4.54	4.61	5.09	1	-0.554	-1.468	0.040	0	-0.488	-1.402	0.068	0	-0.009	-1.006	1.141
1387599_a_at	Nqo1	NAD(P)H dehydrogenase, quinone 1	uniq_HCDvsDR_322	0	7.12	6.56	6.93	7.05	1	-0.557	-1.471	0.042	0	-0.189	-1.140	0.773	0	-0.064	-1.046	1.142
1387607_at	Npffr2	neuropeptide FF receptor 2	uniq_HCDvsDR_322	0	5.65	5.05	5.49	5.64	1	-0.595	-1.511	0.024	0	-0.155	-1.113	0.860	0	-0.010	-1.007	1.123
1387729_at	Ggt5	gamma-glutamyltransferase 5	uniq_HCDvsDR_322	0	6.46	7.21	6.52	6.50	1	0.753	1.685	0.034	0	0.057	1.040	1.064	0	0.039	1.027	1.092
1387762_s_at	Jund	jun D proto-oncogene	uniq_HCDvsDR_322	0	4.62	5.30	4.50	4.63	1	0.680	1.602	0.095	0	-0.114	-1.082	0.720	0	0.013	1.009	1.047
1387882_at	Klf9	Kruppel-like factor 9	uniq_HCDvsDR_322	0	6.37	7.04	6.08	6.78	1	0.670	1.591	0.077	0	-0.287	-1.220	0.456	0	0.412	1.331	0.483
1387908_at	Rasd1	RAS, dexamethasone-induced 1	uniq_HCDvsDR_322	0	8.77	9.68	8.90	8.95	1	0.908	1.877	0.011	0	0.125	1.091	0.876	0	0.178	1.131	0.931
1388004_at	Gpr37l1	G protein-coupled receptor 37-like 1	uniq_HCDvsDR_322	0	5.97	5.42	5.56	5.95	1	-0.549	-1.463	0.045	0	-0.403	-1.323	0.216	0	-0.014	-1.010	1.164
1388168_a_at	---	---	uniq_HCDvsDR_322	0	4.39	3.74	3.93	4.31	1	-0.651	-1.570	0.008	0	-0.457	-1.373	0.113	0	-0.074	-1.052	1.091
1388187_at	Camk2a	calcium/calmodulin-dependent protein kinase II alp	uniq_HCDvsDR_322	0	4.90	4.32	4.69	4.75	1	-0.580	-1.495	0.029	0	-0.211	-1.157	0.694	0	-0.150	-1.110	0.968
1388271_at	Mt2A	metallothionein 2A	uniq_HCDvsDR_322	0	8.93	10.39	9.28	9.40	1	1.459	2.750	0.000	0	0.344	1.270	0.392	0	0.462	1.377	0.376
1388316_at	RGD1566320	RGD1566320	uniq_HCDvsDR_322	0	7.94	8.55	8.48	7.95	1	0.619	1.535	0.094	0	0.540	1.454	0.106	0	0.010	1.007	1.178
1388547_at	Cldn4	claudin 4	uniq_HCDvsDR_322	0	7.00	7.96	7.16	6.71	1	0.956	1.939	0.008	0	0.158	1.115	0.877	0	-0.295	-1.227	0.507
1388872_at	Idi1	isopentenyl-diphosphate delta isomerase 1	uniq_HCDvsDR_322	0	8.24	7.68	8.22	8.29	1	-0.567	-1.482	0.040	0	-0.027	-1.019	1.098	0	0.043	1.030	1.090

Affymetrix Probeset ID	Gene Symbol	Gene Title	Figure 7 A Venn n=2289	Present in any 2 analyses n=423 1=yes 0=no	Mean log2 intensity DR+/+ ND	Mean log2 intensity DR+/+ HCD	Mean log2 intensity DR+/+ B/S	Mean log2 intensity F+/+ day 40 ND	DR+/+HCD vs DR+/+ND FDR<10% Log2 ratio >0.5 n=636	Log2 ratio DR+/+HCD vs DR+/+ ND	Fold-Change DR+/+HCD vs DR+/+ ND	FDR DR+/+HCD vs DR+/+ ND	DR+/+B/S vs DR+/+ND Log2 ratio >0.5 n=350	Log2 ratio DR+/+ B/S vs DR+/+ ND	Fold-Change DR+/+B/S vs DR+/+ ND	FDR DR+/+B/S vs DR+/+ ND	F+/+ vs DR+/+ND Log2 ratio >0.5 n=1209	Log2 ratio F+/+ vs DR+/+ ND	Fold-Change F+/+ vs DR+/+ ND	FDR F+/+ vs DR+/+ ND
1388936_at	Cdh11	cadherin 11	uniq_HCDvsDR_322	0	7.18	6.67	6.74	7.11	1	-0.518	-1.432	0.061	0	-0.448	-1.364	0.125	0	-0.072	-1.051	1.089
1389100_at	Epm2aip1	EPM2A (laforin) interacting protein 1	uniq_HCDvsDR_322	0	4.63	5.44	4.71	5.02	1	0.810	1.753	0.023	0	0.085	1.061	0.985	0	0.395	1.315	0.507
1389408_at	LOC1003595	ribonucleotide reductase M2 polypeptide /// ribonu	uniq_HCDvsDR_322	0	7.59	6.83	7.27	7.45	1	-0.763	-1.697	0.002	0	-0.316	-1.245	0.393	0	-0.138	-1.101	0.994
1389467_at	Tmem100	transmembrane protein 100	uniq_HCDvsDR_322	0	7.21	6.60	6.83	6.98	1	-0.612	-1.528	0.021	0	-0.374	-1.296	0.267	0	-0.226	-1.169	0.737
1389500_at	---	---	uniq_HCDvsDR_322	0	7.59	8.29	7.82	7.41	1	0.701	1.625	0.049	0	0.224	1.168	0.673	0	-0.180	-1.133	0.890
1389503_at	Upk3bl	uroplakin 3B-like	uniq_HCDvsDR_322	0	5.64	6.39	6.02	6.12	1	0.749	1.681	0.038	0	0.388	1.309	0.306	0	0.480	1.395	0.353
1390073_at	Sowaha	sosondowah ankyrin repeat domain family member	uniq_HCDvsDR_322	0	4.64	4.04	4.27	4.27	1	-0.597	-1.513	0.021	0	-0.369	-1.291	0.299	0	-0.369	-1.291	0.307
1390223_at	---	---	uniq_HCDvsDR_322	0	6.00	5.48	5.57	6.02	1	-0.518	-1.432	0.068	0	-0.425	-1.342	0.166	0	0.019	1.013	1.099
1390256_at	---	---	uniq_HCDvsDR_322	0	5.44	4.93	5.06	5.19	1	-0.503	-1.418	0.093	0	-0.372	-1.294	0.294	0	-0.245	-1.185	0.666
1390393_at	---	---	uniq_HCDvsDR_322	0	4.46	3.85	3.97	4.24	1	-0.611	-1.528	0.016	0	-0.483	-1.398	0.081	0	-0.220	-1.165	0.731
1390958_at	Sertm1	serine-rich and transmembrane domain containing	uniq_HCDvsDR_322	0	4.42	3.78	4.45	4.15	1	-0.640	-1.558	0.012	0	0.028	1.019	1.031	0	-0.270	-1.206	0.585
1391021_at	Cgnl1	cingulin-like 1	uniq_HCDvsDR_322	0	6.77	6.27	6.58	6.59	1	-0.501	-1.415	0.096	0	-0.190	-1.141	0.787	0	-0.182	-1.134	0.882
1391022_at	Lamb3	laminin, beta 3	uniq_HCDvsDR_322	0	5.06	4.49	4.81	5.05	1	-0.566	-1.481	0.040	0	-0.246	-1.186	0.603	0	-0.011	-1.007	1.164
1391030_at	Camta1	calmodulin binding transcription activator 1	uniq_HCDvsDR_322	0	5.70	6.42	6.16	5.71	1	0.726	1.654	0.043	0	0.462	1.377	0.198	0	0.008	1.006	1.177
1391042_at	Mtf2	metal response element binding transcription factor	uniq_HCDvsDR_322	0	4.94	5.70	5.36	5.10	1	0.760	1.694	0.045	0	0.427	1.344	0.245	0	0.166	1.122	0.913
1391241_at	Etv1	ets variant 1	uniq_HCDvsDR_322	0	7.80	8.52	8.05	7.99	1	0.716	1.643	0.044	0	0.248	1.188	0.625	0	0.187	1.139	0.930
1391268_at	Art5	ADP-ribosyltransferase 5	uniq_HCDvsDR_322	0	4.46	3.91	4.02	3.99	1	-0.548	-1.462	0.048	0	-0.441	-1.358	0.138	0	-0.475	-1.390	0.131
1391276_at	---	---	uniq_HCDvsDR_322	0	5.37	4.84	4.95	4.97	1	-0.528	-1.442	0.065	0	-0.417	-1.335	0.190	0	-0.392	-1.312	0.246
1391354_at	---	---	uniq_HCDvsDR_322	0	5.74	5.15	5.25	5.56	1	-0.589	-1.504	0.026	0	-0.483	-1.398	0.077	0	-0.179	-1.132	0.858
1391515_at	---	---	uniq_HCDvsDR_322	0	4.18	3.64	3.77	3.84	1	-0.545	-1.459	0.051	0	-0.411	-1.330	0.183	0	-0.339	-1.265	0.365
1391551_at	---	---	uniq_HCDvsDR_322	0	6.01	6.77	5.91	5.97	1	0.763	1.697	0.041	0	-0.100	-1.072	0.967	0	-0.039	-1.028	1.156
1391563_at	Mum1l1	melanoma associated antigen (mutated) 1-like 1	uniq_HCDvsDR_322	0	6.91	7.72	6.95	6.95	1	0.805	1.747	0.023	0	0.035	1.025	1.052	0	0.040	1.028	1.081
1391605_at	---	---	uniq_HCDvsDR_322	0	6.89	7.81	7.42	6.61	1	0.921	1.893	0.009	0	0.534	1.448	0.115	0	-0.279	-1.213	0.550
1391759_at	---	---	uniq_HCDvsDR_322	0	4.96	5.75	4.98	5.34	1	0.793	1.732	0.040	0	0.017	1.012	1.107	0	0.380	1.301	0.543
1391766_at	Hs3st5	heparan sulfate (glucosamine) 3-O-sulfotransferase	uniq_HCDvsDR_322	0	4.01	3.50	3.69	3.93	1	-0.518	-1.432	0.087	0	-0.324	-1.252	0.359	0	-0.079	-1.057	1.003
1391830_at	Cpne8	copine VIII	uniq_HCDvsDR_322	0	5.72	6.46	5.87	5.45	1	0.737	1.667	0.043	0	0.154	1.112	0.817	0	-0.268	-1.204	0.587
1391869_at	---	---	uniq_HCDvsDR_322	0	7.52	6.93	7.25	7.57	1	-0.585	-1.500	0.029	0	-0.266	-1.202	0.487	0	0.056	1.039	1.071
1391916_at	---	---	uniq_HCDvsDR_322	0	6.66	6.10	6.73	6.83	1	-0.560	-1.475	0.040	0	0.067	1.047	1.071	0	0.171	1.126	0.920
1392406_at	Ipp	intracisternal A particle-promoted polypeptide	uniq_HCDvsDR_322	0	4.58	5.30	4.48	4.76	1	0.723	1.650	0.049	0	-0.098	-1.070	0.692	0	0.177	1.131	0.916
1392477_at	Etv1	ets variant 1	uniq_HCDvsDR_322	0	10.22	10.88	10.37	10.15	1	0.665	1.586	0.065	0	0.155	1.114	0.889	0	-0.068	-1.048	1.145
1392613_at	---	---	uniq_HCDvsDR_322	0	5.69	6.57	5.24	6.11	1	0.881	1.841	0.011	0	-0.454	-1.370	0.113	0	0.421	1.339	0.439
1392633_at	---	---	uniq_HCDvsDR_322	0	3.55	4.24	3.74	3.61	1	0.693	1.616	0.050	0	0.191	1.142	0.777	0	0.059	1.042	1.009
1392668_at	Rbpj	recombination signal binding protein for immunoglo	uniq_HCDvsDR_322	0	4.32	5.02	4.47	4.58	1	0.703	1.628	0.071	0	0.146	1.107	0.699	0	0.258	1.196	0.701
1392791_at	---	---	uniq_HCDvsDR_322	0	5.56	6.91	6.09	5.69	1	1.348	2.546	0.001	0	0.529	1.443	0.106	0	0.131	1.095	0.775
1392953_at	Ptpla	protein tyrosine phosphatase-like (proline instead o	uniq_HCDvsDR_322	0	7.04	7.93	7.44	7.34	1	0.893	1.857	0.011	0	0.403	1.322	0.301	0	0.300	1.231	0.693
1393060_at	Adamtsl2	ADAMTS-like 2	uniq_HCDvsDR_322	0	7.92	7.38	7.49	7.64	1	-0.544	-1.458	0.051	0	-0.430	-1.347	0.159	0	-0.279	-1.213	0.562
1393331_at	Heca	headcase homolog (Drosophila)	uniq_HCDvsDR_322	0	6.48	7.24	6.62	6.86	1	0.755	1.688	0.039	0	0.140	1.102	0.808	0	0.380	1.302	0.522
1393389_at	Nr4a3	nuclear receptor subfamily 4, group A, member 3	uniq_HCDvsDR_322	0	5.20	4.64	4.84	5.52	1	-0.560	-1.475	0.041	0	-0.364	-1.287	0.298	0	0.316	1.245	0.654
1393435_at	---	---	uniq_HCDvsDR_322	0	6.24	5.67	6.21	6.54	1	-0.573	-1.487	0.034	0	-0.035	-1.025	1.101	0	0.299	1.230	0.703
1393445_at	---	---	uniq_HCDvsDR_322	0	5.37	4.86	5.14	5.04	1	-0.506	-1.421	0.088	0	-0.225	-1.168	0.664	0	-0.327	-1.254	0.420
1393631_at	---	---	uniq_HCDvsDR_322	0	5.22	5.98	5.65	5.29	1	0.767	1.702	0.038	0	0.438	1.355	0.232	0	0.071	1.050	1.005
1393641_at	Blnk	B-cell linker	uniq_HCDvsDR_322	0	7.62	8.41	8.08	7.54	1	0.792	1.731	0.025	0	0.461	1.377	0.207	0	-0.071	-1.051	1.140
1393649_at	---	---	uniq_HCDvsDR_322	0	6.11	6.82	6.34	6.59	1	0.702	1.627	0.055	0	0.225	1.169	0.654	0	0.480	1.394	0.380
1393653_at	---	---	uniq_HCDvsDR_322	0	6.62	6.10	6.47	6.23	1	-0.515	-1.429	0.076	0	-0.152	-1.111	0.871	0	-0.385	-1.306	0.262
1393659_at	Tram1l1	translocation associated membrane protein 1-like 1	uniq_HCDvsDR_322	0	4.24	4.89	4.40	4.40	1	0.652	1.572	0.085	0	0.162	1.119	0.837	0	0.166	1.122	0.970
1393843_at	Fem1b	fem-1 homolog b (C. elegans)	uniq_HCDvsDR_322	0	5.67	6.29	5.79	5.71	1	0.626	1.543	0.090	0	0.127	1.092	0.933	0	0.045	1.031	1.093
1393849_at	---	---	uniq_HCDvsDR_322	0	4.86	4.33	4.48	4.76	1	-0.532	-1.446	0.064	0	-0.376	-1.297	0.266	0	-0.102	-1.073	1.013
1393888_at	Amh	anti-Mullerian hormone	uniq_HCDvsDR_322	0	5.08	4.58	5.02	4.88	1	-0.507	-1.421	0.085	0	-0.068	-1.049	1.032	0	-0.204	-1.152	0.817
1393932_at	Samd8	sterile alpha motif domain containing 8	uniq_HCDvsDR_322	0	6.21	6.92	6.38	6.65	1	0.713	1.639	0.055	0	0.171	1.125	0.743	0	0.440	1.356	0.424
1393958_at	---	---	uniq_HCDvsDR_322	0	4.80	4.30	4.58	5.11	1	-0.502	-1.416	0.076	0	-0.228	-1.171	0.561	0	0.303	1.234	0.684
1394015_at	---	---	uniq_HCDvsDR_322	0	3.57	3.07	3.40	3.52	1	-0.502	-1.416	0.094	0	-0.178	-1.131	0.830	0	-0.057	-1.040	1.069
1394042_at	LOC681371	hypothetical protein LOC681371	uniq_HCDvsDR_322	0	6.31	6.95	6.49	6.43	1	0.646	1.565	0.084	0	0.186	1.137	0.775	0	0.119	1.086	1.014
1394200_at	Hspa2	heat shock protein 2	uniq_HCDvsDR_322	0	4.92	5.69	5.27	4.91	1	0.771	1.706	0.036	0	0.354	1.279	0.364	0	-0.012	-1.009	1.155
1394241_at	Clip1	CAP-GLY domain containing linker protein 1	uniq_HCDvsDR_322	0	4.51	5.30	5.01	4.83	1	0.792	1.731	0.036	0	0.495	1.409	0.163	0	0.315	1.244	0.647
1394490_at	Abca1	ATP-binding cassette, subfamily A (ABC1), member 1	uniq_HCDvsDR_322	0	6.26	6.91	6.66	6.55	1	0.647	1.566	0.086	0	0.398	1.317	0.306	0	0.287	1.220	0.713
1394580_at	Nprl2	nitrogen permease regulator-like 2 (S. cerevisiae)	uniq_HCDvsDR_322	0	5.64	6.37	6.08	5.83	1	0.734	1.663	0.043	0	0.442	1.359	0.247	0	0.190	1.141	0.904
1394800_at	---	---	uniq_HCDvsDR_322	0	4.74	5.62	5.26	4.98	1	0.881	1.842	0.012	0	0.523	1.437	0.116	0	0.238	1.180	0.807
1394905_at	Kmt2d /// Ml	lysine (K)-specific methyltransferase 2D /// myeloid/	uniq_HCDvsDR_322	0	8.33	7.80	7.87	8.25	1	-0.529	-1.443	0.060	0	-0.460	-1.376	0.095	0	-0.084	-1.060	1.046
1394980_at	Tmem56	transmembrane protein 56	uniq_HCDvsDR_322	0	4.54	4.02	4.19	4.37	1	-0.520	-1.434	0.065	0	-0.348	-1.272	0.335	0	-0.172	-1.127	0.831
1395082_at	Cpsf6	cleavage and polyadenylation specific factor 6, 68kD	uniq_HCDvsDR_322	0	5.43	6.38	5.64	6.21	1	0.946	1.926	0.010	0	0.206	1.154	0.548	0	0.775	1.711	0.100
1395142_at	RGD1561931	similar to KIAA2022 protein	uniq_HCDvsDR_322	0	3.88	4.57	4.22	4.16	1	0.692	1.616	0.066	0	0.347	1.272	0.377	0	0.282	1.216	0.730
1395274_at	Dst	dystonin	uniq_HCDvsDR_322	0	5.59	6.30	5.42	5.67	1	0.708	1.633	0.060	0	-0.169	-1.124	0.630	0	0.083	1.059	1.043
1395307_at	Anks1b	ankyrin repeat and sterile alpha motif domain conta	uniq_HCDvsDR_322	0	7.20	6.64	6.93	6.90	1	-0.556	-1.470	0.040	0	-0.268	-1.204	0.525	0	-0.292	-1.224	0.470
1395403_at	Stac3	SH3 and cysteine rich domain 3	uniq_HCDvsDR_322	0	5.86	5.12	5.41	5.38	1	-0.746	-1.677	0.002	0	-0.452	-1.367	0.115	0	-0.486	-1.401	0.119
1395447_at	Ermn	ermin, ERM-like protein	uniq_HCDvsDR_322	0	4.09	3.58	3.94	3.83	1	-0.510	-1.424	0.082	0	-0.146	-1.106	0.847	0	-0.259	-1.197	0.630
1395586_at	---	---	uniq_HCDvsDR_322	0	7.64	8.48	7.70	8.01	1	0.840	1.790	0.024	0	0.066	1.046	0.823	0	0.374	1.296	0.516
1395761_at	---	---	uniq_HCDvsDR_322	0	5.50	4.98	5.17	5.25	1	-0.516	-1.430	0.075	0	-0.330	-1.257	0.398	0	-0.251	-1.190	0.650
1395821_at	Cbfb	core-binding factor, beta subunit	uniq_HCDvsDR_322	0	7.03	7.97	7.11	7.31	1	0.940	1.919	0.010	0	0.078	1.056	0.886	0	0.281	1.215	0.693
1395910_at	Dot1l	DOT1-like histone H3K79 methyltransferase	uniq_HCDvsDR_322	0	4.04	3.53	3.74	3.65	1	-0.508	-1.422	0.084	0	-0.298	-1.230	0.431	0	-0.388	-1.308	0.269
1396018_at	---	---	uniq_HCDvsDR_322	0	5.07	5.71	5.23	5.27	1	0.637	1.556	0.083	0	0.159	1.117	0.857	0	0.200	1.149	0.915
1396072_at	Appbp2	amyloid beta precursor protein (cytoplasmic tail) bir	uniq_HCDvsDR_322	0	5.29	5.98	5.50	5.90	1	0.690	1.613	0.077	0	0.214	1.160	0.659	0	0.613	1.529	0.217
1396171_at	Ppil4	peptidylprolyl isomerase (cyclophilin)-like 4	uniq_HCDvsDR_322	0	5.51	6.23	5.64	6.05	1	0.723	1.651	0.053	0	0.138	1.101	0.743	0	0.540	1.454	0.284
1396208_at	Ggt5	gamma-glutamyltransferase 5	uniq_HCDvsDR_322	0	6.37	7.63	6.92	6.63	1	1.252	2.381	0.001	0	0.542	1.456	0.105	0	0.253	1.192	0.787
1396406_at	---	---	uniq_HCDvsDR_322	0	5.16	4.66	4.91	4.79	1	-0.505	-1.419	0.096	0	-0.256	-1.194	0.564	0	-0.368	-1.291	0.307
1396523_at	---	---	uniq_HCDvsDR_322	0	5.05	4.53	4.72	4.87	1	-0.525	-1.438	0.061	0	-0.335	-1.261	0.333	0	-0.182	-1.135	0.831
1396524_at	---	---	uniq_HCDvsDR_322	0	5.15	4.64	4.93	4.68	1	-0.512	-1.426	0.081	0	-0.223	-1.167	0.692	0	-0.470	-1.385	0.173
1396638_at	---	---	uniq_HCDvsDR_322	0	4.24	3.72	3.86	3.97	1	-0.522	-1.436	0.069	0	-0.379	-1.301	0.262	0	-0.265	-1.201	0.607
1396666_at	---	---	uniq_HCDvsDR_322	0	5.00	4.45	4.86	4.98	1	-0.555	-1.469	0.051	0	-0.141	-1.103	0.833	0	-0.024	-1.017	0.972
1396736_at	LOC691984	similar to Glypican-6 precursor	uniq_HCDvsDR_322	0	4.98	4.36	4.62	4.56	1	-0.619	-1.536	0.011	0	-0.361	-1.284	0.259	0	-0.418	-1.336	0.213
1396988_at	---	---	uniq_HCDvsDR_322	0	5.65	5.10	5.38	5.45	1	-0.552	-1.466	0.048	0	-0.273	-1.208	0.530	0	-0.201	-1.150	0.787
1397015_at	Etv1	ets variant 1	uniq_HCDvsDR_322	0	7.63	8.55	7.81	7.72	1	0.922	1.895	0.010	0	0.181	1.134	0.829	0	0.098	1.070	1.067
1397220_at	LOC1009112	uncharacterized LOC100911266	uniq_HCDvsDR_322	0	6.52	7.17	7.03	6.53	1	0.643	1.562	0.079	0	0.505	1.419	0.150	0	0.007	1.005	1.177
1397233_at	Dclk3	doublecortin-like kinase 3	uniq_HCDvsDR_322	0	5.49	6.66	5.72	5.38	1	1.164	2.240	0.002	0	0.230	1.173	0.636	0	-0.110	-1.079	1.060
1397255_at	Ccdc86	coiled-coil domain containing 86	uniq_HCDvsDR_322	0	8.78	9.42	8.96	8.87	1	0.644	1.562	0.095	0	0.181	1.134	0.743	0	0.098	1.071	1.029
1397277_at	---	---	uniq_HCDvsDR_322	0	4.67	5.34	5.04	5.05	1	0.671	1.592	0.075	0	0.371	1.293	0.316	0	0.377	1.299	0.539
1397341_at	Pbk	PDZ binding kinase	uniq_HCDvsDR_322	0	6.66	6.07	6.24	6.61	1	-0.588	-1.503	0.026	0	-0.416	-1.335	0.192	0	-0.043	-1.030	1.141
1397392_at	---	---	uniq_HCDvsDR_322	0	4.31	3.78	4.16	4.35	1	-0.527	-1.441	0.065	0	-0.142	-1.104	0.868	0	0.043	1.030	1.001
1397468_at	Hsdl2	hydroxysteroid dehydrogenase like 2	uniq_HCDvsDR_322	0	7.39	8.03	7.58	7.38	1	0.637	1.555	0.094	0	0.184	1.136	0.769	0	-0.013	-1.009	1.171
1397494_at	---	---	uniq_HCDvsDR_322	0	5.66	5.14	5.25	5.38	1	-0.528	-1.442	0.054	0	-0.415	-1.333	0.169	0	-0.287	-1.220	0.529
1397586_at	LOC1003611	hypothetical protein LOC100361177	uniq_HCDvsDR_322	0	5.16	4.64	4.94	4.94	1	-0.512	-1.426	0.075	0	-0.212	-1.158	0.667	0	-0.216	-1.161	0.715
1397693_at	Eif2s3x /// Eif	eukaryotic translation initiation factor 2, subunit 3,	uniq_HCDvsDR_322	0	6.83	7.65	7.04	7.21	1	0.818	1.762	0.028	0	0.205	1.152	0.565	0	0.381	1.302	0.505
1397843_at	Wdr44	WD repeat domain 44	uniq_HCDvsDR_322	0	5.15	5.89	5.41	5.72	1	0.742	1.673	0.049	0	0.269	1.205	0.436	0	0.574	1.488	0.251
1397932_at	---	---	uniq_HCDvsDR_322	0	5.33	4.78	4.96	5.32	1	-0.549	-1.463	0.046	0	-0.368	-1.290	0.286	0	-0.004	-1.003	1.049
1397946_at	Sec14l1	SEC14-like 1 (S. cerevisiae)	uniq_HCDvsDR_322	0	6.55	6.04	6.35	6.49	1	-0.505	-1.419	0.083	0	-0.203	-1.151	0.731	0	-0.063	-1.044	1.145
1398029_at	RGD1561849	similar to RIKEN cDNA 3110035E14	uniq_HCDvsDR_322	0	5.26	4.73	5.06	5.46	1	-0.524	-1.438	0.065	0	-0.195	-1.145	0.746	0	0.205	1.152	0.886
1398052_at	---	---	uniq_HCDvsDR_322	0	6.11	5.61	5.87	6.06	1	-0.501	-1.415	0.096	0	-0.234	-1.176	0.670	0	-0.043	-1.030	1.163
1398151_at	---	---	uniq_HCDvsDR_322	0	5.63	5.07	5.33	5.38	1	-0.558	-1.472	0.041	0	-0.301	-1.232	0.426	0	-0.253	-1.191	0.642
1398188_at	---	---	uniq_HCDvsDR_322	0	4.73	4.18	4.30	4.61	1	-0.547	-1.461	0.050	0	-0.428	-1.345	0.165	0	-0.125	-1.090	1.002
1398190_at	---	---	uniq_HCDvsDR_322	0	4.34	3.79	3.93	3.96	1	-0.549	-1.463	0.043	0	-0.406	-1.325	0.209	0	-0.382	-1.303	0.268
1398219_at	LOC690918	hypothetical protein LOC690918	uniq_HCDvsDR_322	0	11.24	10.59	11.01	11.28	1	-0.652	-1.571	0.011	0	-0.227	-1.170	0.650	0	0.040	1.028	1.110
1398659_at	Vwc2	von Willebrand factor C domain containing 2	uniq_HCDvsDR_322	0	7.10	8.06	7.36	7.02	1	0.961	1.947	0.006	0	0.255	1.193	0.588	0	-0.078	-1.056	1.126
1398739_at	---	---	uniq_HCDvsDR_322	0	4.58	5.52	5.08	4.86	1	0.939	1.918	0.006	0	0.491	1.405	0.168	0	0.279	1.213	0.718
1398756_at	LOC300303 /,	similar to Nucleophosmin (NPM) (Nucleolar phosphc	uniq_HCDvsDR_322	0	7.26	7.96	7.66	7.28	1	0.700	1.624	0.049	0	0.401	1.320	0.300	0	0.020	1.014	1.105
1367557_s_at	Gapdh /// LO	glyceraldehyde-3-phosphate dehydrogenase /// sim	uniq_isletLongDR_555	0	10.30	10.28	10.36	10.33	0	-0.013	-1.009	1.110	0	0.061	1.043	1.074	0	0.034	1.024	1.103
1367576_at	Gpx1	glutathione peroxidase 1	uniq_isletLongDR_555	0	9.85	9.97	9.82	9.55	0	0.122	1.088	1.012	0	-0.025	-1.018	1.071	0	-0.293	-1.225	0.508
1367586_at	Ldha	lactate dehydrogenase A	uniq_isletLongDR_555	0	7.34	7.06	6.88	6.98	0	-0.279	-1.213	0.518	0	-0.467	-1.382	0.114	0	-0.365	-1.288	0.317
1367605_at	Pfn1	profilin 1	uniq_isletLongDR_555	0	8.38	8.58	8.48	8.46	0	0.196	1.146	0.808	0	0.096	1.069	0.948	0	0.078	1.056	1.068

Affymetrix Probeset ID	Gene Symbol	Gene Title	Figure 7 A Venn n=2289	Present in any 2 analyses n=423 1=yes 0=no	Mean log2 intensity DR+/+ ND	Mean log2 intensity DR+/+ HCD	Mean log2 intensity DR+/+ B/S	Mean log2 intensity F+/+ day 40 ND	DR+/+HCD vs DR+/+ND FDR<10% Log2 ratio >0.5 n=636	Log2 ratio DR+/+HCD vs DR+/+ ND	Fold-Change DR+/+H CD vs DR+/+ ND	FDR DR+/+H CD vs DR+/+ ND	DR+/+B/S vs DR+/+ND Log2 ratio >0.5 n=350	Log2 ratio DR+/+ B/S vs DR+/+ ND	Fold-Change DR+/+B /S vs DR+/+ ND	FDR DR+/+B /S vs DR+/+ ND	F+/+ vs DR+/+N D Log2 ratio >0.5 n=1209	Log2 ratio F+/+ vs DR+/+ ND	Fold-Change F+/+ vs DR+/+ ND	FDR F+/+ vs DR+/+ ND
1367652_at	Igfbp3	insulin-like growth factor binding protein 3	uniq_isletLongDR_555	0	8.57	8.91	8.78	8.63	0	0.340	1.266	0.450	0	0.210	1.157	0.712	0	0.061	1.043	1.082
1367675_at	Cib1	calcium and integrin binding 1 (calmyrin)	uniq_isletLongDR_555	0	7.71	7.78	7.88	7.80	0	0.072	1.051	1.120	0	0.168	1.124	0.857	0	0.091	1.065	1.079
1367683_at	Kpna2 /// LOC	karyopherin alpha 2 /// karyopherin alpha 2-like	uniq_isletLongDR_555	0	8.04	7.81	7.94	7.76	0	-0.228	-1.171	0.651	0	-0.106	-1.076	0.983	0	-0.278	-1.213	0.553
1367786_at	Psmb8	proteasome (prosome, macropain) subunit, beta typ	uniq_isletLongDR_555	0	7.60	7.67	7.82	7.13	0	0.071	1.051	1.095	0	0.227	1.171	0.681	0	-0.464	-1.379	0.141
1367791_at	Ramp1	receptor (G protein-coupled) activity modifying prot	uniq_isletLongDR_555	0	5.74	5.48	5.36	5.34	0	-0.258	-1.196	0.600	0	-0.376	-1.298	0.284	0	-0.397	-1.317	0.251
1367794_at	A2m /// LOC1	alpha-2-macroglobulin /// alpha-2-macroglobulin-lil	uniq_isletLongDR_555	0	5.18	5.70	5.59	5.13	0	0.520	1.434	0.193	0	0.418	1.336	0.268	0	-0.044	-1.031	1.135
1367847_at	Nupr1	nuclear protein, transcriptional regulator, 1	uniq_isletLongDR_555	0	7.22	7.65	7.59	7.80	0	0.434	1.351	0.297	0	0.369	1.291	0.344	0	0.583	1.498	0.210
1367850_at	Fcgr2a /// LO	Fc fragment of IgG, low affinity IIa, receptor /// Low	uniq_isletLongDR_555	0	4.19	4.00	4.35	3.88	0	-0.186	-1.138	0.752	0	0.161	1.118	0.546	0	-0.307	-1.237	0.482
1367914_at	Emp3	epithelial membrane protein 3	uniq_isletLongDR_555	0	6.97	6.64	6.59	6.65	0	-0.336	-1.262	0.420	0	-0.380	-1.302	0.265	0	-0.320	-1.248	0.428
1367936_at	Stk10	serine/threonine kinase 10	uniq_isletLongDR_555	0	6.89	6.90	6.93	6.87	0	0.007	1.005	1.159	0	0.037	1.026	1.124	0	-0.023	-1.016	1.175
1367951_at	Pgam2	phosphoglycerate mutase 2 (muscle)	uniq_isletLongDR_555	0	4.16	3.88	3.97	4.02	0	-0.279	-1.213	0.557	0	-0.195	-1.145	0.693	0	-0.139	-1.101	0.994
1367975_at	Anxa3	annexin A3	uniq_isletLongDR_555	0	4.11	3.91	4.02	4.04	0	-0.205	-1.153	0.768	0	-0.099	-1.071	0.937	0	-0.074	-1.053	1.123
1368006_at	Laptm5	lysosomal protein transmembrane 5	uniq_isletLongDR_555	0	5.69	5.75	5.65	5.48	0	0.059	1.042	1.088	0	-0.040	-1.028	1.054	0	-0.205	-1.153	0.776
1368010_at	Ptpn6	protein tyrosine phosphatase, non-receptor type 6	uniq_isletLongDR_555	0	7.57	7.61	7.68	7.38	0	0.039	1.027	1.153	0	0.107	1.077	1.000	0	-0.187	-1.139	0.871
1368073_at	Irf1	interferon regulatory factor 1	uniq_isletLongDR_555	0	8.41	8.89	8.49	8.29	0	0.480	1.395	0.252	0	0.076	1.054	1.059	0	-0.118	-1.085	1.051
1368075_at	Lipa	lipase A, lysosomal acid, cholesterol esterase	uniq_isletLongDR_555	0	7.04	6.85	6.88	6.91	0	-0.187	-1.138	0.807	0	-0.158	-1.115	0.834	0	-0.130	-1.094	0.987
1368109_at	St3gal5	ST3 beta-galactoside alpha-2,3-sialyltransferase 5	uniq_isletLongDR_555	0	7.15	7.40	7.42	7.49	0	0.252	1.191	0.662	0	0.270	1.206	0.574	0	0.335	1.261	0.621
1368114_at	Fgf13	fibroblast growth factor 13	uniq_isletLongDR_555	0	4.12	3.65	3.72	4.02	0	-0.465	-1.381	0.124	0	-0.400	-1.319	0.214	0	-0.094	-1.067	1.038
1368167_at	Ctse	cathepsin E	uniq_isletLongDR_555	0	4.34	4.23	4.30	4.35	0	-0.105	-1.076	0.880	0	-0.034	-1.024	1.049	0	0.008	1.006	1.177
1368186_a_at	Syk	spleen tyrosine kinase	uniq_isletLongDR_555	0	5.76	5.72	5.56	5.95	0	-0.038	-1.027	1.133	0	-0.202	-1.150	0.658	0	0.189	1.140	0.874
1368192_at	Cxcr3	chemokine (C-X-C motif) receptor 3	uniq_isletLongDR_555	0	4.46	4.28	4.12	4.66	0	-0.178	-1.131	0.782	0	-0.336	-1.263	0.344	0	0.197	1.146	0.860
1368207_at	Fxyd5	FXYD domain-containing ion transport regulator 5	uniq_isletLongDR_555	0	6.45	6.42	6.30	6.36	0	-0.035	-1.024	1.093	0	-0.158	-1.115	0.792	0	-0.096	-1.069	1.088
1368251_at	Jak3	Janus kinase 3	uniq_isletLongDR_555	0	5.16	5.07	5.06	5.40	0	-0.081	-1.058	1.049	0	-0.099	-1.071	1.005	0	0.246	1.186	0.821
1368270_at	Apobec1	apolipoprotein B mRNA editing enzyme, catalytic po	uniq_isletLongDR_555	0	3.33	3.26	3.33	3.57	0	-0.067	-1.048	1.098	0	-0.007	-1.005	1.079	0	0.242	1.182	0.818
1368280_at	Ctsc	cathepsin C	uniq_isletLongDR_555	0	5.62	5.85	5.37	5.32	0	0.233	1.175	0.712	0	-0.245	-1.185	0.557	0	-0.299	-1.230	0.487
1368294_at	Dnase1l3	deoxyribonuclease 1-like 3	uniq_isletLongDR_555	0	5.46	5.03	5.38	5.16	0	-0.423	-1.340	0.206	0	-0.075	-1.053	0.956	0	-0.299	-1.231	0.490
1368304_at	Fmo3	flavin containing monooxygenase 3	uniq_isletLongDR_555	0	5.26	5.24	5.35	5.15	0	-0.012	-1.008	1.097	0	0.089	1.064	0.901	0	-0.107	-1.077	1.044
1368308_at	Myc	myelocytomatosis oncogene	uniq_isletLongDR_555	0	5.00	5.01	4.79	4.79	0	0.004	1.003	1.104	0	-0.211	-1.157	0.648	0	-0.218	-1.163	0.771
1368387_at	Bdh1	3-hydroxybutyrate dehydrogenase, type 1	uniq_isletLongDR_555	0	4.96	4.87	5.03	5.64	0	-0.086	-1.061	1.081	0	0.071	1.051	1.023	0	0.679	1.601	0.125
1368482_at	Bcl2a1	BCL2-related protein A1	uniq_isletLongDR_555	0	5.06	4.81	4.93	4.71	0	-0.249	-1.188	0.574	0	-0.130	-1.095	0.877	0	-0.354	-1.278	0.342
1368490_at	Cd14	CD14 molecule	uniq_isletLongDR_555	0	6.94	7.29	7.27	6.68	0	0.351	1.276	0.417	0	0.324	1.252	0.445	0	-0.260	-1.197	0.617
1368492_at	Hpgds	hematopoietic prostaglandin D synthase	uniq_isletLongDR_555	0	5.00	4.71	4.81	5.15	0	-0.295	-1.227	0.482	0	-0.191	-1.142	0.743	0	0.144	1.105	0.964
1368521_at	Napsa	napsin A aspartic peptidase	uniq_isletLongDR_555	0	4.22	3.88	3.91	3.81	0	-0.340	-1.266	0.357	0	-0.313	-1.242	0.423	0	-0.414	-1.333	0.219
1368530_at	Mmp12	matrix metallopeptidase 12	uniq_isletLongDR_555	0	4.69	4.98	4.68	4.67	0	0.286	1.219	0.475	0	-0.009	-1.006	1.017	0	-0.027	-1.019	1.092
1368555_at	Cd37	CD37 molecule	uniq_isletLongDR_555	0	3.68	3.70	3.56	3.71	0	0.018	1.013	1.146	0	-0.113	-1.081	0.981	0	0.032	1.022	1.100
1368563_at	Aspa	aspartoacylase	uniq_isletLongDR_555	0	4.39	4.21	4.14	4.43	0	-0.174	-1.129	0.811	0	-0.245	-1.185	0.599	0	0.044	1.031	1.100
1368605_at	Sh2b2	SH2B adaptor protein 2	uniq_isletLongDR_555	0	3.91	3.90	3.96	4.30	0	-0.015	-1.010	1.111	0	0.050	1.035	0.945	0	0.391	1.311	0.504
1368610_at	Milr1	mast cell immunoglobulin-like receptor 1	uniq_isletLongDR_555	0	3.94	3.96	3.91	4.00	0	0.019	1.014	1.163	0	-0.026	-1.019	1.135	0	0.057	1.041	1.098
1368612_at	Itgb4	integrin, beta 4	uniq_isletLongDR_555	0	5.06	4.67	4.63	4.81	0	-0.390	-1.311	0.277	0	-0.430	-1.347	0.188	0	-0.257	-1.195	0.613
1368655_at	Srgn	serglycin	uniq_isletLongDR_555	0	8.68	8.66	8.68	8.92	0	-0.026	-1.018	1.135	0	-0.006	-1.004	1.112	0	0.242	1.182	0.836
1368674_at	Pygl	phosphorylase, glycogen, liver	uniq_isletLongDR_555	0	5.23	5.19	5.28	5.27	0	-0.042	-1.030	1.110	0	0.048	1.034	1.111	0	0.035	1.025	1.110
1368723_at	Lat	linker for activation of T cells	uniq_isletLongDR_555	0	5.39	5.55	5.17	5.35	0	0.156	1.114	0.783	0	-0.224	-1.168	0.590	0	-0.044	-1.031	1.129
1368762_at	Ubd	ubiquitin D	uniq_isletLongDR_555	0	4.82	4.40	4.79	4.76	0	-0.421	-1.339	0.208	0	-0.032	-1.022	0.975	0	-0.062	-1.044	1.115
1368777_at	Bard1	BRCA1 associated RING domain 1	uniq_isletLongDR_555	0	5.53	5.40	5.33	5.10	0	-0.135	-1.098	0.953	0	-0.198	-1.147	0.726	0	-0.430	-1.348	0.206
1368783_at	Icos	inducible T-cell co-stimulator	uniq_isletLongDR_555	0	3.91	3.83	3.78	3.73	0	-0.081	-1.058	0.935	0	-0.124	-1.090	0.925	0	-0.178	-1.131	0.892
1368906_at	Pgk1	phosphoglycerate kinase 1	uniq_isletLongDR_555	0	2.69	2.66	2.72	2.72	0	-0.031	-1.021	1.140	0	0.032	1.022	1.093	0	0.031	1.022	1.109
1368914_at	Runx1	runt-related transcription factor 1	uniq_isletLongDR_555	0	4.89	4.67	4.61	4.43	0	-0.211	-1.157	0.686	0	-0.278	-1.213	0.484	0	-0.455	-1.370	0.151
1368915_at	Kmo	kynurenine 3-monooxygenase (kynurenine 3-hydro:	uniq_isletLongDR_555	0	3.34	3.21	3.24	3.68	0	-0.125	-1.090	0.900	0	-0.092	-1.066	0.905	0	0.340	1.266	0.602
1368921_a_at	Cd44	Cd44 molecule	uniq_isletLongDR_555	0	5.33	5.18	5.17	5.24	0	-0.155	-1.114	0.852	0	-0.166	-1.122	0.777	0	-0.094	-1.068	1.082
1368930_at	Kcnn4	potassium intermediate/small conductance calcium	uniq_isletLongDR_555	0	3.67	3.35	3.42	3.55	0	-0.325	-1.253	0.420	0	-0.251	-1.190	0.564	0	-0.121	-1.088	0.985
1368941_at	Ptger4	prostaglandin E receptor 4 (subtype EP4)	uniq_isletLongDR_555	0	4.05	3.86	4.02	3.89	0	-0.189	-1.140	0.836	0	-0.026	-1.018	1.112	0	-0.153	-1.112	0.963
1368948_at	Msn	moesin	uniq_isletLongDR_555	0	5.55	5.77	5.21	5.48	0	0.216	1.161	0.763	0	-0.342	-1.268	0.305	0	-0.069	-1.049	1.109
1368975_at	Cd38	CD38 molecule	uniq_isletLongDR_555	0	3.03	2.95	2.99	3.25	0	-0.075	-1.053	1.077	0	-0.037	-1.026	1.085	0	0.226	1.169	0.844
1368976_at	Cd38	CD38 molecule	uniq_isletLongDR_555	0	3.31	3.21	3.22	3.45	0	-0.105	-1.076	1.014	0	-0.087	-1.063	0.997	0	0.137	1.100	0.990
1368983_at	Hbegf	heparin-binding EGF-like growth factor	uniq_isletLongDR_555	0	5.54	5.46	5.52	5.87	0	-0.074	-1.052	1.058	0	-0.014	-1.010	1.123	0	0.329	1.256	0.635
1368990_at	Cyp1b1	cytochrome P450, family 1, subfamily b, polypeptid	uniq_isletLongDR_555	0	4.83	4.59	4.70	4.99	0	-0.247	-1.187	0.671	0	-0.136	-1.099	0.921	0	0.153	1.112	0.980
1369025_at	Cd5 /// LOC10	Cd5 molecule /// T-cell surface glycoprotein CD5-lik	uniq_isletLongDR_555	0	4.83	4.80	5.14	4.90	0	-0.028	-1.020	1.142	0	0.312	1.242	0.460	0	0.071	1.050	1.081
1369093_at	Reln	reelin	uniq_isletLongDR_555	0	3.47	3.22	3.10	3.31	0	-0.249	-1.189	0.663	0	-0.374	-1.296	0.288	0	-0.158	-1.116	0.945
1369110_x_at	RT1-EC2	RT1 class Ib, locus EC2	uniq_isletLongDR_555	0	6.32	5.88	6.07	6.15	0	-0.447	-1.364	0.154	0	-0.254	-1.192	0.565	0	-0.173	-1.127	0.880
1369129_at	Rasgrp1	RAS guanyl releasing protein 1 (calcium and DAG-re	uniq_isletLongDR_555	0	3.06	3.12	2.96	3.09	0	0.055	1.039	1.115	0	-0.101	-1.073	0.921	0	0.025	1.017	1.110
1369130_at	Rasgrp1	RAS guanyl releasing protein 1 (calcium and DAG-re	uniq_isletLongDR_555	0	4.24	3.94	4.07	4.53	0	-0.297	-1.229	0.507	0	-0.170	-1.125	0.687	0	0.289	1.222	0.722
1369181_at	Cybb	cytochrome b-245, beta polypeptide	uniq_isletLongDR_555	0	4.21	4.13	4.14	4.20	0	-0.085	-1.061	0.971	0	-0.072	-1.051	1.046	0	-0.011	-1.008	1.177
1369186_at	Casp1	caspase 1	uniq_isletLongDR_555	0	5.73	5.66	5.71	5.81	0	-0.075	-1.053	1.067	0	-0.027	-1.019	1.098	0	0.076	1.054	1.079
1369190_at	Cd2	Cd2 molecule	uniq_isletLongDR_555	0	4.84	4.75	4.59	4.67	0	-0.084	-1.060	1.018	0	-0.247	-1.187	0.551	0	-0.162	-1.119	0.916
1369204_at	Hck	hemopoietic cell kinase	uniq_isletLongDR_555	0	4.51	4.37	4.22	4.20	0	-0.142	-1.103	0.936	0	-0.290	-1.223	0.470	0	-0.310	-1.239	0.467
1369214_a_at	Ciita	class II, major histocompatibility complex, transactiv	uniq_isletLongDR_555	0	6.00	5.72	5.81	6.14	0	-0.279	-1.214	0.595	0	-0.193	-1.143	0.741	0	0.145	1.106	0.999
1369262_at	Casp8	caspase 8	uniq_isletLongDR_555	0	5.53	5.71	5.53	5.49	0	0.177	1.130	0.893	0	-0.004	-1.003	1.115	0	-0.038	-1.027	1.160
1369281_at	Tnfsf11	tumor necrosis factor (ligand) superfamily, member	uniq_isletLongDR_555	0	3.35	3.26	3.27	3.40	0	-0.091	-1.065	1.072	0	-0.080	-1.057	1.039	0	0.052	1.036	1.094
1369282_at	Tnfsf11	tumor necrosis factor (ligand) superfamily, member	uniq_isletLongDR_555	0	3.03	2.96	2.93	2.96	0	-0.071	-1.050	1.110	0	-0.100	-1.072	0.999	0	-0.072	-1.051	1.138
1369294_at	Bst1	bone marrow stromal cell antigen 1	uniq_isletLongDR_555	0	5.46	5.34	5.39	5.49	0	-0.125	-1.090	1.004	0	-0.076	-1.054	1.047	0	0.029	1.020	1.083
1369306_at	Klrd1	killer cell lectin-like receptor, subfamily D, member 1	uniq_isletLongDR_555	0	2.95	3.00	2.92	3.17	0	0.048	1.034	1.079	0	-0.030	-1.021	1.136	0	0.225	1.169	0.859
1369387_at	Vav1	vav 1 guanine nucleotide exchange factor	uniq_isletLongDR_555	0	4.33	4.05	4.32	4.22	0	-0.283	-1.217	0.569	0	-0.012	-1.009	1.149	0	-0.113	-1.082	1.066
1369393_at	Map3k8	mitogen-activated protein kinase kinase kinase 8	uniq_isletLongDR_555	0	4.66	5.15	5.06	4.78	0	0.493	1.407	0.259	0	0.403	1.322	0.295	0	0.120	1.087	0.994
1369519_at	Edn1	endothelin 1	uniq_isletLongDR_555	0	4.98	4.63	4.54	5.05	0	-0.352	-1.276	0.357	0	-0.437	-1.354	0.175	0	0.071	1.050	1.042
1369552_at	Samsn1	SAM domain, SH3 domain and nuclear localization s	uniq_isletLongDR_555	0	4.39	4.26	4.21	4.21	0	-0.125	-1.090	0.880	0	-0.178	-1.131	0.676	0	-0.175	-1.129	0.902
1369555_at	Ccr4	chemokine (C-C motif) receptor 4	uniq_isletLongDR_555	0	3.97	3.64	3.75	3.95	0	-0.333	-1.260	0.280	0	-0.222	-1.166	0.612	0	-0.020	-1.014	1.147
1369584_at	Socs3	suppressor of cytokine signaling 3	uniq_isletLongDR_555	0	5.20	5.42	5.03	5.76	0	0.221	1.166	0.728	0	-0.173	-1.128	0.811	0	0.555	1.469	0.230
1369683_at	Bid	BH3 interacting domain death agonist	uniq_isletLongDR_555	0	4.07	3.79	3.66	4.08	0	-0.287	-1.220	0.481	0	-0.414	-1.332	0.191	0	0.005	1.003	1.177
1369791_at	Clcn4	chloride channel, voltage-sensitive 4	uniq_isletLongDR_555	0	4.76	4.42	4.58	4.60	0	-0.338	-1.264	0.424	0	-0.180	-1.133	0.819	0	-0.155	-1.114	0.970
1369801_at	Sell	selectin L	uniq_isletLongDR_555	0	4.91	4.63	4.61	4.92	0	-0.286	-1.219	0.545	0	-0.303	-1.233	0.415	0	0.011	1.008	1.170
1369814_at	Ccl20	chemokine (C-C motif) ligand 20	uniq_isletLongDR_555	0	4.56	4.53	4.47	4.55	0	-0.032	-1.022	1.063	0	-0.089	-1.064	0.730	0	-0.010	-1.007	1.120
1369815_at	Ccl3	chemokine (C-C motif) ligand 3	uniq_isletLongDR_555	0	5.27	5.25	5.29	5.80	0	-0.013	-1.009	1.044	0	0.022	1.015	0.948	0	0.533	1.447	0.316
1369872_a_at	Fcer2	Fc fragment of IgE, low affinity II, receptor for (CD23	uniq_isletLongDR_555	0	3.87	3.39	3.44	3.66	0	-0.484	-1.399	0.109	0	-0.436	-1.353	0.148	0	-0.208	-1.155	0.793
1369877_at	Cd8a	CD8a molecule	uniq_isletLongDR_555	0	3.72	3.59	3.73	3.95	0	-0.125	-1.090	0.987	0	0.008	1.005	1.134	0	0.234	1.176	0.805
1369911_at	Cxcr5	chemokine (C-X-C motif) receptor 5	uniq_isletLongDR_555	0	4.09	3.98	3.95	4.12	0	-0.102	-1.074	1.069	0	-0.131	-1.095	0.891	0	0.035	1.024	1.074
1369979_at	Skap2	src kinase associated phosphoprotein 2	uniq_isletLongDR_555	0	7.23	7.19	7.35	7.33	0	-0.050	-1.035	1.098	0	0.115	1.083	0.977	0	0.095	1.068	0.998
1370033_at	Myl1	myosin, light chain 1	uniq_isletLongDR_555	0	3.66	3.59	3.44	3.49	0	-0.066	-1.047	1.038	0	-0.216	-1.162	0.630	0	-0.163	-1.120	0.885
1370059_at	Nefl	neurofilament, light polypeptide	uniq_isletLongDR_555	0	3.61	3.40	3.14	3.47	0	-0.206	-1.153	0.772	0	-0.465	-1.381	0.095	0	-0.139	-1.101	0.992
1370080_at	Hmox1	heme oxygenase (decycling) 1	uniq_isletLongDR_555	0	5.40	5.12	5.04	4.96	0	-0.285	-1.218	0.533	0	-0.358	-1.282	0.324	0	-0.445	-1.361	0.164
1370096_at	Prf1	perforin 1 (pore forming protein)	uniq_isletLongDR_555	0	5.27	5.21	5.20	5.86	0	-0.064	-1.045	1.050	0	-0.076	-1.054	0.952	0	0.590	1.505	0.201
1370104_at	Pde6h	phosphodiesterase 6H, cGMP-specific, cone, gamma	uniq_isletLongDR_555	0	4.66	4.40	4.28	4.47	0	-0.267	-1.203	0.579	0	-0.382	-1.303	0.258	0	-0.193	-1.143	0.831
1370118_at	Ccl17	chemokine (C-C motif) ligand 17	uniq_isletLongDR_555	0	6.40	6.19	6.13	6.52	0	-0.215	-1.160	0.674	0	-0.265	-1.202	0.499	0	0.120	1.087	1.020
1370127_at	Pold1	polymerase (DNA directed), delta 1, catalytic subuni	uniq_isletLongDR_555	0	5.70	5.58	5.68	5.65	0	-0.113	-1.081	0.962	0	-0.011	-1.008	1.088	0	-0.043	-1.030	1.123
1370133_at	Rgs19	regulator of G-protein signaling 19	uniq_isletLongDR_555	0	5.49	5.54	5.36	5.36	0	0.050	1.036	1.062	0	-0.132	-1.096	0.796	0	-0.137	-1.100	0.994
1370136_at	Lbr	lamin B receptor	uniq_isletLongDR_555	0	5.37	5.36	5.12	5.31	0	-0.016	-1.011	1.063	0	-0.252	-1.191	0.508	0	-0.068	-1.048	1.066
1370138_at	Lef1	lymphoid enhancer binding factor 1	uniq_isletLongDR_555	0	3.49	3.49	3.28	3.58	0	0.004	1.003	1.117	0	-0.212	-1.158	0.674	0	0.093	1.067	1.069
1370174_at	Ppp1r15a	protein phosphatase 1, regulatory subunit 15A	uniq_isletLongDR_555	0	8.13	8.47	7.88	8.02	0	0.337	1.263	0.476	0	-0.253	-1.192	0.521	0	-0.111	-1.080	0.993
1370186_at	Psmb9	proteasome (prosome, macropain) subunit, beta typ	uniq_isletLongDR_555	0	7.29	7.48	7.65	7.05	0	0.197	1.146	0.798	0	0.365	1.288	0.365	0	-0.239	-1.180	0.691
1370202_at	Pla2g16	phospholipase A2, group XVI	uniq_isletLongDR_555	0	6.18	6.08	6.07	5.85	0	-0.098	-1.071	0.985	0	-0.116	-1.084	0.839	0	-0.330	-1.257	0.412
1370215_at	C1qb	complement component 1, q subcomponent, B chai	uniq_isletLongDR_555	0	5.66	5.64	5.69	5.87	0	-0.017	-1.012	1.097	0	0.028	1.020	1.067	0	0.209	1.156	0.867
1370219_at	Cyba	cytochrome b-245, alpha polypeptide	uniq_isletLongDR_555	0	6.72	6.67	6.65	6.59	0	-0.051	-1.036	1.122	0	-0.071	-1.051	0.996	0	-0.137	-1.099	0.990
1370249_at	Tspo	translocator protein	uniq_isletLongDR_555	0	6.92	6.53	6.55	6.74	0	-0.390	-1.310	0.281	0	-0.368	-1.291	0.294	0	-0.179	-1.132	0.895
1370355_at	Scd1	stearoyl-Coenzyme A desaturase 1	uniq_isletLongDR_555	0	4.04	3.57	3.86	3.96	0	-0.466	-1.381	0.137	0	-0.175	-1.129	0.810	0	-0.074	-1.053	1.076
1370371_a_at	Ceacam1 ///	carcinoembryonic antigen-related cell adhesion mol	uniq_isletLongDR_555	0	3.30	3.20	3.05	3.45	0	-0.100	-1.072	1.013	0	-0.254	-1.193	0.518	0	0.148	1.108	0.962
1370382_at	RT1-Db1	RT1 class II, locus Db1	uniq_isletLongDR_555	0	2.78	2.71	2.71	2.74	0	-0.076	-1.054	1.072	0	-0.077	-1.055	1.006	0	-0.040	-1.028	1.161
1370383_s_at	RT1-Db1	RT1 class II, locus Db1	uniq_isletLongDR_555	0	7.07	6.95	7.17	7.09	0	-0.127	-1.092	0.885	0	0.099	1.071	0.952	0	0.014	1.010	1.177
1370391_at	Crabp2 /// LC	cellular retinoic acid binding protein 2 /// cellular re	uniq_isletLongDR_555	0	4.70	4.44	4.45	4.40	0	-0.253	-1.192	0.595	0	-0.245	-1.185	0.566	0	-0.299	-1.231	0.474

Affymetrix Probeset ID	Gene Symbol	Gene Title	Figure 7 A Venn n=2289	Present in any 2 analyses n=423 1=yes 0=no	Mean log2 intensity DR+/+ ND	Mean log2 intensity DR+/+ HCD	Mean log2 intensity DR+/+ B/S	Mean log2 intensity F+/+ day 40 ND	DR+/+HCD vs DR+/+ND FDR<10% Log2 ratio >0.5 n=636	Log2 ratio DR+/+HCD vs DR+/+ ND	Fold-Change DR+/+H CD vs DR+/+ ND	FDR DR+/+H CD vs DR+/+ ND	DR+/+B/S vs DR+/+ND Log2 ratio >0.5 n=350	Log2 ratio DR+/+ B/S vs DR+/+ ND	Fold-Change DR+/+B /S vs DR+/+ ND	FDR DR+/+B /S vs DR+/+ ND	F+/+ vs DR+/+N D Log2 ratio >0.5 n=1209	Log2 ratio F+/+ vs DR+/+ ND	Fold-Change F+/+ vs DR+/+ ND	FDR F+/+ vs DR+/+ ND
1370394_at	IgG-2a	gamma-2a immunoglobulin heavy chain	uniq_isletLongDR_555	0	4.95	4.58	4.58	4.53	0	-0.365	-1.288	0.352	0	-0.365	-1.288	0.297	0	-0.416	-1.334	0.216
1370406_a_at	Cd55	Cd55 molecule	uniq_isletLongDR_555	0	4.00	4.09	3.80	3.85	0	0.085	1.061	1.093	0	-0.203	-1.151	0.726	0	-0.151	-1.111	0.959
1370419_a_at	Sh3kbp1	SH3-domain kinase binding protein 1	uniq_isletLongDR_555	0	6.53	6.40	6.50	6.45	0	-0.132	-1.096	0.819	0	-0.032	-1.022	0.988	0	-0.082	-1.059	1.089
1370422_at	Ripk3	receptor-interacting serine-threonine kinase 3	uniq_isletLongDR_555	0	4.65	4.38	4.31	4.45	0	-0.270	-1.206	0.529	0	-0.348	-1.273	0.342	0	-0.203	-1.151	0.815
1370428_x_at	RT1-A2 /// RT	RT1 class Ia, locus A2 /// RT1 class I, locus A3 /// RT1	uniq_isletLongDR_555	0	9.29	9.40	9.15	9.43	0	0.109	1.079	1.022	0	-0.135	-1.098	0.828	0	0.145	1.106	0.986
1370429_at	RT1-EC2	RT1 class Ib, locus EC2	uniq_isletLongDR_555	0	6.33	6.68	6.54	6.77	0	0.349	1.273	0.468	0	0.210	1.157	0.696	0	0.432	1.349	0.436
1370469_at	Ptpn7	protein tyrosine phosphatase, non-receptor type 7	uniq_isletLongDR_555	0	5.65	5.64	5.77	5.89	0	-0.014	-1.010	1.133	0	0.120	1.087	0.876	0	0.233	1.175	0.808
1370516_at	Slc15a3	solute carrier family 15, member 3	uniq_isletLongDR_555	0	4.64	4.68	4.70	4.71	0	0.036	1.025	1.010	0	0.058	1.041	0.979	0	0.063	1.044	1.055
1370544_at	Eml2	echinoderm microtubule associated protein like 2	uniq_isletLongDR_555	0	6.22	6.43	6.21	6.21	0	0.208	1.155	0.788	0	-0.011	-1.008	1.053	0	-0.013	-1.009	1.168
1370621_at	Cd247	Cd247 molecule	uniq_isletLongDR_555	0	4.66	4.68	4.63	4.69	0	0.014	1.010	1.114	0	-0.036	-1.025	1.033	0	0.027	1.019	1.081
1370708_a_at	Akr1c14	aldo-keto reductase family 1, member C14	uniq_isletLongDR_555	0	3.36	3.16	3.13	3.30	0	-0.197	-1.146	0.794	0	-0.222	-1.167	0.636	0	-0.055	-1.039	1.154
1370769_a_at	Icos	inducible T-cell co-stimulator	uniq_isletLongDR_555	0	3.08	3.04	3.12	3.18	0	-0.041	-1.028	1.084	0	0.040	1.028	1.024	0	0.097	1.069	1.070
1370822_at	RT1-Ba	RT1 class II, locus Ba	uniq_isletLongDR_555	0	7.51	7.46	7.60	7.26	0	-0.051	-1.036	1.085	0	0.084	1.060	0.996	0	-0.253	-1.192	0.631
1370826_at	Nap1l1	nucleosome assembly protein 1-like 1	uniq_isletLongDR_555	0	8.69	8.97	8.64	8.74	0	0.271	1.207	0.617	0	-0.049	-1.035	1.050	0	0.042	1.030	1.105
1370875_at	Ezr	ezrin	uniq_isletLongDR_555	0	7.58	7.50	7.49	7.32	0	-0.080	-1.057	1.054	0	-0.080	-1.057	0.996	0	-0.255	-1.194	0.631
1370885_at	Ctsz	cathepsin Z	uniq_isletLongDR_555	0	6.97	7.14	7.08	6.56	0	0.162	1.119	0.906	0	0.105	1.076	0.976	0	-0.412	-1.331	0.245
1370904_at	LOC10090959	class II histocompatibility antigen, M alpha chain-lik	uniq_isletLongDR_555	0	5.79	5.77	5.85	5.88	0	-0.017	-1.012	1.031	0	0.063	1.044	0.949	0	0.091	1.065	1.002
1370924_at	Tcrb	T-cell receptor beta chain	uniq_isletLongDR_555	0	5.85	5.64	5.39	5.64	0	-0.208	-1.155	0.715	0	-0.455	-1.370	0.120	0	-0.204	-1.152	0.696
1370963_at	Gas7	growth arrest specific 7	uniq_isletLongDR_555	0	4.71	4.30	4.43	4.26	0	-0.413	-1.332	0.224	0	-0.286	-1.219	0.474	0	-0.454	-1.370	0.159
1370987_at	Spn	sialophorin	uniq_isletLongDR_555	0	5.11	5.24	5.01	5.15	0	0.131	1.095	0.911	0	-0.101	-1.073	0.988	0	0.041	1.029	1.100
1370990_at	Crlf3	cytokine receptor-like factor 3	uniq_isletLongDR_555	0	7.40	7.53	7.44	7.82	0	0.139	1.101	0.942	0	0.043	1.030	1.106	0	0.425	1.342	0.449
1371015_at	Mx1	myxovirus (influenza virus) resistance 1	uniq_isletLongDR_555	0	3.63	3.86	3.77	3.61	0	0.233	1.175	0.723	0	0.139	1.102	0.842	0	-0.020	-1.014	1.131
1371016_at	LOC290071	similar to RIKEN cDNA A430107P09 gene	uniq_isletLongDR_555	0	4.26	4.31	3.77	3.89	0	0.042	1.030	1.063	0	-0.494	-1.409	0.093	0	-0.373	-1.295	0.303
1371040_at	Slc1a5	solute carrier family 1 (neutral amino acid transport	uniq_isletLongDR_555	0	6.23	6.19	6.14	6.48	0	-0.037	-1.026	1.117	0	-0.094	-1.067	0.971	0	0.248	1.187	0.800
1371078_at	LOC1003649	MHC class Ib alpha chain-like /// similar to RT1 class	uniq_isletLongDR_555	0	5.34	5.08	5.17	5.31	0	-0.265	-1.202	0.614	0	-0.175	-1.129	0.822	0	-0.036	-1.025	1.160
1371089_at	Gsta5	glutathione S-transferase Yc2 subunit	uniq_isletLongDR_555	0	3.11	2.89	2.96	2.92	0	-0.222	-1.166	0.739	0	-0.154	-1.113	0.813	0	-0.189	-1.140	0.849
1371111_at	RT1-EC2	RT1 class Ib, locus EC2	uniq_isletLongDR_555	0	6.88	6.76	6.94	6.87	0	-0.126	-1.091	0.998	0	0.057	1.040	1.089	0	-0.013	-1.009	1.176
1371115_at	Ptpre	protein tyrosine phosphatase, receptor type, E	uniq_isletLongDR_555	0	4.48	4.52	4.32	4.34	0	0.041	1.029	1.111	0	-0.157	-1.115	0.860	0	-0.133	-1.097	0.936
1371123_x_at	RT1-S3	RT1 class Ib, locus S3	uniq_isletLongDR_555	0	6.60	6.87	6.80	6.79	0	0.274	1.209	0.547	0	0.207	1.154	0.707	0	0.192	1.142	0.928
1371171_at	RT1-EC2	RT1 class Ib, locus EC2	uniq_isletLongDR_555	0	4.71	4.42	4.51	4.62	0	-0.290	-1.222	0.447	0	-0.205	-1.152	0.665	0	-0.089	-1.064	1.047
1371210_s_at	RT1-CE5 /// R	RT1 class I, locus CE5 /// RT1 class Ib, locus EC2	uniq_isletLongDR_555	0	3.72	3.78	3.79	3.97	0	0.065	1.046	1.113	0	0.079	1.056	1.064	0	0.249	1.188	0.818
1371256_at	Ptpn18	protein tyrosine phosphatase, non-receptor type 18	uniq_isletLongDR_555	0	5.84	5.66	5.66	5.56	0	-0.189	-1.140	0.778	0	-0.189	-1.140	0.784	0	-0.284	-1.218	0.540
1371262_at	IgG-2a /// Igh	gamma-2a immunoglobulin heavy chain /// immuno	uniq_isletLongDR_555	0	5.40	5.30	5.15	5.03	0	-0.095	-1.068	0.903	0	-0.243	-1.184	0.534	0	-0.363	-1.286	0.323
1371336_at	Hn1	hematological and neurological expressed 1	uniq_isletLongDR_555	0	7.23	7.41	7.24	7.34	0	0.188	1.139	0.824	0	0.012	1.009	1.146	0	0.113	1.082	1.052
1371414_at	Gsn	gelsolin	uniq_isletLongDR_555	0	9.11	8.98	8.87	8.62	0	-0.126	-1.091	0.922	0	-0.235	-1.177	0.632	0	-0.485	-1.400	0.114
1371566_at	Fbxl22	F-box and leucine-rich repeat protein 22	uniq_isletLongDR_555	0	3.73	3.67	3.43	3.50	0	-0.064	-1.046	1.072	0	-0.300	-1.231	0.431	0	-0.233	-1.175	0.665
1371774_at	Sat1	spermidine/spermine N1-acetyl transferase 1	uniq_isletLongDR_555	0	9.40	9.73	9.15	9.34	0	0.336	1.262	0.474	0	-0.251	-1.190	0.528	0	-0.058	-1.041	1.124
1371872_at	---	---	uniq_isletLongDR_555	0	9.56	9.80	9.76	9.42	0	0.244	1.184	0.669	0	0.202	1.150	0.756	0	-0.141	-1.102	1.006
1372070_at	Ifi30	interferon gamma inducible protein 30	uniq_isletLongDR_555	0	9.38	9.41	9.43	9.28	0	0.027	1.019	1.158	0	0.051	1.036	1.116	0	-0.102	-1.073	1.092
1372133_at	Rras2	related RAS viral (r-ras) oncogene homolog 2	uniq_isletLongDR_555	0	7.23	7.24	7.29	6.78	0	0.011	1.008	1.158	0	0.059	1.042	1.061	0	-0.444	-1.361	0.174
1372135_at	LOC684352	similar to twinfilin-like protein	uniq_isletLongDR_555	0	5.94	5.66	5.98	6.06	0	-0.281	-1.215	0.544	0	0.041	1.029	1.096	0	0.122	1.089	1.007
1372161_at	LOC685612	similar to Emu2	uniq_isletLongDR_555	0	5.34	5.17	5.23	5.67	0	-0.170	-1.125	0.757	0	-0.107	-1.077	0.891	0	0.332	1.258	0.576
1372182_at	Pfkp	phosphofructokinase, platelet	uniq_isletLongDR_555	0	6.90	7.21	7.08	6.62	0	0.313	1.243	0.515	0	0.180	1.133	0.808	0	-0.281	-1.215	0.550
1372200_at	Gpsm3	G-protein signaling modulator 3	uniq_isletLongDR_555	0	5.68	5.62	5.61	5.74	0	-0.066	-1.047	1.095	0	-0.073	-1.052	1.037	0	0.057	1.040	1.093
1372246_at	Ostf1	osteoclast stimulating factor 1	uniq_isletLongDR_555	0	8.14	8.21	8.22	8.10	0	0.068	1.048	1.101	0	0.078	1.056	1.074	0	-0.046	-1.032	1.161
1372303_at	Fam49b	family with sequence similarity 49, member B	uniq_isletLongDR_555	0	8.55	8.81	8.59	8.57	0	0.251	1.190	0.674	0	0.038	1.027	1.120	0	0.016	1.011	1.115
1372408_at	Gga2	golgi associated, gamma adaptin ear containing, ARI	uniq_isletLongDR_555	0	8.72	8.91	8.92	8.62	0	0.192	1.142	0.824	0	0.197	1.146	0.786	0	-0.101	-1.072	1.094
1372531_at	Ppfibp2	PTPRF interacting protein, binding protein 2 (liprin b	uniq_isletLongDR_555	0	4.72	4.46	4.51	4.74	0	-0.260	-1.197	0.512	0	-0.205	-1.153	0.746	0	0.025	1.017	1.101
1372725_at	Plscr2	phospholipid scramblase 2	uniq_isletLongDR_555	0	4.36	4.49	4.12	4.24	0	0.130	1.095	0.959	0	-0.240	-1.181	0.572	0	-0.119	-1.086	0.944
1372770_at	Sri	sorcin	uniq_isletLongDR_555	0	8.08	8.45	8.19	8.04	0	0.366	1.289	0.421	0	0.103	1.074	0.946	0	-0.045	-1.032	1.140
1372846_at	Cyb561a3	cytochrome b561 family, member A3	uniq_isletLongDR_555	0	7.34	7.40	7.62	7.40	0	0.064	1.045	1.067	0	0.280	1.214	0.525	0	0.066	1.047	1.085
1372852_at	Ptprcap	protein tyrosine phosphatase, receptor type, C-asso	uniq_isletLongDR_555	0	5.87	5.60	5.59	5.38	0	-0.267	-1.204	0.509	0	-0.275	-1.210	0.459	0	-0.491	-1.405	0.129
1372904_at	Mob3a	MOB kinase activator 3A	uniq_isletLongDR_555	0	6.30	6.23	6.24	5.96	0	-0.066	-1.047	1.079	0	-0.055	-1.039	1.052	0	-0.338	-1.264	0.371
1372930_at	Sp110	SP110 nuclear body protein	uniq_isletLongDR_555	0	8.22	8.20	8.33	8.11	0	-0.026	-1.018	1.152	0	0.106	1.076	1.027	0	-0.108	-1.078	1.085
1372935_at	Tmem119	transmembrane protein 119	uniq_isletLongDR_555	0	4.67	4.54	4.57	4.70	0	-0.129	-1.093	0.989	0	-0.098	-1.071	1.001	0	0.023	1.016	1.079
1373025_at	C1qc	complement component 1, q subcomponent, C chai	uniq_isletLongDR_555	0	6.42	6.21	6.40	6.01	0	-0.209	-1.156	0.772	0	-0.017	-1.012	1.143	0	-0.409	-1.328	0.227
1373065_at	Ptpn18	protein tyrosine phosphatase, non-receptor type 18	uniq_isletLongDR_555	0	7.29	7.29	7.29	6.89	0	-0.005	-1.003	1.159	0	0.003	1.002	1.132	0	-0.400	-1.320	0.237
1373175_at	Plbd1	phospholipase B domain containing 1	uniq_isletLongDR_555	0	4.47	4.20	4.20	4.46	0	-0.271	-1.206	0.515	0	-0.270	-1.206	0.479	0	-0.011	-1.008	1.140
1373252_at	LOC300225	similar to formin-like 3 protein	uniq_isletLongDR_555	0	7.02	6.92	7.06	6.98	0	-0.097	-1.069	1.041	0	0.043	1.030	0.981	0	-0.037	-1.026	1.154
1373290_at	Ezh2	enhancer of zeste homolog 2 (Drosophila)	uniq_isletLongDR_555	0	5.40	5.44	5.51	5.49	0	0.040	1.028	1.132	0	0.102	1.073	0.968	0	0.089	1.064	0.970
1373421_at	Tgif1	TGFB-induced factor homeobox 1	uniq_isletLongDR_555	0	5.66	5.74	5.67	5.76	0	0.086	1.061	1.069	0	0.017	1.012	1.129	0	0.104	1.075	1.052
1373473_a_at	Nap1l1	nucleosome assembly protein 1-like 1	uniq_isletLongDR_555	0	7.77	8.40	8.01	8.15	0	0.628	1.545	0.104	0	0.236	1.177	0.613	0	0.376	1.298	0.543
1373485_at	---	---	uniq_isletLongDR_555	0	4.66	4.27	4.33	4.16	0	-0.387	-1.308	0.249	0	-0.333	-1.260	0.351	0	-0.504	-1.418	0.122
1373528_at	Pik3cd	phosphatidylinositol-4,5-bisphosphate 3-kinase, cat	uniq_isletLongDR_555	0	6.29	6.13	6.18	6.52	0	-0.168	-1.124	0.910	0	-0.118	-1.086	0.969	0	0.231	1.173	0.837
1373530_at	Ccne1	cyclin E1	uniq_isletLongDR_555	0	5.52	5.18	5.23	5.15	0	-0.339	-1.265	0.415	0	-0.294	-1.226	0.454	0	-0.373	-1.295	0.287
1373557_at	Mcm4	minichromosome maintenance complex componen	uniq_isletLongDR_555	0	7.39	7.57	7.62	7.48	0	0.187	1.139	0.829	0	0.234	1.176	0.678	0	0.092	1.066	1.062
1373666_at	Rapgef5	Rap guanine nucleotide exchange factor (GEF) 5	uniq_isletLongDR_555	0	6.30	6.22	6.28	5.99	0	-0.073	-1.052	1.009	0	-0.013	-1.009	1.088	0	-0.304	-1.234	0.475
1373785_at	Pld4	phospholipase D family, member 4	uniq_isletLongDR_555	0	4.05	3.79	4.07	3.98	0	-0.265	-1.202	0.630	0	0.016	1.011	1.144	0	-0.075	-1.053	1.126
1373881_at	Arhgdib	Rho, GDP dissociation inhibitor (GDI) beta	uniq_isletLongDR_555	0	7.47	7.18	7.15	7.27	0	-0.288	-1.221	0.511	0	-0.318	-1.247	0.424	0	-0.199	-1.148	0.837
1373903_at	Rcsd1	RCSD domain containing 1	uniq_isletLongDR_555	0	5.88	5.73	5.67	5.98	0	-0.154	-1.113	0.890	0	-0.208	-1.155	0.746	0	0.099	1.071	1.066
1374055_at	Erf	Ets2 repressor factor	uniq_isletLongDR_555	0	7.57	7.85	7.74	7.32	0	0.284	1.217	0.554	0	0.172	1.126	0.769	0	-0.251	-1.190	0.648
1374101_at	Tbc1d10a	TBC1 domain family, member 10a	uniq_isletLongDR_555	0	6.32	6.18	6.22	6.16	0	-0.137	-1.100	0.776	0	-0.096	-1.069	0.912	0	-0.160	-1.117	0.944
1374113_at	Baz1a	bromodomain adjacent to zinc finger domain, 1A	uniq_isletLongDR_555	0	6.22	6.30	6.21	6.29	0	0.088	1.063	1.068	0	-0.003	-1.002	1.078	0	0.076	1.054	1.066
1374237_at	Lmod1	leiomodin 1 (smooth muscle)	uniq_isletLongDR_555	0	5.06	4.68	4.73	4.65	0	-0.384	-1.305	0.226	0	-0.336	-1.262	0.369	0	-0.413	-1.331	0.245
1374293_at	H2afj /// LOC	H2A histone family, member J /// histone H2A.J-like	uniq_isletLongDR_555	0	8.21	8.13	8.31	8.08	0	-0.084	-1.060	1.094	0	0.097	1.069	1.015	0	-0.138	-1.100	1.019
1374540_at	Cdca7	cell division cycle associated 7	uniq_isletLongDR_555	0	5.15	4.96	4.94	5.01	0	-0.196	-1.146	0.768	0	-0.209	-1.156	0.690	0	-0.146	-1.106	0.942
1374544_at	MGC112715	hypothetical protein LOC690899	uniq_isletLongDR_555	0	5.33	5.40	5.18	5.15	0	0.071	1.050	1.083	0	-0.151	-1.110	0.838	0	-0.183	-1.135	0.880
1374649_at	Rasgrp2	RAS guanyl releasing protein 2 (calcium and DAG-re	uniq_isletLongDR_555	0	5.16	5.22	5.16	5.30	0	0.054	1.038	1.085	0	-0.002	-1.002	1.141	0	0.139	1.101	0.971
1374735_at	Arhgap4	Rho GTPase activating protein 4	uniq_isletLongDR_555	0	6.08	6.11	6.09	6.30	0	0.034	1.024	1.147	0	0.016	1.011	1.093	0	0.221	1.166	0.858
1374816_at	Gcom1	GRINL1A complex locus 1	uniq_isletLongDR_555	0	5.84	5.42	5.61	5.93	0	-0.423	-1.340	0.181	0	-0.231	-1.173	0.565	0	0.094	1.067	1.052
1374830_at	Ccdc3	coiled-coil domain containing 3	uniq_isletLongDR_555	0	5.75	5.38	5.38	5.54	0	-0.378	-1.300	0.290	0	-0.373	-1.295	0.285	0	-0.215	-1.160	0.727
1374838_at	Sp140	SP140 nuclear body protein	uniq_isletLongDR_555	0	7.13	7.01	7.04	7.01	0	-0.120	-1.086	1.002	0	-0.091	-1.065	0.999	0	-0.116	-1.084	1.038
1374872_at	Rasgrp2	RAS guanyl releasing protein 2 (calcium and DAG-re	uniq_isletLongDR_555	0	4.07	3.80	3.89	3.69	0	-0.272	-1.207	0.578	0	-0.177	-1.130	0.791	0	-0.382	-1.303	0.266
1374943_at	Fam210b	family with sequence similarity 210, member B	uniq_isletLongDR_555	0	7.14	7.07	7.18	7.13	0	-0.065	-1.046	1.117	0	0.037	1.026	1.093	0	-0.012	-1.008	1.171
1375015_at	LOC688280	similar to transmembrane channel-like protein 6 iso	uniq_isletLongDR_555	0	6.04	5.79	5.79	5.77	0	-0.257	-1.195	0.588	0	-0.256	-1.194	0.565	0	-0.270	-1.206	0.590
1375367_at	Pdlim2	PDZ and LIM domain 2	uniq_isletLongDR_555	0	5.22	5.00	5.06	5.14	0	-0.226	-1.169	0.635	0	-0.167	-1.123	0.830	0	-0.083	-1.059	1.128
1375686_at	Ppil3	peptidylprolyl isomerase (cyclophilin)-like 3	uniq_isletLongDR_555	0	6.81	6.77	6.86	6.73	0	-0.040	-1.028	1.134	0	0.046	1.033	1.054	0	-0.085	-1.061	1.103
1375709_at	Sipa1	signal-induced proliferation-associated 1	uniq_isletLongDR_555	0	6.52	6.34	6.37	6.46	0	-0.180	-1.133	0.876	0	-0.154	-1.113	0.871	0	-0.061	-1.043	1.149
1375784_at	Egr4	early growth response 4	uniq_isletLongDR_555	0	5.54	5.32	5.21	5.44	0	-0.213	-1.159	0.793	0	-0.323	-1.251	0.415	0	-0.092	-1.066	1.112
1375908_at	Mpzl2	myelin protein zero-like 2	uniq_isletLongDR_555	0	4.94	4.66	4.68	4.87	0	-0.275	-1.210	0.561	0	-0.259	-1.197	0.540	0	-0.072	-1.051	1.111
1375983_at	Mamdc2	MAM domain containing 2	uniq_isletLongDR_555	0	4.61	4.56	4.45	4.21	0	-0.052	-1.037	1.110	0	-0.154	-1.113	0.836	0	-0.393	-1.313	0.246
1375989_a_at	Nfkb2	nuclear factor of kappa light polypeptide gene enha	uniq_isletLongDR_555	0	5.40	5.22	5.31	5.52	0	-0.183	-1.135	0.817	0	-0.097	-1.069	0.960	0	0.119	1.086	1.029
1375996_at	Sept1	septin 1	uniq_isletLongDR_555	0	6.71	6.96	6.82	6.42	0	0.250	1.189	0.634	0	0.108	1.078	0.964	0	-0.290	-1.223	0.523
1376030_at	Acap1	ArfGAP with coiled-coil, ankyrin repeat and PH dom	uniq_isletLongDR_555	0	4.56	4.41	4.22	4.18	0	-0.145	-1.106	0.880	0	-0.335	-1.261	0.351	0	-0.381	-1.302	0.304
1376102_at	Tmbim1	transmembrane BAX inhibitor motif containing 1	uniq_isletLongDR_555	0	5.94	5.81	6.01	5.59	0	-0.138	-1.100	0.704	0	0.064	1.046	0.957	0	-0.352	-1.276	0.342
1376160_at	Relb	v-rel reticuloendotheliosis viral oncogene homolog	uniq_isletLongDR_555	0	5.41	5.20	5.17	5.66	0	-0.214	-1.160	0.760	0	-0.241	-1.182	0.602	0	0.244	1.185	0.774
1376255_at	Map4k1	mitogen activated protein kinase kinase kinase kina	uniq_isletLongDR_555	0	4.55	4.18	4.49	4.53	0	-0.375	-1.297	0.302	0	-0.059	-1.042	0.943	0	-0.022	-1.015	1.063
1376259_at	Prkcq	protein kinase C, theta	uniq_isletLongDR_555	0	4.08	3.98	3.92	4.11	0	-0.099	-1.071	1.021	0	-0.157	-1.115	0.799	0	0.035	1.024	1.103
1376311_at	Ntng1	netrin G1	uniq_isletLongDR_555	0	3.20	3.08	3.11	3.13	0	-0.121	-1.088	1.008	0	-0.089	-1.064	1.021	0	-0.069	-1.049	1.128
1376327_at	Tnfrsf14	tumor necrosis factor receptor superfamily, membe	uniq_isletLongDR_555	0	3.99	4.18	3.84	4.17	0	0.191	1.142	0.709	0	-0.147	-1.107	0.869	0	0.184	1.136	0.912
1376667_at	Cyp26b1	cytochrome P450, family 26, subfamily b, polypepti	uniq_isletLongDR_555	0	5.25	5.02	5.08	5.17	0	-0.233	-1.176	0.734	0	-0.170	-1.125	0.847	0	-0.083	-1.059	1.109
1376688_a_at	LOC1009104	paired immunoglobulin-like type 2 receptor alpha-li	uniq_isletLongDR_555	0	3.97	4.11	4.18	4.18	0	0.139	1.101	0.883	0	0.203	1.151	0.669	0	0.209	1.156	0.847
1376706_at	Tmem47	transmembrane protein 47	uniq_isletLongDR_555	0	7.92	7.74	7.65	7.72	0	-0.180	-1.133	0.867	0	-0.274	-1.209	0.486	0	-0.196	-1.145	0.854
1376708_at	---	---	uniq_isletLongDR_555	0	6.19	6.11	6.11	6.27	0	-0.081	-1.058	0.995	0	-0.086	-1.061	0.910	0	0.075	1.054	1.083
1376766_at	Fmnl1	formin-like 1	uniq_isletLongDR_555	0	5.79	5.56	5.70	5.73	0	-0.234	-1.176	0.733	0	-0.092	-1.066	1.037	0	-0.060	-1.043	1.152
1376775_at	Prr5l	proline rich 5 like	uniq_isletLongDR_555	0	3.99	3.83	3.91	3.75	0	-0.158	-1.115	0.859	0	-0.080	-1.057	0.927	0	-0.242	-1.183	0.673
1376795_at	Pik3ap1	phosphoinositide-3-kinase adaptor protein 1	uniq_isletLongDR_555	0	5.68	5.58	5.62	5.47	0	-0.105	-1.076	0.907	0	-0.059	-1.042	0.964	0	-0.211	-1.158	0.756

Affymetrix Probeset ID	Gene Symbol	Gene Title	Figure 7 A Venn n=2289	Present in any 2 analyses n=423 1=yes 0=no	Mean log2 intensity DR+/+ ND	Mean log2 intensity DR+/+ HCD	Mean log2 intensity DR+/+ B/S	Mean log2 intensity F+/+ day 40 ND	DR+/+HCD vs DR+/+ND FDR<10% Log2 ratio >0.5 n=636	Log2 ratio DR+/+HCD vs DR+/+ ND	Fold-Change DR+/+HCD vs DR+/+ ND	FDR DR+/+HCD vs DR+/+ ND	DR+/+B/S vs DR+/+ND Log2 ratio >0.5 n=350	Log2 ratio DR+/+ B/S vs DR+/+ ND	Fold-Change DR+/+B/S vs DR+/+ ND	FDR DR+/+B/S vs DR+/+ ND	F+/+ vs DR+/+ND Log2 ratio >0.5 n=1209	Log2 ratio F+/+ vs DR+/+ ND	Fold-Change F+/+ vs DR+/+ ND	FDR F+/+ vs DR+/+ ND
1376835_at	---	---	uniq_isletLongDR_555	0	6.81	6.72	6.84	6.73	0	-0.082	-1.059	1.101	0	0.034	1.024	1.126	0	-0.080	-1.057	1.124
1376895_at	Il16	interleukin 16	uniq_isletLongDR_555	0	4.99	4.71	4.91	4.88	0	-0.285	-1.219	0.471	0	-0.078	-1.055	0.911	0	-0.115	-1.083	0.961
1377023_at	Dusp2	dual specificity phosphatase 2	uniq_isletLongDR_555	0	4.42	4.10	4.13	4.15	0	-0.323	-1.251	0.424	0	-0.291	-1.223	0.463	0	-0.267	-1.204	0.593
1377110_at	Arl5c	ADP-ribosylation factor-like 5C	uniq_isletLongDR_555	0	3.06	3.01	3.05	3.02	0	-0.055	-1.039	1.109	0	-0.016	-1.011	1.123	0	-0.043	-1.030	1.151
1377153_a_at	Klhl6	kelch-like family member 6	uniq_isletLongDR_555	0	4.94	4.67	4.75	4.52	0	-0.268	-1.204	0.625	0	-0.187	-1.138	0.759	0	-0.425	-1.342	0.191
1377169_at	LOC684173	similar to TNF receptor-associated factor 5	uniq_isletLongDR_555	0	5.92	6.06	6.08	5.51	0	0.137	1.099	0.941	0	0.155	1.113	0.870	0	-0.410	-1.329	0.215
1377239_at	Apbb1ip	amyloid beta (A4) precursor protein-binding, family	uniq_isletLongDR_555	0	4.63	4.61	4.78	4.82	0	-0.020	-1.014	1.136	0	0.147	1.108	0.749	0	0.190	1.140	0.901
1377334_at	RT1-Ba	RT1 class II, locus Ba	uniq_isletLongDR_555	0	7.84	7.58	7.83	7.62	0	-0.262	-1.199	0.525	0	-0.016	-1.011	1.106	0	-0.223	-1.167	0.712
1377353_a_at	Tnfsf13	tumor necrosis factor (ligand) superfamily, member	uniq_isletLongDR_555	0	5.31	5.24	5.15	5.10	0	-0.074	-1.052	1.077	0	-0.159	-1.116	0.830	0	-0.206	-1.154	0.810
1377494_at	Fli1	Friend leukemia virus integration 1	uniq_isletLongDR_555	0	6.69	6.52	6.46	6.67	0	-0.164	-1.121	0.891	0	-0.222	-1.166	0.709	0	-0.021	-1.015	1.170
1377631_at	Col9a3	collagen, type IX, alpha 3	uniq_isletLongDR_555	0	4.44	4.21	4.44	4.44	0	-0.230	-1.173	0.616	0	0.000	1.000	1.104	0	0.001	1.001	1.174
1377675_at	LOC10030237	hypothetical protein LOC100302372	uniq_isletLongDR_555	0	6.16	6.15	5.94	6.14	0	-0.013	-1.009	1.159	0	-0.225	-1.169	0.665	0	-0.022	-1.015	1.174
1377698_at	Cd40	CD40 molecule, TNF receptor superfamily member 5	uniq_isletLongDR_555	0	5.05	5.04	5.12	5.10	0	-0.012	-1.008	1.169	0	0.066	1.047	1.000	0	0.051	1.036	1.062
1377718_at	Fancb	Fanconi anemia, complementation group B	uniq_isletLongDR_555	0	4.97	4.90	4.98	4.98	0	-0.074	-1.052	1.067	0	0.008	1.005	1.093	0	0.008	1.005	1.177
1377727_at	Baz1a	bromodomain adjacent to zinc finger domain, 1A	uniq_isletLongDR_555	0	5.37	5.53	5.40	5.36	0	0.156	1.115	0.855	0	0.025	1.017	0.970	0	-0.013	-1.009	1.098
1377735_at	Plekha3	pleckstrin homology domain-containing, family A (p	uniq_isletLongDR_555	0	8.16	8.37	8.42	8.21	0	0.218	1.163	0.726	0	0.264	1.201	0.595	0	0.058	1.041	1.097
1377759_at	Bid	BH3 interacting domain death agonist	uniq_isletLongDR_555	0	5.26	5.45	5.36	5.32	0	0.187	1.139	0.785	0	0.095	1.068	0.954	0	0.057	1.040	1.089
1377810_at	---	---	uniq_isletLongDR_555	0	4.94	4.98	4.76	4.69	0	0.038	1.027	1.152	0	-0.181	-1.134	0.676	0	-0.253	-1.192	0.638
1377916_at	Slfn2	schlafen 2	uniq_isletLongDR_555	0	6.31	6.02	6.17	5.81	0	-0.288	-1.221	0.404	0	-0.136	-1.099	0.801	0	-0.494	-1.408	0.126
1377943_at	---	---	uniq_isletLongDR_555	0	3.65	3.68	3.69	3.69	0	0.027	1.019	1.164	0	0.033	1.023	1.128	0	0.034	1.024	1.110
1378028_at	Mad2l1	MAD2 mitotic arrest deficient-like 1 (yeast)	uniq_isletLongDR_555	0	4.80	4.74	4.72	4.95	0	-0.053	-1.037	1.114	0	-0.075	-1.054	1.049	0	0.152	1.111	0.977
1378047_at	Spic	Spi-C transcription factor (Spi-1/PU.1 related)	uniq_isletLongDR_555	0	3.05	2.87	3.02	3.06	0	-0.181	-1.134	0.793	0	-0.031	-1.022	1.127	0	0.010	1.007	1.178
1378057_at	Flrt3	fibronectin leucine rich transmembrane protein 3	uniq_isletLongDR_555	0	5.31	4.96	5.10	5.01	0	-0.346	-1.271	0.318	0	-0.208	-1.155	0.477	0	-0.304	-1.235	0.451
1378156_at	Cys1	cystin 1	uniq_isletLongDR_555	0	6.22	6.32	6.32	6.47	0	0.090	1.065	1.055	0	0.091	1.065	1.049	0	0.243	1.183	0.821
1378185_at	Nxt2	nuclear transport factor 2-like export factor 2	uniq_isletLongDR_555	0	6.63	6.98	6.97	6.85	0	0.354	1.278	0.444	0	0.343	1.268	0.400	0	0.218	1.163	0.877
1378247_at	Eaf2	ELL associated factor 2	uniq_isletLongDR_555	0	2.99	2.97	2.83	2.93	0	-0.014	-1.010	1.170	0	-0.158	-1.116	0.859	0	-0.056	-1.040	1.157
1378377_at	Dapp1	dual adaptor of phosphotyrosine and 3-phosphoinc	uniq_isletLongDR_555	0	5.46	5.24	5.19	5.23	0	-0.222	-1.166	0.716	0	-0.273	-1.208	0.528	0	-0.234	-1.176	0.685
1378419_at	Rnase2	ribonuclease, RNase A family, 2 (liver, eosinophil-de	uniq_isletLongDR_555	0	2.93	2.82	2.78	2.88	0	-0.115	-1.083	0.964	0	-0.153	-1.112	0.891	0	-0.053	-1.037	1.155
1378451_at	Cyba	cytochrome b-245, alpha polypeptide	uniq_isletLongDR_555	0	4.03	3.81	3.88	3.85	0	-0.222	-1.166	0.671	0	-0.146	-1.107	0.833	0	-0.186	-1.137	0.789
1378520_at	Bcl11b	B-cell CLL/lymphoma 11B (zinc finger protein)	uniq_isletLongDR_555	0	3.90	3.77	3.73	4.06	0	-0.127	-1.092	0.818	0	-0.165	-1.121	0.773	0	0.160	1.118	0.943
1378526_at	Gimap6	GTPase, IMAP family member 6	uniq_isletLongDR_555	0	7.00	6.75	6.97	6.72	0	-0.252	-1.191	0.643	0	-0.034	-1.024	1.127	0	-0.283	-1.217	0.548
1379262_at	Acot9	acyl-CoA thioesterase 9	uniq_isletLongDR_555	0	7.47	7.65	7.52	7.47	0	0.183	1.135	0.762	0	0.046	1.033	1.115	0	-0.003	-1.002	1.179
1379275_at	Snx10	sorting nexin 10	uniq_isletLongDR_555	0	5.71	5.88	5.40	5.72	0	0.169	1.125	0.805	0	-0.309	-1.239	0.380	0	0.015	1.011	1.147
1379295_at	Gngt2	guanine nucleotide binding protein (G protein), gam	uniq_isletLongDR_555	0	6.17	5.87	5.95	5.76	0	-0.304	-1.235	0.432	0	-0.229	-1.172	0.577	0	-0.410	-1.329	0.229
1379368_at	Bcl6	B-cell CLL/lymphoma 6	uniq_isletLongDR_555	0	5.45	5.75	5.52	5.55	0	0.295	1.227	0.544	0	0.067	1.048	1.000	0	0.103	1.074	1.011
1379563_at	Irf5	interferon regulatory factor 5	uniq_isletLongDR_555	0	5.41	4.99	5.18	5.22	0	-0.421	-1.339	0.224	0	-0.232	-1.175	0.566	0	-0.194	-1.144	0.852
1379606_at	Rab30	RAB30, member RAS oncogene family	uniq_isletLongDR_555	0	4.75	4.77	4.83	4.95	0	0.021	1.015	1.170	0	0.086	1.061	1.040	0	0.206	1.153	0.907
1379653_a_at	Sh2d1a	SH2 domain containing 1A	uniq_isletLongDR_555	0	2.66	2.85	2.60	2.58	0	0.196	1.146	0.813	0	-0.058	-1.041	1.078	0	-0.076	-1.054	1.130
1379659_at	Bmp2k	BMP-2 inducible kinase	uniq_isletLongDR_555	0	4.16	4.34	4.36	4.64	0	0.180	1.133	0.874	0	0.203	1.151	0.763	0	0.478	1.392	0.357
1379677_at	Tnfsf13	tumor necrosis factor (ligand) superfamily, member	uniq_isletLongDR_555	0	4.82	4.67	4.67	4.51	0	-0.151	-1.110	0.794	0	-0.147	-1.107	0.787	0	-0.311	-1.240	0.467
1379732_at	Stx11	syntaxin 11	uniq_isletLongDR_555	0	3.10	3.18	3.05	3.23	0	0.081	1.058	1.062	0	-0.046	-1.033	1.029	0	0.125	1.091	1.017
1379780_at	Fam65b	family with sequence similarity 65, member B	uniq_isletLongDR_555	0	4.07	3.95	3.88	4.36	0	-0.120	-1.087	1.003	0	-0.186	-1.138	0.784	0	0.295	1.227	0.685
1379791_at	Cd3e	CD3 molecule, epsilon	uniq_isletLongDR_555	0	4.91	4.88	4.75	4.97	0	-0.030	-1.021	1.114	0	-0.161	-1.118	0.781	0	0.062	1.044	1.087
1379932_at	Clcn4	chloride channel, voltage-sensitive 4	uniq_isletLongDR_555	0	4.70	4.66	4.60	5.40	0	-0.040	-1.028	0.981	0	-0.098	-1.071	0.921	0	0.693	1.617	0.121
1379957_at	Slfn13	schlafen family member 13	uniq_isletLongDR_555	0	5.99	5.70	5.96	6.58	0	-0.286	-1.219	0.446	0	-0.032	-1.023	1.127	0	0.588	1.503	0.201
1380021_at	Dennd2d	DENN/MADD domain containing 2D	uniq_isletLongDR_555	0	5.53	5.53	5.50	5.24	0	-0.003	-1.002	1.101	0	-0.028	-1.019	1.050	0	-0.292	-1.224	0.503
1380293_at	LOC361346	similar to chromosome 18 open reading frame 54	uniq_isletLongDR_555	0	5.27	5.44	5.21	4.83	0	0.165	1.121	0.893	0	-0.061	-1.043	0.930	0	-0.448	-1.364	0.170
1380333_at	Ptpn22	protein tyrosine phosphatase, non-receptor type 22	uniq_isletLongDR_555	0	3.72	3.62	3.67	3.53	0	-0.099	-1.071	1.028	0	-0.044	-1.031	1.065	0	-0.186	-1.137	0.849
1380336_at	Irak3	interleukin-1 receptor-associated kinase 3	uniq_isletLongDR_555	0	5.19	4.90	4.90	5.03	0	-0.292	-1.224	0.564	0	-0.295	-1.227	0.493	0	-0.163	-1.120	0.954
1380377_at	Btk	Bruton agammaglobulinemia tyrosine kinase	uniq_isletLongDR_555	0	3.66	3.71	3.83	3.84	0	0.049	1.034	1.111	0	0.162	1.119	0.781	0	0.179	1.132	0.950
1380432_at	Cmah	cytidine monophospho-N-acetylneuraminic acid hyc	uniq_isletLongDR_555	0	3.37	3.15	3.04	3.49	0	-0.216	-1.162	0.542	0	-0.325	-1.252	0.374	0	0.122	1.088	0.907
1380467_at	Slc16a1	solute carrier family 16, member 1 (monocarboxylic	uniq_isletLongDR_555	0	4.67	4.50	4.59	4.57	0	-0.171	-1.126	0.794	0	-0.079	-1.057	0.934	0	-0.104	-1.075	1.037
1380521_at	Hcls1	hematopoietic cell specific Lyn substrate 1	uniq_isletLongDR_555	0	7.02	6.87	6.75	6.94	0	-0.149	-1.109	0.957	0	-0.275	-1.210	0.539	0	-0.082	-1.059	1.113
1380568_a_at	Ddb2	damage specific DNA binding protein 2	uniq_isletLongDR_555	0	4.18	4.05	4.17	4.45	0	-0.122	-1.088	0.998	0	-0.004	-1.003	1.102	0	0.273	1.208	0.748
1380617_at	Gimap5	GTPase, IMAP family member 5	uniq_isletLongDR_555	0	6.54	6.46	6.61	6.11	0	-0.078	-1.055	1.067	0	0.076	1.054	1.002	0	-0.422	-1.340	0.200
1380828_at	Gabra1	gamma-aminobutyric acid (GABA) A receptor, alpha	uniq_isletLongDR_555	0	3.96	3.69	4.16	3.90	0	-0.264	-1.201	0.581	0	0.199	1.148	0.731	0	-0.060	-1.042	1.071
1380930_at	RGD1564142	similar to chromosome 10 open reading frame 64	uniq_isletLongDR_555	0	3.60	3.27	3.35	3.39	0	-0.338	-1.264	0.342	0	-0.259	-1.196	0.498	0	-0.213	-1.159	0.715
1381012_at	Serpinf1	serpin peptidase inhibitor, clade F (alpha-2 antiplasr	uniq_isletLongDR_555	0	6.19	5.95	6.09	5.99	0	-0.244	-1.184	0.645	0	-0.099	-1.071	1.020	0	-0.198	-1.147	0.736
1381219_at	Myh11	myosin, heavy chain 11, smooth muscle	uniq_isletLongDR_555	0	3.64	3.45	3.49	3.49	0	-0.187	-1.139	0.747	0	-0.148	-1.108	0.806	0	-0.144	-1.105	0.955
1381221_at	RGD1311300	similar to T cell receptor V delta 6	uniq_isletLongDR_555	0	4.01	3.66	3.83	3.74	0	-0.351	-1.275	0.358	0	-0.182	-1.134	0.752	0	-0.270	-1.206	0.564
1381311_at	Emr1	EGF-like module containing, mucin-like, hormone re	uniq_isletLongDR_555	0	3.13	3.07	3.08	3.46	0	-0.059	-1.042	1.003	0	-0.058	-1.041	1.075	0	0.327	1.254	0.638
1381328_at	Cr2	complement component (3d/Epstein Barr virus) rec	uniq_isletLongDR_555	0	2.78	2.61	2.81	2.72	0	-0.173	-1.127	0.880	0	0.030	1.021	1.128	0	-0.059	-1.042	1.155
1381357_at	Ptpn22	protein tyrosine phosphatase, non-receptor type 22	uniq_isletLongDR_555	0	2.81	2.85	2.69	2.64	0	0.045	1.032	1.120	0	-0.122	-1.088	0.882	0	-0.173	-1.127	0.851
1381399_at	LOC10036065	ribosomal protein L37-like /// ribosomal protein L37	uniq_isletLongDR_555	0	3.71	3.74	3.49	3.79	0	0.028	1.020	1.158	0	-0.221	-1.165	0.550	0	0.077	1.055	1.076
1381570_at	Nfkbid	nuclear factor of kappa light polypeptide gene enha	uniq_isletLongDR_555	0	6.22	5.97	5.82	6.16	0	-0.251	-1.190	0.653	0	-0.400	-1.320	0.219	0	-0.059	-1.042	1.128
1381593_x_at	RT1-Ba	RT1 class II, locus Ba	uniq_isletLongDR_555	0	7.37	7.06	7.13	7.73	0	-0.305	-1.236	0.522	0	-0.239	-1.180	0.611	0	0.360	1.284	0.567
1381875_at	Nmi	N-myc (and STAT) interactor	uniq_isletLongDR_555	0	5.64	5.85	5.82	5.98	0	0.204	1.152	0.818	0	0.177	1.130	0.834	0	0.335	1.261	0.632
1382026_at	Arhgap9	Rho GTPase activating protein 9	uniq_isletLongDR_555	0	4.06	4.36	3.95	4.13	0	0.302	1.233	0.533	0	-0.107	-1.077	0.987	0	0.070	1.050	1.062
1382043_at	Unc93b1	unc-93 homolog B1 (C. elegans)	uniq_isletLongDR_555	0	6.90	7.15	7.32	7.05	0	0.243	1.183	0.607	0	0.416	1.334	0.256	0	0.148	1.108	0.954
1382058_at	Rras2	related RAS viral (r-ras) oncogene homolog 2	uniq_isletLongDR_555	0	6.10	6.46	6.23	6.13	0	0.363	1.286	0.441	0	0.133	1.096	0.875	0	0.036	1.026	1.096
1382106_at	Ccl6	chemokine (C-C motif) ligand 6	uniq_isletLongDR_555	0	3.34	3.30	3.17	3.52	0	-0.036	-1.026	1.091	0	-0.167	-1.123	0.746	0	0.177	1.131	0.915
1382167_at	Sit1	signaling threshold regulating transmembrane adap	uniq_isletLongDR_555	0	4.21	4.22	4.18	4.19	0	0.006	1.004	1.126	0	-0.028	-1.020	1.101	0	-0.022	-1.015	1.157
1382181_at	Gsap	gamma-secretase activating protein	uniq_isletLongDR_555	0	5.85	5.79	5.73	5.45	0	-0.064	-1.045	1.117	0	-0.127	-1.092	0.931	0	-0.401	-1.321	0.230
1382283_at	Wipf1	WAS/WASL interacting protein family, member 1	uniq_isletLongDR_555	0	4.79	4.60	4.61	4.93	0	-0.186	-1.138	0.662	0	-0.178	-1.131	0.795	0	0.140	1.102	0.925
1382328_at	Gimap1	GTPase, IMAP family member 1	uniq_isletLongDR_555	0	3.38	3.38	3.43	3.24	0	0.002	1.002	1.168	0	0.055	1.039	1.061	0	-0.143	-1.104	0.988
1382337_at	Stap1	signal transducing adaptor family member 1	uniq_isletLongDR_555	0	2.99	2.69	3.01	2.82	0	-0.295	-1.227	0.463	0	0.025	1.017	1.115	0	-0.170	-1.125	0.899
1382378_at	Ctsw	cathepsin W	uniq_isletLongDR_555	0	4.26	4.03	3.96	4.29	0	-0.232	-1.175	0.674	0	-0.299	-1.230	0.442	0	0.033	1.023	1.090
1382439_at	Itgb6	integrin, beta 6	uniq_isletLongDR_555	0	6.56	6.94	6.51	6.28	0	0.375	1.296	0.365	0	-0.056	-1.039	1.044	0	-0.284	-1.217	0.530
1382442_at	Sept6	septin 6	uniq_isletLongDR_555	0	3.98	3.73	3.62	3.78	0	-0.252	-1.191	0.558	0	-0.356	-1.280	0.319	0	-0.201	-1.149	0.708
1382531_at	Tlr7	toll-like receptor 7	uniq_isletLongDR_555	0	3.21	3.02	3.20	3.34	0	-0.186	-1.138	0.825	0	-0.008	-1.005	1.126	0	0.134	1.097	0.999
1382546_at	Phf11	PHD finger protein 11	uniq_isletLongDR_555	0	5.74	5.51	5.51	5.63	0	-0.234	-1.176	0.692	0	-0.237	-1.179	0.636	0	-0.114	-1.082	0.975
1382566_at	Il7r /// LOC10	interleukin 7 receptor /// interleukin-7 receptor sub	uniq_isletLongDR_555	0	4.33	4.34	4.16	4.45	0	0.017	1.012	1.169	0	-0.171	-1.126	0.836	0	0.126	1.091	1.024
1382590_at	Rftn1	raftlin lipid raft linker 1	uniq_isletLongDR_555	0	6.58	6.44	6.41	6.58	0	-0.142	-1.104	0.880	0	-0.169	-1.124	0.784	0	-0.007	-1.005	1.156
1382622_at	Cst7	cystatin F (leukocystatin)	uniq_isletLongDR_555	0	3.84	3.69	3.70	3.90	0	-0.146	-1.107	0.892	0	-0.144	-1.105	0.836	0	0.056	1.040	1.043
1382663_at	Batf	basic leucine zipper transcription factor, ATF-like	uniq_isletLongDR_555	0	5.21	4.96	5.13	5.37	0	-0.250	-1.189	0.443	0	-0.088	-1.063	0.729	0	0.153	1.112	0.833
1382803_at	Map4k1	mitogen activated protein kinase kinase kinase kina	uniq_isletLongDR_555	0	4.31	4.10	3.92	4.28	0	-0.209	-1.156	0.758	0	-0.392	-1.313	0.231	0	-0.037	-1.026	1.163
1382947_at	Rab1b	RAB1B, member RAS oncogene family	uniq_isletLongDR_555	0	7.88	7.52	7.53	7.70	0	-0.357	-1.281	0.368	0	-0.352	-1.276	0.341	0	-0.183	-1.135	0.884
1382955_at	Gpr126	G protein-coupled receptor 126	uniq_isletLongDR_555	0	3.68	3.30	3.42	3.65	0	-0.375	-1.297	0.279	0	-0.257	-1.195	0.547	0	-0.028	-1.020	1.142
1383008_at	Smc4	structural maintenance of chromosomes 4	uniq_isletLongDR_555	0	6.37	6.26	6.48	6.15	0	-0.104	-1.075	0.857	0	0.118	1.085	0.958	0	-0.214	-1.160	0.775
1383015_at	Tbc1d10c	TBC1 domain family, member 10C	uniq_isletLongDR_555	0	5.41	5.79	5.87	5.27	0	0.379	1.300	0.372	0	0.454	1.369	0.230	0	-0.143	-1.104	0.853
1383085_at	Sh3bgrl	SH3 domain binding glutamic acid-rich protein like	uniq_isletLongDR_555	0	9.22	9.05	9.04	8.73	0	-0.175	-1.129	0.748	0	-0.185	-1.137	0.754	0	-0.494	-1.409	0.110
1383131_at	Itgb2	integrin, beta 2	uniq_isletLongDR_555	0	5.24	5.15	5.22	5.36	0	-0.092	-1.066	1.072	0	-0.013	-1.009	1.149	0	0.118	1.085	1.047
1383145_at	Lamp3	lysosomal-associated membrane protein 3	uniq_isletLongDR_555	0	2.75	2.64	2.70	2.68	0	-0.116	-1.084	1.003	0	-0.051	-1.036	1.108	0	-0.069	-1.049	1.137
1383163_at	Igj	immunoglobulin joining chain	uniq_isletLongDR_555	0	5.30	5.09	5.06	5.17	0	-0.212	-1.159	0.674	0	-0.240	-1.181	0.538	0	-0.128	-1.093	0.984
1383291_at	C7 /// Tubb4l	complement component 7 /// tubulin, beta 4B class	uniq_isletLongDR_555	0	6.16	6.28	5.90	5.68	0	0.119	1.086	0.906	0	-0.261	-1.198	0.431	0	-0.484	-1.399	0.139
1383320_at	Lck	lymphocyte-specific protein tyrosine kinase	uniq_isletLongDR_555	0	6.67	6.69	6.78	6.64	0	0.020	1.014	1.166	0	0.102	1.073	1.005	0	-0.037	-1.026	1.158
1383322_at	Rasl11b	RAS-like family 11 member B	uniq_isletLongDR_555	0	4.67	4.58	4.74	4.64	0	-0.088	-1.063	0.968	0	0.075	1.053	0.903	0	-0.028	-1.019	1.099
1383407_at	Sash3	SAM and SH3 domain containing 3	uniq_isletLongDR_555	0	5.81	5.77	5.65	5.64	0	-0.047	-1.033	1.089	0	-0.158	-1.115	0.810	0	-0.176	-1.130	0.880
1383426_at	Pstpip1	proline-serine-threonine phosphatase-interacting pi	uniq_isletLongDR_555	0	4.97	4.70	4.59	4.94	0	-0.274	-1.209	0.558	0	-0.386	-1.307	0.258	0	-0.039	-1.028	1.158
1383506_at	Bcl11a	B-cell CLL/lymphoma 11A (zinc finger protein)	uniq_isletLongDR_555	0	3.96	3.79	3.72	3.94	0	-0.172	-1.127	0.818	0	-0.239	-1.180	0.573	0	-0.027	-1.019	1.100
1383564_at	Irf7	interferon regulatory factor 7	uniq_isletLongDR_555	0	6.57	6.57	6.50	6.52	0	-0.004	-1.003	1.173	0	-0.068	-1.048	1.041	0	-0.054	-1.038	1.149
1383630_at	Dok3	docking protein 3	uniq_isletLongDR_555	0	6.12	5.85	5.83	6.19	0	-0.272	-1.208	0.622	0	-0.290	-1.222	0.507	0	0.071	1.051	1.088
1383658_at	Laptm5	lysosomal protein transmembrane 5	uniq_isletLongDR_555	0	7.22	7.00	6.95	7.19	0	-0.217	-1.163	0.751	0	-0.270	-1.206	0.553	0	-0.032	-1.022	1.162
1383685_at	Heatr1 /// LO	HEAT repeat containing 1 /// HEAT repeat-containin	uniq_isletLongDR_555	0	6.49	6.50	6.48	6.53	0	0.006	1.004	1.171	0	-0.014	-1.010	1.124	0	0.038	1.026	1.108
1383785_at	---	---	uniq_isletLongDR_555	0	5.52	5.40	5.68	5.93	0	-0.125	-1.091	0.974	0	0.155	1.113	0.748	0	0.404	1.323	0.466
1383786_at	Rab32	RAB32, member RAS oncogene family	uniq_isletLongDR_555	0	4.84	4.64	4.43	4.91	0	-0.198	-1.147	0.831	0	-0.407	-1.326	0.207	0	0.072	1.051	1.085
1383833_at	Fam55b	family with sequence similarity 55, member B	uniq_isletLongDR_555	0	3.91	3.95	4.14	3.86	0	0.038	1.026	1.134	0	0.230	1.173	0.592	0	-0.050	-1.035	1.145

Affymetrix Probeset ID	Gene Symbol	Gene Title	Figure 7 A Venn n=2289	Present in any 2 analyses n=423 1=yes 0=no	Mean log2 intensity DR+/+ ND	Mean log2 intensity DR+/+ HCD	Mean log2 intensity DR+/+ B/S	Mean log2 intensity F+/+ day 40 ND	DR+/+HCD vs DR+/+ND FDR<10% Log2 ratio >0.5 n=636	Log2 ratio DR+/+HCD vs DR+/+ ND	Fold-Change DR+/+HCD vs DR+/+ ND	FDR DR+/+HCD vs DR+/+ ND	DR+/+B/S vs DR+/+ND Log2 ratio >0.5 n=350	Log2 ratio DR+/+ B/S vs DR+/+ ND	Fold-Change DR+/+B/S vs DR+/+ ND	FDR DR+/+B/S vs DR+/+ ND	F+/+ vs DR+/+ND Log2 ratio >0.5 n=1209	Log2 ratio F+/+ vs DR+/+ ND	Fold-Change F+/+ vs DR+/+ ND	FDR F+/+ vs DR+/+ ND
1383863_at	Lmo2	LIM domain only 2	uniq_isletLongDR_555	0	8.38	8.15	8.35	8.15	0	-0.237	-1.178	0.645	0	-0.030	-1.021	1.111	0	-0.238	-1.179	0.701
1383893_at	Atp6v1g3	ATPase, H+ transporting, lysosomal V1 subunit G3	uniq_isletLongDR_555	0	3.07	3.08	3.01	3.03	0	0.008	1.006	1.153	0	-0.062	-1.044	1.084	0	-0.043	-1.030	1.157
1383906_at	Neurl3	neuralized homolog 3 (Drosophila)	uniq_isletLongDR_555	0	5.24	5.08	4.90	5.52	0	-0.155	-1.114	0.887	0	-0.336	-1.262	0.335	0	0.283	1.217	0.735
1383926_at	Bub1b	BUB1 mitotic checkpoint serine/threonine kinase B	uniq_isletLongDR_555	0	4.27	4.03	3.97	4.33	0	-0.243	-1.184	0.636	0	-0.305	-1.235	0.452	0	0.057	1.040	1.074
1384017_at	---	---	uniq_isletLongDR_555	0	5.47	5.23	5.37	5.30	0	-0.235	-1.177	0.588	0	-0.103	-1.074	0.869	0	-0.173	-1.127	0.886
1384035_at	---	---	uniq_isletLongDR_555	0	3.22	2.75	3.07	3.07	0	-0.472	-1.387	0.134	0	-0.153	-1.112	0.817	0	-0.154	-1.113	0.944
1384036_s_at	---	---	uniq_isletLongDR_555	0	3.44	3.10	3.31	3.36	0	-0.340	-1.265	0.417	0	-0.133	-1.097	0.922	0	-0.077	-1.055	1.123
1384111_at	Stat4	signal transducer and activator of transcription 4	uniq_isletLongDR_555	0	4.63	4.25	4.38	4.60	0	-0.378	-1.300	0.313	0	-0.250	-1.189	0.599	0	-0.027	-1.019	1.157
1384192_at	Chst1	carbohydrate (keratan sulfate Gal-6) sulfotransferas	uniq_isletLongDR_555	0	5.32	5.36	5.36	5.49	0	0.043	1.030	1.130	0	0.046	1.032	1.085	0	0.174	1.128	0.943
1384208_at	Igj	immunoglobulin joining chain	uniq_isletLongDR_555	0	4.09	3.76	3.84	3.99	0	-0.324	-1.252	0.418	0	-0.252	-1.191	0.558	0	-0.103	-1.074	1.016
1384292_at	Dok1	docking protein 1	uniq_isletLongDR_555	0	5.03	5.01	5.09	4.95	0	-0.018	-1.013	1.137	0	0.054	1.038	1.071	0	-0.080	-1.057	1.106
1384298_at	Myo1f	myosin IF	uniq_isletLongDR_555	0	4.31	3.96	4.03	4.41	0	-0.357	-1.281	0.329	0	-0.289	-1.221	0.455	0	0.099	1.071	1.040
1384350_at	Nckap1l	NCK associated protein 1 like	uniq_isletLongDR_555	0	4.40	4.17	4.18	4.09	0	-0.225	-1.169	0.659	0	-0.221	-1.165	0.649	0	-0.305	-1.235	0.472
1384411_at	Glcci1	glucocorticoid induced transcript 1	uniq_isletLongDR_555	0	7.88	7.97	8.11	7.93	0	0.091	1.065	1.073	0	0.234	1.176	0.643	0	0.056	1.040	1.074
1384456_at	Usp24	ubiquitin specific peptidase 24	uniq_isletLongDR_555	0	6.09	6.55	6.38	6.38	0	0.460	1.376	0.288	0	0.289	1.222	0.521	0	0.289	1.222	0.724
1384476_at	Cmah	cytidine monophospho-N-acetylneuraminic acid hyd	uniq_isletLongDR_555	0	5.99	5.68	5.69	5.94	0	-0.316	-1.245	0.422	0	-0.304	-1.235	0.443	0	-0.047	-1.033	1.147
1384493_at	Gsto2 /// LOC	glutathione S-transferase omega 2 /// glutathione S	uniq_isletLongDR_555	0	5.82	5.84	5.92	5.95	0	0.016	1.011	1.159	0	0.095	1.068	0.955	0	0.130	1.094	0.999
1384507_at	Fam105a	family with sequence similarity 105, member A	uniq_isletLongDR_555	0	4.78	5.20	4.97	4.97	0	0.422	1.340	0.299	0	0.191	1.142	0.733	0	0.186	1.138	0.899
1384555_at	Dennd1c	DENN/MADD domain containing 1C	uniq_isletLongDR_555	0	5.24	4.92	4.92	5.13	0	-0.319	-1.247	0.444	0	-0.315	-1.244	0.412	0	-0.107	-1.077	1.047
1384837_at	Cd69	Cd69 molecule	uniq_isletLongDR_555	0	4.29	4.62	4.60	4.37	0	0.331	1.258	0.400	0	0.308	1.238	0.366	0	0.084	1.060	0.962
1384895_at	Pacs2	phosphofurin acidic cluster sorting protein 2	uniq_isletLongDR_555	0	4.16	4.35	4.36	4.71	0	0.189	1.140	0.758	0	0.208	1.155	0.659	0	0.558	1.472	0.239
1384900_at	Mob3a	MOB kinase activator 3A	uniq_isletLongDR_555	0	4.54	4.33	4.31	4.27	0	-0.213	-1.159	0.692	0	-0.230	-1.173	0.618	0	-0.273	-1.208	0.562
1384944_at	Bcl11b	B-cell CLL/lymphoma 11B (zinc finger protein)	uniq_isletLongDR_555	0	3.00	3.02	2.98	3.17	0	0.022	1.015	1.157	0	-0.018	-1.013	1.089	0	0.173	1.128	0.942
1384946_at	Tlr1	toll-like receptor 1	uniq_isletLongDR_555	0	3.44	3.35	3.35	3.27	0	-0.091	-1.065	1.072	0	-0.090	-1.064	0.950	0	-0.168	-1.124	0.927
1384981_at	Cd6	Cd6 molecule	uniq_isletLongDR_555	0	5.90	5.70	5.81	6.02	0	-0.199	-1.148	0.773	0	-0.095	-1.068	1.001	0	0.113	1.082	1.020
1385019_at	Bank1	B-cell scaffold protein with ankyrin repeats 1	uniq_isletLongDR_555	0	4.92	4.75	4.75	4.78	0	-0.164	-1.121	0.818	0	-0.162	-1.119	0.787	0	-0.135	-1.098	0.945
1385035_at	Usp12	ubiquitin specific peptidase 12	uniq_isletLongDR_555	0	5.58	5.68	5.55	5.95	0	0.103	1.074	1.068	0	-0.029	-1.020	1.119	0	0.368	1.291	0.557
1385047_x_at	LOC685048 /,	similar to paired immunoglobin-like type 2 receptor	uniq_isletLongDR_555	0	5.75	5.51	5.56	5.83	0	-0.236	-1.178	0.636	0	-0.184	-1.136	0.742	0	0.088	1.063	1.047
1385051_at	Gbp4 /// LOC	guanylate binding protein 4 /// guanylate-binding p	uniq_isletLongDR_555	0	2.70	2.67	2.70	2.97	0	-0.032	-1.022	1.159	0	0.000	1.000	1.156	0	0.262	1.199	0.756
1385058_at	Cldn8	claudin 8	uniq_isletLongDR_555	0	3.81	3.37	3.54	3.47	0	-0.441	-1.358	0.122	0	-0.274	-1.209	0.378	0	-0.345	-1.270	0.338
1385160_at	Stab2	stabilin 2	uniq_isletLongDR_555	0	4.75	4.61	4.65	5.18	0	-0.135	-1.098	0.911	0	-0.091	-1.065	0.961	0	0.435	1.352	0.430
1385213_at	Epsti1	epithelial stromal interaction 1 (breast)	uniq_isletLongDR_555	0	3.83	3.66	3.79	3.57	0	-0.178	-1.131	0.805	0	-0.042	-1.030	1.070	0	-0.263	-1.200	0.575
1385270_s_at	LOC10036378	RAB1B, member RAS oncogene family-like /// RAB1/	uniq_isletLongDR_555	0	5.51	5.44	5.71	5.31	0	-0.070	-1.050	0.948	0	0.208	1.155	0.723	0	-0.200	-1.149	0.829
1385397_at	Steap4	STEAP family member 4	uniq_isletLongDR_555	0	7.87	7.88	8.04	7.64	0	0.011	1.008	1.076	0	0.173	1.127	0.674	0	-0.233	-1.175	0.660
1385414_at	Cd8a	CD8a molecule	uniq_isletLongDR_555	0	3.58	3.54	3.52	3.67	0	-0.045	-1.032	1.136	0	-0.066	-1.047	1.037	0	0.083	1.059	1.031
1385440_at	Hcst	hematopoietic cell signal transducer	uniq_isletLongDR_555	0	3.77	3.47	3.39	3.44	0	-0.300	-1.231	0.529	0	-0.380	-1.301	0.252	0	-0.330	-1.257	0.394
1385441_at	Vpreb3	pre-B lymphocyte 3	uniq_isletLongDR_555	0	4.95	4.89	4.98	4.99	0	-0.058	-1.041	1.117	0	0.036	1.025	1.080	0	0.041	1.029	1.103
1385465_at	Siglec5	sialic acid binding Ig-like lectin 5	uniq_isletLongDR_555	0	3.10	3.04	2.99	3.06	0	-0.063	-1.045	1.120	0	-0.116	-1.084	0.857	0	-0.038	-1.027	1.164
1385587_at	Mcoln2	mucolipin 2	uniq_isletLongDR_555	0	3.16	3.00	3.13	3.35	0	-0.154	-1.113	0.857	0	-0.028	-1.020	1.099	0	0.196	1.146	0.897
1385700_at	Cr2	complement component (3d/Epstein Barr virus) rec	uniq_isletLongDR_555	0	4.05	3.80	3.93	3.73	0	-0.245	-1.185	0.525	0	-0.124	-1.089	0.784	0	-0.322	-1.250	0.374
1385702_at	Ifi204	interferon activated gene 204	uniq_isletLongDR_555	0	3.58	3.14	3.29	3.11	0	-0.439	-1.355	0.195	0	-0.286	-1.220	0.463	0	-0.462	-1.378	0.155
1385813_at	Mzb1	marginal zone B and B1 cell-specific protein	uniq_isletLongDR_555	0	4.64	4.47	4.54	4.73	0	-0.167	-1.123	0.876	0	-0.091	-1.065	0.996	0	0.098	1.070	1.060
1385831_at	Sash3	SAM and SH3 domain containing 3	uniq_isletLongDR_555	0	2.92	2.82	2.84	2.88	0	-0.101	-1.072	1.044	0	-0.086	-1.061	1.030	0	-0.040	-1.028	1.159
1385832_s_at	Sash3	SAM and SH3 domain containing 3	uniq_isletLongDR_555	0	4.45	4.32	4.24	4.29	0	-0.125	-1.091	1.003	0	-0.207	-1.154	0.726	0	-0.163	-1.120	0.937
1385853_at	Baz1a	bromodomain adjacent to zinc finger domain, 1A	uniq_isletLongDR_555	0	3.54	3.67	3.39	3.19	0	0.132	1.096	0.960	0	-0.145	-1.106	0.805	0	-0.350	-1.275	0.356
1385962_x_at	Blk	B lymphoid tyrosine kinase	uniq_isletLongDR_555	0	4.00	3.87	3.78	3.72	0	-0.138	-1.100	0.960	0	-0.220	-1.164	0.645	0	-0.287	-1.220	0.526
1386052_at	---	---	uniq_isletLongDR_555	0	2.80	2.99	2.81	2.85	0	0.183	1.135	0.820	0	0.011	1.008	1.128	0	0.050	1.035	1.098
1386162_at	Txk	TXK tyrosine kinase	uniq_isletLongDR_555	0	3.62	3.50	3.55	3.61	0	-0.116	-1.084	0.981	0	-0.074	-1.052	0.962	0	-0.012	-1.008	1.104
1386166_at	Atp6v1g3	ATPase, H+ transporting, lysosomal V1 subunit G3	uniq_isletLongDR_555	0	3.09	2.87	2.75	2.94	0	-0.225	-1.169	0.743	0	-0.341	-1.267	0.336	0	-0.152	-1.111	0.956
1386881_at	Igfbp3	insulin-like growth factor binding protein 3	uniq_isletLongDR_555	0	7.91	8.23	8.14	7.71	0	0.321	1.249	0.450	0	0.237	1.178	0.604	0	-0.199	-1.148	0.828
1386925_at	Arpc1b	actin related protein 2/3 complex, subunit 1B	uniq_isletLongDR_555	0	8.03	8.04	7.93	7.76	0	0.018	1.013	1.156	0	-0.091	-1.065	0.915	0	-0.260	-1.198	0.617
1387005_at	Ctss	cathepsin S	uniq_isletLongDR_555	0	6.89	6.98	7.10	7.38	0	0.097	1.069	1.070	0	0.216	1.161	0.727	0	0.497	1.411	0.332
1387036_at	Hes1	hairy and enhancer of split 1 (Drosophila)	uniq_isletLongDR_555	0	7.26	7.05	6.85	7.08	0	-0.211	-1.158	0.677	0	-0.410	-1.329	0.198	0	-0.181	-1.134	0.880
1387098_at	Polr1a	polymerase (RNA) I polypeptide A	uniq_isletLongDR_555	0	5.52	5.66	5.69	5.73	0	0.140	1.102	0.923	0	0.171	1.126	0.796	0	0.211	1.158	0.854
1387114_at	Prkcd	protein kinase C, delta	uniq_isletLongDR_555	0	7.00	7.04	6.96	6.90	0	0.042	1.029	1.152	0	-0.036	-1.025	1.118	0	-0.097	-1.069	1.110
1387189_at	Slc22a3	solute carrier family 22 (extraneuronal monoamine	uniq_isletLongDR_555	0	3.35	3.08	3.23	3.20	0	-0.267	-1.203	0.575	0	-0.118	-1.086	0.901	0	-0.146	-1.107	0.976
1387190_at	Dgka	diacylglycerol kinase, alpha	uniq_isletLongDR_555	0	7.01	7.08	7.04	6.89	0	0.074	1.053	1.072	0	0.032	1.022	1.129	0	-0.119	-1.086	1.056
1387198_at	Inpp5d	inositol polyphosphate-5-phosphatase D	uniq_isletLongDR_555	0	5.56	5.34	5.37	5.58	0	-0.225	-1.169	0.671	0	-0.195	-1.145	0.671	0	0.014	1.010	1.178
1387227_at	Wipf1	WAS/WASL interacting protein family, member 1	uniq_isletLongDR_555	0	6.76	6.51	6.58	6.35	0	-0.249	-1.188	0.557	0	-0.179	-1.132	0.753	0	-0.411	-1.329	0.220
1387277_at	Lyn	v-yes-1 Yamaguchi sarcoma viral related oncogene h	uniq_isletLongDR_555	0	5.64	5.56	5.49	5.66	0	-0.086	-1.062	1.054	0	-0.152	-1.111	0.856	0	0.019	1.013	1.108
1387315_at	Sftpd	surfactant protein D	uniq_isletLongDR_555	0	5.27	4.88	5.06	5.15	0	-0.393	-1.313	0.266	0	-0.216	-1.162	0.698	0	-0.120	-1.087	0.941
1387361_s_at	Pgk1	phosphoglycerate kinase 1	uniq_isletLongDR_555	0	8.83	8.84	8.88	8.84	0	0.014	1.010	1.172	0	0.052	1.037	1.111	0	0.009	1.006	1.177
1387365_at	Nr1h3	nuclear receptor subfamily 1, group H, member 3	uniq_isletLongDR_555	0	5.30	5.13	5.42	4.99	0	-0.165	-1.121	0.818	0	0.122	1.088	0.809	0	-0.302	-1.233	0.483
1387394_at	Il2rb	interleukin 2 receptor, beta	uniq_isletLongDR_555	0	4.10	4.12	3.85	4.36	0	0.012	1.009	1.135	0	-0.252	-1.191	0.569	0	0.252	1.191	0.791
1387442_at	Egr4	early growth response 4	uniq_isletLongDR_555	0	6.48	6.74	6.30	6.28	0	0.252	1.191	0.576	0	-0.185	-1.137	0.658	0	-0.201	-1.150	0.763
1387483_at	Plcg2	phospholipase C, gamma 2	uniq_isletLongDR_555	0	4.31	4.20	4.07	4.34	0	-0.116	-1.084	0.921	0	-0.237	-1.179	0.499	0	0.033	1.023	1.098
1387502_at	Stk17b	serine/threonine kinase 17b	uniq_isletLongDR_555	0	4.99	5.21	4.79	5.02	0	0.226	1.169	0.644	0	-0.192	-1.142	0.647	0	0.034	1.024	1.050
1387566_at	Pla2g4a	phospholipase A2, group IVA (cytosolic, calcium-dep	uniq_isletLongDR_555	0	3.78	3.39	3.50	3.36	0	-0.394	-1.314	0.251	0	-0.284	-1.218	0.499	0	-0.424	-1.342	0.205
1387588_at	Ehd3	EH-domain containing 3	uniq_isletLongDR_555	0	5.82	5.61	5.66	5.76	0	-0.204	-1.152	0.805	0	-0.159	-1.117	0.856	0	-0.058	-1.041	1.155
1387605_at	Casp12	caspase 12	uniq_isletLongDR_555	0	4.18	4.03	3.98	4.18	0	-0.156	-1.114	0.919	0	-0.206	-1.154	0.646	0	-0.007	-1.005	1.151
1387621_at	Tnfrsf4	tumor necrosis factor receptor superfamily, membe	uniq_isletLongDR_555	0	6.57	6.41	6.49	6.46	0	-0.160	-1.117	0.925	0	-0.085	-1.060	1.032	0	-0.117	-1.084	0.970
1387627_at	Cd86	CD86 molecule	uniq_isletLongDR_555	0	3.72	3.41	3.54	3.83	0	-0.308	-1.238	0.465	0	-0.182	-1.134	0.751	0	0.114	1.082	1.013
1387651_at	Aqp1	aquaporin 1	uniq_isletLongDR_555	0	6.01	5.58	5.63	6.10	0	-0.434	-1.351	0.188	0	-0.382	-1.303	0.258	0	0.086	1.061	1.035
1387711_at	Il10	interleukin 10	uniq_isletLongDR_555	0	3.37	3.31	3.25	3.11	0	-0.056	-1.040	1.117	0	-0.119	-1.086	0.932	0	-0.260	-1.198	0.610
1387739_at	Cd8b	CD8b molecule	uniq_isletLongDR_555	0	4.33	4.15	4.02	4.23	0	-0.183	-1.135	0.827	0	-0.317	-1.245	0.362	0	-0.103	-1.074	1.015
1387794_at	Fcn1	ficolin (collagen/fibrinogen domain containing) 1	uniq_isletLongDR_555	0	4.33	4.01	3.98	4.18	0	-0.313	-1.242	0.441	0	-0.344	-1.269	0.350	0	-0.149	-1.109	0.963
1387799_at	Fxyd2	FXYD domain-containing ion transport regulator 2	uniq_isletLongDR_555	0	4.21	4.02	4.15	4.15	0	-0.194	-1.144	0.835	0	-0.063	-1.045	1.061	0	-0.063	-1.045	1.048
1387870_at	Zfp36	zinc finger protein 36	uniq_isletLongDR_555	0	8.42	8.68	8.17	9.11	0	0.257	1.195	0.637	0	-0.256	-1.194	0.521	0	0.686	1.609	0.140
1387951_at	Cd55	Cd55 molecule	uniq_isletLongDR_555	0	4.27	4.57	4.34	3.82	0	0.296	1.228	0.569	0	0.069	1.049	1.001	0	-0.451	-1.367	0.155
1387952_a_at	Cd44	Cd44 molecule	uniq_isletLongDR_555	0	4.82	4.71	4.51	4.42	0	-0.112	-1.081	0.919	0	-0.311	-1.241	0.378	0	-0.397	-1.317	0.251
1387957_a_at	Sh3kbp1	SH3-domain kinase binding protein 1	uniq_isletLongDR_555	0	7.29	7.29	7.30	7.18	0	-0.001	-1.001	1.165	0	0.006	1.004	1.147	0	-0.114	-1.082	1.057
1387965_at	Havcr1	hepatitis A virus cellular receptor 1	uniq_isletLongDR_555	0	4.07	3.91	3.98	4.03	0	-0.158	-1.116	0.848	0	-0.090	-1.064	0.959	0	-0.042	-1.029	1.130
1388026_at	Cd247	Cd247 molecule	uniq_isletLongDR_555	0	5.60	5.28	5.49	5.55	0	-0.322	-1.250	0.429	0	-0.112	-1.081	0.965	0	-0.052	-1.037	1.072
1388071_x_at	RT1-EC2	RT1 class Ib, locus EC2	uniq_isletLongDR_555	0	8.57	8.51	8.44	8.44	0	-0.057	-1.040	1.101	0	-0.122	-1.088	0.926	0	-0.124	-1.090	1.039
1388131_at	Tubb2b	tubulin, beta 2B class IIb	uniq_isletLongDR_555	0	6.43	6.21	6.06	6.00	0	-0.215	-1.161	0.722	0	-0.374	-1.296	0.287	0	-0.425	-1.343	0.207
1388164_at	RT1-S3	RT1 class Ib, locus S3	uniq_isletLongDR_555	0	7.07	7.26	7.51	7.29	0	0.192	1.142	0.822	0	0.436	1.353	0.248	0	0.221	1.166	0.867
1388181_at	Igd	immunoglobulin delta heavy chain constant region	uniq_isletLongDR_555	0	4.46	4.24	4.32	4.55	0	-0.215	-1.160	0.679	0	-0.140	-1.102	0.838	0	0.092	1.066	1.015
1388202_at	RT1-EC2	RT1 class Ib, locus EC2	uniq_isletLongDR_555	0	4.01	4.07	3.91	4.49	0	0.059	1.042	1.123	0	-0.095	-1.068	1.000	0	0.488	1.402	0.349
1388212_a_at	RT1-S3	RT1 class Ib, locus S3	uniq_isletLongDR_555	0	7.21	7.36	7.56	7.43	0	0.150	1.109	0.920	0	0.344	1.269	0.393	0	0.212	1.158	0.869
1388213_a_at	RT1-S3	RT1 class Ib, locus S3	uniq_isletLongDR_555	0	6.35	6.65	6.50	6.58	0	0.305	1.235	0.562	0	0.155	1.114	0.881	0	0.233	1.175	0.807
1388275_at	LOC10091277	T-cell receptor beta chain T17T-22-like /// T-cell rec	uniq_isletLongDR_555	0	4.87	4.81	4.70	4.53	0	-0.055	-1.039	1.114	0	-0.171	-1.126	0.797	0	-0.335	-1.262	0.372
1388277_at	LOC1003617(	lambda-chain C1-region-like /// Ig lambda chain V-V	uniq_isletLongDR_555	0	5.19	4.73	4.89	4.72	0	-0.459	-1.375	0.144	0	-0.301	-1.232	0.451	0	-0.476	-1.391	0.127
1388318_at	Pgk1	phosphoglycerate kinase 1	uniq_isletLongDR_555	0	9.80	9.85	9.96	9.83	0	0.048	1.034	1.147	0	0.160	1.117	0.874	0	0.031	1.022	1.109
1388340_at	Ns5atp9	NS5A (hepatitis C virus) transactivated protein 9	uniq_isletLongDR_555	0	6.44	6.00	6.12	6.25	0	-0.437	-1.354	0.182	0	-0.324	-1.252	0.396	0	-0.192	-1.142	0.870
1388408_at	Prr13	proline rich 13	uniq_isletLongDR_555	0	8.62	8.58	8.65	8.46	0	-0.035	-1.025	1.143	0	0.038	1.027	1.128	0	-0.153	-1.112	0.977
1388528_at	Fbl	fibrillarin	uniq_isletLongDR_555	0	8.64	8.84	8.80	8.30	0	0.201	1.150	0.822	0	0.164	1.120	0.884	0	-0.340	-1.266	0.370
1388581_at	Hn1	hematological and neurological expressed 1	uniq_isletLongDR_555	0	7.66	7.71	7.74	7.43	0	0.051	1.036	1.126	0	0.078	1.056	1.074	0	-0.231	-1.174	0.727
1388596_at	Cotl1	coactosin-like 1 (Dictyostelium)	uniq_isletLongDR_555	0	8.42	8.40	8.44	8.86	0	-0.026	-1.018	1.149	0	0.023	1.016	1.137	0	0.436	1.353	0.424
1388602_at	Cfd	complement factor D (adipsin)	uniq_isletLongDR_555	0	4.57	4.63	4.44	4.02	0	0.065	1.046	1.028	0	-0.130	-1.094	0.837	0	-0.546	-1.460	0.101
1388629_at	Impdh2	IMP (inosine 5'-monophosphate) dehydrogenase 2	uniq_isletLongDR_555	0	8.91	9.28	9.07	8.63	0	0.377	1.299	0.388	0	0.168	1.123	0.870	0	-0.277	-1.212	0.563
1388663_at	LOC687118	similar to death effector domain-containing DNA bir	uniq_isletLongDR_555	0	5.19	5.52	5.47	5.39	0	0.337	1.263	0.485	0	0.286	1.219	0.515	0	0.205	1.153	0.906
1388694_at	LOC10090979	RT1 class I histocompatibility antigen, AA alpha chai	uniq_isletLongDR_555	0	4.66	4.38	4.37	4.98	0	-0.282	-1.216	0.482	0	-0.294	-1.226	0.459	0	0.321	1.249	0.639
1388740_at	Fermt3	fermitin family member 3	uniq_isletLongDR_555	0	5.24	5.20	5.37	4.98	0	-0.036	-1.025	1.072	0	0.135	1.098	0.802	0	-0.258	-1.196	0.621
1388784_at	Csf1r	colony stimulating factor 1 receptor	uniq_isletLongDR_555	0	5.50	5.22	5.51	5.40	0	-0.277	-1.212	0.563	0	0.013	1.009	1.127	0	-0.102	-1.073	1.016
1388837_at	Slc44a2	solute carrier family 44, member 2	uniq_isletLongDR_555	0	7.19	7.07	7.19	7.03	0	-0.120	-1.087	0.815	0	0.001	1.001	1.132	0	-0.167	-1.123	0.912
1388846_at	Bcl2l12	BCL2-like 12 (proline rich)	uniq_isletLongDR_555	0	4.19	3.72	3.80	3.98	0	-0.472	-1.387	0.121	0	-0.389	-1.310	0.235	0	-0.214	-1.160	0.771
1388864_at	Ddx54	DEAD (Asp-Glu-Ala-Asp) box polypeptide 54	uniq_isletLongDR_555	0	5.48	5.62	5.69	6.04	0	0.147	1.107	0.955	0	0.208	1.155	0.731	0	0.566	1.480	0.242

Affymetrix Probeset ID	Gene Symbol	Gene Title	Figure 7 A Venn n=2289	Present in any 2 analyses n=423 1=yes 0=no	Mean log2 intensity DR+/+ ND	Mean log2 intensity DR+/+ HCD	Mean log2 intensity DR+/+ B/S	Mean log2 intensity F+/+ day 40 ND	DR+/+HCD vs DR+/+ND FDR<10% Log2 ratio >0.5 n=636	Log2 ratio DR+/+HCD vs DR+/+ ND	Fold-Change DR+/+H CD vs DR+/+ ND	FDR DR+/+H CD vs DR+/+ ND	DR+/+B/S vs DR+/+ND Log2 ratio >0.5 n=350	Log2 ratio DR+/+ B/S vs DR+/+ ND	Fold-Change DR+/+B /S vs DR+/+ ND	FDR DR+/+B /S vs DR+/+ ND	F+/+ vs DR+/+N D Log2 ratio >0.5 n=1209	Log2 ratio F+/+ vs DR+/+ ND	Fold-Change F+/+ vs DR+/+ ND	FDR F+/+ vs DR+/+ ND
1388887_at	Ggta1p	glycoprotein, alpha-galactosyltransferase 1,3	uniq_isletLongDR_555	0	7.48	7.08	7.42	7.47	0	-0.394	-1.314	0.254	0	-0.056	-1.040	1.045	0	-0.006	-1.005	1.177
1388924_at	Angptl4	angiopoietin-like 4	uniq_isletLongDR_555	0	5.15	5.53	5.48	5.24	0	0.373	1.295	0.373	0	0.329	1.256	0.399	0	0.088	1.063	1.007
1388940_at	Arhgap25	Rho GTPase activating protein 25	uniq_isletLongDR_555	0	4.33	4.30	4.13	4.53	0	-0.035	-1.024	1.077	0	-0.200	-1.149	0.669	0	0.199	1.148	0.890
1388945_at	Pxdc1	PX domain containing 1	uniq_isletLongDR_555	0	6.28	6.07	6.02	6.16	0	-0.204	-1.152	0.622	0	-0.258	-1.196	0.450	0	-0.119	-1.086	0.958
1389063_at	LOC1009120(	exportin-6-like /// exportin 6	uniq_isletLongDR_555	0	9.14	9.27	9.29	8.89	0	0.129	1.094	1.002	0	0.148	1.108	0.915	0	-0.248	-1.187	0.661
1389074_at	Fchsd2	FCH and double SH3 domains 2	uniq_isletLongDR_555	0	8.68	8.63	8.67	8.49	0	-0.051	-1.036	1.121	0	-0.012	-1.009	1.134	0	-0.191	-1.141	0.865
1389079_at	Dhrs7c	dehydrogenase/reductase (SDR family) member 7C	uniq_isletLongDR_555	0	3.64	3.43	3.46	3.34	0	-0.213	-1.159	0.696	0	-0.180	-1.133	0.677	0	-0.306	-1.236	0.474
1389092_at	Il2rg	interleukin 2 receptor, gamma	uniq_isletLongDR_555	0	5.39	5.10	5.13	5.09	0	-0.290	-1.223	0.472	0	-0.256	-1.194	0.565	0	-0.294	-1.226	0.507
1389110_at	LOC1003600:	mitochondrial ribosomal protein S6-like	uniq_isletLongDR_555	0	7.56	7.56	7.53	7.44	0	0.005	1.003	1.174	0	-0.027	-1.019	1.098	0	-0.113	-1.082	1.063
1389123_at	Ccl6	chemokine (C-C motif) ligand 6	uniq_isletLongDR_555	0	5.52	5.63	5.80	5.74	0	0.109	1.079	1.023	0	0.278	1.213	0.534	0	0.220	1.164	0.871
1389179_at	Cidea	cell death-inducing DFFA-like effector a	uniq_isletLongDR_555	0	4.43	4.04	3.99	4.83	0	-0.390	-1.310	0.269	0	-0.440	-1.357	0.134	0	0.401	1.320	0.495
1389210_at	Lcp1	lymphocyte cytosolic protein 1	uniq_isletLongDR_555	0	6.31	6.27	6.27	6.27	0	-0.047	-1.033	1.030	0	-0.045	-1.032	0.993	0	-0.047	-1.033	1.096
1389227_at	Rhog	ras homolog family member G	uniq_isletLongDR_555	0	7.35	7.51	7.46	7.41	0	0.158	1.116	0.865	0	0.108	1.078	0.977	0	0.059	1.042	1.100
1389351_at	Lrrfip1	leucine rich repeat (in FLII) interacting protein 1	uniq_isletLongDR_555	0	6.58	6.53	6.60	6.60	0	-0.047	-1.033	1.103	0	0.023	1.016	1.136	0	0.014	1.010	1.113
1389359_at	Smc4	structural maintenance of chromosomes 4	uniq_isletLongDR_555	0	5.00	4.98	5.07	4.99	0	-0.026	-1.018	1.135	0	0.062	1.044	1.034	0	-0.013	-1.009	1.169
1389384_at	Ndufaf4	NADH dehydrogenase (ubiquinone) complex I, asser	uniq_isletLongDR_555	0	7.61	7.72	7.72	7.39	0	0.113	1.081	1.031	0	0.116	1.084	0.952	0	-0.220	-1.165	0.758
1389402_at	Csrnp1	cysteine-serine-rich nuclear protein 1	uniq_isletLongDR_555	0	6.19	6.47	6.19	6.42	0	0.280	1.215	0.525	0	0.003	1.002	0.920	0	0.233	1.176	0.782
1389413_at	Evi2a	ecotropic viral integration site 2A	uniq_isletLongDR_555	0	3.48	3.52	3.54	3.63	0	0.049	1.035	1.088	0	0.061	1.043	0.990	0	0.154	1.113	0.967
1389425_at	LOC1003605{	5',3'-nucleotidase, cytosolic	uniq_isletLongDR_555	0	4.90	5.12	4.95	4.69	0	0.220	1.165	0.725	0	0.046	1.032	1.106	0	-0.217	-1.162	0.654
1389538_at	Nfkbia	nuclear factor of kappa light polypeptide gene enha	uniq_isletLongDR_555	0	9.40	9.53	9.05	9.54	0	0.136	1.099	0.920	0	-0.350	-1.275	0.338	0	0.143	1.104	0.974
1389595_at	Dus2l	dihydrouridine synthase 2-like, SMM1 homolog (S. c	uniq_isletLongDR_555	0	6.96	7.07	6.85	6.99	0	0.115	1.083	0.999	0	-0.104	-1.075	0.954	0	0.028	1.020	1.110
1389605_at	Jakmip1	janus kinase and microtubule interacting protein 1	uniq_isletLongDR_555	0	3.48	3.36	3.43	3.49	0	-0.116	-1.084	0.888	0	-0.055	-1.039	0.995	0	0.014	1.010	1.173
1389659_at	Ctla2a	cytotoxic T lymphocyte-associated protein 2 alpha	uniq_isletLongDR_555	0	6.15	5.94	6.01	5.82	0	-0.217	-1.162	0.684	0	-0.146	-1.106	0.856	0	-0.334	-1.260	0.392
1389729_at	Rilpl2	Rab interacting lysosomal protein-like 2	uniq_isletLongDR_555	0	8.80	9.06	8.91	8.67	0	0.263	1.200	0.638	0	0.110	1.079	1.003	0	-0.132	-1.096	0.995
1389732_at	Dram	damage-regulated autophagy modulator	uniq_isletLongDR_555	0	6.79	6.73	6.73	6.80	0	-0.063	-1.045	1.087	0	-0.058	-1.041	1.027	0	0.014	1.010	1.177
1389852_at	Cenpm	centromere protein M	uniq_isletLongDR_555	0	5.25	4.78	5.00	5.16	0	-0.468	-1.383	0.134	0	-0.254	-1.193	0.559	0	-0.093	-1.067	1.062
1389980_at	Cnih4	cornichon homolog 4 (Drosophila)	uniq_isletLongDR_555	0	6.72	7.10	7.01	6.63	0	0.371	1.294	0.423	0	0.285	1.218	0.519	0	-0.093	-1.067	1.097
1389997_at	Cd3e	CD3 molecule, epsilon	uniq_isletLongDR_555	0	3.47	3.43	3.36	3.43	0	-0.037	-1.026	1.145	0	-0.102	-1.073	0.953	0	-0.037	-1.026	1.157
1390034_at	---	---	uniq_isletLongDR_555	0	6.58	6.61	6.70	6.60	0	0.037	1.026	1.153	0	0.124	1.090	0.921	0	0.024	1.017	1.112
1390049_at	Fhl1	four and a half LIM domains 1	uniq_isletLongDR_555	0	4.76	4.75	4.32	4.27	0	-0.014	-1.010	0.946	0	-0.438	-1.354	0.137	0	-0.495	-1.409	0.140
1390195_at	Psd4	pleckstrin and Sec7 domain containing 4	uniq_isletLongDR_555	0	5.20	4.90	4.90	5.05	0	-0.303	-1.233	0.455	0	-0.301	-1.232	0.456	0	-0.151	-1.111	0.975
1390209_at	Syngr3	synaptogyrin 3	uniq_isletLongDR_555	0	5.05	4.67	4.57	4.87	0	-0.381	-1.302	0.259	0	-0.484	-1.398	0.088	0	-0.181	-1.134	0.831
1390282_at	Cyp2s1	cytochrome P450, family 2, subfamily s, polypeptide	uniq_isletLongDR_555	0	4.89	4.92	4.73	4.83	0	0.026	1.018	1.152	0	-0.159	-1.116	0.820	0	-0.065	-1.046	0.871
1390513_at	LOC690130	similar to ras homolog gene family, member f	uniq_isletLongDR_555	0	5.11	4.84	4.87	5.05	0	-0.276	-1.211	0.540	0	-0.246	-1.186	0.575	0	-0.064	-1.045	1.124
1390529_at	Cd83	CD83 molecule	uniq_isletLongDR_555	0	7.20	7.58	7.47	7.33	0	0.386	1.307	0.365	0	0.275	1.210	0.537	0	0.128	1.093	1.026
1390914_at	Fli1	Friend leukemia virus integration 1	uniq_isletLongDR_555	0	6.38	6.31	6.27	6.06	0	-0.065	-1.046	1.007	0	-0.102	-1.074	0.870	0	-0.318	-1.246	0.429
1390948_at	Phemx	pan hematopoietic expression	uniq_isletLongDR_555	0	4.09	3.67	3.79	3.77	0	-0.419	-1.337	0.199	0	-0.302	-1.233	0.418	0	-0.320	-1.248	0.443
1391015_at	Ccdc69	coiled-coil domain containing 69	uniq_isletLongDR_555	0	5.20	4.94	4.94	4.87	0	-0.269	-1.205	0.575	0	-0.265	-1.202	0.537	0	-0.338	-1.264	0.370
1391072_at	Fcer2	Fc fragment of IgE, low affinity II, receptor for (CD23	uniq_isletLongDR_555	0	3.47	3.40	3.44	3.54	0	-0.072	-1.051	1.117	0	-0.026	-1.018	1.144	0	0.077	1.055	0.948
1391112_at	Ggta1p	glycoprotein, alpha-galactosyltransferase 1,3	uniq_isletLongDR_555	0	4.19	4.07	4.21	3.97	0	-0.123	-1.089	0.972	0	0.023	1.016	1.120	0	-0.216	-1.162	0.773
1391214_at	Fam26f	family with sequence similarity 26, member F	uniq_isletLongDR_555	0	3.62	3.29	3.32	3.36	0	-0.322	-1.250	0.461	0	-0.300	-1.231	0.459	0	-0.252	-1.191	0.653
1391323_at	Srprb /// Tf	signal recognition particle receptor, B subunit /// tra	uniq_isletLongDR_555	0	3.53	3.51	3.46	3.56	0	-0.018	-1.013	1.166	0	-0.067	-1.047	1.053	0	0.034	1.024	1.109
1391442_at	---	---	uniq_isletLongDR_555	0	5.34	5.37	5.42	5.37	0	0.028	1.019	1.143	0	0.078	1.055	1.029	0	0.030	1.021	1.082
1391453_at	Ebi3	Epstein-Barr virus induced 3	uniq_isletLongDR_555	0	4.50	4.39	4.53	4.48	0	-0.109	-1.079	1.026	0	0.030	1.021	1.067	0	-0.021	-1.015	1.176
1391612_at	Il22ra2	interleukin 22 receptor, alpha 2	uniq_isletLongDR_555	0	3.25	3.06	3.35	3.00	0	-0.192	-1.143	0.732	0	0.101	1.073	0.933	0	-0.252	-1.190	0.606
1391737_at	Ncf4	neutrophil cytosolic factor 4	uniq_isletLongDR_555	0	4.69	4.58	4.73	4.95	0	-0.112	-1.081	0.964	0	0.041	1.029	1.079	0	0.252	1.191	0.787
1391787_at	Traf3ip3	TRAF3 interacting protein 3	uniq_isletLongDR_555	0	5.19	5.14	5.23	5.20	0	-0.046	-1.032	1.117	0	0.039	1.027	1.122	0	0.008	1.005	1.152
1391797_at	Mef2b	myocyte enhancer factor 2B	uniq_isletLongDR_555	0	5.58	5.47	5.33	5.58	0	-0.113	-1.081	1.003	0	-0.251	-1.190	0.590	0	-0.006	-1.004	1.083
1391835_at	Baz1a	bromodomain adjacent to zinc finger domain, 1A	uniq_isletLongDR_555	0	3.34	3.34	3.24	3.47	0	0.004	1.003	1.096	0	-0.103	-1.074	0.895	0	0.126	1.091	0.987
1391878_at	RGD1560020	similar to Myb proto-oncogene protein (C-myb) (pre	uniq_isletLongDR_555	0	3.14	3.21	3.27	3.11	0	0.070	1.050	1.112	0	0.134	1.097	0.938	0	-0.025	-1.018	1.157
1391948_at	Bcl11b	B-cell CLL/lymphoma 11B (zinc finger protein)	uniq_isletLongDR_555	0	3.81	3.83	3.84	3.90	0	0.020	1.014	1.171	0	0.027	1.019	1.108	0	0.081	1.058	1.081
1392032_at	Grap	GRB2-related adaptor protein	uniq_isletLongDR_555	0	5.37	5.12	5.20	5.21	0	-0.251	-1.190	0.623	0	-0.173	-1.127	0.772	0	-0.159	-1.116	0.903
1392233_at	Cr2	complement component (3d/Epstein Barr virus) rec	uniq_isletLongDR_555	0	3.08	3.04	2.94	3.09	0	-0.035	-1.025	1.114	0	-0.136	-1.099	0.868	0	0.015	1.011	1.177
1392308_at	Pla2g2d	phospholipase A2, group IID	uniq_isletLongDR_555	0	3.58	3.32	3.29	3.75	0	-0.261	-1.198	0.612	0	-0.295	-1.227	0.442	0	0.170	1.125	0.909
1392322_at	Gimap7	GTPase, IMAP family member 7	uniq_isletLongDR_555	0	3.54	3.30	3.27	3.05	0	-0.246	-1.186	0.635	0	-0.275	-1.210	0.523	0	-0.490	-1.404	0.118
1392334_at	RT1-Ba	RT1 class II, locus Ba	uniq_isletLongDR_555	0	7.71	7.31	7.34	7.96	0	-0.398	-1.318	0.269	0	-0.366	-1.289	0.297	0	0.257	1.195	0.783
1392494_at	LOC499331	similar to hypothetical protein D030056L22	uniq_isletLongDR_555	0	7.01	7.00	7.21	6.81	0	-0.006	-1.004	1.175	0	0.195	1.145	0.781	0	-0.204	-1.152	0.829
1392547_at	MGC105649	hypothetical LOC302884	uniq_isletLongDR_555	0	3.70	3.74	3.78	3.86	0	0.035	1.025	1.065	0	0.079	1.056	0.982	0	0.156	1.114	0.935
1392560_at	Pip5k1a	phosphatidylinositol-4-phosphate 5-kinase, type 1,	uniq_isletLongDR_555	0	6.61	6.74	6.81	6.49	0	0.129	1.094	1.004	0	0.197	1.146	0.766	0	-0.117	-1.085	1.047
1392708_at	Stard6	StAR-related lipid transfer (START) domain containir	uniq_isletLongDR_555	0	5.93	5.86	5.88	5.49	0	-0.077	-1.055	1.091	0	-0.051	-1.036	1.086	0	-0.442	-1.358	0.164
1392722_at	Dapp1	dual adaptor of phosphotyrosine and 3-phosphoinc	uniq_isletLongDR_555	0	5.45	5.33	5.39	5.19	0	-0.124	-1.090	0.921	0	-0.067	-1.047	1.029	0	-0.259	-1.196	0.629
1392754_at	Adam8	ADAM metallopeptidase domain 8	uniq_isletLongDR_555	0	5.06	4.89	4.91	4.98	0	-0.170	-1.125	0.781	0	-0.151	-1.111	0.781	0	-0.084	-1.060	1.077
1392976_at	Tpm2	tropomyosin 2, beta	uniq_isletLongDR_555	0	3.76	3.76	3.68	3.84	0	0.002	1.001	1.166	0	-0.077	-1.055	1.059	0	0.074	1.053	1.082
1393049_at	Myo1g	myosin IG	uniq_isletLongDR_555	0	5.77	5.36	5.52	5.50	0	-0.408	-1.327	0.217	0	-0.241	-1.181	0.577	0	-0.265	-1.202	0.565
1393121_at	Agr2	anterior gradient 2 homolog (Xenopus laevis)	uniq_isletLongDR_555	0	3.92	3.46	3.52	3.54	0	-0.454	-1.370	0.154	0	-0.394	-1.314	0.246	0	-0.375	-1.297	0.295
1393144_at	Nmi	N-myc (and STAT) interactor	uniq_isletLongDR_555	0	6.99	6.98	7.15	6.89	0	-0.011	-1.007	1.172	0	0.162	1.119	0.845	0	-0.105	-1.075	1.062
1393171_at	Tmem47	transmembrane protein 47	uniq_isletLongDR_555	0	4.14	4.43	4.03	4.50	0	0.293	1.225	0.562	0	-0.116	-1.084	0.844	0	0.360	1.283	0.567
1393201_at	Lrmp	lymphoid-restricted membrane protein	uniq_isletLongDR_555	0	5.12	4.72	4.88	5.06	0	-0.393	-1.313	0.258	0	-0.240	-1.181	0.622	0	-0.055	-1.039	1.143
1393280_at	Ly86	lymphocyte antigen 86	uniq_isletLongDR_555	0	5.02	4.71	5.07	4.93	0	-0.313	-1.242	0.426	0	0.043	1.030	1.088	0	-0.090	-1.064	1.082
1393297_at	Pou2af1	POU class 2 associating factor 1	uniq_isletLongDR_555	0	3.55	3.37	3.21	3.06	0	-0.180	-1.133	0.802	0	-0.349	-1.273	0.342	0	-0.491	-1.405	0.115
1393315_at	Traf3ip3	TRAF3 interacting protein 3	uniq_isletLongDR_555	0	3.71	3.52	3.75	3.60	0	-0.190	-1.141	0.836	0	0.036	1.025	1.103	0	-0.108	-1.078	1.037
1393319_a_at	Rab17	RAB17, member RAS oncogene family	uniq_isletLongDR_555	0	3.59	3.29	3.16	3.11	0	-0.307	-1.237	0.476	0	-0.428	-1.346	0.171	0	-0.480	-1.395	0.150
1393336_at	Swap70	SWAP switching B-cell complex 70	uniq_isletLongDR_555	0	7.14	6.93	6.80	6.82	0	-0.214	-1.160	0.778	0	-0.337	-1.263	0.376	0	-0.318	-1.247	0.446
1393347_at	Itgal	integrin, alpha L	uniq_isletLongDR_555	0	6.19	5.77	5.95	6.11	0	-0.423	-1.341	0.221	0	-0.239	-1.180	0.657	0	-0.082	-1.058	1.123
1393379_a_at	Blk	B lymphoid tyrosine kinase	uniq_isletLongDR_555	0	4.52	4.37	4.32	4.56	0	-0.145	-1.106	0.958	0	-0.194	-1.144	0.759	0	0.048	1.034	1.099
1393400_at	Zap70	zeta-chain (TCR) associated protein kinase 70	uniq_isletLongDR_555	0	5.79	5.48	5.55	5.46	0	-0.311	-1.240	0.474	0	-0.233	-1.175	0.666	0	-0.325	-1.253	0.421
1393460_at	Lrrc33	leucine rich repeat containing 33	uniq_isletLongDR_555	0	6.61	6.53	6.45	6.66	0	-0.084	-1.060	1.093	0	-0.161	-1.118	0.864	0	0.051	1.036	1.099
1393654_at	Neil1	nei endonuclease VIII-like 1 (E. coli)	uniq_isletLongDR_555	0	6.50	6.78	6.75	6.79	0	0.277	1.212	0.616	0	0.246	1.186	0.615	0	0.293	1.225	0.718
1393663_at	Slc36a2	solute carrier family 36 (proton/amino acid symport	uniq_isletLongDR_555	0	4.63	4.31	4.40	4.52	0	-0.320	-1.249	0.461	0	-0.238	-1.179	0.639	0	-0.116	-1.084	1.047
1393669_at	LOC1003637{	RAB1B, member RAS oncogene family-like /// RAB1/	uniq_isletLongDR_555	0	6.07	6.02	6.25	5.87	0	-0.053	-1.038	0.942	0	0.182	1.134	0.814	0	-0.205	-1.152	0.791
1393682_at	Gpr34	G protein-coupled receptor 34	uniq_isletLongDR_555	0	2.84	2.70	2.78	2.73	0	-0.137	-1.100	0.870	0	-0.061	-1.043	1.053	0	-0.109	-1.078	1.042
1393688_at	LOC685048 /	similar to paired immunoglobin-like type 2 receptor	uniq_isletLongDR_555	0	3.92	3.83	4.07	4.13	0	-0.088	-1.063	1.080	0	0.149	1.109	0.902	0	0.206	1.154	0.887
1393708_at	Bhlhe22	basic helix-loop-helix family, member e22	uniq_isletLongDR_555	0	3.24	2.93	2.94	3.12	0	-0.307	-1.237	0.418	0	-0.297	-1.229	0.413	0	-0.113	-1.082	1.019
1393755_at	Pik3cd	phosphatidylinositol-4,5-bisphosphate 3-kinase, cat	uniq_isletLongDR_555	0	4.80	4.69	4.78	4.79	0	-0.109	-1.078	0.938	0	-0.015	-1.010	1.111	0	-0.010	-1.007	1.171
1393863_at	Cd180	CD180 molecule	uniq_isletLongDR_555	0	2.86	2.80	2.71	2.77	0	-0.058	-1.041	1.085	0	-0.149	-1.109	0.826	0	-0.094	-1.067	1.084
1393926_at	Stap1	signal transducing adaptor family member 1	uniq_isletLongDR_555	0	3.74	3.68	3.65	3.71	0	-0.060	-1.042	1.063	0	-0.092	-1.066	0.984	0	-0.031	-1.022	1.124
1393929_at	Ccr6	chemokine (C-C motif) receptor 6	uniq_isletLongDR_555	0	4.66	4.31	4.40	4.34	0	-0.349	-1.273	0.390	0	-0.260	-1.198	0.558	0	-0.323	-1.251	0.417
1394051_at	P2ry10	purinergic receptor P2Y, G-protein coupled, 10	uniq_isletLongDR_555	0	2.68	2.62	2.70	2.75	0	-0.051	-1.036	1.098	0	0.029	1.020	1.131	0	0.074	1.053	1.087
1394306_at	Zap70	zeta-chain (TCR) associated protein kinase 70	uniq_isletLongDR_555	0	5.81	5.54	5.58	5.84	0	-0.269	-1.205	0.606	0	-0.238	-1.179	0.656	0	0.023	1.016	1.093
1394480_at	LOC1003023	hypothetical protein LOC100302372	uniq_isletLongDR_555	0	5.54	5.31	5.33	5.50	0	-0.232	-1.174	0.647	0	-0.210	-1.157	0.709	0	-0.033	-1.023	1.163
1394501_at	Siglec10	sialic acid binding Ig-like lectin 10	uniq_isletLongDR_555	0	3.75	3.63	3.61	3.87	0	-0.122	-1.088	0.979	0	-0.144	-1.105	0.830	0	0.115	1.083	1.012
1394523_at	Plbd1	phospholipase B domain containing 1	uniq_isletLongDR_555	0	5.74	5.39	5.45	5.80	0	-0.344	-1.269	0.377	0	-0.288	-1.221	0.450	0	0.059	1.041	1.052
1394673_at	Cd33	CD33 molecule	uniq_isletLongDR_555	0	4.99	4.73	4.74	4.95	0	-0.264	-1.200	0.600	0	-0.250	-1.189	0.596	0	-0.044	-1.031	1.030
1394678_at	Fgd2	FYVE, RhoGEF and PH domain containing 2	uniq_isletLongDR_555	0	5.46	5.44	5.60	5.31	0	-0.028	-1.020	1.026	0	0.135	1.098	0.809	0	-0.150	-1.110	0.886
1394699_at	---	---	uniq_isletLongDR_555	0	5.03	4.54	4.61	5.09	0	-0.483	-1.398	0.129	0	-0.415	-1.334	0.231	0	0.063	1.045	1.070
1394750_at	Fhl1	four and a half LIM domains 1	uniq_isletLongDR_555	0	4.17	4.01	4.11	4.06	0	-0.163	-1.120	0.831	0	-0.059	-1.042	1.035	0	-0.108	-1.078	1.062
1394819_at	Sp110	SP110 nuclear body protein	uniq_isletLongDR_555	0	5.67	5.93	5.93	5.69	0	0.259	1.197	0.661	0	0.265	1.202	0.576	0	0.022	1.015	1.110
1395116_at	Cd8a	CD8a molecule	uniq_isletLongDR_555	0	3.95	3.68	3.76	3.85	0	-0.274	-1.209	0.569	0	-0.186	-1.137	0.736	0	-0.101	-1.072	1.071
1395301_x_at	LOC1009108(	paired immunoglobulin-like type 2 receptor alpha-li	uniq_isletLongDR_555	0	5.25	5.04	5.11	5.22	0	-0.207	-1.154	0.716	0	-0.141	-1.103	0.804	0	-0.028	-1.020	1.154
1395313_s_at	Anxa3	annexin A3	uniq_isletLongDR_555	0	6.62	6.24	6.29	6.82	0	-0.375	-1.297	0.329	0	-0.326	-1.253	0.411	0	0.202	1.150	0.902
1395394_at	Gga2	golgi associated, gamma adaptin ear containing, ARI	uniq_isletLongDR_555	0	5.34	5.69	4.95	4.88	0	0.347	1.272	0.408	0	-0.385	-1.306	0.278	0	-0.463	-1.379	0.198
1395635_at	Tnfrsf26	tumor necrosis factor receptor superfamily, membe	uniq_isletLongDR_555	0	4.19	4.03	4.16	4.30	0	-0.159	-1.116	0.839	0	-0.029	-1.020	1.070	0	0.110	1.079	0.963
1395697_at	Ezh2	enhancer of zeste homolog 2 (Drosophila)	uniq_isletLongDR_555	0	5.39	5.45	5.42	5.04	0	0.062	1.044	1.097	0	0.033	1.023	1.102	0	-0.350	-1.274	0.354
1395960_at	LOC688276	similar to epidermodysplasia verruciformis 2	uniq_isletLongDR_555	0	4.08	3.85	3.91	3.82	0	-0.232	-1.175	0.645	0	-0.171	-1.126	0.831	0	-0.267	-1.203	0.600
1396035_at	Sbspon	somatomedin B and thrombospondin, type 1 domai	uniq_isletLongDR_555	0	4.52	4.14	4.12	4.08	0	-0.380	-1.301	0.307	0	-0.399	-1.319	0.225	0	-0.444	-1.361	0.164
1396036_at	Ralgps2	Ral GEF with PH domain and SH3 binding motif 2	uniq_isletLongDR_555	0	4.98	5.28	5.06	4.97	0	0.301	1.232	0.554	0	0.079	1.056	0.906	0	-0.014	-1.010	1.149
1396132_at	Fcrla	Fc receptor-like A	uniq_isletLongDR_555	0	3.67	3.69	3.60	3.44	0	0.014	1.010	1.171	0	-0.076	-1.054	1.049	0	-0.230	-1.173	0.727

Affymetrix Probeset ID	Gene Symbol	Gene Title	Figure 7 A Venn n=2289	Present in any 2 analyses n=423 1=yes 0=no	Mean log2 intensity DR+/+ ND	Mean log2 intensity DR+/+ HCD	Mean log2 intensity DR+/+ B/S	Mean log2 intensity F+/+ day 40 ND	DR+/+HCD vs DR+/+ND FDR<10% Log2 ratio >0.5 n=636	Log2 ratio DR+/+HCD vs DR+/+ ND	Fold-Change DR+/+HCD vs DR+/+ ND	FDR DR+/+HCD vs DR+/+ ND	DR+/+B/S vs DR+/+ND Log2 ratio >0.5 n=350	Log2 ratio DR+/+ B/S vs DR+/+ ND	Fold-Change DR+/+B/S vs DR+/+ ND	FDR DR+/+B/S vs DR+/+ ND	F+/+ vs DR+/+ND Log2 ratio >0.5 n=1209	Log2 ratio F+/+ vs DR+/+ ND	Fold-Change F+/+ vs DR+/+ ND	FDR F+/+ vs DR+/+ ND
1396231_at	Rasal3	RAS protein activator like 3	uniq_isletLongDR_555	0	4.11	3.73	3.90	4.13	0	-0.383	-1.304	0.291	0	-0.210	-1.157	0.723	0	0.015	1.011	1.172
1396268_at	Sp110	SP110 nuclear body protein	uniq_isletLongDR_555	0	6.14	6.35	6.30	6.13	0	0.206	1.154	0.727	0	0.161	1.118	0.751	0	-0.013	-1.009	1.146
1397335_at	Sema3d	sema domain, immunoglobulin domain (Ig), short ba	uniq_isletLongDR_555	0	3.54	3.55	3.71	3.55	0	0.006	1.004	1.140	0	0.166	1.122	0.774	0	0.009	1.006	1.167
1397411_at	Fgd2	FYVE, RhoGEF and PH domain containing 2	uniq_isletLongDR_555	0	5.23	5.23	5.05	5.19	0	0.001	1.001	1.153	0	-0.173	-1.128	0.826	0	-0.042	-1.029	1.157
1397507_at	Apbb1ip	amyloid beta (A4) precursor protein-binding, family	uniq_isletLongDR_555	0	2.64	2.58	2.65	2.73	0	-0.063	-1.044	1.110	0	0.007	1.005	1.142	0	0.085	1.061	1.063
1397548_at	Hmha1	histocompatibility (minor) HA-1	uniq_isletLongDR_555	0	5.37	5.13	5.29	5.33	0	-0.241	-1.182	0.659	0	-0.082	-1.058	0.923	0	-0.037	-1.026	1.163
1397866_at	Serpinb6b	serine (or cysteine) peptidase inhibitor, clade B, mer	uniq_isletLongDR_555	0	4.80	4.53	4.62	4.64	0	-0.273	-1.208	0.591	0	-0.185	-1.137	0.784	0	-0.158	-1.116	0.956
1398246_s_at	Fcgr2a /// LO	Fc fragment of IgG, low affinity IIa, receptor /// low	uniq_isletLongDR_555	0	5.56	5.59	5.89	5.56	0	0.027	1.019	1.071	0	0.334	1.261	0.396	0	0.004	1.003	1.124
1398272_at	B4galnt1	beta-1,4-N-acetyl-galactosaminyl transferase 1	uniq_isletLongDR_555	0	3.96	3.84	3.80	4.14	0	-0.120	-1.087	0.920	0	-0.158	-1.116	0.812	0	0.181	1.134	0.943
1398282_at	Kynu	kynureninase	uniq_isletLongDR_555	0	4.27	4.12	3.97	4.31	0	-0.149	-1.109	0.962	0	-0.296	-1.228	0.485	0	0.040	1.028	1.108
1398297_at	Mapk12	mitogen-activated protein kinase 12	uniq_isletLongDR_555	0	6.77	6.99	6.95	6.28	0	0.222	1.166	0.689	0	0.184	1.136	0.797	0	-0.495	-1.409	0.107
1398356_at	Nudt21	nudix (nucleoside diphosphate linked moiety X)-typ	uniq_isletLongDR_555	0	8.06	8.47	8.36	8.26	0	0.415	1.333	0.345	0	0.300	1.232	0.487	0	0.202	1.150	0.906
1398405_at	Sept6	septin 6	uniq_isletLongDR_555	0	4.75	4.72	4.47	5.09	0	-0.039	-1.027	1.154	0	-0.286	-1.219	0.520	0	0.332	1.259	0.634
1398602_at	Mad2l1	MAD2 mitotic arrest deficient-like 1 (yeast)	uniq_isletLongDR_555	0	5.95	5.91	6.06	5.73	0	-0.040	-1.028	1.086	0	0.105	1.076	1.010	0	-0.220	-1.165	0.752
1398641_at	Plekha3	pleckstrin homology domain-containing, family A (p	uniq_isletLongDR_555	0	7.05	7.28	7.18	7.19	0	0.227	1.171	0.716	0	0.129	1.093	0.919	0	0.137	1.100	0.986
1399152_at	Eps15	epidermal growth factor receptor pathway substrat	uniq_isletLongDR_555	0	6.23	6.48	6.30	6.07	0	0.242	1.183	0.660	0	0.066	1.047	1.048	0	-0.165	-1.121	0.911
1367555_at	Alb	albumin	uniq_RT1uvsDR_883	0	3.99	3.79	3.85	5.29	0	-0.199	-1.148	0.643	0	-0.144	-1.105	0.756	1	1.302	2.466	0.011
1367562_at	Sparc	secreted protein, acidic, cysteine-rich (osteonectin)	uniq_RT1uvsDR_883	0	8.60	8.28	8.41	8.04	0	-0.321	-1.249	0.392	0	-0.192	-1.142	0.657	1	-0.560	-1.474	0.066
1367563_at	Sparc	secreted protein, acidic, cysteine-rich (osteonectin)	uniq_RT1uvsDR_883	0	11.14	11.15	11.03	10.46	0	0.009	1.007	1.157	0	-0.110	-1.079	0.959	1	-0.675	-1.597	0.022
1367612_at	Mgst1	microsomal glutathione S-transferase 1	uniq_RT1uvsDR_883	0	6.61	6.44	6.59	5.35	0	-0.169	-1.124	0.852	0	-0.019	-1.013	1.105	1	-1.258	-2.391	0.000
1367705_at	Glrx	glutaredoxin (thioltransferase)	uniq_RT1uvsDR_883	0	7.90	7.83	8.00	8.71	0	-0.074	-1.052	1.050	0	0.098	1.071	1.006	1	0.813	1.757	0.060
1367706_at	LOC683062 ///	similar to voltage-dependent anion channel 1 /// vo	uniq_RT1uvsDR_883	0	10.53	10.75	10.83	9.99	0	0.215	1.161	0.762	0	0.296	1.228	0.510	1	-0.544	-1.458	0.074
1367811_at	Phgdh	phosphoglycerate dehydrogenase	uniq_RT1uvsDR_883	0	5.76	6.24	6.19	6.66	0	0.489	1.403	0.207	0	0.431	1.348	0.246	1	0.902	1.868	0.042
1367838_at	Cth	cystathionase (cystathionine gamma-lyase)	uniq_RT1uvsDR_883	0	3.66	3.85	3.52	4.53	0	0.193	1.143	0.773	0	-0.133	-1.097	0.881	1	0.871	1.828	0.075
1367860_a_at	Mmp14	matrix metallopeptidase 14 (membrane-inserted)	uniq_RT1uvsDR_883	0	7.77	7.60	7.59	7.26	0	-0.167	-1.123	0.798	0	-0.175	-1.129	0.789	1	-0.508	-1.422	0.093
1367880_at	Lamb2	laminin, beta 2	uniq_RT1uvsDR_883	0	7.61	7.32	7.20	6.80	0	-0.291	-1.224	0.450	0	-0.410	-1.329	0.202	1	-0.812	-1.756	0.007
1367900_at	Gyg1	glycogenin 1	uniq_RT1uvsDR_883	0	8.65	8.96	8.86	8.08	0	0.310	1.239	0.519	0	0.208	1.155	0.746	1	-0.568	-1.482	0.055
1367961_at	Klk1	kallikrein 1	uniq_RT1uvsDR_883	0	8.05	8.48	8.08	8.89	0	0.427	1.345	0.325	0	0.034	1.024	1.021	1	0.839	1.788	0.072
1368008_at	Prom1	prominin 1	uniq_RT1uvsDR_883	0	9.18	9.08	9.30	8.33	0	-0.092	-1.066	1.075	0	0.125	1.090	0.972	1	-0.842	-1.793	0.006
1368089_at	Pde2a	phosphodiesterase 2A, cGMP-stimulated	uniq_RT1uvsDR_883	0	7.93	7.76	7.84	7.01	0	-0.174	-1.128	0.864	0	-0.092	-1.066	1.029	1	-0.922	-1.894	0.003
1368104_at	Tspan2	tetraspanin 2	uniq_RT1uvsDR_883	0	8.01	7.96	7.99	7.24	0	-0.046	-1.032	1.121	0	-0.017	-1.012	1.149	1	-0.768	-1.703	0.009
1368115_at	Cldn3	claudin 3	uniq_RT1uvsDR_883	0	7.89	7.70	7.61	7.33	0	-0.193	-1.143	0.817	0	-0.279	-1.213	0.530	1	-0.564	-1.478	0.064
1368124_at	Dusp5	dual specificity phosphatase 5	uniq_RT1uvsDR_883	0	6.51	6.98	6.90	7.32	0	0.473	1.388	0.273	0	0.391	1.311	0.300	1	0.815	1.759	0.073
1368145_at	Pcp4	Purkinje cell protein 4	uniq_RT1uvsDR_883	0	9.95	9.79	9.95	8.41	0	-0.155	-1.114	0.885	0	0.000	-1.000	1.150	1	-1.533	-2.893	0.000
1368171_at	Lox	lysyl oxidase	uniq_RT1uvsDR_883	0	6.94	6.84	6.65	6.28	0	-0.104	-1.075	0.968	0	-0.295	-1.227	0.432	1	-0.658	-1.578	0.024
1368172_a_at	Lox	lysyl oxidase	uniq_RT1uvsDR_883	0	7.27	7.05	6.92	5.82	0	-0.224	-1.168	0.648	0	-0.349	-1.274	0.295	1	-1.454	-2.739	0.000
1368202_a_at	Dab2	disabled 2, mitogen-responsive phosphoprotein	uniq_RT1uvsDR_883	0	7.83	7.42	7.43	7.26	0	-0.404	-1.323	0.221	0	-0.399	-1.319	0.219	1	-0.570	-1.484	0.053
1368249_at	Klf15	Kruppel-like factor 15	uniq_RT1uvsDR_883	0	5.88	6.15	6.17	6.64	0	0.270	1.206	0.584	0	0.290	1.223	0.519	1	0.760	1.693	0.083
1368259_at	Ptgs1	prostaglandin-endoperoxide synthase 1	uniq_RT1uvsDR_883	0	5.28	5.47	5.21	6.80	0	0.195	1.145	0.794	0	-0.063	-1.044	0.938	1	1.529	2.886	0.006
1368272_at	Got1	glutamic-oxaloacetic transaminase 1, soluble (aspar	uniq_RT1uvsDR_883	0	8.13	8.22	8.07	7.58	0	0.095	1.068	1.074	0	-0.054	-1.038	1.025	1	-0.548	-1.462	0.066
1368289_at	Gc	group specific component	uniq_RT1uvsDR_883	0	6.57	6.81	6.52	7.73	0	0.244	1.184	0.670	0	-0.046	-1.032	1.020	1	1.166	2.244	0.016
1368342_at	Ampd3	adenosine monophosphate deaminase 3	uniq_RT1uvsDR_883	0	6.18	6.29	6.05	5.56	0	0.110	1.080	0.989	0	-0.129	-1.094	0.767	1	-0.625	-1.543	0.035
1368379_at	Scarb2	scavenger receptor class B, member 2	uniq_RT1uvsDR_883	0	7.60	7.81	7.71	7.02	0	0.207	1.154	0.764	0	0.111	1.080	0.995	1	-0.585	-1.500	0.046
1368416_at	Ibsp	integrin-binding sialoprotein	uniq_RT1uvsDR_883	0	2.78	2.83	2.65	3.56	0	0.051	1.036	1.052	0	-0.127	-1.092	0.924	1	0.782	1.720	0.074
1368441_at	Msln	mesothelin	uniq_RT1uvsDR_883	0	13.30	13.42	13.40	12.48	0	0.120	1.087	1.015	0	0.098	1.071	1.025	1	-0.828	-1.776	0.006
1368450_at	Myo5a	myosin VA	uniq_RT1uvsDR_883	0	8.77	9.14	9.13	7.97	0	0.365	1.288	0.441	0	0.358	1.282	0.364	1	-0.805	-1.748	0.008
1368502_at	Gast	gastrin	uniq_RT1uvsDR_883	0	6.80	6.56	7.20	5.97	0	-0.248	-1.188	0.656	0	0.394	1.314	0.305	1	-0.838	-1.788	0.007
1368505_at	Rgs4	regulator of G-protein signaling 4	uniq_RT1uvsDR_883	0	9.98	10.18	10.24	9.14	0	0.201	1.149	0.805	0	0.261	1.198	0.599	1	-0.833	-1.782	0.006
1368522_at	Timeless	timeless circadian clock	uniq_RT1uvsDR_883	0	4.72	4.27	4.45	4.12	0	-0.450	-1.366	0.138	0	-0.266	-1.202	0.526	1	-0.596	-1.511	0.064
1368536_at	Enpp2	ectonucleotide pyrophosphatase/phosphodiesteras	uniq_RT1uvsDR_883	0	8.43	8.39	8.67	7.37	0	-0.047	-1.033	1.111	0	0.241	1.182	0.662	1	-1.063	-2.089	0.001
1368539_at	Scn9a	sodium channel, voltage-gated, type IX, alpha	uniq_RT1uvsDR_883	0	8.31	8.87	8.39	7.67	0	0.558	1.472	0.137	0	0.071	1.051	1.070	1	-0.647	-1.566	0.031
1368540_at	Tpbg	trophoblast glycoprotein	uniq_RT1uvsDR_883	0	4.05	4.42	4.51	3.48	0	0.365	1.288	0.407	0	0.453	1.369	0.220	1	-0.573	-1.487	0.078
1368565_at	Slc1a3	solute carrier family 1 (glial high affinity glutamate t	uniq_RT1uvsDR_883	0	4.66	4.96	4.73	5.80	0	0.297	1.229	0.565	0	0.070	1.050	1.033	1	1.142	2.207	0.018
1368589_at	Ptprj	protein tyrosine phosphatase, receptor type, J	uniq_RT1uvsDR_883	0	9.33	9.34	9.44	8.52	0	0.009	1.007	1.174	0	0.110	1.079	0.964	1	-0.807	-1.750	0.007
1368658_at	Cntf	ciliary neurotrophic factor	uniq_RT1uvsDR_883	0	5.58	5.81	5.99	6.50	0	0.226	1.170	0.710	0	0.404	1.323	0.285	1	0.914	1.885	0.041
1368725_at	Jag1	jagged 1	uniq_RT1uvsDR_883	0	6.21	5.77	5.95	5.30	0	-0.438	-1.355	0.166	0	-0.260	-1.197	0.496	1	-0.906	-1.874	0.004
1368752_at	Tacr3	tachykinin receptor 3	uniq_RT1uvsDR_883	0	5.56	5.49	5.68	6.38	0	-0.070	-1.050	1.026	0	0.119	1.086	0.828	1	0.821	1.766	0.068
1368770_at	Gcnt1	glucosaminyl (N-acetyl) transferase 1, core 2	uniq_RT1uvsDR_883	0	5.66	5.45	5.80	4.84	0	-0.218	-1.163	0.632	0	0.138	1.100	0.859	1	-0.826	-1.773	0.009
1368778_at	Slc6a6	solute carrier family 6 (neurotransmitter transporte	uniq_RT1uvsDR_883	0	9.05	8.87	9.23	8.48	0	-0.177	-1.130	0.852	0	0.178	1.131	0.824	1	-0.564	-1.479	0.063
1368798_at	Abol1	ABO blood group-like 1	uniq_RT1uvsDR_883	0	4.03	3.60	3.68	3.28	0	-0.432	-1.349	0.154	0	-0.353	-1.277	0.261	1	-0.750	-1.682	0.013
1368826_at	Comt	catechol-O-methyltransferase	uniq_RT1uvsDR_883	0	11.20	11.30	11.37	9.73	0	0.097	1.070	1.067	0	0.166	1.122	0.877	1	-1.468	-2.765	0.000
1368977_a_at	Timm10b	translocase of inner mitochondrial membrane 10 hc	uniq_RT1uvsDR_883	0	7.10	7.07	7.17	6.58	0	-0.032	-1.023	1.124	0	0.069	1.049	1.085	1	-0.523	-1.437	0.093
1369000_at	Ntrk1	neurotrophic tyrosine kinase, receptor, type 1	uniq_RT1uvsDR_883	0	8.76	9.04	8.90	7.49	0	0.281	1.215	0.564	0	0.132	1.096	0.940	1	-1.269	-2.410	0.000
1369094_a_at	Ptprs	protein tyrosine phosphatase, receptor type, S	uniq_RT1uvsDR_883	0	6.36	5.87	6.19	5.51	0	-0.493	-1.407	0.085	0	-0.173	-1.128	0.434	1	-0.853	-1.807	0.007
1369104_at	Prkaa1	protein kinase, AMP-activated, alpha 1 catalytic sub	uniq_RT1uvsDR_883	0	3.63	4.02	3.63	5.68	0	0.388	1.309	0.382	0	0.003	1.002	1.123	1	2.052	4.147	0.003
1369113_at	Grem1	gremlin 1	uniq_RT1uvsDR_883	0	6.19	5.73	6.15	5.38	0	-0.453	-1.369	0.128	0	-0.034	-1.024	0.975	1	-0.810	-1.753	0.009
1369182_at	F3	coagulation factor III (thromboplastin, tissue factor)	uniq_RT1uvsDR_883	0	9.18	9.19	9.16	7.11	0	0.016	1.011	1.170	0	-0.014	-1.010	1.111	1	-2.064	-4.180	0.000
1369196_at	Ppy	pancreatic polypeptide	uniq_RT1uvsDR_883	0	14.17	14.27	14.17	13.39	0	0.103	1.074	1.054	0	0.000	1.000	1.156	1	-0.774	-1.710	0.009
1369210_at	Scn1a	sodium channel, voltage-gated, type I, alpha	uniq_RT1uvsDR_883	0	3.50	3.45	3.44	2.85	0	-0.047	-1.033	1.103	0	-0.057	-1.040	1.053	1	-0.649	-1.568	0.026
1369351_at	Cntn3	contactin 3 (plasmacytoma associated)	uniq_RT1uvsDR_883	0	7.03	7.41	7.45	8.01	0	0.379	1.301	0.383	0	0.418	1.336	0.267	1	0.979	1.971	0.031
1369407_at	Tnfrsf11b	tumor necrosis factor receptor superfamily, membe	uniq_RT1uvsDR_883	0	9.57	9.25	9.43	8.51	0	-0.320	-1.249	0.415	0	-0.136	-1.099	0.838	1	-1.055	-2.077	0.001
1369422_at	Fap	fibroblast activation protein, alpha	uniq_RT1uvsDR_883	0	4.66	4.51	4.58	4.11	0	-0.151	-1.110	0.940	0	-0.080	-1.057	1.039	1	-0.544	-1.458	0.074
1369602_at	Fgf17	fibroblast growth factor 17	uniq_RT1uvsDR_883	0	4.35	4.12	4.14	3.85	0	-0.230	-1.173	0.695	0	-0.207	-1.154	0.729	1	-0.505	-1.419	0.100
1369679_a_at	Nfia	nuclear factor I/A	uniq_RT1uvsDR_883	0	8.16	7.91	8.00	7.41	0	-0.255	-1.194	0.649	0	-0.163	-1.120	0.750	1	-0.756	-1.689	0.010
1369691_at	Scn3a	sodium channel, voltage-gated, type III, alpha	uniq_RT1uvsDR_883	0	8.75	8.76	8.70	9.65	0	0.004	1.003	1.122	0	-0.052	-1.037	1.045	1	0.897	1.862	0.042
1369692_at	Tnr	tenascin R	uniq_RT1uvsDR_883	0	5.41	5.82	5.66	4.77	0	0.407	1.326	0.325	0	0.255	1.194	0.604	1	-0.643	-1.561	0.028
1369737_at	Crem	cAMP responsive element modulator	uniq_RT1uvsDR_883	0	5.30	5.23	5.20	6.26	0	-0.063	-1.045	0.958	0	-0.098	-1.071	0.801	1	0.966	1.954	0.042
1369769_at	Kcne1	potassium voltage-gated channel, Isk-related family	uniq_RT1uvsDR_883	0	4.52	4.72	4.21	5.36	0	0.202	1.150	0.746	0	-0.311	-1.241	0.425	1	0.841	1.791	0.056
1369773_at	Bmp3	bone morphogenetic protein 3	uniq_RT1uvsDR_883	0	6.01	6.55	6.25	4.94	0	0.545	1.459	0.163	0	0.239	1.180	0.571	1	-1.073	-2.104	0.001
1369903_at	Gabrb3	gamma-aminobutyric acid (GABA) A receptor, beta 3	uniq_RT1uvsDR_883	0	6.27	6.80	6.22	7.30	0	0.527	1.441	0.200	0	-0.048	-1.033	0.969	1	1.033	2.047	0.026
1369951_at	Ctrb1	chymotrypsinogen B1	uniq_RT1uvsDR_883	0	12.73	13.08	12.38	13.86	0	0.352	1.276	0.429	0	-0.351	-1.275	0.314	1	1.128	2.185	0.018
1369960_at	Fxyd1	FXYD domain-containing ion transport regulator 1	uniq_RT1uvsDR_883	0	6.54	6.35	6.21	5.99	0	-0.188	-1.139	0.786	0	-0.330	-1.257	0.395	1	-0.545	-1.459	0.068
1369964_at	Coro1a	coronin, actin binding protein 1A	uniq_RT1uvsDR_883	0	6.35	6.37	6.12	5.53	0	0.018	1.012	1.039	0	-0.227	-1.170	0.502	1	-0.821	-1.767	0.009
1369965_at	Mlh1	mutL homolog 1, colon cancer, nonpolyposis type 2	uniq_RT1uvsDR_883	0	7.07	7.07	7.22	6.29	0	0.001	1.001	1.174	0	0.150	1.109	0.906	1	-0.780	-1.717	0.009
1369968_at	Ptn	pleiotrophin	uniq_RT1uvsDR_883	0	9.58	9.26	9.44	8.96	0	-0.323	-1.251	0.411	0	-0.140	-1.102	0.769	1	-0.622	-1.539	0.034
1369989_at	Pnpo	pyridoxine 5'-phosphate oxidase	uniq_RT1uvsDR_883	0	7.93	8.04	8.08	7.23	0	0.104	1.075	1.063	0	0.150	1.109	0.881	1	-0.705	-1.630	0.016
1370016_at	Nell2	NEL-like 2 (chicken)	uniq_RT1uvsDR_883	0	6.96	6.97	7.17	6.40	0	0.016	1.011	1.170	0	0.217	1.162	0.723	1	-0.552	-1.466	0.076
1370018_at	Hspb2	heat shock protein beta 2	uniq_RT1uvsDR_883	0	4.25	4.02	4.08	3.67	0	-0.234	-1.176	0.673	0	-0.175	-1.129	0.781	1	-0.580	-1.495	0.061
1370107_at	Ctrl	chymotrypsin-like	uniq_RT1uvsDR_883	0	11.28	11.35	10.87	12.46	0	0.068	1.048	1.042	0	-0.412	-1.331	0.209	1	1.171	2.251	0.016
1370182_at	Ptprn2	protein tyrosine phosphatase, receptor type, N poly	uniq_RT1uvsDR_883	0	9.53	9.40	9.62	11.25	0	-0.125	-1.090	0.950	0	0.097	1.070	1.043	1	1.721	3.296	0.006
1370247_a_at	Pmp22	peripheral myelin protein 22	uniq_RT1uvsDR_883	0	7.36	6.87	6.94	6.31	0	-0.493	-1.408	0.105	0	-0.424	-1.341	0.173	1	-1.049	-2.070	0.001
1370257_at	Pla2g1b	phospholipase A2, group IB, pancreas	uniq_RT1uvsDR_883	0	11.18	11.58	10.73	12.03	0	0.398	1.318	0.377	0	-0.456	-1.372	0.130	1	0.849	1.801	0.059
1370301_at	Mmp2	matrix metallopeptidase 2	uniq_RT1uvsDR_883	0	6.01	5.71	5.68	5.39	0	-0.300	-1.231	0.437	0	-0.328	-1.255	0.340	1	-0.625	-1.542	0.050
1370312_at	Spon1	spondin 1, extracellular matrix protein	uniq_RT1uvsDR_883	0	7.72	7.71	7.82	5.88	0	-0.009	-1.006	1.178	0	0.100	1.072	1.032	1	-1.839	-3.578	0.000
1370334_at	Plekhb1	pleckstrin homology domain containing, family B (ev	uniq_RT1uvsDR_883	0	7.95	8.31	8.13	6.92	0	0.364	1.287	0.378	0	0.178	1.131	0.782	1	-1.026	-2.036	0.001
1370344_at	Hspa4	heat shock protein 4	uniq_RT1uvsDR_883	0	8.27	8.19	8.37	7.70	0	-0.076	-1.054	1.061	0	0.099	1.071	1.031	1	-0.573	-1.487	0.051
1370380_s_at	Nr2f6	nuclear receptor subfamily 2, group F, member 6	uniq_RT1uvsDR_883	0	5.18	4.86	4.81	4.66	0	-0.326	-1.254	0.435	0	-0.370	-1.292	0.297	1	-0.520	-1.434	0.090
1370442_at	Tmsbl1	thymosin beta-like protein 1	uniq_RT1uvsDR_883	0	12.11	11.86	12.17	11.60	0	-0.246	-1.186	0.595	0	0.057	1.040	1.092	1	-0.512	-1.426	0.097
1370517_at	Nptx1	neuronal pentraxin I	uniq_RT1uvsDR_883	0	4.81	4.74	5.09	6.00	0	-0.065	-1.046	1.050	0	0.281	1.215	0.542	1	1.196	2.291	0.014
1370522_at	Gcgr	glucagon receptor	uniq_RT1uvsDR_883	0	7.72	7.59	7.60	8.51	0	-0.128	-1.093	0.974	0	-0.114	-1.083	0.942	1	0.788	1.727	0.070
1370606_at	P2ry1	purinergic receptor P2Y, G-protein coupled, 1	uniq_RT1uvsDR_883	0	9.10	9.26	8.92	10.09	0	0.160	1.118	0.917	0	-0.182	-1.135	0.676	1	0.994	1.991	0.029
1370642_s_at	Pdgfrb	platelet derived growth factor receptor, beta polype	uniq_RT1uvsDR_883	0	7.23	6.88	7.07	6.72	0	-0.355	-1.279	0.259	0	-0.166	-1.122	0.728	1	-0.509	-1.424	0.096
1370687_a_at	Ntrk2	neurotrophic tyrosine kinase, receptor, type 2	uniq_RT1uvsDR_883	0	8.65	8.60	8.44	7.97	0	-0.054	-1.038	1.101	0	-0.207	-1.154	0.670	1	-0.679	-1.601	0.021
1370806_at	Retsat	retinol saturase (all trans retinol 13,14 reductase)	uniq_RT1uvsDR_883	0	8.54	8.83	8.61	7.30	0	0.289	1.222	0.583	0	0.072	1.051	1.078	1	-1.234	-2.352	0.000
1370835_at	Tox4	TOX high mobility group box family member 4	uniq_RT1uvsDR_883	0	5.57	5.62	5.55	6.39	0	0.048	1.034	1.115	0	-0.021	-1.015	1.141	1	0.813	1.757	0.065
1370884_at	Spr	sepiapterin reductase (7,8-dihydrobiopterin:NADP+	uniq_RT1uvsDR_883	0	7.11	7.04	7.07	6.47	0	-0.070	-1.049	1.073	0	-0.040	-1.028	1.109	1	-0.642	-1.561	0.028
1370894_at	Cldn7	claudin 7	uniq_RT1uvsDR_883	0	7.81	8.18	8.12	6.87	0	0.373	1.295	0.417	0	0.309	1.239	0.463	1	-0.937	-1.915	0.003

Affymetrix Probeset ID	Gene Symbol	Gene Title	Figure 7 A Venn n=2289	Present in any 2 analyses n=423 1=yes 0=no	Mean log2 intensity DR+/+ ND	Mean log2 intensity DR+/+ HCD	Mean log2 intensity DR+/+ B/S	Mean log2 intensity F+/+ day 40 ND	DR+/+HCD vs DR+/+ND FDR<10% Log2 ratio >0.5 n=636	Log2 ratio DR+/+HCD vs DR+/+ ND	Fold-Change DR+/+HCD vs DR+/+ ND	FDR DR+/+HCD vs DR+/+ ND	DR+/+B/S vs DR+/+ND Log2 ratio >0.5 n=350	Log2 ratio DR+/+ B/S vs DR+/+ ND	Fold-Change DR+/+B/S vs DR+/+ ND	FDR DR+/+B/S vs DR+/+ ND	F+/+ vs DR+/+ND Log2 ratio >0.5 n=1209	Log2 ratio F+/+ vs DR+/+ ND	Fold-Change F+/+ vs DR+/+ ND	FDR F+/+ vs DR+/+ ND
1370895_at	Col5a2	collagen, type V, alpha 2	uniq_RT1uvsDR_883	0	8.60	8.26	8.28	8.06	0	-0.337	-1.263	0.401	0	-0.315	-1.244	0.410	1	-0.537	-1.450	0.074
1370903_a_at	Ndrg3	NDRG family member 3	uniq_RT1uvsDR_883	0	8.26	8.12	8.26	7.73	0	-0.141	-1.103	0.902	0	0.000	1.000	1.156	1	-0.529	-1.443	0.092
1370905_at	Dock9	dedicator of cytokinesis 9	uniq_RT1uvsDR_883	0	11.09	11.28	11.20	10.22	0	0.196	1.145	0.805	0	0.109	1.078	0.994	1	-0.867	-1.823	0.005
1370932_at	Lrp4	low density lipoprotein receptor-related protein 4	uniq_RT1uvsDR_883	0	4.85	4.45	4.58	4.31	0	-0.398	-1.318	0.250	0	-0.272	-1.208	0.519	1	-0.536	-1.450	0.076
1370935_at	Slc44a1	solute carrier family 44, member 1	uniq_RT1uvsDR_883	0	8.59	8.76	8.68	7.96	0	0.170	1.125	0.909	0	0.083	1.059	1.064	1	-0.634	-1.552	0.030
1370940_at	Tjp2	tight junction protein 2	uniq_RT1uvsDR_883	0	5.75	5.68	5.64	6.66	0	-0.075	-1.054	1.092	0	-0.118	-1.085	0.936	1	0.903	1.869	0.046
1370948_a_at	Marcks	myristoylated alanine rich protein kinase C substrate	uniq_RT1uvsDR_883	0	8.70	9.17	9.01	9.54	0	0.467	1.382	0.263	0	0.313	1.242	0.469	1	0.842	1.793	0.057
1370950_at	Ppap2b	phosphatidic acid phosphatase type 2B	uniq_RT1uvsDR_883	0	9.92	10.17	10.06	9.28	0	0.244	1.184	0.691	0	0.132	1.096	0.948	1	-0.649	-1.568	0.027
1370951_at	Ppap2b	phosphatidic acid phosphatase type 2B	uniq_RT1uvsDR_883	0	7.91	7.97	8.08	7.26	0	0.055	1.039	1.115	0	0.169	1.124	0.832	1	-0.649	-1.569	0.027
1370995_at	Pou2f1	POU class 2 homeobox 1	uniq_RT1uvsDR_883	0	5.60	5.71	5.83	7.04	0	0.112	1.081	0.925	0	0.232	1.174	0.571	1	1.440	2.713	0.007
1371036_at	Nrcam	neuronal cell adhesion molecule	uniq_RT1uvsDR_883	0	8.54	8.72	8.63	7.99	0	0.182	1.135	0.850	0	0.098	1.070	1.033	1	-0.543	-1.457	0.070
1371092_at	LOC286960	preprotrypsinogen IV	uniq_RT1uvsDR_883	0	6.24	6.18	6.36	8.59	0	-0.055	-1.039	1.007	0	0.121	1.087	0.870	1	2.351	5.103	0.001
1371099_at	Pigr	polymeric immunoglobulin receptor	uniq_RT1uvsDR_883	0	7.99	7.67	8.26	7.23	0	-0.317	-1.245	0.394	0	0.278	1.213	0.529	1	-0.760	-1.693	0.011
1371120_s_at	Bdkrb2	bradykinin receptor B2	uniq_RT1uvsDR_883	0	4.46	4.03	3.98	3.84	0	-0.431	-1.348	0.190	0	-0.483	-1.398	0.072	1	-0.616	-1.533	0.040
1371209_at	RT1-CE5	RT1 class I, locus CE5	uniq_RT1uvsDR_883	0	3.07	3.38	2.99	4.04	0	0.308	1.238	0.550	0	-0.076	-1.054	1.025	1	0.970	1.958	0.032
1371243_at	Grm8	glutamate receptor, metabotropic 8	uniq_RT1uvsDR_883	0	4.86	4.43	4.74	4.22	0	-0.428	-1.345	0.208	0	-0.119	-1.086	0.960	1	-0.641	-1.559	0.037
1371245_a_at	LOC1001348	beta globin minor gene /// beta-globin	uniq_RT1uvsDR_883	0	10.05	10.14	10.06	7.34	0	0.087	1.062	0.776	0	0.002	1.001	0.900	1	-2.709	-6.537	0.000
1371310_s_at	Serpinh1	serpin peptidase inhibitor, clade H (heat shock prote	uniq_RT1uvsDR_883	0	8.81	8.45	8.33	7.90	0	-0.357	-1.281	0.341	0	-0.473	-1.388	0.102	1	-0.906	-1.874	0.004
1371394_x_at	---	---	uniq_RT1uvsDR_883	0	7.27	7.21	7.18	8.19	0	-0.054	-1.038	1.005	0	-0.087	-1.062	0.965	1	0.921	1.893	0.040
1371412_a_at	Nrep	neuronal regeneration related protein	uniq_RT1uvsDR_883	0	9.46	9.49	9.49	8.95	0	0.030	1.021	1.168	0	0.032	1.022	1.130	1	-0.518	-1.432	0.090
1371442_at	Hyou1	hypoxia up-regulated 1	uniq_RT1uvsDR_883	0	8.36	8.02	8.20	7.04	0	-0.345	-1.270	0.305	0	-0.159	-1.116	0.729	1	-1.320	-2.497	0.000
1371472_at	---	---	uniq_RT1uvsDR_883	0	10.44	10.17	10.43	9.88	0	-0.265	-1.202	0.507	0	-0.010	-1.007	1.065	1	-0.557	-1.471	0.065
1371491_at	Notch1	notch 1	uniq_RT1uvsDR_883	0	7.55	7.55	7.52	7.03	0	0.005	1.004	1.174	0	-0.028	-1.020	1.124	1	-0.511	-1.425	0.091
1371519_at	Etfdh	electron-transferring-flavoprotein dehydrogenase	uniq_RT1uvsDR_883	0	7.80	7.93	7.96	7.28	0	0.128	1.093	0.981	0	0.158	1.115	0.896	1	-0.521	-1.435	0.084
1371527_at	Emp1	epithelial membrane protein 1	uniq_RT1uvsDR_883	0	8.85	8.49	8.45	8.25	0	-0.359	-1.283	0.333	0	-0.401	-1.320	0.213	1	-0.599	-1.514	0.044
1371541_at	Mylk	myosin light chain kinase	uniq_RT1uvsDR_883	0	8.79	8.50	8.31	8.15	0	-0.293	-1.225	0.482	0	-0.481	-1.396	0.094	1	-0.641	-1.559	0.029
1371633_at	Ctnnbl1	catenin, beta like 1	uniq_RT1uvsDR_883	0	9.89	10.06	10.12	9.34	0	0.177	1.131	0.858	0	0.229	1.172	0.695	1	-0.550	-1.464	0.063
1371665_at	Smarcd3	SWI/SNF related, matrix associated, actin dependen	uniq_RT1uvsDR_883	0	5.46	5.19	5.20	4.87	0	-0.272	-1.208	0.493	0	-0.262	-1.199	0.565	1	-0.589	-1.505	0.045
1371691_at	Rarres2	retinoic acid receptor responder (tazarotene induce	uniq_RT1uvsDR_883	0	7.60	7.11	7.20	7.06	0	-0.491	-1.406	0.098	0	-0.401	-1.320	0.220	1	-0.542	-1.456	0.071
1371732_at	Dpt	dermatopontin	uniq_RT1uvsDR_883	0	8.70	8.45	8.39	7.28	0	-0.249	-1.189	0.577	0	-0.310	-1.240	0.407	1	-1.416	-2.668	0.000
1371756_at	LOC683313	similar to keratin complex 2, basic, gene 6a	uniq_RT1uvsDR_883	0	2.70	2.74	2.55	3.57	0	0.037	1.026	1.097	0	-0.144	-1.105	0.856	1	0.869	1.827	0.047
1371757_s_at	LOC1003652	keratin 6A-like /// similar to keratin complex 2, basic	uniq_RT1uvsDR_883	0	4.60	4.54	4.58	6.00	0	-0.057	-1.041	1.113	0	-0.018	-1.012	1.064	1	1.405	2.648	0.008
1371762_at	Rbp4	retinol binding protein 4, plasma	uniq_RT1uvsDR_883	0	8.28	8.65	8.42	9.06	0	0.379	1.300	0.384	0	0.140	1.102	0.927	1	0.785	1.724	0.076
1371775_at	LOC1009124	short/branched chain specific acyl-CoA dehydrogena	uniq_RT1uvsDR_883	0	9.74	9.85	9.89	9.23	0	0.117	1.084	1.038	0	0.157	1.115	0.900	1	-0.502	-1.416	0.100
1371776_at	---	---	uniq_RT1uvsDR_883	0	5.20	5.18	5.21	6.28	0	-0.023	-1.016	1.152	0	0.008	1.005	1.131	1	1.076	2.108	0.022
1371960_at	Ythdf2	YTH domain family, member 2	uniq_RT1uvsDR_883	0	7.71	7.72	7.87	6.37	0	0.012	1.009	1.135	0	0.156	1.114	0.864	1	-1.343	-2.536	0.000
1371988_at	Man1a1	mannosidase, alpha, class 1A, member 1	uniq_RT1uvsDR_883	0	8.41	8.41	8.41	7.88	0	0.005	1.004	1.176	0	0.000	-1.000	1.156	1	-0.525	-1.439	0.076
1372011_at	---	---	uniq_RT1uvsDR_883	0	6.07	5.64	5.64	5.27	0	-0.433	-1.350	0.190	0	-0.427	-1.345	0.165	1	-0.798	-1.739	0.023
1372025_at	---	---	uniq_RT1uvsDR_883	0	4.64	5.10	4.81	7.01	0	0.453	1.369	0.263	0	0.166	1.122	0.763	1	2.369	5.166	0.001
1372060_at	---	---	uniq_RT1uvsDR_883	0	6.71	6.72	6.86	4.35	0	0.012	1.008	1.171	0	0.152	1.111	0.900	1	-2.358	-5.127	0.000
1372069_at	Kank1	KN motif and ankyrin repeat domains 1	uniq_RT1uvsDR_883	0	8.01	7.96	7.94	7.46	0	-0.050	-1.035	1.117	0	-0.063	-1.045	1.053	1	-0.547	-1.461	0.072
1372095_at	RGD1309821	similar to KIAA1161 protein	uniq_RT1uvsDR_883	0	5.58	5.46	5.70	4.72	0	-0.116	-1.084	1.010	0	0.122	1.088	0.939	1	-0.862	-1.818	0.006
1372097_at	---	---	uniq_RT1uvsDR_883	0	4.94	4.70	4.88	4.03	0	-0.237	-1.178	0.610	0	-0.054	-1.038	1.044	1	-0.911	-1.880	0.005
1372102_at	Ncor1	nuclear receptor co-repressor 1	uniq_RT1uvsDR_883	0	7.67	8.01	7.96	8.82	0	0.345	1.270	0.466	0	0.293	1.225	0.448	1	1.150	2.219	0.017
1372212_at	Paox	polyamine oxidase (exo-N4-amino)	uniq_RT1uvsDR_883	0	7.10	7.24	7.32	8.43	0	0.138	1.100	0.976	0	0.218	1.163	0.723	1	1.330	2.514	0.010
1372236_at	Nod1	nucleotide-binding oligomerization domain containi	uniq_RT1uvsDR_883	0	8.00	7.94	8.16	7.26	0	-0.062	-1.044	1.043	0	0.163	1.120	0.875	1	-0.736	-1.666	0.013
1372294_at	Pxdn	peroxidasin homolog (Drosophila)	uniq_RT1uvsDR_883	0	7.42	7.26	7.11	6.59	0	-0.158	-1.115	0.896	0	-0.312	-1.241	0.420	1	-0.831	-1.779	0.007
1372321_at	Lyl1	lymphoblastic leukemia derived sequence 1	uniq_RT1uvsDR_883	0	5.28	4.86	4.94	4.63	0	-0.420	-1.337	0.209	0	-0.339	-1.265	0.368	1	-0.652	-1.571	0.028
1372322_at	RGD1565023	similar to Suppressor of S. cerevisiae gcr2	uniq_RT1uvsDR_883	0	7.70	7.78	7.78	7.18	0	0.082	1.058	1.088	0	0.084	1.060	1.068	1	-0.518	-1.432	0.087
1372379_at	---	---	uniq_RT1uvsDR_883	0	8.03	8.17	8.17	7.40	0	0.144	1.105	0.967	0	0.145	1.106	0.925	1	-0.622	-1.540	0.035
1372404_at	Rac2	ras-related C3 botulinum toxin substrate 2 (rho fam	uniq_RT1uvsDR_883	0	6.65	6.53	6.31	6.10	0	-0.125	-1.090	0.756	0	-0.339	-1.265	0.335	1	-0.550	-1.464	0.095
1372405_at	Ppp1r37	protein phosphatase 1, regulatory subunit 37	uniq_RT1uvsDR_883	0	6.85	6.65	7.03	6.23	0	-0.198	-1.147	0.706	0	0.179	1.132	0.775	1	-0.616	-1.533	0.038
1372423_at	Perp	PERP, TP53 apoptosis effector	uniq_RT1uvsDR_883	0	8.45	8.63	8.79	7.73	0	0.181	1.134	0.861	0	0.339	1.265	0.415	1	-0.722	-1.649	0.015
1372440_at	Serpine2	serpin peptidase inhibitor, clade E (nexin, plasminog	uniq_RT1uvsDR_883	0	8.17	7.70	7.85	7.39	0	-0.465	-1.380	0.132	0	-0.319	-1.248	0.378	1	-0.783	-1.720	0.009
1372462_at	Acat2	acetyl-CoA acetyltransferase 2	uniq_RT1uvsDR_883	0	8.80	8.63	9.03	7.73	0	-0.170	-1.125	0.793	0	0.229	1.172	0.688	1	-1.072	-2.102	0.001
1372480_at	Zfp462	zinc finger protein 462	uniq_RT1uvsDR_883	0	8.13	8.03	8.26	7.55	0	-0.100	-1.072	0.988	0	0.134	1.097	0.946	1	-0.582	-1.497	0.048
1372481_at	---	---	uniq_RT1uvsDR_883	0	7.41	7.01	7.24	6.86	0	-0.397	-1.317	0.266	0	-0.165	-1.121	0.754	1	-0.546	-1.460	0.074
1372524_at	---	---	uniq_RT1uvsDR_883	0	7.33	7.67	7.51	8.10	0	0.348	1.273	0.459	0	0.188	1.139	0.812	1	0.776	1.713	0.076
1372606_at	---	---	uniq_RT1uvsDR_883	0	7.79	7.83	7.78	7.20	0	0.034	1.024	1.150	0	-0.012	-1.008	1.151	1	-0.595	-1.510	0.044
1372639_at	Trim54	tripartite motif-containing 54	uniq_RT1uvsDR_883	0	4.47	4.32	4.28	3.84	0	-0.154	-1.113	0.786	0	-0.196	-1.146	0.711	1	-0.628	-1.545	0.060
1372646_at	RGD1305645	similar to RIKEN cDNA 1500015O10	uniq_RT1uvsDR_883	0	5.63	5.27	5.69	4.66	0	-0.355	-1.279	0.284	0	0.061	1.043	1.063	1	-0.972	-1.961	0.003
1372681_at	---	---	uniq_RT1uvsDR_883	0	4.96	4.89	4.88	6.32	0	-0.069	-1.049	1.096	0	-0.075	-1.054	1.040	1	1.367	2.580	0.009
1372754_at	Appl2	adaptor protein, phosphotyrosine interaction, PH do	uniq_RT1uvsDR_883	0	10.07	10.36	10.29	8.70	0	0.283	1.217	0.564	0	0.221	1.165	0.721	1	-1.371	-2.587	0.000
1372818_at	Colec12	collectin sub-family member 12	uniq_RT1uvsDR_883	0	6.58	6.61	6.59	5.82	0	0.033	1.023	1.088	0	0.015	1.010	1.094	1	-0.758	-1.692	0.018
1372923_at	---	---	uniq_RT1uvsDR_883	0	4.90	5.01	5.05	7.13	0	0.109	1.078	1.040	0	0.152	1.111	0.885	1	2.226	4.677	0.001
1372927_at	Mrpl50	mitochondrial ribosomal protein L50	uniq_RT1uvsDR_883	0	6.22	6.13	6.25	6.96	0	-0.093	-1.066	1.008	0	0.027	1.019	1.128	1	0.743	1.674	0.090
1372945_at	Ing3	inhibitor of growth family, member 3	uniq_RT1uvsDR_883	0	7.71	7.74	8.13	7.16	0	0.029	1.020	1.143	0	0.418	1.336	0.270	1	-0.548	-1.462	0.075
1372993_at	---	---	uniq_RT1uvsDR_883	0	6.65	6.46	6.57	6.08	0	-0.196	-1.146	0.732	0	-0.081	-1.058	0.992	1	-0.575	-1.489	0.063
1373035_at	---	---	uniq_RT1uvsDR_883	0	7.28	7.53	7.20	8.13	0	0.242	1.183	0.656	0	-0.087	-1.062	0.909	1	0.847	1.799	0.059
1373093_at	Errfi1	ERBB receptor feedback inhibitor 1	uniq_RT1uvsDR_883	0	9.97	10.23	10.22	11.00	0	0.260	1.197	0.637	0	0.244	1.185	0.593	1	1.024	2.034	0.026
1373123_at	Rmi1	RMI1, RecQ mediated genome instability 1, homolog	uniq_RT1uvsDR_883	0	8.85	8.77	8.89	8.25	0	-0.082	-1.058	1.093	0	0.039	1.027	1.122	1	-0.605	-1.521	0.039
1373151_at	Lhfp	lipoma HMGIC fusion partner	uniq_RT1uvsDR_883	0	7.62	7.23	7.29	6.90	0	-0.392	-1.313	0.232	0	-0.328	-1.255	0.344	1	-0.714	-1.641	0.016
1373177_x_at	LOC679608	hypothetical protein LOC679608	uniq_RT1uvsDR_883	0	5.86	5.92	6.30	6.72	0	0.054	1.038	1.120	0	0.431	1.348	0.256	1	0.860	1.815	0.060
1373261_at	---	---	uniq_RT1uvsDR_883	0	6.16	6.10	6.18	5.62	0	-0.060	-1.042	1.108	0	0.021	1.014	1.071	1	-0.546	-1.460	0.067
1373288_at	St5	suppression of tumorigenicity 5	uniq_RT1uvsDR_883	0	7.58	7.65	7.78	6.69	0	0.069	1.049	1.105	0	0.200	1.149	0.764	1	-0.893	-1.858	0.004
1373345_at	Amigo2	adhesion molecule with Ig like domain 2	uniq_RT1uvsDR_883	0	9.45	10.04	9.70	8.68	0	0.591	1.507	0.117	0	0.254	1.192	0.620	1	-0.765	-1.700	0.010
1373374_at	Lmo4	LIM domain only 4	uniq_RT1uvsDR_883	0	9.16	8.90	8.98	8.48	0	-0.259	-1.197	0.585	0	-0.185	-1.137	0.781	1	-0.683	-1.605	0.020
1373401_at	Tnc	tenascin C	uniq_RT1uvsDR_883	0	5.12	4.69	4.69	3.78	0	-0.422	-1.340	0.187	0	-0.429	-1.346	0.166	1	-1.336	-2.525	0.000
1373463_at	Col5a2	collagen, type V, alpha 2	uniq_RT1uvsDR_883	0	6.64	6.38	6.52	6.11	0	-0.263	-1.200	0.558	0	-0.120	-1.086	0.847	1	-0.531	-1.445	0.081
1373487_at	---	---	uniq_RT1uvsDR_883	0	5.01	5.00	4.95	4.34	0	-0.016	-1.011	1.157	0	-0.065	-1.046	1.024	1	-0.671	-1.593	0.025
1373546_at	Atp11a	ATPase, class VI, type 11A	uniq_RT1uvsDR_883	0	8.00	8.10	8.14	9.15	0	0.108	1.078	1.042	0	0.140	1.102	0.914	1	1.158	2.231	0.015
1373615_at	Frzb	frizzled-related protein	uniq_RT1uvsDR_883	0	8.48	8.78	8.75	6.93	0	0.297	1.228	0.551	0	0.274	1.209	0.572	1	-1.546	-2.919	0.000
1373636_at	Spock1	sparc/osteonectin, cwcv and kazal-like domains prot	uniq_RT1uvsDR_883	0	5.64	5.64	5.76	6.93	0	0.000	-1.000	1.171	0	0.128	1.093	0.915	1	1.292	2.449	0.012
1373646_at	Rab15	RAB15, member RAS oncogene family	uniq_RT1uvsDR_883	0	5.98	5.68	5.90	7.03	0	-0.306	-1.237	0.517	0	-0.086	-1.061	0.995	1	1.051	2.072	0.024
1373661_a_at	Cxcr4	chemokine (C-X-C motif) receptor 4	uniq_RT1uvsDR_883	0	7.14	6.98	7.07	6.29	0	-0.162	-1.118	0.864	0	-0.067	-1.048	1.035	1	-0.850	-1.803	0.006
1373674_at	Mfap5	microfibrillar associated protein 5	uniq_RT1uvsDR_883	0	5.82	5.40	5.43	5.01	0	-0.427	-1.345	0.164	0	-0.396	-1.316	0.214	1	-0.811	-1.755	0.007
1373745_at	Gtf2h4	general transcription factor II H, polypeptide 4	uniq_RT1uvsDR_883	0	6.88	7.13	7.09	6.16	0	0.244	1.184	0.703	0	0.210	1.157	0.745	1	-0.722	-1.649	0.019
1373834_at	Mef2bnb	MEF2B neighbor	uniq_RT1uvsDR_883	0	6.72	6.86	6.99	6.09	0	0.141	1.103	0.957	0	0.269	1.205	0.552	1	-0.632	-1.549	0.030
1373900_at	Krt7	keratin 7	uniq_RT1uvsDR_883	0	10.11	10.46	10.43	9.44	0	0.350	1.275	0.422	0	0.318	1.247	0.449	1	-0.664	-1.584	0.023
1373915_at	Dmpk	dystrophia myotonica-protein kinase	uniq_RT1uvsDR_883	0	7.80	7.40	7.71	7.19	0	-0.405	-1.324	0.224	0	-0.088	-1.063	1.027	1	-0.612	-1.528	0.038
1373947_at	Dpt	dermatopontin	uniq_RT1uvsDR_883	0	7.03	6.95	7.00	6.15	0	-0.080	-1.057	1.097	0	-0.025	-1.017	1.089	1	-0.875	-1.834	0.007
1373975_at	Inmt	indolethylamine N-methyltransferase	uniq_RT1uvsDR_883	0	6.02	6.56	6.18	7.47	0	0.536	1.450	0.170	0	0.154	1.112	0.827	1	1.445	2.723	0.007
1374035_at	Rem2	RAS (RAD and GEM) like GTP binding 2	uniq_RT1uvsDR_883	0	8.47	8.73	8.29	9.22	0	0.259	1.197	0.638	0	-0.177	-1.131	0.719	1	0.746	1.677	0.097
1374065_at	---	---	uniq_RT1uvsDR_883	0	7.74	7.50	7.84	7.14	0	-0.244	-1.184	0.659	0	0.097	1.070	1.020	1	-0.600	-1.516	0.042
1374105_at	Higd1a	HIG1 hypoxia inducible domain family, member 1A	uniq_RT1uvsDR_883	0	7.20	7.21	7.23	6.66	0	0.004	1.003	1.174	0	0.034	1.024	1.124	1	-0.538	-1.452	0.074
1374126_at	---	---	uniq_RT1uvsDR_883	0	7.72	7.95	7.89	7.19	0	0.230	1.173	0.708	0	0.164	1.121	0.848	1	-0.527	-1.441	0.083
1374171_at	Abcc9	ATP-binding cassette, subfamily C (CFTR/MRP), mem	uniq_RT1uvsDR_883	0	8.12	7.70	7.71	7.29	0	-0.421	-1.339	0.187	0	-0.412	-1.330	0.170	1	-0.835	-1.784	0.007
1374247_at	Stab1	stabilin 1	uniq_RT1uvsDR_883	0	6.33	5.86	6.01	5.72	0	-0.466	-1.381	0.120	0	-0.314	-1.243	0.425	1	-0.613	-1.529	0.035
1374251_at	---	---	uniq_RT1uvsDR_883	0	6.77	6.35	6.91	5.79	0	-0.422	-1.340	0.219	0	0.143	1.105	0.921	1	-0.976	-1.967	0.002
1374273_at	---	---	uniq_RT1uvsDR_883	0	9.84	9.73	9.90	9.28	0	-0.109	-1.079	1.008	0	0.063	1.044	1.101	1	-0.565	-1.480	0.056
1374283_at	Falz	fetal Alzheimer antigen	uniq_RT1uvsDR_883	0	5.87	6.26	5.96	6.72	0	0.384	1.305	0.325	0	0.086	1.061	0.679	1	0.845	1.796	0.077
1374325_at	---	---	uniq_RT1uvsDR_883	0	6.30	6.12	6.11	5.78	0	-0.172	-1.127	0.653	0	-0.190	-1.141	0.782	1	-0.521	-1.435	0.090
1374344_at	Lppr3	lipid phosphate phosphatase-related protein type 3	uniq_RT1uvsDR_883	0	6.64	6.55	6.63	5.95	0	-0.093	-1.067	1.061	0	-0.014	-1.010	1.135	1	-0.692	-1.616	0.019
1374396_at	Atp6v1c1	ATPase, H+ transporting, lysosomal V1 subunit C1	uniq_RT1uvsDR_883	0	9.54	9.85	9.70	10.36	0	0.311	1.241	0.525	0	0.166	1.122	0.857	1	0.826	1.772	0.059
1374496_at	Tril	TLR4 interactor with leucine-rich repeats	uniq_RT1uvsDR_883	0	7.80	7.53	7.74	7.29	0	-0.273	-1.208	0.536	0	-0.067	-1.048	1.020	1	-0.515	-1.429	0.098
1374554_at	Pithd1	PITH (C-terminal proteasome-interacting domain of	uniq_RT1uvsDR_883	0	8.93	9.31	9.25	8.29	0	0.382	1.303	0.373	0	0.320	1.248	0.456	1	-0.644	-1.563	0.029
1374560_at	Prrc1	proline-rich coiled-coil 1	uniq_RT1uvsDR_883	0	6.33	6.36	6.37	7.05	0	0.027	1.019	1.155	0	0.043	1.031	1.102	1	0.721	1.648	0.096

Affymetrix Probeset ID	Gene Symbol	Gene Title	Figure 7 A Venn n=2289	Present in any 2 analyses n=423 1=yes 0=no	Mean log2 intensity DR+/+ ND	Mean log2 intensity DR+/+ HCD	Mean log2 intensity DR+/+ B/S	Mean log2 intensity F+/+ day 40 ND	DR+/+HCD vs DR+/+ND FDR<10% Log2 ratio >0.5 n=636	Log2 ratio DR+/+HCD vs DR+/+ ND	Fold-Change DR+/+H CD vs DR+/+ ND	FDR DR+/+H CD vs DR+/+ ND	DR+/+B/S vs DR+/+ND Log2 ratio >0.5 n=350	Log2 ratio DR+/+ B/S vs DR+/+ ND	Fold-Change DR+/+B /S vs DR+/+ ND	FDR DR+/+B /S vs DR+/+ ND	F+/+ vs DR+/+N D Log2 ratio >0.5 n=1209	Log2 ratio F+/+ vs DR+/+ ND	Fold-Change F+/+ vs DR+/+ ND	FDR F+/+ vs DR+/+ ND
1374574_at	Fam214a	family with sequence similarity 214, member A	uniq_RT1uvsDR_883	0	6.68	6.80	6.98	8.09	0	0.119	1.086	1.002	0	0.308	1.238	0.449	1	1.412	2.662	0.008
1374591_at	Ptprd	protein tyrosine phosphatase, receptor type, D	uniq_RT1uvsDR_883	0	6.84	7.05	6.92	5.90	0	0.217	1.162	0.704	0	0.085	1.061	0.975	1	-0.934	-1.910	0.003
1374616_at	Pdgfrl	platelet-derived growth factor receptor-like	uniq_RT1uvsDR_883	0	9.73	10.05	9.98	7.44	0	0.316	1.245	0.495	0	0.253	1.192	0.630	1	-2.291	-4.895	0.000
1374695_at	---	---	uniq_RT1uvsDR_883	0	8.53	8.62	8.63	7.80	0	0.094	1.068	1.064	0	0.099	1.071	1.024	1	-0.731	-1.660	0.014
1374699_at	Fam84a	family with sequence similarity 84, member A	uniq_RT1uvsDR_883	0	4.56	4.66	4.57	6.08	0	0.100	1.072	1.062	0	0.011	1.007	1.135	1	1.516	2.859	0.006
1374704_at	Kdelc2	KDEL (Lys-Asp-Glu-Leu) containing 2	uniq_RT1uvsDR_883	0	7.12	7.29	7.35	6.57	0	0.170	1.125	0.905	0	0.237	1.179	0.650	1	-0.542	-1.456	0.085
1374786_at	---	---	uniq_RT1uvsDR_883	0	6.53	6.38	6.88	5.92	0	-0.150	-1.109	0.886	0	0.348	1.273	0.393	1	-0.618	-1.534	0.040
1374806_at	Fxr1	fragile X mental retardation gene 1, autosomal hom	uniq_RT1uvsDR_883	0	5.77	5.37	5.41	5.23	0	-0.406	-1.325	0.232	0	-0.360	-1.283	0.304	1	-0.548	-1.462	0.070
1374855_at	Per1	period circadian clock 1	uniq_RT1uvsDR_883	0	8.09	8.17	7.95	8.91	0	0.076	1.054	1.112	0	-0.148	-1.108	0.866	1	0.816	1.761	0.061
1374859_at	---	---	uniq_RT1uvsDR_883	0	6.12	6.09	6.50	5.56	0	-0.029	-1.020	1.099	0	0.387	1.308	0.323	1	-0.554	-1.468	0.064
1374888_at	Cwc25	CWC25 spliceosome-associated protein homolog (S.	uniq_RT1uvsDR_883	0	6.80	6.95	6.98	5.55	0	0.140	1.102	0.949	0	0.178	1.132	0.813	1	-1.260	-2.394	0.000
1374897_at	Thoc4	THO complex 4	uniq_RT1uvsDR_883	0	8.40	8.55	8.49	7.25	0	0.149	1.109	0.962	0	0.089	1.064	1.053	1	-1.147	-2.214	0.001
1374916_at	---	---	uniq_RT1uvsDR_883	0	5.54	5.76	5.58	6.37	0	0.212	1.158	0.780	0	0.040	1.028	1.103	1	0.824	1.770	0.061
1374924_at	LOC1009102:	uncharacterized LOC100910237	uniq_RT1uvsDR_883	0	6.29	6.69	6.67	3.77	0	0.400	1.319	0.378	0	0.385	1.306	0.331	1	-2.520	-5.735	0.000
1374932_at	LOC1003614	rCG31799-like	uniq_RT1uvsDR_883	0	6.72	7.27	7.14	5.66	0	0.552	1.467	0.142	0	0.425	1.343	0.251	1	-1.057	-2.080	0.001
1374947_at	Bcar3	breast cancer anti-estrogen resistance 3	uniq_RT1uvsDR_883	0	5.68	6.08	5.99	4.08	0	0.402	1.321	0.341	0	0.310	1.240	0.466	1	-1.597	-3.025	0.000
1374959_at	Nqo2	NAD(P)H dehydrogenase, quinone 2	uniq_RT1uvsDR_883	0	5.93	5.92	6.24	7.77	0	-0.009	-1.007	1.057	0	0.313	1.243	0.448	1	1.845	3.591	0.005
1374963_s_at	---	---	uniq_RT1uvsDR_883	0	5.04	5.61	5.56	6.22	0	0.578	1.493	0.144	0	0.520	1.434	0.137	1	1.183	2.271	0.016
1375102_at	Large	like-glycosyltransferase	uniq_RT1uvsDR_883	0	5.11	5.33	5.28	4.49	0	0.218	1.163	0.723	0	0.169	1.124	0.832	1	-0.621	-1.538	0.048
1375116_at	---	---	uniq_RT1uvsDR_883	0	6.99	6.61	6.75	6.39	0	-0.379	-1.300	0.304	0	-0.238	-1.180	0.630	1	-0.607	-1.523	0.040
1375199_at	---	---	uniq_RT1uvsDR_883	0	7.61	7.67	7.72	8.72	0	0.058	1.041	1.065	0	0.116	1.084	0.899	1	1.116	2.167	0.019
1375214_at	Galnt2	UDP-N-acetyl-alpha-D-galactosamine:polypeptide N	uniq_RT1uvsDR_883	0	8.77	8.79	8.86	7.86	0	0.021	1.014	1.150	0	0.082	1.059	0.948	1	-0.913	-1.882	0.003
1375377_at	Igsf3	immunoglobulin superfamily, member 3	uniq_RT1uvsDR_883	0	5.86	6.22	6.11	6.60	0	0.354	1.278	0.391	0	0.247	1.187	0.530	1	0.738	1.668	0.096
1375422_at	---	---	uniq_RT1uvsDR_883	0	9.38	9.56	9.64	8.63	0	0.186	1.137	0.677	0	0.267	1.204	0.516	1	-0.742	-1.672	0.012
1375519_at	LOC287167	globin, alpha	uniq_RT1uvsDR_883	0	8.04	8.20	8.01	7.46	0	0.157	1.115	0.640	0	-0.025	-1.017	0.896	1	-0.585	-1.500	0.064
1375523_at	Marcks	myristoylated alanine rich protein kinase C substrat	uniq_RT1uvsDR_883	0	9.22	9.38	9.40	9.99	0	0.165	1.121	0.925	0	0.185	1.137	0.820	1	0.772	1.708	0.076
1375532_at	Id2	inhibitor of DNA binding 2	uniq_RT1uvsDR_883	0	6.95	7.14	6.99	5.97	0	0.196	1.146	0.805	0	0.047	1.033	1.103	1	-0.976	-1.967	0.002
1375573_at	---	---	uniq_RT1uvsDR_883	0	6.13	6.05	6.15	7.18	0	-0.081	-1.058	1.056	0	0.021	1.015	1.009	1	1.048	2.067	0.024
1375613_at	Gata6	GATA binding protein 6	uniq_RT1uvsDR_883	0	6.67	6.25	6.37	6.07	0	-0.421	-1.339	0.211	0	-0.297	-1.229	0.465	1	-0.606	-1.522	0.038
1375638_at	---	---	uniq_RT1uvsDR_883	0	8.55	8.38	8.57	8.02	0	-0.176	-1.130	0.796	0	0.019	1.013	1.143	1	-0.534	-1.448	0.075
1375640_at	Fkbp9	FK506 binding protein 9	uniq_RT1uvsDR_883	0	8.36	8.08	8.17	7.63	0	-0.283	-1.217	0.568	0	-0.185	-1.137	0.784	1	-0.727	-1.656	0.013
1375676_at	Lin7c	lin-7 homolog C (C. elegans)	uniq_RT1uvsDR_883	0	7.93	8.19	8.37	6.72	0	0.258	1.196	0.564	0	0.445	1.361	0.229	1	-1.207	-2.309	0.001
1375699_at	---	---	uniq_RT1uvsDR_883	0	8.10	8.23	8.04	7.33	0	0.130	1.094	1.003	0	-0.067	-1.047	1.049	1	-0.771	-1.707	0.009
1375707_at	---	---	uniq_RT1uvsDR_883	0	7.27	7.55	7.39	6.70	0	0.281	1.215	0.506	0	0.120	1.087	0.921	1	-0.569	-1.484	0.052
1375730_at	Itfg2	integrin alpha FG-GAP repeat containing 2	uniq_RT1uvsDR_883	0	5.97	5.62	5.71	5.46	0	-0.345	-1.270	0.363	0	-0.256	-1.194	0.550	1	-0.505	-1.420	0.093
1375732_at	---	---	uniq_RT1uvsDR_883	0	7.70	7.75	7.75	6.71	0	0.055	1.039	1.137	0	0.052	1.037	1.085	1	-0.984	-1.977	0.002
1375794_at	---	---	uniq_RT1uvsDR_883	0	4.32	4.32	4.29	5.12	0	0.001	1.001	1.169	0	-0.027	-1.019	1.122	1	0.804	1.746	0.075
1375815_at	---	---	uniq_RT1uvsDR_883	0	5.46	5.14	5.60	8.12	0	-0.325	-1.252	0.348	0	0.136	1.099	0.815	1	2.658	6.311	0.000
1375857_at	Myof	myoferlin	uniq_RT1uvsDR_883	0	6.08	5.62	5.66	5.00	0	-0.460	-1.375	0.144	0	-0.425	-1.342	0.201	1	-1.085	-2.121	0.001
1375920_at	Plekho1	pleckstrin homology domain containing, family O m	uniq_RT1uvsDR_883	0	5.45	5.18	5.19	4.71	0	-0.268	-1.204	0.565	0	-0.257	-1.195	0.505	1	-0.738	-1.668	0.012
1375945_at	Plxdc2	plexin domain containing 2	uniq_RT1uvsDR_883	0	6.11	5.98	6.05	5.51	0	-0.132	-1.095	0.896	0	-0.064	-1.045	0.962	1	-0.603	-1.518	0.047
1375961_at	Frzb	frizzled-related protein	uniq_RT1uvsDR_883	0	9.16	9.23	9.39	7.50	0	0.071	1.051	1.110	0	0.228	1.171	0.704	1	-1.660	-3.161	0.000
1375993_at	---	---	uniq_RT1uvsDR_883	0	3.75	3.59	3.67	4.65	0	-0.155	-1.113	0.844	0	-0.083	-1.059	0.973	1	0.904	1.871	0.042
1376026_at	Donson	downstream neighbor of SON	uniq_RT1uvsDR_883	0	6.84	7.15	7.12	7.79	0	0.307	1.237	0.524	0	0.284	1.217	0.529	1	0.949	1.930	0.035
1376066_at	Rnd3	Rho family GTPase 3	uniq_RT1uvsDR_883	0	7.37	7.41	7.32	6.52	0	0.041	1.029	1.152	0	-0.053	-1.038	1.067	1	-0.850	-1.803	0.006
1376170_at	Col14a1	collagen, type XIV, alpha 1	uniq_RT1uvsDR_883	0	6.97	6.48	7.12	8.48	0	-0.492	-1.406	0.105	0	0.146	1.107	0.909	1	1.503	2.834	0.006
1376200_at	Mettl20	methyltransferase like 20	uniq_RT1uvsDR_883	0	6.65	6.79	6.66	5.22	0	0.143	1.104	0.975	0	0.007	1.005	1.147	1	-1.434	-2.702	0.000
1376226_at	---	---	uniq_RT1uvsDR_883	0	7.53	7.59	7.85	5.42	0	0.064	1.046	1.109	0	0.322	1.250	0.427	1	-2.102	-4.292	0.000
1376232_at	---	---	uniq_RT1uvsDR_883	0	6.30	5.97	6.49	5.49	0	-0.327	-1.254	0.453	0	0.190	1.141	0.750	1	-0.806	-1.749	0.007
1376282_at	---	---	uniq_RT1uvsDR_883	0	6.95	6.70	6.81	6.41	0	-0.249	-1.188	0.560	0	-0.135	-1.098	0.825	1	-0.539	-1.453	0.083
1376302_at	---	---	uniq_RT1uvsDR_883	0	8.30	8.63	8.41	9.23	0	0.332	1.259	0.480	0	0.107	1.077	0.976	1	0.926	1.900	0.039
1376358_at	---	---	uniq_RT1uvsDR_883	0	5.33	5.39	5.43	4.73	0	0.053	1.038	1.134	0	0.096	1.069	1.016	1	-0.606	-1.522	0.040
1376457_at	Crispld2	cysteine-rich secretory protein LCCL domain contain	uniq_RT1uvsDR_883	0	6.10	5.86	5.70	5.16	0	-0.242	-1.182	0.637	0	-0.403	-1.322	0.220	1	-0.935	-1.911	0.003
1376496_at	Apol9a	apolipoprotein L 9a	uniq_RT1uvsDR_883	0	5.11	4.92	4.85	4.50	0	-0.190	-1.141	0.836	0	-0.263	-1.200	0.583	1	-0.608	-1.524	0.038
1376534_at	---	---	uniq_RT1uvsDR_883	0	4.67	4.97	4.70	6.15	0	0.297	1.229	0.477	0	0.032	1.023	1.060	1	1.483	2.795	0.007
1376577_at	---	---	uniq_RT1uvsDR_883	0	9.35	9.03	9.35	10.23	0	-0.322	-1.250	0.420	0	-0.001	-1.001	1.145	1	0.873	1.832	0.047
1376584_at	Ppm1l	protein phosphatase, Mg2+/Mn2+ dependent, 1L	uniq_RT1uvsDR_883	0	7.90	8.12	8.16	8.82	0	0.211	1.157	0.779	0	0.255	1.193	0.623	1	0.913	1.882	0.040
1376594_at	Vezf1	vascular endothelial zinc finger 1	uniq_RT1uvsDR_883	0	8.42	8.63	8.55	7.90	0	0.204	1.152	0.804	0	0.129	1.093	0.894	1	-0.523	-1.437	0.090
1376619_at	Ptprd	protein tyrosine phosphatase, receptor type, D	uniq_RT1uvsDR_883	0	7.09	7.11	7.10	6.14	0	0.021	1.014	1.147	0	0.011	1.008	1.128	1	-0.943	-1.923	0.003
1376630_at	Tarsl2	threonyl-tRNA synthetase-like 2	uniq_RT1uvsDR_883	0	8.56	8.94	8.83	7.88	0	0.384	1.305	0.387	0	0.274	1.209	0.577	1	-0.679	-1.601	0.022
1376654_at	Plcxd3	phosphatidylinositol-specific phospholipase C, X dor	uniq_RT1uvsDR_883	0	7.06	7.17	7.13	7.80	0	0.109	1.078	1.051	0	0.077	1.055	1.069	1	0.741	1.672	0.091
1376710_at	---	---	uniq_RT1uvsDR_883	0	4.33	4.54	4.52	5.97	0	0.210	1.157	0.724	0	0.189	1.140	0.750	1	1.639	3.114	0.005
1376724_at	---	---	uniq_RT1uvsDR_883	0	6.01	6.48	6.31	6.83	0	0.467	1.382	0.281	0	0.292	1.225	0.497	1	0.818	1.763	0.069
1376911_at	---	---	uniq_RT1uvsDR_883	0	7.67	7.51	7.45	7.05	0	-0.155	-1.113	0.791	0	-0.213	-1.159	0.676	1	-0.619	-1.536	0.043
1376912_at	Zswim7	zinc finger, SWIM-type containing 7	uniq_RT1uvsDR_883	0	5.86	5.82	5.92	5.19	0	-0.048	-1.034	1.073	0	0.057	1.040	1.068	1	-0.678	-1.600	0.028
1376920_at	LOC500013	similar to sterile alpha motif domain containing 9-lik	uniq_RT1uvsDR_883	0	6.24	6.31	6.25	5.30	0	0.068	1.048	1.095	0	0.017	1.012	1.130	1	-0.936	-1.913	0.003
1376946_at	---	---	uniq_RT1uvsDR_883	0	9.34	9.25	9.36	8.76	0	-0.084	-1.060	1.054	0	0.024	1.017	1.136	1	-0.574	-1.489	0.053
1376958_at	LOC1009111(	leukocyte elastase inhibitor A-like	uniq_RT1uvsDR_883	0	4.56	4.14	4.22	6.10	0	-0.417	-1.335	0.206	0	-0.341	-1.267	0.332	1	1.537	2.903	0.006
1376977_at	Ptger3	prostaglandin E receptor 3 (subtype EP3)	uniq_RT1uvsDR_883	0	4.32	4.68	4.08	5.21	0	0.362	1.285	0.312	0	-0.233	-1.175	0.630	1	0.889	1.852	0.042
1377001_at	Nrcam	neuronal cell adhesion molecule	uniq_RT1uvsDR_883	0	6.87	6.97	7.01	6.18	0	0.097	1.069	1.041	0	0.135	1.098	0.906	1	-0.690	-1.613	0.019
1377111_at	---	---	uniq_RT1uvsDR_883	0	3.63	3.73	3.81	5.37	0	0.101	1.072	0.961	0	0.177	1.130	0.688	1	1.736	3.331	0.006
1377133_at	---	---	uniq_RT1uvsDR_883	0	3.76	3.97	3.91	6.68	0	0.211	1.157	0.793	0	0.144	1.105	0.907	1	2.917	7.553	0.000
1377217_at	---	---	uniq_RT1uvsDR_883	0	3.98	4.12	4.18	5.00	0	0.147	1.107	0.972	0	0.204	1.152	0.760	1	1.028	2.039	0.024
1377306_at	---	---	uniq_RT1uvsDR_883	0	4.20	4.05	4.30	5.06	0	-0.141	-1.103	0.906	0	0.100	1.072	0.968	1	0.863	1.819	0.052
1377308_a_at	---	---	uniq_RT1uvsDR_883	0	5.39	5.11	5.12	4.75	0	-0.277	-1.212	0.472	0	-0.265	-1.202	0.459	1	-0.635	-1.553	0.035
1377366_at	---	---	uniq_RT1uvsDR_883	0	9.96	10.24	9.96	9.18	0	0.282	1.216	0.563	0	-0.003	-1.002	1.155	1	-0.780	-1.718	0.009
1377406_at	Rpl32	ribosomal protein L32	uniq_RT1uvsDR_883	0	7.15	6.98	7.12	6.39	0	-0.168	-1.123	0.812	0	-0.024	-1.017	1.117	1	-0.756	-1.689	0.010
1377430_at	---	---	uniq_RT1uvsDR_883	0	5.03	5.30	5.38	5.78	0	0.266	1.203	0.610	0	0.352	1.276	0.381	1	0.751	1.683	0.096
1377501_at	Zfp317	zinc finger protein 317	uniq_RT1uvsDR_883	0	4.90	5.10	4.94	5.93	0	0.200	1.148	0.710	0	0.038	1.026	1.066	1	1.027	2.038	0.025
1377534_at	Stk32c	serine/threonine kinase 32C	uniq_RT1uvsDR_883	0	6.75	6.63	7.06	7.50	0	-0.117	-1.084	1.012	0	0.314	1.243	0.422	1	0.754	1.686	0.091
1377541_at	---	---	uniq_RT1uvsDR_883	0	5.63	5.54	5.66	5.05	0	-0.082	-1.059	1.071	0	0.033	1.023	1.113	1	-0.579	-1.493	0.049
1377551_at	---	---	uniq_RT1uvsDR_883	0	8.07	7.96	8.18	7.39	0	-0.113	-1.081	0.992	0	0.105	1.076	0.982	1	-0.677	-1.598	0.021
1377599_at	---	---	uniq_RT1uvsDR_883	0	7.49	7.76	7.59	8.28	0	0.266	1.203	0.635	0	0.093	1.066	1.008	1	0.783	1.721	0.073
1377619_at	LOC688637	similar to WD repeat domain 36	uniq_RT1uvsDR_883	0	5.64	6.19	6.05	6.45	0	0.545	1.459	0.162	0	0.404	1.323	0.283	1	0.809	1.752	0.082
1377642_at	Cav2	caveolin 2	uniq_RT1uvsDR_883	0	7.63	7.42	7.37	6.98	0	-0.211	-1.157	0.565	0	-0.256	-1.194	0.513	1	-0.646	-1.565	0.033
1377651_at	---	---	uniq_RT1uvsDR_883	0	3.40	3.66	3.69	5.75	0	0.267	1.203	0.632	0	0.289	1.222	0.495	1	2.354	5.111	0.001
1377663_at	Rnd3	Rho family GTPase 3	uniq_RT1uvsDR_883	0	6.61	6.69	6.56	5.98	0	0.079	1.056	1.020	0	-0.054	-1.038	0.943	1	-0.631	-1.548	0.043
1377758_at	Hsd17b13	hydroxysteroid (17-beta) dehydrogenase 13	uniq_RT1uvsDR_883	0	3.91	4.19	3.61	4.68	0	0.281	1.215	0.580	0	-0.299	-1.231	0.462	1	0.767	1.701	0.077
1377823_at	---	---	uniq_RT1uvsDR_883	0	5.13	4.92	5.24	5.99	0	-0.213	-1.159	0.722	0	0.111	1.080	0.781	1	0.855	1.809	0.051
1377910_at	Nop58	NOP58 ribonucleoprotein	uniq_RT1uvsDR_883	0	5.40	5.52	5.30	6.13	0	0.124	1.090	0.979	0	-0.100	-1.072	0.944	1	0.734	1.663	0.095
1377934_at	---	---	uniq_RT1uvsDR_883	0	6.84	7.14	7.22	6.33	0	0.302	1.233	0.515	0	0.382	1.303	0.334	1	-0.510	-1.424	0.093
1378080_at	Sco1	SCO1 cytochrome c oxidase assembly protein	uniq_RT1uvsDR_883	0	7.42	7.58	7.62	6.90	0	0.163	1.119	0.891	0	0.202	1.150	0.744	1	-0.520	-1.434	0.085
1378097_at	---	---	uniq_RT1uvsDR_883	0	5.23	5.28	5.48	4.62	0	0.051	1.036	1.135	0	0.250	1.189	0.598	1	-0.613	-1.530	0.042
1378098_at	---	---	uniq_RT1uvsDR_883	0	8.50	8.65	8.54	9.36	0	0.149	1.109	0.940	0	0.032	1.023	1.126	1	0.855	1.808	0.051
1378101_at	Ppp1r17	protein phosphatase 1, regulatory subunit 17	uniq_RT1uvsDR_883	0	3.76	3.63	3.83	4.89	0	-0.138	-1.100	0.885	0	0.063	1.044	0.852	1	1.123	2.178	0.020
1378120_at	Mrto4	mRNA turnover 4 homolog (S. cerevisiae)	uniq_RT1uvsDR_883	0	6.27	6.21	6.28	5.72	0	-0.051	-1.036	1.130	0	0.015	1.010	1.094	1	-0.548	-1.462	0.072
1378187_at	Dnajc7	DnaJ (Hsp40) homolog, subfamily C, member 7	uniq_RT1uvsDR_883	0	3.95	3.63	3.62	3.43	0	-0.323	-1.251	0.446	0	-0.336	-1.262	0.369	1	-0.523	-1.437	0.089
1378203_at	Smarce1	SWI/SNF related, matrix associated, actin dependen	uniq_RT1uvsDR_883	0	7.82	7.91	7.93	7.30	0	0.097	1.070	1.072	0	0.109	1.078	1.005	1	-0.515	-1.429	0.090
1378254_at	RGD1561609	similar to TBC1 domain family member 4	uniq_RT1uvsDR_883	0	4.77	5.04	4.87	6.31	0	0.275	1.210	0.600	0	0.106	1.076	0.973	1	1.544	2.916	0.006
1378271_at	---	---	uniq_RT1uvsDR_883	0	5.97	5.81	5.98	5.36	0	-0.160	-1.117	0.913	0	0.010	1.007	1.128	1	-0.614	-1.530	0.038
1378305_at	Tm4sf1	transmembrane 4 L six family member 1	uniq_RT1uvsDR_883	0	6.37	6.40	6.35	5.67	0	0.024	1.017	1.163	0	-0.019	-1.014	1.053	1	-0.698	-1.622	0.023
1378338_at	Ammecr1l	AMME chromosomal region gene 1-like	uniq_RT1uvsDR_883	0	4.70	4.92	4.77	6.05	0	0.212	1.158	0.793	0	0.064	1.045	1.080	1	1.348	2.545	0.010
1378350_at	---	---	uniq_RT1uvsDR_883	0	6.25	6.43	6.57	7.23	0	0.179	1.132	0.766	0	0.317	1.246	0.423	1	0.981	1.973	0.032
1378379_at	Cox11 /// LO(	cytochrome c oxidase assembly homolog 11 (yeast)	uniq_RT1uvsDR_883	0	5.04	4.68	4.76	4.48	0	-0.356	-1.280	0.358	0	-0.278	-1.212	0.519	1	-0.561	-1.475	0.059
1378391_at	---	---	uniq_RT1uvsDR_883	0	7.47	7.66	7.64	6.88	0	0.193	1.143	0.826	0	0.173	1.127	0.782	1	-0.590	-1.505	0.048
1378421_at	LOC1009109(	uncharacterized LOC100910996	uniq_RT1uvsDR_883	0	11.49	11.68	11.72	10.42	0	0.198	1.147	0.827	0	0.234	1.176	0.683	1	-1.062	-2.088	0.001
1378509_at	Kif3a	kinesin family member 3a	uniq_RT1uvsDR_883	0	6.13	6.35	6.07	5.49	0	0.221	1.166	0.746	0	-0.058	-1.041	0.964	1	-0.637	-1.555	0.035

Affymetrix Probeset ID	Gene Symbol	Gene Title	Figure 7 A Venn n=2289	Present in any 2 analyses n=423 1=yes 0=no	Mean log2 intensity DR+/+ ND	Mean log2 intensity DR+/+ HCD	Mean log2 intensity DR+/+ B/S	Mean log2 intensity F+/+ day 40 ND	DR+/+HCD vs DR+/+ND FDR<10% Log2 ratio >0.5 n=636	Log2 ratio DR+/+HCD vs DR+/+ ND	Fold-Change DR+/+HCD vs DR+/+ ND	FDR DR+/+HCD vs DR+/+ ND	DR+/+B/S vs DR+/+ND Log2 ratio >0.5 n=350	Log2 ratio DR+/+ B/S vs DR+/+ ND	Fold-Change DR+/+B/S vs DR+/+ ND	FDR DR+/+B/S vs DR+/+ ND	F+/+ vs DR+/+ND Log2 ratio >0.5 n=1209	Log2 ratio F+/+ vs DR+/+ ND	Fold-Change F+/+ vs DR+/+ ND	FDR F+/+ vs DR+/+ ND
1378580_at	---	---	uniq_RT1uvsDR_883	0	11.35	11.31	11.46	8.44	0	-0.035	-1.024	1.131	0	0.113	1.081	1.000	1	-2.905	-7.492	0.000
1378665_at	---	---	uniq_RT1uvsDR_883	0	7.85	7.80	7.95	8.61	0	-0.053	-1.038	1.066	0	0.102	1.073	1.027	1	0.765	1.699	0.088
1378678_at	---	---	uniq_RT1uvsDR_883	0	6.16	6.07	6.26	5.41	0	-0.085	-1.060	1.072	0	0.105	1.075	0.989	1	-0.751	-1.683	0.010
1378708_at	---	---	uniq_RT1uvsDR_883	0	4.89	4.56	4.69	4.34	0	-0.331	-1.258	0.416	0	-0.200	-1.148	0.724	1	-0.550	-1.464	0.066
1378738_at	Kcnab1	potassium voltage-gated channel, shaker-related su	uniq_RT1uvsDR_883	0	5.45	5.28	5.36	4.64	0	-0.165	-1.121	0.825	0	-0.090	-1.065	0.888	1	-0.813	-1.757	0.007
1378742_at	---	---	uniq_RT1uvsDR_883	0	7.16	7.03	7.21	6.51	0	-0.132	-1.096	0.821	0	0.046	1.033	1.094	1	-0.649	-1.568	0.028
1378757_at	RGD1310257	similar to RIKEN cDNA 6330408A02 gene	uniq_RT1uvsDR_883	0	5.30	5.22	5.14	4.59	0	-0.073	-1.052	1.092	0	-0.158	-1.115	0.789	1	-0.702	-1.627	0.017
1378803_at	Nkx6-2	NK6 homeobox 2	uniq_RT1uvsDR_883	0	4.12	3.80	3.74	3.61	0	-0.324	-1.251	0.432	0	-0.385	-1.306	0.235	1	-0.514	-1.428	0.096
1378838_at	---	---	uniq_RT1uvsDR_883	0	4.16	4.58	4.08	5.33	0	0.427	1.344	0.309	0	-0.079	-1.056	1.009	1	1.168	2.247	0.015
1378914_a_at	LOC308990	hypothetical protein LOC308990	uniq_RT1uvsDR_883	0	6.26	6.58	6.35	5.47	0	0.323	1.251	0.456	0	0.092	1.066	0.982	1	-0.785	-1.723	0.009
1378915_x_at	LOC308990	hypothetical protein LOC308990	uniq_RT1uvsDR_883	0	5.54	5.45	5.46	4.72	0	-0.093	-1.066	0.858	0	-0.084	-1.060	0.859	1	-0.823	-1.768	0.008
1378930_a_at	LOC1009125	uncharacterized LOC100912518	uniq_RT1uvsDR_883	0	7.22	7.19	7.26	6.52	0	-0.029	-1.021	1.094	0	0.038	1.026	1.094	1	-0.706	-1.631	0.019
1378983_at	---	---	uniq_RT1uvsDR_883	0	4.65	4.18	4.36	4.08	0	-0.467	-1.382	0.129	0	-0.286	-1.219	0.476	1	-0.572	-1.487	0.092
1379069_at	LOC1001923	uncharacterized LOC100192314	uniq_RT1uvsDR_883	0	5.67	5.45	5.49	5.07	0	-0.226	-1.170	0.717	0	-0.177	-1.131	0.711	1	-0.597	-1.513	0.044
1379092_at	---	---	uniq_RT1uvsDR_883	0	6.86	6.95	7.11	8.12	0	0.091	1.065	1.023	0	0.245	1.185	0.588	1	1.256	2.389	0.012
1379105_at	---	---	uniq_RT1uvsDR_883	0	5.31	4.99	5.11	4.40	0	-0.324	-1.252	0.379	0	-0.207	-1.154	0.611	1	-0.917	-1.888	0.003
1379112_at	Arid4a	AT rich interactive domain 4A (Rbp1 like)	uniq_RT1uvsDR_883	0	6.62	6.59	6.63	6.08	0	-0.038	-1.027	0.960	0	0.010	1.007	1.141	1	-0.539	-1.453	0.098
1379126_at	---	---	uniq_RT1uvsDR_883	0	8.22	8.08	8.20	9.05	0	-0.140	-1.102	0.691	0	-0.028	-1.020	1.105	1	0.826	1.773	0.062
1379132_at	---	---	uniq_RT1uvsDR_883	0	3.78	4.28	4.08	4.61	0	0.500	1.414	0.244	0	0.304	1.234	0.481	1	0.832	1.780	0.059
1379139_x_at	---	---	uniq_RT1uvsDR_883	0	4.50	4.26	4.34	3.82	0	-0.238	-1.179	0.667	0	-0.159	-1.117	0.830	1	-0.679	-1.601	0.021
1379188_at	Arid2	AT rich interactive domain 2 (Arid-rfx like)	uniq_RT1uvsDR_883	0	7.83	7.72	7.90	7.20	0	-0.109	-1.079	0.984	0	0.078	1.055	1.061	1	-0.629	-1.547	0.032
1379214_at	---	---	uniq_RT1uvsDR_883	0	2.83	3.10	3.01	5.46	0	0.271	1.207	0.607	0	0.178	1.131	0.834	1	2.627	6.177	0.000
1379249_at	Wtap	Wilms tumor 1 associated protein	uniq_RT1uvsDR_883	0	7.63	7.64	7.78	6.69	0	0.013	1.009	1.170	0	0.150	1.110	0.899	1	-0.934	-1.911	0.003
1379257_at	Epb41l4a	erythrocyte membrane protein band 4.1 like 4A	uniq_RT1uvsDR_883	0	8.17	8.23	8.26	7.65	0	0.053	1.038	1.139	0	0.082	1.058	1.064	1	-0.523	-1.437	0.087
1379286_at	Rnf152	ring finger protein 152	uniq_RT1uvsDR_883	0	5.15	5.10	5.17	6.08	0	-0.046	-1.033	1.110	0	0.021	1.015	1.116	1	0.929	1.904	0.041
1379358_at	---	---	uniq_RT1uvsDR_883	0	3.79	3.70	3.60	3.24	0	-0.094	-1.068	1.043	0	-0.186	-1.138	0.767	1	-0.550	-1.464	0.063
1379446_at	---	---	uniq_RT1uvsDR_883	0	4.75	4.31	4.88	3.59	0	-0.440	-1.357	0.125	0	0.135	1.098	0.396	1	-1.156	-2.229	0.001
1379453_a_at	---	---	uniq_RT1uvsDR_883	0	3.66	3.59	4.02	5.09	0	-0.077	-1.055	0.673	0	0.359	1.282	0.367	1	1.423	2.682	0.007
1379482_at	Tm6sf1	transmembrane 6 superfamily member 1	uniq_RT1uvsDR_883	0	7.61	7.83	7.79	6.78	0	0.220	1.165	0.778	0	0.177	1.131	0.837	1	-0.826	-1.772	0.007
1379497_at	---	---	uniq_RT1uvsDR_883	0	6.55	6.99	6.66	7.47	0	0.437	1.354	0.295	0	0.115	1.083	0.951	1	0.926	1.900	0.037
1379513_at	Tmem30b	transmembrane protein 30B	uniq_RT1uvsDR_883	0	7.05	7.45	7.37	6.27	0	0.398	1.318	0.359	0	0.315	1.244	0.465	1	-0.779	-1.716	0.012
1379573_at	LOC1009121	uncharacterized LOC100912188	uniq_RT1uvsDR_883	0	6.23	6.51	6.52	6.97	0	0.285	1.219	0.555	0	0.293	1.226	0.515	1	0.747	1.679	0.091
1379607_at	---	---	uniq_RT1uvsDR_883	0	6.53	6.49	6.55	7.30	0	-0.047	-1.033	1.097	0	0.017	1.012	1.111	1	0.766	1.701	0.086
1379645_at	Pbrm1	polybromo 1	uniq_RT1uvsDR_883	0	8.75	8.99	8.82	8.24	0	0.248	1.187	0.699	0	0.074	1.053	1.032	1	-0.509	-1.423	0.097
1379673_at	Uap1l1	UDP-N-acteylglucosamine pyrophosphorylase 1-like	uniq_RT1uvsDR_883	0	6.62	7.11	6.93	7.40	0	0.489	1.403	0.253	0	0.309	1.239	0.448	1	0.779	1.716	0.079
1379696_at	---	---	uniq_RT1uvsDR_883	0	4.27	4.12	4.18	5.37	0	-0.145	-1.106	0.770	0	-0.085	-1.061	0.952	1	1.106	2.153	0.020
1379748_at	Ifi44l	interferon-induced protein 44-like	uniq_RT1uvsDR_883	0	4.83	5.21	5.21	6.58	0	0.371	1.293	0.408	0	0.377	1.299	0.345	1	1.742	3.345	0.006
1379772_at	Aplnr	apelin receptor	uniq_RT1uvsDR_883	0	4.89	4.70	4.96	4.00	0	-0.189	-1.140	0.590	0	0.067	1.048	0.947	1	-0.894	-1.859	0.006
1379859_at	---	---	uniq_RT1uvsDR_883	0	5.57	5.56	5.65	4.78	0	-0.016	-1.011	1.111	0	0.080	1.057	1.071	1	-0.795	-1.735	0.008
1379863_at	Kcnd2	potassium voltage-gated channel, Shal-related subf	uniq_RT1uvsDR_883	0	4.42	4.38	4.49	5.72	0	-0.043	-1.031	1.148	0	0.064	1.046	1.064	1	1.295	2.454	0.012
1379879_at	---	---	uniq_RT1uvsDR_883	0	5.78	5.88	5.98	6.81	0	0.096	1.069	1.040	0	0.191	1.142	0.736	1	1.028	2.039	0.025
1379903_at	---	---	uniq_RT1uvsDR_883	0	8.67	8.86	9.09	7.36	0	0.191	1.141	0.838	0	0.421	1.339	0.259	1	-1.307	-2.475	0.000
1379964_at	---	---	uniq_RT1uvsDR_883	0	5.50	5.49	5.73	6.92	0	-0.014	-1.010	0.952	0	0.228	1.172	0.606	1	1.424	2.683	0.008
1379996_at	---	---	uniq_RT1uvsDR_883	0	6.92	6.97	7.38	6.05	0	0.049	1.034	1.150	0	0.466	1.381	0.212	1	-0.866	-1.823	0.005
1380044_x_at	---	---	uniq_RT1uvsDR_883	0	3.46	3.78	3.51	4.44	0	0.315	1.244	0.503	0	0.044	1.031	1.078	1	0.972	1.962	0.030
1380050_at	---	---	uniq_RT1uvsDR_883	0	5.67	5.51	5.86	7.01	0	-0.157	-1.115	0.880	0	0.190	1.141	0.764	1	1.336	2.524	0.010
1380094_a_at	Zfp212	Zinc finger protein 212	uniq_RT1uvsDR_883	0	4.32	4.47	4.45	5.48	0	0.149	1.109	0.837	0	0.129	1.093	0.843	1	1.166	2.243	0.020
1380184_at	Fam19a5	family with sequence similarity 19 (chemokine (C-C m	uniq_RT1uvsDR_883	0	5.87	5.72	6.00	5.08	0	-0.146	-1.106	0.969	0	0.139	1.101	0.923	1	-0.789	-1.727	0.010
1380206_at	---	---	uniq_RT1uvsDR_883	0	6.74	6.71	6.92	5.44	0	-0.033	-1.023	1.096	0	0.178	1.132	0.785	1	-1.298	-2.458	0.000
1380321_at	Mtus1	microtubule associated tumor suppressor 1	uniq_RT1uvsDR_883	0	7.65	8.10	7.80	7.09	0	0.456	1.372	0.274	0	0.153	1.112	0.900	1	-0.560	-1.475	0.062
1380340_at	---	---	uniq_RT1uvsDR_883	0	4.43	4.41	4.61	3.90	0	-0.020	-1.014	1.150	0	0.176	1.130	0.800	1	-0.531	-1.445	0.081
1380358_at	---	---	uniq_RT1uvsDR_883	0	6.18	6.31	6.27	5.56	0	0.128	1.093	0.904	0	0.084	1.060	0.915	1	-0.620	-1.537	0.042
1380366_at	Igh-6 /// LOC	immunoglobulin heavy chain 6 /// similar to single c	uniq_RT1uvsDR_883	0	6.21	5.81	6.05	4.91	0	-0.395	-1.315	0.138	0	-0.160	-1.117	0.434	1	-1.295	-2.454	0.001
1380371_at	Nipbl	Nipped-B homolog (Drosophila)	uniq_RT1uvsDR_883	0	8.65	8.97	8.64	9.58	0	0.315	1.244	0.485	0	-0.008	-1.006	0.909	1	0.927	1.901	0.042
1380376_at	---	---	uniq_RT1uvsDR_883	0	6.36	6.53	6.77	5.78	0	0.168	1.124	0.894	0	0.410	1.329	0.284	1	-0.581	-1.496	0.051
1380383_at	Arl4d	ADP-ribosylation factor-like 4D	uniq_RT1uvsDR_883	0	8.35	8.51	8.58	7.67	0	0.160	1.117	0.911	0	0.228	1.172	0.681	1	-0.678	-1.600	0.020
1380392_at	Brd4	bromodomain containing 4	uniq_RT1uvsDR_883	0	6.78	6.56	6.98	5.66	0	-0.225	-1.169	0.499	0	0.198	1.147	0.777	1	-1.126	-2.182	0.001
1380393_at	Cryz	crystallin, zeta (quinone reductase)	uniq_RT1uvsDR_883	0	5.65	5.81	5.77	5.10	0	0.159	1.116	0.884	0	0.126	1.091	0.901	1	-0.542	-1.456	0.079
1380407_at	RGD1310352	similar to HTGN29 protein; keratinocytes associated	uniq_RT1uvsDR_883	0	9.88	9.94	9.95	8.59	0	0.053	1.038	1.144	0	0.062	1.044	1.093	1	-1.297	-2.458	0.000
1380464_at	Ints6	integrator complex subunit 6	uniq_RT1uvsDR_883	0	3.85	3.64	3.94	4.92	0	-0.204	-1.152	0.623	0	0.089	1.064	0.845	1	1.073	2.103	0.022
1380477_at	---	---	uniq_RT1uvsDR_883	0	6.41	5.99	5.99	5.82	0	-0.417	-1.335	0.213	0	-0.417	-1.335	0.200	1	-0.591	-1.506	0.050
1380489_at	---	---	uniq_RT1uvsDR_883	0	5.94	5.79	5.90	6.70	0	-0.149	-1.109	0.871	0	-0.040	-1.028	1.052	1	0.762	1.695	0.089
1380492_at	Ncs1	neuronal calcium sensor 1	uniq_RT1uvsDR_883	0	5.23	5.01	5.01	4.73	0	-0.228	-1.171	0.690	0	-0.221	-1.166	0.687	1	-0.509	-1.423	0.096
1380514_at	Zic4	Zic family member 4	uniq_RT1uvsDR_883	0	4.64	4.17	4.43	4.03	0	-0.471	-1.386	0.117	0	-0.209	-1.156	0.688	1	-0.613	-1.529	0.035
1380547_at	Clcn3	chloride channel, voltage-sensitive 3	uniq_RT1uvsDR_883	0	8.73	8.81	8.85	8.18	0	0.082	1.058	1.070	0	0.122	1.088	0.890	1	-0.552	-1.466	0.066
1380610_at	---	---	uniq_RT1uvsDR_883	0	5.25	5.56	5.53	4.66	0	0.313	1.242	0.545	0	0.279	1.213	0.519	1	-0.587	-1.503	0.049
1380613_at	---	---	uniq_RT1uvsDR_883	0	4.18	3.84	3.82	3.66	0	-0.340	-1.266	0.413	0	-0.363	-1.286	0.284	1	-0.524	-1.438	0.100
1380638_at	Ncoa3	nuclear receptor coactivator 3	uniq_RT1uvsDR_883	0	6.59	6.49	6.53	5.79	0	-0.100	-1.071	0.990	0	-0.062	-1.044	1.075	1	-0.800	-1.741	0.008
1380670_at	---	---	uniq_RT1uvsDR_883	0	4.15	4.02	4.33	4.96	0	-0.129	-1.094	0.889	0	0.178	1.131	0.757	1	0.812	1.756	0.065
1380688_at	Galnt18	UDP-N-acetyl-alpha-D-galactosamine:polypeptide N	uniq_RT1uvsDR_883	0	7.20	7.42	7.36	6.43	0	0.221	1.165	0.700	0	0.152	1.111	0.879	1	-0.777	-1.713	0.009
1380693_at	---	---	uniq_RT1uvsDR_883	0	7.57	7.76	7.82	5.37	0	0.188	1.139	0.864	0	0.247	1.186	0.632	1	-2.207	-4.617	0.000
1380699_at	---	---	uniq_RT1uvsDR_883	0	6.05	5.67	5.66	5.32	0	-0.375	-1.297	0.226	0	-0.390	-1.311	0.207	1	-0.733	-1.662	0.014
1380726_at	---	---	uniq_RT1uvsDR_883	0	6.77	6.70	6.84	5.86	0	-0.067	-1.048	0.990	0	0.067	1.048	0.967	1	-0.909	-1.878	0.005
1380763_at	---	---	uniq_RT1uvsDR_883	0	6.06	6.34	6.44	7.20	0	0.284	1.218	0.512	0	0.378	1.300	0.292	1	1.144	2.210	0.019
1380786_at	---	---	uniq_RT1uvsDR_883	0	3.89	3.62	3.70	3.38	0	-0.272	-1.208	0.595	0	-0.197	-1.147	0.701	1	-0.515	-1.429	0.087
1380850_at	---	---	uniq_RT1uvsDR_883	0	5.66	6.19	5.76	7.29	0	0.534	1.448	0.191	0	0.107	1.077	0.810	1	1.635	3.105	0.005
1380880_at	---	---	uniq_RT1uvsDR_883	0	3.05	2.90	2.85	2.52	0	-0.159	-1.116	0.854	0	-0.204	-1.152	0.693	1	-0.530	-1.444	0.077
1380915_at	---	---	uniq_RT1uvsDR_883	0	5.69	5.70	5.76	5.10	0	0.014	1.009	1.137	0	0.073	1.052	1.061	1	-0.583	-1.498	0.055
1380964_at	---	---	uniq_RT1uvsDR_883	0	4.56	4.55	4.21	5.32	0	-0.001	-1.000	1.114	0	-0.347	-1.272	0.305	1	0.763	1.697	0.093
1380976_at	---	---	uniq_RT1uvsDR_883	0	5.28	5.11	5.31	6.19	0	-0.168	-1.123	0.833	0	0.032	1.023	1.093	1	0.913	1.883	0.040
1381010_at	---	---	uniq_RT1uvsDR_883	0	3.55	3.61	3.51	4.37	0	0.060	1.042	1.085	0	-0.038	-1.027	1.085	1	0.820	1.765	0.068
1381042_at	---	---	uniq_RT1uvsDR_883	0	3.05	3.01	3.08	3.97	0	-0.049	-1.035	1.134	0	0.024	1.017	1.092	1	0.911	1.880	0.041
1381069_at	Kmt2a	lysine (K)-specific methyltransferase 2A	uniq_RT1uvsDR_883	0	5.80	5.72	6.14	4.64	0	-0.076	-1.054	0.991	0	0.343	1.268	0.398	1	-1.158	-2.232	0.001
1381147_at	---	---	uniq_RT1uvsDR_883	0	4.82	5.41	5.26	5.59	0	0.585	1.500	0.119	0	0.434	1.351	0.235	1	0.765	1.699	0.083
1381165_at	---	---	uniq_RT1uvsDR_883	0	3.42	3.62	3.78	4.41	0	0.208	1.155	0.679	0	0.366	1.289	0.333	1	0.992	1.988	0.031
1381211_at	---	---	uniq_RT1uvsDR_883	0	6.59	6.16	6.29	6.06	0	-0.433	-1.350	0.194	0	-0.309	-1.238	0.424	1	-0.539	-1.453	0.067
1381222_at	---	---	uniq_RT1uvsDR_883	0	4.86	5.16	5.00	5.70	0	0.303	1.233	0.535	0	0.137	1.100	0.889	1	0.841	1.791	0.055
1381228_at	---	---	uniq_RT1uvsDR_883	0	6.04	6.17	6.31	5.18	0	0.128	1.093	1.008	0	0.266	1.203	0.590	1	-0.864	-1.820	0.005
1381247_at	---	---	uniq_RT1uvsDR_883	0	3.68	3.49	3.43	3.13	0	-0.195	-1.145	0.690	0	-0.258	-1.195	0.575	1	-0.550	-1.464	0.080
1381282_at	---	---	uniq_RT1uvsDR_883	0	6.77	6.68	6.82	6.22	0	-0.090	-1.064	1.052	0	0.055	1.039	1.024	1	-0.546	-1.460	0.072
1381386_at	Pop5	processing of precursor 5, ribonuclease P/MRP subu	uniq_RT1uvsDR_883	0	4.62	4.56	4.66	5.81	0	-0.057	-1.040	1.088	0	0.045	1.032	0.991	1	1.194	2.287	0.015
1381482_at	---	---	uniq_RT1uvsDR_883	0	5.17	4.94	5.15	6.07	0	-0.235	-1.177	0.686	0	-0.019	-1.013	1.096	1	0.901	1.868	0.042
1381504_at	Aspn	asporin	uniq_RT1uvsDR_883	0	5.95	6.09	5.98	5.33	0	0.141	1.103	0.908	0	0.028	1.020	0.955	1	-0.623	-1.540	0.041
1381512_at	---	---	uniq_RT1uvsDR_883	0	5.76	5.37	5.57	5.18	0	-0.396	-1.316	0.259	0	-0.197	-1.147	0.759	1	-0.586	-1.501	0.067
1381524_at	---	---	uniq_RT1uvsDR_883	0	5.16	4.74	5.01	4.56	0	-0.428	-1.345	0.199	0	-0.151	-1.110	0.842	1	-0.607	-1.523	0.041
1381576_at	Pik3c2b	phosphatidylinositol-4-phosphate 3-kinase, catalyti	uniq_RT1uvsDR_883	0	5.92	5.66	5.70	5.03	0	-0.256	-1.194	0.542	0	-0.215	-1.161	0.536	1	-0.886	-1.848	0.005
1381590_at	---	---	uniq_RT1uvsDR_883	0	5.83	5.64	6.01	5.14	0	-0.191	-1.142	0.676	0	0.187	1.139	0.710	1	-0.690	-1.613	0.020
1381621_at	---	---	uniq_RT1uvsDR_883	0	6.72	6.86	6.99	5.99	0	0.140	1.102	0.968	0	0.265	1.201	0.579	1	-0.734	-1.663	0.014
1381650_at	Sephs1	selenophosphate synthetase 1	uniq_RT1uvsDR_883	0	6.29	6.47	6.57	5.55	0	0.183	1.135	0.826	0	0.280	1.214	0.512	1	-0.736	-1.665	0.013
1381708_at	Zfyve1	zinc finger, FYVE domain containing 1	uniq_RT1uvsDR_883	0	4.39	4.46	4.37	3.70	0	0.067	1.048	1.101	0	-0.021	-1.014	1.075	1	-0.698	-1.622	0.020
1381709_at	Rnf19b	ring finger protein 19B	uniq_RT1uvsDR_883	0	6.08	5.58	5.80	5.00	0	-0.498	-1.412	0.089	0	-0.286	-1.219	0.477	1	-1.077	-2.109	0.002
1381726_at	---	---	uniq_RT1uvsDR_883	0	4.55	4.17	4.30	3.84	0	-0.386	-1.307	0.306	0	-0.255	-1.194	0.601	1	-0.709	-1.634	0.016
1381741_at	---	---	uniq_RT1uvsDR_883	0	6.01	5.66	5.92	5.10	0	-0.349	-1.274	0.403	0	-0.088	-1.063	1.037	1	-0.910	-1.879	0.003
1381753_at	---	---	uniq_RT1uvsDR_883	0	4.20	3.82	4.25	3.65	0	-0.381	-1.302	0.241	0	0.054	1.038	1.085	1	-0.547	-1.461	0.068
1381764_s_at	Rnf126	ring finger protein 126	uniq_RT1uvsDR_883	0	6.74	6.36	6.67	6.05	0	-0.374	-1.296	0.309	0	-0.066	-1.047	1.059	1	-0.686	-1.609	0.020
1381795_at	---	---	uniq_RT1uvsDR_883	0	4.21	4.21	4.36	3.61	0	-0.002	-1.001	0.990	0	0.154	1.112	0.901	1	-0.595	-1.511	0.042
1381829_at	Zfp318	zinc finger protein 318	uniq_RT1uvsDR_883	0	3.81	3.55	3.72	3.28	0	-0.252	-1.191	0.476	0	-0.085	-1.060	0.932	1	-0.530	-1.444	0.086

Affymetrix Probeset ID	Gene Symbol	Gene Title	Figure 7 A Venn n=2289	Present in any 2 analyses n=423 1=yes 0=no	Mean log2 intensity DR+/+ ND	Mean log2 intensity DR+/+ HCD	Mean log2 intensity DR+/+ B/S	Mean log2 intensity F+/+ day 40 ND	DR+/+HCD vs DR+/+ND FDR<10% Log2 ratio >0.5 n=636	Log2 ratio DR+/+HCD vs DR+/+ ND	Fold-Change DR+/+HCD vs DR+/+ ND	FDR DR+/+HCD vs DR+/+ ND	DR+/+B/S vs DR+/+ND Log2 ratio >0.5 n=350	Log2 ratio DR+/+ B/S vs DR+/+ ND	Fold-Change DR+/+B/S vs DR+/+ ND	FDR DR+/+B/S vs DR+/+ ND	F+/+ vs DR+/+ND Log2 ratio >0.5 n=1209	Log2 ratio F+/+ vs DR+/+ ND	Fold-Change F+/+ vs DR+/+ ND	FDR F+/+ vs DR+/+ ND
1381886_at	Yy1	YY1 transcription factor	uniq_RT1uvsDR_883	0	4.88	4.59	4.79	4.17	0	-0.287	-1.220	0.431	0	-0.089	-1.063	1.005	1	-0.709	-1.635	0.023
1381905_at	---	---	uniq_RT1uvsDR_883	0	7.22	7.23	7.42	6.45	0	0.013	1.009	1.160	0	0.200	1.149	0.731	1	-0.774	-1.710	0.010
1381953_at	St6galnac2	ST6 (alpha-N-acetyl-neuraminyl-2,3-beta-galactosyl-	uniq_RT1uvsDR_883	0	4.25	3.97	4.09	3.72	0	-0.288	-1.221	0.489	0	-0.163	-1.119	0.769	1	-0.531	-1.445	0.083
1381990_at	Galnt1	UDP-N-acetyl-alpha-D-galactosamine:polypeptide N	uniq_RT1uvsDR_883	0	5.87	5.91	6.24	4.32	0	0.037	1.026	1.130	0	0.373	1.295	0.344	1	-1.553	-2.933	0.000
1381993_at	Clic2	chloride intracellular channel 2	uniq_RT1uvsDR_883	0	8.22	7.75	8.06	7.16	0	-0.467	-1.383	0.117	0	-0.161	-1.118	0.778	1	-1.057	-2.081	0.001
1382086_at	---	---	uniq_RT1uvsDR_883	0	5.82	5.68	5.75	5.25	0	-0.145	-1.106	0.770	0	-0.075	-1.054	0.853	1	-0.569	-1.484	0.064
1382088_at	Ryr2	ryanodine receptor 2, cardiac	uniq_RT1uvsDR_883	0	6.72	6.85	6.84	5.48	0	0.127	1.092	0.940	0	0.113	1.081	0.939	1	-1.247	-2.373	0.000
1382136_at	Slc2a9	solute carrier family 2 (facilitated glucose transporte	uniq_RT1uvsDR_883	0	6.03	6.19	6.29	5.40	0	0.162	1.119	0.691	0	0.267	1.204	0.492	1	-0.631	-1.549	0.032
1382203_at	Cers1 /// Gdf	ceramide synthase 1 /// growth differentiation facto	uniq_RT1uvsDR_883	0	8.25	8.20	8.46	7.62	0	-0.051	-1.036	1.134	0	0.203	1.151	0.749	1	-0.635	-1.553	0.035
1382206_a_at	Akap2	A kinase (PRKA) anchor protein 2	uniq_RT1uvsDR_883	0	6.80	6.38	6.46	5.39	0	-0.427	-1.345	0.159	0	-0.339	-1.265	0.339	1	-1.408	-2.654	0.000
1382234_at	---	---	uniq_RT1uvsDR_883	0	6.16	6.17	6.31	5.59	0	0.015	1.010	1.131	0	0.150	1.110	0.852	1	-0.567	-1.481	0.058
1382265_at	Ccser2	coiled-coil serine-rich protein 2	uniq_RT1uvsDR_883	0	7.46	7.48	7.50	6.96	0	0.023	1.016	1.169	0	0.045	1.031	1.116	1	-0.501	-1.415	0.098
1382296_at	---	---	uniq_RT1uvsDR_883	0	4.56	4.35	4.21	3.97	0	-0.218	-1.163	0.591	0	-0.356	-1.280	0.281	1	-0.589	-1.505	0.050
1382333_at	Thumpd3	THUMP domain containing 3	uniq_RT1uvsDR_883	0	5.44	5.67	5.81	6.30	0	0.228	1.171	0.725	0	0.373	1.295	0.357	1	0.865	1.821	0.047
1382351_at	Gem	GTP binding protein overexpressed in skeletal muscl	uniq_RT1uvsDR_883	0	7.34	7.91	7.34	9.24	0	0.568	1.482	0.138	0	0.006	1.004	1.111	1	1.906	3.748	0.004
1382368_at	Trove2	TROVE domain family, member 2	uniq_RT1uvsDR_883	0	4.38	4.79	4.74	5.40	0	0.414	1.332	0.350	0	0.361	1.284	0.350	1	1.017	2.024	0.030
1382499_at	---	---	uniq_RT1uvsDR_883	0	5.04	4.86	5.09	4.29	0	-0.182	-1.134	0.642	0	0.049	1.034	1.064	1	-0.753	-1.686	0.011
1382517_at	---	---	uniq_RT1uvsDR_883	0	4.61	4.91	4.84	5.54	0	0.294	1.226	0.585	0	0.231	1.173	0.688	1	0.927	1.901	0.041
1382522_at	Matr3	matrin 3	uniq_RT1uvsDR_883	0	8.17	8.24	8.34	7.50	0	0.072	1.051	1.097	0	0.171	1.126	0.843	1	-0.676	-1.597	0.022
1382682_at	---	---	uniq_RT1uvsDR_883	0	6.63	6.60	6.58	5.55	0	-0.036	-1.025	1.063	0	-0.052	-1.037	0.983	1	-1.078	-2.111	0.001
1382696_at	Dscam	Down syndrome cell adhesion molecule	uniq_RT1uvsDR_883	0	4.56	4.92	4.89	5.35	0	0.361	1.284	0.450	0	0.332	1.259	0.425	1	0.791	1.730	0.084
1382699_s_at	Hps1	Hermansky-Pudlak syndrome 1 homolog (human)	uniq_RT1uvsDR_883	0	8.42	8.27	8.36	7.80	0	-0.147	-1.107	0.915	0	-0.059	-1.042	1.074	1	-0.623	-1.540	0.034
1382712_at	---	---	uniq_RT1uvsDR_883	0	8.13	8.15	8.29	7.20	0	0.019	1.013	0.773	0	0.157	1.115	0.564	1	-0.930	-1.905	0.005
1382735_at	Atg12	autophagy related 12	uniq_RT1uvsDR_883	0	6.58	7.04	6.46	7.71	0	0.459	1.375	0.281	0	-0.128	-1.093	0.855	1	1.131	2.190	0.018
1382737_at	RGD1304884	similar to RIKEN cDNA 6430548M08	uniq_RT1uvsDR_883	0	6.21	5.83	5.89	5.55	0	-0.382	-1.303	0.268	0	-0.321	-1.249	0.422	1	-0.655	-1.574	0.042
1382775_at	Ryr2	ryanodine receptor 2, cardiac	uniq_RT1uvsDR_883	0	5.39	5.28	5.46	4.74	0	-0.110	-1.079	0.773	0	0.066	1.047	0.743	1	-0.652	-1.571	0.033
1382797_at	Rint1	RAD50 interactor 1	uniq_RT1uvsDR_883	0	6.30	6.17	6.26	5.72	0	-0.137	-1.100	0.782	0	-0.042	-1.030	1.076	1	-0.583	-1.498	0.055
1382814_at	Tenm3	teneurin transmembrane protein 3	uniq_RT1uvsDR_883	0	7.35	7.39	7.57	8.10	0	0.046	1.032	1.152	0	0.221	1.166	0.714	1	0.753	1.686	0.091
1382841_at	Tssc4	tumor suppressing subtransferable candidate 4	uniq_RT1uvsDR_883	0	7.44	7.37	7.27	6.88	0	-0.078	-1.055	1.047	0	-0.176	-1.130	0.805	1	-0.565	-1.479	0.055
1382868_at	Sema6a	sema domain, transmembrane domain (TM), and cy	uniq_RT1uvsDR_883	0	5.99	5.82	6.11	5.29	0	-0.167	-1.123	0.773	0	0.119	1.086	0.632	1	-0.697	-1.621	0.020
1382929_at	LOC1009122	leukocyte receptor cluster member 9-like	uniq_RT1uvsDR_883	0	4.99	4.68	5.10	4.41	0	-0.314	-1.243	0.477	0	0.113	1.081	1.004	1	-0.585	-1.500	0.049
1382981_at	Ahi1	Abelson helper integration site 1	uniq_RT1uvsDR_883	0	7.10	7.46	7.33	8.03	0	0.362	1.286	0.422	0	0.230	1.173	0.480	1	0.934	1.910	0.040
1382998_at	---	---	uniq_RT1uvsDR_883	0	3.70	4.20	4.07	3.15	0	0.494	1.408	0.263	0	0.366	1.289	0.366	1	-0.559	-1.473	0.063
1383047_at	Gas6	growth arrest specific 6	uniq_RT1uvsDR_883	0	8.82	8.71	8.87	8.26	0	-0.104	-1.075	1.046	0	0.050	1.035	1.116	1	-0.560	-1.474	0.059
1383058_at	---	---	uniq_RT1uvsDR_883	0	6.49	6.63	6.67	5.73	0	0.144	1.105	0.943	0	0.187	1.138	0.788	1	-0.756	-1.689	0.011
1383087_at	---	---	uniq_RT1uvsDR_883	0	7.85	8.04	7.82	7.33	0	0.187	1.139	0.840	0	-0.033	-1.023	1.119	1	-0.523	-1.437	0.084
1383088_at	Lipt2	lipoyl(octanoyl) transferase 2 (putative)	uniq_RT1uvsDR_883	0	7.07	7.13	7.14	6.48	0	0.054	1.038	1.148	0	0.067	1.047	1.078	1	-0.589	-1.505	0.045
1383117_at	Pxmp4	peroxisomal membrane protein 4	uniq_RT1uvsDR_883	0	7.88	8.07	8.09	5.27	0	0.191	1.141	0.846	0	0.211	1.157	0.748	1	-2.613	-6.117	0.000
1383135_at	Ntrk2	neurotrophic tyrosine kinase, receptor, type 2	uniq_RT1uvsDR_883	0	9.72	9.45	9.63	9.16	0	-0.271	-1.207	0.590	0	-0.092	-1.066	1.004	1	-0.567	-1.482	0.053
1383227_at	LOC688311	similar to ADP-ribosylation factor-like 1	uniq_RT1uvsDR_883	0	7.64	7.59	7.69	6.75	0	-0.049	-1.034	1.129	0	0.053	1.037	1.067	1	-0.891	-1.855	0.004
1383289_at	Nif3l1	NIF3 NGG1 interacting factor 3-like 1 (S. cerevisiae)	uniq_RT1uvsDR_883	0	7.55	7.65	7.58	6.98	0	0.099	1.071	1.067	0	0.025	1.017	1.134	1	-0.568	-1.482	0.052
1383331_at	---	---	uniq_RT1uvsDR_883	0	4.19	3.95	4.16	7.74	0	-0.238	-1.179	0.597	0	-0.033	-1.023	1.097	1	3.552	11.729	0.000
1383338_at	---	---	uniq_RT1uvsDR_883	0	7.58	7.71	7.74	6.98	0	0.129	1.094	1.002	0	0.164	1.120	0.852	1	-0.594	-1.509	0.053
1383348_at	---	---	uniq_RT1uvsDR_883	0	6.67	6.86	6.90	6.02	0	0.184	1.136	0.838	0	0.226	1.170	0.688	1	-0.655	-1.575	0.027
1383371_at	Srcrb4d	scavenger receptor cysteine rich domain containing,	uniq_RT1uvsDR_883	0	5.71	5.57	5.60	4.95	0	-0.141	-1.103	0.901	0	-0.116	-1.083	0.964	1	-0.766	-1.700	0.010
1383383_at	---	---	uniq_RT1uvsDR_883	0	9.02	9.02	9.07	8.50	0	0.006	1.004	1.173	0	0.057	1.041	1.090	1	-0.513	-1.427	0.098
1383408_at	---	---	uniq_RT1uvsDR_883	0	6.18	6.48	6.42	6.94	0	0.298	1.229	0.548	0	0.239	1.180	0.625	1	0.761	1.695	0.090
1383424_at	Cmpk2	cytidine monophosphate (UMP-CMP) kinase 2, mito	uniq_RT1uvsDR_883	0	7.89	7.84	8.11	7.38	0	-0.055	-1.039	1.110	0	0.219	1.164	0.709	1	-0.516	-1.430	0.087
1383468_at	---	---	uniq_RT1uvsDR_883	0	7.87	7.79	8.07	7.36	0	-0.081	-1.058	1.017	0	0.197	1.147	0.769	1	-0.517	-1.431	0.092
1383552_at	---	---	uniq_RT1uvsDR_883	0	7.44	7.63	7.68	8.50	0	0.196	1.146	0.801	0	0.249	1.188	0.539	1	1.060	2.084	0.023
1383561_at	---	---	uniq_RT1uvsDR_883	0	6.97	7.18	7.08	6.06	0	0.214	1.160	0.778	0	0.107	1.077	0.986	1	-0.905	-1.873	0.004
1383566_at	LOC1009130	uncharacterized LOC100913073	uniq_RT1uvsDR_883	0	7.37	7.90	7.75	5.99	0	0.533	1.447	0.172	0	0.376	1.298	0.334	1	-1.384	-2.610	0.000
1383577_at	---	---	uniq_RT1uvsDR_883	0	3.76	3.44	3.75	4.67	0	-0.324	-1.252	0.448	0	-0.010	-1.007	1.126	1	0.908	1.877	0.063
1383641_at	Ednra	endothelin receptor type A	uniq_RT1uvsDR_883	0	7.14	6.67	6.94	6.34	0	-0.479	-1.394	0.091	0	-0.206	-1.154	0.589	1	-0.800	-1.741	0.009
1383661_at	---	---	uniq_RT1uvsDR_883	0	7.55	7.81	8.03	8.45	0	0.253	1.191	0.654	0	0.479	1.394	0.186	1	0.901	1.867	0.042
1383708_at	Itgbl1	integrin, beta-like 1	uniq_RT1uvsDR_883	0	5.02	4.53	4.86	4.21	0	-0.489	-1.403	0.095	0	-0.159	-1.117	0.826	1	-0.815	-1.759	0.013
1383770_at	---	---	uniq_RT1uvsDR_883	0	7.51	7.25	7.26	6.91	0	-0.254	-1.193	0.587	0	-0.241	-1.182	0.564	1	-0.600	-1.516	0.052
1383823_at	---	---	uniq_RT1uvsDR_883	0	6.72	6.75	6.80	5.96	0	0.034	1.024	1.130	0	0.085	1.061	1.041	1	-0.754	-1.686	0.012
1383852_at	---	---	uniq_RT1uvsDR_883	0	4.11	4.31	4.30	5.01	0	0.200	1.149	0.742	0	0.199	1.148	0.731	1	0.906	1.874	0.040
1383874_at	---	---	uniq_RT1uvsDR_883	0	4.94	5.04	5.14	5.73	0	0.104	1.074	1.001	0	0.204	1.152	0.675	1	0.794	1.733	0.080
1383879_at	LOC688163	hypothetical protein LOC688163	uniq_RT1uvsDR_883	0	7.21	7.58	7.30	8.82	0	0.378	1.299	0.400	0	0.093	1.067	1.004	1	1.615	3.062	0.005
1383886_at	---	---	uniq_RT1uvsDR_883	0	6.13	6.07	6.32	5.49	0	-0.065	-1.046	1.091	0	0.188	1.139	0.769	1	-0.641	-1.560	0.033
1383914_at	Mrvi1	murine retrovirus integration site 1 homolog	uniq_RT1uvsDR_883	0	6.86	6.58	6.53	6.09	0	-0.275	-1.210	0.554	0	-0.328	-1.256	0.371	1	-0.771	-1.706	0.009
1384013_at	Chl1	cell adhesion molecule with homology to L1CAM	uniq_RT1uvsDR_883	0	4.45	4.20	4.07	3.91	0	-0.251	-1.190	0.617	0	-0.380	-1.301	0.246	1	-0.541	-1.455	0.080
1384041_at	Dck	deoxycytidine kinase	uniq_RT1uvsDR_883	0	6.77	6.88	6.83	6.23	0	0.104	1.075	0.992	0	0.062	1.044	1.045	1	-0.546	-1.460	0.072
1384043_at	---	---	uniq_RT1uvsDR_883	0	5.62	6.06	5.87	4.88	0	0.442	1.358	0.325	0	0.255	1.193	0.592	1	-0.731	-1.660	0.022
1384052_at	---	---	uniq_RT1uvsDR_883	0	5.03	5.17	5.13	4.22	0	0.132	1.096	0.884	0	0.094	1.067	0.922	1	-0.816	-1.761	0.007
1384056_at	---	---	uniq_RT1uvsDR_883	0	8.05	7.75	7.89	7.50	0	-0.302	-1.233	0.437	0	-0.160	-1.117	0.784	1	-0.550	-1.464	0.072
1384082_at	LOC688311	similar to ADP-ribosylation factor-like 1	uniq_RT1uvsDR_883	0	5.63	5.77	5.81	5.04	0	0.144	1.105	0.922	0	0.182	1.135	0.764	1	-0.582	-1.497	0.057
1384110_at	---	---	uniq_RT1uvsDR_883	0	7.21	7.59	7.50	7.97	0	0.382	1.303	0.384	0	0.289	1.222	0.511	1	0.758	1.691	0.090
1384136_at	Osbpl3	oxysterol binding protein-like 3	uniq_RT1uvsDR_883	0	7.30	7.25	7.29	6.07	0	-0.053	-1.038	1.117	0	-0.011	-1.008	1.143	1	-1.230	-2.345	0.000
1384144_at	---	---	uniq_RT1uvsDR_883	0	5.64	5.35	5.30	5.11	0	-0.294	-1.226	0.547	0	-0.343	-1.268	0.335	1	-0.530	-1.444	0.073
1384148_at	Rab20	RAB20, member RAS oncogene family	uniq_RT1uvsDR_883	0	5.54	5.54	5.61	6.59	0	-0.003	-1.002	1.161	0	0.068	1.048	1.048	1	1.050	2.071	0.023
1384166_a_at	LOC681647	similar to F43G9.2	uniq_RT1uvsDR_883	0	6.47	6.21	6.74	7.67	0	-0.259	-1.197	0.563	0	0.267	1.203	0.588	1	1.200	2.297	0.013
1384167_at	LOC681647	similar to F43G9.2	uniq_RT1uvsDR_883	0	5.55	5.56	5.82	6.84	0	0.008	1.006	1.038	0	0.277	1.212	0.452	1	1.295	2.454	0.012
1384229_at	Gnal	guanine nucleotide binding protein, alpha stimulatir	uniq_RT1uvsDR_883	0	5.86	5.38	5.56	5.31	0	-0.477	-1.392	0.125	0	-0.304	-1.234	0.443	1	-0.550	-1.464	0.063
1384234_at	Prrx1	paired related homeobox 1	uniq_RT1uvsDR_883	0	4.73	4.51	4.49	4.20	0	-0.217	-1.163	0.674	0	-0.237	-1.178	0.609	1	-0.525	-1.439	0.084
1384253_at	Palld	palladin, cytoskeletal associated protein	uniq_RT1uvsDR_883	0	11.50	11.36	11.49	10.74	0	-0.147	-1.107	0.922	0	-0.015	-1.011	1.118	1	-0.763	-1.697	0.010
1384271_at	IgG-2a	gamma-2a immunoglobulin heavy chain	uniq_RT1uvsDR_883	0	5.36	5.03	5.08	4.74	0	-0.333	-1.260	0.223	0	-0.281	-1.215	0.339	1	-0.622	-1.539	0.067
1384275_at	LOC688328	hypothetical protein LOC688328	uniq_RT1uvsDR_883	0	6.26	6.52	6.52	5.58	0	0.263	1.200	0.635	0	0.259	1.196	0.555	1	-0.680	-1.602	0.026
1384308_at	Meis1	Meis homeobox 1	uniq_RT1uvsDR_883	0	9.47	9.53	9.64	8.95	0	0.060	1.043	1.127	0	0.167	1.123	0.870	1	-0.525	-1.439	0.078
1384312_at	Irx1	iroquois homeobox 1	uniq_RT1uvsDR_883	0	8.09	8.58	8.45	7.39	0	0.490	1.405	0.202	0	0.361	1.285	0.369	1	-0.698	-1.622	0.017
1384346_at	---	---	uniq_RT1uvsDR_883	0	3.55	3.24	3.30	4.38	0	-0.306	-1.236	0.482	0	-0.243	-1.183	0.564	1	0.831	1.778	0.085
1384407_at	Nmral1	NmrA-like family domain containing 1	uniq_RT1uvsDR_883	0	7.79	7.90	7.93	6.93	0	0.108	1.078	1.034	0	0.140	1.102	0.908	1	-0.857	-1.811	0.006
1384422_at	---	---	uniq_RT1uvsDR_883	0	6.18	6.17	6.50	4.96	0	-0.010	-1.007	1.160	0	0.322	1.250	0.425	1	-1.216	-2.323	0.000
1384425_at	LOC304131	similar to C21ORF7	uniq_RT1uvsDR_883	0	5.86	5.43	5.79	4.93	0	-0.429	-1.346	0.207	0	-0.065	-1.046	1.078	1	-0.927	-1.901	0.003
1384537_at	---	---	uniq_RT1uvsDR_883	0	6.57	6.34	6.48	5.38	0	-0.222	-1.166	0.715	0	-0.089	-1.064	0.981	1	-1.187	-2.276	0.001
1384538_at	Msto1	misato homolog 1 (Drosophila)	uniq_RT1uvsDR_883	0	5.67	5.49	5.56	5.08	0	-0.181	-1.134	0.812	0	-0.107	-1.077	0.959	1	-0.584	-1.499	0.050
1384558_at	Plac9	placenta-specific 9	uniq_RT1uvsDR_883	0	5.71	5.22	5.33	5.03	0	-0.490	-1.405	0.106	0	-0.379	-1.300	0.268	1	-0.677	-1.598	0.027
1384582_at	---	---	uniq_RT1uvsDR_883	0	6.30	6.77	6.54	7.05	0	0.465	1.381	0.290	0	0.236	1.178	0.633	1	0.754	1.687	0.091
1384599_at	---	---	uniq_RT1uvsDR_883	0	4.49	4.84	4.69	3.78	0	0.345	1.270	0.422	0	0.195	1.144	0.679	1	-0.716	-1.643	0.020
1384634_at	---	---	uniq_RT1uvsDR_883	0	6.02	6.17	6.27	6.78	0	0.147	1.107	0.942	0	0.252	1.191	0.574	1	0.760	1.694	0.093
1384794_at	---	---	uniq_RT1uvsDR_883	0	5.62	5.72	5.77	4.70	0	0.105	1.076	0.956	0	0.150	1.110	0.808	1	-0.914	-1.885	0.003
1384839_at	---	---	uniq_RT1uvsDR_883	0	8.03	8.45	8.41	8.75	0	0.418	1.337	0.308	0	0.377	1.299	0.345	1	0.723	1.650	0.099
1384871_at	Osbpl6	oxysterol binding protein-like 6	uniq_RT1uvsDR_883	0	4.87	4.83	4.95	4.35	0	-0.038	-1.027	1.071	0	0.088	1.063	0.976	1	-0.514	-1.428	0.096
1384975_at	Ermp1	endoplasmic reticulum metallopeptidase 1	uniq_RT1uvsDR_883	0	5.88	6.23	5.79	5.07	0	0.359	1.283	0.450	0	-0.090	-1.065	0.978	1	-0.801	-1.742	0.008
1385089_at	Grhl1	grainyhead-like 1 (Drosophila)	uniq_RT1uvsDR_883	0	5.62	6.14	5.92	6.40	0	0.518	1.432	0.190	0	0.303	1.234	0.443	1	0.781	1.719	0.085
1385098_at	---	---	uniq_RT1uvsDR_883	0	6.58	6.26	6.63	4.80	0	-0.318	-1.247	0.379	0	0.052	1.037	1.078	1	-1.772	-3.416	0.000
1385119_at	---	---	uniq_RT1uvsDR_883	0	5.15	4.71	5.21	4.35	0	-0.436	-1.353	0.177	0	0.058	1.041	0.915	1	-0.802	-1.744	0.008
1385127_at	Bmp5	bone morphogenetic protein 5	uniq_RT1uvsDR_883	0	4.71	4.52	4.83	4.08	0	-0.197	-1.147	0.811	0	0.121	1.087	0.787	1	-0.636	-1.554	0.034
1385138_at	---	---	uniq_RT1uvsDR_883	0	7.56	7.75	7.79	8.48	0	0.189	1.140	0.837	0	0.230	1.173	0.687	1	0.913	1.883	0.040
1385157_at	---	---	uniq_RT1uvsDR_883	0	6.55	6.55	6.72	5.59	0	0.000	-1.000	1.165	0	0.176	1.130	0.757	1	-0.958	-1.943	0.002
1385205_at	---	---	uniq_RT1uvsDR_883	0	5.99	6.24	6.35	4.76	0	0.256	1.194	0.673	0	0.365	1.288	0.366	1	-1.227	-2.342	0.000
1385215_at	Runx1t1	runt-related transcription factor 1; translocated to,	uniq_RT1uvsDR_883	0	4.08	4.29	3.91	4.90	0	0.204	1.152	0.811	0	-0.167	-1.123	0.766	1	0.820	1.765	0.061
1385314_at	---	---	uniq_RT1uvsDR_883	0	8.34	8.86	8.31	7.73	0	0.519	1.433	0.192	0	-0.026	-1.018	1.041	1	-0.607	-1.523	0.044
1385321_at	Prmt8	protein arginine methyltransferase 8	uniq_RT1uvsDR_883	0	5.49	5.57	5.69	4.87	0	0.083	1.059	1.012	0	0.203	1.151	0.665	1	-0.617	-1.534	0.043
1385333_at	Aatk	apoptosis-associated tyrosine kinase	uniq_RT1uvsDR_883	0	4.53	4.38	4.55	3.97	0	-0.152	-1.111	0.834	0	0.016	1.011	1.135	1	-0.559	-1.473	0.064

Affymetrix Probeset ID	Gene Symbol	Gene Title	Figure 7 A Venn n=2289	Present in any 2 analyses n=423 1=yes 0=no	Mean log2 intensity DR+/+ ND	Mean log2 intensity DR+/+ HCD	Mean log2 intensity DR+/+ B/S	Mean log2 intensity F+/+ day 40 ND	DR+/+HCD vs DR+/+ND FDR<10% Log2 ratio >0.5 n=636	Log2 ratio DR+/+HCD vs DR+/+ ND	Fold-Change DR+/+HCD vs DR+/+ ND	FDR DR+/+HCD vs DR+/+ ND	DR+/+B/S vs DR+/+ND Log2 ratio >0.5 n=350	Log2 ratio DR+/+ B/S vs DR+/+ ND	Fold-Change DR+/+B/S vs DR+/+ ND	FDR DR+/+B/S vs DR+/+ ND	F+/+ vs DR+/+ND Log2 ratio >0.5 n=1209	Log2 ratio F+/+ vs DR+/+ ND	Fold-Change F+/+ vs DR+/+ ND	FDR F+/+ vs DR+/+ ND
1385343_at	Adam22	ADAM metallopeptidase domain 22	uniq_RT1uvsDR_883	0	4.16	4.52	4.33	5.25	0	0.353	1.277	0.422	0	0.167	1.123	0.812	1	1.085	2.121	0.023
1385350_at	---	---	uniq_RT1uvsDR_883	0	7.55	8.17	7.72	8.81	0	0.625	1.542	0.108	0	0.172	1.126	0.604	1	1.262	2.398	0.013
1385362_at	---	---	uniq_RT1uvsDR_883	0	4.65	4.27	4.34	4.12	0	-0.387	-1.308	0.300	0	-0.316	-1.245	0.424	1	-0.537	-1.451	0.090
1385393_at	---	---	uniq_RT1uvsDR_883	0	4.62	4.54	4.52	5.60	0	-0.087	-1.062	1.003	0	-0.101	-1.072	0.881	1	0.979	1.971	0.037
1385442_at	---	---	uniq_RT1uvsDR_883	0	7.30	6.83	7.11	8.24	0	-0.468	-1.383	0.138	0	-0.188	-1.139	0.756	1	0.943	1.923	0.038
1385637_at	---	---	uniq_RT1uvsDR_883	0	6.67	6.49	6.61	6.12	0	-0.181	-1.134	0.689	0	-0.061	-1.043	0.886	1	-0.551	-1.465	0.080
1385729_at	---	---	uniq_RT1uvsDR_883	0	3.38	3.04	3.21	4.14	0	-0.343	-1.268	0.417	0	-0.174	-1.128	0.801	1	0.757	1.690	0.090
1385835_at	Plxdc1	plexin domain containing 1	uniq_RT1uvsDR_883	0	6.30	6.00	6.13	5.74	0	-0.300	-1.231	0.482	0	-0.169	-1.125	0.781	1	-0.551	-1.465	0.068
1385871_at	Dhx36	DEAH (Asp-Glu-Ala-His) box polypeptide 36	uniq_RT1uvsDR_883	0	7.44	7.67	7.78	6.48	0	0.227	1.170	0.709	0	0.339	1.265	0.409	1	-0.956	-1.939	0.003
1385931_at	Hook3	hook homolog 3 (Drosophila)	uniq_RT1uvsDR_883	0	5.25	5.50	5.72	4.54	0	0.248	1.188	0.565	0	0.471	1.386	0.186	1	-0.704	-1.629	0.027
1385981_at	Lppr4	lipid phosphate phosphatase-related protein type 4	uniq_RT1uvsDR_883	0	6.98	7.13	6.77	8.04	0	0.150	1.110	0.921	0	-0.217	-1.163	0.653	1	1.059	2.084	0.025
1385994_at	Spo11	SPO11 meiotic protein covalently bound to DSB hom	uniq_RT1uvsDR_883	0	3.25	3.01	3.13	4.06	0	-0.243	-1.184	0.691	0	-0.123	-1.089	0.964	1	0.816	1.760	0.066
1386051_at	---	---	uniq_RT1uvsDR_883	0	4.12	4.27	4.10	4.96	0	0.145	1.106	0.882	0	-0.018	-1.013	1.060	1	0.833	1.781	0.066
1386061_at	---	---	uniq_RT1uvsDR_883	0	6.52	6.92	6.62	7.45	0	0.399	1.318	0.387	0	0.103	1.074	0.951	1	0.928	1.903	0.040
1386080_at	Hey1	hairy/enhancer-of-split related with YRPW motif 1	uniq_RT1uvsDR_883	0	7.02	6.89	6.75	6.20	0	-0.127	-1.092	0.933	0	-0.268	-1.204	0.508	1	-0.823	-1.769	0.007
1386327_at	Cerkl	ceramide kinase-like	uniq_RT1uvsDR_883	0	5.15	4.95	5.12	4.39	0	-0.196	-1.145	0.732	0	-0.034	-1.024	0.936	1	-0.762	-1.695	0.010
1386420_at	---	---	uniq_RT1uvsDR_883	0	8.08	7.98	8.06	7.42	0	-0.103	-1.074	1.049	0	-0.019	-1.013	1.141	1	-0.661	-1.581	0.025
1386621_at	---	---	uniq_RT1uvsDR_883	0	5.90	6.40	6.25	6.92	0	0.505	1.419	0.252	0	0.357	1.280	0.366	1	1.018	2.026	0.029
1386685_at	Sacs	spastic ataxia of Charlevoix-Saguenay (sacsin)	uniq_RT1uvsDR_883	0	5.98	6.29	6.00	6.81	0	0.302	1.233	0.542	0	0.021	1.014	1.129	1	0.825	1.772	0.061
1386728_at	---	---	uniq_RT1uvsDR_883	0	3.67	3.54	3.41	4.70	0	-0.125	-1.090	1.016	0	-0.259	-1.196	0.597	1	1.034	2.048	0.024
1386855_at	---	---	uniq_RT1uvsDR_883	0	5.55	5.72	5.74	4.48	0	0.176	1.130	0.832	0	0.195	1.144	0.672	1	-1.067	-2.095	0.001
1386908_at	Glrx	glutaredoxin (thioltransferase)	uniq_RT1uvsDR_883	0	9.12	9.10	9.33	9.83	0	-0.020	-1.014	1.157	0	0.208	1.155	0.750	1	0.717	1.644	0.100
1386909_a_at	LOC683062 //	similar to voltage-dependent anion channel 1 /// sir	uniq_RT1uvsDR_883	0	9.30	9.59	9.42	8.59	0	0.291	1.224	0.583	0	0.123	1.089	0.913	1	-0.712	-1.638	0.016
1386911_at	Atp1a2	ATPase, Na+/K+ transporting, alpha 2 polypeptide	uniq_RT1uvsDR_883	0	7.00	6.88	6.88	6.48	0	-0.120	-1.087	0.948	0	-0.123	-1.089	0.784	1	-0.516	-1.430	0.095
1386914_at	Gmpr	guanosine monophosphate reductase	uniq_RT1uvsDR_883	0	7.14	7.53	7.27	6.44	0	0.393	1.313	0.357	0	0.132	1.096	0.914	1	-0.700	-1.624	0.018
1386985_at	Gstm1	glutathione S-transferase mu 1	uniq_RT1uvsDR_883	0	6.79	6.87	6.87	6.14	0	0.086	1.061	1.085	0	0.080	1.057	0.912	1	-0.642	-1.561	0.041
1387023_at	Gstm7	glutathione S-transferase, mu 7	uniq_RT1uvsDR_883	0	6.90	6.60	6.96	7.73	0	-0.307	-1.237	0.453	0	0.055	1.039	1.087	1	0.825	1.772	0.060
1387028_a_at	Id1	inhibitor of DNA binding 1	uniq_RT1uvsDR_883	0	8.49	8.52	8.14	7.97	0	0.037	1.026	1.126	0	-0.344	-1.270	0.338	1	-0.519	-1.433	0.090
1387029_at	Cfh	complement factor H	uniq_RT1uvsDR_883	0	6.11	6.00	6.23	5.59	0	-0.112	-1.080	0.842	0	0.120	1.087	0.884	1	-0.524	-1.438	0.093
1387063_at	Ip6k2	inositol hexakisphosphate kinase 2	uniq_RT1uvsDR_883	0	6.89	7.21	7.00	7.82	0	0.311	1.241	0.553	0	0.109	1.079	0.962	1	0.925	1.898	0.040
1387074_at	Rgs2	regulator of G-protein signaling 2	uniq_RT1uvsDR_883	0	9.22	9.75	9.29	10.73	0	0.530	1.444	0.178	0	0.065	1.046	1.032	1	1.510	2.848	0.006
1387122_at	Plagl1	pleiomorphic adenoma gene-like 1	uniq_RT1uvsDR_883	0	6.71	6.83	6.88	7.70	0	0.123	1.089	0.940	0	0.164	1.120	0.764	1	0.985	1.980	0.034
1387154_at	LOC1009122	pro-neuropeptide Y-like /// neuropeptide Y	uniq_RT1uvsDR_883	0	10.11	10.38	10.19	11.02	0	0.275	1.210	0.597	0	0.087	1.062	1.052	1	0.910	1.879	0.040
1387169_at	Tle3	transducin-like enhancer of split 3 (E(sp1) homolog,	uniq_RT1uvsDR_883	0	8.79	8.99	9.04	8.28	0	0.204	1.152	0.805	0	0.246	1.186	0.610	1	-0.507	-1.421	0.095
1387323_at	Klkb1	kallikrein B, plasma 1	uniq_RT1uvsDR_883	0	3.22	3.18	3.40	5.95	0	-0.040	-1.028	1.145	0	0.177	1.131	0.811	1	2.728	6.624	0.000
1387366_at	Ilf3	interleukin enhancer binding factor 3	uniq_RT1uvsDR_883	0	6.85	6.92	6.90	7.82	0	0.071	1.050	1.115	0	0.050	1.035	1.057	1	0.965	1.952	0.032
1387395_at	Adora2b	adenosine A2B receptor	uniq_RT1uvsDR_883	0	6.70	6.93	7.09	5.74	0	0.226	1.169	0.709	0	0.388	1.309	0.322	1	-0.964	-1.951	0.003
1387471_at	Cela2a	chymotrypsin-like elastase family, member 2A	uniq_RT1uvsDR_883	0	12.10	12.47	11.77	12.96	0	0.367	1.290	0.414	0	-0.332	-1.259	0.351	1	0.858	1.813	0.053
1387476_at	Kcnd2	potassium voltage-gated channel, Shal-related subfa	uniq_RT1uvsDR_883	0	4.90	4.64	4.73	5.95	0	-0.261	-1.198	0.637	0	-0.174	-1.128	0.826	1	1.044	2.063	0.023
1387529_a_at	Tagln3	transgelin 3	uniq_RT1uvsDR_883	0	6.37	6.36	6.55	7.17	0	-0.017	-1.012	1.027	0	0.173	1.127	0.830	1	0.794	1.733	0.073
1387554_at	Galnt5	UDP-N-acetyl-alpha-D-galactosamine:polypeptide N	uniq_RT1uvsDR_883	0	7.44	7.02	7.27	6.71	0	-0.414	-1.333	0.222	0	-0.169	-1.124	0.791	1	-0.729	-1.658	0.013
1387646_a_at	Max	MYC associated factor X	uniq_RT1uvsDR_883	0	5.08	4.96	5.06	4.46	0	-0.120	-1.087	0.931	0	-0.014	-1.010	1.122	1	-0.613	-1.530	0.052
1387769_a_at	Id3	inhibitor of DNA binding 3	uniq_RT1uvsDR_883	0	8.10	7.96	7.83	7.49	0	-0.136	-1.099	0.945	0	-0.262	-1.199	0.519	1	-0.608	-1.525	0.039
1387811_at	Agt	angiotensinogen (serpin peptidase inhibitor, clade A	uniq_RT1uvsDR_883	0	6.91	6.82	6.80	6.30	0	-0.089	-1.064	1.020	0	-0.110	-1.079	0.931	1	-0.607	-1.523	0.047
1387819_at	Cela1	chymotrypsin-like elastase family, member 1	uniq_RT1uvsDR_883	0	11.85	11.86	11.47	12.68	0	0.009	1.006	1.121	0	-0.372	-1.294	0.276	1	0.836	1.785	0.061
1387883_a_at	Tmsb4x	thymosin beta 4, X-linked	uniq_RT1uvsDR_883	0	12.59	12.52	12.48	12.02	0	-0.069	-1.049	1.057	0	-0.107	-1.077	0.944	1	-0.564	-1.478	0.055
1387906_a_at	Gnas	GNAS complex locus	uniq_RT1uvsDR_883	0	10.53	10.93	10.94	11.55	0	0.401	1.320	0.352	0	0.405	1.324	0.294	1	1.013	2.018	0.025
1387922_at	Crispld2	cysteine-rich secretory protein LCCL domain contain	uniq_RT1uvsDR_883	0	6.31	6.07	5.91	5.40	0	-0.244	-1.184	0.629	0	-0.395	-1.315	0.246	1	-0.913	-1.883	0.003
1387947_at	Mafb	v-maf musculoaponeurotic fibrosarcoma oncogene	uniq_RT1uvsDR_883	0	9.09	9.34	9.44	8.45	0	0.251	1.190	0.668	0	0.350	1.274	0.376	1	-0.632	-1.549	0.030
1388006_at	Muc13	mucin 13, cell surface associated	uniq_RT1uvsDR_883	0	5.89	5.94	6.06	5.07	0	0.041	1.029	1.112	0	0.162	1.119	0.810	1	-0.821	-1.766	0.007
1388127_at	Cyp4v3	cytochrome P450, family 4, subfamily v, polypeptide	uniq_RT1uvsDR_883	0	8.66	8.88	8.90	5.73	0	0.222	1.166	0.718	0	0.234	1.176	0.679	1	-2.934	-7.641	0.000
1388143_at	Col18a1	collagen, type XVIII, alpha 1	uniq_RT1uvsDR_883	0	6.45	6.17	6.24	5.67	0	-0.276	-1.211	0.562	0	-0.207	-1.154	0.728	1	-0.774	-1.710	0.009
1388155_at	Krt18	keratin 18	uniq_RT1uvsDR_883	0	10.76	10.94	10.76	10.02	0	0.176	1.130	0.839	0	-0.003	-1.002	1.135	1	-0.745	-1.676	0.012
1388182_at	Prim1	DNA primase, p49 subunit	uniq_RT1uvsDR_883	0	7.81	8.18	8.04	6.75	0	0.371	1.293	0.426	0	0.236	1.177	0.666	1	-1.055	-2.077	0.001
1388190_at	Apob	apolipoprotein B	uniq_RT1uvsDR_883	0	5.02	4.70	4.85	6.46	0	-0.324	-1.252	0.436	0	-0.170	-1.125	0.833	1	1.437	2.708	0.007
1388312_at	---	---	uniq_RT1uvsDR_883	0	7.08	6.76	6.72	6.35	0	-0.322	-1.250	0.415	0	-0.362	-1.285	0.299	1	-0.734	-1.664	0.012
1388363_at	Raly	RNA binding protein, autoantigenic (hnRNP-associat	uniq_RT1uvsDR_883	0	8.50	8.66	8.72	7.72	0	0.160	1.117	0.927	0	0.226	1.170	0.689	1	-0.776	-1.713	0.009
1388366_at	Mrpl4	mitochondrial ribosomal protein L4	uniq_RT1uvsDR_883	0	8.11	8.30	8.20	7.33	0	0.189	1.140	0.846	0	0.090	1.064	1.059	1	-0.782	-1.719	0.009
1388459_at	Col18a1	collagen, type XVIII, alpha 1	uniq_RT1uvsDR_883	0	6.79	6.36	6.33	6.04	0	-0.430	-1.348	0.208	0	-0.464	-1.380	0.109	1	-0.754	-1.687	0.011
1388494_at	Col4a2	collagen, type IV, alpha 2	uniq_RT1uvsDR_883	0	7.97	7.72	7.79	7.42	0	-0.250	-1.189	0.510	0	-0.185	-1.136	0.664	1	-0.555	-1.469	0.065
1388506_at	Dsp	desmoplakin	uniq_RT1uvsDR_883	0	9.64	9.68	9.61	8.25	0	0.045	1.032	1.151	0	-0.029	-1.020	1.105	1	-1.391	-2.623	0.000
1388527_at	---	---	uniq_RT1uvsDR_883	0	5.83	5.38	5.49	5.27	0	-0.457	-1.373	0.154	0	-0.348	-1.273	0.339	1	-0.567	-1.481	0.060
1388557_at	C7 /// Tubb4l	complement component 7 /// tubulin, beta 4B class	uniq_RT1uvsDR_883	0	8.00	7.67	7.63	6.73	0	-0.335	-1.261	0.326	0	-0.375	-1.296	0.258	1	-1.270	-2.411	0.000
1388578_at	Tatdn3	TatD DNase domain containing 3	uniq_RT1uvsDR_883	0	7.81	7.95	7.98	7.30	0	0.139	1.101	0.964	0	0.161	1.118	0.886	1	-0.511	-1.426	0.090
1388598_at	LOC691995	hypothetical protein LOC691995	uniq_RT1uvsDR_883	0	4.56	4.10	4.18	3.91	0	-0.453	-1.369	0.166	0	-0.373	-1.295	0.268	1	-0.649	-1.568	0.032
1388603_a_at	Isca1	iron-sulfur cluster assembly 1 homolog (S. cerevisiae	uniq_RT1uvsDR_883	0	8.97	8.99	9.08	9.85	0	0.022	1.016	1.166	0	0.112	1.080	0.995	1	0.886	1.848	0.042
1388613_at	Isca1	iron-sulfur cluster assembly 1 homolog (S. cerevisiae	uniq_RT1uvsDR_883	0	6.41	6.50	6.47	8.04	0	0.088	1.063	1.032	0	0.063	1.044	1.066	1	1.632	3.098	0.005
1388617_at	Bphl	biphenyl hydrolase-like (serine hydrolase)	uniq_RT1uvsDR_883	0	7.81	8.11	8.02	7.19	0	0.306	1.236	0.551	0	0.208	1.155	0.749	1	-0.615	-1.532	0.036
1388674_at	Cdkn1a	cyclin-dependent kinase inhibitor 1A	uniq_RT1uvsDR_883	0	7.31	7.65	7.30	8.07	0	0.337	1.264	0.455	0	-0.009	-1.006	1.137	1	0.758	1.691	0.083
1388718_at	Tmod1	tropomodulin 1	uniq_RT1uvsDR_883	0	8.30	8.49	8.39	7.75	0	0.195	1.145	0.780	0	0.093	1.067	1.037	1	-0.544	-1.458	0.078
1388773_at	Tnfaip2	tumor necrosis factor, alpha-induced protein 2	uniq_RT1uvsDR_883	0	5.74	5.56	5.42	5.20	0	-0.186	-1.138	0.794	0	-0.317	-1.246	0.426	1	-0.545	-1.460	0.069
1388845_at	---	---	uniq_RT1uvsDR_883	0	6.73	6.76	6.85	6.11	0	0.026	1.018	1.152	0	0.116	1.083	0.933	1	-0.616	-1.533	0.035
1388902_at	Loxl1	lysyl oxidase-like 1	uniq_RT1uvsDR_883	0	7.51	7.33	7.58	6.88	0	-0.179	-1.132	0.696	0	0.067	1.047	1.003	1	-0.634	-1.552	0.034
1388921_at	---	---	uniq_RT1uvsDR_883	0	7.86	8.33	8.30	6.25	0	0.469	1.385	0.252	0	0.439	1.356	0.250	1	-1.608	-3.048	0.000
1388948_at	Stard10	StAR-related lipid transfer (START) domain containir	uniq_RT1uvsDR_883	0	9.13	9.34	9.31	8.62	0	0.211	1.157	0.796	0	0.176	1.130	0.836	1	-0.512	-1.426	0.089
1388966_at	---	---	uniq_RT1uvsDR_883	0	6.39	5.94	6.24	5.74	0	-0.452	-1.368	0.160	0	-0.154	-1.113	0.847	1	-0.650	-1.569	0.027
1389095_at	Boc	BOC cell adhesion associated, oncogene regulated	uniq_RT1uvsDR_883	0	5.19	4.79	4.94	4.64	0	-0.399	-1.319	0.253	0	-0.255	-1.193	0.572	1	-0.552	-1.466	0.070
1389112_at	Nkd1	naked cuticle homolog 1 (Drosophila)	uniq_RT1uvsDR_883	0	4.81	4.36	4.48	4.20	0	-0.450	-1.366	0.144	0	-0.336	-1.262	0.332	1	-0.617	-1.534	0.037
1389115_at	Evpl	envoplakin	uniq_RT1uvsDR_883	0	6.24	6.15	6.15	7.06	0	-0.087	-1.062	1.089	0	-0.089	-1.064	1.030	1	0.819	1.765	0.066
1389137_at	Cit	citron (rho-interacting, serine/threonine kinase 21)	uniq_RT1uvsDR_883	0	6.40	6.53	6.39	7.64	0	0.132	1.096	0.952	0	-0.006	-1.004	1.129	1	1.241	2.364	0.012
1389221_at	Mmd2	monocyte to macrophage differentiation-associatec	uniq_RT1uvsDR_883	0	6.84	6.51	6.91	5.89	0	-0.327	-1.254	0.413	0	0.068	1.048	1.078	1	-0.955	-1.939	0.003
1389244_x_at	Cxcr4	chemokine (C-X-C motif) receptor 4	uniq_RT1uvsDR_883	0	6.97	6.86	6.97	6.35	0	-0.109	-1.078	0.955	0	0.009	1.007	1.149	1	-0.619	-1.536	0.036
1389341_at	Plxnd1	plexin D1	uniq_RT1uvsDR_883	0	7.71	7.24	7.24	7.10	0	-0.469	-1.384	0.127	0	-0.471	-1.386	0.093	1	-0.611	-1.528	0.035
1389374_at	Kifc3	kinesin family member C3	uniq_RT1uvsDR_883	0	7.54	7.61	7.47	7.03	0	0.069	1.049	1.112	0	-0.068	-1.049	1.018	1	-0.512	-1.426	0.095
1389394_at	---	---	uniq_RT1uvsDR_883	0	9.55	9.18	9.28	8.96	0	-0.373	-1.295	0.303	0	-0.269	-1.205	0.505	1	-0.586	-1.502	0.050
1389444_at	Zdhhc20	zinc finger, DHHC-type containing 20	uniq_RT1uvsDR_883	0	8.50	8.63	8.76	9.28	0	0.130	1.095	0.962	0	0.263	1.200	0.576	1	0.780	1.717	0.079
1389448_at	Sept4	septin 4	uniq_RT1uvsDR_883	0	7.68	7.42	7.55	7.16	0	-0.252	-1.191	0.603	0	-0.130	-1.094	0.869	1	-0.511	-1.425	0.093
1389474_at	Mylip	myosin regulatory light chain interacting protein	uniq_RT1uvsDR_883	0	7.78	8.23	8.21	7.19	0	0.448	1.364	0.299	0	0.429	1.346	0.250	1	-0.591	-1.506	0.053
1389514_at	Lingo1	leucine rich repeat and Ig domain containing 1	uniq_RT1uvsDR_883	0	8.54	8.40	8.71	8.02	0	-0.139	-1.101	0.958	0	0.175	1.129	0.820	1	-0.517	-1.431	0.084
1389554_at	---	---	uniq_RT1uvsDR_883	0	7.32	6.99	7.14	6.79	0	-0.334	-1.260	0.329	0	-0.183	-1.135	0.735	1	-0.536	-1.450	0.081
1389600_at	LOC360998 /	hypothetical LOC360998 /// hypothetical protein LC	uniq_RT1uvsDR_883	0	6.40	6.32	6.33	7.61	0	-0.080	-1.057	1.036	0	-0.077	-1.055	0.961	1	1.209	2.312	0.013
1389640_at	Sh3bgrl2	SH3 domain binding glutamic acid-rich protein like 2	uniq_RT1uvsDR_883	0	9.54	9.74	9.66	8.89	0	0.197	1.146	0.824	0	0.118	1.086	0.968	1	-0.654	-1.574	0.026
1389650_at	---	---	uniq_RT1uvsDR_883	0	7.36	7.33	7.51	6.56	0	-0.023	-1.016	1.079	0	0.151	1.110	0.796	1	-0.798	-1.738	0.008
1389748_at	---	---	uniq_RT1uvsDR_883	0	5.54	5.64	5.71	6.69	0	0.103	1.074	0.943	0	0.173	1.128	0.704	1	1.151	2.221	0.018
1389774_at	Zfp612	zinc finger protein 612	uniq_RT1uvsDR_883	0	8.43	8.59	8.67	9.30	0	0.161	1.118	0.929	0	0.240	1.181	0.668	1	0.874	1.833	0.046
1389793_at	---	---	uniq_RT1uvsDR_883	0	6.83	6.88	7.03	6.07	0	0.055	1.039	1.124	0	0.202	1.151	0.750	1	-0.755	-1.688	0.013
1389806_at	---	---	uniq_RT1uvsDR_883	0	6.24	6.34	6.31	7.21	0	0.098	1.070	1.038	0	0.071	1.051	1.087	1	0.974	1.965	0.031
1389811_at	Rasgef1c	RasGEF domain family, member 1C	uniq_RT1uvsDR_883	0	5.98	5.81	6.26	4.89	0	-0.165	-1.121	0.773	0	0.280	1.215	0.551	1	-1.089	-2.128	0.001
1389882_at	Ier5	immediate early response 5	uniq_RT1uvsDR_883	0	5.71	5.81	5.75	5.09	0	0.107	1.077	0.928	0	0.045	1.032	1.066	1	-0.616	-1.533	0.036
1389986_at	LOC1009124	uncharacterized LOC100912411	uniq_RT1uvsDR_883	0	12.12	12.01	12.07	11.55	0	-0.101	-1.073	0.751	0	-0.048	-1.034	0.962	1	-0.570	-1.484	0.063
1390024_at	---	---	uniq_RT1uvsDR_883	0	9.25	8.99	9.37	8.48	0	-0.268	-1.204	0.509	0	0.117	1.084	0.995	1	-0.769	-1.705	0.010
1390063_at	Mfap3	microfibrillar-associated protein 3	uniq_RT1uvsDR_883	0	6.99	7.41	7.41	8.57	0	0.419	1.337	0.343	0	0.421	1.339	0.250	1	1.577	2.983	0.005
1390107_at	LOC1009118	synaptotagmin-like protein 2-like /// synaptotagmir	uniq_RT1uvsDR_883	0	6.50	6.52	6.40	5.87	0	0.018	1.013	1.172	0	-0.103	-1.074	0.972	1	-0.631	-1.549	0.034
1390116_at	LOC1009097	uncharacterized LOC100909788	uniq_RT1uvsDR_883	0	6.62	6.25	6.35	5.99	0	-0.369	-1.291	0.256	0	-0.275	-1.210	0.512	1	-0.629	-1.547	0.036
1390159_at	Rasgrp3	RAS guanyl releasing protein 3 (calcium and DAG-reg	uniq_RT1uvsDR_883	0	8.23	8.41	8.50	7.67	0	0.182	1.135	0.819	0	0.264	1.201	0.574	1	-0.565	-1.480	0.060
1390174_at	Eml1	echinoderm microtubule associated protein like 1	uniq_RT1uvsDR_883	0	5.46	5.54	5.46	7.05	0	0.082	1.058	1.072	0	0.006	1.004	1.127	1	1.595	3.022	0.006
1390191_at	Hunk	hormonally upregulated Neu-associated kinase	uniq_RT1uvsDR_883	0	6.96	7.11	7.14	5.61	0	0.146	1.106	0.963	0	0.180	1.133	0.802	1	-1.353	-2.554	0.000

Affymetrix Probeset ID	Gene Symbol	Gene Title	Figure 7 A Venn n=2289	Present in any 2 analyses n=423 1=yes 0=no	Mean log2 intensity DR+/+ ND	Mean log2 intensity DR+/+ HCD	Mean log2 intensity DR+/+ B/S	Mean log2 intensity F+/+ day 40 ND	DR+/+HCD vs DR+/+ND FDR<10% Log2 ratio >0.5 n=636	Log2 ratio DR+/+HCD vs DR+/+ ND	Fold-Change DR+/+HCD vs DR+/+ ND	FDR DR+/+HCD vs DR+/+ ND	DR+/+B/S vs DR+/+ND Log2 ratio >0.5 n=350	Log2 ratio DR+/+ B/S vs DR+/+ ND	Fold-Change DR+/+B/S vs DR+/+ ND	FDR DR+/+B/S vs DR+/+ ND	F+/+ vs DR+/+ND Log2 ratio >0.5 n=1209	Log2 ratio F+/+ vs DR+/+ ND	Fold-Change F+/+ vs DR+/+ ND	FDR F+/+ vs DR+/+ ND
1390270_at	---	---	uniq_RT1uvsDR_883	0	5.56	5.39	5.54	5.00	0	-0.169	-1.124	0.806	0	-0.017	-1.012	1.143	1	-0.560	-1.474	0.057
1390284_at	Ccdc77	coiled-coil domain containing 77	uniq_RT1uvsDR_883	0	7.75	7.92	7.79	6.69	0	0.161	1.118	0.904	0	0.040	1.028	1.042	1	-1.060	-2.085	0.001
1390300_at	---	---	uniq_RT1uvsDR_883	0	7.07	7.19	7.38	6.44	0	0.118	1.086	1.015	0	0.301	1.232	0.484	1	-0.631	-1.549	0.043
1390406_at	Arhgap18	Rho GTPase activating protein 18	uniq_RT1uvsDR_883	0	8.42	8.51	8.49	7.89	0	0.088	1.063	1.040	0	0.067	1.048	1.086	1	-0.531	-1.445	0.079
1390428_at	---	---	uniq_RT1uvsDR_883	0	6.57	6.65	6.80	6.04	0	0.076	1.054	1.112	0	0.225	1.169	0.669	1	-0.532	-1.445	0.080
1390437_at	Sema5a	sema domain, seven thrombospondin repeats (type	uniq_RT1uvsDR_883	0	8.21	8.17	8.32	7.60	0	-0.047	-1.033	1.121	0	0.108	1.078	1.021	1	-0.609	-1.526	0.036
1390450_a_at	LOC10091085	mimecan-like /// osteoglycin	uniq_RT1uvsDR_883	0	5.59	5.32	5.30	4.96	0	-0.271	-1.206	0.461	0	-0.291	-1.224	0.363	1	-0.635	-1.553	0.046
1390496_at	LOC10091053	protein phosphatase 1 regulatory subunit 29-like	uniq_RT1uvsDR_883	0	5.35	5.01	5.05	4.78	0	-0.342	-1.267	0.411	0	-0.303	-1.234	0.454	1	-0.579	-1.493	0.075
1390530_at	---	---	uniq_RT1uvsDR_883	0	9.27	9.39	9.37	8.71	0	0.124	1.089	0.973	0	0.106	1.077	0.974	1	-0.560	-1.474	0.073
1390561_at	---	---	uniq_RT1uvsDR_883	0	7.37	7.32	7.38	6.27	0	-0.053	-1.037	1.134	0	0.009	1.006	1.141	1	-1.098	-2.140	0.001
1390645_at	---	---	uniq_RT1uvsDR_883	0	6.55	6.52	6.45	7.36	0	-0.024	-1.016	0.967	0	-0.095	-1.068	0.915	1	0.817	1.761	0.059
1390739_at	Zfp609	zinc finger protein 609	uniq_RT1uvsDR_883	0	6.81	6.77	6.80	7.56	0	-0.037	-1.026	0.947	0	-0.004	-1.002	0.934	1	0.755	1.688	0.091
1390751_at	---	---	uniq_RT1uvsDR_883	0	5.16	4.80	4.85	4.59	0	-0.360	-1.283	0.348	0	-0.307	-1.237	0.442	1	-0.562	-1.477	0.063
1390781_at	Abcb10	ATP-binding cassette, subfamily B (MDR/TAP), mem	uniq_RT1uvsDR_883	0	7.42	7.45	7.55	6.85	0	0.027	1.019	1.170	0	0.126	1.091	0.969	1	-0.577	-1.492	0.048
1390783_at	Abca8a	ATP-binding cassette, subfamily A (ABC1), member	uniq_RT1uvsDR_883	0	7.80	7.80	7.82	7.25	0	-0.002	-1.002	1.153	0	0.023	1.016	1.089	1	-0.550	-1.465	0.066
1390798_at	Ptprc	protein tyrosine phosphatase, receptor type, C	uniq_RT1uvsDR_883	0	5.78	5.84	5.75	5.01	0	0.053	1.037	0.986	0	-0.037	-1.026	0.937	1	-0.774	-1.709	0.016
1390813_at	Msi2	musashi RNA-binding protein 2	uniq_RT1uvsDR_883	0	4.96	5.58	5.25	6.89	0	0.619	1.536	0.131	0	0.296	1.227	0.427	1	1.935	3.825	0.004
1390828_at	Npy1r	neuropeptide Y receptor Y1	uniq_RT1uvsDR_883	0	7.07	7.33	7.20	6.14	0	0.255	1.193	0.621	0	0.132	1.096	0.909	1	-0.934	-1.911	0.003
1390837_at	---	---	uniq_RT1uvsDR_883	0	7.23	7.47	7.60	6.67	0	0.241	1.181	0.679	0	0.361	1.285	0.377	1	-0.563	-1.478	0.057
1390912_at	Pcp4l1	Purkinje cell protein 4-like 1	uniq_RT1uvsDR_883	0	4.81	4.50	4.55	4.24	0	-0.312	-1.241	0.496	0	-0.264	-1.201	0.558	1	-0.575	-1.490	0.050
1390937_at	Ppp1r36	protein phosphatase 1, regulatory subunit 36	uniq_RT1uvsDR_883	0	7.26	7.39	7.28	6.40	0	0.130	1.094	0.920	0	0.027	1.019	1.124	1	-0.853	-1.807	0.006
1390943_at	RGD1359529	similar to chromosome 1 open reading frame 63	uniq_RT1uvsDR_883	0	8.68	8.85	8.61	7.85	0	0.170	1.125	0.906	0	-0.073	-1.052	0.943	1	-0.832	-1.781	0.006
1390960_at	Azi2	5-azacytidine induced 2	uniq_RT1uvsDR_883	0	7.05	7.15	7.09	6.49	0	0.093	1.067	1.071	0	0.036	1.026	1.128	1	-0.562	-1.477	0.063
1390979_at	LOC10091023	zinc finger protein 82-like /// zinc finger protein 14 h	uniq_RT1uvsDR_883	0	4.65	4.76	4.63	6.81	0	0.106	1.076	1.010	0	-0.018	-1.013	1.151	1	2.158	4.463	0.001
1391016_at	Wars2	tryptophanyl tRNA synthetase 2 (mitochondrial)	uniq_RT1uvsDR_883	0	5.63	5.64	5.58	6.45	0	0.007	1.005	1.168	0	-0.052	-1.037	1.054	1	0.814	1.758	0.069
1391025_at	---	---	uniq_RT1uvsDR_883	0	6.48	7.10	6.99	7.53	0	0.629	1.547	0.119	0	0.515	1.429	0.147	1	1.056	2.080	0.025
1391090_at	---	---	uniq_RT1uvsDR_883	0	5.42	5.32	5.54	4.76	0	-0.106	-1.076	1.039	0	0.114	1.083	0.976	1	-0.665	-1.586	0.024
1391134_at	Tmco3	transmembrane and coiled-coil domains 3	uniq_RT1uvsDR_883	0	6.80	6.76	6.94	6.25	0	-0.037	-1.026	1.141	0	0.145	1.106	0.881	1	-0.549	-1.463	0.066
1391143_at	---	---	uniq_RT1uvsDR_883	0	6.55	6.62	6.58	5.90	0	0.075	1.054	1.110	0	0.034	1.024	1.118	1	-0.650	-1.570	0.041
1391165_at	Sost	sclerostin	uniq_RT1uvsDR_883	0	4.34	4.00	4.02	3.73	0	-0.342	-1.267	0.397	0	-0.314	-1.243	0.417	1	-0.607	-1.524	0.041
1391170_at	Setd5	SET domain containing 5	uniq_RT1uvsDR_883	0	7.90	8.13	7.85	8.94	0	0.235	1.177	0.638	0	-0.045	-1.032	1.033	1	1.046	2.064	0.025
1391202_at	---	---	uniq_RT1uvsDR_883	0	5.59	5.71	5.75	7.13	0	0.123	1.089	1.020	0	0.161	1.118	0.847	1	1.535	2.897	0.006
1391207_at	Fam19a5	family with sequence similarity 19 (chemokine (C-C	uniq_RT1uvsDR_883	0	7.93	7.76	7.99	7.06	0	-0.168	-1.124	0.875	0	0.062	1.044	1.100	1	-0.869	-1.826	0.005
1391238_at	---	---	uniq_RT1uvsDR_883	0	4.49	4.28	4.57	5.35	0	-0.210	-1.157	0.649	0	0.078	1.056	1.018	1	0.859	1.813	0.052
1391243_at	---	---	uniq_RT1uvsDR_883	0	4.01	3.69	3.83	3.47	0	-0.329	-1.256	0.415	0	-0.189	-1.140	0.761	1	-0.547	-1.461	0.063
1391262_at	LOC690251 /,	similar to SUMO/sentrin specific protease 5 /// simil	uniq_RT1uvsDR_883	0	4.80	4.86	4.95	8.31	0	0.060	1.043	1.095	0	0.154	1.113	0.839	1	3.507	11.367	0.000
1391334_at	---	---	uniq_RT1uvsDR_883	0	6.30	6.23	6.58	7.35	0	-0.068	-1.049	0.965	0	0.275	1.210	0.545	1	1.050	2.071	0.024
1391341_at	Plxdc1	plexin domain containing 1	uniq_RT1uvsDR_883	0	8.05	7.91	8.29	7.38	0	-0.139	-1.101	0.936	0	0.243	1.183	0.657	1	-0.671	-1.592	0.025
1391406_at	---	---	uniq_RT1uvsDR_883	0	8.59	8.36	8.55	8.02	0	-0.236	-1.177	0.671	0	-0.049	-1.034	1.063	1	-0.572	-1.486	0.052
1391408_a_at	Mplkip	M-phase specific PLK1 interacting protein	uniq_RT1uvsDR_883	0	8.58	8.28	8.30	8.04	0	-0.300	-1.231	0.524	0	-0.283	-1.217	0.522	1	-0.537	-1.451	0.071
1391446_at	Ms4a1	membrane-spanning 4-domains, subfamily A, meml	uniq_RT1uvsDR_883	0	5.10	4.61	4.75	4.27	0	-0.495	-1.409	0.096	0	-0.348	-1.273	0.295	1	-0.828	-1.775	0.009
1391447_at	---	---	uniq_RT1uvsDR_883	0	5.43	5.54	5.61	6.18	0	0.106	1.076	1.047	0	0.179	1.132	0.772	1	0.752	1.685	0.085
1391476_at	---	---	uniq_RT1uvsDR_883	0	5.88	6.35	6.23	6.71	0	0.469	1.385	0.229	0	0.348	1.273	0.385	1	0.831	1.779	0.061
1391489_at	Irgm	immunity-related GTPase family, M	uniq_RT1uvsDR_883	0	6.51	6.59	6.75	5.89	0	0.081	1.058	1.054	0	0.238	1.179	0.615	1	-0.626	-1.543	0.036
1391491_a_at	Rad23b	RAD23 homolog B (S. cerevisiae)	uniq_RT1uvsDR_883	0	4.87	5.08	5.16	4.34	0	0.217	1.162	0.745	0	0.295	1.227	0.450	1	-0.522	-1.436	0.085
1391512_at	---	---	uniq_RT1uvsDR_883	0	3.70	3.41	3.78	4.99	0	-0.286	-1.219	0.531	0	0.079	1.056	0.989	1	1.294	2.453	0.012
1391524_at	Phip	pleckstrin homology domain interacting protein	uniq_RT1uvsDR_883	0	8.87	9.11	9.01	8.21	0	0.244	1.184	0.709	0	0.139	1.101	0.909	1	-0.660	-1.581	0.025
1391564_at	Zfp846	zinc finger protein 846	uniq_RT1uvsDR_883	0	6.13	6.31	6.18	5.50	0	0.177	1.130	0.830	0	0.048	1.034	1.082	1	-0.624	-1.541	0.033
1391589_at	---	---	uniq_RT1uvsDR_883	0	6.88	7.02	7.08	6.04	0	0.136	1.099	0.999	0	0.198	1.147	0.781	1	-0.846	-1.797	0.006
1391624_at	---	---	uniq_RT1uvsDR_883	0	5.22	4.72	4.86	4.71	0	-0.500	-1.414	0.083	0	-0.362	-1.285	0.267	1	-0.510	-1.424	0.098
1391625_at	Wasl	Wiskott-Aldrich syndrome-like	uniq_RT1uvsDR_883	0	8.64	8.40	8.58	9.87	0	-0.246	-1.186	0.389	0	-0.061	-1.043	0.806	1	1.222	2.333	0.015
1391635_at	Ctdspl	CTD (carboxy-terminal domain, RNA polymerase II, p	uniq_RT1uvsDR_883	0	7.82	7.89	7.95	6.39	0	0.064	1.045	1.129	0	0.130	1.094	0.956	1	-1.435	-2.705	0.000
1391643_at	---	---	uniq_RT1uvsDR_883	0	8.40	8.53	8.51	9.39	0	0.126	1.091	0.972	0	0.108	1.078	0.904	1	0.990	1.986	0.031
1391757_at	Ptpn4	protein tyrosine phosphatase, non-receptor type 4	uniq_RT1uvsDR_883	0	4.24	4.64	4.08	6.03	0	0.398	1.318	0.379	0	-0.163	-1.119	0.736	1	1.792	3.462	0.005
1391776_at	Cmc1	COX assembly mitochondrial protein homolog (S. ce	uniq_RT1uvsDR_883	0	8.48	8.74	8.78	7.73	0	0.260	1.197	0.629	0	0.299	1.230	0.497	1	-0.746	-1.677	0.011
1391822_at	---	---	uniq_RT1uvsDR_883	0	6.01	5.97	6.11	7.43	0	-0.043	-1.030	1.102	0	0.097	1.070	0.840	1	1.421	2.678	0.007
1391841_at	---	---	uniq_RT1uvsDR_883	0	7.11	6.78	6.74	6.29	0	-0.332	-1.259	0.396	0	-0.368	-1.290	0.276	1	-0.819	-1.764	0.007
1391856_at	Sema3g	sema domain, immunoglobulin domain (Ig), short ba	uniq_RT1uvsDR_883	0	6.45	6.36	6.37	5.82	0	-0.088	-1.063	1.051	0	-0.083	-1.059	1.003	1	-0.632	-1.549	0.033
1391857_at	Mslnl	mesothelin-like	uniq_RT1uvsDR_883	0	7.89	8.28	8.20	7.19	0	0.395	1.315	0.363	0	0.307	1.237	0.476	1	-0.696	-1.620	0.024
1391882_at	Gata2	GATA binding protein 2	uniq_RT1uvsDR_883	0	4.75	4.43	4.50	4.13	0	-0.324	-1.252	0.440	0	-0.249	-1.188	0.602	1	-0.624	-1.541	0.032
1391899_at	Purb	purine rich element binding protein B	uniq_RT1uvsDR_883	0	4.74	4.98	4.83	5.52	0	0.236	1.178	0.683	0	0.088	1.063	0.935	1	0.777	1.714	0.087
1391906_at	Apitd1 /// Co	apoptosis-inducing, TAF9-like domain 1 /// cortistat	uniq_RT1uvsDR_883	0	7.08	6.81	6.91	6.30	0	-0.273	-1.208	0.576	0	-0.167	-1.123	0.854	1	-0.783	-1.721	0.008
1391915_at	Hspa9 /// LO(	heat shock protein 9 /// stress-70 protein, mitochor	uniq_RT1uvsDR_883	0	7.15	7.60	7.46	4.41	0	0.443	1.359	0.295	0	0.302	1.233	0.476	1	-2.747	-6.713	0.000
1391941_at	LOC10091298	uncharacterized LOC100912988	uniq_RT1uvsDR_883	0	5.74	5.76	5.71	5.21	0	0.020	1.014	1.164	0	-0.028	-1.019	1.068	1	-0.528	-1.442	0.093
1391951_at	---	---	uniq_RT1uvsDR_883	0	6.02	6.33	6.21	6.77	0	0.302	1.233	0.540	0	0.183	1.135	0.748	1	0.746	1.677	0.090
1391993_at	Large	like-glycosyltransferase	uniq_RT1uvsDR_883	0	3.62	3.62	3.92	4.43	0	0.003	1.002	1.175	0	0.308	1.238	0.470	1	0.815	1.759	0.061
1392040_at	Sass6	spindle assembly 6 homolog (C. elegans)	uniq_RT1uvsDR_883	0	6.74	7.28	7.05	7.70	0	0.544	1.458	0.168	0	0.310	1.240	0.461	1	0.962	1.949	0.033
1392079_at	Akap7	A kinase (PRKA) anchor protein 7	uniq_RT1uvsDR_883	0	4.67	5.14	4.81	5.48	0	0.469	1.384	0.253	0	0.140	1.102	0.829	1	0.803	1.745	0.073
1392145_at	---	---	uniq_RT1uvsDR_883	0	8.63	9.02	9.06	9.53	0	0.387	1.308	0.362	0	0.430	1.348	0.247	1	0.903	1.870	0.040
1392166_at	---	---	uniq_RT1uvsDR_883	0	10.17	10.35	10.03	9.11	0	0.172	1.127	0.850	0	-0.141	-1.103	0.794	1	-1.068	-2.097	0.001
1392192_at	---	---	uniq_RT1uvsDR_883	0	5.27	5.30	5.24	4.61	0	0.022	1.016	0.997	0	-0.031	-1.022	0.959	1	-0.662	-1.583	0.034
1392206_at	Slc35f4	solute carrier family 35, member F4	uniq_RT1uvsDR_883	0	7.23	7.49	7.26	5.82	0	0.259	1.197	0.573	0	0.030	1.021	1.103	1	-1.407	-2.653	0.000
1392228_at	---	---	uniq_RT1uvsDR_883	0	4.00	3.51	3.51	3.35	0	-0.490	-1.405	0.106	0	-0.487	-1.402	0.098	1	-0.651	-1.571	0.035
1392237_at	---	---	uniq_RT1uvsDR_883	0	3.21	3.00	3.00	2.65	0	-0.206	-1.154	0.707	0	-0.213	-1.159	0.693	1	-0.561	-1.476	0.063
1392306_at	---	---	uniq_RT1uvsDR_883	0	4.27	4.06	4.09	5.02	0	-0.207	-1.154	0.775	0	-0.178	-1.131	0.787	1	0.747	1.678	0.089
1392337_at	Gpatch11	G patch domain containing 11	uniq_RT1uvsDR_883	0	5.76	5.87	5.51	5.24	0	0.110	1.079	1.028	0	-0.250	-1.189	0.579	1	-0.525	-1.439	0.076
1392380_at	---	---	uniq_RT1uvsDR_883	0	5.96	5.88	6.38	5.25	0	-0.077	-1.055	1.026	0	0.424	1.342	0.252	1	-0.704	-1.629	0.027
1392392_at	---	---	uniq_RT1uvsDR_883	0	3.24	3.54	3.40	6.20	0	0.302	1.233	0.565	0	0.163	1.119	0.878	1	2.964	7.802	0.000
1392404_at	---	---	uniq_RT1uvsDR_883	0	5.14	4.90	4.86	4.59	0	-0.240	-1.181	0.632	0	-0.278	-1.213	0.478	1	-0.549	-1.463	0.072
1392448_at	---	---	uniq_RT1uvsDR_883	0	4.53	4.32	4.28	3.86	0	-0.209	-1.156	0.730	0	-0.251	-1.190	0.575	1	-0.670	-1.591	0.024
1392586_at	Ap5m1	adaptor-related protein complex 5, mu 1 subunit	uniq_RT1uvsDR_883	0	6.20	6.23	6.12	7.15	0	0.030	1.021	1.162	0	-0.075	-1.054	1.047	1	0.955	1.939	0.032
1392598_at	Dnajb6	DnaJ (Hsp40) homolog, subfamily B, member 6	uniq_RT1uvsDR_883	0	7.70	7.99	8.07	7.03	0	0.289	1.221	0.558	0	0.369	1.292	0.345	1	-0.663	-1.583	0.028
1392653_at	LOC10091039	uncharacterized LOC100910396	uniq_RT1uvsDR_883	0	7.54	7.52	7.34	6.98	0	-0.018	-1.013	1.081	0	-0.196	-1.146	0.735	1	-0.561	-1.476	0.065
1392702_at	---	---	uniq_RT1uvsDR_883	0	5.25	5.62	5.62	6.09	0	0.373	1.295	0.399	0	0.370	1.292	0.366	1	0.843	1.794	0.053
1392736_at	---	---	uniq_RT1uvsDR_883	0	7.08	7.25	7.21	7.98	0	0.169	1.124	0.908	0	0.130	1.094	0.934	1	0.891	1.855	0.042
1392798_at	Pdia2	protein disulfide isomerase family A, member 2	uniq_RT1uvsDR_883	0	6.15	6.72	5.86	7.17	0	0.568	1.483	0.160	0	-0.293	-1.225	0.424	1	1.022	2.030	0.026
1392860_at	---	---	uniq_RT1uvsDR_883	0	6.49	6.72	6.75	3.91	0	0.229	1.172	0.679	0	0.261	1.198	0.571	1	-2.574	-5.955	0.000
1392870_at	---	---	uniq_RT1uvsDR_883	0	5.58	5.17	5.41	4.52	0	-0.416	-1.334	0.181	0	-0.177	-1.131	0.643	1	-1.065	-2.092	0.001
1392894_at	Fgl2	fibrinogen-like 2	uniq_RT1uvsDR_883	0	7.99	7.85	7.64	7.19	0	-0.140	-1.102	0.950	0	-0.356	-1.279	0.295	1	-0.801	-1.742	0.008
1392935_at	---	---	uniq_RT1uvsDR_883	0	6.83	6.60	6.87	6.12	0	-0.234	-1.176	0.642	0	0.036	1.026	1.112	1	-0.715	-1.642	0.016
1392947_at	---	---	uniq_RT1uvsDR_883	0	5.67	5.88	5.90	6.42	0	0.208	1.155	0.778	0	0.235	1.177	0.674	1	0.753	1.685	0.091
1392963_at	Scrn1	secernin 1	uniq_RT1uvsDR_883	0	6.26	6.42	6.28	7.00	0	0.159	1.117	0.936	0	0.012	1.008	1.143	1	0.732	1.661	0.091
1392967_at	MGC116121	similar to RIKEN cDNA 2700062C07	uniq_RT1uvsDR_883	0	7.67	7.86	7.66	7.10	0	0.187	1.139	0.849	0	-0.013	-1.009	1.133	1	-0.574	-1.489	0.055
1392980_at	Tiam1	T-cell lymphoma invasion and metastasis 1	uniq_RT1uvsDR_883	0	6.86	6.92	6.76	7.95	0	0.056	1.040	1.065	0	-0.103	-1.074	0.880	1	1.087	2.124	0.021
1392990_at	Sox17	SRY (sex determining region Y)-box 17	uniq_RT1uvsDR_883	0	6.89	6.90	6.74	6.24	0	0.008	1.006	1.100	0	-0.155	-1.114	0.746	1	-0.654	-1.573	0.028
1393020_at	Zmym6nb	ZMYM6 neighbor	uniq_RT1uvsDR_883	0	7.34	7.75	7.62	6.37	0	0.405	1.324	0.332	0	0.280	1.215	0.543	1	-0.973	-1.962	0.002
1393030_at	LOC10091044	protein PRRC2C-like /// hypothetical protein LOC68	uniq_RT1uvsDR_883	0	4.34	4.24	4.44	5.41	0	-0.104	-1.074	0.989	0	0.102	1.073	0.987	1	1.072	2.103	0.021
1393068_at	---	---	uniq_RT1uvsDR_883	0	5.00	5.14	5.00	6.21	0	0.136	1.099	0.940	0	-0.004	-1.002	1.085	1	1.208	2.310	0.013
1393069_at	Sfrp5	secreted frizzled-related protein 5	uniq_RT1uvsDR_883	0	6.89	6.51	6.93	6.39	0	-0.383	-1.304	0.207	0	0.038	1.027	1.104	1	-0.507	-1.421	0.092
1393084_at	Hrasls	HRAS-like suppressor	uniq_RT1uvsDR_883	0	5.40	5.28	5.33	4.57	0	-0.118	-1.085	0.985	0	-0.065	-1.046	1.016	1	-0.829	-1.776	0.007
1393140_at	Zc3h12a	zinc finger CCCH type containing 12A	uniq_RT1uvsDR_883	0	4.73	4.32	4.53	4.16	0	-0.407	-1.326	0.224	0	-0.196	-1.145	0.670	1	-0.568	-1.483	0.072
1393174_at	Fam171b	family with sequence similarity 171, member B	uniq_RT1uvsDR_883	0	4.30	4.77	4.39	5.46	0	0.466	1.381	0.289	0	0.090	1.064	0.886	1	1.159	2.234	0.017
1393235_at	---	---	uniq_RT1uvsDR_883	0	8.87	8.65	8.92	8.28	0	-0.217	-1.162	0.736	0	0.049	1.034	1.083	1	-0.596	-1.512	0.041
1393252_at	---	---	uniq_RT1uvsDR_883	0	5.77	5.36	5.44	5.16	0	-0.415	-1.333	0.198	0	-0.335	-1.261	0.324	1	-0.616	-1.533	0.051
1393263_at	Snhg11	small nucleolar RNA host gene 11 (non-protein codi	uniq_RT1uvsDR_883	0	10.96	10.73	10.93	10.14	0	-0.230	-1.173	0.649	0	-0.033	-1.023	1.101	1	-0.815	-1.759	0.007
1393302_at	---	---	uniq_RT1uvsDR_883	0	5.69	5.35	5.68	4.94	0	-0.339	-1.265	0.401	0	-0.015	-1.010	1.124	1	-0.746	-1.678	0.012
1393314_at	---	---	uniq_RT1uvsDR_883	0	7.97	7.55	7.63	6.80	0	-0.419	-1.337	0.206	0	-0.337	-1.263	0.369	1	-1.171	-2.252	0.001

Affymetrix Probeset ID	Gene Symbol	Gene Title	Figure 7 A Venn n=2289	Present in any 2 analyses n=423 1=yes 0=no	Mean log2 intensity DR+/+ ND	Mean log2 intensity DR+/+ HCD	Mean log2 intensity DR+/+ B/S	Mean log2 intensity F+/+ day 40 ND	DR+/+HCD vs DR+/+ND FDR<10% Log2 ratio >0.5 n=636	Log2 ratio DR+/+HCD vs DR+/+ ND	Fold-Change DR+/+HCD vs DR+/+ ND	FDR DR+/+HCD vs DR+/+ ND	DR+/+B/S vs DR+/+ND Log2 ratio >0.5 n=350	Log2 ratio DR+/+ B/S vs DR+/+ ND	Fold-Change DR+/+B/S vs DR+/+ ND	FDR DR+/+B/S vs DR+/+ ND	F+/+ vs DR+/+ND Log2 ratio >0.5 n=1209	Log2 ratio F+/+ vs DR+/+ ND	Fold-Change F+/+ vs DR+/+ ND	FDR F+/+ vs DR+/+ ND
1393324_at	Jam2	junctional adhesion molecule 2	uniq_RT1uvsDR_883	0	5.54	5.44	5.50	4.80	0	-0.102	-1.073	1.014	0	-0.041	-1.029	1.068	1	-0.745	-1.676	0.011
1393330_at	LOC10090953	uncharacterized LOC100909539	uniq_RT1uvsDR_883	0	7.40	7.49	7.63	6.81	0	0.090	1.064	1.083	0	0.232	1.175	0.674	1	-0.589	-1.505	0.051
1393421_at	Pxmp4	peroxisomal membrane protein 4	uniq_RT1uvsDR_883	0	7.17	7.31	7.26	5.68	0	0.140	1.102	0.909	0	0.092	1.066	0.984	1	-1.492	-2.812	0.000
1393469_at	---	---	uniq_RT1uvsDR_883	0	5.72	5.86	5.77	4.72	0	0.144	1.105	0.976	0	0.050	1.035	1.093	1	-0.997	-1.995	0.002
1393491_at	Tbl1x	transducin (beta)-like 1 X-linked	uniq_RT1uvsDR_883	0	5.77	6.40	5.90	6.59	0	0.631	1.549	0.104	0	0.128	1.093	0.855	1	0.825	1.771	0.067
1393585_at	---	---	uniq_RT1uvsDR_883	0	8.35	8.35	8.24	7.80	0	-0.001	-1.001	1.176	0	-0.105	-1.075	0.961	1	-0.548	-1.462	0.063
1393623_at	---	---	uniq_RT1uvsDR_883	0	8.11	8.42	8.26	9.01	0	0.305	1.235	0.540	0	0.142	1.103	0.936	1	0.893	1.857	0.042
1393632_at	C1qtnf7	C1q and tumor necrosis factor related protein 7	uniq_RT1uvsDR_883	0	4.23	4.13	4.28	3.59	0	-0.097	-1.070	0.961	0	0.047	1.033	0.899	1	-0.643	-1.562	0.033
1393672_at	Hmcn1	hemicentin 1	uniq_RT1uvsDR_883	0	4.36	4.14	4.00	3.62	0	-0.217	-1.162	0.693	0	-0.358	-1.281	0.295	1	-0.742	-1.673	0.011
1393723_at	Plk5	polo-like kinase 5	uniq_RT1uvsDR_883	0	6.39	6.15	6.30	5.74	0	-0.240	-1.181	0.690	0	-0.089	-1.064	1.029	1	-0.645	-1.563	0.029
1393743_at	---	---	uniq_RT1uvsDR_883	0	4.07	4.31	4.04	5.75	0	0.242	1.183	0.638	0	-0.034	-1.024	1.062	1	1.680	3.203	0.005
1393746_at	---	---	uniq_RT1uvsDR_883	0	5.93	5.93	6.06	5.19	0	0.000	-1.000	1.163	0	0.127	1.092	0.951	1	-0.737	-1.667	0.013
1393756_at	Dmp1	dentin matrix acidic phosphoprotein 1	uniq_RT1uvsDR_883	0	5.24	5.07	5.05	4.71	0	-0.164	-1.120	0.837	0	-0.192	-1.143	0.630	1	-0.531	-1.445	0.089
1393759_at	---	---	uniq_RT1uvsDR_883	0	5.08	4.98	5.01	4.57	0	-0.097	-1.070	0.991	0	-0.073	-1.052	0.869	1	-0.514	-1.428	0.093
1393762_at	Pcaf	p300/CBP-associated factor	uniq_RT1uvsDR_883	0	4.73	4.93	4.77	5.57	0	0.200	1.149	0.762	0	0.038	1.027	1.031	1	0.842	1.793	0.059
1393765_at	---	---	uniq_RT1uvsDR_883	0	6.14	5.68	5.81	5.06	0	-0.462	-1.378	0.114	0	-0.335	-1.261	0.309	1	-1.080	-2.115	0.001
1393766_at	---	---	uniq_RT1uvsDR_883	0	4.86	4.96	5.20	4.27	0	0.099	1.071	1.015	0	0.337	1.263	0.413	1	-0.590	-1.505	0.048
1393784_at	Vezf1	vascular endothelial zinc finger 1	uniq_RT1uvsDR_883	0	6.47	6.65	6.65	5.88	0	0.174	1.128	0.860	0	0.178	1.131	0.781	1	-0.597	-1.512	0.048
1393785_at	Iqca1	IQ motif containing with AAA domain 1	uniq_RT1uvsDR_883	0	3.34	3.36	3.36	2.76	0	0.020	1.014	1.041	0	0.019	1.013	1.006	1	-0.576	-1.491	0.071
1393795_at	Zeb2	zinc finger E-box binding homeobox 2	uniq_RT1uvsDR_883	0	5.77	5.34	5.41	4.71	0	-0.432	-1.349	0.122	0	-0.365	-1.288	0.279	1	-1.062	-2.088	0.001
1393807_at	---	---	uniq_RT1uvsDR_883	0	4.02	4.00	4.08	4.81	0	-0.012	-1.009	1.149	0	0.061	1.043	0.974	1	0.789	1.727	0.096
1393809_at	Traf6	TNF receptor-associated factor 6, E3 ubiquitin prote	uniq_RT1uvsDR_883	0	5.48	5.46	5.47	7.04	0	-0.022	-1.015	0.992	0	-0.006	-1.004	1.101	1	1.558	2.945	0.006
1393831_at	---	---	uniq_RT1uvsDR_883	0	3.18	3.14	3.19	4.28	0	-0.046	-1.033	1.099	0	0.005	1.004	1.148	1	1.101	2.146	0.021
1393842_at	Ccdc77	coiled-coil domain containing 77	uniq_RT1uvsDR_883	0	7.29	7.46	7.28	5.46	0	0.167	1.123	0.854	0	-0.013	-1.009	1.069	1	-1.831	-3.558	0.000
1393881_at	Naa15	N(alpha)-acetyltransferase 15, NatA auxiliary subuni	uniq_RT1uvsDR_883	0	8.85	8.74	8.93	8.04	0	-0.105	-1.075	0.881	0	0.084	1.060	0.969	1	-0.806	-1.748	0.008
1393901_at	Runx1t1	runt-related transcription factor 1; translocated to,	uniq_RT1uvsDR_883	0	5.17	5.37	5.29	6.30	0	0.201	1.149	0.797	0	0.120	1.087	0.827	1	1.128	2.186	0.020
1393907_at	Pyy	peptide YY (mapped)	uniq_RT1uvsDR_883	0	12.48	12.98	12.36	11.92	0	0.500	1.415	0.199	0	-0.119	-1.086	0.926	1	-0.560	-1.474	0.059
1393920_at	---	---	uniq_RT1uvsDR_883	0	5.80	5.77	5.90	6.54	0	-0.033	-1.023	1.132	0	0.097	1.070	0.907	1	0.739	1.669	0.091
1393937_at	---	---	uniq_RT1uvsDR_883	0	8.09	8.53	8.28	7.56	0	0.446	1.362	0.272	0	0.198	1.147	0.759	1	-0.530	-1.444	0.078
1393995_at	---	---	uniq_RT1uvsDR_883	0	7.62	7.59	7.53	8.50	0	-0.030	-1.021	1.094	0	-0.084	-1.060	0.944	1	0.880	1.840	0.050
1394097_at	Kcnip1	Kv channel-interacting protein 1	uniq_RT1uvsDR_883	0	8.53	9.04	8.87	7.86	0	0.515	1.429	0.179	0	0.343	1.268	0.396	1	-0.663	-1.584	0.024
1394160_at	Tmem2	transmembrane protein 2	uniq_RT1uvsDR_883	0	5.38	5.37	5.43	6.14	0	-0.011	-1.008	1.052	0	0.044	1.031	0.914	1	0.758	1.691	0.095
1394320_at	Cdk2	cyclin dependent kinase 2	uniq_RT1uvsDR_883	0	5.32	5.30	5.12	4.75	0	-0.023	-1.016	1.116	0	-0.206	-1.154	0.720	1	-0.570	-1.484	0.051
1394347_at	Golga4	golgin A4	uniq_RT1uvsDR_883	0	6.26	6.81	6.64	7.55	0	0.542	1.456	0.194	0	0.377	1.298	0.313	1	1.281	2.431	0.012
1394401_at	Elovl6 /// LOC	ELOVL fatty acid elongase 6 /// elongation of very lo	uniq_RT1uvsDR_883	0	7.96	8.51	8.35	7.42	0	0.549	1.463	0.162	0	0.394	1.314	0.306	1	-0.537	-1.451	0.075
1394448_at	---	---	uniq_RT1uvsDR_883	0	7.33	7.37	7.60	6.23	0	0.043	1.030	1.153	0	0.274	1.209	0.554	1	-1.091	-2.130	0.001
1394477_at	LOC682058	similar to nucleolar protein with MIF4G domain 1	uniq_RT1uvsDR_883	0	8.30	8.48	8.45	5.87	0	0.183	1.136	0.855	0	0.155	1.113	0.905	1	-2.433	-5.399	0.000
1394520_at	Setd5	SET domain containing 5	uniq_RT1uvsDR_883	0	5.55	5.78	5.46	4.67	0	0.222	1.167	0.624	0	-0.098	-1.070	0.839	1	-0.882	-1.842	0.005
1394538_at	---	---	uniq_RT1uvsDR_883	0	4.17	3.74	3.75	3.44	0	-0.435	-1.352	0.197	0	-0.421	-1.339	0.190	1	-0.735	-1.664	0.013
1394550_at	LOC691221	similar to CG1998-PA	uniq_RT1uvsDR_883	0	6.32	6.33	6.32	5.65	0	0.011	1.008	1.169	0	0.000	-1.000	1.130	1	-0.672	-1.593	0.024
1394585_at	---	---	uniq_RT1uvsDR_883	0	8.37	8.27	8.41	7.58	0	-0.106	-1.076	1.015	0	0.043	1.030	1.123	1	-0.795	-1.735	0.009
1394620_at	---	---	uniq_RT1uvsDR_883	0	7.08	7.29	7.17	6.11	0	0.211	1.157	0.784	0	0.092	1.066	1.040	1	-0.971	-1.960	0.002
1394671_at	---	---	uniq_RT1uvsDR_883	0	5.87	5.93	5.81	4.72	0	0.056	1.040	1.071	0	-0.065	-1.046	1.015	1	-1.153	-2.224	0.001
1394675_at	---	---	uniq_RT1uvsDR_883	0	5.01	4.57	4.74	4.30	0	-0.438	-1.355	0.187	0	-0.275	-1.210	0.537	1	-0.711	-1.636	0.015
1394693_at	---	---	uniq_RT1uvsDR_883	0	7.43	7.89	7.36	5.68	0	0.463	1.378	0.288	0	-0.067	-1.047	0.931	1	-1.745	-3.352	0.000
1394695_at	Fam46a	family with sequence similarity 46, member A	uniq_RT1uvsDR_883	0	5.93	6.23	6.00	7.02	0	0.293	1.225	0.574	0	0.070	1.050	0.786	1	1.085	2.122	0.021
1394805_at	---	---	uniq_RT1uvsDR_883	0	5.38	5.37	5.70	4.58	0	-0.009	-1.006	1.176	0	0.320	1.248	0.446	1	-0.799	-1.740	0.008
1394807_at	---	---	uniq_RT1uvsDR_883	0	5.69	5.68	5.78	4.85	0	-0.011	-1.007	1.116	0	0.090	1.064	1.061	1	-0.841	-1.791	0.006
1394839_at	---	---	uniq_RT1uvsDR_883	0	3.16	3.13	2.98	6.42	0	-0.027	-1.019	1.149	0	-0.183	-1.135	0.743	1	3.258	9.564	0.000
1394940_at	Fam46a	family with sequence similarity 46, member A	uniq_RT1uvsDR_883	0	8.52	9.14	8.77	10.21	0	0.617	1.534	0.113	0	0.243	1.183	0.623	1	1.685	3.215	0.006
1394946_at	Rgd1310049	uncharacterized LOC292199	uniq_RT1uvsDR_883	0	7.53	7.55	7.37	8.64	0	0.026	1.018	1.169	0	-0.160	-1.117	0.810	1	1.114	2.164	0.018
1394959_at	---	---	uniq_RT1uvsDR_883	0	5.99	6.16	6.18	7.18	0	0.164	1.120	0.886	0	0.189	1.140	0.758	1	1.189	2.280	0.014
1394976_at	---	---	uniq_RT1uvsDR_883	0	5.47	5.13	5.23	4.50	0	-0.334	-1.260	0.376	0	-0.240	-1.181	0.557	1	-0.971	-1.960	0.002
1395058_at	Wdr75	WD repeat domain 75	uniq_RT1uvsDR_883	0	5.86	6.35	6.06	6.62	0	0.495	1.409	0.260	0	0.202	1.150	0.621	1	0.765	1.699	0.096
1395064_at	S100pbp	S100P binding protein	uniq_RT1uvsDR_883	0	8.18	8.22	8.38	7.52	0	0.043	1.030	1.150	0	0.209	1.156	0.735	1	-0.651	-1.570	0.028
1395073_at	---	---	uniq_RT1uvsDR_883	0	5.11	4.71	4.97	4.42	0	-0.407	-1.326	0.236	0	-0.143	-1.104	0.869	1	-0.698	-1.623	0.018
1395076_at	Runx1t1	runt-related transcription factor 1; translocated to,	uniq_RT1uvsDR_883	0	4.45	4.55	4.36	5.40	0	0.097	1.069	0.996	0	-0.091	-1.065	0.833	1	0.944	1.924	0.037
1395080_at	---	---	uniq_RT1uvsDR_883	0	4.78	4.41	4.54	4.18	0	-0.369	-1.291	0.277	0	-0.240	-1.181	0.608	1	-0.594	-1.509	0.049
1395127_at	---	---	uniq_RT1uvsDR_883	0	3.73	3.97	3.75	4.49	0	0.246	1.186	0.690	0	0.025	1.017	1.120	1	0.761	1.695	0.090
1395153_at	---	---	uniq_RT1uvsDR_883	0	4.17	3.69	3.87	3.57	0	-0.472	-1.387	0.105	0	-0.296	-1.228	0.460	1	-0.595	-1.510	0.051
1395166_at	Ehd2	EH-domain containing 2	uniq_RT1uvsDR_883	0	5.93	5.70	5.50	5.34	0	-0.225	-1.169	0.722	0	-0.424	-1.341	0.173	1	-0.585	-1.500	0.051
1395191_at	---	---	uniq_RT1uvsDR_883	0	4.03	4.04	4.27	3.31	0	0.005	1.003	1.141	0	0.237	1.179	0.621	1	-0.729	-1.657	0.018
1395203_at	LOC1009103	uncharacterized LOC100910396	uniq_RT1uvsDR_883	0	6.63	6.49	6.35	5.71	0	-0.146	-1.106	0.809	0	-0.282	-1.216	0.505	1	-0.921	-1.893	0.003
1395221_at	Snx13	sorting nexin 13	uniq_RT1uvsDR_883	0	4.15	4.30	4.55	4.90	0	0.141	1.103	0.873	0	0.391	1.312	0.301	1	0.749	1.681	0.091
1395225_at	Uhrf1bp1	UHRF1 binding protein 1	uniq_RT1uvsDR_883	0	5.84	5.41	5.59	5.18	0	-0.428	-1.345	0.207	0	-0.248	-1.187	0.571	1	-0.657	-1.577	0.026
1395246_at	---	---	uniq_RT1uvsDR_883	0	5.21	5.52	4.98	6.05	0	0.306	1.236	0.539	0	-0.234	-1.176	0.640	1	0.837	1.787	0.059
1395294_at	---	---	uniq_RT1uvsDR_883	0	5.29	5.38	5.46	6.19	0	0.092	1.066	1.003	0	0.171	1.125	0.727	1	0.898	1.864	0.042
1395358_at	Rhobtb3	Rho-related BTB domain containing 3	uniq_RT1uvsDR_883	0	6.05	5.98	6.24	5.49	0	-0.075	-1.053	0.892	0	0.184	1.136	0.673	1	-0.565	-1.479	0.071
1395366_at	---	---	uniq_RT1uvsDR_883	0	6.68	6.80	6.91	5.37	0	0.116	1.083	1.042	0	0.232	1.174	0.686	1	-1.314	-2.486	0.000
1395505_at	---	---	uniq_RT1uvsDR_883	0	6.17	6.74	6.54	5.21	0	0.571	1.486	0.172	0	0.373	1.295	0.321	1	-0.958	-1.942	0.003
1395518_at	---	---	uniq_RT1uvsDR_883	0	5.08	5.20	5.02	6.31	0	0.125	1.091	0.971	0	-0.052	-1.037	1.083	1	1.229	2.343	0.012
1395536_at	---	---	uniq_RT1uvsDR_883	0	8.69	8.92	8.89	7.77	0	0.235	1.177	0.703	0	0.199	1.148	0.763	1	-0.918	-1.889	0.003
1395546_at	---	---	uniq_RT1uvsDR_883	0	5.26	5.36	5.28	3.92	0	0.100	1.072	1.038	0	0.025	1.018	1.033	1	-1.338	-2.527	0.000
1395705_at	---	---	uniq_RT1uvsDR_883	0	6.75	6.51	7.00	5.58	0	-0.240	-1.181	0.649	0	0.251	1.190	0.586	1	-1.171	-2.252	0.001
1395742_at	---	---	uniq_RT1uvsDR_883	0	5.36	5.68	5.51	4.86	0	0.320	1.248	0.480	0	0.145	1.106	0.801	1	-0.502	-1.416	0.098
1395768_at	---	---	uniq_RT1uvsDR_883	0	5.84	5.70	5.90	5.19	0	-0.131	-1.095	1.005	0	0.068	1.049	1.041	1	-0.649	-1.568	0.027
1395806_at	LOC1009094	disks large homolog 5-like /// disks large homolog 5	uniq_RT1uvsDR_883	0	3.66	3.51	3.63	5.21	0	-0.148	-1.108	0.902	0	-0.021	-1.015	1.108	1	1.556	2.940	0.005
1395927_at	---	---	uniq_RT1uvsDR_883	0	6.99	7.10	7.05	6.02	0	0.112	1.081	1.024	0	0.057	1.040	1.017	1	-0.968	-1.957	0.002
1395937_at	Arfgef1	ADP-ribosylation factor guanine nucleotide-exchang	uniq_RT1uvsDR_883	0	5.98	5.53	5.67	5.44	0	-0.451	-1.367	0.159	0	-0.316	-1.245	0.351	1	-0.538	-1.452	0.068
1395990_at	Srcap	Snf2-related CREBBP activator protein	uniq_RT1uvsDR_883	0	6.94	6.66	6.80	6.22	0	-0.286	-1.219	0.576	0	-0.139	-1.101	0.885	1	-0.725	-1.652	0.014
1396020_at	Tlk2	tousled-like kinase 2	uniq_RT1uvsDR_883	0	6.77	6.43	6.45	5.74	0	-0.343	-1.269	0.389	0	-0.324	-1.252	0.409	1	-1.036	-2.050	0.002
1396037_at	---	---	uniq_RT1uvsDR_883	0	6.23	6.08	6.37	5.71	0	-0.150	-1.109	0.919	0	0.144	1.105	0.876	1	-0.525	-1.439	0.077
1396049_x_at	Rpl3	ribosomal protein L3	uniq_RT1uvsDR_883	0	7.05	7.02	7.04	6.07	0	-0.025	-1.018	1.142	0	-0.007	-1.005	1.108	1	-0.974	-1.965	0.002
1396065_at	Rnase6	ribonuclease, RNase A family, 6	uniq_RT1uvsDR_883	0	4.15	4.67	3.88	5.28	0	0.517	1.431	0.208	0	-0.267	-1.204	0.509	1	1.126	2.183	0.021
1396102_at	---	---	uniq_RT1uvsDR_883	0	5.85	5.92	5.64	5.27	0	0.073	1.052	1.109	0	-0.211	-1.157	0.671	1	-0.583	-1.498	0.063
1396223_at	---	---	uniq_RT1uvsDR_883	0	6.68	6.47	6.53	6.11	0	-0.209	-1.156	0.787	0	-0.148	-1.108	0.887	1	-0.574	-1.489	0.052
1396233_at	Arsk	arylsulfatase family, member K	uniq_RT1uvsDR_883	0	3.06	3.40	3.20	3.93	0	0.346	1.271	0.455	0	0.145	1.106	0.888	1	0.870	1.828	0.062
1396246_at	---	---	uniq_RT1uvsDR_883	0	5.42	5.20	5.23	4.59	0	-0.217	-1.162	0.750	0	-0.186	-1.137	0.600	1	-0.831	-1.779	0.006
1396274_at	LOC682431	similar to IQ motif containing C	uniq_RT1uvsDR_883	0	7.32	7.52	7.58	6.63	0	0.196	1.146	0.811	0	0.258	1.196	0.605	1	-0.699	-1.623	0.019
1396288_s_at	---	---	uniq_RT1uvsDR_883	0	4.71	4.84	4.67	4.00	0	0.123	1.089	0.906	0	-0.043	-1.030	1.100	1	-0.714	-1.640	0.015
1396310_at	---	---	uniq_RT1uvsDR_883	0	7.18	7.10	7.19	6.40	0	-0.079	-1.056	1.089	0	0.006	1.004	1.140	1	-0.786	-1.725	0.008
1396331_at	---	---	uniq_RT1uvsDR_883	0	5.63	5.44	5.50	5.06	0	-0.195	-1.144	0.685	0	-0.128	-1.093	0.811	1	-0.572	-1.486	0.063
1396344_at	Nt5dc1	5'-nucleotidase domain containing 1	uniq_RT1uvsDR_883	0	3.46	3.59	3.64	6.94	0	0.131	1.095	0.989	0	0.179	1.132	0.769	1	3.485	11.196	0.000
1396347_at	LOC1009118	uncharacterized LOC100911864	uniq_RT1uvsDR_883	0	3.87	3.41	3.52	2.92	0	-0.451	-1.367	0.139	0	-0.342	-1.268	0.335	1	-0.946	-1.927	0.003
1396420_at	---	---	uniq_RT1uvsDR_883	0	5.45	5.74	5.81	3.39	0	0.297	1.229	0.539	0	0.367	1.290	0.345	1	-2.055	-4.155	0.000
1396442_at	---	---	uniq_RT1uvsDR_883	0	4.71	4.78	4.83	5.57	0	0.066	1.047	1.122	0	0.120	1.086	0.730	1	0.856	1.810	0.079
1396463_at	Pnn	pinin, desmosome associated protein	uniq_RT1uvsDR_883	0	4.84	4.87	5.06	5.63	0	0.030	1.021	1.083	0	0.219	1.164	0.637	1	0.787	1.726	0.090
1396559_at	---	---	uniq_RT1uvsDR_883	0	4.29	4.19	4.26	3.72	0	-0.102	-1.073	1.070	0	-0.033	-1.023	1.123	1	-0.573	-1.488	0.050
1396621_at	---	---	uniq_RT1uvsDR_883	0	7.69	7.99	7.90	8.45	0	0.302	1.233	0.565	0	0.204	1.152	0.673	1	0.757	1.690	0.086
1396764_at	---	---	uniq_RT1uvsDR_883	0	6.73	6.94	6.66	6.13	0	0.204	1.152	0.809	0	-0.068	-1.048	1.029	1	-0.604	-1.520	0.042
1396775_at	---	---	uniq_RT1uvsDR_883	0	5.42	5.57	5.56	7.12	0	0.147	1.107	0.759	0	0.140	1.102	0.674	1	1.695	3.238	0.005
1396797_at	Nova2	neuro-oncological ventral antigen 2	uniq_RT1uvsDR_883	0	3.76	3.31	3.29	3.03	0	-0.451	-1.367	0.159	0	-0.466	-1.381	0.113	1	-0.723	-1.651	0.016
1396831_at	---	---	uniq_RT1uvsDR_883	0	6.97	7.24	7.20	6.25	0	0.268	1.204	0.595	0	0.231	1.174	0.643	1	-0.718	-1.645	0.019
1396846_at	---	---	uniq_RT1uvsDR_883	0	3.95	4.48	3.89	3.31	0	0.530	1.444	0.181	0	-0.058	-1.041	1.070	1	-0.632	-1.549	0.030
1396909_at	---	---	uniq_RT1uvsDR_883	0	5.15	4.90	4.99	4.53	0	-0.247	-1.186	0.690	0	-0.164	-1.120	0.853	1	-0.621	-1.538	0.034
1396917_at	---	---	uniq_RT1uvsDR_883	0	6.71	6.40	6.61	5.68	0	-0.315	-1.244	0.440	0	-0.098	-1.070	0.960	1	-1.033	-2.046	0.001
1396947_at	---	---	uniq_RT1uvsDR_883	0	7.62	7.67	7.91	7.00	0	0.049	1.035	1.126	0	0.294	1.226	0.498	1	-0.616	-1.533	0.043

Affymetrix Probeset ID	Gene Symbol	Gene Title	Figure 7 A Venn n=2289	Present in any 2 analyses n=423 1=yes 0=no	Mean log2 intensity DR+/+ ND	Mean log2 intensity DR+/+ HCD	Mean log2 intensity DR+/+ B/S	Mean log2 intensity F+/+ day 40 ND	DR+/+HCD vs DR+/+ND FDR<10% Log2 ratio >0.5 n=636	Log2 ratio DR+/+HCD vs DR+/+ ND	Fold-Change DR+/+HCD vs DR+/+ ND	FDR DR+/+HCD vs DR+/+ ND	DR+/+B/S vs DR+/+ND Log2 ratio >0.5 n=350	Log2 ratio DR+/+ B/S vs DR+/+ ND	Fold-Change DR+/+B/S vs DR+/+ ND	FDR DR+/+B/S vs DR+/+ ND	F+/+ vs DR+/+ND Log2 ratio >0.5 n=1209	Log2 ratio F+/+ vs DR+/+ ND	Fold-Change F+/+ vs DR+/+ ND	FDR F+/+ vs DR+/+ ND
1397096_at	---	---	uniq_RT1uvsDR_883	0	6.76	6.83	6.61	5.71	0	0.070	1.050	1.113	0	-0.156	-1.114	0.797	1	-1.054	-2.076	0.001
1397184_at	Rgd1310049	uncharacterized LOC292199	uniq_RT1uvsDR_883	0	7.65	7.50	7.62	8.63	0	-0.156	-1.114	0.894	0	-0.036	-1.025	1.121	1	0.975	1.966	0.030
1397200_at	Chd4	chromodomain helicase DNA binding protein 4	uniq_RT1uvsDR_883	0	7.54	7.95	7.53	8.29	0	0.414	1.332	0.370	0	-0.007	-1.005	0.996	1	0.748	1.679	0.100
1397226_at	---	---	uniq_RT1uvsDR_883	0	5.69	6.25	6.02	6.47	0	0.559	1.473	0.163	0	0.323	1.251	0.423	1	0.773	1.709	0.083
1397246_at	Ntrk2	neurotrophic tyrosine kinase, receptor, type 2	uniq_RT1uvsDR_883	0	9.20	8.99	9.19	8.50	0	-0.209	-1.156	0.648	0	-0.005	-1.003	1.147	1	-0.698	-1.622	0.017
1397268_at	Slc17a4	solute carrier family 17 (sodium phosphate), membe	uniq_RT1uvsDR_883	0	5.36	5.28	4.93	6.95	0	-0.080	-1.057	1.083	0	-0.433	-1.350	0.135	1	1.588	3.007	0.006
1397412_at	---	---	uniq_RT1uvsDR_883	0	5.70	5.99	5.99	6.83	0	0.283	1.217	0.599	0	0.284	1.217	0.520	1	1.122	2.176	0.018
1397435_at	---	---	uniq_RT1uvsDR_883	0	3.63	3.74	4.05	3.03	0	0.111	1.080	1.031	0	0.419	1.337	0.269	1	-0.608	-1.524	0.038
1397439_at	LOC497978	similar to diacylglycerol kinase epsilon	uniq_RT1uvsDR_883	0	8.29	8.66	8.35	9.27	0	0.373	1.295	0.409	0	0.065	1.046	1.070	1	0.981	1.974	0.030
1397463_at	Rab14	RAB14, member RAS oncogene family	uniq_RT1uvsDR_883	0	6.01	6.19	6.07	5.41	0	0.179	1.132	0.772	0	0.063	1.045	0.896	1	-0.595	-1.510	0.070
1397478_at	Phb2	prohibitin 2	uniq_RT1uvsDR_883	0	6.26	6.50	6.47	5.63	0	0.233	1.175	0.735	0	0.204	1.152	0.753	1	-0.634	-1.552	0.035
1397495_at	---	---	uniq_RT1uvsDR_883	0	6.21	6.33	6.38	6.96	0	0.120	1.087	0.955	0	0.165	1.121	0.827	1	0.746	1.677	0.095
1397502_at	---	---	uniq_RT1uvsDR_883	0	4.22	3.92	3.98	3.55	0	-0.299	-1.230	0.504	0	-0.240	-1.181	0.639	1	-0.672	-1.594	0.024
1397547_at	Ikbkb	inhibitor of kappa light polypeptide gene enhancer i	uniq_RT1uvsDR_883	0	2.89	2.95	3.28	3.67	0	0.057	1.040	1.114	0	0.388	1.309	0.316	1	0.783	1.720	0.075
1397569_at	---	---	uniq_RT1uvsDR_883	0	7.39	7.55	7.74	5.54	0	0.159	1.117	0.942	0	0.351	1.275	0.368	1	-1.851	-3.608	0.000
1397579_x_at	Apc2	adenomatosis polyposis coli 2	uniq_RT1uvsDR_883	0	8.51	8.38	8.66	7.78	0	-0.134	-1.097	0.901	0	0.150	1.110	0.902	1	-0.734	-1.663	0.012
1397592_at	Ubash3b	ubiquitin associated and SH3 domain containing, B	uniq_RT1uvsDR_883	0	5.06	4.84	4.98	4.56	0	-0.225	-1.169	0.686	0	-0.085	-1.061	0.970	1	-0.507	-1.421	0.097
1397617_at	Kdm5a	lysine (K)-specific demethylase 5A	uniq_RT1uvsDR_883	0	6.38	6.64	6.56	7.12	0	0.257	1.195	0.622	0	0.182	1.134	0.753	1	0.737	1.667	0.095
1397690_at	---	---	uniq_RT1uvsDR_883	0	5.32	5.19	5.22	6.12	0	-0.125	-1.090	0.979	0	-0.096	-1.069	0.888	1	0.805	1.747	0.069
1397745_at	---	---	uniq_RT1uvsDR_883	0	6.03	6.28	6.35	3.44	0	0.247	1.187	0.606	0	0.313	1.242	0.424	1	-2.590	-6.022	0.000
1397808_at	---	---	uniq_RT1uvsDR_883	0	5.73	5.49	5.41	5.12	0	-0.237	-1.179	0.603	0	-0.315	-1.244	0.381	1	-0.606	-1.522	0.041
1397855_at	---	---	uniq_RT1uvsDR_883	0	5.05	4.69	4.59	6.52	0	-0.366	-1.289	0.240	0	-0.460	-1.375	0.105	1	1.465	2.760	0.007
1397897_x_at	---	---	uniq_RT1uvsDR_883	0	6.98	7.31	7.18	6.13	0	0.328	1.255	0.472	0	0.199	1.148	0.742	1	-0.850	-1.802	0.013
1397941_at	Zc3h4	zinc finger CCCH-type containing 4	uniq_RT1uvsDR_883	0	5.27	4.98	4.90	4.69	0	-0.292	-1.225	0.482	0	-0.372	-1.294	0.294	1	-0.586	-1.501	0.044
1398005_at	Kbtbd2	kelch repeat and BTB (POZ) domain containing 2	uniq_RT1uvsDR_883	0	5.29	4.82	5.07	4.78	0	-0.468	-1.383	0.129	0	-0.216	-1.161	0.677	1	-0.508	-1.422	0.092
1398216_at	---	---	uniq_RT1uvsDR_883	0	7.38	7.35	7.72	6.50	0	-0.032	-1.023	1.103	0	0.340	1.266	0.404	1	-0.881	-1.842	0.005
1398273_at	Efna1	ephrin A1	uniq_RT1uvsDR_883	0	5.87	5.94	5.63	5.24	0	0.071	1.051	1.093	0	-0.239	-1.180	0.498	1	-0.628	-1.546	0.034
1398295_at	Slc29a1	solute carrier family 29 (nucleoside transporters), m	uniq_RT1uvsDR_883	0	7.34	7.62	7.47	6.72	0	0.276	1.211	0.624	0	0.130	1.094	0.923	1	-0.619	-1.535	0.035
1398333_at	---	---	uniq_RT1uvsDR_883	0	10.83	10.59	10.71	10.26	0	-0.244	-1.185	0.624	0	-0.124	-1.090	0.873	1	-0.576	-1.491	0.051
1398362_at	---	---	uniq_RT1uvsDR_883	0	6.36	6.10	6.09	5.51	0	-0.253	-1.191	0.533	0	-0.264	-1.201	0.524	1	-0.849	-1.801	0.007
1398365_at	Tppp3	tubulin polymerization-promoting protein family me	uniq_RT1uvsDR_883	0	7.18	6.96	7.02	6.62	0	-0.224	-1.168	0.686	0	-0.165	-1.121	0.803	1	-0.559	-1.473	0.057
1398497_at	---	---	uniq_RT1uvsDR_883	0	7.62	7.75	7.78	8.75	0	0.136	1.099	0.972	0	0.161	1.118	0.825	1	1.133	2.193	0.018
1398507_at	---	---	uniq_RT1uvsDR_883	0	5.82	6.00	6.14	4.93	0	0.179	1.132	0.862	0	0.320	1.249	0.450	1	-0.889	-1.852	0.005
1398538_at	Abhd8	abhydrolase domain containing 8	uniq_RT1uvsDR_883	0	6.00	6.00	5.77	5.49	0	-0.003	-1.002	1.171	0	-0.233	-1.175	0.634	1	-0.515	-1.429	0.094
1398540_at	Rgs1	regulator of G-protein signaling 1	uniq_RT1uvsDR_883	0	5.34	5.76	5.85	6.42	0	0.422	1.339	0.305	0	0.507	1.421	0.140	1	1.083	2.118	0.022
1398544_at	---	---	uniq_RT1uvsDR_883	0	5.68	5.37	5.73	4.58	0	-0.312	-1.241	0.393	0	0.052	1.037	1.038	1	-1.100	-2.143	0.001
1398568_at	---	---	uniq_RT1uvsDR_883	0	4.75	5.12	5.26	5.50	0	0.377	1.299	0.416	0	0.515	1.429	0.130	1	0.755	1.687	0.096
1398612_at	Akr1c12	aldo-keto reductase family 1, member C12	uniq_RT1uvsDR_883	0	7.46	7.63	7.68	8.49	0	0.171	1.126	0.850	0	0.213	1.159	0.731	1	1.023	2.032	0.025
1398646_at	Rgs22	regulator of G-protein signaling 22	uniq_RT1uvsDR_883	0	8.50	9.05	8.43	6.59	0	0.555	1.469	0.158	0	-0.066	-1.047	1.052	1	-1.905	-3.744	0.000
1398707_at	---	---	uniq_RT1uvsDR_883	0	5.11	5.40	5.59	7.25	0	0.284	1.218	0.552	0	0.482	1.396	0.172	1	2.141	4.412	0.001
1398729_s_at	LOC682058	similar to nucleolar protein with MIF4G domain 1	uniq_RT1uvsDR_883	0	5.67	6.02	5.96	4.42	0	0.348	1.273	0.440	0	0.294	1.226	0.480	1	-1.244	-2.368	0.000
1398867_at	LOC1009107ε	pre-mRNA-processing factor 19-like /// PRP19/PSO4	uniq_RT1uvsDR_883	0	9.40	9.76	9.68	8.89	0	0.358	1.282	0.440	0	0.281	1.215	0.544	1	-0.516	-1.430	0.087
1399065_at	Rbpms2	RNA binding protein with multiple splicing 2	uniq_RT1uvsDR_883	0	6.86	6.53	6.68	6.33	0	-0.327	-1.254	0.455	0	-0.177	-1.131	0.817	1	-0.529	-1.443	0.076

Data are available as a sortable spreadsheet upon request

[1] **Data are expressed in a binar fashion as meeting (1) or not meeting (0) the query defined in the header.**
