# Supplementary material for: Modulation of the diet and gastrointestinal microbiota normalizes systemic inflammation and β-cell chemokine expression associated with autoimmune diabetes susceptibility
Source: PLoS One. 2018 Jan 2;13(1):e0190351. doi: 10.1371/journal.pone.0190351 (PMC5749787; doi:10.1371/journal.pone.0190351)
Supplement: S3 Table — (PDF) [file pone.0190351.s003.pdf]

S3 Table: Operational Taxonomic Unit Table. On average, there were 3,607 +/- 966.08 mapped reads per sample. Among samples collected from the ileum, the total number of mapped 16S rRNA gene sequences did not differ between DR+/+ ND rats (4,316.5 +/- 940.7) and F+/+ ND rats (4,223.5 +/- 1,125.4). However, DR+/+ HCD (2,688.2 +/- 664.6) and DR+/+ B/S (2,523.5 +/- 655.5) exhibited fewer mapped reads (p<0.01). The total number of mapped 16S rRNA gene sequences did not differ among cecum samples of DR+/+ ND rats (3,885.2 +/- 601.7), DR+/+ HCD rats (3,966.8 +/- 401.9), DR+/+ B/S rats (3,690.5 +/-882.8), or F+/+ ND rats (3,567.0 +/- 840.7).

Taxon	Cecum DR+/+_ND_A1	Cecum DR+/+_ND_A2	Cecum DR+/+_ND_A3	Cecum DR+/+_ND_A4	Cecum DR+/+_ND_A5	Cecum DR+/+_ND_A6	Cecum DR+/+_HCD_B1	Cecum DR+/+_HCD_B2	Cecum DR+/+_HCD_B3	Cecum DR+/+_HCD_B4	Cecum DR+/+_HCD_B5	Cecum DR+/+_HCD_B6	Cecum DR+/+_B/S_C1	Cecum DR+/+_B/S_C2	Cecum DR+/+_B/S_C3	Cecum DR+/+_B/S_C4	Cecum DR+/+_B/S_C5	Cecum DR+/+_B/S_C6	Cecum F_FND_D1	Cecum F_FND_D2	Cecum F_FND_D3	Cecum F_FND_D4	Cecum F_FND_D5	Cecum F_FND_D6	Ileum DR+/+_ND_E1	Ileum DR+/+_ND_E2	Ileum DR+/+_ND_E3	Ileum DR+/+_ND_E4	Ileum DR+/+_ND_E5	Ileum DR+/+_ND_E6	Ileum DR+/+_HCD_F1	Ileum DR+/+_HCD_F2	Ileum DR+/+_HCD_F3	Ileum DR+/+_HCD_F4	Ileum DR+/+_HCD_F5	Ileum DR+/+_HCD_F6	Ileum DR+/+_B/S_G1	Ileum DR+/+_B/S_G2	Ileum DR+/+_B/S_G3	Ileum DR+/+_B/S_G4	Ileum DR+/+_B/S_G5	Ileum DR+/+_B/S_G6	Ileum F_FND_H1	Ileum F_FND_H2	Ileum F_FND_H3	Ileum F_FND_H4	Ileum F_FND_H5	Ileum F_FND_H6
k__Archaea;p__Euryarchaeota;c__Thermoplasmata;o__E2;f__[Methanomassiliicoccaceae];g__vadinCA11;s__	7	0	0	7	5	6	0	1	1	0	0	2	0	0	0	0	0	0	13	1	8	1	0	0	0	0	0	0	0	0	0	0	0	0	0	0	0	0	0	0	0	0	0	0	0	0	0	0
k__Bacteria;p__Actinobacteria;c__Actinobacteria;o__Actinomycetales;f__;g__;s__	0	0	0	0	0	0	0	0	0	0	0	0	1	0	0	0	0	0	0	0	0	0	0	0	0	0	0	0	0	0	0	0	0	0	0	0	0	0	0	0	1	0	0	0	0	0	0	0
k__Bacteria;p__Actinobacteria;c__Actinobacteria;o__Actinomycetales;f__Corynebacteriaceae;g__Corynebacterium;s__	0	0	0	0	0	0	0	0	0	0	0	0	3	0	0	0	0	0	0	0	0	0	0	0	0	0	0	0	0	0	0	0	0	0	0	0	0	0	0	0	2	0	0	0	0	0	0	0
k__Bacteria;p__Actinobacteria;c__Actinobacteria;o__Actinomycetales;f__Microbacteriaceae;g__Curtobacterium;s__	0	0	0	0	0	0	0	0	0	0	0	0	9	0	0	0	4	0	0	0	0	0	0	0	0	0	0	0	0	0	0	0	0	0	0	0	2	0	0	7	14	7	0	0	0	0	0	0
k__Bacteria;p__Actinobacteria;c__Actinobacteria;o__Actinomycetales;f__Micrococcaceae;g__Arthrobacter;s__	0	0	0	0	0	0	0	0	0	0	0	0	0	0	0	0	0	0	0	0	0	0	0	0	0	0	0	0	0	0	0	0	0	0	0	0	0	0	0	0	2	1	0	0	0	0	0	0
k__Bacteria;p__Actinobacteria;c__Actinobacteria;o__Actinomycetales;f__Micrococcaceae;g__Rothia;s__terrae	0	0	0	0	0	0	0	3	1	0	0	0	0	0	0	0	0	0	0	0	0	0	1	0	0	1	0	0	0	0	0	0	0	49	1	0	0	0	0	0	0	0	0	0	0	0	1	1
k__Bacteria;p__Actinobacteria;c__Actinobacteria;o__Bifidobacteriales;f__Bifidobacteriaceae;g__Bifidobacterium;s__	2	20	51	12	31	38	0	0	3	0	0	8	0	0	0	0	0	0	2	9	7	3	6	4	7	126	48	114	40	79	1	0	32	1	1	5	0	6	0	1	0	1	2	7	28	1	8	18
k__Bacteria;p__Actinobacteria;c__Coriobacteriia;o__Coriobacteriales;f__Coriobacteriaceae;g__;s__	3	3	1	0	4	0	1	0	0	0	1	0	0	0	0	0	0	0	2	0	0	2	1	1	1	1	2	0	0	1	0	0	2	0	0	0	0	1	0	0	1	0	0	0	0	2	0	0
k__Bacteria;p__Actinobacteria;c__Coriobacteriia;o__Coriobacteriales;f__Coriobacteriaceae;g__Adlercreutzia;s__	3	0	1	1	3	0	0	0	1	0	1	1	0	0	0	0	0	0	1	1	0	1	1	0	0	1	1	0	0	0	0	0	0	0	0	0	0	0	0	0	0	0	0	0	0	0	0	0
k__Bacteria;p__Actinobacteria;c__Coriobacteriia;o__Coriobacteriales;f__Coriobacteriaceae;g__Collinsella;s__	0	0	2	1	0	0	1	0	26	0	0	20	0	0	0	0	0	0	0	2	1	0	0	1	0	0	0	0	1	0	0	0	3	0	0	0	0	1	0	0	0	0	0	0	0	0	0	0
k__Bacteria;p__Bacteroidetes;c__Bacteroidia;o__Bacteroidales;f__;g__;s__	7	2	13	8	12	2	21	18	0	33	33	0	0	0	0	0	0	0	4	1	9	3	5	7	0	0	0	0	0	0	0	0	0	3	1	0	0	0	0	0	0	0	0	0	0	0	0	0
k__Bacteria;p__Bacteroidetes;c__Bacteroidia;o__Bacteroidales;f__Bacteroidaceae;g__Bacteroides;s__	3	4	10	5	9	127	704	684	638	783	1023	429	7	0	0	0	0	0	108	53	107	28	253	104	0	0	0	0	0	0	0	1	0	58	11	0	0	1	0	1	1	9	0	0	0	0	0	0
k__Bacteria;p__Bacteroidetes;c__Bacteroidia;o__Bacteroidales;f__Bacteroidaceae;g__Bacteroides;s__acidifaciens	1	3	2	1	0	2	1	0	9	9	21	21	0	0	0	0	0	0	1	0	0	0	0	0	0	0	0	0	0	0	0	0	0	1	2	0	0	0	0	0	0	0	0	0	0	0	0	0
k__Bacteria;p__Bacteroidetes;c__Bacteroidia;o__Bacteroidales;f__Bacteroidaceae;g__Bacteroides;s__uniformis	0	1	0	0	0	152	4	2	42	30	88	42	0	1	0	0	0	0	1	0	4	3	1	0	0	0	0	0	0	0	0	0	0	3	3	0	0	0	0	0	0	2	0	0	0	0	0	0
k__Bacteria;p__Bacteroidetes;c__Bacteroidia;o__Bacteroidales;f__Porphyromonadaceae;g__Parabacteroides;s__	3	5	4	4	2	13	7	2	4	0	1	0	0	0	0	0	0	0	9	2	0	3	1	1	0	0	0	0	0	0	0	0	0	0	2	0	0	0	0	0	0	0	0	0	0	0	0	0
k__Bacteria;p__Bacteroidetes;c__Bacteroidia;o__Bacteroidales;f__Prevotellaceae;g__Prevotella;s__	350	260	339	589	574	739	0	0	0	0	0	0	23	0	0	1	0	0	488	833	928	478	1202	698	0	1	0	0	0	0	0	0	0	0	0	0	1	0	0	1	3	0	0	0	0	0	0	0
k__Bacteria;p__Bacteroidetes;c__Bacteroidia;o__Bacteroidales;f__Prevotellaceae;g__Prevotella;s__copri	148	143	214	103	157	22	1	0	0	0	0	0	27	0	0	1	0	0	705	270	17	315	168	196	0	0	0	0	0	0	0	0	0	0	1	0	1	0	0	0	0	0	0	0	0	0	0	0
k__Bacteria;p__Bacteroidetes;c__Bacteroidia;o__Bacteroidales;f__RF16;g__;s__	78	10	54	48	56	118	28	104	197	25	42	231	6	0	0	0	0	0	121	53	51	60	16	29	0	0	0	0	0	0	0	0	0	3	1	0	0	0	0	1	0	4	0	0	0	0	0	0
k__Bacteria;p__Bacteroidetes;c__Bacteroidia;o__Bacteroidales;f__Rikenellaceae;g__;s__	0	5	5	4	5	5	11	26	12	4	10	10	0	0	0	0	0	0	3	1	0	0	0	0	0	0	0	0	0	0	0	0	0	1	0	0	0	0	0	0	0	0	0	0	0	0	0	0
k__Bacteria;p__Bacteroidetes;c__Bacteroidia;o__Bacteroidales;f__S24-7;g__;s__	122	236	197	396	731	166	129	279	147	35	73	98	1	0	0	0	0	0	118	197	114	50	158	136	2	4	236	1	34	5	0	0	0	3	1	0	3	0	0	1	1	6	1	0	0	1	2	0
k__Bacteria;p__Bacteroidetes;c__Bacteroidia;o__Bacteroidales;f__[Barnesiellaceae];g__;s__	0	0	0	0	0	0	0	0	2	1	0	2	0	0	0	0	0	0	0	0	0	0	0	0	0	0	0	0	0	0	0	0	0	0	0	0	0	0	0	0	0	0	0	0	0	0	0	0
k__Bacteria;p__Bacteroidetes;c__Bacteroidia;o__Bacteroidales;f__[Odoribacteraceae];g__Butyricimonas;s__	0	0	0	0	2	1	7	4	11	3	7	8	0	0	0	0	0	0	0	0	0	0	0	1	0	0	0	0	0	0	0	0	0	0	0	0	0	0	0	0	0	1	0	0	0	0	0	0
k__Bacteria;p__Bacteroidetes;c__Bacteroidia;o__Bacteroidales;f__[Paraprevotellaceae];g__YRC22;s__	1	0	2	1	0	2	0	0	0	0	0	0	1	0	0	0	0	0	41	8	122	4	23	23	0	0	0	0	0	0	1	0	0	0	0	0	0	0	0	0	0	0	0	0	0	0	0	0
k__Bacteria;p__Bacteroidetes;c__Bacteroidia;o__Bacteroidales;f__[Paraprevotellaceae];g__[Prevotella];s__	35	50	75	60	63	212	298	96	288	0	4	86	4	0	0	0	0	0	157	214	107	102	123	171	0	0	0	0	0	0	0	0	0	0	0	0	2	0	1	0	0	2	0	0	0	0	0	0
k__Bacteria;p__Cyanobacteria;c__4C0d-2;o__YS2;f__;g__;s__	0	1	4	10	5	0	4	28	0	3	6	0	0	0	0	0	0	0	0	2	19	0	8	4	0	0	0	0	0	0	0	0	0	0	0	0	0	0	0	0	0	0	0	0	0	0	0	0
k__Bacteria;p__Cyanobacteria;c__Chloroplast;o__Streptophyta;f__;g__;s__	0	0	0	0	0	0	0	0	0	0	0	0	23	0	0	0	7	0	0	0	0	0	0	0	0	0	0	0	0	0	0	0	0	0	0	0	37	5	21	24	17	16	0	0	0	0	0	0
k__Bacteria;p__Deferribacteres;c__Deferribacteres;o__Deferribacterales;f__Deferribacteraceae;g__Mucispirillum;s__schaedleri	17	36	18	26	7	27	34	7	22	3	0	10	4	0	0	0	0	0	109	51	28	34	2	14	0	0	0	0	0	0	0	0	0	0	0	0	0	0	0	0	1	1	0	0	0	0	0	0
k__Bacteria;p__Elusimicrobia;c__Elusimicrobia;o__Elusimicrobiales;f__Elusimicrobiaceae;g__;s__	6	0	0	0	0	0	0	0	0	0	0	0	0	0	0	0	0	0	0	2	0	0	0	20	0	0	0	0	0	0	0	0	0	0	0	0	0	0	0	0	0	0	0	0	0	0	0	0
k__Bacteria;p__Firmicutes;c__Bacilli;o__Bacillales;f__Alicyclobacillaceae;g__Alicyclobacillus;s__	0	0	0	0	0	0	0	0	0	0	0	0	0	0	0	0	0	0	0	0	0	0	0	0	0	0	0	0	0	0	0	0	0	0	0	0	0	4	2	0	0	15	0	0	0	0	0	0
k__Bacteria;p__Firmicutes;c__Bacilli;o__Bacillales;f__Bacillaceae;g__Bacillus;s__	0	0	0	0	0	0	0	0	0	0	0	0	2	0	0	0	3	0	0	0	0	0	0	0	0	0	0	0	0	0	0	0	0	0	0	0	3	1	0	2	2	5	0	0	0	0	0	0
k__Bacteria;p__Firmicutes;c__Bacilli;o__Bacillales;f__Bacillaceae;g__Geobacillus;s__	0	0	0	0	0	0	0	0	0	0	0	0	0	0	0	0	0	0	0	0	0	0	0	0	0	0	0	0	0	0	0	0	0	0	0	0	1	0	0	5	2	0	0	0	0	0	0	0
k__Bacteria;p__Firmicutes;c__Bacilli;o__Bacillales;f__Bacillaceae;g__Oceanobacillus;s__chironomi	0	0	0	0	0	0	0	0	0	0	0	0	0	0	0	0	0	0	0	0	0	0	0	0	0	0	0	0	0	0	0	0	0	0	0	0	0	1	2	0	0	0	0	0	0	0	0	0
k__Bacteria;p__Firmicutes;c__Bacilli;o__Bacillales;f__Paenibacillaceae;g__Brevibacillus;s__reuszeri	0	0	0	0	0	0	0	0	0	0	0	0	1	0	0	0	0	0	0	0	0	0	0	0	0	0	0	0	0	0	0	0	0	0	0	0	0	0	1	0	0	1	0	0	0	0	0	0
k__Bacteria;p__Firmicutes;c__Bacilli;o__Bacillales;f__Paenibacillaceae;g__Paenibacillus;s__	0	0	0	0	0	0	0	0	0	0	0	0	0	0	0	0	2	0	0	0	0	0	0	0	0	0	0	0	0	0	0	0	0	0	0	0	0	0	0	0	0	3	0	0	0	0	0	0
k__Bacteria;p__Firmicutes;c__Bacilli;o__Bacillales;f__Paenibacillaceae;g__Paenibacillus;s__amylolyticus	0	0	0	0	0	0	0	0	0	0	0	0	0	0	0	0	0	0	0	0	0	0	0	0	0	0	0	0	0	0	0	0	0	0	0	0	2	0	0	1	1	0	0	0	0	0	0	0
k__Bacteria;p__Firmicutes;c__Bacilli;o__Bacillales;f__Planococcaceae;g__;s__	0	0	0	0	2	0	0	0	0	0	0	0	6	0	1	1	1	0	2	0	0	0	0	0	0	0	0	0	0	0	0	0	0	0	0	0	3	0	4	3	15	6	0	0	0	0	1	0
k__Bacteria;p__Firmicutes;c__Bacilli;o__Bacillales;f__Planococcaceae;g__Rummeliibacillus;s__	0	0	0	0	0	0	0	0	0	0	0	0	3	0	0	0	1	0	0	0	0	0	0	0	0	0	0	0	0	0	0	0	0	0	0	0	2	0	0	1	52	0	0	0	0	0	0	0
k__Bacteria;p__Firmicutes;c__Bacilli;o__Bacillales;f__Planococcaceae;g__Sporosarcina;s__	0	0	0	0	0	0	0	0	0	0	0	0	0	0	0	0	0	0	0	0	0	0	0	0	0	0	0	0	0	0	0	0	0	0	0	0	0	0	0	0	4	0	0	0	0	0	0	0
k__Bacteria;p__Firmicutes;c__Bacilli;o__Bacillales;f__Staphylococcaceae;g__Jeotgalicoccus;s__	0	0	0	0	0	0	0	0	0	0	0	0	1	0	0	0	0	0	0	0	0	0	0	0	0	0	0	0	0	0	0	0	0	0	0	0	2	0	0	0	0	3	0	0	0	0	0	0
k__Bacteria;p__Firmicutes;c__Bacilli;o__Bacillales;f__Thermoactinomycetaceae;g__Thermoactinomyces;s__	0	0	0	0	0	0	0	0	0	0	0	0	1	0	0	0	0	0	0	0	0	0	0	0	0	0	0	0	0	0	0	0	0	0	0	0	0	0	0	1	0	0	0	0	0	0	0	0
k__Bacteria;p__Firmicutes;c__Bacilli;o__Gemellales;f__Gemellaceae;g__Gemella;s__	0	0	0	0	0	0	0	2	0	0	1	0	1	0	0	0	0	0	0	0	0	0	0	0	0	1	0	0	0	1	3	3	1	26	4	1	0	0	0	0	0	0	1	1	1	0	1	0
k__Bacteria;p__Firmicutes;c__Bacilli;o__Lactobacillales;f__Aerococcaceae;g__Facklamia;s__	0	0	0	0	0	0	0	0	0	0	0	1	0	0	0	0	0	0	0	0	0	0	0	0	0	0	0	0	0	1	0	0	0	3	0	0	0	0	0	0	0	0	0	0	0	0	1	0
k__Bacteria;p__Firmicutes;c__Bacilli;o__Lactobacillales;f__Carnobacteriaceae;g__Granulicatella;s__	0	0	0	0	0	0	0	0	0	0	0	0	2	0	0	0	0	0	0	0	0	0	0	0	0	2	1	1	0	1	0	0	0	8	0	0	3	0	1	0	7	7	0	0	0	2	0	0
k__Bacteria;p__Firmicutes;c__Bacilli;o__Lactobacillales;f__Lactobacillaceae;g__Lactobacillus;s__	336	462	542	135	410	184	372	83	96	481	335	599	81	0	0	1	22	0	156	244	182	80	250	273	1653	698	1115	1824	557	627	2171	1909	1144	1005	1789	1953	85	59	46	64	65	36	818	2261	1537	767	914	824
k__Bacteria;p__Firmicutes;c__Bacilli;o__Lactobacillales;f__Lactobacillaceae;g__Lactobacillus;s__brevis	0	0	0	0	0	0	0	0	0	0	0	0	57	0	0	4	17	0	0	0	0	0	0	0	0	0	0	0	0	0	0	0	0	0	0	0	25	3	11	31	100	27	0	0	0	0	0	0
k__Bacteria;p__Firmicutes;c__Bacilli;o__Lactobacillales;f__Lactobacillaceae;g__Lactobacillus;s__paralimentarius	0	0	0	0	0	0	0	0	0	0	0	0	5	0	0	1	6	0	0	0	0	0	0	0	0	0	0	0	0	0	0	0	0	0	0	0	7	1	1	5	3	7	0	0	0	0	0	0
k__Bacteria;p__Firmicutes;c__Bacilli;o__Lactobacillales;f__Lactobacillaceae;g__Lactobacillus;s__reuteri	47	135	84	21	43	41	27	36	13	97	29	126	1	0	0	0	1	0	26	53	55	30	51	70	178	199	152	221	49	113	147	303	168	488	135	223	3	8	4	0	2	11	112	796	623	246	230	240
k__Bacteria;p__Firmicutes;c__Bacilli;o__Lactobacillales;f__Leuconostocaceae;g__Leuconostoc;s__	0	0	0	0	0	0	0	0	0	0	0	0	0	0	0	0	0	0	0	0	0	0	0	0	0	0	0	0	0	0	0	0	0	0	0	0	0	0	0	0	0	5	0	0	0	0	0	0
k__Bacteria;p__Firmicutes;c__Bacilli;o__Lactobacillales;f__Leuconostocaceae;g__Weissella;s__hellenica	0	0	0	0	0	0	0	0	0	0	0	0	70	0	1	1	11	0	0	0	0	0	0	0	0	0	0	0	0	0	0	0	0	0	0	0	32	2	10	18	44	22	0	0	0	0	0	0
k__Bacteria;p__Firmicutes;c__Bacilli;o__Lactobacillales;f__Streptococcaceae;g__Lactococcus;s__	0	0	0	0	0	0	0	0	0	0	0	0	35	0	0	1	5	0	0	0	0	0	0	0	0	0	0	0	0	0	0	0	0	0	0	0	61	0	0	8	4	2	0	0	0	0	0	0
k__Bacteria;p__Firmicutes;c__Bacilli;o__Lactobacillales;f__Streptococcaceae;g__Streptococcus;s__	1	0	1	1	2	2	5	4	2	8	6	6	881	1	3	31	467	1	7	4	5	4	2	3	2	9	4	5	2	1	6	15	31	103	4	7	933	209	603	931	1483	1008	4	16	6	6	31	26
k__Bacteria;p__Firmicutes;c__Bacilli;o__Turicibacterales;f__Turicibacteraceae;g__Turicibacter;s__	193	206	300	231	323	386	0	0	1	0	0	1	46	0	0	0	8	0	268	96	465	96	212	112	200	1218	624	601	1774	805	1	0	32	0	0	6	41	46	6	42	35	20	1150	375	934	881	1278	1347
k__Bacteria;p__Firmicutes;c__Clostridia;o__Clostridiales;f__;g__;s__	829	627	695	699	719	345	524	787	118	351	485	492	113	0	0	2	2	0	1395	236	232	683	350	201	1	21	11	0	5	1	0	0	0	33	8	0	6	0	3	27	2	4	2	0	0	5	0	0
k__Bacteria;p__Firmicutes;c__Clostridia;o__Clostridiales;f__Christensenellaceae;g__;s__	0	3	1	0	3	5	0	2	5	0	0	0	0	0	0	0	0	0	2	1	1	1	0	0	0	0	0	0	0	0	0	0	0	0	0	0	0	0	0	0	0	0	0	0	0	0	0	0
k__Bacteria;p__Firmicutes;c__Clostridia;o__Clostridiales;f__Christensenellaceae;g__Christensenella;s__	0	0	0	0	0	0	1	0	0	0	0	1	0	0	0	0	0	0	0	0	0	0	0	0	0	0	0	0	0	0	0	0	0	0	0	0	0	0	0	0	0	0	0	0	0	0	0	0
k__Bacteria;p__Firmicutes;c__Clostridia;o__Clostridiales;f__Clostridiaceae;g__;s__	131	57	100	71	98	246	188	33	154	37	39	195	213	1	1	8	62	0	64	41	143	10	49	20	276	274	148	190	519	1153	1046	85	2074	5	3	282	228	56	48	181	249	187	214	100	227	149	384	606
k__Bacteria;p__Firmicutes;c__Clostridia;o__Clostridiales;f__Clostridiaceae;g__Clostridium;s__	0	1	0	0	1	1	0	3	1	1	0	4	8	0	0	0	1	0	5	0	0	0	1	0	0	0	2	0	0	0	0	0	0	0	0	0	21	0	3	10	8	6	0	0	0	0	0	1
k__Bacteria;p__Firmicutes;c__Clostridia;o__Clostridiales;f__Clostridiaceae;g__Clostridium;s__tetani	0	0	0	0	0	0	0	0	0	0	0	0	1	0	0	1	0	0	0	0	0	0	0	0	0	0	0	0	0	0	0	0	0	0	0	0	0	0	0	2	1	0	0	0	0	0	0	0
k__Bacteria;p__Firmicutes;c__Clostridia;o__Clostridiales;f__Clostridiaceae;g__Natronincola_Anaerovirgula;s__	0	0	0	0	0	0	0	0	0	0	0	0	0	0	0	0	0	0	0	0	0	0	0	0	0	0	0	0	0	0	0	0	0	0	0	0	0	0	0	0	3	0	0	0	0	0	0	0
k__Bacteria;p__Firmicutes;c__Clostridia;o__Clostridiales;f__Dehalobacteriaceae;g__Dehalobacterium;s__	0	0	0	0	0	0	0	2	0	0	0	0	0	0	0	0	0	0	0	0	0	1	0	0	0	0	0	0	0	0	0	0	0	0	0	0	0	0	0	0	0	0	0	0	0	0	0	0
k__Bacteria;p__Firmicutes;c__Clostridia;o__Clostridiales;f__Lachnospiraceae;g__;s__	99	217	171	113	279	359	259	359	333	260	171	346	14	0	0	1	2	0	278	77	65	93	139	115	0	11	2	1	3	0	3	2	13	16	2	0	1	0	2	5	3	17	0	0	2	0	1	0
k__Bacteria;p__Firmicutes;c__Clostridia;o__Clostridiales;f__Lachnospiraceae;g__Anaerostipes;s__	11	3	14	1	0	0	0	0	0	0	0	0	0	0	0	0	0	0	0	1	0	0	1	1	0	0	0	0	0	0	0	0	0	0	0	0	0	0	0	0	0	0	0	0	0	0	0	0
k__Bacteria;p__Firmicutes;c__Clostridia;o__Clostridiales;f__Lachnospiraceae;g__Blautia;s__	10	5	37	3	7	28	4	9	126	4	3	74	0	0	0	0	0	0	1	41	39	0	81	23	0	0	0	0	0	0	0	2	6	1	0	2	0	0	1	0	0	7	0	0	0	0	0	0
k__Bacteria;p__Firmicutes;c__Clostridia;o__Clostridiales;f__Lachnospiraceae;g__Blautia;s__producta	0	0	0	0	0	0	0	0	72	0	0	56	0	0	0	0	0	0	0	0	0	0	0	0	0	0	0	0	0	0	0	0	15	0	0	1	0	0	0	0	0	2	0	0	0	0	0	0
k__Bacteria;p__Firmicutes;c__Clostridia;o__Clostridiales;f__Lachnospiraceae;g__Clostridium;s__colinum	0	0	4	0	0	0	0	0	1	0	0	2	4	0	0	0	3	0	38	0	0	11	0	0	0	1	1	0	0	0	0	0	12	0	0	13	3	0	0	0	2	1	1133	0	0	504	0	0
k__Bacteria;p__Firmicutes;c__Clostridia;o__Clostridiales;f__Lachnospiraceae;g__Coprococcus;s__	36	9	11	37	35	5	27	7	48	36	9	29	3	0	0	0	0	0	12	23	4	9	29	3	0	0	0	0	0	0	0	0	0	0	0	0	2	0	0	0	0	3	0	0	0	0	0	0
k__Bacteria;p__Firmicutes;c__Clostridia;o__Clostridiales;f__Lachnospiraceae;g__Dorea;s__	2	0	3	0	1	3	4	1	11	1	2	2	0	0	0	0	0	0	0	3	3	0	5	2	0	0	0	0	0	0	0	0	0	0	0	0	0	0	0	0	0	0	0	0	0	0	0	0
k__Bacteria;p__Firmicutes;c__Clostridia;o__Clostridiales;f__Lachnospiraceae;g__[Ruminococcus];s__gnavus	22	23	28	21	23	13	223	205	642	218	25	492	0	0	0	0	0	0	10	6	3	7	1	8	1	4	4	0	4	0	3	0	2	32	0	0	0	0	0	2	1	14	0	0	0	0	0	0
k__Bacteria;p__Firmicutes;c__Clostridia;o__Clostridiales;f__Peptococcaceae;g__;s__	0	3	1	3	2	1	5	5	2	6	1	9	0	0	0	0	0	0	3	2	2	0	4	0	0	0	0	0	0	0	0	0	0	0	0	0	0	1	0	0	0	0	0	0	0	0	0	0
k__Bacteria;p__Firmicutes;c__Clostridia;o__Clostridiales;f__Peptococcaceae;g__rc4-4;s__	0	0	0	0	0	0	0	4	0	0	4	0	0	0	0	0	0	0	0	0	0	0	2	9	0	0	0	0	1	0	0	0	0	0	0	0	0	0	0	0	0	0	0	0	0	0	0	0
k__Bacteria;p__Firmicutes;c__Clostridia;o__Clostridiales;f__Peptostreptococcaceae;g__;s__	0	0	0	0	0	0	0	0	0	0	0	0	2	0	0	0	1	0	0	0	0	0	0	0	0	0	0	0	0	0	0	0	0	0	0	0	6	0	2	2	1	0	0	0	0	0	0	0
k__Bacteria;p__Firmicutes;c__Clostridia;o__Clostridiales;f__Peptostreptococcaceae;g__Peptostreptococcus;s__	0	0	0	0	0	0	0	0	0	0	0	0	2	0	0	0	1	0	0	0	0	0	0	0	0	0	0	0	0	0	0	0	0	0	0	0	16	0	0	5	5	0	0	0	0	0	0	0
k__Bacteria;p__Firmicutes;c__Clostridia;o__Clostridiales;f__Peptostreptococcaceae;g__Peptostreptococcus;s__anaerobius	0	0	0	0	0	0	0	0	0	0	0	0	5	0	0	1	1	0	0	0	0	0	0	0	0	0	0	0	0	0	0	0	0	0	0	0	0	2	0	0	3	1	0	0	0	0	0	0
k__Bacteria;p__Firmicutes;c__Clostridia;o__Clostridiales;f__Peptostreptococcaceae;g__[Clostridium];s__bifermentans	0	0	0	0	0	0	0	0	0	0	0	0	4	0	0	1	1	0	0	0	0	0	0	0	0	0	0	0	0	0	0	0	0	0	0	0	12	0	0	6	5	5	0	0	0	0	0	0
k__Bacteria;p__Firmicutes;c__Clostridia;o__Clostridiales;f__Ruminococcaceae;g__;s__	89	53	82	96	93	306	102	99	140	26	33	40	14	0	0	0	0	0	267	41	31	86	34	39	0	1	0	0	0	0	0	1	0	1	2	0	0	0	0	3	0	11	0	0	0	0	0	0
k__Bacteria;p__Firmicutes;c__Clostridia;o__Clostridiales;f__Ruminococcaceae;g__Oscillospira;s__	81	117	137	122	127	64	230	244	206	485	127	118	23	0	0	0	0	0	196	69	45	190	48	55	0	0	0	0	0	0	0	1	0	41	3	0	1	0	0	4	3	8	0	0	0	0	0	0
k__Bacteria;p__Firmicutes;c__Clostridia;o__Clostridiales;f__Ruminococcaceae;g__Ruminococcus;s__	42	9	31	44	85	104	15	9	8	3	7	1	376	2306	1487	4326	3482	3039	147	53	47	51	47	76	0	1	0	0	0	0	0	0	0	0	0	1	14	1157	1503	878	3	628	2	0	2	1	1	0
k__Bacteria;p__Firmicutes;c__Clostridia;o__Clostridiales;f__Veillonellaceae;g__Phascolarctobacterium;s__	13	15	29	12	21	19	26	51	42	10	55	16	3	0	0	0	0	0	33	31	27	18	30	32	0	0	0	0	0	0	0	0	0	1	0	1	0	0	0	0	0	3	0	0	0	0	0	0
k__Bacteria;p__Firmicutes;c__Clostridia;o__Clostridiales;f__Veillonellaceae;g__Veillonella;s__parvula	0	0	0	0	0	0	0	0	0	0	0	0	0	0	0	0	0	0	0	0	0	0	0	1	0	0	0	0	2	1	0	0	0	1	0	0	0	0	0	0	1	0	1	2	0	0	3	7
k__Bacteria;p__Firmicutes;c__Clostridia;o__Clostridiales;f__[Mogibacteriaceae];g__;s__	1	2	0	3	2	9	83	41	63	29	54	42	1	0	0	0	0	0	3	3	1	2	8	0	0	0	0	0	0	0	2	1	1	3	3	0	0	0	0	0	0	2	0	0	0	0	0	0
k__Bacteria;p__Firmicutes;c__Clostridia;o__Clostridiales;f__[Tissierellaceae];g__Anaerococcus;s__	0	0	0	0	0	0	0	0	0	0	0	0	5	0	0	0	2	0	0	0	0	0	0	0	0	0	0	0	0	0	0	0	0	0	0	0	9	0	2	3	8	8	0	0	0	0	0	0
k__Bacteria;p__Firmicutes;c__Clostridia;o__Clostridiales;f__[Tissierellaceae];g__Gallicola;s__	0	0	0	0	0	0	0	0	0	0	0	0	1	0	0	0	1	0	0	0	0	0	0	0	0	0	0	0	0	0	0	0	0	0	0	0	1	0	2	1	0	2	0	0	0	0	0	0
k__Bacteria;p__Firmicutes;c__Clostridia;o__Clostridiales;f__[Tissierellaceae];g__Peptoniphilus;s__	0	0	0	0	0	0	0	0	0	0	0	0	0	0	0	0	0	0	0	0	0	0	0	0	0	0	0	0	0	0	0	0	0	0	0	0	2	0	0	0	0	4	0	0	0	0	0	0
k__Bacteria;p__Firmicutes;c__Clostridia;o__Clostridiales;f__[Tissierellaceae];g__Sporanaerobacter;s__	0	0	0	0	0	0	0	0	0	0	0	0	5	0	0	0	0	0	0	0	0	0	0	0	0	0	0	0	0	0	0	0	0	0	0	0	0	0	1	0	3	0	0	0	0	0	0	0
k__Bacteria;p__Firmicutes;c__Clostridia;o__Clostridiales;f__[Tissierellaceae];g__Tepidimicrobium;s__	0	0	0	0	0	0	0	0	0	0	0	0	0	0	0	0	0	0	0	0	0	0	0	0	0	0	0	0	0	0	0	0	0	0	0	0	2	0	0	0	3	0	0	0	0	0	0	0
k__Bacteria;p__Firmicutes;c__Erysipelotrichi;o__Erysipelotrichales;f__Erysipelotrichaceae;g__;s__	1	0	3	1	4	2	0	1	17	0	0	1	0	0	0	0	0	0	1	12	5	0	8	4	0	0	0	0	0	0	0	0	4	4	0	0	0	0	0	0	0	0	0	0	0	0	2	1
k__Bacteria;p__Firmicutes;c__Erysipelotrichi;o__Erysipelotrichales;f__Erysipelotrichaceae;g__Allobaculum;s__	159	83	56	90	381	185	1	1	6	1	0	1	1	0	0	0	0	0	18	47	25	3	14	24	248	115	125	134	150	352	3	0	32	0	0	1	0	6	0	3	1	4	36	33	59	17	30	26
k__Bacteria;p__Firmicutes;c__Erysipelotrichi;o__Erysipelotrichales;f__Erysipelotrichaceae;g__Coprobacillus;s__	0	0	3	1	3	1	2	0	4	1	1	1	0	0	0	0	0	0	0	4	0	0	7	3	0	0	0	0	0	0	0	0	0	0	0	0	0	0	1	0	0	0	0	0	0	0	0	0
k__Bacteria;p__Firmicutes;c__Erysipelotrichi;o__Erysipelotrichales;f__Erysipelotrichaceae;g__Holdemania;s__	0	0	0	1	0	0	1	0	2	0	1	0	0	0	0	0	0	0	0	0	0	0	0	0	0	0	0	0	0	0	0	0	0	0	0	0	0	0	0	0	0	0	0	0	0	0	0	0
k__Bacteria;p__Firmicutes;c__Erysipelotrichi;o__Erysipelotrichales;f__Erysipelotrichaceae;g__Sharpea;s__p-3329-23G2	0	0	0	0	0	0	0	0	0	0	0	0	0	0	0	0	0	0	0	0	0	0	0	0	0	0	0	0	0	0	0	0	0	0	0	0	0	0	2	0	0	0	0	0	0	0	0	0
k__Bacteria;p__Fusobacteria;c__Fusobacteriia;o__Fusobacteriales;f__Fusobacteriaceae;g__Fusobacterium;s__	0	0	0	0	0	0	0	0	0	0	0	0	7	0	0	0	0	0	0	0	0	0	0	0	0	0	0	0	1	0	0	0	0	1	0	0	0	0	0	0	0	0	0	0	0	0	2	0
k__Bacteria;p__Proteobacteria;c__Alphaproteobacteria;o__RF32;f__;g__;s__	0	0	0	0	0	0	0	0	0	0	0	1	0	0	0	0	0	0	0	0	0	0	10	0	0	0	0	0	0	0	0	0	0	1	0	0	0	0	0	0	0	0	0	0	0	0	0	0
k__Bacteria;p__Proteobacteria;c__Alphaproteobacteria;o__Rhizobiales;f__Aurantimonadaceae;g__Aurantimonas;s__ureilytica	0	0	0	0	0	0	0	0	0	0	0	0	0	0	0	0	0	0	0	0	0	0	0	0	0	0	0	0	0	0	0	0	0	0	0	0	0	0	0	0	3	0	0	0	0	0	0	0
k__Bacteria;p__Proteobacteria;c__Alphaproteobacteria;o__Rhizobiales;f__Bradyrhizobiaceae;g__Balneimonas;s__	0	0	0	0	0	0	0	0	0	0	0	0	0	0	0	0	0	0	0	0	0	0	0	0	0	0	0	0	0	0	0	0	0	0	0	0	0	0	0	0	4	0	0	0	0	0	0	0
k__Bacteria;p__Proteobacteria;c__Alphaproteobacteria;o__Rhizobiales;f__Brucellaceae;g__;s__	0	0	0	0	0	0	0	0	0	0	0	0	2	0	0	0	0	0	0	0	0	0	0	0	0	0	0	0	0	0	0	0	0	0	0	0	0	0	1	0	3	1	0	0	0	0	0	0
k__Bacteria;p__Proteobacteria;c__Alphaproteobacteria;o__Rhizobiales;f__Rhizobiaceae;g__Agrobacterium;s__	0	0	0	0	0	0	0	0	0	0	0	0	2	0	0	0	1	0	0	0	0	0	0	0	0	0	0	0	0	0	0	0	0	0	0	0	0	0	0	0	13	1	0	0	0	0	0	0
k__Bacteria;p__Proteobacteria;c__Alphaproteobacteria;o__Rickettsiales;f__mitochondria;g__Zea;s__luxurians	0	0	0	0	0	0	0	0	0	0	0	0	0	0	0	0	0	0	0	0	0	0	0	0	0	0	0	0	0	0	0	0	0	0	0	0	1	0	0	3	2	2	0	0	0	0	0	0
k__Bacteria;p__Proteobacteria;c__Alphaproteobacteria;o__Sphingomonadales;f__Sphingomonadaceae;g__Blastomonas;s__	0	0	0	0	0	0	0	0	0	0	0	0	0	0	0	0	0	0	0	0	0	0	0	0	0	0	0	0	0	0	0	0	0	0	0	0	3	0	0	2	2	0	0	0	0	0	0	0
k__Bacteria;p__Proteobacteria;c__Alphaproteobacteria;o__Sphingomonadales;f__Sphingomonadaceae;g__Sphingobium;s__	0	0	0	0	0	0	0	0	0	0	0	0	3	0	0	2	1	0	0	0	0	0	0	0	0	0	0	0	0	0	0	0	0	0	0	0	1	0	0	0	3	0	0	0	0	0	0	0
k__Bacteria;p__Proteobacteria;c__Alphaproteobacteria;o__Sphingomonadales;f__Sphingomonadaceae;g__Sphingomonas;s__yabuuchiae	0	0	0	0	0	0	0	0	0	0	0	0	0	0	0	0	0	0	0	0	0	0	0	0	0	0	0	0	0	0	0	0	0	0	0	0	0	0	0	0	3	0	0	0	0	0	0	0
k__Bacteria;p__Proteobacteria;c__Betaproteobacteria;o__Burkholderiales;f__Alcaligenaceae;g__Sutterella;s__	13	10	19	12	28	15	3	11	4	1	10	3	1	1619	1597	0	0	1388	0	6	3	2	2	5	4	1	33	2	3	0	0	0	0	0	0	0	0	1722	918	0	0	330	0	0	0	0	0	0
k__Bacteria;p__Proteobacteria;c__Betaproteobacteria;o__Burkholderiales;f__Burkholderiaceae;g__Burkholderia;s__	0	0	0	0	0	0	0	0	0	0	0	0	0	0	0	0	0	0	0	0	0	0	0	0	0	0	0	0	0	0	0	0	0	0	0	0	0	0	3	0	0	3	0	0	0	0	0	0
k__Bacteria;p__Proteobacteria;c__Deltaproteobacteria;o__Desulfovibrionales;f__Desulfovibrionaceae;g__;s__	127	102	158	81	92	163	470	384	358	354	263	241	11	0	0	0	0	0	150	141	59	93	0	11	0	0	0	0	3	0	1	0	0	10	5	0	0	1	0	2	1	3	0	0	0	0	0	0
k__Bacteria;p__Proteobacteria;c__Deltaproteobacteria;o__Desulfovibrionales;f__Desulfovibrionaceae;g__Desulfovibrio;s__	1	1	1	3	0	4	1	0	8	5	7	2	0	0	0	0	0	0	14	6	16	8	5	9	0	0	0	0	0	0	0	0	0	0	0	0	0	0	0	0	0	0	0	0	0	0	0	0
k__Bacteria;p__Proteobacteria;c__Epsilonproteobacteria;o__Campylobacterales;f__Helicobacteraceae;g__;s__	135	107	133	78	115	41	301	212	56	72	38	64	1	0	0	0	0	0	0	0	0	0	0	0	0	0	0	0	0	0	0	0	0	1	1	0	0	0	0	0	0	0	0	0	0	0	0	0
k__Bacteria;p__Proteobacteria;c__Epsilonproteobacteria;o__Campylobacterales;f__Helicobacteraceae;g__Helicobacter;s__apodemus	320	225	243	210	229	213	389	361	38	298	172	50	24	1	0	0	0	0	21	102	31	781	52	139	428	1776	1259	981	2589	1697	80	25	0	270	37	0	5	40	0	2	7	14	443	2078	0	1055	2709	0
k__Bacteria;p__Proteobacteria;c__Epsilonproteobacteria;o__Campylobacterales;f__Helicobacteraceae;g__Helicobacter;s__cholecystus	2	0	0	1	0	0	0	0	0	0	0	0	0	0	0	0	0	0	0	0	0	0	0	0	1	4	0	0	1	0	0	0	0	0	0	0	0	0	0	0	0	0	0	0	0	0	0	0
k__Bacteria;p__Proteobacteria;c__Gammaproteobacteria;o__Enterobacteriales;f__Enterobacteriaceae;g__;s__	0	0	0	0	0	0	0	21	0	19	158	0	8	0	0	3	3	0	0	0	0	0	0	0	0	0	0	0	0	0	0	0	0	2	8	0	1	0	1	1	2	6	0	0	0	0	0	0
k__Bacteria;p__Proteobacteria;c__Gammaproteobacteria;o__Pasteurellales;f__Pasteurellaceae;g__Aggregatibacter;s__	0	1	0	0	0	0	0	0	0	0	0	0	0	0	0	0	0	0	0	0	0	0	1	0	0	0	0	0	7	0	0	2	0	34	0	0	0	0	0	2	0	0	0	0	0	0	0	1
k__Bacteria;p__Proteobacteria;c__Gammaproteobacteria;o__Pseudomonadales;f__Moraxellaceae;g__Acinetobacter;s__guillouiae	0	0	0	0	0	0	0	0	0	0	0	0	2	0	0	0	0	0	0	0	0	0	0	0	0	0	0	0	0	0	0	0	0	0	0	0	1	0	0	0	0	0	0	0	0	0	0	0
k__Bacteria;p__Proteobacteria;c__Gammaproteobacteria;o__Pseudomonadales;f__Moraxellaceae;g__Acinetobacter;s__rhizosphaerae	0	0	0	0	0	0	0	0	0	0	0	0	0	0	0	0	0	0	0	0	0	0	0	0	0	0	0	0	0	0	0	0	0	0	0	0	0	0	0	0	5	1	0	0	0	0	0	0
k__Bacteria;p__Proteobacteria;c__Gammaproteobacteria;o__Xanthomonadales;f__Xanthomonadaceae;g__Xanthomonas;s__	0	0	0	0	0	0	0	0	0	0	0	0	0	0	0	0	2	0	0	0	0	0	0	0	0	0	0	0	0	0	0	0	0	0	0	0	0	0	0	0	0	0	0	0	0	0	0	0
k__Bacteria;p__Spirochaetes;c__Spirochaetes;o__Spirochaetales;f__Spirochaetaceae;g__Treponema;s__	11	53	16	49	15	0	0	0	0	0	0	0	25	0	0	0	0	0	167	183	131	320	29	110	0	0	0	0	0	0	0	0	0	0	0	0	0	0	0	0	0	0	0	0	0	0	0	0
k__Bacteria;p__Tenericutes;c__Mollicutes;o__Acholeplasmatales;f__Acholeplasmataceae;g__Candidatus Phytoplasma;s__	0	0	0	0	0	0	0	0	0	0	0	0	0	0	0	0	2	0	0	0	0	0	0	0	0	0	0	0	0	0	0	0	0	0	0	0	0	0	0	4	0	0	0	0	0	0	0	0
k__Bacteria;p__Tenericutes;c__Mollicutes;o__RF39;f__;g__;s__	0	0	0	0	0	0	0	0	0	0	2	0	0	0	0	0	0	0	0	0	0	0	0	2	0	0	0	0	0	0	0	0	0	0	0	0	0	0	0	0	0	0	0	0	0	0	0	0
k__Bacteria;p__Verrucomicrobia;c__Opitutae;o__[Cerasicoccales];f__[Cerasicoccaceae];g__;s__	1	0	0	0	1	1	0	0	0	0	0	0	0	0	0	0	0	0	0	0	0	0	0	0	0	0	0	0	0	0	0	0	0	0	0	0	0	0	0	0	0	0	0	0	0	0	0	0
k__Bacteria;p__Verrucomicrobia;c__Verrucomicrobiae;o__Verrucomicrobiales;f__Verrucomicrobiaceae;g__Akkermansia;s__muciniphila	0	0	0	0	5	0	0	0	10	0	0	0	0	0	0	0	0	0	0	0	0	0	0	0	0	0	0	0	0	1	0	0	3	0	0	0	0	0	0	0	0	0	0	0	0	0	0	0
